# Accurate full-scale patient-specific Circle of Willis models including aneurysms: A novel manufacturing approach

**DOI:** 10.1371/journal.pone.0328300

**Published:** 2025-07-17

**Authors:** Jan Gottfried Minkenberg, Lara Bender, Christiane Franz, Rebecca May, Omid Nikoubashman, Martin Wiesmann, Thorsten Sichtermann

**Affiliations:** Department of Diagnostic and Interventional Neuroradiology, University Hospital RWTH Aachen, Aachen, Germany; University of Marburg: Philipps-Universitat Marburg, GERMANY

## Abstract

**Background:**

Accurate physical replicas of the Circle of Willis (CoW) are valuable for planning neuroendovascular interventions, validating computational simulations, evaluating medical devices and training physicians. Existing methods often replicate only segments of the CoW or lack geometric precision, which is critical for realistic hemodynamic simulations.

**Objective:**

We introduce a novel, cost-effective manufacturing approach to create full-scale, patient-specific CoW models using fused deposition modeling (FDM) 3D printing and lost core silicone casting. We aim to evaluate the accuracy and reproducibility of this manufacturing process.

**Methods:**

A patient-specific 3D model of the CoW with four saccular aneurysms was generated from time-of-flight magnetic resonance angiography (TOF-MRA) data. Three identical models were printed using FDM with acrylonitrile styrene acrylate (ASA) for the vascular structure and butenediol vinyl alcohol co-polymer (BVOH) as a water-soluble support material. The printed models were encased in a clear silicone block and the ASA core was then dissolved using acetone. Computed tomography (CT) scans were used to assess geometric accuracy through cloud-to-mesh distance calculations and centerline analysis.

**Results:**

The median absolute surface deviation between the replicas and the initial model was approximately 309 µm for the entire CoW, with interquartile ranges (IQR) between 360 µm and 444 µm. The aneurysm surfaces exhibited lower deviations, averaging 90 µm. Centerline analysis showed median absolute deviations in vessel radius ranging from 48 µm to 114 µm across key vascular pathways. Statistical analysis confirmed minimal discrepancies between replicas and the initial model. Each replica costs approximately €100 in materials and requires five days to produce.

**Conclusion:**

The manufacturing approach produces accurate, reproducible full-scale, patient-specific CoW models, including four aneurysms. This method simplifies the production process, reduces costs and maintains high geometric accuracy, making it suitable for hemodynamic studies, device evaluation, and clinical training.

## Introduction

Located at the base of the brain, the complex arterial network known as the Circle of Willis (CoW) facilitates collateral blood flow, ensuring a consistent blood supply to the brain [1,2]. Intracranial aneurysms affect roughly 3% of the population and represent a life-threatening condition in humans [3]. These aneurysms are commonly found in the vessels of the CoW [4]. Intracranial aneurysms typically remain asymptomatic until rupture, which results in subarachnoid hemorrhage [5], a life-threatening condition affecting approximately 9 out of 100,000 people annually [6].

Neuroendovascular treatments, targeting complete occlusion while preserving arterial blood flow, are key in managing aneurysms. These approaches involve filling the aneurysm sac with coils or diverting blood flow to induce thrombosis within the aneurysm [7]. Aneurysms primarily develop due to two key factors: weaknesses in the arterial wall and elevated wall shear stress (WSS) induced by hemodynamic forces [8–10]. WSS is particularly pronounced in areas of irregular arterial geometry, including tortuous segments, bends and bifurcations, contributing significantly to aneurysm formation and their potential for rupture. Peculiarities of the CoW, such as missing collaterals and aneurysm geometry, further complicate these conditions and influence the distribution of WSS and thus the rupture of aneurysms [11], as well as the aneurysm occlusion after treatment [12].

To better understand these interactions, in-vitro and in-silico replicas of the CoW are used. These models aid in planning neuroendovascular interventions by facilitating precise catheter shaping and the selection of other neuroendovascular devices [13,14]. In-vitro CoW replicas validate in-silico simulation results, ensuring computational accuracy in the simulation of aneurysms and hemodynamics [15,16]. These models also aid in evaluating neuroendovascular devices [17,18] and play a crucial role in the training of physicians, both in adopting new techniques and in the treatment of rare pathologies and anatomical anomalies [19,20]. Various methods, from cadaveric models [21] to additive manufacturing techniques [22,23], have been employed to create three-dimensional models of the CoW. Yet, these often focus on replicating only segments of the CoW. Full-scale, patient-specific CoW replicas are crucial for accurately replicating hemodynamic conditions, particularly concerning collaterals [24].

Based on patient image data, additive manufacturing is currently employed to create models with either flexible or rigid properties, typically in a block-like or thin-walled form. Techniques like the lost wax method [22,25–27] and printing of vessel walls [28,29] with various materials are common, each offering different levels of precision, transparency, flexibility, wall thickness and surface roughness [30,31]. Despite these advancements, replicating the entire CoW remains challenging due to its complex geometry and frequent undercuts leading to often only partially replicated CoW. While full-scale CoW models have previously been produced using stereolithography (SLA) or coating processes [32], these often result in mechanically fragile structures. In such approaches, the vessel structures are not supported by an enclosing block, making them prone to deformation, torsion, or displacement—especially during handling or when subjected to flow. Replicating the entire CoW is vital for realistic simulations of hemodynamic properties in the context of Particle Image Velocimetry measurements [33] and device behavior, particularly in the view of different variations of the CoW [24].

This manuscript introduces a novel technique for creating patient-specific replicas of the entire CoW encased within a transparent silicone block. The key innovation lies in the simultaneous 3D printing of both the vascular structure and its surrounding casting enclosure using fused deposition modeling (FDM) with ASA and BVOH filaments. This study presents a technique designed to ensure the geometric stability and detail of vessels and aneurysms. Additionally, it aims to simplify the manufacturing process for entire CoW replicas. We hypothesize that this manufacturing process for replicas of the CoW will yield accurate and reproducible replicas. To evaluate the accuracy and reproducibility of the manufacturing process, three identical silicone replicas of the CoW were fabricated using patient-specific data with four aneurysms. The agreement between these replicas and the initial model was assessed by generating 3D models from high-resolution CT-scans of the replicas and comparing them to the initial 3D model.

## Methods

### Segmentation and 3D model generation

The silicone replicas were generated from patient-specific DICOM images acquired using TOF-MRA. The DICOM images obtained through TOF-MRA featured a spatial resolution of 0.3125 mm, a slice thickness of 1 mm and a spacing between slices of 0.5 mm. Ethical approval was obtained from the Institutional Review Board of the University Hospital RWTH Aachen (EK 257/20). Written informed consent was waived by the Institutional Review Board. The data for this retrospective study were accessed for research purposes between 04.01.2021 and 30.06.2021. While the authors had access to information that could identify individual participants, this information was not relevant to the study’s objectives and did not influence the analysis or results. Segmentation of the CoW was performed using the software 3D Slicer (https://www.slicer.org/) [34] by applying an intensity-threshold function to the DICOM images. Vessels that did not belong to the CoW or were smaller than 1 mm were not included in the model. Surface smoothing was carried out utilizing Chebyshev polynomials before the segmented patient scan was exported as a 3D model.

**Fig 1:**
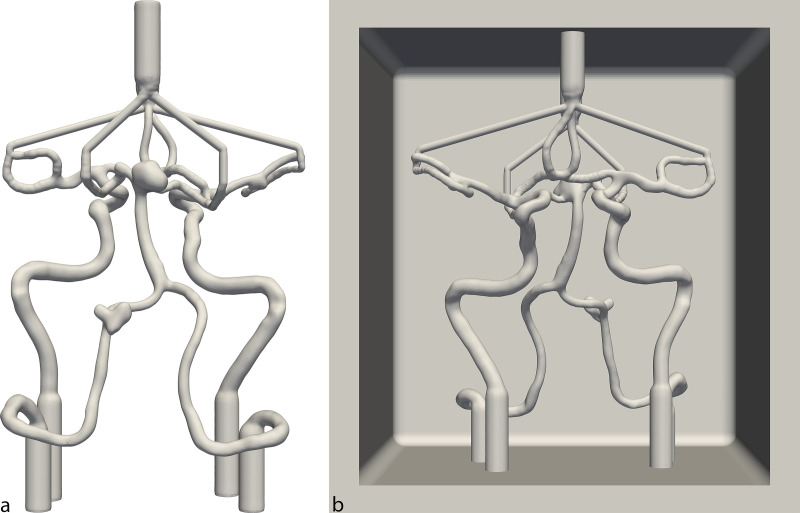
Patient specific 3D Model of the CoW. (a) Posterior view of the segmented CoW derived from patient-specific TOF-MRA data, showing inflow and outflow ports. (b) Anterior view of finalized 3D printing model, consisting of the segmented CoW connected to the cuboid enclosure. CoW: Circle of Willis, TOF-MRA: Time-of-flight magnetic resonance angiography.

The 3D model included key vessels of the CoW, such as the vertebral arteries (VA), the basilar artery (BA), the posterior cerebral arteries with P1 and P2 segments, the internal carotid artery (ICA), the middle cerebral arteries (MCA) with M1 and M2 segments, the anterior cerebral arteries with A1 and A2 segments, as well as the anterior communicating artery. The CoW model exhibits four patient-specific saccular aneurysms located at the BA head, the left VA, the right carotid-T and the right MCA bifurcation. The initial patient-specific 3D model of the CoW is shown in Fig 1a. In preparation for the casting process, the model was imported into the software Meshmixer (Autodesk, USA), where minor artifacts were removed, surface holes were closed and smoothing operations were applied to ensure printability and structural continuity;inflow and outflow ports were also integrated at this stage, while preserving the original geometry of the CoW. This initial 3D model acted as the reference standard for evaluating the accuracy of the final silicone replicas. For the casting process, the 3D model was extended with a cuboid casing, designed to be open on one side, with the inlets and outlets of the model connected to the cuboid’s walls. The 3D model of the CoW with its cuboid casing, which was used for printing, is shown in Fig 1b. The 3D model of the cuboid was generated using the software Autodesk Inventor Professional 2022 (Autodesk, USA).

### 3D printing

**Fig 2:**
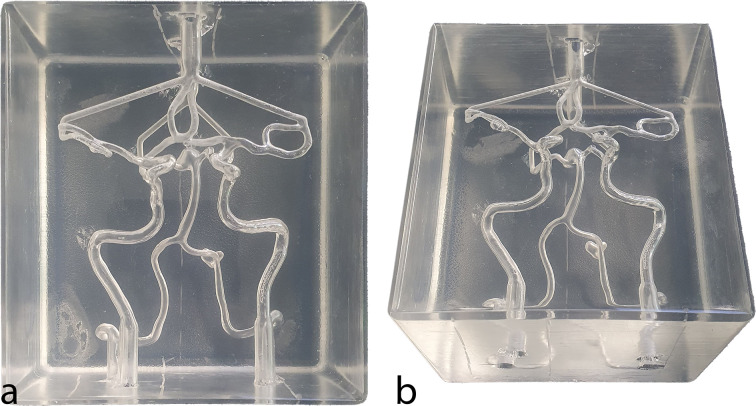
Silicone replica of the patient-specific CoW including inflows and outflows. (a) Anterior perspective. (b) Anterior-caudal perspective. CoW: Circle of Willis.

The GCODE file, containing the commands required for printing, was prepared using the software PrusaSlicer 2.7.2 (Prusa Research, Czech Republic) and printed on the fused deposition 3D printer Prusa i3 MK3S+ (Prusa Research, Czech Republic) equipped with Multi-Material Upgrade 2 (MMU2) (Prusa Research, Czech Republic). BVOH filament (Verbatim, USA) was utilized as support structures due to its water solubility. ASA filament (Prusament ASA Natural, Prusa Research, Czech Republic) was employed for the CoW model and cuboid, chosen for its solubility in acetone. All used filaments for printing had a diameter of 1.75 mm. The layer height was set to 200 µm and a nozzle with a 400 µm diameter was used for the printing process. After printing, the support structures were dissolved in water and thoroughly rinsed to ensure no BVOH residues remained. The remaining ASA surface of the CoW model and the interior of the cuboid were smoothed using acetone. The smoothed 3D print was then filled with clear silicone (Solaris™, Smooth-On, USA). To prevent the entrapment of air bubbles in the two-component mixture, the silicone was degassed under vacuum before casting. After the 24-hour curing time, the silicone cast was demolded by cutting away the cuboid walls and dissolving the CoW structure within the silicone cuboid in an acetone bath. This process necessitated rinsing to eliminate any ASA residue within the silicone model, resulting in a cuboid silicone block that replicates the patient-specific vascular lumens of the CoW as depicted in Fig 2.

### Evaluation

All three replicas were examined using a photon-counting CT (NAEOTOM Alpha, Siemens, Germany), whereby the vessel lumen was filled with air. The imaging was conducted with a slice thickness of 0.2 mm and a spatial resolution of approximately 0.2 mm. The obtained DICOM images were again segmented with 3DSlicer to generate a 3D model of the vessel lumen. For the evaluation, the segmented silicone replicas (R1, R2, R3) were co-registered to the segmented patient scan (R0) using an iterative closest point algorithm in the software CloudCompare 2.12.4 (CloudCompare, Ukraine). To quantify the geometric deviation between replicas and the initial model, the minimum Euclidean distance from each point of the replica surface to the nearest point on the surface of the initial model was determined using the Cloud-to-Mesh Distance calculation in CloudCompare 2.12.4. The absolute median of the minimum Euclidean distances was calculated. The deviation was determined for the entire CoW and separately for the surfaces of the four aneurysms. The data from the Cloud-to-Mesh Distance calculation are provided in Supporting Information S1 C2M CoW and S2 C2M Aneurysms.

Centerlines were created for crucial vessel pathways for each model using the software Vascular Modeling Toolkit (https://www.vmtk.org) in 3DSlicer, with the radius along the centerline acquired at 1 mm increments. Fig 4 shows the course of the four generated centerlines (in orange) through key sections of the CoW: from the right vertebral artery to the top of the basilar artery (labeled as C-VR), from the left vertebral artery to the left posterior cerebral artery (labeled as C-VL), from the left ICA to the anterior cerebral artery-A2 segment (labeled as C-CL) and from the right ICA to the aneurysm at the MCA bifurcation (labeled as C-CR). The median absolute deviation between the centerlines of the silicone replicas and the initial model was determined. The data from the centerline analysis are provided as Supporting Information S3 Centerlines. Additionally, a Wilcoxon signed-rank test was conducted to test for significant differences between the centerline radii of the initial model and the replicas. The statistical analyses were performed using the software JASP 0.18.3 (JASP Team, 2024).

**Fig 3:**
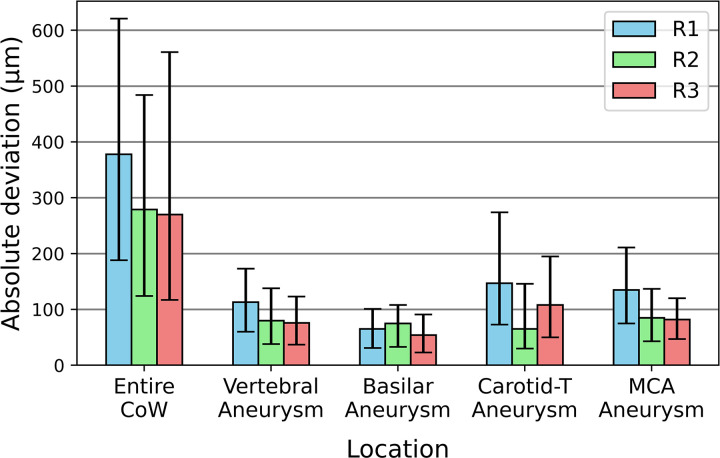
Median absolute deviation between the surfaces of the replicas (R1-R3) and the segmented patient vasculature (R0). The deviations are analyzed across the entire CoW and the areas of the aneurysms. The bars represent the median absolute deviation for each model, with error bars indicating the interquartile ranges. CoW: Circle of Willis. All dimensions are given in µm.

## Results

### Geometric accuracy

The geometric accuracy of the silicone replicas was evaluated by comparing the segmented models obtained from CT scans of the replicas with the initial patient-specific 3D model. The median absolute surface deviation for the entire CoW was 309 µm across all three replicas (R1, R2 and R3), with IQR between 360 µm and 444 µm. The highest deviation occurred in R1 (378 µm), while R2 and R3 showed slightly lower median absolute deviations (279 µm and 270 µm).

For the individual aneurysms, the surface deviation was lower. The VA aneurysm exhibited an average deviation of 90 µm across the three models, while the BA aneurysm showed an average deviation of 65 µm. The carotid-T aneurysm and MCA bifurcation aneurysm had slightly higher deviations, with average values of 107 µm and 101 µm, respectively. Detailed statistics for each model and aneurysm are summarized in Table 1 and Fig 3, which illustrate the median absolute errors and IQR for the CoW and the selected aneurysms.

**Table 1:** Median absolute deviation of the comparison between the surfaces of the replicas (R1-R3) and the segmented patient vasculature (R0) for the entire CoW and the four aneurysms of the model.

Compared surface	Replica	Median absolute deviation to R0	Q1	Q3	(µm)
Entire CoW	R1	378	188	621	
	R2	279	124	484	
	R3	270	117	561	
Vertebral aneurysm	R1	113	60	173	
	R2	80	38	138	
	R3	76	37	123	
Basilar aneurysm	R1	65	31	101	
	R2	75	33	108	
	R3	54	23	91	
Carotid-T aneurysm	R1	147	73	274	
	R2	65	30	146	
	R3	108	50	195	
MCA aneurysm	R1	135	75	211	
	R2	85	43	137	
	R3	82	47	120	

The table includes the first (Q1) and third (Q3) quartiles for each dataset. All dimensions are given in µm.

**Fig 4:**
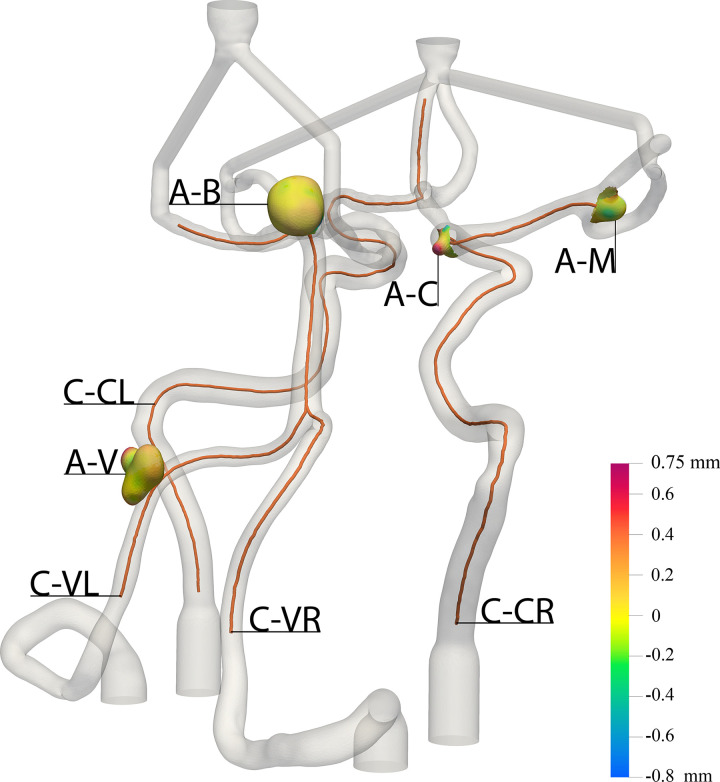
Course of the four evaluated centerlines and heat maps for the deviation in the area of the aneurysms overlaid on the segmented patient vasculature. The orange lines represent the centerlines for: (C-VR) right VA to the top of the BA; (C-VL) left VA to the left posterior cerebral artery; (C-CL) left ICA to the anterior cerebral artery-A2; and (C-CR) right ICA to the small aneurysm at the MCA bifurcation. Heat maps visualize the geometric deviations between R0 and R3 for the surfaces of the aneurysms. (A-V) represents the aneurysm at the left VA, (A-B) highlights aneurysm at the top of the BA, (A-C) shows the aneurysm at the right carotid-T and (A-M) depicts the aneurysm at the right MCA bifurcation. CoW: Circle of Willis, VA: vertebral artery, ICA: internal carotid artery, BA: basilar artery, MCA: middle cerebral artery. All dimensions are given in mm.

A 3D model of the CoW, supplemented by heat maps as shown in Fig 4, visually represents the deviations between R0 and R3 based on the cloud-to-mesh calculations. The heat maps for the aneurysms mostly show small deviations, with some regions showing larger discrepancies, with deviations of up to 800 µm.

**Fig 5:**
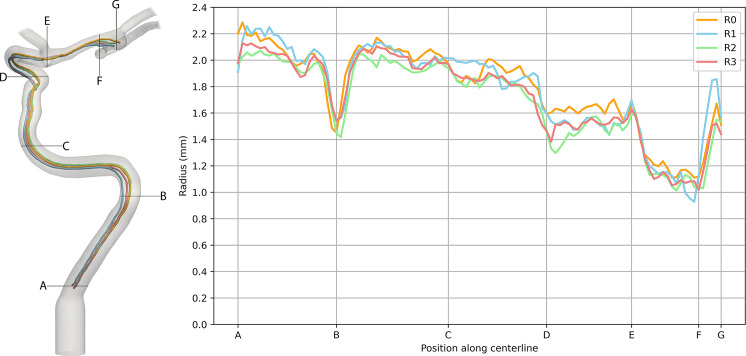
Exemplary results from the centerline analysis. The left side shows the 3D model of the right ICA with the four generated centerlines leading from to the small aneurysm at the MCA bifurcation. The patient’s centerline is displayed in orange, while the centerlines of replicas 1,2 and 3 are shown in blue, green and red, respectively. The right side illustrates the comparison of the radii of the four models from point A to G. ICA: internal carotid artery, MCA: middle cerebral artery.

Centerlines were generated to assess the geometric congruence between the segmented silicone replicas and the initial 3D model. Fig 5 provides a detailed example of the centerline analysis for the right ICA. On the left, the 3D model displays the centerline for R0 (orange) alongside the generated centerlines for R1, R2 and R3 (blue, green, red). Key points along the centerline path, labeled A to G, highlight critical anatomical features and changes in vessel geometry. The right side of Fig 5 shows the radii of the four models from point A to G, illustrating the deviations along the centerline.

From the start of the centerline (Point A) to the end (Point G), the deviations are small, with a maximum variation of 340 µm and an average median absolute deviation of 78 µm for the three replicas compared to R0. At Point B, a local narrowing of the ICA is observed for all four centerlines, likely due to vessel curvature. Moving from Point B to D via C, the radius gradually decreases, from approximately 2 mm at Point C to 1.6 mm for Models R0 and R1, while Models R2 and R3 show a slightly smaller radius of 1.4 mm at Point D. At Point E, a slight increase in radius occurs at the junction with the anterior cerebral artery due to vessel crossing. Following this, the radius sharply decreases toward Point F in all models. Near Point F, in the aneurysm, the radius increases again before decreasing toward Point G, reflecting the rounded shape of the aneurysm.

The median absolute deviations in radius along the centerlines between R1-R3 and R0 are summarized in Table 2. For the ICA, the median absolute deviations ranged from 67 µm to 97 µm across the models, with the left ICA showing slightly smaller deviations. Similarly, the vertebral arteries exhibited median deviations ranging from 48 µm to 114 µm, with the highest deviations in the right vertebral artery for R2.

**Table 2:** Median absolute deviation in radius along various centerlines for all replicas (R1, R2 and R3) compared to the initial 3D model (R0).

Centerline Course	Replica (µm)	Median absolute deviation to R0 (µm)	Q1 (µm)	Q3 (µm)	p-value
Right carotid artery to MCA aneurysm	R1	67	28	118	0.16
	R2	97	67	136	< 0.001
	R3	70	37	106	< 0.001
Left carotid artery to A2 anterior cerebral artery	R1	56	34	101	0.022
	R2	73	47	115	< 0.001
	R3	65	29	128	< 0.001
Right vertebral artery to basilar artery aneurysm	R1	61	28	114	0.081
	R2	114	90	150	< 0.001
	R3	63	44	88	< 0.001
Left vertebral artery to posterior cerebral artery	R1	48	19	105	0.714
	R2	98	48	155	< 0.001
	R3	56	24	119	0.006

The table includes the first (Q1) and third (Q3) quartiles for each dataset. All dimensions are given in µm. p-values were calculated using the Wilcoxon signed-rank test comparing each replica to the initial model (R0). Statistical significance was considered at p < 0.05.

Although Fig 5 shows a good overlay of the centerlines and Table 2 only demonstrates only small median absolute deviations between the centerline radii, the Wilcoxon signed-rank revealed test for most replicas (R2 and R3 across all centerlines and R1 in one centerline), the vessel radius of the initial model R0 was significantly larger (p < 0.05).

However, the actual difference remained minor with a median radius of 1.61 mm for the R0 across all centerlines and a median radius of 1.52 mm across all replicas and centerlines, corresponding to a median difference of only 0.09 mm. Furthermore, both distributions exhibit remarkable consistency as reflected by similar IQR (1.23–1.95 mm for R0 vs. 1.17–1.92 mm for the replicas. Detailed data are provided in Table 3.

**Table 3:** Median radius along all centerlines for the initial model (R0) and the three replicas (R1, R2 and R3), along with the median radius for the combined average of all replicas.

Model	Median radius	Q1	Q3	(mm)
R0	1.61	1.23	1.95	
R1	1.56	1.21	1.99	
R2	1.47	1.13	1.89	
R3	1.52	1.18	1.91	
Average of R1, R2, R3	1.52	1.17	1.92	

The table also includes the first (Q1) and third (Q3) quartiles for each dataset. All measurements are given in mm.

### Silicone replica production: timeline, costs and mechanical properties

The production of a single silicone replica of the entire CoW, as shown in Fig 2, required a total of 5 days to complete. The timeline includes 44 hours of 3D printing, one day for dissolving of the support material, the surface smoothing and the silicone casting, one day for the silicone curing and one additional day to dissolve the ASA core. The material costs for the 3D-printed model included approximately €10 for the ASA filament and €35 for the BVOH filament. Due to the large casting volume, the silicone represented the most significant expense. With dimensions of 11 cm x 10 cm x 7.5 cm (825 mL) and a rate of €0.06 per mL for silicone, the cost amounted to €49.50. Approximately one liter of Acetone was used to dissolve the ASA core, adding around €5 to the total, bringing the overall material cost to approximately €100.

The replicas were cast using clear, transparent silicone; however, complete clarity was only achieved on the upper side. The sides in contact with the walls during curing remained slightly opaque. The replicas therefore demonstrated overall good visual as well as mechanical properties, including slight pulsation when connected to a pulsatile pump.

## Discussion

This study presents a novel and cost-effective manufacturing approach for full-scale, patient-specific CoW models using FDM and lost core silicone casting. A central innovation of the presented method lies in the simultaneous FDM printing of both the vascular structure and its surrounding casting enclosure. This direct-print approach simplifies the lost-core casting process, eliminates the need for separate mold fabrication and improves reproducibility. To our knowledge, this integrated workflow has not been applied to full-scale, patient-specific CoW models including aneurysms and represents a practical and cost-efficient solution for vascular replication. The comparison between the CT-generated 3D models of the silicone replicas and the patient-specific initial model showed a strong geometric similarity, indicating that the approach can reliably reproduce individual anatomical features.

Vessel analysis revealed a generally good agreement between the replicas and the initial model. This is evident in Fig 5, where all four curves closely follow each other, even in tortuous segments, bends and bifurcations, with a maximum deviation of just 340 µm between the initial model and the replicas. Additionally, the median deviations in the centerline analysis, which range from 48 µm to 114 µm, indicate that the silicone models accurately preserved the vascular geometry.

Our results are comparable to those of Falk, Medero and Roldán-Alzate (2019), who performed centerline analysis on silicone models consisting of an aneurysm and the adjacent vessel. Falk et al. reported a mean deviation in the calculated circumference of 0.6 mm, which corresponds to a deviation in radius of 95 µm. Although their method involved calculating the circumference, our results in Table 2, based on the radius, show mostly smaller median deviations ranging from 48–114 µm, underscoring the increased accuracy of our proposed process. Despite the Wilcoxon signed-rank test indicating a statistically significant difference between the initial model and most replicas, the observed differences in vessel radius were small. When analyzed individually, the median radius of the initial model (R0) was 1.61 mm, while the median radii of the replicas ranged from 1.47 mm (R2) to 1.56 mm (R1). To assess the overall deviation across all replicas, we also calculated the median radius of the combined replica data (R1–R3), which resulted in an average value of 1.52 mm. This corresponds to a difference of only 0.09 mm compared to the initial model. Furthermore, the interquartile ranges (IQR) were highly comparable, with 1.23–1.95 mm for R0 and 1.17–1.92 mm for the averaged replicas. These findings suggest that the actual discrepancies between the models are minimal. Nevertheless, these deviations must be interpreted with caution when considering clinical scenarios. In neurovascular interventions, even small dimensional inaccuracies can affect the performance of flow diverters and compromise treatment efficacy, as slight malpositioning have been shown to alter intra-aneurysmal hemodynamics and potentially inhibit thrombus formation [35].

This is further shown when compared with other studies, such as Chivukula et al. (2019) [36], who used cloud-to-mesh comparisons to assess the dimensional accuracy of their patient-specific aneurysm models. Chivukula et al. (2019) reported a mean IQR of 226 µm surface deviation for six patient geometries for individual vascular segments. Our cloud-to-mesh analysis revealed an average IQR of surface deviations for the entire CoW of 390 µm. This comparison is particularly notable, as Chivukula et al.‘s models represent only a single vessel branch with an aneurysm, whereas our model represents the entire CoW with multiple aneurysms.

Aneurysm analysis demonstrated particularly consistent reproduction of aneurysm morphology across all replicas, showing an average median deviation of only 90 µm. This precision is noteworthy when compared to the previously mentioned study by Falk, Medero and Roldán-Alzate (2019), which reported a mean absolute geometric surface deviation of 300 µm across an aneurysm. These small deviations, especially at aneurysm sites, demonstrate that our method preserves intricate vascular geometries with high accuracy. This precision is essential for reliable hemodynamic simulation and the whole CoW, where factors like vessel radius and curvature have a profound impact on WSS and hemodynamics (Meng et al. 2014; Cebral et al. 2015) or on the behavior and efficacy of medical devices [37].

The larger deviation observed in the entire model compared to the aneurysms alone can be attributed to the increased complexity and surface area of the entire CoW structure. The complexity makes it more challenging to precisely overlay the segmented scans for comparison, further contributing to the overall deviation. Nevertheless, the small deviations show that the replicas accurately capture the geometry of both the CoW and the aneurysms, as further reflected in the low IQR, particularly for the aneurysms.

In addition to geometric accuracy, the material properties of the silicone contribute to the functional fidelity of our replicas. The compliance properties of Solaris™ [38], allow the vessels, when connected to a pulsatile pump, to exhibit pulsatile behavior despite the large wall thickness. Additionally, the transparency of Solaris™ makes it ideal for Particle Image Velocimetry measurements, enabling detailed hemodynamic analysis [39].

Simplifying the manufacturing process by directly 3D printing the lost core in combination with the model enclosure using ASA and water-soluble supports eliminate several production steps. Our approach bypasses the need for creating a separate enclosure to house the CoW print [39,40] and avoids the additional step of generating a wax replica for casting [41]. This process allowed us to achieve low material costs of €100 per replica, despite the large amount of silicone used. In comparison, Falk et al. reported material costs of $25–28 for smaller models replicating branches of the CoW using PVA filament and Solaris™ silicone. However, their approach required the manual construction of an acrylic casting box, which was not included in the reported cost estimate and introduces an additional fabrication step. While the replicas of Falk et al. required four days to complete, our production process took five days, primarily due to the longer printing time associated with filament changes on the Prusa i3 MK3S+ with MMU2. However, this could be optimized in future by using a dual-nozzle 3D printer, which would eliminate the need for manual filament swaps and potentially reduce both material waste and overall print time by up to 50%, thereby increasing efficiency and cost-effectiveness. The manufacturing process presented here can also be readily adapted to produce both smaller and larger vascular models.

While cost and simplicity favor FDM for the lost core approach, SLA offers higher resolution and surface fidelity, which may be beneficial for replicating finer anatomical details. However, SLA typically requires more complex post-processing and may be less robust when printing large, hollow structures intended for casting. Future work should investigate whether the reproducibility and accuracy observed with FDM can be matched or improved using SLA in comparable full-scale vascular models.

While the replicas demonstrated a high degree of geometric fidelity to the initial model, several limitations of the underlying imaging and processing pipeline must be considered. The original 3D model was based on TOF-MRA imaging, which has been shown to introduce inaccuracies, particularly in curved vessel segments and smaller caliber arteries [42,43]. Furthermore, although care was taken during mesh post-processing to preserve the anatomical geometry, the use of smoothing and repair operations in Meshmixer may have introduced minor deviations from the original data.

Another limitation lies in the imaging modality used for validating the replicas. While photon-counting CT offers superior spatial resolution compared to TOF-MRA, it is not directly comparable to 3D rotational angiography (3DRA), which is widely regarded as the gold standard for vascular imaging due to its excellent spatial and contrast resolution. However, 3DRA requires the injection of contrast agent under flow conditions and is typically limited to targeted vascular regions, making it challenging to image the entire Circle of Willis in a single acquisition. This reduces its suitability for in vitro validation of full-scale vascular models, where a comprehensive and static imaging approach—such as photon-counting CT, where air-filled lumens provide sufficient contrast—offers practical advantages. These methodological considerations should be taken into account when interpreting the reported geometric deviations.

While this study focused on the geometric accuracy and reproducibility of replicas derived from a single patient dataset, it did not include a comparison with alternative segmentation or modeling pipelines. Including such control methods—e.g., by re-segmenting the same data using different tools or protocols—could further validate the robustness and generalizability of our approach. Future work should therefore incorporate additional segmentation strategies or modeling workflows to benchmark deviations and evaluate the consistency of outcomes across different methods.In addition, future investigations should include a systematic comparison between FDM and stereolithography (SLA) printing techniques. Although FDM offers practical advantages in support material handling, SLA may yield benefits in surface resolution and anatomical detail reproduction. Previous studies have demonstrated the high geometric accuracy of SLA-based vascular models, as shown by previous studies [29].

With the rising application of 3D printing in medical training [31] and pre-operative planning [30], the development of cheap and practical production methods is both necessary and highly beneficial, while providing a high degree of accuracy. Our proposed method employs FDM printing and the lost core process to cheaply and reliably replicate full-scale CoW models from real patient data.

## Conclusion

We have developed a novel and cost-effective manufacturing approach for creating accurate, full-scale, patient-specific CoW models that include four aneurysms. By utilizing FDM 3D printing and lost core silicone casting, we produced replicas with geometric fidelity, as evidenced by minimal deviations in both surface and centerline analyses. This method simplifies the production process, reduces costs and maintains geometric accuracy, making it suitable for hemodynamic studies, device evaluation and clinical training. While the current work focused on validating the manufacturing process in terms of accuracy and reproducibility, the method lays a strong foundation for future applications in medical education and neurointerventional training, where anatomically accurate vascular models are essential for simulating complex endovascular procedures. The ability to accurately replicate complex vascular geometries holds significant potential for advancing neuroendovascular research and improving clinical outcomes.

## Supporting information

S1 C2M CoW Surface deviation data from cloud-to-mesh distance calculations for the entire Circle of Willis.
(CSV)

S2 C2M Aneurysms Surface deviation data from cloud-to-mesh distance calculations focused on the four aneurysm regions.
(PDF)

S3 Centerlines Centerline radius measurements for all models (R0–R3) across four key vascular paths (C-VR, C-VL, C-CL, C-CR).
(PDF)
