# Supplementary material for: Accurate full-scale patient-specific Circle of Willis models including aneurysms: A novel manufacturing approach
Source: PLoS One. 2025 Jul 17;20(7):e0328300. doi: 10.1371/journal.pone.0328300 (PMC12270141; doi:10.1371/journal.pone.0328300)
Supplement: S3 Centerlines — Centerline radius measurements for all models (R0–R3) across four key vascular paths (C-VR, C-VL, C-CL, C-CR). (PDF) [file pone.0328300.s003.pdf]

Centerline	id	X	Y	Z	radius_R0	radius_R1	radius_R2	radius_R3	abs_diff_R0_R1	abs_diff_R0_R2	abs_diff_R0_R3
Carotis_L	1	42,877	-85,870	-992,728	2,1306517526	2,0815323840	2,0080421593	2,0483295596	0,0491193686	0,1226095933	0,0823221930
Carotis_L	2	42,850	-85,687	-991,705	2,1025186848	2,0973600931	1,9169544249	2,0333325266	0,0051585917	0,1855642599	0,0691861582
Carotis_L	3	42,823	-85,666	-990,680	2,0612665097	2,0742279665	1,9667987585	2,0553589969	0,0129614568	0,0944677512	0,0059075128
Carotis_L	4	42,823	-85,623	-989,644	2,0639775633	2,0485451887	1,9623686904	2,0280273777	0,0154323746	0,1016088730	0,0359501856
Carotis_L	5	42,848	-85,404	-988,632	2,0831923028	2,0100597446	1,9260945915	1,9995334480	0,0731325581	0,1570977113	0,0836588547
Carotis_L	6	42,937	-85,289	-987,609	2,0747285269	2,0574698058	1,9785884211	2,0216266564	0,0172587211	0,0961401058	0,0531018705
Carotis_L	7	43,055	-85,148	-986,590	2,0871142104	2,0463790287	1,9865386824	2,0431180729	0,0407351817	0,1005755281	0,0439961376
Carotis_L	8	43,205	-85,007	-985,582	2,0593508711	2,0860441026	1,9858158387	1,9949058787	0,0266932315	0,0735350324	0,0644449925
Carotis_L	9	43,496	-84,812	-984,607	2,0344628037	2,0684227081	1,9803193538	1,9855702694	0,0339599044	0,0541434499	0,0488925343
Carotis_L	10	43,813	-84,666	-983,636	2,0447590651	2,0846743033	1,9690323532	1,9997080841	0,0399152382	0,0757267119	0,0450509810
Carotis_L	11	44,175	-84,642	-982,672	2,0807945163	2,0246488559	1,9541169046	1,9889409790	0,0561456604	0,1266776118	0,0918535374
Carotis_L	12	44,660	-84,545	-981,774	2,0802427122	2,0464746773	1,9825606936	1,9991726662	0,0337680349	0,0976820187	0,0810700461
Carotis_L	13	45,108	-84,361	-980,868	2,0758409354	2,0498306891	1,9606402693	2,0331331425	0,0260102462	0,1152006661	0,0427077928
Carotis_L	14	45,611	-84,226	-979,976	2,0853506732	2,1081727288	1,9846326735	2,0193800892	0,0228220555	0,1007179997	0,0659705841
Carotis_L	15	46,099	-84,166	-979,068	2,0848068081	2,1482238427	2,0377146051	2,0789353272	0,0634170346	0,0470922030	0,0058714809
Carotis_L	16	46,614	-84,099	-978,177	2,1000451810	2,1023456698	1,9983453450	2,0255387065	0,0023004888	0,1016998360	0,0745064745
Carotis_L	17	47,150	-83,996	-977,299	2,0656864485	2,1365809634	2,0157464024	2,0816233960	0,0708945149	0,0499400461	0,0159369475
Carotis_L	18	47,626	-83,853	-976,392	2,0439530077	2,0834222706	1,9957393910	2,0346909843	0,0394692629	0,0482136167	0,0092620234
Carotis_L	19	48,098	-83,686	-975,491	2,0669620197	2,0747228442	1,9571830187	2,0214342811	0,0077608245	0,1097790010	0,0455277386
Carotis_L	20	48,490	-83,380	-974,596	2,0661465139	2,0952953943	1,9288440641	2,0162647340	0,0291488804	0,1373024498	0,0498817799
Carotis_L	21	48,826	-82,975	-973,706	1,9700665533	2,0927220650	1,9516511268	2,0232654013	0,1226555117	0,0184154264	0,0531988481
Carotis_L	22	49,059	-82,534	-972,806	1,9128728157	2,0957575945	1,9648179275	1,9736776625	0,1828847787	0,0519451118	0,0608048468
Carotis_L	23	49,308	-82,338	-971,861	1,8600197134	1,9926334552	1,8975829417	1,8606717084	0,1326137418	0,0375632283	0,0006519950
Carotis_L	24	49,406	-82,264	-970,843	1,7388476468	2,0069326018	1,7854898436	1,8138985577	0,2680849550	0,0466421968	0,0750509109
Carotis_L	25	49,471	-82,554	-969,871	1,6121082413	1,9168179571	1,6371831665	1,7907960720	0,3047097158	0,0250749253	0,1786878307
Carotis_L	26	49,483	-82,831	-968,880	1,6206552343	1,8506685879	1,5600646045	1,6856215130	0,2300133536	0,0605906298	0,0649662787
Carotis_L	27	49,532	-83,207	-967,921	1,7415257563	1,7784154522	1,5513872525	1,5812305913	0,0368896959	0,1901385038	0,1602951650
Carotis_L	28	49,373	-83,727	-967,043	1,8875442176	1,8437922935	1,5868183337	1,6382483434	0,0437519241	0,3007258838	0,2492958742
Carotis_L	29	49,020	-84,258	-966,247	1,9803173047	1,9633427832	1,6973971046	1,7481765765	0,0169745215	0,2829202001	0,2321407282
Carotis_L	30	48,379	-84,770	-965,632	1,9950791697	2,0301917269	1,9069441612	1,8724376670	0,0351125572	0,0881350086	0,1226415028
Carotis_L	31	47,659	-85,401	-965,231	2,0910745309	2,0845569786	2,0526023353	1,9493866774	0,0065175523	0,0384721956	0,1416878534
Carotis_L	32	46,892	-85,995	-964,927	2,1746569037	2,1647453845	2,1096416972	2,0103115869	0,0099115192	0,0650152065	0,1643453169

Carotis_L	33	46,050	-86,614	-965,044	2,3266467589	2,2168922597	2,1763746502	2,0937345817	0,1097544992	0,1502721087	0,2329121772
Carotis_L	34	45,171	-87,153	-965,073	2,3603719458	2,3543702745	2,2407788417	2,2320670784	0,0060016713	0,1195931041	0,1283048674
Carotis_L	35	44,334	-87,747	-965,152	2,2616800927	2,3576753029	2,2547277372	2,3178593575	0,0959952102	0,0069523554	0,0561792648
Carotis_L	36	43,470	-88,305	-965,212	2,1393932610	2,2455180698	2,1624787865	2,2946705886	0,1061248089	0,0230855256	0,1552773277
Carotis_L	37	42,578	-88,822	-965,336	2,1098067534	2,1854245511	2,0923586234	2,1921237425	0,0756177977	0,0174481300	0,0823169891
Carotis_L	38	41,671	-89,312	-965,429	2,1202108614	2,0996113798	2,0603109066	2,1177602205	0,0205994816	0,0598999549	0,0024506409
Carotis_L	39	40,797	-89,851	-965,520	2,1010421813	2,1408908911	2,0535272605	2,0742894914	0,0398487099	0,0475149208	0,0267526899
Carotis_L	40	39,914	-90,378	-965,588	2,0608285931	2,1228219611	2,0574879406	2,0630415305	0,0619933680	0,0033406525	0,0022129374
Carotis_L	41	39,047	-90,931	-965,654	2,0381154629	2,1599659895	1,9988022520	2,0277113004	0,1218505266	0,0393132110	0,0104041625
Carotis_L	42	38,165	-91,464	-965,755	2,0243816407	2,1051510629	1,9469036952	1,9920513299	0,0807694223	0,0774779455	0,0323303108
Carotis_L	43	37,286	-91,995	-965,833	1,9860060824	2,1452309183	1,9114119874	1,9717920866	0,1592248359	0,0745940950	0,0142139958
Carotis_L	44	36,385	-92,499	-965,816	1,9763753774	2,1052389227	1,8888839184	1,9390824924	0,1288635452	0,0874914591	0,0372928851
Carotis_L	45	35,490	-93,021	-965,760	1,9546257150	2,0554184151	1,9099428946	1,9369388124	0,1007927001	0,0446828204	0,0176869026
Carotis_L	46	34,601	-93,544	-965,648	1,9340581703	2,0653493202	1,8856080618	1,9384200187	0,1312911499	0,0484501085	0,0043618484
Carotis_L	47	33,686	-93,980	-965,453	1,9352022070	2,0012343447	1,8948601235	1,8924615157	0,0660321378	0,0403420835	0,0427406913
Carotis_L	48	32,760	-94,337	-965,162	1,9487033712	1,9854704549	1,8779100410	1,8720768880	0,0367670838	0,0707933302	0,0766264832
Carotis_L	49	31,852	-94,568	-964,748	1,9328758956	2,0176584808	1,8788487194	1,9051648546	0,0847825852	0,0540271762	0,0277110410
Carotis_L	50	31,018	-94,702	-964,164	1,8976897556	1,9970203835	1,8826077978	1,9230439328	0,0993306278	0,0150819578	0,0253541772
Carotis_L	51	30,237	-94,832	-963,504	1,8907416452	1,9765371803	1,8531400404	1,9274019571	0,0857955351	0,0376016048	0,0366603119
Carotis_L	52	29,567	-94,877	-962,729	1,8995016891	1,9604284587	1,8429158327	1,9096131842	0,0609267696	0,0565858564	0,0101114951
Carotis_L	53	29,059	-94,863	-961,826	1,8454806577	1,9167635070	1,8252357115	1,9182259436	0,0712828492	0,0202449463	0,0727452858
Carotis_L	54	28,773	-94,898	-960,842	1,7573763997	1,8540896950	1,8040017566	1,9118181300	0,0967132953	0,0466253569	0,1544417303
Carotis_L	55	28,575	-95,009	-959,836	1,7720169495	1,8136441829	1,7613233858	1,8442353346	0,0416272334	0,0106935637	0,0722183851
Carotis_L	56	28,453	-95,033	-958,807	1,7891242676	1,7705128136	1,7448658859	1,7976345033	0,0186114540	0,0442583817	0,0085102357
Carotis_L	57	28,383	-95,188	-957,797	1,7940173047	1,7647361087	1,7288986249	1,7849637543	0,0292811960	0,0651186798	0,0090535504
Carotis_L	58	28,585	-95,417	-956,814	1,7965688259	1,7913604836	1,7436676476	1,7728786621	0,0052083423	0,0529011782	0,0236901637
Carotis_L	59	28,670	-95,435	-955,787	1,7853230135	1,7421989537	1,7207710974	1,7460198771	0,0431240598	0,0645519161	0,0393031364
Carotis_L	60	28,881	-95,366	-954,784	1,8423370306	1,7171346916	1,6952499786	1,7282859248	0,1252023389	0,1470870519	0,1140511057
Carotis_L	61	29,208	-95,351	-953,817	1,8139284222	1,7509030354	1,7253806994	1,7505639446	0,0630253868	0,0885477228	0,0633644776
Carotis_L	62	29,497	-95,173	-952,850	1,9236408590	1,8560306093	1,7002442375	1,8081456229	0,0676102498	0,2233966216	0,1154952361
Carotis_L	63	29,897	-95,358	-951,909	1,9906163631	1,8814649606	1,7271608323	1,8292177072	0,1091514025	0,2634555307	0,1613986558
Carotis_L	64	30,368	-95,764	-951,084	2,0876426758	1,9950531467	1,8587488991	1,8522673358	0,0925895291	0,2288937767	0,2353753400
Carotis_L	65	30,660	-96,472	-950,392	2,1396207649	2,0776651182	2,0222886870	1,9778756225	0,0619556467	0,1173320779	0,1617451424

Carotis_L	66	30,730	-97,359	-949,882	2,1431891392	2,1488641801	2,0754337989	2,0812396446	0,0056750409	0,0677553404	0,0619494946
Carotis_L	67	30,837	-98,361	-949,686	2,0765696695	2,0797673170	2,0475789835	2,1070474330	0,0031976474	0,0289906861	0,0304777635
Carotis_L	68	30,874	-99,393	-949,606	2,0040395170	1,9390978058	1,9673068880	2,0756244717	0,0649417112	0,0367326290	0,0715849547
Carotis_L	69	30,889	-100,425	-949,608	1,9487813188	1,9057919014	1,8877270738	1,9512756720	0,0429894174	0,0610542450	0,0024943532
Carotis_L	70	30,794	-101,442	-949,756	1,9001890773	1,8785270594	1,8072841037	1,9194263855	0,0216620179	0,0929049735	0,0192373082
Carotis_L	71	30,736	-102,462	-949,853	1,7915277490	1,8252376938	1,7550087796	1,8893902306	0,0337099448	0,0365189694	0,0978624816
Carotis_L	72	30,680	-103,479	-949,883	1,6584589357	1,8239757779	1,6875781503	1,7941875242	0,1655168422	0,0291192146	0,1357285885
Carotis_L	73	30,659	-104,499	-949,759	1,4785193829	1,6809197014	1,5808822511	1,7195931951	0,2024003185	0,1023628682	0,2410738122
Carotis_L	74	30,327	-105,452	-949,569	1,3642734108	1,5994419213	1,3977344963	1,6202656160	0,2351685105	0,0334610855	0,2559922052
Carotis_L	75	29,771	-106,241	-949,267	1,3440343060	1,5928107970	1,3132610696	1,4747527267	0,2487764910	0,0307732364	0,1307184207
Carotis_L	76	28,942	-106,723	-949,011	1,4264644067	1,6165105576	1,3096259859	1,3644525718	0,1900461509	0,1168384208	0,0620118349
Carotis_L	77	27,972	-106,773	-948,722	1,5156633563	1,7009719950	1,3816115586	1,3605971266	0,1853086387	0,1340517977	0,1550662297
Carotis_L	78	27,062	-106,495	-948,353	1,5789047667	1,7303668816	1,4001403389	1,4131395374	0,1514621149	0,1787644278	0,1657652294
Carotis_L	79	26,319	-105,890	-948,036	1,5973094189	1,6524596407	1,4178486028	1,4562370990	0,0551502218	0,1794608162	0,1410723199
Carotis_L	80	25,604	-105,178	-947,764	1,6203587371	1,6588586442	1,4488640788	1,4641675655	0,0384999071	0,1714946583	0,1561911716
Carotis_L	81	24,956	-104,580	-947,236	1,6292821631	1,6662857463	1,4813475119	1,4568286099	0,0370035833	0,1479346512	0,1724535532
Carotis_L	82	24,561	-103,856	-946,642	1,6498123822	1,6868517839	1,4960067186	1,5107604448	0,0370394017	0,1538056636	0,1390519374
Carotis_L	83	24,321	-102,979	-946,153	1,6326051301	1,6262457505	1,4591200207	1,5674047637	0,0063593797	0,1734851095	0,0652003664
Carotis_L	84	24,213	-102,022	-945,801	1,6146927466	1,5794890122	1,4452267709	1,5733321697	0,0352037344	0,1694659757	0,0413605769
Carotis_L	85	24,311	-101,012	-945,602	1,6247048521	1,5357929257	1,4324956166	1,5379028938	0,0889119264	0,1922092355	0,0868019583
Carotis_L	86	24,571	-100,008	-945,526	1,6191803875	1,5034368039	1,4437554472	1,4977563867	0,1157435836	0,1754249404	0,1214240008
Carotis_L	87	25,008	-99,074	-945,471	1,6290719746	1,5112442717	1,4524493605	1,4952054312	0,1178277029	0,1766226141	0,1338665433
Carotis_L	88	25,652	-98,284	-945,436	1,6250732647	1,5571171853	1,4662102453	1,5092901449	0,0679560793	0,1588630194	0,1157831198
Carotis_L	89	26,475	-97,694	-945,276	1,5762569176	1,5399042967	1,5032962421	1,5198113497	0,0363526209	0,0729606755	0,0564455679
Carotis_L	90	27,329	-97,255	-944,899	1,4972600260	1,5834370459	1,4963248119	1,5447478682	0,0861770199	0,0009352141	0,0474878423
Carotis_L	91	28,114	-96,901	-944,331	1,4833681147	1,5351045933	1,4585463701	1,5420679616	0,0517364786	0,0248217446	0,0586998469
Carotis_L	92	28,915	-96,727	-943,698	1,4758544158	1,4883057890	1,4157702633	1,5051588394	0,0124513733	0,0600841525	0,0293044236
Carotis_L	93	29,522	-96,771	-942,868	1,4323947413	1,4179719217	1,3467582843	1,4544978228	0,0144228197	0,0856364570	0,0221030815
Carotis_L	94	29,865	-96,662	-941,903	1,2929149567	1,1487969695	1,2200995170	1,4198321399	0,1441179872	0,0728154398	0,1269171832
Carotis_L	95	29,530	-96,863	-940,941	1,0432156685	0,9521412378	0,9924521137	1,3502391275	0,0910744308	0,0507635548	0,3070234590
Carotis_L	96	29,028	-96,894	-940,120	0,9176508976	0,8635098318	0,8083349557	1,1780614929	0,0541410658	0,1093159419	0,2604105953
Carotis_L	97	28,117	-96,904	-939,747	0,9428767476	0,8981146543	0,8017077870	0,9769534126	0,0447620933	0,1411689606	0,0340766650
Carotis_L	98	27,084	-96,829	-939,649	1,0030185810	1,0865198792	0,9014800187	0,8753609421	0,0835012983	0,1015385623	0,1276576389

Carotis_L	99	26,094	-96,767	-939,887	1,1047908540	1,2118195007	1,0330450627	0,9331810190	0,1070286467	0,0717457913	0,1716098350
Carotis_L	100	25,131	-96,680	-940,244	1,1485943364	1,2644344642	1,1314699140	1,0549458049	0,1158401278	0,0171244224	0,0936485315
Carotis_L	101	24,135	-96,608	-940,500	1,1643028242	1,2081184984	1,1680250593	1,1414992840	0,0438156741	0,0037222350	0,0228035402
Carotis_L	102	23,149	-96,596	-940,740	1,1126978481	1,1303643633	1,1201198143	1,1634611937	0,0176665152	0,0074219662	0,0507633456
Carotis_L	103	22,156	-96,644	-940,957	1,0685823229	1,1693722323	1,0165457263	1,1333025378	0,1007899094	0,0520365966	0,0647202149
Carotis_L	104	21,136	-96,751	-941,008	1,1117413857	1,1032361280	1,0001168674	1,0700638738	0,0085052577	0,1116245183	0,0416775119
Carotis_L	105	20,148	-97,045	-941,053	1,0769634527	1,0170159365	0,9819488055	1,0574645266	0,0599475161	0,0950146472	0,0194989261
Carotis_L	106	19,169	-97,355	-941,086	1,0116308021	0,8888183471	0,9447565871	1,0401413559	0,1228124550	0,0668742150	0,0285105539
Carotis_L	107	18,205	-97,701	-941,053	1,0555428008	0,8208884184	0,9177309822	0,9594483155	0,2346543824	0,1378118186	0,0960944853
Carotis_L	108	17,307	-98,183	-941,033	1,0593538305	0,9594557436	0,8893699294	0,9306752984	0,0998980870	0,1699839011	0,1286785322
Carotis_L	109	16,624	-98,886	-940,802	1,0571025793	1,0555465864	0,9563303032	0,9240689657	0,0015559929	0,1007722761	0,1330336136
Carotis_L	110	16,330	-99,767	-940,447	1,1336907976	1,0542581897	1,0689916503	0,9544949852	0,0794326079	0,0646991473	0,1791958124
Carotis_L	111	16,589	-100,573	-940,044	1,1187798199	0,9298204269	1,0256860147	1,0302165210	0,1889593929	0,0930938052	0,0885632989
Carotis_L	112	17,266	-101,091	-939,510	1,0249692508	1,1074453331	0,9547651473	1,0475947282	0,0824760824	0,0702041035	0,0226254774
Carotis_L	113	17,920	-101,489	-938,825	1,0611426758	1,1082145098	1,0269394482	0,9469634423	0,0470718340	0,0342032276	0,1141792335
Carotis_L	114	18,360	-101,867	-937,983	1,1404958341	1,1614814896	1,0534819344	1,0495023809	0,0209856555	0,0870138997	0,0909934532
Carotis_L	115	18,588	-102,161	-937,026	1,1746120869	1,1459392085	1,0832765640	1,1280849148	0,0286728785	0,0913355230	0,0465271722
Carotis_L	116	18,739	-102,371	-936,026	1,1668199446	1,1105005785	1,0812314704	1,1430347635	0,0563193662	0,0855884742	0,0237851811
Carotis_L	117	18,720	-102,364	-935,016	1,0764426146	1,1142864443	1,0510389123	1,1724490302	0,0378438297	0,0254037023	0,0960064156
Carotis_L	118	18,619	-102,120	-934,027	0,9661466967	1,0763436520	1,0418685916	1,1156056677	0,1101969553	0,0757218949	0,1494589709
Carotis_L	119	18,432	-102,107	-933,020	1,0637618192	1,1295119107	1,0533081314	1,0665617204	0,0657500915	0,0104536879	0,0027999012
Carotis_L	120	18,257	-102,074	-932,008	1,1044592639	1,1093648721	1,1029278411	1,0770723070	0,0049056083	0,0015314228	0,0273869568
Carotis_L	121	18,102	-101,960	-931,005	1,1247687776	1,1088821119	1,0284999841	1,1077153989	0,0158866657	0,0962687934	0,0170533786
Carotis_L	122	17,901	-101,931	-929,992	1,1384468593	1,0884980418	1,0853366385	1,1208555982	0,0499488175	0,0531102208	0,0175912611
Carotis_L	123	17,731	-101,936	-928,981	1,1184062342	1,1585132415	1,0370345551	1,0740976738	0,0401070074	0,0813716791	0,0443085604
Carotis_L	124	17,653	-102,011	-927,961	1,1057864281	1,0071989334	1,0461937192	1,0941492386	0,0985874948	0,0595927089	0,0116371895
Carotis_L	125	17,659	-102,222	-926,954	1,1152437908	1,0626786145	1,0640069267	1,0348024983	0,0525651763	0,0512368641	0,0804412925
Carotis_R	1	-1,560	-87,125	-992,246	2,2016519151	1,9112010982	1,9731297884	1,9803243172	0,2904508168	0,2285221267	0,2213275978
Carotis_R	2	-2,146	-86,696	-991,489	2,2859631589	2,1501633844	2,0249851608	2,1277140359	0,1357997745	0,2609779981	0,1582491230
Carotis_R	3	-2,458	-86,365	-990,553	2,1912289880	2,2575735179	2,0586640377	2,1130156842	0,0663445299	0,1325649504	0,0782133039
Carotis_R	4	-2,888	-85,969	-989,680	2,1846854007	2,1958736395	2,0330988714	2,1267134307	0,0111882388	0,1515865293	0,0579719700
Carotis_R	5	-3,446	-85,576	-988,889	2,2093841482	2,2380078956	2,0528538329	2,1045073851	0,0286237474	0,1565303153	0,1048767631
Carotis_R	6	-3,926	-85,230	-988,029	2,1426813626	2,2394158421	2,0704441984	2,0898485811	0,0967344795	0,0722371642	0,0528327815

Carotis_R	7	-4,410	-84,862	-987,176	2,1600575793	2,1803548249	2,0414202940	2,0991721823	0,0202972456	0,1186372854	0,0608853971
Carotis_R	8	-4,918	-84,514	-986,332	2,1673068959	2,2496851172	2,0345026335	2,0606375472	0,0823782213	0,1328042625	0,1066693488
Carotis_R	9	-5,404	-84,227	-985,456	2,1347372761	2,1932666593	2,0502328248	2,0552642191	0,0585293832	0,0845044512	0,0794730570
Carotis_R	10	-5,926	-83,980	-984,582	2,0991557725	2,1847583455	2,0177883735	2,0421019238	0,0856025731	0,0813673989	0,0570538487
Carotis_R	11	-6,513	-83,755	-983,740	2,0798635112	2,1420949294	1,9912942088	2,0473529460	0,0622314182	0,0885693024	0,0325105652
Carotis_R	12	-7,067	-83,489	-982,894	2,0358349704	2,0845993804	1,9872740997	2,0324771541	0,0487644100	0,0485608707	0,0033578162
Carotis_R	13	-7,604	-83,287	-982,023	1,9868977941	2,0998273921	1,9875077573	1,9764612279	0,1129295980	0,0006099632	0,0104365663
Carotis_R	14	-8,202	-83,096	-981,190	1,9574890249	1,9930325585	1,9417536075	1,9214987796	0,0355435336	0,0157354174	0,0359902453
Carotis_R	15	-8,800	-82,959	-980,361	1,9729381188	1,9992540979	1,9079563942	1,8726647503	0,0263159792	0,0649817246	0,1002733685
Carotis_R	16	-9,302	-82,753	-979,482	1,9939837185	1,9706947492	1,9029770867	1,8870503710	0,0232889693	0,0910066319	0,1069333475
Carotis_R	17	-9,764	-82,466	-978,593	2,0449218962	2,0438875018	1,9323720368	1,9868644552	0,0010343944	0,1125498595	0,0580574411
Carotis_R	18	-10,348	-82,176	-977,781	2,0483275109	2,0821034821	1,9717460602	2,0326114323	0,0337759712	0,0765814507	0,0157160786
Carotis_R	19	-10,930	-81,974	-976,929	1,9756749383	2,0585085248	1,9799812214	1,9980495879	0,0828335866	0,0043062831	0,0223746496
Carotis_R	20	-11,215	-81,792	-975,945	1,9302302495	2,0226369390	1,8969155926	1,9673837885	0,0924066896	0,0333146569	0,0371535391
Carotis_R	21	-11,398	-81,974	-974,968	1,6805823412	1,9023361637	1,7958228111	1,8536108821	0,2217538225	0,1152404699	0,1730285409
Carotis_R	22	-11,672	-82,291	-974,020	1,4854800615	1,6544072341	1,6234661363	1,6580928768	0,1689271727	0,1379860748	0,1726128154
Carotis_R	23	-11,822	-82,550	-973,026	1,4526387308	1,5492397467	1,4489724812	1,5386043124	0,0966010159	0,0036662496	0,0859655816
Carotis_R	24	-11,790	-82,887	-972,045	1,6567614790	1,5654103396	1,4171348223	1,5675219777	0,0913511394	0,2396266567	0,0892395013
Carotis_R	25	-11,679	-83,373	-971,153	1,8906378053	1,6536481228	1,5936703631	1,7246772311	0,2369896825	0,2969674422	0,1659605742
Carotis_R	26	-11,323	-83,937	-970,354	1,9871183131	1,9403114753	1,7896495171	1,9071773190	0,0468068378	0,1974687960	0,0799409941
Carotis_R	27	-10,809	-84,599	-969,731	2,0589586300	2,0307467096	1,9631083955	1,9888964545	0,0282119204	0,0958502345	0,0700621756
Carotis_R	28	-10,288	-85,261	-969,131	2,1110629879	2,0768063381	2,0443134035	2,0512626494	0,0342566497	0,0667495843	0,0598003385
Carotis_R	29	-9,568	-85,837	-968,700	2,0954376497	2,1010154065	2,0429453947	2,0822427525	0,0055777567	0,0524922550	0,0131948972
Carotis_R	30	-8,689	-86,379	-968,553	2,0570093841	2,1220620189	2,0121871705	2,0743168296	0,0650526347	0,0448222136	0,0173074454
Carotis_R	31	-7,750	-86,815	-968,525	2,0885483694	2,0990331058	1,9918101096	2,0480208343	0,0104847364	0,0967382598	0,0405275352
Carotis_R	32	-6,767	-87,157	-968,534	2,1693015333	2,1335009734	1,9457883040	2,1045982785	0,0358005599	0,2235132293	0,0647032548
Carotis_R	33	-5,837	-87,628	-968,588	2,1423063832	2,1312758918	2,0391825566	2,0902675956	0,0110304913	0,1031238265	0,0520387876
Carotis_R	34	-4,906	-88,101	-968,686	2,1043609768	2,1045529888	2,0109106626	2,0854426615	0,0001920120	0,0934503142	0,0189183153
Carotis_R	35	-3,992	-88,609	-968,745	2,0882291106	2,1069565288	1,9788272643	2,0525711078	0,0187274182	0,1094018463	0,0356580027
Carotis_R	36	-3,107	-89,160	-968,818	2,0747384811	2,0638554546	1,9947099786	2,0439306842	0,0108830265	0,0800285025	0,0308077969
Carotis_R	37	-2,190	-89,643	-968,909	2,0838388532	2,0709989191	1,9745382576	2,0276753170	0,0128399341	0,1093005956	0,0561635362
Carotis_R	38	-1,286	-90,163	-968,927	2,0674009924	2,0300424466	1,9442568630	2,0266475251	0,0373585457	0,1231441294	0,0407534673
Carotis_R	39	-0,389	-90,683	-968,980	2,0642744262	2,0234245153	1,9278392162	2,0234977748	0,0408499108	0,1364352099	0,0407766513

Carotis_R	40	0,508	-91,207	-968,913	1,9898868842	1,9663285649	1,9074823616	1,9386211546	0,0235583193	0,0824045225	0,0512657295
Carotis_R	41	1,401	-91,736	-968,782	2,0018184516	1,9351746946	1,9073260305	1,9356015851	0,0666437570	0,0944924211	0,0662168665
Carotis_R	42	2,315	-92,183	-968,546	2,0323820328	1,9781812550	1,9141070595	1,9321438002	0,0542007778	0,1182749733	0,1002382326
Carotis_R	43	3,213	-92,629	-968,236	2,0610191934	1,9797031214	1,9247045442	1,9622514096	0,0813160720	0,1363146492	0,0987677838
Carotis_R	44	4,067	-93,047	-967,791	2,0823082845	1,9642577588	1,9493747347	1,9893342917	0,1180505256	0,1329335498	0,0929739928
Carotis_R	45	4,920	-93,302	-967,243	2,0539422826	2,0294338927	1,9653440473	2,0208825939	0,0245083899	0,0885982353	0,0330596887
Carotis_R	46	5,697	-93,564	-966,592	2,0419564531	1,9956119593	1,9980660360	1,9767956287	0,0463444939	0,0438904171	0,0651608244
Carotis_R	47	6,387	-93,744	-965,828	2,0131534117	2,0197497741	1,9615246395	1,9796384040	0,0065963625	0,0516287722	0,0335150077
Carotis_R	48	7,009	-93,866	-965,005	1,9739452399	2,0157779249	1,9386205553	1,9708143979	0,0418326850	0,0353246846	0,0031308419
Carotis_R	49	7,523	-93,819	-964,102	1,9073674606	2,0143261113	1,9085605862	1,9015188316	0,1069586507	0,0011931256	0,0058486290
Carotis_R	50	7,772	-93,787	-963,092	1,8421135528	1,9878329136	1,8380030995	1,8835507996	0,1457193609	0,0041104532	0,0414372469
Carotis_R	51	7,918	-93,823	-962,060	1,8327987793	1,9845052141	1,8253938759	1,8652287825	0,1517064348	0,0074049034	0,0324300032
Carotis_R	52	7,974	-93,959	-961,042	1,8812350701	1,9793856472	1,7956075901	1,8668069096	0,0981505771	0,0856274800	0,0144281605
Carotis_R	53	7,784	-94,241	-960,059	1,8622397903	1,9895617487	1,8561587575	1,8568491288	0,1273219583	0,0060810328	0,0053906615
Carotis_R	54	7,521	-94,720	-959,170	1,8237821124	1,9968812488	1,8638115268	1,8474241707	0,1730991364	0,0400294144	0,0236420584
Carotis_R	55	7,262	-95,283	-958,321	1,8662570259	1,9696077196	1,7920462149	1,8421199681	0,1033506937	0,0742108110	0,0241370578
Carotis_R	56	7,025	-95,959	-957,566	1,9627051532	1,9727645623	1,8349027143	1,8563979409	0,0100594091	0,1278024389	0,1063072124
Carotis_R	57	6,959	-96,750	-956,904	2,0085959384	1,9434485759	1,8736115524	1,9022294714	0,0651473625	0,1349843860	0,1063664670
Carotis_R	58	7,037	-97,509	-956,189	1,9994729082	1,9582226145	1,8852519171	1,8860165729	0,0412502937	0,1142209911	0,1134563353
Carotis_R	59	7,041	-98,252	-955,453	1,9686981639	1,8968493102	1,8795359146	1,8621254369	0,0718488537	0,0891622494	0,1065727270
Carotis_R	60	6,933	-99,023	-954,760	1,9462469584	1,7822105192	1,8648232806	1,8807222304	0,1640364393	0,0814236779	0,0655247281
Carotis_R	61	6,875	-99,802	-954,065	1,9041471528	1,7861901076	1,8348684711	1,8462672306	0,1179570452	0,0692786817	0,0578799222
Carotis_R	62	6,741	-100,650	-953,477	1,9150370770	1,8373049664	1,8176202608	1,8096486045	0,0777321106	0,0974168162	0,1053884726
Carotis_R	63	6,523	-101,481	-952,896	1,9336516479	1,8364093566	1,8250323596	1,8137294659	0,0972422913	0,1086192883	0,1199221820
Carotis_R	64	6,298	-102,387	-952,439	1,9560664450	1,8536977727	1,8047144847	1,8120026773	0,1023686722	0,1513519603	0,1440637677
Carotis_R	65	5,849	-103,221	-951,993	1,8886587710	1,8733571236	1,7495686816	1,7982409586	0,0153016474	0,1390900894	0,0904178124
Carotis_R	66	5,352	-104,063	-951,647	1,8648765253	1,8772469037	1,7091577628	1,7477406574	0,0123703784	0,1557187626	0,1171358679
Carotis_R	67	5,104	-104,995	-951,268	1,8155801152	1,9025624544	1,6840523251	1,7348596453	0,0869823393	0,1315277901	0,0807204699
Carotis_R	68	5,198	-105,762	-950,605	1,7847550698	1,8791498405	1,6671253614	1,5939305801	0,0943947707	0,1176297084	0,1908244896
Carotis_R	69	5,724	-106,235	-949,854	1,6608892145	1,6367401986	1,5962827591	1,5089339860	0,0241490159	0,0646064554	0,1519552284
Carotis_R	70	6,421	-106,385	-949,117	1,5957781676	1,6004976885	1,4565716701	1,4635581453	0,0047195209	0,1392064975	0,1322200224
Carotis_R	71	7,297	-106,218	-948,588	1,6030356003	1,5383424655	1,3314135150	1,3829407296	0,0646931348	0,2716220853	0,2200948707
Carotis_R	72	8,190	-105,828	-948,238	1,6332827209	1,5121741037	1,2976013529	1,5146637630	0,1211086171	0,3356813680	0,1186189579

Carotis_R	73	8,959	-105,180	-947,949	1,6236951876	1,5272621314	1,3385727899	1,5079498829	0,0964330562	0,2851223977	0,1157453047
Carotis_R	74	9,662	-104,452	-947,703	1,6119493350	1,5450159813	1,3884774193	1,5322386530	0,0669333536	0,2234719157	0,0797106820
Carotis_R	75	10,247	-103,652	-947,381	1,6481661142	1,5238266795	1,4487484597	1,5167778319	0,1243394348	0,1994176545	0,1313882823
Carotis_R	76	10,674	-102,783	-946,996	1,6259171361	1,4725184956	1,4259263503	1,4859506836	0,1533986406	0,1999907858	0,1399664525
Carotis_R	77	10,927	-101,856	-946,591	1,5961309602	1,4753676077	1,4475426053	1,4761959612	0,1207633526	0,1485883549	0,1199349990
Carotis_R	78	11,021	-100,906	-946,182	1,6261537237	1,5236239376	1,4869663092	1,5254895992	0,1025297862	0,1391874145	0,1006641245
Carotis_R	79	10,924	-99,923	-945,868	1,6473486070	1,5635666420	1,5342488114	1,5313248100	0,0837819651	0,1130997956	0,1160237970
Carotis_R	80	10,644	-98,936	-945,716	1,6538891799	1,5590529181	1,5687173359	1,5611753492	0,0948362618	0,0851718440	0,0927138307
Carotis_R	81	10,238	-97,972	-945,696	1,6676352550	1,5358504376	1,5740038280	1,5343007883	0,1317848174	0,0936314270	0,1333344667
Carotis_R	82	9,660	-97,112	-945,770	1,6417116419	1,5042717518	1,5395514352	1,5145132520	0,1374398901	0,1021602067	0,1271983899
Carotis_R	83	8,972	-96,343	-945,950	1,5935310175	1,4981864746	1,4694812731	1,5115442752	0,0953445430	0,1240497445	0,0819867424
Carotis_R	84	8,268	-95,616	-946,159	1,6707016684	1,4345135142	1,4387790775	1,5238991844	0,2361881542	0,2319225910	0,1468024840
Carotis_R	85	7,549	-94,885	-946,368	1,7036365720	1,5243649748	1,5132009055	1,5666507777	0,1792715972	0,1904356665	0,1369857943
Carotis_R	86	6,786	-94,205	-946,577	1,6272879927	1,5046452985	1,5204287506	1,5626915024	0,1226426942	0,1068592421	0,0645964902
Carotis_R	87	5,935	-93,688	-946,802	1,5544836687	1,5320180335	1,4696998540	1,5234454199	0,0224656352	0,0847838146	0,0310382488
Carotis_R	88	5,024	-93,209	-946,992	1,5526452680	1,6060208936	1,5047367080	1,5580035836	0,0533756255	0,0479085600	0,0053583155
Carotis_R	89	4,099	-92,774	-947,111	1,6828233847	1,6960258647	1,5884164732	1,6267643769	0,0132024799	0,0944069115	0,0560590078
Carotis_R	90	3,097	-92,840	-946,908	1,5808628893	1,5545093271	1,5854129259	1,5708952176	0,0263535622	0,0045500366	0,0099676717
Carotis_R	91	2,212	-93,332	-946,649	1,3887538707	1,4573311481	1,4201724666	1,4028269071	0,0685772774	0,0314185960	0,0140730364
Carotis_R	92	1,472	-93,981	-946,320	1,2805935946	1,2688393086	1,2316917168	1,2502207846	0,0117542861	0,0489018779	0,0303728101
Carotis_R	93	0,806	-94,768	-946,119	1,2513473891	1,1997452206	1,1314163205	1,1660843464	0,0516021685	0,1199310686	0,0852630427
Carotis_R	94	0,236	-95,608	-946,028	1,2077247105	1,1705534128	1,1404228812	1,1016321081	0,0371712977	0,0673018292	0,1060926024
Carotis_R	95	-0,312	-96,453	-945,762	1,1972991898	1,1393668325	1,1366176174	1,1152632996	0,0579323572	0,0606815723	0,0820358901
Carotis_R	96	-0,932	-97,208	-945,428	1,2350487949	1,1550120055	1,1302776260	1,1524709685	0,0800367894	0,1047711689	0,0825778265
Carotis_R	97	-1,523	-97,949	-945,062	1,1825565681	1,1508068614	1,1107286948	1,1240842751	0,0317497067	0,0718278734	0,0584722931
Carotis_R	98	-2,133	-98,696	-944,746	1,1120420327	1,1235283901	1,0447727147	1,0535645110	0,0114863574	0,0672693180	0,0584775217
Carotis_R	99	-2,912	-99,250	-944,339	1,1133958204	1,0688615130	1,0148612016	1,0628581752	0,0445343074	0,0985346188	0,0505376451
Carotis_R	100	-3,729	-99,722	-943,910	1,1663863293	1,1518618058	1,0695121761	1,0928925988	0,0145245235	0,0968741532	0,0734937305
Carotis_R	101	-4,599	-100,095	-943,499	1,1719467083	0,9958638613	1,1322227292	1,0714179301	0,1760828471	0,0397239791	0,1005287783
Carotis_R	102	-5,505	-100,383	-943,085	1,1463544856	0,9548726185	1,1145554214	1,0814250307	0,1914818671	0,0317990642	0,0649294549
Carotis_R	103	-6,457	-100,673	-942,787	1,1108718802	0,9277563366	1,0407421019	1,0837754938	0,1831155436	0,0701297783	0,0270963864
Carotis_R	104	-7,429	-100,893	-942,484	1,1171511466	1,1324078696	1,0361087186	1,0181131977	0,0152567229	0,0810424280	0,0990379489
Carotis_R	105	-8,443	-101,129	-942,417	1,2243115433	1,4175376772	1,0314742960	1,1586272567	0,1932261339	0,1928372473	0,0656842866

Carotis_R	106	-9,465	-101,375	-942,422	1,3805978482	1,6406967914	1,2190901763	1,3251816615	0,2600989432	0,1615076718	0,0554161867
Carotis_R	107	-10,382	-101,779	-942,664	1,5254916685	1,8474680747	1,3816690779	1,5060346975	0,3219764062	0,1438225906	0,0194569710
Carotis_R	108	-11,227	-102,371	-942,853	1,6718408243	1,8572664426	1,5509991897	1,5203259367	0,1854256183	0,1208416346	0,1515148876
Carotis_R	109	-11,808	-103,223	-942,994	1,5109144827	1,5656074337	1,5347620946	1,4399292310	0,0546929510	0,0238476119	0,0709852518
Vertebralis_L	1	32,064	-63,917	-990,449	1,2960453751	1,1851498374	1,1394844356	1,1730894133	0,1108955377	0,1565609395	0,1229559618
Vertebralis_L	2	31,750	-64,125	-989,469	1,3123366352	1,2055305604	1,1752464475	1,1529277129	0,1068060749	0,1370901878	0,1594089224
Vertebralis_L	3	31,572	-64,223	-988,445	1,4194699580	1,2553748535	1,1529100124	1,2623190883	0,1640951045	0,2665599456	0,1571508697
Vertebralis_L	4	31,373	-64,341	-987,427	1,4650908988	1,2978415226	1,2752354385	1,3576391346	0,1672493762	0,1898554603	0,1074517642
Vertebralis_L	5	31,221	-64,479	-986,400	1,4177201747	1,4008279659	1,3606547342	1,3774527314	0,0168922088	0,0570654405	0,0402674433
Vertebralis_L	6	31,096	-64,636	-985,375	1,3758402014	1,4578531012	1,3665480600	1,4256644440	0,0820128998	0,0092921414	0,0498242426
Vertebralis_L	7	30,944	-64,865	-984,368	1,2877425168	1,4213330885	1,3298881621	1,3795088326	0,1335905717	0,0421456454	0,0917663158
Vertebralis_L	8	30,767	-65,068	-983,369	1,2880278042	1,3521620704	1,2514424670	1,2780027697	0,0641342662	0,0365853372	0,0100250345
Vertebralis_L	9	30,632	-65,326	-982,383	1,2460881455	1,2864560721	1,1477655079	1,2482450688	0,0403679266	0,0983226376	0,0021569233
Vertebralis_L	10	30,523	-65,619	-981,386	1,2056655357	1,2315242473	1,0740178057	1,1423502565	0,0258587116	0,1316477300	0,0633152792
Vertebralis_L	11	30,370	-65,908	-980,397	1,1679560133	1,2262761527	1,1024234131	1,1391892752	0,0583201394	0,0655326003	0,0287667381
Vertebralis_L	12	30,144	-66,176	-979,414	1,1900384118	1,2036279808	1,0391706200	1,1065733622	0,0135895690	0,1508677918	0,0834650496
Vertebralis_L	13	29,938	-66,499	-978,457	1,1991341485	1,1540327756	1,0283143535	1,0752656281	0,0451013729	0,1708197950	0,1238685204
Vertebralis_L	14	29,863	-67,003	-977,567	1,1312577613	1,1489405928	1,0348819794	1,1099780499	0,0176828314	0,0963757819	0,0212797114
Vertebralis_L	15	29,817	-67,684	-976,790	1,0789384472	1,1804327546	1,0936668826	1,1416404974	0,1014943074	0,0147284354	0,0627020502
Vertebralis_L	16	29,696	-68,430	-976,069	1,1556212589	1,2059238536	1,1892297753	1,2106856534	0,0503025947	0,0336085164	0,0550643945
Vertebralis_L	17	29,516	-69,254	-975,453	1,2285305468	1,1166000134	1,0770499909	0,9614126643	0,1119305335	0,1514805559	0,2671178825
Vertebralis_L	18	29,284	-70,108	-974,904	1,2047610280	1,0466014057	1,0015975298	1,0055714532	0,1581596222	0,2031634982	0,1991895747
Vertebralis_L	19	28,953	-71,001	-974,509	1,1745771186	1,0960613203	0,9766342332	1,0561545290	0,0785157983	0,1979428854	0,1184225895
Vertebralis_L	20	28,556	-71,906	-974,181	1,1356896351	1,1753521601	1,0095778906	1,0943760681	0,0396625250	0,1261117445	0,0413135670
Vertebralis_L	21	28,100	-72,770	-973,874	1,0920630484	1,1594601064	1,0580754229	1,0846875142	0,0673970580	0,0339876255	0,0073755343
Vertebralis_L	22	27,420	-73,525	-973,652	1,0959161485	1,1060959182	0,9996383473	1,0579910325	0,0101797697	0,0962778012	0,0379251160
Vertebralis_L	23	26,677	-74,229	-973,593	1,1286011306	1,0239814567	0,9287514056	0,9916669300	0,1046196740	0,1998497250	0,1369342006
Vertebralis_L	24	25,925	-74,943	-973,521	1,1473242380	1,0060747538	0,9189572375	1,0217951979	0,1412494843	0,2283670006	0,1255290402
Vertebralis_L	25	25,023	-75,467	-973,547	1,1854249930	1,1371671285	1,0573354227	1,0343585368	0,0482578645	0,1280895702	0,1510664562
Vertebralis_L	26	24,123	-75,963	-973,555	1,1296710367	1,1676994614	1,0557464884	1,1055075137	0,0380284247	0,0739245483	0,0241635230
Vertebralis_L	27	23,202	-76,443	-973,487	1,0962339044	1,1328106920	1,0513207427	1,0794888602	0,0365767877	0,0449131617	0,0167450442
Vertebralis_L	28	22,248	-76,819	-973,332	1,0596363633	1,0706644305	0,9669030575	1,0202071234	0,0110280672	0,0927333058	0,0394292399
Vertebralis_L	29	21,307	-77,237	-973,210	1,0724122848	0,9801855976	0,9438002727	0,9947850489	0,0922266873	0,1286120121	0,0776272360

Vertebralis_L	30	20,367	-77,592	-972,957	1,1004188779	0,8895707587	0,8955151423	0,9582109037	0,2108481191	0,2049037356	0,1422079742
Vertebralis_L	31	19,474	-77,863	-972,491	1,1237293449	1,0274579393	1,0019234423	1,0934759869	0,0962714056	0,1218059026	0,0302533580
Vertebralis_L	32	18,645	-78,128	-971,917	1,1181684484	1,1254257140	1,0203492779	1,0819636952	0,0072572656	0,0978191705	0,0362047532
Vertebralis_L	33	17,905	-78,351	-971,236	1,0759556072	1,0964660978	0,9915015393	1,0436539258	0,0205104906	0,0844540679	0,0323016814
Vertebralis_L	34	17,380	-78,590	-970,384	1,1205347591	1,1034949845	0,9639981112	1,0440536234	0,0170397746	0,1565366478	0,0764811356
Vertebralis_L	35	17,025	-78,956	-969,482	1,2926566726	1,1308998902	1,0016193575	1,0934389705	0,1617567824	0,2910373151	0,1992177021
Vertebralis_L	36	16,725	-79,404	-968,600	1,5711690326	1,2985809046	1,1318552527	1,2153120530	0,2725881280	0,4393137798	0,3558569795
Vertebralis_L	37	16,704	-79,882	-967,714	1,8059728262	1,5601717697	1,3855457489	1,4216539815	0,2458010565	0,4204270773	0,3843188448
Vertebralis_L	38	16,948	-80,275	-966,790	1,8937014664	1,7956416743	1,6251388600	1,6933155581	0,0980597921	0,2685626064	0,2003859083
Vertebralis_L	39	17,286	-80,595	-965,855	1,8966438595	1,8744763856	1,7661579753	1,9062571065	0,0221674739	0,1304858842	0,0096132470
Vertebralis_L	40	17,707	-80,921	-964,959	1,8830972210	1,8840293246	1,8036220624	1,8630726266	0,0009321036	0,0794751586	0,0200245944
Vertebralis_L	41	18,036	-81,281	-964,044	1,8833977230	1,8648561746	1,7989367580	1,9211294288	0,0185415484	0,0844609650	0,0377317058
Vertebralis_L	42	18,401	-81,737	-963,195	1,8609273843	1,8125696935	1,8124019917	1,8632781404	0,0483576908	0,0485253926	0,0023507561
Vertebralis_L	43	18,734	-82,077	-962,257	1,8148467530	1,7976869887	1,7673057043	1,8317993153	0,0171597643	0,0475410486	0,0169525624
Vertebralis_L	44	19,037	-82,471	-961,337	1,7871665004	1,7791840454	1,7242257915	1,7811249157	0,0079824550	0,0629407089	0,0060415847
Vertebralis_L	45	19,310	-82,832	-960,394	1,7032750739	1,7296332082	1,7245999231	1,7834697620	0,0263581343	0,0213248492	0,0801946881
Vertebralis_L	46	19,541	-83,199	-959,445	1,6448632547	1,6586416298	1,6460247405	1,7238177472	0,0137783752	0,0011614859	0,0789544926
Vertebralis_L	47	19,819	-83,490	-958,480	1,6103559785	1,5709429256	1,5446768712	1,6249264909	0,0394130530	0,0656791074	0,0145705123
Vertebralis_L	48	19,972	-83,829	-957,504	1,6013056758	1,4835514006	1,5203371053	1,6000506250	0,1177542753	0,0809685705	0,0012550508
Vertebralis_L	49	20,197	-84,141	-956,532	1,5385120048	1,4931327216	1,4817215764	1,5098837277	0,0453792832	0,0567904284	0,0286282771
Vertebralis_L	50	20,352	-84,388	-955,532	1,5628363460	1,5238495014	1,4613230313	1,5253674567	0,0389868446	0,1015133147	0,0374688893
Vertebralis_L	51	20,494	-84,588	-954,513	1,5966151414	1,5186416309	1,4162988271	1,5206446667	0,0779735106	0,1803163144	0,0759704748
Vertebralis_L	52	20,627	-84,788	-953,497	1,5092783250	1,5121123331	1,4211864565	1,4882659657	0,0028340080	0,0880918685	0,0210123594
Vertebralis_L	53	20,704	-85,051	-952,489	1,5035975000	1,5008910747	1,3845441944	1,4725469904	0,0027064253	0,1190533057	0,0310505097
Vertebralis_L	54	20,795	-85,230	-951,466	1,4882785130	1,5010492047	1,3644170929	1,4437017109	0,0127706917	0,1238614200	0,0445768021
Vertebralis_L	55	20,749	-85,324	-950,427	1,4539974960	1,5011763942	1,3271354847	1,4627353157	0,0471788982	0,1268620113	0,0087378197
Vertebralis_L	56	20,738	-85,374	-949,396	1,4262417048	1,5524777960	1,3102305991	1,4716866076	0,1262360911	0,1160111057	0,0454449027
Vertebralis_L	57	20,832	-85,307	-948,365	1,4147613171	1,5190127733	1,2822989131	1,4751210117	0,1042514562	0,1324624040	0,0603596946
Vertebralis_L	58	20,846	-85,001	-947,374	1,4199008423	1,5402475106	1,2250162820	1,4115469652	0,1203466683	0,1948845603	0,0083538771
Vertebralis_L	59	20,868	-84,625	-946,403	1,4779745616	1,5596721645	1,2231126771	1,3365992432	0,0816976030	0,2548618844	0,1413753183
Vertebralis_L	60	20,991	-84,214	-945,448	1,6547926483	1,7875814893	1,3900105652	1,3926514113	0,1327888410	0,2647820831	0,2621412370
Vertebralis_L	61	21,153	-83,766	-944,521	1,8407038289	2,0795895661	1,7140502068	1,5100511261	0,2388857372	0,1266536220	0,3306527028
Vertebralis_L	62	21,678	-83,714	-943,715	1,6149870668	2,0651702636	1,9922142265	1,7085034781	0,4501831968	0,3772271597	0,0935164113

Vertebralis_L	63	22,413	-83,938	-943,032	1,3129467578	1,7405539215	1,9691272285	1,8780686878	0,4276071637	0,6561804707	0,5651219300
Vertebralis_L	64	23,090	-84,253	-942,309	1,1407913977	1,3990243132	1,5701892339	1,6900374771	0,2582329155	0,4293978362	0,5492460794
Vertebralis_L	65	23,855	-84,576	-941,686	1,1614757846	1,2187272442	1,1713227065	1,3461470614	0,0572514596	0,0098469219	0,1846712768
Vertebralis_L	66	24,690	-84,982	-941,223	1,2969399199	1,2432532975	1,1346469581	1,1775189705	0,0536866225	0,1622929618	0,1194209494
Vertebralis_L	67	25,397	-85,651	-940,876	1,4459482691	1,3358675249	1,2330051604	1,1588664474	0,1100807441	0,2129431087	0,2870818216
Vertebralis_L	68	25,648	-86,607	-941,076	1,3273288764	1,2826185288	1,2878514565	1,2853160178	0,0447103477	0,0394774200	0,0420128586
Vertebralis_L	69	25,624	-87,529	-941,555	1,2308884183	1,2523333521	1,1582766776	1,2456382924	0,0214449338	0,0726117408	0,0147498741
Vertebralis_L	70	25,693	-88,323	-942,204	1,2524692951	1,2539760459	1,1019210610	1,1910117572	0,0015067509	0,1505482341	0,0614575379
Vertebralis_L	71	26,176	-88,871	-942,935	1,2582480630	1,2414089872	1,1388974677	1,1826409459	0,0168390758	0,1193505953	0,0756071171
Vertebralis_L	72	26,900	-89,163	-943,609	1,1878973761	1,2179146089	1,1416650406	1,2009273893	0,0300172329	0,0462323355	0,0130300132
Vertebralis_L	73	27,737	-89,104	-944,209	1,1871967601	1,1911394058	1,1553074746	1,2080015953	0,0039426457	0,0318892855	0,0208048352
Vertebralis_L	74	28,480	-88,695	-944,792	1,1639030395	1,1252934776	1,1431346065	1,1806294871	0,0386095619	0,0207684330	0,0167264476
Vertebralis_L	75	29,055	-88,030	-945,332	1,0742035540	1,0971594753	1,0637173991	1,1825736756	0,0229559213	0,0104861548	0,1083701217
Vertebralis_L	76	29,452	-87,211	-945,832	1,0613163877	1,0857840411	1,0001260653	1,1135531512	0,0244676534	0,0611903223	0,0522367635
Vertebralis_L	77	29,804	-86,313	-946,192	1,0842175863	1,0659097413	0,9770294154	1,0215251785	0,0183078450	0,1071881709	0,0626924079
Vertebralis_L	78	30,000	-85,329	-946,427	0,9825134283	1,0489802433	0,9698857798	1,0393889767	0,0664668150	0,0126276485	0,0568755484
Vertebralis_L	79	30,167	-84,318	-946,548	0,9265875522	1,0230665982	0,9542876834	1,0705573457	0,0964790460	0,0277001312	0,1439697935
Vertebralis_L	80	30,391	-83,307	-946,578	0,9315075076	1,0088231604	0,8831355284	0,9482626470	0,0773156528	0,0483719791	0,0167551394
Vertebralis_L	81	30,628	-82,295	-946,596	0,9142715537	1,0083943786	0,9202424730	0,9392353252	0,0941228249	0,0059709193	0,0249637715
Vertebralis_L	82	30,898	-81,295	-946,612	0,8989755317	1,0352520000	0,9464303831	0,9774221529	0,1362764683	0,0474548514	0,0784466212
Vertebralis_L	83	31,248	-80,335	-946,523	0,9535136354	1,0424941491	0,8972139424	0,9829407741	0,0889805137	0,0562996930	0,0294271387
Vertebralis_L	84	31,633	-79,387	-946,368	0,9895189230	1,0070564913	0,9860662123	0,9612920001	0,0175375684	0,0034527107	0,0282269229
Vertebralis_L	85	32,000	-78,441	-946,180	0,9293386582	1,0145867699	0,9769581290	1,0232604246	0,0852481117	0,0476194707	0,0939217663
Vertebralis_L	86	32,376	-77,499	-945,944	0,9537983266	0,9923411492	0,8776375613	1,0378030315	0,0385428225	0,0761607653	0,0840047049
Vertebralis_L	87	32,787	-76,567	-945,691	1,0177180713	0,9727762761	0,9069157006	1,0269846527	0,0449417953	0,1108023707	0,0092665813
Vertebralis_L	88	33,193	-75,652	-945,396	0,9794170388	0,9827881958	0,8852752406	0,9724669925	0,0033711569	0,0941417982	0,0069500463
Vertebralis_L	89	33,559	-74,742	-945,037	0,9857684062	1,0030611661	0,9329827975	0,9381725328	0,0172927600	0,0527856087	0,0475958734
Vertebralis_L	90	33,927	-73,886	-944,589	1,0776434272	1,0685154538	0,9505322857	0,9662138456	0,0091279734	0,1271111415	0,1114295816
Vertebralis_R	1	12,644	-64,487	-992,666	1,3140725470	1,5893627997	1,2162843066	1,2378645921	0,2752902527	0,0977882404	0,0762079549
Vertebralis_R	2	12,765	-64,662	-991,651	1,3397416932	1,4954011728	1,2356227893	1,2213175033	0,1556594796	0,1041189039	0,1184241899
Vertebralis_R	3	12,869	-64,734	-990,615	1,3235601883	1,3931452112	1,2371628810	1,2576607756	0,0695850229	0,0863973073	0,0658994128
Vertebralis_R	4	12,808	-64,845	-989,586	1,3055506601	1,3638914881	1,1800439277	1,2565188484	0,0583408281	0,1255067323	0,0490318117
Vertebralis_R	5	12,739	-64,972	-988,555	1,2639585731	1,3186258210	1,2016827692	1,2514476331	0,0546672478	0,0622758039	0,0125109400

Vertebralis_R	6	12,670	-65,007	-987,518	1,2836464593	1,2744039362	1,1865386362	1,2349975923	0,0092425231	0,0971078230	0,0486488669
Vertebralis_R	7	12,418	-65,206	-986,541	1,2389029308	1,3252812863	1,1524639170	1,1801484654	0,0863783555	0,0864390138	0,0587544654
Vertebralis_R	8	12,151	-65,216	-985,536	1,2460210026	1,2520323401	1,1454726034	1,1806757701	0,0060113375	0,1005483992	0,0653452326
Vertebralis_R	9	11,808	-65,287	-984,556	1,2769684378	1,2144076231	1,2239127582	1,2186370512	0,0625608147	0,0530556796	0,0583313867
Vertebralis_R	10	11,425	-65,363	-983,594	1,2838509112	1,2851598353	1,1899208281	1,2353837522	0,0013089241	0,0939300831	0,0484671590
Vertebralis_R	11	11,038	-65,496	-982,642	1,2667007399	1,2653811170	1,1297296057	1,2049678785	0,0013196229	0,1369711342	0,0617328614
Vertebralis_R	12	10,568	-65,648	-981,734	1,2546355793	1,2431004821	1,1256659338	1,2026284653	0,0115350972	0,1289696455	0,0520071140
Vertebralis_R	13	10,095	-65,897	-980,853	1,2434542101	1,2356353179	1,0914990893	1,1834351742	0,0078188922	0,1519551208	0,0600190359
Vertebralis_R	14	9,664	-66,206	-979,967	1,1992801439	1,2255471953	1,0459684304	1,1495384251	0,0262670514	0,1533117135	0,0497417188
Vertebralis_R	15	9,270	-66,633	-979,105	1,1699841386	1,1971733717	1,0508559578	1,1030360703	0,0271892331	0,1191281808	0,0669480683
Vertebralis_R	16	8,907	-67,088	-978,245	1,1718611789	1,1362284667	1,0660838340	1,1136604661	0,0356327122	0,1057773449	0,0582007128
Vertebralis_R	17	8,599	-67,609	-977,402	1,2018037652	1,1145062446	1,0745338252	1,0975978858	0,0872975206	0,1272699399	0,1042058794
Vertebralis_R	18	8,327	-68,194	-976,591	1,2160100626	1,0605116511	1,0735927210	1,1179447314	0,1554984114	0,1424173415	0,0980653311
Vertebralis_R	19	8,093	-68,772	-975,755	1,2221527777	0,9820019277	1,1036553168	1,1435267188	0,2401508501	0,1184974609	0,0786260590
Vertebralis_R	20	7,950	-69,426	-974,971	1,2052233821	1,0773657850	1,1421286783	1,0667346195	0,1278575971	0,0630947038	0,1384887626
Vertebralis_R	21	7,990	-70,181	-974,284	1,2342927375	1,1313724551	1,2016930045	1,0905468912	0,1029202824	0,0325997330	0,1437458463
Vertebralis_R	22	8,132	-70,886	-973,562	1,2903335260	1,1749193409	1,2460223973	1,1400095286	0,1154141851	0,0443111288	0,1503239974
Vertebralis_R	23	8,379	-71,629	-972,897	1,3170417354	1,2085451984	1,2228340653	1,2342914835	0,1084965370	0,0942076701	0,0827502519
Vertebralis_R	24	8,708	-72,447	-972,355	1,3288772578	1,2394670044	1,2447808615	1,2486722217	0,0894102534	0,0840963963	0,0802050361
Vertebralis_R	25	9,093	-73,241	-971,815	1,3830923774	1,2714040814	1,2985796549	1,2899661362	0,1116882960	0,0845127225	0,0931262413
Vertebralis_R	26	9,491	-74,050	-971,297	1,4248402609	1,2986598998	1,3129744370	1,3249153782	0,1261803611	0,1118658238	0,0999248827
Vertebralis_R	27	9,922	-74,888	-970,871	1,3925526180	1,2966249582	1,2300747642	1,2492891439	0,0959276598	0,1624778538	0,1432634741
Vertebralis_R	28	10,261	-75,712	-970,349	1,3652064809	1,2726255181	1,1398999150	1,2345554460	0,0925809627	0,2253065658	0,1306510349
Vertebralis_R	29	10,688	-76,555	-969,937	1,2965574548	1,2436456403	1,0933356265	1,2378305965	0,0529118145	0,2032218283	0,0587268584
Vertebralis_R	30	11,314	-77,314	-969,615	1,2450219843	1,1825299222	1,0633920976	1,1737533442	0,0624920621	0,1816298867	0,0712686401
Vertebralis_R	31	11,944	-78,113	-969,401	1,2636405057	1,2129989030	0,9954879926	1,1987889432	0,0506416027	0,2681525131	0,0648515625
Vertebralis_R	32	12,692	-78,732	-969,100	1,2583512436	1,2051883773	0,9911165205	1,1867401875	0,0531628663	0,2672347232	0,0716110561
Vertebralis_R	33	13,431	-79,404	-968,881	1,2098867460	1,1468474259	1,0727271106	1,1747542074	0,0630393201	0,1371596354	0,0351325386
Vertebralis_R	34	14,384	-79,740	-968,667	1,1659911346	1,2097417942	1,2159421903	1,2765420839	0,0437506596	0,0499510557	0,1105509493
Vertebralis_R	35	15,376	-79,917	-968,416	1,4113923752	1,3684862200	1,4544782282	1,4669953683	0,0429061552	0,0430858530	0,0556029931
Vertebralis_R	36	16,246	-80,062	-967,893	1,7342986552	1,5691455210	1,7740808610	1,7285109841	0,1651531342	0,0397822058	0,0057876711
Vertebralis_R	37	16,830	-80,183	-967,046	1,8929618871	1,7766426213	1,7995272847	1,9094401369	0,1163192658	0,0934346024	0,0164782498
Vertebralis_R	38	17,217	-80,492	-966,135	1,9099624316	1,8760982858	1,7955067367	1,8655384082	0,0338641458	0,1144556950	0,0444240235

Vertebralis_R	39	17,528	-80,854	-965,214	1,8920489081	1,8848931915	1,7993848034	1,9087169192	0,0071557166	0,0926641048	0,0166680111
Vertebralis_R	40	17,945	-81,216	-964,343	1,8766050719	1,8491146221	1,8095557530	1,8675649046	0,0274904498	0,0670493190	0,0090401674
Vertebralis_R	41	18,268	-81,545	-963,416	1,8813362250	1,8068476832	1,7549934425	1,8154427386	0,0744885418	0,1263427825	0,0658934864
Vertebralis_R	42	18,637	-81,991	-962,561	1,8290268066	1,7979721424	1,7231880650	1,7870240411	0,0310546642	0,1058387415	0,0420027655
Vertebralis_R	43	18,942	-82,310	-961,617	1,7925234655	1,7713866927	1,7061460670	1,7771043826	0,0211367728	0,0863773984	0,0154190829
Vertebralis_R	44	19,231	-82,707	-960,703	1,7355265957	1,7204215550	1,6217210866	1,7131739576	0,0151050407	0,1138055091	0,0223526380
Vertebralis_R	45	19,494	-83,088	-959,772	1,6635301982	1,6466195965	1,5412226350	1,6216591011	0,0169106018	0,1223075632	0,0418710972
Vertebralis_R	46	19,743	-83,411	-958,817	1,6214638438	1,5609046501	1,5087908825	1,5863021063	0,0605591936	0,1126729613	0,0351617375
Vertebralis_R	47	19,953	-83,720	-957,850	1,5927662348	1,4732474985	1,4805595347	1,5191110647	0,1195187362	0,1122067000	0,0736551700
Vertebralis_R	48	20,136	-84,039	-956,878	1,5673012670	1,4987831401	1,4459480467	1,5219476657	0,0685181269	0,1213532204	0,0453536013
Vertebralis_R	49	20,300	-84,312	-955,891	1,5571196291	1,5242477880	1,4211793842	1,5172700158	0,0328718411	0,1359402449	0,0398496133
Vertebralis_R	50	20,448	-84,502	-954,881	1,5486756492	1,5193846842	1,4117549383	1,4862816102	0,0292909651	0,1369207110	0,0623940390
Vertebralis_R	51	20,585	-84,720	-953,874	1,5334505560	1,5154525474	1,3890136117	1,4649319395	0,0179980086	0,1444369443	0,0685186165
Vertebralis_R	52	20,683	-84,942	-952,865	1,5113173489	1,4968503696	1,3576029683	1,4463286109	0,0144669793	0,1537143806	0,0649887381
Vertebralis_R	53	20,770	-85,162	-951,852	1,5029750329	1,5015181362	1,3229524041	1,4588186407	0,0014568968	0,1800226289	0,0441563923
Vertebralis_R	54	20,760	-85,312	-950,833	1,4496496357	1,4999083390	1,3082947221	1,4814138723	0,0502587034	0,1413549135	0,0317642366
Vertebralis_R	55	20,751	-85,330	-949,803	1,4324576262	1,5490842890	1,2847753443	1,4761713377	0,1166266628	0,1476822819	0,0437137115
Vertebralis_R	56	20,788	-85,336	-948,775	1,3920151876	1,5213484613	1,2220383678	1,4093844448	0,1293332738	0,1699768198	0,0173692572
Vertebralis_R	57	20,806	-85,157	-947,766	1,4178703296	1,5490065612	1,2162200939	1,3362154915	0,1311362315	0,2016502357	0,0816548381
Vertebralis_R	58	20,846	-84,782	-946,798	1,4359378557	1,5498078971	1,3820380576	1,3973968839	0,1138700414	0,0538997981	0,0385409717
Vertebralis_R	59	20,847	-84,411	-945,827	1,5896067656	1,7341229875	1,6965920573	1,5149086383	0,1445162219	0,1069852917	0,0746981273
Vertebralis_R	60	20,823	-83,908	-944,924	1,8689199819	2,0373938493	2,0948352207	1,6976087607	0,1684738674	0,2259152388	0,1713112213
Vertebralis_R	61	20,529	-83,240	-944,211	2,1584925604	2,3081798682	2,4312652445	1,9546714343	0,1496873078	0,2727726841	0,2038211261
Vertebralis_R	62	20,095	-82,509	-943,621	2,5451903694	2,6590720160	2,7299350665	2,3346175444	0,1138816467	0,1847446972	0,2105728250
Vertebralis_R	63	19,599	-81,679	-943,256	2,8952676833	2,9009174243	2,9969435150	2,7779374763	0,0056497410	0,1016758317	0,1173302071
Vertebralis_R	64	19,086	-80,790	-943,107	3,0612580607	2,9973213271	3,0616621114	2,9300927415	0,0639367336	0,0004040506	0,1311653192
Vertebralis_R	65	18,562	-79,917	-943,234	2,9650936224	2,9222463303	2,8941304723	2,9177062040	0,0428472921	0,0709631501	0,0473874184
Vertebralis_R	66	18,023	-79,113	-943,600	2,8219802287	2,7729197398	2,6452287837	2,7589664386	0,0490604890	0,1767514450	0,0630137901
Vertebralis_R	67	17,529	-78,384	-944,136	2,2901491260	2,3498694107	1,9505389379	2,4091445792	0,0597202848	0,3396101881	0,1189954533
