# Supplementary material for: Accurate full-scale patient-specific Circle of Willis models including aneurysms: A novel manufacturing approach
Source: PLoS One. 2025 Jul 17;20(7):e0328300. doi: 10.1371/journal.pone.0328300 (PMC12270141; doi:10.1371/journal.pone.0328300)
Supplement: S2 C2M Aneurysms — Surface deviation data from cloud-to-mesh distance calculations focused on the four aneurysm regions. (PDF) [file pone.0328300.s002.pdf]

filename	X	Y	Z	C2M signed distances
Cloud_Mesh_Distance_BA_R0_R1	17,11810303	-79,74662781	-946,2822266	-0,042106
Cloud_Mesh_Distance_BA_R0_R1	17,26747513	-79,81388855	-946,2809448	-0,033821
Cloud_Mesh_Distance_BA_R0_R1	17,4538784	-79,90048218	-946,2659302	-0,019841
Cloud_Mesh_Distance_BA_R0_R1	17,85779572	-79,9080658	-946,2620239	-0,009294
Cloud_Mesh_Distance_BA_R0_R1	16,8849926	-79,52400208	-946,2979126	-0,062431
Cloud_Mesh_Distance_BA_R0_R1	16,94293404	-79,70069885	-946,2640381	-0,056828
Cloud_Mesh_Distance_BA_R0_R1	17,346838	-79,63702393	-946,3379517	-0,028109
Cloud_Mesh_Distance_BA_R0_R1	17,58326149	-79,75593567	-946,31073	-0,017477
Cloud_Mesh_Distance_BA_R0_R1	17,80110931	-79,76974487	-946,2997437	-0,011839
Cloud_Mesh_Distance_BA_R0_R1	18,09052849	-79,66937256	-946,2974243	-0,006328
Cloud_Mesh_Distance_BA_R0_R1	16,81423759	-79,32147217	-946,3218384	-0,066575
Cloud_Mesh_Distance_BA_R0_R1	16,69632339	-79,48120117	-946,2625122	-0,071639
Cloud_Mesh_Distance_BA_R0_R1	17,08118248	-79,56887817	-946,3233032	-0,050173
Cloud_Mesh_Distance_BA_R0_R1	17,0280323	-79,36914063	-946,3521729	-0,061385
Cloud_Mesh_Distance_BA_R0_R1	17,23903465	-79,41665649	-946,3695679	-0,045486
Cloud_Mesh_Distance_BA_R0_R1	17,49660492	-79,58929443	-946,3517456	-0,025794
Cloud_Mesh_Distance_BA_R0_R1	17,44449234	-79,45980835	-946,3740234	-0,031323
Cloud_Mesh_Distance_BA_R0_R1	17,74144173	-79,60202026	-946,3400269	-0,018866
Cloud_Mesh_Distance_BA_R0_R1	17,93355751	-79,64012146	-946,3187256	-0,009359
Cloud_Mesh_Distance_BA_R0_R1	18,07460022	-79,52182007	-946,3249512	-0,005927
Cloud_Mesh_Distance_BA_R0_R1	16,97873116	-79,152771	-946,3691406	-0,072364
Cloud_Mesh_Distance_BA_R0_R1	17,62432289	-79,24711609	-946,3986816	-0,027962
Cloud_Mesh_Distance_BA_R0_R1	17,88517952	-79,34780884	-946,3695679	-0,014909
Cloud_Mesh_Distance_BA_R0_R1	17,68621826	-79,42628479	-946,3737183	-0,022247
Cloud_Mesh_Distance_BA_R0_R1	16,6149807	-79,25836182	-946,2835083	-0,073969
Cloud_Mesh_Distance_BA_R0_R1	16,77789116	-79,12034607	-946,3370972	-0,076536
Cloud_Mesh_Distance_BA_R0_R1	17,16679955	-78,97329712	-946,401062	-0,068087
Cloud_Mesh_Distance_BA_R0_R1	17,41351891	-79,23744202	-946,4004517	-0,040265
Cloud_Mesh_Distance_BA_R0_R1	17,20099068	-79,19998169	-946,3915405	-0,056476
Cloud_Mesh_Distance_BA_R0_R1	17,60133934	-79,04608154	-946,4179688	-0,03148
Cloud_Mesh_Distance_BA_R0_R1	17,82885551	-79,22875977	-946,3892822	-0,015091
Cloud_Mesh_Distance_BA_R0_R1	16,44520378	-79,10449219	-946,2480469	-0,079229
Cloud_Mesh_Distance_BA_R0_R1	16,59675407	-79,02049255	-946,3008423	-0,086753
Cloud_Mesh_Distance_BA_R0_R1	17,03464699	-78,86460876	-946,3856812	-0,084517
Cloud_Mesh_Distance_BA_R0_R1	17,02532768	-78,73765564	-946,3812256	-0,094171
Cloud_Mesh_Distance_BA_R0_R1	17,39383125	-79,02096558	-946,416626	-0,045926
Cloud_Mesh_Distance_BA_R0_R1	17,78989029	-79,0615387	-946,4087524	-0,016606
Cloud_Mesh_Distance_BA_R0_R1	17,59416962	-78,83988953	-946,4277344	-0,034005
Cloud_Mesh_Distance_BA_R0_R1	17,98932648	-78,88972473	-946,4033203	0,000461
Cloud_Mesh_Distance_BA_R0_R1	17,79456139	-78,86624146	-946,421936	-0,015198
Cloud_Mesh_Distance_BA_R0_R1	18,18618584	-78,88902283	-946,3769531	0,007558
Cloud_Mesh_Distance_BA_R0_R1	17,93667412	-79,06402588	-946,395813	-0,010064
Cloud_Mesh_Distance_BA_R0_R1	16,87363243	-78,63790894	-946,3521118	-0,113542
Cloud_Mesh_Distance_BA_R0_R1	17,18870354	-78,74047852	-946,4024658	-0,082208
Cloud_Mesh_Distance_BA_R0_R1	17,0699482	-78,58831787	-946,3793945	-0,100564
Cloud_Mesh_Distance_BA_R0_R1	17,38858032	-78,80633545	-946,4207153	-0,055549
Cloud_Mesh_Distance_BA_R0_R1	17,39488029	-78,60836792	-946,4151611	-0,06211
Cloud_Mesh_Distance_BA_R0_R1	17,97955132	-78,69032288	-946,4136963	0,00669
Cloud_Mesh_Distance_BA_R0_R1	16,91444588	-78,47149658	-946,3441162	-0,120488

Cloud_Mesh_Distance_BA_R0_R1	17,24531364	-78,56973267	-946,3988037	-0,083045
Cloud_Mesh_Distance_BA_R0_R1	17,37473488	-78,4462738	-946,3983765	-0,073638
Cloud_Mesh_Distance_BA_R0_R1	17,20547104	-78,44711304	-946,3818359	-0,097167
Cloud_Mesh_Distance_BA_R0_R1	17,58149147	-78,43571472	-946,4106445	-0,04309
Cloud_Mesh_Distance_BA_R0_R1	17,79286385	-78,66136169	-946,4257202	-0,010238
Cloud_Mesh_Distance_BA_R0_R1	17,78370476	-78,4710083	-946,4175415	-0,012487
Cloud_Mesh_Distance_BA_R0_R1	17,59065819	-78,63717651	-946,4262085	-0,034361
Cloud_Mesh_Distance_BA_R0_R1	17,00143433	-78,3445282	-946,3404541	-0,123247
Cloud_Mesh_Distance_BA_R0_R1	17,07558441	-78,43882751	-946,3641968	-0,112878
Cloud_Mesh_Distance_BA_R0_R1	17,500597	-78,29975891	-946,3870239	-0,063289
Cloud_Mesh_Distance_BA_R0_R1	18,00070381	-78,4823761	-946,4077148	0,0123
Cloud_Mesh_Distance_BA_R0_R1	17,76086235	-78,25709534	-946,3894043	-0,025885
Cloud_Mesh_Distance_BA_R0_R1	18,21472931	-78,43991089	-946,3806763	0,029327
Cloud_Mesh_Distance_BA_R0_R1	18,14311981	-78,55236816	-946,3952637	0,022743
Cloud_Mesh_Distance_BA_R0_R1	18,32985306	-78,27618408	-946,3450928	0,036775
Cloud_Mesh_Distance_BA_R0_R1	17,94885826	-78,31613159	-946,3939209	0,002722
Cloud_Mesh_Distance_BA_R0_R1	18,14945412	-78,30331421	-946,3762207	0,026299
Cloud_Mesh_Distance_BA_R0_R1	16,97442818	-81,82469177	-946,128479	0,245784
Cloud_Mesh_Distance_BA_R0_R1	17,20096016	-81,83776855	-946,1506958	0,20162
Cloud_Mesh_Distance_BA_R0_R1	16,80259895	-81,73660278	-946,0675049	0,291541
Cloud_Mesh_Distance_BA_R0_R1	17,47127533	-81,79803467	-946,1530151	0,132347
Cloud_Mesh_Distance_BA_R0_R1	17,73038101	-81,75949097	-946,1571655	0,162129
Cloud_Mesh_Distance_BA_R0_R1	18,11284447	-81,63916016	-946,1616211	0,116129
Cloud_Mesh_Distance_BA_R0_R1	17,97980118	-81,79953003	-946,1832275	0,167873
Cloud_Mesh_Distance_BA_R0_R1	18,16641045	-81,80673218	-946,1973877	0,173951
Cloud_Mesh_Distance_BA_R0_R1	19,07295609	-81,70309448	-946,2303467	-0,133002
Cloud_Mesh_Distance_BA_R0_R1	19,24793243	-81,77330017	-946,2453003	-0,245442
Cloud_Mesh_Distance_BA_R0_R1	19,84938812	-81,79022217	-946,2232666	-0,280343
Cloud_Mesh_Distance_BA_R0_R1	19,65701675	-81,76702881	-946,2329102	-0,213571
Cloud_Mesh_Distance_BA_R0_R1	20,05491829	-81,8102417	-946,2029419	0,325533
Cloud_Mesh_Distance_BA_R0_R1	18,49142456	-81,50312805	-946,1708984	0,025143
Cloud_Mesh_Distance_BA_R0_R1	18,30960846	-81,68101501	-946,1851196	0,074955
Cloud_Mesh_Distance_BA_R0_R1	18,51367188	-81,75773621	-946,2128906	0,189315
Cloud_Mesh_Distance_BA_R0_R1	18,70897865	-81,62083435	-946,2036133	-0,069691
Cloud_Mesh_Distance_BA_R0_R1	18,86992073	-81,71331787	-946,2261353	-0,174972
Cloud_Mesh_Distance_BA_R0_R1	19,79329491	-81,62579346	-946,1853027	-0,107508
Cloud_Mesh_Distance_BA_R0_R1	19,97675323	-81,6416626	-946,1650391	-0,14548
Cloud_Mesh_Distance_BA_R0_R1	20,16671371	-81,63394165	-946,1268921	-0,152693
Cloud_Mesh_Distance_BA_R0_R1	18,36921501	-81,28512573	-946,130188	0,051969
Cloud_Mesh_Distance_BA_R0_R1	18,56765747	-81,29588318	-946,1472778	0,026794
Cloud_Mesh_Distance_BA_R0_R1	18,72732544	-81,39604187	-946,1694336	0,003236
Cloud_Mesh_Distance_BA_R0_R1	18,9382782	-81,50991821	-946,1952515	-0,018742
Cloud_Mesh_Distance_BA_R0_R1	19,14130402	-81,51779175	-946,1986084	-0,028062
Cloud_Mesh_Distance_BA_R0_R1	19,44914436	-81,72564697	-946,2324219	-0,143959
Cloud_Mesh_Distance_BA_R0_R1	19,28313446	-81,60437012	-946,2122192	-0,079958
Cloud_Mesh_Distance_BA_R0_R1	19,62516594	-81,61338806	-946,1975708	-0,061968
Cloud_Mesh_Distance_BA_R0_R1	19,49939728	-81,48999023	-946,1799927	-0,036995
Cloud_Mesh_Distance_BA_R0_R1	19,91738129	-81,48867798	-946,1324463	-0,026321
Cloud_Mesh_Distance_BA_R0_R1	19,28345299	-81,26304626	-946,1489868	-0,031328
Cloud_Mesh_Distance_BA_R0_R1	19,45669937	-81,29315186	-946,1428223	-0,035005

Cloud_Mesh_Distance_BA_R0_R1	19,2959137	-81,41986084	-946,1769409	-0,032478
Cloud_Mesh_Distance_BA_R0_R1	18,03597832	-81,02572632	-946,0737915	0,097037
Cloud_Mesh_Distance_BA_R0_R1	18,27897835	-81,03904724	-946,1002197	0,067065
Cloud_Mesh_Distance_BA_R0_R1	18,70657158	-80,95440674	-946,1192017	0,016099
Cloud_Mesh_Distance_BA_R0_R1	18,48181152	-80,70643616	-946,1083374	0,031135
Cloud_Mesh_Distance_BA_R0_R1	19,11553383	-80,88703918	-946,104126	-0,021539
Cloud_Mesh_Distance_BA_R0_R1	17,71763229	-80,43556213	-946,1060181	0,026327
Cloud_Mesh_Distance_BA_R0_R1	17,52411461	-80,60441589	-946,0394287	0,052379
Cloud_Mesh_Distance_BA_R0_R1	18,51395988	-80,35873413	-946,1296997	0,00882
Cloud_Mesh_Distance_BA_R0_R1	17,09119987	-80,32189941	-946,0495606	-0,016881
Cloud_Mesh_Distance_BA_R0_R1	17,24750137	-80,23651123	-946,1154785	-0,014529
Cloud_Mesh_Distance_BA_R0_R1	17,44861984	-80,36747742	-946,09729	0,009874
Cloud_Mesh_Distance_BA_R0_R1	17,6326561	-80,20362854	-946,1705322	-0,000894
Cloud_Mesh_Distance_BA_R0_R1	17,84723473	-80,20397949	-946,1773071	0,004285
Cloud_Mesh_Distance_BA_R0_R1	17,96319389	-80,36434937	-946,1367798	0,019076
Cloud_Mesh_Distance_BA_R0_R1	18,13555145	-80,30934143	-946,1501465	0,011638
Cloud_Mesh_Distance_BA_R0_R1	18,25821304	-80,16455078	-946,1747437	0,002317
Cloud_Mesh_Distance_BA_R0_R1	16,85757446	-80,00105286	-946,1304932	-0,055308
Cloud_Mesh_Distance_BA_R0_R1	17,05732536	-80,14401245	-946,1177368	-0,03332
Cloud_Mesh_Distance_BA_R0_R1	17,30292702	-79,95802307	-946,2347412	-0,025501
Cloud_Mesh_Distance_BA_R0_R1	17,5886631	-80,03636169	-946,2259522	-0,009842
Cloud_Mesh_Distance_BA_R0_R1	17,41571045	-80,11514282	-946,1862793	-0,01505
Cloud_Mesh_Distance_BA_R0_R1	18,05335999	-80,17453003	-946,1825562	0,003323
Cloud_Mesh_Distance_BA_R0_R1	18,15965462	-79,9883728	-946,2211914	0,00027
Cloud_Mesh_Distance_BA_R0_R1	18,38532257	-79,96098328	-946,2044678	-0,003792
Cloud_Mesh_Distance_BA_R0_R1	18,44693184	-80,17437744	-946,15979	-0,000766
Cloud_Mesh_Distance_BA_R0_R1	18,60126305	-79,99987793	-946,1711426	-0,00483
Cloud_Mesh_Distance_BA_R0_R1	16,77066612	-79,66653442	-946,2324829	-0,066981
Cloud_Mesh_Distance_BA_R0_R1	16,98892021	-79,86131287	-946,2200928	-0,048401
Cloud_Mesh_Distance_BA_R0_R1	16,84462547	-79,81427002	-946,2032471	-0,058602
Cloud_Mesh_Distance_BA_R0_R1	17,04893684	-79,9940033	-946,180603	-0,042031
Cloud_Mesh_Distance_BA_R0_R1	17,20651436	-80,05680847	-946,1826172	-0,029844
Cloud_Mesh_Distance_BA_R0_R1	17,14993668	-79,90176392	-946,234314	-0,035646
Cloud_Mesh_Distance_BA_R0_R1	17,6812973	-79,90675354	-946,2675171	-0,011783
Cloud_Mesh_Distance_BA_R0_R1	17,95631599	-80,02311707	-946,225769	-0,005451
Cloud_Mesh_Distance_BA_R0_R1	17,76406479	-80,04647827	-946,223877	-0,00476
Cloud_Mesh_Distance_BA_R0_R1	18,01416588	-79,81492615	-946,2750855	-0,004497
Cloud_Mesh_Distance_BA_R0_R1	18,20044518	-79,81381226	-946,2566528	-0,004101
Cloud_Mesh_Distance_BA_R0_R1	16,48937416	-79,76269531	-946,1048584	-0,082201
Cloud_Mesh_Distance_BA_R0_R1	16,58784294	-79,62216187	-946,1901245	-0,07497
Cloud_Mesh_Distance_BA_R0_R1	16,68409348	-79,82278442	-946,1524658	-0,071285
Cloud_Mesh_Distance_BA_R0_R1	18,37962532	-79,75134277	-946,2444458	-0,002912
Cloud_Mesh_Distance_BA_R0_R1	18,5830307	-79,80351257	-946,2042847	-0,005882
Cloud_Mesh_Distance_BA_R0_R1	18,79309845	-79,86949158	-946,1585693	-0,012886
Cloud_Mesh_Distance_BA_R0_R1	16,40286827	-79,55635071	-946,1389771	-0,08158
Cloud_Mesh_Distance_BA_R0_R1	16,49029541	-79,4241333	-946,2096558	-0,074811
Cloud_Mesh_Distance_BA_R0_R1	17,87546158	-79,47871399	-946,3515625	-0,013821
Cloud_Mesh_Distance_BA_R0_R1	18,25606728	-79,38995361	-946,3173218	-0,00424
Cloud_Mesh_Distance_BA_R0_R1	18,22070885	-79,62333679	-946,289856	-0,002889
Cloud_Mesh_Distance_BA_R0_R1	18,44492722	-79,34977722	-946,289917	-0,002039

Cloud_Mesh_Distance_BA_R0_R1	18,3875103	-79,53663635	-946,27771	-0,001472
Cloud_Mesh_Distance_BA_R0_R1	18,57666206	-79,59754944	-946,236084	-0,004459
Cloud_Mesh_Distance_BA_R0_R1	18,58005905	-79,402771	-946,2583008	-0,002771
Cloud_Mesh_Distance_BA_R0_R1	18,79270172	-79,63427734	-946,1878662	-0,010377
Cloud_Mesh_Distance_BA_R0_R1	19,03378105	-79,56744385	-946,1362305	-0,020925
Cloud_Mesh_Distance_BA_R0_R1	18,95085335	-79,76226807	-946,1384888	-0,020122
Cloud_Mesh_Distance_BA_R0_R1	16,16202736	-79,29251099	-946,0875855	-0,087301
Cloud_Mesh_Distance_BA_R0_R1	16,42078781	-79,26625061	-946,2163696	-0,077917
Cloud_Mesh_Distance_BA_R0_R1	16,31531906	-79,37187195	-946,1488648	-0,087044
Cloud_Mesh_Distance_BA_R0_R1	18,05532265	-79,35627747	-946,3494263	-0,009186
Cloud_Mesh_Distance_BA_R0_R1	18,06278801	-79,15983582	-946,3718872	-0,006157
Cloud_Mesh_Distance_BA_R0_R1	18,21032333	-79,18516541	-946,3477783	-0,001847
Cloud_Mesh_Distance_BA_R0_R1	18,95637703	-79,42453003	-946,1680908	-0,015061
Cloud_Mesh_Distance_BA_R0_R1	18,75531769	-79,42663574	-946,2184448	-0,005174
Cloud_Mesh_Distance_BA_R0_R1	18,72192764	-79,27142334	-946,2391968	-0,003785
Cloud_Mesh_Distance_BA_R0_R1	19,15747452	-79,40077209	-946,1099243	-0,030138
Cloud_Mesh_Distance_BA_R0_R1	16,27168083	-79,18460083	-946,1643677	-0,081852
Cloud_Mesh_Distance_BA_R0_R1	16,24666023	-79,0072937	-946,1733398	-0,0852
Cloud_Mesh_Distance_BA_R0_R1	18,58077621	-79,01985168	-946,2883301	0,003332
Cloud_Mesh_Distance_BA_R0_R1	18,40838242	-79,1860199	-946,3132935	0,001765
Cloud_Mesh_Distance_BA_R0_R1	18,37210274	-78,95585632	-946,3380127	0,006968
Cloud_Mesh_Distance_BA_R0_R1	18,58769035	-79,21958923	-946,2738648	0,000093
Cloud_Mesh_Distance_BA_R0_R1	18,77276421	-79,07774353	-946,237793	-0,003232
Cloud_Mesh_Distance_BA_R0_R1	18,90184021	-79,22546387	-946,196167	-0,008625
Cloud_Mesh_Distance_BA_R0_R1	19,08540916	-79,24621582	-946,1394653	-0,022905
Cloud_Mesh_Distance_BA_R0_R1	16,12274742	-78,90055847	-946,112793	-0,092675
Cloud_Mesh_Distance_BA_R0_R1	16,92055702	-78,98657227	-946,3693237	-0,084444
Cloud_Mesh_Distance_BA_R0_R1	16,89972496	-78,80029297	-946,3648682	-0,101721
Cloud_Mesh_Distance_BA_R0_R1	18,14337921	-79,02268982	-946,3726807	0,000273
Cloud_Mesh_Distance_BA_R0_R1	18,57794762	-78,81045532	-946,2992554	0,009907
Cloud_Mesh_Distance_BA_R0_R1	18,45204163	-78,74624634	-946,331543	0,015169
Cloud_Mesh_Distance_BA_R0_R1	18,74432373	-78,8639679	-946,2527466	0,000916
Cloud_Mesh_Distance_BA_R0_R1	18,90840149	-78,89634705	-946,2033691	-0,007422
Cloud_Mesh_Distance_BA_R0_R1	16,09254265	-78,67037964	-946,0855713	-0,096191
Cloud_Mesh_Distance_BA_R0_R1	16,31512642	-78,74472046	-946,2057495	-0,099519
Cloud_Mesh_Distance_BA_R0_R1	16,3609066	-78,9072113	-946,2305908	-0,087991
Cloud_Mesh_Distance_BA_R0_R1	16,55900383	-78,79907227	-946,2929077	-0,099757
Cloud_Mesh_Distance_BA_R0_R1	16,76375198	-78,92636108	-946,3421631	-0,088976
Cloud_Mesh_Distance_BA_R0_R1	16,65049553	-78,67042542	-946,3084717	-0,113047
Cloud_Mesh_Distance_BA_R0_R1	16,74902534	-78,78518677	-946,3363648	-0,102813
Cloud_Mesh_Distance_BA_R0_R1	18,14299965	-78,71798706	-946,3925781	0,01412
Cloud_Mesh_Distance_BA_R0_R1	18,29726219	-78,762146	-946,364563	0,015898
Cloud_Mesh_Distance_BA_R0_R1	18,7245636	-78,66339111	-946,2600098	0,010092
Cloud_Mesh_Distance_BA_R0_R1	16,31759834	-78,27970886	-946,1469116	-0,112217
Cloud_Mesh_Distance_BA_R0_R1	16,29445839	-78,54977417	-946,1796265	-0,107044
Cloud_Mesh_Distance_BA_R0_R1	16,48435783	-78,59321594	-946,2560425	-0,11119
Cloud_Mesh_Distance_BA_R0_R1	16,4499321	-78,38136292	-946,2178345	-0,120977
Cloud_Mesh_Distance_BA_R0_R1	18,27984619	-78,57827759	-946,3729248	0,026881
Cloud_Mesh_Distance_BA_R0_R1	18,38750267	-78,42202759	-946,3460083	0,033805
Cloud_Mesh_Distance_BA_R0_R1	18,42350006	-78,59396362	-946,3413086	0,024501

Cloud_Mesh_Distance_BA_R0_R1	18,56993675	-78,61836243	-946,3042603	0,019675
Cloud_Mesh_Distance_BA_R0_R1	18,52536392	-78,46392822	-946,3138428	0,029482
Cloud_Mesh_Distance_BA_R0_R1	18,86124039	-78,52896118	-946,2131348	0,007114
Cloud_Mesh_Distance_BA_R0_R1	16,70018387	-78,4566803	-946,3001099	-0,126551
Cloud_Mesh_Distance_BA_R0_R1	17,15626335	-78,31590271	-946,3572388	-0,111216
Cloud_Mesh_Distance_BA_R0_R1	17,26487541	-78,16212463	-946,3391724	-0,106667
Cloud_Mesh_Distance_BA_R0_R1	17,32438087	-78,30545044	-946,373291	-0,090296
Cloud_Mesh_Distance_BA_R0_R1	17,4524498	-78,15386963	-946,3532105	-0,081627
Cloud_Mesh_Distance_BA_R0_R1	18,53178978	-78,25909424	-946,2936401	0,032907
Cloud_Mesh_Distance_BA_R0_R1	18,68821907	-78,45013428	-946,2658081	0,022542
Cloud_Mesh_Distance_BA_R0_R1	16,47412109	-78,04318237	-946,1525879	-0,115248
Cloud_Mesh_Distance_BA_R0_R1	16,46996689	-78,18345642	-946,1871338	-0,118808
Cloud_Mesh_Distance_BA_R0_R1	16,87063026	-78,23095703	-946,3002319	-0,133668
Cloud_Mesh_Distance_BA_R0_R1	16,78886414	-77,99252319	-946,2318115	-0,128497
Cloud_Mesh_Distance_BA_R0_R1	16,64403915	-78,21536255	-946,2470703	-0,131558
Cloud_Mesh_Distance_BA_R0_R1	17,08176422	-78,18612671	-946,3233643	-0,12561
Cloud_Mesh_Distance_BA_R0_R1	17,01934433	-78,0227356	-946,2783203	-0,129433
Cloud_Mesh_Distance_BA_R0_R1	17,19063377	-78,0413208	-946,3027954	-0,120934
Cloud_Mesh_Distance_BA_R0_R1	17,60504341	-78,01794434	-946,3245239	-0,072011
Cloud_Mesh_Distance_BA_R0_R1	17,89282799	-78,18510437	-946,3722534	-0,009019
Cloud_Mesh_Distance_BA_R0_R1	17,63753128	-78,22492981	-946,380127	-0,046433
Cloud_Mesh_Distance_BA_R0_R1	17,987957	-78,06768799	-946,3371582	-0,008531
Cloud_Mesh_Distance_BA_R0_R1	18,24641037	-78,16647339	-946,3380737	0,026738
Cloud_Mesh_Distance_BA_R0_R1	18,07087326	-78,17163086	-946,3583984	0,011581
Cloud_Mesh_Distance_BA_R0_R1	18,3931694	-78,07542419	-946,289978	0,031906
Cloud_Mesh_Distance_BA_R0_R1	18,17119598	-78,01899719	-946,3081055	0,009853
Cloud_Mesh_Distance_BA_R0_R1	18,73088074	-78,27445984	-946,2363281	0,023746
Cloud_Mesh_Distance_BA_R0_R1	16,61013985	-77,92707825	-946,1647949	-0,120797
Cloud_Mesh_Distance_BA_R0_R1	16,59718704	-78,06851196	-946,201294	-0,124416
Cloud_Mesh_Distance_BA_R0_R1	16,98794365	-77,83204651	-946,218811	-0,12734
Cloud_Mesh_Distance_BA_R0_R1	17,39118767	-77,96934509	-946,2991943	-0,101788
Cloud_Mesh_Distance_BA_R0_R1	17,57077026	-77,81654358	-946,2546387	-0,089255
Cloud_Mesh_Distance_BA_R0_R1	17,80410767	-78,06230164	-946,3408203	-0,033681
Cloud_Mesh_Distance_BA_R0_R1	17,98246384	-77,91664124	-946,2888794	-0,020257
Cloud_Mesh_Distance_BA_R0_R1	18,56012344	-78,07354736	-946,2514648	0,033834
Cloud_Mesh_Distance_BA_R0_R1	18,64745331	-77,98905945	-946,2054443	0,027487
Cloud_Mesh_Distance_BA_R0_R1	16,78327751	-77,79386902	-946,1691284	-0,114143
Cloud_Mesh_Distance_BA_R0_R1	17,13151169	-77,71604919	-946,1930542	-0,123186
Cloud_Mesh_Distance_BA_R0_R1	16,93652344	-77,67518616	-946,1525269	-0,114926
Cloud_Mesh_Distance_BA_R0_R1	17,1985569	-77,87133789	-946,2546387	-0,119549
Cloud_Mesh_Distance_BA_R0_R1	17,7635994	-77,89308167	-946,2852783	-0,055432
Cloud_Mesh_Distance_BA_R0_R1	17,05619431	-77,58584595	-946,1271362	-0,123245
Cloud_Mesh_Distance_BA_R0_R1	16,60453606	-81,64573669	-945,9834595	0,380166
Cloud_Mesh_Distance_BA_R0_R1	17,33550262	-81,59225464	-946,0734863	0,127324
Cloud_Mesh_Distance_BA_R0_R1	17,03084183	-81,62182617	-946,0483398	0,137332
Cloud_Mesh_Distance_BA_R0_R1	20,6902771	-81,87121582	-946,0697632	0,284728
Cloud_Mesh_Distance_BA_R0_R1	16,4201889	-81,56433105	-945,8809815	0,395407
Cloud_Mesh_Distance_BA_R0_R1	16,60219193	-81,46377563	-945,888855	0,298633
Cloud_Mesh_Distance_BA_R0_R1	16,79265976	-81,54217529	-945,9733276	0,224903
Cloud_Mesh_Distance_BA_R0_R1	16,94360924	-81,43225098	-945,9603882	0,199504

Cloud_Mesh_Distance_BA_R0_R1	17,560112	-81,63053894	-946,1083984	0,1079
Cloud_Mesh_Distance_BA_R0_R1	17,37787628	-81,3973999	-946,0219116	0,181588
Cloud_Mesh_Distance_BA_R0_R1	17,71784019	-81,53474426	-946,0996094	0,117422
Cloud_Mesh_Distance_BA_R0_R1	20,30012894	-81,81530762	-946,159668	0,307739
Cloud_Mesh_Distance_BA_R0_R1	20,37156868	-81,58239746	-946,0592041	0,0454
Cloud_Mesh_Distance_BA_R0_R1	20,53255653	-81,73223877	-946,0647583	0,167136
Cloud_Mesh_Distance_BA_R0_R1	16,7794857	-81,3553772	-945,8959351	0,266082
Cloud_Mesh_Distance_BA_R0_R1	17,5240097	-81,29455566	-946,0224609	0,174549
Cloud_Mesh_Distance_BA_R0_R1	17,53417397	-81,47143555	-946,0623779	0,147082
Cloud_Mesh_Distance_BA_R0_R1	18,05460167	-81,47232056	-946,1247559	0,084006
Cloud_Mesh_Distance_BA_R0_R1	17,91440964	-81,59651184	-946,1341553	0,090751
Cloud_Mesh_Distance_BA_R0_R1	18,24331284	-81,47668457	-946,1452026	0,058707
Cloud_Mesh_Distance_BA_R0_R1	19,72910309	-81,47268677	-946,1547241	-0,033464
Cloud_Mesh_Distance_BA_R0_R1	20,05238914	-81,36978149	-946,0756226	-0,026223
Cloud_Mesh_Distance_BA_R0_R1	20,21678734	-81,44873047	-946,05896	-0,016816
Cloud_Mesh_Distance_BA_R0_R1	20,06868553	-81,51426697	-946,1123657	-0,027169
Cloud_Mesh_Distance_BA_R0_R1	16,79023743	-81,16033936	-945,8461304	0,297043
Cloud_Mesh_Distance_BA_R0_R1	16,96845818	-81,25759888	-945,9127197	0,284838
Cloud_Mesh_Distance_BA_R0_R1	17,32805443	-81,2348175	-945,9788818	0,213924
Cloud_Mesh_Distance_BA_R0_R1	17,16085434	-81,38952637	-945,9853516	0,212417
Cloud_Mesh_Distance_BA_R0_R1	17,25139809	-81,05543518	-945,9434815	0,197939
Cloud_Mesh_Distance_BA_R0_R1	17,67605972	-81,37715149	-946,0604858	0,145555
Cloud_Mesh_Distance_BA_R0_R1	17,9108429	-81,16876221	-946,0653076	0,118499
Cloud_Mesh_Distance_BA_R0_R1	17,71387291	-81,20932007	-946,0395508	0,145994
Cloud_Mesh_Distance_BA_R0_R1	18,11407089	-81,26754761	-946,1008911	0,08841
Cloud_Mesh_Distance_BA_R0_R1	17,87582779	-81,37530518	-946,0863037	0,115958
Cloud_Mesh_Distance_BA_R0_R1	18,92299843	-81,03010559	-946,1270752	-0,001965
Cloud_Mesh_Distance_BA_R0_R1	18,93155289	-81,26496887	-946,1569824	-0,009723
Cloud_Mesh_Distance_BA_R0_R1	19,12278366	-81,35029602	-946,1691284	-0,02337
Cloud_Mesh_Distance_BA_R0_R1	19,39582825	-81,12472534	-946,1170044	-0,033677
Cloud_Mesh_Distance_BA_R0_R1	19,6078701	-81,14627075	-946,0976563	-0,038883
Cloud_Mesh_Distance_BA_R0_R1	19,81597137	-81,16744995	-946,071228	-0,038305
Cloud_Mesh_Distance_BA_R0_R1	19,66470528	-81,31411743	-946,1264648	-0,033648
Cloud_Mesh_Distance_BA_R0_R1	19,86943626	-81,33093262	-946,1002808	-0,032055
Cloud_Mesh_Distance_BA_R0_R1	20,01358795	-81,19981384	-946,0401611	-0,034973
Cloud_Mesh_Distance_BA_R0_R1	20,20498085	-81,256073	-946,0077515	-0,027704
Cloud_Mesh_Distance_BA_R0_R1	17,14488602	-81,1946106	-945,9373779	0,248405
Cloud_Mesh_Distance_BA_R0_R1	17,01488876	-81,04469299	-945,8901367	0,218499
Cloud_Mesh_Distance_BA_R0_R1	17,77323151	-80,97105408	-946,036499	0,12156
Cloud_Mesh_Distance_BA_R0_R1	17,49951935	-81,06962585	-945,9918823	0,166887
Cloud_Mesh_Distance_BA_R0_R1	18,52249336	-81,11016846	-946,1245728	0,035761
Cloud_Mesh_Distance_BA_R0_R1	18,49298096	-80,89321899	-946,1096191	0,034185
Cloud_Mesh_Distance_BA_R0_R1	18,72920036	-81,17642212	-946,1408691	0,012429
Cloud_Mesh_Distance_BA_R0_R1	19,15573311	-81,12353516	-946,1321411	-0,023881
Cloud_Mesh_Distance_BA_R0_R1	19,54611206	-80,97358704	-946,0742188	-0,03788
Cloud_Mesh_Distance_BA_R0_R1	19,95518112	-81,02827454	-946,0125732	-0,042235
Cloud_Mesh_Distance_BA_R0_R1	20,15166283	-81,06625366	-945,9732056	-0,038171
Cloud_Mesh_Distance_BA_R0_R1	17,38629723	-80,71942139	-945,9923096	0,081809
Cloud_Mesh_Distance_BA_R0_R1	17,54405785	-80,82730103	-946,0059815	0,108111
Cloud_Mesh_Distance_BA_R0_R1	17,34168434	-80,88357544	-945,9633179	0,136701

Cloud_Mesh_Distance_BA_R0_R1	19,3296051	-80,94180298	-946,0939941	-0,031319
Cloud_Mesh_Distance_BA_R0_R1	19,28270721	-80,74850464	-946,0736694	-0,030294
Cloud_Mesh_Distance_BA_R0_R1	19,69159889	-80,83248901	-946,0266113	-0,046471
Cloud_Mesh_Distance_BA_R0_R1	19,48898315	-80,79066467	-946,0521851	-0,040078
Cloud_Mesh_Distance_BA_R0_R1	19,88360023	-80,86276245	-945,9935913	-0,046345
Cloud_Mesh_Distance_BA_R0_R1	19,75452042	-81,0009613	-946,0463867	-0,043901
Cloud_Mesh_Distance_BA_R0_R1	20,07718468	-80,887146	-945,9519653	-0,04863
Cloud_Mesh_Distance_BA_R0_R1	16,98854256	-80,8200531	-945,8876343	0,122448
Cloud_Mesh_Distance_BA_R0_R1	16,97528839	-80,61036682	-945,9224243	0,044363
Cloud_Mesh_Distance_BA_R0_R1	16,79279137	-80,71090698	-945,8476563	0,07747
Cloud_Mesh_Distance_BA_R0_R1	17,32496452	-80,55212402	-946,0185547	0,041569
Cloud_Mesh_Distance_BA_R0_R1	18,14161873	-80,86529541	-946,085083	0,068892
Cloud_Mesh_Distance_BA_R0_R1	17,95653343	-80,8001709	-946,0689697	0,076883
Cloud_Mesh_Distance_BA_R0_R1	17,9326458	-80,57600403	-946,0934448	0,047152
Cloud_Mesh_Distance_BA_R0_R1	18,13310814	-80,67584229	-946,0948486	0,049666
Cloud_Mesh_Distance_BA_R0_R1	18,31037903	-80,79153442	-946,0993652	0,05014
Cloud_Mesh_Distance_BA_R0_R1	18,34954834	-80,55792236	-946,1138916	0,029137
Cloud_Mesh_Distance_BA_R0_R1	18,59909248	-80,53799438	-946,1130371	0,013322
Cloud_Mesh_Distance_BA_R0_R1	18,66779137	-80,73599243	-946,109314	0,016628
Cloud_Mesh_Distance_BA_R0_R1	18,90904045	-80,77285767	-946,1043701	-0,00298
Cloud_Mesh_Distance_BA_R0_R1	19,03629875	-80,55654907	-946,0856323	-0,020166
Cloud_Mesh_Distance_BA_R0_R1	19,11050034	-80,70582581	-946,0864258	-0,021331
Cloud_Mesh_Distance_BA_R0_R1	19,43624306	-80,5977478	-946,0368042	-0,042742
Cloud_Mesh_Distance_BA_R0_R1	16,7563839	-80,31674194	-945,960083	-0,035191
Cloud_Mesh_Distance_BA_R0_R1	16,78261757	-80,50790405	-945,8961792	0,006048
Cloud_Mesh_Distance_BA_R0_R1	17,73644257	-80,7021637	-946,0521851	0,073048
Cloud_Mesh_Distance_BA_R0_R1	18,13116264	-80,47421265	-946,1204834	0,026386
Cloud_Mesh_Distance_BA_R0_R1	18,3017807	-80,34858704	-946,1394043	0,013246
Cloud_Mesh_Distance_BA_R0_R1	18,82555389	-80,54624939	-946,102478	-0,003858
Cloud_Mesh_Distance_BA_R0_R1	19,2317009	-80,56782532	-946,0636597	-0,031332
Cloud_Mesh_Distance_BA_R0_R1	16,60151863	-80,39826965	-945,8723145	-0,030465
Cloud_Mesh_Distance_BA_R0_R1	16,58807755	-80,20300293	-945,9515381	-0,061816
Cloud_Mesh_Distance_BA_R0_R1	16,42276955	-80,08520508	-945,9373169	-0,081234
Cloud_Mesh_Distance_BA_R0_R1	16,90663719	-80,22592163	-946,0440674	-0,037746
Cloud_Mesh_Distance_BA_R0_R1	16,94078445	-80,4147644	-945,9754028	-0,005794
Cloud_Mesh_Distance_BA_R0_R1	17,13197517	-80,50090027	-945,9935303	0,020576
Cloud_Mesh_Distance_BA_R0_R1	17,25222588	-80,39683533	-946,0550537	0,006351
Cloud_Mesh_Distance_BA_R0_R1	18,62563133	-80,18133545	-946,1419067	-0,004823
Cloud_Mesh_Distance_BA_R0_R1	18,76444626	-80,3203125	-946,1147461	-0,008256
Cloud_Mesh_Distance_BA_R0_R1	19,17951393	-80,39257813	-946,0614014	-0,030882
Cloud_Mesh_Distance_BA_R0_R1	18,98979378	-80,36393738	-946,0883789	-0,021592
Cloud_Mesh_Distance_BA_R0_R1	18,97900009	-80,16186523	-946,0968018	-0,025004
Cloud_Mesh_Distance_BA_R0_R1	19,37108421	-80,40959167	-946,0310059	-0,045403
Cloud_Mesh_Distance_BA_R0_R1	19,79457474	-80,34614563	-945,9327393	-0,065654
Cloud_Mesh_Distance_BA_R0_R1	16,28314972	-79,97741699	-945,9187012	-0,09782
Cloud_Mesh_Distance_BA_R0_R1	16,60157204	-79,99052429	-946,053833	-0,072912
Cloud_Mesh_Distance_BA_R0_R1	16,73309898	-80,13986206	-946,0310669	-0,057424
Cloud_Mesh_Distance_BA_R0_R1	18,79618454	-80,07998657	-946,1325073	-0,011157
Cloud_Mesh_Distance_BA_R0_R1	19,16245651	-80,20834351	-946,0628052	-0,03562
Cloud_Mesh_Distance_BA_R0_R1	18,9751873	-79,95697021	-946,1134644	-0,025048

Cloud_Mesh_Distance_BA_R0_R1	19,48193741	-80,23014832	-945,998291	-0,055503
Cloud_Mesh_Distance_BA_R0_R1	19,30819511	-80,25840759	-946,0363159	-0,043794
Cloud_Mesh_Distance_BA_R0_R1	16,38319778	-79,88919067	-946,0072632	-0,087897
Cloud_Mesh_Distance_BA_R0_R1	16,21730232	-79,80883789	-945,9560547	-0,099084
Cloud_Mesh_Distance_BA_R0_R1	19,3594017	-80,06806946	-946,0216675	-0,052908
Cloud_Mesh_Distance_BA_R0_R1	19,16392899	-80,00862122	-946,0682373	-0,037536
Cloud_Mesh_Distance_BA_R0_R1	19,34429169	-79,85147095	-946,0288696	-0,050402
Cloud_Mesh_Distance_BA_R0_R1	19,57278824	-80,04516602	-945,9666748	-0,06722
Cloud_Mesh_Distance_BA_R0_R1	19,49273491	-79,90452576	-945,9853516	-0,064926
Cloud_Mesh_Distance_BA_R0_R1	16,05957603	-79,74281311	-945,8880615	-0,113103
Cloud_Mesh_Distance_BA_R0_R1	16,22893906	-79,4866333	-946,0760498	-0,08917
Cloud_Mesh_Distance_BA_R0_R1	16,30964851	-79,68640137	-946,0523071	-0,094093
Cloud_Mesh_Distance_BA_R0_R1	19,15151215	-79,78469849	-946,0858765	-0,035176
Cloud_Mesh_Distance_BA_R0_R1	19,18662834	-79,58004761	-946,0898438	-0,035664
Cloud_Mesh_Distance_BA_R0_R1	19,34870338	-79,647995	-946,0345459	-0,050893
Cloud_Mesh_Distance_BA_R0_R1	19,55550194	-79,70806885	-945,9650879	-0,068642
Cloud_Mesh_Distance_BA_R0_R1	15,8298521	-79,41540527	-945,8270874	-0,111435
Cloud_Mesh_Distance_BA_R0_R1	16,06652451	-79,41082764	-946,001648	-0,098497
Cloud_Mesh_Distance_BA_R0_R1	16,1395092	-79,60809326	-945,9868774	-0,102448
Cloud_Mesh_Distance_BA_R0_R1	19,50052071	-79,5335083	-945,9848633	-0,062406
Cloud_Mesh_Distance_BA_R0_R1	15,91960526	-79,33590698	-945,9181519	-0,101958
Cloud_Mesh_Distance_BA_R0_R1	16,12579346	-79,10253906	-946,0982056	-0,086346
Cloud_Mesh_Distance_BA_R0_R1	19,36562729	-79,42262268	-946,0354004	-0,051124
Cloud_Mesh_Distance_BA_R0_R1	15,88031006	-79,14457703	-945,9227905	-0,09574
Cloud_Mesh_Distance_BA_R0_R1	15,73463821	-79,05169678	-945,8042603	-0,097711
Cloud_Mesh_Distance_BA_R0_R1	16,01895332	-79,2191925	-946,0131836	-0,098484
Cloud_Mesh_Distance_BA_R0_R1	18,94328499	-79,04270935	-946,1900024	-0,01208
Cloud_Mesh_Distance_BA_R0_R1	19,19118118	-78,97613525	-946,1044312	-0,029105
Cloud_Mesh_Distance_BA_R0_R1	19,06277466	-79,08862305	-946,1505737	-0,021073
Cloud_Mesh_Distance_BA_R0_R1	19,26940155	-79,24455261	-946,0749512	-0,037491
Cloud_Mesh_Distance_BA_R0_R1	19,18468094	-79,12081909	-946,1077271	-0,030073
Cloud_Mesh_Distance_BA_R0_R1	19,31030083	-79,05265808	-946,0582886	-0,042763
Cloud_Mesh_Distance_BA_R0_R1	19,44306183	-79,19319153	-946,0070801	-0,055657
Cloud_Mesh_Distance_BA_R0_R1	15,94460773	-78,84187317	-945,9932861	-0,09284
Cloud_Mesh_Distance_BA_R0_R1	15,98412037	-79,0239563	-946,0150757	-0,09059
Cloud_Mesh_Distance_BA_R0_R1	19,05861282	-78,92419434	-946,1530762	-0,018453
Cloud_Mesh_Distance_BA_R0_R1	19,19757652	-78,78988647	-946,097168	-0,029844
Cloud_Mesh_Distance_BA_R0_R1	15,83245373	-78,90222168	-945,9020996	-0,092061
Cloud_Mesh_Distance_BA_R0_R1	15,85501862	-78,67233276	-945,9122315	-0,086785
Cloud_Mesh_Distance_BA_R0_R1	16,218853	-78,42153931	-946,1265869	-0,102174
Cloud_Mesh_Distance_BA_R0_R1	18,88044357	-78,71624756	-946,2116089	-0,001297
Cloud_Mesh_Distance_BA_R0_R1	18,99137688	-78,5942688	-946,1687012	-0,007322
Cloud_Mesh_Distance_BA_R0_R1	19,03339005	-78,75112915	-946,1588135	-0,015933
Cloud_Mesh_Distance_BA_R0_R1	16,01223946	-78,4848938	-946,0124512	-0,090357
Cloud_Mesh_Distance_BA_R0_R1	16,07245636	-78,32943726	-946,0230713	-0,094456
Cloud_Mesh_Distance_BA_R0_R1	19,1854763	-78,42074585	-946,076294	-0,020809
Cloud_Mesh_Distance_BA_R0_R1	19,14742088	-78,58494568	-946,1063843	-0,022402
Cloud_Mesh_Distance_BA_R0_R1	19,3500061	-78,66171265	-946,0230103	-0,041068
Cloud_Mesh_Distance_BA_R0_R1	19,28826141	-78,49906921	-946,0371094	-0,032864
Cloud_Mesh_Distance_BA_R0_R1	16,04301071	-78,20303345	-945,9673462	-0,090534

Cloud_Mesh_Distance_BA_R0_R1	15,94273186	-78,36213684	-945,9362183	-0,082482
Cloud_Mesh_Distance_BA_R0_R1	16,19575691	-78,22050476	-946,0708008	-0,097924
Cloud_Mesh_Distance_BA_R0_R1	18,83954048	-78,36497498	-946,2094116	0,014256
Cloud_Mesh_Distance_BA_R0_R1	19,01997185	-78,39091492	-946,1435547	-0,003544
Cloud_Mesh_Distance_BA_R0_R1	15,87966919	-78,25808716	-945,8538818	-0,072012
Cloud_Mesh_Distance_BA_R0_R1	16,09458733	-78,05545044	-945,951416	-0,097199
Cloud_Mesh_Distance_BA_R0_R1	18,80067635	-77,97990417	-946,1571655	0,02173
Cloud_Mesh_Distance_BA_R0_R1	18,69588661	-78,1242981	-946,2228394	0,02899
Cloud_Mesh_Distance_BA_R0_R1	18,8767662	-78,18948364	-946,1738892	0,017266
Cloud_Mesh_Distance_BA_R0_R1	18,96147537	-78,03656006	-946,1135254	0,013287
Cloud_Mesh_Distance_BA_R0_R1	19,05637741	-78,17120361	-946,1012573	0,003201
Cloud_Mesh_Distance_BA_R0_R1	19,26306725	-78,03857422	-945,980835	-0,014183
Cloud_Mesh_Distance_BA_R0_R1	16,18263435	-77,95100403	-945,968689	-0,102173
Cloud_Mesh_Distance_BA_R0_R1	16,43398666	-77,93167114	-946,098877	-0,112737
Cloud_Mesh_Distance_BA_R0_R1	16,24819756	-78,0453949	-946,0454102	-0,104503
Cloud_Mesh_Distance_BA_R0_R1	16,36020088	-78,087677	-946,1182251	-0,11429
Cloud_Mesh_Distance_BA_R0_R1	16,41311646	-77,72515869	-945,9983521	-0,112967
Cloud_Mesh_Distance_BA_R0_R1	18,16060638	-77,86618042	-946,256958	-0,003264
Cloud_Mesh_Distance_BA_R0_R1	18,32372665	-77,89894104	-946,2474976	0,012716
Cloud_Mesh_Distance_BA_R0_R1	18,5081768	-77,93980408	-946,2269898	0,024504
Cloud_Mesh_Distance_BA_R0_R1	18,65130806	-77,84178162	-946,1591187	0,0244
Cloud_Mesh_Distance_BA_R0_R1	19,10774422	-77,99914551	-946,0455933	0,001007
Cloud_Mesh_Distance_BA_R0_R1	16,6118679	-77,81420898	-946,1245117	-0,119892
Cloud_Mesh_Distance_BA_R0_R1	17,69870567	-77,75112915	-946,2300415	-0,079041
Cloud_Mesh_Distance_BA_R0_R1	17,43128014	-77,77435303	-946,2346191	-0,108519
Cloud_Mesh_Distance_BA_R0_R1	17,58409691	-77,64802551	-946,1864014	-0,097719
Cloud_Mesh_Distance_BA_R0_R1	17,8962059	-77,75442505	-946,2285156	-0,051266
Cloud_Mesh_Distance_BA_R0_R1	18,06964874	-77,77478027	-946,2279663	-0,023699
Cloud_Mesh_Distance_BA_R0_R1	18,23696709	-77,73516846	-946,1952515	-0,008633
Cloud_Mesh_Distance_BA_R0_R1	18,44470215	-77,77987671	-946,1827393	0,016182
Cloud_Mesh_Distance_BA_R0_R1	18,35966682	-77,615448	-946,1264648	-0,003562
Cloud_Mesh_Distance_BA_R0_R1	18,79483604	-77,84451294	-946,1192017	0,023783
Cloud_Mesh_Distance_BA_R0_R1	18,94480515	-77,85124207	-946,0710449	0,018127
Cloud_Mesh_Distance_BA_R0_R1	16,62513924	-77,69017029	-946,0721436	-0,116887
Cloud_Mesh_Distance_BA_R0_R1	16,73493576	-77,52102661	-946,0187378	-0,118857
Cloud_Mesh_Distance_BA_R0_R1	16,98054695	-77,45994568	-946,0500488	-0,118936
Cloud_Mesh_Distance_BA_R0_R1	17,22545242	-77,61149597	-946,156311	-0,122293
Cloud_Mesh_Distance_BA_R0_R1	17,29615402	-77,71844482	-946,2063599	-0,120581
Cloud_Mesh_Distance_BA_R0_R1	17,23020172	-77,47036743	-946,0876465	-0,121596
Cloud_Mesh_Distance_BA_R0_R1	17,40176582	-77,58226013	-946,1521606	-0,116475
Cloud_Mesh_Distance_BA_R0_R1	17,79140663	-77,59133911	-946,1591187	-0,075176
Cloud_Mesh_Distance_BA_R0_R1	18,05630302	-77,61315918	-946,1571045	-0,038225
Cloud_Mesh_Distance_BA_R0_R1	18,12016869	-77,4405365	-946,067627	-0,040567
Cloud_Mesh_Distance_BA_R0_R1	18,47738457	-77,5173645	-946,0595093	0,000106
Cloud_Mesh_Distance_BA_R0_R1	18,21594238	-77,56787109	-946,1226807	-0,019061
Cloud_Mesh_Distance_BA_R0_R1	18,56282043	-77,66690063	-946,1124878	0,014546
Cloud_Mesh_Distance_BA_R0_R1	18,7672596	-77,71205139	-946,0787354	0,017693
Cloud_Mesh_Distance_BA_R0_R1	18,6530323	-77,55178833	-946,0391846	0,011715
Cloud_Mesh_Distance_BA_R0_R1	18,9817524	-77,68849182	-945,9996948	0,015811
Cloud_Mesh_Distance_BA_R0_R1	17,41908264	-77,43771362	-946,0818481	-0,118685

Cloud_Mesh_Distance_BA_R0_R1	17,33473396	-77,32420349	-946,0150147	-0,11661
Cloud_Mesh_Distance_BA_R0_R1	17,59628105	-77,48408508	-946,1090088	-0,105068
Cloud_Mesh_Distance_BA_R0_R1	17,67601204	-77,36376953	-946,0458374	-0,094467
Cloud_Mesh_Distance_BA_R0_R1	18,30989075	-77,43161011	-946,0425415	-0,01693
Cloud_Mesh_Distance_BA_R0_R1	20,89645576	-81,84275818	-945,9729004	0,123328
Cloud_Mesh_Distance_BA_R0_R1	20,74406242	-81,67901611	-945,9671021	0,070583
Cloud_Mesh_Distance_BA_R0_R1	20,95323753	-81,65145874	-945,8612061	0,025397
Cloud_Mesh_Distance_BA_R0_R1	16,27859116	-81,32896423	-945,6778565	0,433404
Cloud_Mesh_Distance_BA_R0_R1	16,26579285	-81,48895264	-945,7615356	0,428765
Cloud_Mesh_Distance_BA_R0_R1	16,43065643	-81,39212036	-945,7909546	0,357625
Cloud_Mesh_Distance_BA_R0_R1	16,60156631	-81,28266907	-945,8161621	0,345188
Cloud_Mesh_Distance_BA_R0_R1	20,58401108	-81,52264404	-945,9689941	0,003734
Cloud_Mesh_Distance_BA_R0_R1	20,62504578	-81,34324646	-945,8926392	-0,011248
Cloud_Mesh_Distance_BA_R0_R1	16,43362617	-81,22108459	-945,7248535	0,356008
Cloud_Mesh_Distance_BA_R0_R1	16,28946686	-80,98086548	-945,5969849	0,175817
Cloud_Mesh_Distance_BA_R0_R1	20,41856384	-81,3470459	-945,9697266	-0,015457
Cloud_Mesh_Distance_BA_R0_R1	20,35211945	-81,11758423	-945,9261475	-0,03272
Cloud_Mesh_Distance_BA_R0_R1	20,75312233	-81,22737122	-945,8006592	-0,019807
Cloud_Mesh_Distance_BA_R0_R1	20,55853271	-81,16418457	-945,8643799	-0,028263
Cloud_Mesh_Distance_BA_R0_R1	16,43244934	-81,05206299	-945,6885986	0,239451
Cloud_Mesh_Distance_BA_R0_R1	16,59631538	-81,10327148	-945,7703857	0,276537
Cloud_Mesh_Distance_BA_R0_R1	16,56717682	-80,95600891	-945,744751	0,189685
Cloud_Mesh_Distance_BA_R0_R1	17,16152954	-80,90316772	-945,9228516	0,150974
Cloud_Mesh_Distance_BA_R0_R1	20,70825195	-80,99371338	-945,7523193	-0,038813
Cloud_Mesh_Distance_BA_R0_R1	16,44481087	-80,68943787	-945,7156372	0,050011
Cloud_Mesh_Distance_BA_R0_R1	16,43821335	-80,87927246	-945,68573	0,13837
Cloud_Mesh_Distance_BA_R0_R1	16,76834679	-80,94361877	-945,8182373	0,180447
Cloud_Mesh_Distance_BA_R0_R1	20,18306351	-80,73666382	-945,8886108	-0,0554
Cloud_Mesh_Distance_BA_R0_R1	20,27609825	-80,9203949	-945,9007568	-0,046956
Cloud_Mesh_Distance_BA_R0_R1	20,48076248	-80,95640564	-945,8374634	-0,041775
Cloud_Mesh_Distance_BA_R0_R1	20,38444138	-80,76104736	-945,8258667	-0,055325
Cloud_Mesh_Distance_BA_R0_R1	20,58472824	-80,77871704	-945,7515259	-0,055485
Cloud_Mesh_Distance_BA_R0_R1	16,60538864	-80,79550171	-945,7684937	0,108214
Cloud_Mesh_Distance_BA_R0_R1	17,19093704	-80,71311951	-945,9520264	0,082968
Cloud_Mesh_Distance_BA_R0_R1	19,64716721	-80,65013123	-946,0056763	-0,054619
Cloud_Mesh_Distance_BA_R0_R1	19,81217003	-80,71975708	-945,9834595	-0,051572
Cloud_Mesh_Distance_BA_R0_R1	19,98213577	-80,71688843	-945,9419556	-0,057171
Cloud_Mesh_Distance_BA_R0_R1	20,477911	-80,59118652	-945,7523804	-0,066592
Cloud_Mesh_Distance_BA_R0_R1	16,24172211	-80,34732056	-945,7248535	-0,06608
Cloud_Mesh_Distance_BA_R0_R1	16,4272728	-80,28030396	-945,8500366	-0,060778
Cloud_Mesh_Distance_BA_R0_R1	16,43553352	-80,48416138	-945,7713623	-0,018298
Cloud_Mesh_Distance_BA_R0_R1	16,61251831	-80,5954895	-945,8110352	0,027523
Cloud_Mesh_Distance_BA_R0_R1	19,60664558	-80,42362976	-945,9862061	-0,05781
Cloud_Mesh_Distance_BA_R0_R1	20,07320976	-80,55845642	-945,8869629	-0,06388
Cloud_Mesh_Distance_BA_R0_R1	19,84155464	-80,54103088	-945,9469605	-0,058711
Cloud_Mesh_Distance_BA_R0_R1	19,9715023	-80,37783813	-945,8875122	-0,072652
Cloud_Mesh_Distance_BA_R0_R1	20,37974167	-80,38432312	-945,7484131	-0,078122
Cloud_Mesh_Distance_BA_R0_R1	20,28087425	-80,57424927	-945,8227539	-0,067612
Cloud_Mesh_Distance_BA_R0_R1	15,94501877	-80,20907593	-945,5853272	-0,116196
Cloud_Mesh_Distance_BA_R0_R1	16,07736778	-80,12123108	-945,729187	-0,109702

Cloud_Mesh_Distance_BA_R0_R1	20,23639297	-80,2591095	-945,7807007	-0,080872
Cloud_Mesh_Distance_BA_R0_R1	20,17020988	-80,39941406	-945,8280029	-0,074926
Cloud_Mesh_Distance_BA_R0_R1	20,07471275	-80,22834778	-945,8330689	-0,081706
Cloud_Mesh_Distance_BA_R0_R1	15,98805904	-79,88000488	-945,781189	-0,120786
Cloud_Mesh_Distance_BA_R0_R1	16,1452446	-79,947052	-945,8551025	-0,106577
Cloud_Mesh_Distance_BA_R0_R1	16,26630211	-80,13366699	-945,8372803	-0,087376
Cloud_Mesh_Distance_BA_R0_R1	19,78298378	-79,99835205	-945,902771	-0,080138
Cloud_Mesh_Distance_BA_R0_R1	19,6347084	-79,88871765	-945,9423218	-0,073683
Cloud_Mesh_Distance_BA_R0_R1	19,88255692	-80,19581604	-945,8901978	-0,075685
Cloud_Mesh_Distance_BA_R0_R1	19,68511772	-80,20266724	-945,9463501	-0,068164
Cloud_Mesh_Distance_BA_R0_R1	20,1861763	-80,09997559	-945,7756348	-0,086538
Cloud_Mesh_Distance_BA_R0_R1	19,99902153	-80,0430603	-945,835144	-0,08505
Cloud_Mesh_Distance_BA_R0_R1	15,74745941	-79,88058472	-945,5808716	-0,138238
Cloud_Mesh_Distance_BA_R0_R1	15,8427	-79,78344727	-945,7097778	-0,133001
Cloud_Mesh_Distance_BA_R0_R1	19,93876457	-79,85360718	-945,8384399	-0,08775
Cloud_Mesh_Distance_BA_R0_R1	20,06892204	-79,72886658	-945,7769775	-0,093641
Cloud_Mesh_Distance_BA_R0_R1	15,95655155	-79,53620911	-945,8909912	-0,110423
Cloud_Mesh_Distance_BA_R0_R1	15,92572308	-79,69564819	-945,8122559	-0,119521
Cloud_Mesh_Distance_BA_R0_R1	19,76325989	-79,78527832	-945,895752	-0,082299
Cloud_Mesh_Distance_BA_R0_R1	19,74576759	-79,59741211	-945,8943481	-0,082627
Cloud_Mesh_Distance_BA_R0_R1	15,79734421	-79,57151794	-945,7515869	-0,124652
Cloud_Mesh_Distance_BA_R0_R1	15,78708839	-79,25505066	-945,8251953	-0,10402
Cloud_Mesh_Distance_BA_R0_R1	15,70922565	-79,37506104	-945,7271118	-0,11836
Cloud_Mesh_Distance_BA_R0_R1	15,627985	-79,52659607	-945,602356	-0,128163
Cloud_Mesh_Distance_BA_R0_R1	19,62902451	-79,51609802	-945,935669	-0,073925
Cloud_Mesh_Distance_BA_R0_R1	19,88430977	-79,49066162	-945,8330078	-0,09006
Cloud_Mesh_Distance_BA_R0_R1	19,90814018	-79,66929626	-945,8358154	-0,089725
Cloud_Mesh_Distance_BA_R0_R1	20,01461601	-79,5645752	-945,7841187	-0,093021
Cloud_Mesh_Distance_BA_R0_R1	15,64443111	-79,17088318	-945,6981812	-0,103829
Cloud_Mesh_Distance_BA_R0_R1	19,57455635	-79,36816406	-945,9549561	-0,070001
Cloud_Mesh_Distance_BA_R0_R1	19,6804657	-79,25469971	-945,9067383	-0,077502
Cloud_Mesh_Distance_BA_R0_R1	19,56804085	-79,16482544	-945,9525147	-0,067371
Cloud_Mesh_Distance_BA_R0_R1	19,6853447	-79,11790466	-945,8968506	-0,077484
Cloud_Mesh_Distance_BA_R0_R1	19,72851372	-78,93260193	-945,8618164	-0,076472
Cloud_Mesh_Distance_BA_R0_R1	15,58929539	-78,96511841	-945,649292	-0,09593
Cloud_Mesh_Distance_BA_R0_R1	19,44688034	-78,95462036	-945,996521	-0,056225
Cloud_Mesh_Distance_BA_R0_R1	19,32108498	-78,88395691	-946,0492554	-0,043929
Cloud_Mesh_Distance_BA_R0_R1	19,58544159	-78,84265137	-945,9234009	-0,067179
Cloud_Mesh_Distance_BA_R0_R1	19,57402229	-79,02790833	-945,942688	-0,06975
Cloud_Mesh_Distance_BA_R0_R1	15,71114731	-78,83329773	-945,7844238	-0,090649
Cloud_Mesh_Distance_BA_R0_R1	15,67596531	-78,6401062	-945,7290649	-0,083409
Cloud_Mesh_Distance_BA_R0_R1	19,4374485	-78,78755188	-945,9911499	-0,051952
Cloud_Mesh_Distance_BA_R0_R1	19,28910065	-78,34895325	-946,0198364	-0,029989
Cloud_Mesh_Distance_BA_R0_R1	19,70210457	-78,71075439	-945,8491211	-0,074871
Cloud_Mesh_Distance_BA_R0_R1	19,69115257	-78,50421143	-945,8237915	-0,072116
Cloud_Mesh_Distance_BA_R0_R1	19,52736664	-78,61508179	-945,9298096	-0,062211
Cloud_Mesh_Distance_BA_R0_R1	15,80622482	-78,46217346	-945,8406982	-0,079222
Cloud_Mesh_Distance_BA_R0_R1	19,17096901	-78,27856445	-946,0665894	-0,015772
Cloud_Mesh_Distance_BA_R0_R1	19,56584358	-78,40892029	-945,8808594	-0,056582
Cloud_Mesh_Distance_BA_R0_R1	19,42079353	-78,42315674	-945,9619751	-0,048352

Cloud_Mesh_Distance_BA_R0_R1	15,69842243	-78,12635803	-945,5899658	-0,071482
Cloud_Mesh_Distance_BA_R0_R1	15,9316721	-78,1085968	-945,8431397	-0,079652
Cloud_Mesh_Distance_BA_R0_R1	15,80974579	-77,99006653	-945,6550293	-0,077043
Cloud_Mesh_Distance_BA_R0_R1	19,17843437	-78,13769531	-946,0409546	-0,009907
Cloud_Mesh_Distance_BA_R0_R1	19,27957344	-78,20143127	-946,003479	-0,022266
Cloud_Mesh_Distance_BA_R0_R1	19,50048065	-78,24258423	-945,8899536	-0,04793
Cloud_Mesh_Distance_BA_R0_R1	19,37690926	-78,26971436	-945,9636231	-0,037595
Cloud_Mesh_Distance_BA_R0_R1	19,530159	-78,09483337	-945,8408203	-0,047592
Cloud_Mesh_Distance_BA_R0_R1	15,99839783	-77,69213867	-945,6531982	-0,092774
Cloud_Mesh_Distance_BA_R0_R1	15,93843174	-77,95257568	-945,7702026	-0,08893
Cloud_Mesh_Distance_BA_R0_R1	16,31055069	-77,88163757	-946,0164185	-0,106734
Cloud_Mesh_Distance_BA_R0_R1	16,06539154	-77,94963074	-945,8815308	-0,09575
Cloud_Mesh_Distance_BA_R0_R1	16,20528603	-77,83099365	-945,9249878	-0,104074
Cloud_Mesh_Distance_BA_R0_R1	19,3847847	-78,1342926	-945,9346924	-0,033524
Cloud_Mesh_Distance_BA_R0_R1	16,06902313	-77,7986145	-945,7984009	-0,098385
Cloud_Mesh_Distance_BA_R0_R1	16,15899086	-77,59918213	-945,7404785	-0,104129
Cloud_Mesh_Distance_BA_R0_R1	16,26578522	-77,67022705	-945,8737793	-0,101279
Cloud_Mesh_Distance_BA_R0_R1	16,48313713	-77,56723022	-945,9390869	-0,114305
Cloud_Mesh_Distance_BA_R0_R1	16,43207169	-77,42285156	-945,8109741	-0,107803
Cloud_Mesh_Distance_BA_R0_R1	16,87003899	-77,37388611	-945,9694824	-0,117661
Cloud_Mesh_Distance_BA_R0_R1	17,92887688	-77,46347046	-946,0917969	-0,066543
Cloud_Mesh_Distance_BA_R0_R1	17,76666451	-77,44223022	-946,0865479	-0,084731
Cloud_Mesh_Distance_BA_R0_R1	18,03404427	-77,35914612	-946,0300293	-0,050767
Cloud_Mesh_Distance_BA_R0_R1	17,83619499	-77,31817627	-946,0174561	-0,075144
Cloud_Mesh_Distance_BA_R0_R1	18,55444145	-77,39553833	-945,9803467	0,006705
Cloud_Mesh_Distance_BA_R0_R1	18,73640442	-77,45452881	-945,9667358	0,012721
Cloud_Mesh_Distance_BA_R0_R1	18,79961586	-77,57846069	-946,0119019	0,016765
Cloud_Mesh_Distance_BA_R0_R1	16,63190079	-77,40684509	-945,9047241	-0,114354
Cloud_Mesh_Distance_BA_R0_R1	17,10063744	-77,32568359	-945,9908447	-0,117906
Cloud_Mesh_Distance_BA_R0_R1	17,09727097	-77,13441467	-945,8616943	-0,110839
Cloud_Mesh_Distance_BA_R0_R1	17,26109695	-77,18289185	-945,9216309	-0,118858
Cloud_Mesh_Distance_BA_R0_R1	17,52495956	-77,34101868	-946,0327148	-0,110658
Cloud_Mesh_Distance_BA_R0_R1	17,99067497	-77,2180481	-945,9519043	-0,056251
Cloud_Mesh_Distance_BA_R0_R1	18,53875542	-77,27142334	-945,9115601	0,000567
Cloud_Mesh_Distance_BA_R0_R1	17,41258812	-77,08360291	-945,8670044	-0,105383
Cloud_Mesh_Distance_BA_R0_R1	17,65257454	-77,24331665	-945,9778442	-0,096896
Cloud_Mesh_Distance_BA_R0_R1	17,46612167	-77,20782471	-945,953125	-0,10729
Cloud_Mesh_Distance_BA_R0_R1	17,60928535	-77,09004211	-945,8812866	-0,091829
Cloud_Mesh_Distance_BA_R0_R1	17,94590187	-77,07946777	-945,8676148	-0,052225
Cloud_Mesh_Distance_BA_R0_R1	17,80205727	-77,15582275	-945,9224854	-0,079184
Cloud_Mesh_Distance_BA_R0_R1	18,2070179	-77,29171753	-945,9769287	-0,029528
Cloud_Mesh_Distance_BA_R0_R1	18,40749168	-77,28067017	-945,9430542	-0,008882
Cloud_Mesh_Distance_BA_R0_R1	18,30721283	-77,15606689	-945,8816528	-0,013766
Cloud_Mesh_Distance_BA_R0_R1	18,45679665	-77,18962097	-945,8771362	-0,001915
Cloud_Mesh_Distance_BA_R0_R1	17,50897026	-76,97058105	-945,7896118	-0,095715
Cloud_Mesh_Distance_BA_R0_R1	21,63587189	-82,040802	-945,6403198	0,171173
Cloud_Mesh_Distance_BA_R0_R1	21,23024368	-81,93344116	-945,8488159	0,195454
Cloud_Mesh_Distance_BA_R0_R1	21,40207291	-81,96582031	-945,7614746	0,132715
Cloud_Mesh_Distance_BA_R0_R1	21,09696198	-81,81332397	-945,861023	0,049957
Cloud_Mesh_Distance_BA_R0_R1	21,14972496	-81,64227295	-945,7521973	0,026184

Cloud_Mesh_Distance_BA_R0_R1	21,48033333	-81,82774353	-945,6320801	0,04252
Cloud_Mesh_Distance_BA_R0_R1	21,28451729	-81,79882813	-945,7462158	0,085197
Cloud_Mesh_Distance_BA_R0_R1	21,19225883	-81,49502563	-945,6651001	0,004135
Cloud_Mesh_Distance_BA_R0_R1	15,99255848	-81,22583008	-945,3603516	0,2696
Cloud_Mesh_Distance_BA_R0_R1	16,13794518	-81,27406311	-945,5443115	0,364472
Cloud_Mesh_Distance_BA_R0_R1	16,28391838	-81,16007996	-945,6195679	0,298509
Cloud_Mesh_Distance_BA_R0_R1	20,9419651	-81,28921509	-945,7287598	-0,013594
Cloud_Mesh_Distance_BA_R0_R1	21,02777481	-81,47792053	-945,751709	0,00462
Cloud_Mesh_Distance_BA_R0_R1	21,12627983	-81,33532715	-945,6437988	-0,011581
Cloud_Mesh_Distance_BA_R0_R1	16,14278984	-81,08964539	-945,4909668	0,218012
Cloud_Mesh_Distance_BA_R0_R1	20,87636566	-81,11517334	-945,7073364	-0,031026
Cloud_Mesh_Distance_BA_R0_R1	20,80475616	-81,45565796	-945,854187	-0,000732
Cloud_Mesh_Distance_BA_R0_R1	21,22111702	-81,22874451	-945,5490723	-0,025388
Cloud_Mesh_Distance_BA_R0_R1	16,15128326	-80,89025879	-945,4943848	0,10104
Cloud_Mesh_Distance_BA_R0_R1	16,02750969	-80,77313232	-945,4014282	0,013121
Cloud_Mesh_Distance_BA_R0_R1	16,29288673	-80,79019165	-945,6119385	0,076212
Cloud_Mesh_Distance_BA_R0_R1	16,14982414	-80,68991089	-945,5348511	0,009907
Cloud_Mesh_Distance_BA_R0_R1	16,28221893	-80,58529663	-945,6565552	-0,005243
Cloud_Mesh_Distance_BA_R0_R1	20,91378784	-80,93890381	-945,6376953	-0,044844
Cloud_Mesh_Distance_BA_R0_R1	15,86410999	-80,47860718	-945,3613281	-0,103673
Cloud_Mesh_Distance_BA_R0_R1	15,9277029	-80,65856934	-945,3384399	-0,051391
Cloud_Mesh_Distance_BA_R0_R1	20,55991173	-80,44059753	-945,6858521	-0,074167
Cloud_Mesh_Distance_BA_R0_R1	16,12472916	-80,48922729	-945,5892334	-0,054
Cloud_Mesh_Distance_BA_R0_R1	16,01386642	-80,58363342	-945,4581909	-0,050605
Cloud_Mesh_Distance_BA_R0_R1	15,98154831	-80,3976593	-945,5177612	-0,092961
Cloud_Mesh_Distance_BA_R0_R1	15,73666954	-80,29197693	-945,3329468	-0,138609
Cloud_Mesh_Distance_BA_R0_R1	15,7771349	-80,10902405	-945,4880371	-0,139272
Cloud_Mesh_Distance_BA_R0_R1	15,89454842	-79,99888611	-945,6529541	-0,130298
Cloud_Mesh_Distance_BA_R0_R1	15,86203575	-80,2999115	-945,461792	-0,12417
Cloud_Mesh_Distance_BA_R0_R1	16,07463455	-80,31430054	-945,632019	-0,09127
Cloud_Mesh_Distance_BA_R0_R1	20,35917282	-80,16731262	-945,7174072	-0,089013
Cloud_Mesh_Distance_BA_R0_R1	15,62203979	-79,74351501	-945,513916	-0,140793
Cloud_Mesh_Distance_BA_R0_R1	20,30101013	-79,9793396	-945,7127686	-0,093829
Cloud_Mesh_Distance_BA_R0_R1	20,12574196	-79,91622925	-945,7747803	-0,090884
Cloud_Mesh_Distance_BA_R0_R1	20,23838806	-79,78370667	-945,7124634	-0,096801
Cloud_Mesh_Distance_BA_R0_R1	15,72332191	-79,69828796	-945,6381836	-0,133316
Cloud_Mesh_Distance_BA_R0_R1	15,50714588	-79,60122681	-945,4352417	-0,134718
Cloud_Mesh_Distance_BA_R0_R1	15,58926296	-79,31134033	-945,6132202	-0,111055
Cloud_Mesh_Distance_BA_R0_R1	19,85279465	-79,31600952	-945,8348389	-0,088811
Cloud_Mesh_Distance_BA_R0_R1	20,03227806	-79,39320374	-945,7622681	-0,091646
Cloud_Mesh_Distance_BA_R0_R1	20,16314507	-79,58033752	-945,7224731	-0,096227
Cloud_Mesh_Distance_BA_R0_R1	20,22927666	-79,42323303	-945,6740723	-0,091331
Cloud_Mesh_Distance_BA_R0_R1	20,36282539	-79,63639832	-945,637085	-0,094852
Cloud_Mesh_Distance_BA_R0_R1	15,4932642	-79,15196228	-945,5176392	-0,103396
Cloud_Mesh_Distance_BA_R0_R1	15,49984169	-79,39216614	-945,4881592	-0,121031
Cloud_Mesh_Distance_BA_R0_R1	19,73389435	-79,42219543	-945,8914185	-0,081077
Cloud_Mesh_Distance_BA_R0_R1	19,80632782	-79,1217041	-945,8414917	-0,084014
Cloud_Mesh_Distance_BA_R0_R1	19,95215034	-79,16781616	-945,7772827	-0,086489
Cloud_Mesh_Distance_BA_R0_R1	20,32679558	-79,30053711	-945,6092529	-0,082449
Cloud_Mesh_Distance_BA_R0_R1	19,90342522	-78,96559143	-945,7774048	-0,083455

Cloud_Mesh_Distance_BA_R0_R1	20,07563591	-79,01426697	-945,6972046	-0,080258
Cloud_Mesh_Distance_BA_R0_R1	20,24729919	-79,10848999	-945,6226807	-0,077044
Cloud_Mesh_Distance_BA_R0_R1	20,14550781	-79,24275208	-945,6928101	-0,085677
Cloud_Mesh_Distance_BA_R0_R1	15,45349026	-78,91442871	-945,463562	-0,093515
Cloud_Mesh_Distance_BA_R0_R1	20,10127258	-78,85620117	-945,6565552	-0,080065
Cloud_Mesh_Distance_BA_R0_R1	20,19840431	-78,9455719	-945,6196899	-0,078851
Cloud_Mesh_Distance_BA_R0_R1	15,56517982	-78,77810669	-945,6088867	-0,092075
Cloud_Mesh_Distance_BA_R0_R1	15,43847275	-78,68328857	-945,4034424	-0,087821
Cloud_Mesh_Distance_BA_R0_R1	19,82707977	-78,81298828	-945,796814	-0,078518
Cloud_Mesh_Distance_BA_R0_R1	20,02554512	-78,69239807	-945,6665039	-0,080167
Cloud_Mesh_Distance_BA_R0_R1	19,9718914	-78,81134033	-945,7189941	-0,078682
Cloud_Mesh_Distance_BA_R0_R1	15,75325489	-78,24249268	-945,7174683	-0,076693
Cloud_Mesh_Distance_BA_R0_R1	19,84919548	-78,6265564	-945,7545776	-0,076375
Cloud_Mesh_Distance_BA_R0_R1	15,64054585	-78,4473877	-945,6467285	-0,078289
Cloud_Mesh_Distance_BA_R0_R1	19,81551361	-78,40568542	-945,7297974	-0,078212
Cloud_Mesh_Distance_BA_R0_R1	15,61191177	-78,10722351	-945,4480591	-0,071834
Cloud_Mesh_Distance_BA_R0_R1	19,68092918	-78,26513672	-945,7841797	-0,070917
Cloud_Mesh_Distance_BA_R0_R1	19,68347931	-78,03857422	-945,7248535	-0,061855
Cloud_Mesh_Distance_BA_R0_R1	19,79911232	-78,14575195	-945,6741943	-0,072287
Cloud_Mesh_Distance_BA_R0_R1	19,80368805	-78,27310181	-945,7056274	-0,075242
Cloud_Mesh_Distance_BA_R0_R1	19,92057991	-78,27633667	-945,6264038	-0,079762
Cloud_Mesh_Distance_BA_R0_R1	15,91415215	-77,84815979	-945,6793213	-0,08896
Cloud_Mesh_Distance_BA_R0_R1	15,84947586	-77,79127502	-945,5576782	-0,086387
Cloud_Mesh_Distance_BA_R0_R1	19,3900032	-77,97625732	-945,8959961	-0,029579
Cloud_Mesh_Distance_BA_R0_R1	19,21256638	-77,82637024	-945,9504395	-0,001294
Cloud_Mesh_Distance_BA_R0_R1	19,5347805	-77,73435974	-945,7225342	-0,043355
Cloud_Mesh_Distance_BA_R0_R1	19,53789711	-77,9316864	-945,7913208	-0,042366
Cloud_Mesh_Distance_BA_R0_R1	19,42031479	-77,60475159	-945,7392578	-0,028004
Cloud_Mesh_Distance_BA_R0_R1	19,37592888	-77,78421021	-945,8427124	-0,022498
Cloud_Mesh_Distance_BA_R0_R1	16,31035423	-77,50991821	-945,793457	-0,108335
Cloud_Mesh_Distance_BA_R0_R1	16,27946091	-77,39848328	-945,6809082	-0,107418
Cloud_Mesh_Distance_BA_R0_R1	19,03378868	-77,59347534	-945,9350586	0,011126
Cloud_Mesh_Distance_BA_R0_R1	19,11186028	-77,70301819	-945,9489746	0,005746
Cloud_Mesh_Distance_BA_R0_R1	19,21403313	-77,58813477	-945,8476563	-0,004793
Cloud_Mesh_Distance_BA_R0_R1	16,5021534	-77,30830383	-945,7634888	-0,107891
Cloud_Mesh_Distance_BA_R0_R1	16,66559792	-77,1608429	-945,7315064	-0,114911
Cloud_Mesh_Distance_BA_R0_R1	16,86392021	-77,14363098	-945,8021851	-0,114897
Cloud_Mesh_Distance_BA_R0_R1	16,74654198	-77,26712036	-945,8527832	-0,117308
Cloud_Mesh_Distance_BA_R0_R1	16,94838715	-77,23782349	-945,9000855	-0,121146
Cloud_Mesh_Distance_BA_R0_R1	18,6783371	-77,31564331	-945,9034424	0,007512
Cloud_Mesh_Distance_BA_R0_R1	18,81261635	-77,21786499	-945,7975464	0,012512
Cloud_Mesh_Distance_BA_R0_R1	18,82612991	-77,38249207	-945,8991699	0,012668
Cloud_Mesh_Distance_BA_R0_R1	19,00301933	-77,41741943	-945,8527832	0,008308
Cloud_Mesh_Distance_BA_R0_R1	18,8915081	-77,51438904	-945,9499512	0,011167
Cloud_Mesh_Distance_BA_R0_R1	19,04404449	-77,25590515	-945,7313843	0,000559
Cloud_Mesh_Distance_BA_R0_R1	19,17727089	-77,38650513	-945,7546387	-0,00335
Cloud_Mesh_Distance_BA_R0_R1	19,33306313	-77,45718384	-945,7132568	-0,019968
Cloud_Mesh_Distance_BA_R0_R1	16,80543327	-77,02416992	-945,6748047	-0,114111
Cloud_Mesh_Distance_BA_R0_R1	17,00557709	-77,04917908	-945,7702637	-0,114397
Cloud_Mesh_Distance_BA_R0_R1	17,93688393	-76,97657776	-945,7948608	-0,049407

Cloud_Mesh_Distance_BA_R0_R1	18,11917114	-77,12411499	-945,8837891	-0,034649
Cloud_Mesh_Distance_BA_R0_R1	18,2155323	-77,00796509	-945,7924805	-0,016967
Cloud_Mesh_Distance_BA_R0_R1	18,05231285	-77,01559448	-945,8157349	-0,035977
Cloud_Mesh_Distance_BA_R0_R1	18,43743706	-77,06643677	-945,7962036	0,001135
Cloud_Mesh_Distance_BA_R0_R1	18,59267998	-77,1862793	-945,8434448	0,007177
Cloud_Mesh_Distance_BA_R0_R1	18,63146782	-77,0730896	-945,7525024	0,007832
Cloud_Mesh_Distance_BA_R0_R1	19,24229622	-77,26730347	-945,6393433	-0,016229
Cloud_Mesh_Distance_BA_R0_R1	16,60048485	-77,07055664	-945,6126099	-0,111188
Cloud_Mesh_Distance_BA_R0_R1	16,8290596	-76,89712524	-945,5563355	-0,1074
Cloud_Mesh_Distance_BA_R0_R1	16,66301155	-76,96470642	-945,5414429	-0,102444
Cloud_Mesh_Distance_BA_R0_R1	17,01581955	-76,91741943	-945,6531372	-0,110578
Cloud_Mesh_Distance_BA_R0_R1	17,19207764	-76,98245239	-945,7592773	-0,109252
Cloud_Mesh_Distance_BA_R0_R1	17,31121445	-77,03237915	-945,8197022	-0,107987
Cloud_Mesh_Distance_BA_R0_R1	17,36019135	-76,89355469	-945,7092285	-0,102326
Cloud_Mesh_Distance_BA_R0_R1	17,65699577	-76,94099426	-945,7722778	-0,079453
Cloud_Mesh_Distance_BA_R0_R1	17,86042595	-76,88975525	-945,7295532	-0,058036
Cloud_Mesh_Distance_BA_R0_R1	17,78117561	-77,00744629	-945,8229981	-0,070675
Cloud_Mesh_Distance_BA_R0_R1	18,05139351	-76,90660095	-945,7323608	-0,036975
Cloud_Mesh_Distance_BA_R0_R1	17,95884132	-76,78921509	-945,6366577	-0,041095
Cloud_Mesh_Distance_BA_R0_R1	21,83652306	-82,07373047	-945,4970093	0,078714
Cloud_Mesh_Distance_BA_R0_R1	21,6461792	-81,84747314	-945,5212402	0,038497
Cloud_Mesh_Distance_BA_R0_R1	21,77341461	-81,73339844	-945,3601074	0,020018
Cloud_Mesh_Distance_BA_R0_R1	21,34795761	-81,62393188	-945,6226196	0,020367
Cloud_Mesh_Distance_BA_R0_R1	21,55699921	-81,68504333	-945,5059815	0,023195
Cloud_Mesh_Distance_BA_R0_R1	15,85745239	-81,29052734	-945,1616211	0,248694
Cloud_Mesh_Distance_BA_R0_R1	21,31402397	-81,39726257	-945,5499878	-0,006716
Cloud_Mesh_Distance_BA_R0_R1	21,62210274	-81,537323	-945,3890991	0,000078
Cloud_Mesh_Distance_BA_R0_R1	21,46733475	-81,51989746	-945,4958496	0,00287
Cloud_Mesh_Distance_BA_R0_R1	21,4989872	-81,34492493	-945,4031372	-0,020351
Cloud_Mesh_Distance_BA_R0_R1	16,00222588	-80,98724365	-945,3339233	0,10502
Cloud_Mesh_Distance_BA_R0_R1	21,06181526	-81,12307739	-945,6092529	-0,032337
Cloud_Mesh_Distance_BA_R0_R1	21,09469414	-80,89608765	-945,524292	-0,052135
Cloud_Mesh_Distance_BA_R0_R1	21,25716972	-81,05589294	-945,4713135	-0,042709
Cloud_Mesh_Distance_BA_R0_R1	15,79224586	-80,80456543	-945,0853882	-0,057844
Cloud_Mesh_Distance_BA_R0_R1	15,90799713	-80,80841064	-945,2523193	-0,010247
Cloud_Mesh_Distance_BA_R0_R1	15,81709099	-80,64187622	-945,2111816	-0,087504
Cloud_Mesh_Distance_BA_R0_R1	20,77842331	-80,76797485	-945,6605225	-0,056848
Cloud_Mesh_Distance_BA_R0_R1	20,95812416	-80,73179626	-945,5588379	-0,061955
Cloud_Mesh_Distance_BA_R0_R1	15,70984554	-80,46214294	-945,1845703	-0,135631
Cloud_Mesh_Distance_BA_R0_R1	20,83569145	-80,56350708	-945,5825806	-0,070071
Cloud_Mesh_Distance_BA_R0_R1	20,66064835	-80,59088135	-945,6738281	-0,067143
Cloud_Mesh_Distance_BA_R0_R1	20,91217422	-80,34599304	-945,4925537	-0,08302
Cloud_Mesh_Distance_BA_R0_R1	21,09415245	-80,43582153	-945,4091797	-0,081125
Cloud_Mesh_Distance_BA_R0_R1	20,98666382	-80,54904175	-945,4985352	-0,073082
Cloud_Mesh_Distance_BA_R0_R1	15,58568859	-80,26795959	-945,1566162	-0,157468
Cloud_Mesh_Distance_BA_R0_R1	15,62433243	-80,11222839	-945,3162842	-0,157617
Cloud_Mesh_Distance_BA_R0_R1	20,76078224	-80,17192078	-945,5343628	-0,088592
Cloud_Mesh_Distance_BA_R0_R1	20,71532059	-80,39108276	-945,6036377	-0,07869
Cloud_Mesh_Distance_BA_R0_R1	20,55734634	-80,23068237	-945,6447144	-0,08458
Cloud_Mesh_Distance_BA_R0_R1	15,63500118	-79,93927002	-945,4326172	-0,150264

Cloud_Mesh_Distance_BA_R0_R1	15,53559875	-79,96343994	-945,2973023	-0,15456
Cloud_Mesh_Distance_BA_R0_R1	20,63780022	-80,01005554	-945,5638428	-0,093029
Cloud_Mesh_Distance_BA_R0_R1	20,46850204	-80,02229309	-945,6468506	-0,091493
Cloud_Mesh_Distance_BA_R0_R1	20,52225685	-79,82852173	-945,5883789	-0,095734
Cloud_Mesh_Distance_BA_R0_R1	21,00801849	-79,97238159	-945,3514404	-0,09233
Cloud_Mesh_Distance_BA_R0_R1	21,08457184	-80,1995697	-945,3583374	-0,090927
Cloud_Mesh_Distance_BA_R0_R1	20,91510963	-80,12313843	-945,440979	-0,090865
Cloud_Mesh_Distance_BA_R0_R1	15,39252281	-79,64523315	-945,258667	-0,134819
Cloud_Mesh_Distance_BA_R0_R1	20,39217567	-79,84989929	-945,6531982	-0,094434
Cloud_Mesh_Distance_BA_R0_R1	15,51508141	-79,79911804	-945,3607788	-0,146149
Cloud_Mesh_Distance_BA_R0_R1	20,75568199	-79,6938324	-945,4390259	-0,090977
Cloud_Mesh_Distance_BA_R0_R1	20,56188583	-79,6083374	-945,5321045	-0,087819
Cloud_Mesh_Distance_BA_R0_R1	20,65885735	-79,81712341	-945,5169678	-0,095493
Cloud_Mesh_Distance_BA_R0_R1	15,42272568	-79,47351074	-945,3630371	-0,123355
Cloud_Mesh_Distance_BA_R0_R1	15,33891582	-79,42475891	-945,2477417	-0,120578
Cloud_Mesh_Distance_BA_R0_R1	15,39899921	-79,29304504	-945,3672485	-0,114705
Cloud_Mesh_Distance_BA_R0_R1	20,40496254	-79,47015381	-945,5941162	-0,08624
Cloud_Mesh_Distance_BA_R0_R1	20,69605827	-79,47360229	-945,4301148	-0,082045
Cloud_Mesh_Distance_BA_R0_R1	20,51522064	-79,34765625	-945,5142212	-0,081568
Cloud_Mesh_Distance_BA_R0_R1	15,3556881	-79,0737915	-945,3152466	-0,094867
Cloud_Mesh_Distance_BA_R0_R1	15,34874916	-78,83676147	-945,2804565	-0,091822
Cloud_Mesh_Distance_BA_R0_R1	20,38980293	-79,18490601	-945,5567627	-0,077397
Cloud_Mesh_Distance_BA_R0_R1	20,52482986	-79,10258484	-945,4610596	-0,068153
Cloud_Mesh_Distance_BA_R0_R1	15,34332275	-78,66731262	-945,2271729	-0,081507
Cloud_Mesh_Distance_BA_R0_R1	20,36643982	-78,96252441	-945,5271606	-0,068043
Cloud_Mesh_Distance_BA_R0_R1	20,19760132	-78,77484131	-945,5835571	-0,071635
Cloud_Mesh_Distance_BA_R0_R1	20,33406639	-78,74169922	-945,4902954	-0,06577
Cloud_Mesh_Distance_BA_R0_R1	15,55279541	-78,60342407	-945,5638428	-0,08194
Cloud_Mesh_Distance_BA_R0_R1	20,45096207	-78,81048584	-945,4336548	-0,05845
Cloud_Mesh_Distance_BA_R0_R1	20,5262413	-78,71090698	-945,3462524	-0,05173
Cloud_Mesh_Distance_BA_R0_R1	15,51424313	-78,48104858	-945,4716187	-0,078844
Cloud_Mesh_Distance_BA_R0_R1	15,38956356	-78,47979736	-945,2463989	-0,078735
Cloud_Mesh_Distance_BA_R0_R1	20,08800316	-78,57835388	-945,5999756	-0,076578
Cloud_Mesh_Distance_BA_R0_R1	20,23867035	-78,59042358	-945,5064697	-0,068585
Cloud_Mesh_Distance_BA_R0_R1	20,3131237	-78,42185974	-945,3927002	-0,066753
Cloud_Mesh_Distance_BA_R0_R1	15,51173973	-78,17607117	-945,3184204	-0,069935
Cloud_Mesh_Distance_BA_R0_R1	15,59863663	-78,27624512	-945,5222168	-0,070808
Cloud_Mesh_Distance_BA_R0_R1	19,94724846	-78,49243164	-945,6663208	-0,084329
Cloud_Mesh_Distance_BA_R0_R1	20,15343094	-78,40458679	-945,5023804	-0,078369
Cloud_Mesh_Distance_BA_R0_R1	20,05242729	-78,28839111	-945,534668	-0,082583
Cloud_Mesh_Distance_BA_R0_R1	20,02978325	-78,40898132	-945,5892944	-0,080443
Cloud_Mesh_Distance_BA_R0_R1	20,18085289	-78,24847412	-945,4226685	-0,078904
Cloud_Mesh_Distance_BA_R0_R1	20,02034378	-78,13580322	-945,5042725	-0,088176
Cloud_Mesh_Distance_BA_R0_R1	15,70563984	-77,98057556	-945,5064697	-0,075776
Cloud_Mesh_Distance_BA_R0_R1	15,75353718	-77,7991333	-945,4312744	-0,078756
Cloud_Mesh_Distance_BA_R0_R1	19,69335938	-77,82044983	-945,6433106	-0,064906
Cloud_Mesh_Distance_BA_R0_R1	15,89060307	-77,65750122	-945,4938355	-0,089284
Cloud_Mesh_Distance_BA_R0_R1	19,58286476	-77,63526917	-945,6459351	-0,041901
Cloud_Mesh_Distance_BA_R0_R1	19,82267761	-77,76486206	-945,5170898	-0,072547
Cloud_Mesh_Distance_BA_R0_R1	19,83784294	-77,90815735	-945,5657959	-0,077484

Cloud_Mesh_Distance_BA_R0_R1	16,16093445	-77,37796021	-945,5487671	-0,100547
Cloud_Mesh_Distance_BA_R0_R1	16,02044487	-77,39710999	-945,4024048	-0,090217
Cloud_Mesh_Distance_BA_R0_R1	16,03804588	-77,52516174	-945,5531006	-0,0883
Cloud_Mesh_Distance_BA_R0_R1	16,31905365	-77,27033997	-945,5977783	-0,103187
Cloud_Mesh_Distance_BA_R0_R1	16,15839195	-77,24879456	-945,4122925	-0,096318
Cloud_Mesh_Distance_BA_R0_R1	19,3657856	-77,26522827	-945,5623169	-0,027382
Cloud_Mesh_Distance_BA_R0_R1	19,3933754	-77,371521	-945,6231079	-0,02498
Cloud_Mesh_Distance_BA_R0_R1	19,54912376	-77,4924469	-945,5951538	-0,039049
Cloud_Mesh_Distance_BA_R0_R1	19,67107391	-77,47926331	-945,4926758	-0,055205
Cloud_Mesh_Distance_BA_R0_R1	16,49243355	-77,05560303	-945,5253296	-0,102788
Cloud_Mesh_Distance_BA_R0_R1	16,44041634	-77,17353821	-945,6047974	-0,106272
Cloud_Mesh_Distance_BA_R0_R1	18,94598961	-77,08444214	-945,6434326	0,00471
Cloud_Mesh_Distance_BA_R0_R1	19,13691139	-77,16423035	-945,6147461	-0,005219
Cloud_Mesh_Distance_BA_R0_R1	19,29904175	-77,11590576	-945,4784546	-0,023507
Cloud_Mesh_Distance_BA_R0_R1	19,47553635	-77,21002197	-945,4439697	-0,037727
Cloud_Mesh_Distance_BA_R0_R1	16,8165493	-76,79187012	-945,423584	-0,107604
Cloud_Mesh_Distance_BA_R0_R1	17,45210838	-76,77305603	-945,604187	-0,089441
Cloud_Mesh_Distance_BA_R0_R1	17,61234093	-76,79711914	-945,6442261	-0,074973
Cloud_Mesh_Distance_BA_R0_R1	17,52184105	-76,86367798	-945,7001343	-0,086049
Cloud_Mesh_Distance_BA_R0_R1	18,34468079	-76,92829895	-945,7096558	-0,004804
Cloud_Mesh_Distance_BA_R0_R1	18,61757851	-76,8586731	-945,5754395	0,017315
Cloud_Mesh_Distance_BA_R0_R1	18,51796722	-76,95266724	-945,6898193	0,008381
Cloud_Mesh_Distance_BA_R0_R1	18,66407394	-76,97502136	-945,6637573	0,013591
Cloud_Mesh_Distance_BA_R0_R1	18,79340172	-76,94360352	-945,5884399	0,012404
Cloud_Mesh_Distance_BA_R0_R1	18,76847458	-77,0567627	-945,694458	0,010613
Cloud_Mesh_Distance_BA_R0_R1	16,95341682	-76,8286438	-945,5350342	-0,105411
Cloud_Mesh_Distance_BA_R0_R1	17,12152672	-76,79454041	-945,5561523	-0,105793
Cloud_Mesh_Distance_BA_R0_R1	16,95952034	-76,73100281	-945,4140015	-0,107388
Cloud_Mesh_Distance_BA_R0_R1	17,15332222	-76,69998169	-945,4522705	-0,101703
Cloud_Mesh_Distance_BA_R0_R1	17,24132347	-76,8433075	-945,6408081	-0,103527
Cloud_Mesh_Distance_BA_R0_R1	17,29178238	-76,72555542	-945,524231	-0,096439
Cloud_Mesh_Distance_BA_R0_R1	17,54371262	-76,59962463	-945,4116821	-0,073966
Cloud_Mesh_Distance_BA_R0_R1	17,44236755	-76,66471863	-945,4805298	-0,083104
Cloud_Mesh_Distance_BA_R0_R1	17,76874161	-76,78475952	-945,6373291	-0,060277
Cloud_Mesh_Distance_BA_R0_R1	17,64466667	-76,68299866	-945,5264282	-0,066484
Cloud_Mesh_Distance_BA_R0_R1	17,86543083	-76,67980957	-945,5283814	-0,042855
Cloud_Mesh_Distance_BA_R0_R1	18,24038887	-76,86349487	-945,6740723	-0,00779
Cloud_Mesh_Distance_BA_R0_R1	18,13892555	-76,80426025	-945,6343384	-0,016431
Cloud_Mesh_Distance_BA_R0_R1	18,42097282	-76,83647156	-945,6118774	0,009042
Cloud_Mesh_Distance_BA_R0_R1	21,98178482	-82,18037415	-945,4316406	0,152099
Cloud_Mesh_Distance_BA_R0_R1	21,94595718	-81,96481323	-945,3334351	0,035394
Cloud_Mesh_Distance_BA_R0_R1	22,12113953	-82,13235474	-945,2600098	0,072382
Cloud_Mesh_Distance_BA_R0_R1	21,77080917	-81,93414307	-945,4697876	0,043384
Cloud_Mesh_Distance_BA_R0_R1	21,67757797	-81,3881073	-945,2832031	-0,0208
Cloud_Mesh_Distance_BA_R0_R1	15,754776	-81,43083191	-944,9671631	0,285135
Cloud_Mesh_Distance_BA_R0_R1	21,34722328	-81,23141479	-945,4678955	-0,028856
Cloud_Mesh_Distance_BA_R0_R1	15,86610508	-80,97621155	-945,1400147	0,039733
Cloud_Mesh_Distance_BA_R0_R1	15,90718842	-81,12742615	-945,2005005	0,150877
Cloud_Mesh_Distance_BA_R0_R1	21,44427872	-81,12878418	-945,3668823	-0,040456
Cloud_Mesh_Distance_BA_R0_R1	21,23979759	-80,85513306	-945,4245606	-0,059792

Cloud_Mesh_Distance_BA_R0_R1	21,48748589	-80,8072052	-945,2398071	-0,073366
Cloud_Mesh_Distance_BA_R0_R1	21,38568115	-80,92945862	-945,3467407	-0,059123
Cloud_Mesh_Distance_BA_R0_R1	21,13580704	-80,67216492	-945,4412842	-0,069489
Cloud_Mesh_Distance_BA_R0_R1	21,32227325	-80,72851563	-945,3356934	-0,0707
Cloud_Mesh_Distance_BA_R0_R1	21,25376892	-80,5244751	-945,329773	-0,081228
Cloud_Mesh_Distance_BA_R0_R1	21,34989929	-80,39608765	-945,2321167	-0,090512
Cloud_Mesh_Distance_BA_R0_R1	15,40885544	-80,19741821	-944,9285278	-0,165804
Cloud_Mesh_Distance_BA_R0_R1	15,37336636	-79,97052002	-945,0473023	-0,156106
Cloud_Mesh_Distance_BA_R0_R1	15,48798656	-80,06991577	-945,1602173	-0,163355
Cloud_Mesh_Distance_BA_R0_R1	21,20633316	-80,34672546	-945,317688	-0,088388
Cloud_Mesh_Distance_BA_R0_R1	15,43038845	-79,87446594	-945,2009888	-0,151569
Cloud_Mesh_Distance_BA_R0_R1	21,27394295	-80,20883179	-945,2382202	-0,092961
Cloud_Mesh_Distance_BA_R0_R1	21,42487144	-80,27058411	-945,1452026	-0,096078
Cloud_Mesh_Distance_BA_R0_R1	21,40411758	-80,08493042	-945,1105957	-0,095041
Cloud_Mesh_Distance_BA_R0_R1	15,31578159	-79,77217102	-945,071167	-0,148129
Cloud_Mesh_Distance_BA_R0_R1	21,09553146	-79,83375549	-945,260376	-0,089302
Cloud_Mesh_Distance_BA_R0_R1	21,08360863	-79,68318176	-945,2270508	-0,086929
Cloud_Mesh_Distance_BA_R0_R1	21,22992706	-79,74958801	-945,1427002	-0,090729
Cloud_Mesh_Distance_BA_R0_R1	15,27701283	-79,54743958	-945,1036377	-0,123675
Cloud_Mesh_Distance_BA_R0_R1	15,21323872	-79,6590271	-944,927063	-0,129233
Cloud_Mesh_Distance_BA_R0_R1	20,94041252	-79,75216675	-945,3403931	-0,090212
Cloud_Mesh_Distance_BA_R0_R1	20,8165493	-79,93330383	-945,4541016	-0,093318
Cloud_Mesh_Distance_BA_R0_R1	20,97608948	-79,5500946	-945,2650757	-0,08446
Cloud_Mesh_Distance_BA_R0_R1	15,31828403	-79,25201416	-945,2435913	-0,104332
Cloud_Mesh_Distance_BA_R0_R1	15,25579643	-79,335495	-945,1195679	-0,108774
Cloud_Mesh_Distance_BA_R0_R1	20,8405056	-79,38095093	-945,3148804	-0,076794
Cloud_Mesh_Distance_BA_R0_R1	20,83768272	-79,57684326	-945,3637695	-0,084758
Cloud_Mesh_Distance_BA_R0_R1	15,26145458	-79,14123535	-945,1467285	-0,094644
Cloud_Mesh_Distance_BA_R0_R1	21,00800133	-79,39407349	-945,1984253	-0,077531
Cloud_Mesh_Distance_BA_R0_R1	15,27213192	-78,94944763	-945,1538696	-0,090064
Cloud_Mesh_Distance_BA_R0_R1	20,54955482	-78,90188599	-945,3936157	-0,056105
Cloud_Mesh_Distance_BA_R0_R1	20,67949867	-79,23103333	-945,3885498	-0,069533
Cloud_Mesh_Distance_BA_R0_R1	20,66333199	-79,00372314	-945,3401489	-0,059438
Cloud_Mesh_Distance_BA_R0_R1	15,27570915	-78,75782776	-945,1177979	-0,083847
Cloud_Mesh_Distance_BA_R0_R1	15,29759693	-78,59335327	-945,1046143	-0,081049
Cloud_Mesh_Distance_BA_R0_R1	20,39624405	-78,61956787	-945,40802	-0,063026
Cloud_Mesh_Distance_BA_R0_R1	15,46548176	-78,34011841	-945,3275147	-0,071172
Cloud_Mesh_Distance_BA_R0_R1	20,47204018	-78,50564575	-945,305481	-0,054708
Cloud_Mesh_Distance_BA_R0_R1	20,42298126	-78,26335144	-945,2306519	-0,061502
Cloud_Mesh_Distance_BA_R0_R1	19,90251732	-78,06399536	-945,5701904	-0,083404
Cloud_Mesh_Distance_BA_R0_R1	15,64998722	-77,79750061	-945,2571411	-0,077725
Cloud_Mesh_Distance_BA_R0_R1	15,61445236	-77,95426941	-945,3389282	-0,073388
Cloud_Mesh_Distance_BA_R0_R1	19,9912529	-77,95159912	-945,4545288	-0,082629
Cloud_Mesh_Distance_BA_R0_R1	15,80430222	-77,54481506	-945,2477417	-0,078155
Cloud_Mesh_Distance_BA_R0_R1	15,78325748	-77,66488647	-945,345398	-0,080783
Cloud_Mesh_Distance_BA_R0_R1	19,72912025	-77,6262207	-945,5285034	-0,060154
Cloud_Mesh_Distance_BA_R0_R1	19,94991112	-77,77380371	-945,4104614	-0,079352
Cloud_Mesh_Distance_BA_R0_R1	20,08486557	-77,82868958	-945,3135376	-0,084893
Cloud_Mesh_Distance_BA_R0_R1	15,89373398	-77,42370605	-945,2497559	-0,089248
Cloud_Mesh_Distance_BA_R0_R1	15,90149689	-77,53410339	-945,3852539	-0,089253

Cloud_Mesh_Distance_BA_R0_R1	19,9119339	-77,63815308	-945,3751831	-0,074278
Cloud_Mesh_Distance_BA_R0_R1	16,00341606	-77,28436279	-945,2479248	-0,08955
Cloud_Mesh_Distance_BA_R0_R1	16,33750725	-77,10906982	-945,4510498	-0,093319
Cloud_Mesh_Distance_BA_R0_R1	16,22390556	-77,10487366	-945,3221436	-0,095148
Cloud_Mesh_Distance_BA_R0_R1	16,35173988	-76,97070313	-945,2911377	-0,093569
Cloud_Mesh_Distance_BA_R0_R1	19,10707474	-77,03109741	-945,5142822	-0,011428
Cloud_Mesh_Distance_BA_R0_R1	19,32745552	-76,95527649	-945,3049316	-0,025557
Cloud_Mesh_Distance_BA_R0_R1	19,63527298	-77,1947937	-945,3078613	-0,051078
Cloud_Mesh_Distance_BA_R0_R1	19,70114708	-77,35345459	-945,3822632	-0,052629
Cloud_Mesh_Distance_BA_R0_R1	19,53440094	-77,34100342	-945,5057373	-0,03978
Cloud_Mesh_Distance_BA_R0_R1	16,59824562	-76,80209351	-945,2841187	-0,100793
Cloud_Mesh_Distance_BA_R0_R1	16,69492531	-76,85339355	-945,4294434	-0,101317
Cloud_Mesh_Distance_BA_R0_R1	16,47960281	-76,97111511	-945,418457	-0,09937
Cloud_Mesh_Distance_BA_R0_R1	18,95929718	-76,93855286	-945,5059815	0,004895
Cloud_Mesh_Distance_BA_R0_R1	18,97477531	-76,80406189	-945,3588867	0,003316
Cloud_Mesh_Distance_BA_R0_R1	19,21728134	-76,97035217	-945,3912964	-0,016996
Cloud_Mesh_Distance_BA_R0_R1	19,08460999	-76,91749573	-945,4169312	-0,005296
Cloud_Mesh_Distance_BA_R0_R1	19,13441658	-76,81477356	-945,2765503	-0,010675
Cloud_Mesh_Distance_BA_R0_R1	16,74918938	-76,69673157	-945,2357178	-0,102428
Cloud_Mesh_Distance_BA_R0_R1	17,33945465	-76,60585022	-945,3806763	-0,089092
Cloud_Mesh_Distance_BA_R0_R1	17,79195023	-76,59712219	-945,4298706	-0,046713
Cloud_Mesh_Distance_BA_R0_R1	17,99577141	-76,60351563	-945,4302368	-0,029335
Cloud_Mesh_Distance_BA_R0_R1	18,2831974	-76,73051453	-945,5372315	-0,000123
Cloud_Mesh_Distance_BA_R0_R1	18,07139397	-76,69836426	-945,5341187	-0,024711
Cloud_Mesh_Distance_BA_R0_R1	18,49706459	-76,73783875	-945,4915161	0,012486
Cloud_Mesh_Distance_BA_R0_R1	18,80997276	-76,82244873	-945,4636841	0,01335
Cloud_Mesh_Distance_BA_R0_R1	18,68335915	-76,75045776	-945,4403076	0,012203
Cloud_Mesh_Distance_BA_R0_R1	17,73506546	-76,47564697	-945,2435913	-0,058858
Cloud_Mesh_Distance_BA_R0_R1	18,09548378	-76,53599548	-945,3274536	-0,022348
Cloud_Mesh_Distance_BA_R0_R1	17,91169167	-76,51957703	-945,3178101	-0,039047
Cloud_Mesh_Distance_BA_R0_R1	18,18926239	-76,61157227	-945,4154053	-0,011744
Cloud_Mesh_Distance_BA_R0_R1	18,39636421	-76,62600708	-945,3883667	0,002886
Cloud_Mesh_Distance_BA_R0_R1	18,58584976	-76,65290833	-945,3624878	0,014415
Cloud_Mesh_Distance_BA_R0_R1	18,53075409	-76,55247498	-945,2462158	0,013382
Cloud_Mesh_Distance_BA_R0_R1	18,76265526	-76,65823364	-945,2976685	0,018398
Cloud_Mesh_Distance_BA_R0_R1	22,13258553	-81,9339447	-945,1282959	0,01701
Cloud_Mesh_Distance_BA_R0_R1	22,25131035	-82,3031311	-945,2250977	0,139895
Cloud_Mesh_Distance_BA_R0_R1	22,28018951	-82,12028503	-945,0727539	0,017252
Cloud_Mesh_Distance_BA_R0_R1	15,66501999	-81,8014679	-944,470398	0,389111
Cloud_Mesh_Distance_BA_R0_R1	22,15055275	-81,71322632	-944,9897461	-0,002564
Cloud_Mesh_Distance_BA_R0_R1	15,70755768	-81,51171875	-944,7983398	0,293018
Cloud_Mesh_Distance_BA_R0_R1	15,68539333	-81,62356567	-944,5889893	0,292602
Cloud_Mesh_Distance_BA_R0_R1	21,98594856	-81,75714111	-945,1813355	0,013236
Cloud_Mesh_Distance_BA_R0_R1	21,88379669	-81,5861969	-945,1923828	-0,004889
Cloud_Mesh_Distance_BA_R0_R1	22,01282501	-81,60345459	-945,0784302	-0,010954
Cloud_Mesh_Distance_BA_R0_R1	21,75588799	-81,53567505	-945,2811279	-0,005177
Cloud_Mesh_Distance_BA_R0_R1	21,81596184	-81,42236328	-945,1813355	-0,023586
Cloud_Mesh_Distance_BA_R0_R1	21,94835281	-81,45381165	-945,0737305	-0,023836
Cloud_Mesh_Distance_BA_R0_R1	15,80982304	-81,16122437	-945,0007935	0,102444
Cloud_Mesh_Distance_BA_R0_R1	21,74899292	-81,27603149	-945,1814575	-0,037105

Cloud_Mesh_Distance_BA_R0_R1	21,87039757	-81,28491211	-945,0778809	-0,041889
Cloud_Mesh_Distance_BA_R0_R1	22,00960732	-81,31758118	-944,9592285	-0,045108
Cloud_Mesh_Distance_BA_R0_R1	15,76435947	-80,96575928	-944,9505005	-0,017454
Cloud_Mesh_Distance_BA_R0_R1	21,5557003	-81,00489807	-945,2451172	-0,059462
Cloud_Mesh_Distance_BA_R0_R1	21,61540604	-81,20368958	-945,2633057	-0,040942
Cloud_Mesh_Distance_BA_R0_R1	21,63191986	-80,88348389	-945,1488648	-0,073488
Cloud_Mesh_Distance_BA_R0_R1	21,92848969	-80,95054626	-944,9095459	-0,081923
Cloud_Mesh_Distance_BA_R0_R1	21,75780296	-81,07420349	-945,1030273	-0,063274
Cloud_Mesh_Distance_BA_R0_R1	15,61831284	-80,80307007	-944,7554932	-0,113466
Cloud_Mesh_Distance_BA_R0_R1	15,68976688	-80,79838562	-944,9065552	-0,097435
Cloud_Mesh_Distance_BA_R0_R1	15,69282627	-80,63265991	-945,036438	-0,122533
Cloud_Mesh_Distance_BA_R0_R1	21,59568596	-80,69952393	-945,1262817	-0,086686
Cloud_Mesh_Distance_BA_R0_R1	15,57655716	-80,63598633	-944,8289795	-0,145
Cloud_Mesh_Distance_BA_R0_R1	15,5691061	-80,44293213	-944,9902344	-0,15686
Cloud_Mesh_Distance_BA_R0_R1	21,42516899	-80,59968567	-945,2301025	-0,084962
Cloud_Mesh_Distance_BA_R0_R1	21,54398537	-80,45959473	-945,1032715	-0,096352
Cloud_Mesh_Distance_BA_R0_R1	15,25854206	-79,91386414	-944,8649902	-0,149911
Cloud_Mesh_Distance_BA_R0_R1	15,16193771	-79,7396698	-944,7561035	-0,135068
Cloud_Mesh_Distance_BA_R0_R1	21,38154411	-79,8711853	-945,0645142	-0,095476
Cloud_Mesh_Distance_BA_R0_R1	21,20975876	-79,98800659	-945,2243042	-0,09315
Cloud_Mesh_Distance_BA_R0_R1	15,12557125	-79,52819824	-944,7667847	-0,114952
Cloud_Mesh_Distance_BA_R0_R1	21,32050514	-79,59120178	-945,0216065	-0,088863
Cloud_Mesh_Distance_BA_R0_R1	21,36144829	-79,73184204	-945,0366211	-0,094901
Cloud_Mesh_Distance_BA_R0_R1	15,19079399	-79,43740845	-944,9624634	-0,112912
Cloud_Mesh_Distance_BA_R0_R1	15,1815443	-79,20892334	-944,9762573	-0,096966
Cloud_Mesh_Distance_BA_R0_R1	21,14185905	-79,36965942	-945,0877686	-0,075922
Cloud_Mesh_Distance_BA_R0_R1	21,15680504	-79,53398132	-945,1304321	-0,08559
Cloud_Mesh_Distance_BA_R0_R1	15,13213634	-79,33572388	-944,8400269	-0,098458
Cloud_Mesh_Distance_BA_R0_R1	15,15452766	-79,00984192	-944,8933106	-0,084434
Cloud_Mesh_Distance_BA_R0_R1	15,212286	-79,03443909	-945,0387573	-0,093731
Cloud_Mesh_Distance_BA_R0_R1	20,9223671	-79,21743774	-945,2089233	-0,068281
Cloud_Mesh_Distance_BA_R0_R1	20,79799271	-79,16221619	-945,2859497	-0,067396
Cloud_Mesh_Distance_BA_R0_R1	21,06625175	-79,10305786	-945,0530396	-0,058481
Cloud_Mesh_Distance_BA_R0_R1	21,06166458	-79,25552368	-945,1119995	-0,070338
Cloud_Mesh_Distance_BA_R0_R1	15,22123241	-78,65260315	-944,9476318	-0,081003
Cloud_Mesh_Distance_BA_R0_R1	15,20966244	-78,86753845	-945,0003662	-0,087235
Cloud_Mesh_Distance_BA_R0_R1	20,78429604	-78,92201233	-945,2192993	-0,048998
Cloud_Mesh_Distance_BA_R0_R1	20,68138695	-78,82836914	-945,2683106	-0,047429
Cloud_Mesh_Distance_BA_R0_R1	20,76473045	-78,70811462	-945,1501465	-0,043085
Cloud_Mesh_Distance_BA_R0_R1	20,6546917	-78,63302612	-945,2112427	-0,042777
Cloud_Mesh_Distance_BA_R0_R1	20,89345551	-78,81130981	-945,0827026	-0,042343
Cloud_Mesh_Distance_BA_R0_R1	20,73996925	-78,41017151	-945,0253296	-0,030882
Cloud_Mesh_Distance_BA_R0_R1	20,56803131	-78,41110229	-945,1810303	-0,045971
Cloud_Mesh_Distance_BA_R0_R1	15,29114246	-78,43397522	-945,0057373	-0,07118
Cloud_Mesh_Distance_BA_R0_R1	15,31740093	-78,25079346	-944,9423828	-0,067779
Cloud_Mesh_Distance_BA_R0_R1	15,49673176	-77,98422241	-945,1380615	-0,064958
Cloud_Mesh_Distance_BA_R0_R1	15,38961315	-78,25064087	-945,1171875	-0,073125
Cloud_Mesh_Distance_BA_R0_R1	20,11286736	-78,02513123	-945,3827515	-0,084108
Cloud_Mesh_Distance_BA_R0_R1	20,31326485	-77,99905396	-945,1893921	-0,07122
Cloud_Mesh_Distance_BA_R0_R1	20,27186584	-78,16967773	-945,3123779	-0,073515

Cloud_Mesh_Distance_BA_R0_R1	20,41617012	-78,07806396	-945,1381226	-0,060963
Cloud_Mesh_Distance_BA_R0_R1	20,57076836	-78,21150208	-945,0703125	-0,047383
Cloud_Mesh_Distance_BA_R0_R1	15,43758869	-77,9037323	-944,918396	-0,060967
Cloud_Mesh_Distance_BA_R0_R1	15,55771446	-77,77719116	-945,0542603	-0,064646
Cloud_Mesh_Distance_BA_R0_R1	20,20770645	-77,91964722	-945,2459106	-0,077739
Cloud_Mesh_Distance_BA_R0_R1	20,18510437	-77,76660156	-945,1844482	-0,070356
Cloud_Mesh_Distance_BA_R0_R1	20,35220718	-77,82263184	-945,0517578	-0,058617
Cloud_Mesh_Distance_BA_R0_R1	15,65280724	-77,5297699	-944,9457398	-0,070407
Cloud_Mesh_Distance_BA_R0_R1	15,68821335	-77,64239502	-945,1575317	-0,071913
Cloud_Mesh_Distance_BA_R0_R1	20,02906609	-77,69689941	-945,2979736	-0,076803
Cloud_Mesh_Distance_BA_R0_R1	15,81118774	-77,28723145	-944,9136353	-0,07169
Cloud_Mesh_Distance_BA_R0_R1	15,88558674	-77,32821655	-945,1154785	-0,080489
Cloud_Mesh_Distance_BA_R0_R1	15,77892113	-77,45571899	-945,0949097	-0,076988
Cloud_Mesh_Distance_BA_R0_R1	19,97544479	-77,55310059	-945,2650147	-0,067401
Cloud_Mesh_Distance_BA_R0_R1	15,9661808	-77,19296265	-945,0640869	-0,082388
Cloud_Mesh_Distance_BA_R0_R1	19,84590149	-77,37117004	-945,2664795	-0,060413
Cloud_Mesh_Distance_BA_R0_R1	19,79550362	-77,49386597	-945,395752	-0,063355
Cloud_Mesh_Distance_BA_R0_R1	19,88352585	-77,24765015	-945,1329956	-0,055996
Cloud_Mesh_Distance_BA_R0_R1	19,48517227	-77,06407166	-945,3034668	-0,038822
Cloud_Mesh_Distance_BA_R0_R1	19,76942635	-77,2592926	-945,2486572	-0,057743
Cloud_Mesh_Distance_BA_R0_R1	19,77138329	-77,14311218	-945,1455078	-0,048255
Cloud_Mesh_Distance_BA_R0_R1	19,64879799	-77,05325317	-945,1651611	-0,045722
Cloud_Mesh_Distance_BA_R0_R1	16,24094009	-77,00408936	-945,2034912	-0,09164
Cloud_Mesh_Distance_BA_R0_R1	16,11044121	-77,09103394	-945,1508179	-0,087915
Cloud_Mesh_Distance_BA_R0_R1	16,40670586	-76,82043457	-945,1083374	-0,101322
Cloud_Mesh_Distance_BA_R0_R1	19,24871635	-76,77407837	-945,1553955	-0,016799
Cloud_Mesh_Distance_BA_R0_R1	16,6140995	-76,66064453	-945,0401611	-0,105469
Cloud_Mesh_Distance_BA_R0_R1	16,56595802	-76,7434845	-945,1506348	-0,098929
Cloud_Mesh_Distance_BA_R0_R1	16,87603951	-76,59721375	-945,1519165	-0,108985
Cloud_Mesh_Distance_BA_R0_R1	16,94064331	-76,64497375	-945,2770996	-0,108071
Cloud_Mesh_Distance_BA_R0_R1	17,1031208	-76,63383484	-945,3400879	-0,103624
Cloud_Mesh_Distance_BA_R0_R1	17,03996086	-76,57145691	-945,2076416	-0,106486
Cloud_Mesh_Distance_BA_R0_R1	17,24037361	-76,54785156	-945,2576294	-0,097689
Cloud_Mesh_Distance_BA_R0_R1	17,29607391	-76,45179749	-945,0959473	-0,098269
Cloud_Mesh_Distance_BA_R0_R1	17,47624779	-76,42330933	-945,0979004	-0,084269
Cloud_Mesh_Distance_BA_R0_R1	17,48766518	-76,50991821	-945,2647095	-0,076926
Cloud_Mesh_Distance_BA_R0_R1	17,76874352	-76,38858032	-945,078125	-0,062285
Cloud_Mesh_Distance_BA_R0_R1	18,29729843	-76,50679016	-945,2476196	-0,005043
Cloud_Mesh_Distance_BA_R0_R1	17,63156319	-76,4221344	-945,1303101	-0,071425
Cloud_Mesh_Distance_BA_R0_R1	17,57291603	-76,35842896	-944,9718628	-0,083995
Cloud_Mesh_Distance_BA_R0_R1	22,53763008	-82,35282898	-944,8745117	-0,070285
Cloud_Mesh_Distance_BA_R0_R1	22,40527344	-82,31974792	-945,0413208	0,219203
Cloud_Mesh_Distance_BA_R0_R1	22,45632553	-82,12017822	-944,8418579	0,010849
Cloud_Mesh_Distance_BA_R0_R1	22,29407692	-81,91229248	-944,9315186	0,005239
Cloud_Mesh_Distance_BA_R0_R1	22,2283783	-81,55026245	-944,8226318	-0,027699
Cloud_Mesh_Distance_BA_R0_R1	15,74005604	-81,30429077	-944,7883911	0,134672
Cloud_Mesh_Distance_BA_R0_R1	15,6977253	-81,24798584	-944,586792	0,039442
Cloud_Mesh_Distance_BA_R0_R1	22,08533669	-81,49925232	-944,9573364	-0,02605
Cloud_Mesh_Distance_BA_R0_R1	15,72772789	-81,11329651	-944,7862549	0,018331
Cloud_Mesh_Distance_BA_R0_R1	21,92905807	-81,15704346	-944,9769287	-0,060161

Cloud_Mesh_Distance_BA_R0_R1	22,0583744	-81,15130615	-944,8471069	-0,067137
Cloud_Mesh_Distance_BA_R0_R1	15,60089493	-80,89505005	-944,616333	-0,104272
Cloud_Mesh_Distance_BA_R0_R1	15,67890644	-80,94155884	-944,7717896	-0,064667
Cloud_Mesh_Distance_BA_R0_R1	15,65911293	-81,06324768	-944,6040039	-0,04569
Cloud_Mesh_Distance_BA_R0_R1	15,53701401	-80,73167419	-944,6444092	-0,140967
Cloud_Mesh_Distance_BA_R0_R1	21,72484589	-80,61915588	-944,9973145	-0,097553
Cloud_Mesh_Distance_BA_R0_R1	21,85773087	-80,74194336	-944,9128418	-0,096878
Cloud_Mesh_Distance_BA_R0_R1	21,75316048	-80,82809448	-945,0317383	-0,085067
Cloud_Mesh_Distance_BA_R0_R1	21,7209034	-80,41537476	-944,942749	-0,106474
Cloud_Mesh_Distance_BA_R0_R1	15,35143948	-80,30621338	-944,7081299	-0,169293
Cloud_Mesh_Distance_BA_R0_R1	15,45678997	-80,44073486	-944,7891235	-0,163359
Cloud_Mesh_Distance_BA_R0_R1	21,87963486	-80,35708618	-944,777832	-0,116072
Cloud_Mesh_Distance_BA_R0_R1	15,27812862	-80,29766846	-944,5297241	-0,161908
Cloud_Mesh_Distance_BA_R0_R1	15,26222134	-80,12464905	-944,6868897	-0,160333
Cloud_Mesh_Distance_BA_R0_R1	21,73161697	-80,25257874	-944,8852539	-0,112212
Cloud_Mesh_Distance_BA_R0_R1	15,18425655	-80,11433411	-944,4673462	-0,157918
Cloud_Mesh_Distance_BA_R0_R1	15,10937309	-79,95741272	-944,3637695	-0,145884
Cloud_Mesh_Distance_BA_R0_R1	15,15470219	-79,90562439	-944,6021729	-0,144761
Cloud_Mesh_Distance_BA_R0_R1	21,56978798	-79,96704102	-944,9399414	-0,106647
Cloud_Mesh_Distance_BA_R0_R1	21,68182755	-80,11224365	-944,887146	-0,112167
Cloud_Mesh_Distance_BA_R0_R1	21,57990074	-80,22265625	-945,008728	-0,102767
Cloud_Mesh_Distance_BA_R0_R1	21,72522545	-79,99229431	-944,8067017	-0,115767
Cloud_Mesh_Distance_BA_R0_R1	21,51088715	-79,69718933	-944,8994141	-0,097751
Cloud_Mesh_Distance_BA_R0_R1	15,10946369	-79,16809082	-944,7835693	-0,087304
Cloud_Mesh_Distance_BA_R0_R1	15,08081055	-79,33666992	-944,6794434	-0,101335
Cloud_Mesh_Distance_BA_R0_R1	15,05488682	-78,94674683	-944,5053101	-0,091778
Cloud_Mesh_Distance_BA_R0_R1	15,09708214	-78,97459412	-944,7064819	-0,087914
Cloud_Mesh_Distance_BA_R0_R1	15,05251312	-79,16088867	-944,5681763	-0,097747
Cloud_Mesh_Distance_BA_R0_R1	15,15075111	-78,81483459	-944,8244019	-0,083265
Cloud_Mesh_Distance_BA_R0_R1	20,89984894	-79,01818848	-945,1591187	-0,055081
Cloud_Mesh_Distance_BA_R0_R1	21,18577576	-79,23620605	-945,0039063	-0,069551
Cloud_Mesh_Distance_BA_R0_R1	15,15812397	-78,62176514	-944,7424316	-0,078776
Cloud_Mesh_Distance_BA_R0_R1	20,98916435	-78,93548584	-945,052002	-0,047449
Cloud_Mesh_Distance_BA_R0_R1	21,10818863	-78,94816589	-944,9532471	-0,04351
Cloud_Mesh_Distance_BA_R0_R1	21,15625381	-78,76113892	-944,8171387	-0,026676
Cloud_Mesh_Distance_BA_R0_R1	21,02565956	-78,79307556	-944,9556274	-0,036286
Cloud_Mesh_Distance_BA_R0_R1	21,22977066	-79,06929016	-944,8988648	-0,051797
Cloud_Mesh_Distance_BA_R0_R1	21,21665001	-78,90704346	-944,835144	-0,040038
Cloud_Mesh_Distance_BA_R0_R1	15,21583843	-78,49118042	-944,8388062	-0,071656
Cloud_Mesh_Distance_BA_R0_R1	20,80897522	-78,58917236	-945,0546265	-0,032808
Cloud_Mesh_Distance_BA_R0_R1	15,18162632	-78,41731262	-944,6601563	-0,07196
Cloud_Mesh_Distance_BA_R0_R1	20,95527649	-78,63841248	-944,9430542	-0,027361
Cloud_Mesh_Distance_BA_R0_R1	15,25441933	-78,32800293	-944,8286743	-0,069102
Cloud_Mesh_Distance_BA_R0_R1	15,36962318	-78,09692383	-944,9437866	-0,061084
Cloud_Mesh_Distance_BA_R0_R1	20,71989441	-78,19169617	-944,9163208	-0,024964
Cloud_Mesh_Distance_BA_R0_R1	20,81166458	-78,30645752	-944,8960571	-0,020733
Cloud_Mesh_Distance_BA_R0_R1	20,89702797	-78,44316101	-944,8916626	-0,022341
Cloud_Mesh_Distance_BA_R0_R1	15,40600204	-77,80789185	-944,7109375	-0,048778
Cloud_Mesh_Distance_BA_R0_R1	20,52131271	-77,96983337	-944,9744873	-0,042655
Cloud_Mesh_Distance_BA_R0_R1	15,50983143	-77,70610046	-944,8552856	-0,060377

Cloud_Mesh_Distance_BA_R0_R1	15,5778017	-77,52850342	-944,7679443	-0,053018
Cloud_Mesh_Distance_BA_R0_R1	20,22298241	-77,65826416	-945,0813599	-0,064499
Cloud_Mesh_Distance_BA_R0_R1	20,11260986	-77,64492798	-945,1852417	-0,071385
Cloud_Mesh_Distance_BA_R0_R1	20,17970276	-77,51345825	-945,0314331	-0,058345
Cloud_Mesh_Distance_BA_R0_R1	20,05781174	-77,50079346	-945,149292	-0,066834
Cloud_Mesh_Distance_BA_R0_R1	20,31161499	-77,5796814	-944,9359131	-0,047152
Cloud_Mesh_Distance_BA_R0_R1	20,13510513	-77,38165283	-944,9829102	-0,047125
Cloud_Mesh_Distance_BA_R0_R1	20,25913239	-77,43710327	-944,8897705	-0,034988
Cloud_Mesh_Distance_BA_R0_R1	16,06620979	-77,02148438	-944,9597168	-0,084852
Cloud_Mesh_Distance_BA_R0_R1	19,97529602	-77,37464905	-945,144104	-0,063111
Cloud_Mesh_Distance_BA_R0_R1	15,93943882	-77,10887146	-944,8720093	-0,078175
Cloud_Mesh_Distance_BA_R0_R1	19,55773735	-76,94059753	-945,1222534	-0,039987
Cloud_Mesh_Distance_BA_R0_R1	15,99660397	-76,99963379	-944,769043	-0,07811
Cloud_Mesh_Distance_BA_R0_R1	16,14769745	-76,89111328	-944,8410645	-0,09363
Cloud_Mesh_Distance_BA_R0_R1	16,23364449	-76,91725159	-945,0457153	-0,090418
Cloud_Mesh_Distance_BA_R0_R1	19,41859245	-76,90907288	-945,1918335	-0,031518
Cloud_Mesh_Distance_BA_R0_R1	19,48881912	-76,77682495	-944,9890747	-0,020434
Cloud_Mesh_Distance_BA_R0_R1	19,72400856	-76,99359131	-945,0427856	-0,040011
Cloud_Mesh_Distance_BA_R0_R1	16,45957565	-76,72103882	-944,9777222	-0,104858
Cloud_Mesh_Distance_BA_R0_R1	16,51852608	-76,62823486	-944,8388062	-0,111463
Cloud_Mesh_Distance_BA_R0_R1	19,34911346	-76,74043274	-945,0458984	-0,016006
Cloud_Mesh_Distance_BA_R0_R1	16,67300415	-76,58184814	-944,9296875	-0,107675
Cloud_Mesh_Distance_BA_R0_R1	16,96967125	-76,52111816	-945,0655518	-0,107858
Cloud_Mesh_Distance_BA_R0_R1	16,9065609	-76,48173523	-944,9228516	-0,115677
Cloud_Mesh_Distance_BA_R0_R1	16,78058624	-76,58070374	-945,0408325	-0,110771
Cloud_Mesh_Distance_BA_R0_R1	17,12400436	-76,50205994	-945,1198731	-0,103986
Cloud_Mesh_Distance_BA_R0_R1	18,08770943	-76,45568848	-945,2005005	-0,023079
Cloud_Mesh_Distance_BA_R0_R1	18,25974083	-76,40884399	-945,0881348	-0,016069
Cloud_Mesh_Distance_BA_R0_R1	18,69996262	-76,50689697	-945,1057129	0,012298
Cloud_Mesh_Distance_BA_R0_R1	18,50358772	-76,45339966	-945,0969238	0,000261
Cloud_Mesh_Distance_BA_R0_R1	18,68446922	-76,42205811	-944,961853	-0,000624
Cloud_Mesh_Distance_BA_R0_R1	18,93028259	-76,68258667	-945,2397461	0,008675
Cloud_Mesh_Distance_BA_R0_R1	18,8819313	-76,57901001	-945,1245117	0,0132
Cloud_Mesh_Distance_BA_R0_R1	19,04891014	-76,67428589	-945,1589966	0,00113
Cloud_Mesh_Distance_BA_R0_R1	18,89734268	-76,46228027	-944,9264526	0,000564
Cloud_Mesh_Distance_BA_R0_R1	17,20814323	-76,3621521	-944,8264771	-0,110881
Cloud_Mesh_Distance_BA_R0_R1	17,40277672	-76,32421875	-944,8170166	-0,101452
Cloud_Mesh_Distance_BA_R0_R1	17,27512741	-76,38973999	-944,9416504	-0,102811
Cloud_Mesh_Distance_BA_R0_R1	17,40106201	-76,38746643	-944,9909058	-0,096603
Cloud_Mesh_Distance_BA_R0_R1	17,60326195	-76,29994202	-944,8140259	-0,090702
Cloud_Mesh_Distance_BA_R0_R1	18,02910042	-76,38624573	-945,0723877	-0,038296
Cloud_Mesh_Distance_BA_R0_R1	17,89383507	-76,41047668	-945,1290283	-0,046735
Cloud_Mesh_Distance_BA_R0_R1	18,17447853	-76,33508301	-944,9360962	-0,035
Cloud_Mesh_Distance_BA_R0_R1	18,37411308	-76,33700562	-944,8955078	-0,023394
Cloud_Mesh_Distance_BA_R0_R1	22,78145599	-82,61694336	-944,6322632	-0,125014
Cloud_Mesh_Distance_BA_R0_R1	22,66301918	-82,52615356	-944,7872925	-0,045559
Cloud_Mesh_Distance_BA_R0_R1	22,86201096	-82,59815979	-944,4537354	0,142453
Cloud_Mesh_Distance_BA_R0_R1	15,73105907	-81,86634827	-944,2592773	0,332892
Cloud_Mesh_Distance_BA_R0_R1	15,76120186	-82,03791809	-944,1724854	0,405202
Cloud_Mesh_Distance_BA_R0_R1	22,6271534	-82,27642822	-944,678894	-0,000977

Cloud_Mesh_Distance_BA_R0_R1	22,42731285	-81,85501099	-944,7242432	-0,010634
Cloud_Mesh_Distance_BA_R0_R1	22,52212906	-81,75012207	-944,5252686	-0,021209
Cloud_Mesh_Distance_BA_R0_R1	15,70879173	-81,44871521	-944,5792847	0,160586
Cloud_Mesh_Distance_BA_R0_R1	15,71702099	-81,63671875	-944,3572998	0,213695
Cloud_Mesh_Distance_BA_R0_R1	22,29871559	-81,72877502	-944,8259277	-0,011004
Cloud_Mesh_Distance_BA_R0_R1	15,6938324	-81,38642883	-944,3380737	0,060778
Cloud_Mesh_Distance_BA_R0_R1	15,58313656	-81,084198	-944,2528687	-0,074685
Cloud_Mesh_Distance_BA_R0_R1	15,64746857	-81,17294312	-944,4108887	-0,031058
Cloud_Mesh_Distance_BA_R0_R1	22,14994621	-81,34832764	-944,8235474	-0,049014
Cloud_Mesh_Distance_BA_R0_R1	15,5139761	-80,81582642	-944,4725952	-0,132118
Cloud_Mesh_Distance_BA_R0_R1	15,50389385	-80,90182495	-944,2877197	-0,116624
Cloud_Mesh_Distance_BA_R0_R1	15,58660889	-80,99334717	-944,4418335	-0,095344
Cloud_Mesh_Distance_BA_R0_R1	15,43859196	-80,63595581	-944,5164185	-0,15709
Cloud_Mesh_Distance_BA_R0_R1	15,4761343	-80,58364868	-944,6756592	-0,154463
Cloud_Mesh_Distance_BA_R0_R1	15,3214283	-80,49311829	-944,3786621	-0,155493
Cloud_Mesh_Distance_BA_R0_R1	15,38192749	-80,46144104	-944,6000977	-0,164459
Cloud_Mesh_Distance_BA_R0_R1	21,99288177	-80,75125122	-944,7832642	-0,104984
Cloud_Mesh_Distance_BA_R0_R1	21,86895752	-80,57165527	-944,8527222	-0,107709
Cloud_Mesh_Distance_BA_R0_R1	15,21342945	-80,28236389	-944,3276367	-0,158431
Cloud_Mesh_Distance_BA_R0_R1	22,0246315	-80,55775452	-944,6925659	-0,118573
Cloud_Mesh_Distance_BA_R0_R1	15,14324284	-80,11988831	-944,2805176	-0,152996
Cloud_Mesh_Distance_BA_R0_R1	21,80628586	-80,14170837	-944,779541	-0,12189
Cloud_Mesh_Distance_BA_R0_R1	22,07528877	-80,23068237	-944,5297852	-0,139297
Cloud_Mesh_Distance_BA_R0_R1	15,08680153	-79,79727173	-944,4510498	-0,134881
Cloud_Mesh_Distance_BA_R0_R1	21,86988068	-79,95097351	-944,645874	-0,129711
Cloud_Mesh_Distance_BA_R0_R1	21,95161819	-80,18745422	-944,6481934	-0,13183
Cloud_Mesh_Distance_BA_R0_R1	15,09894371	-79,68496704	-944,5963135	-0,12562
Cloud_Mesh_Distance_BA_R0_R1	21,69649696	-79,82131958	-944,7722778	-0,113193
Cloud_Mesh_Distance_BA_R0_R1	15,06346035	-79,49902344	-944,5645142	-0,113503
Cloud_Mesh_Distance_BA_R0_R1	15,01893234	-79,28970337	-944,3779297	-0,105518
Cloud_Mesh_Distance_BA_R0_R1	21,28199959	-79,38400269	-944,9790039	-0,080034
Cloud_Mesh_Distance_BA_R0_R1	21,50553703	-79,35829163	-944,7709351	-0,077601
Cloud_Mesh_Distance_BA_R0_R1	21,4381752	-79,4785614	-944,8812256	-0,088975
Cloud_Mesh_Distance_BA_R0_R1	15,0429163	-79,34634399	-944,5193481	-0,108774
Cloud_Mesh_Distance_BA_R0_R1	21,40078163	-79,29316711	-944,8405151	-0,071041
Cloud_Mesh_Distance_BA_R0_R1	21,29525375	-79,21951294	-944,9036255	-0,066118
Cloud_Mesh_Distance_BA_R0_R1	21,3705368	-79,11975098	-944,7932129	-0,056478
Cloud_Mesh_Distance_BA_R0_R1	21,49516106	-79,20184326	-944,711853	-0,063404
Cloud_Mesh_Distance_BA_R0_R1	21,32631302	-78,94390869	-944,749939	-0,035196
Cloud_Mesh_Distance_BA_R0_R1	15,03156471	-78,94648743	-944,3317871	-0,098072
Cloud_Mesh_Distance_BA_R0_R1	21,28775406	-78,81039429	-944,7165527	-0,021397
Cloud_Mesh_Distance_BA_R0_R1	21,39571762	-78,80570984	-944,6056519	-0,015251
Cloud_Mesh_Distance_BA_R0_R1	15,10059166	-78,77804565	-944,6256104	-0,082771
Cloud_Mesh_Distance_BA_R0_R1	21,058918	-78,65040588	-944,8511963	-0,02335
Cloud_Mesh_Distance_BA_R0_R1	21,14021492	-78,5554657	-944,7145996	-0,007726
Cloud_Mesh_Distance_BA_R0_R1	21,27330971	-78,66040039	-944,6431885	-0,003471
Cloud_Mesh_Distance_BA_R0_R1	15,11767197	-78,58576965	-944,5446167	-0,080649
Cloud_Mesh_Distance_BA_R0_R1	15,24213791	-78,21124268	-944,6734009	-0,061112
Cloud_Mesh_Distance_BA_R0_R1	21,01299095	-78,47184753	-944,7930298	-0,011533
Cloud_Mesh_Distance_BA_R0_R1	15,29861832	-78,17366028	-944,8210449	-0,060225

Cloud_Mesh_Distance_BA_R0_R1	15,2486496	-78,0625	-944,4979858	-0,050465
Cloud_Mesh_Distance_BA_R0_R1	21,08038902	-78,39485168	-944,6754761	0,001627
Cloud_Mesh_Distance_BA_R0_R1	15,32310581	-78,02372742	-944,7340088	-0,055841
Cloud_Mesh_Distance_BA_R0_R1	15,32577133	-77,90409851	-944,5800171	-0,047258
Cloud_Mesh_Distance_BA_R0_R1	20,63107872	-78,06828308	-944,927063	-0,029701
Cloud_Mesh_Distance_BA_R0_R1	20,62440491	-77,87719727	-944,8098145	-0,020873
Cloud_Mesh_Distance_BA_R0_R1	15,48509884	-77,63484192	-944,6834717	-0,048125
Cloud_Mesh_Distance_BA_R0_R1	15,52534199	-77,49777222	-944,5578003	-0,047062
Cloud_Mesh_Distance_BA_R0_R1	20,44925117	-77,74066162	-944,8996582	-0,039529
Cloud_Mesh_Distance_BA_R0_R1	15,6630373	-77,3923645	-944,7547607	-0,054343
Cloud_Mesh_Distance_BA_R0_R1	15,74786758	-77,24650574	-944,6929932	-0,061026
Cloud_Mesh_Distance_BA_R0_R1	15,64309502	-77,3168335	-944,5441895	-0,048478
Cloud_Mesh_Distance_BA_R0_R1	15,86259174	-77,12644958	-944,7302246	-0,076873
Cloud_Mesh_Distance_BA_R0_R1	20,1871109	-77,2910614	-944,8519287	-0,032487
Cloud_Mesh_Distance_BA_R0_R1	20,01372719	-77,24700928	-945,0029297	-0,045614
Cloud_Mesh_Distance_BA_R0_R1	20,01875305	-77,08850098	-944,8530884	-0,024746
Cloud_Mesh_Distance_BA_R0_R1	15,74668026	-77,17181396	-944,5105591	-0,057346
Cloud_Mesh_Distance_BA_R0_R1	16,05458832	-76,89665222	-944,6417236	-0,089154
Cloud_Mesh_Distance_BA_R0_R1	16,30071259	-76,79298401	-944,8955078	-0,097709
Cloud_Mesh_Distance_BA_R0_R1	19,58421707	-76,69186401	-944,8041382	-0,00694
Cloud_Mesh_Distance_BA_R0_R1	19,70397568	-76,86825562	-944,9219971	-0,025303
Cloud_Mesh_Distance_BA_R0_R1	19,90753174	-77,10957336	-944,9871826	-0,035277
Cloud_Mesh_Distance_BA_R0_R1	19,87950325	-76,96722412	-944,8702393	-0,025544
Cloud_Mesh_Distance_BA_R0_R1	16,24157333	-76,76333618	-944,7063599	-0,09717
Cloud_Mesh_Distance_BA_R0_R1	16,38999748	-76,64587402	-944,6615601	-0,111814
Cloud_Mesh_Distance_BA_R0_R1	16,38414192	-76,70315552	-944,8188477	-0,104004
Cloud_Mesh_Distance_BA_R0_R1	19,44113731	-76,64427185	-944,8504028	-0,011903
Cloud_Mesh_Distance_BA_R0_R1	16,7692852	-76,50942993	-944,8551636	-0,110065
Cloud_Mesh_Distance_BA_R0_R1	16,67368317	-76,51170349	-944,7371216	-0,115115
Cloud_Mesh_Distance_BA_R0_R1	17,09353828	-76,4336853	-944,9459229	-0,112728
Cloud_Mesh_Distance_BA_R0_R1	18,81563759	-76,38226318	-944,8128052	0,00339
Cloud_Mesh_Distance_BA_R0_R1	19,1678772	-76,66017151	-945,0645752	-0,00363
Cloud_Mesh_Distance_BA_R0_R1	19,27342224	-76,6144104	-944,9295654	-0,007804
Cloud_Mesh_Distance_BA_R0_R1	19,1013279	-76,55389404	-944,9553833	0,000158
Cloud_Mesh_Distance_BA_R0_R1	17,0760746	-76,37515259	-944,7665405	-0,118831
Cloud_Mesh_Distance_BA_R0_R1	17,21790314	-76,30380249	-944,6342163	-0,116149
Cloud_Mesh_Distance_BA_R0_R1	17,75398445	-76,30926514	-944,8739014	-0,07596
Cloud_Mesh_Distance_BA_R0_R1	17,91849709	-76,26385498	-944,7443237	-0,07567
Cloud_Mesh_Distance_BA_R0_R1	17,94553185	-76,32746887	-944,9355469	-0,05681
Cloud_Mesh_Distance_BA_R0_R1	18,11407089	-76,27243042	-944,7645874	-0,057912
Cloud_Mesh_Distance_BA_R0_R1	18,32158089	-76,27862549	-944,7449951	-0,042608
Cloud_Mesh_Distance_BA_R0_R1	18,482687	-76,38041687	-944,9623413	-0,01055
Cloud_Mesh_Distance_BA_R0_R1	18,54534721	-76,29890442	-944,7317505	-0,024589
Cloud_Mesh_Distance_BA_R0_R1	18,64857101	-76,3584137	-944,8426514	-0,003556
Cloud_Mesh_Distance_BA_R0_R1	22,96081352	-82,80226135	-944,3728638	-0,2363
Cloud_Mesh_Distance_BA_R0_R1	22,75576973	-82,43748474	-944,5574341	-0,000175
Cloud_Mesh_Distance_BA_R0_R1	22,84699059	-82,39105225	-944,3362427	0,014249
Cloud_Mesh_Distance_BA_R0_R1	22,73346901	-82,21626282	-944,4465942	0,00588
Cloud_Mesh_Distance_BA_R0_R1	22,59428024	-82,00672913	-944,5595703	-0,004022
Cloud_Mesh_Distance_BA_R0_R1	22,64040947	-81,82281494	-944,3672485	-0,012489

Cloud_Mesh_Distance_BA_R0_R1	15,74381447	-81,57852173	-944,0961304	0,108171
Cloud_Mesh_Distance_BA_R0_R1	15,68165779	-81,36627197	-944,093689	0,011299
Cloud_Mesh_Distance_BA_R0_R1	22,3818264	-81,60992432	-944,6576538	-0,026861
Cloud_Mesh_Distance_BA_R0_R1	22,49204826	-81,54898071	-944,4674072	-0,041579
Cloud_Mesh_Distance_BA_R0_R1	15,64439774	-81,24266052	-944,2300415	-0,025102
Cloud_Mesh_Distance_BA_R0_R1	22,39923859	-81,43707275	-944,5516357	-0,050837
Cloud_Mesh_Distance_BA_R0_R1	22,35642815	-81,26657104	-944,5380859	-0,069016
Cloud_Mesh_Distance_BA_R0_R1	22,31991768	-81,06811523	-944,5101318	-0,092907
Cloud_Mesh_Distance_BA_R0_R1	22,28993797	-81,39697266	-944,6789551	-0,050043
Cloud_Mesh_Distance_BA_R0_R1	22,21405602	-81,1655426	-944,6793823	-0,074327
Cloud_Mesh_Distance_BA_R0_R1	22,43464088	-81,17095947	-944,3875122	-0,088417
Cloud_Mesh_Distance_BA_R0_R1	22,4691391	-81,36238098	-944,4158936	-0,064581
Cloud_Mesh_Distance_BA_R0_R1	15,41811466	-80,79733276	-944,1403198	-0,126263
Cloud_Mesh_Distance_BA_R0_R1	15,41799259	-80,71322632	-944,3288574	-0,140963
Cloud_Mesh_Distance_BA_R0_R1	22,09646225	-80,9564209	-944,7393799	-0,090614
Cloud_Mesh_Distance_BA_R0_R1	22,21323776	-80,96743774	-944,6096191	-0,097483
Cloud_Mesh_Distance_BA_R0_R1	22,282547	-80,89486694	-944,4988403	-0,11005
Cloud_Mesh_Distance_BA_R0_R1	22,40886307	-80,95309448	-944,3455811	-0,113067
Cloud_Mesh_Distance_BA_R0_R1	15,3321991	-80,616745	-944,1750488	-0,142235
Cloud_Mesh_Distance_BA_R0_R1	22,15865326	-80,7673645	-944,6085815	-0,114986
Cloud_Mesh_Distance_BA_R0_R1	22,20269775	-80,33946228	-944,4190674	-0,14495
Cloud_Mesh_Distance_BA_R0_R1	22,18148232	-80,550354	-944,513916	-0,133018
Cloud_Mesh_Distance_BA_R0_R1	22,05207443	-80,37120056	-944,6033325	-0,13014
Cloud_Mesh_Distance_BA_R0_R1	15,16174316	-80,23860168	-944,1312866	-0,148766
Cloud_Mesh_Distance_BA_R0_R1	15,08755875	-80,00823975	-944,100647	-0,142572
Cloud_Mesh_Distance_BA_R0_R1	22,03272629	-80,06990051	-944,5158081	-0,137173
Cloud_Mesh_Distance_BA_R0_R1	15,05666733	-79,7991333	-944,2715454	-0,136029
Cloud_Mesh_Distance_BA_R0_R1	15,02328968	-79,61585999	-944,2327881	-0,125028
Cloud_Mesh_Distance_BA_R0_R1	15,05162048	-79,6428833	-944,4177246	-0,127554
Cloud_Mesh_Distance_BA_R0_R1	21,9985733	-79,89775085	-944,4824829	-0,13559
Cloud_Mesh_Distance_BA_R0_R1	21,80560303	-79,75082397	-944,6327515	-0,120208
Cloud_Mesh_Distance_BA_R0_R1	21,91801071	-79,74612427	-944,5056763	-0,13026
Cloud_Mesh_Distance_BA_R0_R1	22,13752365	-79,9704895	-944,3505859	-0,148318
Cloud_Mesh_Distance_BA_R0_R1	21,6921711	-79,63653564	-944,7037964	-0,106238
Cloud_Mesh_Distance_BA_R0_R1	21,8225193	-79,5872345	-944,5411987	-0,113373
Cloud_Mesh_Distance_BA_R0_R1	15,02719498	-79,4684906	-944,3886719	-0,113159
Cloud_Mesh_Distance_BA_R0_R1	15,00645161	-79,43269348	-944,2121582	-0,121521
Cloud_Mesh_Distance_BA_R0_R1	21,57405663	-79,50540161	-944,7668457	-0,092885
Cloud_Mesh_Distance_BA_R0_R1	21,70671272	-79,46212769	-944,612915	-0,096357
Cloud_Mesh_Distance_BA_R0_R1	15,00314617	-79,24926758	-944,1986084	-0,116561
Cloud_Mesh_Distance_BA_R0_R1	15,02219582	-79,10494995	-944,3655396	-0,10037
Cloud_Mesh_Distance_BA_R0_R1	21,60220528	-79,32104492	-944,6585083	-0,079524
Cloud_Mesh_Distance_BA_R0_R1	21,70179939	-79,28518677	-944,5349731	-0,073497
Cloud_Mesh_Distance_BA_R0_R1	21,46709633	-79,00871277	-944,6446533	-0,037759
Cloud_Mesh_Distance_BA_R0_R1	21,61048889	-79,13072205	-944,5583496	-0,052715
Cloud_Mesh_Distance_BA_R0_R1	21,71725845	-79,02664185	-944,3773804	-0,042617
Cloud_Mesh_Distance_BA_R0_R1	21,56642342	-78,94683838	-944,5064697	-0,030246
Cloud_Mesh_Distance_BA_R0_R1	15,06281471	-78,7593689	-944,3939819	-0,091818
Cloud_Mesh_Distance_BA_R0_R1	15,09726906	-78,54037476	-944,3476563	-0,086376
Cloud_Mesh_Distance_BA_R0_R1	15,0593853	-78,68106079	-944,1777344	-0,097886

Cloud_Mesh_Distance_BA_R0_R1	15,13792515	-78,35290527	-944,3216553	-0,076763
Cloud_Mesh_Distance_BA_R0_R1	15,13968182	-78,42926025	-944,4769287	-0,072725
Cloud_Mesh_Distance_BA_R0_R1	15,18331623	-78,27175903	-944,4924927	-0,05889
Cloud_Mesh_Distance_BA_R0_R1	20,84228325	-78,15414429	-944,7692261	-0,007162
Cloud_Mesh_Distance_BA_R0_R1	20,81084251	-77,97814941	-944,68396	0,00511
Cloud_Mesh_Distance_BA_R0_R1	20,98978043	-78,26611328	-944,6875	0,007325
Cloud_Mesh_Distance_BA_R0_R1	15,39735031	-77,70324707	-944,506897	-0,040174
Cloud_Mesh_Distance_BA_R0_R1	15,3070507	-77,85699463	-944,4040527	-0,039217
Cloud_Mesh_Distance_BA_R0_R1	15,37689304	-77,67089844	-944,3272705	-0,032224
Cloud_Mesh_Distance_BA_R0_R1	20,631073	-77,57174683	-944,5715942	0,008799
Cloud_Mesh_Distance_BA_R0_R1	20,70212364	-77,75843811	-944,6414795	0,005948
Cloud_Mesh_Distance_BA_R0_R1	20,43061638	-77,51954651	-944,7598267	-0,022697
Cloud_Mesh_Distance_BA_R0_R1	20,54019928	-77,63273621	-944,7247925	-0,012419
Cloud_Mesh_Distance_BA_R0_R1	20,25522614	-77,23023987	-944,7163696	-0,016573
Cloud_Mesh_Distance_BA_R0_R1	20,11991692	-77,08743286	-944,7367554	-0,013562
Cloud_Mesh_Distance_BA_R0_R1	20,35815239	-77,38101196	-944,7294312	-0,016934
Cloud_Mesh_Distance_BA_R0_R1	20,47141457	-77,30014038	-944,5112305	0,010205
Cloud_Mesh_Distance_BA_R0_R1	20,49612045	-77,44088745	-944,6173096	0,001336
Cloud_Mesh_Distance_BA_R0_R1	15,91645432	-76,93205261	-944,2872315	-0,076571
Cloud_Mesh_Distance_BA_R0_R1	15,90480328	-77,00653076	-944,5385742	-0,070821
Cloud_Mesh_Distance_BA_R0_R1	16,1349411	-76,78330994	-944,4900513	-0,094167
Cloud_Mesh_Distance_BA_R0_R1	19,90397263	-76,72994995	-944,5393066	0,013458
Cloud_Mesh_Distance_BA_R0_R1	19,75971794	-76,66333008	-944,5946655	0,007526
Cloud_Mesh_Distance_BA_R0_R1	20,00386238	-76,95309448	-944,7218018	-0,013979
Cloud_Mesh_Distance_BA_R0_R1	19,87747955	-76,83522034	-944,7158814	-0,003291
Cloud_Mesh_Distance_BA_R0_R1	16,52459335	-76,50085449	-944,3869629	-0,118804
Cloud_Mesh_Distance_BA_R0_R1	16,44096184	-76,57073975	-944,4957275	-0,117578
Cloud_Mesh_Distance_BA_R0_R1	16,54932594	-76,54197693	-944,6282959	-0,114876
Cloud_Mesh_Distance_BA_R0_R1	16,8907814	-76,42228699	-944,7324829	-0,117463
Cloud_Mesh_Distance_BA_R0_R1	16,91509819	-76,36387634	-944,5454712	-0,119981
Cloud_Mesh_Distance_BA_R0_R1	19,10383797	-76,41470337	-944,708313	0,005432
Cloud_Mesh_Distance_BA_R0_R1	19,33504868	-76,52857971	-944,7504273	-0,000284
Cloud_Mesh_Distance_BA_R0_R1	19,58568573	-76,60369873	-944,6694336	0,004915
Cloud_Mesh_Distance_BA_R0_R1	19,70609093	-76,71517944	-944,7277222	-0,001497
Cloud_Mesh_Distance_BA_R0_R1	17,02789879	-76,35014343	-944,6333008	-0,120026
Cloud_Mesh_Distance_BA_R0_R1	17,4160614	-76,26742554	-944,6248779	-0,108629
Cloud_Mesh_Distance_BA_R0_R1	17,56627464	-76,26251221	-944,6724243	-0,104933
Cloud_Mesh_Distance_BA_R0_R1	17,73833084	-76,25907898	-944,7070923	-0,093292
Cloud_Mesh_Distance_BA_R0_R1	17,54595757	-76,22366333	-944,4990234	-0,108855
Cloud_Mesh_Distance_BA_R0_R1	17,71464348	-76,21954346	-944,5407105	-0,100737
Cloud_Mesh_Distance_BA_R0_R1	18,07931519	-76,22259521	-944,5876465	-0,07551
Cloud_Mesh_Distance_BA_R0_R1	18,46051407	-76,22409058	-944,5096436	-0,054442
Cloud_Mesh_Distance_BA_R0_R1	18,71968842	-76,25523376	-944,5041504	-0,036825
Cloud_Mesh_Distance_BA_R0_R1	18,77075768	-76,31968689	-944,6824951	-0,00717
Cloud_Mesh_Distance_BA_R0_R1	18,95794868	-76,37200928	-944,7093506	0,003967
Cloud_Mesh_Distance_BA_R0_R1	23,04843521	-82,74940491	-944,1190186	0,137086
Cloud_Mesh_Distance_BA_R0_R1	22,95133781	-82,56872559	-944,2276611	0,018012
Cloud_Mesh_Distance_BA_R0_R1	15,9715147	-82,39060974	-943,9384155	0,440556
Cloud_Mesh_Distance_BA_R0_R1	22,82925606	-82,17758179	-944,2200317	0,013655
Cloud_Mesh_Distance_BA_R0_R1	15,85804558	-82,16575623	-944,0244141	0,396087

Cloud_Mesh_Distance_BA_R0_R1	15,97751427	-82,07235718	-943,7519531	0,214642
Cloud_Mesh_Distance_BA_R0_R1	15,82712364	-81,79589844	-943,9382935	0,171074
Cloud_Mesh_Distance_BA_R0_R1	15,89751434	-82,01679993	-943,8972778	0,25569
Cloud_Mesh_Distance_BA_R0_R1	15,80791569	-81,94078064	-944,0738525	0,295284
Cloud_Mesh_Distance_BA_R0_R1	15,76621914	-81,76364136	-944,145813	0,219623
Cloud_Mesh_Distance_BA_R0_R1	22,72650337	-82,00636292	-944,3182983	-0,002484
Cloud_Mesh_Distance_BA_R0_R1	15,78771782	-81,62132263	-943,8739014	0,075465
Cloud_Mesh_Distance_BA_R0_R1	22,72999191	-81,84210205	-944,2068481	-0,006865
Cloud_Mesh_Distance_BA_R0_R1	22,5841732	-81,65498352	-944,371521	-0,033316
Cloud_Mesh_Distance_BA_R0_R1	15,66182709	-81,32049561	-943,9316406	-0,020152
Cloud_Mesh_Distance_BA_R0_R1	22,585495	-81,47833252	-944,2796021	-0,051759
Cloud_Mesh_Distance_BA_R0_R1	15,50691605	-80,98155212	-944,0913086	-0,097291
Cloud_Mesh_Distance_BA_R0_R1	22,53691673	-81,27322388	-944,2689209	-0,078332
Cloud_Mesh_Distance_BA_R0_R1	15,32557297	-80,67672729	-943,8633423	-0,111662
Cloud_Mesh_Distance_BA_R0_R1	15,35038471	-80,70098877	-944,0234985	-0,124356
Cloud_Mesh_Distance_BA_R0_R1	22,3043499	-80,73799133	-944,420105	-0,12576
Cloud_Mesh_Distance_BA_R0_R1	22,55033112	-80,96212769	-944,1260376	-0,121009
Cloud_Mesh_Distance_BA_R0_R1	15,24297142	-80,42947388	-944,1670532	-0,145763
Cloud_Mesh_Distance_BA_R0_R1	15,20060539	-80,38195801	-943,9728394	-0,129853
Cloud_Mesh_Distance_BA_R0_R1	22,33230209	-80,5382843	-944,3186035	-0,143546
Cloud_Mesh_Distance_BA_R0_R1	22,14441872	-80,1627655	-944,4237061	-0,146696
Cloud_Mesh_Distance_BA_R0_R1	22,36123085	-80,33242798	-944,2094727	-0,1568
Cloud_Mesh_Distance_BA_R0_R1	22,17502403	-79,77618408	-944,2098389	-0,147235
Cloud_Mesh_Distance_BA_R0_R1	15,01056099	-79,58074951	-944,0452271	-0,130536
Cloud_Mesh_Distance_BA_R0_R1	15,00029373	-79,39808655	-944,0285645	-0,125184
Cloud_Mesh_Distance_BA_R0_R1	22,03540802	-79,74771118	-944,3684082	-0,141834
Cloud_Mesh_Distance_BA_R0_R1	15,01096344	-79,06773376	-944,1861572	-0,114858
Cloud_Mesh_Distance_BA_R0_R1	21,73942947	-79,18499756	-944,4404297	-0,061795
Cloud_Mesh_Distance_BA_R0_R1	21,81939697	-79,37023926	-944,4404297	-0,090077
Cloud_Mesh_Distance_BA_R0_R1	15,01417351	-79,02523804	-944,0040894	-0,119527
Cloud_Mesh_Distance_BA_R0_R1	15,0296011	-78,88183594	-944,1860352	-0,111461
Cloud_Mesh_Distance_BA_R0_R1	21,82661247	-79,01669312	-944,2255859	-0,041104
Cloud_Mesh_Distance_BA_R0_R1	21,84000587	-79,18545532	-944,3140259	-0,070628
Cloud_Mesh_Distance_BA_R0_R1	21,63220596	-78,84994507	-944,3679199	-0,011778
Cloud_Mesh_Distance_BA_R0_R1	21,72984886	-78,85728455	-944,2461548	-0,015399
Cloud_Mesh_Distance_BA_R0_R1	15,07145596	-78,62895203	-943,9732056	-0,11443
Cloud_Mesh_Distance_BA_R0_R1	21,50811768	-78,76919556	-944,4581909	-0,006678
Cloud_Mesh_Distance_BA_R0_R1	15,10205269	-78,4617157	-944,1403809	-0,091215
Cloud_Mesh_Distance_BA_R0_R1	15,10734081	-78,4624939	-943,946106	-0,105423
Cloud_Mesh_Distance_BA_R0_R1	21,38125229	-78,57885742	-944,4700928	0,00449
Cloud_Mesh_Distance_BA_R0_R1	21,22934532	-78,44352722	-944,5460205	0,010493
Cloud_Mesh_Distance_BA_R0_R1	21,39096832	-78,425354	-944,3410034	0,027048
Cloud_Mesh_Distance_BA_R0_R1	15,14874077	-78,27325439	-944,1746826	-0,076013
Cloud_Mesh_Distance_BA_R0_R1	15,18614769	-78,17118835	-944,3085327	-0,060804
Cloud_Mesh_Distance_BA_R0_R1	15,19451618	-78,10935974	-944,0874634	-0,066243
Cloud_Mesh_Distance_BA_R0_R1	15,24587727	-77,97279358	-944,267334	-0,050162
Cloud_Mesh_Distance_BA_R0_R1	21,26504326	-78,25634766	-944,362793	0,037651
Cloud_Mesh_Distance_BA_R0_R1	20,98327255	-78,08551025	-944,5697022	0,02259
Cloud_Mesh_Distance_BA_R0_R1	21,11126328	-78,21342468	-944,5149536	0,024773
Cloud_Mesh_Distance_BA_R0_R1	21,08260727	-77,99650574	-944,3848267	0,04764

Cloud_Mesh_Distance_BA_R0_R1	21,20783615	-78,08998108	-944,3009033	0,048938
Cloud_Mesh_Distance_BA_R0_R1	20,95009804	-77,89758301	-944,4680786	0,039394
Cloud_Mesh_Distance_BA_R0_R1	15,32010937	-77,76786804	-944,2145386	-0,041985
Cloud_Mesh_Distance_BA_R0_R1	15,46072483	-77,52081299	-944,3458252	-0,033103
Cloud_Mesh_Distance_BA_R0_R1	20,84283829	-77,77502441	-944,4956055	0,032366
Cloud_Mesh_Distance_BA_R0_R1	20,75164604	-77,63183594	-944,4812622	0,027089
Cloud_Mesh_Distance_BA_R0_R1	15,55940819	-77,38269043	-944,4009399	-0,039137
Cloud_Mesh_Distance_BA_R0_R1	15,64433193	-77,2462616	-944,3336182	-0,047966
Cloud_Mesh_Distance_BA_R0_R1	15,53784657	-77,36271667	-944,187439	-0,033513
Cloud_Mesh_Distance_BA_R0_R1	20,93148232	-77,68833923	-944,3135376	0,059198
Cloud_Mesh_Distance_BA_R0_R1	20,28908157	-77,12435913	-944,5649414	0,006311
Cloud_Mesh_Distance_BA_R0_R1	20,51615906	-77,13470459	-944,2498779	0,036197
Cloud_Mesh_Distance_BA_R0_R1	20,58939934	-77,41285706	-944,4752808	0,022691
Cloud_Mesh_Distance_BA_R0_R1	16,00761414	-76,87759399	-944,4310913	-0,08405
Cloud_Mesh_Distance_BA_R0_R1	16,08332634	-76,77706909	-944,2505493	-0,095287
Cloud_Mesh_Distance_BA_R0_R1	20,01438713	-76,85452271	-944,5858154	0,010625
Cloud_Mesh_Distance_BA_R0_R1	20,05056381	-76,7645874	-944,404541	0,027358
Cloud_Mesh_Distance_BA_R0_R1	20,14596176	-76,97663879	-944,5784912	0,004288
Cloud_Mesh_Distance_BA_R0_R1	16,21712494	-76,69088745	-944,3594971	-0,108171
Cloud_Mesh_Distance_BA_R0_R1	16,28982353	-76,67272949	-944,5161133	-0,105969
Cloud_Mesh_Distance_BA_R0_R1	16,43794632	-76,51461792	-944,1290283	-0,112113
Cloud_Mesh_Distance_BA_R0_R1	16,34744263	-76,59100342	-944,3006592	-0,111507
Cloud_Mesh_Distance_BA_R0_R1	19,89484024	-76,56974792	-944,229187	0,017448
Cloud_Mesh_Distance_BA_R0_R1	19,81497383	-76,60705566	-944,428833	0,016939
Cloud_Mesh_Distance_BA_R0_R1	16,73299026	-76,42324829	-944,5008545	-0,116484
Cloud_Mesh_Distance_BA_R0_R1	16,62246895	-76,43244934	-944,2651367	-0,122257
Cloud_Mesh_Distance_BA_R0_R1	16,88715553	-76,33964539	-944,3773193	-0,117281
Cloud_Mesh_Distance_BA_R0_R1	19,47698593	-76,39990234	-944,3346558	0,003865
Cloud_Mesh_Distance_BA_R0_R1	19,51577568	-76,49861145	-944,5360718	0,002313
Cloud_Mesh_Distance_BA_R0_R1	19,66657257	-76,57328796	-944,5334473	0,007997
Cloud_Mesh_Distance_BA_R0_R1	17,22523499	-76,25668335	-944,4315186	-0,119529
Cloud_Mesh_Distance_BA_R0_R1	17,05905914	-76,30099487	-944,4568481	-0,119777
Cloud_Mesh_Distance_BA_R0_R1	17,16799545	-76,24539185	-944,2954102	-0,113762
Cloud_Mesh_Distance_BA_R0_R1	17,3720417	-76,23851013	-944,4678345	-0,116216
Cloud_Mesh_Distance_BA_R0_R1	17,33140182	-76,21234131	-944,2855225	-0,113803
Cloud_Mesh_Distance_BA_R0_R1	17,70920563	-76,18457031	-944,3542481	-0,117197
Cloud_Mesh_Distance_BA_R0_R1	17,52133942	-76,19081116	-944,3032227	-0,116359
Cloud_Mesh_Distance_BA_R0_R1	17,8903141	-76,21853638	-944,5672607	-0,091355
Cloud_Mesh_Distance_BA_R0_R1	18,04621315	-76,17990112	-944,3843384	-0,097412
Cloud_Mesh_Distance_BA_R0_R1	17,8546257	-76,18870544	-944,4163208	-0,103297
Cloud_Mesh_Distance_BA_R0_R1	18,25577927	-76,19358826	-944,4364624	-0,07702
Cloud_Mesh_Distance_BA_R0_R1	18,2490139	-76,23419189	-944,6104736	-0,060482
Cloud_Mesh_Distance_BA_R0_R1	18,93518257	-76,29580688	-944,5108032	-0,017606
Cloud_Mesh_Distance_BA_R0_R1	19,29985619	-76,40179443	-944,524353	0,000312
Cloud_Mesh_Distance_BA_R0_R1	19,14335823	-76,35955811	-944,5482178	-0,005246
Cloud_Mesh_Distance_BA_R0_R1	18,37966156	-76,17875671	-944,328125	-0,077275
Cloud_Mesh_Distance_BA_R0_R1	16,63449287	-82,9619751	-943,581665	0,237045
Cloud_Mesh_Distance_BA_R0_R1	16,77447128	-83,11026001	-943,585144	0,222541
Cloud_Mesh_Distance_BA_R0_R1	16,92072868	-83,25546265	-943,5857544	0,205556
Cloud_Mesh_Distance_BA_R0_R1	23,14837646	-82,9201355	-943,9777222	0,139092

Cloud_Mesh_Distance_BA_R0_R1	16,41002083	-82,76400757	-943,6672974	0,2989
Cloud_Mesh_Distance_BA_R0_R1	16,25688553	-82,67533875	-943,7817383	0,375513
Cloud_Mesh_Distance_BA_R0_R1	23,10760498	-82,71278381	-943,9301148	0,086934
Cloud_Mesh_Distance_BA_R0_R1	16,31153297	-82,55709839	-943,5780029	0,245737
Cloud_Mesh_Distance_BA_R0_R1	16,06939697	-82,19850159	-943,6618652	0,219591
Cloud_Mesh_Distance_BA_R0_R1	16,12717628	-82,42431641	-943,7507935	0,319973
Cloud_Mesh_Distance_BA_R0_R1	22,81448174	-81,96922302	-944,1066895	0,005579
Cloud_Mesh_Distance_BA_R0_R1	22,9309082	-82,34623718	-944,1069336	0,031811
Cloud_Mesh_Distance_BA_R0_R1	22,90676117	-82,13204956	-944,0018311	0,030659
Cloud_Mesh_Distance_BA_R0_R1	15,98398209	-82,21180725	-943,8377075	0,308949
Cloud_Mesh_Distance_BA_R0_R1	15,85643291	-81,72850037	-943,7258301	0,090325
Cloud_Mesh_Distance_BA_R0_R1	15,92027569	-81,91351318	-943,7497559	0,15787
Cloud_Mesh_Distance_BA_R0_R1	15,68479443	-81,35769653	-943,7687988	-0,014497
Cloud_Mesh_Distance_BA_R0_R1	15,72416496	-81,46542358	-943,9105835	0,021845
Cloud_Mesh_Distance_BA_R0_R1	22,67365265	-81,67181396	-944,2182617	-0,034491
Cloud_Mesh_Distance_BA_R0_R1	15,60805416	-81,21037292	-943,829773	-0,053209
Cloud_Mesh_Distance_BA_R0_R1	15,63194847	-81,2454071	-943,6710205	-0,044347
Cloud_Mesh_Distance_BA_R0_R1	22,68235207	-81,28016663	-944,0064087	-0,083729
Cloud_Mesh_Distance_BA_R0_R1	22,62218475	-81,34152222	-944,1489258	-0,074085
Cloud_Mesh_Distance_BA_R0_R1	22,59443092	-81,17739868	-944,1292114	-0,097614
Cloud_Mesh_Distance_BA_R0_R1	15,52186775	-81,04618835	-943,888794	-0,084055
Cloud_Mesh_Distance_BA_R0_R1	15,60245323	-81,18321228	-944,0465088	-0,05058
Cloud_Mesh_Distance_BA_R0_R1	22,50691223	-81,10705566	-944,2513428	-0,099905
Cloud_Mesh_Distance_BA_R0_R1	22,65752029	-81,09411621	-943,9798584	-0,108162
Cloud_Mesh_Distance_BA_R0_R1	15,27729321	-80,55465698	-944,0045166	-0,125152
Cloud_Mesh_Distance_BA_R0_R1	15,41949272	-80,8515625	-943,9280396	-0,110021
Cloud_Mesh_Distance_BA_R0_R1	22,44405174	-80,7434845	-944,2237549	-0,135786
Cloud_Mesh_Distance_BA_R0_R1	15,24870968	-80,51174927	-943,8203735	-0,117816
Cloud_Mesh_Distance_BA_R0_R1	15,1353159	-80,20243835	-943,9306641	-0,138602
Cloud_Mesh_Distance_BA_R0_R1	15,18237495	-80,340271	-943,7746582	-0,125741
Cloud_Mesh_Distance_BA_R0_R1	15,12979889	-80,16848755	-943,7330933	-0,128641
Cloud_Mesh_Distance_BA_R0_R1	15,09393597	-80,05844116	-943,8706665	-0,136558
Cloud_Mesh_Distance_BA_R0_R1	15,0887394	-79,99688721	-943,7116699	-0,131282
Cloud_Mesh_Distance_BA_R0_R1	15,05771255	-79,89833069	-943,8868408	-0,136135
Cloud_Mesh_Distance_BA_R0_R1	15,02854824	-79,71517944	-943,8799439	-0,1367
Cloud_Mesh_Distance_BA_R0_R1	22,25999069	-80,15800476	-944,2788086	-0,156034
Cloud_Mesh_Distance_BA_R0_R1	22,28873825	-79,96530151	-944,1569214	-0,159965
Cloud_Mesh_Distance_BA_R0_R1	22,40935898	-79,9526825	-943,9822998	-0,163162
Cloud_Mesh_Distance_BA_R0_R1	15,03520679	-79,77171326	-944,0673828	-0,137635
Cloud_Mesh_Distance_BA_R0_R1	22,31415558	-79,77789307	-944,0256958	-0,150785
Cloud_Mesh_Distance_BA_R0_R1	22,07008171	-79,59494019	-944,2455444	-0,128086
Cloud_Mesh_Distance_BA_R0_R1	21,94734955	-79,53807068	-944,3695679	-0,116419
Cloud_Mesh_Distance_BA_R0_R1	21,9372654	-79,32069397	-944,263794	-0,094991
Cloud_Mesh_Distance_BA_R0_R1	15,00178146	-79,21409607	-944,0151367	-0,120737
Cloud_Mesh_Distance_BA_R0_R1	22,05522919	-79,25386047	-944,05896	-0,08617
Cloud_Mesh_Distance_BA_R0_R1	15,03755283	-78,82931519	-943,9934082	-0,11746
Cloud_Mesh_Distance_BA_R0_R1	15,03259659	-78,98124695	-943,8073731	-0,130958
Cloud_Mesh_Distance_BA_R0_R1	15,06198215	-78,77648926	-943,7922363	-0,125574
Cloud_Mesh_Distance_BA_R0_R1	21,93638992	-79,12962341	-944,1465454	-0,063341
Cloud_Mesh_Distance_BA_R0_R1	21,49210739	-78,55705261	-944,3199463	0,016506

Cloud_Mesh_Distance_BA_R0_R1	21,62167931	-78,69676208	-944,265625	0,002466
Cloud_Mesh_Distance_BA_R0_R1	21,62002754	-78,53408813	-944,1279297	0,021718
Cloud_Mesh_Distance_BA_R0_R1	21,82640076	-78,85369873	-944,1049194	-0,014324
Cloud_Mesh_Distance_BA_R0_R1	21,7342968	-78,69198608	-944,1046143	0,003919
Cloud_Mesh_Distance_BA_R0_R1	15,14103222	-78,30706787	-944,0136108	-0,09131
Cloud_Mesh_Distance_BA_R0_R1	15,23718929	-78,01205444	-943,8726807	-0,065879
Cloud_Mesh_Distance_BA_R0_R1	15,26913071	-77,88485718	-944,0587769	-0,045533
Cloud_Mesh_Distance_BA_R0_R1	15,41356373	-77,55873108	-944,1311035	-0,031801
Cloud_Mesh_Distance_BA_R0_R1	15,34282017	-77,70124817	-944,0249634	-0,037732
Cloud_Mesh_Distance_BA_R0_R1	15,42381191	-77,55593872	-943,8479614	-0,039396
Cloud_Mesh_Distance_BA_R0_R1	20,96741676	-77,58760071	-944,1618652	0,080218
Cloud_Mesh_Distance_BA_R0_R1	21,09078217	-77,73382568	-944,1412964	0,082687
Cloud_Mesh_Distance_BA_R0_R1	21,04695702	-77,82704163	-944,2893066	0,059995
Cloud_Mesh_Distance_BA_R0_R1	21,08093262	-77,61654663	-944,0251465	0,095036
Cloud_Mesh_Distance_BA_R0_R1	20,84142494	-77,53071594	-944,2709961	0,058892
Cloud_Mesh_Distance_BA_R0_R1	20,69859695	-77,46168518	-944,3845825	0,036612
Cloud_Mesh_Distance_BA_R0_R1	15,66558647	-77,18414307	-944,1247559	-0,048137
Cloud_Mesh_Distance_BA_R0_R1	15,727952	-77,1053772	-943,9310913	-0,057902
Cloud_Mesh_Distance_BA_R0_R1	20,60977364	-77,30706787	-944,3327637	0,032831
Cloud_Mesh_Distance_BA_R0_R1	20,75984001	-77,35081482	-944,1725464	0,058831
Cloud_Mesh_Distance_BA_R0_R1	15,78147316	-77,07875061	-944,3225098	-0,060566
Cloud_Mesh_Distance_BA_R0_R1	15,80614662	-77,02111816	-944,1083984	-0,067011
Cloud_Mesh_Distance_BA_R0_R1	20,42760277	-77,12631226	-944,3717651	0,026734
Cloud_Mesh_Distance_BA_R0_R1	20,25916862	-76,963974	-944,4049683	0,018328
Cloud_Mesh_Distance_BA_R0_R1	20,62240601	-77,18389893	-944,1540527	0,049581
Cloud_Mesh_Distance_BA_R0_R1	15,96590805	-76,86289978	-944,111023	-0,076437
Cloud_Mesh_Distance_BA_R0_R1	16,76429558	-76,37355042	-944,2965088	-0,118131
Cloud_Mesh_Distance_BA_R0_R1	16,7390213	-76,36482239	-944,1254273	-0,112371
Cloud_Mesh_Distance_BA_R0_R1	19,69530106	-76,50720215	-944,3539429	0,008063
Cloud_Mesh_Distance_BA_R0_R1	16,8731308	-76,31855774	-944,19104	-0,116853
Cloud_Mesh_Distance_BA_R0_R1	16,87507629	-76,30400085	-944,0085449	-0,10462
Cloud_Mesh_Distance_BA_R0_R1	17,00200462	-76,26568604	-944,0846558	-0,106709
Cloud_Mesh_Distance_BA_R0_R1	17,01986313	-76,27818298	-944,253418	-0,114842
Cloud_Mesh_Distance_BA_R0_R1	17,20209885	-76,21640015	-944,1260376	-0,111615
Cloud_Mesh_Distance_BA_R0_R1	17,71289825	-76,1550293	-944,1305542	-0,118501
Cloud_Mesh_Distance_BA_R0_R1	18,53553581	-76,18988037	-944,3197632	-0,064399
Cloud_Mesh_Distance_BA_R0_R1	18,7170639	-76,20480347	-944,27948	-0,053745
Cloud_Mesh_Distance_BA_R0_R1	18,92676544	-76,21058655	-944,1176758	-0,05827
Cloud_Mesh_Distance_BA_R0_R1	18,92112541	-76,24514771	-944,3221436	-0,033715
Cloud_Mesh_Distance_BA_R0_R1	19,11337471	-76,28886414	-944,3394775	-0,016284
Cloud_Mesh_Distance_BA_R0_R1	19,10860634	-76,24891663	-944,1481934	-0,036867
Cloud_Mesh_Distance_BA_R0_R1	19,31933594	-76,35362244	-944,3688355	-0,003089
Cloud_Mesh_Distance_BA_R0_R1	19,47166061	-76,35647583	-944,1876831	-0,008415
Cloud_Mesh_Distance_BA_R0_R1	18,22643471	-76,14196777	-944,0693359	-0,111894
Cloud_Mesh_Distance_BA_R0_R1	18,22769356	-76,16238403	-944,2614746	-0,091959
Cloud_Mesh_Distance_BA_R0_R1	18,41555595	-76,1567688	-944,1612549	-0,088405
Cloud_Mesh_Distance_BA_R0_R1	16,87417603	-83,14134216	-943,4090576	0,151862
Cloud_Mesh_Distance_BA_R0_R1	16,5241642	-82,83837891	-943,5822144	0,246839
Cloud_Mesh_Distance_BA_R0_R1	23,17386436	-82,76452637	-943,7827148	0,093173
Cloud_Mesh_Distance_BA_R0_R1	16,46057129	-82,70718384	-943,5014648	0,210102

Cloud_Mesh_Distance_BA_R0_R1	23,12053108	-82,60379028	-943,798645	0,09014
Cloud_Mesh_Distance_BA_R0_R1	16,40737915	-82,57669067	-943,4106445	0,169762
Cloud_Mesh_Distance_BA_R0_R1	23,03824806	-82,52423096	-943,9674072	0,059543
Cloud_Mesh_Distance_BA_R0_R1	16,11269379	-82,1348114	-943,4793701	0,140819
Cloud_Mesh_Distance_BA_R0_R1	16,19450378	-82,33035278	-943,5358276	0,196628
Cloud_Mesh_Distance_BA_R0_R1	23,03198624	-82,17323303	-943,6771851	0,070285
Cloud_Mesh_Distance_BA_R0_R1	22,99608231	-82,28038025	-943,8795776	0,062721
Cloud_Mesh_Distance_BA_R0_R1	23,09288788	-82,40713501	-943,7006836	0,094017
Cloud_Mesh_Distance_BA_R0_R1	22,96347618	-82,09672546	-943,8217773	0,046751
Cloud_Mesh_Distance_BA_R0_R1	22,8956337	-81,91671753	-943,8717041	0,018203
Cloud_Mesh_Distance_BA_R0_R1	16,03738213	-81,95471191	-943,427002	0,089133
Cloud_Mesh_Distance_BA_R0_R1	16,01112175	-82,01986694	-943,6087036	0,154006
Cloud_Mesh_Distance_BA_R0_R1	15,94375896	-81,84329224	-943,5682373	0,093877
Cloud_Mesh_Distance_BA_R0_R1	15,7743454	-81,52775574	-943,6905518	0,021488
Cloud_Mesh_Distance_BA_R0_R1	22,78584862	-81,73484802	-944,0205078	-0,016804
Cloud_Mesh_Distance_BA_R0_R1	15,70114899	-81,35745239	-943,5765991	-0,025269
Cloud_Mesh_Distance_BA_R0_R1	22,76779938	-81,37535095	-943,8678589	-0,070192
Cloud_Mesh_Distance_BA_R0_R1	22,79857635	-81,55386353	-943,8878174	-0,038394
Cloud_Mesh_Distance_BA_R0_R1	22,86876106	-81,68963623	-943,7912598	-0,014314
Cloud_Mesh_Distance_BA_R0_R1	22,73824501	-81,18792725	-943,852356	-0,098205
Cloud_Mesh_Distance_BA_R0_R1	22,70422363	-81,49230957	-944,0615234	-0,048649
Cloud_Mesh_Distance_BA_R0_R1	15,53332615	-81,03198242	-943,4429932	-0,059038
Cloud_Mesh_Distance_BA_R0_R1	15,54441738	-81,08711243	-943,6685791	-0,064091
Cloud_Mesh_Distance_BA_R0_R1	15,46121693	-80,94096375	-943,7595825	-0,087095
Cloud_Mesh_Distance_BA_R0_R1	15,452178	-80,91081238	-943,5818481	-0,078309
Cloud_Mesh_Distance_BA_R0_R1	15,36881638	-80,73902893	-943,5269775	-0,08396
Cloud_Mesh_Distance_BA_R0_R1	15,38378239	-80,79490662	-943,7235107	-0,100102
Cloud_Mesh_Distance_BA_R0_R1	22,65137672	-80,91166687	-943,9281616	-0,131132
Cloud_Mesh_Distance_BA_R0_R1	15,23901463	-80,46603394	-943,6223145	-0,104232
Cloud_Mesh_Distance_BA_R0_R1	15,30557919	-80,63189697	-943,6772461	-0,102964
Cloud_Mesh_Distance_BA_R0_R1	15,29990196	-80,58247375	-943,50354	-0,086983
Cloud_Mesh_Distance_BA_R0_R1	22,59293938	-80,59672546	-943,9360962	-0,157028
Cloud_Mesh_Distance_BA_R0_R1	22,56731796	-80,74633789	-944,0262451	-0,145569
Cloud_Mesh_Distance_BA_R0_R1	22,48198318	-80,5282135	-944,0980225	-0,151876
Cloud_Mesh_Distance_BA_R0_R1	22,66514969	-80,73052979	-943,8447266	-0,151985
Cloud_Mesh_Distance_BA_R0_R1	15,1811924	-80,28683472	-943,5731201	-0,109061
Cloud_Mesh_Distance_BA_R0_R1	22,4812603	-80,29981995	-944,0217896	-0,16502
Cloud_Mesh_Distance_BA_R0_R1	22,66608429	-80,54273987	-943,7873535	-0,163399
Cloud_Mesh_Distance_BA_R0_R1	22,57644272	-80,42697144	-943,9118652	-0,163899
Cloud_Mesh_Distance_BA_R0_R1	15,05598927	-79,81764221	-943,7097168	-0,137025
Cloud_Mesh_Distance_BA_R0_R1	22,38814163	-80,13653564	-944,0966797	-0,163301
Cloud_Mesh_Distance_BA_R0_R1	15,03427982	-79,66069031	-943,7298584	-0,137621
Cloud_Mesh_Distance_BA_R0_R1	15,0303688	-79,51266479	-943,6783447	-0,136872
Cloud_Mesh_Distance_BA_R0_R1	15,0129137	-79,54153442	-943,8640137	-0,134518
Cloud_Mesh_Distance_BA_R0_R1	22,42200661	-79,76745605	-943,8618774	-0,154232
Cloud_Mesh_Distance_BA_R0_R1	15,00867653	-79,36408997	-943,8422241	-0,132548
Cloud_Mesh_Distance_BA_R0_R1	22,06722641	-79,45542908	-944,1696777	-0,117954
Cloud_Mesh_Distance_BA_R0_R1	22,19744873	-79,60682678	-944,0856934	-0,137558
Cloud_Mesh_Distance_BA_R0_R1	15,0308876	-79,32562256	-943,6561279	-0,135089
Cloud_Mesh_Distance_BA_R0_R1	15,0147438	-79,17718506	-943,8259888	-0,135571

Cloud_Mesh_Distance_BA_R0_R1	22,18623543	-79,45579529	-944,0073853	-0,119153
Cloud_Mesh_Distance_BA_R0_R1	22,21771622	-79,3527832	-943,8909302	-0,103708
Cloud_Mesh_Distance_BA_R0_R1	15,09691525	-78,72261047	-943,6220703	-0,136711
Cloud_Mesh_Distance_BA_R0_R1	22,01160431	-79,06015015	-943,9854126	-0,056656
Cloud_Mesh_Distance_BA_R0_R1	22,04808044	-78,93814087	-943,8269653	-0,034279
Cloud_Mesh_Distance_BA_R0_R1	21,94251442	-78,89935303	-943,9633179	-0,031348
Cloud_Mesh_Distance_BA_R0_R1	21,89817238	-78,98417664	-944,0997315	-0,039199
Cloud_Mesh_Distance_BA_R0_R1	22,15041924	-79,16116333	-943,8502808	-0,071371
Cloud_Mesh_Distance_BA_R0_R1	15,1055212	-78,56071472	-943,763794	-0,122518
Cloud_Mesh_Distance_BA_R0_R1	21,7102375	-78,52560425	-943,9829102	0,026712
Cloud_Mesh_Distance_BA_R0_R1	21,8486805	-78,71287537	-943,9463501	-0,002294
Cloud_Mesh_Distance_BA_R0_R1	15,14559364	-78,35299683	-943,8220215	-0,106861
Cloud_Mesh_Distance_BA_R0_R1	15,1804018	-78,19342041	-943,8917236	-0,082779
Cloud_Mesh_Distance_BA_R0_R1	15,15887833	-78,41059875	-943,6478882	-0,119772
Cloud_Mesh_Distance_BA_R0_R1	21,60782242	-78,354599	-943,9680176	0,043634
Cloud_Mesh_Distance_BA_R0_R1	21,71925354	-78,41342163	-943,8466187	0,035577
Cloud_Mesh_Distance_BA_R0_R1	21,37005043	-78,14889526	-944,1279907	0,053397
Cloud_Mesh_Distance_BA_R0_R1	21,4532547	-78,33485413	-944,1799316	0,04288
Cloud_Mesh_Distance_BA_R0_R1	15,29701424	-77,92221069	-943,6754761	-0,07496
Cloud_Mesh_Distance_BA_R0_R1	15,31610394	-77,79191589	-943,8617554	-0,051105
Cloud_Mesh_Distance_BA_R0_R1	15,36664581	-77,72886658	-943,6972046	-0,056763
Cloud_Mesh_Distance_BA_R0_R1	21,50138855	-78,18902588	-943,9636841	0,054909
Cloud_Mesh_Distance_BA_R0_R1	21,39095688	-77,98718262	-943,9312744	0,078546
Cloud_Mesh_Distance_BA_R0_R1	21,21099091	-77,91711426	-944,145752	0,072534
Cloud_Mesh_Distance_BA_R0_R1	21,22669792	-77,78326416	-943,9846191	0,091783
Cloud_Mesh_Distance_BA_R0_R1	15,49116898	-77,42022705	-943,9968262	-0,034744
Cloud_Mesh_Distance_BA_R0_R1	15,59402657	-77,27119446	-943,970459	-0,042391
Cloud_Mesh_Distance_BA_R0_R1	15,53837204	-77,36239624	-943,8449707	-0,03479
Cloud_Mesh_Distance_BA_R0_R1	20,92977333	-77,45751953	-944,0562134	0,079311
Cloud_Mesh_Distance_BA_R0_R1	20,72388268	-77,18997192	-943,9943237	0,063026
Cloud_Mesh_Distance_BA_R0_R1	20,85037613	-77,31822205	-943,9837647	0,07881
Cloud_Mesh_Distance_BA_R0_R1	15,8946619	-76,92547607	-943,9468384	-0,069069
Cloud_Mesh_Distance_BA_R0_R1	15,82033348	-77,01502991	-943,7823486	-0,065039
Cloud_Mesh_Distance_BA_R0_R1	20,53647614	-77,04727173	-944,0794067	0,04837
Cloud_Mesh_Distance_BA_R0_R1	20,45145798	-76,92552185	-944,0059815	0,039495
Cloud_Mesh_Distance_BA_R0_R1	20,38699341	-76,97149658	-944,2092285	0,029731
Cloud_Mesh_Distance_BA_R0_R1	20,58821869	-77,01261902	-943,918396	0,051972
Cloud_Mesh_Distance_BA_R0_R1	15,98424149	-76,84880066	-943,7884522	-0,072026
Cloud_Mesh_Distance_BA_R0_R1	16,08211708	-76,75549316	-943,9743652	-0,085248
Cloud_Mesh_Distance_BA_R0_R1	16,23270226	-76,64532471	-944,1104736	-0,096221
Cloud_Mesh_Distance_BA_R0_R1	16,23768234	-76,63616943	-943,8347168	-0,081142
Cloud_Mesh_Distance_BA_R0_R1	20,08845711	-76,67153931	-944,1393433	0,022975
Cloud_Mesh_Distance_BA_R0_R1	20,21336937	-76,80661011	-944,2154541	0,033166
Cloud_Mesh_Distance_BA_R0_R1	20,24348831	-76,73519897	-943,9841309	0,027822
Cloud_Mesh_Distance_BA_R0_R1	16,49005127	-76,47599792	-943,9553223	-0,101806
Cloud_Mesh_Distance_BA_R0_R1	16,60878372	-76,41424561	-943,8383179	-0,095125
Cloud_Mesh_Distance_BA_R0_R1	16,74337959	-76,35340881	-943,9247437	-0,106576
Cloud_Mesh_Distance_BA_R0_R1	16,60268211	-76,42166138	-944,0482178	-0,112169
Cloud_Mesh_Distance_BA_R0_R1	19,67862701	-76,43101501	-944,1455078	-0,000508
Cloud_Mesh_Distance_BA_R0_R1	17,47092628	-76,16642761	-944,0657959	-0,11429

Cloud_Mesh_Distance_BA_R0_R1	17,29748344	-76,18362427	-943,9432373	-0,109709
Cloud_Mesh_Distance_BA_R0_R1	17,71624374	-76,13858032	-943,8790894	-0,123983
Cloud_Mesh_Distance_BA_R0_R1	17,91222191	-76,15823364	-944,2241211	-0,111818
Cloud_Mesh_Distance_BA_R0_R1	17,94067764	-76,13699341	-943,9750977	-0,130756
Cloud_Mesh_Distance_BA_R0_R1	18,09104156	-76,14680481	-944,1400757	-0,110578
Cloud_Mesh_Distance_BA_R0_R1	18,75354958	-76,1787262	-944,0628662	-0,080511
Cloud_Mesh_Distance_BA_R0_R1	18,55402374	-76,15100098	-943,9714966	-0,097949
Cloud_Mesh_Distance_BA_R0_R1	18,76407051	-76,16650391	-943,8542481	-0,100134
Cloud_Mesh_Distance_BA_R0_R1	18,59451866	-76,17303467	-944,1739502	-0,075149
Cloud_Mesh_Distance_BA_R0_R1	18,94003677	-76,19177246	-943,9145508	-0,07755
Cloud_Mesh_Distance_BA_R0_R1	19,28283882	-76,29414368	-944,1693726	-0,023414
Cloud_Mesh_Distance_BA_R0_R1	19,28129005	-76,26348877	-943,9807129	-0,037721
Cloud_Mesh_Distance_BA_R0_R1	19,43828773	-76,31062317	-944,008606	-0,025699
Cloud_Mesh_Distance_BA_R0_R1	17,08940506	-83,33708191	-943,3024292	0,110133
Cloud_Mesh_Distance_BA_R0_R1	16,98358154	-83,20001221	-943,2246704	0,091869
Cloud_Mesh_Distance_BA_R0_R1	17,0051651	-83,28425598	-943,4237671	0,146246
Cloud_Mesh_Distance_BA_R0_R1	16,82874489	-83,02104187	-943,2197876	0,093285
Cloud_Mesh_Distance_BA_R0_R1	16,66727448	-82,82481384	-943,2216187	0,098035
Cloud_Mesh_Distance_BA_R0_R1	16,58947945	-82,83148193	-943,4389648	0,179071
Cloud_Mesh_Distance_BA_R0_R1	16,72877121	-82,98106384	-943,4101563	0,160405
Cloud_Mesh_Distance_BA_R0_R1	23,24018097	-82,95053101	-943,7455444	0,089529
Cloud_Mesh_Distance_BA_R0_R1	23,18256378	-82,6101532	-943,6096802	0,124239
Cloud_Mesh_Distance_BA_R0_R1	23,23258781	-82,790802	-943,62854	0,118395
Cloud_Mesh_Distance_BA_R0_R1	16,54693604	-82,62518311	-943,1278687	0,070474
Cloud_Mesh_Distance_BA_R0_R1	16,42737961	-82,51574707	-943,2538452	0,108501
Cloud_Mesh_Distance_BA_R0_R1	16,51715088	-82,67967224	-943,3270264	0,135891
Cloud_Mesh_Distance_BA_R0_R1	16,22224236	-82,24378967	-943,3423462	0,112984
Cloud_Mesh_Distance_BA_R0_R1	16,31676102	-82,42697144	-943,3886108	0,15135
Cloud_Mesh_Distance_BA_R0_R1	22,98069572	-81,97714233	-943,6730957	0,044335
Cloud_Mesh_Distance_BA_R0_R1	16,13642693	-82,06347656	-943,2900391	0,076857
Cloud_Mesh_Distance_BA_R0_R1	15,8680954	-81,66175842	-943,519043	0,042723
Cloud_Mesh_Distance_BA_R0_R1	15,96036339	-81,77767944	-943,3695068	0,044349
Cloud_Mesh_Distance_BA_R0_R1	15,87913036	-81,60121155	-943,3040772	0,009671
Cloud_Mesh_Distance_BA_R0_R1	22,9364357	-81,81123352	-943,6834106	0,017541
Cloud_Mesh_Distance_BA_R0_R1	22,93133163	-81,64503479	-943,5887451	-0,010648
Cloud_Mesh_Distance_BA_R0_R1	15,78737354	-81,48374939	-943,4495239	-0,002045
Cloud_Mesh_Distance_BA_R0_R1	22,93132401	-81,45977783	-943,4853516	-0,043392
Cloud_Mesh_Distance_BA_R0_R1	22,85782814	-81,46870422	-943,6955566	-0,046079
Cloud_Mesh_Distance_BA_R0_R1	15,53809834	-80,97537231	-943,2307129	-0,041892
Cloud_Mesh_Distance_BA_R0_R1	15,62116623	-81,19995117	-943,5050049	-0,044172
Cloud_Mesh_Distance_BA_R0_R1	22,81403923	-81,25683594	-943,708252	-0,085229
Cloud_Mesh_Distance_BA_R0_R1	22,71516037	-81,03430176	-943,8432617	-0,119439
Cloud_Mesh_Distance_BA_R0_R1	15,35861206	-80,63893127	-943,303894	-0,072406
Cloud_Mesh_Distance_BA_R0_R1	22,7818718	-81,07382202	-943,7125855	-0,110614
Cloud_Mesh_Distance_BA_R0_R1	15,25427628	-80,41688538	-943,3907471	-0,093057
Cloud_Mesh_Distance_BA_R0_R1	22,76068878	-80,68916321	-943,6413574	-0,151145
Cloud_Mesh_Distance_BA_R0_R1	22,84730911	-80,73043823	-943,4586792	-0,149642
Cloud_Mesh_Distance_BA_R0_R1	15,13242149	-80,09889221	-943,5474243	-0,121854
Cloud_Mesh_Distance_BA_R0_R1	22,73523521	-80,48057556	-943,6340332	-0,164913
Cloud_Mesh_Distance_BA_R0_R1	15,09479523	-79,91098022	-943,5389404	-0,130755

Cloud_Mesh_Distance_BA_R0_R1	22,50219727	-80,10818481	-943,9138794	-0,164575
Cloud_Mesh_Distance_BA_R0_R1	22,58114052	-80,22386169	-943,8323975	-0,167953
Cloud_Mesh_Distance_BA_R0_R1	22,57895851	-80,04574585	-943,7595825	-0,161981
Cloud_Mesh_Distance_BA_R0_R1	22,6613884	-80,34152222	-943,7317505	-0,16875
Cloud_Mesh_Distance_BA_R0_R1	15,06548119	-79,6968689	-943,5397949	-0,134095
Cloud_Mesh_Distance_BA_R0_R1	15,14534092	-79,99967957	-943,3674927	-0,123659
Cloud_Mesh_Distance_BA_R0_R1	15,11260033	-79,82591248	-943,3842773	-0,127269
Cloud_Mesh_Distance_BA_R0_R1	22,60523033	-79,90943909	-943,6426392	-0,154102
Cloud_Mesh_Distance_BA_R0_R1	22,50819206	-79,9181366	-943,8127441	-0,161572
Cloud_Mesh_Distance_BA_R0_R1	22,51998711	-79,73989868	-943,6878662	-0,1416
Cloud_Mesh_Distance_BA_R0_R1	15,0586853	-79,27488708	-943,5057373	-0,140724
Cloud_Mesh_Distance_BA_R0_R1	15,04169941	-79,13911438	-943,6358643	-0,135408
Cloud_Mesh_Distance_BA_R0_R1	22,28908348	-79,27474976	-943,7202759	-0,088886
Cloud_Mesh_Distance_BA_R0_R1	22,4314537	-79,60069275	-943,739563	-0,131438
Cloud_Mesh_Distance_BA_R0_R1	22,32414818	-79,58139038	-943,8919067	-0,132895
Cloud_Mesh_Distance_BA_R0_R1	22,36829185	-79,43695068	-943,720459	-0,108654
Cloud_Mesh_Distance_BA_R0_R1	15,08049583	-79,12872314	-943,444519	-0,139814
Cloud_Mesh_Distance_BA_R0_R1	22,20760345	-79,08468628	-943,6915283	-0,060452
Cloud_Mesh_Distance_BA_R0_R1	15,12754345	-78,77224731	-943,4405518	-0,136352
Cloud_Mesh_Distance_BA_R0_R1	15,07202911	-78,92840576	-943,5840454	-0,137815
Cloud_Mesh_Distance_BA_R0_R1	15,14374733	-78,57606506	-943,5358887	-0,130317
Cloud_Mesh_Distance_BA_R0_R1	21,95905685	-78,72239685	-943,7704468	-0,001621
Cloud_Mesh_Distance_BA_R0_R1	15,21725368	-78,18692017	-943,6571045	-0,105477
Cloud_Mesh_Distance_BA_R0_R1	15,20643044	-78,39305115	-943,4599609	-0,12473
Cloud_Mesh_Distance_BA_R0_R1	15,41553307	-77,90159607	-943,2910767	-0,095883
Cloud_Mesh_Distance_BA_R0_R1	21,41812134	-77,86828613	-943,7354126	0,088571
Cloud_Mesh_Distance_BA_R0_R1	15,45356464	-77,59698486	-943,5836182	-0,057212
Cloud_Mesh_Distance_BA_R0_R1	15,65651512	-77,21427917	-943,7565308	-0,050569
Cloud_Mesh_Distance_BA_R0_R1	15,66379929	-77,2767334	-943,5250855	-0,054198
Cloud_Mesh_Distance_BA_R0_R1	20,95590401	-77,36671448	-943,8779907	0,093487
Cloud_Mesh_Distance_BA_R0_R1	21,02526283	-77,35414124	-943,7209473	0,103183
Cloud_Mesh_Distance_BA_R0_R1	21,06677055	-77,49635315	-943,8840942	0,096326
Cloud_Mesh_Distance_BA_R0_R1	20,73506355	-77,09649658	-943,795044	0,073315
Cloud_Mesh_Distance_BA_R0_R1	16,03292465	-76,83230591	-943,5884399	-0,061364
Cloud_Mesh_Distance_BA_R0_R1	16,12761688	-76,7288208	-943,7227783	-0,067566
Cloud_Mesh_Distance_BA_R0_R1	16,36863899	-76,54675293	-943,9096069	-0,093298
Cloud_Mesh_Distance_BA_R0_R1	16,25426483	-76,63871765	-943,6333008	-0,06675
Cloud_Mesh_Distance_BA_R0_R1	16,377285	-76,54911804	-943,6901245	-0,075067
Cloud_Mesh_Distance_BA_R0_R1	16,48234749	-76,48086548	-943,7887573	-0,088603
Cloud_Mesh_Distance_BA_R0_R1	20,12798119	-76,62579346	-943,9077759	0,020692
Cloud_Mesh_Distance_BA_R0_R1	20,27811813	-76,69711304	-943,7750855	0,023614
Cloud_Mesh_Distance_BA_R0_R1	20,33477211	-76,84283447	-944,0593872	0,035542
Cloud_Mesh_Distance_BA_R0_R1	16,56041908	-76,45373535	-943,5725098	-0,069148
Cloud_Mesh_Distance_BA_R0_R1	17,00727654	-76,26216125	-943,6994019	-0,097839
Cloud_Mesh_Distance_BA_R0_R1	16,88944817	-76,29623413	-943,8348999	-0,101573
Cloud_Mesh_Distance_BA_R0_R1	16,78642464	-76,34130859	-943,6923828	-0,090887
Cloud_Mesh_Distance_BA_R0_R1	19,58983231	-76,33886719	-943,8571167	-0,025742
Cloud_Mesh_Distance_BA_R0_R1	19,57331848	-76,36276245	-944,0410156	-0,013763
Cloud_Mesh_Distance_BA_R0_R1	19,72378349	-76,40116882	-943,9150391	-0,012125
Cloud_Mesh_Distance_BA_R0_R1	19,87820435	-76,44906616	-943,7666626	-0,008486

Cloud_Mesh_Distance_BA_R0_R1	19,9872303	-76,56504822	-944,0269165	0,018608
Cloud_Mesh_Distance_BA_R0_R1	19,8596344	-76,47952271	-943,9782105	0,009701
Cloud_Mesh_Distance_BA_R0_R1	20,00748444	-76,53094482	-943,8325806	0,00882
Cloud_Mesh_Distance_BA_R0_R1	17,0712719	-76,23762512	-943,9225464	-0,102031
Cloud_Mesh_Distance_BA_R0_R1	17,25214958	-76,19425964	-943,7450562	-0,105419
Cloud_Mesh_Distance_BA_R0_R1	17,75587082	-76,14257813	-943,6318359	-0,132755
Cloud_Mesh_Distance_BA_R0_R1	17,9674778	-76,13499451	-943,7200317	-0,138557
Cloud_Mesh_Distance_BA_R0_R1	18,31720161	-76,13751221	-943,9362183	-0,118342
Cloud_Mesh_Distance_BA_R0_R1	18,20429993	-76,13577271	-943,7340698	-0,138347
Cloud_Mesh_Distance_BA_R0_R1	18,42437363	-76,14205933	-943,7294922	-0,135642
Cloud_Mesh_Distance_BA_R0_R1	19,11320496	-76,22398376	-943,9533081	-0,060092
Cloud_Mesh_Distance_BA_R0_R1	18,13391495	-76,13417053	-943,9257813	-0,125155
Cloud_Mesh_Distance_BA_R0_R1	17,11749649	-83,3261261	-943,1430664	0,073159
Cloud_Mesh_Distance_BA_R0_R1	17,13531113	-83,3013916	-942,958252	0,033773
Cloud_Mesh_Distance_BA_R0_R1	17,04085159	-83,21554565	-943,0480957	0,04936
Cloud_Mesh_Distance_BA_R0_R1	16,92823219	-83,0743103	-943,0175171	0,039608
Cloud_Mesh_Distance_BA_R0_R1	23,29935837	-82,90916443	-943,5368652	0,126404
Cloud_Mesh_Distance_BA_R0_R1	16,78788376	-82,90551758	-943,0311279	0,042434
Cloud_Mesh_Distance_BA_R0_R1	23,22333336	-82,56462097	-943,4169922	0,159276
Cloud_Mesh_Distance_BA_R0_R1	23,33710098	-82,83261108	-943,3378906	0,160313
Cloud_Mesh_Distance_BA_R0_R1	23,26070023	-82,7230835	-943,4691162	0,146052
Cloud_Mesh_Distance_BA_R0_R1	16,33168602	-82,34324646	-943,2008667	0,084636
Cloud_Mesh_Distance_BA_R0_R1	16,23821831	-82,16650391	-943,149292	0,061993
Cloud_Mesh_Distance_BA_R0_R1	16,15113831	-81,99356079	-943,0944824	0,037249
Cloud_Mesh_Distance_BA_R0_R1	16,34109497	-82,26397705	-943,0008545	0,04105
Cloud_Mesh_Distance_BA_R0_R1	23,10286713	-82,2376709	-943,4941406	0,112124
Cloud_Mesh_Distance_BA_R0_R1	16,05515671	-81,88819885	-943,2304077	0,045881
Cloud_Mesh_Distance_BA_R0_R1	23,06340981	-81,9246521	-943,3330078	0,065178
Cloud_Mesh_Distance_BA_R0_R1	23,04969788	-82,0393219	-943,4941406	0,074357
Cloud_Mesh_Distance_BA_R0_R1	15,99341297	-81,64775085	-942,927063	0,009644
Cloud_Mesh_Distance_BA_R0_R1	15,97429657	-81,71343994	-943,159668	0,02076
Cloud_Mesh_Distance_BA_R0_R1	22,99960327	-81,82388306	-943,4899292	0,030053
Cloud_Mesh_Distance_BA_R0_R1	22,98741341	-81,61433411	-943,3876343	-0,001576
Cloud_Mesh_Distance_BA_R0_R1	15,79489136	-81,42726135	-943,2322998	-0,011998
Cloud_Mesh_Distance_BA_R0_R1	15,7106905	-81,2562561	-943,161377	-0,021311
Cloud_Mesh_Distance_BA_R0_R1	15,70476913	-81,31430054	-943,3705444	-0,027189
Cloud_Mesh_Distance_BA_R0_R1	15,62032127	-81,146698	-943,2994995	-0,036598
Cloud_Mesh_Distance_BA_R0_R1	15,88950825	-81,53642273	-943,0881958	0,001341
Cloud_Mesh_Distance_BA_R0_R1	22,88399315	-81,29116821	-943,5427856	-0,074081
Cloud_Mesh_Distance_BA_R0_R1	15,62988377	-81,08621216	-943,0924072	-0,027551
Cloud_Mesh_Distance_BA_R0_R1	15,4470911	-80,8525238	-943,3724976	-0,06199
Cloud_Mesh_Distance_BA_R0_R1	22,84620285	-81,13365173	-943,5817871	-0,102628
Cloud_Mesh_Distance_BA_R0_R1	15,31962967	-80,43432617	-943,1419678	-0,074003
Cloud_Mesh_Distance_BA_R0_R1	22,73694992	-80,89498901	-943,7515259	-0,133823
Cloud_Mesh_Distance_BA_R0_R1	15,24731827	-80,27363586	-943,2208252	-0,091333
Cloud_Mesh_Distance_BA_R0_R1	15,18870926	-80,19891357	-943,3795166	-0,110214
Cloud_Mesh_Distance_BA_R0_R1	15,20880795	-80,087677	-943,1921997	-0,099355
Cloud_Mesh_Distance_BA_R0_R1	22,7748661	-80,12971497	-943,4223633	-0,155421
Cloud_Mesh_Distance_BA_R0_R1	22,67270851	-80,12518311	-943,6269531	-0,164991
Cloud_Mesh_Distance_BA_R0_R1	22,75875664	-80,31768799	-943,5325317	-0,163844

Cloud_Mesh_Distance_BA_R0_R1	22,7430706	-79,97515869	-943,4121704	-0,145458
Cloud_Mesh_Distance_BA_R0_R1	15,1118927	-79,65412903	-943,314148	-0,131253
Cloud_Mesh_Distance_BA_R0_R1	15,06669426	-79,46398926	-943,4628296	-0,138159
Cloud_Mesh_Distance_BA_R0_R1	15,09574223	-79,29878235	-943,3436279	-0,136965
Cloud_Mesh_Distance_BA_R0_R1	22,51140022	-79,53929138	-943,5560913	-0,116959
Cloud_Mesh_Distance_BA_R0_R1	15,1295414	-79,14775085	-943,2536011	-0,137127
Cloud_Mesh_Distance_BA_R0_R1	15,17548561	-78,99191284	-943,1619873	-0,135769
Cloud_Mesh_Distance_BA_R0_R1	15,16726017	-78,82954407	-943,2625732	-0,142375
Cloud_Mesh_Distance_BA_R0_R1	15,11942291	-78,9672699	-943,3561401	-0,137922
Cloud_Mesh_Distance_BA_R0_R1	22,11533928	-78,89332581	-943,6696777	-0,029189
Cloud_Mesh_Distance_BA_R0_R1	22,18937492	-78,85169983	-943,4873047	-0,013571
Cloud_Mesh_Distance_BA_R0_R1	15,28459454	-78,37739563	-943,2200317	-0,128572
Cloud_Mesh_Distance_BA_R0_R1	21,83212471	-78,5443573	-943,7996826	0,020706
Cloud_Mesh_Distance_BA_R0_R1	15,26867294	-78,21717834	-943,4115601	-0,11047
Cloud_Mesh_Distance_BA_R0_R1	21,80729675	-78,40856934	-943,6824341	0,035738
Cloud_Mesh_Distance_BA_R0_R1	21,8039341	-78,2756958	-943,5091553	0,054918
Cloud_Mesh_Distance_BA_R0_R1	21,96073914	-78,52134705	-943,5316162	0,026887
Cloud_Mesh_Distance_BA_R0_R1	15,3144474	-78,02114868	-943,4672241	-0,093267
Cloud_Mesh_Distance_BA_R0_R1	15,34965038	-78,13063049	-943,2474976	-0,111709
Cloud_Mesh_Distance_BA_R0_R1	21,66317749	-78,24606323	-943,7468262	0,054913
Cloud_Mesh_Distance_BA_R0_R1	21,51047707	-78,03285217	-943,7676392	0,073764
Cloud_Mesh_Distance_BA_R0_R1	21,62459564	-78,06410217	-943,5796509	0,068753
Cloud_Mesh_Distance_BA_R0_R1	21,49134064	-77,85720825	-943,562439	0,092837
Cloud_Mesh_Distance_BA_R0_R1	15,50733662	-77,65002441	-943,3380127	-0,076232
Cloud_Mesh_Distance_BA_R0_R1	15,3829813	-77,80058289	-943,5083618	-0,067876
Cloud_Mesh_Distance_BA_R0_R1	15,55885601	-77,46089172	-943,477478	-0,058657
Cloud_Mesh_Distance_BA_R0_R1	21,31943321	-77,78182983	-943,8161621	0,09651
Cloud_Mesh_Distance_BA_R0_R1	21,21652412	-77,64855957	-943,8314209	0,107167
Cloud_Mesh_Distance_BA_R0_R1	15,54283237	-77,40255737	-943,6656494	-0,046784
Cloud_Mesh_Distance_BA_R0_R1	15,66237545	-77,40310669	-943,2921753	-0,066047
Cloud_Mesh_Distance_BA_R0_R1	21,04605675	-77,29650879	-943,5478516	0,104174
Cloud_Mesh_Distance_BA_R0_R1	21,18586159	-77,44519043	-943,538269	0,109661
Cloud_Mesh_Distance_BA_R0_R1	21,2601223	-77,57467651	-943,6202393	0,110205
Cloud_Mesh_Distance_BA_R0_R1	21,16503906	-77,51377869	-943,7218018	0,107997
Cloud_Mesh_Distance_BA_R0_R1	15,83109665	-77,25682068	-943,1229248	-0,061318
Cloud_Mesh_Distance_BA_R0_R1	15,81047916	-77,14813232	-943,3677979	-0,06193
Cloud_Mesh_Distance_BA_R0_R1	15,78855419	-77,0894165	-943,598938	-0,058466
Cloud_Mesh_Distance_BA_R0_R1	20,86493301	-77,24104309	-943,8256226	0,085106
Cloud_Mesh_Distance_BA_R0_R1	15,90827084	-76,94244385	-943,6576538	-0,065423
Cloud_Mesh_Distance_BA_R0_R1	20,57525826	-76,92001343	-943,7248535	0,052851
Cloud_Mesh_Distance_BA_R0_R1	20,70713234	-76,98977661	-943,585144	0,068585
Cloud_Mesh_Distance_BA_R0_R1	20,88862801	-77,18133545	-943,6497192	0,084513
Cloud_Mesh_Distance_BA_R0_R1	16,18507385	-76,71453857	-943,4967041	-0,055601
Cloud_Mesh_Distance_BA_R0_R1	20,43041992	-76,84664917	-943,8657227	0,037429
Cloud_Mesh_Distance_BA_R0_R1	20,4146347	-76,77310181	-943,6740112	0,035184
Cloud_Mesh_Distance_BA_R0_R1	20,1534214	-76,59399414	-943,6972046	0,013114
Cloud_Mesh_Distance_BA_R0_R1	16,78317451	-76,36817932	-943,4476929	-0,070718
Cloud_Mesh_Distance_BA_R0_R1	19,73241043	-76,35612488	-943,4969482	-0,030369
Cloud_Mesh_Distance_BA_R0_R1	19,74145317	-76,3782196	-943,7050781	-0,021473
Cloud_Mesh_Distance_BA_R0_R1	17,16109276	-76,23318481	-943,5419922	-0,096486

Cloud_Mesh_Distance_BA_R0_R1	17,62828636	-76,16201782	-943,496582	-0,122436
Cloud_Mesh_Distance_BA_R0_R1	17,49574852	-76,15054321	-943,7744141	-0,111031
Cloud_Mesh_Distance_BA_R0_R1	17,81313515	-76,15704346	-943,4422607	-0,134579
Cloud_Mesh_Distance_BA_R0_R1	18,36293221	-76,14904785	-943,5374146	-0,14665
Cloud_Mesh_Distance_BA_R0_R1	18,56849861	-76,15725708	-943,5366211	-0,141213
Cloud_Mesh_Distance_BA_R0_R1	18,63437843	-76,1541748	-943,72052	-0,123782
Cloud_Mesh_Distance_BA_R0_R1	18,7690258	-76,17047119	-943,5266724	-0,129006
Cloud_Mesh_Distance_BA_R0_R1	18,81349373	-76,16986084	-943,6721802	-0,116297
Cloud_Mesh_Distance_BA_R0_R1	18,95624352	-76,18585205	-943,7176514	-0,098461
Cloud_Mesh_Distance_BA_R0_R1	19,13861465	-76,20968628	-943,567688	-0,090171
Cloud_Mesh_Distance_BA_R0_R1	19,12047005	-76,21124268	-943,7607422	-0,077706
Cloud_Mesh_Distance_BA_R0_R1	19,27347755	-76,23371887	-943,6256714	-0,072803
Cloud_Mesh_Distance_BA_R0_R1	19,28315735	-76,24453735	-943,7883301	-0,05912
Cloud_Mesh_Distance_BA_R0_R1	19,44309616	-76,28692627	-943,8145752	-0,042694
Cloud_Mesh_Distance_BA_R0_R1	17,48139954	-83,6466217	-942,7929077	0,038471
Cloud_Mesh_Distance_BA_R0_R1	17,3585434	-83,54095459	-942,9245606	0,041935
Cloud_Mesh_Distance_BA_R0_R1	17,35548401	-83,49606323	-942,713562	0,018346
Cloud_Mesh_Distance_BA_R0_R1	17,2378788	-83,4355011	-943,0473633	0,056915
Cloud_Mesh_Distance_BA_R0_R1	16,95317841	-83,04820251	-942,8158569	0,005212
Cloud_Mesh_Distance_BA_R0_R1	17,03902054	-83,17298889	-942,8920898	0,019204
Cloud_Mesh_Distance_BA_R0_R1	16,86235809	-82,95446777	-942,8880005	0,0146
Cloud_Mesh_Distance_BA_R0_R1	16,66911316	-82,66281128	-942,7805176	-0,003788
Cloud_Mesh_Distance_BA_R0_R1	16,66719627	-82,73294067	-942,9880371	0,034155
Cloud_Mesh_Distance_BA_R0_R1	23,22173309	-82,43582153	-943,2545776	0,17657
Cloud_Mesh_Distance_BA_R0_R1	23,16445923	-82,42256165	-943,4690552	0,140192
Cloud_Mesh_Distance_BA_R0_R1	23,28370285	-82,63873291	-943,2835693	0,180083
Cloud_Mesh_Distance_BA_R0_R1	23,29594803	-82,54624939	-943,0901489	0,212622
Cloud_Mesh_Distance_BA_R0_R1	16,44278336	-82,44366455	-943,0554199	0,054149
Cloud_Mesh_Distance_BA_R0_R1	23,15719604	-82,28652954	-943,3338623	0,146874
Cloud_Mesh_Distance_BA_R0_R1	23,18129349	-82,24156189	-943,1602173	0,159242
Cloud_Mesh_Distance_BA_R0_R1	16,24642944	-82,09118652	-942,9559326	0,028132
Cloud_Mesh_Distance_BA_R0_R1	16,33781433	-82,17788696	-942,8100586	0,015213
Cloud_Mesh_Distance_BA_R0_R1	16,16186714	-81,92572021	-942,9009399	0,018757
Cloud_Mesh_Distance_BA_R0_R1	16,07043266	-81,82559204	-943,029419	0,020669
Cloud_Mesh_Distance_BA_R0_R1	23,10497475	-81,90663147	-943,1209106	0,074921
Cloud_Mesh_Distance_BA_R0_R1	23,11593819	-82,10174561	-943,2901611	0,113629
Cloud_Mesh_Distance_BA_R0_R1	23,15161705	-82,08624268	-943,092041	0,131681
Cloud_Mesh_Distance_BA_R0_R1	23,04441643	-81,75744629	-943,2684326	0,027884
Cloud_Mesh_Distance_BA_R0_R1	23,07597733	-81,71072388	-943,0849609	0,032049
Cloud_Mesh_Distance_BA_R0_R1	15,80400181	-81,36123657	-943,0215454	-0,009522
Cloud_Mesh_Distance_BA_R0_R1	15,89814281	-81,46118164	-942,8807983	-0,000143
Cloud_Mesh_Distance_BA_R0_R1	22,93853188	-81,30825806	-943,3931885	-0,067627
Cloud_Mesh_Distance_BA_R0_R1	22,97910309	-81,24711609	-943,239502	-0,078355
Cloud_Mesh_Distance_BA_R0_R1	15,55809021	-80,91964722	-943,0284424	-0,033343
Cloud_Mesh_Distance_BA_R0_R1	15,72313404	-81,18838501	-942,9547119	-0,016054
Cloud_Mesh_Distance_BA_R0_R1	22,91249847	-81,11593628	-943,4000244	-0,101162
Cloud_Mesh_Distance_BA_R0_R1	15,46463585	-80,79719543	-943,1542358	-0,051483
Cloud_Mesh_Distance_BA_R0_R1	15,41348171	-80,61572266	-943,0501709	-0,058856
Cloud_Mesh_Distance_BA_R0_R1	22,83190727	-80,92755127	-943,550293	-0,128475
Cloud_Mesh_Distance_BA_R0_R1	22,89075279	-80,65820313	-943,3325806	-0,148265

Cloud_Mesh_Distance_BA_R0_R1	22,82400131	-80,52824402	-943,4570923	-0,154995
Cloud_Mesh_Distance_BA_R0_R1	15,16865444	-79,84710693	-943,1854248	-0,116753
Cloud_Mesh_Distance_BA_R0_R1	22,62941933	-79,73937988	-943,4885254	-0,131226
Cloud_Mesh_Distance_BA_R0_R1	15,14194584	-79,3013916	-943,1846314	-0,133278
Cloud_Mesh_Distance_BA_R0_R1	15,12365723	-79,45825195	-943,2393189	-0,131378
Cloud_Mesh_Distance_BA_R0_R1	15,18309116	-79,40367126	-943,0573731	-0,125812
Cloud_Mesh_Distance_BA_R0_R1	22,59893417	-79,54959106	-943,3960571	-0,1066
Cloud_Mesh_Distance_BA_R0_R1	22,62414551	-79,43782043	-943,2289429	-0,090409
Cloud_Mesh_Distance_BA_R0_R1	22,41072464	-79,34295654	-943,5704956	-0,095763
Cloud_Mesh_Distance_BA_R0_R1	22,51741982	-79,35772705	-943,3793335	-0,085588
Cloud_Mesh_Distance_BA_R0_R1	15,19308853	-79,18795776	-943,0547485	-0,126486
Cloud_Mesh_Distance_BA_R0_R1	22,44104958	-79,14355469	-943,3088379	-0,052931
Cloud_Mesh_Distance_BA_R0_R1	22,3566227	-79,16888428	-943,5061035	-0,062121
Cloud_Mesh_Distance_BA_R0_R1	22,31414223	-78,94261169	-943,340271	-0,028205
Cloud_Mesh_Distance_BA_R0_R1	22,2298336	-78,98806763	-943,5585327	-0,038181
Cloud_Mesh_Distance_BA_R0_R1	15,19999218	-78,61645508	-943,2940064	-0,134859
Cloud_Mesh_Distance_BA_R0_R1	15,27951336	-78,58052063	-943,09021	-0,133052
Cloud_Mesh_Distance_BA_R0_R1	22,07696152	-78,59007263	-943,3847656	0,02312
Cloud_Mesh_Distance_BA_R0_R1	22,07121468	-78,71728516	-943,5602417	-0,001847
Cloud_Mesh_Distance_BA_R0_R1	22,18827629	-78,73220825	-943,34021	0,00244
Cloud_Mesh_Distance_BA_R0_R1	15,39012623	-78,26312256	-943,0367432	-0,116311
Cloud_Mesh_Distance_BA_R0_R1	21,91373444	-78,34320068	-943,3733521	0,044833
Cloud_Mesh_Distance_BA_R0_R1	21,83314323	-78,18978882	-943,3085327	0,060418
Cloud_Mesh_Distance_BA_R0_R1	21,43014145	-77,70704651	-943,4505005	0,105492
Cloud_Mesh_Distance_BA_R0_R1	21,48423576	-77,68617249	-943,2528076	0,100921
Cloud_Mesh_Distance_BA_R0_R1	21,33403969	-77,57424927	-943,4326172	0,110792
Cloud_Mesh_Distance_BA_R0_R1	21,35486984	-77,70368958	-943,6263428	0,103312
Cloud_Mesh_Distance_BA_R0_R1	21,1417923	-77,34262085	-943,4050903	0,106025
Cloud_Mesh_Distance_BA_R0_R1	20,95931435	-77,18934631	-943,4907227	0,093579
Cloud_Mesh_Distance_BA_R0_R1	15,92351723	-76,97026062	-943,4811401	-0,059199
Cloud_Mesh_Distance_BA_R0_R1	16,07482529	-76,8540802	-943,3522339	-0,049424
Cloud_Mesh_Distance_BA_R0_R1	16,02371407	-76,97862244	-943,1801148	-0,048157
Cloud_Mesh_Distance_BA_R0_R1	16,14419365	-76,86517334	-943,1459961	-0,035979
Cloud_Mesh_Distance_BA_R0_R1	20,85355377	-77,08581543	-943,4741211	0,083838
Cloud_Mesh_Distance_BA_R0_R1	16,27289963	-76,70637512	-943,2514648	-0,0314
Cloud_Mesh_Distance_BA_R0_R1	20,49370003	-76,80093384	-943,5401611	0,038003
Cloud_Mesh_Distance_BA_R0_R1	16,35663795	-76,58837891	-943,4766846	-0,05288
Cloud_Mesh_Distance_BA_R0_R1	16,49694061	-76,54064941	-943,2780151	-0,03568
Cloud_Mesh_Distance_BA_R0_R1	20,02415657	-76,50546265	-943,6287842	-0,006449
Cloud_Mesh_Distance_BA_R0_R1	20,15596962	-76,56559753	-943,5125122	-0,002206
Cloud_Mesh_Distance_BA_R0_R1	20,2142086	-76,58135986	-943,3389282	-0,002834
Cloud_Mesh_Distance_BA_R0_R1	20,40842056	-76,7068634	-943,355957	0,026311
Cloud_Mesh_Distance_BA_R0_R1	20,30580521	-76,66741943	-943,5516357	0,016872
Cloud_Mesh_Distance_BA_R0_R1	20,59007454	-76,84292603	-943,385437	0,04855
Cloud_Mesh_Distance_BA_R0_R1	16,65542412	-76,43946838	-943,3527222	-0,049741
Cloud_Mesh_Distance_BA_R0_R1	16,98422432	-76,28779602	-943,5076904	-0,086794
Cloud_Mesh_Distance_BA_R0_R1	17,21397972	-76,24847412	-943,340332	-0,087463
Cloud_Mesh_Distance_BA_R0_R1	19,56059074	-76,29660034	-943,4616089	-0,046022
Cloud_Mesh_Distance_BA_R0_R1	19,67010498	-76,32775879	-943,3396606	-0,042349
Cloud_Mesh_Distance_BA_R0_R1	19,88676643	-76,42677307	-943,5586548	-0,014498

Cloud_Mesh_Distance_BA_R0_R1	17,38904953	-76,18955994	-943,5101318	-0,109513
Cloud_Mesh_Distance_BA_R0_R1	17,57407188	-76,19252014	-943,3081665	-0,113389
Cloud_Mesh_Distance_BA_R0_R1	17,9619236	-76,14889526	-943,4869995	-0,141385
Cloud_Mesh_Distance_BA_R0_R1	17,95589828	-76,16830444	-943,3035889	-0,146269
Cloud_Mesh_Distance_BA_R0_R1	18,15628624	-76,14605713	-943,5227661	-0,150072
Cloud_Mesh_Distance_BA_R0_R1	18,7189045	-76,17828369	-943,3555908	-0,143956
Cloud_Mesh_Distance_BA_R0_R1	18,9591732	-76,18731689	-943,531311	-0,114271
Cloud_Mesh_Distance_BA_R0_R1	18,92928123	-76,19354248	-943,3435669	-0,125516
Cloud_Mesh_Distance_BA_R0_R1	19,14154625	-76,21437073	-943,3641357	-0,104355
Cloud_Mesh_Distance_BA_R0_R1	19,35613632	-76,24703979	-943,4243164	-0,075483
Cloud_Mesh_Distance_BA_R0_R1	19,43496323	-76,26925659	-943,6131592	-0,052744
Cloud_Mesh_Distance_BA_R0_R1	19,59466362	-76,31871033	-943,6559448	-0,03012
Cloud_Mesh_Distance_BA_R0_R1	17,13411522	-83,25648499	-942,7723999	0,007021
Cloud_Mesh_Distance_BA_R0_R1	17,04261398	-83,1375885	-942,7347412	-0,005166
Cloud_Mesh_Distance_BA_R0_R1	17,23611832	-83,3460083	-942,6403198	-0,0011
Cloud_Mesh_Distance_BA_R0_R1	17,24255562	-83,3971405	-942,8430176	0,024059
Cloud_Mesh_Distance_BA_R0_R1	16,77182007	-82,82366943	-942,8450317	0,00702
Cloud_Mesh_Distance_BA_R0_R1	16,57122231	-82,49603271	-942,7042236	-0,005638
Cloud_Mesh_Distance_BA_R0_R1	23,36637306	-82,6383667	-942,9267578	0,241628
Cloud_Mesh_Distance_BA_R0_R1	16,55798912	-82,55387878	-942,9143677	0,022751
Cloud_Mesh_Distance_BA_R0_R1	16,45009804	-82,35943604	-942,824585	0,009934
Cloud_Mesh_Distance_BA_R0_R1	23,24368095	-82,36361694	-943,0291138	0,207312
Cloud_Mesh_Distance_BA_R0_R1	16,25061417	-82,01641846	-942,7657471	0,014462
Cloud_Mesh_Distance_BA_R0_R1	16,26057625	-81,95942688	-942,5975342	0,009886
Cloud_Mesh_Distance_BA_R0_R1	23,15789223	-82,00494385	-942,9250488	0,119832
Cloud_Mesh_Distance_BA_R0_R1	16,08996582	-81,78289795	-942,8557129	0,018146
Cloud_Mesh_Distance_BA_R0_R1	23,12360382	-81,77583313	-942,8535767	0,050469
Cloud_Mesh_Distance_BA_R0_R1	16,0618763	-81,67222595	-942,7219238	0,00971
Cloud_Mesh_Distance_BA_R0_R1	23,0279808	-81,39022827	-943,1121826	-0,045837
Cloud_Mesh_Distance_BA_R0_R1	23,03229141	-81,57382202	-943,1907959	-0,006467
Cloud_Mesh_Distance_BA_R0_R1	23,07262611	-81,55270386	-942,987854	-0,007549
Cloud_Mesh_Distance_BA_R0_R1	15,81906128	-81,28915405	-942,8171387	-0,000293
Cloud_Mesh_Distance_BA_R0_R1	15,8438406	-81,21710205	-942,6217041	0,010486
Cloud_Mesh_Distance_BA_R0_R1	15,74380302	-81,11064148	-942,7507324	0,00091
Cloud_Mesh_Distance_BA_R0_R1	22,98317146	-81,4307251	-943,3010254	-0,041707
Cloud_Mesh_Distance_BA_R0_R1	15,64741135	-81,01504517	-942,8931274	-0,015023
Cloud_Mesh_Distance_BA_R0_R1	15,50471497	-80,76046753	-942,9532471	-0,036267
Cloud_Mesh_Distance_BA_R0_R1	15,55663872	-80,72944641	-942,7680054	-0,0208
Cloud_Mesh_Distance_BA_R0_R1	15,47294903	-80,61663818	-942,8703613	-0,033398
Cloud_Mesh_Distance_BA_R0_R1	15,58380508	-80,86767578	-942,8632202	-0,02443
Cloud_Mesh_Distance_BA_R0_R1	23,01523781	-80,7509613	-942,9902954	-0,140126
Cloud_Mesh_Distance_BA_R0_R1	22,91824532	-80,85855103	-943,3114014	-0,131891
Cloud_Mesh_Distance_BA_R0_R1	22,99197769	-80,87432861	-943,0947876	-0,128854
Cloud_Mesh_Distance_BA_R0_R1	15,39951801	-80,42407227	-942,8868408	-0,056078
Cloud_Mesh_Distance_BA_R0_R1	22,95537758	-80,6665802	-943,1593628	-0,144311
Cloud_Mesh_Distance_BA_R0_R1	15,29403877	-80,20906067	-943,0122681	-0,084765
Cloud_Mesh_Distance_BA_R0_R1	22,89753914	-80,52082825	-943,27771	-0,149326
Cloud_Mesh_Distance_BA_R0_R1	22,85884857	-80,31987	-943,30896	-0,156593
Cloud_Mesh_Distance_BA_R0_R1	15,31394196	-80,05133057	-942,8665772	-0,078654
Cloud_Mesh_Distance_BA_R0_R1	15,24745846	-79,9732666	-943,005188	-0,09701

Cloud_Mesh_Distance_BA_R0_R1	15,37626553	-80,19277954	-942,7865601	-0,06435
Cloud_Mesh_Distance_BA_R0_R1	22,8460598	-80,07092285	-943,2266846	-0,143184
Cloud_Mesh_Distance_BA_R0_R1	22,92933846	-80,24252319	-943,0932617	-0,146033
Cloud_Mesh_Distance_BA_R0_R1	15,3043251	-79,66720581	-942,7783203	-0,088598
Cloud_Mesh_Distance_BA_R0_R1	15,30278206	-79,8745575	-942,8265381	-0,086584
Cloud_Mesh_Distance_BA_R0_R1	15,37254429	-79,99609375	-942,7086182	-0,070775
Cloud_Mesh_Distance_BA_R0_R1	15,23941708	-79,53511047	-942,9158936	-0,112027
Cloud_Mesh_Distance_BA_R0_R1	15,23334026	-79,75335693	-942,9644775	-0,105778
Cloud_Mesh_Distance_BA_R0_R1	22,76553535	-79,84436035	-943,2728882	-0,128359
Cloud_Mesh_Distance_BA_R0_R1	22,68219566	-79,63226318	-943,2893677	-0,112624
Cloud_Mesh_Distance_BA_R0_R1	15,17113495	-79,6109314	-943,1065674	-0,126232
Cloud_Mesh_Distance_BA_R0_R1	15,25294971	-79,31887817	-942,887207	-0,113926
Cloud_Mesh_Distance_BA_R0_R1	22,70917892	-79,5128479	-943,1015015	-0,092249
Cloud_Mesh_Distance_BA_R0_R1	22,56477928	-79,25941467	-943,1635132	-0,063912
Cloud_Mesh_Distance_BA_R0_R1	15,26517773	-79,13296509	-942,8880615	-0,119349
Cloud_Mesh_Distance_BA_R0_R1	15,23443222	-79,0123291	-942,9951782	-0,125521
Cloud_Mesh_Distance_BA_R0_R1	15,2400465	-78,80299377	-943,0671997	-0,13015
Cloud_Mesh_Distance_BA_R0_R1	22,40563011	-78,92576599	-943,1066284	-0,01053
Cloud_Mesh_Distance_BA_R0_R1	22,41584015	-79,02444458	-943,2177124	-0,033578
Cloud_Mesh_Distance_BA_R0_R1	15,34414768	-78,45620728	-943,0091553	-0,123165
Cloud_Mesh_Distance_BA_R0_R1	22,14391518	-78,55754089	-943,1826782	0,02682
Cloud_Mesh_Distance_BA_R0_R1	22,01667404	-78,44573975	-943,3026733	0,037119
Cloud_Mesh_Distance_BA_R0_R1	22,06058311	-78,39131165	-943,1016235	0,043197
Cloud_Mesh_Distance_BA_R0_R1	15,54615402	-77,89697266	-942,9879761	-0,100698
Cloud_Mesh_Distance_BA_R0_R1	15,5697794	-77,9866333	-942,8633423	-0,099731
Cloud_Mesh_Distance_BA_R0_R1	21,71562195	-78,01148987	-943,2817383	0,078512
Cloud_Mesh_Distance_BA_R0_R1	21,95043182	-78,28094482	-943,1815186	0,052933
Cloud_Mesh_Distance_BA_R0_R1	15,46506119	-78,02287292	-943,0559082	-0,108833
Cloud_Mesh_Distance_BA_R0_R1	15,52646828	-77,80584717	-943,1167603	-0,090286
Cloud_Mesh_Distance_BA_R0_R1	21,6518116	-77,87968445	-943,190979	0,085538
Cloud_Mesh_Distance_BA_R0_R1	21,71702385	-78,09216309	-943,4178467	0,06927
Cloud_Mesh_Distance_BA_R0_R1	15,62383842	-77,60620117	-943,1129761	-0,08219
Cloud_Mesh_Distance_BA_R0_R1	21,61363983	-77,93760681	-943,4050293	0,084887
Cloud_Mesh_Distance_BA_R0_R1	21,52534294	-77,79806519	-943,3754273	0,095705
Cloud_Mesh_Distance_BA_R0_R1	15,8314085	-77,41688538	-942,9102173	-0,067338
Cloud_Mesh_Distance_BA_R0_R1	15,7215929	-77,57417297	-942,9451904	-0,077179
Cloud_Mesh_Distance_BA_R0_R1	15,72872925	-77,44760132	-943,078064	-0,071634
Cloud_Mesh_Distance_BA_R0_R1	21,2500267	-77,43145752	-943,3231812	0,111661
Cloud_Mesh_Distance_BA_R0_R1	21,16939735	-77,31594849	-943,2283936	0,101363
Cloud_Mesh_Distance_BA_R0_R1	15,97635365	-77,21377563	-942,8930664	-0,047797
Cloud_Mesh_Distance_BA_R0_R1	15,93114853	-77,04241943	-943,2711182	-0,054257
Cloud_Mesh_Distance_BA_R0_R1	15,96500587	-77,10501099	-943,0759277	-0,049961
Cloud_Mesh_Distance_BA_R0_R1	21,05535316	-77,23707581	-943,3497925	0,097391
Cloud_Mesh_Distance_BA_R0_R1	16,10558891	-76,99642944	-942,9736328	-0,036694
Cloud_Mesh_Distance_BA_R0_R1	20,7648716	-76,93443298	-943,1057739	0,059287
Cloud_Mesh_Distance_BA_R0_R1	20,76327133	-76,97140503	-943,3321533	0,063193
Cloud_Mesh_Distance_BA_R0_R1	20,87242508	-77,02902222	-943,1628418	0,068727
Cloud_Mesh_Distance_BA_R0_R1	16,77646065	-76,44638062	-943,078064	-0,025167
Cloud_Mesh_Distance_BA_R0_R1	16,67036247	-76,4657135	-943,1962891	-0,035221
Cloud_Mesh_Distance_BA_R0_R1	20,03351402	-76,48800659	-943,4293823	-0,012226

Cloud_Mesh_Distance_BA_R0_R1	20,33013725	-76,63952637	-943,1737061	0,0039
Cloud_Mesh_Distance_BA_R0_R1	20,5110569	-76,75587463	-943,163147	0,032244
Cloud_Mesh_Distance_BA_R0_R1	16,80527496	-76,39685059	-943,2340088	-0,044387
Cloud_Mesh_Distance_BA_R0_R1	16,98010254	-76,32466125	-943,2763672	-0,066501
Cloud_Mesh_Distance_BA_R0_R1	17,37201309	-76,2559967	-943,1119995	-0,081196
Cloud_Mesh_Distance_BA_R0_R1	19,52384758	-76,28631592	-943,2625122	-0,061243
Cloud_Mesh_Distance_BA_R0_R1	19,70102882	-76,33981323	-943,1610718	-0,044026
Cloud_Mesh_Distance_BA_R0_R1	19,85204124	-76,39642334	-943,3345947	-0,030249
Cloud_Mesh_Distance_BA_R0_R1	17,41071892	-76,21923828	-943,2793579	-0,095238
Cloud_Mesh_Distance_BA_R0_R1	17,53227615	-76,22277832	-943,1500855	-0,098413
Cloud_Mesh_Distance_BA_R0_R1	17,77024841	-76,17687988	-943,298645	-0,128738
Cloud_Mesh_Distance_BA_R0_R1	17,70969582	-76,2074585	-943,1253052	-0,115544
Cloud_Mesh_Distance_BA_R0_R1	18,13964081	-76,16360474	-943,3273926	-0,151897
Cloud_Mesh_Distance_BA_R0_R1	18,11921501	-76,18551636	-943,1478882	-0,145881
Cloud_Mesh_Distance_BA_R0_R1	18,33197212	-76,16377258	-943,3491211	-0,158408
Cloud_Mesh_Distance_BA_R0_R1	18,4999733	-76,18482971	-943,1911621	-0,152482
Cloud_Mesh_Distance_BA_R0_R1	18,65877914	-76,18756104	-943,2193604	-0,148108
Cloud_Mesh_Distance_BA_R0_R1	18,52344513	-76,16828918	-943,3634644	-0,153491
Cloud_Mesh_Distance_BA_R0_R1	19,11735153	-76,22770691	-943,1340332	-0,113955
Cloud_Mesh_Distance_BA_R0_R1	19,33046341	-76,25041199	-943,1967163	-0,081163
Cloud_Mesh_Distance_BA_R0_R1	17,62022209	-83,76412964	-942,6517334	0,042577
Cloud_Mesh_Distance_BA_R0_R1	17,34617615	-83,44137573	-942,5010986	-0,003265
Cloud_Mesh_Distance_BA_R0_R1	17,32787514	-83,36520386	-942,2636108	-0,017186
Cloud_Mesh_Distance_BA_R0_R1	17,47300529	-83,59638977	-942,5761108	0,018945
Cloud_Mesh_Distance_BA_R0_R1	17,45910645	-83,53689575	-942,3659668	0,000567
Cloud_Mesh_Distance_BA_R0_R1	16,97666359	-83,02853394	-942,6317139	-0,018086
Cloud_Mesh_Distance_BA_R0_R1	16,86985779	-82,917099	-942,7268066	-0,012064
Cloud_Mesh_Distance_BA_R0_R1	16,88215065	-82,87748718	-942,5296631	-0,02783
Cloud_Mesh_Distance_BA_R0_R1	16,77370834	-82,76559448	-942,6497803	-0,017961
Cloud_Mesh_Distance_BA_R0_R1	16,66137314	-82,50593567	-942,3449097	-0,026307
Cloud_Mesh_Distance_BA_R0_R1	16,58521461	-82,45303345	-942,5233154	-0,01803
Cloud_Mesh_Distance_BA_R0_R1	16,67271996	-82,59953308	-942,5744629	-0,02024
Cloud_Mesh_Distance_BA_R0_R1	23,30215645	-82,47235107	-942,90979	0,241719
Cloud_Mesh_Distance_BA_R0_R1	23,34783554	-82,5123291	-942,7068481	0,279482
Cloud_Mesh_Distance_BA_R0_R1	16,42142105	-82,18231201	-942,4900513	-0,004339
Cloud_Mesh_Distance_BA_R0_R1	16,40722847	-82,23300171	-942,6724854	0,003848
Cloud_Mesh_Distance_BA_R0_R1	23,26043892	-82,3125	-942,7946777	0,226647
Cloud_Mesh_Distance_BA_R0_R1	23,19935226	-82,1865387	-942,9628906	0,171503
Cloud_Mesh_Distance_BA_R0_R1	16,33441734	-82,09780884	-942,6309204	0,009861
Cloud_Mesh_Distance_BA_R0_R1	23,16941643	-81,96618652	-942,7478638	0,112705
Cloud_Mesh_Distance_BA_R0_R1	16,16635704	-81,84091187	-942,6930542	0,014237
Cloud_Mesh_Distance_BA_R0_R1	15,97813129	-81,53883362	-942,7522583	0,008042
Cloud_Mesh_Distance_BA_R0_R1	16,02809715	-81,52261353	-942,5542603	0,022497
Cloud_Mesh_Distance_BA_R0_R1	15,78287411	-81,0418396	-942,5512085	0,013471
Cloud_Mesh_Distance_BA_R0_R1	15,91617298	-81,39056396	-942,6824341	0,009976
Cloud_Mesh_Distance_BA_R0_R1	15,8735714	-81,15310669	-942,4560547	0,022901
Cloud_Mesh_Distance_BA_R0_R1	15,94282055	-81,31491089	-942,484314	0,020732
Cloud_Mesh_Distance_BA_R0_R1	23,06839371	-81,35635376	-942,9120483	-0,055204
Cloud_Mesh_Distance_BA_R0_R1	23,0274353	-81,19892883	-943,0460815	-0,082632
Cloud_Mesh_Distance_BA_R0_R1	23,0750103	-81,13850403	-942,8163452	-0,095972

Cloud_Mesh_Distance_BA_R0_R1	23,09836388	-81,3513031	-942,7323608	-0,055483
Cloud_Mesh_Distance_BA_R0_R1	15,50947571	-80,54011536	-942,7064209	-0,028346
Cloud_Mesh_Distance_BA_R0_R1	22,97789764	-81,05154419	-943,1835938	-0,108041
Cloud_Mesh_Distance_BA_R0_R1	23,02546501	-81,03086853	-943,0109253	-0,112248
Cloud_Mesh_Distance_BA_R0_R1	23,08354759	-80,94268799	-942,7445679	-0,124712
Cloud_Mesh_Distance_BA_R0_R1	23,00838089	-80,58218384	-942,9741211	-0,14544
Cloud_Mesh_Distance_BA_R0_R1	23,04470253	-80,90956116	-942,9121704	-0,125602
Cloud_Mesh_Distance_BA_R0_R1	15,4662962	-80,33331299	-942,6608276	-0,040696
Cloud_Mesh_Distance_BA_R0_R1	15,61153698	-80,35395813	-942,3765259	-0,001961
Cloud_Mesh_Distance_BA_R0_R1	15,53464031	-80,25265503	-942,477356	-0,01886
Cloud_Mesh_Distance_BA_R0_R1	15,44931602	-80,12728882	-942,5953369	-0,045551
Cloud_Mesh_Distance_BA_R0_R1	22,94468498	-80,44836426	-943,1297607	-0,15214
Cloud_Mesh_Distance_BA_R0_R1	22,99645805	-80,38513184	-942,9501953	-0,14705
Cloud_Mesh_Distance_BA_R0_R1	22,98447227	-80,20236206	-942,9025269	-0,139701
Cloud_Mesh_Distance_BA_R0_R1	22,92087173	-80,05310059	-943,0109863	-0,128631
Cloud_Mesh_Distance_BA_R0_R1	22,75418472	-79,67459106	-943,1555786	-0,107647
Cloud_Mesh_Distance_BA_R0_R1	22,82452774	-79,76620483	-943,0552368	-0,109696
Cloud_Mesh_Distance_BA_R0_R1	22,85628891	-79,91885376	-943,0985107	-0,126093
Cloud_Mesh_Distance_BA_R0_R1	15,31803894	-79,44873047	-942,7368164	-0,092565
Cloud_Mesh_Distance_BA_R0_R1	22,65525818	-79,35081482	-943,0484619	-0,065296
Cloud_Mesh_Distance_BA_R0_R1	22,49858475	-79,08869934	-943,1038208	-0,036874
Cloud_Mesh_Distance_BA_R0_R1	22,5096035	-78,98750305	-942,9199829	-0,012984
Cloud_Mesh_Distance_BA_R0_R1	15,31925392	-78,94325256	-942,8233643	-0,114513
Cloud_Mesh_Distance_BA_R0_R1	15,35478973	-78,64978027	-942,8719482	-0,11639
Cloud_Mesh_Distance_BA_R0_R1	15,4332695	-78,79931641	-942,6464844	-0,097747
Cloud_Mesh_Distance_BA_R0_R1	22,27565384	-78,74612427	-943,1599731	0,00812
Cloud_Mesh_Distance_BA_R0_R1	15,48408985	-78,56015015	-942,6573486	-0,10116
Cloud_Mesh_Distance_BA_R0_R1	15,43153191	-78,40882874	-942,8451538	-0,116452
Cloud_Mesh_Distance_BA_R0_R1	15,54372787	-78,33569336	-942,6695557	-0,098971
Cloud_Mesh_Distance_BA_R0_R1	22,16282845	-78,45803833	-942,9487915	0,047208
Cloud_Mesh_Distance_BA_R0_R1	15,60968399	-78,12614441	-942,6831055	-0,097833
Cloud_Mesh_Distance_BA_R0_R1	15,49747849	-78,18995667	-942,8551025	-0,108184
Cloud_Mesh_Distance_BA_R0_R1	22,03236771	-78,28475952	-942,9666748	0,061577
Cloud_Mesh_Distance_BA_R0_R1	21,85084534	-78,0954895	-943,0842896	0,073466
Cloud_Mesh_Distance_BA_R0_R1	15,63539124	-77,77282715	-942,9180908	-0,090585
Cloud_Mesh_Distance_BA_R0_R1	21,73892975	-77,9083252	-942,9868774	0,086935
Cloud_Mesh_Distance_BA_R0_R1	15,80570698	-77,63421631	-942,7294922	-0,074728
Cloud_Mesh_Distance_BA_R0_R1	21,46951103	-77,60914612	-943,0924683	0,104444
Cloud_Mesh_Distance_BA_R0_R1	21,38812828	-77,49307251	-943,0136719	0,101843
Cloud_Mesh_Distance_BA_R0_R1	21,60211182	-77,74945068	-943,0251465	0,09861
Cloud_Mesh_Distance_BA_R0_R1	21,28085327	-77,40341187	-943,1207275	0,102015
Cloud_Mesh_Distance_BA_R0_R1	21,36765289	-77,52676392	-943,210083	0,10565
Cloud_Mesh_Distance_BA_R0_R1	20,94270134	-77,11627197	-943,2921143	0,088127
Cloud_Mesh_Distance_BA_R0_R1	21,07896233	-77,20767212	-943,1462402	0,091878
Cloud_Mesh_Distance_BA_R0_R1	21,19245148	-77,29244995	-943,0305176	0,095022
Cloud_Mesh_Distance_BA_R0_R1	16,28442383	-76,91343689	-942,7890625	-0,011143
Cloud_Mesh_Distance_BA_R0_R1	16,26485252	-76,81156921	-943,0076294	-0,018361
Cloud_Mesh_Distance_BA_R0_R1	16,44371223	-76,79541016	-942,7365723	0,007324
Cloud_Mesh_Distance_BA_R0_R1	16,4303627	-76,6574707	-943,045166	-0,010363
Cloud_Mesh_Distance_BA_R0_R1	20,64760971	-76,85939026	-943,2138672	0,05104

Cloud_Mesh_Distance_BA_R0_R1	16,61834145	-76,53460693	-943,0510864	-0,013605
Cloud_Mesh_Distance_BA_R0_R1	16,5871582	-76,65005493	-942,8063965	0,004664
Cloud_Mesh_Distance_BA_R0_R1	20,17278481	-76,54708862	-943,1080933	-0,010897
Cloud_Mesh_Distance_BA_R0_R1	20,41962624	-76,68812561	-943,0286865	0,012487
Cloud_Mesh_Distance_BA_R0_R1	16,81215668	-76,48902893	-942,888855	-0,003022
Cloud_Mesh_Distance_BA_R0_R1	16,95991516	-76,38162231	-943,0428467	-0,034545
Cloud_Mesh_Distance_BA_R0_R1	17,15010071	-76,36933899	-942,8517456	-0,025656
Cloud_Mesh_Distance_BA_R0_R1	17,17120171	-76,30633545	-943,0845947	-0,052638
Cloud_Mesh_Distance_BA_R0_R1	17,37004852	-76,29916382	-942,9074707	-0,05448
Cloud_Mesh_Distance_BA_R0_R1	19,00733185	-76,25798035	-942,8029785	-0,11349
Cloud_Mesh_Distance_BA_R0_R1	19,17444611	-76,26022339	-942,8953857	-0,104797
Cloud_Mesh_Distance_BA_R0_R1	19,83633423	-76,3971405	-942,9859619	-0,038233
Cloud_Mesh_Distance_BA_R0_R1	20,03494644	-76,47799683	-943,2337036	-0,01991
Cloud_Mesh_Distance_BA_R0_R1	19,89300537	-76,41220093	-943,157898	-0,030735
Cloud_Mesh_Distance_BA_R0_R1	17,91750145	-76,19352722	-943,12677	-0,131784
Cloud_Mesh_Distance_BA_R0_R1	18,31162643	-76,1834259	-943,1686401	-0,152725
Cloud_Mesh_Distance_BA_R0_R1	18,48340797	-76,20602417	-943,0146484	-0,149194
Cloud_Mesh_Distance_BA_R0_R1	18,67479515	-76,20695496	-943,0543213	-0,143293
Cloud_Mesh_Distance_BA_R0_R1	18,84434509	-76,23068237	-942,9272461	-0,131308
Cloud_Mesh_Distance_BA_R0_R1	19,01150513	-76,23422241	-942,9845581	-0,119399
Cloud_Mesh_Distance_BA_R0_R1	18,8575573	-76,20552063	-943,1459961	-0,137418
Cloud_Mesh_Distance_BA_R0_R1	19,34811783	-76,27389526	-942,9682617	-0,088622
Cloud_Mesh_Distance_BA_R0_R1	19,65575027	-76,33891296	-942,970398	-0,054841
Cloud_Mesh_Distance_BA_R0_R1	17,81205177	-83,89984131	-942,3779297	0,052207
Cloud_Mesh_Distance_BA_R0_R1	17,80048943	-83,84378052	-942,180481	0,035854
Cloud_Mesh_Distance_BA_R0_R1	17,69874573	-83,80969238	-942,475769	0,041266
Cloud_Mesh_Distance_BA_R0_R1	17,68941307	-83,76394653	-942,3039551	0,026838
Cloud_Mesh_Distance_BA_R0_R1	17,58366776	-83,68453979	-942,4353027	0,023676
Cloud_Mesh_Distance_BA_R0_R1	17,11032295	-83,17790222	-942,5619507	-0,017488
Cloud_Mesh_Distance_BA_R0_R1	17,23333549	-83,29951477	-942,4505005	-0,017664
Cloud_Mesh_Distance_BA_R0_R1	17,00418854	-83,00747681	-942,4187622	-0,035412
Cloud_Mesh_Distance_BA_R0_R1	16,89513588	-82,83258057	-942,3137817	-0,041107
Cloud_Mesh_Distance_BA_R0_R1	17,15218353	-83,1607666	-942,2868652	-0,033739
Cloud_Mesh_Distance_BA_R0_R1	16,77814102	-82,70724487	-942,4385986	-0,034427
Cloud_Mesh_Distance_BA_R0_R1	16,52902412	-82,31648254	-942,3897705	-0,013905
Cloud_Mesh_Distance_BA_R0_R1	16,49365997	-82,34130859	-942,6015625	-0,002948
Cloud_Mesh_Distance_BA_R0_R1	23,20416069	-82,12063599	-942,7741089	0,166321
Cloud_Mesh_Distance_BA_R0_R1	16,43855095	-82,08570862	-942,2039185	0,006771
Cloud_Mesh_Distance_BA_R0_R1	16,31231499	-81,90087891	-942,2874756	0,015883
Cloud_Mesh_Distance_BA_R0_R1	16,33107758	-82,01016235	-942,4432373	0,003979
Cloud_Mesh_Distance_BA_R0_R1	16,23888397	-81,73382568	-942,2375488	0,029219
Cloud_Mesh_Distance_BA_R0_R1	16,12267876	-81,69766235	-942,5579834	0,015356
Cloud_Mesh_Distance_BA_R0_R1	16,22331619	-81,82545471	-942,4539795	0,014329
Cloud_Mesh_Distance_BA_R0_R1	23,13656807	-81,67843628	-942,595398	0,026494
Cloud_Mesh_Distance_BA_R0_R1	23,10429573	-81,52844238	-942,7763062	-0,01305
Cloud_Mesh_Distance_BA_R0_R1	23,12279892	-81,47436523	-942,5759277	-0,035138
Cloud_Mesh_Distance_BA_R0_R1	15,6644659	-80,90202332	-942,68573	-0,00579
Cloud_Mesh_Distance_BA_R0_R1	15,74057961	-80,85571289	-942,4545288	0,011314
Cloud_Mesh_Distance_BA_R0_R1	15,84738731	-81,00389099	-942,3555298	0,024547
Cloud_Mesh_Distance_BA_R0_R1	15,72352219	-80,68925476	-942,3549805	0,01567

Cloud_Mesh_Distance_BA_R0_R1	15,62679482	-80,67222595	-942,5462647	-0,004272
Cloud_Mesh_Distance_BA_R0_R1	23,06342888	-80,74438477	-942,8057251	-0,140912
Cloud_Mesh_Distance_BA_R0_R1	15,56436443	-80,44747925	-942,5194702	-0,015764
Cloud_Mesh_Distance_BA_R0_R1	15,66390419	-80,51803589	-942,3623047	0,007637
Cloud_Mesh_Distance_BA_R0_R1	23,10036469	-80,65925598	-942,6152954	-0,149864
Cloud_Mesh_Distance_BA_R0_R1	23,05327415	-80,52639771	-942,7941284	-0,14919
Cloud_Mesh_Distance_BA_R0_R1	23,0393734	-80,34094238	-942,7765503	-0,141942
Cloud_Mesh_Distance_BA_R0_R1	15,52954865	-80,05435181	-942,4155273	-0,023666
Cloud_Mesh_Distance_BA_R0_R1	23,02611923	-80,19514465	-942,7440796	-0,136397
Cloud_Mesh_Distance_BA_R0_R1	23,07179451	-80,30203247	-942,6106567	-0,142038
Cloud_Mesh_Distance_BA_R0_R1	23,0477562	-80,1395874	-942,6029663	-0,132059
Cloud_Mesh_Distance_BA_R0_R1	22,96199989	-79,87986755	-942,7168579	-0,105149
Cloud_Mesh_Distance_BA_R0_R1	22,98335648	-80,03727722	-942,789978	-0,124252
Cloud_Mesh_Distance_BA_R0_R1	15,44824696	-79,92419434	-942,5338135	-0,049584
Cloud_Mesh_Distance_BA_R0_R1	15,45605087	-79,71936035	-942,4835205	-0,051518
Cloud_Mesh_Distance_BA_R0_R1	15,3738699	-79,79620361	-942,6528931	-0,072788
Cloud_Mesh_Distance_BA_R0_R1	15,38501549	-79,58889771	-942,6048584	-0,075042
Cloud_Mesh_Distance_BA_R0_R1	22,85756302	-79,7010498	-942,8876953	-0,091874
Cloud_Mesh_Distance_BA_R0_R1	22,8472538	-79,55834961	-942,7347412	-0,069826
Cloud_Mesh_Distance_BA_R0_R1	22,90658569	-79,86712646	-942,9034424	-0,113533
Cloud_Mesh_Distance_BA_R0_R1	15,47060776	-79,51972961	-942,4469605	-0,052731
Cloud_Mesh_Distance_BA_R0_R1	22,79048157	-79,60064697	-942,977417	-0,091537
Cloud_Mesh_Distance_BA_R0_R1	15,34295559	-79,19961548	-942,7089233	-0,096252
Cloud_Mesh_Distance_BA_R0_R1	15,40837097	-79,37913513	-942,5629883	-0,074642
Cloud_Mesh_Distance_BA_R0_R1	15,48954105	-79,35107422	-942,421814	-0,054065
Cloud_Mesh_Distance_BA_R0_R1	22,74131393	-79,4052124	-942,8731079	-0,063452
Cloud_Mesh_Distance_BA_R0_R1	15,40827465	-79,00793457	-942,6253662	-0,093166
Cloud_Mesh_Distance_BA_R0_R1	22,62147903	-79,19018555	-942,9173584	-0,037594
Cloud_Mesh_Distance_BA_R0_R1	22,48418045	-78,86297607	-942,7670898	0,008781
Cloud_Mesh_Distance_BA_R0_R1	22,39561081	-78,81158447	-942,9493408	0,009451
Cloud_Mesh_Distance_BA_R0_R1	22,2702179	-78,63970947	-942,9952393	0,025842
Cloud_Mesh_Distance_BA_R0_R1	15,7268858	-78,0667572	-942,5189819	-0,080226
Cloud_Mesh_Distance_BA_R0_R1	15,69228077	-77,90579224	-942,6962891	-0,086872
Cloud_Mesh_Distance_BA_R0_R1	21,84687424	-78,01416016	-942,9002075	0,082237
Cloud_Mesh_Distance_BA_R0_R1	21,7716217	-77,88825989	-942,802002	0,085612
Cloud_Mesh_Distance_BA_R0_R1	21,98575211	-78,15975952	-942,8115845	0,074161
Cloud_Mesh_Distance_BA_R0_R1	21,59736061	-77,68252563	-942,8082886	0,093501
Cloud_Mesh_Distance_BA_R0_R1	21,68024445	-77,79862976	-942,8759766	0,091186
Cloud_Mesh_Distance_BA_R0_R1	15,96139717	-77,38729858	-942,7106323	-0,051872
Cloud_Mesh_Distance_BA_R0_R1	21,50493431	-77,56109619	-942,7137451	0,096558
Cloud_Mesh_Distance_BA_R0_R1	21,49586487	-77,59014893	-942,9124146	0,097549
Cloud_Mesh_Distance_BA_R0_R1	16,04836464	-77,24462891	-942,7268066	-0,042941
Cloud_Mesh_Distance_BA_R0_R1	21,29990768	-77,37962341	-942,921814	0,096964
Cloud_Mesh_Distance_BA_R0_R1	21,31052971	-77,36180115	-942,7254639	0,089664
Cloud_Mesh_Distance_BA_R0_R1	21,20547676	-77,27325439	-942,8314209	0,091138
Cloud_Mesh_Distance_BA_R0_R1	21,40386963	-77,46954346	-942,8167114	0,096163
Cloud_Mesh_Distance_BA_R0_R1	21,09692383	-77,18807983	-942,9431152	0,08681
Cloud_Mesh_Distance_BA_R0_R1	21,10240364	-77,17089844	-942,7418823	0,077377
Cloud_Mesh_Distance_BA_R0_R1	20,98376274	-77,10621643	-943,0615845	0,078402
Cloud_Mesh_Distance_BA_R0_R1	16,13278389	-77,11170959	-942,753479	-0,025703

Cloud_Mesh_Distance_BA_R0_R1	20,7628212	-76,9125061	-942,8996582	0,051112
Cloud_Mesh_Distance_BA_R0_R1	20,88000488	-77,00909424	-942,9787598	0,068098
Cloud_Mesh_Distance_BA_R0_R1	16,39533234	-76,75854492	-942,8792725	-0,000365
Cloud_Mesh_Distance_BA_R0_R1	20,4131031	-76,6819458	-942,8676148	0,010985
Cloud_Mesh_Distance_BA_R0_R1	20,53297615	-76,75665283	-942,9329224	0,024491
Cloud_Mesh_Distance_BA_R0_R1	20,64916039	-76,84011841	-943,0227051	0,041106
Cloud_Mesh_Distance_BA_R0_R1	20,6459465	-76,8276825	-942,8389893	0,034143
Cloud_Mesh_Distance_BA_R0_R1	16,98156548	-76,42288208	-942,8666382	-0,015189
Cloud_Mesh_Distance_BA_R0_R1	16,7902298	-76,57527161	-942,697876	0,012804
Cloud_Mesh_Distance_BA_R0_R1	20,2887249	-76,61830139	-942,7227783	-0,003085
Cloud_Mesh_Distance_BA_R0_R1	20,2947979	-76,6137085	-942,9694214	0,001327
Cloud_Mesh_Distance_BA_R0_R1	17,5728035	-76,24807739	-942,9827881	-0,084412
Cloud_Mesh_Distance_BA_R0_R1	17,60469246	-76,29374695	-942,7443848	-0,055007
Cloud_Mesh_Distance_BA_R0_R1	18,0100956	-76,28712463	-942,5758667	-0,065424
Cloud_Mesh_Distance_BA_R0_R1	18,0455761	-76,24372864	-942,7897339	-0,105022
Cloud_Mesh_Distance_BA_R0_R1	17,82324409	-76,23419189	-942,9156494	-0,103626
Cloud_Mesh_Distance_BA_R0_R1	17,86119652	-76,27429199	-942,6934815	-0,070893
Cloud_Mesh_Distance_BA_R0_R1	18,25417519	-76,23303223	-942,8190918	-0,125074
Cloud_Mesh_Distance_BA_R0_R1	18,65426636	-76,23078918	-942,8676148	-0,132866
Cloud_Mesh_Distance_BA_R0_R1	18,45560074	-76,23060608	-942,8378906	-0,132343
Cloud_Mesh_Distance_BA_R0_R1	18,62304878	-76,25737	-942,6837158	-0,116316
Cloud_Mesh_Distance_BA_R0_R1	18,82361031	-76,25814819	-942,7254028	-0,116534
Cloud_Mesh_Distance_BA_R0_R1	19,17569733	-76,29019165	-942,6885986	-0,095379
Cloud_Mesh_Distance_BA_R0_R1	19,34731293	-76,30232239	-942,7545166	-0,08441
Cloud_Mesh_Distance_BA_R0_R1	19,95442009	-76,45605469	-942,8265381	-0,033217
Cloud_Mesh_Distance_BA_R0_R1	20,01483345	-76,46899414	-943,0337524	-0,022935
Cloud_Mesh_Distance_BA_R0_R1	18,08006859	-76,21240234	-942,9680176	-0,128934
Cloud_Mesh_Distance_BA_R0_R1	18,28709221	-76,20657349	-942,9920044	-0,140879
Cloud_Mesh_Distance_BA_R0_R1	19,50265312	-76,29267883	-943,0647583	-0,069248
Cloud_Mesh_Distance_BA_R0_R1	17,93292618	-83,9800415	-942,2335815	0,063321
Cloud_Mesh_Distance_BA_R0_R1	17,90299606	-83,90951538	-942,0626831	0,042815
Cloud_Mesh_Distance_BA_R0_R1	17,67931557	-83,66430664	-941,9495239	0,005306
Cloud_Mesh_Distance_BA_R0_R1	17,68119621	-83,71133423	-942,1182251	0,015425
Cloud_Mesh_Distance_BA_R0_R1	17,46302032	-83,49673462	-942,1766968	-0,010235
Cloud_Mesh_Distance_BA_R0_R1	17,57128716	-83,6282959	-942,2398682	0,005971
Cloud_Mesh_Distance_BA_R0_R1	17,01699448	-82,95983887	-942,1850586	-0,041403
Cloud_Mesh_Distance_BA_R0_R1	17,25268173	-83,23170471	-942,0906982	-0,034053
Cloud_Mesh_Distance_BA_R0_R1	17,02025032	-82,89619446	-941,9620361	-0,044389
Cloud_Mesh_Distance_BA_R0_R1	17,1359787	-83,0721283	-942,0389404	-0,039521
Cloud_Mesh_Distance_BA_R0_R1	16,70140266	-82,47958374	-942,1013184	-0,027905
Cloud_Mesh_Distance_BA_R0_R1	16,90726662	-82,78184509	-942,0933228	-0,041825
Cloud_Mesh_Distance_BA_R0_R1	16,79087448	-82,65454102	-942,2150879	-0,035793
Cloud_Mesh_Distance_BA_R0_R1	23,35175896	-82,47740173	-942,4813843	0,311659
Cloud_Mesh_Distance_BA_R0_R1	16,63633537	-82,32366943	-941,975769	-0,008551
Cloud_Mesh_Distance_BA_R0_R1	16,58355141	-82,31938171	-942,1737671	-0,01553
Cloud_Mesh_Distance_BA_R0_R1	23,29079437	-82,34051514	-942,552063	0,265817
Cloud_Mesh_Distance_BA_R0_R1	23,23896027	-82,20245361	-942,6289063	0,212291
Cloud_Mesh_Distance_BA_R0_R1	23,20093346	-82,05844116	-942,5961304	0,15203
Cloud_Mesh_Distance_BA_R0_R1	23,19082832	-82,00280762	-942,4036255	0,133723
Cloud_Mesh_Distance_BA_R0_R1	16,33331299	-81,84049988	-942,1096191	0,021705

Cloud_Mesh_Distance_BA_R0_R1	16,13737679	-81,62249756	-942,3768311	0,026244
Cloud_Mesh_Distance_BA_R0_R1	16,05015373	-81,4176178	-942,3276367	0,030747
Cloud_Mesh_Distance_BA_R0_R1	23,16397476	-81,88024902	-942,5960083	0,084236
Cloud_Mesh_Distance_BA_R0_R1	23,13378334	-81,4120636	-942,3849487	-0,059256
Cloud_Mesh_Distance_BA_R0_R1	15,96247673	-81,18696594	-942,2738037	0,035116
Cloud_Mesh_Distance_BA_R0_R1	16,04897499	-81,11959839	-942,0087891	0,057295
Cloud_Mesh_Distance_BA_R0_R1	15,92345715	-80,96636963	-942,1547852	0,041659
Cloud_Mesh_Distance_BA_R0_R1	23,11556435	-81,27455139	-942,5812988	-0,076948
Cloud_Mesh_Distance_BA_R0_R1	23,1124649	-81,07113647	-942,5878296	-0,118566
Cloud_Mesh_Distance_BA_R0_R1	23,13392448	-81,21054077	-942,390564	-0,101602
Cloud_Mesh_Distance_BA_R0_R1	23,13680077	-81,00340271	-942,3944092	-0,136541
Cloud_Mesh_Distance_BA_R0_R1	15,81611824	-80,81112671	-942,2561646	0,032258
Cloud_Mesh_Distance_BA_R0_R1	23,1100502	-80,8646698	-942,5916748	-0,138768
Cloud_Mesh_Distance_BA_R0_R1	23,08729553	-80,47119141	-942,6229858	-0,150383
Cloud_Mesh_Distance_BA_R0_R1	15,69586277	-80,35464478	-942,2229614	0,01381
Cloud_Mesh_Distance_BA_R0_R1	15,61405087	-80,19036865	-942,3070068	-0,005427
Cloud_Mesh_Distance_BA_R0_R1	23,11371803	-80,43182373	-942,4560547	-0,155731
Cloud_Mesh_Distance_BA_R0_R1	23,09719658	-80,26712036	-942,4424439	-0,143335
Cloud_Mesh_Distance_BA_R0_R1	15,53494263	-79,84609985	-942,3603516	-0,026882
Cloud_Mesh_Distance_BA_R0_R1	15,62277126	-79,98080444	-942,2357788	0,000461
Cloud_Mesh_Distance_BA_R0_R1	23,01733017	-79,97850037	-942,585083	-0,115977
Cloud_Mesh_Distance_BA_R0_R1	23,04073524	-79,93772888	-942,3960571	-0,112522
Cloud_Mesh_Distance_BA_R0_R1	23,07367516	-80,1038208	-942,4207153	-0,128344
Cloud_Mesh_Distance_BA_R0_R1	15,55457401	-79,44117737	-942,3085327	-0,035368
Cloud_Mesh_Distance_BA_R0_R1	15,54426861	-79,63833618	-942,3242188	-0,03004
Cloud_Mesh_Distance_BA_R0_R1	15,63537121	-79,54469299	-942,1787109	-0,009142
Cloud_Mesh_Distance_BA_R0_R1	22,97971344	-79,81903076	-942,5579834	-0,095226
Cloud_Mesh_Distance_BA_R0_R1	22,91498756	-79,73693848	-942,7269898	-0,086029
Cloud_Mesh_Distance_BA_R0_R1	15,46418381	-79,16722107	-942,4894409	-0,06953
Cloud_Mesh_Distance_BA_R0_R1	15,55872536	-79,24998474	-942,3195801	-0,042675
Cloud_Mesh_Distance_BA_R0_R1	15,64925003	-79,1456604	-942,1976318	-0,030964
Cloud_Mesh_Distance_BA_R0_R1	22,71542358	-79,24130249	-942,6931763	-0,030843
Cloud_Mesh_Distance_BA_R0_R1	22,87299538	-79,4864502	-942,5004273	-0,054205
Cloud_Mesh_Distance_BA_R0_R1	22,79949379	-79,39404297	-942,6498413	-0,047971
Cloud_Mesh_Distance_BA_R0_R1	15,59019375	-78,88293457	-942,3499756	-0,061478
Cloud_Mesh_Distance_BA_R0_R1	15,50129414	-78,93487549	-942,4797363	-0,076237
Cloud_Mesh_Distance_BA_R0_R1	15,56772518	-79,05477905	-942,3408203	-0,05244
Cloud_Mesh_Distance_BA_R0_R1	22,60778236	-79,04180908	-942,6986694	-0,007089
Cloud_Mesh_Distance_BA_R0_R1	22,54661751	-78,89291382	-942,5958252	0,016499
Cloud_Mesh_Distance_BA_R0_R1	15,70367527	-78,554245	-942,2919922	-0,060653
Cloud_Mesh_Distance_BA_R0_R1	15,60567951	-78,46490479	-942,4891357	-0,083933
Cloud_Mesh_Distance_BA_R0_R1	15,56507111	-78,70758057	-942,4501343	-0,076701
Cloud_Mesh_Distance_BA_R0_R1	15,65514946	-78,26377869	-942,5150757	-0,084957
Cloud_Mesh_Distance_BA_R0_R1	15,70265388	-78,37089539	-942,3786621	-0,070852
Cloud_Mesh_Distance_BA_R0_R1	15,79835415	-78,35838318	-942,2389526	-0,057213
Cloud_Mesh_Distance_BA_R0_R1	22,31480598	-78,56816101	-942,690979	0,046663
Cloud_Mesh_Distance_BA_R0_R1	22,2457695	-78,51530457	-942,8123169	0,04619
Cloud_Mesh_Distance_BA_R0_R1	22,36307526	-78,6925354	-942,8170166	0,027316
Cloud_Mesh_Distance_BA_R0_R1	22,43815994	-78,72561646	-942,6135864	0,037006
Cloud_Mesh_Distance_BA_R0_R1	15,76257229	-78,21133423	-942,371521	-0,069762

Cloud_Mesh_Distance_BA_R0_R1	22,22560692	-78,40887451	-942,595398	0,060333
Cloud_Mesh_Distance_BA_R0_R1	22,12781715	-78,33457947	-942,7736816	0,063637
Cloud_Mesh_Distance_BA_R0_R1	15,82768154	-78,04350281	-942,3728027	-0,067065
Cloud_Mesh_Distance_BA_R0_R1	15,84743404	-77,84060669	-942,4846802	-0,069162
Cloud_Mesh_Distance_BA_R0_R1	15,88945675	-78,15873718	-942,210083	-0,05364
Cloud_Mesh_Distance_BA_R0_R1	21,88702202	-77,95584106	-942,5020752	0,087413
Cloud_Mesh_Distance_BA_R0_R1	21,87747955	-77,99060059	-942,7074585	0,084418
Cloud_Mesh_Distance_BA_R0_R1	21,60412025	-77,65341187	-942,6105957	0,094965
Cloud_Mesh_Distance_BA_R0_R1	21,69176102	-77,76863098	-942,7035523	0,092127
Cloud_Mesh_Distance_BA_R0_R1	16,12890816	-77,277771	-942,5604858	-0,032747
Cloud_Mesh_Distance_BA_R0_R1	16,00040054	-77,55630493	-942,4818115	-0,05335
Cloud_Mesh_Distance_BA_R0_R1	16,31451416	-77,20957947	-942,3713379	-0,013008
Cloud_Mesh_Distance_BA_R0_R1	21,50941658	-77,54301453	-942,5214233	0,089317
Cloud_Mesh_Distance_BA_R0_R1	21,41166306	-77,45228577	-942,6245117	0,090137
Cloud_Mesh_Distance_BA_R0_R1	21,31975365	-77,35562134	-942,5266113	0,083474
Cloud_Mesh_Distance_BA_R0_R1	16,28118706	-77,06639099	-942,5808716	-0,013
Cloud_Mesh_Distance_BA_R0_R1	20,99182701	-77,08717346	-942,8583984	0,073446
Cloud_Mesh_Distance_BA_R0_R1	21,21120071	-77,2600708	-942,6318359	0,081716
Cloud_Mesh_Distance_BA_R0_R1	21,10081482	-77,16000366	-942,5360718	0,070878
Cloud_Mesh_Distance_BA_R0_R1	16,4410038	-77,02940369	-942,4098511	0,001988
Cloud_Mesh_Distance_BA_R0_R1	16,42050743	-76,90673828	-942,5963135	0,00329
Cloud_Mesh_Distance_BA_R0_R1	16,54151726	-76,8977356	-942,4506226	0,013528
Cloud_Mesh_Distance_BA_R0_R1	20,87214851	-76,98574829	-942,7705078	0,058896
Cloud_Mesh_Distance_BA_R0_R1	16,86306572	-76,68952942	-942,3950806	0,033827
Cloud_Mesh_Distance_BA_R0_R1	16,69504738	-76,81774902	-942,3809815	0,023024
Cloud_Mesh_Distance_BA_R0_R1	16,56291389	-76,7850647	-942,5898438	0,013401
Cloud_Mesh_Distance_BA_R0_R1	20,3978405	-76,69158936	-942,496521	0,008195
Cloud_Mesh_Distance_BA_R0_R1	20,71669579	-76,8719635	-942,6691284	0,03962
Cloud_Mesh_Distance_BA_R0_R1	20,5195179	-76,74702454	-942,7150879	0,020228
Cloud_Mesh_Distance_BA_R0_R1	16,72117805	-76,68310547	-942,5664063	0,020331
Cloud_Mesh_Distance_BA_R0_R1	16,87587929	-76,59561157	-942,5532837	0,022159
Cloud_Mesh_Distance_BA_R0_R1	17,29678345	-76,44213867	-942,4867554	0,012547
Cloud_Mesh_Distance_BA_R0_R1	17,05468178	-76,55102539	-942,4590454	0,025725
Cloud_Mesh_Distance_BA_R0_R1	16,98410988	-76,48083496	-942,6973877	0,005118
Cloud_Mesh_Distance_BA_R0_R1	20,13857079	-76,53408813	-942,8791504	-0,016737
Cloud_Mesh_Distance_BA_R0_R1	17,17485619	-76,42553711	-942,645813	0,000435
Cloud_Mesh_Distance_BA_R0_R1	17,35676003	-76,35578918	-942,6950684	-0,02127
Cloud_Mesh_Distance_BA_R0_R1	17,76311874	-76,32296753	-942,5309448	-0,033134
Cloud_Mesh_Distance_BA_R0_R1	18,21903801	-76,26219177	-942,6530151	-0,094723
Cloud_Mesh_Distance_BA_R0_R1	18,42061424	-76,25798035	-942,6629639	-0,105703
Cloud_Mesh_Distance_BA_R0_R1	18,78501892	-76,28715515	-942,5322876	-0,094216
Cloud_Mesh_Distance_BA_R0_R1	18,58436775	-76,28704834	-942,501648	-0,088679
Cloud_Mesh_Distance_BA_R0_R1	19,00057983	-76,29290771	-942,5718384	-0,094086
Cloud_Mesh_Distance_BA_R0_R1	19,33269501	-76,32917786	-942,5716553	-0,076115
Cloud_Mesh_Distance_BA_R0_R1	19,18263626	-76,31918335	-942,5148315	-0,081154
Cloud_Mesh_Distance_BA_R0_R1	19,54090309	-76,33163452	-942,7976074	-0,068116
Cloud_Mesh_Distance_BA_R0_R1	19,75324249	-76,38803101	-942,793335	-0,048863
Cloud_Mesh_Distance_BA_R0_R1	18,29751015	-84,12654114	-941,7121582	0,056284
Cloud_Mesh_Distance_BA_R0_R1	18,15353203	-84,07116699	-941,8760986	0,06186
Cloud_Mesh_Distance_BA_R0_R1	18,32102585	-84,21054077	-941,9072266	0,088714

Cloud_Mesh_Distance_BA_R0_R1	18,4696331	-84,26329041	-941,7575684	0,077339
Cloud_Mesh_Distance_BA_R0_R1	18,03757858	-84,02786255	-942,0587158	0,065968
Cloud_Mesh_Distance_BA_R0_R1	17,95835114	-83,89874268	-941,8534546	0,034943
Cloud_Mesh_Distance_BA_R0_R1	17,79731369	-83,79138184	-941,9909058	0,023627
Cloud_Mesh_Distance_BA_R0_R1	17,59003067	-83,53181458	-941,8104248	-0,012497
Cloud_Mesh_Distance_BA_R0_R1	17,56246948	-83,56977844	-942,0445557	-0,005396
Cloud_Mesh_Distance_BA_R0_R1	17,40482712	-83,37762451	-941,9741821	-0,02169
Cloud_Mesh_Distance_BA_R0_R1	17,25424767	-83,17601013	-941,8912964	-0,036864
Cloud_Mesh_Distance_BA_R0_R1	17,13378716	-83,00093079	-941,8193359	-0,042132
Cloud_Mesh_Distance_BA_R0_R1	17,0225029	-82,8276062	-941,7533569	-0,037326
Cloud_Mesh_Distance_BA_R0_R1	16,81300926	-82,61244202	-941,99823	-0,033585
Cloud_Mesh_Distance_BA_R0_R1	16,83662796	-82,56999207	-941,7942505	-0,028647
Cloud_Mesh_Distance_BA_R0_R1	16,91966057	-82,72883606	-941,8848877	-0,036379
Cloud_Mesh_Distance_BA_R0_R1	16,54683685	-82,1946106	-942,0299072	-0,001473
Cloud_Mesh_Distance_BA_R0_R1	16,58632278	-82,16860962	-941,8352051	0,001614
Cloud_Mesh_Distance_BA_R0_R1	16,73525238	-82,45315552	-941,8987427	-0,020324
Cloud_Mesh_Distance_BA_R0_R1	23,2386055	-82,18528748	-942,4416504	0,217094
Cloud_Mesh_Distance_BA_R0_R1	23,21508789	-82,14233398	-942,2173462	0,18871
Cloud_Mesh_Distance_BA_R0_R1	16,41849709	-81,9155426	-941,9552002	0,020611
Cloud_Mesh_Distance_BA_R0_R1	16,26452637	-81,67382813	-942,0667114	0,033306
Cloud_Mesh_Distance_BA_R0_R1	23,15836334	-81,81111145	-942,3933106	0,063125
Cloud_Mesh_Distance_BA_R0_R1	23,14037132	-81,6125946	-942,3855591	-0,005145
Cloud_Mesh_Distance_BA_R0_R1	23,13705635	-81,54968262	-942,1825562	-0,037471
Cloud_Mesh_Distance_BA_R0_R1	16,0753727	-81,32220459	-942,1434937	0,044028
Cloud_Mesh_Distance_BA_R0_R1	16,16658592	-81,52522278	-942,1466675	0,038825
Cloud_Mesh_Distance_BA_R0_R1	16,15883636	-81,34625244	-941,9597168	0,054853
Cloud_Mesh_Distance_BA_R0_R1	23,13956261	-81,34783936	-942,1920776	-0,085476
Cloud_Mesh_Distance_BA_R0_R1	16,07625198	-80,92878723	-941,8106079	0,06955
Cloud_Mesh_Distance_BA_R0_R1	15,9849062	-80,90432739	-941,9803467	0,059479
Cloud_Mesh_Distance_BA_R0_R1	23,15462494	-80,94471741	-942,1942749	-0,1577
Cloud_Mesh_Distance_BA_R0_R1	23,13816643	-80,77322388	-942,392395	-0,158637
Cloud_Mesh_Distance_BA_R0_R1	15,89612865	-80,76124573	-942,0632324	0,047776
Cloud_Mesh_Distance_BA_R0_R1	15,81364727	-80,33824158	-942,0134888	0,045201
Cloud_Mesh_Distance_BA_R0_R1	15,78239155	-80,57803345	-942,171875	0,033214
Cloud_Mesh_Distance_BA_R0_R1	23,12138748	-80,58337402	-942,4742432	-0,160298
Cloud_Mesh_Distance_BA_R0_R1	15,71361351	-80,14198303	-942,1220703	0,023113
Cloud_Mesh_Distance_BA_R0_R1	15,63074112	-79,75830078	-942,1924439	-0,004767
Cloud_Mesh_Distance_BA_R0_R1	15,73857307	-79,89440918	-942,0370483	0,024178
Cloud_Mesh_Distance_BA_R0_R1	15,72816277	-79,64698792	-942,0368652	0,017612
Cloud_Mesh_Distance_BA_R0_R1	15,82281303	-79,51715088	-941,9012451	0,030104
Cloud_Mesh_Distance_BA_R0_R1	15,82942963	-79,73022461	-941,887146	0,041427
Cloud_Mesh_Distance_BA_R0_R1	22,93058205	-79,65545654	-942,5361328	-0,075225
Cloud_Mesh_Distance_BA_R0_R1	15,64163971	-79,34301758	-942,1813355	-0,018757
Cloud_Mesh_Distance_BA_R0_R1	15,72806168	-79,43769836	-942,0447388	0,004833
Cloud_Mesh_Distance_BA_R0_R1	22,94630241	-79,59841919	-942,3289795	-0,059265
Cloud_Mesh_Distance_BA_R0_R1	15,73805714	-79,23443604	-942,0524902	-0,004557
Cloud_Mesh_Distance_BA_R0_R1	15,65908623	-78,95588684	-942,2235718	-0,039982
Cloud_Mesh_Distance_BA_R0_R1	15,7444334	-79,03552246	-942,0810547	-0,018362
Cloud_Mesh_Distance_BA_R0_R1	15,7469101	-78,8518219	-942,1235962	-0,031202
Cloud_Mesh_Distance_BA_R0_R1	22,79716492	-79,3114624	-942,4918823	-0,029192

Cloud_Mesh_Distance_BA_R0_R1	22,70518875	-79,13757324	-942,5112305	-0,009189
Cloud_Mesh_Distance_BA_R0_R1	22,78620911	-79,22631836	-942,3209229	-0,011505
Cloud_Mesh_Distance_BA_R0_R1	15,66847515	-78,77165222	-942,2643433	-0,052936
Cloud_Mesh_Distance_BA_R0_R1	22,62593269	-78,9813385	-942,4716187	0,011245
Cloud_Mesh_Distance_BA_R0_R1	22,63594246	-78,94174194	-942,272522	0,024168
Cloud_Mesh_Distance_BA_R0_R1	15,75959015	-78,68884277	-942,1572876	-0,041678
Cloud_Mesh_Distance_BA_R0_R1	22,54263306	-78,82676697	-942,4169312	0,030092
Cloud_Mesh_Distance_BA_R0_R1	15,84874153	-78,52819824	-942,090332	-0,035935
Cloud_Mesh_Distance_BA_R0_R1	15,90308952	-78,32971191	-942,1011963	-0,041531
Cloud_Mesh_Distance_BA_R0_R1	22,34800148	-78,55728149	-942,5094605	0,05432
Cloud_Mesh_Distance_BA_R0_R1	22,27102089	-78,42483521	-942,4136353	0,068193
Cloud_Mesh_Distance_BA_R0_R1	15,9861536	-78,21453857	-942,0437622	-0,036001
Cloud_Mesh_Distance_BA_R0_R1	15,93944359	-77,93649292	-942,2752075	-0,055132
Cloud_Mesh_Distance_BA_R0_R1	21,98157883	-78,08839417	-942,5900879	0,083127
Cloud_Mesh_Distance_BA_R0_R1	22,06588745	-78,16757202	-942,4724731	0,082524
Cloud_Mesh_Distance_BA_R0_R1	22,09488106	-78,23963928	-942,6171265	0,073768
Cloud_Mesh_Distance_BA_R0_R1	21,79968262	-77,84483337	-942,4269409	0,088277
Cloud_Mesh_Distance_BA_R0_R1	21,79010391	-77,86260986	-942,6072998	0,091214
Cloud_Mesh_Distance_BA_R0_R1	21,86151314	-77,89979553	-942,2764893	0,088531
Cloud_Mesh_Distance_BA_R0_R1	21,98324013	-78,05204773	-942,3907471	0,086192
Cloud_Mesh_Distance_BA_R0_R1	15,99054527	-77,75079346	-942,3343506	-0,051515
Cloud_Mesh_Distance_BA_R0_R1	21,72097588	-77,75057983	-942,3220215	0,087614
Cloud_Mesh_Distance_BA_R0_R1	21,7040329	-77,74925232	-942,5101929	0,090918
Cloud_Mesh_Distance_BA_R0_R1	16,19281769	-77,41897583	-942,3283691	-0,03017
Cloud_Mesh_Distance_BA_R0_R1	16,11380577	-77,62442017	-942,2589111	-0,041529
Cloud_Mesh_Distance_BA_R0_R1	21,41794777	-77,44718933	-942,4536133	0,083208
Cloud_Mesh_Distance_BA_R0_R1	21,61060333	-77,64002991	-942,4151001	0,085523
Cloud_Mesh_Distance_BA_R0_R1	21,35427284	-77,38607788	-942,3030396	0,073761
Cloud_Mesh_Distance_BA_R0_R1	20,97907066	-77,06248474	-942,4400024	0,057804
Cloud_Mesh_Distance_BA_R0_R1	21,09371567	-77,1574707	-942,3238525	0,062677
Cloud_Mesh_Distance_BA_R0_R1	16,60440254	-76,97128296	-942,2821045	0,017801
Cloud_Mesh_Distance_BA_R0_R1	20,98735619	-77,06994629	-942,6509399	0,066048
Cloud_Mesh_Distance_BA_R0_R1	20,85761261	-76,97013855	-942,5509033	0,0497
Cloud_Mesh_Distance_BA_R0_R1	17,23984337	-76,55848694	-942,2709351	0,039322
Cloud_Mesh_Distance_BA_R0_R1	20,58454514	-76,79190063	-942,5369263	0,024181
Cloud_Mesh_Distance_BA_R0_R1	19,99271774	-76,51278687	-942,4854736	-0,02459
Cloud_Mesh_Distance_BA_R0_R1	20,07888603	-76,56454468	-942,359314	-0,014424
Cloud_Mesh_Distance_BA_R0_R1	20,17927551	-76,58586121	-942,5073242	-0,009677
Cloud_Mesh_Distance_BA_R0_R1	20,06882286	-76,519104	-942,6720581	-0,021142
Cloud_Mesh_Distance_BA_R0_R1	17,53503609	-76,36572266	-942,520752	-0,00765
Cloud_Mesh_Distance_BA_R0_R1	17,47927284	-76,45269775	-942,3125	0,025913
Cloud_Mesh_Distance_BA_R0_R1	17,91977501	-76,3494873	-942,3535767	-0,015762
Cloud_Mesh_Distance_BA_R0_R1	18,11889076	-76,33178711	-942,347168	-0,03184
Cloud_Mesh_Distance_BA_R0_R1	18,37765121	-76,28910828	-942,4916992	-0,079053
Cloud_Mesh_Distance_BA_R0_R1	18,17425728	-76,29441833	-942,4967651	-0,063979
Cloud_Mesh_Distance_BA_R0_R1	18,31980133	-76,32504272	-942,3272095	-0,04273
Cloud_Mesh_Distance_BA_R0_R1	18,53398132	-76,32200623	-942,3170776	-0,050799
Cloud_Mesh_Distance_BA_R0_R1	18,91459846	-76,31793213	-942,3914795	-0,071783
Cloud_Mesh_Distance_BA_R0_R1	19,10500526	-76,34265137	-942,3377686	-0,062294
Cloud_Mesh_Distance_BA_R0_R1	19,47927666	-76,34506226	-942,6251221	-0,06962

Cloud_Mesh_Distance_BA_R0_R1	19,66035652	-76,39312744	-942,5801392	-0,051309
Cloud_Mesh_Distance_BA_R0_R1	19,81471443	-76,46005249	-942,4376831	-0,038443
Cloud_Mesh_Distance_BA_R0_R1	19,87411308	-76,449646	-942,6290283	-0,035292
Cloud_Mesh_Distance_BA_R0_R1	18,12583542	-83,97625732	-941,6605225	0,034497
Cloud_Mesh_Distance_BA_R0_R1	17,77704239	-83,71182251	-941,7875977	0,006835
Cloud_Mesh_Distance_BA_R0_R1	17,71777344	-83,58740234	-941,5842896	-0,012158
Cloud_Mesh_Distance_BA_R0_R1	17,25942421	-83,10520935	-941,6547241	-0,037651
Cloud_Mesh_Distance_BA_R0_R1	17,3552494	-83,26036072	-941,772583	-0,033688
Cloud_Mesh_Distance_BA_R0_R1	17,46021843	-83,37258911	-941,7485962	-0,027276
Cloud_Mesh_Distance_BA_R0_R1	16,93182182	-82,67932129	-941,7072754	-0,032775
Cloud_Mesh_Distance_BA_R0_R1	17,12740707	-82,9206543	-941,6161499	-0,039049
Cloud_Mesh_Distance_BA_R0_R1	16,67694283	-82,31869507	-941,814148	-0,008858
Cloud_Mesh_Distance_BA_R0_R1	23,30245399	-82,37217712	-942,2711792	0,299208
Cloud_Mesh_Distance_BA_R0_R1	16,50059319	-81,97787476	-941,7883911	0,021603
Cloud_Mesh_Distance_BA_R0_R1	16,4965229	-82,06958008	-941,961792	0,010475
Cloud_Mesh_Distance_BA_R0_R1	23,16961861	-81,94555664	-942,1982422	0,103854
Cloud_Mesh_Distance_BA_R0_R1	16,3358078	-81,72135925	-941,9205933	0,039772
Cloud_Mesh_Distance_BA_R0_R1	23,14549637	-81,75068665	-942,1890869	0,029113
Cloud_Mesh_Distance_BA_R0_R1	23,13764954	-81,88696289	-941,9977417	0,062702
Cloud_Mesh_Distance_BA_R0_R1	23,12414169	-81,68991089	-941,9829102	-0,010862
Cloud_Mesh_Distance_BA_R0_R1	16,33818054	-81,56248474	-941,7205811	0,051722
Cloud_Mesh_Distance_BA_R0_R1	16,25037193	-81,53829956	-941,9313965	0,047663
Cloud_Mesh_Distance_BA_R0_R1	16,24365807	-81,38983154	-941,7919312	0,055992
Cloud_Mesh_Distance_BA_R0_R1	23,12672615	-81,47201538	-941,9672241	-0,077863
Cloud_Mesh_Distance_BA_R0_R1	23,14654732	-81,14868164	-942,1965332	-0,131432
Cloud_Mesh_Distance_BA_R0_R1	23,1408577	-81,27790833	-942,0306397	-0,117863
Cloud_Mesh_Distance_BA_R0_R1	23,16455841	-80,86828613	-942,0180054	-0,182998
Cloud_Mesh_Distance_BA_R0_R1	15,91801167	-80,39717102	-941,8560791	0,063864
Cloud_Mesh_Distance_BA_R0_R1	15,89588833	-80,55975342	-941,9575806	0,053666
Cloud_Mesh_Distance_BA_R0_R1	15,98656845	-80,73698425	-941,8770142	0,065848
Cloud_Mesh_Distance_BA_R0_R1	23,15838432	-80,76998901	-942,1884155	-0,178381
Cloud_Mesh_Distance_BA_R0_R1	23,14656448	-80,58251953	-942,2792358	-0,168417
Cloud_Mesh_Distance_BA_R0_R1	23,13115311	-80,39556885	-942,303833	-0,160661
Cloud_Mesh_Distance_BA_R0_R1	15,89642811	-80,23764038	-941,847168	0,061516
Cloud_Mesh_Distance_BA_R0_R1	15,82210827	-80,0952301	-941,9361572	0,046513
Cloud_Mesh_Distance_BA_R0_R1	23,12001038	-80,25360107	-942,2550659	-0,147707
Cloud_Mesh_Distance_BA_R0_R1	23,09441376	-80,08047485	-942,2248535	-0,131566
Cloud_Mesh_Distance_BA_R0_R1	23,05860519	-79,90866089	-942,1975098	-0,107378
Cloud_Mesh_Distance_BA_R0_R1	23,06946373	-79,88864136	-941,9933472	-0,108876
Cloud_Mesh_Distance_BA_R0_R1	23,10769463	-80,06155396	-942,0197144	-0,13069
Cloud_Mesh_Distance_BA_R0_R1	22,99882317	-79,77134705	-942,3674316	-0,088195
Cloud_Mesh_Distance_BA_R0_R1	15,81397533	-79,33125305	-941,9309082	0,016801
Cloud_Mesh_Distance_BA_R0_R1	22,87637329	-79,41375732	-942,3039551	-0,034409
Cloud_Mesh_Distance_BA_R0_R1	15,92405796	-79,35179138	-941,772522	0,038278
Cloud_Mesh_Distance_BA_R0_R1	15,86417294	-79,10372925	-941,8981934	0,009823
Cloud_Mesh_Distance_BA_R0_R1	15,91245651	-78,85229492	-941,8926392	-0,000866
Cloud_Mesh_Distance_BA_R0_R1	15,82015896	-78,91819763	-942,001648	-0,012683
Cloud_Mesh_Distance_BA_R0_R1	15,99542713	-78,94845581	-941,7531738	0,02055
Cloud_Mesh_Distance_BA_R0_R1	15,83837509	-78,74560547	-942,0267944	-0,021836
Cloud_Mesh_Distance_BA_R0_R1	22,70049667	-79,07255554	-942,3463135	0,005827

Cloud_Mesh_Distance_BA_R0_R1	22,74109077	-79,09762573	-942,1380615	0,011753
Cloud_Mesh_Distance_BA_R0_R1	15,94952202	-78,63903809	-941,9091187	-0,012193
Cloud_Mesh_Distance_BA_R0_R1	22,44587135	-78,66404724	-942,3665161	0,047088
Cloud_Mesh_Distance_BA_R0_R1	22,43050575	-78,60552979	-942,1652222	0,060249
Cloud_Mesh_Distance_BA_R0_R1	22,54320335	-78,7800293	-942,210144	0,043331
Cloud_Mesh_Distance_BA_R0_R1	16,00666046	-78,39332581	-941,9294434	-0,022479
Cloud_Mesh_Distance_BA_R0_R1	22,35048676	-78,51391602	-942,3065796	0,061478
Cloud_Mesh_Distance_BA_R0_R1	22,33268738	-78,46684265	-942,1395874	0,068475
Cloud_Mesh_Distance_BA_R0_R1	22,25006294	-78,35214233	-942,034668	0,074384
Cloud_Mesh_Distance_BA_R0_R1	22,23722649	-78,35083008	-942,2440186	0,073815
Cloud_Mesh_Distance_BA_R0_R1	16,04406548	-78,0193634	-942,0754395	-0,037749
Cloud_Mesh_Distance_BA_R0_R1	16,26499748	-77,86279297	-941,8881836	-0,020435
Cloud_Mesh_Distance_BA_R0_R1	16,17124367	-77,84628296	-942,0174561	-0,030324
Cloud_Mesh_Distance_BA_R0_R1	22,09367752	-78,17419434	-942,2990723	0,080253
Cloud_Mesh_Distance_BA_R0_R1	22,16960907	-78,29222107	-942,4307251	0,074355
Cloud_Mesh_Distance_BA_R0_R1	16,07012749	-77,81091309	-942,1766968	-0,041301
Cloud_Mesh_Distance_BA_R0_R1	21,98139572	-78,03063965	-942,1734619	0,083926
Cloud_Mesh_Distance_BA_R0_R1	16,18184471	-77,69946289	-942,109314	-0,031487
Cloud_Mesh_Distance_BA_R0_R1	16,25861359	-77,53826904	-942,1374512	-0,025189
Cloud_Mesh_Distance_BA_R0_R1	21,73887634	-77,76638794	-942,1520996	0,082985
Cloud_Mesh_Distance_BA_R0_R1	16,40681076	-77,48242188	-942,0023193	-0,012518
Cloud_Mesh_Distance_BA_R0_R1	21,50048828	-77,52552795	-942,3309937	0,081232
Cloud_Mesh_Distance_BA_R0_R1	16,49934006	-77,31744385	-942,0397949	-0,005303
Cloud_Mesh_Distance_BA_R0_R1	16,50998688	-77,15457153	-942,1865845	0,00466
Cloud_Mesh_Distance_BA_R0_R1	16,36022949	-77,34785461	-942,1773071	-0,017542
Cloud_Mesh_Distance_BA_R0_R1	16,70825958	-77,0284729	-942,100769	0,020137
Cloud_Mesh_Distance_BA_R0_R1	16,74614334	-76,90679932	-942,2044678	0,030669
Cloud_Mesh_Distance_BA_R0_R1	16,85735321	-77,03497314	-941,9403076	0,031985
Cloud_Mesh_Distance_BA_R0_R1	21,21668434	-77,25930786	-942,4246216	0,073475
Cloud_Mesh_Distance_BA_R0_R1	16,86164474	-76,80117798	-942,2250977	0,036096
Cloud_Mesh_Distance_BA_R0_R1	16,86742401	-76,90740967	-942,0783081	0,035708
Cloud_Mesh_Distance_BA_R0_R1	16,99549866	-76,79460144	-942,1013184	0,043534
Cloud_Mesh_Distance_BA_R0_R1	20,86102295	-76,97912598	-942,3571777	0,048895
Cloud_Mesh_Distance_BA_R0_R1	20,95009995	-77,05499268	-942,204834	0,052973
Cloud_Mesh_Distance_BA_R0_R1	17,02668953	-76,68243408	-942,2416382	0,042818
Cloud_Mesh_Distance_BA_R0_R1	20,1336689	-76,63464355	-942,0647583	0,002491
Cloud_Mesh_Distance_BA_R0_R1	20,23101997	-76,63642883	-942,2938232	-0,001954
Cloud_Mesh_Distance_BA_R0_R1	20,32739258	-76,70330811	-942,1268921	0,010191
Cloud_Mesh_Distance_BA_R0_R1	20,54506111	-76,78100586	-942,3739014	0,02243
Cloud_Mesh_Distance_BA_R0_R1	20,40610886	-76,71624756	-942,3011475	0,010982
Cloud_Mesh_Distance_BA_R0_R1	20,49555969	-76,77503967	-942,1885376	0,019232
Cloud_Mesh_Distance_BA_R0_R1	20,63223267	-76,8421936	-942,2523804	0,02985
Cloud_Mesh_Distance_BA_R0_R1	20,71751022	-76,87994385	-942,4182129	0,036267
Cloud_Mesh_Distance_BA_R0_R1	17,43753433	-76,56413269	-942,098999	0,050427
Cloud_Mesh_Distance_BA_R0_R1	17,87697601	-76,40977478	-942,1809082	0,018022
Cloud_Mesh_Distance_BA_R0_R1	18,24283218	-76,359375	-942,2055664	-0,01256
Cloud_Mesh_Distance_BA_R0_R1	19,2643795	-76,4241333	-942,0323486	-0,016352
Cloud_Mesh_Distance_BA_R0_R1	19,64251137	-76,42718506	-942,3579102	-0,042406
Cloud_Mesh_Distance_BA_R0_R1	19,7754612	-76,47735596	-942,2752686	-0,030851
Cloud_Mesh_Distance_BA_R0_R1	20,03373337	-76,57452393	-942,1952515	-0,011746

Cloud_Mesh_Distance_BA_R0_R1	19,92855835	-76,51776123	-942,3134766	-0,023338
Cloud_Mesh_Distance_BA_R0_R1	17,70928764	-76,38548279	-942,3439331	0,003248
Cloud_Mesh_Distance_BA_R0_R1	18,07348251	-76,37776184	-942,1951904	0,00031
Cloud_Mesh_Distance_BA_R0_R1	18,61728096	-76,41355896	-941,9484253	0,01388
Cloud_Mesh_Distance_BA_R0_R1	18,68844414	-76,35977173	-942,1471558	-0,025114
Cloud_Mesh_Distance_BA_R0_R1	18,7446537	-76,31965637	-942,342041	-0,063657
Cloud_Mesh_Distance_BA_R0_R1	19,11764336	-76,39204407	-942,0999146	-0,028077
Cloud_Mesh_Distance_BA_R0_R1	18,9158783	-76,35966492	-942,1801148	-0,037071
Cloud_Mesh_Distance_BA_R0_R1	19,28087425	-76,38903809	-942,2036133	-0,04081
Cloud_Mesh_Distance_BA_R0_R1	19,30985641	-76,35696411	-942,3927612	-0,062701
Cloud_Mesh_Distance_BA_R0_R1	19,4649353	-76,40548706	-942,2714233	-0,043304
Cloud_Mesh_Distance_BA_R0_R1	19,48692513	-76,37445068	-942,4614258	-0,05929
Cloud_Mesh_Distance_BA_R0_R1	18,94936943	-84,40826416	-941,4706421	0,046316
Cloud_Mesh_Distance_BA_R0_R1	18,62878799	-84,30018616	-941,5977783	0,05872
Cloud_Mesh_Distance_BA_R0_R1	20,88817024	-84,45428467	-941,2099609	0,207049
Cloud_Mesh_Distance_BA_R0_R1	18,45056725	-84,17425537	-941,5596924	0,04402
Cloud_Mesh_Distance_BA_R0_R1	18,28501701	-84,04211426	-941,5141602	0,026753
Cloud_Mesh_Distance_BA_R0_R1	18,43873215	-84,08518982	-941,3690186	0,011041
Cloud_Mesh_Distance_BA_R0_R1	18,27421188	-83,95233154	-941,319519	-0,000876
Cloud_Mesh_Distance_BA_R0_R1	18,14166832	-83,91668701	-941,4694824	0,010056
Cloud_Mesh_Distance_BA_R0_R1	17,9425621	-83,78016663	-941,5430298	0,003874
Cloud_Mesh_Distance_BA_R0_R1	17,55354118	-83,42199707	-941,5905151	-0,023945
Cloud_Mesh_Distance_BA_R0_R1	17,21481514	-82,98710632	-941,4883423	-0,039636
Cloud_Mesh_Distance_BA_R0_R1	17,41005516	-83,26473999	-941,5944824	-0,033344
Cloud_Mesh_Distance_BA_R0_R1	17,13323975	-82,86227417	-941,4491577	-0,035858
Cloud_Mesh_Distance_BA_R0_R1	17,01756668	-82,73413086	-941,5338745	-0,033085
Cloud_Mesh_Distance_BA_R0_R1	16,87459946	-82,53219604	-941,5703125	-0,023019
Cloud_Mesh_Distance_BA_R0_R1	16,75971603	-82,4082489	-941,7070313	-0,015349
Cloud_Mesh_Distance_BA_R0_R1	16,67613411	-82,23561096	-941,6532593	0,000155
Cloud_Mesh_Distance_BA_R0_R1	16,58822632	-82,05131531	-941,6260986	0,016646
Cloud_Mesh_Distance_BA_R0_R1	16,50756454	-81,86705017	-941,6049805	0,033145
Cloud_Mesh_Distance_BA_R0_R1	23,22707558	-82,27391052	-942,0033569	0,261576
Cloud_Mesh_Distance_BA_R0_R1	23,16922569	-82,07672119	-942,0030518	0,147589
Cloud_Mesh_Distance_BA_R0_R1	23,11593819	-82,01229858	-941,8157349	0,100018
Cloud_Mesh_Distance_BA_R0_R1	16,44256401	-81,68191528	-941,5651245	0,04494
Cloud_Mesh_Distance_BA_R0_R1	16,42124748	-81,78872681	-941,7575684	0,037141
Cloud_Mesh_Distance_BA_R0_R1	16,51888847	-81,77346802	-941,4556885	0,035721
Cloud_Mesh_Distance_BA_R0_R1	23,09767151	-81,83187866	-941,8029785	0,02286
Cloud_Mesh_Distance_BA_R0_R1	16,3953228	-81,47955322	-941,4900513	0,055908
Cloud_Mesh_Distance_BA_R0_R1	16,29672623	-81,33035278	-941,6082764	0,064791
Cloud_Mesh_Distance_BA_R0_R1	16,36312294	-81,26425171	-941,3998413	0,066231
Cloud_Mesh_Distance_BA_R0_R1	23,15259361	-81,09054565	-941,9732666	-0,16433
Cloud_Mesh_Distance_BA_R0_R1	16,2710762	-81,11509705	-941,5131226	0,070973
Cloud_Mesh_Distance_BA_R0_R1	16,18077087	-81,16552734	-941,7501221	0,067267
Cloud_Mesh_Distance_BA_R0_R1	23,15985489	-80,93878174	-941,8200684	-0,200166
Cloud_Mesh_Distance_BA_R0_R1	16,08184242	-80,71890259	-941,687439	0,071716
Cloud_Mesh_Distance_BA_R0_R1	15,99254799	-80,53945923	-941,7761231	0,072259
Cloud_Mesh_Distance_BA_R0_R1	16,17293358	-80,67468262	-941,4951172	0,083748
Cloud_Mesh_Distance_BA_R0_R1	23,17019653	-80,61320496	-941,8682251	-0,207669
Cloud_Mesh_Distance_BA_R0_R1	23,16617584	-80,67694092	-942,0540772	-0,187828

Cloud_Mesh_Distance_BA_R0_R1	23,16896629	-80,77783203	-941,8865967	-0,20493
Cloud_Mesh_Distance_BA_R0_R1	16,08519936	-80,5083313	-941,5992432	0,086135
Cloud_Mesh_Distance_BA_R0_R1	23,155056	-80,45201111	-942,0928955	-0,179438
Cloud_Mesh_Distance_BA_R0_R1	23,16174126	-80,41300964	-941,8535767	-0,185254
Cloud_Mesh_Distance_BA_R0_R1	15,99977875	-80,30992126	-941,6920776	0,080573
Cloud_Mesh_Distance_BA_R0_R1	23,1415863	-80,22116089	-941,8313599	-0,163037
Cloud_Mesh_Distance_BA_R0_R1	15,84936333	-79,91516113	-941,8676758	0,052318
Cloud_Mesh_Distance_BA_R0_R1	15,9394207	-80,051651	-941,743042	0,068069
Cloud_Mesh_Distance_BA_R0_R1	23,13657761	-80,24401855	-942,0455322	-0,156117
Cloud_Mesh_Distance_BA_R0_R1	16,01360703	-79,70678711	-941,6126099	0,071938
Cloud_Mesh_Distance_BA_R0_R1	15,92626286	-79,8152771	-941,7444458	0,064114
Cloud_Mesh_Distance_BA_R0_R1	23,01284218	-79,73501587	-942,1621704	-0,077345
Cloud_Mesh_Distance_BA_R0_R1	23,02261925	-79,72119141	-941,9556274	-0,078894
Cloud_Mesh_Distance_BA_R0_R1	23,07105446	-79,87342834	-941,7938843	-0,107114
Cloud_Mesh_Distance_BA_R0_R1	23,11087227	-80,04144287	-941,8134766	-0,134238
Cloud_Mesh_Distance_BA_R0_R1	16,02314758	-79,48947144	-941,6140137	0,064715
Cloud_Mesh_Distance_BA_R0_R1	15,92228222	-79,6008606	-941,7525024	0,053692
Cloud_Mesh_Distance_BA_R0_R1	22,94891739	-79,53652954	-942,1028442	-0,047923
Cloud_Mesh_Distance_BA_R0_R1	22,9770031	-79,58358765	-941,899353	-0,058752
Cloud_Mesh_Distance_BA_R0_R1	15,98219872	-79,1444397	-941,7258301	0,035466
Cloud_Mesh_Distance_BA_R0_R1	16,04918861	-79,26760864	-941,6077881	0,055655
Cloud_Mesh_Distance_BA_R0_R1	16,08455086	-79,04376221	-941,6066895	0,041226
Cloud_Mesh_Distance_BA_R0_R1	22,85225487	-79,31298828	-942,1248169	-0,017631
Cloud_Mesh_Distance_BA_R0_R1	22,82214165	-79,22924805	-941,9406128	-0,001774
Cloud_Mesh_Distance_BA_R0_R1	16,12390518	-78,78573608	-941,6255493	0,026
Cloud_Mesh_Distance_BA_R0_R1	16,0130043	-78,75750732	-941,7842407	0,007476
Cloud_Mesh_Distance_BA_R0_R1	22,63266182	-78,90104675	-942,0593262	0,034905
Cloud_Mesh_Distance_BA_R0_R1	22,60577583	-78,84857178	-941,8706055	0,044499
Cloud_Mesh_Distance_BA_R0_R1	22,71515083	-79,0295105	-941,8740845	0,026428
Cloud_Mesh_Distance_BA_R0_R1	16,08446312	-78,56806946	-941,7546997	0,003582
Cloud_Mesh_Distance_BA_R0_R1	22,11604691	-78,18656921	-942,0722656	0,080614
Cloud_Mesh_Distance_BA_R0_R1	22,36791992	-78,50521851	-941,9716187	0,070339
Cloud_Mesh_Distance_BA_R0_R1	16,37879944	-77,80358887	-941,7901001	-0,01133
Cloud_Mesh_Distance_BA_R0_R1	16,18151665	-77,99887085	-941,9064331	-0,022436
Cloud_Mesh_Distance_BA_R0_R1	16,10229874	-78,1750946	-941,9085693	-0,023962
Cloud_Mesh_Distance_BA_R0_R1	16,21960068	-78,11616516	-941,7894898	-0,012091
Cloud_Mesh_Distance_BA_R0_R1	16,29933929	-77,97288513	-941,7745972	-0,012365
Cloud_Mesh_Distance_BA_R0_R1	16,30215263	-77,69024658	-941,9636231	-0,020766
Cloud_Mesh_Distance_BA_R0_R1	16,56830788	-77,41264343	-941,8771973	-0,002779
Cloud_Mesh_Distance_BA_R0_R1	16,46804237	-77,61276245	-941,8258667	-0,01006
Cloud_Mesh_Distance_BA_R0_R1	21,83736992	-77,87368774	-942,0291748	0,08142
Cloud_Mesh_Distance_BA_R0_R1	21,31806374	-77,36483765	-942,1035767	0,06607
Cloud_Mesh_Distance_BA_R0_R1	21,35492134	-77,41801453	-941,9358521	0,059804
Cloud_Mesh_Distance_BA_R0_R1	21,47476196	-77,50862122	-942,1083984	0,075769
Cloud_Mesh_Distance_BA_R0_R1	21,61797333	-77,64257813	-942,2147827	0,083637
Cloud_Mesh_Distance_BA_R0_R1	21,4967289	-77,55157471	-941,8999634	0,06486
Cloud_Mesh_Distance_BA_R0_R1	21,1777401	-77,2633667	-941,9544067	0,057194
Cloud_Mesh_Distance_BA_R0_R1	16,67083359	-77,20257568	-941,9591065	0,012413
Cloud_Mesh_Distance_BA_R0_R1	17,02845192	-77,04917908	-941,7598267	0,048895
Cloud_Mesh_Distance_BA_R0_R1	21,08736992	-77,16752625	-942,1270142	0,057698

Cloud_Mesh_Distance_BA_R0_R1	21,21322441	-77,2644043	-942,210144	0,067127
Cloud_Mesh_Distance_BA_R0_R1	17,03556252	-76,88250732	-941,9477539	0,047871
Cloud_Mesh_Distance_BA_R0_R1	20,76497841	-76,93078613	-942,1807251	0,038963
Cloud_Mesh_Distance_BA_R0_R1	17,19825935	-76,70625305	-942,0474854	0,053776
Cloud_Mesh_Distance_BA_R0_R1	17,66641045	-76,46934509	-942,1430054	0,036487
Cloud_Mesh_Distance_BA_R0_R1	17,85027122	-76,48699951	-941,9943237	0,049463
Cloud_Mesh_Distance_BA_R0_R1	18,2317028	-76,40438843	-942,0548706	0,017162
Cloud_Mesh_Distance_BA_R0_R1	18,03686333	-76,43615723	-942,0325317	0,032584
Cloud_Mesh_Distance_BA_R0_R1	18,44117355	-76,36967468	-942,1235352	-0,01124
Cloud_Mesh_Distance_BA_R0_R1	19,42252922	-76,43659973	-942,0831299	-0,02355
Cloud_Mesh_Distance_BA_R0_R1	19,92431641	-76,57403564	-942,0079956	-0,00204
Cloud_Mesh_Distance_BA_R0_R1	19,84372902	-76,52153015	-942,1463013	-0,017182
Cloud_Mesh_Distance_BA_R0_R1	19,63410568	-76,46543884	-942,1495361	-0,025559
Cloud_Mesh_Distance_BA_R0_R1	18,80642128	-76,39874268	-941,9989014	-0,004698
Cloud_Mesh_Distance_BA_R0_R1	19,13433456	-84,45887756	-941,4204102	0,038175
Cloud_Mesh_Distance_BA_R0_R1	19,26915741	-84,39006042	-941,2057495	-0,008976
Cloud_Mesh_Distance_BA_R0_R1	19,50833511	-84,4707489	-941,2396851	0,011168
Cloud_Mesh_Distance_BA_R0_R1	18,78632927	-84,31263733	-941,4311523	0,033455
Cloud_Mesh_Distance_BA_R0_R1	18,74359131	-84,20300293	-941,2492676	-0,004788
Cloud_Mesh_Distance_BA_R0_R1	19,12528419	-84,24995422	-941,0407105	-0,055304
Cloud_Mesh_Distance_BA_R0_R1	19,71332741	-84,52032471	-941,2618408	0,031504
Cloud_Mesh_Distance_BA_R0_R1	19,91050148	-84,54811096	-941,2710571	0,049035
Cloud_Mesh_Distance_BA_R0_R1	19,84142685	-84,4624176	-941,1447144	0,007676
Cloud_Mesh_Distance_BA_R0_R1	21,32710457	-84,49597168	-941,3175049	0,346432
Cloud_Mesh_Distance_BA_R0_R1	18,602108	-84,20341492	-941,4103394	0,022527
Cloud_Mesh_Distance_BA_R0_R1	21,15564156	-84,402771	-941,1968994	0,265223
Cloud_Mesh_Distance_BA_R0_R1	21,02766228	-84,28588867	-941,0496826	0,190488
Cloud_Mesh_Distance_BA_R0_R1	18,24399567	-83,83227539	-941,1072388	-0,035342
Cloud_Mesh_Distance_BA_R0_R1	18,09614372	-83,8163147	-941,3130493	-0,01106
Cloud_Mesh_Distance_BA_R0_R1	17,87733459	-83,57614136	-941,1785889	-0,034037
Cloud_Mesh_Distance_BA_R0_R1	17,81147385	-83,59976196	-941,3771362	-0,019018
Cloud_Mesh_Distance_BA_R0_R1	17,95150757	-83,6965332	-941,3091431	-0,018756
Cloud_Mesh_Distance_BA_R0_R1	17,62091064	-83,3515625	-941,2242432	-0,038893
Cloud_Mesh_Distance_BA_R0_R1	17,65574646	-83,45993042	-941,4041748	-0,026095
Cloud_Mesh_Distance_BA_R0_R1	17,74314499	-83,4823761	-941,24646	-0,031467
Cloud_Mesh_Distance_BA_R0_R1	17,22273445	-82,92689514	-941,3192749	-0,03739
Cloud_Mesh_Distance_BA_R0_R1	17,47693634	-83,18891907	-941,2075195	-0,040802
Cloud_Mesh_Distance_BA_R0_R1	17,34377861	-83,11529541	-941,404541	-0,03871
Cloud_Mesh_Distance_BA_R0_R1	17,50661087	-83,30281067	-941,4031372	-0,034163
Cloud_Mesh_Distance_BA_R0_R1	16,77416801	-82,33334351	-941,5097046	-0,007646
Cloud_Mesh_Distance_BA_R0_R1	16,86357307	-82,4223938	-941,3876343	-0,013069
Cloud_Mesh_Distance_BA_R0_R1	16,96190834	-82,5687561	-941,3588867	-0,024328
Cloud_Mesh_Distance_BA_R0_R1	16,5881443	-81,92518616	-941,444397	0,02575
Cloud_Mesh_Distance_BA_R0_R1	16,68828011	-82,12663269	-941,4317017	0,010334
Cloud_Mesh_Distance_BA_R0_R1	23,08548164	-82,27200317	-941,581604	0,194199
Cloud_Mesh_Distance_BA_R0_R1	23,1489563	-82,19271851	-941,8015747	0,187455
Cloud_Mesh_Distance_BA_R0_R1	22,89523315	-82,0994873	-941,2705078	0,035776
Cloud_Mesh_Distance_BA_R0_R1	23,00743103	-82,10760498	-941,4834595	0,091826
Cloud_Mesh_Distance_BA_R0_R1	23,05725861	-81,95800781	-941,6338501	0,05031
Cloud_Mesh_Distance_BA_R0_R1	23,08325005	-82,11105347	-941,663208	0,126213

Cloud_Mesh_Distance_BA_R0_R1	22,97591591	-81,87014771	-941,4124146	-0,019221
Cloud_Mesh_Distance_BA_R0_R1	23,09269714	-81,62858582	-941,7634277	-0,053865
Cloud_Mesh_Distance_BA_R0_R1	23,05435753	-81,79309082	-941,6294556	-0,01527
Cloud_Mesh_Distance_BA_R0_R1	23,10847092	-81,47135925	-941,796814	-0,094185
Cloud_Mesh_Distance_BA_R0_R1	23,08442879	-81,45751953	-941,6351929	-0,11462
Cloud_Mesh_Distance_BA_R0_R1	23,10801125	-81,27018738	-941,6281128	-0,158143
Cloud_Mesh_Distance_BA_R0_R1	23,12618446	-81,31204224	-941,8223267	-0,131543
Cloud_Mesh_Distance_BA_R0_R1	23,13818359	-81,11978149	-941,7283936	-0,180814
Cloud_Mesh_Distance_BA_R0_R1	16,26635361	-80,97348022	-941,4403687	0,076314
Cloud_Mesh_Distance_BA_R0_R1	16,3389473	-81,03453064	-941,3155518	0,073425
Cloud_Mesh_Distance_BA_R0_R1	16,18660545	-80,91596985	-941,5768433	0,081221
Cloud_Mesh_Distance_BA_R0_R1	23,1628418	-80,78744507	-941,6868897	-0,226107
Cloud_Mesh_Distance_BA_R0_R1	16,10050011	-80,3352356	-941,5263672	0,088047
Cloud_Mesh_Distance_BA_R0_R1	16,17183113	-80,46774292	-941,4309082	0,094062
Cloud_Mesh_Distance_BA_R0_R1	16,16633606	-80,2890625	-941,4020996	0,092351
Cloud_Mesh_Distance_BA_R0_R1	16,09561539	-79,83093262	-941,4835205	0,089519
Cloud_Mesh_Distance_BA_R0_R1	16,18297195	-79,93817139	-941,3424683	0,097808
Cloud_Mesh_Distance_BA_R0_R1	16,08671379	-80,10032654	-941,5114136	0,089906
Cloud_Mesh_Distance_BA_R0_R1	16,215065	-80,15139771	-941,298584	0,09899
Cloud_Mesh_Distance_BA_R0_R1	16,0112381	-79,89930725	-941,6164551	0,079437
Cloud_Mesh_Distance_BA_R0_R1	23,06208611	-79,85479736	-941,6275635	-0,107654
Cloud_Mesh_Distance_BA_R0_R1	23,02394104	-79,7166748	-941,7596436	-0,080989
Cloud_Mesh_Distance_BA_R0_R1	23,10046959	-80,00878906	-941,6158447	-0,136176
Cloud_Mesh_Distance_BA_R0_R1	16,10591888	-79,61376953	-941,4779663	0,083951
Cloud_Mesh_Distance_BA_R0_R1	16,19977379	-79,52984619	-941,3428955	0,089029
Cloud_Mesh_Distance_BA_R0_R1	16,12755775	-79,39872742	-941,4723511	0,075759
Cloud_Mesh_Distance_BA_R0_R1	22,91034698	-79,41915894	-941,9334106	-0,02926
Cloud_Mesh_Distance_BA_R0_R1	22,95591927	-79,53294373	-941,7246094	-0,04548
Cloud_Mesh_Distance_BA_R0_R1	16,16370201	-79,17532349	-941,4633179	0,064925
Cloud_Mesh_Distance_BA_R0_R1	16,23449898	-79,32064819	-941,3271484	0,083624
Cloud_Mesh_Distance_BA_R0_R1	16,20771027	-78,94146729	-941,4627686	0,052273
Cloud_Mesh_Distance_BA_R0_R1	22,80740547	-79,20507813	-941,739502	0,002729
Cloud_Mesh_Distance_BA_R0_R1	22,87837219	-79,34637451	-941,796814	-0,01898
Cloud_Mesh_Distance_BA_R0_R1	22,73064804	-79,07006836	-941,6697388	0,019143
Cloud_Mesh_Distance_BA_R0_R1	22,5115757	-78,70823669	-941,9932861	0,057866
Cloud_Mesh_Distance_BA_R0_R1	16,23005104	-78,53695679	-941,5752564	0,019479
Cloud_Mesh_Distance_BA_R0_R1	16,18985939	-78,31546021	-941,7247315	-0,002037
Cloud_Mesh_Distance_BA_R0_R1	16,32977104	-78,12115479	-941,6499023	0,000064
Cloud_Mesh_Distance_BA_R0_R1	22,17163467	-78,25796509	-941,8778076	0,076599
Cloud_Mesh_Distance_BA_R0_R1	22,21989822	-78,33081055	-941,710083	0,073828
Cloud_Mesh_Distance_BA_R0_R1	22,26682091	-78,37495422	-941,8704834	0,072112
Cloud_Mesh_Distance_BA_R0_R1	16,42812538	-77,9329834	-941,6455078	-0,003742
Cloud_Mesh_Distance_BA_R0_R1	22,05836868	-78,12960815	-941,8414917	0,077948
Cloud_Mesh_Distance_BA_R0_R1	21,73679924	-77,83021545	-941,6120606	0,063537
Cloud_Mesh_Distance_BA_R0_R1	21,97273254	-78,0241394	-941,9614258	0,080299
Cloud_Mesh_Distance_BA_R0_R1	16,52028275	-77,74691772	-941,6660156	-0,002331
Cloud_Mesh_Distance_BA_R0_R1	16,77258492	-77,60487366	-941,4963379	0,020754
Cloud_Mesh_Distance_BA_R0_R1	16,64875603	-77,54402161	-941,6787109	0,00507
Cloud_Mesh_Distance_BA_R0_R1	21,65011978	-77,6870575	-941,9903565	0,076229
Cloud_Mesh_Distance_BA_R0_R1	21,58457375	-77,66120911	-941,7318115	0,061517

Cloud_Mesh_Distance_BA_R0_R1	21,84813499	-77,90814209	-941,7961426	0,072688
Cloud_Mesh_Distance_BA_R0_R1	21,74913979	-77,80410767	-941,8259277	0,069524
Cloud_Mesh_Distance_BA_R0_R1	21,27130699	-77,37014771	-941,7894287	0,053446
Cloud_Mesh_Distance_BA_R0_R1	16,74215508	-77,32403564	-941,769104	0,014108
Cloud_Mesh_Distance_BA_R0_R1	16,8371048	-77,16445923	-941,8243408	0,025067
Cloud_Mesh_Distance_BA_R0_R1	17,00150681	-77,39584351	-941,4411011	0,039771
Cloud_Mesh_Distance_BA_R0_R1	16,90186119	-77,22833252	-941,6973267	0,030135
Cloud_Mesh_Distance_BA_R0_R1	17,06451225	-77,2237854	-941,5446167	0,05097
Cloud_Mesh_Distance_BA_R0_R1	21,03904152	-77,18469238	-941,7861939	0,044967
Cloud_Mesh_Distance_BA_R0_R1	20,97554588	-77,10707092	-941,9489746	0,050014
Cloud_Mesh_Distance_BA_R0_R1	17,54387474	-76,72914124	-941,7407837	0,083157
Cloud_Mesh_Distance_BA_R0_R1	17,27601242	-76,84152222	-941,7897339	0,06799
Cloud_Mesh_Distance_BA_R0_R1	20,69753838	-76,96943665	-941,7234497	0,03939
Cloud_Mesh_Distance_BA_R0_R1	20,70825386	-76,93061829	-941,940918	0,037209
Cloud_Mesh_Distance_BA_R0_R1	20,84753799	-77,00466919	-942,0263062	0,046083
Cloud_Mesh_Distance_BA_R0_R1	20,82183456	-77,01646423	-941,8602295	0,040759
Cloud_Mesh_Distance_BA_R0_R1	17,41705894	-76,67703247	-941,9146729	0,069577
Cloud_Mesh_Distance_BA_R0_R1	17,6460743	-76,584198	-941,9134522	0,069321
Cloud_Mesh_Distance_BA_R0_R1	17,73939896	-76,6594696	-941,7220459	0,088165
Cloud_Mesh_Distance_BA_R0_R1	17,87763023	-76,57118225	-941,8018799	0,074029
Cloud_Mesh_Distance_BA_R0_R1	19,82840538	-76,63148499	-941,6668091	0,022536
Cloud_Mesh_Distance_BA_R0_R1	19,99950981	-76,6315918	-941,8554688	0,011412
Cloud_Mesh_Distance_BA_R0_R1	20,41870117	-76,76852417	-941,9859619	0,021802
Cloud_Mesh_Distance_BA_R0_R1	20,22061157	-76,69706726	-941,914978	0,018881
Cloud_Mesh_Distance_BA_R0_R1	20,59551048	-76,84587097	-942,0635986	0,029753
Cloud_Mesh_Distance_BA_R0_R1	18,10687637	-76,55963135	-941,715332	0,076236
Cloud_Mesh_Distance_BA_R0_R1	18,00848579	-76,49998474	-941,8858032	0,061424
Cloud_Mesh_Distance_BA_R0_R1	18,18283653	-76,4697113	-941,8868408	0,048845
Cloud_Mesh_Distance_BA_R0_R1	18,39273834	-76,43714905	-941,9123535	0,033926
Cloud_Mesh_Distance_BA_R0_R1	18,32377434	-76,50953674	-941,7446899	0,06458
Cloud_Mesh_Distance_BA_R0_R1	18,53894234	-76,48468018	-941,7538452	0,048192
Cloud_Mesh_Distance_BA_R0_R1	18,97164154	-76,41705322	-941,953186	0,002206
Cloud_Mesh_Distance_BA_R0_R1	19,14947319	-76,44448853	-941,9012451	0,004337
Cloud_Mesh_Distance_BA_R0_R1	19,53600311	-76,48171997	-941,9785767	-0,009774
Cloud_Mesh_Distance_BA_R0_R1	19,77139473	-76,57217407	-941,8296509	0,00925
Cloud_Mesh_Distance_BA_R0_R1	19,71429253	-76,52088928	-941,9830322	-0,006801
Cloud_Mesh_Distance_BA_R0_R1	18,86519432	-84,14306641	-941,0301514	-0,051742
Cloud_Mesh_Distance_BA_R0_R1	18,98290443	-84,31747437	-941,2592773	-0,004693
Cloud_Mesh_Distance_BA_R0_R1	19,47579956	-84,35717773	-941,0620728	-0,035969
Cloud_Mesh_Distance_BA_R0_R1	19,67487717	-84,41639709	-941,1027832	-0,015661
Cloud_Mesh_Distance_BA_R0_R1	20,11290741	-84,57215881	-941,2924805	0,077675
Cloud_Mesh_Distance_BA_R0_R1	20,27005386	-84,4889679	-941,171875	0,070901
Cloud_Mesh_Distance_BA_R0_R1	20,46941185	-84,5144043	-941,2232666	0,119661
Cloud_Mesh_Distance_BA_R0_R1	20,65893555	-84,55204773	-941,2980957	0,17798
Cloud_Mesh_Distance_BA_R0_R1	21,49461365	-84,41664124	-941,2510986	0,375904
Cloud_Mesh_Distance_BA_R0_R1	21,67095947	-84,41056824	-941,2498169	0,426757
Cloud_Mesh_Distance_BA_R0_R1	21,36448479	-84,33996582	-941,1658325	0,311481
Cloud_Mesh_Distance_BA_R0_R1	21,70161057	-84,10910034	-941,0205078	0,359539
Cloud_Mesh_Distance_BA_R0_R1	21,4057827	-84,20776367	-941,053894	0,285943
Cloud_Mesh_Distance_BA_R0_R1	21,56365776	-84,26527405	-941,1233521	0,354995

Cloud_Mesh_Distance_BA_R0_R1	18,45913315	-83,99658203	-941,15448	-0,027883
Cloud_Mesh_Distance_BA_R0_R1	18,57777405	-84,1191864	-941,2603149	-0,005394
Cloud_Mesh_Distance_BA_R0_R1	18,65300751	-84,0917511	-941,1242676	-0,029593
Cloud_Mesh_Distance_BA_R0_R1	18,04370117	-83,69696045	-941,1376953	-0,033589
Cloud_Mesh_Distance_BA_R0_R1	17,76327896	-83,41412354	-941,0519409	-0,049492
Cloud_Mesh_Distance_BA_R0_R1	17,46928596	-83,08760071	-941,0094605	-0,049694
Cloud_Mesh_Distance_BA_R0_R1	17,34376717	-83,01422119	-941,1695557	-0,042281
Cloud_Mesh_Distance_BA_R0_R1	17,22374153	-82,83441162	-941,1188355	-0,039702
Cloud_Mesh_Distance_BA_R0_R1	17,11897278	-82,65707397	-941,0661011	-0,03167
Cloud_Mesh_Distance_BA_R0_R1	17,09618378	-82,73825073	-941,2890015	-0,034885
Cloud_Mesh_Distance_BA_R0_R1	16,91860771	-82,40585327	-941,192749	-0,014281
Cloud_Mesh_Distance_BA_R0_R1	17,02135849	-82,57196045	-941,1849365	-0,025393
Cloud_Mesh_Distance_BA_R0_R1	16,80229759	-82,26795959	-941,309082	-0,00436
Cloud_Mesh_Distance_BA_R0_R1	17,02589989	-82,48471069	-941,0223389	-0,024684
Cloud_Mesh_Distance_BA_R0_R1	22,83484077	-82,42880249	-941,1567383	0,155296
Cloud_Mesh_Distance_BA_R0_R1	22,95312881	-82,31767273	-941,3338013	0,158738
Cloud_Mesh_Distance_BA_R0_R1	16,83834457	-82,20530701	-941,1113892	-0,004137
Cloud_Mesh_Distance_BA_R0_R1	16,7486763	-82,11039734	-941,2307129	0,006997
Cloud_Mesh_Distance_BA_R0_R1	16,57696724	-81,77456665	-941,296875	0,033746
Cloud_Mesh_Distance_BA_R0_R1	16,48955154	-81,61834717	-941,3724976	0,048166
Cloud_Mesh_Distance_BA_R0_R1	16,66660881	-81,95449829	-941,2582398	0,01888
Cloud_Mesh_Distance_BA_R0_R1	16,65620804	-81,79129028	-941,1034546	0,026609
Cloud_Mesh_Distance_BA_R0_R1	23,03915215	-81,65441895	-941,5373535	-0,072842
Cloud_Mesh_Distance_BA_R0_R1	22,980196	-81,64050293	-941,3442993	-0,102333
Cloud_Mesh_Distance_BA_R0_R1	16,52040672	-81,35263062	-941,0831909	0,048365
Cloud_Mesh_Distance_BA_R0_R1	16,45747185	-81,41740417	-941,2825317	0,055298
Cloud_Mesh_Distance_BA_R0_R1	16,43194008	-81,20394897	-941,1994019	0,062052
Cloud_Mesh_Distance_BA_R0_R1	23,03530502	-81,50796509	-941,4487915	-0,123591
Cloud_Mesh_Distance_BA_R0_R1	16,42342377	-81,00305176	-941,1137085	0,067303
Cloud_Mesh_Distance_BA_R0_R1	23,06732559	-81,334198	-941,460083	-0,164678
Cloud_Mesh_Distance_BA_R0_R1	23,11002159	-81,11685181	-941,499939	-0,209874
Cloud_Mesh_Distance_BA_R0_R1	16,26914978	-80,82403564	-941,3640747	0,078125
Cloud_Mesh_Distance_BA_R0_R1	23,14546585	-80,9753418	-941,6398315	-0,216669
Cloud_Mesh_Distance_BA_R0_R1	16,31520081	-80,70648193	-941,2264404	0,080356
Cloud_Mesh_Distance_BA_R0_R1	16,2505722	-80,60568237	-941,321228	0,09028
Cloud_Mesh_Distance_BA_R0_R1	16,35784721	-80,84898376	-941,18927	0,078121
Cloud_Mesh_Distance_BA_R0_R1	16,31938362	-80,56497192	-941,1757202	0,088115
Cloud_Mesh_Distance_BA_R0_R1	23,1348114	-80,90487671	-941,4831543	-0,240233
Cloud_Mesh_Distance_BA_R0_R1	23,149786	-80,52839661	-941,4528809	-0,234111
Cloud_Mesh_Distance_BA_R0_R1	16,26109314	-80,38861084	-941,2485352	0,091962
Cloud_Mesh_Distance_BA_R0_R1	23,16580582	-80,5741272	-941,6589966	-0,224402
Cloud_Mesh_Distance_BA_R0_R1	23,15647697	-80,37887573	-941,6389771	-0,199298
Cloud_Mesh_Distance_BA_R0_R1	23,13770676	-80,33546448	-941,4308472	-0,199246
Cloud_Mesh_Distance_BA_R0_R1	23,13461304	-80,19062805	-941,6236572	-0,170526
Cloud_Mesh_Distance_BA_R0_R1	16,27854919	-79,98880005	-941,180481	0,101094
Cloud_Mesh_Distance_BA_R0_R1	16,24858475	-79,82312012	-941,2359619	0,100796
Cloud_Mesh_Distance_BA_R0_R1	16,18191528	-79,7315979	-941,3502808	0,094809
Cloud_Mesh_Distance_BA_R0_R1	16,26703644	-79,65530396	-941,2208862	0,099471
Cloud_Mesh_Distance_BA_R0_R1	16,29271317	-79,46490479	-941,2089233	0,095733
Cloud_Mesh_Distance_BA_R0_R1	16,42564201	-79,09729004	-941,1019898	0,084148

Cloud_Mesh_Distance_BA_R0_R1	16,2972641	-79,08407593	-941,2939453	0,073811
Cloud_Mesh_Distance_BA_R0_R1	22,88306427	-79,37713623	-941,6199341	-0,025528
Cloud_Mesh_Distance_BA_R0_R1	16,33040237	-78,8500824	-941,3224487	0,056332
Cloud_Mesh_Distance_BA_R0_R1	16,37166977	-78,68315125	-941,3295898	0,048767
Cloud_Mesh_Distance_BA_R0_R1	22,68515778	-79,02058411	-941,5076904	0,027613
Cloud_Mesh_Distance_BA_R0_R1	22,64228058	-78,91717529	-941,69104	0,038488
Cloud_Mesh_Distance_BA_R0_R1	16,35730743	-78,53819275	-941,4089966	0,032324
Cloud_Mesh_Distance_BA_R0_R1	16,26737595	-78,71986389	-941,454895	0,040147
Cloud_Mesh_Distance_BA_R0_R1	22,48175621	-78,70297241	-941,5045776	0,063168
Cloud_Mesh_Distance_BA_R0_R1	22,40654373	-78,57957458	-941,6135864	0,066583
Cloud_Mesh_Distance_BA_R0_R1	22,52538872	-78,73397827	-941,7186279	0,052721
Cloud_Mesh_Distance_BA_R0_R1	22,5796051	-78,84684753	-941,513916	0,045407
Cloud_Mesh_Distance_BA_R0_R1	16,45892525	-78,41687012	-941,3352051	0,035559
Cloud_Mesh_Distance_BA_R0_R1	16,36990356	-78,3175354	-941,4967651	0,017897
Cloud_Mesh_Distance_BA_R0_R1	22,42998695	-78,59162903	-941,8154907	0,065336
Cloud_Mesh_Distance_BA_R0_R1	16,63855362	-77,9914856	-941,3618774	0,019138
Cloud_Mesh_Distance_BA_R0_R1	16,48024178	-78,07749939	-941,4942017	0,012212
Cloud_Mesh_Distance_BA_R0_R1	22,12145233	-78,215271	-941,7153931	0,075002
Cloud_Mesh_Distance_BA_R0_R1	22,33006668	-78,46295166	-941,7522583	0,071712
Cloud_Mesh_Distance_BA_R0_R1	16,56888962	-77,87464905	-941,5206299	0,009178
Cloud_Mesh_Distance_BA_R0_R1	21,93909836	-78,00727844	-941,7717896	0,073352
Cloud_Mesh_Distance_BA_R0_R1	16,71436691	-77,7901001	-941,4161377	0,020278
Cloud_Mesh_Distance_BA_R0_R1	21,88444328	-77,98320007	-941,583313	0,067143
Cloud_Mesh_Distance_BA_R0_R1	16,64278412	-77,71157837	-941,553772	0,006874
Cloud_Mesh_Distance_BA_R0_R1	16,90613937	-77,51567078	-941,4300537	0,032106
Cloud_Mesh_Distance_BA_R0_R1	21,58048058	-77,72247314	-941,4512329	0,052688
Cloud_Mesh_Distance_BA_R0_R1	16,8579998	-77,40124512	-941,5797119	0,024655
Cloud_Mesh_Distance_BA_R0_R1	21,40637016	-77,4944458	-941,7540283	0,056042
Cloud_Mesh_Distance_BA_R0_R1	21,28168106	-77,42420959	-941,5884399	0,04671
Cloud_Mesh_Distance_BA_R0_R1	21,45286942	-77,57124329	-941,5891113	0,049431
Cloud_Mesh_Distance_BA_R0_R1	17,27522087	-77,16946411	-941,4121094	0,071598
Cloud_Mesh_Distance_BA_R0_R1	21,14353752	-77,28370667	-941,7037964	0,045695
Cloud_Mesh_Distance_BA_R0_R1	17,49066353	-76,89245605	-941,5565796	0,086137
Cloud_Mesh_Distance_BA_R0_R1	17,40570641	-77,04270935	-941,4428711	0,08097
Cloud_Mesh_Distance_BA_R0_R1	20,87963676	-77,09025574	-941,7006836	0,043769
Cloud_Mesh_Distance_BA_R0_R1	17,25193405	-77,02685547	-941,5855713	0,068896
Cloud_Mesh_Distance_BA_R0_R1	17,70762253	-76,88418579	-941,4197388	0,101728
Cloud_Mesh_Distance_BA_R0_R1	20,51781273	-76,85179138	-941,8092651	0,032161
Cloud_Mesh_Distance_BA_R0_R1	20,71547318	-77,03701782	-941,5168457	0,038699
Cloud_Mesh_Distance_BA_R0_R1	18,16828918	-76,65509033	-941,5155029	0,095761
Cloud_Mesh_Distance_BA_R0_R1	18,38981438	-76,62567139	-941,4889526	0,088798
Cloud_Mesh_Distance_BA_R0_R1	20,33225632	-76,82058716	-941,5960083	0,034525
Cloud_Mesh_Distance_BA_R0_R1	20,31703949	-76,76235962	-941,7943115	0,028643
Cloud_Mesh_Distance_BA_R0_R1	18,28352547	-76,57781982	-941,6126709	0,080804
Cloud_Mesh_Distance_BA_R0_R1	18,43803978	-76,55032349	-941,6223145	0,074509
Cloud_Mesh_Distance_BA_R0_R1	18,65443611	-76,56616211	-941,5442505	0,071407
Cloud_Mesh_Distance_BA_R0_R1	18,79256821	-76,46109009	-941,7922974	0,032018
Cloud_Mesh_Distance_BA_R0_R1	19,00488472	-76,46835327	-941,784668	0,026361
Cloud_Mesh_Distance_BA_R0_R1	18,92623138	-76,52444458	-941,6207275	0,051631
Cloud_Mesh_Distance_BA_R0_R1	19,35369301	-76,47540283	-941,8782959	0,003385

Cloud_Mesh_Distance_BA_R0_R1	19,3965435	-76,5406189	-941,6769409	0,029552
Cloud_Mesh_Distance_BA_R0_R1	19,55773926	-76,52416992	-941,8273315	0,009248
Cloud_Mesh_Distance_BA_R0_R1	20,23857689	-84,38240051	-941,0220947	0,027266
Cloud_Mesh_Distance_BA_R0_R1	20,04717255	-84,44822693	-941,1080933	0,021809
Cloud_Mesh_Distance_BA_R0_R1	20,42736435	-84,39538574	-941,0599365	0,067606
Cloud_Mesh_Distance_BA_R0_R1	20,64137459	-84,41755676	-941,1217041	0,122531
Cloud_Mesh_Distance_BA_R0_R1	18,62906647	-83,97735596	-940,935669	-0,069534
Cloud_Mesh_Distance_BA_R0_R1	19,06924248	-84,11521912	-940,8500366	-0,096879
Cloud_Mesh_Distance_BA_R0_R1	19,44889069	-84,24230957	-940,890564	-0,078906
Cloud_Mesh_Distance_BA_R0_R1	19,31206894	-84,29142761	-941,0170898	-0,055471
Cloud_Mesh_Distance_BA_R0_R1	19,65356827	-84,29914856	-940,9273682	-0,0625
Cloud_Mesh_Distance_BA_R0_R1	19,61538696	-84,1592865	-940,7321167	-0,112043
Cloud_Mesh_Distance_BA_R0_R1	19,85987091	-84,36227417	-940,9929199	-0,027903
Cloud_Mesh_Distance_BA_R0_R1	19,84872818	-84,22946167	-940,8063965	-0,07684
Cloud_Mesh_Distance_BA_R0_R1	20,0909481	-84,2897644	-940,8884277	-0,02882
Cloud_Mesh_Distance_BA_R0_R1	20,24820518	-84,15296936	-940,7306519	-0,045454
Cloud_Mesh_Distance_BA_R0_R1	20,35098457	-84,27288818	-940,8934937	0,008471
Cloud_Mesh_Distance_BA_R0_R1	20,5848465	-84,27661133	-940,9403687	0,062328
Cloud_Mesh_Distance_BA_R0_R1	21,12367249	-84,13275146	-940,9233398	0,165103
Cloud_Mesh_Distance_BA_R0_R1	20,94475365	-84,17294312	-940,9136963	0,12482
Cloud_Mesh_Distance_BA_R0_R1	20,80972862	-84,29434204	-941,0093384	0,13317
Cloud_Mesh_Distance_BA_R0_R1	21,23641586	-84,24354553	-941,0526733	0,239454
Cloud_Mesh_Distance_BA_R0_R1	21,63799858	-83,99273682	-940,9338379	0,29813
Cloud_Mesh_Distance_BA_R0_R1	18,41988182	-83,88143921	-940,9759522	-0,056068
Cloud_Mesh_Distance_BA_R0_R1	18,48196411	-83,82223511	-940,8022461	-0,089862
Cloud_Mesh_Distance_BA_R0_R1	21,34662628	-83,91398621	-940,8043213	0,166575
Cloud_Mesh_Distance_BA_R0_R1	21,19924736	-84,00457764	-940,831543	0,14703
Cloud_Mesh_Distance_BA_R0_R1	21,33513641	-84,08226013	-940,9337158	0,218015
Cloud_Mesh_Distance_BA_R0_R1	21,5252552	-84,10342407	-940,9921875	0,291342
Cloud_Mesh_Distance_BA_R0_R1	21,65268707	-83,86003113	-940,8538208	0,257687
Cloud_Mesh_Distance_BA_R0_R1	17,96997833	-83,54943848	-940,9555054	-0,055567
Cloud_Mesh_Distance_BA_R0_R1	18,14165115	-83,69062805	-940,9696045	-0,05591
Cloud_Mesh_Distance_BA_R0_R1	21,50214958	-83,96026611	-940,8823242	0,238091
Cloud_Mesh_Distance_BA_R0_R1	21,93936729	-83,75192261	-940,8538208	0,337787
Cloud_Mesh_Distance_BA_R0_R1	21,8031559	-83,92907715	-940,9215698	0,342081
Cloud_Mesh_Distance_BA_R0_R1	21,78392982	-83,77210999	-940,8344116	0,278795
Cloud_Mesh_Distance_BA_R0_R1	17,84807396	-83,39648438	-940,8602295	-0,065952
Cloud_Mesh_Distance_BA_R0_R1	18,16671944	-83,59936523	-940,7651978	-0,079332
Cloud_Mesh_Distance_BA_R0_R1	17,97971535	-83,44044495	-940,7349243	-0,080026
Cloud_Mesh_Distance_BA_R0_R1	22,05930519	-83,52978516	-940,7942505	0,307473
Cloud_Mesh_Distance_BA_R0_R1	21,8447094	-83,61857605	-940,7758789	0,246315
Cloud_Mesh_Distance_BA_R0_R1	17,60037613	-83,24610901	-941,0393677	-0,047524
Cloud_Mesh_Distance_BA_R0_R1	17,71105957	-83,27120972	-940,8666992	-0,059245
Cloud_Mesh_Distance_BA_R0_R1	17,58218956	-83,1459198	-940,8791504	-0,059433
Cloud_Mesh_Distance_BA_R0_R1	17,34907341	-82,91760254	-940,9622803	-0,045546
Cloud_Mesh_Distance_BA_R0_R1	17,48525047	-82,99812317	-940,8079834	-0,057605
Cloud_Mesh_Distance_BA_R0_R1	22,70301628	-82,74130249	-940,9841309	0,246523
Cloud_Mesh_Distance_BA_R0_R1	22,52944756	-82,84605408	-940,8699951	0,218273
Cloud_Mesh_Distance_BA_R0_R1	17,13216972	-82,56330872	-940,8759155	-0,035538
Cloud_Mesh_Distance_BA_R0_R1	22,77605629	-82,58599854	-941,0688477	0,205003

Cloud_Mesh_Distance_BA_R0_R1	22,93753624	-82,54251099	-941,230835	0,259561
Cloud_Mesh_Distance_BA_R0_R1	16,96837807	-82,24131775	-940,821167	-0,019972
Cloud_Mesh_Distance_BA_R0_R1	16,8776741	-82,13623047	-940,9159546	-0,006279
Cloud_Mesh_Distance_BA_R0_R1	16,94198799	-82,3114624	-940,9815064	-0,015361
Cloud_Mesh_Distance_BA_R0_R1	22,80943871	-82,27124023	-941,1427612	0,076998
Cloud_Mesh_Distance_BA_R0_R1	16,64932823	-81,64559937	-940,9868164	0,026085
Cloud_Mesh_Distance_BA_R0_R1	16,76197815	-81,99575806	-941,048645	0,008527
Cloud_Mesh_Distance_BA_R0_R1	22,85424805	-81,91540527	-941,1656494	-0,047473
Cloud_Mesh_Distance_BA_R0_R1	16,69217682	-81,58398438	-940,8341675	0,018139
Cloud_Mesh_Distance_BA_R0_R1	16,60788155	-81,47627258	-940,9573364	0,034719
Cloud_Mesh_Distance_BA_R0_R1	16,5616188	-81,58277893	-941,1536255	0,041253
Cloud_Mesh_Distance_BA_R0_R1	22,91320992	-81,75502014	-941,2286377	-0,079156
Cloud_Mesh_Distance_BA_R0_R1	22,92056084	-81,58984375	-941,163208	-0,128418
Cloud_Mesh_Distance_BA_R0_R1	22,83405876	-81,73390198	-941,0548706	-0,112684
Cloud_Mesh_Distance_BA_R0_R1	22,99879646	-81,43255615	-941,2782593	-0,155241
Cloud_Mesh_Distance_BA_R0_R1	16,50101852	-81,1471405	-941,008606	0,054621
Cloud_Mesh_Distance_BA_R0_R1	16,58064651	-81,284729	-940,8999634	0,035228
Cloud_Mesh_Distance_BA_R0_R1	16,57130241	-81,09533691	-940,8257446	0,04159
Cloud_Mesh_Distance_BA_R0_R1	16,49322701	-80,94226074	-940,9351196	0,057365
Cloud_Mesh_Distance_BA_R0_R1	16,42499352	-80,83610535	-941,0432739	0,068452
Cloud_Mesh_Distance_BA_R0_R1	23,08861923	-80,99488831	-941,2938843	-0,244012
Cloud_Mesh_Distance_BA_R0_R1	23,14886665	-80,7171936	-941,4780273	-0,245911
Cloud_Mesh_Distance_BA_R0_R1	23,11857986	-80,82580566	-941,3309326	-0,256591
Cloud_Mesh_Distance_BA_R0_R1	16,37961197	-80,68292236	-941,0879517	0,07933
Cloud_Mesh_Distance_BA_R0_R1	16,31987762	-80,19784546	-941,1148071	0,095973
Cloud_Mesh_Distance_BA_R0_R1	23,11135483	-80,1388092	-941,4217529	-0,160956
Cloud_Mesh_Distance_BA_R0_R1	16,40985298	-79,67446899	-940,9805298	0,100481
Cloud_Mesh_Distance_BA_R0_R1	23,06537628	-79,92539978	-941,4306641	-0,125716
Cloud_Mesh_Distance_BA_R0_R1	23,02780533	-79,89860535	-941,239502	-0,121345
Cloud_Mesh_Distance_BA_R0_R1	16,35046196	-79,58706665	-941,0939331	0,09906
Cloud_Mesh_Distance_BA_R0_R1	16,37404442	-79,42697144	-941,0884399	0,097877
Cloud_Mesh_Distance_BA_R0_R1	23,01711845	-79,72583008	-941,5588379	-0,083783
Cloud_Mesh_Distance_BA_R0_R1	22,94915009	-79,56037903	-941,4937134	-0,056416
Cloud_Mesh_Distance_BA_R0_R1	22,99699402	-79,74157715	-941,3475342	-0,093429
Cloud_Mesh_Distance_BA_R0_R1	22,91684914	-79,55911255	-941,2943726	-0,059261
Cloud_Mesh_Distance_BA_R0_R1	16,46120262	-79,30274963	-940,9835205	0,094531
Cloud_Mesh_Distance_BA_R0_R1	22,87978172	-79,4153595	-941,4416504	-0,032129
Cloud_Mesh_Distance_BA_R0_R1	22,79589844	-79,22103882	-941,5151978	-0,001257
Cloud_Mesh_Distance_BA_R0_R1	16,34443665	-79,28036499	-941,1699219	0,087409
Cloud_Mesh_Distance_BA_R0_R1	22,82804489	-79,36126709	-941,2988892	-0,0284
Cloud_Mesh_Distance_BA_R0_R1	16,40212631	-78,93536377	-941,1914063	0,069282
Cloud_Mesh_Distance_BA_R0_R1	16,5170536	-78,90493774	-941,0407715	0,076104
Cloud_Mesh_Distance_BA_R0_R1	16,60428619	-78,69532776	-941,0133667	0,063612
Cloud_Mesh_Distance_BA_R0_R1	16,45815086	-78,7461853	-941,1877441	0,061944
Cloud_Mesh_Distance_BA_R0_R1	16,54582977	-78,58677673	-941,1417236	0,051756
Cloud_Mesh_Distance_BA_R0_R1	22,62166977	-78,96772766	-941,3287354	0,029878
Cloud_Mesh_Distance_BA_R0_R1	16,45676613	-78,56729126	-941,2672119	0,042726
Cloud_Mesh_Distance_BA_R0_R1	16,55799484	-78,23120117	-941,3116455	0,027495
Cloud_Mesh_Distance_BA_R0_R1	16,55929565	-78,431427	-941,2011108	0,044516
Cloud_Mesh_Distance_BA_R0_R1	16,66101265	-78,4874115	-941,0437012	0,058273

Cloud_Mesh_Distance_BA_R0_R1	22,38038826	-78,59468079	-941,3822632	0,065339
Cloud_Mesh_Distance_BA_R0_R1	22,28684425	-78,43684387	-941,5459595	0,072273
Cloud_Mesh_Distance_BA_R0_R1	22,23359871	-78,42021179	-941,3470459	0,070134
Cloud_Mesh_Distance_BA_R0_R1	22,0207119	-78,11576843	-941,6375732	0,072178
Cloud_Mesh_Distance_BA_R0_R1	22,13747406	-78,26713562	-941,5164185	0,072966
Cloud_Mesh_Distance_BA_R0_R1	16,72898483	-78,09295654	-941,1921387	0,037696
Cloud_Mesh_Distance_BA_R0_R1	21,97735214	-78,11590576	-941,4249268	0,064997
Cloud_Mesh_Distance_BA_R0_R1	16,79578781	-77,889328	-941,2543335	0,034356
Cloud_Mesh_Distance_BA_R0_R1	16,88311386	-77,68099976	-941,3170776	0,031353
Cloud_Mesh_Distance_BA_R0_R1	21,78728676	-77,93991089	-941,3724976	0,05896
Cloud_Mesh_Distance_BA_R0_R1	17,05858612	-77,67176819	-941,1437378	0,05385
Cloud_Mesh_Distance_BA_R0_R1	17,33452797	-77,27160645	-941,257019	0,076584
Cloud_Mesh_Distance_BA_R0_R1	17,16373062	-77,34986877	-941,3307495	0,055822
Cloud_Mesh_Distance_BA_R0_R1	17,1601181	-77,5052948	-941,1893921	0,060697
Cloud_Mesh_Distance_BA_R0_R1	17,02664185	-77,51460266	-941,3098755	0,049947
Cloud_Mesh_Distance_BA_R0_R1	21,21831512	-77,43777466	-941,3659668	0,039452
Cloud_Mesh_Distance_BA_R0_R1	17,45185852	-77,14970398	-941,2904053	0,085979
Cloud_Mesh_Distance_BA_R0_R1	17,64539146	-77,13485718	-941,166687	0,101307
Cloud_Mesh_Distance_BA_R0_R1	21,0067997	-77,19844055	-941,6270142	0,042572
Cloud_Mesh_Distance_BA_R0_R1	20,92028427	-77,23817444	-941,3117676	0,033745
Cloud_Mesh_Distance_BA_R0_R1	21,07902336	-77,29360962	-941,4804077	0,040766
Cloud_Mesh_Distance_BA_R0_R1	17,77712822	-76,98936462	-941,2474365	0,104365
Cloud_Mesh_Distance_BA_R0_R1	17,57654953	-77,01031494	-941,3538818	0,094135
Cloud_Mesh_Distance_BA_R0_R1	20,55444336	-77,00984192	-941,3406982	0,036346
Cloud_Mesh_Distance_BA_R0_R1	20,74019814	-77,11883545	-941,3244629	0,035834
Cloud_Mesh_Distance_BA_R0_R1	17,87353706	-76,87623596	-941,3313599	0,107205
Cloud_Mesh_Distance_BA_R0_R1	17,84560966	-76,79391479	-941,4575195	0,100937
Cloud_Mesh_Distance_BA_R0_R1	20,36214256	-76,90983582	-941,3652954	0,039574
Cloud_Mesh_Distance_BA_R0_R1	20,53290176	-76,92584229	-941,5531006	0,040364
Cloud_Mesh_Distance_BA_R0_R1	17,92329979	-76,67727661	-941,5914307	0,096842
Cloud_Mesh_Distance_BA_R0_R1	17,70496368	-76,76701355	-941,5788574	0,094183
Cloud_Mesh_Distance_BA_R0_R1	18,03978157	-76,78465271	-941,37146	0,105932
Cloud_Mesh_Distance_BA_R0_R1	18,28471947	-76,73484802	-941,3421021	0,104444
Cloud_Mesh_Distance_BA_R0_R1	18,52420235	-76,69313049	-941,333313	0,09697
Cloud_Mesh_Distance_BA_R0_R1	18,75912094	-76,66239929	-941,3378296	0,087027
Cloud_Mesh_Distance_BA_R0_R1	18,87331009	-76,59223938	-941,4636231	0,071113
Cloud_Mesh_Distance_BA_R0_R1	18,97175217	-76,65007019	-941,3392334	0,082868
Cloud_Mesh_Distance_BA_R0_R1	19,06380272	-76,58009338	-941,4909058	0,059913
Cloud_Mesh_Distance_BA_R0_R1	19,18282318	-76,64698792	-941,3521729	0,06964
Cloud_Mesh_Distance_BA_R0_R1	19,60459328	-76,58010864	-941,6656494	0,025956
Cloud_Mesh_Distance_BA_R0_R1	19,4222393	-76,65777588	-941,3727417	0,060339
Cloud_Mesh_Distance_BA_R0_R1	19,65138054	-76,64892578	-941,4882202	0,040839
Cloud_Mesh_Distance_BA_R0_R1	19,61388779	-76,70700073	-941,3225708	0,058796
Cloud_Mesh_Distance_BA_R0_R1	19,79614258	-76,73200989	-941,3508911	0,051226
Cloud_Mesh_Distance_BA_R0_R1	19,97098351	-76,75227356	-941,4163208	0,041273
Cloud_Mesh_Distance_BA_R0_R1	19,86519623	-76,68882751	-941,513916	0,03708
Cloud_Mesh_Distance_BA_R0_R1	20,08821297	-76,71434021	-941,6466065	0,030064
Cloud_Mesh_Distance_BA_R0_R1	20,15367699	-76,81723022	-941,3984375	0,040815
Cloud_Mesh_Distance_BA_R0_R1	19,16605568	-76,51101685	-941,6876221	0,033198
Cloud_Mesh_Distance_BA_R0_R1	19,2770977	-76,58448792	-941,5134888	0,051636

Cloud_Mesh_Distance_BA_R0_R1	19,46250343	-76,59786987	-941,538269	0,042138
Cloud_Mesh_Distance_BA_R0_R1	19,22310257	-84,09199524	-940,741272	-0,120691
Cloud_Mesh_Distance_BA_R0_R1	19,25888062	-84,19100952	-940,8786011	-0,087038
Cloud_Mesh_Distance_BA_R0_R1	20,03384781	-84,14352417	-940,6953735	-0,081015
Cloud_Mesh_Distance_BA_R0_R1	20,16307259	-84,05430603	-940,598938	-0,086419
Cloud_Mesh_Distance_BA_R0_R1	20,5274601	-83,93566895	-940,5490723	-0,048516
Cloud_Mesh_Distance_BA_R0_R1	20,33402824	-84,01608276	-940,5863037	-0,06926
Cloud_Mesh_Distance_BA_R0_R1	20,5075016	-84,1153717	-940,737793	-0,009618
Cloud_Mesh_Distance_BA_R0_R1	20,76368904	-84,14282227	-940,8336792	0,063417
Cloud_Mesh_Distance_BA_R0_R1	20,99417114	-83,99821472	-940,7599487	0,08268
Cloud_Mesh_Distance_BA_R0_R1	19,09008217	-83,98690796	-940,640686	-0,143127
Cloud_Mesh_Distance_BA_R0_R1	18,86682701	-84	-940,7852173	-0,106183
Cloud_Mesh_Distance_BA_R0_R1	19,31408119	-84,02493286	-940,6104126	-0,147954
Cloud_Mesh_Distance_BA_R0_R1	19,48539543	-84,06161499	-940,6189575	-0,14218
Cloud_Mesh_Distance_BA_R0_R1	19,39023018	-84,13122559	-940,7416382	-0,118319
Cloud_Mesh_Distance_BA_R0_R1	21,19416809	-83,89039612	-940,7352905	0,106831
Cloud_Mesh_Distance_BA_R0_R1	18,29905128	-83,75796509	-940,8892822	-0,069999
Cloud_Mesh_Distance_BA_R0_R1	18,52230263	-83,72671509	-940,6053467	-0,119141
Cloud_Mesh_Distance_BA_R0_R1	18,68140221	-83,86584473	-940,7006226	-0,113118
Cloud_Mesh_Distance_BA_R0_R1	21,33481216	-83,73544312	-940,668396	0,10731
Cloud_Mesh_Distance_BA_R0_R1	18,33213806	-83,57307434	-940,5357056	-0,122382
Cloud_Mesh_Distance_BA_R0_R1	18,11809349	-83,44845581	-940,5675049	-0,107985
Cloud_Mesh_Distance_BA_R0_R1	21,50889969	-83,80386353	-940,7774048	0,187441
Cloud_Mesh_Distance_BA_R0_R1	21,67364311	-83,70184326	-940,7670898	0,213153
Cloud_Mesh_Distance_BA_R0_R1	21,70576859	-83,50469971	-940,673523	0,157111
Cloud_Mesh_Distance_BA_R0_R1	21,90057182	-83,45404053	-940,7219238	0,209816
Cloud_Mesh_Distance_BA_R0_R1	17,83245468	-83,29251099	-940,6974487	-0,077782
Cloud_Mesh_Distance_BA_R0_R1	17,66137505	-83,11434937	-940,6762695	-0,073984
Cloud_Mesh_Distance_BA_R0_R1	22,15622711	-83,11329651	-940,7147827	0,177692
Cloud_Mesh_Distance_BA_R0_R1	22,03252411	-83,31170654	-940,7193604	0,205992
Cloud_Mesh_Distance_BA_R0_R1	22,23274612	-83,30300903	-940,7824707	0,29535
Cloud_Mesh_Distance_BA_R0_R1	17,36168671	-82,81608582	-940,7574463	-0,05575
Cloud_Mesh_Distance_BA_R0_R1	17,51439095	-82,88752747	-940,5736694	-0,07156
Cloud_Mesh_Distance_BA_R0_R1	22,36892509	-83,07060242	-940,7998047	0,250715
Cloud_Mesh_Distance_BA_R0_R1	17,23484039	-82,73973083	-940,9155273	-0,041388
Cloud_Mesh_Distance_BA_R0_R1	22,45895195	-82,68057251	-940,8189697	0,113018
Cloud_Mesh_Distance_BA_R0_R1	22,35299873	-82,58010864	-940,7191773	0,037473
Cloud_Mesh_Distance_BA_R0_R1	22,52321053	-82,50154114	-940,8509522	0,062802
Cloud_Mesh_Distance_BA_R0_R1	22,61465073	-82,63031006	-940,934082	0,151949
Cloud_Mesh_Distance_BA_R0_R1	22,69275475	-82,42634583	-941,0065308	0,093966
Cloud_Mesh_Distance_BA_R0_R1	17,05109978	-82,27932739	-940,6766968	-0,032284
Cloud_Mesh_Distance_BA_R0_R1	17,04140091	-82,39117432	-940,8429565	-0,025567
Cloud_Mesh_Distance_BA_R0_R1	22,42977905	-82,37286377	-940,7336426	-0,01444
Cloud_Mesh_Distance_BA_R0_R1	22,58410645	-82,31072998	-940,8803711	0,007387
Cloud_Mesh_Distance_BA_R0_R1	22,70132446	-82,2482605	-941,0048218	0,024294
Cloud_Mesh_Distance_BA_R0_R1	22,77217293	-82,09799194	-941,0753784	-0,003482
Cloud_Mesh_Distance_BA_R0_R1	16,8321228	-81,97561646	-940,8469238	-0,000996
Cloud_Mesh_Distance_BA_R0_R1	16,74241638	-81,79122925	-940,8848877	0,012623
Cloud_Mesh_Distance_BA_R0_R1	22,64506149	-81,82051086	-940,7814331	-0,125988
Cloud_Mesh_Distance_BA_R0_R1	22,73836517	-81,69252014	-940,8619385	-0,13992

Cloud_Mesh_Distance_BA_R0_R1	22,72579002	-81,91317749	-940,9473877	-0,086275
Cloud_Mesh_Distance_BA_R0_R1	16,66013527	-81,40429688	-940,7872315	0,024296
Cloud_Mesh_Distance_BA_R0_R1	16,77980232	-81,67134094	-940,696228	0,003643
Cloud_Mesh_Distance_BA_R0_R1	22,93681908	-81,42956543	-941,1056519	-0,174244
Cloud_Mesh_Distance_BA_R0_R1	22,98456192	-81,2902832	-941,1403809	-0,195536
Cloud_Mesh_Distance_BA_R0_R1	23,00340652	-81,07185364	-941,0517578	-0,245187
Cloud_Mesh_Distance_BA_R0_R1	23,04778099	-81,20802307	-941,2854614	-0,20492
Cloud_Mesh_Distance_BA_R0_R1	16,57849312	-80,8739624	-940,7271729	0,039072
Cloud_Mesh_Distance_BA_R0_R1	23,0727253	-80,83093262	-941,137085	-0,266889
Cloud_Mesh_Distance_BA_R0_R1	16,47423553	-80,69937134	-940,8965454	0,064575
Cloud_Mesh_Distance_BA_R0_R1	23,12246895	-80,66867065	-941,2861939	-0,261283
Cloud_Mesh_Distance_BA_R0_R1	16,39183998	-80,43588257	-941,0078125	0,081921
Cloud_Mesh_Distance_BA_R0_R1	23,11992264	-80,4788208	-941,2549439	-0,238217
Cloud_Mesh_Distance_BA_R0_R1	23,09766006	-80,27333069	-941,2103272	-0,194818
Cloud_Mesh_Distance_BA_R0_R1	16,37443733	-80,01599121	-941,0106201	0,098167
Cloud_Mesh_Distance_BA_R0_R1	16,40493965	-80,19819641	-940,9572144	0,091569
Cloud_Mesh_Distance_BA_R0_R1	16,48024559	-80,27075195	-940,8179321	0,079998
Cloud_Mesh_Distance_BA_R0_R1	16,5548172	-80,12556458	-940,6759033	0,072142
Cloud_Mesh_Distance_BA_R0_R1	16,52662468	-79,94390869	-940,7402344	0,088278
Cloud_Mesh_Distance_BA_R0_R1	16,44796944	-79,8425293	-940,8911133	0,098358
Cloud_Mesh_Distance_BA_R0_R1	16,33797646	-79,79821777	-941,0866699	0,10274
Cloud_Mesh_Distance_BA_R0_R1	16,46393013	-80,0664978	-940,8464966	0,087592
Cloud_Mesh_Distance_BA_R0_R1	23,07035637	-80,06539917	-941,2421265	-0,153244
Cloud_Mesh_Distance_BA_R0_R1	23,01287842	-79,97996521	-941,0838013	-0,139407
Cloud_Mesh_Distance_BA_R0_R1	16,43303108	-79,51287842	-940,9732666	0,100844
Cloud_Mesh_Distance_BA_R0_R1	16,59213448	-79,27249146	-940,7888794	0,0927
Cloud_Mesh_Distance_BA_R0_R1	16,55312347	-79,08839417	-940,9164429	0,086201
Cloud_Mesh_Distance_BA_R0_R1	22,72891045	-79,15843201	-941,3197022	0,005236
Cloud_Mesh_Distance_BA_R0_R1	16,80071449	-78,3416748	-940,9533691	0,053503
Cloud_Mesh_Distance_BA_R0_R1	22,50697708	-78,7857666	-941,3362427	0,05266
Cloud_Mesh_Distance_BA_R0_R1	22,40865517	-78,71679688	-941,1548462	0,047773
Cloud_Mesh_Distance_BA_R0_R1	16,68531418	-78,2930603	-941,1212158	0,042187
Cloud_Mesh_Distance_BA_R0_R1	16,9542141	-78,16439819	-940,8868408	0,060616
Cloud_Mesh_Distance_BA_R0_R1	16,81240845	-78,17391968	-941,043335	0,047299
Cloud_Mesh_Distance_BA_R0_R1	21,91459656	-78,11697388	-941,2130737	0,061243
Cloud_Mesh_Distance_BA_R0_R1	22,08132553	-78,27470398	-941,2602539	0,067289
Cloud_Mesh_Distance_BA_R0_R1	22,19513512	-78,44352722	-941,1522217	0,06461
Cloud_Mesh_Distance_BA_R0_R1	17,06044769	-77,95671082	-940,9176025	0,063478
Cloud_Mesh_Distance_BA_R0_R1	16,8978653	-77,98626709	-941,0718994	0,046753
Cloud_Mesh_Distance_BA_R0_R1	17,15212631	-77,78433228	-940,958374	0,066533
Cloud_Mesh_Distance_BA_R0_R1	21,75755882	-77,99778748	-941,1228027	0,055803
Cloud_Mesh_Distance_BA_R0_R1	16,92826653	-77,80973816	-941,168457	0,045087
Cloud_Mesh_Distance_BA_R0_R1	17,01778984	-77,83818054	-941,0515747	0,056052
Cloud_Mesh_Distance_BA_R0_R1	21,61854172	-77,83544922	-941,2092896	0,048372
Cloud_Mesh_Distance_BA_R0_R1	17,30202293	-77,41375732	-941,1461792	0,073206
Cloud_Mesh_Distance_BA_R0_R1	17,41628456	-77,47854614	-940,9885864	0,085652
Cloud_Mesh_Distance_BA_R0_R1	17,4930439	-77,30567932	-941,0963745	0,089262
Cloud_Mesh_Distance_BA_R0_R1	21,30809402	-77,55258179	-941,24646	0,039048
Cloud_Mesh_Distance_BA_R0_R1	21,38881874	-77,56304932	-941,4158325	0,045325
Cloud_Mesh_Distance_BA_R0_R1	17,73069	-77,27232361	-940,9628906	0,104938

Cloud_Mesh_Distance_BA_R0_R1	21,14243126	-77,46130371	-941,1507568	0,031313
Cloud_Mesh_Distance_BA_R0_R1	21,06739426	-77,34899902	-941,2921753	0,033349
Cloud_Mesh_Distance_BA_R0_R1	18,19509697	-76,84649658	-941,2143555	0,112405
Cloud_Mesh_Distance_BA_R0_R1	18,41332626	-76,83021545	-941,1549683	0,109508
Cloud_Mesh_Distance_BA_R0_R1	18,4452095	-76,95799255	-940,9682617	0,111038
Cloud_Mesh_Distance_BA_R0_R1	20,38873672	-77,00744629	-941,1591187	0,039381
Cloud_Mesh_Distance_BA_R0_R1	20,77568245	-77,21127319	-941,1547241	0,029372
Cloud_Mesh_Distance_BA_R0_R1	20,89051819	-77,15710449	-941,4879761	0,03997
Cloud_Mesh_Distance_BA_R0_R1	18,00936699	-76,94511414	-941,1726074	0,111686
Cloud_Mesh_Distance_BA_R0_R1	18,99982834	-76,79318237	-941,0806274	0,095331
Cloud_Mesh_Distance_BA_R0_R1	19,29675293	-76,72084045	-941,2130737	0,077412
Cloud_Mesh_Distance_BA_R0_R1	19,50323486	-76,741745	-941,2097168	0,069495
Cloud_Mesh_Distance_BA_R0_R1	19,19665146	-76,79058838	-941,0774536	0,087634
Cloud_Mesh_Distance_BA_R0_R1	19,40177727	-76,80114746	-941,0735474	0,079779
Cloud_Mesh_Distance_BA_R0_R1	19,58159256	-76,81373596	-941,0853882	0,072822
Cloud_Mesh_Distance_BA_R0_R1	19,95651436	-76,83799744	-941,1964111	0,054359
Cloud_Mesh_Distance_BA_R0_R1	18,66808319	-76,77696228	-941,1618652	0,103915
Cloud_Mesh_Distance_BA_R0_R1	19,08590317	-76,71746826	-941,2084351	0,083892
Cloud_Mesh_Distance_BA_R0_R1	18,88273621	-76,72976685	-941,2001343	0,091608
Cloud_Mesh_Distance_BA_R0_R1	19,70516014	-76,77923584	-941,1973267	0,061011
Cloud_Mesh_Distance_BA_R0_R1	20,00905037	-84,01800537	-940,5374756	-0,114161
Cloud_Mesh_Distance_BA_R0_R1	20,73406792	-83,97845459	-940,6567993	0,009345
Cloud_Mesh_Distance_BA_R0_R1	19,24024391	-83,90420532	-940,4639282	-0,174256
Cloud_Mesh_Distance_BA_R0_R1	19,64612198	-84,02482605	-940,5461426	-0,144388
Cloud_Mesh_Distance_BA_R0_R1	19,83278465	-84,09356689	-940,6252441	-0,1123
Cloud_Mesh_Distance_BA_R0_R1	20,36939812	-83,89315796	-940,4598389	-0,088278
Cloud_Mesh_Distance_BA_R0_R1	18,57916451	-83,61157227	-940,3834229	-0,159684
Cloud_Mesh_Distance_BA_R0_R1	18,72257233	-83,76055908	-940,505127	-0,147592
Cloud_Mesh_Distance_BA_R0_R1	18,88381004	-83,87895203	-940,583374	-0,146742
Cloud_Mesh_Distance_BA_R0_R1	18,90924072	-83,77322388	-940,4129028	-0,172928
Cloud_Mesh_Distance_BA_R0_R1	19,04706001	-83,86322021	-940,477417	-0,168204
Cloud_Mesh_Distance_BA_R0_R1	20,71301651	-83,80802917	-940,4841309	-0,038882
Cloud_Mesh_Distance_BA_R0_R1	20,89422607	-83,85046387	-940,5906982	0,012013
Cloud_Mesh_Distance_BA_R0_R1	20,93479347	-83,66470337	-940,4454346	-0,022957
Cloud_Mesh_Distance_BA_R0_R1	21,09241486	-83,79112244	-940,6154785	0,046408
Cloud_Mesh_Distance_BA_R0_R1	18,38853455	-83,46125793	-940,3156128	-0,149018
Cloud_Mesh_Distance_BA_R0_R1	18,35732269	-83,69955444	-940,7185669	-0,096498
Cloud_Mesh_Distance_BA_R0_R1	21,53751755	-83,64901733	-940,6896973	0,143736
Cloud_Mesh_Distance_BA_R0_R1	21,4585247	-83,4954834	-940,5597534	0,071843
Cloud_Mesh_Distance_BA_R0_R1	18,06259537	-83,29490662	-940,3946533	-0,115208
Cloud_Mesh_Distance_BA_R0_R1	17,8528614	-83,17747498	-940,4812622	-0,096975
Cloud_Mesh_Distance_BA_R0_R1	17,96564293	-83,33404541	-940,5741577	-0,092898
Cloud_Mesh_Distance_BA_R0_R1	21,66172981	-83,30508423	-940,5484009	0,079194
Cloud_Mesh_Distance_BA_R0_R1	21,85474205	-83,3081665	-940,6430054	0,141046
Cloud_Mesh_Distance_BA_R0_R1	21,62845802	-83,13134766	-940,4370117	0,019438
Cloud_Mesh_Distance_BA_R0_R1	17,59640503	-82,84429932	-940,3803711	-0,092395
Cloud_Mesh_Distance_BA_R0_R1	21,91334724	-82,8923645	-940,499939	0,027151
Cloud_Mesh_Distance_BA_R0_R1	21,83344269	-83,1078949	-940,545166	0,066924
Cloud_Mesh_Distance_BA_R0_R1	21,98500252	-83,1656189	-940,6494751	0,134299
Cloud_Mesh_Distance_BA_R0_R1	22,04107666	-82,98912048	-940,6182251	0,091641

Cloud_Mesh_Distance_BA_R0_R1	17,24649239	-82,63645935	-940,7248535	-0,04489
Cloud_Mesh_Distance_BA_R0_R1	17,47862625	-82,71655273	-940,4011231	-0,078811
Cloud_Mesh_Distance_BA_R0_R1	17,36718178	-82,6978302	-940,5693359	-0,060206
Cloud_Mesh_Distance_BA_R0_R1	17,36676598	-82,5847168	-940,4240112	-0,068899
Cloud_Mesh_Distance_BA_R0_R1	22,2694416	-82,84191895	-940,716919	0,110462
Cloud_Mesh_Distance_BA_R0_R1	22,26011276	-82,43571472	-940,5927734	-0,029833
Cloud_Mesh_Distance_BA_R0_R1	22,16831207	-82,64163208	-940,5882568	0,012497
Cloud_Mesh_Distance_BA_R0_R1	17,1460247	-82,459198	-940,6941528	-0,039313
Cloud_Mesh_Distance_BA_R0_R1	17,1654129	-82,34373474	-940,512085	-0,048808
Cloud_Mesh_Distance_BA_R0_R1	16,85244942	-81,86630249	-940,6925659	-0,006735
Cloud_Mesh_Distance_BA_R0_R1	16,94050407	-82,08432007	-940,7059937	-0,017282
Cloud_Mesh_Distance_BA_R0_R1	22,50906944	-82,17675781	-940,7556763	-0,055165
Cloud_Mesh_Distance_BA_R0_R1	22,64729881	-82,12522888	-940,9086914	-0,041078
Cloud_Mesh_Distance_BA_R0_R1	22,58783913	-81,98674011	-940,7792969	-0,090052
Cloud_Mesh_Distance_BA_R0_R1	22,54027557	-81,83911133	-940,6453247	-0,126861
Cloud_Mesh_Distance_BA_R0_R1	16,87633133	-81,72859192	-940,5287476	-0,013959
Cloud_Mesh_Distance_BA_R0_R1	16,72999954	-81,50793457	-940,6932983	0,00997
Cloud_Mesh_Distance_BA_R0_R1	16,80323982	-81,53648376	-940,5521851	-0,005169
Cloud_Mesh_Distance_BA_R0_R1	16,63755608	-81,23883057	-940,7456055	0,025446
Cloud_Mesh_Distance_BA_R0_R1	16,72402382	-81,33355713	-940,604187	0,008052
Cloud_Mesh_Distance_BA_R0_R1	22,84755707	-81,51673889	-940,9621582	-0,170356
Cloud_Mesh_Distance_BA_R0_R1	22,91651535	-81,30215454	-940,9779053	-0,215574
Cloud_Mesh_Distance_BA_R0_R1	16,65662003	-81,10379028	-940,6427002	0,021314
Cloud_Mesh_Distance_BA_R0_R1	22,94949532	-80,90298462	-940,8327637	-0,262375
Cloud_Mesh_Distance_BA_R0_R1	22,87177467	-81,14382935	-940,791626	-0,236038
Cloud_Mesh_Distance_BA_R0_R1	16,6381321	-80,7063446	-940,5610962	0,033175
Cloud_Mesh_Distance_BA_R0_R1	23,0168457	-80,8875885	-940,9884644	-0,267376
Cloud_Mesh_Distance_BA_R0_R1	23,02194786	-80,73872375	-940,9450684	-0,273253
Cloud_Mesh_Distance_BA_R0_R1	16,55804062	-80,64785767	-940,7116699	0,052652
Cloud_Mesh_Distance_BA_R0_R1	22,94507408	-80,68634033	-940,7540283	-0,264401
Cloud_Mesh_Distance_BA_R0_R1	23,07909203	-80,43362427	-941,0826416	-0,233789
Cloud_Mesh_Distance_BA_R0_R1	23,08083344	-80,61158752	-941,0944824	-0,26531
Cloud_Mesh_Distance_BA_R0_R1	16,50997162	-80,47619629	-940,779419	0,065636
Cloud_Mesh_Distance_BA_R0_R1	16,54642677	-79,76896667	-940,7295532	0,090424
Cloud_Mesh_Distance_BA_R0_R1	16,62342262	-79,91975403	-940,5700073	0,073583
Cloud_Mesh_Distance_BA_R0_R1	23,05010223	-80,1449585	-941,0865479	-0,173917
Cloud_Mesh_Distance_BA_R0_R1	22,93493462	-79,90324402	-940,9140015	-0,128396
Cloud_Mesh_Distance_BA_R0_R1	16,48698235	-79,64079285	-940,855957	0,095695
Cloud_Mesh_Distance_BA_R0_R1	16,5665226	-79,60044861	-940,7304077	0,093294
Cloud_Mesh_Distance_BA_R0_R1	22,95227051	-79,75509644	-941,1201782	-0,095719
Cloud_Mesh_Distance_BA_R0_R1	16,5283699	-79,46224976	-940,8305054	0,096562
Cloud_Mesh_Distance_BA_R0_R1	22,85452271	-79,52416992	-941,1063843	-0,057452
Cloud_Mesh_Distance_BA_R0_R1	22,75837326	-79,31576538	-941,1193848	-0,023419
Cloud_Mesh_Distance_BA_R0_R1	22,65772629	-79,11734009	-941,1380005	0,004259
Cloud_Mesh_Distance_BA_R0_R1	16,68022346	-78,87147522	-940,8265991	0,078847
Cloud_Mesh_Distance_BA_R0_R1	16,66628456	-79,09396362	-940,7467041	0,084461
Cloud_Mesh_Distance_BA_R0_R1	22,48789978	-79,03627014	-940,8373413	0,001929
Cloud_Mesh_Distance_BA_R0_R1	22,54535675	-78,92138672	-941,1522217	0,029574
Cloud_Mesh_Distance_BA_R0_R1	22,57352066	-79,07723999	-940,9711304	0,002127
Cloud_Mesh_Distance_BA_R0_R1	16,78300095	-78,57373047	-940,8429565	0,065723

Cloud_Mesh_Distance_BA_R0_R1	22,46040154	-78,89190674	-940,9758301	0,030163
Cloud_Mesh_Distance_BA_R0_R1	22,32203674	-78,71337891	-940,9419556	0,047419
Cloud_Mesh_Distance_BA_R0_R1	22,28249931	-78,58425903	-941,0775757	0,058368
Cloud_Mesh_Distance_BA_R0_R1	16,9181118	-78,36993408	-940,7957153	0,065783
Cloud_Mesh_Distance_BA_R0_R1	22,30464745	-78,54946899	-941,2218018	0,065167
Cloud_Mesh_Distance_BA_R0_R1	21,98873138	-78,23783875	-941,097168	0,063194
Cloud_Mesh_Distance_BA_R0_R1	22,15042686	-78,50363159	-940,9254761	0,057049
Cloud_Mesh_Distance_BA_R0_R1	22,08460045	-78,35099792	-941,0740967	0,064079
Cloud_Mesh_Distance_BA_R0_R1	17,11493874	-78,07698059	-940,772522	0,071038
Cloud_Mesh_Distance_BA_R0_R1	17,2325592	-77,91204834	-940,7781982	0,076162
Cloud_Mesh_Distance_BA_R0_R1	21,86494446	-78,15310669	-941,0054932	0,061058
Cloud_Mesh_Distance_BA_R0_R1	17,25852394	-77,60108948	-941,0128784	0,073646
Cloud_Mesh_Distance_BA_R0_R1	17,29186249	-77,74514771	-940,8584595	0,077979
Cloud_Mesh_Distance_BA_R0_R1	17,37568474	-77,80218506	-940,7349854	0,087739
Cloud_Mesh_Distance_BA_R0_R1	21,61932945	-77,91003418	-941,0301514	0,046911
Cloud_Mesh_Distance_BA_R0_R1	17,44375992	-77,63673401	-940,8212891	0,089732
Cloud_Mesh_Distance_BA_R0_R1	21,47156334	-77,77642822	-941,0388184	0,038903
Cloud_Mesh_Distance_BA_R0_R1	21,44775009	-77,67547607	-941,2311401	0,043802
Cloud_Mesh_Distance_BA_R0_R1	21,3020668	-77,62129211	-941,0695801	0,033791
Cloud_Mesh_Distance_BA_R0_R1	17,59733009	-77,45155334	-940,8753662	0,096177
Cloud_Mesh_Distance_BA_R0_R1	21,13462448	-77,56016541	-940,9352417	0,028151
Cloud_Mesh_Distance_BA_R0_R1	17,86357307	-77,10700989	-941,0597534	0,109583
Cloud_Mesh_Distance_BA_R0_R1	17,94735146	-77,23362732	-940,8726196	0,111122
Cloud_Mesh_Distance_BA_R0_R1	20,9763279	-77,42233276	-940,9841919	0,024836
Cloud_Mesh_Distance_BA_R0_R1	20,95265007	-77,32713318	-941,1491699	0,029673
Cloud_Mesh_Distance_BA_R0_R1	18,2434063	-76,9564209	-941,0498047	0,113868
Cloud_Mesh_Distance_BA_R0_R1	18,08724213	-77,08895874	-940,9604492	0,112602
Cloud_Mesh_Distance_BA_R0_R1	18,56995583	-76,87768555	-941,0375977	0,109703
Cloud_Mesh_Distance_BA_R0_R1	18,52187538	-77,04322815	-940,8322754	0,11262
Cloud_Mesh_Distance_BA_R0_R1	18,62302589	-76,96020508	-940,9075928	0,111942
Cloud_Mesh_Distance_BA_R0_R1	18,81059837	-76,96020508	-940,8590698	0,103989
Cloud_Mesh_Distance_BA_R0_R1	18,91185951	-76,87191772	-940,9675903	0,101757
Cloud_Mesh_Distance_BA_R0_R1	19,18041229	-76,9349823	-940,8433228	0,092571
Cloud_Mesh_Distance_BA_R0_R1	19,10050201	-76,8653717	-940,9555664	0,094736
Cloud_Mesh_Distance_BA_R0_R1	19,29559898	-76,87327576	-940,9396973	0,088045
Cloud_Mesh_Distance_BA_R0_R1	20,19558525	-77,01824951	-940,982666	0,045052
Cloud_Mesh_Distance_BA_R0_R1	19,96445274	-76,94943237	-940,9754028	0,0549
Cloud_Mesh_Distance_BA_R0_R1	20,19244766	-76,91648865	-941,1872559	0,046717
Cloud_Mesh_Distance_BA_R0_R1	20,63381195	-77,20983887	-940,9934082	0,031508
Cloud_Mesh_Distance_BA_R0_R1	20,58731651	-77,1053009	-941,1546021	0,037654
Cloud_Mesh_Distance_BA_R0_R1	20,43183899	-77,11846924	-940,9754028	0,032611
Cloud_Mesh_Distance_BA_R0_R1	18,73114967	-76,87333679	-941,0011597	0,104782
Cloud_Mesh_Distance_BA_R0_R1	18,83449364	-76,8061676	-941,0814819	0,099772
Cloud_Mesh_Distance_BA_R0_R1	19,52928162	-76,89820862	-940,9223023	0,075773
Cloud_Mesh_Distance_BA_R0_R1	20,07915115	-76,93003845	-941,076416	0,050194
Cloud_Mesh_Distance_BA_R0_R1	19,75404358	-76,87232971	-941,0287476	0,065143
Cloud_Mesh_Distance_BA_R0_R1	19,45833397	-83,95716858	-940,4819336	-0,170783
Cloud_Mesh_Distance_BA_R0_R1	19,83308029	-83,96257019	-940,4597778	-0,142934
Cloud_Mesh_Distance_BA_R0_R1	18,77725029	-83,68257141	-940,3554077	-0,17576
Cloud_Mesh_Distance_BA_R0_R1	19,41787148	-83,81072998	-940,2960205	-0,190898

Cloud_Mesh_Distance_BA_R0_R1	19,85682678	-83,817276	-940,286377	-0,163713
Cloud_Mesh_Distance_BA_R0_R1	20,18203545	-83,92886353	-940,4574585	-0,110693
Cloud_Mesh_Distance_BA_R0_R1	19,99696922	-83,90802002	-940,4048462	-0,13505
Cloud_Mesh_Distance_BA_R0_R1	18,22535324	-83,42367554	-940,411438	-0,126147
Cloud_Mesh_Distance_BA_R0_R1	21,19749069	-83,59353638	-940,5053711	0,028123
Cloud_Mesh_Distance_BA_R0_R1	21,07275772	-83,46958923	-940,3526611	-0,030162
Cloud_Mesh_Distance_BA_R0_R1	21,47712708	-83,27867126	-940,4350586	0,019336
Cloud_Mesh_Distance_BA_R0_R1	21,28442001	-83,39201355	-940,40448	0,001812
Cloud_Mesh_Distance_BA_R0_R1	17,96641159	-83,04203796	-940,1618042	-0,129735
Cloud_Mesh_Distance_BA_R0_R1	21,4836483	-83,13256836	-940,350647	-0,01129
Cloud_Mesh_Distance_BA_R0_R1	17,69034958	-82,9942627	-940,4443359	-0,089358
Cloud_Mesh_Distance_BA_R0_R1	21,73517227	-82,96279907	-940,4157715	0,002915
Cloud_Mesh_Distance_BA_R0_R1	17,45017433	-82,54336548	-940,2456665	-0,091787
Cloud_Mesh_Distance_BA_R0_R1	22,07621574	-82,81269836	-940,5795898	0,042818
Cloud_Mesh_Distance_BA_R0_R1	17,26074409	-82,52737427	-940,5463257	-0,057122
Cloud_Mesh_Distance_BA_R0_R1	21,98188591	-82,69943237	-940,4664917	-0,013801
Cloud_Mesh_Distance_BA_R0_R1	22,06377029	-82,47799683	-940,435669	-0,050019
Cloud_Mesh_Distance_BA_R0_R1	17,35744476	-82,33303833	-940,1767578	-0,085629
Cloud_Mesh_Distance_BA_R0_R1	17,29533768	-82,42654419	-940,3665772	-0,065637
Cloud_Mesh_Distance_BA_R0_R1	17,18950272	-82,03883362	-940,1856079	-0,0701
Cloud_Mesh_Distance_BA_R0_R1	17,06360626	-82,14396667	-940,5025635	-0,038887
Cloud_Mesh_Distance_BA_R0_R1	16,96387291	-81,93301392	-940,5098267	-0,028612
Cloud_Mesh_Distance_BA_R0_R1	22,19124794	-82,31211853	-940,477478	-0,069333
Cloud_Mesh_Distance_BA_R0_R1	22,06595421	-82,27177429	-940,3414307	-0,087373
Cloud_Mesh_Distance_BA_R0_R1	22,21171379	-82,14585876	-940,4202271	-0,097543
Cloud_Mesh_Distance_BA_R0_R1	22,3436203	-82,21884155	-940,5887451	-0,072224
Cloud_Mesh_Distance_BA_R0_R1	22,4081192	-81,87150574	-940,4962769	-0,130656
Cloud_Mesh_Distance_BA_R0_R1	22,45558548	-82,02590942	-940,6298828	-0,100997
Cloud_Mesh_Distance_BA_R0_R1	16,88915253	-81,58636475	-940,4071045	-0,026009
Cloud_Mesh_Distance_BA_R0_R1	16,75498772	-81,1529541	-940,4575806	-0,000473
Cloud_Mesh_Distance_BA_R0_R1	22,6342411	-81,66365051	-940,6832275	-0,158828
Cloud_Mesh_Distance_BA_R0_R1	16,83831024	-81,37283325	-940,3898315	-0,017919
Cloud_Mesh_Distance_BA_R0_R1	22,6287384	-81,47480774	-940,5729981	-0,193267
Cloud_Mesh_Distance_BA_R0_R1	22,73898125	-81,5007019	-940,7557373	-0,178743
Cloud_Mesh_Distance_BA_R0_R1	22,82679558	-81,35379028	-940,8253174	-0,205946
Cloud_Mesh_Distance_BA_R0_R1	16,68825531	-80,93421936	-940,5175781	0,015687
Cloud_Mesh_Distance_BA_R0_R1	22,75574493	-81,0970459	-940,5716553	-0,234571
Cloud_Mesh_Distance_BA_R0_R1	22,85136223	-80,9670105	-940,6693726	-0,253964
Cloud_Mesh_Distance_BA_R0_R1	22,87844276	-80,8192749	-940,6633911	-0,259641
Cloud_Mesh_Distance_BA_R0_R1	16,68041611	-80,55754089	-940,4550781	0,027813
Cloud_Mesh_Distance_BA_R0_R1	16,67389107	-80,37283325	-940,4526978	0,039352
Cloud_Mesh_Distance_BA_R0_R1	22,84202003	-80,66990662	-940,5666504	-0,243976
Cloud_Mesh_Distance_BA_R0_R1	16,60255623	-80,49697876	-940,5988159	0,046697
Cloud_Mesh_Distance_BA_R0_R1	16,57751083	-80,31321716	-940,6322632	0,063563
Cloud_Mesh_Distance_BA_R0_R1	23,01599312	-80,50183105	-940,8904419	-0,251958
Cloud_Mesh_Distance_BA_R0_R1	22,9492321	-80,26190186	-940,7651367	-0,201834
Cloud_Mesh_Distance_BA_R0_R1	22,90533447	-80,4826355	-940,6551514	-0,231472
Cloud_Mesh_Distance_BA_R0_R1	23,04061699	-80,28839111	-940,987793	-0,203137
Cloud_Mesh_Distance_BA_R0_R1	22,99231148	-80,10441589	-940,9289551	-0,165601
Cloud_Mesh_Distance_BA_R0_R1	16,72288513	-79,76817322	-940,4296265	0,068577

Cloud_Mesh_Distance_BA_R0_R1	22,91618156	-80,0456543	-940,7772217	-0,159334
Cloud_Mesh_Distance_BA_R0_R1	16,7134819	-79,58535767	-940,493103	0,077993
Cloud_Mesh_Distance_BA_R0_R1	16,6341095	-79,68218994	-940,5975952	0,083475
Cloud_Mesh_Distance_BA_R0_R1	22,82466888	-79,85417175	-940,7053833	-0,126521
Cloud_Mesh_Distance_BA_R0_R1	22,76569557	-79,63020325	-940,7615356	-0,090191
Cloud_Mesh_Distance_BA_R0_R1	22,85822105	-79,68927002	-940,9145508	-0,093904
Cloud_Mesh_Distance_BA_R0_R1	16,71751213	-79,24102783	-940,6092529	0,083955
Cloud_Mesh_Distance_BA_R0_R1	16,76967239	-79,41641235	-940,4624023	0,076377
Cloud_Mesh_Distance_BA_R0_R1	16,64351463	-79,46298218	-940,6430664	0,089134
Cloud_Mesh_Distance_BA_R0_R1	22,56213188	-79,21105957	-940,7797852	-0,024798
Cloud_Mesh_Distance_BA_R0_R1	22,67098808	-79,2747345	-940,937561	-0,027618
Cloud_Mesh_Distance_BA_R0_R1	22,76790237	-79,47872925	-940,9207764	-0,06183
Cloud_Mesh_Distance_BA_R0_R1	16,8492775	-79,20974731	-940,4317627	0,076264
Cloud_Mesh_Distance_BA_R0_R1	16,80528259	-79,00582886	-940,5892944	0,081566
Cloud_Mesh_Distance_BA_R0_R1	16,92932129	-79,03489685	-940,4065552	0,070226
Cloud_Mesh_Distance_BA_R0_R1	16,86392403	-78,7479248	-940,6433106	0,074057
Cloud_Mesh_Distance_BA_R0_R1	16,95941353	-78,85360718	-940,4633789	0,070907
Cloud_Mesh_Distance_BA_R0_R1	17,00035858	-78,67796326	-940,5128784	0,071696
Cloud_Mesh_Distance_BA_R0_R1	22,36306763	-78,89459229	-940,7745972	0,015117
Cloud_Mesh_Distance_BA_R0_R1	22,0598278	-78,5553894	-940,6936646	0,040875
Cloud_Mesh_Distance_BA_R0_R1	17,08275604	-78,27174377	-940,671936	0,069352
Cloud_Mesh_Distance_BA_R0_R1	16,9783287	-78,51535034	-940,6353149	0,069073
Cloud_Mesh_Distance_BA_R0_R1	21,8164959	-78,23739624	-940,7693481	0,047411
Cloud_Mesh_Distance_BA_R0_R1	21,98637009	-78,31008911	-940,9412231	0,060376
Cloud_Mesh_Distance_BA_R0_R1	21,98967552	-78,40292358	-940,7911377	0,050071
Cloud_Mesh_Distance_BA_R0_R1	21,70292473	-78,04260254	-940,9167481	0,0469
Cloud_Mesh_Distance_BA_R0_R1	21,52419281	-77,90936279	-940,8670654	0,041393
Cloud_Mesh_Distance_BA_R0_R1	17,52059937	-77,78413391	-940,6289673	0,092111
Cloud_Mesh_Distance_BA_R0_R1	17,65995598	-77,61343384	-940,6761475	0,100424
Cloud_Mesh_Distance_BA_R0_R1	21,41958046	-77,93626404	-940,6825562	0,035255
Cloud_Mesh_Distance_BA_R0_R1	17,86348724	-77,42210388	-940,7258301	0,103928
Cloud_Mesh_Distance_BA_R0_R1	21,11064148	-77,63890076	-940,7706909	0,023415
Cloud_Mesh_Distance_BA_R0_R1	20,68814087	-77,3170166	-940,8550415	0,01961
Cloud_Mesh_Distance_BA_R0_R1	20,74779701	-77,42861938	-940,7315674	0,013898
Cloud_Mesh_Distance_BA_R0_R1	20,8378315	-77,39581299	-940,8712769	0,02254
Cloud_Mesh_Distance_BA_R0_R1	18,14112282	-77,22573853	-940,7785034	0,110551
Cloud_Mesh_Distance_BA_R0_R1	18,01666832	-77,31109619	-940,7510376	0,109967
Cloud_Mesh_Distance_BA_R0_R1	18,53974724	-77,15237427	-940,6914673	0,108
Cloud_Mesh_Distance_BA_R0_R1	18,3302784	-77,09579468	-940,8409424	0,112848
Cloud_Mesh_Distance_BA_R0_R1	20,59074211	-77,34860229	-940,720398	0,016191
Cloud_Mesh_Distance_BA_R0_R1	20,81269836	-77,30880737	-941,0007935	0,027781
Cloud_Mesh_Distance_BA_R0_R1	18,69115829	-77,056427	-940,7608643	0,107199
Cloud_Mesh_Distance_BA_R0_R1	19,00915527	-76,94966125	-940,8381958	0,0997
Cloud_Mesh_Distance_BA_R0_R1	19,34331131	-76,98112488	-940,7713013	0,085904
Cloud_Mesh_Distance_BA_R0_R1	19,76408005	-76,99499512	-940,81604	0,066699
Cloud_Mesh_Distance_BA_R0_R1	19,64394569	-83,89550781	-940,3795776	-0,171294
Cloud_Mesh_Distance_BA_R0_R1	19,64406013	-83,77723694	-940,2335815	-0,184562
Cloud_Mesh_Distance_BA_R0_R1	18,91832161	-83,67520142	-940,2719116	-0,192873
Cloud_Mesh_Distance_BA_R0_R1	19,11994362	-83,75683594	-940,3013916	-0,196489
Cloud_Mesh_Distance_BA_R0_R1	19,29426575	-83,66218567	-940,1314697	-0,204783

Cloud_Mesh_Distance_BA_R0_R1	19,52746773	-83,68231201	-940,1253662	-0,195123
Cloud_Mesh_Distance_BA_R0_R1	20,00379562	-83,68630981	-940,1591187	-0,155005
Cloud_Mesh_Distance_BA_R0_R1	19,76918602	-83,66140747	-940,1034546	-0,181168
Cloud_Mesh_Distance_BA_R0_R1	20,09261513	-83,80369568	-940,302063	-0,139394
Cloud_Mesh_Distance_BA_R0_R1	20,50219727	-83,78457642	-940,3885498	-0,0838
Cloud_Mesh_Distance_BA_R0_R1	20,29696274	-83,7913208	-940,3344116	-0,111303
Cloud_Mesh_Distance_BA_R0_R1	18,40390396	-83,34992981	-940,1533814	-0,16371
Cloud_Mesh_Distance_BA_R0_R1	18,57099533	-83,45895386	-940,177124	-0,170264
Cloud_Mesh_Distance_BA_R0_R1	18,77000237	-83,57684326	-940,2147217	-0,185729
Cloud_Mesh_Distance_BA_R0_R1	18,76804161	-83,43148804	-940,0310059	-0,198617
Cloud_Mesh_Distance_BA_R0_R1	18,53989601	-83,28842163	-939,9874878	-0,187809
Cloud_Mesh_Distance_BA_R0_R1	19,96327782	-83,55740356	-940,0194092	-0,161581
Cloud_Mesh_Distance_BA_R0_R1	20,12296677	-83,58447266	-940,0803833	-0,150526
Cloud_Mesh_Distance_BA_R0_R1	20,20726395	-83,6764679	-940,1934815	-0,135323
Cloud_Mesh_Distance_BA_R0_R1	20,42063713	-83,64785767	-940,2288208	-0,109929
Cloud_Mesh_Distance_BA_R0_R1	20,68077087	-83,61576843	-940,2957153	-0,078652
Cloud_Mesh_Distance_BA_R0_R1	20,5309124	-83,46965027	-940,1077271	-0,111518
Cloud_Mesh_Distance_BA_R0_R1	18,29417038	-83,19697571	-940,050293	-0,164907
Cloud_Mesh_Distance_BA_R0_R1	18,10285187	-83,17950439	-940,1948853	-0,137198
Cloud_Mesh_Distance_BA_R0_R1	18,24255943	-83,32209778	-940,2506714	-0,142463
Cloud_Mesh_Distance_BA_R0_R1	20,88643074	-83,50469971	-940,2911987	-0,057119
Cloud_Mesh_Distance_BA_R0_R1	20,76892853	-83,40795898	-940,1590576	-0,087893
Cloud_Mesh_Distance_BA_R0_R1	20,97133255	-83,35539246	-940,2155762	-0,060219
Cloud_Mesh_Distance_BA_R0_R1	21,156147	-83,29492188	-940,2684326	-0,041119
Cloud_Mesh_Distance_BA_R0_R1	21,21225739	-83,12081909	-940,1827393	-0,053939
Cloud_Mesh_Distance_BA_R0_R1	17,83336258	-83,03022766	-940,2984619	-0,110474
Cloud_Mesh_Distance_BA_R0_R1	17,96034241	-83,14813232	-940,3044434	-0,122116
Cloud_Mesh_Distance_BA_R0_R1	21,33677101	-83,21078491	-940,3133545	-0,023669
Cloud_Mesh_Distance_BA_R0_R1	21,56902313	-83,00024414	-940,3282471	-0,022106
Cloud_Mesh_Distance_BA_R0_R1	17,71338654	-82,88665771	-940,2700195	-0,106916
Cloud_Mesh_Distance_BA_R0_R1	17,83952713	-82,91316223	-940,1484985	-0,126609
Cloud_Mesh_Distance_BA_R0_R1	17,60448074	-82,7230835	-940,2225342	-0,09852
Cloud_Mesh_Distance_BA_R0_R1	21,80062485	-82,78359985	-940,371521	-0,026745
Cloud_Mesh_Distance_BA_R0_R1	21,65379524	-82,86291504	-940,3091431	-0,03287
Cloud_Mesh_Distance_BA_R0_R1	21,71486855	-82,47601318	-940,1525269	-0,08108
Cloud_Mesh_Distance_BA_R0_R1	21,65086937	-82,69847107	-940,22052	-0,053991
Cloud_Mesh_Distance_BA_R0_R1	21,85600853	-82,58805847	-940,3181152	-0,056307
Cloud_Mesh_Distance_BA_R0_R1	21,9076786	-82,39056396	-940,2628174	-0,083127
Cloud_Mesh_Distance_BA_R0_R1	17,08530807	-81,99534607	-940,3283691	-0,049037
Cloud_Mesh_Distance_BA_R0_R1	17,13121223	-81,86630249	-940,1551514	-0,066709
Cloud_Mesh_Distance_BA_R0_R1	17,29615974	-82,14337158	-940,1060791	-0,083215
Cloud_Mesh_Distance_BA_R0_R1	17,20168877	-82,20758057	-940,3082275	-0,063941
Cloud_Mesh_Distance_BA_R0_R1	17,25343132	-81,9659729	-940,0388184	-0,081578
Cloud_Mesh_Distance_BA_R0_R1	21,92816544	-82,21040344	-940,1901856	-0,100117
Cloud_Mesh_Distance_BA_R0_R1	22,07907486	-82,08300781	-940,2612305	-0,111022
Cloud_Mesh_Distance_BA_R0_R1	22,33461952	-82,04180908	-940,4978638	-0,106423
Cloud_Mesh_Distance_BA_R0_R1	17,07672882	-81,65550232	-940,1135864	-0,064093
Cloud_Mesh_Distance_BA_R0_R1	16,99108887	-81,7588501	-940,3238525	-0,037332
Cloud_Mesh_Distance_BA_R0_R1	22,20461845	-81,73745728	-940,2121582	-0,133738
Cloud_Mesh_Distance_BA_R0_R1	22,35275269	-81,71998596	-940,3565064	-0,144463

Cloud_Mesh_Distance_BA_R0_R1	17,0442543	-81,44348145	-940,0593872	-0,061768
Cloud_Mesh_Distance_BA_R0_R1	16,93220711	-81,29293823	-940,1804199	-0,038925
Cloud_Mesh_Distance_BA_R0_R1	16,95593262	-81,52075195	-940,2448731	-0,038902
Cloud_Mesh_Distance_BA_R0_R1	22,30887032	-81,55728149	-940,2324829	-0,155283
Cloud_Mesh_Distance_BA_R0_R1	22,50504494	-81,65545654	-940,5023804	-0,162975
Cloud_Mesh_Distance_BA_R0_R1	22,42698288	-81,5528717	-940,3569336	-0,166734
Cloud_Mesh_Distance_BA_R0_R1	22,74320984	-81,30574036	-940,6538086	-0,215633
Cloud_Mesh_Distance_BA_R0_R1	22,52346039	-81,46247864	-940,4275513	-0,18408
Cloud_Mesh_Distance_BA_R0_R1	16,84248734	-81,164505	-940,2926025	-0,022128
Cloud_Mesh_Distance_BA_R0_R1	16,9176712	-81,01832581	-940,114563	-0,042778
Cloud_Mesh_Distance_BA_R0_R1	16,79235649	-80,97462463	-940,3251343	-0,010972
Cloud_Mesh_Distance_BA_R0_R1	22,61147881	-81,2665863	-940,4465942	-0,208704
Cloud_Mesh_Distance_BA_R0_R1	16,74176407	-80,7338562	-940,3653565	0,006426
Cloud_Mesh_Distance_BA_R0_R1	16,83571053	-80,82598877	-940,2109985	-0,017068
Cloud_Mesh_Distance_BA_R0_R1	22,64794159	-81,02534485	-940,3943481	-0,22012
Cloud_Mesh_Distance_BA_R0_R1	22,64903259	-80,82824707	-940,3365479	-0,217866
Cloud_Mesh_Distance_BA_R0_R1	22,77321625	-80,87121582	-940,5115356	-0,242375
Cloud_Mesh_Distance_BA_R0_R1	22,73830414	-80,69367981	-940,4204102	-0,226903
Cloud_Mesh_Distance_BA_R0_R1	16,76609612	-80,24961853	-940,2896729	0,028145
Cloud_Mesh_Distance_BA_R0_R1	16,75456238	-80,49429321	-940,3118286	0,014987
Cloud_Mesh_Distance_BA_R0_R1	16,65679359	-80,16004944	-940,4866943	0,054219
Cloud_Mesh_Distance_BA_R0_R1	22,75841713	-80,47071838	-940,4262085	-0,205722
Cloud_Mesh_Distance_BA_R0_R1	16,73855019	-79,99716187	-940,3607178	0,051246
Cloud_Mesh_Distance_BA_R0_R1	22,8485775	-80,30517578	-940,5686646	-0,194467
Cloud_Mesh_Distance_BA_R0_R1	16,84011078	-79,82542419	-940,2303467	0,046801
Cloud_Mesh_Distance_BA_R0_R1	22,83930588	-80,10180664	-940,6053467	-0,168239
Cloud_Mesh_Distance_BA_R0_R1	16,80595398	-79,60423279	-940,3436279	0,066751
Cloud_Mesh_Distance_BA_R0_R1	16,90747452	-79,40867615	-940,265625	0,060543
Cloud_Mesh_Distance_BA_R0_R1	22,58064461	-79,52780151	-940,5250855	-0,078667
Cloud_Mesh_Distance_BA_R0_R1	22,68870163	-79,681427	-940,5829468	-0,100334
Cloud_Mesh_Distance_BA_R0_R1	22,4665184	-79,48832703	-940,3939819	-0,068074
Cloud_Mesh_Distance_BA_R0_R1	22,65585327	-79,4468689	-940,7171021	-0,06144
Cloud_Mesh_Distance_BA_R0_R1	22,38370705	-79,32525635	-940,402832	-0,048133
Cloud_Mesh_Distance_BA_R0_R1	22,4961834	-79,32009888	-940,5632935	-0,046619
Cloud_Mesh_Distance_BA_R0_R1	17,1185379	-78,71568298	-940,3516846	0,067728
Cloud_Mesh_Distance_BA_R0_R1	22,43505287	-79,09823608	-940,6723633	-0,010444
Cloud_Mesh_Distance_BA_R0_R1	17,09124374	-78,56292725	-940,4755859	0,068795
Cloud_Mesh_Distance_BA_R0_R1	17,23737526	-78,4901123	-940,3624268	0,073062
Cloud_Mesh_Distance_BA_R0_R1	22,24334526	-78,85221863	-940,6265869	0,014056
Cloud_Mesh_Distance_BA_R0_R1	22,32040405	-78,98553467	-940,6062012	-0,000748
Cloud_Mesh_Distance_BA_R0_R1	17,12703323	-78,42228699	-940,524292	0,072698
Cloud_Mesh_Distance_BA_R0_R1	21,95927238	-78,57504272	-940,5346069	0,033932
Cloud_Mesh_Distance_BA_R0_R1	22,09362984	-78,74008179	-940,5351563	0,018501
Cloud_Mesh_Distance_BA_R0_R1	22,21316528	-78,70529175	-940,74823	0,031108
Cloud_Mesh_Distance_BA_R0_R1	21,90607643	-78,42810059	-940,6348877	0,041686
Cloud_Mesh_Distance_BA_R0_R1	17,24931526	-78,28253174	-940,489502	0,074948
Cloud_Mesh_Distance_BA_R0_R1	17,24551773	-78,09738159	-940,6253662	0,077874
Cloud_Mesh_Distance_BA_R0_R1	17,40558624	-78,11778259	-940,4584351	0,084879
Cloud_Mesh_Distance_BA_R0_R1	17,55883026	-77,9465332	-940,4625855	0,090845
Cloud_Mesh_Distance_BA_R0_R1	17,38957214	-77,94104004	-940,6096191	0,086899

Cloud_Mesh_Distance_BA_R0_R1	17,69909096	-77,77680969	-940,5006714	0,097505
Cloud_Mesh_Distance_BA_R0_R1	21,60787201	-78,0692749	-940,7252808	0,042586
Cloud_Mesh_Distance_BA_R0_R1	21,66091156	-78,21005249	-940,5982056	0,044025
Cloud_Mesh_Distance_BA_R0_R1	17,85831642	-77,77268982	-940,3950806	0,09518
Cloud_Mesh_Distance_BA_R0_R1	17,85020447	-77,62712097	-940,5338135	0,100486
Cloud_Mesh_Distance_BA_R0_R1	20,99799156	-77,62869263	-940,6691284	0,013465
Cloud_Mesh_Distance_BA_R0_R1	21,3032608	-77,74957275	-940,8275757	0,030384
Cloud_Mesh_Distance_BA_R0_R1	21,14370346	-77,76324463	-940,6242065	0,020682
Cloud_Mesh_Distance_BA_R0_R1	21,28876305	-77,84922791	-940,6560669	0,02979
Cloud_Mesh_Distance_BA_R0_R1	18,12321663	-77,37931824	-940,6220703	0,104898
Cloud_Mesh_Distance_BA_R0_R1	18,01077652	-77,52230835	-940,5375977	0,103774
Cloud_Mesh_Distance_BA_R0_R1	18,44132996	-77,43026733	-940,4168091	0,096556
Cloud_Mesh_Distance_BA_R0_R1	20,75953484	-77,62258911	-940,4752808	0,004226
Cloud_Mesh_Distance_BA_R0_R1	20,9408493	-77,69316101	-940,5300903	0,009606
Cloud_Mesh_Distance_BA_R0_R1	18,28090477	-77,40075684	-940,5201416	0,104748
Cloud_Mesh_Distance_BA_R0_R1	20,2520256	-77,27062988	-940,6065064	0,028009
Cloud_Mesh_Distance_BA_R0_R1	20,33844757	-77,37966919	-940,5053711	0,015148
Cloud_Mesh_Distance_BA_R0_R1	20,5879879	-77,46929932	-940,5467529	0,008379
Cloud_Mesh_Distance_BA_R0_R1	20,95054817	-77,51327515	-940,7959595	0,020641
Cloud_Mesh_Distance_BA_R0_R1	20,83176231	-77,55453491	-940,621521	0,009509
Cloud_Mesh_Distance_BA_R0_R1	18,35057259	-77,27706909	-940,6223145	0,10785
Cloud_Mesh_Distance_BA_R0_R1	18,60135841	-77,28610229	-940,5128174	0,103092
Cloud_Mesh_Distance_BA_R0_R1	18,91854095	-77,06109619	-940,6984253	0,101052
Cloud_Mesh_Distance_BA_R0_R1	18,75558472	-77,16943359	-940,6003418	0,101481
Cloud_Mesh_Distance_BA_R0_R1	19,0835743	-77,10655212	-940,6103516	0,094144
Cloud_Mesh_Distance_BA_R0_R1	19,25299072	-77,09016418	-940,6166382	0,087785
Cloud_Mesh_Distance_BA_R0_R1	19,15889549	-77,18476868	-940,5007324	0,087791
Cloud_Mesh_Distance_BA_R0_R1	19,35326195	-77,17826843	-940,4967041	0,076707
Cloud_Mesh_Distance_BA_R0_R1	19,5464592	-77,1799469	-940,5031738	0,070032
Cloud_Mesh_Distance_BA_R0_R1	19,44946289	-77,08491516	-940,6255493	0,079654
Cloud_Mesh_Distance_BA_R0_R1	19,73580551	-77,2046814	-940,4995728	0,056108
Cloud_Mesh_Distance_BA_R0_R1	19,65254974	-77,09472656	-940,6356812	0,066271
Cloud_Mesh_Distance_BA_R0_R1	19,86737442	-77,1293335	-940,640625	0,055418
Cloud_Mesh_Distance_BA_R0_R1	19,9346714	-77,25308228	-940,489624	0,043323
Cloud_Mesh_Distance_BA_R0_R1	20,42577934	-77,30909729	-940,657898	0,019646
Cloud_Mesh_Distance_BA_R0_R1	20,50729179	-77,23974609	-940,8255005	0,024969
Cloud_Mesh_Distance_BA_R0_R1	19,13207054	-77,01853943	-940,7255249	0,095129
Cloud_Mesh_Distance_BA_R0_R1	19,54719543	-77,0052948	-940,751648	0,076195
Cloud_Mesh_Distance_BA_R0_R1	20,27542496	-77,16876221	-940,774292	0,032007
Cloud_Mesh_Distance_BA_R0_R1	20,02586555	-77,06997681	-940,7954102	0,049374
Cloud_Mesh_Distance_BA_R0_R1	20,08255005	-77,19584656	-940,6280518	0,039499
Cloud_Mesh_Distance_BA_R0_R1	19,25126648	-83,54753113	-940,0028687	-0,204587
Cloud_Mesh_Distance_BA_R0_R1	19,43005753	-83,57354736	-940,007019	-0,200424
Cloud_Mesh_Distance_BA_R0_R1	19,60949516	-83,58180237	-940,0091553	-0,193758
Cloud_Mesh_Distance_BA_R0_R1	19,02725601	-83,56788635	-940,0890503	-0,209765
Cloud_Mesh_Distance_BA_R0_R1	18,96187782	-83,38476563	-939,8969116	-0,200351
Cloud_Mesh_Distance_BA_R0_R1	19,35278511	-83,4649353	-939,8959961	-0,196507
Cloud_Mesh_Distance_BA_R0_R1	20,13610268	-83,46151733	-939,9677124	-0,143608
Cloud_Mesh_Distance_BA_R0_R1	20,2973938	-83,53262329	-940,0805664	-0,132887
Cloud_Mesh_Distance_BA_R0_R1	20,65032578	-83,25318909	-939,9838867	-0,101237

Cloud_Mesh_Distance_BA_R0_R1	20,88586044	-83,23344421	-940,0820313	-0,084038
Cloud_Mesh_Distance_BA_R0_R1	18,10409164	-82,90168762	-939,8859253	-0,165728
Cloud_Mesh_Distance_BA_R0_R1	18,26420784	-83,02302551	-939,8839722	-0,170535
Cloud_Mesh_Distance_BA_R0_R1	18,11334419	-83,04711914	-940,0275879	-0,157307
Cloud_Mesh_Distance_BA_R0_R1	20,82888794	-83,10806274	-939,9649658	-0,087277
Cloud_Mesh_Distance_BA_R0_R1	21,05309105	-83,21258545	-940,1557007	-0,063221
Cloud_Mesh_Distance_BA_R0_R1	17,73713875	-82,79946899	-940,1395264	-0,11937
Cloud_Mesh_Distance_BA_R0_R1	17,70595932	-82,67575073	-940,048584	-0,125899
Cloud_Mesh_Distance_BA_R0_R1	21,03510666	-83,05290222	-940,0375977	-0,075334
Cloud_Mesh_Distance_BA_R0_R1	21,39978981	-83,04095459	-940,2445679	-0,037636
Cloud_Mesh_Distance_BA_R0_R1	21,28389549	-82,90666199	-940,0923462	-0,063619
Cloud_Mesh_Distance_BA_R0_R1	21,50369072	-82,8722229	-940,2132568	-0,048532
Cloud_Mesh_Distance_BA_R0_R1	17,58304024	-82,57531738	-940,0978394	-0,111639
Cloud_Mesh_Distance_BA_R0_R1	17,67207146	-82,50427246	-939,9320679	-0,134019
Cloud_Mesh_Distance_BA_R0_R1	17,82685089	-82,65054321	-939,8997803	-0,144139
Cloud_Mesh_Distance_BA_R0_R1	21,45266151	-82,72370911	-940,0963135	-0,067859
Cloud_Mesh_Distance_BA_R0_R1	21,55714417	-82,58259583	-940,0915527	-0,074322
Cloud_Mesh_Distance_BA_R0_R1	17,50645638	-82,42062378	-940,0522461	-0,109601
Cloud_Mesh_Distance_BA_R0_R1	17,580019	-82,31735229	-939,8840332	-0,130059
Cloud_Mesh_Distance_BA_R0_R1	21,42756271	-82,58511353	-940,0042725	-0,074772
Cloud_Mesh_Distance_BA_R0_R1	17,22514534	-81,76187134	-939,9551392	-0,08843
Cloud_Mesh_Distance_BA_R0_R1	17,33314514	-81,90826416	-939,8973389	-0,102326
Cloud_Mesh_Distance_BA_R0_R1	21,80211067	-82,31387329	-940,1387329	-0,092432
Cloud_Mesh_Distance_BA_R0_R1	22,24199677	-81,93093872	-940,3446045	-0,128398
Cloud_Mesh_Distance_BA_R0_R1	17,15291214	-81,56504822	-939,9537964	-0,081545
Cloud_Mesh_Distance_BA_R0_R1	22,0709362	-81,73031616	-940,0862427	-0,12307
Cloud_Mesh_Distance_BA_R0_R1	17,15429497	-81,38867188	-939,8768921	-0,08569
Cloud_Mesh_Distance_BA_R0_R1	17,05576897	-81,01985168	-939,9013062	-0,065469
Cloud_Mesh_Distance_BA_R0_R1	22,51227951	-81,13275146	-940,2734375	-0,191626
Cloud_Mesh_Distance_BA_R0_R1	22,43358994	-81,36413574	-940,2787476	-0,175062
Cloud_Mesh_Distance_BA_R0_R1	22,55493927	-80,95487976	-940,2630005	-0,20232
Cloud_Mesh_Distance_BA_R0_R1	16,83247757	-80,63449097	-940,1870117	-0,007491
Cloud_Mesh_Distance_BA_R0_R1	16,84744644	-80,42211914	-940,1501465	-0,00028
Cloud_Mesh_Distance_BA_R0_R1	16,86903572	-80,24221802	-940,1216431	0,008506
Cloud_Mesh_Distance_BA_R0_R1	22,6358223	-80,63583374	-940,286377	-0,20162
Cloud_Mesh_Distance_BA_R0_R1	22,64839172	-80,32002258	-940,2929688	-0,17287
Cloud_Mesh_Distance_BA_R0_R1	22,74036789	-80,20809937	-940,4257813	-0,169875
Cloud_Mesh_Distance_BA_R0_R1	22,58879089	-80,12896729	-940,2543945	-0,143511
Cloud_Mesh_Distance_BA_R0_R1	22,69065094	-79,9331665	-940,4447022	-0,128147
Cloud_Mesh_Distance_BA_R0_R1	16,97694397	-79,76489258	-940,050354	0,027327
Cloud_Mesh_Distance_BA_R0_R1	16,90371323	-79,62715149	-940,1920776	0,049425
Cloud_Mesh_Distance_BA_R0_R1	22,48858643	-79,88087463	-940,2173462	-0,116674
Cloud_Mesh_Distance_BA_R0_R1	22,56317711	-79,69186401	-940,3931885	-0,098525
Cloud_Mesh_Distance_BA_R0_R1	17,00703239	-79,53691101	-940,0835571	0,041762
Cloud_Mesh_Distance_BA_R0_R1	17,06365204	-79,3203125	-940,0993652	0,04883
Cloud_Mesh_Distance_BA_R0_R1	17,16316414	-79,08407593	-940,09552	0,050797
Cloud_Mesh_Distance_BA_R0_R1	16,99015236	-79,16496277	-940,2630615	0,062565
Cloud_Mesh_Distance_BA_R0_R1	22,16302872	-79,09329224	-940,3118897	-0,018024
Cloud_Mesh_Distance_BA_R0_R1	22,33112717	-79,14387512	-940,4769898	-0,024073
Cloud_Mesh_Distance_BA_R0_R1	22,27385521	-79,25460815	-940,3204956	-0,038683

Cloud_Mesh_Distance_BA_R0_R1	17,22460556	-78,83953857	-940,1608276	0,058422
Cloud_Mesh_Distance_BA_R0_R1	17,05987549	-78,94723511	-940,2880249	0,067704
Cloud_Mesh_Distance_BA_R0_R1	17,40767288	-78,50080872	-940,1870117	0,066678
Cloud_Mesh_Distance_BA_R0_R1	17,27876091	-78,64968872	-940,2183228	0,06429
Cloud_Mesh_Distance_BA_R0_R1	22,20218086	-78,94839478	-940,4780273	-0,002145
Cloud_Mesh_Distance_BA_R0_R1	17,53606224	-78,3495636	-940,1759644	0,068923
Cloud_Mesh_Distance_BA_R0_R1	17,58733368	-78,50543213	-940,0272217	0,058153
Cloud_Mesh_Distance_BA_R0_R1	21,72260475	-78,53588867	-940,3183594	0,03041
Cloud_Mesh_Distance_BA_R0_R1	21,89546204	-78,67599487	-940,3655396	0,020525
Cloud_Mesh_Distance_BA_R0_R1	21,81228065	-78,4781189	-940,465332	0,035762
Cloud_Mesh_Distance_BA_R0_R1	17,69946671	-78,29425049	-940,085083	0,069363
Cloud_Mesh_Distance_BA_R0_R1	21,60385513	-78,33062744	-940,4021606	0,037805
Cloud_Mesh_Distance_BA_R0_R1	21,76387024	-78,33276367	-940,5706787	0,042057
Cloud_Mesh_Distance_BA_R0_R1	17,40293884	-78,3117218	-940,3200684	0,074524
Cloud_Mesh_Distance_BA_R0_R1	21,36190796	-78,1675415	-940,3466187	0,030521
Cloud_Mesh_Distance_BA_R0_R1	17,59388161	-78,14506531	-940,2782593	0,081489
Cloud_Mesh_Distance_BA_R0_R1	17,75040627	-77,93014526	-940,3318481	0,089899
Cloud_Mesh_Distance_BA_R0_R1	17,82493019	-78,08894348	-940,1500244	0,076373
Cloud_Mesh_Distance_BA_R0_R1	17,97859573	-77,868927	-940,2338257	0,085431
Cloud_Mesh_Distance_BA_R0_R1	21,13932228	-78,03726196	-940,2964478	0,02123
Cloud_Mesh_Distance_BA_R0_R1	21,2768631	-77,95536804	-940,5061646	0,026703
Cloud_Mesh_Distance_BA_R0_R1	21,49343109	-78,10647583	-940,5390625	0,041075
Cloud_Mesh_Distance_BA_R0_R1	18,01476669	-77,66934204	-940,3927612	0,097109
Cloud_Mesh_Distance_BA_R0_R1	18,20599556	-77,5271759	-940,4268799	0,100173
Cloud_Mesh_Distance_BA_R0_R1	18,18932724	-77,71286011	-940,2547607	0,089208
Cloud_Mesh_Distance_BA_R0_R1	20,93006706	-77,9185791	-940,2628784	0,015125
Cloud_Mesh_Distance_BA_R0_R1	21,05083656	-77,85137939	-940,4285889	0,01424
Cloud_Mesh_Distance_BA_R0_R1	18,38726044	-77,59829712	-940,2694092	0,086688
Cloud_Mesh_Distance_BA_R0_R1	18,57231331	-77,53024292	-940,2614746	0,086316
Cloud_Mesh_Distance_BA_R0_R1	18,69405556	-77,41503906	-940,3386231	0,089251
Cloud_Mesh_Distance_BA_R0_R1	20,40913391	-77,5	-940,3935547	0,010559
Cloud_Mesh_Distance_BA_R0_R1	20,8403759	-77,75814819	-940,3726196	0,00638
Cloud_Mesh_Distance_BA_R0_R1	19,16732407	-77,39204407	-940,2565918	0,070079
Cloud_Mesh_Distance_BA_R0_R1	19,3759346	-77,37008667	-940,265564	0,061484
Cloud_Mesh_Distance_BA_R0_R1	19,2586422	-77,27655029	-940,3786011	0,077556
Cloud_Mesh_Distance_BA_R0_R1	19,56485939	-77,35290527	-940,2919922	0,054795
Cloud_Mesh_Distance_BA_R0_R1	19,61872673	-77,26580811	-940,4012451	0,06039
Cloud_Mesh_Distance_BA_R0_R1	20,21508789	-77,43838501	-940,3681641	0,018038
Cloud_Mesh_Distance_BA_R0_R1	18,83202553	-77,28883362	-940,437439	0,094546
Cloud_Mesh_Distance_BA_R0_R1	18,96429443	-77,18147278	-940,5353394	0,094626
Cloud_Mesh_Distance_BA_R0_R1	19,05081367	-77,2858429	-940,3934326	0,082137
Cloud_Mesh_Distance_BA_R0_R1	19,45378113	-77,26821899	-940,3856201	0,066241
Cloud_Mesh_Distance_BA_R0_R1	20,13726997	-77,3145752	-940,4880981	0,030599
Cloud_Mesh_Distance_BA_R0_R1	19,15955925	-83,447052	-939,9092407	-0,202894
Cloud_Mesh_Distance_BA_R0_R1	19,1056633	-83,33311462	-939,8026123	-0,193374
Cloud_Mesh_Distance_BA_R0_R1	19,46333504	-83,36282349	-939,7860107	-0,177623
Cloud_Mesh_Distance_BA_R0_R1	19,54839897	-83,47885132	-939,8997803	-0,184098
Cloud_Mesh_Distance_BA_R0_R1	19,66474342	-83,36802673	-939,7974243	-0,171518
Cloud_Mesh_Distance_BA_R0_R1	19,7708149	-83,49493408	-939,928833	-0,173347
Cloud_Mesh_Distance_BA_R0_R1	19,86579514	-83,3480835	-939,8051758	-0,15603

Cloud_Mesh_Distance_BA_R0_R1	19,96816444	-83,43580627	-939,9035645	-0,155133
Cloud_Mesh_Distance_BA_R0_R1	20,0985775	-83,29989624	-939,817627	-0,141344
Cloud_Mesh_Distance_BA_R0_R1	20,35667229	-83,33934021	-939,9328003	-0,127768
Cloud_Mesh_Distance_BA_R0_R1	18,76217842	-83,26933289	-939,8521729	-0,194407
Cloud_Mesh_Distance_BA_R0_R1	19,17934799	-83,23629761	-939,6972046	-0,178857
Cloud_Mesh_Distance_BA_R0_R1	19,27020264	-83,35328674	-939,7919312	-0,18968
Cloud_Mesh_Distance_BA_R0_R1	20,28692436	-83,18533325	-939,7871704	-0,12179
Cloud_Mesh_Distance_BA_R0_R1	18,23808479	-82,85813904	-939,7501221	-0,176847
Cloud_Mesh_Distance_BA_R0_R1	18,42094612	-83,12924194	-939,8866577	-0,179953
Cloud_Mesh_Distance_BA_R0_R1	18,58807182	-83,13606262	-939,7996216	-0,187586
Cloud_Mesh_Distance_BA_R0_R1	20,13702011	-83,09356689	-939,673523	-0,118327
Cloud_Mesh_Distance_BA_R0_R1	20,53904724	-82,96592712	-939,7356567	-0,092272
Cloud_Mesh_Distance_BA_R0_R1	17,96466255	-82,77999878	-939,8890991	-0,156276
Cloud_Mesh_Distance_BA_R0_R1	17,83722115	-82,79092407	-940,0196533	-0,132587
Cloud_Mesh_Distance_BA_R0_R1	17,96788025	-82,91522217	-940,0171509	-0,146545
Cloud_Mesh_Distance_BA_R0_R1	20,48059464	-83,14112854	-939,828064	-0,107718
Cloud_Mesh_Distance_BA_R0_R1	20,72382545	-82,86517334	-939,7573853	-0,084335
Cloud_Mesh_Distance_BA_R0_R1	20,88121033	-82,9446106	-939,8847656	-0,08425
Cloud_Mesh_Distance_BA_R0_R1	20,6855545	-83,0484314	-939,8547974	-0,096639
Cloud_Mesh_Distance_BA_R0_R1	21,08699608	-82,83190918	-939,9295044	-0,076361
Cloud_Mesh_Distance_BA_R0_R1	20,91751289	-82,75212097	-939,789978	-0,075005
Cloud_Mesh_Distance_BA_R0_R1	18,18541718	-82,64845276	-939,62146	-0,175549
Cloud_Mesh_Distance_BA_R0_R1	18,09163094	-82,76821899	-939,7766113	-0,172621
Cloud_Mesh_Distance_BA_R0_R1	17,97678566	-82,64054871	-939,7612915	-0,164088
Cloud_Mesh_Distance_BA_R0_R1	21,11218643	-82,6307373	-939,8319702	-0,070748
Cloud_Mesh_Distance_BA_R0_R1	17,82915306	-82,50021362	-939,7774048	-0,157713
Cloud_Mesh_Distance_BA_R0_R1	17,82878494	-82,33906555	-939,6656494	-0,16145
Cloud_Mesh_Distance_BA_R0_R1	21,12482452	-82,44657898	-939,7476196	-0,058409
Cloud_Mesh_Distance_BA_R0_R1	21,28067017	-82,69589233	-939,9691773	-0,069211
Cloud_Mesh_Distance_BA_R0_R1	21,30984497	-82,49275208	-939,8809204	-0,072449
Cloud_Mesh_Distance_BA_R0_R1	17,71239281	-82,37709045	-939,7928467	-0,145465
Cloud_Mesh_Distance_BA_R0_R1	21,26333618	-82,34156799	-939,7793579	-0,061803
Cloud_Mesh_Distance_BA_R0_R1	21,47725296	-82,16537476	-939,8342896	-0,071097
Cloud_Mesh_Distance_BA_R0_R1	21,52998543	-82,43099976	-939,9950562	-0,07904
Cloud_Mesh_Distance_BA_R0_R1	21,41725731	-82,31185913	-939,8612061	-0,073173
Cloud_Mesh_Distance_BA_R0_R1	17,37550545	-82,06011963	-939,9398193	-0,101961
Cloud_Mesh_Distance_BA_R0_R1	17,43263435	-82,23704529	-939,9951782	-0,10854
Cloud_Mesh_Distance_BA_R0_R1	17,5174408	-82,13594055	-939,8258667	-0,131371
Cloud_Mesh_Distance_BA_R0_R1	17,45355415	-81,9591217	-939,7874756	-0,125148
Cloud_Mesh_Distance_BA_R0_R1	17,62609482	-82,01219177	-939,6459961	-0,145166
Cloud_Mesh_Distance_BA_R0_R1	17,67422295	-82,22276306	-939,7255249	-0,150245
Cloud_Mesh_Distance_BA_R0_R1	21,62186813	-82,04103088	-939,8770752	-0,080949
Cloud_Mesh_Distance_BA_R0_R1	21,58485794	-82,24050903	-939,9404297	-0,082209
Cloud_Mesh_Distance_BA_R0_R1	21,68186569	-82,32548523	-940,0519409	-0,088932
Cloud_Mesh_Distance_BA_R0_R1	21,76703072	-82,16485596	-940,0380249	-0,093091
Cloud_Mesh_Distance_BA_R0_R1	21,93165779	-82,02987671	-940,1055298	-0,107953
Cloud_Mesh_Distance_BA_R0_R1	21,79859161	-81,98420715	-939,9786987	-0,09645
Cloud_Mesh_Distance_BA_R0_R1	21,92187119	-81,8296814	-940,0067139	-0,106964
Cloud_Mesh_Distance_BA_R0_R1	22,07820892	-81,89349365	-940,1679688	-0,11587
Cloud_Mesh_Distance_BA_R0_R1	21,98060417	-81,60284424	-939,9609985	-0,111967

Cloud_Mesh_Distance_BA_R0_R1	22,15633965	-81,57225037	-940,0942993	-0,13277
Cloud_Mesh_Distance_BA_R0_R1	17,2965107	-81,56422424	-939,7687988	-0,105594
Cloud_Mesh_Distance_BA_R0_R1	21,83296204	-81,70092773	-939,8876343	-0,096873
Cloud_Mesh_Distance_BA_R0_R1	17,03596497	-81,22721863	-939,9884644	-0,068677
Cloud_Mesh_Distance_BA_R0_R1	22,26102448	-81,38006592	-940,1132202	-0,150856
Cloud_Mesh_Distance_BA_R0_R1	17,15711021	-81,18521118	-939,8054199	-0,085639
Cloud_Mesh_Distance_BA_R0_R1	22,36197853	-81,1836853	-940,1382446	-0,171568
Cloud_Mesh_Distance_BA_R0_R1	22,31469727	-81,03715515	-940,0524292	-0,163377
Cloud_Mesh_Distance_BA_R0_R1	16,99167633	-80,86294556	-939,9631348	-0,051154
Cloud_Mesh_Distance_BA_R0_R1	16,91268539	-80,76626587	-940,0705566	-0,032169
Cloud_Mesh_Distance_BA_R0_R1	17,17397881	-80,99360657	-939,7374878	-0,084698
Cloud_Mesh_Distance_BA_R0_R1	22,35853577	-80,87051392	-940,0524292	-0,16477
Cloud_Mesh_Distance_BA_R0_R1	22,43854523	-80,99594116	-940,1547852	-0,182326
Cloud_Mesh_Distance_BA_R0_R1	16,9991951	-80,68840027	-939,9291992	-0,042982
Cloud_Mesh_Distance_BA_R0_R1	16,92268944	-80,56373596	-940,0352173	-0,022757
Cloud_Mesh_Distance_BA_R0_R1	17,00597191	-80,5199585	-939,9099731	-0,034815
Cloud_Mesh_Distance_BA_R0_R1	22,2603817	-80,74304199	-939,9512329	-0,143409
Cloud_Mesh_Distance_BA_R0_R1	22,51031876	-80,79377747	-940,1789551	-0,186896
Cloud_Mesh_Distance_BA_R0_R1	17,08707237	-80,59790039	-939,8024902	-0,051353
Cloud_Mesh_Distance_BA_R0_R1	17,07515335	-80,39956665	-939,8197632	-0,03543
Cloud_Mesh_Distance_BA_R0_R1	22,39697266	-80,70071411	-940,0576172	-0,162209
Cloud_Mesh_Distance_BA_R0_R1	22,33722115	-80,52282715	-939,9923706	-0,143711
Cloud_Mesh_Distance_BA_R0_R1	16,94271469	-80,12402344	-940,024353	0,005323
Cloud_Mesh_Distance_BA_R0_R1	16,95735931	-80,34866333	-939,982605	-0,014294
Cloud_Mesh_Distance_BA_R0_R1	22,61522675	-80,46607971	-940,2510376	-0,182506
Cloud_Mesh_Distance_BA_R0_R1	22,49731064	-80,34463501	-940,1331177	-0,154231
Cloud_Mesh_Distance_BA_R0_R1	22,50417519	-80,57539368	-940,1431885	-0,171019
Cloud_Mesh_Distance_BA_R0_R1	16,84306335	-80,06526184	-940,1807251	0,027856
Cloud_Mesh_Distance_BA_R0_R1	17,01716805	-79,96530151	-939,9491577	0,005
Cloud_Mesh_Distance_BA_R0_R1	16,92129326	-79,95027161	-940,083252	0,022073
Cloud_Mesh_Distance_BA_R0_R1	22,3474884	-79,91015625	-940,0675049	-0,107418
Cloud_Mesh_Distance_BA_R0_R1	22,34863281	-79,76211548	-940,1193237	-0,094403
Cloud_Mesh_Distance_BA_R0_R1	22,32484818	-79,42730713	-940,2637329	-0,06369
Cloud_Mesh_Distance_BA_R0_R1	22,41140175	-79,63729858	-940,2415161	-0,087616
Cloud_Mesh_Distance_BA_R0_R1	17,29631615	-79,1157074	-939,9377441	0,034848
Cloud_Mesh_Distance_BA_R0_R1	22,14401627	-79,29782104	-940,1548462	-0,040172
Cloud_Mesh_Distance_BA_R0_R1	17,21268272	-79,27600098	-939,9476929	0,033939
Cloud_Mesh_Distance_BA_R0_R1	17,41166115	-79,04684448	-939,8621216	0,029583
Cloud_Mesh_Distance_BA_R0_R1	17,33516884	-78,94245911	-939,9901123	0,041251
Cloud_Mesh_Distance_BA_R0_R1	21,8775444	-78,83349609	-940,21698	0,009288
Cloud_Mesh_Distance_BA_R0_R1	22,03673935	-78,88815308	-940,3345947	0,001487
Cloud_Mesh_Distance_BA_R0_R1	17,41679382	-78,71032715	-940,0460205	0,052554
Cloud_Mesh_Distance_BA_R0_R1	17,52127647	-78,87045288	-939,8602905	0,032175
Cloud_Mesh_Distance_BA_R0_R1	21,73174667	-78,68771362	-940,1983643	0,024664
Cloud_Mesh_Distance_BA_R0_R1	21,55828667	-78,51791382	-940,1886597	0,031593
Cloud_Mesh_Distance_BA_R0_R1	21,57115936	-78,69799805	-940,0582886	0,027461
Cloud_Mesh_Distance_BA_R0_R1	17,76177216	-78,42584229	-939,9465942	0,051947
Cloud_Mesh_Distance_BA_R0_R1	21,42243385	-78,34454346	-940,2276611	0,031998
Cloud_Mesh_Distance_BA_R0_R1	17,91431046	-78,26435852	-939,9573364	0,062069
Cloud_Mesh_Distance_BA_R0_R1	21,20475388	-78,26002502	-940,1342163	0,031696

Cloud_Mesh_Distance_BA_R0_R1	18,03731537	-78,0504303	-940,0465698	0,069433
Cloud_Mesh_Distance_BA_R0_R1	18,51800728	-77,782547	-940,0415649	0,06323
Cloud_Mesh_Distance_BA_R0_R1	18,70061111	-77,73997498	-940,0162354	0,054161
Cloud_Mesh_Distance_BA_R0_R1	18,38428116	-77,89353943	-939,9995728	0,05957
Cloud_Mesh_Distance_BA_R0_R1	20,97779655	-78,09164429	-940,1259766	0,023869
Cloud_Mesh_Distance_BA_R0_R1	18,36946869	-77,75085449	-940,1321411	0,075895
Cloud_Mesh_Distance_BA_R0_R1	18,55534363	-77,65696716	-940,1436157	0,075301
Cloud_Mesh_Distance_BA_R0_R1	20,58548546	-77,66018677	-940,3115845	0,003375
Cloud_Mesh_Distance_BA_R0_R1	20,75354958	-77,82640076	-940,2371216	0,008039
Cloud_Mesh_Distance_BA_R0_R1	20,79275131	-77,96574402	-940,1231079	0,017792
Cloud_Mesh_Distance_BA_R0_R1	18,78402328	-77,56411743	-940,1574097	0,068159
Cloud_Mesh_Distance_BA_R0_R1	19,49641228	-77,44364929	-940,1866455	0,046995
Cloud_Mesh_Distance_BA_R0_R1	19,87969398	-77,4680481	-940,2145386	0,02945
Cloud_Mesh_Distance_BA_R0_R1	20,09254074	-77,51176453	-940,2320557	0,016435
Cloud_Mesh_Distance_BA_R0_R1	20,3122654	-77,57614136	-940,2545166	0,007446
Cloud_Mesh_Distance_BA_R0_R1	18,9324379	-77,40855408	-940,2788696	0,081211
Cloud_Mesh_Distance_BA_R0_R1	19,06466675	-77,53215027	-940,1253662	0,056878
Cloud_Mesh_Distance_BA_R0_R1	19,32768059	-77,50810242	-940,1207886	0,050867
Cloud_Mesh_Distance_BA_R0_R1	19,77441406	-77,33370972	-940,3449097	0,042439
Cloud_Mesh_Distance_BA_R0_R1	19,67174911	-77,4495697	-940,1956787	0,0407
Cloud_Mesh_Distance_BA_R0_R1	20,0043869	-77,37997437	-940,3532715	0,029654
Cloud_Mesh_Distance_BA_R0_R1	18,96737671	-83,22207642	-939,7321167	-0,189474
Cloud_Mesh_Distance_BA_R0_R1	19,28281212	-83,1265564	-939,5945435	-0,160157
Cloud_Mesh_Distance_BA_R0_R1	19,3688488	-83,24769592	-939,6870728	-0,16824
Cloud_Mesh_Distance_BA_R0_R1	19,44474411	-83,14353943	-939,6009522	-0,152504
Cloud_Mesh_Distance_BA_R0_R1	19,58808899	-83,07302856	-939,5558472	-0,140752
Cloud_Mesh_Distance_BA_R0_R1	19,75920677	-83,13569641	-939,6191406	-0,138094
Cloud_Mesh_Distance_BA_R0_R1	19,57827187	-83,233078	-939,6745606	-0,154926
Cloud_Mesh_Distance_BA_R0_R1	19,75955772	-83,26130676	-939,7156372	-0,148949
Cloud_Mesh_Distance_BA_R0_R1	19,92721939	-83,20144653	-939,6981812	-0,138184
Cloud_Mesh_Distance_BA_R0_R1	18,43228912	-82,97848511	-939,7374268	-0,183825
Cloud_Mesh_Distance_BA_R0_R1	18,76976395	-83,1116333	-939,6972046	-0,186488
Cloud_Mesh_Distance_BA_R0_R1	18,62079048	-83,00714111	-939,668396	-0,182972
Cloud_Mesh_Distance_BA_R0_R1	18,77712822	-82,93170166	-939,550415	-0,175643
Cloud_Mesh_Distance_BA_R0_R1	18,92010307	-83,06973267	-939,6135864	-0,179486
Cloud_Mesh_Distance_BA_R0_R1	19,09226418	-83,10403442	-939,6021729	-0,171466
Cloud_Mesh_Distance_BA_R0_R1	19,00549507	-82,95401001	-939,5080566	-0,160489
Cloud_Mesh_Distance_BA_R0_R1	19,93736649	-83,04063416	-939,5864868	-0,117897
Cloud_Mesh_Distance_BA_R0_R1	18,54813004	-82,85383606	-939,5796509	-0,175231
Cloud_Mesh_Distance_BA_R0_R1	20,35445976	-83,03608704	-939,7073975	-0,105217
Cloud_Mesh_Distance_BA_R0_R1	20,56315994	-82,79675293	-939,6464844	-0,077264
Cloud_Mesh_Distance_BA_R0_R1	20,94431686	-82,55863953	-939,7041626	-0,060876
Cloud_Mesh_Distance_BA_R0_R1	17,98483849	-82,48017883	-939,6384888	-0,165745
Cloud_Mesh_Distance_BA_R0_R1	20,98109818	-82,39248657	-939,6459961	-0,050444
Cloud_Mesh_Distance_BA_R0_R1	21,13251495	-82,25680542	-939,6694336	-0,051728
Cloud_Mesh_Distance_BA_R0_R1	21,32714272	-82,1965332	-939,7543335	-0,061066
Cloud_Mesh_Distance_BA_R0_R1	21,42591858	-82,0342865	-939,7476196	-0,063919
Cloud_Mesh_Distance_BA_R0_R1	21,23723412	-82,06396484	-939,6503906	-0,049384
Cloud_Mesh_Distance_BA_R0_R1	17,64952469	-81,81440735	-939,5283814	-0,144488
Cloud_Mesh_Distance_BA_R0_R1	17,51827621	-81,84646606	-939,6617432	-0,129382

Cloud_Mesh_Distance_BA_R0_R1	21,06097794	-82,10012817	-939,5718994	-0,039272
Cloud_Mesh_Distance_BA_R0_R1	21,53897858	-81,84085083	-939,7446289	-0,070216
Cloud_Mesh_Distance_BA_R0_R1	21,3384304	-81,89112854	-939,6452026	-0,052048
Cloud_Mesh_Distance_BA_R0_R1	17,43495941	-81,43597412	-939,5712891	-0,1169
Cloud_Mesh_Distance_BA_R0_R1	17,38199615	-81,78521729	-939,7728882	-0,116374
Cloud_Mesh_Distance_BA_R0_R1	21,40956116	-81,74479675	-939,6373901	-0,053125
Cloud_Mesh_Distance_BA_R0_R1	21,73711014	-81,84744263	-939,8760986	-0,089525
Cloud_Mesh_Distance_BA_R0_R1	21,68884468	-81,67744446	-939,7814941	-0,080762
Cloud_Mesh_Distance_BA_R0_R1	21,65527534	-81,49107361	-939,7037354	-0,073077
Cloud_Mesh_Distance_BA_R0_R1	21,52742958	-81,63848877	-939,6709595	-0,06153
Cloud_Mesh_Distance_BA_R0_R1	21,8214283	-81,53729248	-939,822937	-0,094386
Cloud_Mesh_Distance_BA_R0_R1	21,80527878	-81,31547546	-939,7492065	-0,090807
Cloud_Mesh_Distance_BA_R0_R1	22,09210205	-81,40446472	-939,9783325	-0,130158
Cloud_Mesh_Distance_BA_R0_R1	17,28151894	-81,3276825	-939,6968384	-0,102992
Cloud_Mesh_Distance_BA_R0_R1	17,27701759	-81,128479	-939,6451416	-0,098339
Cloud_Mesh_Distance_BA_R0_R1	17,40496254	-81,23513794	-939,5385742	-0,109221
Cloud_Mesh_Distance_BA_R0_R1	21,94325447	-81,4241333	-939,8719482	-0,109892
Cloud_Mesh_Distance_BA_R0_R1	22,18888664	-81,17970276	-939,9858398	-0,145288
Cloud_Mesh_Distance_BA_R0_R1	21,99945259	-81,24853516	-939,8615112	-0,115846
Cloud_Mesh_Distance_BA_R0_R1	21,8953228	-81,11706543	-939,7612915	-0,098482
Cloud_Mesh_Distance_BA_R0_R1	22,04860687	-81,03492737	-939,8461304	-0,122339
Cloud_Mesh_Distance_BA_R0_R1	17,20270729	-80,84338379	-939,6784668	-0,080263
Cloud_Mesh_Distance_BA_R0_R1	22,08995628	-80,81114197	-939,8360596	-0,121039
Cloud_Mesh_Distance_BA_R0_R1	22,21325493	-80,94815063	-939,9490967	-0,141002
Cloud_Mesh_Distance_BA_R0_R1	17,09513855	-80,81430054	-939,8102417	-0,063488
Cloud_Mesh_Distance_BA_R0_R1	17,18845367	-80,6791687	-939,6808472	-0,06888
Cloud_Mesh_Distance_BA_R0_R1	17,30300713	-80,56033325	-939,5529175	-0,068847
Cloud_Mesh_Distance_BA_R0_R1	17,17882919	-80,48220825	-939,6901245	-0,055033
Cloud_Mesh_Distance_BA_R0_R1	22,14958382	-80,5965271	-939,8547363	-0,121737
Cloud_Mesh_Distance_BA_R0_R1	17,06494522	-80,16592407	-939,8529053	-0,01708
Cloud_Mesh_Distance_BA_R0_R1	17,26418114	-80,37399292	-939,6013794	-0,060923
Cloud_Mesh_Distance_BA_R0_R1	17,19901276	-80,06536865	-939,7109985	-0,031532
Cloud_Mesh_Distance_BA_R0_R1	17,17647552	-80,28027344	-939,7056274	-0,040977
Cloud_Mesh_Distance_BA_R0_R1	22,02848244	-80,24957275	-939,770752	-0,095112
Cloud_Mesh_Distance_BA_R0_R1	22,04081726	-80,46150208	-939,7748413	-0,106188
Cloud_Mesh_Distance_BA_R0_R1	22,18700409	-80,3888855	-939,874939	-0,119565
Cloud_Mesh_Distance_BA_R0_R1	22,33167839	-80,30963135	-939,9874268	-0,130722
Cloud_Mesh_Distance_BA_R0_R1	22,39613152	-80,10662842	-940,0666504	-0,128785
Cloud_Mesh_Distance_BA_R0_R1	22,21752357	-80,18222046	-939,9080811	-0,112485
Cloud_Mesh_Distance_BA_R0_R1	22,09371758	-80,03717041	-939,8364868	-0,092429
Cloud_Mesh_Distance_BA_R0_R1	17,21076393	-79,84365845	-939,7476807	-0,012447
Cloud_Mesh_Distance_BA_R0_R1	17,09536552	-79,80558777	-939,8889771	0,005714
Cloud_Mesh_Distance_BA_R0_R1	17,11550713	-79,97120667	-939,8253784	-0,009972
Cloud_Mesh_Distance_BA_R0_R1	17,34003067	-79,6434021	-939,6781616	-0,01432
Cloud_Mesh_Distance_BA_R0_R1	22,24158287	-79,98841858	-939,9574585	-0,101526
Cloud_Mesh_Distance_BA_R0_R1	22,23416519	-79,79968262	-940,0022583	-0,089294
Cloud_Mesh_Distance_BA_R0_R1	22,23529816	-79,57620239	-940,092041	-0,069561
Cloud_Mesh_Distance_BA_R0_R1	17,078228	-79,64279175	-939,9574585	0,023305
Cloud_Mesh_Distance_BA_R0_R1	17,18532562	-79,640625	-939,8352661	0,007941
Cloud_Mesh_Distance_BA_R0_R1	17,13336945	-79,47341919	-939,9527588	0,026623

Cloud_Mesh_Distance_BA_R0_R1	22,06622314	-79,45297241	-939,9989624	-0,045778
Cloud_Mesh_Distance_BA_R0_R1	21,91361237	-79,46875	-939,8706665	-0,030959
Cloud_Mesh_Distance_BA_R0_R1	22,06950951	-79,67269897	-939,911377	-0,061517
Cloud_Mesh_Distance_BA_R0_R1	17,27070427	-79,44537354	-939,8161011	0,012692
Cloud_Mesh_Distance_BA_R0_R1	21,94051933	-79,26635742	-939,9885864	-0,02034
Cloud_Mesh_Distance_BA_R0_R1	21,76298141	-79,28396606	-939,8439941	-0,006269
Cloud_Mesh_Distance_BA_R0_R1	21,95870209	-79,04394531	-940,1405029	-0,009259
Cloud_Mesh_Distance_BA_R0_R1	21,75859642	-79,07655334	-939,9519653	0,002516
Cloud_Mesh_Distance_BA_R0_R1	17,58428192	-78,68299866	-939,916687	0,044941
Cloud_Mesh_Distance_BA_R0_R1	21,30771446	-78,8008728	-939,8053589	0,045233
Cloud_Mesh_Distance_BA_R0_R1	21,74321938	-78,870224	-940,0717773	0,014367
Cloud_Mesh_Distance_BA_R0_R1	21,15686226	-78,64608765	-939,8101807	0,049749
Cloud_Mesh_Distance_BA_R0_R1	21,37196541	-78,64814758	-939,9480591	0,039165
Cloud_Mesh_Distance_BA_R0_R1	21,46918678	-78,58248901	-940,0665283	0,031568
Cloud_Mesh_Distance_BA_R0_R1	18,10878372	-78,3470459	-939,7853394	0,034328
Cloud_Mesh_Distance_BA_R0_R1	18,30124855	-78,30912781	-939,7178955	0,022438
Cloud_Mesh_Distance_BA_R0_R1	21,17645836	-78,46289063	-939,9500732	0,04183
Cloud_Mesh_Distance_BA_R0_R1	21,36934471	-78,46723938	-940,0827026	0,033461
Cloud_Mesh_Distance_BA_R0_R1	18,10651588	-78,17608643	-939,9094238	0,051369
Cloud_Mesh_Distance_BA_R0_R1	18,26584244	-78,18367004	-939,8232422	0,040587
Cloud_Mesh_Distance_BA_R0_R1	20,72093391	-78,19239807	-939,8823242	0,035732
Cloud_Mesh_Distance_BA_R0_R1	18,19495583	-77,88224792	-940,0997925	0,074308
Cloud_Mesh_Distance_BA_R0_R1	18,23157692	-78,0357666	-939,9528198	0,059108
Cloud_Mesh_Distance_BA_R0_R1	18,42375565	-78,0382843	-939,8641968	0,038799
Cloud_Mesh_Distance_BA_R0_R1	18,57875252	-77,89099121	-939,925293	0,047171
Cloud_Mesh_Distance_BA_R0_R1	18,7962265	-77,87019348	-939,8751221	0,030108
Cloud_Mesh_Distance_BA_R0_R1	19,00745392	-77,86444092	-939,8330689	0,018936
Cloud_Mesh_Distance_BA_R0_R1	19,19292641	-77,87380981	-939,8001099	0,011443
Cloud_Mesh_Distance_BA_R0_R1	19,13835716	-77,76916504	-939,8931885	0,025837
Cloud_Mesh_Distance_BA_R0_R1	19,3502388	-77,88212585	-939,782959	0,003798
Cloud_Mesh_Distance_BA_R0_R1	20,54813576	-78,10067749	-939,8665772	0,02821
Cloud_Mesh_Distance_BA_R0_R1	20,43272018	-77,92835999	-939,9588623	0,012984
Cloud_Mesh_Distance_BA_R0_R1	20,60374832	-77,8500824	-940,121582	0,007523
Cloud_Mesh_Distance_BA_R0_R1	20,6541729	-78,02005005	-939,9909058	0,021624
Cloud_Mesh_Distance_BA_R0_R1	20,8218708	-78,10766602	-940,0107422	0,027808
Cloud_Mesh_Distance_BA_R0_R1	19,31005859	-77,77076721	-939,8765869	0,018138
Cloud_Mesh_Distance_BA_R0_R1	18,95947647	-77,70678711	-939,979126	0,044081
Cloud_Mesh_Distance_BA_R0_R1	19,7598629	-77,82688904	-939,84729	0,007802
Cloud_Mesh_Distance_BA_R0_R1	19,65075874	-77,68157959	-939,9622192	0,017802
Cloud_Mesh_Distance_BA_R0_R1	20,00738716	-77,8494873	-939,8763428	0,00809
Cloud_Mesh_Distance_BA_R0_R1	20,21450424	-77,79721069	-939,9868774	0,007625
Cloud_Mesh_Distance_BA_R0_R1	19,43024826	-77,65774536	-939,9735718	0,027964
Cloud_Mesh_Distance_BA_R0_R1	19,20766258	-77,65167236	-939,9910278	0,035347
Cloud_Mesh_Distance_BA_R0_R1	19,55618286	-77,54794312	-940,0825195	0,033405
Cloud_Mesh_Distance_BA_R0_R1	19,766222	-77,56939697	-940,085144	0,023685
Cloud_Mesh_Distance_BA_R0_R1	19,86823463	-77,70018005	-939,9780273	0,01064
Cloud_Mesh_Distance_BA_R0_R1	20,1731205	-77,64836121	-940,1164551	0,009633
Cloud_Mesh_Distance_BA_R0_R1	19,97210121	-77,59611511	-940,1035156	0,017612
Cloud_Mesh_Distance_BA_R0_R1	20,04843903	-77,71231079	-940,0119019	0,010509
Cloud_Mesh_Distance_BA_R0_R1	20,38945198	-77,73574829	-940,1216431	0,003735

Cloud_Mesh_Distance_BA_R0_R1	19,12860489	-82,82067871	-939,4050293	-0,13959
Cloud_Mesh_Distance_BA_R0_R1	19,21094704	-82,98103333	-939,4973755	-0,15095
Cloud_Mesh_Distance_BA_R0_R1	19,4091835	-83,0151062	-939,5103149	-0,143443
Cloud_Mesh_Distance_BA_R0_R1	19,57626534	-82,90397644	-939,4484253	-0,122187
Cloud_Mesh_Distance_BA_R0_R1	19,94695091	-82,87046814	-939,4873657	-0,101539
Cloud_Mesh_Distance_BA_R0_R1	19,76289558	-82,97422791	-939,5123901	-0,120179
Cloud_Mesh_Distance_BA_R0_R1	18,36487007	-82,8057251	-939,6327515	-0,177211
Cloud_Mesh_Distance_BA_R0_R1	18,6781292	-82,69664001	-939,4257202	-0,155512
Cloud_Mesh_Distance_BA_R0_R1	19,06105995	-82,65203857	-939,321167	-0,124754
Cloud_Mesh_Distance_BA_R0_R1	19,22936249	-82,69416809	-939,3293457	-0,120828
Cloud_Mesh_Distance_BA_R0_R1	19,35802269	-82,82797241	-939,3974609	-0,130396
Cloud_Mesh_Distance_BA_R0_R1	19,96623039	-82,68612671	-939,3979492	-0,082666
Cloud_Mesh_Distance_BA_R0_R1	20,08767891	-82,94090271	-939,5618897	-0,10228
Cloud_Mesh_Distance_BA_R0_R1	20,25731277	-82,9181366	-939,5994873	-0,093476
Cloud_Mesh_Distance_BA_R0_R1	20,41979027	-82,89678955	-939,6450806	-0,088748
Cloud_Mesh_Distance_BA_R0_R1	20,38288879	-82,75973511	-939,5557861	-0,072974
Cloud_Mesh_Distance_BA_R0_R1	18,43025589	-82,66885376	-939,5032349	-0,166823
Cloud_Mesh_Distance_BA_R0_R1	18,51980591	-82,48979187	-939,361084	-0,149992
Cloud_Mesh_Distance_BA_R0_R1	18,91726875	-82,78067017	-939,4143066	-0,148178
Cloud_Mesh_Distance_BA_R0_R1	18,86639214	-82,58972168	-939,3187256	-0,13609
Cloud_Mesh_Distance_BA_R0_R1	19,76937866	-82,78923035	-939,4100342	-0,103433
Cloud_Mesh_Distance_BA_R0_R1	20,00455284	-82,52084351	-939,3358154	-0,063793
Cloud_Mesh_Distance_BA_R0_R1	20,15816116	-82,76416016	-939,4856567	-0,082243
Cloud_Mesh_Distance_BA_R0_R1	20,56004333	-82,60945129	-939,5510864	-0,059429
Cloud_Mesh_Distance_BA_R0_R1	20,74862099	-82,67346191	-939,6644287	-0,067229
Cloud_Mesh_Distance_BA_R0_R1	18,12632751	-82,44020081	-939,5195923	-0,163697
Cloud_Mesh_Distance_BA_R0_R1	18,30155945	-82,48379517	-939,4507446	-0,16056
Cloud_Mesh_Distance_BA_R0_R1	18,37459564	-82,35134888	-939,3451538	-0,1488
Cloud_Mesh_Distance_BA_R0_R1	20,38239288	-82,35838318	-939,3815308	-0,040444
Cloud_Mesh_Distance_BA_R0_R1	20,33508492	-82,55801392	-939,4448242	-0,058002
Cloud_Mesh_Distance_BA_R0_R1	20,54925728	-82,40686035	-939,4595337	-0,042538
Cloud_Mesh_Distance_BA_R0_R1	17,81339645	-82,1505127	-939,5652466	-0,159397
Cloud_Mesh_Distance_BA_R0_R1	17,98584938	-82,29623413	-939,5239868	-0,163328
Cloud_Mesh_Distance_BA_R0_R1	18,18946075	-82,2754364	-939,3925781	-0,149689
Cloud_Mesh_Distance_BA_R0_R1	18,35886955	-82,19236755	-939,2761231	-0,135669
Cloud_Mesh_Distance_BA_R0_R1	20,66003036	-82,07618713	-939,3875732	-0,021601
Cloud_Mesh_Distance_BA_R0_R1	20,77640533	-82,44859314	-939,5723267	-0,048112
Cloud_Mesh_Distance_BA_R0_R1	20,92521477	-82,24609375	-939,5601196	-0,039056
Cloud_Mesh_Distance_BA_R0_R1	20,71864319	-82,2388916	-939,4647217	-0,032764
Cloud_Mesh_Distance_BA_R0_R1	17,79840279	-81,9473877	-939,4735107	-0,153328
Cloud_Mesh_Distance_BA_R0_R1	17,99856567	-82,07414246	-939,3994751	-0,154212
Cloud_Mesh_Distance_BA_R0_R1	20,87675095	-82,02908325	-939,4629517	-0,025857
Cloud_Mesh_Distance_BA_R0_R1	21,12746239	-81,89607239	-939,5370483	-0,035087
Cloud_Mesh_Distance_BA_R0_R1	17,58252144	-81,50193787	-939,460022	-0,128824
Cloud_Mesh_Distance_BA_R0_R1	20,93126106	-81,75013733	-939,4078369	-0,019912
Cloud_Mesh_Distance_BA_R0_R1	21,27835083	-81,75856018	-939,5714722	-0,041959
Cloud_Mesh_Distance_BA_R0_R1	21,15448189	-81,66511536	-939,4852905	-0,030029
Cloud_Mesh_Distance_BA_R0_R1	17,50120735	-81,6582489	-939,5922852	-0,123449
Cloud_Mesh_Distance_BA_R0_R1	17,7307148	-81,45733643	-939,333313	-0,126414
Cloud_Mesh_Distance_BA_R0_R1	21,35393143	-81,58947754	-939,5629883	-0,043905

Cloud_Mesh_Distance_BA_R0_R1	21,50144386	-81,48175049	-939,612854	-0,055579
Cloud_Mesh_Distance_BA_R0_R1	21,56656265	-81,28894043	-939,6034546	-0,061515
Cloud_Mesh_Distance_BA_R0_R1	21,39313698	-81,39871216	-939,5371704	-0,045513
Cloud_Mesh_Distance_BA_R0_R1	17,38665199	-80,87324524	-939,4871826	-0,092883
Cloud_Mesh_Distance_BA_R0_R1	17,38827705	-81,05230713	-939,5128784	-0,101985
Cloud_Mesh_Distance_BA_R0_R1	21,33638	-81,20013428	-939,4700317	-0,043371
Cloud_Mesh_Distance_BA_R0_R1	21,72503853	-81,08766174	-939,6522827	-0,078102
Cloud_Mesh_Distance_BA_R0_R1	17,28281212	-80,94709778	-939,6025391	-0,089085
Cloud_Mesh_Distance_BA_R0_R1	17,28812981	-80,77026367	-939,5758667	-0,080767
Cloud_Mesh_Distance_BA_R0_R1	21,52397346	-81,04298401	-939,5368042	-0,060073
Cloud_Mesh_Distance_BA_R0_R1	17,38510132	-80,70727539	-939,4747925	-0,082768
Cloud_Mesh_Distance_BA_R0_R1	17,47110558	-80,55032349	-939,3966065	-0,082185
Cloud_Mesh_Distance_BA_R0_R1	21,96741295	-80,64122009	-939,7377319	-0,10364
Cloud_Mesh_Distance_BA_R0_R1	21,76270103	-80,65585327	-939,618103	-0,082511
Cloud_Mesh_Distance_BA_R0_R1	21,88175392	-80,88945007	-939,7130127	-0,095896
Cloud_Mesh_Distance_BA_R0_R1	21,65556335	-80,86624146	-939,5812988	-0,073606
Cloud_Mesh_Distance_BA_R0_R1	17,2987175	-80,19146729	-939,5861206	-0,048667
Cloud_Mesh_Distance_BA_R0_R1	17,3806572	-80,35562134	-939,4880981	-0,066588
Cloud_Mesh_Distance_BA_R0_R1	21,72945213	-80,29237366	-939,5897827	-0,068729
Cloud_Mesh_Distance_BA_R0_R1	21,86378288	-80,42225647	-939,6652222	-0,085354
Cloud_Mesh_Distance_BA_R0_R1	17,35357857	-79,9337616	-939,5831299	-0,039179
Cloud_Mesh_Distance_BA_R0_R1	21,69525528	-80,13165283	-939,579895	-0,061516
Cloud_Mesh_Distance_BA_R0_R1	21,84237671	-80,19732666	-939,6587524	-0,075518
Cloud_Mesh_Distance_BA_R0_R1	21,79484749	-80,03936768	-939,6456299	-0,060663
Cloud_Mesh_Distance_BA_R0_R1	21,82428169	-79,87838745	-939,6882935	-0,058297
Cloud_Mesh_Distance_BA_R0_R1	21,93172264	-80,04566956	-939,727478	-0,072666
Cloud_Mesh_Distance_BA_R0_R1	17,62491608	-79,77430725	-939,4024048	-0,049537
Cloud_Mesh_Distance_BA_R0_R1	17,64476776	-79,60014343	-939,442627	-0,039268
Cloud_Mesh_Distance_BA_R0_R1	17,49446106	-79,7288208	-939,5158081	-0,035704
Cloud_Mesh_Distance_BA_R0_R1	21,99110031	-79,865448	-939,7984619	-0,068205
Cloud_Mesh_Distance_BA_R0_R1	22,1300869	-79,87554932	-939,8962402	-0,08043
Cloud_Mesh_Distance_BA_R0_R1	17,4011116	-79,42179871	-939,701294	-0,000345
Cloud_Mesh_Distance_BA_R0_R1	17,50909424	-79,50817871	-939,5774536	-0,021497
Cloud_Mesh_Distance_BA_R0_R1	17,67988777	-79,38626099	-939,4961548	-0,027373
Cloud_Mesh_Distance_BA_R0_R1	21,7346344	-79,48022461	-939,7424316	-0,018125
Cloud_Mesh_Distance_BA_R0_R1	21,87155151	-79,67645264	-939,767334	-0,044592
Cloud_Mesh_Distance_BA_R0_R1	21,66254044	-79,70092773	-939,6312256	-0,028956
Cloud_Mesh_Distance_BA_R0_R1	17,37631989	-79,23629761	-939,8029175	0,017337
Cloud_Mesh_Distance_BA_R0_R1	17,73408318	-79,13504028	-939,5658569	-0,011326
Cloud_Mesh_Distance_BA_R0_R1	17,53414726	-79,29057312	-939,6431274	-0,006573
Cloud_Mesh_Distance_BA_R0_R1	21,44717407	-79,24766541	-939,65802	0,02417
Cloud_Mesh_Distance_BA_R0_R1	17,54191208	-79,07743835	-939,7354126	0,012594
Cloud_Mesh_Distance_BA_R0_R1	17,96006012	-79,09928894	-939,4464111	-0,030404
Cloud_Mesh_Distance_BA_R0_R1	21,58101273	-79,12940979	-939,7954712	0,015395
Cloud_Mesh_Distance_BA_R0_R1	21,618927	-79,31401062	-939,7340088	0,004269
Cloud_Mesh_Distance_BA_R0_R1	17,67611313	-78,74862671	-939,8068237	0,03068
Cloud_Mesh_Distance_BA_R0_R1	17,70038795	-78,90534973	-939,7026978	0,01483
Cloud_Mesh_Distance_BA_R0_R1	17,85554123	-78,70394897	-939,7094727	0,020588
Cloud_Mesh_Distance_BA_R0_R1	21,33615494	-79,00909424	-939,7045898	0,042775
Cloud_Mesh_Distance_BA_R0_R1	17,72523689	-78,58996582	-939,8656006	0,042664

Cloud_Mesh_Distance_BA_R0_R1	18,06012344	-78,55752563	-939,6757813	0,017219
Cloud_Mesh_Distance_BA_R0_R1	21,12783432	-78,82382202	-939,6855469	0,059733
Cloud_Mesh_Distance_BA_R0_R1	20,96051025	-78,65777588	-939,6896973	0,062879
Cloud_Mesh_Distance_BA_R0_R1	21,52981186	-78,88699341	-939,8999023	0,025777
Cloud_Mesh_Distance_BA_R0_R1	17,89952087	-78,48065186	-939,817688	0,041112
Cloud_Mesh_Distance_BA_R0_R1	20,98141479	-78,48933411	-939,8098755	0,054137
Cloud_Mesh_Distance_BA_R0_R1	18,44929314	-78,19274902	-939,7381592	0,020372
Cloud_Mesh_Distance_BA_R0_R1	18,44690514	-78,35562134	-939,62854	0,003055
Cloud_Mesh_Distance_BA_R0_R1	20,95628548	-78,27926636	-939,9508667	0,038639
Cloud_Mesh_Distance_BA_R0_R1	20,79120445	-78,37210083	-939,7854004	0,049803
Cloud_Mesh_Distance_BA_R0_R1	18,78014565	-78,12644958	-939,6813355	0,000573
Cloud_Mesh_Distance_BA_R0_R1	19,0716114	-77,99082947	-939,723877	0,002134
Cloud_Mesh_Distance_BA_R0_R1	19,54132652	-78,10896301	-939,6124878	-0,006757
Cloud_Mesh_Distance_BA_R0_R1	20,2245369	-78,09393311	-939,7401733	0,020757
Cloud_Mesh_Distance_BA_R0_R1	20,38364792	-78,09448242	-939,7974854	0,023059
Cloud_Mesh_Distance_BA_R0_R1	18,64669418	-78,04600525	-939,7785034	0,020226
Cloud_Mesh_Distance_BA_R0_R1	18,87525368	-77,99986267	-939,7527466	0,011511
Cloud_Mesh_Distance_BA_R0_R1	19,2600956	-77,98934937	-939,7044678	-0,001479
Cloud_Mesh_Distance_BA_R0_R1	19,44650078	-77,97662354	-939,7077637	-0,001189
Cloud_Mesh_Distance_BA_R0_R1	19,51710701	-77,81817627	-939,8347168	0,009495
Cloud_Mesh_Distance_BA_R0_R1	19,64663315	-77,9662323	-939,7230225	0,000163
Cloud_Mesh_Distance_BA_R0_R1	19,86055374	-77,98083496	-939,7379761	0,003388
Cloud_Mesh_Distance_BA_R0_R1	20,223629	-77,95692444	-939,8493652	0,011524
Cloud_Mesh_Distance_BA_R0_R1	20,06505013	-78,02748108	-939,7442627	0,012337
Cloud_Mesh_Distance_BA_R0_R1	19,21792984	-82,52133179	-939,2491455	-0,102962
Cloud_Mesh_Distance_BA_R0_R1	19,57308388	-82,70797729	-939,345459	-0,104719
Cloud_Mesh_Distance_BA_R0_R1	19,38733864	-82,62593079	-939,2961426	-0,104608
Cloud_Mesh_Distance_BA_R0_R1	18,64863014	-82,38195801	-939,2666016	-0,127773
Cloud_Mesh_Distance_BA_R0_R1	18,70712662	-82,51356506	-939,3153076	-0,134759
Cloud_Mesh_Distance_BA_R0_R1	19,13802528	-82,35400391	-939,1849976	-0,091845
Cloud_Mesh_Distance_BA_R0_R1	19,01595116	-82,47592163	-939,2427979	-0,107824
Cloud_Mesh_Distance_BA_R0_R1	19,55888939	-82,50613403	-939,2557983	-0,082114
Cloud_Mesh_Distance_BA_R0_R1	19,39203835	-82,47010803	-939,2302246	-0,085826
Cloud_Mesh_Distance_BA_R0_R1	19,49114799	-82,33322144	-939,1860352	-0,072866
Cloud_Mesh_Distance_BA_R0_R1	19,7195797	-82,31286621	-939,2071533	-0,057104
Cloud_Mesh_Distance_BA_R0_R1	19,79266167	-82,55947876	-939,309021	-0,077792
Cloud_Mesh_Distance_BA_R0_R1	20,18091011	-82,3986969	-939,3347168	-0,048703
Cloud_Mesh_Distance_BA_R0_R1	20,12848091	-82,58843994	-939,3944702	-0,06645
Cloud_Mesh_Distance_BA_R0_R1	18,51257896	-82,29656982	-939,2680054	-0,130719
Cloud_Mesh_Distance_BA_R0_R1	18,68053818	-82,20349121	-939,184082	-0,108863
Cloud_Mesh_Distance_BA_R0_R1	18,99028969	-82,3271637	-939,1842041	-0,097285
Cloud_Mesh_Distance_BA_R0_R1	18,82828522	-82,3883667	-939,2307739	-0,11734
Cloud_Mesh_Distance_BA_R0_R1	19,07608795	-82,20626831	-939,1351929	-0,082671
Cloud_Mesh_Distance_BA_R0_R1	19,22275352	-82,23123169	-939,1401367	-0,072947
Cloud_Mesh_Distance_BA_R0_R1	19,30828857	-82,34802246	-939,1817627	-0,078467
Cloud_Mesh_Distance_BA_R0_R1	19,57839012	-82,18251038	-939,147644	-0,054111
Cloud_Mesh_Distance_BA_R0_R1	19,95855713	-82,35644531	-939,2649536	-0,050548
Cloud_Mesh_Distance_BA_R0_R1	20,51984978	-82,21670532	-939,3800049	-0,028753
Cloud_Mesh_Distance_BA_R0_R1	19,9241066	-82,14059448	-939,1907349	-0,036188
Cloud_Mesh_Distance_BA_R0_R1	20,13027763	-82,18563843	-939,2512207	-0,03368

Cloud_Mesh_Distance_BA_R0_R1	20,32813454	-82,20361328	-939,3120117	-0,028795
Cloud_Mesh_Distance_BA_R0_R1	17,78878021	-81,77612305	-939,4053345	-0,143987
Cloud_Mesh_Distance_BA_R0_R1	17,88113022	-81,62553406	-939,2897339	-0,130074
Cloud_Mesh_Distance_BA_R0_R1	18,08997345	-81,91940308	-939,281189	-0,137587
Cloud_Mesh_Distance_BA_R0_R1	18,07881355	-81,73629761	-939,2194824	-0,126657
Cloud_Mesh_Distance_BA_R0_R1	17,93723297	-81,8480835	-939,3377075	-0,145072
Cloud_Mesh_Distance_BA_R0_R1	18,23740387	-81,8692627	-939,1958618	-0,120873
Cloud_Mesh_Distance_BA_R0_R1	18,2064476	-82,06015015	-939,2827759	-0,139779
Cloud_Mesh_Distance_BA_R0_R1	19,73124886	-82,08351135	-939,1410523	-0,041845
Cloud_Mesh_Distance_BA_R0_R1	20,47893524	-82,00898743	-939,3034668	-0,017108
Cloud_Mesh_Distance_BA_R0_R1	20,27614021	-82,04492188	-939,2507324	-0,021001
Cloud_Mesh_Distance_BA_R0_R1	20,70259094	-81,88226318	-939,3500366	-0,015258
Cloud_Mesh_Distance_BA_R0_R1	20,73130035	-81,65600586	-939,3068848	-0,012839
Cloud_Mesh_Distance_BA_R0_R1	20,5301075	-81,76751709	-939,2613525	-0,012085
Cloud_Mesh_Distance_BA_R0_R1	17,68597603	-81,64091492	-939,4274902	-0,137765
Cloud_Mesh_Distance_BA_R0_R1	18,07721519	-81,48400879	-939,1436157	-0,101509
Cloud_Mesh_Distance_BA_R0_R1	17,78729248	-81,23666382	-939,2382202	-0,1056
Cloud_Mesh_Distance_BA_R0_R1	17,88824081	-81,41705322	-939,2215576	-0,11217
Cloud_Mesh_Distance_BA_R0_R1	20,56608963	-81,53713989	-939,2255859	-0,011507
Cloud_Mesh_Distance_BA_R0_R1	20,88694763	-81,55636597	-939,3444824	-0,016079
Cloud_Mesh_Distance_BA_R0_R1	20,76759529	-81,44364929	-939,2782593	-0,015127
Cloud_Mesh_Distance_BA_R0_R1	21,02952957	-81,55657959	-939,4037476	-0,022677
Cloud_Mesh_Distance_BA_R0_R1	17,57645416	-81,30244446	-939,4042969	-0,115815
Cloud_Mesh_Distance_BA_R0_R1	20,97538757	-81,37660217	-939,3445435	-0,021395
Cloud_Mesh_Distance_BA_R0_R1	21,19811821	-81,43266296	-939,4506836	-0,031349
Cloud_Mesh_Distance_BA_R0_R1	21,13332176	-81,23944092	-939,3861084	-0,030845
Cloud_Mesh_Distance_BA_R0_R1	17,49749756	-81,133255	-939,4295044	-0,1053
Cloud_Mesh_Distance_BA_R0_R1	17,63293839	-81,08381653	-939,3119507	-0,102207
Cloud_Mesh_Distance_BA_R0_R1	17,50147629	-80,96899414	-939,3960571	-0,094031
Cloud_Mesh_Distance_BA_R0_R1	20,8282032	-81,26324463	-939,269165	-0,018496
Cloud_Mesh_Distance_BA_R0_R1	20,79815292	-81,07688904	-939,2335205	-0,023801
Cloud_Mesh_Distance_BA_R0_R1	20,98318863	-81,14874268	-939,3104858	-0,025234
Cloud_Mesh_Distance_BA_R0_R1	17,49451828	-80,77540588	-939,382019	-0,087279
Cloud_Mesh_Distance_BA_R0_R1	17,63213348	-80,87202454	-939,282959	-0,094147
Cloud_Mesh_Distance_BA_R0_R1	17,61657143	-80,66619873	-939,2816773	-0,086606
Cloud_Mesh_Distance_BA_R0_R1	21,17033577	-81,04351807	-939,3716431	-0,036683
Cloud_Mesh_Distance_BA_R0_R1	20,99251556	-80,90158081	-939,2844238	-0,031254
Cloud_Mesh_Distance_BA_R0_R1	21,35438728	-80,95672607	-939,4418335	-0,047452
Cloud_Mesh_Distance_BA_R0_R1	21,49073219	-80,85997009	-939,4950562	-0,059135
Cloud_Mesh_Distance_BA_R0_R1	17,74321938	-80,75500488	-939,1989136	-0,086582
Cloud_Mesh_Distance_BA_R0_R1	17,62951279	-80,46440125	-939,2761231	-0,081528
Cloud_Mesh_Distance_BA_R0_R1	21,37970734	-80,77825928	-939,4343262	-0,052417
Cloud_Mesh_Distance_BA_R0_R1	17,53077888	-80,32006836	-939,3630371	-0,073819
Cloud_Mesh_Distance_BA_R0_R1	17,66201782	-80,29751587	-939,2681274	-0,073421
Cloud_Mesh_Distance_BA_R0_R1	17,76815224	-80,38278198	-939,1914063	-0,076249
Cloud_Mesh_Distance_BA_R0_R1	21,29916	-80,59750366	-939,3848877	-0,04626
Cloud_Mesh_Distance_BA_R0_R1	21,53174019	-80,66087341	-939,4977417	-0,061212
Cloud_Mesh_Distance_BA_R0_R1	17,44793892	-80,14059448	-939,4568481	-0,06043
Cloud_Mesh_Distance_BA_R0_R1	17,6278019	-80,11965942	-939,3192139	-0,068279
Cloud_Mesh_Distance_BA_R0_R1	17,77359581	-80,20837402	-939,2097168	-0,073577

Cloud_Mesh_Distance_BA_R0_R1	21,09656525	-80,33200073	-939,2940064	-0,033241
Cloud_Mesh_Distance_BA_R0_R1	21,26310921	-80,35423279	-939,3623657	-0,041764
Cloud_Mesh_Distance_BA_R0_R1	21,45813942	-80,41943359	-939,4506836	-0,054559
Cloud_Mesh_Distance_BA_R0_R1	21,39927864	-80,19450378	-939,4268799	-0,042992
Cloud_Mesh_Distance_BA_R0_R1	21,58200645	-80,25848389	-939,5131836	-0,056059
Cloud_Mesh_Distance_BA_R0_R1	21,65704918	-80,45162964	-939,5507813	-0,067848
Cloud_Mesh_Distance_BA_R0_R1	17,70303535	-79,90962219	-939,3127441	-0,060837
Cloud_Mesh_Distance_BA_R0_R1	21,6734581	-79,94302368	-939,5894775	-0,047421
Cloud_Mesh_Distance_BA_R0_R1	17,53549576	-79,93182373	-939,4284058	-0,055474
Cloud_Mesh_Distance_BA_R0_R1	21,36762238	-79,97219849	-939,4291992	-0,024458
Cloud_Mesh_Distance_BA_R0_R1	21,50855446	-79,84896851	-939,5159912	-0,025176
Cloud_Mesh_Distance_BA_R0_R1	21,54010963	-80,05950928	-939,5043335	-0,044383
Cloud_Mesh_Distance_BA_R0_R1	17,77241325	-79,7179718	-939,3214111	-0,056659
Cloud_Mesh_Distance_BA_R0_R1	17,90624428	-79,88626099	-939,199585	-0,067267
Cloud_Mesh_Distance_BA_R0_R1	21,22393608	-79,81845093	-939,3849487	-0,001295
Cloud_Mesh_Distance_BA_R0_R1	21,44616318	-79,63148499	-939,5298462	-0,004251
Cloud_Mesh_Distance_BA_R0_R1	21,26309204	-79,60420227	-939,4445801	0,01692
Cloud_Mesh_Distance_BA_R0_R1	21,35224724	-79,77883911	-939,4502564	-0,00555
Cloud_Mesh_Distance_BA_R0_R1	17,82367325	-79,50946045	-939,3577881	-0,046635
Cloud_Mesh_Distance_BA_R0_R1	17,96206284	-79,64924622	-939,2363892	-0,064634
Cloud_Mesh_Distance_BA_R0_R1	18,03989792	-79,23590088	-939,3458252	-0,044236
Cloud_Mesh_Distance_BA_R0_R1	21,12710381	-79,41151428	-939,4334717	0,042636
Cloud_Mesh_Distance_BA_R0_R1	21,33934212	-79,41459656	-939,5363159	0,026334
Cloud_Mesh_Distance_BA_R0_R1	21,54535866	-79,45903015	-939,6348267	0,002524
Cloud_Mesh_Distance_BA_R0_R1	17,87403107	-79,29681396	-939,4093628	-0,035614
Cloud_Mesh_Distance_BA_R0_R1	18,09987259	-78,92379761	-939,4577637	-0,023936
Cloud_Mesh_Distance_BA_R0_R1	18,14853859	-79,09417725	-939,3561401	-0,043931
Cloud_Mesh_Distance_BA_R0_R1	18,32316399	-79,10772705	-939,2807617	-0,056504
Cloud_Mesh_Distance_BA_R0_R1	21,05737305	-79,20553589	-939,4707031	0,059213
Cloud_Mesh_Distance_BA_R0_R1	21,22951889	-79,22592163	-939,5471802	0,046201
Cloud_Mesh_Distance_BA_R0_R1	17,89659882	-78,9161377	-939,5696411	-0,003928
Cloud_Mesh_Distance_BA_R0_R1	18,06028175	-78,74424744	-939,5689087	-0,003813
Cloud_Mesh_Distance_BA_R0_R1	18,44836426	-78,97834778	-939,2944946	-0,054812
Cloud_Mesh_Distance_BA_R0_R1	20,42752647	-78,94494629	-939,3207398	0,076412
Cloud_Mesh_Distance_BA_R0_R1	20,50345421	-78,78781128	-939,4147339	0,077606
Cloud_Mesh_Distance_BA_R0_R1	20,60437965	-78,94924927	-939,380127	0,081628
Cloud_Mesh_Distance_BA_R0_R1	20,8278656	-79,06314087	-939,4240723	0,076002
Cloud_Mesh_Distance_BA_R0_R1	20,90782738	-78,8327179	-939,56604	0,06975
Cloud_Mesh_Distance_BA_R0_R1	21,08640671	-79,01690674	-939,5648804	0,063052
Cloud_Mesh_Distance_BA_R0_R1	18,20980835	-78,61477661	-939,5689087	-0,003283
Cloud_Mesh_Distance_BA_R0_R1	18,26255798	-78,75830078	-939,4677734	-0,024419
Cloud_Mesh_Distance_BA_R0_R1	18,49996758	-78,78297424	-939,3703003	-0,045556
Cloud_Mesh_Distance_BA_R0_R1	18,68114281	-78,5737915	-939,4277954	-0,035742
Cloud_Mesh_Distance_BA_R0_R1	18,42539215	-78,55908203	-939,5134888	-0,017554
Cloud_Mesh_Distance_BA_R0_R1	18,76756096	-78,75408936	-939,315918	-0,05358
Cloud_Mesh_Distance_BA_R0_R1	20,08677483	-78,65551758	-939,3571167	0,048009
Cloud_Mesh_Distance_BA_R0_R1	20,29681587	-78,75123596	-939,3649292	0,067607
Cloud_Mesh_Distance_BA_R0_R1	20,42632294	-78,60961914	-939,4775391	0,06428
Cloud_Mesh_Distance_BA_R0_R1	20,6940403	-78,82035828	-939,4747315	0,077465
Cloud_Mesh_Distance_BA_R0_R1	18,25481796	-78,45758057	-939,6426392	0,008658

Cloud_Mesh_Distance_BA_R0_R1	19,06090927	-78,41983032	-939,440918	-0,033079
Cloud_Mesh_Distance_BA_R0_R1	19,54085159	-78,59880066	-939,3204346	-0,009641
Cloud_Mesh_Distance_BA_R0_R1	19,71971512	-78,49104309	-939,3866577	0,00741
Cloud_Mesh_Distance_BA_R0_R1	19,89511108	-78,57298279	-939,3638306	0,026315
Cloud_Mesh_Distance_BA_R0_R1	19,71780586	-78,35479736	-939,4639893	0,002512
Cloud_Mesh_Distance_BA_R0_R1	20,04632759	-78,46669006	-939,4477539	0,033957
Cloud_Mesh_Distance_BA_R0_R1	19,86566734	-78,40263367	-939,4526367	0,016438
Cloud_Mesh_Distance_BA_R0_R1	20,18567467	-78,35310364	-939,5490112	0,036117
Cloud_Mesh_Distance_BA_R0_R1	20,23810196	-78,53897095	-939,4556885	0,051274
Cloud_Mesh_Distance_BA_R0_R1	20,74793625	-78,63806152	-939,5948486	0,069833
Cloud_Mesh_Distance_BA_R0_R1	20,58434105	-78,67062378	-939,5047607	0,071932
Cloud_Mesh_Distance_BA_R0_R1	20,83567238	-78,5241394	-939,7067871	0,058597
Cloud_Mesh_Distance_BA_R0_R1	20,69684601	-78,46586609	-939,6745606	0,057111
Cloud_Mesh_Distance_BA_R0_R1	18,63817406	-78,3923645	-939,5440674	-0,015245
Cloud_Mesh_Distance_BA_R0_R1	18,84889984	-78,40762329	-939,4839478	-0,027545
Cloud_Mesh_Distance_BA_R0_R1	19,00136757	-78,26025391	-939,5469971	-0,019826
Cloud_Mesh_Distance_BA_R0_R1	19,3041935	-78,47094727	-939,3913574	-0,028496
Cloud_Mesh_Distance_BA_R0_R1	19,55697823	-78,39892578	-939,4292603	-0,011182
Cloud_Mesh_Distance_BA_R0_R1	19,63381767	-78,24429321	-939,5261231	-0,00492
Cloud_Mesh_Distance_BA_R0_R1	20,38013268	-78,41920471	-939,5702515	0,049559
Cloud_Mesh_Distance_BA_R0_R1	20,5628624	-78,51242065	-939,5849609	0,062831
Cloud_Mesh_Distance_BA_R0_R1	18,62688828	-78,22566223	-939,6548462	0,002847
Cloud_Mesh_Distance_BA_R0_R1	18,80622292	-78,2520752	-939,5899048	-0,012546
Cloud_Mesh_Distance_BA_R0_R1	18,94577599	-78,1211853	-939,6499634	-0,005538
Cloud_Mesh_Distance_BA_R0_R1	19,21193123	-78,2696228	-939,5162354	-0,021041
Cloud_Mesh_Distance_BA_R0_R1	19,13924599	-78,11993408	-939,621521	-0,011167
Cloud_Mesh_Distance_BA_R0_R1	19,33995056	-78,11665344	-939,6083984	-0,01514
Cloud_Mesh_Distance_BA_R0_R1	19,43102455	-78,26296997	-939,5092773	-0,014842
Cloud_Mesh_Distance_BA_R0_R1	19,74007797	-78,11117554	-939,625061	0,000984
Cloud_Mesh_Distance_BA_R0_R1	19,93553162	-78,13769531	-939,6342773	0,009008
Cloud_Mesh_Distance_BA_R0_R1	19,81559563	-78,2539978	-939,5369873	0,007986
Cloud_Mesh_Distance_BA_R0_R1	19,99744225	-78,29614258	-939,5404053	0,022316
Cloud_Mesh_Distance_BA_R0_R1	20,12331963	-78,18563843	-939,6433716	0,020987
Cloud_Mesh_Distance_BA_R0_R1	20,31119347	-78,23452759	-939,6664429	0,033054
Cloud_Mesh_Distance_BA_R0_R1	20,55052757	-78,2933197	-939,7224121	0,044466
Cloud_Mesh_Distance_BA_R0_R1	18,50192451	-82,13868713	-939,2041016	-0,117231
Cloud_Mesh_Distance_BA_R0_R1	18,89684868	-82,19950867	-939,1470947	-0,093631
Cloud_Mesh_Distance_BA_R0_R1	19,00903702	-82,03771973	-939,0854492	-0,072747
Cloud_Mesh_Distance_BA_R0_R1	18,38194084	-82,01145935	-939,1936646	-0,122175
Cloud_Mesh_Distance_BA_R0_R1	18,41654778	-81,81648254	-939,1160889	-0,101247
Cloud_Mesh_Distance_BA_R0_R1	18,80329132	-82,01818848	-939,0977173	-0,084938
Cloud_Mesh_Distance_BA_R0_R1	18,58424187	-81,97424316	-939,123169	-0,098529
Cloud_Mesh_Distance_BA_R0_R1	19,15126038	-81,88845825	-939,0412598	-0,054105
Cloud_Mesh_Distance_BA_R0_R1	19,20213318	-82,07888794	-939,0928955	-0,064181
Cloud_Mesh_Distance_BA_R0_R1	19,36190605	-81,94076538	-939,0623779	-0,047683
Cloud_Mesh_Distance_BA_R0_R1	19,3894577	-82,16252136	-939,1251221	-0,063138
Cloud_Mesh_Distance_BA_R0_R1	18,26276588	-81,65809631	-939,118042	-0,10291
Cloud_Mesh_Distance_BA_R0_R1	18,45727158	-81,6156311	-939,0487671	-0,080569
Cloud_Mesh_Distance_BA_R0_R1	18,59072113	-81,75802612	-939,0563965	-0,080485
Cloud_Mesh_Distance_BA_R0_R1	18,92785263	-81,82618713	-939,0316773	-0,062785

Cloud_Mesh_Distance_BA_R0_R1	18,73049545	-81,85346985	-939,0587158	-0,078677
Cloud_Mesh_Distance_BA_R0_R1	19,30160904	-81,72921753	-939,0108643	-0,040713
Cloud_Mesh_Distance_BA_R0_R1	19,55727959	-82,00959778	-939,0974731	-0,044954
Cloud_Mesh_Distance_BA_R0_R1	19,56068039	-81,76972961	-939,0428467	-0,033196
Cloud_Mesh_Distance_BA_R0_R1	19,7123909	-81,90919495	-939,0939331	-0,033811
Cloud_Mesh_Distance_BA_R0_R1	19,88885307	-81,93301392	-939,130127	-0,028386
Cloud_Mesh_Distance_BA_R0_R1	20,11275101	-81,95664978	-939,1849365	-0,022184
Cloud_Mesh_Distance_BA_R0_R1	20,02921677	-81,7550354	-939,1206665	-0,018763
Cloud_Mesh_Distance_BA_R0_R1	18,75662231	-81,68490601	-939,0125732	-0,061525
Cloud_Mesh_Distance_BA_R0_R1	19,10274696	-81,71495056	-939,0008545	-0,04756
Cloud_Mesh_Distance_BA_R0_R1	19,18382263	-81,5521698	-938,9710083	-0,038866
Cloud_Mesh_Distance_BA_R0_R1	19,81442642	-81,74397278	-939,0753784	-0,024703
Cloud_Mesh_Distance_BA_R0_R1	19,71914482	-81,53994751	-939,0231934	-0,02328
Cloud_Mesh_Distance_BA_R0_R1	20,182621	-81,58039856	-939,1240845	-0,013493
Cloud_Mesh_Distance_BA_R0_R1	20,32905769	-81,86608887	-939,2214966	-0,014327
Cloud_Mesh_Distance_BA_R0_R1	20,36624527	-81,65606689	-939,1867065	-0,010785
Cloud_Mesh_Distance_BA_R0_R1	20,20486069	-81,76263428	-939,1647949	-0,015031
Cloud_Mesh_Distance_BA_R0_R1	18,32687187	-81,41494751	-939,0361328	-0,076542
Cloud_Mesh_Distance_BA_R0_R1	18,61597061	-81,59848022	-939,0119629	-0,068328
Cloud_Mesh_Distance_BA_R0_R1	18,74202347	-81,49276733	-938,9724121	-0,052112
Cloud_Mesh_Distance_BA_R0_R1	18,95176125	-81,57922363	-938,975647	-0,051123
Cloud_Mesh_Distance_BA_R0_R1	18,90203285	-81,36126709	-938,9385376	-0,045639
Cloud_Mesh_Distance_BA_R0_R1	19,06802177	-81,42149353	-938,9459229	-0,039434
Cloud_Mesh_Distance_BA_R0_R1	19,4412632	-81,50592041	-938,9805908	-0,030016
Cloud_Mesh_Distance_BA_R0_R1	19,96341515	-81,54975891	-939,0687866	-0,017041
Cloud_Mesh_Distance_BA_R0_R1	17,97140884	-81,26947021	-939,1419067	-0,096019
Cloud_Mesh_Distance_BA_R0_R1	18,15507698	-81,24914551	-939,0593872	-0,082127
Cloud_Mesh_Distance_BA_R0_R1	20,20989227	-81,0816803	-939,0631714	-0,022341
Cloud_Mesh_Distance_BA_R0_R1	20,08771324	-80,9846344	-939,0261841	-0,024669
Cloud_Mesh_Distance_BA_R0_R1	20,37914467	-81,42715454	-939,1506958	-0,012243
Cloud_Mesh_Distance_BA_R0_R1	20,39125252	-81,18273926	-939,1205444	-0,01684
Cloud_Mesh_Distance_BA_R0_R1	20,14560699	-81,33117676	-939,0767212	-0,017764
Cloud_Mesh_Distance_BA_R0_R1	20,61803055	-81,27810669	-939,2005005	-0,015946
Cloud_Mesh_Distance_BA_R0_R1	17,9037056	-81,13923645	-939,1511231	-0,09657
Cloud_Mesh_Distance_BA_R0_R1	18,02697182	-81,08267212	-939,0830689	-0,083063
Cloud_Mesh_Distance_BA_R0_R1	18,22699738	-81,04093933	-939,0026856	-0,069753
Cloud_Mesh_Distance_BA_R0_R1	20,23100281	-80,8802948	-939,0508423	-0,026049
Cloud_Mesh_Distance_BA_R0_R1	17,83356476	-80,96380615	-939,1647339	-0,089403
Cloud_Mesh_Distance_BA_R0_R1	20,06709099	-80,8249054	-939,0103149	-0,027553
Cloud_Mesh_Distance_BA_R0_R1	20,59346581	-81,02355957	-939,1605225	-0,02206
Cloud_Mesh_Distance_BA_R0_R1	20,40020561	-80,95295715	-939,0996094	-0,024621
Cloud_Mesh_Distance_BA_R0_R1	20,77223778	-80,85621643	-939,2018433	-0,027687
Cloud_Mesh_Distance_BA_R0_R1	17,75709724	-80,57171631	-939,1875	-0,081238
Cloud_Mesh_Distance_BA_R0_R1	17,897892	-80,69245911	-939,1104126	-0,079283
Cloud_Mesh_Distance_BA_R0_R1	20,22951317	-80,67907715	-939,0383301	-0,025282
Cloud_Mesh_Distance_BA_R0_R1	20,39825821	-80,74572754	-939,083313	-0,025725
Cloud_Mesh_Distance_BA_R0_R1	20,39073944	-80,57028198	-939,0734253	-0,024696
Cloud_Mesh_Distance_BA_R0_R1	20,57972908	-80,79983521	-939,137146	-0,0276
Cloud_Mesh_Distance_BA_R0_R1	21,21539307	-80,82244873	-939,3655396	-0,042316
Cloud_Mesh_Distance_BA_R0_R1	21,08765984	-80,66656494	-939,3004761	-0,037583

Cloud_Mesh_Distance_BA_R0_R1	17,91343689	-80,46672058	-939,1072388	-0,076378
Cloud_Mesh_Distance_BA_R0_R1	18,07904243	-80,56253052	-939,0305786	-0,074343
Cloud_Mesh_Distance_BA_R0_R1	18,21118546	-80,46780396	-938,9884644	-0,073059
Cloud_Mesh_Distance_BA_R0_R1	20,4971962	-80,39932251	-939,0982666	-0,017697
Cloud_Mesh_Distance_BA_R0_R1	20,55932426	-80,58854675	-939,1199341	-0,027264
Cloud_Mesh_Distance_BA_R0_R1	20,75284195	-80,63648987	-939,1801148	-0,029962
Cloud_Mesh_Distance_BA_R0_R1	21,14198494	-80,48736572	-939,3143311	-0,040609
Cloud_Mesh_Distance_BA_R0_R1	20,91294479	-80,70858765	-939,2387085	-0,033678
Cloud_Mesh_Distance_BA_R0_R1	17,89485741	-80,2940979	-939,1311646	-0,076214
Cloud_Mesh_Distance_BA_R0_R1	20,77829552	-80,14929199	-939,1847534	-0,004805
Cloud_Mesh_Distance_BA_R0_R1	20,73037529	-80,38394165	-939,1660767	-0,021699
Cloud_Mesh_Distance_BA_R0_R1	20,95446205	-80,49134827	-939,2434082	-0,034161
Cloud_Mesh_Distance_BA_R0_R1	20,94345474	-80,23530579	-939,2385864	-0,01784
Cloud_Mesh_Distance_BA_R0_R1	21,16674614	-80,09255981	-939,3311157	-0,02235
Cloud_Mesh_Distance_BA_R0_R1	17,92650604	-80,12071228	-939,1405029	-0,075137
Cloud_Mesh_Distance_BA_R0_R1	17,78339577	-80,04655457	-939,2321777	-0,069908
Cloud_Mesh_Distance_BA_R0_R1	18,10898781	-80,29702759	-939,0375366	-0,075699
Cloud_Mesh_Distance_BA_R0_R1	18,1163559	-80,00163269	-939,0791626	-0,076347
Cloud_Mesh_Distance_BA_R0_R1	18,11488342	-79,77502441	-939,1310425	-0,071757
Cloud_Mesh_Distance_BA_R0_R1	18,28642845	-79,8578186	-939,0499878	-0,081064
Cloud_Mesh_Distance_BA_R0_R1	18,29973221	-79,66407776	-939,0953369	-0,080946
Cloud_Mesh_Distance_BA_R0_R1	20,49790001	-79,88589478	-939,117981	0,024071
Cloud_Mesh_Distance_BA_R0_R1	20,67942619	-79,9715271	-939,1641846	0,012761
Cloud_Mesh_Distance_BA_R0_R1	20,78128242	-79,80255127	-939,2138672	0,028486
Cloud_Mesh_Distance_BA_R0_R1	20,63083267	-79,75041199	-939,1724243	0,036069
Cloud_Mesh_Distance_BA_R0_R1	20,41780472	-79,49888611	-939,15271	0,058767
Cloud_Mesh_Distance_BA_R0_R1	20,59807205	-79,52909851	-939,199585	0,057805
Cloud_Mesh_Distance_BA_R0_R1	20,9176445	-79,68939209	-939,2810059	0,032425
Cloud_Mesh_Distance_BA_R0_R1	20,74752998	-79,64512634	-939,2268677	0,044546
Cloud_Mesh_Distance_BA_R0_R1	21,09974289	-79,63298035	-939,364563	0,027929
Cloud_Mesh_Distance_BA_R0_R1	20,91257668	-79,96688843	-939,2424316	0,006065
Cloud_Mesh_Distance_BA_R0_R1	21,08097267	-79,83694458	-939,3217773	0,007535
Cloud_Mesh_Distance_BA_R0_R1	18,36558151	-79,25390625	-939,208374	-0,067506
Cloud_Mesh_Distance_BA_R0_R1	18,28651237	-79,44003296	-939,1679688	-0,071977
Cloud_Mesh_Distance_BA_R0_R1	18,19721985	-79,26002502	-939,2667236	-0,057671
Cloud_Mesh_Distance_BA_R0_R1	18,13806915	-79,59132385	-939,1738281	-0,068116
Cloud_Mesh_Distance_BA_R0_R1	18,04129028	-79,42544556	-939,2728272	-0,056902
Cloud_Mesh_Distance_BA_R0_R1	18,45385933	-79,54714966	-939,0836182	-0,080523
Cloud_Mesh_Distance_BA_R0_R1	20,92490578	-79,31430054	-939,3747559	0,063168
Cloud_Mesh_Distance_BA_R0_R1	20,97425652	-79,49902344	-939,3425293	0,046821
Cloud_Mesh_Distance_BA_R0_R1	18,50032616	-79,12478638	-939,2182007	-0,070442
Cloud_Mesh_Distance_BA_R0_R1	18,81158638	-79,34996033	-939,0718384	-0,080277
Cloud_Mesh_Distance_BA_R0_R1	18,53381729	-79,33779907	-939,1303101	-0,078561
Cloud_Mesh_Distance_BA_R0_R1	20,28524208	-79,3637085	-939,147583	0,060748
Cloud_Mesh_Distance_BA_R0_R1	20,32371521	-79,12796021	-939,2242432	0,072788
Cloud_Mesh_Distance_BA_R0_R1	20,47615242	-79,33329773	-939,2078247	0,068026
Cloud_Mesh_Distance_BA_R0_R1	20,57953262	-79,14395142	-939,2976685	0,080628
Cloud_Mesh_Distance_BA_R0_R1	20,6414032	-79,3449707	-939,2577515	0,069219
Cloud_Mesh_Distance_BA_R0_R1	20,79587746	-79,46604919	-939,2816162	0,058095
Cloud_Mesh_Distance_BA_R0_R1	20,75407791	-79,26330566	-939,3219605	0,074377

Cloud_Mesh_Distance_BA_R0_R1	18,29467583	-78,93917847	-939,3664551	-0,041539
Cloud_Mesh_Distance_BA_R0_R1	18,71328354	-79,13342285	-939,1645508	-0,075665
Cloud_Mesh_Distance_BA_R0_R1	18,95303917	-79,12060547	-939,130188	-0,068718
Cloud_Mesh_Distance_BA_R0_R1	20,11145782	-79,21118164	-939,1481323	0,053322
Cloud_Mesh_Distance_BA_R0_R1	20,10988808	-78,98991394	-939,2197876	0,057031
Cloud_Mesh_Distance_BA_R0_R1	18,63085938	-78,94799805	-939,2576294	-0,060411
Cloud_Mesh_Distance_BA_R0_R1	18,86236954	-78,93444824	-939,2161865	-0,062016
Cloud_Mesh_Distance_BA_R0_R1	19,09275818	-78,93292236	-939,1862183	-0,054435
Cloud_Mesh_Distance_BA_R0_R1	19,28361511	-78,92799377	-939,1749878	-0,042866
Cloud_Mesh_Distance_BA_R0_R1	19,66869545	-78,95846558	-939,1695557	0,004997
Cloud_Mesh_Distance_BA_R0_R1	19,91015244	-79,0688324	-939,1568604	0,033446
Cloud_Mesh_Distance_BA_R0_R1	20,25957108	-78,92504883	-939,2793579	0,06894
Cloud_Mesh_Distance_BA_R0_R1	20,13304901	-78,82202148	-939,2917481	0,054883
Cloud_Mesh_Distance_BA_R0_R1	19,02256203	-78,73716736	-939,2808838	-0,051505
Cloud_Mesh_Distance_BA_R0_R1	18,91200447	-78,57260132	-939,3796997	-0,041452
Cloud_Mesh_Distance_BA_R0_R1	19,45339394	-78,80964661	-939,2216187	-0,019765
Cloud_Mesh_Distance_BA_R0_R1	19,23274422	-78,78129578	-939,2402954	-0,03971
Cloud_Mesh_Distance_BA_R0_R1	19,90373039	-78,80307007	-939,2578125	0,031131
Cloud_Mesh_Distance_BA_R0_R1	19,7449131	-78,64097595	-939,3119507	0,012664
Cloud_Mesh_Distance_BA_R0_R1	19,66345596	-78,75776672	-939,2511597	0,004417
Cloud_Mesh_Distance_BA_R0_R1	19,14863014	-78,60214233	-939,3320923	-0,038818
Cloud_Mesh_Distance_BA_R0_R1	19,34556007	-78,65653992	-939,2927856	-0,030748
Cloud_Mesh_Distance_BA_R0_R1	18,32330513	-81,19946289	-938,9972534	-0,069681
Cloud_Mesh_Distance_BA_R0_R1	18,55157661	-81,44329834	-938,9893799	-0,066021
Cloud_Mesh_Distance_BA_R0_R1	18,49404144	-81,24650574	-938,9661865	-0,063479
Cloud_Mesh_Distance_BA_R0_R1	19,07645226	-81,25419617	-938,9225464	-0,03822
Cloud_Mesh_Distance_BA_R0_R1	19,24144173	-81,36068726	-938,9425049	-0,034107
Cloud_Mesh_Distance_BA_R0_R1	19,6428318	-81,31903076	-938,9790039	-0,024472
Cloud_Mesh_Distance_BA_R0_R1	18,43728828	-81,04589844	-938,9501343	-0,060674
Cloud_Mesh_Distance_BA_R0_R1	18,70219803	-81,29223633	-938,9424439	-0,052158
Cloud_Mesh_Distance_BA_R0_R1	18,89991379	-81,12915039	-938,9095459	-0,045159
Cloud_Mesh_Distance_BA_R0_R1	19,25695992	-81,16720581	-938,9205933	-0,034324
Cloud_Mesh_Distance_BA_R0_R1	19,11091042	-81,0854187	-938,9056397	-0,040877
Cloud_Mesh_Distance_BA_R0_R1	19,42979431	-81,26008606	-938,9452515	-0,030509
Cloud_Mesh_Distance_BA_R0_R1	19,58602905	-81,11312866	-938,9481812	-0,028601
Cloud_Mesh_Distance_BA_R0_R1	19,87990761	-81,3401947	-939,020874	-0,019077
Cloud_Mesh_Distance_BA_R0_R1	18,65523529	-81,08135986	-938,9210815	-0,053721
Cloud_Mesh_Distance_BA_R0_R1	18,59731293	-80,86618042	-938,90979	-0,057025
Cloud_Mesh_Distance_BA_R0_R1	18,82750893	-80,90583801	-938,8936768	-0,049752
Cloud_Mesh_Distance_BA_R0_R1	19,05194283	-80,92398071	-938,8915405	-0,043911
Cloud_Mesh_Distance_BA_R0_R1	19,28165627	-80,95571899	-938,9038086	-0,037985
Cloud_Mesh_Distance_BA_R0_R1	19,50790787	-80,93670654	-938,9246826	-0,033705
Cloud_Mesh_Distance_BA_R0_R1	19,41160965	-81,0869751	-938,9250488	-0,033622
Cloud_Mesh_Distance_BA_R0_R1	19,93103409	-80,97451782	-938,9934082	-0,026328
Cloud_Mesh_Distance_BA_R0_R1	19,79585266	-81,13954163	-938,982666	-0,02446
Cloud_Mesh_Distance_BA_R0_R1	20,00759888	-81,14535522	-939,0241089	-0,02215
Cloud_Mesh_Distance_BA_R0_R1	18,27523232	-80,6187439	-938,9661255	-0,068709
Cloud_Mesh_Distance_BA_R0_R1	18,35736656	-80,8377533	-938,9503174	-0,064641
Cloud_Mesh_Distance_BA_R0_R1	18,7815094	-80,70188904	-938,8878784	-0,057266
Cloud_Mesh_Distance_BA_R0_R1	19,61146355	-80,78009033	-938,9297485	-0,037436

Cloud_Mesh_Distance_BA_R0_R1	19,73116493	-80,923172	-938,9543457	-0,030021
Cloud_Mesh_Distance_BA_R0_R1	18,09295654	-80,829422	-939,0307007	-0,073485
Cloud_Mesh_Distance_BA_R0_R1	18,54458809	-80,61917114	-938,9102783	-0,062793
Cloud_Mesh_Distance_BA_R0_R1	18,39285851	-80,38700867	-938,946167	-0,069743
Cloud_Mesh_Distance_BA_R0_R1	18,98928642	-80,73425293	-938,8829346	-0,052189
Cloud_Mesh_Distance_BA_R0_R1	19,20705986	-80,74055481	-938,8894043	-0,046479
Cloud_Mesh_Distance_BA_R0_R1	18,93925095	-80,5824585	-938,8814087	-0,05852
Cloud_Mesh_Distance_BA_R0_R1	19,11915779	-80,56242371	-938,8829346	-0,054814
Cloud_Mesh_Distance_BA_R0_R1	19,42953682	-80,75408936	-938,9075928	-0,041276
Cloud_Mesh_Distance_BA_R0_R1	19,90678787	-80,8069458	-938,9779053	-0,030906
Cloud_Mesh_Distance_BA_R0_R1	18,63567924	-80,40693665	-938,9050293	-0,069387
Cloud_Mesh_Distance_BA_R0_R1	18,79078293	-80,50518799	-938,887207	-0,065506
Cloud_Mesh_Distance_BA_R0_R1	19,00301933	-80,41891479	-938,8834229	-0,063298
Cloud_Mesh_Distance_BA_R0_R1	19,3608532	-80,52331543	-938,8979492	-0,050521
Cloud_Mesh_Distance_BA_R0_R1	19,60365677	-80,5880127	-938,9237061	-0,041758
Cloud_Mesh_Distance_BA_R0_R1	20,02477837	-80,60688782	-938,9932861	-0,028704
Cloud_Mesh_Distance_BA_R0_R1	20,07442284	-80,37309265	-938,9998779	-0,020767
Cloud_Mesh_Distance_BA_R0_R1	19,78629684	-80,69018555	-938,9528809	-0,032781
Cloud_Mesh_Distance_BA_R0_R1	20,21931076	-80,20819092	-939,0317993	-0,009721
Cloud_Mesh_Distance_BA_R0_R1	18,62009621	-80,2106781	-938,9221802	-0,078951
Cloud_Mesh_Distance_BA_R0_R1	18,83658409	-80,30133057	-938,8942261	-0,073263
Cloud_Mesh_Distance_BA_R0_R1	19,19678688	-80,39154053	-938,8885498	-0,061338
Cloud_Mesh_Distance_BA_R0_R1	19,36506653	-80,28091431	-938,9037476	-0,055407
Cloud_Mesh_Distance_BA_R0_R1	19,54507065	-80,13137817	-938,9280396	-0,048464
Cloud_Mesh_Distance_BA_R0_R1	19,58471298	-80,3513031	-938,9229126	-0,044982
Cloud_Mesh_Distance_BA_R0_R1	19,83923531	-80,42303467	-938,958313	-0,032122
Cloud_Mesh_Distance_BA_R0_R1	20,26647377	-80,43768311	-939,0412598	-0,019948
Cloud_Mesh_Distance_BA_R0_R1	18,38120461	-80,07551575	-938,984375	-0,082495
Cloud_Mesh_Distance_BA_R0_R1	18,53543091	-79,76586914	-939,0087891	-0,088634
Cloud_Mesh_Distance_BA_R0_R1	18,63829613	-79,99804688	-938,9464722	-0,085254
Cloud_Mesh_Distance_BA_R0_R1	19,06220245	-79,91714478	-938,9262085	-0,076391
Cloud_Mesh_Distance_BA_R0_R1	18,81861496	-79,84439087	-938,9509888	-0,085278
Cloud_Mesh_Distance_BA_R0_R1	18,84846115	-80,07783508	-938,9140015	-0,07928
Cloud_Mesh_Distance_BA_R0_R1	19,29397202	-79,95680237	-938,9240723	-0,063136
Cloud_Mesh_Distance_BA_R0_R1	19,3630867	-80,10670471	-938,9138184	-0,059752
Cloud_Mesh_Distance_BA_R0_R1	19,77287865	-80,1585083	-938,9545288	-0,030289
Cloud_Mesh_Distance_BA_R0_R1	20,00779724	-80,18530273	-938,9911499	-0,017469
Cloud_Mesh_Distance_BA_R0_R1	20,35997391	-80,03295898	-939,071167	0,008107
Cloud_Mesh_Distance_BA_R0_R1	20,5163765	-80,06105042	-939,1101074	0,007097
Cloud_Mesh_Distance_BA_R0_R1	20,41493988	-80,22229004	-939,0777588	-0,006666
Cloud_Mesh_Distance_BA_R0_R1	20,6067009	-80,19662476	-939,1298218	-0,00439
Cloud_Mesh_Distance_BA_R0_R1	19,47540474	-79,98130798	-938,932251	-0,049941
Cloud_Mesh_Distance_BA_R0_R1	20,11424065	-79,7747345	-939,0394898	0,02075
Cloud_Mesh_Distance_BA_R0_R1	19,94616699	-79,95152283	-938,9927979	-0,00815
Cloud_Mesh_Distance_BA_R0_R1	20,31422424	-79,82650757	-939,0775757	0,025413
Cloud_Mesh_Distance_BA_R0_R1	20,16594505	-79,9962616	-939,0304565	0,003598
Cloud_Mesh_Distance_BA_R0_R1	18,62579155	-79,52235413	-939,0523071	-0,083102
Cloud_Mesh_Distance_BA_R0_R1	18,8057766	-79,60661316	-939,0023804	-0,088348
Cloud_Mesh_Distance_BA_R0_R1	19,03268433	-79,68675232	-938,9660645	-0,079701
Cloud_Mesh_Distance_BA_R0_R1	19,25730705	-79,74871826	-938,9516602	-0,065454

Cloud_Mesh_Distance_BA_R0_R1	19,23293495	-79,53475952	-938,9916382	-0,062383
Cloud_Mesh_Distance_BA_R0_R1	19,65465164	-79,6522522	-938,9879761	-0,019299
Cloud_Mesh_Distance_BA_R0_R1	19,4716053	-79,80523682	-938,9523926	-0,045782
Cloud_Mesh_Distance_BA_R0_R1	19,68536377	-79,90859985	-938,9598999	-0,027901
Cloud_Mesh_Distance_BA_R0_R1	19,89588165	-79,69213867	-939,0120239	0,006074
Cloud_Mesh_Distance_BA_R0_R1	19,96132278	-79,4981842	-939,0537109	0,023949
Cloud_Mesh_Distance_BA_R0_R1	20,07151604	-79,59509277	-939,055603	0,027525
Cloud_Mesh_Distance_BA_R0_R1	20,45698738	-79,67953491	-939,1314697	0,041835
Cloud_Mesh_Distance_BA_R0_R1	19,02327156	-79,46321106	-939,0167236	-0,078105
Cloud_Mesh_Distance_BA_R0_R1	19,04166985	-79,27389526	-939,0697632	-0,069879
Cloud_Mesh_Distance_BA_R0_R1	19,41699409	-79,39404297	-939,0244751	-0,034921
Cloud_Mesh_Distance_BA_R0_R1	19,22109032	-79,32839966	-939,0424194	-0,055395
Cloud_Mesh_Distance_BA_R0_R1	19,44277191	-79,59469604	-938,9831543	-0,039726
Cloud_Mesh_Distance_BA_R0_R1	19,60752678	-79,45054626	-939,0211792	-0,013881
Cloud_Mesh_Distance_BA_R0_R1	19,91159248	-79,30874634	-939,0872192	0,028716
Cloud_Mesh_Distance_BA_R0_R1	20,10426903	-79,42608643	-939,09375	0,043848
Cloud_Mesh_Distance_BA_R0_R1	20,25687027	-79,59364319	-939,0942383	0,041995
Cloud_Mesh_Distance_BA_R0_R1	19,20433044	-79,11218262	-939,1106567	-0,050483
Cloud_Mesh_Distance_BA_R0_R1	19,73009491	-79,33265686	-939,0584106	0,006145
Cloud_Mesh_Distance_BA_R0_R1	19,79837227	-79,48457336	-939,03302	0,006768
Cloud_Mesh_Distance_BA_R0_R1	19,57972527	-79,27384949	-939,0621948	-0,014092
Cloud_Mesh_Distance_BA_R0_R1	19,5664444	-79,12037659	-939,1072388	-0,010715
Cloud_Mesh_Distance_BA_R0_R1	19,40528679	-79,20385742	-939,076416	-0,031539
Cloud_Mesh_Distance_BA_R0_R1	19,43195724	-79,01797485	-939,1384277	-0,023973
Cloud_Mesh_Distance_BA_R0_R1	19,73180389	-79,17170715	-939,1029053	0,011464
Cloud_Mesh_Distance_BA_R0_R1	19,12244606	-80,18128967	-938,8978272	-0,070103
Cloud_Mesh_Distance_BA_R0_R2	17,45834923	-78,78816223	-946,3978272	-0,075186
Cloud_Mesh_Distance_BA_R0_R2	17,4753418	-78,81703186	-946,3987427	-0,073263
Cloud_Mesh_Distance_BA_R0_R2	17,99739838	-79,9312439	-946,2457275	-0,00634
Cloud_Mesh_Distance_BA_R0_R2	18,04758644	-80,1084137	-946,1830444	-0,016189
Cloud_Mesh_Distance_BA_R0_R2	18,40240479	-80,08332825	-946,1647949	-0,016085
Cloud_Mesh_Distance_BA_R0_R2	16,55277061	-79,66462708	-946,1582031	-0,083463
Cloud_Mesh_Distance_BA_R0_R2	17,25386429	-79,82705688	-946,2327881	-0,073731
Cloud_Mesh_Distance_BA_R0_R2	17,42531204	-79,70414734	-946,2886353	-0,057497
Cloud_Mesh_Distance_BA_R0_R2	17,65315056	-79,93302917	-946,2364502	-0,033001
Cloud_Mesh_Distance_BA_R0_R2	18,19223595	-79,97419739	-946,22229	0,001308
Cloud_Mesh_Distance_BA_R0_R2	18,17473221	-79,80734253	-946,2723389	0,006901
Cloud_Mesh_Distance_BA_R0_R2	18,33294868	-79,84588623	-946,2445679	0,006189
Cloud_Mesh_Distance_BA_R0_R2	18,46643829	-79,9974823	-946,1817017	-0,008552
Cloud_Mesh_Distance_BA_R0_R2	18,35318565	-79,99124146	-946,2011719	-0,003647
Cloud_Mesh_Distance_BA_R0_R2	16,84558487	-79,49810791	-946,2698364	-0,08869
Cloud_Mesh_Distance_BA_R0_R2	16,57986069	-79,64762878	-946,1724243	-0,082696
Cloud_Mesh_Distance_BA_R0_R2	17,20634079	-79,63710022	-946,2839356	-0,078381
Cloud_Mesh_Distance_BA_R0_R2	17,2462616	-79,45848083	-946,3277588	-0,078223
Cloud_Mesh_Distance_BA_R0_R2	17,63087082	-79,75489807	-946,289917	-0,035129
Cloud_Mesh_Distance_BA_R0_R2	17,61511803	-79,56942749	-946,3323975	-0,040769
Cloud_Mesh_Distance_BA_R0_R2	18,03496742	-79,7648468	-946,2932129	0,003858
Cloud_Mesh_Distance_BA_R0_R2	17,84608269	-79,80274963	-946,2851563	-0,012916
Cloud_Mesh_Distance_BA_R0_R2	18,16849327	-79,64195251	-946,3112183	0,013542
Cloud_Mesh_Distance_BA_R0_R2	18,46731758	-79,8853302	-946,2128296	-0,000303

Cloud_Mesh_Distance_BA_R0_R2	18,47713852	-79,73100281	-946,2463379	0,011115
Cloud_Mesh_Distance_BA_R0_R2	18,60645294	-79,79905701	-946,2028198	-0,004822
Cloud_Mesh_Distance_BA_R0_R2	16,37956429	-79,3296051	-946,1958618	-0,07462
Cloud_Mesh_Distance_BA_R0_R2	16,48265076	-79,34994507	-946,2251587	-0,074507
Cloud_Mesh_Distance_BA_R0_R2	17,02692604	-79,47360229	-946,3006592	-0,090752
Cloud_Mesh_Distance_BA_R0_R2	17,05115318	-79,35813904	-946,324707	-0,09192
Cloud_Mesh_Distance_BA_R0_R2	17,41624451	-79,51960754	-946,3300171	-0,06351
Cloud_Mesh_Distance_BA_R0_R2	17,61249161	-79,37892151	-946,3638916	-0,04544
Cloud_Mesh_Distance_BA_R0_R2	17,8106842	-79,41921997	-946,3616943	-0,021582
Cloud_Mesh_Distance_BA_R0_R2	17,81442833	-79,60935974	-946,3310547	-0,018262
Cloud_Mesh_Distance_BA_R0_R2	18,14613533	-79,49984741	-946,3365479	0,013069
Cloud_Mesh_Distance_BA_R0_R2	17,99960327	-79,63130188	-946,3243408	0,001475
Cloud_Mesh_Distance_BA_R0_R2	18,00152779	-79,45545959	-946,352478	-0,000568
Cloud_Mesh_Distance_BA_R0_R2	18,32886505	-79,67533875	-946,2845459	0,015846
Cloud_Mesh_Distance_BA_R0_R2	18,33539009	-79,4650116	-946,3165283	0,018592
Cloud_Mesh_Distance_BA_R0_R2	16,48284912	-79,0627594	-946,2574463	-0,088226
Cloud_Mesh_Distance_BA_R0_R2	16,35463715	-79,18684387	-946,2062988	-0,078132
Cloud_Mesh_Distance_BA_R0_R2	16,48493195	-79,23254395	-946,2435913	-0,076919
Cloud_Mesh_Distance_BA_R0_R2	16,5713768	-79,30093384	-946,2566528	-0,079308
Cloud_Mesh_Distance_BA_R0_R2	16,68465614	-79,15531921	-946,3005981	-0,09006
Cloud_Mesh_Distance_BA_R0_R2	16,67327499	-79,34033203	-946,2733154	-0,086043
Cloud_Mesh_Distance_BA_R0_R2	16,87146759	-79,32814026	-946,3075562	-0,089399
Cloud_Mesh_Distance_BA_R0_R2	16,9958725	-79,30505371	-946,3268433	-0,093784
Cloud_Mesh_Distance_BA_R0_R2	16,9875164	-79,36331177	-946,3162842	-0,094623
Cloud_Mesh_Distance_BA_R0_R2	17,05950356	-79,29978943	-946,3353272	-0,08985
Cloud_Mesh_Distance_BA_R0_R2	17,06786728	-79,18309021	-946,3526001	-0,089587
Cloud_Mesh_Distance_BA_R0_R2	17,1886673	-79,30036926	-946,348938	-0,084222
Cloud_Mesh_Distance_BA_R0_R2	17,25925255	-79,11584473	-946,3764648	-0,085757
Cloud_Mesh_Distance_BA_R0_R2	17,41347122	-79,33529663	-946,3614502	-0,0676
Cloud_Mesh_Distance_BA_R0_R2	18,01400948	-79,25892639	-946,3721314	-0,00376
Cloud_Mesh_Distance_BA_R0_R2	18,38050652	-79,31037903	-946,3225098	0,013892
Cloud_Mesh_Distance_BA_R0_R2	18,49829102	-79,39894104	-946,2896118	0,01322
Cloud_Mesh_Distance_BA_R0_R2	18,63407898	-79,61863708	-946,2277222	-0,000464
Cloud_Mesh_Distance_BA_R0_R2	18,484375	-79,5539093	-946,2752686	0,013965
Cloud_Mesh_Distance_BA_R0_R2	16,50184441	-78,91578674	-946,2665405	-0,098714
Cloud_Mesh_Distance_BA_R0_R2	16,66814995	-78,90040588	-946,3092041	-0,105467
Cloud_Mesh_Distance_BA_R0_R2	16,90941238	-79,11802673	-946,3413086	-0,096162
Cloud_Mesh_Distance_BA_R0_R2	17,41633034	-79,14375305	-946,3829956	-0,068284
Cloud_Mesh_Distance_BA_R0_R2	17,22063828	-79,0042572	-946,3820801	-0,087228
Cloud_Mesh_Distance_BA_R0_R2	17,61550713	-79,18099976	-946,3856201	-0,049723
Cloud_Mesh_Distance_BA_R0_R2	17,82056999	-79,01092529	-946,3933106	-0,031324
Cloud_Mesh_Distance_BA_R0_R2	18,01593208	-79,05554199	-946,381958	-0,010551
Cloud_Mesh_Distance_BA_R0_R2	17,81548882	-79,22047424	-946,3823242	-0,024507
Cloud_Mesh_Distance_BA_R0_R2	18,23185158	-79,30012512	-946,3474731	0,009686
Cloud_Mesh_Distance_BA_R0_R2	18,20454788	-79,0925293	-946,3618164	0,004545
Cloud_Mesh_Distance_BA_R0_R2	16,72625923	-78,69560242	-946,3164673	-0,121022
Cloud_Mesh_Distance_BA_R0_R2	16,56608582	-78,78384399	-946,2805786	-0,114487
Cloud_Mesh_Distance_BA_R0_R2	16,87697601	-78,943573	-946,3476563	-0,1019
Cloud_Mesh_Distance_BA_R0_R2	16,88119507	-78,74043274	-946,3485107	-0,116545
Cloud_Mesh_Distance_BA_R0_R2	17,29911995	-78,80897522	-946,3929443	-0,08756

Cloud_Mesh_Distance_BA_R0_R2	17,07184219	-78,97235107	-946,3707275	-0,093503
Cloud_Mesh_Distance_BA_R0_R2	17,07315636	-78,77397156	-946,3743897	-0,103218
Cloud_Mesh_Distance_BA_R0_R2	17,43544388	-78,76013184	-946,3968506	-0,077448
Cloud_Mesh_Distance_BA_R0_R2	17,41868591	-78,8213501	-946,3972778	-0,077414
Cloud_Mesh_Distance_BA_R0_R2	17,41202927	-78,93510437	-946,3960571	-0,071503
Cloud_Mesh_Distance_BA_R0_R2	17,61437607	-78,97444153	-946,3977051	-0,054064
Cloud_Mesh_Distance_BA_R0_R2	18,01858521	-78,84666443	-946,382019	-0,016863
Cloud_Mesh_Distance_BA_R0_R2	18,37154388	-79,10765076	-946,3345337	0,010189
Cloud_Mesh_Distance_BA_R0_R2	18,49022484	-79,12663269	-946,3086548	0,00864
Cloud_Mesh_Distance_BA_R0_R2	16,73342514	-78,56634521	-946,3081665	-0,129885
Cloud_Mesh_Distance_BA_R0_R2	16,9028244	-78,53434753	-946,3392334	-0,128002
Cloud_Mesh_Distance_BA_R0_R2	17,49228096	-78,62840271	-946,3930664	-0,079211
Cloud_Mesh_Distance_BA_R0_R2	17,49198914	-78,75601196	-946,3983154	-0,073301
Cloud_Mesh_Distance_BA_R0_R2	17,58307648	-78,78796387	-946,3994141	-0,063119
Cloud_Mesh_Distance_BA_R0_R2	17,82500076	-78,77661133	-946,3921509	-0,039881
Cloud_Mesh_Distance_BA_R0_R2	18,20371246	-78,89808655	-946,3634033	-0,002343
Cloud_Mesh_Distance_BA_R0_R2	18,52150726	-78,9881897	-946,3043213	0,005561
Cloud_Mesh_Distance_BA_R0_R2	18,37282181	-78,93913269	-946,3363648	0,005149
Cloud_Mesh_Distance_BA_R0_R2	16,79013443	-78,4261322	-946,3046265	-0,136127
Cloud_Mesh_Distance_BA_R0_R2	17,31746101	-78,43685913	-946,3724976	-0,101091
Cloud_Mesh_Distance_BA_R0_R2	17,26137543	-78,58602905	-946,3837891	-0,097653
Cloud_Mesh_Distance_BA_R0_R2	17,05692291	-78,60406494	-946,3664551	-0,112527
Cloud_Mesh_Distance_BA_R0_R2	17,41045189	-78,4773407	-946,3803101	-0,092463
Cloud_Mesh_Distance_BA_R0_R2	17,65969849	-78,42140198	-946,3721924	-0,07008
Cloud_Mesh_Distance_BA_R0_R2	17,85565758	-78,66390991	-946,3850708	-0,041374
Cloud_Mesh_Distance_BA_R0_R2	17,7440567	-78,62893677	-946,3892212	-0,052243
Cloud_Mesh_Distance_BA_R0_R2	17,86740494	-78,61268616	-946,3814087	-0,042842
Cloud_Mesh_Distance_BA_R0_R2	17,92025757	-78,61398315	-946,3777466	-0,037318
Cloud_Mesh_Distance_BA_R0_R2	18,00864983	-78,63439941	-946,3721924	-0,027573
Cloud_Mesh_Distance_BA_R0_R2	18,2075882	-78,51698303	-946,3404541	-0,016969
Cloud_Mesh_Distance_BA_R0_R2	18,04598427	-78,43475342	-946,3495483	-0,033812
Cloud_Mesh_Distance_BA_R0_R2	18,37320709	-78,58082581	-946,3208008	-0,005689
Cloud_Mesh_Distance_BA_R0_R2	18,20456886	-78,70439148	-946,3560181	-0,008985
Cloud_Mesh_Distance_BA_R0_R2	18,53354073	-78,62731934	-946,2906494	-0,001967
Cloud_Mesh_Distance_BA_R0_R2	18,37611961	-78,75704956	-946,331665	-0,000435
Cloud_Mesh_Distance_BA_R0_R2	16,83891106	-78,25692749	-946,2878418	-0,144688
Cloud_Mesh_Distance_BA_R0_R2	17,16753578	-78,40318298	-946,3581543	-0,118159
Cloud_Mesh_Distance_BA_R0_R2	16,97050858	-78,3273468	-946,3245239	-0,13571
Cloud_Mesh_Distance_BA_R0_R2	17,33545876	-78,35548401	-946,3626099	-0,104182
Cloud_Mesh_Distance_BA_R0_R2	17,34337616	-78,24201965	-946,3428345	-0,114315
Cloud_Mesh_Distance_BA_R0_R2	17,13760757	-78,1865387	-946,3197022	-0,132801
Cloud_Mesh_Distance_BA_R0_R2	17,52504539	-78,27560425	-946,3519898	-0,093268
Cloud_Mesh_Distance_BA_R0_R2	17,67695618	-78,21221924	-946,3361206	-0,083195
Cloud_Mesh_Distance_BA_R0_R2	17,88207626	-78,52098083	-946,3718872	-0,045501
Cloud_Mesh_Distance_BA_R0_R2	17,88063622	-78,31005859	-946,3440552	-0,056855
Cloud_Mesh_Distance_BA_R0_R2	16,97777557	-78,18621826	-946,2993774	-0,141068
Cloud_Mesh_Distance_BA_R0_R2	17,29237175	-78,07817078	-946,3051148	-0,12414
Cloud_Mesh_Distance_BA_R0_R2	17,82553291	-78,12089539	-946,3080444	-0,072314
Cloud_Mesh_Distance_BA_R0_R2	17,49030304	-78,10450745	-946,3152466	-0,106568
Cloud_Mesh_Distance_BA_R0_R2	17,98251724	-78,18354797	-946,3115234	-0,052339

Cloud_Mesh_Distance_BA_R0_R2	17,66018295	-78,06057739	-946,3005981	-0,094661
Cloud_Mesh_Distance_BA_R0_R2	17,15395927	-81,8187561	-946,09021	-0,147137
Cloud_Mesh_Distance_BA_R0_R2	16,74290466	-81,70066833	-946,019104	0,293208
Cloud_Mesh_Distance_BA_R0_R2	16,94509125	-81,78329468	-946,0715332	0,225754
Cloud_Mesh_Distance_BA_R0_R2	17,52288055	-81,73678589	-946,0524902	0,037962
Cloud_Mesh_Distance_BA_R0_R2	17,34976196	-81,85931396	-946,0979614	-0,135847
Cloud_Mesh_Distance_BA_R0_R2	17,70649529	-81,77510071	-946,0514526	-0,108107
Cloud_Mesh_Distance_BA_R0_R2	17,86470604	-81,81665039	-946,0515137	-0,185856
Cloud_Mesh_Distance_BA_R0_R2	18,07391357	-81,78294373	-946,0371094	-0,152058
Cloud_Mesh_Distance_BA_R0_R2	18,47303963	-81,83512878	-946,0533447	-0,274937
Cloud_Mesh_Distance_BA_R0_R2	16,67604446	-81,51699829	-945,9370728	0,285415
Cloud_Mesh_Distance_BA_R0_R2	17,128685	-81,64701843	-946,0324707	0,072136
Cloud_Mesh_Distance_BA_R0_R2	16,95396423	-81,62031555	-946,0141602	0,143122
Cloud_Mesh_Distance_BA_R0_R2	17,09711647	-81,48646545	-945,9824219	0,15715
Cloud_Mesh_Distance_BA_R0_R2	16,8866787	-81,48085022	-945,9589233	0,180971
Cloud_Mesh_Distance_BA_R0_R2	17,33116913	-81,68397522	-946,045105	0,049043
Cloud_Mesh_Distance_BA_R0_R2	17,53562737	-81,55561829	-946,0076294	0,061265
Cloud_Mesh_Distance_BA_R0_R2	17,68661118	-81,62944031	-946,0188599	0,011198
Cloud_Mesh_Distance_BA_R0_R2	18,02889824	-81,6186676	-946,0073853	-0,058641
Cloud_Mesh_Distance_BA_R0_R2	18,30047798	-81,75941467	-946,0343018	-0,14463
Cloud_Mesh_Distance_BA_R0_R2	18,12139702	-81,60105896	-946,0038452	-0,07973
Cloud_Mesh_Distance_BA_R0_R2	18,67811012	-81,6817627	-946,036438	-0,194021
Cloud_Mesh_Distance_BA_R0_R2	19,12729454	-81,88304138	-946,0847168	-0,354644
Cloud_Mesh_Distance_BA_R0_R2	19,09289742	-81,72233582	-946,0586548	-0,236461
Cloud_Mesh_Distance_BA_R0_R2	18,92622375	-81,88354492	-946,0799561	-0,353169
Cloud_Mesh_Distance_BA_R0_R2	18,88441467	-81,72073364	-946,052063	-0,2453
Cloud_Mesh_Distance_BA_R0_R2	19,30190468	-81,72935486	-946,0622559	-0,25696
Cloud_Mesh_Distance_BA_R0_R2	19,49728012	-81,76351929	-946,0631104	-0,24684
Cloud_Mesh_Distance_BA_R0_R2	19,96840668	-81,88977051	-946,0472412	-0,405991
Cloud_Mesh_Distance_BA_R0_R2	20,36691284	-81,89320374	-945,975769	-0,34588
Cloud_Mesh_Distance_BA_R0_R2	20,17522049	-81,8992157	-946,0178223	-0,405908
Cloud_Mesh_Distance_BA_R0_R2	16,736063	-81,27471924	-945,866394	0,326491
Cloud_Mesh_Distance_BA_R0_R2	17,32764626	-81,48623657	-945,9918823	0,125139
Cloud_Mesh_Distance_BA_R0_R2	17,51279831	-81,24589539	-945,9518433	0,123983
Cloud_Mesh_Distance_BA_R0_R2	17,56988525	-81,35487366	-945,9690552	0,097666
Cloud_Mesh_Distance_BA_R0_R2	17,79459381	-81,44517517	-945,9829712	0,023792
Cloud_Mesh_Distance_BA_R0_R2	17,98898697	-81,54820251	-945,9958496	-0,040338
Cloud_Mesh_Distance_BA_R0_R2	17,88410187	-81,63871765	-946,0133667	-0,04352
Cloud_Mesh_Distance_BA_R0_R2	18,2629528	-81,58236694	-946,0039063	-0,103177
Cloud_Mesh_Distance_BA_R0_R2	18,16244125	-81,49382019	-945,989502	-0,073549
Cloud_Mesh_Distance_BA_R0_R2	18,4718132	-81,62329102	-946,017395	-0,143995
Cloud_Mesh_Distance_BA_R0_R2	19,04498482	-81,58226013	-946,0363159	-0,19759
Cloud_Mesh_Distance_BA_R0_R2	18,87707901	-81,53305054	-946,022583	-0,18553
Cloud_Mesh_Distance_BA_R0_R2	19,49362946	-81,58226013	-946,036499	-0,19695
Cloud_Mesh_Distance_BA_R0_R2	19,67239571	-81,79768372	-946,0592651	-0,29548
Cloud_Mesh_Distance_BA_R0_R2	19,67792511	-81,63288879	-946,03302	-0,201065
Cloud_Mesh_Distance_BA_R0_R2	19,81651688	-81,69667053	-946,0285034	-0,238623
Cloud_Mesh_Distance_BA_R0_R2	19,83167267	-81,83158875	-946,0513916	-0,343551
Cloud_Mesh_Distance_BA_R0_R2	16,78229332	-81,37361145	-945,90802	0,260254
Cloud_Mesh_Distance_BA_R0_R2	17,63009262	-81,20910645	-945,9526367	0,090167

Cloud_Mesh_Distance_BA_R0_R2	17,78882217	-81,21470642	-945,9577026	0,046352
Cloud_Mesh_Distance_BA_R0_R2	18,0701046	-81,38104248	-945,9747925	-0,040293
Cloud_Mesh_Distance_BA_R0_R2	18,25831604	-81,40409851	-945,9810181	-0,085926
Cloud_Mesh_Distance_BA_R0_R2	18,61119461	-81,29545593	-945,984314	-0,139014
Cloud_Mesh_Distance_BA_R0_R2	18,49376297	-81,24702454	-945,9770508	-0,11889
Cloud_Mesh_Distance_BA_R0_R2	18,44548416	-81,40834045	-945,987915	-0,123072
Cloud_Mesh_Distance_BA_R0_R2	18,34592819	-81,24162292	-945,9718018	-0,089863
Cloud_Mesh_Distance_BA_R0_R2	18,67350769	-81,45695496	-946,0029907	-0,161348
Cloud_Mesh_Distance_BA_R0_R2	18,88777161	-81,3371582	-945,9991455	-0,171321
Cloud_Mesh_Distance_BA_R0_R2	19,09258842	-81,40184021	-946,0149536	-0,18353
Cloud_Mesh_Distance_BA_R0_R2	19,27602768	-81,51953125	-946,0325928	-0,193619
Cloud_Mesh_Distance_BA_R0_R2	19,50065231	-81,41210938	-946,0151978	-0,181268
Cloud_Mesh_Distance_BA_R0_R2	20,0074749	-81,70652771	-946,0031738	-0,251892
Cloud_Mesh_Distance_BA_R0_R2	20,14195633	-81,5960083	-945,9539185	-0,184754
Cloud_Mesh_Distance_BA_R0_R2	17,71115112	-81,11793518	-945,9503784	0,068607
Cloud_Mesh_Distance_BA_R0_R2	17,82429504	-81,06672668	-945,9545898	0,038192
Cloud_Mesh_Distance_BA_R0_R2	17,99861908	-81,26957703	-945,9658203	-0,012229
Cloud_Mesh_Distance_BA_R0_R2	18,17327118	-81,29866028	-945,9700928	-0,056367
Cloud_Mesh_Distance_BA_R0_R2	18,20598221	-81,10227966	-945,9653931	-0,049814
Cloud_Mesh_Distance_BA_R0_R2	18,82618713	-81,20907593	-945,9874268	-0,158193
Cloud_Mesh_Distance_BA_R0_R2	18,9015007	-81,11268616	-945,9863892	-0,150862
Cloud_Mesh_Distance_BA_R0_R2	19,27515221	-81,35247803	-946,0134277	-0,18137
Cloud_Mesh_Distance_BA_R0_R2	19,05522156	-81,23738098	-945,998169	-0,170391
Cloud_Mesh_Distance_BA_R0_R2	19,76766777	-81,29107666	-945,9787598	-0,159976
Cloud_Mesh_Distance_BA_R0_R2	19,86690521	-81,22483826	-945,9555054	-0,151385
Cloud_Mesh_Distance_BA_R0_R2	19,86782074	-81,35282898	-945,9698486	-0,162028
Cloud_Mesh_Distance_BA_R0_R2	19,69194794	-81,43928528	-946,0043945	-0,176224
Cloud_Mesh_Distance_BA_R0_R2	19,87664986	-81,53225708	-945,9936523	-0,175925
Cloud_Mesh_Distance_BA_R0_R2	20,04133606	-81,53196716	-945,9637451	-0,167934
Cloud_Mesh_Distance_BA_R0_R2	17,9858036	-81,04832458	-945,9603882	-0,00204
Cloud_Mesh_Distance_BA_R0_R2	18,19471931	-80,82658386	-945,982544	-0,04733
Cloud_Mesh_Distance_BA_R0_R2	18,94009018	-81,20278931	-945,991394	-0,163881
Cloud_Mesh_Distance_BA_R0_R2	19,02350235	-81,10787964	-945,991272	-0,152874
Cloud_Mesh_Distance_BA_R0_R2	19,15571976	-81,1267395	-945,9953003	-0,159616
Cloud_Mesh_Distance_BA_R0_R2	19,24431229	-80,91189575	-945,9944458	-0,129407
Cloud_Mesh_Distance_BA_R0_R2	19,3268013	-80,92619324	-945,993103	-0,129592
Cloud_Mesh_Distance_BA_R0_R2	17,87153244	-80,59632874	-946,0116577	-0,020679
Cloud_Mesh_Distance_BA_R0_R2	18,06425476	-80,61543274	-946,0160523	-0,030594
Cloud_Mesh_Distance_BA_R0_R2	17,88074112	-80,72906494	-945,9848023	-0,006519
Cloud_Mesh_Distance_BA_R0_R2	18,00339127	-80,78605652	-945,9818115	-0,017048
Cloud_Mesh_Distance_BA_R0_R2	18,40540695	-80,81216431	-945,9876099	-0,071531
Cloud_Mesh_Distance_BA_R0_R2	18,54127502	-80,75926208	-945,9935913	-0,086853
Cloud_Mesh_Distance_BA_R0_R2	18,67443085	-80,72000122	-945,998291	-0,09513
Cloud_Mesh_Distance_BA_R0_R2	19,00919914	-80,6672821	-946,0021973	-0,105187
Cloud_Mesh_Distance_BA_R0_R2	19,32622337	-80,84327698	-945,9935913	-0,119026
Cloud_Mesh_Distance_BA_R0_R2	16,91165543	-80,31607056	-945,9602661	-0,063883
Cloud_Mesh_Distance_BA_R0_R2	17,19017601	-80,26774597	-946,0447388	-0,057284
Cloud_Mesh_Distance_BA_R0_R2	17,57639503	-80,39033508	-946,0529175	-0,032707
Cloud_Mesh_Distance_BA_R0_R2	17,72120857	-80,52095032	-946,0235596	-0,013326
Cloud_Mesh_Distance_BA_R0_R2	18,32526398	-80,46853638	-946,0514526	-0,048278

Cloud_Mesh_Distance_BA_R0_R2	18,28218842	-80,58564758	-946,0244751	-0,047053
Cloud_Mesh_Distance_BA_R0_R2	18,44617462	-80,64967346	-946,0108643	-0,071022
Cloud_Mesh_Distance_BA_R0_R2	18,44864273	-80,45623779	-946,0495606	-0,058855
Cloud_Mesh_Distance_BA_R0_R2	18,61096573	-80,63739014	-946,0093994	-0,081892
Cloud_Mesh_Distance_BA_R0_R2	19,11071014	-80,7303009	-945,9992065	-0,109453
Cloud_Mesh_Distance_BA_R0_R2	19,2241478	-80,78852844	-945,9962769	-0,113194
Cloud_Mesh_Distance_BA_R0_R2	19,42179108	-80,68367004	-945,9911499	-0,097653
Cloud_Mesh_Distance_BA_R0_R2	19,32907104	-80,7321167	-945,9957886	-0,105328
Cloud_Mesh_Distance_BA_R0_R2	19,74378967	-80,59254456	-945,946228	-0,086244
Cloud_Mesh_Distance_BA_R0_R2	19,76712227	-80,60858154	-945,9415894	-0,087848
Cloud_Mesh_Distance_BA_R0_R2	19,78988647	-80,63536072	-945,937561	-0,090309
Cloud_Mesh_Distance_BA_R0_R2	16,53106117	-80,07937622	-945,9593506	-0,100608
Cloud_Mesh_Distance_BA_R0_R2	16,60826874	-79,96559143	-946,0448608	-0,092742
Cloud_Mesh_Distance_BA_R0_R2	16,83594131	-80,21054077	-945,9882813	-0,080993
Cloud_Mesh_Distance_BA_R0_R2	16,98897552	-80,05288696	-946,1002808	-0,080537
Cloud_Mesh_Distance_BA_R0_R2	16,9935379	-80,20077515	-946,0338135	-0,07204
Cloud_Mesh_Distance_BA_R0_R2	17,37763786	-80,32704163	-946,052002	-0,042599
Cloud_Mesh_Distance_BA_R0_R2	17,68866539	-80,2664032	-946,1102905	-0,032934
Cloud_Mesh_Distance_BA_R0_R2	17,76322174	-80,37045288	-946,0765381	-0,029649
Cloud_Mesh_Distance_BA_R0_R2	17,87516975	-80,23577881	-946,1331787	-0,025055
Cloud_Mesh_Distance_BA_R0_R2	17,95100403	-80,4354248	-946,062561	-0,028876
Cloud_Mesh_Distance_BA_R0_R2	18,10694122	-80,38960266	-946,0791626	-0,033891
Cloud_Mesh_Distance_BA_R0_R2	18,37870407	-80,41447449	-946,0634766	-0,050006
Cloud_Mesh_Distance_BA_R0_R2	18,19783401	-80,33537292	-946,0957642	-0,029722
Cloud_Mesh_Distance_BA_R0_R2	18,20738029	-80,25971985	-946,1207886	-0,026078
Cloud_Mesh_Distance_BA_R0_R2	18,28913498	-80,26924133	-946,1136475	-0,027469
Cloud_Mesh_Distance_BA_R0_R2	18,64898491	-80,47288513	-946,0358276	-0,071977
Cloud_Mesh_Distance_BA_R0_R2	18,62131119	-80,24963379	-946,0866699	-0,047146
Cloud_Mesh_Distance_BA_R0_R2	19,10752296	-80,62779236	-946,0042114	-0,100572
Cloud_Mesh_Distance_BA_R0_R2	18,8406105	-80,28297424	-946,0532227	-0,067122
Cloud_Mesh_Distance_BA_R0_R2	19,22360229	-80,65237427	-946,0003052	-0,09954
Cloud_Mesh_Distance_BA_R0_R2	19,15445518	-80,52058411	-946,0068359	-0,091007
Cloud_Mesh_Distance_BA_R0_R2	19,68946075	-80,45202637	-945,9489746	-0,079062
Cloud_Mesh_Distance_BA_R0_R2	16,46044922	-79,96812439	-945,9923706	-0,095347
Cloud_Mesh_Distance_BA_R0_R2	16,57463455	-79,76756287	-946,125	-0,087236
Cloud_Mesh_Distance_BA_R0_R2	16,75073624	-79,86997986	-946,1290894	-0,090541
Cloud_Mesh_Distance_BA_R0_R2	16,82334328	-80,06030273	-946,0587158	-0,086175
Cloud_Mesh_Distance_BA_R0_R2	16,90893364	-79,9445343	-946,1322022	-0,085437
Cloud_Mesh_Distance_BA_R0_R2	17,14536285	-80,10681152	-946,1069336	-0,068414
Cloud_Mesh_Distance_BA_R0_R2	17,52793694	-80,2207489	-946,1143799	-0,04242
Cloud_Mesh_Distance_BA_R0_R2	17,33600235	-80,16548157	-946,1120606	-0,058201
Cloud_Mesh_Distance_BA_R0_R2	17,88547516	-80,07319641	-946,1958008	-0,02002
Cloud_Mesh_Distance_BA_R0_R2	17,70303917	-80,11006165	-946,1737671	-0,033235
Cloud_Mesh_Distance_BA_R0_R2	18,09103584	-80,27558899	-946,1195679	-0,028387
Cloud_Mesh_Distance_BA_R0_R2	18,39188004	-80,1864624	-946,1329956	-0,027689
Cloud_Mesh_Distance_BA_R0_R2	18,24795723	-80,146698	-946,159668	-0,017674
Cloud_Mesh_Distance_BA_R0_R2	18,54271317	-80,0813446	-946,1453857	-0,022684
Cloud_Mesh_Distance_BA_R0_R2	18,94564056	-80,36677551	-946,0305786	-0,080086
Cloud_Mesh_Distance_BA_R0_R2	18,9999733	-80,25263977	-946,0372925	-0,077229
Cloud_Mesh_Distance_BA_R0_R2	18,99792671	-80,12072754	-946,0532227	-0,069403

Cloud_Mesh_Distance_BA_R0_R2	19,66771698	-80,42234802	-945,9520874	-0,078081
Cloud_Mesh_Distance_BA_R0_R2	16,25283813	-79,60368347	-946,0722656	-0,074638
Cloud_Mesh_Distance_BA_R0_R2	16,53141022	-79,6809845	-946,1459351	-0,083206
Cloud_Mesh_Distance_BA_R0_R2	16,45002365	-79,83628845	-946,0543213	-0,088713
Cloud_Mesh_Distance_BA_R0_R2	16,58322144	-79,68678284	-946,1592407	-0,084404
Cloud_Mesh_Distance_BA_R0_R2	17,08134651	-79,93202209	-946,1686401	-0,078953
Cloud_Mesh_Distance_BA_R0_R2	17,09029007	-79,77403259	-946,2282105	-0,082436
Cloud_Mesh_Distance_BA_R0_R2	17,29063416	-79,99916077	-946,1744995	-0,066608
Cloud_Mesh_Distance_BA_R0_R2	17,45370102	-79,8833313	-946,236023	-0,053915
Cloud_Mesh_Distance_BA_R0_R2	17,49136162	-80,05508423	-946,1766357	-0,049791
Cloud_Mesh_Distance_BA_R0_R2	17,81410789	-79,97221375	-946,2304688	-0,021067
Cloud_Mesh_Distance_BA_R0_R2	18,55629349	-79,93562317	-946,1813965	-0,009782
Cloud_Mesh_Distance_BA_R0_R2	18,71780396	-80,03730774	-946,1226807	-0,036413
Cloud_Mesh_Distance_BA_R0_R2	18,84808731	-80,08091736	-946,0870361	-0,052053
Cloud_Mesh_Distance_BA_R0_R2	16,15535355	-79,50152588	-946,0602417	-0,069709
Cloud_Mesh_Distance_BA_R0_R2	16,52794647	-79,64231873	-946,1591797	-0,080421
Cloud_Mesh_Distance_BA_R0_R2	16,43046761	-79,64375305	-946,1271362	-0,080002
Cloud_Mesh_Distance_BA_R0_R2	16,70650864	-79,48802185	-946,2487183	-0,085106
Cloud_Mesh_Distance_BA_R0_R2	16,69683647	-79,66995239	-946,1935425	-0,086542
Cloud_Mesh_Distance_BA_R0_R2	16,55915451	-79,56938171	-946,1919556	-0,07734
Cloud_Mesh_Distance_BA_R0_R2	16,8742485	-79,61428833	-946,2452393	-0,089122
Cloud_Mesh_Distance_BA_R0_R2	16,77600479	-79,57023621	-946,239563	-0,087924
Cloud_Mesh_Distance_BA_R0_R2	16,91302109	-79,78582764	-946,1958008	-0,0869
Cloud_Mesh_Distance_BA_R0_R2	17,01749039	-79,6620636	-946,2532349	-0,085813
Cloud_Mesh_Distance_BA_R0_R2	18,87488937	-79,86045837	-946,1190796	-0,041546
Cloud_Mesh_Distance_BA_R0_R2	18,68679047	-79,90570068	-946,1598511	-0,021757
Cloud_Mesh_Distance_BA_R0_R2	18,96693802	-79,6869812	-946,1150513	-0,046504
Cloud_Mesh_Distance_BA_R0_R2	18,94768524	-79,74581909	-946,1135864	-0,04649
Cloud_Mesh_Distance_BA_R0_R2	19,02519798	-79,9334259	-946,0693359	-0,062695
Cloud_Mesh_Distance_BA_R0_R2	19,16246986	-80,01579285	-946,0303955	-0,074561
Cloud_Mesh_Distance_BA_R0_R2	16,26698112	-79,28607178	-946,1602783	-0,069886
Cloud_Mesh_Distance_BA_R0_R2	16,13207626	-79,3184967	-946,0927734	-0,064667
Cloud_Mesh_Distance_BA_R0_R2	16,21744919	-79,13560486	-946,156189	-0,071835
Cloud_Mesh_Distance_BA_R0_R2	16,07344055	-79,13819885	-946,086731	-0,06883
Cloud_Mesh_Distance_BA_R0_R2	16,28694916	-79,45153809	-946,1333008	-0,070478
Cloud_Mesh_Distance_BA_R0_R2	16,44420242	-79,46224976	-946,1882935	-0,071266
Cloud_Mesh_Distance_BA_R0_R2	16,56623459	-79,41976929	-946,2332153	-0,077428
Cloud_Mesh_Distance_BA_R0_R2	18,89957809	-79,74279785	-946,1292114	-0,039523
Cloud_Mesh_Distance_BA_R0_R2	18,91873169	-79,68397522	-946,1307373	-0,040324
Cloud_Mesh_Distance_BA_R0_R2	18,97098351	-79,56492615	-946,1275635	-0,041458
Cloud_Mesh_Distance_BA_R0_R2	18,80172157	-79,70780945	-946,1643677	-0,022274
Cloud_Mesh_Distance_BA_R0_R2	18,79425621	-79,48127747	-946,1959839	-0,015526
Cloud_Mesh_Distance_BA_R0_R2	19,04395676	-79,7243042	-946,0872192	-0,056321
Cloud_Mesh_Distance_BA_R0_R2	15,83843899	-79,04466248	-945,9459229	-0,063682
Cloud_Mesh_Distance_BA_R0_R2	15,84045219	-79,01852417	-945,9476929	-0,064323
Cloud_Mesh_Distance_BA_R0_R2	15,8601284	-79,02915955	-945,9610596	-0,066256
Cloud_Mesh_Distance_BA_R0_R2	16,28310776	-78,94927979	-946,1889648	-0,09164
Cloud_Mesh_Distance_BA_R0_R2	16,34081268	-79,06333923	-946,2110596	-0,086909
Cloud_Mesh_Distance_BA_R0_R2	16,39995956	-78,97099304	-946,2340698	-0,094019
Cloud_Mesh_Distance_BA_R0_R2	17,01823044	-79,3349762	-946,3247681	-0,092988

Cloud_Mesh_Distance_BA_R0_R2	18,44330025	-79,34333801	-946,3079224	0,014436
Cloud_Mesh_Distance_BA_R0_R2	18,50686455	-79,31564331	-946,2949829	0,011056
Cloud_Mesh_Distance_BA_R0_R2	18,62710571	-79,43638611	-946,2521362	0,003787
Cloud_Mesh_Distance_BA_R0_R2	19,1274929	-79,39433289	-946,0848389	-0,061844
Cloud_Mesh_Distance_BA_R0_R2	19,12653542	-79,56048584	-946,0739746	-0,064756
Cloud_Mesh_Distance_BA_R0_R2	15,84106731	-78,97651672	-945,947937	-0,064192
Cloud_Mesh_Distance_BA_R0_R2	16,18638992	-78,81970215	-946,1376953	-0,10077
Cloud_Mesh_Distance_BA_R0_R2	16,33511543	-78,79289246	-946,2019043	-0,109458
Cloud_Mesh_Distance_BA_R0_R2	18,3949604	-79,21434021	-946,3261719	0,013758
Cloud_Mesh_Distance_BA_R0_R2	18,59072113	-79,2926178	-946,2747803	0,006326
Cloud_Mesh_Distance_BA_R0_R2	18,49571419	-79,24014282	-946,3023071	0,011069
Cloud_Mesh_Distance_BA_R0_R2	18,75207901	-79,28164673	-946,2261963	-0,00991
Cloud_Mesh_Distance_BA_R0_R2	18,86608696	-79,11161804	-946,1939087	-0,024282
Cloud_Mesh_Distance_BA_R0_R2	18,96305466	-79,28253174	-946,1518555	-0,036301
Cloud_Mesh_Distance_BA_R0_R2	19,11553574	-79,06439209	-946,1025391	-0,052736
Cloud_Mesh_Distance_BA_R0_R2	19,05114365	-79,13708496	-946,125061	-0,047977
Cloud_Mesh_Distance_BA_R0_R2	16,3263588	-78,65081787	-946,1830444	-0,123786
Cloud_Mesh_Distance_BA_R0_R2	16,48096085	-78,68106079	-946,246582	-0,120674
Cloud_Mesh_Distance_BA_R0_R2	16,4224968	-78,85142517	-946,2382813	-0,1038
Cloud_Mesh_Distance_BA_R0_R2	18,65894699	-79,09996033	-946,2645264	-0,001542
Cloud_Mesh_Distance_BA_R0_R2	18,98192215	-79,1118927	-946,1515503	-0,038918
Cloud_Mesh_Distance_BA_R0_R2	18,9759407	-78,99694824	-946,1570435	-0,036387
Cloud_Mesh_Distance_BA_R0_R2	19,14233208	-79,19247437	-946,088501	-0,058463
Cloud_Mesh_Distance_BA_R0_R2	16,17177963	-78,644104	-946,1099243	-0,116954
Cloud_Mesh_Distance_BA_R0_R2	16,62475395	-78,6516571	-946,2878418	-0,126104
Cloud_Mesh_Distance_BA_R0_R2	16,62923241	-78,52960205	-946,2763062	-0,136405
Cloud_Mesh_Distance_BA_R0_R2	17,89601326	-78,63848877	-946,3813477	-0,038426
Cloud_Mesh_Distance_BA_R0_R2	17,90851402	-78,6650238	-946,3814087	-0,036485
Cloud_Mesh_Distance_BA_R0_R2	18,68219185	-78,87045288	-946,2589722	-0,006285
Cloud_Mesh_Distance_BA_R0_R2	18,53420067	-78,80705261	-946,2994385	0,000257
Cloud_Mesh_Distance_BA_R0_R2	18,85272598	-78,91738892	-946,2026978	-0,022014
Cloud_Mesh_Distance_BA_R0_R2	18,81386948	-78,75463867	-946,2147827	-0,015679
Cloud_Mesh_Distance_BA_R0_R2	19,18388367	-79,07225037	-946,0755615	-0,06046
Cloud_Mesh_Distance_BA_R0_R2	16,37057495	-78,26113892	-946,1398926	-0,137936
Cloud_Mesh_Distance_BA_R0_R2	16,48769379	-78,49713135	-946,2258301	-0,137225
Cloud_Mesh_Distance_BA_R0_R2	16,31741333	-78,46063232	-946,1506958	-0,135524
Cloud_Mesh_Distance_BA_R0_R2	16,66455078	-78,35818481	-946,2619629	-0,144845
Cloud_Mesh_Distance_BA_R0_R2	17,39555931	-78,39389038	-946,3699341	-0,097244
Cloud_Mesh_Distance_BA_R0_R2	17,45964622	-78,39964294	-946,3719482	-0,091465
Cloud_Mesh_Distance_BA_R0_R2	17,51331711	-78,45428467	-946,3787842	-0,083995
Cloud_Mesh_Distance_BA_R0_R2	17,5228157	-78,37130737	-946,3681641	-0,086072
Cloud_Mesh_Distance_BA_R0_R2	18,69070435	-78,68222046	-946,2512207	-0,006994
Cloud_Mesh_Distance_BA_R0_R2	18,68547249	-78,47544861	-946,2363281	-0,00923
Cloud_Mesh_Distance_BA_R0_R2	18,86882401	-78,58328247	-946,1861572	-0,022155
Cloud_Mesh_Distance_BA_R0_R2	18,95926476	-78,74403381	-946,161499	-0,031996
Cloud_Mesh_Distance_BA_R0_R2	16,25733376	-78,21630859	-946,0803833	-0,121324
Cloud_Mesh_Distance_BA_R0_R2	16,51579857	-78,3031311	-946,2041016	-0,14783
Cloud_Mesh_Distance_BA_R0_R2	16,72162819	-78,14950562	-946,2373047	-0,149151
Cloud_Mesh_Distance_BA_R0_R2	17,43442535	-78,33343506	-946,3621826	-0,097827
Cloud_Mesh_Distance_BA_R0_R2	18,05649948	-78,28794861	-946,3247681	-0,041214

Cloud_Mesh_Distance_BA_R0_R2	18,20133591	-78,34233093	-946,3186035	-0,02326
Cloud_Mesh_Distance_BA_R0_R2	18,37037277	-78,41030884	-946,3026733	-0,01062
Cloud_Mesh_Distance_BA_R0_R2	18,50795555	-78,4793396	-946,2832031	-0,00575
Cloud_Mesh_Distance_BA_R0_R2	18,72935295	-78,2999115	-946,197937	-0,016854
Cloud_Mesh_Distance_BA_R0_R2	19,01349068	-78,479599	-946,121582	-0,035347
Cloud_Mesh_Distance_BA_R0_R2	16,52970505	-78,10276794	-946,1697998	-0,13607
Cloud_Mesh_Distance_BA_R0_R2	16,89644623	-78,11801147	-946,2703247	-0,147356
Cloud_Mesh_Distance_BA_R0_R2	16,897686	-77,95227051	-946,2260742	-0,145131
Cloud_Mesh_Distance_BA_R0_R2	17,03254318	-78,06660461	-946,2793579	-0,142377
Cloud_Mesh_Distance_BA_R0_R2	17,16341782	-78,04344177	-946,2875366	-0,135684
Cloud_Mesh_Distance_BA_R0_R2	17,10717201	-77,94184875	-946,2521362	-0,136515
Cloud_Mesh_Distance_BA_R0_R2	18,14671898	-78,16851807	-946,2911377	-0,038915
Cloud_Mesh_Distance_BA_R0_R2	18,34628677	-78,23469543	-946,2769775	-0,020988
Cloud_Mesh_Distance_BA_R0_R2	18,49154663	-78,12104797	-946,2236939	-0,01783
Cloud_Mesh_Distance_BA_R0_R2	18,54873466	-78,31118774	-946,2495728	-0,011764
Cloud_Mesh_Distance_BA_R0_R2	18,99005127	-78,21627808	-946,0863648	-0,038233
Cloud_Mesh_Distance_BA_R0_R2	18,88360786	-78,3662262	-946,156311	-0,026262
Cloud_Mesh_Distance_BA_R0_R2	18,83633041	-78,19134521	-946,1408081	-0,024875
Cloud_Mesh_Distance_BA_R0_R2	16,60651779	-77,79200745	-946,1069336	-0,124645
Cloud_Mesh_Distance_BA_R0_R2	16,58264732	-77,91914368	-946,1405029	-0,129827
Cloud_Mesh_Distance_BA_R0_R2	16,69705009	-77,98486328	-946,1923218	-0,139781
Cloud_Mesh_Distance_BA_R0_R2	16,74363327	-77,82142639	-946,1535034	-0,129699
Cloud_Mesh_Distance_BA_R0_R2	17,23659897	-77,96800232	-946,270874	-0,135461
Cloud_Mesh_Distance_BA_R0_R2	17,37182236	-77,95477295	-946,2717896	-0,125728
Cloud_Mesh_Distance_BA_R0_R2	17,75296783	-77,93710327	-946,2612305	-0,09108
Cloud_Mesh_Distance_BA_R0_R2	17,54811478	-77,93922424	-946,2683106	-0,112058
Cloud_Mesh_Distance_BA_R0_R2	18,13991547	-78,04386902	-946,2617798	-0,045797
Cloud_Mesh_Distance_BA_R0_R2	17,98523331	-78,0296936	-946,2728882	-0,064698
Cloud_Mesh_Distance_BA_R0_R2	18,13085747	-77,91812134	-946,2264404	-0,051546
Cloud_Mesh_Distance_BA_R0_R2	18,29698181	-77,90301514	-946,1990967	-0,039517
Cloud_Mesh_Distance_BA_R0_R2	18,28867149	-78,05067444	-946,242981	-0,032261
Cloud_Mesh_Distance_BA_R0_R2	18,67245293	-78,16221619	-946,1875	-0,017416
Cloud_Mesh_Distance_BA_R0_R2	19,07822609	-78,10392761	-946,0176392	-0,056594
Cloud_Mesh_Distance_BA_R0_R2	18,9406395	-78,06880188	-946,0682373	-0,041287
Cloud_Mesh_Distance_BA_R0_R2	16,54218102	-77,69487	-946,0483398	-0,11207
Cloud_Mesh_Distance_BA_R0_R2	16,680233	-77,69955444	-946,0906982	-0,118033
Cloud_Mesh_Distance_BA_R0_R2	17,44285774	-77,71374512	-946,1859741	-0,133701
Cloud_Mesh_Distance_BA_R0_R2	17,38895607	-77,63296509	-946,1508789	-0,137157
Cloud_Mesh_Distance_BA_R0_R2	17,59649277	-77,77333069	-946,2096558	-0,116882
Cloud_Mesh_Distance_BA_R0_R2	17,50458336	-77,63821411	-946,1554565	-0,129258
Cloud_Mesh_Distance_BA_R0_R2	17,62059212	-77,64155579	-946,1571045	-0,118139
Cloud_Mesh_Distance_BA_R0_R2	17,75569534	-77,69900513	-946,1769409	-0,103933
Cloud_Mesh_Distance_BA_R0_R2	17,91356468	-77,68788147	-946,1655884	-0,083879
Cloud_Mesh_Distance_BA_R0_R2	17,90687561	-77,83302307	-946,2185059	-0,079353
Cloud_Mesh_Distance_BA_R0_R2	17,75063515	-77,8105011	-946,2189331	-0,100107
Cloud_Mesh_Distance_BA_R0_R2	18,22286224	-77,80487061	-946,1777954	-0,048785
Cloud_Mesh_Distance_BA_R0_R2	18,39511871	-77,7203064	-946,116333	-0,037933
Cloud_Mesh_Distance_BA_R0_R2	17,43086624	-77,55038452	-946,1137695	-0,137704
Cloud_Mesh_Distance_BA_R0_R2	18,40016365	-77,69824219	-946,105957	-0,039252
Cloud_Mesh_Distance_BA_R0_R2	18,41384125	-77,66397095	-946,0897827	-0,039937

Cloud_Mesh_Distance_BA_R0_R2	16,29750824	-81,30636597	-945,7252197	0,450885
Cloud_Mesh_Distance_BA_R0_R2	16,40357018	-81,36708069	-945,7973633	0,390295
Cloud_Mesh_Distance_BA_R0_R2	16,53300476	-81,50286865	-945,8980103	0,340443
Cloud_Mesh_Distance_BA_R0_R2	18,07191086	-81,5280304	-945,9924316	-0,057275
Cloud_Mesh_Distance_BA_R0_R2	20,52437782	-81,94700623	-945,9534302	-0,362902
Cloud_Mesh_Distance_BA_R0_R2	20,63703918	-81,81484985	-945,8790894	-0,219461
Cloud_Mesh_Distance_BA_R0_R2	20,43990517	-81,7746582	-945,9224243	-0,228579
Cloud_Mesh_Distance_BA_R0_R2	20,84978676	-81,92959595	-945,859375	-0,174035
Cloud_Mesh_Distance_BA_R0_R2	21,35631371	-81,99092102	-945,7199707	0,131459
Cloud_Mesh_Distance_BA_R0_R2	16,5025425	-81,41426086	-945,8519898	0,344507
Cloud_Mesh_Distance_BA_R0_R2	16,6336441	-81,39190674	-945,8812256	0,301126
Cloud_Mesh_Distance_BA_R0_R2	17,22429657	-81,20288086	-945,9251709	0,207451
Cloud_Mesh_Distance_BA_R0_R2	17,06482697	-81,33757019	-945,9381104	0,231556
Cloud_Mesh_Distance_BA_R0_R2	17,24997902	-81,34439087	-945,954773	0,183255
Cloud_Mesh_Distance_BA_R0_R2	17,40856171	-81,3303833	-945,9605103	0,146468
Cloud_Mesh_Distance_BA_R0_R2	18,02247238	-81,46708679	-945,9848023	-0,037568
Cloud_Mesh_Distance_BA_R0_R2	20,30897141	-81,59864807	-945,9109497	-0,161607
Cloud_Mesh_Distance_BA_R0_R2	20,29623032	-81,7709198	-945,9591065	-0,260036
Cloud_Mesh_Distance_BA_R0_R2	20,64845657	-81,66105652	-945,8234253	-0,135667
Cloud_Mesh_Distance_BA_R0_R2	20,78378868	-81,74269104	-945,8074341	-0,118151
Cloud_Mesh_Distance_BA_R0_R2	21,01847839	-81,95426941	-945,8197022	-0,117009
Cloud_Mesh_Distance_BA_R0_R2	21,1846199	-82,02638245	-945,7989502	-0,241941
Cloud_Mesh_Distance_BA_R0_R2	16,5823307	-81,10058594	-945,7671509	0,277703
Cloud_Mesh_Distance_BA_R0_R2	16,39852715	-81,09388733	-945,6923218	0,276378
Cloud_Mesh_Distance_BA_R0_R2	16,43292046	-81,22412109	-945,7512207	0,378298
Cloud_Mesh_Distance_BA_R0_R2	16,58701515	-81,26618958	-945,8222656	0,36688
Cloud_Mesh_Distance_BA_R0_R2	16,82492256	-81,09083557	-945,8388062	0,252012
Cloud_Mesh_Distance_BA_R0_R2	16,8569622	-81,24946594	-945,8848877	0,315389
Cloud_Mesh_Distance_BA_R0_R2	16,9209671	-81,32524109	-945,9169312	0,263793
Cloud_Mesh_Distance_BA_R0_R2	17,06341171	-81,01916504	-945,8773193	0,183738
Cloud_Mesh_Distance_BA_R0_R2	17,02521896	-81,18229675	-945,8969727	0,25926
Cloud_Mesh_Distance_BA_R0_R2	17,39216805	-81,2199707	-945,9418945	0,158024
Cloud_Mesh_Distance_BA_R0_R2	17,3938427	-81,07885742	-945,9259033	0,140035
Cloud_Mesh_Distance_BA_R0_R2	17,22058487	-81,05227661	-945,9046631	0,170155
Cloud_Mesh_Distance_BA_R0_R2	17,5646553	-81,11595154	-945,9419556	0,102724
Cloud_Mesh_Distance_BA_R0_R2	17,9317646	-81,49952698	-945,9888916	-0,020506
Cloud_Mesh_Distance_BA_R0_R2	17,96461105	-81,40992737	-945,9780884	-0,015603
Cloud_Mesh_Distance_BA_R0_R2	18,09797096	-81,45222473	-945,9833984	-0,054956
Cloud_Mesh_Distance_BA_R0_R2	18,15407944	-81,40391541	-945,9782105	-0,06289
Cloud_Mesh_Distance_BA_R0_R2	18,74220848	-81,30314636	-945,9896851	-0,157048
Cloud_Mesh_Distance_BA_R0_R2	19,38999939	-81,36199951	-946,013794	-0,177408
Cloud_Mesh_Distance_BA_R0_R2	20,18718719	-81,70973206	-945,9690552	-0,242468
Cloud_Mesh_Distance_BA_R0_R2	20,1583519	-81,45462036	-945,921814	-0,163103
Cloud_Mesh_Distance_BA_R0_R2	20,29873657	-81,38807678	-945,8660889	-0,15971
Cloud_Mesh_Distance_BA_R0_R2	20,50911713	-81,64149475	-945,8615112	-0,148399
Cloud_Mesh_Distance_BA_R0_R2	16,84114456	-80,90498352	-945,8150024	0,13974
Cloud_Mesh_Distance_BA_R0_R2	17,56241417	-80,95394897	-945,9377441	0,079127
Cloud_Mesh_Distance_BA_R0_R2	17,38699341	-80,9284668	-945,9204102	0,101413
Cloud_Mesh_Distance_BA_R0_R2	17,73841095	-80,98817444	-945,9497681	0,050172
Cloud_Mesh_Distance_BA_R0_R2	18,37763786	-81,08955383	-945,9699097	-0,081219

Cloud_Mesh_Distance_BA_R0_R2	18,46535873	-81,1459198	-945,972229	-0,106596
Cloud_Mesh_Distance_BA_R0_R2	18,60204887	-81,14231873	-945,9768066	-0,12489
Cloud_Mesh_Distance_BA_R0_R2	18,51129913	-81,0105896	-945,9747315	-0,100752
Cloud_Mesh_Distance_BA_R0_R2	18,71054459	-81,20755005	-945,9827881	-0,143297
Cloud_Mesh_Distance_BA_R0_R2	19,38271523	-81,23506165	-946,0026856	-0,165913
Cloud_Mesh_Distance_BA_R0_R2	19,21870422	-81,23466492	-946,0025024	-0,169938
Cloud_Mesh_Distance_BA_R0_R2	19,49746323	-81,28466797	-946,0029297	-0,169267
Cloud_Mesh_Distance_BA_R0_R2	19,55938721	-81,12628174	-945,9888306	-0,146604
Cloud_Mesh_Distance_BA_R0_R2	19,75801277	-81,16165161	-945,9686279	-0,145908
Cloud_Mesh_Distance_BA_R0_R2	19,63346481	-81,28526306	-945,9934692	-0,162405
Cloud_Mesh_Distance_BA_R0_R2	20,01209259	-81,39118958	-945,9472656	-0,16204
Cloud_Mesh_Distance_BA_R0_R2	20,31345367	-81,24371338	-945,8341675	-0,155131
Cloud_Mesh_Distance_BA_R0_R2	20,58729362	-81,53074646	-945,8027954	-0,147654
Cloud_Mesh_Distance_BA_R0_R2	20,44325638	-81,47923279	-945,8378296	-0,157249
Cloud_Mesh_Distance_BA_R0_R2	17,03686142	-80,65612793	-945,8820801	0,022843
Cloud_Mesh_Distance_BA_R0_R2	17,01709938	-80,86967468	-945,8580933	0,115966
Cloud_Mesh_Distance_BA_R0_R2	17,21571732	-80,90223694	-945,8969116	0,114464
Cloud_Mesh_Distance_BA_R0_R2	17,21971512	-80,74342346	-945,9075317	0,051985
Cloud_Mesh_Distance_BA_R0_R2	17,99101448	-80,89833069	-945,968689	-0,004367
Cloud_Mesh_Distance_BA_R0_R2	17,88554382	-80,97206116	-945,9586792	0,021135
Cloud_Mesh_Distance_BA_R0_R2	18,36301994	-80,95213318	-945,9750977	-0,073096
Cloud_Mesh_Distance_BA_R0_R2	18,6375103	-80,86482239	-945,9855347	-0,102489
Cloud_Mesh_Distance_BA_R0_R2	18,65628242	-81,00590515	-945,9782105	-0,11829
Cloud_Mesh_Distance_BA_R0_R2	18,77186966	-81,08143616	-945,9817505	-0,136627
Cloud_Mesh_Distance_BA_R0_R2	18,94347954	-80,95608521	-945,9875488	-0,134989
Cloud_Mesh_Distance_BA_R0_R2	19,33175659	-81,04182434	-945,994812	-0,141811
Cloud_Mesh_Distance_BA_R0_R2	19,95098877	-81,0736084	-945,9240723	-0,135706
Cloud_Mesh_Distance_BA_R0_R2	19,97099495	-81,25444031	-945,9376221	-0,153314
Cloud_Mesh_Distance_BA_R0_R2	20,14217949	-81,24130249	-945,8901978	-0,15282
Cloud_Mesh_Distance_BA_R0_R2	20,14072418	-81,03768921	-945,8659668	-0,137595
Cloud_Mesh_Distance_BA_R0_R2	16,60866165	-80,52761841	-945,7734375	-0,038921
Cloud_Mesh_Distance_BA_R0_R2	16,6853199	-80,43023682	-945,8384399	-0,059856
Cloud_Mesh_Distance_BA_R0_R2	17,41955185	-80,65184021	-945,9560547	0,014436
Cloud_Mesh_Distance_BA_R0_R2	17,59547043	-80,63459778	-945,9806519	0,001954
Cloud_Mesh_Distance_BA_R0_R2	17,54420662	-80,78305054	-945,9487915	0,039672
Cloud_Mesh_Distance_BA_R0_R2	17,36188126	-80,80163574	-945,9242554	0,062489
Cloud_Mesh_Distance_BA_R0_R2	17,72507858	-80,83267212	-945,9595337	0,028138
Cloud_Mesh_Distance_BA_R0_R2	17,72685242	-80,68209839	-945,9829102	0,003332
Cloud_Mesh_Distance_BA_R0_R2	17,87401009	-80,86633301	-945,9658814	0,009484
Cloud_Mesh_Distance_BA_R0_R2	18,48945236	-80,87857056	-945,9817505	-0,092989
Cloud_Mesh_Distance_BA_R0_R2	18,76800728	-80,93211365	-945,9841309	-0,125271
Cloud_Mesh_Distance_BA_R0_R2	19,00078583	-80,79309082	-945,9956055	-0,11604
Cloud_Mesh_Distance_BA_R0_R2	19,10976028	-80,86352539	-945,9943237	-0,128262
Cloud_Mesh_Distance_BA_R0_R2	19,13631439	-81,00245667	-945,993042	-0,143647
Cloud_Mesh_Distance_BA_R0_R2	19,3931694	-80,90194702	-945,9911499	-0,12624
Cloud_Mesh_Distance_BA_R0_R2	19,42776489	-80,79417419	-945,9909058	-0,108233
Cloud_Mesh_Distance_BA_R0_R2	19,53732872	-80,89744568	-945,9836426	-0,116787
Cloud_Mesh_Distance_BA_R0_R2	19,74222183	-80,97433472	-945,9612427	-0,12475
Cloud_Mesh_Distance_BA_R0_R2	19,88554955	-80,85266113	-945,9261475	-0,109847
Cloud_Mesh_Distance_BA_R0_R2	16,18259048	-80,13858032	-945,7713623	-0,107166

Cloud_Mesh_Distance_BA_R0_R2	16,31339645	-80,1834259	-945,8097534	-0,103913
Cloud_Mesh_Distance_BA_R0_R2	16,37627602	-80,3578949	-945,7438355	-0,089992
Cloud_Mesh_Distance_BA_R0_R2	16,53886032	-80,42214966	-945,7843018	-0,071982
Cloud_Mesh_Distance_BA_R0_R2	16,76142693	-80,30880737	-945,9190674	-0,074498
Cloud_Mesh_Distance_BA_R0_R2	16,89878082	-80,47766113	-945,8917847	-0,03626
Cloud_Mesh_Distance_BA_R0_R2	17,05585098	-80,36726379	-945,9752808	-0,050833
Cloud_Mesh_Distance_BA_R0_R2	17,09559631	-80,50224304	-945,9349365	-0,021831
Cloud_Mesh_Distance_BA_R0_R2	17,22901344	-80,42292786	-945,9898682	-0,035461
Cloud_Mesh_Distance_BA_R0_R2	17,23784065	-80,57801819	-945,9431152	0,000132
Cloud_Mesh_Distance_BA_R0_R2	17,40076065	-80,48522949	-945,9980469	-0,016157
Cloud_Mesh_Distance_BA_R0_R2	17,54592514	-80,53663635	-946,0009155	-0,013769
Cloud_Mesh_Distance_BA_R0_R2	18,21163177	-80,49223328	-946,0469971	-0,044082
Cloud_Mesh_Distance_BA_R0_R2	18,3017025	-80,3813324	-946,0772705	-0,041093
Cloud_Mesh_Distance_BA_R0_R2	18,84085464	-80,75511169	-945,9954224	-0,108115
Cloud_Mesh_Distance_BA_R0_R2	19,34641647	-80,63343811	-945,9963989	-0,096144
Cloud_Mesh_Distance_BA_R0_R2	19,45589066	-80,55830383	-945,9884644	-0,084628
Cloud_Mesh_Distance_BA_R0_R2	19,54373932	-80,70323181	-945,9812012	-0,095522
Cloud_Mesh_Distance_BA_R0_R2	19,74466133	-80,62510681	-945,9473267	-0,089026
Cloud_Mesh_Distance_BA_R0_R2	19,73253059	-80,73414612	-945,9544067	-0,09819
Cloud_Mesh_Distance_BA_R0_R2	19,91016388	-80,63105774	-945,9053345	-0,09589
Cloud_Mesh_Distance_BA_R0_R2	20,06937218	-80,85063171	-945,8721314	-0,120749
Cloud_Mesh_Distance_BA_R0_R2	20,21395111	-80,76651001	-945,8130493	-0,122107
Cloud_Mesh_Distance_BA_R0_R2	16,00916672	-79,98196411	-945,7647705	-0,10567
Cloud_Mesh_Distance_BA_R0_R2	15,92554092	-79,87254333	-945,7709351	-0,098546
Cloud_Mesh_Distance_BA_R0_R2	16,06773376	-79,9316864	-945,8267212	-0,104284
Cloud_Mesh_Distance_BA_R0_R2	16,086586	-80,0539093	-945,7688599	-0,108854
Cloud_Mesh_Distance_BA_R0_R2	16,5349617	-80,25062561	-945,8673096	-0,09404
Cloud_Mesh_Distance_BA_R0_R2	16,68331718	-80,14950562	-945,9736939	-0,091418
Cloud_Mesh_Distance_BA_R0_R2	18,10504532	-80,4717865	-946,053772	-0,034528
Cloud_Mesh_Distance_BA_R0_R2	18,18563461	-80,41705322	-946,069519	-0,038364
Cloud_Mesh_Distance_BA_R0_R2	18,43585968	-80,33976746	-946,0810547	-0,047718
Cloud_Mesh_Distance_BA_R0_R2	18,47637749	-80,23164368	-946,1086426	-0,038164
Cloud_Mesh_Distance_BA_R0_R2	18,75745201	-80,59172058	-946,0131226	-0,093137
Cloud_Mesh_Distance_BA_R0_R2	18,82792473	-80,44667053	-946,0296021	-0,079959
Cloud_Mesh_Distance_BA_R0_R2	18,95964813	-80,53051758	-946,0147095	-0,089259
Cloud_Mesh_Distance_BA_R0_R2	19,05428505	-80,58314514	-946,0070801	-0,099323
Cloud_Mesh_Distance_BA_R0_R2	19,11254692	-80,3367157	-946,0186157	-0,080858
Cloud_Mesh_Distance_BA_R0_R2	19,30267525	-80,54823303	-945,9993897	-0,090018
Cloud_Mesh_Distance_BA_R0_R2	19,33952522	-80,38946533	-945,996582	-0,083182
Cloud_Mesh_Distance_BA_R0_R2	19,64731789	-80,39753723	-945,9539795	-0,078767
Cloud_Mesh_Distance_BA_R0_R2	19,6478157	-80,44406128	-945,9577026	-0,078267
Cloud_Mesh_Distance_BA_R0_R2	19,66992188	-80,55342102	-945,9592896	-0,08418
Cloud_Mesh_Distance_BA_R0_R2	19,77408028	-80,44789124	-945,9291382	-0,081156
Cloud_Mesh_Distance_BA_R0_R2	19,77869987	-80,57835388	-945,9373169	-0,08583
Cloud_Mesh_Distance_BA_R0_R2	19,93979263	-80,40742493	-945,8754883	-0,095979
Cloud_Mesh_Distance_BA_R0_R2	20,07894897	-80,50544739	-945,8370972	-0,103282
Cloud_Mesh_Distance_BA_R0_R2	16,36898232	-80,04548645	-945,9130859	-0,102063
Cloud_Mesh_Distance_BA_R0_R2	16,20124817	-80,0408783	-945,8369751	-0,105673
Cloud_Mesh_Distance_BA_R0_R2	16,35208702	-79,89837646	-945,9855347	-0,092993
Cloud_Mesh_Distance_BA_R0_R2	18,38069153	-80,28543091	-946,1021729	-0,037868

Cloud_Mesh_Distance_BA_R0_R2	19,11159325	-80,17193604	-946,029419	-0,07577
Cloud_Mesh_Distance_BA_R0_R2	19,29224014	-80,15162659	-946,0010376	-0,084096
Cloud_Mesh_Distance_BA_R0_R2	19,562397	-80,40943909	-945,9695435	-0,080945
Cloud_Mesh_Distance_BA_R0_R2	19,68867493	-80,40541077	-945,9462891	-0,078233
Cloud_Mesh_Distance_BA_R0_R2	19,6701889	-80,3170166	-945,9420166	-0,081333
Cloud_Mesh_Distance_BA_R0_R2	20,05609703	-80,34809875	-945,8268433	-0,104391
Cloud_Mesh_Distance_BA_R0_R2	15,87871933	-79,71218872	-945,8151245	-0,088446
Cloud_Mesh_Distance_BA_R0_R2	16,00799751	-79,7509613	-945,8826294	-0,088807
Cloud_Mesh_Distance_BA_R0_R2	16,29644966	-79,75486755	-946,0303955	-0,085019
Cloud_Mesh_Distance_BA_R0_R2	16,19812202	-79,87220764	-945,9280396	-0,093535
Cloud_Mesh_Distance_BA_R0_R2	19,48815727	-80,0189209	-945,9577026	-0,092947
Cloud_Mesh_Distance_BA_R0_R2	19,33539963	-79,93011475	-945,9929199	-0,087083
Cloud_Mesh_Distance_BA_R0_R2	19,50492477	-80,22712708	-945,9674683	-0,082229
Cloud_Mesh_Distance_BA_R0_R2	19,68130875	-80,10046387	-945,9161377	-0,094318
Cloud_Mesh_Distance_BA_R0_R2	19,83311272	-80,25189209	-945,8911743	-0,091227
Cloud_Mesh_Distance_BA_R0_R2	19,97361183	-80,2671814	-945,8461304	-0,099725
Cloud_Mesh_Distance_BA_R0_R2	15,85898209	-79,36250305	-945,9194946	-0,06152
Cloud_Mesh_Distance_BA_R0_R2	15,75813675	-79,50263977	-945,8034668	-0,075382
Cloud_Mesh_Distance_BA_R0_R2	16,00593948	-79,61482239	-945,9386597	-0,081057
Cloud_Mesh_Distance_BA_R0_R2	16,01291466	-79,46862793	-945,9917603	-0,070539
Cloud_Mesh_Distance_BA_R0_R2	15,89947128	-79,5786438	-945,8827515	-0,07647
Cloud_Mesh_Distance_BA_R0_R2	16,12603951	-79,65516663	-945,9910889	-0,079044
Cloud_Mesh_Distance_BA_R0_R2	19,6337738	-79,89453125	-945,9106445	-0,104684
Cloud_Mesh_Distance_BA_R0_R2	18,93680382	-79,71513367	-946,121582	-0,043289
Cloud_Mesh_Distance_BA_R0_R2	19,21381569	-79,80586243	-946,0289307	-0,080402
Cloud_Mesh_Distance_BA_R0_R2	19,20308304	-79,64067078	-946,0418091	-0,075937
Cloud_Mesh_Distance_BA_R0_R2	19,48615456	-79,83454895	-945,9511108	-0,099987
Cloud_Mesh_Distance_BA_R0_R2	19,37214279	-79,77554321	-945,9830322	-0,091873
Cloud_Mesh_Distance_BA_R0_R2	19,47716713	-79,68540955	-945,9514771	-0,102099
Cloud_Mesh_Distance_BA_R0_R2	19,6115284	-79,72314453	-945,9088745	-0,108999
Cloud_Mesh_Distance_BA_R0_R2	19,7411747	-79,77687073	-945,8679199	-0,115345
Cloud_Mesh_Distance_BA_R0_R2	15,83269024	-79,12135315	-945,9387817	-0,057079
Cloud_Mesh_Distance_BA_R0_R2	15,76748562	-79,02394104	-945,8922119	-0,055814
Cloud_Mesh_Distance_BA_R0_R2	15,82341194	-79,032547	-945,9353638	-0,061712
Cloud_Mesh_Distance_BA_R0_R2	15,95863914	-79,00366211	-946,0248413	-0,069706
Cloud_Mesh_Distance_BA_R0_R2	15,96961403	-79,2355957	-946,0159302	-0,065549
Cloud_Mesh_Distance_BA_R0_R2	19,27469826	-79,48252869	-946,0266113	-0,080419
Cloud_Mesh_Distance_BA_R0_R2	19,34620857	-79,63676453	-945,9953613	-0,089943
Cloud_Mesh_Distance_BA_R0_R2	19,51262093	-79,47337341	-945,9402466	-0,101414
Cloud_Mesh_Distance_BA_R0_R2	19,55697632	-79,36465454	-945,9259033	-0,10284
Cloud_Mesh_Distance_BA_R0_R2	16,07047462	-78,90301514	-946,0855713	-0,08749
Cloud_Mesh_Distance_BA_R0_R2	15,88458061	-78,84732056	-945,9700928	-0,072181
Cloud_Mesh_Distance_BA_R0_R2	16,12506676	-78,99311829	-946,1173706	-0,081614
Cloud_Mesh_Distance_BA_R0_R2	19,2732296	-79,28929138	-946,0351563	-0,074447
Cloud_Mesh_Distance_BA_R0_R2	19,38640594	-79,19262695	-945,994812	-0,084973
Cloud_Mesh_Distance_BA_R0_R2	19,42506027	-79,35844421	-945,9755249	-0,092372
Cloud_Mesh_Distance_BA_R0_R2	19,68753433	-79,22183228	-945,871582	-0,10658
Cloud_Mesh_Distance_BA_R0_R2	19,65382004	-79,32756042	-945,8869019	-0,108364
Cloud_Mesh_Distance_BA_R0_R2	19,73136902	-79,4098053	-945,8555298	-0,115027
Cloud_Mesh_Distance_BA_R0_R2	16,03115463	-78,7910614	-946,0541992	-0,094072

Cloud_Mesh_Distance_BA_R0_R2	16,05113792	-78,6292572	-946,0436401	-0,10556
Cloud_Mesh_Distance_BA_R0_R2	19,04685211	-79,04701233	-946,1287231	-0,045874
Cloud_Mesh_Distance_BA_R0_R2	19,15855217	-79,01242065	-946,0864868	-0,055763
Cloud_Mesh_Distance_BA_R0_R2	19,27105331	-79,11955261	-946,0411377	-0,070943
Cloud_Mesh_Distance_BA_R0_R2	19,54753304	-79,20184326	-945,9306641	-0,095897
Cloud_Mesh_Distance_BA_R0_R2	15,93301773	-78,68475342	-945,9812622	-0,087349
Cloud_Mesh_Distance_BA_R0_R2	15,99590302	-78,51557922	-945,9924927	-0,099391
Cloud_Mesh_Distance_BA_R0_R2	16,14058495	-78,50520325	-946,0722046	-0,11677
Cloud_Mesh_Distance_BA_R0_R2	16,21944237	-78,36080933	-946,0876465	-0,124843
Cloud_Mesh_Distance_BA_R0_R2	19,10472488	-78,94052124	-946,1082764	-0,048821
Cloud_Mesh_Distance_BA_R0_R2	18,99526215	-78,87576294	-946,1502075	-0,037274
Cloud_Mesh_Distance_BA_R0_R2	19,07607079	-78,80133057	-946,1185913	-0,045427
Cloud_Mesh_Distance_BA_R0_R2	19,22707939	-78,98278809	-946,0596314	-0,062701
Cloud_Mesh_Distance_BA_R0_R2	19,23278618	-78,77024841	-946,0537109	-0,059853
Cloud_Mesh_Distance_BA_R0_R2	19,40341759	-78,99452209	-945,9894409	-0,078048
Cloud_Mesh_Distance_BA_R0_R2	15,93939686	-78,22802734	-945,8994141	-0,074085
Cloud_Mesh_Distance_BA_R0_R2	16,15296555	-78,17068481	-946,0178833	-0,106644
Cloud_Mesh_Distance_BA_R0_R2	16,1305294	-78,30335999	-946,03125	-0,114664
Cloud_Mesh_Distance_BA_R0_R2	19,08207703	-78,69746399	-946,1124878	-0,042784
Cloud_Mesh_Distance_BA_R0_R2	19,01136208	-78,61244202	-946,1357422	-0,034725
Cloud_Mesh_Distance_BA_R0_R2	19,41828156	-78,79238892	-945,9769898	-0,07202
Cloud_Mesh_Distance_BA_R0_R2	19,38267899	-78,63563538	-945,9799194	-0,068375
Cloud_Mesh_Distance_BA_R0_R2	16,0445385	-78,13418579	-945,9470825	-0,087945
Cloud_Mesh_Distance_BA_R0_R2	16,28580856	-78,07470703	-946,064209	-0,116877
Cloud_Mesh_Distance_BA_R0_R2	16,37720108	-78,1285553	-946,1166382	-0,1317
Cloud_Mesh_Distance_BA_R0_R2	19,38484001	-78,47140503	-945,956665	-0,071334
Cloud_Mesh_Distance_BA_R0_R2	19,12485123	-78,58148193	-946,0863648	-0,047706
Cloud_Mesh_Distance_BA_R0_R2	19,25809669	-78,53639221	-946,024231	-0,059622
Cloud_Mesh_Distance_BA_R0_R2	19,51511383	-78,69749451	-945,9246216	-0,080591
Cloud_Mesh_Distance_BA_R0_R2	16,04292488	-78,00320435	-945,9128418	-0,076532
Cloud_Mesh_Distance_BA_R0_R2	15,984972	-77,89492798	-945,8365479	-0,056604
Cloud_Mesh_Distance_BA_R0_R2	16,17078972	-77,85319519	-945,9428101	-0,081954
Cloud_Mesh_Distance_BA_R0_R2	16,1690712	-78,0249176	-945,9927979	-0,099243
Cloud_Mesh_Distance_BA_R0_R2	16,47563934	-77,84204102	-946,0809326	-0,113976
Cloud_Mesh_Distance_BA_R0_R2	16,31697655	-77,92085266	-946,0393066	-0,104934
Cloud_Mesh_Distance_BA_R0_R2	16,43030167	-77,99525452	-946,1079102	-0,124652
Cloud_Mesh_Distance_BA_R0_R2	19,03808403	-78,34085083	-946,0913086	-0,040832
Cloud_Mesh_Distance_BA_R0_R2	19,14424324	-78,41957092	-946,0588379	-0,049624
Cloud_Mesh_Distance_BA_R0_R2	19,27290154	-78,36386108	-945,9899292	-0,065239
Cloud_Mesh_Distance_BA_R0_R2	19,15324402	-78,24003601	-946,0199585	-0,058113
Cloud_Mesh_Distance_BA_R0_R2	19,29448128	-78,2210083	-945,9468384	-0,07251
Cloud_Mesh_Distance_BA_R0_R2	16,24910355	-77,66514587	-945,9082642	-0,065473
Cloud_Mesh_Distance_BA_R0_R2	16,36543465	-77,75213623	-946,00354	-0,099045
Cloud_Mesh_Distance_BA_R0_R2	17,06170845	-77,80484009	-946,2005615	-0,141765
Cloud_Mesh_Distance_BA_R0_R2	16,91147232	-77,77159119	-946,1671753	-0,137355
Cloud_Mesh_Distance_BA_R0_R2	17,24227333	-77,84066772	-946,2285156	-0,138352
Cloud_Mesh_Distance_BA_R0_R2	17,41882896	-77,81988525	-946,2272339	-0,129603
Cloud_Mesh_Distance_BA_R0_R2	18,44678497	-77,95256042	-946,1870728	-0,0304
Cloud_Mesh_Distance_BA_R0_R2	18,38755608	-77,77540588	-946,138855	-0,036525
Cloud_Mesh_Distance_BA_R0_R2	18,5773468	-77,8447876	-946,119751	-0,031696

Cloud_Mesh_Distance_BA_R0_R2	18,62001228	-78,0067749	-946,161499	-0,02433
Cloud_Mesh_Distance_BA_R0_R2	18,88670921	-77,89910889	-946,0336914	-0,045675
Cloud_Mesh_Distance_BA_R0_R2	18,78361511	-78,04016113	-946,119812	-0,031129
Cloud_Mesh_Distance_BA_R0_R2	16,29655075	-77,5425415	-945,866333	-0,067091
Cloud_Mesh_Distance_BA_R0_R2	16,44242477	-77,56384277	-945,9481812	-0,088832
Cloud_Mesh_Distance_BA_R0_R2	16,56986046	-77,43920898	-945,9213867	-0,093169
Cloud_Mesh_Distance_BA_R0_R2	16,89822388	-77,51731873	-946,0476074	-0,130318
Cloud_Mesh_Distance_BA_R0_R2	16,68344879	-77,54541016	-946,0170898	-0,118519
Cloud_Mesh_Distance_BA_R0_R2	16,81763077	-77,4251709	-945,979126	-0,123796
Cloud_Mesh_Distance_BA_R0_R2	16,82380104	-77,66151428	-946,1062622	-0,123103
Cloud_Mesh_Distance_BA_R0_R2	17,08049393	-77,46870422	-946,0470581	-0,145539
Cloud_Mesh_Distance_BA_R0_R2	17,18212891	-77,70751953	-946,1719971	-0,144767
Cloud_Mesh_Distance_BA_R0_R2	17,0300293	-77,63768005	-946,126648	-0,141199
Cloud_Mesh_Distance_BA_R0_R2	17,26234245	-77,55784607	-946,109436	-0,144006
Cloud_Mesh_Distance_BA_R0_R2	17,32072067	-77,7101593	-946,1814575	-0,13987
Cloud_Mesh_Distance_BA_R0_R2	18,06913567	-77,74981689	-946,1760254	-0,066329
Cloud_Mesh_Distance_BA_R0_R2	18,36449814	-77,68118286	-946,1068115	-0,041734
Cloud_Mesh_Distance_BA_R0_R2	18,72593498	-77,90859985	-946,09729	-0,032643
Cloud_Mesh_Distance_BA_R0_R2	18,91115379	-77,74790955	-945,9637451	-0,058468
Cloud_Mesh_Distance_BA_R0_R2	19,05467415	-77,96580505	-945,9838867	-0,061994
Cloud_Mesh_Distance_BA_R0_R2	19,02220535	-77,8177948	-945,9425049	-0,066955
Cloud_Mesh_Distance_BA_R0_R2	19,14270401	-77,87361145	-945,9065552	-0,077083
Cloud_Mesh_Distance_BA_R0_R2	16,26432419	-77,46917725	-945,7977295	-0,060289
Cloud_Mesh_Distance_BA_R0_R2	16,38176155	-77,45916748	-945,855896	-0,075217
Cloud_Mesh_Distance_BA_R0_R2	16,46941376	-77,45510864	-945,8929443	-0,081796
Cloud_Mesh_Distance_BA_R0_R2	16,96245956	-77,38687134	-945,9816284	-0,134885
Cloud_Mesh_Distance_BA_R0_R2	17,09830856	-77,358078	-945,9840088	-0,140688
Cloud_Mesh_Distance_BA_R0_R2	17,23969078	-77,37167358	-946,0080566	-0,14184
Cloud_Mesh_Distance_BA_R0_R2	17,44492531	-77,40664673	-946,0418091	-0,138055
Cloud_Mesh_Distance_BA_R0_R2	17,57095909	-77,52416992	-946,1040649	-0,127484
Cloud_Mesh_Distance_BA_R0_R2	17,69604301	-77,57836914	-946,1281128	-0,113335
Cloud_Mesh_Distance_BA_R0_R2	17,83022118	-77,5740509	-946,1227417	-0,095883
Cloud_Mesh_Distance_BA_R0_R2	18,01987839	-77,58520508	-946,1152344	-0,073463
Cloud_Mesh_Distance_BA_R0_R2	17,89469337	-77,443573	-946,0598145	-0,089759
Cloud_Mesh_Distance_BA_R0_R2	18,22719955	-77,66714478	-946,1256714	-0,051012
Cloud_Mesh_Distance_BA_R0_R2	18,51806068	-77,6706543	-946,0683594	-0,037297
Cloud_Mesh_Distance_BA_R0_R2	18,42337227	-77,70950317	-946,1052856	-0,037794
Cloud_Mesh_Distance_BA_R0_R2	18,35809326	-77,54222107	-946,0498657	-0,045888
Cloud_Mesh_Distance_BA_R0_R2	18,74537659	-77,74447632	-946,02771	-0,045163
Cloud_Mesh_Distance_BA_R0_R2	18,6829834	-77,56143188	-945,9696655	-0,045817
Cloud_Mesh_Distance_BA_R0_R2	17,58130074	-77,32868958	-946,0044556	-0,124185
Cloud_Mesh_Distance_BA_R0_R2	17,60329437	-77,40742493	-946,0470581	-0,123401
Cloud_Mesh_Distance_BA_R0_R2	18,16726685	-77,52786255	-946,0753174	-0,057442
Cloud_Mesh_Distance_BA_R0_R2	18,05245018	-77,45713806	-946,0546875	-0,073001
Cloud_Mesh_Distance_BA_R0_R2	18,18280411	-77,3946991	-946,0080566	-0,057259
Cloud_Mesh_Distance_BA_R0_R2	18,53378677	-77,51597595	-945,9946289	-0,041002
Cloud_Mesh_Distance_BA_R0_R2	18,62574387	-77,41726685	-945,9154663	-0,041345
Cloud_Mesh_Distance_BA_R0_R2	17,37260056	-77,27510071	-945,9613648	-0,138297
Cloud_Mesh_Distance_BA_R0_R2	17,51612473	-77,26216125	-945,9631348	-0,130147
Cloud_Mesh_Distance_BA_R0_R2	17,60771942	-77,16040039	-945,9030762	-0,114369

Cloud_Mesh_Distance_BA_R0_R2	18,00729179	-77,34707642	-946,0032959	-0,07248
Cloud_Mesh_Distance_BA_R0_R2	18,11147308	-77,25202942	-945,9379273	-0,063174
Cloud_Mesh_Distance_BA_R0_R2	18,33146286	-77,37449646	-945,9719238	-0,045881
Cloud_Mesh_Distance_BA_R0_R2	18,25381851	-77,28581238	-945,9363403	-0,05087
Cloud_Mesh_Distance_BA_R0_R2	16,17736626	-81,36839294	-945,6917725	0,502323
Cloud_Mesh_Distance_BA_R0_R2	21,46760368	-82,09301758	-945,7227783	0,207957
Cloud_Mesh_Distance_BA_R0_R2	21,56219864	-82,1267395	-945,6908569	0,246087
Cloud_Mesh_Distance_BA_R0_R2	21,53352737	-82,02404785	-945,6520996	0,133083
Cloud_Mesh_Distance_BA_R0_R2	15,99175453	-81,24653625	-945,5131836	0,385099
Cloud_Mesh_Distance_BA_R0_R2	16,1235714	-81,0960083	-945,5482788	0,27006
Cloud_Mesh_Distance_BA_R0_R2	16,33217621	-81,21022034	-945,7026367	0,370162
Cloud_Mesh_Distance_BA_R0_R2	20,89087105	-81,65632629	-945,7387695	-0,108545
Cloud_Mesh_Distance_BA_R0_R2	20,94491959	-81,81239319	-945,7836914	-0,087196
Cloud_Mesh_Distance_BA_R0_R2	21,22327995	-81,88859558	-945,7213745	-0,143318
Cloud_Mesh_Distance_BA_R0_R2	21,02234459	-81,73353577	-945,7241211	-0,082153
Cloud_Mesh_Distance_BA_R0_R2	21,15646362	-81,74446106	-945,6782227	-0,058206
Cloud_Mesh_Distance_BA_R0_R2	21,09405899	-81,85922241	-945,7546997	-0,074656
Cloud_Mesh_Distance_BA_R0_R2	21,30982018	-81,82142639	-945,6523438	-0,033045
Cloud_Mesh_Distance_BA_R0_R2	21,45919037	-82,00254822	-945,6795654	0,108276
Cloud_Mesh_Distance_BA_R0_R2	16,2855835	-80,89506531	-945,5933228	0,124173
Cloud_Mesh_Distance_BA_R0_R2	16,20300293	-81,21495056	-945,6379395	0,372823
Cloud_Mesh_Distance_BA_R0_R2	20,73423195	-81,58106995	-945,7667847	-0,134241
Cloud_Mesh_Distance_BA_R0_R2	20,8419857	-81,47346497	-945,6906128	-0,131354
Cloud_Mesh_Distance_BA_R0_R2	21,25361061	-81,69021606	-945,6098633	-0,054165
Cloud_Mesh_Distance_BA_R0_R2	16,2298317	-80,82270813	-945,55896	0,076352
Cloud_Mesh_Distance_BA_R0_R2	16,34918594	-80,78491211	-945,6177368	0,060171
Cloud_Mesh_Distance_BA_R0_R2	16,54595184	-80,978302	-945,723999	0,191046
Cloud_Mesh_Distance_BA_R0_R2	16,6982975	-80,95692444	-945,7750244	0,174367
Cloud_Mesh_Distance_BA_R0_R2	16,42353821	-80,91099548	-945,6612549	0,142566
Cloud_Mesh_Distance_BA_R0_R2	20,42847252	-81,32296753	-945,8074951	-0,155703
Cloud_Mesh_Distance_BA_R0_R2	20,56651115	-81,26008606	-945,7407837	-0,154161
Cloud_Mesh_Distance_BA_R0_R2	20,53635406	-81,40281677	-945,7860107	-0,154475
Cloud_Mesh_Distance_BA_R0_R2	20,66521835	-81,42427063	-945,7429199	-0,147426
Cloud_Mesh_Distance_BA_R0_R2	20,73717308	-81,29827881	-945,6807861	-0,14647
Cloud_Mesh_Distance_BA_R0_R2	20,98540878	-81,42648315	-945,6154175	-0,120352
Cloud_Mesh_Distance_BA_R0_R2	21,02291107	-81,59577942	-945,6651001	-0,097636
Cloud_Mesh_Distance_BA_R0_R2	21,1584301	-81,6190033	-945,6188965	-0,07821
Cloud_Mesh_Distance_BA_R0_R2	16,48516655	-80,77293396	-945,6785889	0,057386
Cloud_Mesh_Distance_BA_R0_R2	16,6120224	-80,83418274	-945,7324829	0,100754
Cloud_Mesh_Distance_BA_R0_R2	16,58929443	-80,66374207	-945,732666	0,00887
Cloud_Mesh_Distance_BA_R0_R2	16,76090622	-80,83787537	-945,7858276	0,102379
Cloud_Mesh_Distance_BA_R0_R2	16,74725723	-80,72241211	-945,7853394	0,043913
Cloud_Mesh_Distance_BA_R0_R2	16,88334274	-80,7726593	-945,8253784	0,071983
Cloud_Mesh_Distance_BA_R0_R2	20,2787056	-81,10221863	-945,8255615	-0,147321
Cloud_Mesh_Distance_BA_R0_R2	20,52120209	-81,09327698	-945,7280884	-0,152728
Cloud_Mesh_Distance_BA_R0_R2	20,41802979	-81,17008972	-945,7821045	-0,152958
Cloud_Mesh_Distance_BA_R0_R2	20,66832733	-81,11051941	-945,6690064	-0,151246
Cloud_Mesh_Distance_BA_R0_R2	16,4281292	-80,67155457	-945,6621704	0,004366
Cloud_Mesh_Distance_BA_R0_R2	16,46312332	-80,5309906	-945,7109375	-0,0491
Cloud_Mesh_Distance_BA_R0_R2	16,86663055	-80,64297485	-945,8363037	0,015609

Cloud_Mesh_Distance_BA_R0_R2	16,73847008	-80,579422	-945,8075562	-0,015022
Cloud_Mesh_Distance_BA_R0_R2	20,40028191	-81,00114441	-945,7642212	-0,144796
Cloud_Mesh_Distance_BA_R0_R2	20,24890137	-80,92481995	-945,8150635	-0,136309
Cloud_Mesh_Distance_BA_R0_R2	20,5529213	-80,92573547	-945,6896973	-0,147687
Cloud_Mesh_Distance_BA_R0_R2	20,7837944	-81,04908752	-945,6064453	-0,148279
Cloud_Mesh_Distance_BA_R0_R2	15,8424511	-80,06007385	-945,6112671	-0,111186
Cloud_Mesh_Distance_BA_R0_R2	16,10119057	-80,15376282	-945,7198486	-0,111461
Cloud_Mesh_Distance_BA_R0_R2	16,21015358	-80,24726868	-945,723938	-0,104536
Cloud_Mesh_Distance_BA_R0_R2	20,10849762	-80,66972351	-945,8429565	-0,110673
Cloud_Mesh_Distance_BA_R0_R2	20,37496567	-80,79234314	-945,7494507	-0,134117
Cloud_Mesh_Distance_BA_R0_R2	20,41048431	-80,61581421	-945,7141113	-0,129303
Cloud_Mesh_Distance_BA_R0_R2	20,53067017	-80,71829224	-945,6728516	-0,138153
Cloud_Mesh_Distance_BA_R0_R2	15,75110054	-79,99205017	-945,5802002	-0,104962
Cloud_Mesh_Distance_BA_R0_R2	15,69664001	-79,85275269	-945,6079102	-0,096115
Cloud_Mesh_Distance_BA_R0_R2	15,96686172	-80,06190491	-945,6929321	-0,109656
Cloud_Mesh_Distance_BA_R0_R2	20,25194168	-80,595047	-945,7785645	-0,120566
Cloud_Mesh_Distance_BA_R0_R2	20,3670845	-80,43264771	-945,7089844	-0,12511
Cloud_Mesh_Distance_BA_R0_R2	20,67063904	-80,80780029	-945,6206665	-0,143509
Cloud_Mesh_Distance_BA_R0_R2	15,76763344	-79,72418213	-945,7268677	-0,09046
Cloud_Mesh_Distance_BA_R0_R2	15,65052414	-79,71359253	-945,6304321	-0,088263
Cloud_Mesh_Distance_BA_R0_R2	15,81955719	-79,92837524	-945,6673584	-0,104997
Cloud_Mesh_Distance_BA_R0_R2	20,09092331	-80,18162537	-945,7893066	-0,114236
Cloud_Mesh_Distance_BA_R0_R2	20,27364922	-80,24142456	-945,7225952	-0,120865
Cloud_Mesh_Distance_BA_R0_R2	20,18870544	-80,38345337	-945,7783203	-0,114225
Cloud_Mesh_Distance_BA_R0_R2	20,51601219	-80,54135132	-945,6571655	-0,130986
Cloud_Mesh_Distance_BA_R0_R2	15,82124233	-79,62937927	-945,8071899	-0,08281
Cloud_Mesh_Distance_BA_R0_R2	19,94299316	-80,15496826	-945,8417358	-0,102253
Cloud_Mesh_Distance_BA_R0_R2	20,09670258	-80,02342224	-945,7628174	-0,121015
Cloud_Mesh_Distance_BA_R0_R2	20,36472702	-80,13121033	-945,6646729	-0,130265
Cloud_Mesh_Distance_BA_R0_R2	20,40470695	-80,27745056	-945,670044	-0,127614
Cloud_Mesh_Distance_BA_R0_R2	20,21690941	-80,04690552	-945,7158203	-0,12653
Cloud_Mesh_Distance_BA_R0_R2	15,63215828	-79,37870789	-945,7243652	-0,066542
Cloud_Mesh_Distance_BA_R0_R2	15,64913559	-79,57502747	-945,6824951	-0,080562
Cloud_Mesh_Distance_BA_R0_R2	19,84487152	-80,08537292	-945,8657837	-0,103006
Cloud_Mesh_Distance_BA_R0_R2	19,79706764	-79,93395996	-945,8638306	-0,109261
Cloud_Mesh_Distance_BA_R0_R2	19,97029114	-80,00007629	-945,8098755	-0,114829
Cloud_Mesh_Distance_BA_R0_R2	20,2503891	-79,89888	-945,6776733	-0,135874
Cloud_Mesh_Distance_BA_R0_R2	20,33127975	-79,98994446	-945,656189	-0,134383
Cloud_Mesh_Distance_BA_R0_R2	15,63110924	-79,14486694	-945,7611084	-0,05469
Cloud_Mesh_Distance_BA_R0_R2	15,73347855	-79,26951599	-945,8404541	-0,058014
Cloud_Mesh_Distance_BA_R0_R2	15,73056698	-79,12068176	-945,8568726	-0,054683
Cloud_Mesh_Distance_BA_R0_R2	19,91107178	-79,7646637	-945,8042603	-0,123007
Cloud_Mesh_Distance_BA_R0_R2	20,07673836	-79,64547729	-945,7209473	-0,1348
Cloud_Mesh_Distance_BA_R0_R2	20,10743141	-79,87278748	-945,7371216	-0,129052
Cloud_Mesh_Distance_BA_R0_R2	15,52371216	-79,11849976	-945,6461182	-0,046519
Cloud_Mesh_Distance_BA_R0_R2	15,70223999	-79,06370544	-945,8348389	-0,053015
Cloud_Mesh_Distance_BA_R0_R2	19,40670586	-79,50447083	-945,9785156	-0,094542
Cloud_Mesh_Distance_BA_R0_R2	19,46543503	-79,57499695	-945,9558106	-0,100418
Cloud_Mesh_Distance_BA_R0_R2	19,57657242	-79,58135986	-945,9173584	-0,106558
Cloud_Mesh_Distance_BA_R0_R2	19,63265991	-79,45672607	-945,8964233	-0,109771

Cloud_Mesh_Distance_BA_R0_R2	19,75067902	-79,58026123	-945,8533936	-0,119357
Cloud_Mesh_Distance_BA_R0_R2	19,93003273	-79,5566864	-945,7781372	-0,130247
Cloud_Mesh_Distance_BA_R0_R2	20,04681015	-79,41885376	-945,7108154	-0,135685
Cloud_Mesh_Distance_BA_R0_R2	15,47540283	-78,66848755	-945,5615845	-0,029351
Cloud_Mesh_Distance_BA_R0_R2	15,57433701	-78,75059509	-945,6901245	-0,043193
Cloud_Mesh_Distance_BA_R0_R2	15,6839838	-78,98088074	-945,819397	-0,053019
Cloud_Mesh_Distance_BA_R0_R2	15,68013954	-78,81755066	-945,8051148	-0,052617
Cloud_Mesh_Distance_BA_R0_R2	15,77274227	-78,8952179	-945,8919067	-0,059792
Cloud_Mesh_Distance_BA_R0_R2	15,57104683	-78,95773315	-945,7032471	-0,047904
Cloud_Mesh_Distance_BA_R0_R2	19,7497673	-79,3000946	-945,84552	-0,112487
Cloud_Mesh_Distance_BA_R0_R2	19,85738373	-79,3782196	-945,7991333	-0,124135
Cloud_Mesh_Distance_BA_R0_R2	20,16363525	-79,34805298	-945,6385498	-0,14204
Cloud_Mesh_Distance_BA_R0_R2	15,80157948	-78,73672485	-945,8971558	-0,069531
Cloud_Mesh_Distance_BA_R0_R2	19,66631126	-79,11502075	-945,8788452	-0,100894
Cloud_Mesh_Distance_BA_R0_R2	19,57616997	-79,02960205	-945,9172974	-0,092403
Cloud_Mesh_Distance_BA_R0_R2	19,83497238	-78,96653748	-945,7843018	-0,108944
Cloud_Mesh_Distance_BA_R0_R2	19,80391312	-79,1525116	-945,8132324	-0,113595
Cloud_Mesh_Distance_BA_R0_R2	19,94073105	-79,03971863	-945,7300415	-0,118418
Cloud_Mesh_Distance_BA_R0_R2	19,97097969	-79,22398376	-945,7312622	-0,125899
Cloud_Mesh_Distance_BA_R0_R2	15,73257065	-78,39590454	-945,777771	-0,054997
Cloud_Mesh_Distance_BA_R0_R2	15,7091198	-78,59326172	-945,7973023	-0,057011
Cloud_Mesh_Distance_BA_R0_R2	15,62065506	-78,46214294	-945,6870728	-0,042591
Cloud_Mesh_Distance_BA_R0_R2	15,85991001	-78,58647156	-945,9158936	-0,078133
Cloud_Mesh_Distance_BA_R0_R2	16,10124588	-78,41459656	-946,0352783	-0,112391
Cloud_Mesh_Distance_BA_R0_R2	19,70011139	-78,95823669	-945,8549805	-0,095876
Cloud_Mesh_Distance_BA_R0_R2	19,79621506	-78,80680847	-945,7893677	-0,098821
Cloud_Mesh_Distance_BA_R0_R2	20,02449989	-78,92976379	-945,6622315	-0,121964
Cloud_Mesh_Distance_BA_R0_R2	15,66441536	-78,29483032	-945,6884155	-0,038299
Cloud_Mesh_Distance_BA_R0_R2	15,81314373	-78,22572327	-945,8048096	-0,053043
Cloud_Mesh_Distance_BA_R0_R2	16,02593422	-78,38208008	-945,986023	-0,103725
Cloud_Mesh_Distance_BA_R0_R2	15,88298512	-78,40020752	-945,8970947	-0,077849
Cloud_Mesh_Distance_BA_R0_R2	16,02803612	-78,27067566	-945,9663086	-0,098202
Cloud_Mesh_Distance_BA_R0_R2	19,6547966	-78,75274658	-945,8605347	-0,089937
Cloud_Mesh_Distance_BA_R0_R2	19,5983696	-78,57711792	-945,8671875	-0,083198
Cloud_Mesh_Distance_BA_R0_R2	19,54740906	-78,87117004	-945,9232788	-0,084379
Cloud_Mesh_Distance_BA_R0_R2	19,75536728	-78,60165405	-945,7837524	-0,094178
Cloud_Mesh_Distance_BA_R0_R2	19,92181587	-78,86376953	-945,7192383	-0,110237
Cloud_Mesh_Distance_BA_R0_R2	15,85494137	-78,02928162	-945,7811279	-0,048629
Cloud_Mesh_Distance_BA_R0_R2	15,93967819	-78,10543823	-945,8699951	-0,065758
Cloud_Mesh_Distance_BA_R0_R2	15,95491791	-78,00892639	-945,8535156	-0,062761
Cloud_Mesh_Distance_BA_R0_R2	19,51255608	-78,43347168	-945,8873291	-0,077618
Cloud_Mesh_Distance_BA_R0_R2	19,47932625	-78,56115723	-945,9249268	-0,07852
Cloud_Mesh_Distance_BA_R0_R2	19,66242409	-78,42102051	-945,8040161	-0,087275
Cloud_Mesh_Distance_BA_R0_R2	19,8948288	-78,68704224	-945,7106934	-0,101592
Cloud_Mesh_Distance_BA_R0_R2	15,85049248	-77,8658905	-945,7127686	-0,029385
Cloud_Mesh_Distance_BA_R0_R2	15,95985889	-77,7784729	-945,7671509	-0,039307
Cloud_Mesh_Distance_BA_R0_R2	19,40115738	-78,31654358	-945,9179077	-0,078866
Cloud_Mesh_Distance_BA_R0_R2	15,89243889	-77,73786926	-945,6851196	-0,023766
Cloud_Mesh_Distance_BA_R0_R2	16,08270073	-77,7348938	-945,8391113	-0,051851
Cloud_Mesh_Distance_BA_R0_R2	15,99948502	-77,66508484	-945,7379761	-0,034297

Cloud_Mesh_Distance_BA_R0_R2	19,39765739	-78,16177368	-945,8773804	-0,085366
Cloud_Mesh_Distance_BA_R0_R2	19,24667931	-78,03762817	-945,9147339	-0,079978
Cloud_Mesh_Distance_BA_R0_R2	19,54347992	-78,2651062	-945,8308106	-0,08725
Cloud_Mesh_Distance_BA_R0_R2	16,14961624	-77,57173157	-945,7940674	-0,049273
Cloud_Mesh_Distance_BA_R0_R2	16,7089386	-77,40174866	-945,9379883	-0,115969
Cloud_Mesh_Distance_BA_R0_R2	19,39499283	-78,05081177	-945,8425903	-0,094743
Cloud_Mesh_Distance_BA_R0_R2	19,64131737	-78,15977478	-945,7457275	-0,096393
Cloud_Mesh_Distance_BA_R0_R2	19,50410843	-78,09240723	-945,7991943	-0,094092
Cloud_Mesh_Distance_BA_R0_R2	19,61024475	-78,00730896	-945,7094727	-0,100132
Cloud_Mesh_Distance_BA_R0_R2	16,48634338	-77,36676025	-945,8364258	-0,080092
Cloud_Mesh_Distance_BA_R0_R2	16,63934898	-77,32507324	-945,8618774	-0,105506
Cloud_Mesh_Distance_BA_R0_R2	17,04422379	-77,28001404	-945,9248047	-0,138797
Cloud_Mesh_Distance_BA_R0_R2	17,16563416	-77,29109192	-945,9501343	-0,143207
Cloud_Mesh_Distance_BA_R0_R2	17,7246151	-77,46057129	-946,0741577	-0,110389
Cloud_Mesh_Distance_BA_R0_R2	18,88554192	-77,58273315	-945,8978882	-0,062136
Cloud_Mesh_Distance_BA_R0_R2	19,12358856	-77,72665405	-945,8529663	-0,083478
Cloud_Mesh_Distance_BA_R0_R2	18,99912643	-77,69702148	-945,9002075	-0,069148
Cloud_Mesh_Distance_BA_R0_R2	16,6751976	-77,19039917	-945,7583618	-0,119562
Cloud_Mesh_Distance_BA_R0_R2	16,86903763	-77,16003418	-945,7941284	-0,132578
Cloud_Mesh_Distance_BA_R0_R2	16,84036064	-77,28884888	-945,8907471	-0,130738
Cloud_Mesh_Distance_BA_R0_R2	17,00926399	-77,17689514	-945,8421021	-0,138396
Cloud_Mesh_Distance_BA_R0_R2	17,25817108	-77,26278687	-945,9430542	-0,144965
Cloud_Mesh_Distance_BA_R0_R2	17,7108326	-77,33006287	-946,0070801	-0,1073
Cloud_Mesh_Distance_BA_R0_R2	17,64089203	-77,25013733	-945,960144	-0,11639
Cloud_Mesh_Distance_BA_R0_R2	17,79333878	-77,38513184	-946,0345459	-0,098599
Cloud_Mesh_Distance_BA_R0_R2	17,85692787	-77,31788635	-945,9967651	-0,089889
Cloud_Mesh_Distance_BA_R0_R2	18,4689846	-77,39555359	-945,9507446	-0,039487
Cloud_Mesh_Distance_BA_R0_R2	18,72153091	-77,32606506	-945,8259888	-0,050566
Cloud_Mesh_Distance_BA_R0_R2	18,76502991	-77,44944763	-945,8809204	-0,050998
Cloud_Mesh_Distance_BA_R0_R2	19,03869247	-77,61360168	-945,8413086	-0,076992
Cloud_Mesh_Distance_BA_R0_R2	17,33859062	-77,07748413	-945,8204956	-0,13597
Cloud_Mesh_Distance_BA_R0_R2	17,18383408	-77,19064331	-945,8828735	-0,142751
Cloud_Mesh_Distance_BA_R0_R2	17,32315445	-77,19300842	-945,9031982	-0,140351
Cloud_Mesh_Distance_BA_R0_R2	17,45090675	-77,16877747	-945,8995361	-0,128663
Cloud_Mesh_Distance_BA_R0_R2	17,70291138	-77,05592346	-945,8320313	-0,101387
Cloud_Mesh_Distance_BA_R0_R2	17,62496758	-76,96917725	-945,7623901	-0,107914
Cloud_Mesh_Distance_BA_R0_R2	17,53552437	-77,06083679	-945,8286743	-0,123888
Cloud_Mesh_Distance_BA_R0_R2	17,79343796	-77,18490601	-945,9193115	-0,100173
Cloud_Mesh_Distance_BA_R0_R2	17,95624352	-77,23957825	-945,9459839	-0,077995
Cloud_Mesh_Distance_BA_R0_R2	18,01789665	-77,11701965	-945,8598023	-0,067583
Cloud_Mesh_Distance_BA_R0_R2	18,38812828	-77,26985168	-945,8983765	-0,045945
Cloud_Mesh_Distance_BA_R0_R2	18,25284195	-77,15621948	-945,8521118	-0,049685
Cloud_Mesh_Distance_BA_R0_R2	18,56149101	-77,27325439	-945,8513184	-0,042784
Cloud_Mesh_Distance_BA_R0_R2	17,78673935	-76,9352417	-945,7356567	-0,094319
Cloud_Mesh_Distance_BA_R0_R2	17,85717773	-77,04815674	-945,8225708	-0,084126
Cloud_Mesh_Distance_BA_R0_R2	17,97967529	-76,96980286	-945,7514648	-0,072053
Cloud_Mesh_Distance_BA_R0_R2	18,17174721	-77,00852966	-945,755188	-0,055473
Cloud_Mesh_Distance_BA_R0_R2	18,36874008	-77,04148865	-945,7391357	-0,045061
Cloud_Mesh_Distance_BA_R0_R2	18,44984245	-77,15917969	-945,8078613	-0,040344
Cloud_Mesh_Distance_BA_R0_R2	22,08427048	-82,30860901	-945,3666992	0,269176

Cloud_Mesh_Distance_BA_R0_R2	21,6367836	-82,07173157	-945,6165161	0,177355
Cloud_Mesh_Distance_BA_R0_R2	21,72237206	-81,95695496	-945,4848633	0,02102
Cloud_Mesh_Distance_BA_R0_R2	21,75001144	-82,13604736	-945,5714111	0,174244
Cloud_Mesh_Distance_BA_R0_R2	21,96491432	-82,26951599	-945,4620972	0,231843
Cloud_Mesh_Distance_BA_R0_R2	15,87010098	-81,1688385	-945,3776856	0,306163
Cloud_Mesh_Distance_BA_R0_R2	16,01007652	-81,05799866	-945,4614868	0,230805
Cloud_Mesh_Distance_BA_R0_R2	21,46458435	-81,89634705	-945,6174927	0,003346
Cloud_Mesh_Distance_BA_R0_R2	21,58650208	-81,77262878	-945,4645996	-0,017956
Cloud_Mesh_Distance_BA_R0_R2	21,59171677	-81,94543457	-945,5716553	0,045743
Cloud_Mesh_Distance_BA_R0_R2	21,38642502	-81,71405029	-945,5546265	-0,03676
Cloud_Mesh_Distance_BA_R0_R2	15,90883636	-80,98959351	-945,3685913	0,172648
Cloud_Mesh_Distance_BA_R0_R2	16,26518631	-81,02409363	-945,607666	0,217387
Cloud_Mesh_Distance_BA_R0_R2	21,74049568	-81,81903076	-945,3776245	-0,009001
Cloud_Mesh_Distance_BA_R0_R2	21,83779716	-81,89349365	-945,3453369	-0,00153
Cloud_Mesh_Distance_BA_R0_R2	16,02317047	-80,91357422	-945,4454956	0,128489
Cloud_Mesh_Distance_BA_R0_R2	15,95054817	-80,6630249	-945,4115601	-0,009528
Cloud_Mesh_Distance_BA_R0_R2	16,10584641	-80,76060486	-945,493042	0,038401
Cloud_Mesh_Distance_BA_R0_R2	16,15020752	-80,93771362	-945,5264282	0,151888
Cloud_Mesh_Distance_BA_R0_R2	20,86922646	-81,3303833	-945,6343994	-0,136665
Cloud_Mesh_Distance_BA_R0_R2	21,11142921	-81,37841797	-945,5393677	-0,115931
Cloud_Mesh_Distance_BA_R0_R2	21,10565567	-81,51409912	-945,5964966	-0,099495
Cloud_Mesh_Distance_BA_R0_R2	21,24761963	-81,54673767	-945,5419312	-0,080449
Cloud_Mesh_Distance_BA_R0_R2	21,42830276	-81,36178589	-945,3452148	-0,109494
Cloud_Mesh_Distance_BA_R0_R2	21,44094849	-81,54878235	-945,4287109	-0,07191
Cloud_Mesh_Distance_BA_R0_R2	16,06361198	-80,54872131	-945,5106812	-0,055513
Cloud_Mesh_Distance_BA_R0_R2	16,3189373	-80,68492126	-945,6100464	0,005996
Cloud_Mesh_Distance_BA_R0_R2	16,24728584	-80,73852539	-945,5701904	0,028944
Cloud_Mesh_Distance_BA_R0_R2	20,96493912	-81,26319885	-945,5722656	-0,134829
Cloud_Mesh_Distance_BA_R0_R2	21,09495735	-81,16279602	-945,4796753	-0,133808
Cloud_Mesh_Distance_BA_R0_R2	20,91133499	-81,08695984	-945,553894	-0,143783
Cloud_Mesh_Distance_BA_R0_R2	21,23607254	-81,1869812	-945,4064331	-0,130398
Cloud_Mesh_Distance_BA_R0_R2	21,27066422	-81,35046387	-945,4423218	-0,111239
Cloud_Mesh_Distance_BA_R0_R2	15,75499535	-80,28764343	-945,4187012	-0,095977
Cloud_Mesh_Distance_BA_R0_R2	15,92270565	-80,34552002	-945,5100708	-0,096377
Cloud_Mesh_Distance_BA_R0_R2	16,2176075	-80,63859558	-945,5684204	-0,019622
Cloud_Mesh_Distance_BA_R0_R2	16,21305847	-80,50740051	-945,6022339	-0,067638
Cloud_Mesh_Distance_BA_R0_R2	16,3250618	-80,47438049	-945,6690674	-0,069724
Cloud_Mesh_Distance_BA_R0_R2	16,33225822	-80,58651733	-945,6356812	-0,035551
Cloud_Mesh_Distance_BA_R0_R2	20,69931221	-80,95834351	-945,6290894	-0,14766
Cloud_Mesh_Distance_BA_R0_R2	20,82178116	-81,18968201	-945,617981	-0,147198
Cloud_Mesh_Distance_BA_R0_R2	21,10539246	-80,99620056	-945,4343872	-0,140125
Cloud_Mesh_Distance_BA_R0_R2	21,18509674	-81,07685852	-945,4072266	-0,136398
Cloud_Mesh_Distance_BA_R0_R2	15,65852356	-80,15861511	-945,4072876	-0,103961
Cloud_Mesh_Distance_BA_R0_R2	15,54190063	-80,0721283	-945,3400269	-0,097811
Cloud_Mesh_Distance_BA_R0_R2	15,7791996	-80,14215088	-945,5185547	-0,105638
Cloud_Mesh_Distance_BA_R0_R2	15,91243362	-80,17391968	-945,5952759	-0,107955
Cloud_Mesh_Distance_BA_R0_R2	16,21716309	-80,37741089	-945,6590576	-0,092393
Cloud_Mesh_Distance_BA_R0_R2	16,0978756	-80,40830994	-945,5834351	-0,091112
Cloud_Mesh_Distance_BA_R0_R2	16,06145477	-80,25471497	-945,6402588	-0,109259
Cloud_Mesh_Distance_BA_R0_R2	20,8192234	-80,90118408	-945,5643921	-0,144991

Cloud_Mesh_Distance_BA_R0_R2	21,07155609	-80,83898926	-945,425415	-0,139085
Cloud_Mesh_Distance_BA_R0_R2	20,98582077	-80,95439148	-945,4909668	-0,143185
Cloud_Mesh_Distance_BA_R0_R2	20,91534424	-80,73686218	-945,4920044	-0,140769
Cloud_Mesh_Distance_BA_R0_R2	15,63948059	-79,97949219	-945,4907227	-0,101121
Cloud_Mesh_Distance_BA_R0_R2	20,76707649	-80,71725464	-945,5632935	-0,14036
Cloud_Mesh_Distance_BA_R0_R2	20,64832115	-80,62890625	-945,6081543	-0,137177
Cloud_Mesh_Distance_BA_R0_R2	20,7776165	-80,51390076	-945,5300903	-0,134726
Cloud_Mesh_Distance_BA_R0_R2	15,58709431	-79,78138733	-945,5391846	-0,090364
Cloud_Mesh_Distance_BA_R0_R2	20,50922012	-80,37677002	-945,6380005	-0,129045
Cloud_Mesh_Distance_BA_R0_R2	20,50190926	-80,20704651	-945,6139526	-0,131144
Cloud_Mesh_Distance_BA_R0_R2	20,61906624	-80,47720337	-945,600769	-0,133483
Cloud_Mesh_Distance_BA_R0_R2	15,5793972	-79,64073181	-945,5892334	-0,083195
Cloud_Mesh_Distance_BA_R0_R2	15,48735428	-79,64724731	-945,4830933	-0,08145
Cloud_Mesh_Distance_BA_R0_R2	20,63674736	-80,32627869	-945,5700073	-0,133396
Cloud_Mesh_Distance_BA_R0_R2	20,45482063	-80,02973938	-945,6045532	-0,136279
Cloud_Mesh_Distance_BA_R0_R2	20,90931511	-80,38568115	-945,4442139	-0,132197
Cloud_Mesh_Distance_BA_R0_R2	20,76988983	-80,28050232	-945,4978027	-0,13275
Cloud_Mesh_Distance_BA_R0_R2	15,45477009	-79,4490509	-945,505249	-0,072194
Cloud_Mesh_Distance_BA_R0_R2	15,54763317	-79,4957428	-945,6038208	-0,072644
Cloud_Mesh_Distance_BA_R0_R2	20,36764526	-79,8503418	-945,6137695	-0,140112
Cloud_Mesh_Distance_BA_R0_R2	20,63387871	-80,1035614	-945,5324707	-0,136745
Cloud_Mesh_Distance_BA_R0_R2	20,74075127	-80,01704407	-945,4628296	-0,136584
Cloud_Mesh_Distance_BA_R0_R2	15,43216038	-79,31428528	-945,5043335	-0,060788
Cloud_Mesh_Distance_BA_R0_R2	15,51797676	-79,29438782	-945,6170044	-0,054492
Cloud_Mesh_Distance_BA_R0_R2	15,44458771	-79,17634583	-945,5402832	-0,050639
Cloud_Mesh_Distance_BA_R0_R2	20,22747803	-79,74412537	-945,6633911	-0,141696
Cloud_Mesh_Distance_BA_R0_R2	20,32936859	-79,68377686	-945,6040039	-0,144918
Cloud_Mesh_Distance_BA_R0_R2	20,5263195	-79,90232849	-945,5464478	-0,142059
Cloud_Mesh_Distance_BA_R0_R2	15,47257233	-79,03282166	-945,5861206	-0,045489
Cloud_Mesh_Distance_BA_R0_R2	15,37134933	-79,04740906	-945,442688	-0,047594
Cloud_Mesh_Distance_BA_R0_R2	15,46833897	-78,87435913	-945,5767212	-0,041368
Cloud_Mesh_Distance_BA_R0_R2	20,2324028	-79,52687073	-945,6286621	-0,143663
Cloud_Mesh_Distance_BA_R0_R2	20,36744499	-79,59217834	-945,5671997	-0,147681
Cloud_Mesh_Distance_BA_R0_R2	20,45309067	-79,71749878	-945,5466309	-0,145359
Cloud_Mesh_Distance_BA_R0_R2	15,37678814	-78,7973938	-945,440918	-0,030023
Cloud_Mesh_Distance_BA_R0_R2	20,37417984	-79,46742249	-945,5407715	-0,146512
Cloud_Mesh_Distance_BA_R0_R2	15,57653809	-78,59866333	-945,6695557	-0,042517
Cloud_Mesh_Distance_BA_R0_R2	15,52608967	-78,51846313	-945,5943604	-0,028014
Cloud_Mesh_Distance_BA_R0_R2	20,08804703	-79,25813293	-945,670105	-0,134998
Cloud_Mesh_Distance_BA_R0_R2	20,27222633	-79,33410645	-945,5738525	-0,144561
Cloud_Mesh_Distance_BA_R0_R2	20,17861938	-79,21072388	-945,6082764	-0,137822
Cloud_Mesh_Distance_BA_R0_R2	20,18978119	-79,07524109	-945,5772095	-0,139001
Cloud_Mesh_Distance_BA_R0_R2	20,28552628	-79,16548157	-945,5333862	-0,144633
Cloud_Mesh_Distance_BA_R0_R2	20,07779121	-79,09196472	-945,6528931	-0,129191
Cloud_Mesh_Distance_BA_R0_R2	15,545825	-78,36735535	-945,5775147	-0,023566
Cloud_Mesh_Distance_BA_R0_R2	15,55090523	-78,07818604	-945,4638672	-0,006499
Cloud_Mesh_Distance_BA_R0_R2	15,71585846	-78,1242981	-945,685791	-0,03225
Cloud_Mesh_Distance_BA_R0_R2	15,75473976	-77,98144531	-945,6694946	-0,025312
Cloud_Mesh_Distance_BA_R0_R2	15,59401894	-78,18914795	-945,5741577	-0,014071
Cloud_Mesh_Distance_BA_R0_R2	19,87886429	-78,51605225	-945,6898193	-0,101226

Cloud_Mesh_Distance_BA_R0_R2	20,08565712	-78,540802	-945,5427856	-0,115361
Cloud_Mesh_Distance_BA_R0_R2	19,97646523	-78,58323669	-945,6347046	-0,112978
Cloud_Mesh_Distance_BA_R0_R2	20,03246307	-78,74160767	-945,6235352	-0,122326
Cloud_Mesh_Distance_BA_R0_R2	15,64999771	-78,02355957	-945,569397	-0,015126
Cloud_Mesh_Distance_BA_R0_R2	19,67619133	-78,28266907	-945,7618408	-0,095797
Cloud_Mesh_Distance_BA_R0_R2	19,78439713	-78,44674683	-945,7368164	-0,094517
Cloud_Mesh_Distance_BA_R0_R2	19,75340271	-78,3447113	-945,7321167	-0,095222
Cloud_Mesh_Distance_BA_R0_R2	19,77328682	-78,22390747	-945,6864014	-0,094831
Cloud_Mesh_Distance_BA_R0_R2	19,86342239	-78,34687805	-945,6629028	-0,096968
Cloud_Mesh_Distance_BA_R0_R2	19,98945808	-78,42391968	-945,5913086	-0,105124
Cloud_Mesh_Distance_BA_R0_R2	15,78371429	-77,67085266	-945,5139771	-0,009477
Cloud_Mesh_Distance_BA_R0_R2	15,91326523	-77,63632202	-945,6377564	-0,016081
Cloud_Mesh_Distance_BA_R0_R2	20,00485229	-78,0594635	-945,4735718	-0,096722
Cloud_Mesh_Distance_BA_R0_R2	19,86180878	-78,1083374	-945,5927734	-0,096978
Cloud_Mesh_Distance_BA_R0_R2	19,97770691	-78,23547363	-945,5507813	-0,099551
Cloud_Mesh_Distance_BA_R0_R2	19,87106705	-78,23028564	-945,625061	-0,095477
Cloud_Mesh_Distance_BA_R0_R2	20,08560944	-78,15597534	-945,4438477	-0,098061
Cloud_Mesh_Distance_BA_R0_R2	15,84955788	-77,6282959	-945,5605469	-0,010603
Cloud_Mesh_Distance_BA_R0_R2	15,9971714	-77,44081116	-945,5524292	-0,017969
Cloud_Mesh_Distance_BA_R0_R2	16,16200638	-77,41262817	-945,6785278	-0,045411
Cloud_Mesh_Distance_BA_R0_R2	16,01668549	-77,54258728	-945,6652832	-0,024286
Cloud_Mesh_Distance_BA_R0_R2	19,4309082	-77,91731262	-945,7731323	-0,100291
Cloud_Mesh_Distance_BA_R0_R2	19,35394478	-77,72131348	-945,7275391	-0,108799
Cloud_Mesh_Distance_BA_R0_R2	19,73039246	-78,08888245	-945,6687622	-0,098885
Cloud_Mesh_Distance_BA_R0_R2	19,58893585	-77,84486389	-945,654419	-0,112652
Cloud_Mesh_Distance_BA_R0_R2	19,75291443	-77,94036865	-945,5988159	-0,106985
Cloud_Mesh_Distance_BA_R0_R2	16,28828621	-77,29856873	-945,6624146	-0,065309
Cloud_Mesh_Distance_BA_R0_R2	16,33727264	-77,37324524	-945,7647095	-0,069811
Cloud_Mesh_Distance_BA_R0_R2	16,56314468	-77,28211975	-945,7979126	-0,095809
Cloud_Mesh_Distance_BA_R0_R2	19,23391914	-77,69151306	-945,777771	-0,09773
Cloud_Mesh_Distance_BA_R0_R2	19,25727844	-77,82640076	-945,8276978	-0,09504
Cloud_Mesh_Distance_BA_R0_R2	19,27185059	-77,61340332	-945,7196045	-0,10272
Cloud_Mesh_Distance_BA_R0_R2	16,45336914	-77,25366211	-945,7188721	-0,089993
Cloud_Mesh_Distance_BA_R0_R2	16,31304741	-77,17602539	-945,541687	-0,071392
Cloud_Mesh_Distance_BA_R0_R2	16,52673721	-77,12434387	-945,6207886	-0,108704
Cloud_Mesh_Distance_BA_R0_R2	16,6774826	-77,06156921	-945,6270142	-0,126125
Cloud_Mesh_Distance_BA_R0_R2	19,01710129	-77,4929657	-945,7888794	-0,076908
Cloud_Mesh_Distance_BA_R0_R2	19,1531105	-77,57675171	-945,7639771	-0,092862
Cloud_Mesh_Distance_BA_R0_R2	19,26089096	-77,4871521	-945,6572876	-0,103604
Cloud_Mesh_Distance_BA_R0_R2	19,52494812	-77,41136169	-945,4666138	-0,109664
Cloud_Mesh_Distance_BA_R0_R2	19,34752083	-77,59754944	-945,670166	-0,1112
Cloud_Mesh_Distance_BA_R0_R2	19,57748604	-77,43304443	-945,4483643	-0,109563
Cloud_Mesh_Distance_BA_R0_R2	16,57833862	-77,05003357	-945,5623779	-0,117811
Cloud_Mesh_Distance_BA_R0_R2	16,97308159	-77,08786011	-945,7580566	-0,140416
Cloud_Mesh_Distance_BA_R0_R2	16,83605194	-77,0715332	-945,7009888	-0,133911
Cloud_Mesh_Distance_BA_R0_R2	16,80145645	-76,97564697	-945,5841675	-0,138329
Cloud_Mesh_Distance_BA_R0_R2	17,00159454	-76,97367859	-945,6560059	-0,14532
Cloud_Mesh_Distance_BA_R0_R2	17,13036346	-77,06947327	-945,7788696	-0,139756
Cloud_Mesh_Distance_BA_R0_R2	17,46828079	-76,9528656	-945,7355347	-0,123589
Cloud_Mesh_Distance_BA_R0_R2	18,57730484	-77,16583252	-945,7721558	-0,041901

Cloud_Mesh_Distance_BA_R0_R2	18,6781292	-77,21252441	-945,7687378	-0,044064
Cloud_Mesh_Distance_BA_R0_R2	18,88989258	-77,37533569	-945,7826538	-0,06235
Cloud_Mesh_Distance_BA_R0_R2	18,78913689	-77,25390625	-945,7512817	-0,050152
Cloud_Mesh_Distance_BA_R0_R2	18,88652611	-77,22241211	-945,6842041	-0,059586
Cloud_Mesh_Distance_BA_R0_R2	19,00943375	-77,39912415	-945,7387085	-0,075995
Cloud_Mesh_Distance_BA_R0_R2	16,89060783	-76,87843323	-945,5059204	-0,143104
Cloud_Mesh_Distance_BA_R0_R2	17,27563477	-76,82138062	-945,5743408	-0,137705
Cloud_Mesh_Distance_BA_R0_R2	17,25210381	-76,93365479	-945,6824341	-0,139493
Cloud_Mesh_Distance_BA_R0_R2	17,61347771	-76,85559082	-945,6606445	-0,103438
Cloud_Mesh_Distance_BA_R0_R2	17,4169693	-76,84288025	-945,6262207	-0,128064
Cloud_Mesh_Distance_BA_R0_R2	18,53843689	-77,07788086	-945,7171631	-0,044631
Cloud_Mesh_Distance_BA_R0_R2	18,64858246	-77,12625122	-945,7165527	-0,045202
Cloud_Mesh_Distance_BA_R0_R2	18,8429184	-77,1068573	-945,6159058	-0,057487
Cloud_Mesh_Distance_BA_R0_R2	18,75673103	-77,15557861	-945,694519	-0,047709
Cloud_Mesh_Distance_BA_R0_R2	17,35838318	-76,76222229	-945,5300903	-0,124325
Cloud_Mesh_Distance_BA_R0_R2	22,17701721	-82,35412598	-945,2932129	0,227121
Cloud_Mesh_Distance_BA_R0_R2	15,73104382	-81,24761963	-945,2468872	0,337747
Cloud_Mesh_Distance_BA_R0_R2	22,05172729	-82,15907288	-945,3173218	0,110539
Cloud_Mesh_Distance_BA_R0_R2	22,1653595	-82,22967529	-945,2409668	0,109048
Cloud_Mesh_Distance_BA_R0_R2	22,15281677	-82,08580017	-945,1682129	0,007486
Cloud_Mesh_Distance_BA_R0_R2	15,80445099	-81,07481384	-945,2842407	0,220676
Cloud_Mesh_Distance_BA_R0_R2	21,97609901	-81,83155823	-945,1695557	-0,02388
Cloud_Mesh_Distance_BA_R0_R2	21,89779282	-82,06636047	-945,4057007	0,027729
Cloud_Mesh_Distance_BA_R0_R2	22,03940773	-82,00898743	-945,2337036	0,006622
Cloud_Mesh_Distance_BA_R0_R2	22,26314926	-82,16914368	-945,1005249	0,009373
Cloud_Mesh_Distance_BA_R0_R2	15,80867386	-80,90274048	-945,2700806	0,104184
Cloud_Mesh_Distance_BA_R0_R2	21,72638893	-81,68217468	-945,2947388	-0,045585
Cloud_Mesh_Distance_BA_R0_R2	21,59885025	-81,60784912	-945,3502808	-0,05946
Cloud_Mesh_Distance_BA_R0_R2	21,78759575	-81,54634094	-945,1508179	-0,08748
Cloud_Mesh_Distance_BA_R0_R2	21,67251015	-81,55247498	-945,2560425	-0,077689
Cloud_Mesh_Distance_BA_R0_R2	21,86440659	-81,67622375	-945,1662598	-0,056683
Cloud_Mesh_Distance_BA_R0_R2	21,82842255	-81,78242493	-945,2745972	-0,02493
Cloud_Mesh_Distance_BA_R0_R2	15,73857307	-80,72891235	-945,2178345	0,008259
Cloud_Mesh_Distance_BA_R0_R2	15,71240997	-80,90286255	-945,1580811	0,087484
Cloud_Mesh_Distance_BA_R0_R2	15,85227585	-80,74055481	-945,3232422	0,022143
Cloud_Mesh_Distance_BA_R0_R2	15,94073105	-80,82685852	-945,3848877	0,072201
Cloud_Mesh_Distance_BA_R0_R2	21,56449127	-81,46359253	-945,2937622	-0,092285
Cloud_Mesh_Distance_BA_R0_R2	15,68265724	-80,47817993	-945,2557373	-0,066653
Cloud_Mesh_Distance_BA_R0_R2	15,55289459	-80,49046326	-945,0897827	-0,06946
Cloud_Mesh_Distance_BA_R0_R2	15,91315079	-80,51501465	-945,4302368	-0,060993
Cloud_Mesh_Distance_BA_R0_R2	15,82273674	-80,59094238	-945,3369141	-0,039596
Cloud_Mesh_Distance_BA_R0_R2	21,29807854	-81,08628845	-945,3395996	-0,135545
Cloud_Mesh_Distance_BA_R0_R2	21,33438873	-81,24159241	-945,3627319	-0,123635
Cloud_Mesh_Distance_BA_R0_R2	21,5209198	-81,33436584	-945,2650147	-0,117637
Cloud_Mesh_Distance_BA_R0_R2	15,54622459	-80,26998901	-945,2232666	-0,0908
Cloud_Mesh_Distance_BA_R0_R2	15,65871239	-80,28981018	-945,3324585	-0,092861
Cloud_Mesh_Distance_BA_R0_R2	15,7234211	-80,36875916	-945,3495483	-0,084111
Cloud_Mesh_Distance_BA_R0_R2	15,80216599	-80,42979431	-945,3848877	-0,076675
Cloud_Mesh_Distance_BA_R0_R2	21,29855919	-80,91090393	-945,3016968	-0,139879
Cloud_Mesh_Distance_BA_R0_R2	15,59887695	-80,19410706	-945,3289795	-0,09927

Cloud_Mesh_Distance_BA_R0_R2	21,19112778	-80,94168091	-945,3740845	-0,140912
Cloud_Mesh_Distance_BA_R0_R2	21,40491104	-81,0193634	-945,2523193	-0,140544
Cloud_Mesh_Distance_BA_R0_R2	15,42745781	-79,94709778	-945,2719727	-0,095834
Cloud_Mesh_Distance_BA_R0_R2	15,45258617	-80,10801697	-945,2109375	-0,094292
Cloud_Mesh_Distance_BA_R0_R2	15,36824703	-80,02923584	-945,1381226	-0,091019
Cloud_Mesh_Distance_BA_R0_R2	20,94361877	-80,56088257	-945,4528198	-0,135061
Cloud_Mesh_Distance_BA_R0_R2	21,03940582	-80,67253113	-945,4175415	-0,136576
Cloud_Mesh_Distance_BA_R0_R2	21,07203102	-80,49586487	-945,3742065	-0,131196
Cloud_Mesh_Distance_BA_R0_R2	21,21858025	-80,53282166	-945,2945557	-0,130476
Cloud_Mesh_Distance_BA_R0_R2	21,19201279	-80,72915649	-945,3381958	-0,137714
Cloud_Mesh_Distance_BA_R0_R2	15,29694176	-79,66088867	-945,206604	-0,079902
Cloud_Mesh_Distance_BA_R0_R2	15,39868259	-79,75566101	-945,3258667	-0,085324
Cloud_Mesh_Distance_BA_R0_R2	15,50667763	-79,8625946	-945,4136353	-0,090391
Cloud_Mesh_Distance_BA_R0_R2	21,18160248	-80,34434509	-945,2873535	-0,126153
Cloud_Mesh_Distance_BA_R0_R2	21,01151085	-80,24354553	-945,3661499	-0,128183
Cloud_Mesh_Distance_BA_R0_R2	20,87225723	-80,21885681	-945,4354858	-0,131395
Cloud_Mesh_Distance_BA_R0_R2	20,78112411	-80,13400269	-945,4659424	-0,133728
Cloud_Mesh_Distance_BA_R0_R2	20,88278198	-80,04800415	-945,3952637	-0,132615
Cloud_Mesh_Distance_BA_R0_R2	15,3831501	-79,5554657	-945,3777466	-0,073241
Cloud_Mesh_Distance_BA_R0_R2	15,37031746	-79,26341248	-945,4224243	-0,060834
Cloud_Mesh_Distance_BA_R0_R2	15,3869133	-79,39982605	-945,4232788	-0,06707
Cloud_Mesh_Distance_BA_R0_R2	20,650177	-79,93421936	-945,4911499	-0,139929
Cloud_Mesh_Distance_BA_R0_R2	20,75035095	-79,86401367	-945,4230957	-0,139478
Cloud_Mesh_Distance_BA_R0_R2	20,87310791	-79,88822937	-945,3629761	-0,133763
Cloud_Mesh_Distance_BA_R0_R2	21,137043	-80,12225342	-945,2692261	-0,123318
Cloud_Mesh_Distance_BA_R0_R2	21,00809097	-79,92628479	-945,2964478	-0,128882
Cloud_Mesh_Distance_BA_R0_R2	21,01151848	-80,08625793	-945,3338013	-0,127786
Cloud_Mesh_Distance_BA_R0_R2	15,23953629	-79,18656921	-945,2040405	-0,051092
Cloud_Mesh_Distance_BA_R0_R2	15,29771805	-79,22514343	-945,3082886	-0,054668
Cloud_Mesh_Distance_BA_R0_R2	21,02408981	-79,73474121	-945,2316895	-0,127745
Cloud_Mesh_Distance_BA_R0_R2	21,1723671	-79,95970154	-945,2076416	-0,120105
Cloud_Mesh_Distance_BA_R0_R2	21,10061836	-79,84843445	-945,2196045	-0,122847
Cloud_Mesh_Distance_BA_R0_R2	15,28470993	-79,08732605	-945,2964478	-0,041885
Cloud_Mesh_Distance_BA_R0_R2	15,29579163	-78,93212891	-945,3168335	-0,034578
Cloud_Mesh_Distance_BA_R0_R2	15,22724915	-79,04804993	-945,1851196	-0,041116
Cloud_Mesh_Distance_BA_R0_R2	20,53567886	-79,6446991	-945,487793	-0,145115
Cloud_Mesh_Distance_BA_R0_R2	20,5958252	-79,78630066	-945,4869995	-0,14466
Cloud_Mesh_Distance_BA_R0_R2	20,52301979	-79,48854065	-945,4594116	-0,148533
Cloud_Mesh_Distance_BA_R0_R2	20,65716553	-79,66267395	-945,4259644	-0,145141
Cloud_Mesh_Distance_BA_R0_R2	20,84516716	-79,71092224	-945,3314209	-0,138572
Cloud_Mesh_Distance_BA_R0_R2	15,30364513	-78,77763367	-945,3172607	-0,026178
Cloud_Mesh_Distance_BA_R0_R2	15,34683609	-78,63874817	-945,3668823	-0,019014
Cloud_Mesh_Distance_BA_R0_R2	15,40582752	-78,64935303	-945,4619141	-0,024754
Cloud_Mesh_Distance_BA_R0_R2	20,45022964	-79,57926941	-945,5206909	-0,145796
Cloud_Mesh_Distance_BA_R0_R2	20,67730331	-79,33451843	-945,3272095	-0,142634
Cloud_Mesh_Distance_BA_R0_R2	20,69173431	-79,48797607	-945,3605957	-0,144237
Cloud_Mesh_Distance_BA_R0_R2	20,76528168	-79,40652466	-945,2920532	-0,141459
Cloud_Mesh_Distance_BA_R0_R2	15,34929466	-78,5004425	-945,3337402	-0,008707
Cloud_Mesh_Distance_BA_R0_R2	15,4321022	-78,49272156	-945,4631958	-0,018506
Cloud_Mesh_Distance_BA_R0_R2	20,55474091	-79,29255676	-945,3927002	-0,149422

Cloud_Mesh_Distance_BA_R0_R2	20,40274048	-79,30145264	-945,4891968	-0,149452
Cloud_Mesh_Distance_BA_R0_R2	20,63316727	-79,22869873	-945,3257446	-0,145198
Cloud_Mesh_Distance_BA_R0_R2	15,47483063	-78,2440033	-945,4338989	-0,006539
Cloud_Mesh_Distance_BA_R0_R2	20,26525497	-79,00822449	-945,512207	-0,143882
Cloud_Mesh_Distance_BA_R0_R2	20,41521072	-79,07728577	-945,4275513	-0,14734
Cloud_Mesh_Distance_BA_R0_R2	20,55371094	-79,14378357	-945,3540039	-0,14699
Cloud_Mesh_Distance_BA_R0_R2	15,32932663	-78,26220703	-945,178772	0,001814
Cloud_Mesh_Distance_BA_R0_R2	15,377388	-78,35620117	-945,3266602	-0,001361
Cloud_Mesh_Distance_BA_R0_R2	20,21000099	-78,63131714	-945,4627075	-0,129146
Cloud_Mesh_Distance_BA_R0_R2	20,30014229	-78,8263092	-945,4431152	-0,142463
Cloud_Mesh_Distance_BA_R0_R2	20,15036011	-78,90617371	-945,5704956	-0,136968
Cloud_Mesh_Distance_BA_R0_R2	20,25224304	-78,65142822	-945,4346314	-0,134387
Cloud_Mesh_Distance_BA_R0_R2	20,32794952	-78,68307495	-945,3839111	-0,137512
Cloud_Mesh_Distance_BA_R0_R2	20,44910812	-78,88652039	-945,3536987	-0,141065
Cloud_Mesh_Distance_BA_R0_R2	20,40707016	-78,7383728	-945,3413696	-0,137166
Cloud_Mesh_Distance_BA_R0_R2	20,20729065	-78,59649658	-945,4567871	-0,127094
Cloud_Mesh_Distance_BA_R0_R2	20,17589569	-78,67382813	-945,4998779	-0,127129
Cloud_Mesh_Distance_BA_R0_R2	20,5924511	-78,71231079	-945,1960449	-0,117576
Cloud_Mesh_Distance_BA_R0_R2	20,51411819	-78,7820282	-945,2758789	-0,131794
Cloud_Mesh_Distance_BA_R0_R2	15,69454193	-77,74272156	-945,4489746	-0,001245
Cloud_Mesh_Distance_BA_R0_R2	15,59775162	-77,73310852	-945,274231	0,000599
Cloud_Mesh_Distance_BA_R0_R2	15,59532928	-77,90524292	-945,4223633	0,000124
Cloud_Mesh_Distance_BA_R0_R2	15,53091621	-77,87692261	-945,2867432	0,003644
Cloud_Mesh_Distance_BA_R0_R2	15,72607994	-77,86399841	-945,5771484	-0,013344
Cloud_Mesh_Distance_BA_R0_R2	15,81165504	-77,78401184	-945,6286011	-0,017808
Cloud_Mesh_Distance_BA_R0_R2	20,248312	-78,29727173	-945,348938	-0,101598
Cloud_Mesh_Distance_BA_R0_R2	15,6820755	-77,66734314	-945,3572388	0,001025
Cloud_Mesh_Distance_BA_R0_R2	15,75061417	-77,61654663	-945,4160156	-0,00343
Cloud_Mesh_Distance_BA_R0_R2	15,83404732	-77,70162964	-945,5997315	-0,012737
Cloud_Mesh_Distance_BA_R0_R2	15,76737404	-77,75248718	-945,5559082	-0,010724
Cloud_Mesh_Distance_BA_R0_R2	20,189785	-78,09030151	-945,3345337	-0,087374
Cloud_Mesh_Distance_BA_R0_R2	20,14480209	-78,31451416	-945,4405518	-0,109516
Cloud_Mesh_Distance_BA_R0_R2	20,33847237	-78,29299927	-945,2700195	-0,096289
Cloud_Mesh_Distance_BA_R0_R2	15,84796715	-77,4604187	-945,3874512	-0,013561
Cloud_Mesh_Distance_BA_R0_R2	15,90407753	-77,54736328	-945,5533447	-0,009832
Cloud_Mesh_Distance_BA_R0_R2	15,82368279	-77,58450317	-945,4891968	-0,008914
Cloud_Mesh_Distance_BA_R0_R2	15,99378586	-77,35351563	-945,4508667	-0,024716
Cloud_Mesh_Distance_BA_R0_R2	16,08658981	-77,34764099	-945,545105	-0,036894
Cloud_Mesh_Distance_BA_R0_R2	19,47620583	-77,76675415	-945,6818848	-0,114625
Cloud_Mesh_Distance_BA_R0_R2	19,89689636	-77,96864319	-945,5164795	-0,099427
Cloud_Mesh_Distance_BA_R0_R2	19,6971035	-77,7277832	-945,5376587	-0,109244
Cloud_Mesh_Distance_BA_R0_R2	20,09981537	-77,96678162	-945,3631592	-0,087398
Cloud_Mesh_Distance_BA_R0_R2	20,09751129	-78,07092285	-945,4051514	-0,092263
Cloud_Mesh_Distance_BA_R0_R2	19,44684219	-77,64343262	-945,638794	-0,112373
Cloud_Mesh_Distance_BA_R0_R2	19,55277252	-77,70597839	-945,6108398	-0,112113
Cloud_Mesh_Distance_BA_R0_R2	19,85865402	-77,82806396	-945,4818726	-0,099448
Cloud_Mesh_Distance_BA_R0_R2	19,95306396	-77,75454712	-945,3798218	-0,093179
Cloud_Mesh_Distance_BA_R0_R2	16,10711479	-77,24757385	-945,4447632	-0,046838
Cloud_Mesh_Distance_BA_R0_R2	16,18909073	-77,12574768	-945,3613281	-0,066688
Cloud_Mesh_Distance_BA_R0_R2	16,1883812	-77,29391479	-945,5797119	-0,052785

Cloud_Mesh_Distance_BA_R0_R2	19,39593124	-77,52664185	-945,6057129	-0,112299
Cloud_Mesh_Distance_BA_R0_R2	19,54568481	-77,54170227	-945,5296631	-0,112988
Cloud_Mesh_Distance_BA_R0_R2	19,56782722	-77,46551514	-945,4734497	-0,114274
Cloud_Mesh_Distance_BA_R0_R2	19,62326431	-77,46469116	-945,4399414	-0,109726
Cloud_Mesh_Distance_BA_R0_R2	16,31870651	-77,07049561	-945,4055176	-0,083722
Cloud_Mesh_Distance_BA_R0_R2	16,46608543	-77,04354858	-945,4827271	-0,106443
Cloud_Mesh_Distance_BA_R0_R2	16,60344124	-76,86686707	-945,3242798	-0,135592
Cloud_Mesh_Distance_BA_R0_R2	16,6354351	-76,97950745	-945,5057983	-0,123078
Cloud_Mesh_Distance_BA_R0_R2	16,51408386	-76,93650818	-945,366272	-0,119053
Cloud_Mesh_Distance_BA_R0_R2	16,65073776	-76,90333557	-945,4108276	-0,130506
Cloud_Mesh_Distance_BA_R0_R2	19,10606194	-77,42782593	-945,7058106	-0,086175
Cloud_Mesh_Distance_BA_R0_R2	19,0141468	-77,31277466	-945,6821289	-0,078864
Cloud_Mesh_Distance_BA_R0_R2	19,21591187	-77,17930603	-945,4816895	-0,087885
Cloud_Mesh_Distance_BA_R0_R2	19,16557503	-77,31797791	-945,6062622	-0,093795
Cloud_Mesh_Distance_BA_R0_R2	19,35589218	-77,21473694	-945,4311523	-0,0962
Cloud_Mesh_Distance_BA_R0_R2	19,37112045	-77,36665344	-945,5255737	-0,106979
Cloud_Mesh_Distance_BA_R0_R2	19,48957443	-77,27560425	-945,3968506	-0,101722
Cloud_Mesh_Distance_BA_R0_R2	19,59518814	-77,37864685	-945,4026489	-0,10344
Cloud_Mesh_Distance_BA_R0_R2	16,74744415	-76,91156006	-945,4784546	-0,134443
Cloud_Mesh_Distance_BA_R0_R2	17,0861702	-76,74403381	-945,4205933	-0,14604
Cloud_Mesh_Distance_BA_R0_R2	16,93145752	-76,77061462	-945,3845215	-0,147469
Cloud_Mesh_Distance_BA_R0_R2	17,09878922	-76,83337402	-945,5349121	-0,143973
Cloud_Mesh_Distance_BA_R0_R2	17,22297287	-76,74687195	-945,4750977	-0,135324
Cloud_Mesh_Distance_BA_R0_R2	17,78212547	-76,82258606	-945,6328735	-0,08861
Cloud_Mesh_Distance_BA_R0_R2	17,93123627	-76,85334778	-945,6533814	-0,076956
Cloud_Mesh_Distance_BA_R0_R2	18,22066307	-76,7890625	-945,5388794	-0,05352
Cloud_Mesh_Distance_BA_R0_R2	18,10777283	-76,88374329	-945,6574707	-0,062899
Cloud_Mesh_Distance_BA_R0_R2	18,49344063	-76,96273804	-945,631958	-0,045262
Cloud_Mesh_Distance_BA_R0_R2	18,29892731	-76,9161377	-945,6464844	-0,050014
Cloud_Mesh_Distance_BA_R0_R2	18,6847496	-77,03823853	-945,6276856	-0,049385
Cloud_Mesh_Distance_BA_R0_R2	18,86767006	-76,9826355	-945,4931641	-0,059994
Cloud_Mesh_Distance_BA_R0_R2	19,01296806	-77,15957642	-945,5734863	-0,071029
Cloud_Mesh_Distance_BA_R0_R2	17,50273132	-76,74980164	-945,5429077	-0,10977
Cloud_Mesh_Distance_BA_R0_R2	17,75115395	-76,65119934	-945,4500732	-0,080779
Cloud_Mesh_Distance_BA_R0_R2	17,6859951	-76,74284363	-945,5511475	-0,097327
Cloud_Mesh_Distance_BA_R0_R2	18,04216385	-76,78884888	-945,5759277	-0,065603
Cloud_Mesh_Distance_BA_R0_R2	17,88175201	-76,73904419	-945,5430908	-0,077351
Cloud_Mesh_Distance_BA_R0_R2	18,2315731	-76,69616699	-945,4334717	-0,058109
Cloud_Mesh_Distance_BA_R0_R2	18,06835175	-76,69172668	-945,4653931	-0,064861
Cloud_Mesh_Distance_BA_R0_R2	18,42862701	-76,83898926	-945,53125	-0,046109
Cloud_Mesh_Distance_BA_R0_R2	18,55416679	-76,76463318	-945,4039307	-0,052722
Cloud_Mesh_Distance_BA_R0_R2	18,63679123	-76,89253235	-945,5083618	-0,046948
Cloud_Mesh_Distance_BA_R0_R2	19,08551979	-77,03559875	-945,432312	-0,076305
Cloud_Mesh_Distance_BA_R0_R2	19,01340103	-76,89060974	-945,3261719	-0,067128
Cloud_Mesh_Distance_BA_R0_R2	19,18596458	-76,96437073	-945,3082275	-0,076838
Cloud_Mesh_Distance_BA_R0_R2	17,64645767	-76,58248901	-945,361145	-0,086398
Cloud_Mesh_Distance_BA_R0_R2	17,81303406	-76,55630493	-945,3309937	-0,07257
Cloud_Mesh_Distance_BA_R0_R2	17,90512848	-76,64308167	-945,4325562	-0,069919
Cloud_Mesh_Distance_BA_R0_R2	18,01252365	-76,58564758	-945,3480225	-0,063303
Cloud_Mesh_Distance_BA_R0_R2	22,56184769	-82,54890442	-944,9301758	-0,205113

Cloud_Mesh_Distance_BA_R0_R2	22,2686615	-82,32572937	-945,1790772	0,14358
Cloud_Mesh_Distance_BA_R0_R2	22,38529396	-82,40158081	-945,0795288	0,267432
Cloud_Mesh_Distance_BA_R0_R2	22,44707489	-82,29067993	-944,9499512	-0,154269
Cloud_Mesh_Distance_BA_R0_R2	15,57555676	-81,31619263	-944,9015503	0,293429
Cloud_Mesh_Distance_BA_R0_R2	15,66083717	-81,23323059	-945,1210327	0,296688
Cloud_Mesh_Distance_BA_R0_R2	15,61050224	-81,3115387	-945,0363159	0,3353
Cloud_Mesh_Distance_BA_R0_R2	22,09516144	-81,8193512	-945,0404663	-0,033509
Cloud_Mesh_Distance_BA_R0_R2	22,13991165	-81,92408752	-945,0700073	-0,014145
Cloud_Mesh_Distance_BA_R0_R2	22,25210953	-82,03157043	-945,0236816	-0,005348
Cloud_Mesh_Distance_BA_R0_R2	22,33563614	-82,13322449	-944,9946899	0,036383
Cloud_Mesh_Distance_BA_R0_R2	22,35407257	-82,01455688	-944,8919678	-0,00717
Cloud_Mesh_Distance_BA_R0_R2	22,39986229	-82,11581421	-944,904541	0,073243
Cloud_Mesh_Distance_BA_R0_R2	15,628685	-81,17024231	-945,0302124	0,232469
Cloud_Mesh_Distance_BA_R0_R2	15,589468	-81,10682678	-944,9064331	0,156326
Cloud_Mesh_Distance_BA_R0_R2	15,71102905	-81,07263184	-945,1651611	0,193825
Cloud_Mesh_Distance_BA_R0_R2	15,64623833	-80,99147034	-945,0501709	0,120266
Cloud_Mesh_Distance_BA_R0_R2	15,66030312	-80,83287048	-945,093689	0,041801
Cloud_Mesh_Distance_BA_R0_R2	21,56886673	-81,24855042	-945,1939697	-0,130012
Cloud_Mesh_Distance_BA_R0_R2	21,60252953	-81,36494446	-945,2141724	-0,115359
Cloud_Mesh_Distance_BA_R0_R2	21,68264771	-81,44013977	-945,184082	-0,104988
Cloud_Mesh_Distance_BA_R0_R2	21,67351913	-81,30813599	-945,1299439	-0,127636
Cloud_Mesh_Distance_BA_R0_R2	21,79168892	-81,37562561	-945,0547485	-0,124674
Cloud_Mesh_Distance_BA_R0_R2	21,88309097	-81,58390808	-945,0857544	-0,083927
Cloud_Mesh_Distance_BA_R0_R2	21,98458862	-81,61654663	-945,0076294	-0,084136
Cloud_Mesh_Distance_BA_R0_R2	15,62515259	-80,67773438	-945,0935669	-0,023714
Cloud_Mesh_Distance_BA_R0_R2	15,47835732	-80,51829529	-944,9447022	-0,08287
Cloud_Mesh_Distance_BA_R0_R2	21,43982124	-81,20271301	-945,2766724	-0,12899
Cloud_Mesh_Distance_BA_R0_R2	21,64839554	-81,15504456	-945,0977173	-0,145335
Cloud_Mesh_Distance_BA_R0_R2	15,45694923	-80,34284973	-945,0510864	-0,092144
Cloud_Mesh_Distance_BA_R0_R2	15,36962128	-80,28303528	-944,9466553	-0,093441
Cloud_Mesh_Distance_BA_R0_R2	21,52696991	-81,08920288	-945,1779175	-0,143072
Cloud_Mesh_Distance_BA_R0_R2	21,61912537	-80,95933533	-945,0776978	-0,148813
Cloud_Mesh_Distance_BA_R0_R2	15,34400749	-80,1395874	-945,0202026	-0,093726
Cloud_Mesh_Distance_BA_R0_R2	15,45061016	-80,21107483	-945,138916	-0,096287
Cloud_Mesh_Distance_BA_R0_R2	15,40368271	-80,21887207	-945,0612793	-0,097243
Cloud_Mesh_Distance_BA_R0_R2	15,4025383	-80,13771057	-945,1196289	-0,09361
Cloud_Mesh_Distance_BA_R0_R2	21,38525963	-80,88365173	-945,2385254	-0,142352
Cloud_Mesh_Distance_BA_R0_R2	21,41848183	-80,76567078	-945,1975708	-0,140478
Cloud_Mesh_Distance_BA_R0_R2	21,48311424	-80,91769409	-945,175415	-0,143562
Cloud_Mesh_Distance_BA_R0_R2	21,54646301	-80,76490784	-945,1051025	-0,141363
Cloud_Mesh_Distance_BA_R0_R2	15,24889183	-79,80534363	-945,0568237	-0,088226
Cloud_Mesh_Distance_BA_R0_R2	15,33045292	-79,86434937	-945,1724243	-0,086894
Cloud_Mesh_Distance_BA_R0_R2	21,3229599	-80,78858948	-945,2648926	-0,138638
Cloud_Mesh_Distance_BA_R0_R2	21,45281792	-80,58157349	-945,1494751	-0,13176
Cloud_Mesh_Distance_BA_R0_R2	15,19222069	-79,75491333	-944,9638672	-0,081078
Cloud_Mesh_Distance_BA_R0_R2	21,32255554	-80,44908142	-945,2177734	-0,126737
Cloud_Mesh_Distance_BA_R0_R2	21,32144165	-80,64247131	-945,2462158	-0,133176
Cloud_Mesh_Distance_BA_R0_R2	15,22688675	-79,53422546	-945,118042	-0,073187
Cloud_Mesh_Distance_BA_R0_R2	15,22441292	-79,67932129	-945,0648193	-0,078956
Cloud_Mesh_Distance_BA_R0_R2	15,16982079	-79,63224792	-944,9627686	-0,078923

Cloud_Mesh_Distance_BA_R0_R2	15,15966225	-79,45381165	-944,9910278	-0,071639
Cloud_Mesh_Distance_BA_R0_R2	15,3080616	-79,42597961	-945,293335	-0,072097
Cloud_Mesh_Distance_BA_R0_R2	21,43865204	-80,36923218	-945,1278076	-0,120736
Cloud_Mesh_Distance_BA_R0_R2	21,55115318	-80,46005249	-945,0618286	-0,124513
Cloud_Mesh_Distance_BA_R0_R2	21,5500412	-80,25819397	-945,0256958	-0,115129
Cloud_Mesh_Distance_BA_R0_R2	21,24188614	-80,06924438	-945,192688	-0,11992
Cloud_Mesh_Distance_BA_R0_R2	21,31644058	-80,21974182	-945,1804199	-0,118235
Cloud_Mesh_Distance_BA_R0_R2	21,43524551	-80,24230957	-945,1054688	-0,116106
Cloud_Mesh_Distance_BA_R0_R2	15,22932434	-79,34320068	-945,1644898	-0,064306
Cloud_Mesh_Distance_BA_R0_R2	21,11081314	-79,78149414	-945,1923828	-0,12317
Cloud_Mesh_Distance_BA_R0_R2	21,17945099	-79,82647705	-945,1623535	-0,120778
Cloud_Mesh_Distance_BA_R0_R2	21,25342941	-79,99755859	-945,166626	-0,117127
Cloud_Mesh_Distance_BA_R0_R2	20,92373848	-79,59321594	-945,249939	-0,135273
Cloud_Mesh_Distance_BA_R0_R2	20,88912582	-79,42250061	-945,2163086	-0,140869
Cloud_Mesh_Distance_BA_R0_R2	21,03276062	-79,56433105	-945,1695557	-0,133494
Cloud_Mesh_Distance_BA_R0_R2	15,22983742	-78,84013367	-945,1801148	-0,029321
Cloud_Mesh_Distance_BA_R0_R2	20,82329941	-79,52490234	-945,2909546	-0,139407
Cloud_Mesh_Distance_BA_R0_R2	20,74818802	-79,26229858	-945,2593994	-0,142851
Cloud_Mesh_Distance_BA_R0_R2	15,27739525	-78,68447876	-945,2510986	-0,015757
Cloud_Mesh_Distance_BA_R0_R2	15,3029623	-78,44485474	-945,2228394	-0,007077
Cloud_Mesh_Distance_BA_R0_R2	15,21786404	-78,60098267	-945,0951538	-0,016227
Cloud_Mesh_Distance_BA_R0_R2	15,29743004	-78,57246399	-945,2602539	-0,009624
Cloud_Mesh_Distance_BA_R0_R2	20,64235878	-79,11781311	-945,2868652	-0,143584
Cloud_Mesh_Distance_BA_R0_R2	20,88057518	-79,23796082	-945,1606445	-0,135279
Cloud_Mesh_Distance_BA_R0_R2	20,76185989	-79,0891571	-945,1953125	-0,131583
Cloud_Mesh_Distance_BA_R0_R2	20,9660759	-79,17904663	-945,0764771	-0,130803
Cloud_Mesh_Distance_BA_R0_R2	20,53729057	-79,0141449	-945,3289795	-0,142054
Cloud_Mesh_Distance_BA_R0_R2	20,73560715	-78,91760254	-945,1575317	-0,122427
Cloud_Mesh_Distance_BA_R0_R2	20,83120346	-79,00354004	-945,1149292	-0,122753
Cloud_Mesh_Distance_BA_R0_R2	20,63776779	-78,97679138	-945,2479248	-0,135813
Cloud_Mesh_Distance_BA_R0_R2	15,40375996	-78,20021057	-945,2917481	0,004911
Cloud_Mesh_Distance_BA_R0_R2	20,31957626	-78,58157349	-945,3638916	-0,131974
Cloud_Mesh_Distance_BA_R0_R2	20,38868713	-78,628479	-945,3237915	-0,130669
Cloud_Mesh_Distance_BA_R0_R2	20,54800224	-78,89646912	-945,2872925	-0,138069
Cloud_Mesh_Distance_BA_R0_R2	20,62314606	-78,83358765	-945,2131958	-0,1246
Cloud_Mesh_Distance_BA_R0_R2	15,38561153	-78,11442566	-945,194458	0,003978
Cloud_Mesh_Distance_BA_R0_R2	15,47144032	-78,03067017	-945,302002	0,003876
Cloud_Mesh_Distance_BA_R0_R2	20,22367477	-78,61012268	-945,446167	-0,130297
Cloud_Mesh_Distance_BA_R0_R2	20,19941139	-78,50335693	-945,4415283	-0,123304
Cloud_Mesh_Distance_BA_R0_R2	20,24698257	-78,5660553	-945,4171753	-0,131013
Cloud_Mesh_Distance_BA_R0_R2	20,49266815	-78,63757324	-945,2472534	-0,121508
Cloud_Mesh_Distance_BA_R0_R2	20,31001472	-78,45742798	-945,3386231	-0,118669
Cloud_Mesh_Distance_BA_R0_R2	20,61807251	-78,58987427	-945,1342773	-0,107342
Cloud_Mesh_Distance_BA_R0_R2	15,54386044	-77,70327759	-945,121521	0,003209
Cloud_Mesh_Distance_BA_R0_R2	20,24687958	-78,37359619	-945,3696899	-0,110903
Cloud_Mesh_Distance_BA_R0_R2	20,41748047	-78,37579346	-945,2266235	-0,104443
Cloud_Mesh_Distance_BA_R0_R2	20,53389359	-78,51347351	-945,1754761	-0,109204
Cloud_Mesh_Distance_BA_R0_R2	20,42248344	-78,51156616	-945,263794	-0,117674
Cloud_Mesh_Distance_BA_R0_R2	15,69916344	-77,57772827	-945,2876587	0,003687
Cloud_Mesh_Distance_BA_R0_R2	20,26513672	-78,20373535	-945,3063355	-0,092289

Cloud_Mesh_Distance_BA_R0_R2	20,28460693	-78,10415649	-945,255188	-0,085389
Cloud_Mesh_Distance_BA_R0_R2	20,37524796	-78,11590576	-945,1766968	-0,080648
Cloud_Mesh_Distance_BA_R0_R2	15,74085236	-77,43507385	-945,1732788	-0,005941
Cloud_Mesh_Distance_BA_R0_R2	15,84063911	-77,34216309	-945,2168579	-0,019516
Cloud_Mesh_Distance_BA_R0_R2	19,9871788	-77,89483643	-945,4205322	-0,090561
Cloud_Mesh_Distance_BA_R0_R2	20,30204391	-77,99938965	-945,197876	-0,073899
Cloud_Mesh_Distance_BA_R0_R2	15,88865852	-77,25382996	-945,1598511	-0,025416
Cloud_Mesh_Distance_BA_R0_R2	15,97718048	-77,17471313	-945,1712036	-0,040642
Cloud_Mesh_Distance_BA_R0_R2	16,06144905	-77,17959595	-945,2961426	-0,042712
Cloud_Mesh_Distance_BA_R0_R2	15,95338058	-77,2842865	-945,3063355	-0,030291
Cloud_Mesh_Distance_BA_R0_R2	19,71464539	-77,55764771	-945,4371948	-0,11019
Cloud_Mesh_Distance_BA_R0_R2	19,84158707	-77,66822815	-945,4146118	-0,101347
Cloud_Mesh_Distance_BA_R0_R2	20,08527374	-77,77627563	-945,2867432	-0,080188
Cloud_Mesh_Distance_BA_R0_R2	20,20964432	-77,89544678	-945,2368774	-0,072368
Cloud_Mesh_Distance_BA_R0_R2	20,23048973	-77,6947937	-945,1074219	-0,057857
Cloud_Mesh_Distance_BA_R0_R2	19,83130646	-77,53601074	-945,3464356	-0,093922
Cloud_Mesh_Distance_BA_R0_R2	19,73467636	-77,39891052	-945,3256836	-0,099766
Cloud_Mesh_Distance_BA_R0_R2	19,88042068	-77,45428467	-945,2573242	-0,084705
Cloud_Mesh_Distance_BA_R0_R2	19,98909569	-77,41856384	-945,1410523	-0,076777
Cloud_Mesh_Distance_BA_R0_R2	19,97408104	-77,59407043	-945,274292	-0,081108
Cloud_Mesh_Distance_BA_R0_R2	20,10671806	-77,62889099	-945,1837769	-0,069924
Cloud_Mesh_Distance_BA_R0_R2	20,15629196	-77,56192017	-945,092041	-0,063457
Cloud_Mesh_Distance_BA_R0_R2	16,38135147	-76,99707031	-945,3510132	-0,099646
Cloud_Mesh_Distance_BA_R0_R2	16,25579643	-77,02516174	-945,2765503	-0,086402
Cloud_Mesh_Distance_BA_R0_R2	16,503582	-76,8682251	-945,2493286	-0,120737
Cloud_Mesh_Distance_BA_R0_R2	19,54937744	-77,15168762	-945,2606812	-0,09624
Cloud_Mesh_Distance_BA_R0_R2	19,62046051	-77,28022766	-945,3173218	-0,097454
Cloud_Mesh_Distance_BA_R0_R2	19,72955704	-77,20294189	-945,1751709	-0,091125
Cloud_Mesh_Distance_BA_R0_R2	16,60205078	-76,80291748	-945,2200317	-0,131175
Cloud_Mesh_Distance_BA_R0_R2	16,74248695	-76,83377075	-945,3677979	-0,140663
Cloud_Mesh_Distance_BA_R0_R2	19,29971695	-77,12574768	-945,392273	-0,09156
Cloud_Mesh_Distance_BA_R0_R2	19,40597343	-77,13964844	-945,3427124	-0,095738
Cloud_Mesh_Distance_BA_R0_R2	19,31144905	-77,04219055	-945,3125	-0,08551
Cloud_Mesh_Distance_BA_R0_R2	19,436203	-77,04499817	-945,2380981	-0,087667
Cloud_Mesh_Distance_BA_R0_R2	16,9099865	-76,70005798	-945,2686768	-0,14547
Cloud_Mesh_Distance_BA_R0_R2	17,01458168	-76,70370483	-945,3325195	-0,144625
Cloud_Mesh_Distance_BA_R0_R2	17,3249321	-76,56414795	-945,269104	-0,112032
Cloud_Mesh_Distance_BA_R0_R2	17,25877953	-76,53390503	-945,199707	-0,116251
Cloud_Mesh_Distance_BA_R0_R2	17,17662621	-76,58399963	-945,2381592	-0,129632
Cloud_Mesh_Distance_BA_R0_R2	17,34914589	-76,65885925	-945,4041748	-0,118878
Cloud_Mesh_Distance_BA_R0_R2	17,14212799	-76,6756897	-945,3529663	-0,139276
Cloud_Mesh_Distance_BA_R0_R2	17,47846413	-76,56874084	-945,3183594	-0,100977
Cloud_Mesh_Distance_BA_R0_R2	17,57073975	-76,65605164	-945,4448853	-0,097587
Cloud_Mesh_Distance_BA_R0_R2	18,36843109	-76,73257446	-945,4348755	-0,052514
Cloud_Mesh_Distance_BA_R0_R2	18,64208031	-76,68075562	-945,2683106	-0,046869
Cloud_Mesh_Distance_BA_R0_R2	18,78960991	-76,79901123	-945,3392944	-0,059713
Cloud_Mesh_Distance_BA_R0_R2	18,99096298	-76,74780273	-945,1744995	-0,064193
Cloud_Mesh_Distance_BA_R0_R2	19,16253471	-76,84259033	-945,1887817	-0,077452
Cloud_Mesh_Distance_BA_R0_R2	19,21809578	-77,06622314	-945,3869019	-0,085168
Cloud_Mesh_Distance_BA_R0_R2	19,33234596	-76,93110657	-945,1844482	-0,08422

Cloud_Mesh_Distance_BA_R0_R2	19,53687477	-77,05241394	-945,1766968	-0,088725
Cloud_Mesh_Distance_BA_R0_R2	17,80213928	-76,46820068	-945,2106323	-0,064089
Cloud_Mesh_Distance_BA_R0_R2	17,95992851	-76,49330139	-945,2341919	-0,05429
Cloud_Mesh_Distance_BA_R0_R2	18,18644333	-76,61885071	-945,3531494	-0,055526
Cloud_Mesh_Distance_BA_R0_R2	18,31797409	-76,65541077	-945,3606567	-0,054534
Cloud_Mesh_Distance_BA_R0_R2	18,3455143	-76,57077026	-945,2457275	-0,04601
Cloud_Mesh_Distance_BA_R0_R2	18,46148682	-76,66419983	-945,3218994	-0,048553
Cloud_Mesh_Distance_BA_R0_R2	18,68499756	-76,60974121	-945,1598511	-0,041566
Cloud_Mesh_Distance_BA_R0_R2	18,54236221	-76,5922699	-945,1998291	-0,041274
Cloud_Mesh_Distance_BA_R0_R2	22,70317459	-82,63514709	-944,7919922	-0,112119
Cloud_Mesh_Distance_BA_R0_R2	15,5501976	-81,42552185	-944,7454834	0,315001
Cloud_Mesh_Distance_BA_R0_R2	22,35063171	-82,25617981	-945,0490723	0,13526
Cloud_Mesh_Distance_BA_R0_R2	22,40328979	-82,20062256	-944,9541016	0,135607
Cloud_Mesh_Distance_BA_R0_R2	22,5101738	-82,41687012	-944,9345703	-0,159855
Cloud_Mesh_Distance_BA_R0_R2	22,47674561	-82,18028259	-944,8468018	-0,022621
Cloud_Mesh_Distance_BA_R0_R2	22,27275276	-81,89770508	-944,9032593	-0,024328
Cloud_Mesh_Distance_BA_R0_R2	22,26241493	-81,80697632	-944,8480225	-0,04299
Cloud_Mesh_Distance_BA_R0_R2	22,38662338	-81,90309143	-944,7687988	-0,029602
Cloud_Mesh_Distance_BA_R0_R2	15,549366	-81,04847717	-944,7604981	0,081157
Cloud_Mesh_Distance_BA_R0_R2	15,51756096	-80,84440613	-944,7766724	-0,011089
Cloud_Mesh_Distance_BA_R0_R2	15,58761215	-80,87969971	-944,9470825	0,037317
Cloud_Mesh_Distance_BA_R0_R2	22,08123779	-81,72529602	-944,9854126	-0,056051
Cloud_Mesh_Distance_BA_R0_R2	22,25259018	-81,73069763	-944,8041992	-0,059097
Cloud_Mesh_Distance_BA_R0_R2	22,18190765	-81,79173279	-944,9273071	-0,045316
Cloud_Mesh_Distance_BA_R0_R2	22,32903099	-81,79658508	-944,7614746	-0,048372
Cloud_Mesh_Distance_BA_R0_R2	15,53751183	-80,67488098	-944,9454956	-0,042506
Cloud_Mesh_Distance_BA_R0_R2	21,89454269	-81,48797607	-945,017273	-0,106981
Cloud_Mesh_Distance_BA_R0_R2	22,0881691	-81,54963684	-944,8623657	-0,09979
Cloud_Mesh_Distance_BA_R0_R2	22,08338165	-81,63600159	-944,9208374	-0,080519
Cloud_Mesh_Distance_BA_R0_R2	15,46826649	-80,65522766	-944,803772	-0,065318
Cloud_Mesh_Distance_BA_R0_R2	21,903965	-81,36309814	-944,9447632	-0,131184
Cloud_Mesh_Distance_BA_R0_R2	21,95929337	-81,2875824	-944,8612671	-0,145693
Cloud_Mesh_Distance_BA_R0_R2	15,40319824	-80,51473999	-944,7855835	-0,087378
Cloud_Mesh_Distance_BA_R0_R2	15,44074059	-80,5370636	-944,8484497	-0,083649
Cloud_Mesh_Distance_BA_R0_R2	15,41533089	-80,42852783	-944,9009399	-0,088357
Cloud_Mesh_Distance_BA_R0_R2	21,74588013	-81,229599	-945,0373535	-0,144136
Cloud_Mesh_Distance_BA_R0_R2	21,85748863	-81,22236633	-944,9343872	-0,148066
Cloud_Mesh_Distance_BA_R0_R2	21,75879097	-81,06095886	-944,9801025	-0,152688
Cloud_Mesh_Distance_BA_R0_R2	21,8789978	-81,06098938	-944,8727417	-0,155697
Cloud_Mesh_Distance_BA_R0_R2	15,26377964	-80,1900177	-944,8104248	-0,098761
Cloud_Mesh_Distance_BA_R0_R2	15,20490074	-80,00830078	-944,8353882	-0,091487
Cloud_Mesh_Distance_BA_R0_R2	15,27437973	-79,98834229	-944,998291	-0,092727
Cloud_Mesh_Distance_BA_R0_R2	21,65141678	-80,80775452	-945,0297241	-0,146777
Cloud_Mesh_Distance_BA_R0_R2	21,73708344	-80,71875	-944,9492798	-0,142143
Cloud_Mesh_Distance_BA_R0_R2	21,72179413	-80,88877869	-944,982544	-0,151375
Cloud_Mesh_Distance_BA_R0_R2	15,1971035	-79,87672424	-944,9093628	-0,084983
Cloud_Mesh_Distance_BA_R0_R2	21,56892204	-80,61036682	-945,0693359	-0,134046
Cloud_Mesh_Distance_BA_R0_R2	21,63298035	-80,69166565	-945,0302734	-0,139148
Cloud_Mesh_Distance_BA_R0_R2	21,66311264	-80,54554749	-944,9890747	-0,12904
Cloud_Mesh_Distance_BA_R0_R2	21,77954483	-80,54872131	-944,8947144	-0,131248

Cloud_Mesh_Distance_BA_R0_R2	21,86804581	-80,65460205	-944,8307495	-0,137512
Cloud_Mesh_Distance_BA_R0_R2	15,13208199	-79,76828003	-944,8091431	-0,084711
Cloud_Mesh_Distance_BA_R0_R2	21,63326263	-80,38975525	-944,9889526	-0,119206
Cloud_Mesh_Distance_BA_R0_R2	15,10519695	-79,58491516	-944,8137817	-0,079329
Cloud_Mesh_Distance_BA_R0_R2	21,74051476	-80,39071655	-944,9036865	-0,11975
Cloud_Mesh_Distance_BA_R0_R2	21,6655159	-80,28947449	-944,944519	-0,114074
Cloud_Mesh_Distance_BA_R0_R2	15,09015656	-79,38389587	-944,8197022	-0,065523
Cloud_Mesh_Distance_BA_R0_R2	15,1678257	-79,2288208	-945,0444946	-0,063377
Cloud_Mesh_Distance_BA_R0_R2	21,43592072	-80,11764526	-945,0740356	-0,112849
Cloud_Mesh_Distance_BA_R0_R2	21,56490135	-80,03514099	-944,9539185	-0,113493
Cloud_Mesh_Distance_BA_R0_R2	21,67128754	-80,15513611	-944,906311	-0,112911
Cloud_Mesh_Distance_BA_R0_R2	15,10224533	-79,16508484	-944,8789063	-0,050318
Cloud_Mesh_Distance_BA_R0_R2	15,16152573	-79,01603699	-945,0367432	-0,04519
Cloud_Mesh_Distance_BA_R0_R2	21,27222443	-79,9128418	-945,1278076	-0,117239
Cloud_Mesh_Distance_BA_R0_R2	21,32423401	-80,03858948	-945,1306152	-0,114854
Cloud_Mesh_Distance_BA_R0_R2	21,41821289	-79,90447998	-945,0214233	-0,116536
Cloud_Mesh_Distance_BA_R0_R2	15,10884094	-78,86166382	-944,8736572	-0,03339
Cloud_Mesh_Distance_BA_R0_R2	15,07632065	-78,95539856	-944,7822266	-0,042201
Cloud_Mesh_Distance_BA_R0_R2	15,07905388	-78,8319397	-944,7568359	-0,039072
Cloud_Mesh_Distance_BA_R0_R2	15,10852051	-78,98654175	-944,8920898	-0,039784
Cloud_Mesh_Distance_BA_R0_R2	21,16882706	-79,55886841	-945,0720825	-0,130317
Cloud_Mesh_Distance_BA_R0_R2	21,13362885	-79,70332336	-945,1503906	-0,125823
Cloud_Mesh_Distance_BA_R0_R2	21,31298065	-79,54003906	-944,9560547	-0,126631
Cloud_Mesh_Distance_BA_R0_R2	21,27651596	-79,73602295	-945,0628662	-0,124577
Cloud_Mesh_Distance_BA_R0_R2	21,59945679	-79,79148865	-944,8326416	-0,120417
Cloud_Mesh_Distance_BA_R0_R2	21,43531799	-79,68901062	-944,9234619	-0,122591
Cloud_Mesh_Distance_BA_R0_R2	15,15600967	-78,83427429	-945,0040894	-0,031058
Cloud_Mesh_Distance_BA_R0_R2	21,05119133	-79,35891724	-945,0802002	-0,130878
Cloud_Mesh_Distance_BA_R0_R2	21,27903748	-79,39796448	-944,9205322	-0,129785
Cloud_Mesh_Distance_BA_R0_R2	15,13214111	-78,71873474	-944,9035645	-0,025215
Cloud_Mesh_Distance_BA_R0_R2	15,09862232	-78,64912415	-944,7399292	-0,030769
Cloud_Mesh_Distance_BA_R0_R2	15,15621758	-78,56411743	-944,9057617	-0,013395
Cloud_Mesh_Distance_BA_R0_R2	15,20533371	-78,39877319	-944,9490967	-0,003083
Cloud_Mesh_Distance_BA_R0_R2	15,26274014	-78,38967896	-945,1038208	-0,001236
Cloud_Mesh_Distance_BA_R0_R2	20,87596512	-79,10740662	-945,1174316	-0,129703
Cloud_Mesh_Distance_BA_R0_R2	21,20072556	-79,4335022	-944,9973755	-0,129352
Cloud_Mesh_Distance_BA_R0_R2	15,24544621	-78,19552612	-944,8775024	-0,003829
Cloud_Mesh_Distance_BA_R0_R2	15,27181816	-78,26231384	-945,0353394	0,001408
Cloud_Mesh_Distance_BA_R0_R2	20,95169258	-79,02043152	-945,0261231	-0,11897
Cloud_Mesh_Distance_BA_R0_R2	15,29549599	-78,06311035	-944,8827515	-0,00114
Cloud_Mesh_Distance_BA_R0_R2	15,33049202	-78,09068298	-945,034668	0,002551
Cloud_Mesh_Distance_BA_R0_R2	20,73289108	-78,72567749	-945,0911255	-0,110852
Cloud_Mesh_Distance_BA_R0_R2	21,04309082	-78,79336548	-944,8475342	-0,097152
Cloud_Mesh_Distance_BA_R0_R2	20,96306038	-78,849823	-944,9459229	-0,105198
Cloud_Mesh_Distance_BA_R0_R2	20,87033081	-78,75190735	-944,9857788	-0,098217
Cloud_Mesh_Distance_BA_R0_R2	20,8447876	-78,87872314	-945,0573731	-0,116145
Cloud_Mesh_Distance_BA_R0_R2	21,11351395	-78,95478821	-944,8580933	-0,108772
Cloud_Mesh_Distance_BA_R0_R2	15,34705353	-77,93490601	-944,8740234	-0,006129
Cloud_Mesh_Distance_BA_R0_R2	15,40901184	-78,00024414	-945,1455078	0,006345
Cloud_Mesh_Distance_BA_R0_R2	15,47822952	-77,84399414	-945,1412964	0,00302

Cloud_Mesh_Distance_BA_R0_R2	20,62947083	-78,43911743	-945,069519	-0,088436
Cloud_Mesh_Distance_BA_R0_R2	20,74035263	-78,51872253	-945,0039673	-0,084729
Cloud_Mesh_Distance_BA_R0_R2	20,85329056	-78,60922241	-944,940979	-0,084394
Cloud_Mesh_Distance_BA_R0_R2	15,39527512	-77,93496704	-945,036438	-0,001156
Cloud_Mesh_Distance_BA_R0_R2	15,42411995	-77,82504272	-944,9673462	-0,004261
Cloud_Mesh_Distance_BA_R0_R2	20,51896477	-78,39500427	-945,1480713	-0,094256
Cloud_Mesh_Distance_BA_R0_R2	20,75330353	-78,31532288	-944,8996582	-0,063025
Cloud_Mesh_Distance_BA_R0_R2	15,61452484	-77,5688324	-945,0996094	-0,001805
Cloud_Mesh_Distance_BA_R0_R2	20,50238991	-78,224823	-945,1000977	-0,080204
Cloud_Mesh_Distance_BA_R0_R2	20,58343315	-78,09442139	-944,9628906	-0,055619
Cloud_Mesh_Distance_BA_R0_R2	20,65278244	-78,17802429	-944,9326782	-0,056362
Cloud_Mesh_Distance_BA_R0_R2	20,61231422	-78,30984497	-945,0324097	-0,077621
Cloud_Mesh_Distance_BA_R0_R2	15,685112	-77,39511108	-944,9829712	-0,014114
Cloud_Mesh_Distance_BA_R0_R2	20,32064629	-77,84138489	-945,1021729	-0,056854
Cloud_Mesh_Distance_BA_R0_R2	20,41107559	-77,94212341	-945,0632324	-0,057803
Cloud_Mesh_Distance_BA_R0_R2	20,50097084	-78,0406189	-945,0203857	-0,057261
Cloud_Mesh_Distance_BA_R0_R2	15,89588642	-77,18603516	-945,0546875	-0,029074
Cloud_Mesh_Distance_BA_R0_R2	15,80037308	-77,28720093	-945,0497437	-0,016334
Cloud_Mesh_Distance_BA_R0_R2	20,3336792	-77,73474121	-945,0250855	-0,052817
Cloud_Mesh_Distance_BA_R0_R2	16,10570145	-77,07745361	-945,1876221	-0,060185
Cloud_Mesh_Distance_BA_R0_R2	16,10395432	-77,00628662	-945,0556641	-0,065732
Cloud_Mesh_Distance_BA_R0_R2	16,20159149	-76,97427368	-945,1265259	-0,077217
Cloud_Mesh_Distance_BA_R0_R2	20,06793594	-77,44012451	-945,0866089	-0,069342
Cloud_Mesh_Distance_BA_R0_R2	20,07255936	-77,51733398	-945,1402588	-0,070305
Cloud_Mesh_Distance_BA_R0_R2	20,1298008	-77,46389771	-945,045166	-0,060349
Cloud_Mesh_Distance_BA_R0_R2	20,41498375	-77,77983093	-944,9649048	-0,048728
Cloud_Mesh_Distance_BA_R0_R2	16,15773582	-76,95223999	-945,0266113	-0,074237
Cloud_Mesh_Distance_BA_R0_R2	16,361063	-76,9241333	-945,2142334	-0,102476
Cloud_Mesh_Distance_BA_R0_R2	16,25543976	-76,87486267	-945,001709	-0,092619
Cloud_Mesh_Distance_BA_R0_R2	16,44260406	-76,82333374	-945,1141968	-0,117916
Cloud_Mesh_Distance_BA_R0_R2	19,85211754	-77,22950745	-945,098999	-0,081791
Cloud_Mesh_Distance_BA_R0_R2	19,8616581	-77,32368469	-945,1741943	-0,088824
Cloud_Mesh_Distance_BA_R0_R2	20,16485596	-77,40142822	-944,9545898	-0,05139
Cloud_Mesh_Distance_BA_R0_R2	16,38925171	-76,77633667	-944,9630127	-0,112148
Cloud_Mesh_Distance_BA_R0_R2	16,56729698	-76,7164917	-945,032898	-0,130129
Cloud_Mesh_Distance_BA_R0_R2	16,7612133	-76,73208618	-945,2223511	-0,141215
Cloud_Mesh_Distance_BA_R0_R2	19,62638664	-77,08909607	-945,147583	-0,089759
Cloud_Mesh_Distance_BA_R0_R2	16,83606339	-76,60668945	-945,0621338	-0,14258
Cloud_Mesh_Distance_BA_R0_R2	16,73561096	-76,66700745	-945,0894775	-0,144981
Cloud_Mesh_Distance_BA_R0_R2	16,84596062	-76,66668701	-945,1734009	-0,144526
Cloud_Mesh_Distance_BA_R0_R2	16,98920059	-76,63587952	-945,2176514	-0,1462
Cloud_Mesh_Distance_BA_R0_R2	17,00112724	-76,53935242	-945,0662842	-0,131847
Cloud_Mesh_Distance_BA_R0_R2	18,94262695	-76,66783142	-945,1003418	-0,055117
Cloud_Mesh_Distance_BA_R0_R2	19,15806961	-76,7354126	-945,0580444	-0,068615
Cloud_Mesh_Distance_BA_R0_R2	19,29922867	-76,80679321	-945,0563965	-0,079296
Cloud_Mesh_Distance_BA_R0_R2	19,59155273	-77,01550293	-945,0960693	-0,08962
Cloud_Mesh_Distance_BA_R0_R2	19,48759842	-76,9727478	-945,1265259	-0,088922
Cloud_Mesh_Distance_BA_R0_R2	19,58553886	-76,93774414	-945,0075073	-0,085966
Cloud_Mesh_Distance_BA_R0_R2	17,28054047	-76,35647583	-944,9083862	-0,085418
Cloud_Mesh_Distance_BA_R0_R2	17,17718697	-76,42498779	-944,9766846	-0,10852

Cloud_Mesh_Distance_BA_R0_R2	17,17433739	-76,50610352	-945,1153565	-0,118329
Cloud_Mesh_Distance_BA_R0_R2	17,36061668	-76,48023987	-945,1571655	-0,098913
Cloud_Mesh_Distance_BA_R0_R2	17,72136688	-76,39909363	-945,1054077	-0,060686
Cloud_Mesh_Distance_BA_R0_R2	17,51978111	-76,39561462	-945,0697632	-0,075725
Cloud_Mesh_Distance_BA_R0_R2	17,6187973	-76,48876953	-945,2310791	-0,07759
Cloud_Mesh_Distance_BA_R0_R2	17,90406799	-76,41044617	-945,1201172	-0,050125
Cloud_Mesh_Distance_BA_R0_R2	18,14589119	-76,52323914	-945,2402954	-0,049915
Cloud_Mesh_Distance_BA_R0_R2	18,81975746	-76,6678009	-945,1667481	-0,048521
Cloud_Mesh_Distance_BA_R0_R2	18,77794075	-76,57458496	-945,067749	-0,042003
Cloud_Mesh_Distance_BA_R0_R2	17,3517189	-76,3934021	-945,0106201	-0,091005
Cloud_Mesh_Distance_BA_R0_R2	17,6469593	-76,31600952	-944,9606934	-0,050535
Cloud_Mesh_Distance_BA_R0_R2	18,01258087	-76,35368347	-945,0206299	-0,037589
Cloud_Mesh_Distance_BA_R0_R2	18,08713913	-76,42721558	-945,1186523	-0,041523
Cloud_Mesh_Distance_BA_R0_R2	18,28525543	-76,45747375	-945,1116333	-0,04013
Cloud_Mesh_Distance_BA_R0_R2	18,4765625	-76,50003052	-945,1033325	-0,039412
Cloud_Mesh_Distance_BA_R0_R2	18,6017952	-76,45225525	-944,9841309	-0,037636
Cloud_Mesh_Distance_BA_R0_R2	18,74579811	-76,49452209	-944,9754639	-0,03973
Cloud_Mesh_Distance_BA_R0_R2	18,63998604	-76,5365448	-945,083374	-0,039072
Cloud_Mesh_Distance_BA_R0_R2	23,02908707	-82,83203125	-944,4476929	0,318464
Cloud_Mesh_Distance_BA_R0_R2	22,8972435	-82,67048645	-944,5608521	0,237482
Cloud_Mesh_Distance_BA_R0_R2	22,97202873	-82,6988678	-944,4702148	0,238339
Cloud_Mesh_Distance_BA_R0_R2	15,55897141	-81,56364441	-944,5933228	0,339613
Cloud_Mesh_Distance_BA_R0_R2	15,59275055	-81,68490601	-944,4664307	0,362086
Cloud_Mesh_Distance_BA_R0_R2	22,66085434	-82,45545959	-944,7607422	-0,000114
Cloud_Mesh_Distance_BA_R0_R2	22,77284431	-82,56384277	-944,6702881	0,069729
Cloud_Mesh_Distance_BA_R0_R2	22,75926399	-82,47399902	-944,6390991	0,023158
Cloud_Mesh_Distance_BA_R0_R2	15,55619526	-81,36842346	-944,6270752	0,226246
Cloud_Mesh_Distance_BA_R0_R2	22,5569973	-82,28919983	-944,8067017	-0,001079
Cloud_Mesh_Distance_BA_R0_R2	22,7595787	-82,37121582	-944,5720215	0,030153
Cloud_Mesh_Distance_BA_R0_R2	15,5588398	-81,24568176	-944,7546387	0,192782
Cloud_Mesh_Distance_BA_R0_R2	22,56440353	-82,1525116	-944,7075806	-0,000087
Cloud_Mesh_Distance_BA_R0_R2	22,56619453	-82,06809998	-944,6419067	-0,004425
Cloud_Mesh_Distance_BA_R0_R2	15,51518059	-81,01226807	-944,5858765	0,017101
Cloud_Mesh_Distance_BA_R0_R2	22,40055084	-81,82455444	-944,6918335	-0,042722
Cloud_Mesh_Distance_BA_R0_R2	22,48015594	-82,03367615	-944,7409058	-0,006984
Cloud_Mesh_Distance_BA_R0_R2	22,477705	-81,85324097	-944,6080933	-0,035504
Cloud_Mesh_Distance_BA_R0_R2	15,41961861	-80,63449097	-944,6879273	-0,080383
Cloud_Mesh_Distance_BA_R0_R2	15,3851099	-80,64749146	-944,5617676	-0,093768
Cloud_Mesh_Distance_BA_R0_R2	15,42048454	-80,71800232	-944,5793457	-0,079322
Cloud_Mesh_Distance_BA_R0_R2	22,10121536	-81,46614075	-944,8016357	-0,1166
Cloud_Mesh_Distance_BA_R0_R2	22,17125702	-81,64683533	-944,8355103	-0,08091
Cloud_Mesh_Distance_BA_R0_R2	22,24941444	-81,65708923	-944,7573853	-0,073752
Cloud_Mesh_Distance_BA_R0_R2	15,36219215	-80,52470398	-944,666626	-0,094395
Cloud_Mesh_Distance_BA_R0_R2	15,33583832	-80,55633545	-944,539978	-0,104274
Cloud_Mesh_Distance_BA_R0_R2	15,43330002	-80,58331299	-944,7863159	-0,079544
Cloud_Mesh_Distance_BA_R0_R2	22,01374626	-81,42840576	-944,8696899	-0,122633
Cloud_Mesh_Distance_BA_R0_R2	22,12820053	-81,38117981	-944,7346802	-0,1321
Cloud_Mesh_Distance_BA_R0_R2	22,16848755	-81,50466919	-944,7526856	-0,108874
Cloud_Mesh_Distance_BA_R0_R2	15,34769821	-80,39045715	-944,7927856	-0,095358
Cloud_Mesh_Distance_BA_R0_R2	15,28235817	-80,40556335	-944,6029663	-0,104259

Cloud_Mesh_Distance_BA_R0_R2	15,18556213	-80,23287964	-944,5519409	-0,097918
Cloud_Mesh_Distance_BA_R0_R2	21,9656868	-81,15112305	-944,814209	-0,154917
Cloud_Mesh_Distance_BA_R0_R2	15,15687943	-80,05563354	-944,6685181	-0,093042
Cloud_Mesh_Distance_BA_R0_R2	21,8434906	-80,87895203	-944,8764038	-0,152884
Cloud_Mesh_Distance_BA_R0_R2	21,9697628	-80,97424316	-944,7751465	-0,155751
Cloud_Mesh_Distance_BA_R0_R2	15,15004158	-79,92127991	-944,7634888	-0,088517
Cloud_Mesh_Distance_BA_R0_R2	15,07863808	-79,88008118	-944,5753784	-0,083763
Cloud_Mesh_Distance_BA_R0_R2	21,97284508	-80,77359009	-944,7479248	-0,147097
Cloud_Mesh_Distance_BA_R0_R2	15,06856918	-79,69909668	-944,6547241	-0,07869
Cloud_Mesh_Distance_BA_R0_R2	15,04513931	-79,5479126	-944,6323853	-0,07447
Cloud_Mesh_Distance_BA_R0_R2	21,98488998	-80,57156372	-944,7156982	-0,131532
Cloud_Mesh_Distance_BA_R0_R2	21,99433899	-80,44430542	-944,6893311	-0,121239
Cloud_Mesh_Distance_BA_R0_R2	15,04229546	-79,41319275	-944,6553955	-0,072107
Cloud_Mesh_Distance_BA_R0_R2	21,81805801	-80,28022766	-944,81604	-0,113736
Cloud_Mesh_Distance_BA_R0_R2	21,88988304	-80,42857361	-944,7819824	-0,119477
Cloud_Mesh_Distance_BA_R0_R2	15,05183411	-79,27528381	-944,7102661	-0,061627
Cloud_Mesh_Distance_BA_R0_R2	21,73886871	-80,24742126	-944,8757935	-0,114066
Cloud_Mesh_Distance_BA_R0_R2	21,82768059	-79,92547607	-944,689148	-0,118587
Cloud_Mesh_Distance_BA_R0_R2	21,8055954	-80,11524963	-944,7808838	-0,115224
Cloud_Mesh_Distance_BA_R0_R2	15,06196022	-79,10017395	-944,74823	-0,049669
Cloud_Mesh_Distance_BA_R0_R2	21,70316124	-79,97705078	-944,821167	-0,114872
Cloud_Mesh_Distance_BA_R0_R2	21,84485245	-79,76573181	-944,5985107	-0,122906
Cloud_Mesh_Distance_BA_R0_R2	21,90966415	-79,8553772	-944,578186	-0,121718
Cloud_Mesh_Distance_BA_R0_R2	15,03478813	-79,01295471	-944,6209717	-0,059147
Cloud_Mesh_Distance_BA_R0_R2	21,53604889	-79,62634277	-944,8120728	-0,126158
Cloud_Mesh_Distance_BA_R0_R2	21,74328041	-79,83135986	-944,7258301	-0,120694
Cloud_Mesh_Distance_BA_R0_R2	15,04553604	-78,82249451	-944,5883789	-0,045971
Cloud_Mesh_Distance_BA_R0_R2	21,32496262	-79,28149414	-944,8273315	-0,123738
Cloud_Mesh_Distance_BA_R0_R2	21,3651886	-79,42445374	-944,8618164	-0,12754
Cloud_Mesh_Distance_BA_R0_R2	21,44880867	-79,36933899	-944,7634888	-0,122517
Cloud_Mesh_Distance_BA_R0_R2	21,4570179	-79,52352905	-944,8309326	-0,128643
Cloud_Mesh_Distance_BA_R0_R2	21,54503632	-79,54614258	-944,7661743	-0,126364
Cloud_Mesh_Distance_BA_R0_R2	15,14485931	-78,48320007	-944,8021851	-0,015709
Cloud_Mesh_Distance_BA_R0_R2	21,2129364	-79,14286804	-944,8586426	-0,117602
Cloud_Mesh_Distance_BA_R0_R2	21,19126892	-79,29794312	-944,9473877	-0,128911
Cloud_Mesh_Distance_BA_R0_R2	15,15155315	-78,31698608	-944,5839233	-0,028367
Cloud_Mesh_Distance_BA_R0_R2	15,17267227	-78,34191895	-944,7630615	-0,019839
Cloud_Mesh_Distance_BA_R0_R2	15,10669041	-78,49760437	-944,618103	-0,033124
Cloud_Mesh_Distance_BA_R0_R2	21,24929237	-79,02812195	-944,7695923	-0,110018
Cloud_Mesh_Distance_BA_R0_R2	21,08003616	-79,16072083	-944,9786987	-0,123394
Cloud_Mesh_Distance_BA_R0_R2	21,32313538	-79,02340698	-944,6976929	-0,104119
Cloud_Mesh_Distance_BA_R0_R2	21,32436752	-79,12693787	-944,75177	-0,115257
Cloud_Mesh_Distance_BA_R0_R2	15,20454693	-78,20729065	-944,706543	-0,019019
Cloud_Mesh_Distance_BA_R0_R2	20,9678936	-78,69517517	-944,8723145	-0,087559
Cloud_Mesh_Distance_BA_R0_R2	21,27897835	-78,89096069	-944,6680908	-0,09424
Cloud_Mesh_Distance_BA_R0_R2	15,26447105	-78,05607605	-944,7242432	-0,017546
Cloud_Mesh_Distance_BA_R0_R2	15,33438683	-77,86410522	-944,6329956	-0,029899
Cloud_Mesh_Distance_BA_R0_R2	20,95555305	-78,52888489	-944,8032227	-0,06982
Cloud_Mesh_Distance_BA_R0_R2	21,05289841	-78,61872864	-944,7495728	-0,073715
Cloud_Mesh_Distance_BA_R0_R2	21,04730415	-78,71453857	-944,8048096	-0,086923

Cloud_Mesh_Distance_BA_R0_R2	15,50120163	-77,67649841	-944,9647217	-0,005752
Cloud_Mesh_Distance_BA_R0_R2	20,85415649	-78,44067383	-944,8618774	-0,068644
Cloud_Mesh_Distance_BA_R0_R2	20,58455658	-77,9919281	-944,9059448	-0,045346
Cloud_Mesh_Distance_BA_R0_R2	20,86924362	-78,22750854	-944,7279053	-0,041685
Cloud_Mesh_Distance_BA_R0_R2	20,85906029	-78,33201599	-944,7989502	-0,05399
Cloud_Mesh_Distance_BA_R0_R2	15,58410549	-77,4901886	-944,8762207	-0,011897
Cloud_Mesh_Distance_BA_R0_R2	15,66238213	-77,340271	-944,8007202	-0,0152
Cloud_Mesh_Distance_BA_R0_R2	20,63534164	-77,91104126	-944,7965698	-0,035643
Cloud_Mesh_Distance_BA_R0_R2	20,66275406	-78,03640747	-944,8446655	-0,038753
Cloud_Mesh_Distance_BA_R0_R2	20,7831955	-78,09362793	-944,7426148	-0,037035
Cloud_Mesh_Distance_BA_R0_R2	15,74296188	-77,27183533	-944,8867798	-0,021227
Cloud_Mesh_Distance_BA_R0_R2	15,84906292	-77,14511108	-944,8733521	-0,033823
Cloud_Mesh_Distance_BA_R0_R2	15,81495285	-77,11604309	-944,7104492	-0,037365
Cloud_Mesh_Distance_BA_R0_R2	15,99098778	-77,07806396	-945,0177612	-0,051168
Cloud_Mesh_Distance_BA_R0_R2	20,23295593	-77,50839233	-944,9717407	-0,048791
Cloud_Mesh_Distance_BA_R0_R2	20,51367188	-77,66899109	-944,7648926	-0,028491
Cloud_Mesh_Distance_BA_R0_R2	20,35882187	-77,62239075	-944,9180298	-0,041573
Cloud_Mesh_Distance_BA_R0_R2	15,96341133	-77,02325439	-944,8494873	-0,046242
Cloud_Mesh_Distance_BA_R0_R2	16,08758545	-76,95509338	-944,9262085	-0,067156
Cloud_Mesh_Distance_BA_R0_R2	16,02329636	-76,92800903	-944,739563	-0,059433
Cloud_Mesh_Distance_BA_R0_R2	16,16213989	-76,8445282	-944,7945557	-0,083861
Cloud_Mesh_Distance_BA_R0_R2	16,29122162	-76,78248596	-944,848938	-0,100486
Cloud_Mesh_Distance_BA_R0_R2	20,07806778	-77,3631897	-945,0116577	-0,06142
Cloud_Mesh_Distance_BA_R0_R2	20,14281082	-77,3089447	-944,8895264	-0,053224
Cloud_Mesh_Distance_BA_R0_R2	20,23538589	-77,37615967	-944,8505249	-0,040979
Cloud_Mesh_Distance_BA_R0_R2	16,26180649	-76,7310791	-944,6706543	-0,095347
Cloud_Mesh_Distance_BA_R0_R2	16,67792702	-76,62329102	-944,9553833	-0,135919
Cloud_Mesh_Distance_BA_R0_R2	16,55139923	-76,6468811	-944,8709717	-0,133451
Cloud_Mesh_Distance_BA_R0_R2	19,84567261	-77,14710999	-945,022522	-0,077383
Cloud_Mesh_Distance_BA_R0_R2	19,96922302	-77,25250244	-945,0150757	-0,068984
Cloud_Mesh_Distance_BA_R0_R2	20,0902462	-77,18725586	-944,8126221	-0,059142
Cloud_Mesh_Distance_BA_R0_R2	20,07104301	-77,27534485	-944,9333496	-0,060546
Cloud_Mesh_Distance_BA_R0_R2	20,20894051	-77,27052307	-944,7677612	-0,0481
Cloud_Mesh_Distance_BA_R0_R2	16,78350449	-76,527771	-944,8619385	-0,128891
Cloud_Mesh_Distance_BA_R0_R2	19,27493286	-76,70547485	-944,9354248	-0,074187
Cloud_Mesh_Distance_BA_R0_R2	19,42201233	-76,77120972	-944,9139404	-0,082609
Cloud_Mesh_Distance_BA_R0_R2	19,44780159	-76,87301636	-945,0337524	-0,084513
Cloud_Mesh_Distance_BA_R0_R2	19,72641373	-77,03863525	-945,0089111	-0,086442
Cloud_Mesh_Distance_BA_R0_R2	19,69805336	-76,90823364	-944,862915	-0,086427
Cloud_Mesh_Distance_BA_R0_R2	19,8465271	-77,06790161	-944,9297485	-0,078101
Cloud_Mesh_Distance_BA_R0_R2	19,87001801	-76,98439026	-944,7927856	-0,077841
Cloud_Mesh_Distance_BA_R0_R2	17,17375183	-76,35519409	-944,8376465	-0,094168
Cloud_Mesh_Distance_BA_R0_R2	17,08265305	-76,48297119	-945,0220337	-0,123516
Cloud_Mesh_Distance_BA_R0_R2	16,98789787	-76,4629364	-944,9146118	-0,125147
Cloud_Mesh_Distance_BA_R0_R2	18,86054611	-76,53468323	-944,9700317	-0,044569
Cloud_Mesh_Distance_BA_R0_R2	18,90870476	-76,60682678	-945,0404663	-0,049316
Cloud_Mesh_Distance_BA_R0_R2	19,05268097	-76,65065002	-945,0125122	-0,059025
Cloud_Mesh_Distance_BA_R0_R2	19,17096519	-76,67018127	-944,9607544	-0,067369
Cloud_Mesh_Distance_BA_R0_R2	19,54742432	-76,74928284	-944,7650757	-0,085239
Cloud_Mesh_Distance_BA_R0_R2	19,65664291	-76,81881714	-944,7669067	-0,088482

Cloud_Mesh_Distance_BA_R0_R2	19,56040192	-76,83985901	-944,8944702	-0,085907
Cloud_Mesh_Distance_BA_R0_R2	17,43551636	-76,31758118	-944,9058228	-0,066221
Cloud_Mesh_Distance_BA_R0_R2	17,84728241	-76,3306427	-944,9979858	-0,041338
Cloud_Mesh_Distance_BA_R0_R2	18,21476746	-76,3387146	-944,9537354	-0,025318
Cloud_Mesh_Distance_BA_R0_R2	18,16965866	-76,25515747	-944,8265381	-0,009089
Cloud_Mesh_Distance_BA_R0_R2	18,28418732	-76,26792908	-944,8189087	-0,007663
Cloud_Mesh_Distance_BA_R0_R2	18,4335022	-76,40084839	-944,9786377	-0,029202
Cloud_Mesh_Distance_BA_R0_R2	18,58420181	-76,36611938	-944,8648682	-0,017214
Cloud_Mesh_Distance_BA_R0_R2	18,71175385	-76,420578	-944,8868408	-0,024166
Cloud_Mesh_Distance_BA_R0_R2	18,99377823	-76,54724121	-944,9081421	-0,052209
Cloud_Mesh_Distance_BA_R0_R2	23,09135437	-82,82600403	-944,3511353	0,284698
Cloud_Mesh_Distance_BA_R0_R2	23,14863014	-82,90319824	-944,2921753	0,358411
Cloud_Mesh_Distance_BA_R0_R2	15,66667557	-81,84214783	-944,3309937	0,378645
Cloud_Mesh_Distance_BA_R0_R2	22,93761635	-82,57505798	-944,4501343	0,144256
Cloud_Mesh_Distance_BA_R0_R2	15,59857845	-81,56500244	-944,4248657	0,267317
Cloud_Mesh_Distance_BA_R0_R2	22,84649658	-82,50013733	-944,5332642	0,080055
Cloud_Mesh_Distance_BA_R0_R2	22,9988842	-82,50732422	-944,2942505	0,100863
Cloud_Mesh_Distance_BA_R0_R2	22,93667412	-82,45074463	-944,3546753	0,081961
Cloud_Mesh_Distance_BA_R0_R2	15,54694557	-81,20899963	-944,5968018	0,118289
Cloud_Mesh_Distance_BA_R0_R2	22,75508499	-82,23057556	-944,4701538	0,029632
Cloud_Mesh_Distance_BA_R0_R2	22,66538429	-82,24572754	-944,6237183	0,013694
Cloud_Mesh_Distance_BA_R0_R2	15,54554939	-81,18626404	-944,4122925	0,051142
Cloud_Mesh_Distance_BA_R0_R2	22,5772438	-81,80682373	-944,4182739	-0,038574
Cloud_Mesh_Distance_BA_R0_R2	22,62749672	-82,10572815	-944,5772095	0,002418
Cloud_Mesh_Distance_BA_R0_R2	22,5886879	-81,97758484	-944,5362549	-0,012982
Cloud_Mesh_Distance_BA_R0_R2	22,65478516	-81,90452576	-944,3615723	-0,021454
Cloud_Mesh_Distance_BA_R0_R2	15,43953419	-80,8782196	-944,3053589	-0,077138
Cloud_Mesh_Distance_BA_R0_R2	15,4603672	-80,80488586	-944,6083984	-0,054896
Cloud_Mesh_Distance_BA_R0_R2	22,39922142	-81,73410034	-944,6294556	-0,056994
Cloud_Mesh_Distance_BA_R0_R2	22,37265396	-81,56001282	-944,5527344	-0,088655
Cloud_Mesh_Distance_BA_R0_R2	22,48843575	-81,67919922	-944,4680176	-0,068112
Cloud_Mesh_Distance_BA_R0_R2	15,42006207	-80,78271484	-944,4504395	-0,083973
Cloud_Mesh_Distance_BA_R0_R2	22,31189728	-81,67756653	-944,6980591	-0,069705
Cloud_Mesh_Distance_BA_R0_R2	22,25744247	-81,57496643	-944,6966553	-0,090102
Cloud_Mesh_Distance_BA_R0_R2	22,38864517	-81,35977173	-944,4362793	-0,116208
Cloud_Mesh_Distance_BA_R0_R2	15,35788727	-80,66197205	-944,4265747	-0,097522
Cloud_Mesh_Distance_BA_R0_R2	15,29253197	-80,53620911	-944,4190674	-0,102837
Cloud_Mesh_Distance_BA_R0_R2	15,21538353	-80,3921051	-944,3999634	-0,098547
Cloud_Mesh_Distance_BA_R0_R2	22,17017937	-81,22085571	-944,6347656	-0,142652
Cloud_Mesh_Distance_BA_R0_R2	22,25635147	-81,3999176	-944,6077271	-0,12184
Cloud_Mesh_Distance_BA_R0_R2	22,0519104	-81,26109314	-944,7640381	-0,145488
Cloud_Mesh_Distance_BA_R0_R2	22,37386894	-81,07322693	-944,3737793	-0,135916
Cloud_Mesh_Distance_BA_R0_R2	22,30534363	-81,18310547	-944,4804077	-0,135956
Cloud_Mesh_Distance_BA_R0_R2	15,04979134	-79,94276428	-944,3987427	-0,086025
Cloud_Mesh_Distance_BA_R0_R2	15,09767532	-80,07835388	-944,4423828	-0,090326
Cloud_Mesh_Distance_BA_R0_R2	22,08362961	-80,8813324	-944,6556397	-0,149135
Cloud_Mesh_Distance_BA_R0_R2	22,19219208	-80,77182007	-944,5345459	-0,139643
Cloud_Mesh_Distance_BA_R0_R2	22,07426834	-81,08421326	-944,6959229	-0,153441
Cloud_Mesh_Distance_BA_R0_R2	22,20487404	-81,0025177	-944,5517578	-0,146001
Cloud_Mesh_Distance_BA_R0_R2	15,02980042	-79,84159851	-944,4039307	-0,085067

Cloud_Mesh_Distance_BA_R0_R2	22,08640099	-80,67202759	-944,6305542	-0,136565
Cloud_Mesh_Distance_BA_R0_R2	15,00244713	-79,57814026	-944,4299316	-0,081011
Cloud_Mesh_Distance_BA_R0_R2	14,9991951	-79,47595215	-944,4468384	-0,076068
Cloud_Mesh_Distance_BA_R0_R2	22,18506622	-80,57745361	-944,5192871	-0,130372
Cloud_Mesh_Distance_BA_R0_R2	22,18324471	-80,46159363	-944,5026245	-0,126425
Cloud_Mesh_Distance_BA_R0_R2	15,02100849	-79,47627258	-944,5557251	-0,076415
Cloud_Mesh_Distance_BA_R0_R2	15,01383972	-79,55784607	-944,4963989	-0,081725
Cloud_Mesh_Distance_BA_R0_R2	21,9198494	-80,21507263	-944,7077637	-0,114185
Cloud_Mesh_Distance_BA_R0_R2	22,00990677	-80,32214355	-944,6495361	-0,117654
Cloud_Mesh_Distance_BA_R0_R2	22,0965786	-80,45509338	-944,5917969	-0,122178
Cloud_Mesh_Distance_BA_R0_R2	15,01904488	-79,36743164	-944,5668335	-0,07692
Cloud_Mesh_Distance_BA_R0_R2	14,99861526	-79,33555603	-944,4628906	-0,073572
Cloud_Mesh_Distance_BA_R0_R2	15,02214527	-79,21406555	-944,590332	-0,067845
Cloud_Mesh_Distance_BA_R0_R2	21,95433044	-80,00080872	-944,5994873	-0,117519
Cloud_Mesh_Distance_BA_R0_R2	22,03509521	-80,14378357	-944,5744019	-0,116868
Cloud_Mesh_Distance_BA_R0_R2	21,92058754	-79,77880859	-944,5292969	-0,124134
Cloud_Mesh_Distance_BA_R0_R2	22,05135155	-80,02966309	-944,5125732	-0,115312
Cloud_Mesh_Distance_BA_R0_R2	22,07016945	-79,92419434	-944,444397	-0,118674
Cloud_Mesh_Distance_BA_R0_R2	15,00609779	-78,99559021	-944,4227905	-0,063837
Cloud_Mesh_Distance_BA_R0_R2	21,73605537	-79,68823242	-944,6640015	-0,122445
Cloud_Mesh_Distance_BA_R0_R2	21,73948669	-79,57989502	-944,6040649	-0,122429
Cloud_Mesh_Distance_BA_R0_R2	21,99943542	-79,80027771	-944,4577637	-0,124631
Cloud_Mesh_Distance_BA_R0_R2	21,92733955	-79,68891907	-944,4737549	-0,125487
Cloud_Mesh_Distance_BA_R0_R2	15,02645206	-78,80627441	-944,4089356	-0,06053
Cloud_Mesh_Distance_BA_R0_R2	15,06240845	-78,67350769	-944,5585938	-0,041868
Cloud_Mesh_Distance_BA_R0_R2	21,54589844	-79,45619202	-944,7208252	-0,123118
Cloud_Mesh_Distance_BA_R0_R2	21,54145622	-79,32139587	-944,6547241	-0,120497
Cloud_Mesh_Distance_BA_R0_R2	21,63687706	-79,57106018	-944,6964722	-0,124239
Cloud_Mesh_Distance_BA_R0_R2	15,10083389	-78,4498291	-944,4204102	-0,053055
Cloud_Mesh_Distance_BA_R0_R2	21,54225922	-79,23599243	-944,6071777	-0,113231
Cloud_Mesh_Distance_BA_R0_R2	21,63526917	-79,40055847	-944,609436	-0,1213
Cloud_Mesh_Distance_BA_R0_R2	15,14853668	-78,28993225	-944,4035034	-0,049634
Cloud_Mesh_Distance_BA_R0_R2	21,38857651	-79,08058167	-944,6674805	-0,106258
Cloud_Mesh_Distance_BA_R0_R2	21,45056534	-79,2035675	-944,6761475	-0,114986
Cloud_Mesh_Distance_BA_R0_R2	21,55619431	-79,15779114	-944,5493774	-0,105716
Cloud_Mesh_Distance_BA_R0_R2	21,34697914	-78,76191711	-944,519043	-0,076899
Cloud_Mesh_Distance_BA_R0_R2	21,37997437	-78,9916687	-944,6260376	-0,098411
Cloud_Mesh_Distance_BA_R0_R2	15,30298424	-77,89668274	-944,4223023	-0,048419
Cloud_Mesh_Distance_BA_R0_R2	21,04653168	-78,46835327	-944,6709595	-0,058498
Cloud_Mesh_Distance_BA_R0_R2	21,17001724	-78,56185913	-944,5877075	-0,064142
Cloud_Mesh_Distance_BA_R0_R2	21,1345768	-78,74972534	-944,7358398	-0,083929
Cloud_Mesh_Distance_BA_R0_R2	15,41447449	-77,75796509	-944,8021851	-0,011892
Cloud_Mesh_Distance_BA_R0_R2	15,37640476	-77,73872375	-944,4440918	-0,046572
Cloud_Mesh_Distance_BA_R0_R2	15,41117764	-77,70404053	-944,6327515	-0,030018
Cloud_Mesh_Distance_BA_R0_R2	20,95625687	-78,34970093	-944,7023926	-0,045211
Cloud_Mesh_Distance_BA_R0_R2	15,44797897	-77,61109924	-944,5440674	-0,037157
Cloud_Mesh_Distance_BA_R0_R2	15,51622963	-77,48231506	-944,5249634	-0,039536
Cloud_Mesh_Distance_BA_R0_R2	15,49026012	-77,5834198	-944,720459	-0,019476
Cloud_Mesh_Distance_BA_R0_R2	21,03749275	-78,37211609	-944,6217651	-0,046993
Cloud_Mesh_Distance_BA_R0_R2	15,58438492	-77,41044617	-944,6784668	-0,018926

Cloud_Mesh_Distance_BA_R0_R2	20,52762985	-77,87492371	-944,8978882	-0,044366
Cloud_Mesh_Distance_BA_R0_R2	20,602808	-77,7960968	-944,7532959	-0,026623
Cloud_Mesh_Distance_BA_R0_R2	20,69689178	-77,8182373	-944,6495972	-0,022009
Cloud_Mesh_Distance_BA_R0_R2	20,72000885	-77,95144653	-944,7214356	-0,030775
Cloud_Mesh_Distance_BA_R0_R2	20,87802887	-78,12690735	-944,6505127	-0,0307
Cloud_Mesh_Distance_BA_R0_R2	15,65828991	-77,29164124	-944,6530151	-0,02128
Cloud_Mesh_Distance_BA_R0_R2	15,69911671	-77,21299744	-944,5758667	-0,0314
Cloud_Mesh_Distance_BA_R0_R2	15,78760529	-77,09771729	-944,5510864	-0,034677
Cloud_Mesh_Distance_BA_R0_R2	15,74100685	-77,20675659	-944,7225342	-0,027441
Cloud_Mesh_Distance_BA_R0_R2	20,62719917	-77,71128845	-944,6513672	-0,020746
Cloud_Mesh_Distance_BA_R0_R2	20,70538902	-77,6855011	-944,5162354	-0,01627
Cloud_Mesh_Distance_BA_R0_R2	15,89541626	-77,02566528	-944,6912231	-0,047029
Cloud_Mesh_Distance_BA_R0_R2	20,42511749	-77,52693176	-944,7550049	-0,032735
Cloud_Mesh_Distance_BA_R0_R2	20,5068264	-77,47012329	-944,5836792	-0,020518
Cloud_Mesh_Distance_BA_R0_R2	20,60644722	-77,59440613	-944,5690308	-0,022292
Cloud_Mesh_Distance_BA_R0_R2	15,89173412	-76,97698975	-944,5174561	-0,046521
Cloud_Mesh_Distance_BA_R0_R2	16,09367943	-76,82048035	-944,5858765	-0,071996
Cloud_Mesh_Distance_BA_R0_R2	20,31827354	-77,32931519	-944,6928101	-0,036076
Cloud_Mesh_Distance_BA_R0_R2	20,40089798	-77,42645264	-944,6871338	-0,031392
Cloud_Mesh_Distance_BA_R0_R2	20,3314476	-77,46130371	-944,8139038	-0,034839
Cloud_Mesh_Distance_BA_R0_R2	20,41502571	-77,35903931	-944,5908814	-0,028578
Cloud_Mesh_Distance_BA_R0_R2	16,41547394	-76,69897461	-944,8239136	-0,116667
Cloud_Mesh_Distance_BA_R0_R2	16,38891983	-76,6502533	-944,6615601	-0,11484
Cloud_Mesh_Distance_BA_R0_R2	16,32906914	-76,63877869	-944,5155029	-0,103356
Cloud_Mesh_Distance_BA_R0_R2	20,00854111	-77,0660553	-944,7498779	-0,070395
Cloud_Mesh_Distance_BA_R0_R2	20,24568748	-77,17242432	-944,5901489	-0,041343
Cloud_Mesh_Distance_BA_R0_R2	16,46422386	-76,57820129	-944,5778198	-0,118022
Cloud_Mesh_Distance_BA_R0_R2	16,50162125	-76,61558533	-944,7363892	-0,121949
Cloud_Mesh_Distance_BA_R0_R2	16,61876297	-76,58940125	-944,8199463	-0,130456
Cloud_Mesh_Distance_BA_R0_R2	16,71710205	-76,46760559	-944,6433106	-0,125738
Cloud_Mesh_Distance_BA_R0_R2	19,94074631	-77,11299133	-944,8894043	-0,067442
Cloud_Mesh_Distance_BA_R0_R2	20,10758972	-77,08268738	-944,6483154	-0,056114
Cloud_Mesh_Distance_BA_R0_R2	16,87084198	-76,43148804	-944,7376709	-0,117786
Cloud_Mesh_Distance_BA_R0_R2	16,93375206	-76,35202026	-944,6052856	-0,105328
Cloud_Mesh_Distance_BA_R0_R2	17,0373764	-76,38778687	-944,8001099	-0,109241
Cloud_Mesh_Distance_BA_R0_R2	19,19894028	-76,59013367	-944,824585	-0,064987
Cloud_Mesh_Distance_BA_R0_R2	19,06056595	-76,45903015	-944,7318726	-0,03497
Cloud_Mesh_Distance_BA_R0_R2	19,39816856	-76,6701355	-944,7801514	-0,079948
Cloud_Mesh_Distance_BA_R0_R2	19,69278717	-76,753479	-944,6113892	-0,08666
Cloud_Mesh_Distance_BA_R0_R2	17,11029434	-76,31672668	-944,7003174	-0,090374
Cloud_Mesh_Distance_BA_R0_R2	17,20399284	-76,25292969	-944,6212158	-0,068424
Cloud_Mesh_Distance_BA_R0_R2	17,28205872	-76,29309082	-944,7781982	-0,075485
Cloud_Mesh_Distance_BA_R0_R2	17,41604614	-76,26052856	-944,7816773	-0,056341
Cloud_Mesh_Distance_BA_R0_R2	17,48587418	-76,14845276	-944,5480347	-0,020148
Cloud_Mesh_Distance_BA_R0_R2	17,5405159	-76,17262268	-944,638794	-0,024965
Cloud_Mesh_Distance_BA_R0_R2	17,64791298	-76,16442871	-944,6588135	-0,014956
Cloud_Mesh_Distance_BA_R0_R2	17,57634926	-76,22860718	-944,7768555	-0,038523
Cloud_Mesh_Distance_BA_R0_R2	18,00925827	-76,27084351	-944,8829956	-0,020566
Cloud_Mesh_Distance_BA_R0_R2	17,79537582	-76,24905396	-944,8538818	-0,026797
Cloud_Mesh_Distance_BA_R0_R2	17,93183899	-76,19358826	-944,75	-0,008005

Cloud_Mesh_Distance_BA_R0_R2	18,41930008	-76,30401611	-944,8334351	-0,0121
Cloud_Mesh_Distance_BA_R0_R2	18,57221222	-76,28430176	-944,7402954	-0,001496
Cloud_Mesh_Distance_BA_R0_R2	18,74123001	-76,34207153	-944,75177	-0,004875
Cloud_Mesh_Distance_BA_R0_R2	18,92368126	-76,36553955	-944,6810303	-0,010634
Cloud_Mesh_Distance_BA_R0_R2	18,86639023	-76,45040894	-944,8463745	-0,030015
Cloud_Mesh_Distance_BA_R0_R2	18,08731651	-76,19789124	-944,7401123	-0,003693
Cloud_Mesh_Distance_BA_R0_R2	18,24066925	-76,20631409	-944,7232056	0,002076
Cloud_Mesh_Distance_BA_R0_R2	18,38129997	-76,17446899	-944,6225586	0,017808
Cloud_Mesh_Distance_BA_R0_R2	18,14261055	-76,11555481	-944,5688477	0,026305
Cloud_Mesh_Distance_BA_R0_R2	18,45717049	-76,24325562	-944,718689	0,002062
Cloud_Mesh_Distance_BA_R0_R2	18,36038971	-76,23857117	-944,7449951	-0,000822
Cloud_Mesh_Distance_BA_R0_R2	19,04524612	-76,37754822	-944,6133423	-0,019232
Cloud_Mesh_Distance_BA_R0_R2	19,13810921	-76,41424561	-944,5982056	-0,031659
Cloud_Mesh_Distance_BA_R0_R2	15,76668358	-81,96160889	-944,2042847	0,357871
Cloud_Mesh_Distance_BA_R0_R2	15,86160088	-82,02218628	-944,0935669	0,309043
Cloud_Mesh_Distance_BA_R0_R2	15,79853058	-81,86540222	-944,0997925	0,257194
Cloud_Mesh_Distance_BA_R0_R2	15,72571468	-81,8212738	-944,2128906	0,297435
Cloud_Mesh_Distance_BA_R0_R2	15,65334511	-81,67698669	-944,3078003	0,272075
Cloud_Mesh_Distance_BA_R0_R2	23,06243515	-82,69529724	-944,3251343	0,170911
Cloud_Mesh_Distance_BA_R0_R2	15,61887932	-81,49345398	-944,3145142	0,178173
Cloud_Mesh_Distance_BA_R0_R2	15,57475281	-81,39112854	-944,4591065	0,179551
Cloud_Mesh_Distance_BA_R0_R2	22,84273529	-82,32209778	-944,4004517	0,048428
Cloud_Mesh_Distance_BA_R0_R2	22,99934006	-82,5841217	-944,3591919	0,118811
Cloud_Mesh_Distance_BA_R0_R2	23,05297661	-82,54460144	-944,2196045	0,117465
Cloud_Mesh_Distance_BA_R0_R2	22,99815559	-82,39543152	-944,1724854	0,09694
Cloud_Mesh_Distance_BA_R0_R2	15,5970192	-81,3417511	-944,2711792	0,083815
Cloud_Mesh_Distance_BA_R0_R2	22,80045128	-82,18495178	-944,3468628	0,032588
Cloud_Mesh_Distance_BA_R0_R2	22,85737228	-82,16880798	-944,2125855	0,03946
Cloud_Mesh_Distance_BA_R0_R2	15,50874519	-81,04031372	-944,1008301	-0,061625
Cloud_Mesh_Distance_BA_R0_R2	15,55762386	-81,17892456	-944,2174072	-0,004578
Cloud_Mesh_Distance_BA_R0_R2	15,61814594	-81,31100464	-944,1188355	0,023432
Cloud_Mesh_Distance_BA_R0_R2	15,58996964	-81,20854187	-944,0529785	-0,024567
Cloud_Mesh_Distance_BA_R0_R2	22,71767044	-81,86448669	-944,1989136	-0,023721
Cloud_Mesh_Distance_BA_R0_R2	22,71673393	-82,07014465	-944,3950806	0,004649
Cloud_Mesh_Distance_BA_R0_R2	22,79980659	-82,01263428	-944,1658325	0,004386
Cloud_Mesh_Distance_BA_R0_R2	22,79485893	-82,10993958	-944,2800903	0,021132
Cloud_Mesh_Distance_BA_R0_R2	15,46764278	-80,94908142	-944,2369995	-0,070213
Cloud_Mesh_Distance_BA_R0_R2	15,43513489	-80,88912964	-944,1790161	-0,087153
Cloud_Mesh_Distance_BA_R0_R2	15,48592377	-80,98130798	-944,3638916	-0,040163
Cloud_Mesh_Distance_BA_R0_R2	22,64575386	-81,78636169	-944,2800903	-0,046664
Cloud_Mesh_Distance_BA_R0_R2	15,39099121	-80,77746582	-944,2977905	-0,091291
Cloud_Mesh_Distance_BA_R0_R2	15,32600403	-80,65861511	-944,2739868	-0,09943
Cloud_Mesh_Distance_BA_R0_R2	15,37293243	-80,77677917	-944,1400147	-0,100002
Cloud_Mesh_Distance_BA_R0_R2	22,59491348	-81,62895203	-944,262146	-0,075688
Cloud_Mesh_Distance_BA_R0_R2	22,4916687	-81,47975159	-944,3476563	-0,096992
Cloud_Mesh_Distance_BA_R0_R2	22,47055244	-81,32295227	-944,3133545	-0,11408
Cloud_Mesh_Distance_BA_R0_R2	22,56733322	-81,472229	-944,2219849	-0,095805
Cloud_Mesh_Distance_BA_R0_R2	15,1165638	-80,25663757	-944,2019653	-0,095134
Cloud_Mesh_Distance_BA_R0_R2	15,12549019	-80,30258179	-944,109375	-0,091849
Cloud_Mesh_Distance_BA_R0_R2	15,13921833	-80,25117493	-944,3458862	-0,098462

Cloud_Mesh_Distance_BA_R0_R2	15,16946602	-80,37789917	-944,2017822	-0,100426
Cloud_Mesh_Distance_BA_R0_R2	22,31202698	-81,01051331	-944,4362183	-0,138113
Cloud_Mesh_Distance_BA_R0_R2	22,50165939	-81,14820862	-944,2160645	-0,127376
Cloud_Mesh_Distance_BA_R0_R2	22,42592621	-81,19787598	-944,3355103	-0,129038
Cloud_Mesh_Distance_BA_R0_R2	22,48784637	-81,24812317	-944,2633667	-0,121064
Cloud_Mesh_Distance_BA_R0_R2	15,08163452	-80,14320374	-944,2495728	-0,092081
Cloud_Mesh_Distance_BA_R0_R2	15,0452776	-80,00700378	-944,2789307	-0,088172
Cloud_Mesh_Distance_BA_R0_R2	22,29476547	-80,8531189	-944,4329834	-0,138244
Cloud_Mesh_Distance_BA_R0_R2	22,37800407	-80,93135071	-944,3447266	-0,135565
Cloud_Mesh_Distance_BA_R0_R2	15,01934624	-79,88356018	-944,2927856	-0,084646
Cloud_Mesh_Distance_BA_R0_R2	22,29354858	-80,6427002	-944,4081421	-0,129814
Cloud_Mesh_Distance_BA_R0_R2	22,38612938	-80,60350037	-944,2901611	-0,128838
Cloud_Mesh_Distance_BA_R0_R2	22,38258743	-80,75517273	-944,3157959	-0,133101
Cloud_Mesh_Distance_BA_R0_R2	15,00739384	-79,78736877	-944,3226929	-0,081921
Cloud_Mesh_Distance_BA_R0_R2	14,99525642	-79,72111511	-944,2937622	-0,080337
Cloud_Mesh_Distance_BA_R0_R2	15,01487541	-79,68916321	-944,4435425	-0,08332
Cloud_Mesh_Distance_BA_R0_R2	14,99035645	-79,57206726	-944,3550415	-0,079795
Cloud_Mesh_Distance_BA_R0_R2	14,99020767	-79,65402222	-944,3067627	-0,080354
Cloud_Mesh_Distance_BA_R0_R2	22,25464439	-80,46762085	-944,4256592	-0,123993
Cloud_Mesh_Distance_BA_R0_R2	22,20009422	-80,35198975	-944,4614868	-0,121
Cloud_Mesh_Distance_BA_R0_R2	22,12109947	-80,24960327	-944,5194702	-0,117447
Cloud_Mesh_Distance_BA_R0_R2	22,3482399	-80,47900391	-944,3162231	-0,123654
Cloud_Mesh_Distance_BA_R0_R2	14,9965992	-79,19294739	-944,4381104	-0,068662
Cloud_Mesh_Distance_BA_R0_R2	22,22623062	-80,18579102	-944,3809815	-0,122495
Cloud_Mesh_Distance_BA_R0_R2	22,32657242	-80,09976196	-944,2194214	-0,127635
Cloud_Mesh_Distance_BA_R0_R2	14,99837208	-78,97615051	-944,2292481	-0,08606
Cloud_Mesh_Distance_BA_R0_R2	22,08534241	-79,82313538	-944,3740845	-0,127204
Cloud_Mesh_Distance_BA_R0_R2	22,2474556	-79,97538757	-944,2608032	-0,129267
Cloud_Mesh_Distance_BA_R0_R2	22,14773941	-80,05128479	-944,4179077	-0,118236
Cloud_Mesh_Distance_BA_R0_R2	22,23729897	-80,07522583	-944,3222046	-0,125201
Cloud_Mesh_Distance_BA_R0_R2	21,83929062	-79,58378601	-944,5078125	-0,123757
Cloud_Mesh_Distance_BA_R0_R2	21,93129158	-79,59434509	-944,4154053	-0,123406
Cloud_Mesh_Distance_BA_R0_R2	22,09501076	-79,72459412	-944,3059082	-0,128092
Cloud_Mesh_Distance_BA_R0_R2	15,02691841	-78,77830505	-944,2266846	-0,083653
Cloud_Mesh_Distance_BA_R0_R2	15,05865574	-78,62612915	-944,4147949	-0,059299
Cloud_Mesh_Distance_BA_R0_R2	21,71813965	-79,3193512	-944,4807739	-0,110799
Cloud_Mesh_Distance_BA_R0_R2	21,73805618	-79,47291565	-944,5481567	-0,123358
Cloud_Mesh_Distance_BA_R0_R2	21,83897018	-79,38615417	-944,392395	-0,113401
Cloud_Mesh_Distance_BA_R0_R2	21,61426353	-79,2648468	-944,5544434	-0,110513
Cloud_Mesh_Distance_BA_R0_R2	21,78480721	-79,24020386	-944,3623047	-0,102675
Cloud_Mesh_Distance_BA_R0_R2	21,78007317	-79,11253357	-944,28302	-0,092961
Cloud_Mesh_Distance_BA_R0_R2	21,67931366	-79,03126526	-944,3405762	-0,089938
Cloud_Mesh_Distance_BA_R0_R2	21,67593384	-79,16236877	-944,4296875	-0,100157
Cloud_Mesh_Distance_BA_R0_R2	15,20012951	-78,16567993	-944,5526733	-0,028673
Cloud_Mesh_Distance_BA_R0_R2	15,1965332	-78,15356445	-944,3660889	-0,054283
Cloud_Mesh_Distance_BA_R0_R2	21,24274635	-78,71887207	-944,6044922	-0,072553
Cloud_Mesh_Distance_BA_R0_R2	21,28957748	-78,63572693	-944,49823	-0,06579
Cloud_Mesh_Distance_BA_R0_R2	21,37223053	-78,67266846	-944,4282837	-0,069766
Cloud_Mesh_Distance_BA_R0_R2	21,45700836	-78,75497437	-944,3909302	-0,072345
Cloud_Mesh_Distance_BA_R0_R2	21,44558716	-78,90774536	-944,508667	-0,087885

Cloud_Mesh_Distance_BA_R0_R2	21,46675301	-79,06034851	-944,5800781	-0,100241
Cloud_Mesh_Distance_BA_R0_R2	21,57446289	-79,03039551	-944,4526978	-0,095079
Cloud_Mesh_Distance_BA_R0_R2	21,74336815	-78,98680115	-944,2349854	-0,076426
Cloud_Mesh_Distance_BA_R0_R2	15,25362015	-78,02009583	-944,4992065	-0,039972
Cloud_Mesh_Distance_BA_R0_R2	15,24464035	-78,03549194	-944,2698364	-0,073104
Cloud_Mesh_Distance_BA_R0_R2	21,26662064	-78,5296936	-944,449585	-0,05983
Cloud_Mesh_Distance_BA_R0_R2	21,04133415	-78,28823853	-944,5601196	-0,039408
Cloud_Mesh_Distance_BA_R0_R2	21,05327415	-78,20480347	-944,4813843	-0,029801
Cloud_Mesh_Distance_BA_R0_R2	21,10606766	-78,39059448	-944,5504761	-0,046121
Cloud_Mesh_Distance_BA_R0_R2	21,12064362	-78,266922	-944,4387817	-0,031668
Cloud_Mesh_Distance_BA_R0_R2	21,21779823	-78,40156555	-944,4158325	-0,042913
Cloud_Mesh_Distance_BA_R0_R2	15,52430248	-77,43962097	-944,3330078	-0,04578
Cloud_Mesh_Distance_BA_R0_R2	15,58980846	-77,32316589	-944,2736816	-0,048297
Cloud_Mesh_Distance_BA_R0_R2	20,80720139	-77,90357971	-944,5717773	-0,018607
Cloud_Mesh_Distance_BA_R0_R2	20,88376236	-78,03164673	-944,5722656	-0,019928
Cloud_Mesh_Distance_BA_R0_R2	20,96887016	-78,16142273	-944,5606079	-0,029453
Cloud_Mesh_Distance_BA_R0_R2	15,61500168	-77,31037903	-944,4788208	-0,038091
Cloud_Mesh_Distance_BA_R0_R2	15,67241859	-77,20005798	-944,3083496	-0,047132
Cloud_Mesh_Distance_BA_R0_R2	20,75971985	-77,77905273	-944,5269775	-0,014593
Cloud_Mesh_Distance_BA_R0_R2	20,81440926	-77,77503967	-944,4424439	-0,014058
Cloud_Mesh_Distance_BA_R0_R2	20,88605881	-77,94114685	-944,4938355	-0,014648
Cloud_Mesh_Distance_BA_R0_R2	15,70308876	-77,18104553	-944,4626465	-0,038893
Cloud_Mesh_Distance_BA_R0_R2	15,83918095	-76,99533081	-944,3284302	-0,051372
Cloud_Mesh_Distance_BA_R0_R2	20,59705734	-77,50035095	-944,4807739	-0,016669
Cloud_Mesh_Distance_BA_R0_R2	20,67405701	-77,54402161	-944,4107056	-0,014303
Cloud_Mesh_Distance_BA_R0_R2	20,76786995	-77,72016907	-944,4564819	-0,01413
Cloud_Mesh_Distance_BA_R0_R2	15,97328568	-76,92063904	-944,5913086	-0,06163
Cloud_Mesh_Distance_BA_R0_R2	15,98333931	-76,86878967	-944,4185791	-0,062452
Cloud_Mesh_Distance_BA_R0_R2	16,09753799	-76,77307129	-944,4160767	-0,076395
Cloud_Mesh_Distance_BA_R0_R2	16,16127396	-76,70808411	-944,3312378	-0,087917
Cloud_Mesh_Distance_BA_R0_R2	16,19833565	-76,71279907	-944,4737549	-0,087432
Cloud_Mesh_Distance_BA_R0_R2	20,15698624	-77,02427673	-944,4832764	-0,056339
Cloud_Mesh_Distance_BA_R0_R2	20,37006378	-77,26950073	-944,5439453	-0,033429
Cloud_Mesh_Distance_BA_R0_R2	20,28849792	-77,07115173	-944,3624878	-0,041005
Cloud_Mesh_Distance_BA_R0_R2	20,47107697	-77,31507874	-944,4494629	-0,023736
Cloud_Mesh_Distance_BA_R0_R2	20,38842392	-77,18826294	-944,3970337	-0,028302
Cloud_Mesh_Distance_BA_R0_R2	16,41859436	-76,55314636	-944,3979492	-0,109362
Cloud_Mesh_Distance_BA_R0_R2	16,70374489	-76,42686462	-944,5045776	-0,108572
Cloud_Mesh_Distance_BA_R0_R2	16,60441971	-76,54107666	-944,6854248	-0,123131
Cloud_Mesh_Distance_BA_R0_R2	16,79792786	-76,40811157	-944,5880737	-0,110756
Cloud_Mesh_Distance_BA_R0_R2	19,77775574	-76,85656738	-944,694458	-0,082488
Cloud_Mesh_Distance_BA_R0_R2	19,91214371	-76,88626099	-944,578125	-0,0766
Cloud_Mesh_Distance_BA_R0_R2	20,02143478	-76,98405457	-944,6051636	-0,069323
Cloud_Mesh_Distance_BA_R0_R2	16,79019547	-76,36837769	-944,4512329	-0,095618
Cloud_Mesh_Distance_BA_R0_R2	16,91127586	-76,30157471	-944,4127808	-0,08487
Cloud_Mesh_Distance_BA_R0_R2	17,06008148	-76,27290344	-944,5270386	-0,080635
Cloud_Mesh_Distance_BA_R0_R2	19,33480263	-76,47395325	-944,5103149	-0,05533
Cloud_Mesh_Distance_BA_R0_R2	19,55569649	-76,57139587	-944,4299927	-0,081839
Cloud_Mesh_Distance_BA_R0_R2	19,38845062	-76,56163025	-944,6082153	-0,070556
Cloud_Mesh_Distance_BA_R0_R2	19,54468727	-76,65953064	-944,6126709	-0,081634

Cloud_Mesh_Distance_BA_R0_R2	19,8231945	-76,80831909	-944,5492554	-0,086809
Cloud_Mesh_Distance_BA_R0_R2	17,20881081	-76,18876648	-944,4278565	-0,050874
Cloud_Mesh_Distance_BA_R0_R2	17,38345718	-76,20713806	-944,6395264	-0,043609
Cloud_Mesh_Distance_BA_R0_R2	17,38670158	-76,14962769	-944,4800415	-0,02946
Cloud_Mesh_Distance_BA_R0_R2	17,76672554	-76,17852783	-944,7131348	-0,014688
Cloud_Mesh_Distance_BA_R0_R2	17,90994835	-76,12400818	-944,6061401	0,010955
Cloud_Mesh_Distance_BA_R0_R2	18,01921654	-76,15660095	-944,6690064	0,006827
Cloud_Mesh_Distance_BA_R0_R2	19,22885895	-76,48124695	-944,6283569	-0,04764
Cloud_Mesh_Distance_BA_R0_R2	17,51248169	-76,09906006	-944,4171143	-0,006111
Cloud_Mesh_Distance_BA_R0_R2	17,61930466	-76,12013245	-944,5394898	-0,003654
Cloud_Mesh_Distance_BA_R0_R2	17,63729095	-76,07899475	-944,4302979	0,008275
Cloud_Mesh_Distance_BA_R0_R2	17,74401474	-76,1282196	-944,5988159	0,002726
Cloud_Mesh_Distance_BA_R0_R2	17,78169632	-76,06652832	-944,4481812	0,021687
Cloud_Mesh_Distance_BA_R0_R2	17,98238754	-76,06112671	-944,460083	0,03349
Cloud_Mesh_Distance_BA_R0_R2	18,09740067	-76,04994202	-944,4261475	0,043014
Cloud_Mesh_Distance_BA_R0_R2	18,53163338	-76,21788025	-944,6445313	0,01249
Cloud_Mesh_Distance_BA_R0_R2	18,55443573	-76,15301514	-944,512207	0,028378
Cloud_Mesh_Distance_BA_R0_R2	18,78340912	-76,17623901	-944,43396	0,027448
Cloud_Mesh_Distance_BA_R0_R2	18,67227936	-76,24000549	-944,6174316	0,010662
Cloud_Mesh_Distance_BA_R0_R2	18,82403183	-76,2809906	-944,6034546	0,006445
Cloud_Mesh_Distance_BA_R0_R2	18,97730255	-76,29200745	-944,5174561	-0,001093
Cloud_Mesh_Distance_BA_R0_R2	18,99581718	-76,20822144	-944,3377075	0,025024
Cloud_Mesh_Distance_BA_R0_R2	19,10781479	-76,35595703	-944,524231	-0,021941
Cloud_Mesh_Distance_BA_R0_R2	19,12999725	-76,29907227	-944,3955689	-0,00462
Cloud_Mesh_Distance_BA_R0_R2	16,49778557	-82,67610168	-943,8955078	0,195486
Cloud_Mesh_Distance_BA_R0_R2	16,25592422	-82,45172119	-943,9307251	0,255273
Cloud_Mesh_Distance_BA_R0_R2	16,38201904	-82,60842896	-943,9707642	0,252816
Cloud_Mesh_Distance_BA_R0_R2	16,05233002	-82,21968079	-943,9666138	0,275527
Cloud_Mesh_Distance_BA_R0_R2	16,17743874	-82,43937683	-944,0229492	0,318464
Cloud_Mesh_Distance_BA_R0_R2	16,34141922	-82,51913452	-943,8937988	0,224961
Cloud_Mesh_Distance_BA_R0_R2	16,31572723	-82,42948914	-943,7995606	0,181758
Cloud_Mesh_Distance_BA_R0_R2	16,43839645	-82,58082581	-943,8248291	0,178523
Cloud_Mesh_Distance_BA_R0_R2	15,95825768	-82,12800598	-944,0253296	0,295759
Cloud_Mesh_Distance_BA_R0_R2	15,94403076	-82,02792358	-943,9653931	0,235505
Cloud_Mesh_Distance_BA_R0_R2	23,26654053	-82,97897339	-944,1096191	0,284753
Cloud_Mesh_Distance_BA_R0_R2	15,77731419	-81,72819519	-944,0519409	0,175448
Cloud_Mesh_Distance_BA_R0_R2	15,85536957	-81,7936554	-943,939148	0,148966
Cloud_Mesh_Distance_BA_R0_R2	23,21656036	-82,88490295	-944,1569214	0,332619
Cloud_Mesh_Distance_BA_R0_R2	23,14388657	-82,7721405	-944,2280273	0,243956
Cloud_Mesh_Distance_BA_R0_R2	23,22268677	-82,74156189	-944,0280151	0,233303
Cloud_Mesh_Distance_BA_R0_R2	23,26956177	-82,8805542	-944,0374146	0,251389
Cloud_Mesh_Distance_BA_R0_R2	15,71719551	-81,659729	-944,1396484	0,187449
Cloud_Mesh_Distance_BA_R0_R2	15,80640888	-81,64982605	-943,9188232	0,086098
Cloud_Mesh_Distance_BA_R0_R2	23,13130379	-82,63703918	-944,1400147	0,15877
Cloud_Mesh_Distance_BA_R0_R2	15,72303486	-81,47790527	-943,9597168	0,037424
Cloud_Mesh_Distance_BA_R0_R2	15,74717331	-81,58816528	-944,0092773	0,098805
Cloud_Mesh_Distance_BA_R0_R2	22,94587708	-82,21829224	-944,0661011	0,065435
Cloud_Mesh_Distance_BA_R0_R2	22,91474724	-82,30323792	-944,2451782	0,070268
Cloud_Mesh_Distance_BA_R0_R2	23,02034187	-82,30657959	-943,9957886	0,104195
Cloud_Mesh_Distance_BA_R0_R2	15,73969269	-81,4453125	-943,8624878	-0,003971

Cloud_Mesh_Distance_BA_R0_R2	15,67198563	-81,48503113	-944,130127	0,103632
Cloud_Mesh_Distance_BA_R0_R2	15,77782822	-81,54069519	-943,8752441	0,028851
Cloud_Mesh_Distance_BA_R0_R2	15,5186367	-81,03102112	-943,8698731	-0,088908
Cloud_Mesh_Distance_BA_R0_R2	15,59248924	-81,16880798	-943,9031372	-0,063128
Cloud_Mesh_Distance_BA_R0_R2	22,78382874	-81,86175537	-944,0411987	-0,021864
Cloud_Mesh_Distance_BA_R0_R2	22,88254166	-82,07955933	-944,0429077	0,030899
Cloud_Mesh_Distance_BA_R0_R2	15,43405819	-80,89584351	-943,9887695	-0,095199
Cloud_Mesh_Distance_BA_R0_R2	15,35805988	-80,76445007	-943,991333	-0,100575
Cloud_Mesh_Distance_BA_R0_R2	22,67637062	-81,71914673	-944,1656494	-0,0599
Cloud_Mesh_Distance_BA_R0_R2	22,7392292	-81,69120789	-943,9995728	-0,064332
Cloud_Mesh_Distance_BA_R0_R2	15,30128479	-80,65379333	-944,0993042	-0,103473
Cloud_Mesh_Distance_BA_R0_R2	22,69625854	-81,52964783	-943,9938355	-0,088635
Cloud_Mesh_Distance_BA_R0_R2	22,66810989	-81,60621643	-944,1028442	-0,078778
Cloud_Mesh_Distance_BA_R0_R2	15,15497208	-80,37904358	-944,0508423	-0,088942
Cloud_Mesh_Distance_BA_R0_R2	15,24992466	-80,52534485	-944,2415772	-0,102921
Cloud_Mesh_Distance_BA_R0_R2	22,63606453	-81,25479126	-944,0084229	-0,123128
Cloud_Mesh_Distance_BA_R0_R2	15,11618423	-80,29211426	-943,9857178	-0,091563
Cloud_Mesh_Distance_BA_R0_R2	22,44338989	-81,03242493	-944,2761231	-0,132296
Cloud_Mesh_Distance_BA_R0_R2	22,54656982	-81,31330872	-944,1918335	-0,113788
Cloud_Mesh_Distance_BA_R0_R2	15,04125404	-80,04867554	-944,1257324	-0,086722
Cloud_Mesh_Distance_BA_R0_R2	15,0813179	-80,18968201	-944,0820923	-0,090331
Cloud_Mesh_Distance_BA_R0_R2	22,45698357	-80,85671997	-944,2300415	-0,133004
Cloud_Mesh_Distance_BA_R0_R2	22,51965714	-80,97668457	-944,1548462	-0,131492
Cloud_Mesh_Distance_BA_R0_R2	22,57904816	-81,1088562	-944,0802612	-0,130559
Cloud_Mesh_Distance_BA_R0_R2	15,01417542	-79,8866272	-943,8954468	-0,096533
Cloud_Mesh_Distance_BA_R0_R2	15,01655388	-79,94874573	-944,0328369	-0,09135
Cloud_Mesh_Distance_BA_R0_R2	14,99437332	-79,81117249	-944,0421143	-0,09338
Cloud_Mesh_Distance_BA_R0_R2	15,01321888	-79,91738892	-944,1608276	-0,084008
Cloud_Mesh_Distance_BA_R0_R2	22,54491615	-80,68000793	-944,0701904	-0,137233
Cloud_Mesh_Distance_BA_R0_R2	22,53442955	-80,79727173	-944,1048584	-0,134914
Cloud_Mesh_Distance_BA_R0_R2	14,98453522	-79,67503357	-944,2251587	-0,083317
Cloud_Mesh_Distance_BA_R0_R2	14,99321747	-79,7771759	-944,2021484	-0,083779
Cloud_Mesh_Distance_BA_R0_R2	22,42439842	-80,50335693	-944,2206421	-0,124257
Cloud_Mesh_Distance_BA_R0_R2	22,47580147	-80,64741516	-944,1728516	-0,129915
Cloud_Mesh_Distance_BA_R0_R2	14,97638798	-79,61140442	-944,1069946	-0,094745
Cloud_Mesh_Distance_BA_R0_R2	14,98064804	-79,59039307	-944,2507324	-0,082172
Cloud_Mesh_Distance_BA_R0_R2	22,31223679	-80,30659485	-944,317627	-0,122706
Cloud_Mesh_Distance_BA_R0_R2	22,40906906	-80,36820984	-944,2086792	-0,124078
Cloud_Mesh_Distance_BA_R0_R2	14,97809982	-79,41944885	-944,2937622	-0,080503
Cloud_Mesh_Distance_BA_R0_R2	14,98677921	-79,16181946	-944,0489502	-0,102291
Cloud_Mesh_Distance_BA_R0_R2	22,40559196	-80,12400818	-944,119751	-0,133217
Cloud_Mesh_Distance_BA_R0_R2	22,39856911	-80,22155762	-944,1740112	-0,129588
Cloud_Mesh_Distance_BA_R0_R2	14,98103428	-79,18534851	-944,2484131	-0,085253
Cloud_Mesh_Distance_BA_R0_R2	22,17383575	-79,84684753	-944,2813721	-0,131373
Cloud_Mesh_Distance_BA_R0_R2	22,25917816	-79,87600708	-944,187561	-0,13371
Cloud_Mesh_Distance_BA_R0_R2	22,46323586	-80,16880798	-944,0548096	-0,135205
Cloud_Mesh_Distance_BA_R0_R2	22,1009655	-79,62538147	-944,2356567	-0,124968
Cloud_Mesh_Distance_BA_R0_R2	22,28274918	-79,76965332	-944,0873413	-0,135267
Cloud_Mesh_Distance_BA_R0_R2	15,04497337	-78,74807739	-944,0413208	-0,109466
Cloud_Mesh_Distance_BA_R0_R2	15,06450462	-78,59228516	-944,2356567	-0,078408

Cloud_Mesh_Distance_BA_R0_R2	21,93733788	-79,37194824	-944,2717285	-0,112987
Cloud_Mesh_Distance_BA_R0_R2	21,93344688	-79,49607849	-944,3552856	-0,117399
Cloud_Mesh_Distance_BA_R0_R2	22,01527405	-79,44032288	-944,2219238	-0,116856
Cloud_Mesh_Distance_BA_R0_R2	22,01660156	-79,60987854	-944,3280029	-0,122429
Cloud_Mesh_Distance_BA_R0_R2	15,083848	-78,56494141	-944,0601807	-0,105494
Cloud_Mesh_Distance_BA_R0_R2	15,10912323	-78,42207336	-944,2401123	-0,0783
Cloud_Mesh_Distance_BA_R0_R2	15,16896629	-78,26309204	-944,1199951	-0,096408
Cloud_Mesh_Distance_BA_R0_R2	21,89154053	-79,23466492	-944,2352905	-0,102761
Cloud_Mesh_Distance_BA_R0_R2	15,1552763	-78,27738953	-944,237793	-0,078592
Cloud_Mesh_Distance_BA_R0_R2	15,19295883	-78,17622375	-944,2132568	-0,085867
Cloud_Mesh_Distance_BA_R0_R2	21,66429138	-78,77388	-944,1589966	-0,062876
Cloud_Mesh_Distance_BA_R0_R2	21,60958862	-78,87843323	-944,3087158	-0,073397
Cloud_Mesh_Distance_BA_R0_R2	15,31717491	-77,86454773	-944,2479858	-0,075656
Cloud_Mesh_Distance_BA_R0_R2	21,34757233	-78,57785034	-944,3873291	-0,061618
Cloud_Mesh_Distance_BA_R0_R2	21,42707634	-78,63967896	-944,3386231	-0,063267
Cloud_Mesh_Distance_BA_R0_R2	21,54242706	-78,55784607	-944,1174927	-0,048485
Cloud_Mesh_Distance_BA_R0_R2	21,6631279	-78,65408325	-944,0465088	-0,049494
Cloud_Mesh_Distance_BA_R0_R2	21,54129028	-78,68080139	-944,2320557	-0,059382
Cloud_Mesh_Distance_BA_R0_R2	21,42259407	-78,51881409	-944,2420044	-0,049007
Cloud_Mesh_Distance_BA_R0_R2	15,38862038	-77,70602417	-944,2268066	-0,072388
Cloud_Mesh_Distance_BA_R0_R2	15,33789921	-77,84475708	-944,0491943	-0,0923
Cloud_Mesh_Distance_BA_R0_R2	15,40506077	-77,69145203	-944,032898	-0,088636
Cloud_Mesh_Distance_BA_R0_R2	21,31409836	-78,48001099	-944,3514404	-0,0519
Cloud_Mesh_Distance_BA_R0_R2	21,32205772	-78,37942505	-944,2542114	-0,040012
Cloud_Mesh_Distance_BA_R0_R2	21,40517998	-78,39411926	-944,1488648	-0,037479
Cloud_Mesh_Distance_BA_R0_R2	15,45035744	-77,58197021	-944,3798218	-0,048758
Cloud_Mesh_Distance_BA_R0_R2	15,52970219	-77,4256897	-944,1362915	-0,065082
Cloud_Mesh_Distance_BA_R0_R2	21,35608673	-78,26940918	-944,0939941	-0,033287
Cloud_Mesh_Distance_BA_R0_R2	21,07925415	-78,11270142	-944,364624	-0,022436
Cloud_Mesh_Distance_BA_R0_R2	21,21915436	-78,22172546	-944,256897	-0,030109
Cloud_Mesh_Distance_BA_R0_R2	15,61790085	-77,27528381	-944,0977173	-0,062652
Cloud_Mesh_Distance_BA_R0_R2	20,88511276	-77,85858154	-944,4171753	-0,015424
Cloud_Mesh_Distance_BA_R0_R2	20,96123314	-77,81341553	-944,2455444	-0,013369
Cloud_Mesh_Distance_BA_R0_R2	20,96299553	-77,9778595	-944,4138184	-0,018084
Cloud_Mesh_Distance_BA_R0_R2	15,74615097	-77,09725952	-944,2694702	-0,050657
Cloud_Mesh_Distance_BA_R0_R2	15,74826813	-77,10945129	-944,3862915	-0,042685
Cloud_Mesh_Distance_BA_R0_R2	15,77371979	-77,05583191	-944,1402588	-0,060723
Cloud_Mesh_Distance_BA_R0_R2	20,84585381	-77,57298279	-944,1506958	-0,008193
Cloud_Mesh_Distance_BA_R0_R2	20,78914261	-77,64630127	-944,3422852	-0,013309
Cloud_Mesh_Distance_BA_R0_R2	20,86777115	-77,7542572	-944,3353882	-0,014789
Cloud_Mesh_Distance_BA_R0_R2	20,59624672	-77,40112305	-944,3605957	-0,015559
Cloud_Mesh_Distance_BA_R0_R2	20,73577118	-77,45410156	-944,1904907	-0,012192
Cloud_Mesh_Distance_BA_R0_R2	16,06791687	-76,77153015	-944,2703857	-0,078121
Cloud_Mesh_Distance_BA_R0_R2	16,29875565	-76,58927917	-944,1781616	-0,090102
Cloud_Mesh_Distance_BA_R0_R2	20,20211601	-76,97302246	-944,3172607	-0,057334
Cloud_Mesh_Distance_BA_R0_R2	20,52986908	-77,22634888	-944,2192383	-0,019183
Cloud_Mesh_Distance_BA_R0_R2	16,37726974	-76,54812622	-944,2495117	-0,099768
Cloud_Mesh_Distance_BA_R0_R2	16,27615929	-76,62748718	-944,3293457	-0,095522
Cloud_Mesh_Distance_BA_R0_R2	16,52094841	-76,46159363	-944,2424316	-0,101876
Cloud_Mesh_Distance_BA_R0_R2	16,66155815	-76,40895081	-944,359436	-0,102376

Cloud_Mesh_Distance_BA_R0_R2	16,57787704	-76,48695374	-944,4852295	-0,113409
Cloud_Mesh_Distance_BA_R0_R2	19,94046211	-76,78662109	-944,3345337	-0,080688
Cloud_Mesh_Distance_BA_R0_R2	20,18528175	-76,90286255	-944,1964111	-0,058086
Cloud_Mesh_Distance_BA_R0_R2	20,05249786	-76,88609314	-944,3753662	-0,068506
Cloud_Mesh_Distance_BA_R0_R2	20,09853172	-76,83770752	-944,1942139	-0,068861
Cloud_Mesh_Distance_BA_R0_R2	20,31007004	-76,99098206	-944,1725464	-0,047171
Cloud_Mesh_Distance_BA_R0_R2	16,76888275	-76,32806396	-944,2355347	-0,084303
Cloud_Mesh_Distance_BA_R0_R2	16,89454079	-76,25590515	-944,156189	-0,064723
Cloud_Mesh_Distance_BA_R0_R2	19,43264771	-76,50437927	-944,4534302	-0,063868
Cloud_Mesh_Distance_BA_R0_R2	19,90398407	-76,70898438	-944,2131348	-0,091598
Cloud_Mesh_Distance_BA_R0_R2	19,81265259	-76,73547363	-944,4165039	-0,08838
Cloud_Mesh_Distance_BA_R0_R2	19,66967583	-76,66644287	-944,4720459	-0,087451
Cloud_Mesh_Distance_BA_R0_R2	19,91031456	-76,81912231	-944,4500122	-0,078582
Cloud_Mesh_Distance_BA_R0_R2	16,92531967	-76,26277161	-944,2748413	-0,07536
Cloud_Mesh_Distance_BA_R0_R2	17,02604675	-76,24157715	-944,3692627	-0,068769
Cloud_Mesh_Distance_BA_R0_R2	19,3812027	-76,37313843	-944,2466431	-0,039209
Cloud_Mesh_Distance_BA_R0_R2	19,49323273	-76,48886108	-944,3430786	-0,063669
Cloud_Mesh_Distance_BA_R0_R2	19,37808037	-76,42802429	-944,3701782	-0,046759
Cloud_Mesh_Distance_BA_R0_R2	19,47855759	-76,43069458	-944,2348633	-0,05431
Cloud_Mesh_Distance_BA_R0_R2	17,3608551	-76,11631775	-944,3381958	-0,019316
Cloud_Mesh_Distance_BA_R0_R2	17,47647858	-76,06469727	-944,2514038	0,004206
Cloud_Mesh_Distance_BA_R0_R2	17,80304718	-76,01199341	-944,2824097	0,040515
Cloud_Mesh_Distance_BA_R0_R2	17,64492035	-76,03967285	-944,2993774	0,02289
Cloud_Mesh_Distance_BA_R0_R2	18,35629654	-76,07321167	-944,4216309	0,044139
Cloud_Mesh_Distance_BA_R0_R2	18,38377762	-75,99528503	-944,206543	0,07488
Cloud_Mesh_Distance_BA_R0_R2	18,74821281	-76,09141541	-944,270813	0,056734
Cloud_Mesh_Distance_BA_R0_R2	18,88312149	-76,12966919	-944,2601318	0,046024
Cloud_Mesh_Distance_BA_R0_R2	19,23092079	-76,3971405	-944,4796753	-0,034708
Cloud_Mesh_Distance_BA_R0_R2	19,27328873	-76,33654785	-944,3109131	-0,023587
Cloud_Mesh_Distance_BA_R0_R2	17,91918373	-75,97541809	-944,1800537	0,060952
Cloud_Mesh_Distance_BA_R0_R2	17,92657661	-76,01687622	-944,3319092	0,045982
Cloud_Mesh_Distance_BA_R0_R2	18,0446701	-75,98777771	-944,2484131	0,062894
Cloud_Mesh_Distance_BA_R0_R2	18,03574944	-76,02352905	-944,3595581	0,045853
Cloud_Mesh_Distance_BA_R0_R2	18,16284561	-75,97885132	-944,2123413	0,070373
Cloud_Mesh_Distance_BA_R0_R2	18,19462395	-76,04930115	-944,4090576	0,047027
Cloud_Mesh_Distance_BA_R0_R2	18,24595261	-76,01083374	-944,2966309	0,064622
Cloud_Mesh_Distance_BA_R0_R2	18,12955093	-76,01387024	-944,3258057	0,056519
Cloud_Mesh_Distance_BA_R0_R2	18,81074905	-76,06402588	-944,1478272	0,060677
Cloud_Mesh_Distance_BA_R0_R2	19,00914764	-76,15818787	-944,2088623	0,036648
Cloud_Mesh_Distance_BA_R0_R2	16,85606766	-82,94810486	-943,6606445	0,064263
Cloud_Mesh_Distance_BA_R0_R2	16,96204185	-83,06057739	-943,7232666	0,087184
Cloud_Mesh_Distance_BA_R0_R2	16,83428764	-82,96328735	-943,8424072	0,111308
Cloud_Mesh_Distance_BA_R0_R2	17,07203293	-83,16265869	-943,69104	0,113968
Cloud_Mesh_Distance_BA_R0_R2	16,63978577	-82,77430725	-943,8081055	0,135808
Cloud_Mesh_Distance_BA_R0_R2	16,75468445	-82,8372345	-943,6248779	0,069791
Cloud_Mesh_Distance_BA_R0_R2	16,65753937	-82,72566223	-943,6011353	0,074674
Cloud_Mesh_Distance_BA_R0_R2	16,75949478	-82,87931824	-943,7786865	0,104474
Cloud_Mesh_Distance_BA_R0_R2	16,11487198	-82,15956116	-943,8045654	0,171607
Cloud_Mesh_Distance_BA_R0_R2	16,18931389	-82,32366943	-943,8720093	0,222405
Cloud_Mesh_Distance_BA_R0_R2	16,39420319	-82,50636292	-943,7764282	0,164494

Cloud_Mesh_Distance_BA_R0_R2	16,40662003	-82,4546051	-943,6538696	0,112498
Cloud_Mesh_Distance_BA_R0_R2	16,30533791	-82,32066345	-943,6477661	0,109041
Cloud_Mesh_Distance_BA_R0_R2	15,93568611	-81,91981506	-943,8895874	0,163454
Cloud_Mesh_Distance_BA_R0_R2	16,02445793	-82,0461731	-943,8449097	0,172632
Cloud_Mesh_Distance_BA_R0_R2	16,11601639	-82,08839417	-943,7166138	0,117106
Cloud_Mesh_Distance_BA_R0_R2	16,09440994	-81,99766541	-943,6464844	0,075793
Cloud_Mesh_Distance_BA_R0_R2	23,26241493	-82,64472961	-943,7962036	0,21333
Cloud_Mesh_Distance_BA_R0_R2	23,32213593	-82,97662354	-943,9819946	0,220508
Cloud_Mesh_Distance_BA_R0_R2	23,30655098	-82,82214355	-943,8896484	0,217624
Cloud_Mesh_Distance_BA_R0_R2	15,9199543	-81,78114319	-943,7857056	0,074471
Cloud_Mesh_Distance_BA_R0_R2	15,99898911	-81,86663818	-943,701355	0,066929
Cloud_Mesh_Distance_BA_R0_R2	23,17720985	-82,55839539	-943,9266968	0,173881
Cloud_Mesh_Distance_BA_R0_R2	15,86177635	-81,61300659	-943,7301636	0,010287
Cloud_Mesh_Distance_BA_R0_R2	22,99396896	-82,19151306	-943,8952637	0,078136
Cloud_Mesh_Distance_BA_R0_R2	23,06510544	-82,28761292	-943,8366699	0,119162
Cloud_Mesh_Distance_BA_R0_R2	23,08734703	-82,44606018	-944,0170288	0,136046
Cloud_Mesh_Distance_BA_R0_R2	15,78350258	-81,48750305	-943,7837524	-0,010751
Cloud_Mesh_Distance_BA_R0_R2	15,67141724	-81,34124756	-943,939209	-0,014057
Cloud_Mesh_Distance_BA_R0_R2	22,81797981	-81,79762268	-943,8866577	-0,040821
Cloud_Mesh_Distance_BA_R0_R2	22,87506485	-81,88070679	-943,8145752	-0,009897
Cloud_Mesh_Distance_BA_R0_R2	22,84944153	-81,94532776	-943,9630737	-0,005454
Cloud_Mesh_Distance_BA_R0_R2	15,45143986	-80,89982605	-943,7033691	-0,099301
Cloud_Mesh_Distance_BA_R0_R2	15,38956451	-80,81373596	-943,8186035	-0,099643
Cloud_Mesh_Distance_BA_R0_R2	15,46272755	-80,93229675	-943,8132324	-0,096931
Cloud_Mesh_Distance_BA_R0_R2	15,5230093	-81,00650024	-943,6814575	-0,094017
Cloud_Mesh_Distance_BA_R0_R2	15,29907894	-80,66392517	-943,8999634	-0,094419
Cloud_Mesh_Distance_BA_R0_R2	22,62956047	-81,45640564	-944,0977173	-0,096367
Cloud_Mesh_Distance_BA_R0_R2	22,80259895	-81,64265442	-943,7941895	-0,071908
Cloud_Mesh_Distance_BA_R0_R2	15,16088867	-80,39653015	-943,9032593	-0,088481
Cloud_Mesh_Distance_BA_R0_R2	15,16574955	-80,38264465	-943,7473755	-0,09404
Cloud_Mesh_Distance_BA_R0_R2	15,21736526	-80,50813293	-944,0283814	-0,090563
Cloud_Mesh_Distance_BA_R0_R2	22,69403458	-81,37915039	-943,927124	-0,112166
Cloud_Mesh_Distance_BA_R0_R2	22,74806976	-81,50434875	-943,8518677	-0,097318
Cloud_Mesh_Distance_BA_R0_R2	15,08171272	-80,19212341	-943,9310303	-0,091189
Cloud_Mesh_Distance_BA_R0_R2	22,66875458	-81,04129028	-943,8922119	-0,137057
Cloud_Mesh_Distance_BA_R0_R2	22,69664574	-81,2223053	-943,8687134	-0,130652
Cloud_Mesh_Distance_BA_R0_R2	15,04356575	-80,05787659	-943,9453125	-0,093489
Cloud_Mesh_Distance_BA_R0_R2	22,59481239	-80,91340637	-944,0187378	-0,136446
Cloud_Mesh_Distance_BA_R0_R2	15,02430534	-79,80610657	-943,7547607	-0,111656
Cloud_Mesh_Distance_BA_R0_R2	22,65287399	-80,84495544	-943,8981323	-0,14207
Cloud_Mesh_Distance_BA_R0_R2	22,49585342	-80,43484497	-944,0987549	-0,127654
Cloud_Mesh_Distance_BA_R0_R2	22,56123161	-80,56721497	-944,0231934	-0,135678
Cloud_Mesh_Distance_BA_R0_R2	22,60395813	-80,7171936	-943,9732666	-0,138902
Cloud_Mesh_Distance_BA_R0_R2	22,65277672	-80,52125549	-943,8444214	-0,144587
Cloud_Mesh_Distance_BA_R0_R2	14,97374344	-79,37442017	-944,079895	-0,0989
Cloud_Mesh_Distance_BA_R0_R2	22,57613182	-80,42324829	-943,9631958	-0,136784
Cloud_Mesh_Distance_BA_R0_R2	22,46300888	-80,28910828	-944,1053467	-0,12955
Cloud_Mesh_Distance_BA_R0_R2	22,61801147	-80,3384552	-943,8601074	-0,144526
Cloud_Mesh_Distance_BA_R0_R2	14,99238586	-79,3469696	-943,8864136	-0,116488
Cloud_Mesh_Distance_BA_R0_R2	22,53947639	-80,27531433	-943,9768677	-0,140967

Cloud_Mesh_Distance_BA_R0_R2	22,52991676	-80,10762024	-943,9138184	-0,141378
Cloud_Mesh_Distance_BA_R0_R2	15,01316261	-79,14453125	-943,8480835	-0,130956
Cloud_Mesh_Distance_BA_R0_R2	22,43091965	-80,02119446	-944,0275269	-0,142507
Cloud_Mesh_Distance_BA_R0_R2	15,0116272	-78,95178223	-944,0297852	-0,107825
Cloud_Mesh_Distance_BA_R0_R2	22,32858658	-79,92254639	-944,1204834	-0,138556
Cloud_Mesh_Distance_BA_R0_R2	15,07360935	-78,70417786	-943,8991089	-0,131027
Cloud_Mesh_Distance_BA_R0_R2	22,10481644	-79,52174377	-944,1624756	-0,123266
Cloud_Mesh_Distance_BA_R0_R2	22,17932701	-79,65223694	-944,1508789	-0,131559
Cloud_Mesh_Distance_BA_R0_R2	22,20759583	-79,47624207	-943,9866333	-0,12364
Cloud_Mesh_Distance_BA_R0_R2	22,26831818	-79,62136841	-944,0038452	-0,138984
Cloud_Mesh_Distance_BA_R0_R2	21,98913383	-79,31509399	-944,1707153	-0,109957
Cloud_Mesh_Distance_BA_R0_R2	22,08741188	-79,34371948	-944,0618286	-0,109261
Cloud_Mesh_Distance_BA_R0_R2	21,99558067	-79,20089722	-944,0808716	-0,092833
Cloud_Mesh_Distance_BA_R0_R2	15,13080406	-78,39517212	-944,0686035	-0,104145
Cloud_Mesh_Distance_BA_R0_R2	15,16582489	-78,30908203	-944,0126343	-0,115558
Cloud_Mesh_Distance_BA_R0_R2	15,18256664	-78,31651306	-943,887146	-0,130944
Cloud_Mesh_Distance_BA_R0_R2	21,88253212	-79,0410614	-944,105896	-0,076486
Cloud_Mesh_Distance_BA_R0_R2	22,01169968	-79,09822083	-943,9786987	-0,078619
Cloud_Mesh_Distance_BA_R0_R2	22,085886	-79,09568787	-943,8678589	-0,07549
Cloud_Mesh_Distance_BA_R0_R2	22,08736229	-79,19602966	-943,9500122	-0,085421
Cloud_Mesh_Distance_BA_R0_R2	22,14289474	-79,17759705	-943,8508911	-0,085094
Cloud_Mesh_Distance_BA_R0_R2	15,20504951	-78,1786499	-944,0620728	-0,102246
Cloud_Mesh_Distance_BA_R0_R2	15,23500538	-78,17393494	-943,8667603	-0,125238
Cloud_Mesh_Distance_BA_R0_R2	21,75288773	-78,88555908	-944,1428223	-0,064004
Cloud_Mesh_Distance_BA_R0_R2	21,85623741	-78,86880493	-943,9920654	-0,059157
Cloud_Mesh_Distance_BA_R0_R2	15,26958275	-78,01062012	-944,0601807	-0,096629
Cloud_Mesh_Distance_BA_R0_R2	21,74946976	-78,77998352	-944,0528565	-0,057522
Cloud_Mesh_Distance_BA_R0_R2	21,80704498	-78,72496033	-943,9157105	-0,051266
Cloud_Mesh_Distance_BA_R0_R2	15,36449432	-77,83213806	-943,8612671	-0,111657
Cloud_Mesh_Distance_BA_R0_R2	21,48716354	-78,44935608	-944,0853882	-0,043239
Cloud_Mesh_Distance_BA_R0_R2	15,45907021	-77,55976868	-944,1872559	-0,069839
Cloud_Mesh_Distance_BA_R0_R2	15,46908188	-77,55810547	-943,9990234	-0,081495
Cloud_Mesh_Distance_BA_R0_R2	21,37438011	-78,15263367	-943,9260254	-0,030964
Cloud_Mesh_Distance_BA_R0_R2	21,45770264	-78,27966309	-943,9405518	-0,036348
Cloud_Mesh_Distance_BA_R0_R2	21,51467514	-78,38209534	-943,9699097	-0,039469
Cloud_Mesh_Distance_BA_R0_R2	21,44297981	-78,35517883	-944,0515137	-0,03781
Cloud_Mesh_Distance_BA_R0_R2	15,53447342	-77,44224548	-943,9116211	-0,08027
Cloud_Mesh_Distance_BA_R0_R2	21,21008492	-77,97718811	-943,9990234	-0,019433
Cloud_Mesh_Distance_BA_R0_R2	21,05515862	-77,94909668	-944,2385864	-0,019339
Cloud_Mesh_Distance_BA_R0_R2	21,15782738	-78,03031921	-944,1550903	-0,0245
Cloud_Mesh_Distance_BA_R0_R2	15,70217133	-77,15278625	-944,0042114	-0,067392
Cloud_Mesh_Distance_BA_R0_R2	15,70141602	-77,15039063	-944,1674194	-0,054684
Cloud_Mesh_Distance_BA_R0_R2	15,80290604	-77,02667236	-943,9407349	-0,069933
Cloud_Mesh_Distance_BA_R0_R2	20,95357513	-77,66175842	-944,0679321	-0,005805
Cloud_Mesh_Distance_BA_R0_R2	21,02444649	-77,6566925	-943,9182739	-0,011205
Cloud_Mesh_Distance_BA_R0_R2	21,10224533	-77,82621765	-944,0036011	-0,015187
Cloud_Mesh_Distance_BA_R0_R2	15,86580753	-76,94927979	-944,1123657	-0,063779
Cloud_Mesh_Distance_BA_R0_R2	15,97217178	-76,84558105	-943,9231567	-0,069568
Cloud_Mesh_Distance_BA_R0_R2	20,96141434	-77,57228088	-943,9224243	-0,009209
Cloud_Mesh_Distance_BA_R0_R2	20,88427925	-77,68759155	-944,229126	-0,009219

Cloud_Mesh_Distance_BA_R0_R2	15,94565582	-76,86657715	-944,0609131	-0,064307
Cloud_Mesh_Distance_BA_R0_R2	15,95485687	-76,86480713	-944,211853	-0,069714
Cloud_Mesh_Distance_BA_R0_R2	16,04883957	-76,77223206	-944,0675049	-0,073651
Cloud_Mesh_Distance_BA_R0_R2	20,63673019	-77,32446289	-944,182251	-0,012894
Cloud_Mesh_Distance_BA_R0_R2	20,69429016	-77,30050659	-944,0372315	-0,007787
Cloud_Mesh_Distance_BA_R0_R2	20,81955719	-77,48260498	-944,0736694	-0,010392
Cloud_Mesh_Distance_BA_R0_R2	20,88780975	-77,52049255	-943,9957275	-0,009088
Cloud_Mesh_Distance_BA_R0_R2	16,07482719	-76,75721741	-943,8595581	-0,077781
Cloud_Mesh_Distance_BA_R0_R2	16,1824398	-76,6706543	-944,1690674	-0,082135
Cloud_Mesh_Distance_BA_R0_R2	16,37434769	-76,52622986	-943,9559937	-0,081729
Cloud_Mesh_Distance_BA_R0_R2	16,39164925	-76,52146912	-944,1050415	-0,092604
Cloud_Mesh_Distance_BA_R0_R2	20,40369987	-77,0974884	-944,2183228	-0,031847
Cloud_Mesh_Distance_BA_R0_R2	20,48289108	-77,08839417	-944,0587158	-0,022191
Cloud_Mesh_Distance_BA_R0_R2	20,61867905	-77,25444031	-944,1044922	-0,013229
Cloud_Mesh_Distance_BA_R0_R2	20,01203156	-76,78898621	-944,2246704	-0,080419
Cloud_Mesh_Distance_BA_R0_R2	19,99676895	-76,74154663	-944,1341553	-0,086219
Cloud_Mesh_Distance_BA_R0_R2	20,16335678	-76,83605957	-944,0784912	-0,063472
Cloud_Mesh_Distance_BA_R0_R2	20,40106964	-77,03559875	-944,1032105	-0,035544
Cloud_Mesh_Distance_BA_R0_R2	20,39655304	-76,96954346	-943,9680786	-0,038607
Cloud_Mesh_Distance_BA_R0_R2	16,52301216	-76,43322754	-943,9700928	-0,081744
Cloud_Mesh_Distance_BA_R0_R2	16,70973587	-76,32951355	-944,0001221	-0,074963
Cloud_Mesh_Distance_BA_R0_R2	16,64025497	-76,39550781	-944,242981	-0,09686
Cloud_Mesh_Distance_BA_R0_R2	16,62744522	-76,38679504	-944,1383667	-0,087885
Cloud_Mesh_Distance_BA_R0_R2	19,71865463	-76,56141663	-944,1584473	-0,087417
Cloud_Mesh_Distance_BA_R0_R2	19,72983932	-76,62173462	-944,2865601	-0,088682
Cloud_Mesh_Distance_BA_R0_R2	19,82234764	-76,60821533	-944,098938	-0,085384
Cloud_Mesh_Distance_BA_R0_R2	19,99716949	-76,69171143	-944,0025635	-0,082556
Cloud_Mesh_Distance_BA_R0_R2	20,06754494	-76,77798462	-944,102356	-0,076585
Cloud_Mesh_Distance_BA_R0_R2	20,12589645	-76,77423096	-943,9877319	-0,073516
Cloud_Mesh_Distance_BA_R0_R2	16,82050896	-76,27999878	-944,0712891	-0,063887
Cloud_Mesh_Distance_BA_R0_R2	16,93288803	-76,2224884	-944,0037842	-0,046166
Cloud_Mesh_Distance_BA_R0_R2	17,05467415	-76,19281006	-944,1873169	-0,047547
Cloud_Mesh_Distance_BA_R0_R2	17,43177795	-76,05407715	-944,1281128	0,008562
Cloud_Mesh_Distance_BA_R0_R2	17,24522781	-76,12727356	-944,2182007	-0,022905
Cloud_Mesh_Distance_BA_R0_R2	17,32698441	-76,08021545	-944,0844727	-0,005827
Cloud_Mesh_Distance_BA_R0_R2	17,44485474	-76,03442383	-943,9875488	0,014556
Cloud_Mesh_Distance_BA_R0_R2	19,43692017	-76,36177063	-944,1323242	-0,044016
Cloud_Mesh_Distance_BA_R0_R2	19,60864639	-76,51325989	-944,2250977	-0,07535
Cloud_Mesh_Distance_BA_R0_R2	19,70732689	-76,50549316	-944,0300293	-0,078466
Cloud_Mesh_Distance_BA_R0_R2	17,36340904	-76,08714294	-944,2070313	-0,005378
Cloud_Mesh_Distance_BA_R0_R2	17,57355118	-76,01329041	-944,0978394	0,025785
Cloud_Mesh_Distance_BA_R0_R2	17,75573349	-75,98516846	-944,1386108	0,048407
Cloud_Mesh_Distance_BA_R0_R2	18,25536346	-75,97897339	-944,1954346	0,075642
Cloud_Mesh_Distance_BA_R0_R2	18,54893684	-75,99771118	-944,1315918	0,073818
Cloud_Mesh_Distance_BA_R0_R2	18,58054543	-76,06719971	-944,3122559	0,058387
Cloud_Mesh_Distance_BA_R0_R2	18,67876434	-76,03948975	-944,1789551	0,068023
Cloud_Mesh_Distance_BA_R0_R2	19,0487442	-76,14048767	-944,1159058	0,036338
Cloud_Mesh_Distance_BA_R0_R2	19,11474991	-76,13279724	-943,9943237	0,033244
Cloud_Mesh_Distance_BA_R0_R2	19,24931526	-76,19483948	-943,9691162	0,013315
Cloud_Mesh_Distance_BA_R0_R2	19,17192078	-76,22303772	-944,1762695	0,012677

Cloud_Mesh_Distance_BA_R0_R2	19,36681366	-76,2802887	-944,024353	-0,017268
Cloud_Mesh_Distance_BA_R0_R2	19,32830429	-76,31298828	-944,1798706	-0,024896
Cloud_Mesh_Distance_BA_R0_R2	17,72087097	-75,96569824	-943,9594727	0,051239
Cloud_Mesh_Distance_BA_R0_R2	18,20940018	-75,9593811	-944,1323853	0,077282
Cloud_Mesh_Distance_BA_R0_R2	18,4466877	-75,9705658	-944,0924683	0,081909
Cloud_Mesh_Distance_BA_R0_R2	18,50428772	-75,95050049	-943,9713135	0,093388
Cloud_Mesh_Distance_BA_R0_R2	18,70714188	-75,99806213	-944,0147705	0,079435
Cloud_Mesh_Distance_BA_R0_R2	18,9026165	-76,04898071	-944,0001221	0,065241
Cloud_Mesh_Distance_BA_R0_R2	19,00535202	-76,09898376	-944,0445557	0,046103
Cloud_Mesh_Distance_BA_R0_R2	18,94341087	-76,10368347	-944,1341553	0,049942
Cloud_Mesh_Distance_BA_R0_R2	17,14630508	-83,22917175	-943,5532227	0,016814
Cloud_Mesh_Distance_BA_R0_R2	17,17960739	-83,26153564	-943,3868408	-0,000572
Cloud_Mesh_Distance_BA_R0_R2	17,04351807	-83,12638855	-943,576355	0,028144
Cloud_Mesh_Distance_BA_R0_R2	16,59746552	-82,64764404	-943,5700684	0,071843
Cloud_Mesh_Distance_BA_R0_R2	16,54711151	-82,64134216	-943,704834	0,119202
Cloud_Mesh_Distance_BA_R0_R2	16,74747467	-82,78352356	-943,4093628	0,023384
Cloud_Mesh_Distance_BA_R0_R2	16,85646248	-82,91990662	-943,4712524	0,029014
Cloud_Mesh_Distance_BA_R0_R2	16,52829742	-82,5488739	-943,5256348	0,062575
Cloud_Mesh_Distance_BA_R0_R2	16,46445847	-82,5460968	-943,6995239	0,125985
Cloud_Mesh_Distance_BA_R0_R2	16,2003746	-82,1990509	-943,6875	0,118306
Cloud_Mesh_Distance_BA_R0_R2	16,41950035	-82,38035583	-943,4597778	0,04591
Cloud_Mesh_Distance_BA_R0_R2	16,26246262	-82,20655823	-943,5565186	0,067845
Cloud_Mesh_Distance_BA_R0_R2	16,32862091	-82,26358032	-943,4830322	0,04754
Cloud_Mesh_Distance_BA_R0_R2	16,06505585	-81,8739624	-943,5422363	0,019322
Cloud_Mesh_Distance_BA_R0_R2	16,14188766	-81,95498657	-943,4550781	0,007622
Cloud_Mesh_Distance_BA_R0_R2	23,34703445	-82,76098633	-943,6868286	0,243626
Cloud_Mesh_Distance_BA_R0_R2	15,94262123	-81,73110962	-943,6688843	0,025447
Cloud_Mesh_Distance_BA_R0_R2	15,93314743	-81,66687012	-943,6017456	-0,005854
Cloud_Mesh_Distance_BA_R0_R2	23,20477867	-82,49766541	-943,7490845	0,189213
Cloud_Mesh_Distance_BA_R0_R2	23,13844681	-82,40216064	-943,7995606	0,151587
Cloud_Mesh_Distance_BA_R0_R2	23,30630684	-82,60804749	-943,5916748	0,239937
Cloud_Mesh_Distance_BA_R0_R2	15,83755398	-81,47120667	-943,5625	-0,061513
Cloud_Mesh_Distance_BA_R0_R2	15,79717064	-81,46214294	-943,6921997	-0,038345
Cloud_Mesh_Distance_BA_R0_R2	23,23726082	-82,47341919	-943,59729	0,211804
Cloud_Mesh_Distance_BA_R0_R2	23,17615891	-82,39179993	-943,6594238	0,174503
Cloud_Mesh_Distance_BA_R0_R2	15,67338181	-81,25773621	-943,7285767	-0,069475
Cloud_Mesh_Distance_BA_R0_R2	15,73836327	-81,39080811	-943,7700195	-0,036378
Cloud_Mesh_Distance_BA_R0_R2	15,6449852	-81,18305969	-943,6349487	-0,089035
Cloud_Mesh_Distance_BA_R0_R2	15,79524803	-81,36508179	-943,473938	-0,081032
Cloud_Mesh_Distance_BA_R0_R2	15,75932121	-81,36186218	-943,621582	-0,070125
Cloud_Mesh_Distance_BA_R0_R2	22,98455238	-81,98765564	-943,6152344	0,033519
Cloud_Mesh_Distance_BA_R0_R2	23,11770248	-82,24363708	-943,5922852	0,136315
Cloud_Mesh_Distance_BA_R0_R2	23,02331543	-82,13827515	-943,720459	0,076174
Cloud_Mesh_Distance_BA_R0_R2	15,60666084	-81,10993958	-943,5755005	-0,095847
Cloud_Mesh_Distance_BA_R0_R2	15,55291271	-81,01713562	-943,5187378	-0,098332
Cloud_Mesh_Distance_BA_R0_R2	15,5939827	-81,12736511	-943,7321777	-0,085576
Cloud_Mesh_Distance_BA_R0_R2	22,85892296	-81,75471497	-943,727417	-0,04523
Cloud_Mesh_Distance_BA_R0_R2	22,91841888	-81,86677551	-943,6665039	-0,00478
Cloud_Mesh_Distance_BA_R0_R2	22,93361473	-82,03337097	-943,8395386	0,030332
Cloud_Mesh_Distance_BA_R0_R2	15,47691536	-80,90991211	-943,5466309	-0,094317

Cloud_Mesh_Distance_BA_R0_R2	15,22732544	-80,52209473	-943,7747803	-0,093439
Cloud_Mesh_Distance_BA_R0_R2	15,40752125	-80,82191467	-943,6500244	-0,095054
Cloud_Mesh_Distance_BA_R0_R2	15,32683659	-80,69108582	-943,6727295	-0,095774
Cloud_Mesh_Distance_BA_R0_R2	15,39971161	-80,7769928	-943,5032959	-0,087212
Cloud_Mesh_Distance_BA_R0_R2	22,89705467	-81,71012878	-943,5661621	-0,050234
Cloud_Mesh_Distance_BA_R0_R2	22,84712791	-81,62730408	-943,6520996	-0,073952
Cloud_Mesh_Distance_BA_R0_R2	15,14459419	-80,29321289	-943,656189	-0,089468
Cloud_Mesh_Distance_BA_R0_R2	15,11464787	-80,26652527	-943,8134766	-0,089152
Cloud_Mesh_Distance_BA_R0_R2	15,07276917	-80,13056946	-943,8148193	-0,093055
Cloud_Mesh_Distance_BA_R0_R2	15,16082287	-80,24038696	-943,4985352	-0,092836
Cloud_Mesh_Distance_BA_R0_R2	22,73644257	-81,00917053	-943,7352905	-0,143325
Cloud_Mesh_Distance_BA_R0_R2	22,72990417	-81,13450623	-943,770752	-0,136044
Cloud_Mesh_Distance_BA_R0_R2	22,75345421	-81,30151367	-943,751648	-0,125812
Cloud_Mesh_Distance_BA_R0_R2	22,79063797	-81,10238647	-943,6089478	-0,143474
Cloud_Mesh_Distance_BA_R0_R2	15,0613575	-79,76396179	-943,5704346	-0,1271
Cloud_Mesh_Distance_BA_R0_R2	15,04929352	-79,9704895	-943,7377319	-0,101835
Cloud_Mesh_Distance_BA_R0_R2	15,08670521	-79,96990967	-943,5592041	-0,113664
Cloud_Mesh_Distance_BA_R0_R2	22,71652985	-80,84901428	-943,762207	-0,146714
Cloud_Mesh_Distance_BA_R0_R2	22,67250443	-80,71417236	-943,8405762	-0,146676
Cloud_Mesh_Distance_BA_R0_R2	15,02329922	-79,69177246	-943,7140503	-0,121994
Cloud_Mesh_Distance_BA_R0_R2	14,9960947	-79,69833374	-943,8792114	-0,106132
Cloud_Mesh_Distance_BA_R0_R2	14,98305511	-79,51634216	-943,9336548	-0,105409
Cloud_Mesh_Distance_BA_R0_R2	22,72589302	-80,65290833	-943,7177124	-0,149538
Cloud_Mesh_Distance_BA_R0_R2	22,65237427	-80,11911011	-943,6929321	-0,152179
Cloud_Mesh_Distance_BA_R0_R2	22,60770798	-80,19692993	-943,8193359	-0,145759
Cloud_Mesh_Distance_BA_R0_R2	15,07718086	-79,02461243	-943,6176758	-0,158621
Cloud_Mesh_Distance_BA_R0_R2	15,05061722	-78,9203949	-943,802124	-0,140825
Cloud_Mesh_Distance_BA_R0_R2	22,50111389	-79,75944519	-943,7271118	-0,147224
Cloud_Mesh_Distance_BA_R0_R2	22,52773285	-79,93969727	-943,8126221	-0,151583
Cloud_Mesh_Distance_BA_R0_R2	22,59074211	-80,06684875	-943,781189	-0,146709
Cloud_Mesh_Distance_BA_R0_R2	22,62553787	-79,98344421	-943,6621704	-0,152285
Cloud_Mesh_Distance_BA_R0_R2	22,58196831	-79,8434906	-943,6437378	-0,147947
Cloud_Mesh_Distance_BA_R0_R2	15,12005424	-78,84152222	-943,5644531	-0,169675
Cloud_Mesh_Distance_BA_R0_R2	15,09024906	-78,90716553	-943,6350098	-0,158833
Cloud_Mesh_Distance_BA_R0_R2	22,34022713	-79,67651367	-943,9333496	-0,145438
Cloud_Mesh_Distance_BA_R0_R2	22,40724564	-79,81556702	-943,9285889	-0,146658
Cloud_Mesh_Distance_BA_R0_R2	15,099226	-78,74609375	-943,7225952	-0,151125
Cloud_Mesh_Distance_BA_R0_R2	22,319067	-79,54603577	-943,8682861	-0,131055
Cloud_Mesh_Distance_BA_R0_R2	15,15492725	-78,46696472	-943,7870483	-0,146765
Cloud_Mesh_Distance_BA_R0_R2	15,14753246	-78,40608215	-943,9259644	-0,126995
Cloud_Mesh_Distance_BA_R0_R2	15,11293793	-78,55297852	-943,8809815	-0,131852
Cloud_Mesh_Distance_BA_R0_R2	22,18190765	-79,28302002	-943,8774414	-0,101124
Cloud_Mesh_Distance_BA_R0_R2	22,18655396	-79,20025635	-943,7997437	-0,091148
Cloud_Mesh_Distance_BA_R0_R2	22,28465652	-79,39021301	-943,8002319	-0,116204
Cloud_Mesh_Distance_BA_R0_R2	15,27285576	-78,17938232	-943,6600952	-0,153664
Cloud_Mesh_Distance_BA_R0_R2	15,29923058	-78,00090027	-943,861023	-0,118877
Cloud_Mesh_Distance_BA_R0_R2	22,04142761	-78,91181946	-943,758728	-0,052777
Cloud_Mesh_Distance_BA_R0_R2	21,99192047	-78,97581482	-943,8994141	-0,063929
Cloud_Mesh_Distance_BA_R0_R2	15,40089798	-77,80932617	-943,6854248	-0,121475
Cloud_Mesh_Distance_BA_R0_R2	21,67214203	-78,4646759	-943,8146973	-0,040209

Cloud_Mesh_Distance_BA_R0_R2	21,89318085	-78,65351868	-943,6917114	-0,036802
Cloud_Mesh_Distance_BA_R0_R2	21,73272514	-78,58078003	-943,8627319	-0,041092
Cloud_Mesh_Distance_BA_R0_R2	21,93900681	-78,82244873	-943,8234863	-0,049927
Cloud_Mesh_Distance_BA_R0_R2	15,42491913	-77,68531799	-943,8638916	-0,10139
Cloud_Mesh_Distance_BA_R0_R2	21,5886879	-78,33537292	-943,7874146	-0,032722
Cloud_Mesh_Distance_BA_R0_R2	21,5999794	-78,48901367	-943,9598389	-0,043071
Cloud_Mesh_Distance_BA_R0_R2	15,47315788	-77,58119202	-943,8512573	-0,094904
Cloud_Mesh_Distance_BA_R0_R2	21,27004051	-78,10964966	-944,055481	-0,027089
Cloud_Mesh_Distance_BA_R0_R2	21,47184372	-78,18154907	-943,7891235	-0,030465
Cloud_Mesh_Distance_BA_R0_R2	15,6245842	-77,28868103	-943,8640747	-0,070369
Cloud_Mesh_Distance_BA_R0_R2	21,22132683	-77,90858459	-943,8857422	-0,018084
Cloud_Mesh_Distance_BA_R0_R2	21,2974472	-78,01461792	-943,8858643	-0,021606
Cloud_Mesh_Distance_BA_R0_R2	15,6451931	-77,30162048	-943,6583862	-0,07915
Cloud_Mesh_Distance_BA_R0_R2	15,71360207	-77,16244507	-943,7987671	-0,073005
Cloud_Mesh_Distance_BA_R0_R2	15,80630302	-77,05673218	-943,7097168	-0,071301
Cloud_Mesh_Distance_BA_R0_R2	21,00706482	-77,57495117	-943,829773	-0,011327
Cloud_Mesh_Distance_BA_R0_R2	21,21797943	-77,80595398	-943,7417603	-0,01857
Cloud_Mesh_Distance_BA_R0_R2	21,27690506	-77,92012024	-943,7928467	-0,018791
Cloud_Mesh_Distance_BA_R0_R2	15,89448643	-76,92073059	-943,9748535	-0,065474
Cloud_Mesh_Distance_BA_R0_R2	20,9342556	-77,45695496	-943,7960205	-0,004028
Cloud_Mesh_Distance_BA_R0_R2	21,09703255	-77,65557861	-943,7634888	-0,009784
Cloud_Mesh_Distance_BA_R0_R2	21,01955986	-77,53974915	-943,7435303	-0,007096
Cloud_Mesh_Distance_BA_R0_R2	20,80921555	-77,39128113	-943,9553223	-0,003375
Cloud_Mesh_Distance_BA_R0_R2	20,74276924	-77,22668457	-943,8067627	-0,004476
Cloud_Mesh_Distance_BA_R0_R2	16,18570137	-76,67419434	-943,7746582	-0,071878
Cloud_Mesh_Distance_BA_R0_R2	16,17244339	-76,67105103	-943,9640503	-0,081152
Cloud_Mesh_Distance_BA_R0_R2	16,28089523	-76,59146118	-944,0401611	-0,088203
Cloud_Mesh_Distance_BA_R0_R2	16,28407288	-76,59111023	-943,868103	-0,077072
Cloud_Mesh_Distance_BA_R0_R2	20,60968971	-77,17414856	-943,980896	-0,012871
Cloud_Mesh_Distance_BA_R0_R2	16,40894318	-76,50996399	-943,7919922	-0,074035
Cloud_Mesh_Distance_BA_R0_R2	20,53406906	-77,04440308	-943,866394	-0,022203
Cloud_Mesh_Distance_BA_R0_R2	16,65988731	-76,35482788	-943,7987671	-0,06084
Cloud_Mesh_Distance_BA_R0_R2	16,78831673	-76,28544617	-943,8253784	-0,052219
Cloud_Mesh_Distance_BA_R0_R2	19,86507034	-76,56994629	-943,9054565	-0,07938
Cloud_Mesh_Distance_BA_R0_R2	20,30836868	-76,88002014	-943,9196167	-0,050801
Cloud_Mesh_Distance_BA_R0_R2	20,27479744	-76,82681274	-943,8424683	-0,057777
Cloud_Mesh_Distance_BA_R0_R2	20,26733208	-76,89996338	-944,0465088	-0,051661
Cloud_Mesh_Distance_BA_R0_R2	20,22086716	-76,82601929	-943,9472046	-0,061842
Cloud_Mesh_Distance_BA_R0_R2	16,82306671	-76,26991272	-943,9535523	-0,055761
Cloud_Mesh_Distance_BA_R0_R2	17,02369499	-76,17912292	-943,8049316	-0,032857
Cloud_Mesh_Distance_BA_R0_R2	16,88887978	-76,23681641	-943,8606567	-0,046293
Cloud_Mesh_Distance_BA_R0_R2	17,07036972	-76,16363525	-943,9798584	-0,030331
Cloud_Mesh_Distance_BA_R0_R2	17,31823158	-76,0700531	-943,9118042	-0,002038
Cloud_Mesh_Distance_BA_R0_R2	17,19277573	-76,12184143	-944,0407105	-0,025403
Cloud_Mesh_Distance_BA_R0_R2	17,18047333	-76,11752319	-943,8499146	-0,015284
Cloud_Mesh_Distance_BA_R0_R2	19,61047363	-76,40490723	-943,9230347	-0,062048
Cloud_Mesh_Distance_BA_R0_R2	18,2884407	-75,96340942	-944,1293335	0,080407
Cloud_Mesh_Distance_BA_R0_R2	18,36051941	-75,95217896	-944,0568848	0,084695
Cloud_Mesh_Distance_BA_R0_R2	19,08725357	-76,08932495	-943,8679199	0,047209
Cloud_Mesh_Distance_BA_R0_R2	19,21370888	-76,13677979	-943,8051148	0,023025

Cloud_Mesh_Distance_BA_R0_R2	19,55849838	-76,42677307	-944,092041	-0,06485
Cloud_Mesh_Distance_BA_R0_R2	19,49607468	-76,35604858	-944,0077515	-0,044655
Cloud_Mesh_Distance_BA_R0_R2	19,49886131	-76,31568909	-943,8637695	-0,040068
Cloud_Mesh_Distance_BA_R0_R2	17,89927101	-75,943573	-943,9971924	0,064626
Cloud_Mesh_Distance_BA_R0_R2	18,25065994	-75,94387817	-944,0554199	0,080439
Cloud_Mesh_Distance_BA_R0_R2	18,10450363	-75,9437561	-944,0699463	0,078307
Cloud_Mesh_Distance_BA_R0_R2	18,50828362	-75,92263794	-943,7910156	0,098347
Cloud_Mesh_Distance_BA_R0_R2	18,66348648	-75,95069885	-943,8175049	0,092357
Cloud_Mesh_Distance_BA_R0_R2	19,01947784	-76,08016968	-943,9539185	0,053354
Cloud_Mesh_Distance_BA_R0_R2	17,26998329	-83,35621643	-943,2770386	-0,009207
Cloud_Mesh_Distance_BA_R0_R2	17,07175636	-83,14825439	-943,3972778	0,005517
Cloud_Mesh_Distance_BA_R0_R2	16,95729065	-83,01783752	-943,3053589	-0,000094
Cloud_Mesh_Distance_BA_R0_R2	16,95928192	-83,03605652	-943,5228882	0,028695
Cloud_Mesh_Distance_BA_R0_R2	16,6394558	-82,66624451	-943,4649658	0,040969
Cloud_Mesh_Distance_BA_R0_R2	16,68000031	-82,68817139	-943,3579102	0,013108
Cloud_Mesh_Distance_BA_R0_R2	16,61671066	-82,59460449	-943,3153076	0,004915
Cloud_Mesh_Distance_BA_R0_R2	16,36827278	-82,25128174	-943,3269653	0,000427
Cloud_Mesh_Distance_BA_R0_R2	16,19346619	-82,09272766	-943,5446167	0,053081
Cloud_Mesh_Distance_BA_R0_R2	16,22825432	-82,0436554	-943,3495483	-0,010546
Cloud_Mesh_Distance_BA_R0_R2	16,28284264	-82,16340637	-943,4185181	0,019059
Cloud_Mesh_Distance_BA_R0_R2	23,4044342	-82,71377563	-943,3773804	0,270494
Cloud_Mesh_Distance_BA_R0_R2	23,36756706	-82,70722961	-943,5281372	0,256786
Cloud_Mesh_Distance_BA_R0_R2	15,99886131	-81,74429321	-943,5287476	-0,010278
Cloud_Mesh_Distance_BA_R0_R2	23,2938633	-82,49745178	-943,4338989	0,247022
Cloud_Mesh_Distance_BA_R0_R2	23,34733391	-82,6158905	-943,4469605	0,264859
Cloud_Mesh_Distance_BA_R0_R2	15,92825127	-81,59220886	-943,480957	-0,047409
Cloud_Mesh_Distance_BA_R0_R2	16,0238018	-81,6824646	-943,321228	-0,05745
Cloud_Mesh_Distance_BA_R0_R2	23,19151497	-82,35612488	-943,5422363	0,186544
Cloud_Mesh_Distance_BA_R0_R2	15,70370865	-81,2331543	-943,4997559	-0,091105
Cloud_Mesh_Distance_BA_R0_R2	15,79919147	-81,31486511	-943,3102417	-0,095107
Cloud_Mesh_Distance_BA_R0_R2	15,86974049	-81,44683838	-943,3747559	-0,082596
Cloud_Mesh_Distance_BA_R0_R2	23,04541016	-82,08056641	-943,5567627	0,073529
Cloud_Mesh_Distance_BA_R0_R2	23,17445183	-82,28746033	-943,4732666	0,17792
Cloud_Mesh_Distance_BA_R0_R2	15,62101364	-81,07962036	-943,3811646	-0,090984
Cloud_Mesh_Distance_BA_R0_R2	15,53800583	-80,96525574	-943,4033203	-0,085964
Cloud_Mesh_Distance_BA_R0_R2	23,03588486	-81,92739868	-943,3542481	0,042082
Cloud_Mesh_Distance_BA_R0_R2	23,02273178	-81,9757843	-943,4710693	0,05011
Cloud_Mesh_Distance_BA_R0_R2	15,26743412	-80,55934143	-943,5852051	-0,083024
Cloud_Mesh_Distance_BA_R0_R2	15,3295517	-80,64308167	-943,4719849	-0,081519
Cloud_Mesh_Distance_BA_R0_R2	15,33289146	-80,58628845	-943,3305664	-0,081481
Cloud_Mesh_Distance_BA_R0_R2	22,87164688	-81,55232239	-943,5229492	-0,084704
Cloud_Mesh_Distance_BA_R0_R2	22,81146812	-81,47351074	-943,6610107	-0,101105
Cloud_Mesh_Distance_BA_R0_R2	22,85762215	-81,38954163	-943,4848633	-0,115032
Cloud_Mesh_Distance_BA_R0_R2	22,92718315	-81,62837219	-943,3980713	-0,058115
Cloud_Mesh_Distance_BA_R0_R2	15,20272732	-80,41070557	-943,5761108	-0,084357
Cloud_Mesh_Distance_BA_R0_R2	15,27276421	-80,50457764	-943,4265137	-0,081845
Cloud_Mesh_Distance_BA_R0_R2	22,81248474	-81,2926178	-943,5872192	-0,128518
Cloud_Mesh_Distance_BA_R0_R2	15,10178757	-80,13923645	-943,6431274	-0,101766
Cloud_Mesh_Distance_BA_R0_R2	22,77100754	-80,92668152	-943,6412354	-0,148563
Cloud_Mesh_Distance_BA_R0_R2	15,11129189	-79,88972473	-943,4331055	-0,126235

Cloud_Mesh_Distance_BA_R0_R2	22,77948952	-80,77081299	-943,607666	-0,151107
Cloud_Mesh_Distance_BA_R0_R2	22,78691292	-80,58998108	-943,5697632	-0,151794
Cloud_Mesh_Distance_BA_R0_R2	22,83826828	-80,7098999	-943,4577637	-0,156725
Cloud_Mesh_Distance_BA_R0_R2	15,01085663	-79,53991699	-943,7526245	-0,12731
Cloud_Mesh_Distance_BA_R0_R2	15,09659576	-79,61561584	-943,4302368	-0,155301
Cloud_Mesh_Distance_BA_R0_R2	15,0364418	-79,63514709	-943,6444702	-0,12864
Cloud_Mesh_Distance_BA_R0_R2	15,11458969	-79,56033325	-943,3746338	-0,158903
Cloud_Mesh_Distance_BA_R0_R2	22,73258972	-80,4908905	-943,6723023	-0,151067
Cloud_Mesh_Distance_BA_R0_R2	22,7842865	-80,41067505	-943,5379639	-0,152241
Cloud_Mesh_Distance_BA_R0_R2	15,02810192	-79,34104919	-943,696106	-0,13811
Cloud_Mesh_Distance_BA_R0_R2	15,05523109	-79,53370667	-943,5671387	-0,141985
Cloud_Mesh_Distance_BA_R0_R2	15,07899284	-79,32614136	-943,5059204	-0,162173
Cloud_Mesh_Distance_BA_R0_R2	15,09976578	-79,49638367	-943,4196777	-0,159265
Cloud_Mesh_Distance_BA_R0_R2	22,84399605	-80,33169556	-943,37854	-0,145867
Cloud_Mesh_Distance_BA_R0_R2	22,77822495	-80,24935913	-943,4967651	-0,149354
Cloud_Mesh_Distance_BA_R0_R2	15,05192757	-79,14990234	-943,6604004	-0,149659
Cloud_Mesh_Distance_BA_R0_R2	15,08847427	-79,14237976	-943,5251465	-0,16574
Cloud_Mesh_Distance_BA_R0_R2	22,69884682	-80,29562378	-943,6824341	-0,149732
Cloud_Mesh_Distance_BA_R0_R2	22,8214817	-80,15403748	-943,3579712	-0,141786
Cloud_Mesh_Distance_BA_R0_R2	15,11513042	-79,00273132	-943,4908447	-0,17487
Cloud_Mesh_Distance_BA_R0_R2	22,55791473	-79,73110962	-943,6002197	-0,142803
Cloud_Mesh_Distance_BA_R0_R2	22,60910416	-79,77462769	-943,5383911	-0,141471
Cloud_Mesh_Distance_BA_R0_R2	15,16519356	-78,54989624	-943,6424561	-0,163063
Cloud_Mesh_Distance_BA_R0_R2	15,14362621	-78,69888306	-943,5834351	-0,169062
Cloud_Mesh_Distance_BA_R0_R2	15,1330719	-78,60282898	-943,7202759	-0,155443
Cloud_Mesh_Distance_BA_R0_R2	15,16277885	-78,85084534	-943,4116821	-0,177025
Cloud_Mesh_Distance_BA_R0_R2	22,41197777	-79,64353943	-943,7904663	-0,141841
Cloud_Mesh_Distance_BA_R0_R2	22,50259972	-79,59255981	-943,5877686	-0,138174
Cloud_Mesh_Distance_BA_R0_R2	22,38472748	-79,48396301	-943,7073975	-0,123454
Cloud_Mesh_Distance_BA_R0_R2	15,19971848	-78,60083008	-943,4636231	-0,177019
Cloud_Mesh_Distance_BA_R0_R2	15,20525169	-78,37922668	-943,677002	-0,15778
Cloud_Mesh_Distance_BA_R0_R2	22,2521019	-79,22322083	-943,7113037	-0,092727
Cloud_Mesh_Distance_BA_R0_R2	22,34323692	-79,34654236	-943,6617432	-0,112135
Cloud_Mesh_Distance_BA_R0_R2	15,26089096	-78,37794495	-943,4553833	-0,169234
Cloud_Mesh_Distance_BA_R0_R2	22,16349602	-79,12860107	-943,7739868	-0,081831
Cloud_Mesh_Distance_BA_R0_R2	22,28163147	-79,13792419	-943,576355	-0,074787
Cloud_Mesh_Distance_BA_R0_R2	22,19933891	-79,0749054	-943,6612549	-0,072749
Cloud_Mesh_Distance_BA_R0_R2	15,33668137	-77,98957825	-943,6698608	-0,136627
Cloud_Mesh_Distance_BA_R0_R2	22,10442352	-79,01815796	-943,7659302	-0,068258
Cloud_Mesh_Distance_BA_R0_R2	22,11290932	-78,84402466	-943,5507813	-0,045098
Cloud_Mesh_Distance_BA_R0_R2	22,13310814	-78,93937683	-943,631897	-0,055739
Cloud_Mesh_Distance_BA_R0_R2	22,18306923	-78,90924072	-943,500061	-0,046559
Cloud_Mesh_Distance_BA_R0_R2	22,03116798	-78,81481934	-943,6625977	-0,044497
Cloud_Mesh_Distance_BA_R0_R2	22,15704536	-78,83288574	-943,4475708	-0,040931
Cloud_Mesh_Distance_BA_R0_R2	15,42141914	-77,83564758	-943,5343018	-0,121695
Cloud_Mesh_Distance_BA_R0_R2	15,45332146	-77,71252441	-943,6135864	-0,113403
Cloud_Mesh_Distance_BA_R0_R2	15,44826126	-77,66888428	-943,7490234	-0,104545
Cloud_Mesh_Distance_BA_R0_R2	15,49419212	-77,57376099	-943,7241821	-0,098067
Cloud_Mesh_Distance_BA_R0_R2	21,66944885	-78,35769653	-943,6727905	-0,03765
Cloud_Mesh_Distance_BA_R0_R2	21,68451309	-78,41282654	-943,723938	-0,038811

Cloud_Mesh_Distance_BA_R0_R2	21,76015091	-78,50085449	-943,7136231	-0,040198
Cloud_Mesh_Distance_BA_R0_R2	21,84259415	-78,49197388	-943,5499268	-0,035383
Cloud_Mesh_Distance_BA_R0_R2	15,51108551	-77,62388611	-943,5154419	-0,104915
Cloud_Mesh_Distance_BA_R0_R2	15,59568787	-77,45036316	-943,4975586	-0,088418
Cloud_Mesh_Distance_BA_R0_R2	21,46416473	-78,08453369	-943,6639404	-0,029416
Cloud_Mesh_Distance_BA_R0_R2	15,56278229	-77,44096375	-943,6972656	-0,086825
Cloud_Mesh_Distance_BA_R0_R2	15,6736393	-77,31387329	-943,482666	-0,073147
Cloud_Mesh_Distance_BA_R0_R2	21,39732933	-77,96665955	-943,6190186	-0,024576
Cloud_Mesh_Distance_BA_R0_R2	21,39066505	-78,06236267	-943,7755127	-0,026589
Cloud_Mesh_Distance_BA_R0_R2	21,55400848	-78,17225647	-943,6183472	-0,031612
Cloud_Mesh_Distance_BA_R0_R2	15,72827244	-77,18869019	-943,5988159	-0,068904
Cloud_Mesh_Distance_BA_R0_R2	21,1762619	-77,70098877	-943,6622315	-0,011822
Cloud_Mesh_Distance_BA_R0_R2	21,2291069	-77,72348022	-943,5818481	-0,015624
Cloud_Mesh_Distance_BA_R0_R2	21,33509827	-77,97270203	-943,7546997	-0,023443
Cloud_Mesh_Distance_BA_R0_R2	21,31024551	-77,89723206	-943,6911011	-0,023101
Cloud_Mesh_Distance_BA_R0_R2	15,89302254	-76,93713379	-943,8193359	-0,07361
Cloud_Mesh_Distance_BA_R0_R2	15,91111469	-76,95881653	-943,609375	-0,070591
Cloud_Mesh_Distance_BA_R0_R2	15,99231529	-76,84799194	-943,7407837	-0,071127
Cloud_Mesh_Distance_BA_R0_R2	21,00057602	-77,48432922	-943,6904297	-0,001434
Cloud_Mesh_Distance_BA_R0_R2	16,09881592	-76,76223755	-943,6740112	-0,065681
Cloud_Mesh_Distance_BA_R0_R2	20,83826065	-77,32563782	-943,7818604	0,002251
Cloud_Mesh_Distance_BA_R0_R2	20,88620758	-77,31855774	-943,6553955	0,001144
Cloud_Mesh_Distance_BA_R0_R2	20,98962975	-77,41349792	-943,5855713	0,002802
Cloud_Mesh_Distance_BA_R0_R2	16,20229149	-76,69059753	-943,5969238	-0,065815
Cloud_Mesh_Distance_BA_R0_R2	16,45359993	-76,5123291	-943,557312	-0,061569
Cloud_Mesh_Distance_BA_R0_R2	16,30268288	-76,59832764	-943,6766357	-0,073717
Cloud_Mesh_Distance_BA_R0_R2	20,64240837	-77,10604858	-943,7753296	-0,003927
Cloud_Mesh_Distance_BA_R0_R2	20,58146286	-76,99397278	-943,6352539	-0,012098
Cloud_Mesh_Distance_BA_R0_R2	20,77808762	-77,16470337	-943,5880737	0,002149
Cloud_Mesh_Distance_BA_R0_R2	20,78188896	-77,19221497	-943,6422119	0,001012
Cloud_Mesh_Distance_BA_R0_R2	20,72749519	-77,11399841	-943,5911255	0,001574
Cloud_Mesh_Distance_BA_R0_R2	20,83153915	-77,2272644	-943,5986328	0,001635
Cloud_Mesh_Distance_BA_R0_R2	20,81583405	-77,25436401	-943,696106	-0,000013
Cloud_Mesh_Distance_BA_R0_R2	16,54265404	-76,42959595	-943,7208252	-0,063373
Cloud_Mesh_Distance_BA_R0_R2	20,36831284	-76,87966919	-943,7978516	-0,04309
Cloud_Mesh_Distance_BA_R0_R2	20,44995689	-76,89427185	-943,6584473	-0,029163
Cloud_Mesh_Distance_BA_R0_R2	20,46922112	-76,95429993	-943,7845459	-0,028685
Cloud_Mesh_Distance_BA_R0_R2	16,64919662	-76,37361145	-943,6422119	-0,053354
Cloud_Mesh_Distance_BA_R0_R2	16,77533913	-76,3057251	-943,6268311	-0,046078
Cloud_Mesh_Distance_BA_R0_R2	16,9033432	-76,23574829	-943,7044678	-0,037955
Cloud_Mesh_Distance_BA_R0_R2	19,89162827	-76,53242493	-943,7150269	-0,082373
Cloud_Mesh_Distance_BA_R0_R2	20,16924858	-76,74250793	-943,8099976	-0,069497
Cloud_Mesh_Distance_BA_R0_R2	20,00822067	-76,62492371	-943,7752075	-0,078589
Cloud_Mesh_Distance_BA_R0_R2	19,9941597	-76,57730103	-943,6306763	-0,081883
Cloud_Mesh_Distance_BA_R0_R2	20,29483604	-76,80760193	-943,7437744	-0,053687
Cloud_Mesh_Distance_BA_R0_R2	20,28676224	-76,75866699	-943,597168	-0,055666
Cloud_Mesh_Distance_BA_R0_R2	17,04656029	-76,18528748	-943,5823364	-0,022035
Cloud_Mesh_Distance_BA_R0_R2	17,32899475	-76,06956482	-943,7116699	0,000942
Cloud_Mesh_Distance_BA_R0_R2	17,47508049	-76,01994324	-943,7835693	0,021309
Cloud_Mesh_Distance_BA_R0_R2	17,46834946	-76,03395081	-943,6021118	0,016881

Cloud_Mesh_Distance_BA_R0_R2	19,75320816	-76,46862793	-943,8155518	-0,077461
Cloud_Mesh_Distance_BA_R0_R2	17,61431503	-75,98193359	-943,7600708	0,040875
Cloud_Mesh_Distance_BA_R0_R2	17,58103371	-75,99211121	-943,9031982	0,035232
Cloud_Mesh_Distance_BA_R0_R2	19,36691856	-76,22567749	-943,8278809	-0,00715
Cloud_Mesh_Distance_BA_R0_R2	19,35168839	-76,18060303	-943,6253052	0,001425
Cloud_Mesh_Distance_BA_R0_R2	19,63677216	-76,37112427	-943,7306519	-0,052807
Cloud_Mesh_Distance_BA_R0_R2	17,69503021	-75,96266174	-943,8197632	0,050519
Cloud_Mesh_Distance_BA_R0_R2	17,85632706	-75,93251038	-943,7888794	0,070733
Cloud_Mesh_Distance_BA_R0_R2	18,06991768	-75,91261292	-943,8360596	0,086961
Cloud_Mesh_Distance_BA_R0_R2	17,8812294	-75,93273926	-943,5925293	0,069979
Cloud_Mesh_Distance_BA_R0_R2	18,30774879	-75,91716003	-943,8843994	0,094151
Cloud_Mesh_Distance_BA_R0_R2	18,24129105	-75,89901733	-943,6445313	0,095156
Cloud_Mesh_Distance_BA_R0_R2	18,44485664	-75,90597534	-943,644104	0,097714
Cloud_Mesh_Distance_BA_R0_R2	18,62666321	-75,92695618	-943,6394653	0,09779
Cloud_Mesh_Distance_BA_R0_R2	18,83903885	-75,9947052	-943,8323364	0,07786
Cloud_Mesh_Distance_BA_R0_R2	19,09714508	-76,06932068	-943,7282715	0,047695
Cloud_Mesh_Distance_BA_R0_R2	18,96973419	-76,01283264	-943,6553345	0,065066
Cloud_Mesh_Distance_BA_R0_R2	18,98730278	-76,04420471	-943,8432617	0,064071
Cloud_Mesh_Distance_BA_R0_R2	17,4525528	-83,5426178	-943,003479	-0,019185
Cloud_Mesh_Distance_BA_R0_R2	17,35064507	-83,44087219	-943,114563	-0,016232
Cloud_Mesh_Distance_BA_R0_R2	17,22774315	-83,31201172	-943,0566406	-0,021036
Cloud_Mesh_Distance_BA_R0_R2	17,05864143	-83,12263489	-943,0569458	-0,024867
Cloud_Mesh_Distance_BA_R0_R2	17,12586212	-83,20178223	-943,1020508	-0,020032
Cloud_Mesh_Distance_BA_R0_R2	17,05984497	-83,13053894	-943,2058716	-0,01221
Cloud_Mesh_Distance_BA_R0_R2	17,15522003	-83,23570251	-943,2367554	-0,011255
Cloud_Mesh_Distance_BA_R0_R2	16,99443054	-83,0418396	-943,0210571	-0,031475
Cloud_Mesh_Distance_BA_R0_R2	16,90510559	-82,93591309	-943,1010742	-0,026209
Cloud_Mesh_Distance_BA_R0_R2	16,99456978	-83,05102539	-943,1377564	-0,01836
Cloud_Mesh_Distance_BA_R0_R2	16,65396881	-82,60261536	-943,1466065	-0,028327
Cloud_Mesh_Distance_BA_R0_R2	16,71624756	-82,70530701	-943,2209473	-0,0141
Cloud_Mesh_Distance_BA_R0_R2	16,71064949	-82,66247559	-943,0591431	-0,041988
Cloud_Mesh_Distance_BA_R0_R2	16,7951889	-82,77624512	-943,0299072	-0,043246
Cloud_Mesh_Distance_BA_R0_R2	16,80470848	-82,82093811	-943,2136231	-0,012787
Cloud_Mesh_Distance_BA_R0_R2	16,85522842	-82,89668274	-943,3008423	-0,000099
Cloud_Mesh_Distance_BA_R0_R2	16,46347809	-82,35682678	-943,2357788	-0,016871
Cloud_Mesh_Distance_BA_R0_R2	16,39728546	-82,21530151	-943,1165772	-0,037769
Cloud_Mesh_Distance_BA_R0_R2	16,5702858	-82,48143005	-943,1400147	-0,032784
Cloud_Mesh_Distance_BA_R0_R2	16,52125359	-82,47264099	-943,336853	0,012356
Cloud_Mesh_Distance_BA_R0_R2	16,31097412	-82,13027954	-943,2470703	-0,022399
Cloud_Mesh_Distance_BA_R0_R2	16,08774757	-81,82228088	-943,3814697	-0,030781
Cloud_Mesh_Distance_BA_R0_R2	16,25867271	-82,01490784	-943,1709595	-0,044521
Cloud_Mesh_Distance_BA_R0_R2	16,17552948	-81,91578674	-943,2627564	-0,037793
Cloud_Mesh_Distance_BA_R0_R2	23,29994202	-82,4138031	-943,2242432	0,255033
Cloud_Mesh_Distance_BA_R0_R2	23,36472511	-82,51983643	-943,1403809	0,287159
Cloud_Mesh_Distance_BA_R0_R2	23,35745239	-82,56629944	-943,2991333	0,27781
Cloud_Mesh_Distance_BA_R0_R2	15,89111137	-81,40084839	-943,1627808	-0,09011
Cloud_Mesh_Distance_BA_R0_R2	15,95794487	-81,5406189	-943,2490234	-0,079181
Cloud_Mesh_Distance_BA_R0_R2	23,22794724	-82,35185242	-943,3988648	0,210947
Cloud_Mesh_Distance_BA_R0_R2	15,6612978	-81,07849121	-943,199646	-0,08979
Cloud_Mesh_Distance_BA_R0_R2	15,7203207	-81,18501282	-943,2628784	-0,091903

Cloud_Mesh_Distance_BA_R0_R2	15,72648907	-81,13426208	-943,1011353	-0,086074
Cloud_Mesh_Distance_BA_R0_R2	23,16568756	-82,20449829	-943,3497925	0,162155
Cloud_Mesh_Distance_BA_R0_R2	23,08356094	-82,0639801	-943,3977661	0,095831
Cloud_Mesh_Distance_BA_R0_R2	23,12797165	-82,0635376	-943,2282715	0,116329
Cloud_Mesh_Distance_BA_R0_R2	23,23175621	-82,27693176	-943,2248535	0,210594
Cloud_Mesh_Distance_BA_R0_R2	15,67146683	-81,0280304	-943,046875	-0,075329
Cloud_Mesh_Distance_BA_R0_R2	23,02055359	-81,83042908	-943,2781982	0,009238
Cloud_Mesh_Distance_BA_R0_R2	23,0756073	-81,92715454	-943,2037354	0,054539
Cloud_Mesh_Distance_BA_R0_R2	15,48039436	-80,80738831	-943,2174683	-0,074575
Cloud_Mesh_Distance_BA_R0_R2	15,56954575	-80,94406128	-943,2044678	-0,077581
Cloud_Mesh_Distance_BA_R0_R2	15,47516441	-80,85710144	-943,3640747	-0,080399
Cloud_Mesh_Distance_BA_R0_R2	23,03981018	-81,7769165	-943,1314087	0,004023
Cloud_Mesh_Distance_BA_R0_R2	22,97136879	-81,82775879	-943,447937	-0,006982
Cloud_Mesh_Distance_BA_R0_R2	15,30423546	-80,46685791	-943,255188	-0,086948
Cloud_Mesh_Distance_BA_R0_R2	15,44548607	-80,6975708	-943,1349487	-0,074237
Cloud_Mesh_Distance_BA_R0_R2	15,37256432	-80,58380127	-943,1921387	-0,077712
Cloud_Mesh_Distance_BA_R0_R2	15,40098953	-80,71235657	-943,3151856	-0,078931
Cloud_Mesh_Distance_BA_R0_R2	22,90097046	-81,4818573	-943,3887329	-0,095238
Cloud_Mesh_Distance_BA_R0_R2	22,90398598	-81,3394928	-943,3226318	-0,121506
Cloud_Mesh_Distance_BA_R0_R2	22,85691452	-81,19654846	-943,4364014	-0,136584
Cloud_Mesh_Distance_BA_R0_R2	22,90908051	-81,18858337	-943,2749634	-0,138712
Cloud_Mesh_Distance_BA_R0_R2	15,18641567	-80,17352295	-943,3461304	-0,109734
Cloud_Mesh_Distance_BA_R0_R2	15,18572235	-80,06066895	-943,2803345	-0,115835
Cloud_Mesh_Distance_BA_R0_R2	15,22925949	-80,33013916	-943,3460083	-0,093049
Cloud_Mesh_Distance_BA_R0_R2	22,94057655	-81,11471558	-943,171814	-0,14293
Cloud_Mesh_Distance_BA_R0_R2	15,16427994	-79,90556335	-943,2810059	-0,133043
Cloud_Mesh_Distance_BA_R0_R2	15,13680935	-80,05986023	-943,4310913	-0,112371
Cloud_Mesh_Distance_BA_R0_R2	22,84196854	-81,05455017	-943,4626465	-0,149259
Cloud_Mesh_Distance_BA_R0_R2	22,89265251	-81,01197815	-943,3162842	-0,15333
Cloud_Mesh_Distance_BA_R0_R2	15,12667561	-79,72523499	-943,3486939	-0,148747
Cloud_Mesh_Distance_BA_R0_R2	22,82815933	-80,91213989	-943,493042	-0,15379
Cloud_Mesh_Distance_BA_R0_R2	22,879776	-80,8213501	-943,3530273	-0,154559
Cloud_Mesh_Distance_BA_R0_R2	15,12771225	-79,59567261	-943,3375244	-0,158055
Cloud_Mesh_Distance_BA_R0_R2	15,16737366	-79,54023743	-943,2255859	-0,164479
Cloud_Mesh_Distance_BA_R0_R2	22,84807205	-80,52171326	-943,4130249	-0,147344
Cloud_Mesh_Distance_BA_R0_R2	15,12822533	-79,52236938	-943,3346558	-0,161494
Cloud_Mesh_Distance_BA_R0_R2	15,12661552	-79,40603638	-943,3460083	-0,166103
Cloud_Mesh_Distance_BA_R0_R2	15,12796116	-79,20536804	-943,3778076	-0,173552
Cloud_Mesh_Distance_BA_R0_R2	22,86264038	-80,07752991	-943,2226563	-0,13041
Cloud_Mesh_Distance_BA_R0_R2	15,18299866	-79,17399597	-943,2260132	-0,180805
Cloud_Mesh_Distance_BA_R0_R2	15,15655136	-79,04444885	-943,3411255	-0,177917
Cloud_Mesh_Distance_BA_R0_R2	22,65396118	-79,73408508	-943,4180298	-0,137404
Cloud_Mesh_Distance_BA_R0_R2	22,6578846	-79,8767395	-943,5239258	-0,146616
Cloud_Mesh_Distance_BA_R0_R2	22,72657394	-80,06452942	-943,5126343	-0,146343
Cloud_Mesh_Distance_BA_R0_R2	22,8060379	-79,98893738	-943,2978516	-0,134626
Cloud_Mesh_Distance_BA_R0_R2	15,20292187	-78,98539734	-943,2272339	-0,184221
Cloud_Mesh_Distance_BA_R0_R2	15,22750473	-78,77935791	-943,25	-0,184166
Cloud_Mesh_Distance_BA_R0_R2	22,5856514	-79,68644714	-943,5112305	-0,13853
Cloud_Mesh_Distance_BA_R0_R2	22,59513474	-79,59851074	-943,4155884	-0,126367
Cloud_Mesh_Distance_BA_R0_R2	22,42991257	-79,39689636	-943,5493164	-0,118875

Cloud_Mesh_Distance_BA_R0_R2	22,65908241	-79,62788391	-943,3139038	-0,122735
Cloud_Mesh_Distance_BA_R0_R2	22,35265923	-79,24928284	-943,5543213	-0,091754
Cloud_Mesh_Distance_BA_R0_R2	22,44842529	-79,2694397	-943,3847046	-0,089851
Cloud_Mesh_Distance_BA_R0_R2	15,35975456	-78,21820068	-943,2738648	-0,16101
Cloud_Mesh_Distance_BA_R0_R2	15,32647324	-78,16795349	-943,4477539	-0,154355
Cloud_Mesh_Distance_BA_R0_R2	22,32234383	-79,03669739	-943,3757324	-0,064122
Cloud_Mesh_Distance_BA_R0_R2	22,24843407	-79,01921082	-943,5071411	-0,061376
Cloud_Mesh_Distance_BA_R0_R2	22,34521866	-79,13468933	-943,4477539	-0,073632
Cloud_Mesh_Distance_BA_R0_R2	15,38392639	-78,07904053	-943,3395386	-0,152787
Cloud_Mesh_Distance_BA_R0_R2	15,37918282	-77,96507263	-943,5100708	-0,135776
Cloud_Mesh_Distance_BA_R0_R2	22,34729767	-79,01037598	-943,2822266	-0,061737
Cloud_Mesh_Distance_BA_R0_R2	15,42403126	-77,91864014	-943,3938599	-0,1334
Cloud_Mesh_Distance_BA_R0_R2	21,95758438	-78,57926941	-943,4630737	-0,035996
Cloud_Mesh_Distance_BA_R0_R2	21,89725876	-78,47053528	-943,4068604	-0,034529
Cloud_Mesh_Distance_BA_R0_R2	22,07046127	-78,62892151	-943,3052979	-0,030284
Cloud_Mesh_Distance_BA_R0_R2	22,16860199	-78,76817322	-943,3201904	-0,039443
Cloud_Mesh_Distance_BA_R0_R2	22,0577507	-78,72969055	-943,4966431	-0,039722
Cloud_Mesh_Distance_BA_R0_R2	15,45731163	-77,78106689	-943,47052	-0,118285
Cloud_Mesh_Distance_BA_R0_R2	21,80893898	-78,35128784	-943,3892822	-0,028292
Cloud_Mesh_Distance_BA_R0_R2	21,97973824	-78,50857544	-943,2957764	-0,029667
Cloud_Mesh_Distance_BA_R0_R2	21,73889923	-78,39924622	-943,6057739	-0,033324
Cloud_Mesh_Distance_BA_R0_R2	21,67049217	-78,27900696	-943,5529785	-0,032139
Cloud_Mesh_Distance_BA_R0_R2	21,71153641	-78,22280884	-943,3760376	-0,027213
Cloud_Mesh_Distance_BA_R0_R2	15,70913029	-77,30804443	-943,3613892	-0,06883
Cloud_Mesh_Distance_BA_R0_R2	21,50407791	-78,03793335	-943,508728	-0,024626
Cloud_Mesh_Distance_BA_R0_R2	15,82000637	-77,10516357	-943,4741211	-0,06762
Cloud_Mesh_Distance_BA_R0_R2	15,73793793	-77,22531128	-943,4505005	-0,068523
Cloud_Mesh_Distance_BA_R0_R2	15,77522182	-77,22973633	-943,3168945	-0,06685
Cloud_Mesh_Distance_BA_R0_R2	21,26636505	-77,67355347	-943,4018555	-0,00791
Cloud_Mesh_Distance_BA_R0_R2	21,31966209	-77,81887817	-943,5421143	-0,017219
Cloud_Mesh_Distance_BA_R0_R2	21,15795898	-77,61973572	-943,5626831	-0,009987
Cloud_Mesh_Distance_BA_R0_R2	16,02727509	-76,85691833	-943,5459595	-0,067984
Cloud_Mesh_Distance_BA_R0_R2	20,90310097	-77,25883484	-943,480835	0,004821
Cloud_Mesh_Distance_BA_R0_R2	21,06033707	-77,53311157	-943,6383057	-0,005458
Cloud_Mesh_Distance_BA_R0_R2	16,20801544	-76,71647644	-943,4730225	-0,065184
Cloud_Mesh_Distance_BA_R0_R2	16,13126373	-76,77035522	-943,5133667	-0,065409
Cloud_Mesh_Distance_BA_R0_R2	16,45418549	-76,58044434	-943,2972412	-0,051584
Cloud_Mesh_Distance_BA_R0_R2	20,66587257	-77,02723694	-943,5142822	-0,004121
Cloud_Mesh_Distance_BA_R0_R2	16,58711815	-76,42323303	-943,5614014	-0,053767
Cloud_Mesh_Distance_BA_R0_R2	16,69540787	-76,36947632	-943,4968262	-0,043499
Cloud_Mesh_Distance_BA_R0_R2	16,73345947	-76,37692261	-943,3630981	-0,036115
Cloud_Mesh_Distance_BA_R0_R2	20,63087463	-76,96276855	-943,4004517	-0,011669
Cloud_Mesh_Distance_BA_R0_R2	20,79843521	-77,18681335	-943,5892334	0,002133
Cloud_Mesh_Distance_BA_R0_R2	20,80836105	-77,17643738	-943,5393677	0,005547
Cloud_Mesh_Distance_BA_R0_R2	16,83503342	-76,31268311	-943,3978272	-0,030505
Cloud_Mesh_Distance_BA_R0_R2	20,12117195	-76,64810181	-943,5881958	-0,071074
Cloud_Mesh_Distance_BA_R0_R2	20,36265564	-76,83337402	-943,6721802	-0,042656
Cloud_Mesh_Distance_BA_R0_R2	20,49992752	-76,88420105	-943,4971314	-0,026189
Cloud_Mesh_Distance_BA_R0_R2	20,40112114	-76,83068848	-943,5689087	-0,038531
Cloud_Mesh_Distance_BA_R0_R2	17,06112671	-76,20796204	-943,3974609	-0,015386

Cloud_Mesh_Distance_BA_R0_R2	16,92753601	-76,24581909	-943,513855	-0,029969
Cloud_Mesh_Distance_BA_R0_R2	17,38143921	-76,07984924	-943,4716187	0,003308
Cloud_Mesh_Distance_BA_R0_R2	17,18839264	-76,1245575	-943,6414795	-0,014424
Cloud_Mesh_Distance_BA_R0_R2	17,20397949	-76,14831543	-943,423584	-0,008808
Cloud_Mesh_Distance_BA_R0_R2	19,92574501	-76,52114868	-943,5750732	-0,075704
Cloud_Mesh_Distance_BA_R0_R2	19,79629517	-76,44328308	-943,5916748	-0,067832
Cloud_Mesh_Distance_BA_R0_R2	20,02022934	-76,56533813	-943,5036621	-0,074734
Cloud_Mesh_Distance_BA_R0_R2	20,0398407	-76,55218506	-943,3617554	-0,071209
Cloud_Mesh_Distance_BA_R0_R2	20,11218834	-76,60812378	-943,4294434	-0,07131
Cloud_Mesh_Distance_BA_R0_R2	17,71823692	-75,99203491	-943,4104004	0,042954
Cloud_Mesh_Distance_BA_R0_R2	17,78051949	-75,95968628	-943,520752	0,056197
Cloud_Mesh_Distance_BA_R0_R2	17,61645889	-75,99134827	-943,600708	0,037859
Cloud_Mesh_Distance_BA_R0_R2	17,56757927	-76,02783203	-943,4390869	0,023377
Cloud_Mesh_Distance_BA_R0_R2	19,16757393	-76,07722473	-943,4761963	0,039248
Cloud_Mesh_Distance_BA_R0_R2	19,10120773	-76,05543518	-943,581604	0,04852
Cloud_Mesh_Distance_BA_R0_R2	19,20839882	-76,10462952	-943,6039429	0,031996
Cloud_Mesh_Distance_BA_R0_R2	19,50257874	-76,27442932	-943,6679077	-0,026064
Cloud_Mesh_Distance_BA_R0_R2	17,7343483	-75,95611572	-943,6824951	0,051368
Cloud_Mesh_Distance_BA_R0_R2	17,97653198	-75,94387817	-943,3670654	0,070921
Cloud_Mesh_Distance_BA_R0_R2	17,8988266	-75,9427948	-943,4649658	0,068433
Cloud_Mesh_Distance_BA_R0_R2	18,03879356	-75,91023254	-943,6264648	0,082755
Cloud_Mesh_Distance_BA_R0_R2	18,01250458	-75,92424011	-943,4672241	0,081573
Cloud_Mesh_Distance_BA_R0_R2	18,4027977	-75,90589905	-943,4515381	0,099766
Cloud_Mesh_Distance_BA_R0_R2	18,75748825	-75,94764709	-943,4753418	0,088798
Cloud_Mesh_Distance_BA_R0_R2	18,7946701	-75,96166992	-943,6526489	0,085853
Cloud_Mesh_Distance_BA_R0_R2	19,04618645	-76,02877808	-943,4458618	0,055128
Cloud_Mesh_Distance_BA_R0_R2	17,43613434	-83,51896667	-942,8163452	-0,025052
Cloud_Mesh_Distance_BA_R0_R2	17,3523941	-83,42973328	-942,7627564	-0,028938
Cloud_Mesh_Distance_BA_R0_R2	17,23489761	-83,30522156	-942,7886963	-0,032989
Cloud_Mesh_Distance_BA_R0_R2	17,34001923	-83,42698669	-942,9240112	-0,025941
Cloud_Mesh_Distance_BA_R0_R2	17,13269424	-83,20283508	-942,9537964	-0,031589
Cloud_Mesh_Distance_BA_R0_R2	17,04946327	-83,10067749	-942,9196777	-0,036405
Cloud_Mesh_Distance_BA_R0_R2	16,73308182	-82,65644836	-942,8969727	-0,062138
Cloud_Mesh_Distance_BA_R0_R2	16,8803978	-82,873703	-942,9071045	-0,050155
Cloud_Mesh_Distance_BA_R0_R2	16,65345001	-82,56297302	-942,9936523	-0,053923
Cloud_Mesh_Distance_BA_R0_R2	16,66792107	-82,52914429	-942,7984009	-0,078182
Cloud_Mesh_Distance_BA_R0_R2	16,49592781	-82,33784485	-943,0308838	-0,047587
Cloud_Mesh_Distance_BA_R0_R2	16,32588196	-82,09611511	-943,0997315	-0,048056
Cloud_Mesh_Distance_BA_R0_R2	16,1391983	-81,71986389	-942,942688	-0,074436
Cloud_Mesh_Distance_BA_R0_R2	16,11549187	-81,77635193	-943,1764526	-0,061216
Cloud_Mesh_Distance_BA_R0_R2	16,29305458	-82,01448059	-943,0255127	-0,058922
Cloud_Mesh_Distance_BA_R0_R2	16,21563339	-81,8910675	-943,0409546	-0,065803
Cloud_Mesh_Distance_BA_R0_R2	16,29657745	-81,9624939	-942,8768311	-0,069342
Cloud_Mesh_Distance_BA_R0_R2	23,33614159	-82,42732239	-943,0453491	0,283825
Cloud_Mesh_Distance_BA_R0_R2	16,0020752	-81,45878601	-942,883728	-0,085801
Cloud_Mesh_Distance_BA_R0_R2	15,98056602	-81,49293518	-943,039978	-0,089597
Cloud_Mesh_Distance_BA_R0_R2	15,92543125	-81,35006714	-942,9156494	-0,084371
Cloud_Mesh_Distance_BA_R0_R2	16,04641151	-81,63017273	-943,1083374	-0,080032
Cloud_Mesh_Distance_BA_R0_R2	16,05770302	-81,56620789	-942,9129639	-0,081378
Cloud_Mesh_Distance_BA_R0_R2	23,33917236	-82,40153503	-942,8789063	0,292855

Cloud_Mesh_Distance_BA_R0_R2	15,81101704	-81,24909973	-943,0809937	-0,085208
Cloud_Mesh_Distance_BA_R0_R2	23,26729774	-82,27119446	-943,0095215	0,2375
Cloud_Mesh_Distance_BA_R0_R2	23,18989372	-82,15252686	-943,1369629	0,17311
Cloud_Mesh_Distance_BA_R0_R2	15,6939106	-80,98521423	-942,9057617	-0,071385
Cloud_Mesh_Distance_BA_R0_R2	23,0872612	-81,85954285	-943,0384522	0,047078
Cloud_Mesh_Distance_BA_R0_R2	15,5225172	-80,80293274	-943,0729981	-0,075632
Cloud_Mesh_Distance_BA_R0_R2	22,98538971	-81,69664001	-943,2554321	-0,034665
Cloud_Mesh_Distance_BA_R0_R2	23,01329613	-81,61816406	-943,0693359	-0,042437
Cloud_Mesh_Distance_BA_R0_R2	15,35351181	-80,44664001	-943,0962524	-0,084174
Cloud_Mesh_Distance_BA_R0_R2	15,29187489	-80,32176208	-943,1536255	-0,091629
Cloud_Mesh_Distance_BA_R0_R2	15,40963554	-80,39555359	-942,9299927	-0,093128
Cloud_Mesh_Distance_BA_R0_R2	22,95239067	-81,4984436	-943,2224731	-0,083518
Cloud_Mesh_Distance_BA_R0_R2	22,95598793	-81,28175354	-943,1317139	-0,121533
Cloud_Mesh_Distance_BA_R0_R2	15,34454727	-80,32228088	-943,0259399	-0,09537
Cloud_Mesh_Distance_BA_R0_R2	22,99542618	-81,10163879	-942,9884644	-0,139026
Cloud_Mesh_Distance_BA_R0_R2	23,00498009	-81,28800964	-942,9468994	-0,115673
Cloud_Mesh_Distance_BA_R0_R2	15,22066879	-79,8417511	-943,1212158	-0,143237
Cloud_Mesh_Distance_BA_R0_R2	15,2162323	-79,98931885	-943,1709595	-0,122394
Cloud_Mesh_Distance_BA_R0_R2	15,23789215	-80,15190125	-943,1860352	-0,108546
Cloud_Mesh_Distance_BA_R0_R2	15,31166172	-80,18721008	-943,0272217	-0,112585
Cloud_Mesh_Distance_BA_R0_R2	22,98692322	-80,9105835	-943,0401001	-0,147157
Cloud_Mesh_Distance_BA_R0_R2	22,95106888	-80,99182129	-943,1431274	-0,148749
Cloud_Mesh_Distance_BA_R0_R2	15,28623199	-79,96713257	-943,0037842	-0,133209
Cloud_Mesh_Distance_BA_R0_R2	15,22402382	-79,66537476	-943,0892334	-0,161114
Cloud_Mesh_Distance_BA_R0_R2	22,89839935	-80,65698242	-943,2995606	-0,15189
Cloud_Mesh_Distance_BA_R0_R2	22,93776321	-80,82374573	-943,1921997	-0,152697
Cloud_Mesh_Distance_BA_R0_R2	15,17860413	-79,73921204	-943,2086792	-0,152838
Cloud_Mesh_Distance_BA_R0_R2	22,97434044	-80,41319275	-943,0686035	-0,137605
Cloud_Mesh_Distance_BA_R0_R2	22,92087173	-80,44389343	-943,2174072	-0,142495
Cloud_Mesh_Distance_BA_R0_R2	15,28671074	-79,58076477	-942,9419556	-0,161058
Cloud_Mesh_Distance_BA_R0_R2	15,26747131	-79,41923523	-942,9828491	-0,169382
Cloud_Mesh_Distance_BA_R0_R2	15,23150063	-79,51095581	-943,0645752	-0,168341
Cloud_Mesh_Distance_BA_R0_R2	22,95647812	-80,27212524	-943,0769043	-0,131508
Cloud_Mesh_Distance_BA_R0_R2	23,00982475	-80,34431458	-942,9501953	-0,129748
Cloud_Mesh_Distance_BA_R0_R2	15,16328239	-79,33462524	-943,2506104	-0,174512
Cloud_Mesh_Distance_BA_R0_R2	22,89188004	-80,22163391	-943,2231445	-0,137887
Cloud_Mesh_Distance_BA_R0_R2	22,733181	-79,86366272	-943,3623657	-0,137661
Cloud_Mesh_Distance_BA_R0_R2	22,85976028	-79,96763611	-943,1598511	-0,120243
Cloud_Mesh_Distance_BA_R0_R2	15,26769257	-78,5703125	-943,2616577	-0,175278
Cloud_Mesh_Distance_BA_R0_R2	15,29176521	-78,73452759	-943,0904541	-0,174245
Cloud_Mesh_Distance_BA_R0_R2	22,62395096	-79,50169373	-943,2629395	-0,109535
Cloud_Mesh_Distance_BA_R0_R2	22,69040298	-79,50621033	-943,118042	-0,100825
Cloud_Mesh_Distance_BA_R0_R2	22,71981049	-79,71020508	-943,2628174	-0,119724
Cloud_Mesh_Distance_BA_R0_R2	22,69120598	-79,59510803	-943,213562	-0,11108
Cloud_Mesh_Distance_BA_R0_R2	22,52894211	-79,45030212	-943,4075928	-0,112105
Cloud_Mesh_Distance_BA_R0_R2	22,57199287	-79,28645325	-943,1236572	-0,081614
Cloud_Mesh_Distance_BA_R0_R2	22,55312538	-79,35020447	-943,2501831	-0,095476
Cloud_Mesh_Distance_BA_R0_R2	15,37703514	-78,29696655	-943,1452637	-0,156773
Cloud_Mesh_Distance_BA_R0_R2	15,3222084	-78,37313843	-943,2476807	-0,170758
Cloud_Mesh_Distance_BA_R0_R2	15,3959341	-78,16964722	-943,2036743	-0,152039

Cloud_Mesh_Distance_BA_R0_R2	22,39142036	-79,0350647	-943,2109375	-0,06023
Cloud_Mesh_Distance_BA_R0_R2	22,54456329	-79,19734192	-943,062561	-0,070597
Cloud_Mesh_Distance_BA_R0_R2	22,45625114	-79,06663513	-943,0913696	-0,059082
Cloud_Mesh_Distance_BA_R0_R2	22,49948883	-79,21134949	-943,199707	-0,078082
Cloud_Mesh_Distance_BA_R0_R2	15,43276882	-78,0350647	-943,2242432	-0,141908
Cloud_Mesh_Distance_BA_R0_R2	15,43313503	-78,21383667	-943,0540772	-0,145757
Cloud_Mesh_Distance_BA_R0_R2	22,40420532	-79,10752869	-943,2848511	-0,070369
Cloud_Mesh_Distance_BA_R0_R2	15,48653793	-77,82312012	-943,2948608	-0,126799
Cloud_Mesh_Distance_BA_R0_R2	22,27441406	-78,82749939	-943,1633301	-0,039946
Cloud_Mesh_Distance_BA_R0_R2	22,2595253	-78,9183197	-943,3499146	-0,050067
Cloud_Mesh_Distance_BA_R0_R2	21,88034058	-78,37145996	-943,2679443	-0,028394
Cloud_Mesh_Distance_BA_R0_R2	21,90098953	-78,42250061	-943,3151856	-0,029705
Cloud_Mesh_Distance_BA_R0_R2	22,0422287	-78,49684143	-943,1253662	-0,028486
Cloud_Mesh_Distance_BA_R0_R2	22,04662323	-78,55523682	-943,2267456	-0,02804
Cloud_Mesh_Distance_BA_R0_R2	15,56545067	-77,6164856	-943,3099976	-0,101362
Cloud_Mesh_Distance_BA_R0_R2	15,64883327	-77,43806458	-943,31427	-0,079595
Cloud_Mesh_Distance_BA_R0_R2	21,61959839	-78,11630249	-943,3921509	-0,023194
Cloud_Mesh_Distance_BA_R0_R2	21,69582367	-78,15814209	-943,2960205	-0,024254
Cloud_Mesh_Distance_BA_R0_R2	21,87211227	-78,3014679	-943,1569214	-0,025022
Cloud_Mesh_Distance_BA_R0_R2	21,52147675	-77,93537903	-943,2885132	-0,016402
Cloud_Mesh_Distance_BA_R0_R2	21,53636932	-77,99414063	-943,3626099	-0,018971
Cloud_Mesh_Distance_BA_R0_R2	21,58532906	-77,98905945	-943,2391357	-0,015623
Cloud_Mesh_Distance_BA_R0_R2	21,35720062	-77,77302551	-943,3719482	-0,011665
Cloud_Mesh_Distance_BA_R0_R2	21,40538406	-77,76679993	-943,2405396	-0,011385
Cloud_Mesh_Distance_BA_R0_R2	21,44648361	-77,8939209	-943,3875732	-0,016016
Cloud_Mesh_Distance_BA_R0_R2	21,51356506	-77,86543274	-943,1695557	-0,009909
Cloud_Mesh_Distance_BA_R0_R2	15,8655777	-77,13365173	-943,2666626	-0,0611
Cloud_Mesh_Distance_BA_R0_R2	15,93291092	-77,07862854	-943,2144775	-0,060928
Cloud_Mesh_Distance_BA_R0_R2	16,2032299	-76,79052734	-943,263916	-0,056191
Cloud_Mesh_Distance_BA_R0_R2	15,96278572	-76,98707581	-943,3479004	-0,065203
Cloud_Mesh_Distance_BA_R0_R2	16,22803497	-76,85263062	-943,0727539	-0,050358
Cloud_Mesh_Distance_BA_R0_R2	21,09367561	-77,49465942	-943,4819946	-0,004291
Cloud_Mesh_Distance_BA_R0_R2	21,17419052	-77,56195068	-943,4111939	-0,005711
Cloud_Mesh_Distance_BA_R0_R2	21,33950424	-77,71299744	-943,296936	-0,008828
Cloud_Mesh_Distance_BA_R0_R2	16,17295265	-76,76699829	-943,4030762	-0,065133
Cloud_Mesh_Distance_BA_R0_R2	16,09579277	-76,84417725	-943,3775024	-0,063602
Cloud_Mesh_Distance_BA_R0_R2	16,29741287	-76,76065063	-943,1437378	-0,052686
Cloud_Mesh_Distance_BA_R0_R2	21,03948975	-77,37004089	-943,3561401	0,000835
Cloud_Mesh_Distance_BA_R0_R2	21,21327019	-77,54606628	-943,2731323	-0,002131
Cloud_Mesh_Distance_BA_R0_R2	21,13315964	-77,4797821	-943,3448486	0,000432
Cloud_Mesh_Distance_BA_R0_R2	16,31323242	-76,6934967	-943,27948	-0,054268
Cloud_Mesh_Distance_BA_R0_R2	16,30621719	-76,64041138	-943,4661865	-0,061828
Cloud_Mesh_Distance_BA_R0_R2	16,23833466	-76,72016907	-943,3786011	-0,061032
Cloud_Mesh_Distance_BA_R0_R2	16,51802826	-76,60871887	-943,0956421	-0,035996
Cloud_Mesh_Distance_BA_R0_R2	20,71774292	-77,04908752	-943,4359131	0,001306
Cloud_Mesh_Distance_BA_R0_R2	20,81301117	-77,12954712	-943,3966065	0,009343
Cloud_Mesh_Distance_BA_R0_R2	20,83730125	-77,09977722	-943,2150879	0,007943
Cloud_Mesh_Distance_BA_R0_R2	20,93935966	-77,21266174	-943,2407227	0,009031
Cloud_Mesh_Distance_BA_R0_R2	16,61100388	-76,51322937	-943,1724243	-0,03755
Cloud_Mesh_Distance_BA_R0_R2	16,60772514	-76,44953918	-943,3823853	-0,044247

Cloud_Mesh_Distance_BA_R0_R2	20,52175903	-76,8533783	-943,3007813	-0,01511
Cloud_Mesh_Distance_BA_R0_R2	20,74369621	-77,029953	-943,2852173	0,000685
Cloud_Mesh_Distance_BA_R0_R2	16,79157257	-76,3583374	-943,3069458	-0,02798
Cloud_Mesh_Distance_BA_R0_R2	17,0478096	-76,2406311	-943,2745361	-0,010861
Cloud_Mesh_Distance_BA_R0_R2	16,96385956	-76,2555542	-943,3701172	-0,01998
Cloud_Mesh_Distance_BA_R0_R2	16,8560276	-76,34222412	-943,2376709	-0,020062
Cloud_Mesh_Distance_BA_R0_R2	20,13294029	-76,60250854	-943,3233032	-0,067235
Cloud_Mesh_Distance_BA_R0_R2	20,24112892	-76,67776489	-943,3709106	-0,056133
Cloud_Mesh_Distance_BA_R0_R2	20,39400864	-76,78913879	-943,4241333	-0,03558
Cloud_Mesh_Distance_BA_R0_R2	20,39271927	-76,74812317	-943,2102051	-0,033177
Cloud_Mesh_Distance_BA_R0_R2	17,19177437	-76,19821167	-943,2151489	-0,003482
Cloud_Mesh_Distance_BA_R0_R2	17,57040405	-76,0682373	-943,244812	0,019034
Cloud_Mesh_Distance_BA_R0_R2	17,72312164	-76,04223633	-943,1829224	0,031453
Cloud_Mesh_Distance_BA_R0_R2	19,63856697	-76,31408691	-943,3521118	-0,041581
Cloud_Mesh_Distance_BA_R0_R2	19,68522072	-76,33093262	-943,1890259	-0,041785
Cloud_Mesh_Distance_BA_R0_R2	19,82140923	-76,40492249	-943,1642456	-0,056658
Cloud_Mesh_Distance_BA_R0_R2	19,92289543	-76,49310303	-943,435791	-0,070627
Cloud_Mesh_Distance_BA_R0_R2	19,78426933	-76,39900208	-943,3540039	-0,063368
Cloud_Mesh_Distance_BA_R0_R2	20,08195305	-76,56227112	-943,2529907	-0,067007
Cloud_Mesh_Distance_BA_R0_R2	19,96179008	-76,48913574	-943,2264404	-0,064447
Cloud_Mesh_Distance_BA_R0_R2	17,82799911	-76,00469971	-943,2268066	0,044394
Cloud_Mesh_Distance_BA_R0_R2	17,67846489	-76,02488708	-943,3009033	0,031159
Cloud_Mesh_Distance_BA_R0_R2	18,10488892	-75,93327332	-943,3188477	0,079066
Cloud_Mesh_Distance_BA_R0_R2	17,86598587	-75,96505737	-943,3686523	0,056245
Cloud_Mesh_Distance_BA_R0_R2	18,31087494	-75,94247437	-943,1702881	0,084983
Cloud_Mesh_Distance_BA_R0_R2	18,23835182	-75,92901611	-943,2675171	0,085261
Cloud_Mesh_Distance_BA_R0_R2	19,29727554	-76,1341095	-943,4060669	0,015721
Cloud_Mesh_Distance_BA_R0_R2	19,49611664	-76,2401123	-943,4338379	-0,022419
Cloud_Mesh_Distance_BA_R0_R2	19,56869888	-76,27009583	-943,2640991	-0,029078
Cloud_Mesh_Distance_BA_R0_R2	19,64518356	-76,33753967	-943,5214233	-0,043736
Cloud_Mesh_Distance_BA_R0_R2	18,20053101	-75,90975952	-943,4315186	0,089925
Cloud_Mesh_Distance_BA_R0_R2	18,58646584	-75,91941833	-943,4650879	0,094678
Cloud_Mesh_Distance_BA_R0_R2	18,71467972	-75,94569397	-943,3218384	0,087317
Cloud_Mesh_Distance_BA_R0_R2	18,89863968	-75,98213196	-943,5018921	0,073982
Cloud_Mesh_Distance_BA_R0_R2	19,16568184	-76,07476807	-943,3501587	0,036899
Cloud_Mesh_Distance_BA_R0_R2	17,66485977	-83,71546936	-942,5889893	-0,018279
Cloud_Mesh_Distance_BA_R0_R2	17,80094528	-83,83372498	-942,5670166	-0,009678
Cloud_Mesh_Distance_BA_R0_R2	17,61942291	-83,69145203	-942,767334	-0,087816
Cloud_Mesh_Distance_BA_R0_R2	17,53482819	-83,61779785	-942,8623657	-0,024631
Cloud_Mesh_Distance_BA_R0_R2	17,52754593	-83,59611511	-942,6708984	-0,024949
Cloud_Mesh_Distance_BA_R0_R2	17,33916092	-83,39801025	-942,6137695	-0,037042
Cloud_Mesh_Distance_BA_R0_R2	17,00025749	-83,00804138	-942,7329102	-0,054913
Cloud_Mesh_Distance_BA_R0_R2	17,03886986	-83,07218933	-942,8025513	-0,045342
Cloud_Mesh_Distance_BA_R0_R2	17,1117382	-83,16314697	-942,7971191	-0,040623
Cloud_Mesh_Distance_BA_R0_R2	17,01053238	-82,98716736	-942,5822144	-0,066548
Cloud_Mesh_Distance_BA_R0_R2	16,96819687	-82,99121094	-942,8857422	-0,044089
Cloud_Mesh_Distance_BA_R0_R2	16,8200798	-82,7635498	-942,8059082	-0,06655
Cloud_Mesh_Distance_BA_R0_R2	16,7552681	-82,63095093	-942,6904297	-0,084573
Cloud_Mesh_Distance_BA_R0_R2	16,59658051	-82,38241577	-942,6973267	-0,084225
Cloud_Mesh_Distance_BA_R0_R2	16,58304405	-82,43489075	-942,9171143	-0,064011

Cloud_Mesh_Distance_BA_R0_R2	16,44638634	-82,16487122	-942,7603149	-0,077507
Cloud_Mesh_Distance_BA_R0_R2	16,51473999	-82,30322266	-942,8386841	-0,075726
Cloud_Mesh_Distance_BA_R0_R2	16,52276039	-82,26028442	-942,6866455	-0,081518
Cloud_Mesh_Distance_BA_R0_R2	16,44449806	-82,22659302	-942,9365845	-0,062946
Cloud_Mesh_Distance_BA_R0_R2	16,36723137	-82,10359192	-942,939148	-0,067028
Cloud_Mesh_Distance_BA_R0_R2	16,23610306	-81,76994324	-942,6799316	-0,076034
Cloud_Mesh_Distance_BA_R0_R2	16,22983551	-81,82833862	-942,8295288	-0,074316
Cloud_Mesh_Distance_BA_R0_R2	16,36715698	-82,03401184	-942,7610474	-0,072869
Cloud_Mesh_Distance_BA_R0_R2	16,3092308	-81,89222717	-942,6646729	-0,07724
Cloud_Mesh_Distance_BA_R0_R2	16,12654305	-81,61291504	-942,7587891	-0,082082
Cloud_Mesh_Distance_BA_R0_R2	16,18465233	-81,72138977	-942,769043	-0,077388
Cloud_Mesh_Distance_BA_R0_R2	23,39758492	-82,54852295	-942,9680786	0,306676
Cloud_Mesh_Distance_BA_R0_R2	23,39137077	-82,51789856	-942,7589722	0,318796
Cloud_Mesh_Distance_BA_R0_R2	16,05206299	-81,46853638	-942,7368164	-0,083785
Cloud_Mesh_Distance_BA_R0_R2	23,28964233	-82,28317261	-942,7978516	0,256131
Cloud_Mesh_Distance_BA_R0_R2	15,84030342	-81,16194153	-942,8161011	-0,080162
Cloud_Mesh_Distance_BA_R0_R2	15,84917736	-81,22210693	-942,8982544	-0,084822
Cloud_Mesh_Distance_BA_R0_R2	15,98588085	-81,3223114	-942,6867676	-0,080798
Cloud_Mesh_Distance_BA_R0_R2	15,9918375	-81,40266418	-942,8074341	-0,084485
Cloud_Mesh_Distance_BA_R0_R2	23,19945717	-82,11109924	-942,9719238	0,174208
Cloud_Mesh_Distance_BA_R0_R2	15,61409378	-80,8862915	-942,9555664	-0,071805
Cloud_Mesh_Distance_BA_R0_R2	15,67739296	-80,92112732	-942,8527832	-0,071947
Cloud_Mesh_Distance_BA_R0_R2	15,76967621	-81,10803223	-942,9124756	-0,080596
Cloud_Mesh_Distance_BA_R0_R2	23,17013931	-82,01365662	-942,8790283	0,130006
Cloud_Mesh_Distance_BA_R0_R2	23,11874771	-81,86927795	-942,8648071	0,062526
Cloud_Mesh_Distance_BA_R0_R2	23,13920975	-81,99829102	-943,0449829	0,10801
Cloud_Mesh_Distance_BA_R0_R2	15,53625298	-80,68603516	-942,8912354	-0,067043
Cloud_Mesh_Distance_BA_R0_R2	15,55020618	-80,76725769	-942,949646	-0,070754
Cloud_Mesh_Distance_BA_R0_R2	15,59013367	-80,75393677	-942,8446045	-0,068636
Cloud_Mesh_Distance_BA_R0_R2	23,08490372	-81,70359802	-942,7599487	-0,007587
Cloud_Mesh_Distance_BA_R0_R2	23,04575729	-81,5677948	-942,8623047	-0,050347
Cloud_Mesh_Distance_BA_R0_R2	23,06425095	-81,73123169	-942,9523926	0,002535
Cloud_Mesh_Distance_BA_R0_R2	15,49247456	-80,68457031	-942,9907837	-0,071328
Cloud_Mesh_Distance_BA_R0_R2	15,42313385	-80,56582642	-943,0339356	-0,080232
Cloud_Mesh_Distance_BA_R0_R2	15,49160576	-80,5589447	-942,8743286	-0,07357
Cloud_Mesh_Distance_BA_R0_R2	23,00145912	-81,4400177	-943,0015259	-0,09197
Cloud_Mesh_Distance_BA_R0_R2	23,04289246	-81,38594055	-942,7805786	-0,096159
Cloud_Mesh_Distance_BA_R0_R2	15,3698597	-80,246521	-942,9317017	-0,107033
Cloud_Mesh_Distance_BA_R0_R2	15,4903059	-80,3815155	-942,7654419	-0,095636
Cloud_Mesh_Distance_BA_R0_R2	15,36717701	-80,09443665	-942,8770752	-0,119499
Cloud_Mesh_Distance_BA_R0_R2	15,37003708	-79,92985535	-942,8269653	-0,136156
Cloud_Mesh_Distance_BA_R0_R2	15,2692728	-79,7641449	-942,9978638	-0,151414
Cloud_Mesh_Distance_BA_R0_R2	22,95450592	-80,63165283	-943,1485596	-0,147562
Cloud_Mesh_Distance_BA_R0_R2	22,99540329	-80,74987793	-943,0296021	-0,149154
Cloud_Mesh_Distance_BA_R0_R2	22,99404907	-80,60620117	-943,0368042	-0,142973
Cloud_Mesh_Distance_BA_R0_R2	15,34069729	-79,75482178	-942,8502197	-0,144694
Cloud_Mesh_Distance_BA_R0_R2	15,36567593	-79,5447998	-942,7800293	-0,154931
Cloud_Mesh_Distance_BA_R0_R2	22,97105789	-80,51686096	-943,0946655	-0,145329
Cloud_Mesh_Distance_BA_R0_R2	23,00946999	-80,49542236	-942,9829102	-0,13787
Cloud_Mesh_Distance_BA_R0_R2	23,03769493	-80,64353943	-942,9042969	-0,138327

Cloud_Mesh_Distance_BA_R0_R2	23,06222534	-80,45704651	-942,8103027	-0,130793
Cloud_Mesh_Distance_BA_R0_R2	15,21735191	-79,33982849	-943,1077881	-0,177629
Cloud_Mesh_Distance_BA_R0_R2	15,28190041	-79,26153564	-942,9611816	-0,171821
Cloud_Mesh_Distance_BA_R0_R2	15,32426834	-79,39631653	-942,8598633	-0,163152
Cloud_Mesh_Distance_BA_R0_R2	22,92395401	-80,06159973	-943,0603638	-0,11322
Cloud_Mesh_Distance_BA_R0_R2	22,9976387	-80,16046143	-942,909668	-0,116502
Cloud_Mesh_Distance_BA_R0_R2	15,30329704	-79,07096863	-942,9415283	-0,170569
Cloud_Mesh_Distance_BA_R0_R2	15,23977852	-79,12806702	-943,0856323	-0,178757
Cloud_Mesh_Distance_BA_R0_R2	15,2628212	-78,93037415	-943,0831299	-0,176852
Cloud_Mesh_Distance_BA_R0_R2	22,8830452	-79,90382385	-943,0534058	-0,11018
Cloud_Mesh_Distance_BA_R0_R2	22,93558121	-79,88841248	-942,8942261	-0,098281
Cloud_Mesh_Distance_BA_R0_R2	22,96863937	-80,02424622	-942,9088745	-0,107587
Cloud_Mesh_Distance_BA_R0_R2	15,32749557	-78,87776184	-942,9378662	-0,17014
Cloud_Mesh_Distance_BA_R0_R2	15,355196	-78,6897583	-942,9470825	-0,15994
Cloud_Mesh_Distance_BA_R0_R2	22,81120682	-79,8134613	-943,1518555	-0,111344
Cloud_Mesh_Distance_BA_R0_R2	22,87158966	-79,79949951	-942,9884644	-0,099888
Cloud_Mesh_Distance_BA_R0_R2	22,88966942	-79,74423218	-942,8778076	-0,082905
Cloud_Mesh_Distance_BA_R0_R2	15,327672	-78,55256653	-943,0952759	-0,17058
Cloud_Mesh_Distance_BA_R0_R2	22,75154114	-79,63145447	-943,1148071	-0,104295
Cloud_Mesh_Distance_BA_R0_R2	15,38334942	-78,51535034	-942,9697266	-0,157101
Cloud_Mesh_Distance_BA_R0_R2	15,44844818	-78,41244507	-942,8740234	-0,145233
Cloud_Mesh_Distance_BA_R0_R2	22,68333626	-79,41362	-943,0146484	-0,082931
Cloud_Mesh_Distance_BA_R0_R2	22,71320915	-79,37825012	-942,8724365	-0,073062
Cloud_Mesh_Distance_BA_R0_R2	22,6274395	-79,38978577	-943,125061	-0,091079
Cloud_Mesh_Distance_BA_R0_R2	22,62473679	-79,28860474	-942,9859619	-0,071485
Cloud_Mesh_Distance_BA_R0_R2	15,36088848	-78,42416382	-943,0916748	-0,160001
Cloud_Mesh_Distance_BA_R0_R2	15,39938927	-78,37434387	-943,022644	-0,152257
Cloud_Mesh_Distance_BA_R0_R2	15,5116024	-78,19618225	-942,8685913	-0,128008
Cloud_Mesh_Distance_BA_R0_R2	22,35416985	-78,95802307	-943,1826172	-0,050464
Cloud_Mesh_Distance_BA_R0_R2	15,49257851	-78,00791931	-943,0723267	-0,132133
Cloud_Mesh_Distance_BA_R0_R2	22,38226318	-78,89804077	-942,9899902	-0,041221
Cloud_Mesh_Distance_BA_R0_R2	15,6037302	-77,85881042	-942,9233398	-0,106706
Cloud_Mesh_Distance_BA_R0_R2	15,55823326	-77,98753357	-942,920105	-0,115738
Cloud_Mesh_Distance_BA_R0_R2	15,63937759	-77,71511841	-942,9751587	-0,091185
Cloud_Mesh_Distance_BA_R0_R2	22,09564972	-78,58747864	-943,1692505	-0,030623
Cloud_Mesh_Distance_BA_R0_R2	22,17970848	-78,68267822	-943,1384888	-0,032922
Cloud_Mesh_Distance_BA_R0_R2	15,56175423	-77,7973938	-943,0906372	-0,10668
Cloud_Mesh_Distance_BA_R0_R2	15,65910339	-77,83837891	-942,8173828	-0,091166
Cloud_Mesh_Distance_BA_R0_R2	15,71642494	-77,66867065	-942,8496094	-0,077081
Cloud_Mesh_Distance_BA_R0_R2	21,94318199	-78,41178894	-943,1999512	-0,026108
Cloud_Mesh_Distance_BA_R0_R2	21,98497772	-78,37538147	-943,0223999	-0,019503
Cloud_Mesh_Distance_BA_R0_R2	15,69502449	-77,55155945	-943,0222168	-0,077815
Cloud_Mesh_Distance_BA_R0_R2	15,73952675	-77,42019653	-943,086731	-0,06894
Cloud_Mesh_Distance_BA_R0_R2	15,68362045	-77,46748352	-943,1629028	-0,078334
Cloud_Mesh_Distance_BA_R0_R2	15,62700272	-77,59344482	-943,1468506	-0,089485
Cloud_Mesh_Distance_BA_R0_R2	21,80149651	-78,15731812	-943,0418091	-0,011534
Cloud_Mesh_Distance_BA_R0_R2	21,76351166	-78,21138	-943,2407227	-0,026347
Cloud_Mesh_Distance_BA_R0_R2	15,72519302	-77,35290527	-943,2268677	-0,071826
Cloud_Mesh_Distance_BA_R0_R2	15,82272911	-77,28048706	-943,100769	-0,060911
Cloud_Mesh_Distance_BA_R0_R2	21,68920517	-78,08444214	-943,1742554	-0,018841

Cloud_Mesh_Distance_BA_R0_R2	21,72648048	-78,02520752	-942,9533691	-0,003491
Cloud_Mesh_Distance_BA_R0_R2	15,8751421	-77,32977295	-942,9196777	-0,054638
Cloud_Mesh_Distance_BA_R0_R2	15,91142654	-77,14749146	-943,1275635	-0,058007
Cloud_Mesh_Distance_BA_R0_R2	16,1029129	-76,91481018	-943,1843262	-0,059836
Cloud_Mesh_Distance_BA_R0_R2	16,00530624	-77,0554657	-943,0993042	-0,055737
Cloud_Mesh_Distance_BA_R0_R2	21,32296753	-77,63912964	-943,1821899	-0,004888
Cloud_Mesh_Distance_BA_R0_R2	21,44014931	-77,72145081	-943,0464478	0,00189
Cloud_Mesh_Distance_BA_R0_R2	16,27325821	-76,88731384	-942,9281616	-0,043202
Cloud_Mesh_Distance_BA_R0_R2	16,35716629	-76,79188538	-942,9719849	-0,043117
Cloud_Mesh_Distance_BA_R0_R2	16,3997097	-76,6849823	-943,1276856	-0,045721
Cloud_Mesh_Distance_BA_R0_R2	21,00394821	-77,22921753	-943,062439	0,00684
Cloud_Mesh_Distance_BA_R0_R2	21,13973618	-77,43261719	-943,2131348	0,003348
Cloud_Mesh_Distance_BA_R0_R2	21,06288338	-77,32133484	-943,1531372	0,00525
Cloud_Mesh_Distance_BA_R0_R2	21,14964676	-77,39186096	-943,071106	0,002354
Cloud_Mesh_Distance_BA_R0_R2	21,23245621	-77,49615479	-943,0952759	0,00164
Cloud_Mesh_Distance_BA_R0_R2	16,56298065	-76,63040161	-942,9714966	-0,03039
Cloud_Mesh_Distance_BA_R0_R2	20,89653969	-77,11309814	-943,0299683	0,008311
Cloud_Mesh_Distance_BA_R0_R2	16,61685753	-76,56767273	-943,0256348	-0,029182
Cloud_Mesh_Distance_BA_R0_R2	16,74234772	-76,41137695	-943,223877	-0,027346
Cloud_Mesh_Distance_BA_R0_R2	20,52824974	-76,82780457	-943,1303101	-0,014516
Cloud_Mesh_Distance_BA_R0_R2	20,59099007	-76,85041809	-942,9608154	-0,01297
Cloud_Mesh_Distance_BA_R0_R2	20,69215584	-76,92633057	-942,9716187	0,000131
Cloud_Mesh_Distance_BA_R0_R2	20,66294861	-76,93087769	-943,1439209	-0,000666
Cloud_Mesh_Distance_BA_R0_R2	20,78603745	-77,03144836	-943,1286621	0,004855
Cloud_Mesh_Distance_BA_R0_R2	16,95043945	-76,32225037	-943,1442261	-0,012298
Cloud_Mesh_Distance_BA_R0_R2	16,95388794	-76,28697205	-943,2592773	-0,019395
Cloud_Mesh_Distance_BA_R0_R2	17,00432014	-76,34465027	-943,0048828	-0,004303
Cloud_Mesh_Distance_BA_R0_R2	17,07292938	-76,27114868	-943,1263428	-0,002362
Cloud_Mesh_Distance_BA_R0_R2	20,18433762	-76,6164856	-943,1972656	-0,057413
Cloud_Mesh_Distance_BA_R0_R2	20,29957581	-76,66760254	-943,010437	-0,040222
Cloud_Mesh_Distance_BA_R0_R2	20,28170967	-76,67567444	-943,1973877	-0,051563
Cloud_Mesh_Distance_BA_R0_R2	17,37830544	-76,12954712	-943,2330933	0,007504
Cloud_Mesh_Distance_BA_R0_R2	17,56361771	-76,12879944	-943,0360107	0,012884
Cloud_Mesh_Distance_BA_R0_R2	17,72113991	-76,07858276	-943,0567627	0,021198
Cloud_Mesh_Distance_BA_R0_R2	17,87657166	-76,07395935	-942,9481201	0,029465
Cloud_Mesh_Distance_BA_R0_R2	20,08562088	-76,55407715	-943,1550903	-0,063223
Cloud_Mesh_Distance_BA_R0_R2	17,82606316	-76,0428772	-943,0877075	0,034736
Cloud_Mesh_Distance_BA_R0_R2	18,0174427	-75,96765137	-943,2137451	0,063209
Cloud_Mesh_Distance_BA_R0_R2	18,1789875	-75,99111938	-943,0262451	0,062819
Cloud_Mesh_Distance_BA_R0_R2	18,19622993	-75,95127869	-943,1732788	0,078934
Cloud_Mesh_Distance_BA_R0_R2	18,32607651	-75,96792603	-943,0533447	0,077597
Cloud_Mesh_Distance_BA_R0_R2	18,47432518	-75,97810364	-942,991394	0,077626
Cloud_Mesh_Distance_BA_R0_R2	18,40739059	-75,95080566	-943,1139526	0,082999
Cloud_Mesh_Distance_BA_R0_R2	18,92757797	-76,01625061	-943,0893555	0,06403
Cloud_Mesh_Distance_BA_R0_R2	19,04106331	-76,0410614	-943,1484375	0,05567
Cloud_Mesh_Distance_BA_R0_R2	19,2144146	-76,1022644	-943,1567383	0,031829
Cloud_Mesh_Distance_BA_R0_R2	19,36321259	-76,17791748	-942,9998169	0,002622
Cloud_Mesh_Distance_BA_R0_R2	19,51424408	-76,24557495	-943,0192871	-0,018206
Cloud_Mesh_Distance_BA_R0_R2	19,43741608	-76,20117188	-943,2023926	-0,005776
Cloud_Mesh_Distance_BA_R0_R2	19,57056236	-76,26924133	-943,144043	-0,028667

Cloud_Mesh_Distance_BA_R0_R2	18,3806839	-75,92546082	-943,2581177	0,090794
Cloud_Mesh_Distance_BA_R0_R2	18,53550339	-75,95274353	-943,1155396	0,088449
Cloud_Mesh_Distance_BA_R0_R2	18,56294823	-75,93017578	-943,28302	0,093837
Cloud_Mesh_Distance_BA_R0_R2	18,90643692	-75,98963928	-943,2924805	0,073956
Cloud_Mesh_Distance_BA_R0_R2	19,07975769	-76,04420471	-943,2750244	0,051857
Cloud_Mesh_Distance_BA_R0_R2	17,5743885	-83,61940002	-942,501709	-0,024583
Cloud_Mesh_Distance_BA_R0_R2	17,7514286	-83,76554871	-942,3954468	-0,018621
Cloud_Mesh_Distance_BA_R0_R2	17,87325668	-83,87286377	-942,4060059	-0,008135
Cloud_Mesh_Distance_BA_R0_R2	17,42102242	-83,49104309	-942,6712036	-0,029172
Cloud_Mesh_Distance_BA_R0_R2	17,15733719	-83,1945343	-942,6386719	-0,048215
Cloud_Mesh_Distance_BA_R0_R2	17,2471199	-83,28741455	-942,5639038	-0,046494
Cloud_Mesh_Distance_BA_R0_R2	17,33506393	-83,35528564	-942,4069214	-0,047011
Cloud_Mesh_Distance_BA_R0_R2	17,4593029	-83,50413513	-942,4904175	-0,032951
Cloud_Mesh_Distance_BA_R0_R2	17,18706894	-83,20336914	-942,5040283	-0,052151
Cloud_Mesh_Distance_BA_R0_R2	17,10858917	-83,10165405	-942,5018921	-0,06117
Cloud_Mesh_Distance_BA_R0_R2	17,0694313	-83,0900116	-942,6766968	-0,054443
Cloud_Mesh_Distance_BA_R0_R2	16,92059326	-82,88975525	-942,7110596	-0,068261
Cloud_Mesh_Distance_BA_R0_R2	16,84497643	-82,74009705	-942,5932007	-0,085132
Cloud_Mesh_Distance_BA_R0_R2	16,69061089	-82,49464417	-942,5950317	-0,093153
Cloud_Mesh_Distance_BA_R0_R2	16,56332016	-82,26649475	-942,5440674	-0,092201
Cloud_Mesh_Distance_BA_R0_R2	16,41993713	-82,03552246	-942,5632935	-0,082677
Cloud_Mesh_Distance_BA_R0_R2	16,49368286	-82,17481995	-942,5964966	-0,087478
Cloud_Mesh_Distance_BA_R0_R2	16,19260406	-81,66293335	-942,6275635	-0,080087
Cloud_Mesh_Distance_BA_R0_R2	23,33213043	-82,38523865	-942,6943359	0,291376
Cloud_Mesh_Distance_BA_R0_R2	23,31656265	-82,38296509	-942,5435791	0,292548
Cloud_Mesh_Distance_BA_R0_R2	15,89831734	-81,2197113	-942,7567749	-0,079996
Cloud_Mesh_Distance_BA_R0_R2	15,88694	-81,0067749	-942,5098877	-0,07218
Cloud_Mesh_Distance_BA_R0_R2	15,84124184	-81,08006287	-942,6934204	-0,070713
Cloud_Mesh_Distance_BA_R0_R2	23,1215744	-81,82550049	-942,6679688	0,042783
Cloud_Mesh_Distance_BA_R0_R2	23,22503662	-82,13386536	-942,796875	0,190485
Cloud_Mesh_Distance_BA_R0_R2	23,16431427	-81,96986389	-942,72229	0,105748
Cloud_Mesh_Distance_BA_R0_R2	23,14799881	-81,92533875	-942,5396729	0,075977
Cloud_Mesh_Distance_BA_R0_R2	15,66077423	-80,83694458	-942,786438	-0,070875
Cloud_Mesh_Distance_BA_R0_R2	15,72648907	-80,79582214	-942,6168213	-0,070402
Cloud_Mesh_Distance_BA_R0_R2	15,73791409	-80,95587158	-942,7610474	-0,071798
Cloud_Mesh_Distance_BA_R0_R2	15,55999279	-80,64324951	-942,8032227	-0,06942
Cloud_Mesh_Distance_BA_R0_R2	15,63063335	-80,67198181	-942,6915283	-0,07517
Cloud_Mesh_Distance_BA_R0_R2	15,55837154	-80,52333069	-942,7214966	-0,081694
Cloud_Mesh_Distance_BA_R0_R2	15,63087845	-80,49575806	-942,5784912	-0,086196
Cloud_Mesh_Distance_BA_R0_R2	15,70225716	-80,44229126	-942,4331055	-0,082366
Cloud_Mesh_Distance_BA_R0_R2	15,56508732	-80,36578369	-942,6270142	-0,093857
Cloud_Mesh_Distance_BA_R0_R2	23,03223038	-81,22956848	-942,8322144	-0,122608
Cloud_Mesh_Distance_BA_R0_R2	23,04613876	-81,10780334	-942,7965698	-0,132065
Cloud_Mesh_Distance_BA_R0_R2	15,46068382	-80,00392151	-942,6826782	-0,127839
Cloud_Mesh_Distance_BA_R0_R2	15,4305172	-80,22302246	-942,8047485	-0,111352
Cloud_Mesh_Distance_BA_R0_R2	23,09219551	-80,93962097	-942,6629639	-0,134532
Cloud_Mesh_Distance_BA_R0_R2	23,10035133	-80,8367157	-942,6618042	-0,136263
Cloud_Mesh_Distance_BA_R0_R2	15,41202545	-79,82580566	-942,7293701	-0,141548
Cloud_Mesh_Distance_BA_R0_R2	15,52746105	-80,00396729	-942,5728149	-0,12288
Cloud_Mesh_Distance_BA_R0_R2	23,05056381	-80,88987732	-942,8328857	-0,141532

Cloud_Mesh_Distance_BA_R0_R2	23,0957737	-80,67097473	-942,7056885	-0,133514
Cloud_Mesh_Distance_BA_R0_R2	15,43106174	-79,51237488	-942,6582031	-0,144698
Cloud_Mesh_Distance_BA_R0_R2	15,4193058	-79,66461182	-942,6919556	-0,146898
Cloud_Mesh_Distance_BA_R0_R2	15,49946404	-79,57328796	-942,5466309	-0,133437
Cloud_Mesh_Distance_BA_R0_R2	15,47336578	-79,42150879	-942,5802612	-0,136176
Cloud_Mesh_Distance_BA_R0_R2	23,04453468	-80,28005981	-942,8186035	-0,117851
Cloud_Mesh_Distance_BA_R0_R2	23,11223984	-80,49162292	-942,6366577	-0,125535
Cloud_Mesh_Distance_BA_R0_R2	15,40616989	-79,35688782	-942,6990356	-0,147434
Cloud_Mesh_Distance_BA_R0_R2	15,34988785	-79,20819092	-942,8170166	-0,160609
Cloud_Mesh_Distance_BA_R0_R2	23,04462624	-80,16345215	-942,7579346	-0,109038
Cloud_Mesh_Distance_BA_R0_R2	15,42683983	-79,14402771	-942,6708374	-0,143474
Cloud_Mesh_Distance_BA_R0_R2	15,44712448	-78,94639587	-942,6634522	-0,14011
Cloud_Mesh_Distance_BA_R0_R2	15,37235832	-79,01278687	-942,800354	-0,156381
Cloud_Mesh_Distance_BA_R0_R2	22,82608414	-79,66113281	-942,9589233	-0,089973
Cloud_Mesh_Distance_BA_R0_R2	22,87078285	-79,64552307	-942,8048706	-0,072974
Cloud_Mesh_Distance_BA_R0_R2	22,93297005	-79,74494934	-942,7352295	-0,071776
Cloud_Mesh_Distance_BA_R0_R2	15,39560699	-78,82022095	-942,8016968	-0,150613
Cloud_Mesh_Distance_BA_R0_R2	15,46747589	-78,75498962	-942,6747437	-0,135055
Cloud_Mesh_Distance_BA_R0_R2	15,42076111	-78,6282196	-942,8220215	-0,145004
Cloud_Mesh_Distance_BA_R0_R2	22,74889183	-79,51342773	-942,9780884	-0,085699
Cloud_Mesh_Distance_BA_R0_R2	15,56540871	-78,25619507	-942,7120972	-0,115515
Cloud_Mesh_Distance_BA_R0_R2	22,48488808	-79,00108337	-942,8717651	-0,051102
Cloud_Mesh_Distance_BA_R0_R2	22,43331146	-78,897995	-942,8153687	-0,044658
Cloud_Mesh_Distance_BA_R0_R2	22,65095901	-79,22914124	-942,7873535	-0,064162
Cloud_Mesh_Distance_BA_R0_R2	22,57057381	-79,06513977	-942,697876	-0,050596
Cloud_Mesh_Distance_BA_R0_R2	22,55885887	-79,13938904	-942,9160156	-0,061476
Cloud_Mesh_Distance_BA_R0_R2	15,58747482	-78,10765076	-942,762207	-0,113374
Cloud_Mesh_Distance_BA_R0_R2	22,48910332	-78,94725037	-942,7213135	-0,044235
Cloud_Mesh_Distance_BA_R0_R2	15,61306	-77,96141052	-942,8151856	-0,102684
Cloud_Mesh_Distance_BA_R0_R2	15,66844749	-78,01707458	-942,6629639	-0,094626
Cloud_Mesh_Distance_BA_R0_R2	22,21311951	-78,64875793	-942,973999	-0,028392
Cloud_Mesh_Distance_BA_R0_R2	22,13745117	-78,57658386	-943,0369873	-0,027632
Cloud_Mesh_Distance_BA_R0_R2	22,18518639	-78,57737732	-942,8964233	-0,022748
Cloud_Mesh_Distance_BA_R0_R2	22,27072906	-78,66026306	-942,8119507	-0,02641
Cloud_Mesh_Distance_BA_R0_R2	22,28357315	-78,75082397	-942,9833374	-0,033054
Cloud_Mesh_Distance_BA_R0_R2	15,73660469	-77,82000732	-942,6799316	-0,078854
Cloud_Mesh_Distance_BA_R0_R2	22,10110474	-78,47164917	-942,9093628	-0,015297
Cloud_Mesh_Distance_BA_R0_R2	22,19499016	-78,53326416	-942,7514648	-0,014828
Cloud_Mesh_Distance_BA_R0_R2	15,7947197	-77,46925354	-942,9035034	-0,065191
Cloud_Mesh_Distance_BA_R0_R2	21,91621399	-78,24346924	-942,9209595	-0,007411
Cloud_Mesh_Distance_BA_R0_R2	21,96619225	-78,21246338	-942,6832886	0,002464
Cloud_Mesh_Distance_BA_R0_R2	22,02655602	-78,33804321	-942,8170166	-0,00689
Cloud_Mesh_Distance_BA_R0_R2	15,89634418	-77,39840698	-942,7954102	-0,051145
Cloud_Mesh_Distance_BA_R0_R2	15,92984581	-77,31132507	-942,8445435	-0,050608
Cloud_Mesh_Distance_BA_R0_R2	21,62379265	-77,94602966	-943,0549316	-0,007905
Cloud_Mesh_Distance_BA_R0_R2	21,66059875	-77,90783691	-942,8595581	0,003092
Cloud_Mesh_Distance_BA_R0_R2	16,0743866	-77,16789246	-942,7962036	-0,043566
Cloud_Mesh_Distance_BA_R0_R2	16,05855942	-77,09790039	-942,9264526	-0,048265
Cloud_Mesh_Distance_BA_R0_R2	15,95430183	-77,20869446	-942,9423218	-0,050741
Cloud_Mesh_Distance_BA_R0_R2	21,55668831	-77,817276	-942,9423218	0,003506

Cloud_Mesh_Distance_BA_R0_R2	21,48044014	-77,68533325	-942,8238525	0,007962
Cloud_Mesh_Distance_BA_R0_R2	21,60619736	-77,79434204	-942,7132568	0,013456
Cloud_Mesh_Distance_BA_R0_R2	16,12094116	-77,08499146	-942,8439941	-0,043342
Cloud_Mesh_Distance_BA_R0_R2	16,15726852	-76,98747253	-942,9425659	-0,04876
Cloud_Mesh_Distance_BA_R0_R2	21,21094894	-77,41049194	-942,9129028	0,010854
Cloud_Mesh_Distance_BA_R0_R2	21,34589195	-77,56771851	-942,9307861	0,005339
Cloud_Mesh_Distance_BA_R0_R2	21,30508804	-77,46514893	-942,7371826	0,01701
Cloud_Mesh_Distance_BA_R0_R2	16,4768486	-76,75883484	-942,8485718	-0,031893
Cloud_Mesh_Distance_BA_R0_R2	16,47584915	-76,69117737	-942,982605	-0,036935
Cloud_Mesh_Distance_BA_R0_R2	16,39788246	-76,84838867	-942,8048096	-0,035072
Cloud_Mesh_Distance_BA_R0_R2	16,33168411	-76,86753845	-942,8721314	-0,04151
Cloud_Mesh_Distance_BA_R0_R2	16,55239296	-76,68235779	-942,8820801	-0,031949
Cloud_Mesh_Distance_BA_R0_R2	21,09614944	-77,30691528	-942,9880981	0,0106
Cloud_Mesh_Distance_BA_R0_R2	21,20908165	-77,3669281	-942,7529907	0,018093
Cloud_Mesh_Distance_BA_R0_R2	21,1240387	-77,29508972	-942,8270874	0,017834
Cloud_Mesh_Distance_BA_R0_R2	16,65807343	-76,60865784	-942,875	-0,02481
Cloud_Mesh_Distance_BA_R0_R2	16,74318886	-76,64468384	-942,6939087	-0,017561
Cloud_Mesh_Distance_BA_R0_R2	21,01801491	-77,20581055	-942,9099731	0,013968
Cloud_Mesh_Distance_BA_R0_R2	21,04909325	-77,20599365	-942,7554932	0,017149
Cloud_Mesh_Distance_BA_R0_R2	16,86633873	-76,38562012	-943,0882568	-0,013813
Cloud_Mesh_Distance_BA_R0_R2	16,7423172	-76,48904419	-943,0136108	-0,019531
Cloud_Mesh_Distance_BA_R0_R2	16,91043854	-76,43441772	-942,9061279	-0,009915
Cloud_Mesh_Distance_BA_R0_R2	20,72486877	-76,93304443	-942,8070068	0,00131
Cloud_Mesh_Distance_BA_R0_R2	20,85926247	-77,05653381	-942,9140015	0,012757
Cloud_Mesh_Distance_BA_R0_R2	20,78088188	-76,99880981	-942,9765625	0,008131
Cloud_Mesh_Distance_BA_R0_R2	17,29670906	-76,26089478	-942,901062	0,00419
Cloud_Mesh_Distance_BA_R0_R2	17,15093231	-76,30014038	-942,9485474	0,002035
Cloud_Mesh_Distance_BA_R0_R2	17,23666954	-76,32650757	-942,7987671	0,00065
Cloud_Mesh_Distance_BA_R0_R2	17,08164787	-76,3853302	-942,8197022	-0,00233
Cloud_Mesh_Distance_BA_R0_R2	20,15572357	-76,58221436	-942,9505005	-0,050901
Cloud_Mesh_Distance_BA_R0_R2	20,20266724	-76,60704041	-942,7820435	-0,042889
Cloud_Mesh_Distance_BA_R0_R2	20,15441132	-76,58691406	-943,0683594	-0,054505
Cloud_Mesh_Distance_BA_R0_R2	20,46603012	-76,7603302	-942,8788452	-0,019577
Cloud_Mesh_Distance_BA_R0_R2	20,4585495	-76,76856995	-943,0377808	-0,02818
Cloud_Mesh_Distance_BA_R0_R2	17,21731377	-76,24058533	-943,0405884	0,00314
Cloud_Mesh_Distance_BA_R0_R2	17,42271614	-76,23745728	-942,8493042	0,004448
Cloud_Mesh_Distance_BA_R0_R2	17,37617111	-76,19351196	-943,0131836	0,004319
Cloud_Mesh_Distance_BA_R0_R2	17,49210548	-76,25718689	-942,7449341	0,006699
Cloud_Mesh_Distance_BA_R0_R2	17,77291107	-76,13421631	-942,8494873	0,017999
Cloud_Mesh_Distance_BA_R0_R2	19,72542572	-76,37019348	-942,7502441	-0,037971
Cloud_Mesh_Distance_BA_R0_R2	19,90298843	-76,44602966	-943,0552368	-0,056972
Cloud_Mesh_Distance_BA_R0_R2	19,76006889	-76,37023926	-942,9948731	-0,044288
Cloud_Mesh_Distance_BA_R0_R2	20,07400322	-76,53721619	-942,8656006	-0,049485
Cloud_Mesh_Distance_BA_R0_R2	20,03873253	-76,51966858	-943,0265503	-0,055744
Cloud_Mesh_Distance_BA_R0_R2	17,98573685	-76,02436829	-943,0274048	0,045619
Cloud_Mesh_Distance_BA_R0_R2	18,16804504	-76,05104065	-942,838501	0,050881
Cloud_Mesh_Distance_BA_R0_R2	18,37867355	-76,01628113	-942,8703003	0,070512
Cloud_Mesh_Distance_BA_R0_R2	18,75987434	-76,01359558	-942,9281616	0,075898
Cloud_Mesh_Distance_BA_R0_R2	18,87195396	-76,02441406	-942,9677124	0,066561
Cloud_Mesh_Distance_BA_R0_R2	18,85739708	-76,06359863	-942,7835693	0,059867

Cloud_Mesh_Distance_BA_R0_R2	19,05145836	-76,07110596	-942,9533081	0,050787
Cloud_Mesh_Distance_BA_R0_R2	19,22819519	-76,12950134	-942,949646	0,025151
Cloud_Mesh_Distance_BA_R0_R2	19,18096161	-76,13728333	-942,8088989	0,030197
Cloud_Mesh_Distance_BA_R0_R2	19,6312542	-76,30215454	-943,0484009	-0,036007
Cloud_Mesh_Distance_BA_R0_R2	18,62809372	-75,98983765	-942,9683838	0,082111
Cloud_Mesh_Distance_BA_R0_R2	18,74285316	-75,97492981	-943,114624	0,079971
Cloud_Mesh_Distance_BA_R0_R2	17,97930527	-83,94038391	-942,2838135	-0,001182
Cloud_Mesh_Distance_BA_R0_R2	17,58161354	-83,59532166	-942,3272095	-0,0334
Cloud_Mesh_Distance_BA_R0_R2	17,58763123	-83,55360413	-942,1223145	-0,044526
Cloud_Mesh_Distance_BA_R0_R2	17,64891434	-83,67771912	-942,4262085	-0,023097
Cloud_Mesh_Distance_BA_R0_R2	17,67809486	-83,6577301	-942,1873779	-0,033241
Cloud_Mesh_Distance_BA_R0_R2	17,68022156	-83,68721008	-942,3164673	-0,027069
Cloud_Mesh_Distance_BA_R0_R2	17,35523224	-83,33224487	-942,2272949	-0,054923
Cloud_Mesh_Distance_BA_R0_R2	17,18538284	-83,17051697	-942,3848877	-0,061627
Cloud_Mesh_Distance_BA_R0_R2	17,24236298	-83,25315857	-942,428833	-0,053132
Cloud_Mesh_Distance_BA_R0_R2	17,19135094	-83,14157104	-942,2647095	-0,07239
Cloud_Mesh_Distance_BA_R0_R2	16,99202347	-82,86192322	-942,2929688	-0,097159
Cloud_Mesh_Distance_BA_R0_R2	17,0402565	-82,97392273	-942,3961792	-0,082326
Cloud_Mesh_Distance_BA_R0_R2	16,84789658	-82,65086365	-942,3476563	-0,108242
Cloud_Mesh_Distance_BA_R0_R2	16,78379822	-82,58840942	-942,4501343	-0,103286
Cloud_Mesh_Distance_BA_R0_R2	16,91120529	-82,72763062	-942,2827148	-0,110907
Cloud_Mesh_Distance_BA_R0_R2	16,86415672	-82,71107483	-942,428833	-0,099895
Cloud_Mesh_Distance_BA_R0_R2	16,93804169	-82,84391785	-942,4700928	-0,086365
Cloud_Mesh_Distance_BA_R0_R2	16,74031448	-82,43891907	-942,2809448	-0,115925
Cloud_Mesh_Distance_BA_R0_R2	16,70545197	-82,45399475	-942,4379883	-0,106445
Cloud_Mesh_Distance_BA_R0_R2	16,8347435	-82,57505798	-942,2299805	-0,116476
Cloud_Mesh_Distance_BA_R0_R2	16,67527199	-82,37057495	-942,3666382	-0,107615
Cloud_Mesh_Distance_BA_R0_R2	16,63993073	-82,37281799	-942,4990845	-0,1012
Cloud_Mesh_Distance_BA_R0_R2	16,61267281	-82,26054382	-942,3565674	-0,1059
Cloud_Mesh_Distance_BA_R0_R2	16,51856995	-82,13700867	-942,427124	-0,096233
Cloud_Mesh_Distance_BA_R0_R2	16,35939026	-81,86328125	-942,4359131	-0,080082
Cloud_Mesh_Distance_BA_R0_R2	16,20646095	-81,60424805	-942,486084	-0,073616
Cloud_Mesh_Distance_BA_R0_R2	16,18635941	-81,51023865	-942,4060059	-0,071251
Cloud_Mesh_Distance_BA_R0_R2	16,26663399	-81,73130798	-942,5046387	-0,077996
Cloud_Mesh_Distance_BA_R0_R2	16,1377182	-81,52084351	-942,5612793	-0,072041
Cloud_Mesh_Distance_BA_R0_R2	16,06708717	-81,37294006	-942,5413818	-0,072779
Cloud_Mesh_Distance_BA_R0_R2	16,20444107	-81,45602417	-942,2872925	-0,067279
Cloud_Mesh_Distance_BA_R0_R2	23,20941925	-82,15208435	-942,4413452	0,181746
Cloud_Mesh_Distance_BA_R0_R2	23,2683773	-82,32939148	-942,4025269	0,253833
Cloud_Mesh_Distance_BA_R0_R2	23,33342743	-82,46731567	-942,4359131	0,301427
Cloud_Mesh_Distance_BA_R0_R2	16,01941299	-81,26341248	-942,5159912	-0,076631
Cloud_Mesh_Distance_BA_R0_R2	23,26683044	-82,24414063	-942,6101074	0,240055
Cloud_Mesh_Distance_BA_R0_R2	23,19863319	-82,07096863	-942,5939331	0,152102
Cloud_Mesh_Distance_BA_R0_R2	15,95309734	-81,15104675	-942,5299683	-0,0718
Cloud_Mesh_Distance_BA_R0_R2	15,95324612	-80,96841431	-942,3412476	-0,067133
Cloud_Mesh_Distance_BA_R0_R2	16,01989746	-81,1988678	-942,4358521	-0,073321
Cloud_Mesh_Distance_BA_R0_R2	16,03534317	-81,1134491	-942,3110962	-0,0678
Cloud_Mesh_Distance_BA_R0_R2	16,07011223	-81,25785828	-942,3865356	-0,074164
Cloud_Mesh_Distance_BA_R0_R2	23,11560249	-81,79704285	-942,5267334	0,02102
Cloud_Mesh_Distance_BA_R0_R2	15,91117668	-80,8260498	-942,303833	-0,062077

Cloud_Mesh_Distance_BA_R0_R2	15,8228817	-80,73918152	-942,399353	-0,067696
Cloud_Mesh_Distance_BA_R0_R2	15,87729645	-80,89537048	-942,4230957	-0,06842
Cloud_Mesh_Distance_BA_R0_R2	15,81301117	-80,9178772	-942,5653687	-0,072375
Cloud_Mesh_Distance_BA_R0_R2	23,09713173	-81,70597839	-942,6025391	-0,00624
Cloud_Mesh_Distance_BA_R0_R2	15,71329498	-80,61598206	-942,5071411	-0,076103
Cloud_Mesh_Distance_BA_R0_R2	23,07390594	-81,53289795	-942,6198731	-0,06474
Cloud_Mesh_Distance_BA_R0_R2	15,76018906	-80,38005066	-942,3134155	-0,080822
Cloud_Mesh_Distance_BA_R0_R2	15,7021389	-80,2881012	-942,3736572	-0,089665
Cloud_Mesh_Distance_BA_R0_R2	15,78399372	-80,52400208	-942,3379517	-0,073717
Cloud_Mesh_Distance_BA_R0_R2	23,0668602	-81,37579346	-942,6275024	-0,098252
Cloud_Mesh_Distance_BA_R0_R2	23,10041618	-81,1235199	-942,536377	-0,128742
Cloud_Mesh_Distance_BA_R0_R2	15,56376457	-80,14567566	-942,550354	-0,109976
Cloud_Mesh_Distance_BA_R0_R2	15,57989407	-80,24317932	-942,5553589	-0,099624
Cloud_Mesh_Distance_BA_R0_R2	15,63321495	-80,3344574	-942,5011597	-0,08974
Cloud_Mesh_Distance_BA_R0_R2	23,10463715	-81,00993347	-942,5787964	-0,134529
Cloud_Mesh_Distance_BA_R0_R2	23,06888962	-81,26356506	-942,6447754	-0,114351
Cloud_Mesh_Distance_BA_R0_R2	23,07870865	-81,05509949	-942,6777344	-0,13333
Cloud_Mesh_Distance_BA_R0_R2	23,12630272	-81,02532959	-942,4519043	-0,135029
Cloud_Mesh_Distance_BA_R0_R2	23,11875153	-81,15177917	-942,3883667	-0,128256
Cloud_Mesh_Distance_BA_R0_R2	15,51355553	-80,20628357	-942,6531372	-0,107973
Cloud_Mesh_Distance_BA_R0_R2	15,52652836	-80,07887268	-942,5927734	-0,119185
Cloud_Mesh_Distance_BA_R0_R2	15,57394123	-80,04164124	-942,5062866	-0,115005
Cloud_Mesh_Distance_BA_R0_R2	23,11974907	-80,91279602	-942,5513306	-0,133518
Cloud_Mesh_Distance_BA_R0_R2	15,48205185	-79,77763367	-942,6009522	-0,136472
Cloud_Mesh_Distance_BA_R0_R2	15,55302811	-79,89857483	-942,508667	-0,122992
Cloud_Mesh_Distance_BA_R0_R2	23,13311195	-80,77613831	-942,5371094	-0,134332
Cloud_Mesh_Distance_BA_R0_R2	15,59504128	-79,53753662	-942,3945923	-0,121252
Cloud_Mesh_Distance_BA_R0_R2	15,51272392	-79,06532288	-942,5271606	-0,123648
Cloud_Mesh_Distance_BA_R0_R2	15,4911871	-79,26538086	-942,5496216	-0,133848
Cloud_Mesh_Distance_BA_R0_R2	23,06436157	-80,10647583	-942,6433106	-0,099614
Cloud_Mesh_Distance_BA_R0_R2	23,09544373	-80,28869629	-942,6366577	-0,111297
Cloud_Mesh_Distance_BA_R0_R2	15,60917473	-78,95585632	-942,3862915	-0,106053
Cloud_Mesh_Distance_BA_R0_R2	23,05216217	-79,99375916	-942,5856934	-0,08928
Cloud_Mesh_Distance_BA_R0_R2	23,00932693	-79,99987793	-942,757019	-0,096621
Cloud_Mesh_Distance_BA_R0_R2	22,97280884	-79,86320496	-942,7453613	-0,08349
Cloud_Mesh_Distance_BA_R0_R2	15,52550888	-78,86833191	-942,5377808	-0,119963
Cloud_Mesh_Distance_BA_R0_R2	15,59161949	-78,78396606	-942,4464722	-0,107648
Cloud_Mesh_Distance_BA_R0_R2	15,49176311	-78,56471252	-942,6990356	-0,129949
Cloud_Mesh_Distance_BA_R0_R2	15,54109287	-78,68678284	-942,5588379	-0,117061
Cloud_Mesh_Distance_BA_R0_R2	15,56578636	-78,50737	-942,5817871	-0,114064
Cloud_Mesh_Distance_BA_R0_R2	22,82946396	-79,44529724	-942,5720215	-0,059858
Cloud_Mesh_Distance_BA_R0_R2	22,885746	-79,59129333	-942,6581421	-0,065195
Cloud_Mesh_Distance_BA_R0_R2	22,90813828	-79,55580139	-942,4729004	-0,060822
Cloud_Mesh_Distance_BA_R0_R2	22,94710922	-79,67964172	-942,5698853	-0,064049
Cloud_Mesh_Distance_BA_R0_R2	15,58468628	-78,35905457	-942,6168823	-0,112032
Cloud_Mesh_Distance_BA_R0_R2	15,52533913	-78,38458252	-942,7199097	-0,123432
Cloud_Mesh_Distance_BA_R0_R2	22,74304199	-79,33612061	-942,677124	-0,061434
Cloud_Mesh_Distance_BA_R0_R2	22,80714417	-79,50575256	-942,7896118	-0,069959
Cloud_Mesh_Distance_BA_R0_R2	15,64847565	-78,41020203	-942,4796753	-0,098965
Cloud_Mesh_Distance_BA_R0_R2	15,7197876	-78,1973114	-942,4640503	-0,087957

Cloud_Mesh_Distance_BA_R0_R2	15,62591743	-78,22555542	-942,6113892	-0,102246
Cloud_Mesh_Distance_BA_R0_R2	22,43482018	-78,84977722	-942,6766357	-0,033685
Cloud_Mesh_Distance_BA_R0_R2	22,36046791	-78,72312927	-942,6240845	-0,020133
Cloud_Mesh_Distance_BA_R0_R2	22,36388779	-78,79576111	-942,8170776	-0,035873
Cloud_Mesh_Distance_BA_R0_R2	22,51033974	-78,92471313	-942,548645	-0,037478
Cloud_Mesh_Distance_BA_R0_R2	15,80820179	-77,64024353	-942,7023315	-0,065526
Cloud_Mesh_Distance_BA_R0_R2	15,87534523	-77,67037964	-942,5646973	-0,058922
Cloud_Mesh_Distance_BA_R0_R2	22,11966705	-78,41726685	-942,7085571	-0,006644
Cloud_Mesh_Distance_BA_R0_R2	22,11777306	-78,36962891	-942,5700684	0,00128
Cloud_Mesh_Distance_BA_R0_R2	22,05862236	-78,3208313	-942,6591187	-0,00124
Cloud_Mesh_Distance_BA_R0_R2	22,27298546	-78,60102844	-942,6345825	-0,013794
Cloud_Mesh_Distance_BA_R0_R2	15,86037827	-77,50839233	-942,7376099	-0,055575
Cloud_Mesh_Distance_BA_R0_R2	15,88419724	-77,57606506	-942,6323242	-0,055937
Cloud_Mesh_Distance_BA_R0_R2	15,93643379	-77,47438049	-942,6477661	-0,048285
Cloud_Mesh_Distance_BA_R0_R2	21,97292709	-78,16242981	-942,4835815	0,018044
Cloud_Mesh_Distance_BA_R0_R2	22,054142	-78,2741394	-942,5224609	0,009713
Cloud_Mesh_Distance_BA_R0_R2	16,00627136	-77,31890869	-942,7084351	-0,047502
Cloud_Mesh_Distance_BA_R0_R2	16,04148865	-77,46539307	-942,5026245	-0,04438
Cloud_Mesh_Distance_BA_R0_R2	21,74103928	-77,97850037	-942,7936401	0,003316
Cloud_Mesh_Distance_BA_R0_R2	21,83300018	-78,09251404	-942,8022461	0,001162
Cloud_Mesh_Distance_BA_R0_R2	16,1258831	-77,22343445	-942,6448364	-0,041479
Cloud_Mesh_Distance_BA_R0_R2	16,20381737	-77,09338379	-942,7038574	-0,03647
Cloud_Mesh_Distance_BA_R0_R2	16,22568512	-77,21313477	-942,5209961	-0,037788
Cloud_Mesh_Distance_BA_R0_R2	21,50914001	-77,65240479	-942,5874023	0,023509
Cloud_Mesh_Distance_BA_R0_R2	21,69941711	-77,90203857	-942,7141724	0,011467
Cloud_Mesh_Distance_BA_R0_R2	21,61481285	-77,75506592	-942,5183106	0,023082
Cloud_Mesh_Distance_BA_R0_R2	21,78054047	-77,97703552	-942,6490479	0,013833
Cloud_Mesh_Distance_BA_R0_R2	21,7030201	-77,86451721	-942,5640869	0,019794
Cloud_Mesh_Distance_BA_R0_R2	16,35554123	-77,03094482	-942,5829468	-0,035185
Cloud_Mesh_Distance_BA_R0_R2	16,49355888	-76,81896973	-942,7195435	-0,029568
Cloud_Mesh_Distance_BA_R0_R2	16,30479431	-76,94483948	-942,7810669	-0,041724
Cloud_Mesh_Distance_BA_R0_R2	21,38905716	-77,52401733	-942,5944824	0,0221
Cloud_Mesh_Distance_BA_R0_R2	21,40792656	-77,57670593	-942,7338257	0,017179
Cloud_Mesh_Distance_BA_R0_R2	16,59738922	-76,72840881	-942,7331543	-0,026361
Cloud_Mesh_Distance_BA_R0_R2	16,57009125	-76,84870911	-942,5717773	-0,026707
Cloud_Mesh_Distance_BA_R0_R2	21,24765778	-77,38514709	-942,6459961	0,019641
Cloud_Mesh_Distance_BA_R0_R2	16,71145248	-76,75379944	-942,5490112	-0,018435
Cloud_Mesh_Distance_BA_R0_R2	20,82789993	-77,01956177	-942,8482666	0,012258
Cloud_Mesh_Distance_BA_R0_R2	20,8386631	-77,0161438	-942,7442017	0,011441
Cloud_Mesh_Distance_BA_R0_R2	20,92791748	-77,10063171	-942,8049316	0,0153
Cloud_Mesh_Distance_BA_R0_R2	16,86111259	-76,58462524	-942,6643677	-0,012083
Cloud_Mesh_Distance_BA_R0_R2	16,97298431	-76,49552917	-942,7086792	-0,00709
Cloud_Mesh_Distance_BA_R0_R2	16,81126976	-76,53889465	-942,8092651	-0,012486
Cloud_Mesh_Distance_BA_R0_R2	17,11533737	-76,44294739	-942,6687012	-0,002006
Cloud_Mesh_Distance_BA_R0_R2	20,57956123	-76,82772827	-942,7645874	-0,007448
Cloud_Mesh_Distance_BA_R0_R2	17,5983963	-76,25567627	-942,6708374	0,006832
Cloud_Mesh_Distance_BA_R0_R2	17,37685013	-76,28971863	-942,762207	0,005138
Cloud_Mesh_Distance_BA_R0_R2	20,04259872	-76,52798462	-942,7070923	-0,044678
Cloud_Mesh_Distance_BA_R0_R2	20,48015213	-76,76367188	-942,7305908	-0,016364
Cloud_Mesh_Distance_BA_R0_R2	20,36755371	-76,69807434	-942,7686768	-0,030217

Cloud_Mesh_Distance_BA_R0_R2	20,30175209	-76,6663208	-942,6052856	-0,026493
Cloud_Mesh_Distance_BA_R0_R2	20,53821182	-76,79862976	-942,6338501	-0,011517
Cloud_Mesh_Distance_BA_R0_R2	17,5698719	-76,19946289	-942,8278198	0,009244
Cloud_Mesh_Distance_BA_R0_R2	17,94237328	-76,10713196	-942,8083496	0,029196
Cloud_Mesh_Distance_BA_R0_R2	18,07382965	-76,10354614	-942,7390137	0,03995
Cloud_Mesh_Distance_BA_R0_R2	18,27640533	-76,13607788	-942,5598145	0,04742
Cloud_Mesh_Distance_BA_R0_R2	19,56535339	-76,29850769	-942,7420044	-0,019826
Cloud_Mesh_Distance_BA_R0_R2	19,92093849	-76,45767212	-942,8615112	-0,051025
Cloud_Mesh_Distance_BA_R0_R2	18,01013565	-76,07041931	-942,869812	0,040729
Cloud_Mesh_Distance_BA_R0_R2	18,20143127	-76,10101318	-942,6826782	0,047424
Cloud_Mesh_Distance_BA_R0_R2	18,55430794	-76,01408386	-942,8613892	0,073289
Cloud_Mesh_Distance_BA_R0_R2	18,34315872	-76,07984924	-942,6877441	0,057618
Cloud_Mesh_Distance_BA_R0_R2	18,68184662	-76,0279541	-942,8366699	0,073561
Cloud_Mesh_Distance_BA_R0_R2	18,82775497	-76,12069702	-942,5761719	0,05623
Cloud_Mesh_Distance_BA_R0_R2	18,99603081	-76,13056946	-942,6330566	0,045674
Cloud_Mesh_Distance_BA_R0_R2	19,04710007	-76,10748291	-942,7662354	0,045342
Cloud_Mesh_Distance_BA_R0_R2	19,13362122	-76,15141296	-942,6809082	0,03351
Cloud_Mesh_Distance_BA_R0_R2	19,29692459	-76,20335388	-942,6785889	0,011302
Cloud_Mesh_Distance_BA_R0_R2	19,30737686	-76,17419434	-942,8515015	0,014754
Cloud_Mesh_Distance_BA_R0_R2	19,6107769	-76,30104065	-942,8974609	-0,028077
Cloud_Mesh_Distance_BA_R0_R2	19,44906235	-76,23435974	-942,8352661	-0,008142
Cloud_Mesh_Distance_BA_R0_R2	18,36719704	-84,17094421	-941,9598389	0,061648
Cloud_Mesh_Distance_BA_R0_R2	18,1132946	-84,02575684	-942,1688843	0,103601
Cloud_Mesh_Distance_BA_R0_R2	18,15877151	-84,01066589	-941,9327393	0,001115
Cloud_Mesh_Distance_BA_R0_R2	18,24918175	-84,10739136	-942,0599976	0,014741
Cloud_Mesh_Distance_BA_R0_R2	17,85858345	-83,82635498	-942,2164917	-0,015865
Cloud_Mesh_Distance_BA_R0_R2	17,89336014	-83,8102417	-942,0064087	-0,020788
Cloud_Mesh_Distance_BA_R0_R2	17,7798748	-83,73182678	-942,1000977	-0,028455
Cloud_Mesh_Distance_BA_R0_R2	17,7481575	-83,73474121	-942,2434082	-0,025593
Cloud_Mesh_Distance_BA_R0_R2	18,00095367	-83,91694641	-942,076294	-0,009424
Cloud_Mesh_Distance_BA_R0_R2	17,50948524	-83,44898987	-942,0401611	-0,05408
Cloud_Mesh_Distance_BA_R0_R2	17,70020103	-83,63522339	-942,0115967	-0,039217
Cloud_Mesh_Distance_BA_R0_R2	17,45950508	-83,45120239	-942,2407227	-0,048448
Cloud_Mesh_Distance_BA_R0_R2	17,40888977	-83,34719849	-942,0722656	-0,06166
Cloud_Mesh_Distance_BA_R0_R2	17,12544632	-83,07250977	-942,3264771	-0,076526
Cloud_Mesh_Distance_BA_R0_R2	17,26363182	-83,23898315	-942,2820435	-0,060279
Cloud_Mesh_Distance_BA_R0_R2	17,31692696	-83,25788879	-942,1328125	-0,067358
Cloud_Mesh_Distance_BA_R0_R2	17,07962608	-82,96266174	-942,2070923	-0,092323
Cloud_Mesh_Distance_BA_R0_R2	17,15559769	-83,07203674	-942,2050781	-0,084761
Cloud_Mesh_Distance_BA_R0_R2	17,10237122	-82,92037964	-942,0219116	-0,103743
Cloud_Mesh_Distance_BA_R0_R2	16,99925232	-82,79386902	-942,1068115	-0,114211
Cloud_Mesh_Distance_BA_R0_R2	16,79133987	-82,44396973	-942,1161499	-0,124807
Cloud_Mesh_Distance_BA_R0_R2	16,91254997	-82,65022278	-942,1099243	-0,118467
Cloud_Mesh_Distance_BA_R0_R2	16,64649391	-82,23573303	-942,1988525	-0,112847
Cloud_Mesh_Distance_BA_R0_R2	16,67863655	-82,32392883	-942,2609253	-0,113351
Cloud_Mesh_Distance_BA_R0_R2	16,57172394	-82,11097717	-942,208252	-0,101861
Cloud_Mesh_Distance_BA_R0_R2	16,59746742	-82,05567932	-942,0325928	-0,103652
Cloud_Mesh_Distance_BA_R0_R2	16,38985062	-81,81907654	-942,2687378	-0,079131
Cloud_Mesh_Distance_BA_R0_R2	16,45824051	-81,96359253	-942,3009033	-0,086428
Cloud_Mesh_Distance_BA_R0_R2	16,32542801	-81,72253418	-942,3102417	-0,071756

Cloud_Mesh_Distance_BA_R0_R2	16,32348061	-81,59700012	-942,1418457	-0,071228
Cloud_Mesh_Distance_BA_R0_R2	16,25044632	-81,58421326	-942,328064	-0,071427
Cloud_Mesh_Distance_BA_R0_R2	16,37284279	-81,72755432	-942,180481	-0,075831
Cloud_Mesh_Distance_BA_R0_R2	23,1894474	-82,3119812	-942,1570435	0,204863
Cloud_Mesh_Distance_BA_R0_R2	23,23669434	-82,35832214	-942,2450562	0,238003
Cloud_Mesh_Distance_BA_R0_R2	23,22462654	-82,43789673	-942,1218262	0,251741
Cloud_Mesh_Distance_BA_R0_R2	16,14081764	-81,38420105	-942,3631592	-0,071652
Cloud_Mesh_Distance_BA_R0_R2	16,18901062	-81,35458374	-942,208313	-0,066577
Cloud_Mesh_Distance_BA_R0_R2	23,19937134	-82,22740173	-942,28125	0,189969
Cloud_Mesh_Distance_BA_R0_R2	23,14363861	-82,18890381	-942,1369019	0,140757
Cloud_Mesh_Distance_BA_R0_R2	23,15849686	-81,99391174	-942,4415283	0,099863
Cloud_Mesh_Distance_BA_R0_R2	16,08310127	-81,03889465	-942,1417236	-0,05796
Cloud_Mesh_Distance_BA_R0_R2	16,1421051	-81,19880676	-942,1583862	-0,058956
Cloud_Mesh_Distance_BA_R0_R2	23,09068298	-81,75508118	-942,2820435	-0,019028
Cloud_Mesh_Distance_BA_R0_R2	23,14726639	-82,04200745	-942,3012085	0,100744
Cloud_Mesh_Distance_BA_R0_R2	23,11903763	-81,86151123	-942,3847656	0,035382
Cloud_Mesh_Distance_BA_R0_R2	23,06979752	-81,74966431	-942,128479	-0,043483
Cloud_Mesh_Distance_BA_R0_R2	23,09492874	-81,68397522	-942,4451904	-0,02785
Cloud_Mesh_Distance_BA_R0_R2	23,08492661	-81,55397034	-942,4390259	-0,0689
Cloud_Mesh_Distance_BA_R0_R2	15,90812016	-80,71852112	-942,2358398	-0,063977
Cloud_Mesh_Distance_BA_R0_R2	15,99436951	-80,79402161	-942,1287231	-0,05591
Cloud_Mesh_Distance_BA_R0_R2	15,94045448	-80,63841248	-942,1318359	-0,054576
Cloud_Mesh_Distance_BA_R0_R2	23,08269882	-81,60450745	-942,2735596	-0,07018
Cloud_Mesh_Distance_BA_R0_R2	23,09769058	-81,2407074	-942,4690552	-0,118558
Cloud_Mesh_Distance_BA_R0_R2	23,08832932	-81,40431213	-942,4203491	-0,102151
Cloud_Mesh_Distance_BA_R0_R2	23,10800934	-81,26269531	-942,3511353	-0,122428
Cloud_Mesh_Distance_BA_R0_R2	15,7673378	-80,27244568	-942,2644043	-0,081966
Cloud_Mesh_Distance_BA_R0_R2	15,80213833	-80,19561768	-942,1862183	-0,074295
Cloud_Mesh_Distance_BA_R0_R2	15,7270565	-80,12861633	-942,2870483	-0,090373
Cloud_Mesh_Distance_BA_R0_R2	15,84726334	-80,36434937	-942,1657715	-0,067793
Cloud_Mesh_Distance_BA_R0_R2	23,12357712	-81,20018005	-942,270874	-0,132058
Cloud_Mesh_Distance_BA_R0_R2	15,63098907	-80,16394043	-942,4481201	-0,103636
Cloud_Mesh_Distance_BA_R0_R2	15,74105835	-79,96159363	-942,2296143	-0,090576
Cloud_Mesh_Distance_BA_R0_R2	23,18081665	-80,8674469	-942,204834	-0,141075
Cloud_Mesh_Distance_BA_R0_R2	15,56587982	-79,70506287	-942,4559937	-0,122782
Cloud_Mesh_Distance_BA_R0_R2	15,71952248	-79,78723145	-942,2358398	-0,09867
Cloud_Mesh_Distance_BA_R0_R2	15,626688	-79,80027771	-942,3765869	-0,11391
Cloud_Mesh_Distance_BA_R0_R2	15,65106773	-79,96774292	-942,3695068	-0,104005
Cloud_Mesh_Distance_BA_R0_R2	23,19812775	-80,69107056	-942,1867676	-0,136252
Cloud_Mesh_Distance_BA_R0_R2	23,14270401	-80,61175537	-942,5167236	-0,13044
Cloud_Mesh_Distance_BA_R0_R2	23,15044212	-80,89607239	-942,3964233	-0,135984
Cloud_Mesh_Distance_BA_R0_R2	23,16852951	-80,73420715	-942,3678589	-0,134322
Cloud_Mesh_Distance_BA_R0_R2	15,66936111	-79,51507568	-942,2824707	-0,104508
Cloud_Mesh_Distance_BA_R0_R2	15,65275764	-79,65034485	-942,3187256	-0,109684
Cloud_Mesh_Distance_BA_R0_R2	15,70766354	-79,44065857	-942,2241211	-0,097021
Cloud_Mesh_Distance_BA_R0_R2	15,7495575	-79,56306458	-942,170044	-0,088158
Cloud_Mesh_Distance_BA_R0_R2	23,14473724	-80,4393158	-942,4857178	-0,120159
Cloud_Mesh_Distance_BA_R0_R2	23,14014053	-80,29118347	-942,44104	-0,107977
Cloud_Mesh_Distance_BA_R0_R2	23,15928268	-80,23580933	-942,2913818	-0,100162
Cloud_Mesh_Distance_BA_R0_R2	15,71182156	-79,24656677	-942,2167358	-0,09453

Cloud_Mesh_Distance_BA_R0_R2	15,64652634	-79,38624573	-942,3104248	-0,10639
Cloud_Mesh_Distance_BA_R0_R2	15,59771729	-79,19926453	-942,3823853	-0,110518
Cloud_Mesh_Distance_BA_R0_R2	15,54808617	-79,3976593	-942,4581909	-0,123218
Cloud_Mesh_Distance_BA_R0_R2	23,10647583	-80,10884094	-942,4577026	-0,092587
Cloud_Mesh_Distance_BA_R0_R2	23,13904953	-80,10154724	-942,2625122	-0,090672
Cloud_Mesh_Distance_BA_R0_R2	15,76196098	-78,95637512	-942,1694946	-0,083677
Cloud_Mesh_Distance_BA_R0_R2	15,68990707	-79,06565857	-942,2561646	-0,093382
Cloud_Mesh_Distance_BA_R0_R2	15,77511978	-79,10110474	-942,137146	-0,082917
Cloud_Mesh_Distance_BA_R0_R2	23,0628376	-79,94091797	-942,4680176	-0,080962
Cloud_Mesh_Distance_BA_R0_R2	23,00549698	-79,83848572	-942,5846558	-0,075042
Cloud_Mesh_Distance_BA_R0_R2	23,04274368	-79,83898926	-942,4011841	-0,074006
Cloud_Mesh_Distance_BA_R0_R2	15,6950016	-78,9190979	-942,2653809	-0,091169
Cloud_Mesh_Distance_BA_R0_R2	15,61822891	-78,62324524	-942,4478149	-0,104965
Cloud_Mesh_Distance_BA_R0_R2	15,70529366	-78,56356812	-942,3326416	-0,088978
Cloud_Mesh_Distance_BA_R0_R2	22,8491745	-79,41424561	-942,364563	-0,052572
Cloud_Mesh_Distance_BA_R0_R2	22,98859024	-79,69610596	-942,3908691	-0,06086
Cloud_Mesh_Distance_BA_R0_R2	22,7794857	-79,27487183	-942,2554321	-0,048285
Cloud_Mesh_Distance_BA_R0_R2	22,76276588	-79,29621887	-942,4649658	-0,053382
Cloud_Mesh_Distance_BA_R0_R2	15,79137707	-78,25723267	-942,3234863	-0,075089
Cloud_Mesh_Distance_BA_R0_R2	15,82090187	-78,11094666	-942,3534546	-0,073441
Cloud_Mesh_Distance_BA_R0_R2	22,67251015	-79,18339539	-942,5733032	-0,052194
Cloud_Mesh_Distance_BA_R0_R2	22,60237885	-79,04490662	-942,4699707	-0,044615
Cloud_Mesh_Distance_BA_R0_R2	15,7703495	-77,97602844	-942,5099487	-0,076516
Cloud_Mesh_Distance_BA_R0_R2	15,92121315	-78,07621765	-942,2322998	-0,063718
Cloud_Mesh_Distance_BA_R0_R2	22,43754959	-78,81167603	-942,5377197	-0,026841
Cloud_Mesh_Distance_BA_R0_R2	22,40129852	-78,73651123	-942,4562378	-0,019879
Cloud_Mesh_Distance_BA_R0_R2	15,87884331	-77,92518616	-942,3762207	-0,065052
Cloud_Mesh_Distance_BA_R0_R2	15,82668495	-77,79486084	-942,5445557	-0,067261
Cloud_Mesh_Distance_BA_R0_R2	15,95979691	-77,94085693	-942,2568359	-0,060262
Cloud_Mesh_Distance_BA_R0_R2	22,31965637	-78,61416626	-942,4435425	-0,011188
Cloud_Mesh_Distance_BA_R0_R2	22,47072029	-78,82553101	-942,3917847	-0,028664
Cloud_Mesh_Distance_BA_R0_R2	15,91642952	-77,75250244	-942,4387817	-0,057052
Cloud_Mesh_Distance_BA_R0_R2	16,0197525	-77,7020874	-942,335083	-0,05148
Cloud_Mesh_Distance_BA_R0_R2	15,96207237	-77,83601379	-942,3187866	-0,059533
Cloud_Mesh_Distance_BA_R0_R2	22,2045269	-78,48017883	-942,5498657	-0,005416
Cloud_Mesh_Distance_BA_R0_R2	22,24008751	-78,47758484	-942,3294067	0,003006
Cloud_Mesh_Distance_BA_R0_R2	16,04188919	-77,58108521	-942,4003296	-0,049638
Cloud_Mesh_Distance_BA_R0_R2	15,95587826	-77,60018921	-942,5026856	-0,055596
Cloud_Mesh_Distance_BA_R0_R2	21,90703583	-78,07194519	-942,4337769	0,019864
Cloud_Mesh_Distance_BA_R0_R2	16,11912918	-77,43783569	-942,4247437	-0,043004
Cloud_Mesh_Distance_BA_R0_R2	16,27149391	-77,3228302	-942,3443604	-0,038311
Cloud_Mesh_Distance_BA_R0_R2	16,13627434	-77,34054565	-942,498291	-0,041522
Cloud_Mesh_Distance_BA_R0_R2	16,33054543	-77,19110107	-942,4125366	-0,033815
Cloud_Mesh_Distance_BA_R0_R2	21,88135529	-78,07722473	-942,5820313	0,014031
Cloud_Mesh_Distance_BA_R0_R2	21,89725876	-78,03988647	-942,3068848	0,022523
Cloud_Mesh_Distance_BA_R0_R2	16,38554764	-77,10212708	-942,4515991	-0,033787
Cloud_Mesh_Distance_BA_R0_R2	16,29898262	-77,1242981	-942,5327759	-0,037955
Cloud_Mesh_Distance_BA_R0_R2	16,42921448	-77,15180969	-942,338501	-0,032113
Cloud_Mesh_Distance_BA_R0_R2	16,37619781	-77,24342346	-942,2993774	-0,032147
Cloud_Mesh_Distance_BA_R0_R2	21,6029129	-77,72496033	-942,4181519	0,022869

Cloud_Mesh_Distance_BA_R0_R2	21,56766129	-77,69668579	-942,484436	0,024242
Cloud_Mesh_Distance_BA_R0_R2	16,51854324	-77,01989746	-942,3953247	-0,025824
Cloud_Mesh_Distance_BA_R0_R2	16,43705368	-76,9163208	-942,6414185	-0,03354
Cloud_Mesh_Distance_BA_R0_R2	16,68593597	-76,87141418	-942,4018555	-0,021133
Cloud_Mesh_Distance_BA_R0_R2	21,15067101	-77,28755188	-942,6459961	0,020543
Cloud_Mesh_Distance_BA_R0_R2	21,34938812	-77,46606445	-942,496521	0,024304
Cloud_Mesh_Distance_BA_R0_R2	21,31066895	-77,44117737	-942,5946655	0,021035
Cloud_Mesh_Distance_BA_R0_R2	21,25593567	-77,3722229	-942,5012817	0,025475
Cloud_Mesh_Distance_BA_R0_R2	21,34156418	-77,44515991	-942,3641968	0,024483
Cloud_Mesh_Distance_BA_R0_R2	16,95317078	-76,58348083	-942,5693359	-0,010469
Cloud_Mesh_Distance_BA_R0_R2	16,84336853	-76,66967773	-942,5379028	-0,011226
Cloud_Mesh_Distance_BA_R0_R2	16,79499435	-76,73852539	-942,4794922	-0,011528
Cloud_Mesh_Distance_BA_R0_R2	16,86599922	-76,74169922	-942,4006958	-0,00918
Cloud_Mesh_Distance_BA_R0_R2	20,90074921	-77,05027771	-942,4885864	0,016575
Cloud_Mesh_Distance_BA_R0_R2	21,01202583	-77,13961792	-942,3657227	0,020507
Cloud_Mesh_Distance_BA_R0_R2	21,01457405	-77,15281677	-942,5933228	0,020674
Cloud_Mesh_Distance_BA_R0_R2	17,10415268	-76,52584839	-942,5282593	-0,003422
Cloud_Mesh_Distance_BA_R0_R2	17,2350235	-76,43475342	-942,5806274	-0,00077
Cloud_Mesh_Distance_BA_R0_R2	17,28005219	-76,50013733	-942,4290772	-0,004822
Cloud_Mesh_Distance_BA_R0_R2	17,40960884	-76,41127014	-942,4903565	0,004516
Cloud_Mesh_Distance_BA_R0_R2	17,5633297	-76,43164063	-942,3439331	0,002603
Cloud_Mesh_Distance_BA_R0_R2	17,69844246	-76,3671875	-942,3724365	0,009951
Cloud_Mesh_Distance_BA_R0_R2	20,65636444	-76,87432861	-942,5802612	-0,001238
Cloud_Mesh_Distance_BA_R0_R2	20,89939308	-77,05760193	-942,649292	0,015624
Cloud_Mesh_Distance_BA_R0_R2	20,80218887	-76,97831726	-942,6148071	0,008799
Cloud_Mesh_Distance_BA_R0_R2	17,19657326	-76,38691711	-942,7053223	-0,000803
Cloud_Mesh_Distance_BA_R0_R2	17,32268143	-76,35774231	-942,6588135	0,000809
Cloud_Mesh_Distance_BA_R0_R2	17,56644821	-76,36308289	-942,4700317	0,006367
Cloud_Mesh_Distance_BA_R0_R2	17,49084091	-76,31611633	-942,6171265	0,005305
Cloud_Mesh_Distance_BA_R0_R2	17,67414474	-76,29925537	-942,5245972	0,00986
Cloud_Mesh_Distance_BA_R0_R2	20,00689507	-76,5300293	-942,5203857	-0,039254
Cloud_Mesh_Distance_BA_R0_R2	20,15575027	-76,59431458	-942,5759277	-0,035569
Cloud_Mesh_Distance_BA_R0_R2	20,24814606	-76,65080261	-942,460144	-0,027813
Cloud_Mesh_Distance_BA_R0_R2	20,43578911	-76,73893738	-942,6106567	-0,020145
Cloud_Mesh_Distance_BA_R0_R2	20,38471222	-76,72143555	-942,4406128	-0,019554
Cloud_Mesh_Distance_BA_R0_R2	17,78837776	-76,31690979	-942,4128418	0,017104
Cloud_Mesh_Distance_BA_R0_R2	17,81217766	-76,27253723	-942,4909058	0,017469
Cloud_Mesh_Distance_BA_R0_R2	17,76353836	-76,21955872	-942,6400147	0,017517
Cloud_Mesh_Distance_BA_R0_R2	17,97397041	-76,16166687	-942,6473999	0,029659
Cloud_Mesh_Distance_BA_R0_R2	18,1786232	-76,20982361	-942,4237671	0,041391
Cloud_Mesh_Distance_BA_R0_R2	18,14148331	-76,1408844	-942,6070557	0,043693
Cloud_Mesh_Distance_BA_R0_R2	18,8433609	-76,16680908	-942,4467163	0,048073
Cloud_Mesh_Distance_BA_R0_R2	18,70656395	-76,14762878	-942,4707031	0,052555
Cloud_Mesh_Distance_BA_R0_R2	19,45851898	-76,26535034	-942,6730347	-0,007471
Cloud_Mesh_Distance_BA_R0_R2	19,38223648	-76,30754089	-942,390625	-0,000335
Cloud_Mesh_Distance_BA_R0_R2	19,57017136	-76,33280945	-942,5609741	-0,019055
Cloud_Mesh_Distance_BA_R0_R2	19,74499321	-76,40560913	-942,5648804	-0,03458
Cloud_Mesh_Distance_BA_R0_R2	19,5745163	-76,37713623	-942,3795166	-0,020959
Cloud_Mesh_Distance_BA_R0_R2	19,88345528	-76,45748901	-942,6398926	-0,040099
Cloud_Mesh_Distance_BA_R0_R2	18,59553909	-76,0741272	-942,6711426	0,061231

Cloud_Mesh_Distance_BA_R0_R2	18,43555832	-76,126297	-942,5392456	0,05703
Cloud_Mesh_Distance_BA_R0_R2	19,40013695	-76,26777649	-942,560669	-0,001528
Cloud_Mesh_Distance_BA_R0_R2	18,61046028	-84,28773499	-941,7816773	0,025062
Cloud_Mesh_Distance_BA_R0_R2	18,2852459	-84,10049438	-941,9087524	0,009381
Cloud_Mesh_Distance_BA_R0_R2	17,90354729	-83,78265381	-941,8620606	-0,024726
Cloud_Mesh_Distance_BA_R0_R2	17,81138039	-83,71784973	-941,9299316	-0,032307
Cloud_Mesh_Distance_BA_R0_R2	17,60929298	-83,51979065	-941,9259033	-0,054366
Cloud_Mesh_Distance_BA_R0_R2	17,633461	-83,48475647	-941,7386475	-0,061293
Cloud_Mesh_Distance_BA_R0_R2	17,33296013	-83,21598816	-941,9558106	-0,083181
Cloud_Mesh_Distance_BA_R0_R2	17,30359268	-83,11523438	-941,7940064	-0,092572
Cloud_Mesh_Distance_BA_R0_R2	17,20905304	-83,04441833	-941,9411621	-0,094635
Cloud_Mesh_Distance_BA_R0_R2	17,23048401	-83,14527893	-942,1260376	-0,08123
Cloud_Mesh_Distance_BA_R0_R2	17,15443039	-83,0304718	-942,1002197	-0,090619
Cloud_Mesh_Distance_BA_R0_R2	17,04938126	-82,79559326	-941,9311523	-0,116736
Cloud_Mesh_Distance_BA_R0_R2	17,13075256	-82,88658142	-941,84552	-0,114399
Cloud_Mesh_Distance_BA_R0_R2	16,959589	-82,6308136	-941,9038696	-0,125467
Cloud_Mesh_Distance_BA_R0_R2	16,80164528	-82,37110901	-941,9431152	-0,125187
Cloud_Mesh_Distance_BA_R0_R2	16,86266708	-82,52331543	-942,0280762	-0,127114
Cloud_Mesh_Distance_BA_R0_R2	16,52541733	-81,9584198	-942,0891724	-0,094491
Cloud_Mesh_Distance_BA_R0_R2	16,71863937	-82,31428528	-942,1107178	-0,117643
Cloud_Mesh_Distance_BA_R0_R2	16,65176582	-82,17054749	-942,0636597	-0,111462
Cloud_Mesh_Distance_BA_R0_R2	16,72130394	-82,21974182	-941,9294434	-0,116448
Cloud_Mesh_Distance_BA_R0_R2	16,59487152	-81,9198761	-941,8173828	-0,091148
Cloud_Mesh_Distance_BA_R0_R2	16,51768112	-81,85015869	-941,9423218	-0,086679
Cloud_Mesh_Distance_BA_R0_R2	16,37561798	-81,58415222	-941,9845581	-0,065197
Cloud_Mesh_Distance_BA_R0_R2	16,39041519	-81,68109131	-942,0666504	-0,073406
Cloud_Mesh_Distance_BA_R0_R2	16,44032478	-81,81185913	-942,109314	-0,083717
Cloud_Mesh_Distance_BA_R0_R2	23,14234161	-82,35697937	-941,9990234	0,188377
Cloud_Mesh_Distance_BA_R0_R2	16,39829826	-81,50498962	-941,831543	-0,058191
Cloud_Mesh_Distance_BA_R0_R2	16,44569969	-81,68157959	-941,9129028	-0,06751
Cloud_Mesh_Distance_BA_R0_R2	16,21184158	-81,29891968	-942,093689	-0,058404
Cloud_Mesh_Distance_BA_R0_R2	16,32501411	-81,45277405	-941,9689941	-0,057895
Cloud_Mesh_Distance_BA_R0_R2	16,25124741	-81,43348694	-942,1383667	-0,063849
Cloud_Mesh_Distance_BA_R0_R2	23,1038723	-82,05104065	-942,1203613	0,064277
Cloud_Mesh_Distance_BA_R0_R2	23,06223297	-81,98512268	-942,0106201	0,018208
Cloud_Mesh_Distance_BA_R0_R2	16,0271244	-80,96635437	-942,194458	-0,061529
Cloud_Mesh_Distance_BA_R0_R2	16,26494408	-81,28140259	-941,9476318	-0,053283
Cloud_Mesh_Distance_BA_R0_R2	16,20798111	-81,22032166	-942,0270386	-0,055295
Cloud_Mesh_Distance_BA_R0_R2	23,09190559	-81,89582825	-942,1810303	0,016999
Cloud_Mesh_Distance_BA_R0_R2	16,06713676	-80,81216431	-942,0033569	-0,043186
Cloud_Mesh_Distance_BA_R0_R2	16,07216263	-80,90974426	-942,0614624	-0,051824
Cloud_Mesh_Distance_BA_R0_R2	16,13795853	-81,00160217	-941,9960938	-0,052566
Cloud_Mesh_Distance_BA_R0_R2	23,06085014	-81,62419128	-942,0231323	-0,089856
Cloud_Mesh_Distance_BA_R0_R2	15,87048244	-80,58288574	-942,2232056	-0,063355
Cloud_Mesh_Distance_BA_R0_R2	16,11977577	-80,84480286	-941,9207764	-0,039529
Cloud_Mesh_Distance_BA_R0_R2	23,08682251	-81,47459412	-942,1802979	-0,106201
Cloud_Mesh_Distance_BA_R0_R2	16,00042725	-80,59184265	-941,9995117	-0,046098
Cloud_Mesh_Distance_BA_R0_R2	15,92601776	-80,38356018	-942,0402832	-0,052257
Cloud_Mesh_Distance_BA_R0_R2	15,92963791	-80,49537659	-942,0800171	-0,054148
Cloud_Mesh_Distance_BA_R0_R2	23,10804939	-81,32110596	-942,2055054	-0,121937

Cloud_Mesh_Distance_BA_R0_R2	23,08813858	-81,42634583	-941,9885254	-0,129236
Cloud_Mesh_Distance_BA_R0_R2	15,86772919	-80,1587677	-942,0704956	-0,059421
Cloud_Mesh_Distance_BA_R0_R2	15,93254566	-80,26846313	-941,9925537	-0,047274
Cloud_Mesh_Distance_BA_R0_R2	23,1295681	-81,20497131	-942,1656494	-0,138052
Cloud_Mesh_Distance_BA_R0_R2	23,14373589	-81,16197205	-942,0446777	-0,147129
Cloud_Mesh_Distance_BA_R0_R2	23,14810944	-81,06396484	-942,2454834	-0,139162
Cloud_Mesh_Distance_BA_R0_R2	15,80838776	-79,87782288	-942,1133423	-0,079546
Cloud_Mesh_Distance_BA_R0_R2	15,80370522	-80,05020142	-942,149353	-0,077039
Cloud_Mesh_Distance_BA_R0_R2	23,17565918	-81,00404358	-942,0308838	-0,15359
Cloud_Mesh_Distance_BA_R0_R2	23,17700005	-80,56576538	-942,3425903	-0,127852
Cloud_Mesh_Distance_BA_R0_R2	15,8181572	-79,69776917	-942,0804443	-0,077463
Cloud_Mesh_Distance_BA_R0_R2	23,17518234	-80,3989563	-942,3104248	-0,117889
Cloud_Mesh_Distance_BA_R0_R2	23,20341682	-80,5249176	-942,1645508	-0,127831
Cloud_Mesh_Distance_BA_R0_R2	23,19883537	-80,34867859	-942,1116943	-0,112407
Cloud_Mesh_Distance_BA_R0_R2	15,84099579	-79,46568298	-942,0368652	-0,074664
Cloud_Mesh_Distance_BA_R0_R2	15,83016014	-79,57826233	-942,0546875	-0,074344
Cloud_Mesh_Distance_BA_R0_R2	15,70811081	-79,36283875	-942,2213745	-0,095365
Cloud_Mesh_Distance_BA_R0_R2	15,84471893	-79,11026001	-942,0441895	-0,074305
Cloud_Mesh_Distance_BA_R0_R2	23,17209816	-80,186203	-942,1436157	-0,099065
Cloud_Mesh_Distance_BA_R0_R2	15,89881134	-79,04310608	-941,9804077	-0,06653
Cloud_Mesh_Distance_BA_R0_R2	15,83854485	-78,98954773	-942,0646362	-0,073923
Cloud_Mesh_Distance_BA_R0_R2	15,90254402	-79,18475342	-941,9611816	-0,065277
Cloud_Mesh_Distance_BA_R0_R2	23,14593506	-80,04582214	-942,1079712	-0,083281
Cloud_Mesh_Distance_BA_R0_R2	23,09583664	-79,95274353	-942,2972412	-0,078375
Cloud_Mesh_Distance_BA_R0_R2	15,74543953	-78,83146667	-942,2103882	-0,084095
Cloud_Mesh_Distance_BA_R0_R2	15,67376709	-78,76905823	-942,3240356	-0,094558
Cloud_Mesh_Distance_BA_R0_R2	15,83302498	-78,81307983	-942,0979004	-0,073254
Cloud_Mesh_Distance_BA_R0_R2	23,05010605	-79,79551697	-942,2523193	-0,063842
Cloud_Mesh_Distance_BA_R0_R2	23,00746346	-79,668396	-942,1644287	-0,051654
Cloud_Mesh_Distance_BA_R0_R2	23,096138	-79,87811279	-942,1133423	-0,070673
Cloud_Mesh_Distance_BA_R0_R2	15,79675484	-78,49827576	-942,2224121	-0,076273
Cloud_Mesh_Distance_BA_R0_R2	15,76805115	-78,68255615	-942,2103272	-0,081551
Cloud_Mesh_Distance_BA_R0_R2	22,86807632	-79,40013123	-942,1408081	-0,049764
Cloud_Mesh_Distance_BA_R0_R2	22,93104935	-79,53965759	-942,2750244	-0,050175
Cloud_Mesh_Distance_BA_R0_R2	15,81659317	-78,35453796	-942,2463989	-0,074333
Cloud_Mesh_Distance_BA_R0_R2	22,6862011	-79,14509583	-942,3411865	-0,047962
Cloud_Mesh_Distance_BA_R0_R2	22,71772003	-79,16183472	-942,1572266	-0,043155
Cloud_Mesh_Distance_BA_R0_R2	15,74393368	-78,38200378	-942,3404541	-0,08284
Cloud_Mesh_Distance_BA_R0_R2	15,89333344	-78,39233398	-942,1313477	-0,068801
Cloud_Mesh_Distance_BA_R0_R2	15,86353493	-78,23538208	-942,2333374	-0,069124
Cloud_Mesh_Distance_BA_R0_R2	15,94921112	-78,2177124	-942,1289063	-0,063646
Cloud_Mesh_Distance_BA_R0_R2	22,61250877	-79,02488708	-942,3074951	-0,040738
Cloud_Mesh_Distance_BA_R0_R2	15,99363995	-78,10336304	-942,126709	-0,0613
Cloud_Mesh_Distance_BA_R0_R2	22,50535202	-78,83921814	-942,1709595	-0,024187
Cloud_Mesh_Distance_BA_R0_R2	22,54855347	-78,93519592	-942,3508301	-0,037752
Cloud_Mesh_Distance_BA_R0_R2	22,64211082	-79,04606628	-942,1604004	-0,03778
Cloud_Mesh_Distance_BA_R0_R2	22,57860947	-78,95773315	-942,218811	-0,03407
Cloud_Mesh_Distance_BA_R0_R2	15,99837303	-78,00492859	-942,1715088	-0,058773
Cloud_Mesh_Distance_BA_R0_R2	22,40704918	-78,71038818	-942,2918091	-0,020417
Cloud_Mesh_Distance_BA_R0_R2	16,08226395	-77,61979675	-942,3167725	-0,049741

Cloud_Mesh_Distance_BA_R0_R2	16,04602051	-77,86679077	-942,190979	-0,056085
Cloud_Mesh_Distance_BA_R0_R2	16,15205383	-77,67420959	-942,1903687	-0,049557
Cloud_Mesh_Distance_BA_R0_R2	16,17401314	-77,8825531	-942,0299683	-0,054835
Cloud_Mesh_Distance_BA_R0_R2	22,26018143	-78,48039246	-942,1611328	0,003405
Cloud_Mesh_Distance_BA_R0_R2	22,14247704	-78,36251831	-942,4138184	0,008055
Cloud_Mesh_Distance_BA_R0_R2	16,11788368	-77,52780151	-942,3460693	-0,044581
Cloud_Mesh_Distance_BA_R0_R2	16,18305969	-77,42420959	-942,3554077	-0,041539
Cloud_Mesh_Distance_BA_R0_R2	16,2139225	-77,50642395	-942,2456665	-0,041998
Cloud_Mesh_Distance_BA_R0_R2	21,96265793	-78,1237793	-942,3537598	0,020927
Cloud_Mesh_Distance_BA_R0_R2	22,05169487	-78,22927856	-942,3276978	0,015044
Cloud_Mesh_Distance_BA_R0_R2	16,24271011	-77,42066956	-942,2857056	-0,039428
Cloud_Mesh_Distance_BA_R0_R2	16,38477898	-77,3343811	-942,1975708	-0,036062
Cloud_Mesh_Distance_BA_R0_R2	21,89948654	-78,02978516	-942,151062	0,022405
Cloud_Mesh_Distance_BA_R0_R2	21,97624969	-78,12315369	-942,2175293	0,020008
Cloud_Mesh_Distance_BA_R0_R2	16,46539497	-77,2252655	-942,2149658	-0,027774
Cloud_Mesh_Distance_BA_R0_R2	21,62057495	-77,72637939	-942,164917	0,026694
Cloud_Mesh_Distance_BA_R0_R2	21,69034004	-77,81230164	-942,3527222	0,02512
Cloud_Mesh_Distance_BA_R0_R2	21,8133564	-77,96522522	-942,4459839	0,01965
Cloud_Mesh_Distance_BA_R0_R2	21,81268311	-77,93710327	-942,2417603	0,025231
Cloud_Mesh_Distance_BA_R0_R2	16,50304794	-77,13858032	-942,269104	-0,028065
Cloud_Mesh_Distance_BA_R0_R2	16,73329353	-76,99082947	-942,1907959	-0,013695
Cloud_Mesh_Distance_BA_R0_R2	16,94707489	-76,84133911	-942,1777344	-0,008976
Cloud_Mesh_Distance_BA_R0_R2	16,83463669	-76,84344482	-942,2840576	-0,01141
Cloud_Mesh_Distance_BA_R0_R2	21,59731674	-77,70848083	-942,3267822	0,024533
Cloud_Mesh_Distance_BA_R0_R2	21,54210663	-77,65888977	-942,4187012	0,023396
Cloud_Mesh_Distance_BA_R0_R2	21,44275475	-77,55438232	-942,4259033	0,023555
Cloud_Mesh_Distance_BA_R0_R2	16,77470589	-76,78900146	-942,4243774	-0,013188
Cloud_Mesh_Distance_BA_R0_R2	21,23829842	-77,34216309	-942,3173218	0,024713
Cloud_Mesh_Distance_BA_R0_R2	21,32289505	-77,42326355	-942,2000122	0,026291
Cloud_Mesh_Distance_BA_R0_R2	16,97114754	-76,76416016	-942,2668457	-0,010952
Cloud_Mesh_Distance_BA_R0_R2	17,00662994	-76,65261841	-942,4057007	-0,007567
Cloud_Mesh_Distance_BA_R0_R2	17,19793701	-76,59281921	-942,3381958	-0,002636
Cloud_Mesh_Distance_BA_R0_R2	17,48033142	-76,58551025	-942,1343384	0,00231
Cloud_Mesh_Distance_BA_R0_R2	21,12598991	-77,23942566	-942,2404175	0,026383
Cloud_Mesh_Distance_BA_R0_R2	21,13678551	-77,25242615	-942,4450073	0,023639
Cloud_Mesh_Distance_BA_R0_R2	17,33082771	-76,55325317	-942,3009033	-0,00477
Cloud_Mesh_Distance_BA_R0_R2	17,55524063	-76,51745605	-942,1965332	0,004579
Cloud_Mesh_Distance_BA_R0_R2	17,42440414	-76,55506897	-942,2278442	-0,001405
Cloud_Mesh_Distance_BA_R0_R2	17,42472076	-76,49395752	-942,331665	-0,000547
Cloud_Mesh_Distance_BA_R0_R2	17,73314285	-76,44503784	-942,2006836	0,013127
Cloud_Mesh_Distance_BA_R0_R2	20,49025345	-76,78616333	-942,3435059	-0,010423
Cloud_Mesh_Distance_BA_R0_R2	20,62068748	-76,86065674	-942,3410034	0,001496
Cloud_Mesh_Distance_BA_R0_R2	20,7715683	-76,9540863	-942,4186401	0,009518
Cloud_Mesh_Distance_BA_R0_R2	20,88092041	-77,03755188	-942,289978	0,019149
Cloud_Mesh_Distance_BA_R0_R2	17,86561584	-76,35725403	-942,2818604	0,018848
Cloud_Mesh_Distance_BA_R0_R2	18,01931763	-76,37513733	-942,1495972	0,034669
Cloud_Mesh_Distance_BA_R0_R2	18,03338242	-76,295578	-942,3077393	0,029115
Cloud_Mesh_Distance_BA_R0_R2	18,16983604	-76,33241272	-942,1571655	0,042401
Cloud_Mesh_Distance_BA_R0_R2	20,03376007	-76,58195496	-942,2744751	-0,032669
Cloud_Mesh_Distance_BA_R0_R2	20,12004089	-76,59979248	-942,3910523	-0,035187

Cloud_Mesh_Distance_BA_R0_R2	20,2688427	-76,68069458	-942,3010864	-0,023433
Cloud_Mesh_Distance_BA_R0_R2	20,5245266	-76,79467773	-942,4900513	-0,008468
Cloud_Mesh_Distance_BA_R0_R2	17,88894081	-76,29486084	-942,3943481	0,021417
Cloud_Mesh_Distance_BA_R0_R2	17,94213867	-76,23959351	-942,4824219	0,021464
Cloud_Mesh_Distance_BA_R0_R2	18,35787964	-76,17132568	-942,4458618	0,047985
Cloud_Mesh_Distance_BA_R0_R2	18,16863632	-76,27813721	-942,2736816	0,039919
Cloud_Mesh_Distance_BA_R0_R2	18,2938652	-76,28077698	-942,2142944	0,046375
Cloud_Mesh_Distance_BA_R0_R2	18,56482506	-76,14117432	-942,4824829	0,055606
Cloud_Mesh_Distance_BA_R0_R2	18,6144352	-76,19667053	-942,340332	0,053549
Cloud_Mesh_Distance_BA_R0_R2	18,58348274	-76,25444031	-942,204834	0,050959
Cloud_Mesh_Distance_BA_R0_R2	18,92536163	-76,20161438	-942,3796997	0,041957
Cloud_Mesh_Distance_BA_R0_R2	18,80032539	-76,20239258	-942,342041	0,048777
Cloud_Mesh_Distance_BA_R0_R2	19,1771698	-76,28311157	-942,2895508	0,017703
Cloud_Mesh_Distance_BA_R0_R2	19,51171875	-76,39710999	-942,2324829	-0,014384
Cloud_Mesh_Distance_BA_R0_R2	19,32072258	-76,35484314	-942,1888428	0,001138
Cloud_Mesh_Distance_BA_R0_R2	19,71315575	-76,45379639	-942,2807617	-0,029011
Cloud_Mesh_Distance_BA_R0_R2	19,90432358	-76,53146362	-942,2653198	-0,036593
Cloud_Mesh_Distance_BA_R0_R2	19,98328972	-76,54177856	-942,3762817	-0,034698
Cloud_Mesh_Distance_BA_R0_R2	19,84800911	-76,47233582	-942,4396973	-0,037157
Cloud_Mesh_Distance_BA_R0_R2	19,69101906	-76,40661621	-942,4433594	-0,02731
Cloud_Mesh_Distance_BA_R0_R2	18,47798729	-76,17036438	-942,4185791	0,054496
Cloud_Mesh_Distance_BA_R0_R2	18,96452904	-76,16938782	-942,4892578	0,039775
Cloud_Mesh_Distance_BA_R0_R2	19,13635445	-76,19078064	-942,5396729	0,030401
Cloud_Mesh_Distance_BA_R0_R2	19,06204605	-76,22161865	-942,390686	0,030093
Cloud_Mesh_Distance_BA_R0_R2	19,28561401	-76,24102783	-942,5146484	0,01164
Cloud_Mesh_Distance_BA_R0_R2	19,20842171	-76,24633789	-942,4213867	0,014528
Cloud_Mesh_Distance_BA_R0_R2	18,36177444	-84,13877869	-941,8377686	0,01291
Cloud_Mesh_Distance_BA_R0_R2	18,40741539	-84,14605713	-941,7389526	0,012379
Cloud_Mesh_Distance_BA_R0_R2	18,45850754	-84,20278931	-941,8288574	0,019893
Cloud_Mesh_Distance_BA_R0_R2	18,65446472	-84,25816345	-941,5739746	0,014792
Cloud_Mesh_Distance_BA_R0_R2	18,51334572	-84,19807434	-941,668335	0,014658
Cloud_Mesh_Distance_BA_R0_R2	18,76506805	-84,34317017	-941,6573486	0,025526
Cloud_Mesh_Distance_BA_R0_R2	18,29655647	-84,07188416	-941,7564087	0,004695
Cloud_Mesh_Distance_BA_R0_R2	18,16615295	-83,96150208	-941,7109985	-0,008111
Cloud_Mesh_Distance_BA_R0_R2	18,41344643	-84,11512756	-941,6047363	0,005261
Cloud_Mesh_Distance_BA_R0_R2	17,94752884	-83,78387451	-941,7265625	-0,028873
Cloud_Mesh_Distance_BA_R0_R2	18,01683807	-83,87695313	-941,8511353	-0,016468
Cloud_Mesh_Distance_BA_R0_R2	18,06276131	-83,85955811	-941,6450195	-0,02254
Cloud_Mesh_Distance_BA_R0_R2	17,57360268	-83,40686035	-941,7005615	-0,069684
Cloud_Mesh_Distance_BA_R0_R2	17,74569321	-83,6192627	-941,8035889	-0,047403
Cloud_Mesh_Distance_BA_R0_R2	17,85405731	-83,71929932	-941,7944946	-0,034346
Cloud_Mesh_Distance_BA_R0_R2	17,86725616	-83,68722534	-941,6472168	-0,043943
Cloud_Mesh_Distance_BA_R0_R2	17,49104118	-83,36212158	-941,8357544	-0,069922
Cloud_Mesh_Distance_BA_R0_R2	17,38705444	-83,22128296	-941,7890015	-0,085611
Cloud_Mesh_Distance_BA_R0_R2	17,5388031	-83,34265137	-941,6306763	-0,078814
Cloud_Mesh_Distance_BA_R0_R2	17,36849022	-83,15840149	-941,6879273	-0,091288
Cloud_Mesh_Distance_BA_R0_R2	17,22291946	-82,98109436	-941,7437134	-0,106384
Cloud_Mesh_Distance_BA_R0_R2	17,30931091	-83,06130981	-941,6433716	-0,10035
Cloud_Mesh_Distance_BA_R0_R2	17,00510406	-82,6061554	-941,6919556	-0,12811
Cloud_Mesh_Distance_BA_R0_R2	17,07295418	-82,69821167	-941,6405029	-0,122249

Cloud_Mesh_Distance_BA_R0_R2	17,06007385	-82,74163818	-941,7785034	-0,119786
Cloud_Mesh_Distance_BA_R0_R2	17,14706421	-82,82585144	-941,6539917	-0,116173
Cloud_Mesh_Distance_BA_R0_R2	16,87795258	-82,47221375	-941,8762817	-0,12874
Cloud_Mesh_Distance_BA_R0_R2	16,84863663	-82,37759399	-941,7943726	-0,126867
Cloud_Mesh_Distance_BA_R0_R2	16,85582733	-82,33383179	-941,6834106	-0,125655
Cloud_Mesh_Distance_BA_R0_R2	16,65427017	-82,0958252	-941,9246826	-0,110764
Cloud_Mesh_Distance_BA_R0_R2	16,70800591	-82,11817932	-941,7903442	-0,112736
Cloud_Mesh_Distance_BA_R0_R2	16,64575195	-82,02593994	-941,8317261	-0,102613
Cloud_Mesh_Distance_BA_R0_R2	16,58952522	-81,98298645	-941,935669	-0,096898
Cloud_Mesh_Distance_BA_R0_R2	16,66958427	-82,00050354	-941,710144	-0,0967
Cloud_Mesh_Distance_BA_R0_R2	16,35388565	-81,32279968	-941,7633057	-0,050983
Cloud_Mesh_Distance_BA_R0_R2	23,05680084	-82,32699585	-941,8328247	0,132175
Cloud_Mesh_Distance_BA_R0_R2	23,02310944	-82,19586182	-941,8178711	0,067516
Cloud_Mesh_Distance_BA_R0_R2	23,07662964	-82,16430664	-941,9683228	0,090325
Cloud_Mesh_Distance_BA_R0_R2	23,02144814	-82,06048584	-941,8569336	0,020081
Cloud_Mesh_Distance_BA_R0_R2	16,25745773	-81,00517273	-941,7384644	-0,032235
Cloud_Mesh_Distance_BA_R0_R2	16,19807053	-80,91000366	-941,8018799	-0,034888
Cloud_Mesh_Distance_BA_R0_R2	16,21474266	-81,1076355	-941,9130249	-0,050572
Cloud_Mesh_Distance_BA_R0_R2	23,02692986	-81,90307617	-941,9024048	-0,026794
Cloud_Mesh_Distance_BA_R0_R2	23,05799294	-81,85865784	-942,0394287	-0,021548
Cloud_Mesh_Distance_BA_R0_R2	23,04339409	-81,77554321	-941,9749146	-0,059349
Cloud_Mesh_Distance_BA_R0_R2	16,07884979	-80,699646	-941,911377	-0,036872
Cloud_Mesh_Distance_BA_R0_R2	16,07203484	-80,51431274	-941,8318481	-0,027489
Cloud_Mesh_Distance_BA_R0_R2	16,08132362	-80,335495	-941,7479248	-0,013273
Cloud_Mesh_Distance_BA_R0_R2	23,11769676	-81,28068542	-941,9054565	-0,149087
Cloud_Mesh_Distance_BA_R0_R2	23,11854362	-81,27992249	-942,0615234	-0,135956
Cloud_Mesh_Distance_BA_R0_R2	15,99316788	-80,40071106	-941,9299927	-0,038648
Cloud_Mesh_Distance_BA_R0_R2	23,1530571	-81,13348389	-941,866333	-0,156827
Cloud_Mesh_Distance_BA_R0_R2	23,18785095	-80,97589111	-941,8325195	-0,165163
Cloud_Mesh_Distance_BA_R0_R2	15,94691277	-80,08973694	-941,9244995	-0,046846
Cloud_Mesh_Distance_BA_R0_R2	15,87935543	-79,97943115	-942,0165405	-0,061714
Cloud_Mesh_Distance_BA_R0_R2	16,01540184	-80,20762634	-941,8312988	-0,028751
Cloud_Mesh_Distance_BA_R0_R2	15,96033096	-79,90596008	-941,8757324	-0,038745
Cloud_Mesh_Distance_BA_R0_R2	23,20721817	-80,81288147	-941,9890137	-0,151738
Cloud_Mesh_Distance_BA_R0_R2	23,21208763	-80,83755493	-941,788269	-0,169302
Cloud_Mesh_Distance_BA_R0_R2	15,97486591	-79,72807312	-941,8406372	-0,0409
Cloud_Mesh_Distance_BA_R0_R2	15,89176559	-79,79563904	-941,975647	-0,058337
Cloud_Mesh_Distance_BA_R0_R2	16,02855301	-80,01661682	-941,7717285	-0,022265
Cloud_Mesh_Distance_BA_R0_R2	23,21700668	-80,640625	-942,0348511	-0,13919
Cloud_Mesh_Distance_BA_R0_R2	23,22193909	-80,50819397	-941,9857178	-0,134824
Cloud_Mesh_Distance_BA_R0_R2	16,05394745	-79,64125061	-941,7092896	-0,019346
Cloud_Mesh_Distance_BA_R0_R2	23,22825432	-80,66389465	-941,8400879	-0,157209
Cloud_Mesh_Distance_BA_R0_R2	15,7674942	-79,37562561	-942,1383057	-0,086647
Cloud_Mesh_Distance_BA_R0_R2	15,9365406	-79,36639404	-941,9026489	-0,057529
Cloud_Mesh_Distance_BA_R0_R2	15,85110664	-79,3235321	-942,0238648	-0,07226
Cloud_Mesh_Distance_BA_R0_R2	15,91104603	-79,59127808	-941,9364014	-0,057836
Cloud_Mesh_Distance_BA_R0_R2	15,98797035	-79,61245728	-941,8178711	-0,038719
Cloud_Mesh_Distance_BA_R0_R2	16,00910378	-79,49621582	-941,7879639	-0,035126
Cloud_Mesh_Distance_BA_R0_R2	23,22385025	-80,3664856	-941,8206787	-0,117176
Cloud_Mesh_Distance_BA_R0_R2	23,21920013	-80,384552	-941,9352417	-0,116625

Cloud_Mesh_Distance_BA_R0_R2	23,23165131	-80,48851013	-941,821106	-0,136222
Cloud_Mesh_Distance_BA_R0_R2	15,80843449	-79,2247467	-942,0861816	-0,079231
Cloud_Mesh_Distance_BA_R0_R2	23,19799614	-80,21710205	-941,9313965	-0,099591
Cloud_Mesh_Distance_BA_R0_R2	15,919981	-78,90068054	-941,97229	-0,064786
Cloud_Mesh_Distance_BA_R0_R2	23,06562042	-79,76603699	-941,9929199	-0,058695
Cloud_Mesh_Distance_BA_R0_R2	16,0208683	-78,8157959	-941,8605957	-0,055666
Cloud_Mesh_Distance_BA_R0_R2	15,98759556	-79,02420044	-941,8669434	-0,055693
Cloud_Mesh_Distance_BA_R0_R2	15,94277382	-78,71128845	-941,9796143	-0,063803
Cloud_Mesh_Distance_BA_R0_R2	22,99677658	-79,61181641	-941,9851074	-0,051227
Cloud_Mesh_Distance_BA_R0_R2	23,05544281	-79,77241516	-942,1328735	-0,059111
Cloud_Mesh_Distance_BA_R0_R2	15,9722271	-78,51156616	-941,9949341	-0,062452
Cloud_Mesh_Distance_BA_R0_R2	15,86564445	-78,60298157	-942,1026001	-0,070798
Cloud_Mesh_Distance_BA_R0_R2	16,05083847	-78,61952209	-941,8699341	-0,056571
Cloud_Mesh_Distance_BA_R0_R2	22,94719124	-79,53405762	-942,1049805	-0,048059
Cloud_Mesh_Distance_BA_R0_R2	22,8477993	-79,34410095	-941,9624023	-0,041693
Cloud_Mesh_Distance_BA_R0_R2	16,0844326	-78,42114258	-941,8893433	-0,059114
Cloud_Mesh_Distance_BA_R0_R2	22,69312477	-79,10301208	-941,9671631	-0,035051
Cloud_Mesh_Distance_BA_R0_R2	22,78297615	-79,2509613	-942,0599365	-0,045113
Cloud_Mesh_Distance_BA_R0_R2	16,00540161	-78,32209778	-942,0181274	-0,061891
Cloud_Mesh_Distance_BA_R0_R2	22,61885071	-78,99482727	-942,0231934	-0,033797
Cloud_Mesh_Distance_BA_R0_R2	16,02703476	-78,1791687	-942,0511475	-0,06106
Cloud_Mesh_Distance_BA_R0_R2	16,07613182	-78,04167175	-942,0575562	-0,056713
Cloud_Mesh_Distance_BA_R0_R2	22,58096123	-78,94615173	-942,1080322	-0,030933
Cloud_Mesh_Distance_BA_R0_R2	22,47996902	-78,78118896	-941,9728394	-0,013078
Cloud_Mesh_Distance_BA_R0_R2	16,36884499	-77,81842041	-941,8456421	-0,045291
Cloud_Mesh_Distance_BA_R0_R2	16,29419708	-77,74203491	-941,9786987	-0,046048
Cloud_Mesh_Distance_BA_R0_R2	22,31505775	-78,54818726	-942,069458	0,003404
Cloud_Mesh_Distance_BA_R0_R2	22,32666588	-78,58222961	-942,2264404	-0,006484
Cloud_Mesh_Distance_BA_R0_R2	22,33564377	-78,57017517	-941,9034424	0,004517
Cloud_Mesh_Distance_BA_R0_R2	22,4016819	-78,6729126	-942,0728149	-0,00453
Cloud_Mesh_Distance_BA_R0_R2	16,29993057	-77,43380737	-942,2057495	-0,038298
Cloud_Mesh_Distance_BA_R0_R2	16,29069519	-77,56878662	-942,1048584	-0,042385
Cloud_Mesh_Distance_BA_R0_R2	16,39317513	-77,60838318	-941,9584351	-0,043061
Cloud_Mesh_Distance_BA_R0_R2	22,1668129	-78,35855103	-942,1906128	0,010177
Cloud_Mesh_Distance_BA_R0_R2	22,06267166	-78,22045898	-942,1137085	0,019594
Cloud_Mesh_Distance_BA_R0_R2	16,3971138	-77,46064758	-942,0698853	-0,035587
Cloud_Mesh_Distance_BA_R0_R2	16,50095367	-77,33181763	-942,0674439	-0,030146
Cloud_Mesh_Distance_BA_R0_R2	21,96961403	-78,10824585	-942,0529785	0,02103
Cloud_Mesh_Distance_BA_R0_R2	21,90340042	-78,0330658	-942,0378418	0,021154
Cloud_Mesh_Distance_BA_R0_R2	16,65101242	-77,3266449	-941,9068604	-0,020957
Cloud_Mesh_Distance_BA_R0_R2	16,72116852	-77,19294739	-941,9660645	-0,0136
Cloud_Mesh_Distance_BA_R0_R2	21,57603455	-77,68959045	-941,9763794	0,021636
Cloud_Mesh_Distance_BA_R0_R2	21,52409744	-77,62776184	-942,2808228	0,026976
Cloud_Mesh_Distance_BA_R0_R2	21,73216248	-77,84495544	-942,1522827	0,023722
Cloud_Mesh_Distance_BA_R0_R2	16,60171127	-77,1625061	-942,1292114	-0,020932
Cloud_Mesh_Distance_BA_R0_R2	16,89717484	-76,92147827	-942,1149902	-0,007872
Cloud_Mesh_Distance_BA_R0_R2	16,7468586	-77,10351563	-942,0393066	-0,014437
Cloud_Mesh_Distance_BA_R0_R2	17,10139656	-76,86862183	-942,0001831	0,001017
Cloud_Mesh_Distance_BA_R0_R2	17,01176453	-76,94892883	-941,9708862	-0,001791
Cloud_Mesh_Distance_BA_R0_R2	21,50330162	-77,60604858	-942,1065674	0,027878

Cloud_Mesh_Distance_BA_R0_R2	16,99883461	-76,87626648	-942,0812378	-0,004778
Cloud_Mesh_Distance_BA_R0_R2	17,13560104	-76,93695068	-941,8757324	0,006719
Cloud_Mesh_Distance_BA_R0_R2	17,17412567	-76,79518127	-942,0426636	-0,000171
Cloud_Mesh_Distance_BA_R0_R2	21,22575378	-77,33352661	-942,1466065	0,026221
Cloud_Mesh_Distance_BA_R0_R2	21,2226963	-77,34054565	-942,0303345	0,022764
Cloud_Mesh_Distance_BA_R0_R2	21,42462158	-77,52401733	-942,2419434	0,028431
Cloud_Mesh_Distance_BA_R0_R2	21,2935276	-77,40412903	-942,0477905	0,022298
Cloud_Mesh_Distance_BA_R0_R2	17,05318832	-76,80796814	-942,1295776	-0,006048
Cloud_Mesh_Distance_BA_R0_R2	17,1494503	-76,70941162	-942,1933594	-0,004828
Cloud_Mesh_Distance_BA_R0_R2	17,32985497	-76,74038696	-941,9984131	0,005776
Cloud_Mesh_Distance_BA_R0_R2	17,34313202	-76,62602234	-942,1707153	-0,001279
Cloud_Mesh_Distance_BA_R0_R2	17,64982605	-76,60453796	-941,9748535	0,020392
Cloud_Mesh_Distance_BA_R0_R2	20,84958076	-77,02734375	-942,1276245	0,018802
Cloud_Mesh_Distance_BA_R0_R2	20,98491859	-77,12802124	-942,1341553	0,021109
Cloud_Mesh_Distance_BA_R0_R2	17,69262886	-76,5362854	-942,0634155	0,017196
Cloud_Mesh_Distance_BA_R0_R2	17,57701111	-76,57879639	-942,0734253	0,010077
Cloud_Mesh_Distance_BA_R0_R2	17,89561272	-76,42098999	-942,1356201	0,026237
Cloud_Mesh_Distance_BA_R0_R2	17,99404526	-76,44046021	-942,03479	0,036528
Cloud_Mesh_Distance_BA_R0_R2	17,86240768	-76,49591064	-942,015686	0,028386
Cloud_Mesh_Distance_BA_R0_R2	20,35554886	-76,73934937	-942,1783447	-0,015248
Cloud_Mesh_Distance_BA_R0_R2	20,50405502	-76,81307983	-942,173523	-0,00603
Cloud_Mesh_Distance_BA_R0_R2	20,62752914	-76,87896729	-942,1873779	0,004424
Cloud_Mesh_Distance_BA_R0_R2	20,50558472	-76,83683777	-942,0217896	-0,002946
Cloud_Mesh_Distance_BA_R0_R2	20,76608658	-76,98019409	-942,0528565	0,014359
Cloud_Mesh_Distance_BA_R0_R2	20,73985672	-76,94572449	-942,2061768	0,00947
Cloud_Mesh_Distance_BA_R0_R2	18,15421677	-76,41716003	-941,9886475	0,046474
Cloud_Mesh_Distance_BA_R0_R2	18,35103226	-76,36343384	-942,0151367	0,054697
Cloud_Mesh_Distance_BA_R0_R2	18,79393578	-76,32151794	-942,055542	0,043179
Cloud_Mesh_Distance_BA_R0_R2	19,44265747	-76,42941284	-942,0702515	-0,00956
Cloud_Mesh_Distance_BA_R0_R2	19,55698395	-76,50474548	-941,942749	-0,013501
Cloud_Mesh_Distance_BA_R0_R2	19,74987411	-76,55761719	-941,9580689	-0,023919
Cloud_Mesh_Distance_BA_R0_R2	20,02240181	-76,61244202	-942,1106567	-0,033212
Cloud_Mesh_Distance_BA_R0_R2	19,82062912	-76,5358429	-942,1170654	-0,029792
Cloud_Mesh_Distance_BA_R0_R2	20,2214756	-76,68807983	-942,130249	-0,024449
Cloud_Mesh_Distance_BA_R0_R2	20,13460732	-76,63104248	-942,2389526	-0,029182
Cloud_Mesh_Distance_BA_R0_R2	20,4029789	-76,74728394	-942,284729	-0,015072
Cloud_Mesh_Distance_BA_R0_R2	18,42444992	-76,22192383	-942,3066406	0,050435
Cloud_Mesh_Distance_BA_R0_R2	18,47789383	-76,29598999	-942,12677	0,051841
Cloud_Mesh_Distance_BA_R0_R2	18,65476608	-76,30865479	-942,0775147	0,052389
Cloud_Mesh_Distance_BA_R0_R2	18,75978279	-76,26170349	-942,1907349	0,043267
Cloud_Mesh_Distance_BA_R0_R2	18,96613693	-76,25630188	-942,2526245	0,035215
Cloud_Mesh_Distance_BA_R0_R2	19,63353539	-76,47743225	-942,0988159	-0,021648
Cloud_Mesh_Distance_BA_R0_R2	19,06423187	-84,43327332	-941,4963379	0,022125
Cloud_Mesh_Distance_BA_R0_R2	18,91353226	-84,39146423	-941,5682983	0,0243
Cloud_Mesh_Distance_BA_R0_R2	18,80755615	-84,31156921	-941,4736939	0,015231
Cloud_Mesh_Distance_BA_R0_R2	20,41987228	-84,54473877	-941,4202881	0,060348
Cloud_Mesh_Distance_BA_R0_R2	20,24106598	-84,52693176	-941,3646851	0,030521
Cloud_Mesh_Distance_BA_R0_R2	20,61016846	-84,53355408	-941,4285278	0,10672
Cloud_Mesh_Distance_BA_R0_R2	20,7773819	-84,51750183	-941,427124	0,158579
Cloud_Mesh_Distance_BA_R0_R2	20,94762421	-84,5018158	-941,4276733	0,171899

Cloud_Mesh_Distance_BA_R0_R2	21,2268486	-84,50737	-941,4585571	0,250765
Cloud_Mesh_Distance_BA_R0_R2	21,36295891	-84,51411438	-941,4717407	0,28905
Cloud_Mesh_Distance_BA_R0_R2	18,16346359	-83,86108398	-941,37677	-0,028574
Cloud_Mesh_Distance_BA_R0_R2	18,08840752	-83,83795166	-941,5002441	-0,029762
Cloud_Mesh_Distance_BA_R0_R2	18,1783886	-83,91856384	-941,5239258	-0,017576
Cloud_Mesh_Distance_BA_R0_R2	18,2962532	-84,01939392	-941,560791	-0,005752
Cloud_Mesh_Distance_BA_R0_R2	18,54627991	-84,16978455	-941,4906006	0,004126
Cloud_Mesh_Distance_BA_R0_R2	18,4319973	-84,07516479	-941,4216309	-0,005056
Cloud_Mesh_Distance_BA_R0_R2	21,03229141	-84,41943359	-941,3341065	0,172256
Cloud_Mesh_Distance_BA_R0_R2	21,59955597	-84,47903442	-941,4371948	0,342314
Cloud_Mesh_Distance_BA_R0_R2	17,80950546	-83,53044128	-941,3511353	-0,062512
Cloud_Mesh_Distance_BA_R0_R2	17,70124245	-83,4546814	-941,4485474	-0,069133
Cloud_Mesh_Distance_BA_R0_R2	17,75669289	-83,55575562	-941,5731201	-0,058572
Cloud_Mesh_Distance_BA_R0_R2	17,49593544	-83,25541687	-941,5316773	-0,087033
Cloud_Mesh_Distance_BA_R0_R2	17,63269234	-83,43286133	-941,5910034	-0,069029
Cloud_Mesh_Distance_BA_R0_R2	17,4459343	-83,24822998	-941,6704102	-0,087329
Cloud_Mesh_Distance_BA_R0_R2	17,24475479	-82,94091797	-941,5765381	-0,110737
Cloud_Mesh_Distance_BA_R0_R2	17,32159996	-83,02241516	-941,5093384	-0,106286
Cloud_Mesh_Distance_BA_R0_R2	17,19380188	-82,817276	-941,4725952	-0,114728
Cloud_Mesh_Distance_BA_R0_R2	17,04382133	-82,61160278	-941,5623169	-0,127272
Cloud_Mesh_Distance_BA_R0_R2	17,05340004	-82,55989075	-941,4187012	-0,121966
Cloud_Mesh_Distance_BA_R0_R2	16,78978157	-82,2532196	-941,7606201	-0,123091
Cloud_Mesh_Distance_BA_R0_R2	16,89696884	-82,34269714	-941,5496826	-0,121292
Cloud_Mesh_Distance_BA_R0_R2	16,83012581	-82,24156189	-941,5953369	-0,117756
Cloud_Mesh_Distance_BA_R0_R2	16,91707802	-82,46340942	-941,7200317	-0,127788
Cloud_Mesh_Distance_BA_R0_R2	16,97964478	-82,49151611	-941,5474243	-0,124869
Cloud_Mesh_Distance_BA_R0_R2	16,75844574	-82,10964966	-941,5999146	-0,105123
Cloud_Mesh_Distance_BA_R0_R2	16,57039642	-81,70970154	-941,581543	-0,068981
Cloud_Mesh_Distance_BA_R0_R2	16,53554916	-81,76651001	-941,7675171	-0,07487
Cloud_Mesh_Distance_BA_R0_R2	16,61729431	-81,86399841	-941,6567383	-0,080722
Cloud_Mesh_Distance_BA_R0_R2	16,6888237	-81,95613098	-941,5681763	-0,089356
Cloud_Mesh_Distance_BA_R0_R2	23,02735519	-82,59130859	-941,6292725	0,212401
Cloud_Mesh_Distance_BA_R0_R2	22,96566963	-82,63825989	-941,5186157	0,204124
Cloud_Mesh_Distance_BA_R0_R2	23,11495972	-82,53700256	-941,8069458	0,222288
Cloud_Mesh_Distance_BA_R0_R2	16,42092896	-81,41635132	-941,6768189	-0,04996
Cloud_Mesh_Distance_BA_R0_R2	16,4082489	-81,30012512	-941,6012573	-0,037733
Cloud_Mesh_Distance_BA_R0_R2	16,48031235	-81,57337952	-941,680542	-0,058649
Cloud_Mesh_Distance_BA_R0_R2	22,92748642	-82,30528259	-941,5766602	0,069399
Cloud_Mesh_Distance_BA_R0_R2	22,99551773	-82,28144836	-941,7236328	0,083896
Cloud_Mesh_Distance_BA_R0_R2	23,00659943	-82,42655945	-941,6843262	0,143918
Cloud_Mesh_Distance_BA_R0_R2	16,29657936	-81,14056396	-941,7492065	-0,040102
Cloud_Mesh_Distance_BA_R0_R2	16,46990967	-81,38514709	-941,5110474	-0,041878
Cloud_Mesh_Distance_BA_R0_R2	22,97483253	-82,15206909	-941,7106934	0,028025
Cloud_Mesh_Distance_BA_R0_R2	22,91446686	-82,09593201	-941,5608521	-0,014183
Cloud_Mesh_Distance_BA_R0_R2	22,97654152	-81,97581482	-941,7287598	-0,029607
Cloud_Mesh_Distance_BA_R0_R2	22,93008423	-81,87242126	-941,5391846	-0,081744
Cloud_Mesh_Distance_BA_R0_R2	16,25956726	-80,89970398	-941,6605225	-0,023834
Cloud_Mesh_Distance_BA_R0_R2	23,00805855	-81,77798462	-941,795105	-0,079048
Cloud_Mesh_Distance_BA_R0_R2	22,98799896	-81,68969727	-941,6328125	-0,117615
Cloud_Mesh_Distance_BA_R0_R2	23,03968811	-81,58132935	-941,8015137	-0,123479

Cloud_Mesh_Distance_BA_R0_R2	16,13441086	-80,6008606	-941,7526245	-0,023839
Cloud_Mesh_Distance_BA_R0_R2	16,16286659	-80,73828125	-941,7683716	-0,030294
Cloud_Mesh_Distance_BA_R0_R2	16,23406601	-80,779953	-941,6432495	-0,023217
Cloud_Mesh_Distance_BA_R0_R2	16,1463356	-80,45449829	-941,666626	-0,007883
Cloud_Mesh_Distance_BA_R0_R2	16,21001816	-80,59883118	-941,5997925	-0,010089
Cloud_Mesh_Distance_BA_R0_R2	23,07518768	-81,41404724	-941,7609863	-0,142566
Cloud_Mesh_Distance_BA_R0_R2	16,1510849	-80,30168152	-941,6049805	-0,000623
Cloud_Mesh_Distance_BA_R0_R2	23,1554985	-81,10462952	-941,6810303	-0,170338
Cloud_Mesh_Distance_BA_R0_R2	16,09629822	-79,93632507	-941,6411743	-0,001853
Cloud_Mesh_Distance_BA_R0_R2	16,04090881	-79,83001709	-941,7329102	-0,018328
Cloud_Mesh_Distance_BA_R0_R2	23,21844673	-80,77680969	-941,6367188	-0,172883
Cloud_Mesh_Distance_BA_R0_R2	16,13232422	-79,75837708	-941,5699463	0,00525
Cloud_Mesh_Distance_BA_R0_R2	23,22036743	-80,31315613	-941,6972046	-0,118335
Cloud_Mesh_Distance_BA_R0_R2	23,2303257	-80,43659973	-941,614502	-0,140853
Cloud_Mesh_Distance_BA_R0_R2	23,23156166	-80,43522644	-941,7155762	-0,13272
Cloud_Mesh_Distance_BA_R0_R2	23,23324203	-80,57637024	-941,628479	-0,158876
Cloud_Mesh_Distance_BA_R0_R2	16,04907608	-79,31083679	-941,7428589	-0,033721
Cloud_Mesh_Distance_BA_R0_R2	16,11161995	-79,46551514	-941,6257324	-0,011485
Cloud_Mesh_Distance_BA_R0_R2	16,12003326	-79,6098175	-941,5974121	-0,000879
Cloud_Mesh_Distance_BA_R0_R2	15,99167824	-79,19889832	-941,8387451	-0,050329
Cloud_Mesh_Distance_BA_R0_R2	23,14309692	-79,96867371	-941,8831787	-0,074934
Cloud_Mesh_Distance_BA_R0_R2	16,08065987	-79,11218262	-941,7252808	-0,03613
Cloud_Mesh_Distance_BA_R0_R2	23,09244156	-79,81098938	-941,8272705	-0,059883
Cloud_Mesh_Distance_BA_R0_R2	23,07930374	-79,77310181	-941,7454834	-0,057872
Cloud_Mesh_Distance_BA_R0_R2	16,0990696	-78,91490173	-941,737854	-0,042575
Cloud_Mesh_Distance_BA_R0_R2	22,92431259	-79,47164917	-941,9489746	-0,045124
Cloud_Mesh_Distance_BA_R0_R2	22,94389534	-79,49554443	-941,7939453	-0,039557
Cloud_Mesh_Distance_BA_R0_R2	22,98158264	-79,56852722	-941,8585205	-0,045803
Cloud_Mesh_Distance_BA_R0_R2	23,04134178	-79,68864441	-941,8272705	-0,052141
Cloud_Mesh_Distance_BA_R0_R2	23,05947685	-79,72691345	-941,6408691	-0,05112
Cloud_Mesh_Distance_BA_R0_R2	16,12598991	-78,72601318	-941,7481079	-0,048857
Cloud_Mesh_Distance_BA_R0_R2	16,16172791	-78,53561401	-941,7590942	-0,05068
Cloud_Mesh_Distance_BA_R0_R2	22,78606033	-79,23954773	-941,8721314	-0,03629
Cloud_Mesh_Distance_BA_R0_R2	16,1146431	-78,21511841	-941,9320068	-0,057841
Cloud_Mesh_Distance_BA_R0_R2	16,30433083	-78,3331604	-941,6593628	-0,045237
Cloud_Mesh_Distance_BA_R0_R2	16,20754623	-78,33169556	-941,7764893	-0,052537
Cloud_Mesh_Distance_BA_R0_R2	16,30615425	-78,19174194	-941,7194824	-0,051292
Cloud_Mesh_Distance_BA_R0_R2	22,61956787	-78,98771667	-941,9122925	-0,027929
Cloud_Mesh_Distance_BA_R0_R2	16,21853256	-78,17144775	-941,8301392	-0,055016
Cloud_Mesh_Distance_BA_R0_R2	16,27304077	-78,07702637	-941,8121948	-0,05151
Cloud_Mesh_Distance_BA_R0_R2	16,19141197	-78,05155945	-941,9188843	-0,054597
Cloud_Mesh_Distance_BA_R0_R2	22,55717468	-78,89683533	-941,9696045	-0,02405
Cloud_Mesh_Distance_BA_R0_R2	22,58198547	-78,92779541	-941,8294678	-0,02184
Cloud_Mesh_Distance_BA_R0_R2	16,43407822	-77,77903748	-941,795044	-0,041106
Cloud_Mesh_Distance_BA_R0_R2	16,35132217	-78,04702759	-941,7362061	-0,047148
Cloud_Mesh_Distance_BA_R0_R2	16,42696381	-77,92073059	-941,7180786	-0,044335
Cloud_Mesh_Distance_BA_R0_R2	16,29656219	-77,93255615	-941,8612061	-0,05318
Cloud_Mesh_Distance_BA_R0_R2	22,26583862	-78,47637939	-941,8409424	0,006819
Cloud_Mesh_Distance_BA_R0_R2	22,30823135	-78,53523254	-941,7717285	0,006972
Cloud_Mesh_Distance_BA_R0_R2	22,37997437	-78,63664246	-941,6999512	0,002398

Cloud_Mesh_Distance_BA_R0_R2	22,42231178	-78,69270325	-941,8829956	-0,001917
Cloud_Mesh_Distance_BA_R0_R2	22,50464249	-78,81222534	-941,7765503	-0,012207
Cloud_Mesh_Distance_BA_R0_R2	16,39225006	-77,72213745	-941,880249	-0,042609
Cloud_Mesh_Distance_BA_R0_R2	16,47060204	-77,66654968	-941,8289185	-0,0393
Cloud_Mesh_Distance_BA_R0_R2	22,24088669	-78,44317627	-941,9932861	0,009079
Cloud_Mesh_Distance_BA_R0_R2	22,17985153	-78,36872864	-941,7980347	0,013949
Cloud_Mesh_Distance_BA_R0_R2	16,57709122	-77,62582397	-941,737915	-0,028564
Cloud_Mesh_Distance_BA_R0_R2	22,04782486	-78,20397949	-941,8932495	0,018748
Cloud_Mesh_Distance_BA_R0_R2	22,14276123	-78,31636047	-941,9970093	0,014711
Cloud_Mesh_Distance_BA_R0_R2	16,65484619	-77,48826599	-941,7580566	-0,021869
Cloud_Mesh_Distance_BA_R0_R2	16,51845932	-77,49884033	-941,9013062	-0,031127
Cloud_Mesh_Distance_BA_R0_R2	21,71043015	-77,85357666	-941,7710571	0,012934
Cloud_Mesh_Distance_BA_R0_R2	21,8008213	-77,93519592	-941,850647	0,018009
Cloud_Mesh_Distance_BA_R0_R2	21,86383057	-78,01594543	-941,7436523	0,013772
Cloud_Mesh_Distance_BA_R0_R2	21,82557678	-77,94714355	-942,0421753	0,022712
Cloud_Mesh_Distance_BA_R0_R2	21,91324806	-78,05102539	-941,9020996	0,020491
Cloud_Mesh_Distance_BA_R0_R2	16,80554771	-77,24176025	-941,8255615	-0,00546
Cloud_Mesh_Distance_BA_R0_R2	16,91101646	-77,21871948	-941,7440796	0,002055
Cloud_Mesh_Distance_BA_R0_R2	16,90892982	-77,14302063	-941,8293457	0,000466
Cloud_Mesh_Distance_BA_R0_R2	16,81034851	-77,14234924	-941,9294434	-0,009911
Cloud_Mesh_Distance_BA_R0_R2	21,45917702	-77,58197021	-941,9008179	0,016981
Cloud_Mesh_Distance_BA_R0_R2	21,58249664	-77,72969055	-941,7490234	0,010096
Cloud_Mesh_Distance_BA_R0_R2	21,69651031	-77,81524658	-941,958374	0,019919
Cloud_Mesh_Distance_BA_R0_R2	16,88034821	-77,04185486	-941,9786377	-0,005646
Cloud_Mesh_Distance_BA_R0_R2	21,22923279	-77,3661499	-941,8895264	0,019643
Cloud_Mesh_Distance_BA_R0_R2	21,2227211	-77,39607239	-941,7116699	0,009245
Cloud_Mesh_Distance_BA_R0_R2	21,33552742	-77,48956299	-941,7556152	0,011266
Cloud_Mesh_Distance_BA_R0_R2	21,39491844	-77,49972534	-942,0739746	0,025136
Cloud_Mesh_Distance_BA_R0_R2	17,25127792	-76,85578918	-941,8898315	0,009252
Cloud_Mesh_Distance_BA_R0_R2	17,24910545	-76,95968628	-941,7468262	0,018082
Cloud_Mesh_Distance_BA_R0_R2	21,09850121	-77,26026917	-941,8439331	0,017916
Cloud_Mesh_Distance_BA_R0_R2	21,34600639	-77,46733093	-941,9353638	0,01767
Cloud_Mesh_Distance_BA_R0_R2	17,3921814	-76,80986023	-941,8406982	0,02099
Cloud_Mesh_Distance_BA_R0_R2	17,50987244	-76,65084839	-942,0020752	0,009185
Cloud_Mesh_Distance_BA_R0_R2	17,77634048	-76,58071899	-941,9230957	0,030432
Cloud_Mesh_Distance_BA_R0_R2	20,7345314	-76,97767639	-941,9390869	0,01353
Cloud_Mesh_Distance_BA_R0_R2	20,75302696	-77,01834106	-941,7866821	0,016667
Cloud_Mesh_Distance_BA_R0_R2	20,85901642	-77,05561829	-941,9615479	0,019338
Cloud_Mesh_Distance_BA_R0_R2	21,00172615	-77,19525146	-941,7769165	0,015534
Cloud_Mesh_Distance_BA_R0_R2	20,99946404	-77,16622925	-941,921875	0,020557
Cloud_Mesh_Distance_BA_R0_R2	20,89758682	-77,11573792	-941,7843018	0,017131
Cloud_Mesh_Distance_BA_R0_R2	21,1310997	-77,25952148	-942,0307007	0,022895
Cloud_Mesh_Distance_BA_R0_R2	17,9036026	-76,55664063	-941,8764648	0,042148
Cloud_Mesh_Distance_BA_R0_R2	18,03386116	-76,50340271	-941,8895264	0,05096
Cloud_Mesh_Distance_BA_R0_R2	19,99378204	-76,64880371	-941,9188232	-0,023313
Cloud_Mesh_Distance_BA_R0_R2	19,89380646	-76,59286499	-942,0005493	-0,02691
Cloud_Mesh_Distance_BA_R0_R2	19,92882156	-76,6544342	-941,8193359	-0,018193
Cloud_Mesh_Distance_BA_R0_R2	19,87503052	-76,61416626	-941,9011841	-0,023452
Cloud_Mesh_Distance_BA_R0_R2	20,16555786	-76,70310974	-941,951355	-0,017197
Cloud_Mesh_Distance_BA_R0_R2	20,36927986	-76,77357483	-942,0161743	-0,01129

Cloud_Mesh_Distance_BA_R0_R2	20,6490097	-76,94023132	-941,868103	0,009041
Cloud_Mesh_Distance_BA_R0_R2	20,63258553	-76,9019928	-942,0374756	0,006647
Cloud_Mesh_Distance_BA_R0_R2	18,20230865	-76,49032593	-941,8210449	0,061615
Cloud_Mesh_Distance_BA_R0_R2	18,30873299	-76,44073486	-941,8703003	0,063972
Cloud_Mesh_Distance_BA_R0_R2	18,57320404	-76,36572266	-941,9586182	0,055402
Cloud_Mesh_Distance_BA_R0_R2	18,88681221	-76,36045837	-941,9803467	0,040453
Cloud_Mesh_Distance_BA_R0_R2	18,75849342	-76,36766052	-941,9485474	0,047359
Cloud_Mesh_Distance_BA_R0_R2	18,93641281	-76,42504883	-941,8438721	0,038589
Cloud_Mesh_Distance_BA_R0_R2	19,0337429	-76,37919617	-941,9761963	0,029924
Cloud_Mesh_Distance_BA_R0_R2	19,18754196	-76,4022522	-941,9818726	0,01177
Cloud_Mesh_Distance_BA_R0_R2	19,1518364	-76,44602966	-941,8579712	0,021092
Cloud_Mesh_Distance_BA_R0_R2	19,34700394	-76,46055603	-941,9173584	0,002213
Cloud_Mesh_Distance_BA_R0_R2	19,65877914	-76,56752014	-941,8408203	-0,013505
Cloud_Mesh_Distance_BA_R0_R2	18,92518234	-76,31665039	-942,0912476	0,037956
Cloud_Mesh_Distance_BA_R0_R2	19,12651825	-76,33761597	-942,1154175	0,017918
Cloud_Mesh_Distance_BA_R0_R2	19,28420258	-76,39535522	-942,0530396	0,005813
Cloud_Mesh_Distance_BA_R0_R2	19,20833588	-84,46707153	-941,4467163	0,016337
Cloud_Mesh_Distance_BA_R0_R2	19,4647007	-84,53244019	-941,4490967	0,017314
Cloud_Mesh_Distance_BA_R0_R2	18,9670105	-84,35948181	-941,3924561	0,011362
Cloud_Mesh_Distance_BA_R0_R2	19,34443855	-84,49949646	-941,4313355	0,012924
Cloud_Mesh_Distance_BA_R0_R2	19,61484528	-84,53665161	-941,4012451	0,016917
Cloud_Mesh_Distance_BA_R0_R2	19,74710846	-84,49583435	-941,2799072	0,001081
Cloud_Mesh_Distance_BA_R0_R2	19,80200958	-84,56098938	-941,4215088	0,015824
Cloud_Mesh_Distance_BA_R0_R2	18,69577408	-84,22506714	-941,3833618	0,002388
Cloud_Mesh_Distance_BA_R0_R2	18,86039352	-84,27566528	-941,28302	0,000193
Cloud_Mesh_Distance_BA_R0_R2	20,07441711	-84,51799011	-941,3287354	0,012241
Cloud_Mesh_Distance_BA_R0_R2	20,54169083	-84,46156311	-941,3032837	0,06095
Cloud_Mesh_Distance_BA_R0_R2	20,36276245	-84,46055603	-941,2651978	0,027995
Cloud_Mesh_Distance_BA_R0_R2	20,69213867	-84,46343994	-941,3345337	0,097121
Cloud_Mesh_Distance_BA_R0_R2	21,3446064	-84,43531799	-941,3881836	0,265752
Cloud_Mesh_Distance_BA_R0_R2	18,4654274	-84,03601074	-941,2283325	-0,014368
Cloud_Mesh_Distance_BA_R0_R2	18,30314445	-83,96177673	-941,3513184	-0,015747
Cloud_Mesh_Distance_BA_R0_R2	18,58005905	-84,13331604	-941,3032227	-0,003589
Cloud_Mesh_Distance_BA_R0_R2	20,84934425	-84,42352295	-941,3070068	0,125234
Cloud_Mesh_Distance_BA_R0_R2	21,09339523	-84,48599243	-941,4225464	0,20574
Cloud_Mesh_Distance_BA_R0_R2	21,1898632	-84,42155457	-941,3582764	0,218086
Cloud_Mesh_Distance_BA_R0_R2	21,13493729	-84,34121704	-941,2581787	0,179254
Cloud_Mesh_Distance_BA_R0_R2	21,22719765	-84,26411438	-941,1934204	0,184892
Cloud_Mesh_Distance_BA_R0_R2	21,46171188	-84,46806335	-941,427002	0,309453
Cloud_Mesh_Distance_BA_R0_R2	21,51781464	-84,35745239	-941,3267212	0,298836
Cloud_Mesh_Distance_BA_R0_R2	21,30347061	-84,34074402	-941,286438	0,230797
Cloud_Mesh_Distance_BA_R0_R2	21,73305702	-84,28038025	-941,2774048	0,333039
Cloud_Mesh_Distance_BA_R0_R2	21,58235359	-84,23257446	-941,2284546	0,281506
Cloud_Mesh_Distance_BA_R0_R2	18,05431747	-83,77590942	-941,397522	-0,03672
Cloud_Mesh_Distance_BA_R0_R2	17,91996384	-83,66455078	-941,427063	-0,048776
Cloud_Mesh_Distance_BA_R0_R2	17,98864746	-83,76971436	-941,5574951	-0,034688
Cloud_Mesh_Distance_BA_R0_R2	17,93543243	-83,60215759	-941,2126465	-0,060116
Cloud_Mesh_Distance_BA_R0_R2	18,13055611	-83,79899597	-941,2728272	-0,035419
Cloud_Mesh_Distance_BA_R0_R2	18,04291344	-83,7144928	-941,2492676	-0,044881
Cloud_Mesh_Distance_BA_R0_R2	17,6116333	-83,2898407	-941,2762451	-0,086555

Cloud_Mesh_Distance_BA_R0_R2	17,59815788	-83,34130859	-941,4470215	-0,083678
Cloud_Mesh_Distance_BA_R0_R2	17,37911606	-83,03619385	-941,3582764	-0,105134
Cloud_Mesh_Distance_BA_R0_R2	17,47298813	-83,081604	-941,1798096	-0,101954
Cloud_Mesh_Distance_BA_R0_R2	17,50178909	-83,1950531	-941,3622437	-0,091761
Cloud_Mesh_Distance_BA_R0_R2	17,38373947	-82,97413635	-941,2028809	-0,105027
Cloud_Mesh_Distance_BA_R0_R2	17,27925491	-82,92433167	-941,4226074	-0,109379
Cloud_Mesh_Distance_BA_R0_R2	17,40155602	-83,14115906	-941,5390015	-0,094494
Cloud_Mesh_Distance_BA_R0_R2	17,2345829	-82,81877136	-941,3370972	-0,112438
Cloud_Mesh_Distance_BA_R0_R2	17,15241814	-82,68060303	-941,3209229	-0,118362
Cloud_Mesh_Distance_BA_R0_R2	17,11001968	-82,70033264	-941,5130005	-0,121143
Cloud_Mesh_Distance_BA_R0_R2	16,96778488	-82,37342834	-941,3479004	-0,11215
Cloud_Mesh_Distance_BA_R0_R2	17,07414818	-82,50004578	-941,2219849	-0,114375
Cloud_Mesh_Distance_BA_R0_R2	16,84889603	-82,18435669	-941,4117432	-0,106257
Cloud_Mesh_Distance_BA_R0_R2	16,74622726	-81,96836853	-941,3880005	-0,087352
Cloud_Mesh_Distance_BA_R0_R2	16,65120506	-81,82763672	-941,4902344	-0,075754
Cloud_Mesh_Distance_BA_R0_R2	16,6821785	-81,78395081	-941,3241577	-0,066403
Cloud_Mesh_Distance_BA_R0_R2	22,93935585	-82,50714111	-941,5376587	0,144666
Cloud_Mesh_Distance_BA_R0_R2	16,60996819	-81,67430115	-941,4121094	-0,060619
Cloud_Mesh_Distance_BA_R0_R2	16,58510971	-81,54748535	-941,3410645	-0,046014
Cloud_Mesh_Distance_BA_R0_R2	16,54259109	-81,40289307	-941,3236694	-0,039462
Cloud_Mesh_Distance_BA_R0_R2	16,54060936	-81,54943848	-941,4765015	-0,051098
Cloud_Mesh_Distance_BA_R0_R2	16,64272308	-81,62229919	-941,24823	-0,053385
Cloud_Mesh_Distance_BA_R0_R2	22,8591671	-82,03465271	-941,4041138	-0,051817
Cloud_Mesh_Distance_BA_R0_R2	16,37464905	-81,15472412	-941,5678711	-0,033013
Cloud_Mesh_Distance_BA_R0_R2	16,48152924	-81,21899414	-941,3396606	-0,029155
Cloud_Mesh_Distance_BA_R0_R2	16,43182755	-81,23674011	-941,4858398	-0,032644
Cloud_Mesh_Distance_BA_R0_R2	16,31601906	-80,97648621	-941,581604	-0,023418
Cloud_Mesh_Distance_BA_R0_R2	16,39617348	-80,9848175	-941,3944092	-0,017723
Cloud_Mesh_Distance_BA_R0_R2	16,42684746	-81,13056946	-941,4169312	-0,027517
Cloud_Mesh_Distance_BA_R0_R2	22,8512516	-81,84712219	-941,2696533	-0,105876
Cloud_Mesh_Distance_BA_R0_R2	22,89664841	-81,81358337	-941,3811646	-0,10815
Cloud_Mesh_Distance_BA_R0_R2	22,87044334	-81,91110229	-941,3736572	-0,085165
Cloud_Mesh_Distance_BA_R0_R2	16,28396606	-80,70481873	-941,494812	-0,007169
Cloud_Mesh_Distance_BA_R0_R2	16,28761292	-80,83544922	-941,559082	-0,016514
Cloud_Mesh_Distance_BA_R0_R2	16,34057808	-80,8243866	-941,4317017	-0,013807
Cloud_Mesh_Distance_BA_R0_R2	23,00812149	-81,49131775	-941,4403076	-0,15379
Cloud_Mesh_Distance_BA_R0_R2	23,02045822	-81,54118347	-941,6018066	-0,141215
Cloud_Mesh_Distance_BA_R0_R2	16,28336906	-80,5406189	-941,4215088	0,00994
Cloud_Mesh_Distance_BA_R0_R2	16,22808838	-80,3752594	-941,4749756	0,013078
Cloud_Mesh_Distance_BA_R0_R2	16,29174805	-80,42774963	-941,362793	0,017563
Cloud_Mesh_Distance_BA_R0_R2	23,04862595	-81,33300781	-941,3624268	-0,164236
Cloud_Mesh_Distance_BA_R0_R2	23,10974121	-81,22372437	-941,5319824	-0,17158
Cloud_Mesh_Distance_BA_R0_R2	23,06374359	-81,38142395	-941,565979	-0,155712
Cloud_Mesh_Distance_BA_R0_R2	23,11493301	-81,25968933	-941,7159424	-0,158877
Cloud_Mesh_Distance_BA_R0_R2	16,18663406	-80,21087646	-941,5131836	0,008585
Cloud_Mesh_Distance_BA_R0_R2	16,18970108	-80,09059143	-941,4837036	0,017979
Cloud_Mesh_Distance_BA_R0_R2	16,11368561	-80,13108826	-941,6353149	0,000939
Cloud_Mesh_Distance_BA_R0_R2	23,19082642	-80,94723511	-941,6502075	-0,180016
Cloud_Mesh_Distance_BA_R0_R2	16,25109863	-80,15492249	-941,3740845	0,028582
Cloud_Mesh_Distance_BA_R0_R2	16,20234871	-79,71263123	-941,4424439	0,026356

Cloud_Mesh_Distance_BA_R0_R2	16,18156815	-79,58102417	-941,4914551	0,016425
Cloud_Mesh_Distance_BA_R0_R2	23,22247696	-80,37123108	-941,5494385	-0,134008
Cloud_Mesh_Distance_BA_R0_R2	23,22530174	-80,49266052	-941,4888916	-0,154775
Cloud_Mesh_Distance_BA_R0_R2	23,22372627	-80,59703064	-941,4799194	-0,169544
Cloud_Mesh_Distance_BA_R0_R2	23,19322968	-80,1509552	-941,7382813	-0,096939
Cloud_Mesh_Distance_BA_R0_R2	16,16202545	-79,23556519	-941,579773	-0,010967
Cloud_Mesh_Distance_BA_R0_R2	16,2417202	-79,26290894	-941,4450073	0,00886
Cloud_Mesh_Distance_BA_R0_R2	23,20147705	-80,22845459	-941,5168457	-0,113307
Cloud_Mesh_Distance_BA_R0_R2	23,16854477	-80,04498291	-941,6605835	-0,088043
Cloud_Mesh_Distance_BA_R0_R2	23,15340042	-80,01731873	-941,4864502	-0,083731
Cloud_Mesh_Distance_BA_R0_R2	16,256464	-79,11647034	-941,4591065	-0,001472
Cloud_Mesh_Distance_BA_R0_R2	23,1191597	-79,87971497	-941,6777954	-0,068492
Cloud_Mesh_Distance_BA_R0_R2	16,19101906	-78,99699402	-941,590332	-0,022882
Cloud_Mesh_Distance_BA_R0_R2	16,33523178	-78,88098145	-941,4150391	-0,009447
Cloud_Mesh_Distance_BA_R0_R2	16,28125763	-78,96728516	-941,4661255	-0,00895
Cloud_Mesh_Distance_BA_R0_R2	23,1002655	-79,84846497	-941,487915	-0,065564
Cloud_Mesh_Distance_BA_R0_R2	23,05007172	-79,72746277	-941,4619141	-0,050657
Cloud_Mesh_Distance_BA_R0_R2	16,23683739	-78,65791321	-941,6257324	-0,03809
Cloud_Mesh_Distance_BA_R0_R2	16,27079391	-78,82447815	-941,5264893	-0,024715
Cloud_Mesh_Distance_BA_R0_R2	16,18523407	-78,82206726	-941,6455078	-0,036825
Cloud_Mesh_Distance_BA_R0_R2	16,31542206	-78,68962097	-941,5108643	-0,026779
Cloud_Mesh_Distance_BA_R0_R2	22,86789322	-79,37254333	-941,5602417	-0,032445
Cloud_Mesh_Distance_BA_R0_R2	22,9393158	-79,48765564	-941,6470947	-0,034571
Cloud_Mesh_Distance_BA_R0_R2	22,99658966	-79,59231567	-941,7226563	-0,039549
Cloud_Mesh_Distance_BA_R0_R2	23,00133705	-79,61030579	-941,5509033	-0,038022
Cloud_Mesh_Distance_BA_R0_R2	16,2646656	-78,46897888	-941,6549072	-0,045369
Cloud_Mesh_Distance_BA_R0_R2	16,33025742	-78,55493164	-941,5405884	-0,035521
Cloud_Mesh_Distance_BA_R0_R2	22,87325668	-79,37509155	-941,7822266	-0,03777
Cloud_Mesh_Distance_BA_R0_R2	22,79022598	-79,24310303	-941,6749268	-0,030206
Cloud_Mesh_Distance_BA_R0_R2	22,78380013	-79,24938965	-941,461731	-0,021126
Cloud_Mesh_Distance_BA_R0_R2	16,34509659	-78,41532898	-941,5764771	-0,039348
Cloud_Mesh_Distance_BA_R0_R2	16,3961792	-78,26983643	-941,5761108	-0,041847
Cloud_Mesh_Distance_BA_R0_R2	22,55228233	-78,889328	-941,5897217	-0,008244
Cloud_Mesh_Distance_BA_R0_R2	22,61930656	-78,98550415	-941,6282349	-0,01608
Cloud_Mesh_Distance_BA_R0_R2	22,69802094	-79,1055603	-941,5928345	-0,022097
Cloud_Mesh_Distance_BA_R0_R2	22,64606857	-79,02275085	-941,7749023	-0,024213
Cloud_Mesh_Distance_BA_R0_R2	22,72342491	-79,13996887	-941,7766724	-0,028481
Cloud_Mesh_Distance_BA_R0_R2	16,3913784	-78,12619019	-941,6503906	-0,044338
Cloud_Mesh_Distance_BA_R0_R2	16,4145546	-78,04394531	-941,6646118	-0,043523
Cloud_Mesh_Distance_BA_R0_R2	22,58115387	-78,92599487	-941,7183838	-0,017436
Cloud_Mesh_Distance_BA_R0_R2	16,55435944	-77,89672852	-941,5842285	-0,031356
Cloud_Mesh_Distance_BA_R0_R2	16,62591553	-77,7412262	-941,6019898	-0,025813
Cloud_Mesh_Distance_BA_R0_R2	16,51350021	-77,77090454	-941,7103882	-0,036675
Cloud_Mesh_Distance_BA_R0_R2	22,23551369	-78,45289612	-941,6359253	0,015042
Cloud_Mesh_Distance_BA_R0_R2	22,29805183	-78,52812195	-941,6800537	0,011504
Cloud_Mesh_Distance_BA_R0_R2	22,46453667	-78,76599121	-941,5582886	0,003153
Cloud_Mesh_Distance_BA_R0_R2	22,38044357	-78,65895081	-941,4882202	0,012523
Cloud_Mesh_Distance_BA_R0_R2	16,69036484	-77,59580994	-941,6344605	-0,017589
Cloud_Mesh_Distance_BA_R0_R2	22,25818825	-78,47122192	-941,7440796	0,011945
Cloud_Mesh_Distance_BA_R0_R2	16,73083115	-77,50019836	-941,6658325	-0,014406

Cloud_Mesh_Distance_BA_R0_R2	22,07736206	-78,25630188	-941,6983032	0,015664
Cloud_Mesh_Distance_BA_R0_R2	21,96260071	-78,12971497	-941,6902466	0,014274
Cloud_Mesh_Distance_BA_R0_R2	16,77653313	-77,39328003	-941,7103882	-0,006613
Cloud_Mesh_Distance_BA_R0_R2	16,87850571	-77,29693604	-941,6971436	0,002875
Cloud_Mesh_Distance_BA_R0_R2	16,80907249	-77,52687073	-941,5612183	-0,002714
Cloud_Mesh_Distance_BA_R0_R2	16,92522812	-77,38867188	-941,5628052	0,006048
Cloud_Mesh_Distance_BA_R0_R2	16,9841671	-77,25350952	-941,637207	0,012102
Cloud_Mesh_Distance_BA_R0_R2	17,14567375	-77,25228882	-941,4923706	0,02628
Cloud_Mesh_Distance_BA_R0_R2	21,77950668	-77,93927002	-941,6817627	0,012077
Cloud_Mesh_Distance_BA_R0_R2	21,75629616	-77,95149231	-941,5195923	0,002663
Cloud_Mesh_Distance_BA_R0_R2	21,85279465	-78,03213501	-941,59021	0,010344
Cloud_Mesh_Distance_BA_R0_R2	16,98581696	-77,16819763	-941,7276611	0,007816
Cloud_Mesh_Distance_BA_R0_R2	17,16876602	-77,16366577	-941,5667114	0,024137
Cloud_Mesh_Distance_BA_R0_R2	17,08250809	-77,22993469	-941,5707398	0,022088
Cloud_Mesh_Distance_BA_R0_R2	17,07546425	-77,06253052	-941,7687378	0,013875
Cloud_Mesh_Distance_BA_R0_R2	17,05806732	-77,17364502	-941,6547241	0,014601
Cloud_Mesh_Distance_BA_R0_R2	17,30537987	-77,05818176	-941,5772705	0,031198
Cloud_Mesh_Distance_BA_R0_R2	17,4410305	-76,96182251	-941,588562	0,038308
Cloud_Mesh_Distance_BA_R0_R2	17,34635544	-76,96697998	-941,6574707	0,030501
Cloud_Mesh_Distance_BA_R0_R2	21,13363838	-77,34468079	-941,6080933	0,010829
Cloud_Mesh_Distance_BA_R0_R2	21,15137291	-77,32469177	-941,7494507	0,011582
Cloud_Mesh_Distance_BA_R0_R2	21,44672012	-77,60614014	-941,7059937	0,007172
Cloud_Mesh_Distance_BA_R0_R2	17,60809898	-76,7817688	-941,7149658	0,038159
Cloud_Mesh_Distance_BA_R0_R2	17,38868332	-76,89152527	-941,7252808	0,025286
Cloud_Mesh_Distance_BA_R0_R2	17,48937988	-76,82003784	-941,748291	0,02838
Cloud_Mesh_Distance_BA_R0_R2	17,67470741	-76,82130432	-941,6070557	0,046233
Cloud_Mesh_Distance_BA_R0_R2	17,73204422	-76,75576782	-941,6643677	0,047486
Cloud_Mesh_Distance_BA_R0_R2	21,05716896	-77,25970459	-941,6893921	0,014538
Cloud_Mesh_Distance_BA_R0_R2	17,50348091	-76,74589539	-941,8512573	0,022217
Cloud_Mesh_Distance_BA_R0_R2	17,78708458	-76,80310059	-941,5563965	0,0535
Cloud_Mesh_Distance_BA_R0_R2	17,69114113	-76,67808533	-941,8191528	0,03393
Cloud_Mesh_Distance_BA_R0_R2	17,85130692	-76,61941528	-941,802124	0,040954
Cloud_Mesh_Distance_BA_R0_R2	17,98488426	-76,59098816	-941,7624512	0,053956
Cloud_Mesh_Distance_BA_R0_R2	20,60652542	-76,94824219	-941,7233887	0,01128
Cloud_Mesh_Distance_BA_R0_R2	20,69322014	-77,01826477	-941,6343384	0,014641
Cloud_Mesh_Distance_BA_R0_R2	18,12696838	-76,55345154	-941,7440186	0,06161
Cloud_Mesh_Distance_BA_R0_R2	18,49578857	-76,51690674	-941,664978	0,071221
Cloud_Mesh_Distance_BA_R0_R2	18,59079361	-76,56646729	-941,5543823	0,072621
Cloud_Mesh_Distance_BA_R0_R2	18,63503838	-76,50305176	-941,6679077	0,065154
Cloud_Mesh_Distance_BA_R0_R2	18,77460098	-76,50819397	-941,6491089	0,057886
Cloud_Mesh_Distance_BA_R0_R2	19,27314568	-76,56004333	-941,630188	0,018684
Cloud_Mesh_Distance_BA_R0_R2	19,4200058	-76,57997131	-941,6469727	0,008653
Cloud_Mesh_Distance_BA_R0_R2	19,89842033	-76,67077637	-941,7321167	-0,013863
Cloud_Mesh_Distance_BA_R0_R2	20,30487061	-76,7908783	-941,8043213	-0,003885
Cloud_Mesh_Distance_BA_R0_R2	20,06613922	-76,72090149	-941,7388916	-0,012235
Cloud_Mesh_Distance_BA_R0_R2	20,50531006	-76,86442566	-941,8729858	0,002042
Cloud_Mesh_Distance_BA_R0_R2	20,44465256	-76,88339233	-941,6659546	0,007829
Cloud_Mesh_Distance_BA_R0_R2	18,23617935	-76,53826904	-941,7161865	0,067815
Cloud_Mesh_Distance_BA_R0_R2	18,48907089	-76,44334412	-941,8112793	0,062555
Cloud_Mesh_Distance_BA_R0_R2	18,72228622	-76,43997192	-941,7907715	0,057153

Cloud_Mesh_Distance_BA_R0_R2	19,11031723	-76,50967407	-941,6934204	0,030263
Cloud_Mesh_Distance_BA_R0_R2	19,30114746	-76,5153656	-941,7522583	0,011355
Cloud_Mesh_Distance_BA_R0_R2	19,55599213	-76,60353088	-941,6607056	-0,00208
Cloud_Mesh_Distance_BA_R0_R2	19,64235497	-76,64167786	-941,6107178	-0,001526
Cloud_Mesh_Distance_BA_R0_R2	19,5033493	-76,54788208	-941,7810669	-0,007578
Cloud_Mesh_Distance_BA_R0_R2	19,80212784	-76,65437317	-941,6956177	-0,014666
Cloud_Mesh_Distance_BA_R0_R2	19,674366	-76,61308289	-941,7173462	-0,01026
Cloud_Mesh_Distance_BA_R0_R2	19,80035782	-76,6184082	-941,8087769	-0,019524
Cloud_Mesh_Distance_BA_R0_R2	19,12828064	-84,40203857	-941,3365479	0,006902
Cloud_Mesh_Distance_BA_R0_R2	19,28652573	-84,43963623	-941,310669	0,003082
Cloud_Mesh_Distance_BA_R0_R2	19,45554352	-84,47557068	-941,3077393	0,004729
Cloud_Mesh_Distance_BA_R0_R2	19,50480652	-84,42828369	-941,1744385	-0,008396
Cloud_Mesh_Distance_BA_R0_R2	19,60653496	-84,46669006	-941,2373047	-0,005319
Cloud_Mesh_Distance_BA_R0_R2	19,71173477	-84,44299316	-941,1667481	-0,012039
Cloud_Mesh_Distance_BA_R0_R2	19,90797806	-84,50915527	-941,3027954	0,001717
Cloud_Mesh_Distance_BA_R0_R2	20,01424026	-84,44981384	-941,1873169	-0,008883
Cloud_Mesh_Distance_BA_R0_R2	19,84522629	-84,44563293	-941,1672363	-0,015489
Cloud_Mesh_Distance_BA_R0_R2	18,73813248	-84,18432617	-941,190979	-0,003818
Cloud_Mesh_Distance_BA_R0_R2	19,03806305	-84,32493591	-941,2126465	-0,004158
Cloud_Mesh_Distance_BA_R0_R2	18,92843819	-84,2263031	-941,0656128	-0,014289
Cloud_Mesh_Distance_BA_R0_R2	19,37587547	-84,40704346	-941,1765137	-0,008344
Cloud_Mesh_Distance_BA_R0_R2	19,21389771	-84,37063599	-941,1824341	-0,007214
Cloud_Mesh_Distance_BA_R0_R2	20,18751717	-84,45593262	-941,2243042	0,005625
Cloud_Mesh_Distance_BA_R0_R2	20,30271149	-84,38685608	-941,1229858	0,003689
Cloud_Mesh_Distance_BA_R0_R2	20,13084984	-84,38597107	-941,0827637	-0,012839
Cloud_Mesh_Distance_BA_R0_R2	18,29127502	-83,84527588	-941,0446167	-0,03629
Cloud_Mesh_Distance_BA_R0_R2	18,36767578	-83,9382019	-941,1411133	-0,023159
Cloud_Mesh_Distance_BA_R0_R2	18,50745392	-83,99781799	-941,0438232	-0,022336
Cloud_Mesh_Distance_BA_R0_R2	20,48288536	-84,38256836	-941,1610718	0,030967
Cloud_Mesh_Distance_BA_R0_R2	20,40946579	-84,30032349	-941,0073242	-0,000309
Cloud_Mesh_Distance_BA_R0_R2	20,68095779	-84,38027954	-941,2089844	0,069013
Cloud_Mesh_Distance_BA_R0_R2	20,59693718	-84,30108643	-941,0661621	0,033642
Cloud_Mesh_Distance_BA_R0_R2	20,8373642	-84,3409729	-941,1914673	0,103069
Cloud_Mesh_Distance_BA_R0_R2	20,97525787	-84,34266663	-941,2269287	0,139202
Cloud_Mesh_Distance_BA_R0_R2	21,07784271	-84,25892639	-941,151062	0,140571
Cloud_Mesh_Distance_BA_R0_R2	18,23486328	-83,86485291	-941,2177124	-0,028443
Cloud_Mesh_Distance_BA_R0_R2	18,1631031	-83,77297974	-941,1251831	-0,040942
Cloud_Mesh_Distance_BA_R0_R2	20,91570473	-84,27030945	-941,1205444	0,105513
Cloud_Mesh_Distance_BA_R0_R2	21,4017868	-84,22563171	-941,1925659	0,228422
Cloud_Mesh_Distance_BA_R0_R2	21,39917374	-84,10258484	-941,0831909	0,19053
Cloud_Mesh_Distance_BA_R0_R2	17,83876228	-83,49031067	-941,1689453	-0,070606
Cloud_Mesh_Distance_BA_R0_R2	17,71536636	-83,40319824	-941,2728272	-0,077121
Cloud_Mesh_Distance_BA_R0_R2	17,85447311	-83,453125	-941,0377808	-0,074279
Cloud_Mesh_Distance_BA_R0_R2	17,94903946	-83,5385437	-941,0192261	-0,066354
Cloud_Mesh_Distance_BA_R0_R2	21,30500031	-84,0774231	-941,0339356	0,15237
Cloud_Mesh_Distance_BA_R0_R2	21,69107056	-84,16894531	-941,1960449	0,298075
Cloud_Mesh_Distance_BA_R0_R2	21,56208038	-84,13047791	-941,1455078	0,244466
Cloud_Mesh_Distance_BA_R0_R2	21,53412056	-84,01753235	-941,0560913	0,199737
Cloud_Mesh_Distance_BA_R0_R2	17,7790184	-83,40423584	-941,1080933	-0,080994
Cloud_Mesh_Distance_BA_R0_R2	21,62924957	-83,89683533	-941,0045776	0,188349

Cloud_Mesh_Distance_BA_R0_R2	21,75654984	-84,03036499	-941,1193237	0,273724
Cloud_Mesh_Distance_BA_R0_R2	21,83920288	-83,82580566	-941,0323486	0,233295
Cloud_Mesh_Distance_BA_R0_R2	17,56887817	-83,20291138	-941,1873169	-0,091431
Cloud_Mesh_Distance_BA_R0_R2	17,42224693	-82,97790527	-941,0983276	-0,104063
Cloud_Mesh_Distance_BA_R0_R2	17,34520149	-82,86885071	-941,0942993	-0,107891
Cloud_Mesh_Distance_BA_R0_R2	17,24501038	-82,76449585	-941,187439	-0,113382
Cloud_Mesh_Distance_BA_R0_R2	17,30924034	-82,89924622	-941,2698975	-0,108086
Cloud_Mesh_Distance_BA_R0_R2	17,16996002	-82,59315491	-941,0872192	-0,111235
Cloud_Mesh_Distance_BA_R0_R2	16,91797256	-82,20344543	-941,1983032	-0,100501
Cloud_Mesh_Distance_BA_R0_R2	16,83255386	-82,05479431	-941,232605	-0,088187
Cloud_Mesh_Distance_BA_R0_R2	16,89131165	-82,07695007	-941,0731201	-0,08538
Cloud_Mesh_Distance_BA_R0_R2	16,82062531	-81,93659973	-941,0890503	-0,074781
Cloud_Mesh_Distance_BA_R0_R2	22,63869476	-82,81707764	-941,1508179	0,145874
Cloud_Mesh_Distance_BA_R0_R2	22,76607132	-82,72798157	-941,2670288	0,16472
Cloud_Mesh_Distance_BA_R0_R2	16,76605988	-81,86248779	-941,1599121	-0,07011
Cloud_Mesh_Distance_BA_R0_R2	16,758358	-81,9017334	-941,2388916	-0,074521
Cloud_Mesh_Distance_BA_R0_R2	22,66289139	-82,62583923	-941,1660767	0,084256
Cloud_Mesh_Distance_BA_R0_R2	22,87485886	-82,63171387	-941,4066773	0,161769
Cloud_Mesh_Distance_BA_R0_R2	16,71809006	-81,73997498	-941,1530151	-0,058655
Cloud_Mesh_Distance_BA_R0_R2	22,68774414	-82,44552612	-941,1760254	0,030608
Cloud_Mesh_Distance_BA_R0_R2	22,7142067	-82,30844116	-941,1887207	-0,004568
Cloud_Mesh_Distance_BA_R0_R2	22,85004044	-82,43304443	-941,416687	0,079837
Cloud_Mesh_Distance_BA_R0_R2	22,76709938	-82,54103088	-941,2884522	0,089854
Cloud_Mesh_Distance_BA_R0_R2	16,60039139	-81,48919678	-941,2375488	-0,041198
Cloud_Mesh_Distance_BA_R0_R2	22,76988602	-82,36808777	-941,2883911	0,029166
Cloud_Mesh_Distance_BA_R0_R2	22,76522255	-82,23968506	-941,2602539	-0,014807
Cloud_Mesh_Distance_BA_R0_R2	22,79222488	-82,11456299	-941,2755127	-0,04868
Cloud_Mesh_Distance_BA_R0_R2	22,83745575	-82,22921753	-941,3987427	0,00483
Cloud_Mesh_Distance_BA_R0_R2	16,55078888	-81,24537659	-941,1740723	-0,033211
Cloud_Mesh_Distance_BA_R0_R2	16,64190865	-81,47067261	-941,1015625	-0,042879
Cloud_Mesh_Distance_BA_R0_R2	16,58792114	-81,38285828	-941,1787109	-0,036424
Cloud_Mesh_Distance_BA_R0_R2	22,81852913	-81,97186279	-941,2703857	-0,083285
Cloud_Mesh_Distance_BA_R0_R2	22,76144981	-82,03059387	-941,1682739	-0,072238
Cloud_Mesh_Distance_BA_R0_R2	16,45347404	-81,06855774	-941,3063355	-0,022212
Cloud_Mesh_Distance_BA_R0_R2	16,50891876	-81,05671692	-941,1589966	-0,019624
Cloud_Mesh_Distance_BA_R0_R2	16,45247841	-80,95394897	-941,239624	-0,012954
Cloud_Mesh_Distance_BA_R0_R2	22,89031219	-81,74932861	-941,2990112	-0,119508
Cloud_Mesh_Distance_BA_R0_R2	22,94801903	-81,66928101	-941,4248047	-0,13254
Cloud_Mesh_Distance_BA_R0_R2	16,34507751	-80,65541077	-941,3375244	0,002518
Cloud_Mesh_Distance_BA_R0_R2	16,40774536	-80,58659363	-941,171814	0,01159
Cloud_Mesh_Distance_BA_R0_R2	16,34020996	-80,48323059	-941,279541	0,017084
Cloud_Mesh_Distance_BA_R0_R2	16,3212471	-80,13970947	-941,2336426	0,038269
Cloud_Mesh_Distance_BA_R0_R2	16,40163803	-80,3764801	-941,1208496	0,024774
Cloud_Mesh_Distance_BA_R0_R2	16,36838722	-80,21710205	-941,1539307	0,034918
Cloud_Mesh_Distance_BA_R0_R2	16,31402206	-80,31114197	-941,2828369	0,021939
Cloud_Mesh_Distance_BA_R0_R2	23,1068821	-81,16999817	-941,383606	-0,177834
Cloud_Mesh_Distance_BA_R0_R2	23,07196999	-81,17411804	-941,206665	-0,183185
Cloud_Mesh_Distance_BA_R0_R2	23,13999939	-81,02922058	-941,3361206	-0,190296
Cloud_Mesh_Distance_BA_R0_R2	23,15232086	-81,06703186	-941,5060425	-0,18834
Cloud_Mesh_Distance_BA_R0_R2	16,23064613	-79,89851379	-941,3867188	0,032916

Cloud_Mesh_Distance_BA_R0_R2	16,16891479	-79,94824219	-941,507019	0,018568
Cloud_Mesh_Distance_BA_R0_R2	16,22827339	-80,0078125	-941,3995972	0,030068
Cloud_Mesh_Distance_BA_R0_R2	23,12060547	-80,97801208	-941,1688843	-0,196702
Cloud_Mesh_Distance_BA_R0_R2	23,18750954	-80,90296936	-941,4764404	-0,188699
Cloud_Mesh_Distance_BA_R0_R2	23,1737442	-80,85845947	-941,3067627	-0,195056
Cloud_Mesh_Distance_BA_R0_R2	16,19812012	-79,81758118	-941,4474487	0,025386
Cloud_Mesh_Distance_BA_R0_R2	16,30189133	-79,94671631	-941,2525024	0,044915
Cloud_Mesh_Distance_BA_R0_R2	16,25644302	-79,77398682	-941,3389893	0,038405
Cloud_Mesh_Distance_BA_R0_R2	23,21130753	-80,59957886	-941,3638916	-0,176987
Cloud_Mesh_Distance_BA_R0_R2	23,21278763	-80,72036743	-941,451416	-0,180378
Cloud_Mesh_Distance_BA_R0_R2	16,25977707	-79,49711609	-941,3640137	0,029703
Cloud_Mesh_Distance_BA_R0_R2	16,24264145	-79,63761902	-941,3741455	0,034516
Cloud_Mesh_Distance_BA_R0_R2	23,21211243	-80,48191833	-941,366333	-0,161878
Cloud_Mesh_Distance_BA_R0_R2	16,31406784	-79,62843323	-941,2445068	0,048118
Cloud_Mesh_Distance_BA_R0_R2	23,19553185	-80,38941956	-941,2891235	-0,148074
Cloud_Mesh_Distance_BA_R0_R2	23,21500206	-80,391922	-941,4398804	-0,144147
Cloud_Mesh_Distance_BA_R0_R2	16,20489311	-79,39744568	-941,4785156	0,01133
Cloud_Mesh_Distance_BA_R0_R2	16,31166458	-79,22058105	-941,3394165	0,020316
Cloud_Mesh_Distance_BA_R0_R2	16,27262878	-79,3528595	-941,3710327	0,024846
Cloud_Mesh_Distance_BA_R0_R2	16,34521675	-79,39309692	-941,2349854	0,04259
Cloud_Mesh_Distance_BA_R0_R2	16,40628052	-79,17936707	-941,1965332	0,033878
Cloud_Mesh_Distance_BA_R0_R2	16,34738922	-78,77186584	-941,4371338	-0,016974
Cloud_Mesh_Distance_BA_R0_R2	16,42396927	-78,82315063	-941,3076782	-0,000361
Cloud_Mesh_Distance_BA_R0_R2	16,34963036	-79,02635193	-941,3416138	0,009961
Cloud_Mesh_Distance_BA_R0_R2	23,04445267	-79,7432251	-941,3544312	-0,053218
Cloud_Mesh_Distance_BA_R0_R2	23,00305176	-79,6491394	-941,3637085	-0,047062
Cloud_Mesh_Distance_BA_R0_R2	16,3885746	-78,65020752	-941,4261475	-0,020374
Cloud_Mesh_Distance_BA_R0_R2	22,93933105	-79,54248047	-941,300415	-0,032999
Cloud_Mesh_Distance_BA_R0_R2	22,93919373	-79,50596619	-941,4605713	-0,034931
Cloud_Mesh_Distance_BA_R0_R2	16,48363495	-78,61122131	-941,3151856	-0,015172
Cloud_Mesh_Distance_BA_R0_R2	22,77046204	-79,27197266	-941,250061	-0,019547
Cloud_Mesh_Distance_BA_R0_R2	16,60691833	-78,14993286	-941,3822022	-0,024748
Cloud_Mesh_Distance_BA_R0_R2	16,62340546	-78,32519531	-941,2738648	-0,020856
Cloud_Mesh_Distance_BA_R0_R2	16,53848267	-78,44828796	-941,3182983	-0,018744
Cloud_Mesh_Distance_BA_R0_R2	16,42768669	-78,46102905	-941,453186	-0,029469
Cloud_Mesh_Distance_BA_R0_R2	22,61609077	-78,99447632	-941,4718628	-0,008263
Cloud_Mesh_Distance_BA_R0_R2	22,69467545	-79,12867737	-941,3620606	-0,009899
Cloud_Mesh_Distance_BA_R0_R2	16,48753738	-78,08610535	-941,5565796	-0,036649
Cloud_Mesh_Distance_BA_R0_R2	16,51246643	-78,28889465	-941,4252319	-0,031263
Cloud_Mesh_Distance_BA_R0_R2	22,51875496	-78,86506653	-941,3927002	0,003758
Cloud_Mesh_Distance_BA_R0_R2	22,43723869	-78,7477417	-941,4119873	0,013257
Cloud_Mesh_Distance_BA_R0_R2	16,60770798	-78,00224304	-941,4615479	-0,028999
Cloud_Mesh_Distance_BA_R0_R2	16,67569542	-77,8646698	-941,4667358	-0,021151
Cloud_Mesh_Distance_BA_R0_R2	22,30447197	-78,55041504	-941,5532837	0,012713
Cloud_Mesh_Distance_BA_R0_R2	16,7997818	-77,83699036	-941,3481445	-0,005734
Cloud_Mesh_Distance_BA_R0_R2	16,75852966	-77,69894409	-941,4846802	-0,012465
Cloud_Mesh_Distance_BA_R0_R2	16,85469055	-77,7199707	-941,3688965	-0,003259
Cloud_Mesh_Distance_BA_R0_R2	22,21781731	-78,4576416	-941,4608765	0,01531
Cloud_Mesh_Distance_BA_R0_R2	22,14588737	-78,35159302	-941,5847168	0,015394
Cloud_Mesh_Distance_BA_R0_R2	16,92861938	-77,50912476	-941,4542847	0,007214

Cloud_Mesh_Distance_BA_R0_R2	16,87586212	-77,61636353	-941,4227905	0,002005
Cloud_Mesh_Distance_BA_R0_R2	21,92308235	-78,12948608	-941,4746094	0,00962
Cloud_Mesh_Distance_BA_R0_R2	22,03764153	-78,23977661	-941,5251465	0,014341
Cloud_Mesh_Distance_BA_R0_R2	22,11246681	-78,34973145	-941,394043	0,016176
Cloud_Mesh_Distance_BA_R0_R2	17,05914879	-77,44514465	-941,3866577	0,019936
Cloud_Mesh_Distance_BA_R0_R2	17,17571259	-77,33161926	-941,3876953	0,026329
Cloud_Mesh_Distance_BA_R0_R2	17,0605526	-77,31462097	-941,5054932	0,021358
Cloud_Mesh_Distance_BA_R0_R2	21,65437698	-77,88053894	-941,4176636	-0,001852
Cloud_Mesh_Distance_BA_R0_R2	17,24390602	-77,22885132	-941,4338989	0,032104
Cloud_Mesh_Distance_BA_R0_R2	17,39946747	-77,17610168	-941,3677368	0,043506
Cloud_Mesh_Distance_BA_R0_R2	21,36917496	-77,56083679	-941,5922241	0,001686
Cloud_Mesh_Distance_BA_R0_R2	21,46951294	-77,67347717	-941,5267944	-0,001723
Cloud_Mesh_Distance_BA_R0_R2	21,58641243	-77,78256226	-941,5353394	-0,001666
Cloud_Mesh_Distance_BA_R0_R2	21,68064117	-77,85507202	-941,6122437	0,006201
Cloud_Mesh_Distance_BA_R0_R2	17,2791214	-77,15574646	-941,4846802	0,032368
Cloud_Mesh_Distance_BA_R0_R2	17,49259377	-77,03703308	-941,4558716	0,042664
Cloud_Mesh_Distance_BA_R0_R2	17,56088829	-76,95036316	-941,5135498	0,047013
Cloud_Mesh_Distance_BA_R0_R2	21,19294357	-77,48487854	-941,3341675	-0,004492
Cloud_Mesh_Distance_BA_R0_R2	21,24604797	-77,45953369	-941,5505371	0,004001
Cloud_Mesh_Distance_BA_R0_R2	21,33121872	-77,57797241	-941,4199829	-0,004682
Cloud_Mesh_Distance_BA_R0_R2	17,55089569	-76,87353516	-941,6239624	0,03864
Cloud_Mesh_Distance_BA_R0_R2	17,62904739	-76,98776245	-941,4177246	0,054024
Cloud_Mesh_Distance_BA_R0_R2	17,68744469	-76,89387512	-941,4978027	0,056082
Cloud_Mesh_Distance_BA_R0_R2	20,84846497	-77,20524597	-941,3500366	0,011729
Cloud_Mesh_Distance_BA_R0_R2	20,99746895	-77,26101685	-941,5119019	0,009619
Cloud_Mesh_Distance_BA_R0_R2	18,0918541	-76,63040161	-941,6330566	0,069044
Cloud_Mesh_Distance_BA_R0_R2	17,89147949	-76,698349	-941,6463623	0,056791
Cloud_Mesh_Distance_BA_R0_R2	17,90669441	-76,79216003	-941,4953613	0,06364
Cloud_Mesh_Distance_BA_R0_R2	18,07970047	-76,74163818	-941,4699097	0,072286
Cloud_Mesh_Distance_BA_R0_R2	18,25903702	-76,63822937	-941,5369263	0,077512
Cloud_Mesh_Distance_BA_R0_R2	20,21212387	-76,90011597	-941,3099365	0,019296
Cloud_Mesh_Distance_BA_R0_R2	20,87879944	-77,17051697	-941,5178223	0,015961
Cloud_Mesh_Distance_BA_R0_R2	20,78538704	-77,12042236	-941,4753418	0,016668
Cloud_Mesh_Distance_BA_R0_R2	20,70939827	-77,05795288	-941,5249634	0,016027
Cloud_Mesh_Distance_BA_R0_R2	20,94299316	-77,1809082	-941,6489258	0,015536
Cloud_Mesh_Distance_BA_R0_R2	20,81070518	-77,09481812	-941,6208496	0,018746
Cloud_Mesh_Distance_BA_R0_R2	18,20303917	-76,58312988	-941,6531982	0,069472
Cloud_Mesh_Distance_BA_R0_R2	18,41001701	-76,65725708	-941,4487915	0,081094
Cloud_Mesh_Distance_BA_R0_R2	18,42018509	-76,57987976	-941,572937	0,078134
Cloud_Mesh_Distance_BA_R0_R2	18,54708481	-76,63374329	-941,4469605	0,080491
Cloud_Mesh_Distance_BA_R0_R2	18,70692253	-76,62400818	-941,4334717	0,076021
Cloud_Mesh_Distance_BA_R0_R2	18,74786377	-76,56056213	-941,546814	0,065197
Cloud_Mesh_Distance_BA_R0_R2	19,18481827	-76,57763672	-941,5596314	0,032943
Cloud_Mesh_Distance_BA_R0_R2	19,53108597	-76,63705444	-941,5570679	0,007313
Cloud_Mesh_Distance_BA_R0_R2	19,34939575	-76,6211853	-941,5134888	0,021714
Cloud_Mesh_Distance_BA_R0_R2	19,51187706	-76,68687439	-941,4197998	0,022843
Cloud_Mesh_Distance_BA_R0_R2	19,74216461	-76,66195679	-941,6240845	-0,005704
Cloud_Mesh_Distance_BA_R0_R2	19,88004303	-76,77256775	-941,399231	0,011893
Cloud_Mesh_Distance_BA_R0_R2	20,02680969	-76,82556152	-941,3590698	0,012956
Cloud_Mesh_Distance_BA_R0_R2	19,88274002	-76,70726013	-941,597168	-0,006737

Cloud_Mesh_Distance_BA_R0_R2	20,12973213	-76,85267639	-941,3691406	0,0137
Cloud_Mesh_Distance_BA_R0_R2	20,07201004	-76,79260254	-941,4976196	0,003453
Cloud_Mesh_Distance_BA_R0_R2	20,25714302	-76,82444763	-941,600708	0,002805
Cloud_Mesh_Distance_BA_R0_R2	20,39677238	-76,91343689	-941,482544	0,012575
Cloud_Mesh_Distance_BA_R0_R2	18,29402924	-76,56686401	-941,640686	0,073765
Cloud_Mesh_Distance_BA_R0_R2	18,34923553	-76,50744629	-941,7268677	0,070923
Cloud_Mesh_Distance_BA_R0_R2	18,87229729	-76,54942322	-941,567749	0,057954
Cloud_Mesh_Distance_BA_R0_R2	18,90515327	-76,49682617	-941,6810913	0,047237
Cloud_Mesh_Distance_BA_R0_R2	19,02524376	-76,56422424	-941,553772	0,045
Cloud_Mesh_Distance_BA_R0_R2	19,61110115	-84,40974426	-941,1068115	-0,018195
Cloud_Mesh_Distance_BA_R0_R2	18,61481094	-84,09114075	-941,1189575	-0,011453
Cloud_Mesh_Distance_BA_R0_R2	18,76693153	-84,07788086	-940,8786621	-0,027834
Cloud_Mesh_Distance_BA_R0_R2	18,76283836	-84,13063049	-941,012207	-0,015379
Cloud_Mesh_Distance_BA_R0_R2	19,1413784	-84,29162598	-941,0394287	-0,020341
Cloud_Mesh_Distance_BA_R0_R2	19,47933197	-84,37481689	-941,0612793	-0,019595
Cloud_Mesh_Distance_BA_R0_R2	19,32146645	-84,33857727	-941,0410767	-0,021278
Cloud_Mesh_Distance_BA_R0_R2	19,45196915	-84,30467224	-940,9195557	-0,032384
Cloud_Mesh_Distance_BA_R0_R2	19,75274658	-84,39057922	-941,0528565	-0,026246
Cloud_Mesh_Distance_BA_R0_R2	19,7942009	-84,31086731	-940,8966675	-0,040326
Cloud_Mesh_Distance_BA_R0_R2	19,95722389	-84,29728699	-940,8881226	-0,041224
Cloud_Mesh_Distance_BA_R0_R2	19,94161415	-84,37321472	-941,0272217	-0,025883
Cloud_Mesh_Distance_BA_R0_R2	20,24040985	-84,32740784	-941,0033569	-0,014757
Cloud_Mesh_Distance_BA_R0_R2	18,41571045	-83,90849304	-940,9724731	-0,0309
Cloud_Mesh_Distance_BA_R0_R2	18,54583168	-83,95005798	-940,8667603	-0,032809
Cloud_Mesh_Distance_BA_R0_R2	18,64727402	-84,04533386	-940,9509277	-0,023455
Cloud_Mesh_Distance_BA_R0_R2	20,09771538	-84,31211853	-940,9412842	-0,030652
Cloud_Mesh_Distance_BA_R0_R2	20,43460846	-84,2146759	-940,8823242	-0,012519
Cloud_Mesh_Distance_BA_R0_R2	20,25873947	-84,24458313	-940,8704224	-0,029822
Cloud_Mesh_Distance_BA_R0_R2	20,76241112	-84,2870636	-941,0978394	0,07106
Cloud_Mesh_Distance_BA_R0_R2	20,69649124	-84,21525574	-940,9776001	0,037796
Cloud_Mesh_Distance_BA_R0_R2	20,84295273	-84,21162415	-941,0241089	0,07164
Cloud_Mesh_Distance_BA_R0_R2	18,35129738	-83,8338623	-940,9041748	-0,040966
Cloud_Mesh_Distance_BA_R0_R2	18,44049835	-83,87368774	-940,8490601	-0,039568
Cloud_Mesh_Distance_BA_R0_R2	18,39020157	-83,79460144	-940,7529297	-0,050761
Cloud_Mesh_Distance_BA_R0_R2	20,99494553	-84,17915344	-941,0363159	0,09727
Cloud_Mesh_Distance_BA_R0_R2	21,07396698	-84,07646179	-940,951416	0,086917
Cloud_Mesh_Distance_BA_R0_R2	21,23141861	-84,04393005	-940,9776001	0,120452
Cloud_Mesh_Distance_BA_R0_R2	21,21442223	-84,15333557	-941,0776978	0,148342
Cloud_Mesh_Distance_BA_R0_R2	17,90093422	-83,45657349	-940,9371338	-0,075101
Cloud_Mesh_Distance_BA_R0_R2	18,06488991	-83,66270447	-941,0604248	-0,055042
Cloud_Mesh_Distance_BA_R0_R2	21,38512039	-83,92008972	-940,930542	0,12582
Cloud_Mesh_Distance_BA_R0_R2	21,37261009	-84,00743103	-940,9961548	0,14959
Cloud_Mesh_Distance_BA_R0_R2	21,27046585	-83,95350647	-940,9127808	0,10217
Cloud_Mesh_Distance_BA_R0_R2	17,68232727	-83,29602051	-941,1002808	-0,087489
Cloud_Mesh_Distance_BA_R0_R2	17,81180191	-83,37060547	-940,949585	-0,084503
Cloud_Mesh_Distance_BA_R0_R2	21,48035049	-83,93531799	-940,9780273	0,160707
Cloud_Mesh_Distance_BA_R0_R2	21,83745193	-83,69873047	-940,9699707	0,184769
Cloud_Mesh_Distance_BA_R0_R2	22,01031876	-83,78855896	-941,0615234	0,265557
Cloud_Mesh_Distance_BA_R0_R2	21,96105766	-83,69490051	-941,0117798	0,221789
Cloud_Mesh_Distance_BA_R0_R2	17,72149849	-83,24253845	-940,8832398	-0,090415

Cloud_Mesh_Distance_BA_R0_R2	17,58568001	-83,14068604	-941,0027466	-0,097865
Cloud_Mesh_Distance_BA_R0_R2	21,78876686	-83,63041687	-940,9175415	0,144801
Cloud_Mesh_Distance_BA_R0_R2	21,90882111	-83,59642029	-940,9520874	0,17205
Cloud_Mesh_Distance_BA_R0_R2	22,106987	-83,68118286	-941,048523	0,262862
Cloud_Mesh_Distance_BA_R0_R2	22,05173302	-83,56697083	-940,9954224	0,20159
Cloud_Mesh_Distance_BA_R0_R2	17,43297195	-82,92987061	-940,9705811	-0,104612
Cloud_Mesh_Distance_BA_R0_R2	17,48657608	-83,02896118	-941,0270386	-0,102455
Cloud_Mesh_Distance_BA_R0_R2	21,97945023	-83,46282959	-940,9282837	0,146726
Cloud_Mesh_Distance_BA_R0_R2	22,14383888	-83,4094696	-940,9799805	0,176966
Cloud_Mesh_Distance_BA_R0_R2	17,2753067	-82,71853638	-941,0044556	-0,111816
Cloud_Mesh_Distance_BA_R0_R2	17,31529999	-82,68777466	-940,8397217	-0,10931
Cloud_Mesh_Distance_BA_R0_R2	22,1454792	-83,27597046	-940,9398804	0,137765
Cloud_Mesh_Distance_BA_R0_R2	22,27606392	-83,25547791	-940,9949951	0,174587
Cloud_Mesh_Distance_BA_R0_R2	17,09502602	-82,45240784	-941,0630493	-0,109539
Cloud_Mesh_Distance_BA_R0_R2	17,0786705	-82,3848877	-940,9953003	-0,104787
Cloud_Mesh_Distance_BA_R0_R2	17,02009964	-82,34127808	-941,104187	-0,10519
Cloud_Mesh_Distance_BA_R0_R2	22,4280014	-83,10275269	-941,0388184	0,169407
Cloud_Mesh_Distance_BA_R0_R2	16,93776894	-82,09259033	-940,9541626	-0,085915
Cloud_Mesh_Distance_BA_R0_R2	16,96520805	-82,19265747	-941,0240479	-0,095537
Cloud_Mesh_Distance_BA_R0_R2	17,139431	-82,44407654	-940,914917	-0,107555
Cloud_Mesh_Distance_BA_R0_R2	17,05839348	-82,29782104	-940,9129028	-0,100751
Cloud_Mesh_Distance_BA_R0_R2	22,34450531	-82,97445679	-940,9633789	0,097263
Cloud_Mesh_Distance_BA_R0_R2	22,60079384	-83,0040741	-941,1228027	0,207776
Cloud_Mesh_Distance_BA_R0_R2	22,49201965	-82,89961243	-941,0479736	0,123176
Cloud_Mesh_Distance_BA_R0_R2	22,39734459	-82,78648376	-940,9492188	0,054522
Cloud_Mesh_Distance_BA_R0_R2	16,75675583	-81,71270752	-941,0061646	-0,059217
Cloud_Mesh_Distance_BA_R0_R2	16,77597618	-81,81538391	-941,0670776	-0,06599
Cloud_Mesh_Distance_BA_R0_R2	22,53842163	-82,70491028	-941,052124	0,07271
Cloud_Mesh_Distance_BA_R0_R2	22,45268059	-82,58529663	-940,9344482	0,007965
Cloud_Mesh_Distance_BA_R0_R2	22,58129311	-82,50837708	-941,0534058	0,020682
Cloud_Mesh_Distance_BA_R0_R2	16,78516006	-81,66362	-940,8751831	-0,058455
Cloud_Mesh_Distance_BA_R0_R2	16,67843628	-81,6006012	-941,1183472	-0,052251
Cloud_Mesh_Distance_BA_R0_R2	16,71766281	-81,56126404	-940,9689941	-0,051481
Cloud_Mesh_Distance_BA_R0_R2	22,63416672	-82,31251526	-941,0634155	-0,025607
Cloud_Mesh_Distance_BA_R0_R2	22,71563721	-82,16917419	-941,1437378	-0,04393
Cloud_Mesh_Distance_BA_R0_R2	22,64617348	-82,15820313	-941,0147095	-0,063603
Cloud_Mesh_Distance_BA_R0_R2	16,58822441	-81,11819458	-940,9942627	-0,024925
Cloud_Mesh_Distance_BA_R0_R2	16,61189079	-81,29914856	-941,0487671	-0,037789
Cloud_Mesh_Distance_BA_R0_R2	22,6983757	-82,01020813	-941,0234985	-0,085393
Cloud_Mesh_Distance_BA_R0_R2	16,55474854	-80,93011475	-940,9790649	-0,016902
Cloud_Mesh_Distance_BA_R0_R2	22,72868156	-81,84492493	-940,9589233	-0,120853
Cloud_Mesh_Distance_BA_R0_R2	22,78624725	-81,87678528	-941,1194458	-0,107763
Cloud_Mesh_Distance_BA_R0_R2	22,80265999	-81,67599487	-940,9689331	-0,139174
Cloud_Mesh_Distance_BA_R0_R2	16,48931885	-80,86854553	-941,1055908	-0,008715
Cloud_Mesh_Distance_BA_R0_R2	16,41462326	-80,79527283	-941,2456055	-0,006003
Cloud_Mesh_Distance_BA_R0_R2	22,90748596	-81,65638733	-941,2525635	-0,133442
Cloud_Mesh_Distance_BA_R0_R2	22,88960266	-81,59260559	-941,1134644	-0,140619
Cloud_Mesh_Distance_BA_R0_R2	22,8532486	-81,7374115	-941,1697998	-0,12382
Cloud_Mesh_Distance_BA_R0_R2	22,9631691	-81,50836182	-941,2601318	-0,154976
Cloud_Mesh_Distance_BA_R0_R2	16,47543716	-80,69555664	-941,0637817	0,001739

Cloud_Mesh_Distance_BA_R0_R2	23,0046711	-81,34463501	-941,1774902	-0,16235
Cloud_Mesh_Distance_BA_R0_R2	16,3768158	-80,06596375	-941,118103	0,045885
Cloud_Mesh_Distance_BA_R0_R2	16,48322296	-80,33135986	-940,946228	0,023998
Cloud_Mesh_Distance_BA_R0_R2	23,06129837	-81,0811615	-941,0494385	-0,189771
Cloud_Mesh_Distance_BA_R0_R2	16,3861866	-79,9238739	-941,0931397	0,050905
Cloud_Mesh_Distance_BA_R0_R2	16,45763397	-79,97714233	-940,9616699	0,047124
Cloud_Mesh_Distance_BA_R0_R2	23,15515327	-80,78236389	-941,1500855	-0,198612
Cloud_Mesh_Distance_BA_R0_R2	16,31084442	-79,78053284	-941,236145	0,049495
Cloud_Mesh_Distance_BA_R0_R2	23,18686295	-80,64480591	-941,2288208	-0,189181
Cloud_Mesh_Distance_BA_R0_R2	23,19202232	-80,72209167	-941,2945557	-0,190388
Cloud_Mesh_Distance_BA_R0_R2	16,37449265	-79,77436829	-941,1196289	0,05592
Cloud_Mesh_Distance_BA_R0_R2	23,19655037	-80,53897095	-941,2647095	-0,174765
Cloud_Mesh_Distance_BA_R0_R2	23,16027069	-80,57835388	-941,0969238	-0,186184
Cloud_Mesh_Distance_BA_R0_R2	16,44438553	-79,33227539	-941,0836182	0,050826
Cloud_Mesh_Distance_BA_R0_R2	23,11357498	-80,12232971	-941,0806885	-0,1069
Cloud_Mesh_Distance_BA_R0_R2	16,52850342	-79,14839172	-941,0159912	0,041339
Cloud_Mesh_Distance_BA_R0_R2	16,48860741	-79,07943726	-941,1054688	0,03392
Cloud_Mesh_Distance_BA_R0_R2	23,04087067	-79,88710022	-941,0581055	-0,071643
Cloud_Mesh_Distance_BA_R0_R2	23,06078339	-80,01217651	-940,9903565	-0,088226
Cloud_Mesh_Distance_BA_R0_R2	23,07975769	-79,95231628	-941,1268921	-0,076687
Cloud_Mesh_Distance_BA_R0_R2	23,11338425	-79,95587158	-941,2925415	-0,079493
Cloud_Mesh_Distance_BA_R0_R2	23,1661377	-80,17277527	-941,2886353	-0,106494
Cloud_Mesh_Distance_BA_R0_R2	16,43570137	-79,00479126	-941,2163086	0,019978
Cloud_Mesh_Distance_BA_R0_R2	16,5125103	-78,91633606	-941,1398926	0,017811
Cloud_Mesh_Distance_BA_R0_R2	22,99477768	-79,77198792	-941,0540772	-0,055486
Cloud_Mesh_Distance_BA_R0_R2	23,04619217	-79,79801941	-941,2248535	-0,056921
Cloud_Mesh_Distance_BA_R0_R2	22,97376633	-79,65092468	-941,1879273	-0,038521
Cloud_Mesh_Distance_BA_R0_R2	23,07357216	-79,81079102	-941,3652344	-0,059994
Cloud_Mesh_Distance_BA_R0_R2	16,61133766	-78,83413696	-941,0421143	0,014538
Cloud_Mesh_Distance_BA_R0_R2	16,54275322	-78,73190308	-941,1820068	0,00328
Cloud_Mesh_Distance_BA_R0_R2	22,86519432	-79,4045105	-941,3312988	-0,025219
Cloud_Mesh_Distance_BA_R0_R2	16,58158875	-78,55841064	-941,2125855	-0,011837
Cloud_Mesh_Distance_BA_R0_R2	16,64729309	-78,64907837	-941,0852051	-0,003294
Cloud_Mesh_Distance_BA_R0_R2	22,67891884	-79,1675415	-941,1329956	-0,006976
Cloud_Mesh_Distance_BA_R0_R2	22,75668716	-79,30751038	-941,0880737	-0,012689
Cloud_Mesh_Distance_BA_R0_R2	16,60437393	-78,4485321	-941,2365723	-0,01525
Cloud_Mesh_Distance_BA_R0_R2	16,73593712	-78,2960968	-941,1574707	-0,017614
Cloud_Mesh_Distance_BA_R0_R2	16,67494774	-78,46243286	-941,1443481	-0,010581
Cloud_Mesh_Distance_BA_R0_R2	22,60160828	-79,01023865	-941,2695313	-0,002974
Cloud_Mesh_Distance_BA_R0_R2	16,80236053	-78,13618469	-941,1693726	-0,011917
Cloud_Mesh_Distance_BA_R0_R2	16,72246742	-78,01676941	-941,3237915	-0,02002
Cloud_Mesh_Distance_BA_R0_R2	16,69670486	-78,18441772	-941,2601929	-0,017279
Cloud_Mesh_Distance_BA_R0_R2	16,93704987	-78,10760498	-941,0407105	-0,006124
Cloud_Mesh_Distance_BA_R0_R2	22,43355942	-78,77102661	-941,2772827	0,01393
Cloud_Mesh_Distance_BA_R0_R2	16,91769409	-77,77867126	-941,2644653	0,001414
Cloud_Mesh_Distance_BA_R0_R2	17,02322388	-77,96228027	-941,039917	0,002521
Cloud_Mesh_Distance_BA_R0_R2	16,88555908	-77,94998169	-941,1878662	-0,005334
Cloud_Mesh_Distance_BA_R0_R2	22,30264473	-78,58058167	-941,3652954	0,016356
Cloud_Mesh_Distance_BA_R0_R2	22,27955437	-78,58392334	-941,234436	0,022005
Cloud_Mesh_Distance_BA_R0_R2	22,34656143	-78,66732788	-941,2362671	0,017888

Cloud_Mesh_Distance_BA_R0_R2	22,38146019	-78,68412781	-941,3545532	0,015596
Cloud_Mesh_Distance_BA_R0_R2	16,93185234	-77,67276001	-941,3245239	0,00287
Cloud_Mesh_Distance_BA_R0_R2	17,03349304	-77,67712402	-941,2231445	0,012361
Cloud_Mesh_Distance_BA_R0_R2	17,01432991	-77,82554626	-941,137085	0,007582
Cloud_Mesh_Distance_BA_R0_R2	17,12884903	-77,73912048	-941,0891724	0,016679
Cloud_Mesh_Distance_BA_R0_R2	22,00250244	-78,30438232	-941,135437	0,018575
Cloud_Mesh_Distance_BA_R0_R2	22,07864189	-78,35136414	-941,241333	0,019513
Cloud_Mesh_Distance_BA_R0_R2	22,19094086	-78,47622681	-941,2425537	0,023303
Cloud_Mesh_Distance_BA_R0_R2	16,98609543	-77,57711792	-941,3446655	0,013391
Cloud_Mesh_Distance_BA_R0_R2	17,1687851	-77,6108551	-941,1506348	0,023409
Cloud_Mesh_Distance_BA_R0_R2	17,09884071	-77,55184937	-941,2601318	0,022653
Cloud_Mesh_Distance_BA_R0_R2	21,76205444	-78,072052	-941,1398315	0,003821
Cloud_Mesh_Distance_BA_R0_R2	21,86263275	-78,11485291	-941,2994995	0,008327
Cloud_Mesh_Distance_BA_R0_R2	21,89517975	-78,19573975	-941,1428223	0,011299
Cloud_Mesh_Distance_BA_R0_R2	21,98979759	-78,23764038	-941,3145752	0,014793
Cloud_Mesh_Distance_BA_R0_R2	17,25421143	-77,46028137	-941,2040405	0,031689
Cloud_Mesh_Distance_BA_R0_R2	17,29784775	-77,30168152	-941,3161621	0,036189
Cloud_Mesh_Distance_BA_R0_R2	21,68870354	-77,96278381	-941,2598877	-0,002115
Cloud_Mesh_Distance_BA_R0_R2	21,74014091	-77,97171021	-941,3851318	0,001583
Cloud_Mesh_Distance_BA_R0_R2	21,81974411	-78,03710938	-941,4284058	0,00431
Cloud_Mesh_Distance_BA_R0_R2	21,77567673	-78,03051758	-941,3019409	0,003892
Cloud_Mesh_Distance_BA_R0_R2	17,54556847	-77,22805786	-941,2045898	0,050186
Cloud_Mesh_Distance_BA_R0_R2	17,80817795	-77,07981873	-941,1939087	0,06353
Cloud_Mesh_Distance_BA_R0_R2	17,85748291	-77,14794922	-941,0874023	0,064731
Cloud_Mesh_Distance_BA_R0_R2	21,41184425	-77,68844604	-941,315918	-0,008983
Cloud_Mesh_Distance_BA_R0_R2	21,47194672	-77,78356934	-941,2093506	-0,01313
Cloud_Mesh_Distance_BA_R0_R2	21,53162003	-77,77906799	-941,3729248	-0,005995
Cloud_Mesh_Distance_BA_R0_R2	17,62285805	-77,09709167	-941,2944946	0,05354
Cloud_Mesh_Distance_BA_R0_R2	17,92286301	-77,0776825	-941,1277466	0,069554
Cloud_Mesh_Distance_BA_R0_R2	21,1154995	-77,50198364	-941,1364746	-0,010351
Cloud_Mesh_Distance_BA_R0_R2	21,10678101	-77,37458801	-941,4353638	0,005339
Cloud_Mesh_Distance_BA_R0_R2	21,27492142	-77,60771179	-941,2076416	-0,012444
Cloud_Mesh_Distance_BA_R0_R2	17,78203964	-76,96437073	-941,3466797	0,06107
Cloud_Mesh_Distance_BA_R0_R2	18,01984596	-77,04891968	-941,1064453	0,074293
Cloud_Mesh_Distance_BA_R0_R2	17,96352768	-76,98002625	-941,2201538	0,070921
Cloud_Mesh_Distance_BA_R0_R2	18,14908409	-76,86146545	-941,2704468	0,077227
Cloud_Mesh_Distance_BA_R0_R2	20,93236923	-77,31774902	-941,21875	0,003212
Cloud_Mesh_Distance_BA_R0_R2	21,05830383	-77,39772034	-941,2651978	-0,003448
Cloud_Mesh_Distance_BA_R0_R2	20,99134064	-77,30969238	-941,3534546	0,006244
Cloud_Mesh_Distance_BA_R0_R2	17,82295799	-76,86253357	-941,4501953	0,06166
Cloud_Mesh_Distance_BA_R0_R2	17,94959259	-76,86407471	-941,3727417	0,069164
Cloud_Mesh_Distance_BA_R0_R2	18,31443596	-76,74317932	-941,3562622	0,083347
Cloud_Mesh_Distance_BA_R0_R2	18,32807541	-76,83447266	-941,2233887	0,087314
Cloud_Mesh_Distance_BA_R0_R2	18,47124481	-76,79620361	-941,2208862	0,08538
Cloud_Mesh_Distance_BA_R0_R2	18,51185036	-76,71064758	-941,3340454	0,083668
Cloud_Mesh_Distance_BA_R0_R2	18,68501854	-76,77301025	-941,1921997	0,086725
Cloud_Mesh_Distance_BA_R0_R2	19,03322601	-76,75151062	-941,1850586	0,068826
Cloud_Mesh_Distance_BA_R0_R2	19,8358593	-76,81460571	-941,2594605	0,023688
Cloud_Mesh_Distance_BA_R0_R2	20,08687019	-76,88301086	-941,2496338	0,019477
Cloud_Mesh_Distance_BA_R0_R2	20,50315666	-77,00076294	-941,3598023	0,018017

Cloud_Mesh_Distance_BA_R0_R2	20,34240341	-76,94252014	-941,3262329	0,01643
Cloud_Mesh_Distance_BA_R0_R2	20,67341232	-77,08255005	-941,3815918	0,018038
Cloud_Mesh_Distance_BA_R0_R2	20,57795525	-76,98258972	-941,5345459	0,017022
Cloud_Mesh_Distance_BA_R0_R2	18,64884186	-76,68391418	-941,3413086	0,082802
Cloud_Mesh_Distance_BA_R0_R2	18,798666	-76,69268799	-941,3033447	0,075394
Cloud_Mesh_Distance_BA_R0_R2	18,94491768	-76,70506287	-941,2703247	0,072507
Cloud_Mesh_Distance_BA_R0_R2	19,08532333	-76,69995117	-941,2839966	0,059936
Cloud_Mesh_Distance_BA_R0_R2	18,91726494	-76,62561035	-941,4190064	0,063993
Cloud_Mesh_Distance_BA_R0_R2	19,16872978	-76,63825989	-941,421936	0,042951
Cloud_Mesh_Distance_BA_R0_R2	19,35235023	-76,6794281	-941,3776856	0,033467
Cloud_Mesh_Distance_BA_R0_R2	19,69724464	-76,69354248	-941,5005493	0,004953
Cloud_Mesh_Distance_BA_R0_R2	19,95196533	-76,83432007	-941,2805786	0,021035
Cloud_Mesh_Distance_BA_R0_R2	20,23779678	-76,86856079	-941,4295654	0,010014
Cloud_Mesh_Distance_BA_R0_R2	18,89416695	-84,13725281	-940,8844605	-0,029126
Cloud_Mesh_Distance_BA_R0_R2	19,08374405	-84,19857788	-940,8676148	-0,03265
Cloud_Mesh_Distance_BA_R0_R2	19,27409363	-84,26164246	-940,8946533	-0,031918
Cloud_Mesh_Distance_BA_R0_R2	19,41233063	-84,22512817	-940,7741699	-0,046909
Cloud_Mesh_Distance_BA_R0_R2	19,70637894	-84,19010925	-940,6809082	-0,059575
Cloud_Mesh_Distance_BA_R0_R2	19,78116417	-84,23782349	-940,7647095	-0,05478
Cloud_Mesh_Distance_BA_R0_R2	19,61654282	-84,34501648	-940,9695435	-0,034311
Cloud_Mesh_Distance_BA_R0_R2	18,77033424	-83,97377014	-940,6575317	-0,046905
Cloud_Mesh_Distance_BA_R0_R2	18,69678879	-84,00746155	-940,8013306	-0,039339
Cloud_Mesh_Distance_BA_R0_R2	18,94068909	-84,02067566	-940,611145	-0,055062
Cloud_Mesh_Distance_BA_R0_R2	18,84165955	-84,05760193	-940,7593384	-0,038267
Cloud_Mesh_Distance_BA_R0_R2	18,99559784	-84,10922241	-940,7421265	-0,042663
Cloud_Mesh_Distance_BA_R0_R2	19,8965435	-84,25045776	-940,7971802	-0,048538
Cloud_Mesh_Distance_BA_R0_R2	20,05515289	-84,22982788	-940,7919922	-0,044925
Cloud_Mesh_Distance_BA_R0_R2	20,54515457	-84,23628235	-940,9535523	0,009031
Cloud_Mesh_Distance_BA_R0_R2	20,58408356	-84,1446991	-940,8408203	-0,003966
Cloud_Mesh_Distance_BA_R0_R2	18,50577927	-83,87611389	-940,7592773	-0,044065
Cloud_Mesh_Distance_BA_R0_R2	20,67847061	-84,02934265	-940,736023	-0,014795
Cloud_Mesh_Distance_BA_R0_R2	20,84187317	-84,10331726	-940,8911133	0,037135
Cloud_Mesh_Distance_BA_R0_R2	18,03118706	-83,4853363	-940,7277222	-0,075421
Cloud_Mesh_Distance_BA_R0_R2	17,97624588	-83,49916077	-940,868042	-0,073227
Cloud_Mesh_Distance_BA_R0_R2	18,08821869	-83,59358215	-940,8470459	-0,062973
Cloud_Mesh_Distance_BA_R0_R2	18,25706673	-83,65681458	-940,6773071	-0,061325
Cloud_Mesh_Distance_BA_R0_R2	18,22618103	-83,74043274	-940,9133301	-0,049712
Cloud_Mesh_Distance_BA_R0_R2	18,13538742	-83,55311584	-940,673645	-0,0695
Cloud_Mesh_Distance_BA_R0_R2	20,84813309	-83,96928406	-940,744812	0,004199
Cloud_Mesh_Distance_BA_R0_R2	21,00991249	-83,98670959	-940,8336182	0,045772
Cloud_Mesh_Distance_BA_R0_R2	21,14959717	-83,99021912	-940,8960571	0,082778
Cloud_Mesh_Distance_BA_R0_R2	20,98202324	-83,88259888	-940,7209473	0,00785
Cloud_Mesh_Distance_BA_R0_R2	17,83973885	-83,31147766	-940,7619019	-0,089359
Cloud_Mesh_Distance_BA_R0_R2	17,98010063	-83,36260986	-940,5858154	-0,086418
Cloud_Mesh_Distance_BA_R0_R2	17,9208622	-83,41612244	-940,8060913	-0,083621
Cloud_Mesh_Distance_BA_R0_R2	20,94807053	-83,82115173	-940,6490479	-0,017562
Cloud_Mesh_Distance_BA_R0_R2	21,07785416	-83,75010681	-940,65271	-0,008896
Cloud_Mesh_Distance_BA_R0_R2	21,16187859	-83,88577271	-940,8068848	0,052182
Cloud_Mesh_Distance_BA_R0_R2	21,32296944	-83,86961365	-940,8655396	0,092503
Cloud_Mesh_Distance_BA_R0_R2	21,47769165	-83,82905579	-940,9020996	0,124921

Cloud_Mesh_Distance_BA_R0_R2	21,32032967	-83,75294495	-940,7759399	0,056721
Cloud_Mesh_Distance_BA_R0_R2	21,68290138	-83,73822021	-940,930481	0,15032
Cloud_Mesh_Distance_BA_R0_R2	17,56910133	-82,9427948	-940,6674805	-0,108052
Cloud_Mesh_Distance_BA_R0_R2	17,52980042	-83,00669861	-940,8712158	-0,10405
Cloud_Mesh_Distance_BA_R0_R2	17,76811028	-83,21351624	-940,7214356	-0,095169
Cloud_Mesh_Distance_BA_R0_R2	17,66027069	-83,10638428	-940,7548218	-0,10309
Cloud_Mesh_Distance_BA_R0_R2	21,55396461	-83,5438385	-940,7543945	0,051132
Cloud_Mesh_Distance_BA_R0_R2	21,68175888	-83,59510803	-940,8497315	0,103391
Cloud_Mesh_Distance_BA_R0_R2	21,82840538	-83,51457214	-940,8793335	0,116682
Cloud_Mesh_Distance_BA_R0_R2	17,47853661	-82,82511902	-940,6739502	-0,11458
Cloud_Mesh_Distance_BA_R0_R2	17,40001678	-82,76582336	-940,7584839	-0,112505
Cloud_Mesh_Distance_BA_R0_R2	17,46092033	-82,88066101	-940,8076172	-0,108739
Cloud_Mesh_Distance_BA_R0_R2	21,7401371	-83,28691101	-940,7138062	0,021883
Cloud_Mesh_Distance_BA_R0_R2	21,5762043	-83,39622498	-940,6774292	0,012249
Cloud_Mesh_Distance_BA_R0_R2	21,7128334	-83,44883728	-940,7854004	0,062971
Cloud_Mesh_Distance_BA_R0_R2	21,89637947	-83,34230042	-940,8336182	0,079239
Cloud_Mesh_Distance_BA_R0_R2	17,36843109	-82,8092804	-940,9136963	-0,110024
Cloud_Mesh_Distance_BA_R0_R2	22,04620552	-83,33763123	-940,9120483	0,12275
Cloud_Mesh_Distance_BA_R0_R2	22,06748199	-83,18988037	-940,8651123	0,082167
Cloud_Mesh_Distance_BA_R0_R2	17,23064995	-82,58460999	-940,8927002	-0,109412
Cloud_Mesh_Distance_BA_R0_R2	17,22184372	-82,47145081	-940,736084	-0,110392
Cloud_Mesh_Distance_BA_R0_R2	17,2848053	-82,58796692	-940,7559204	-0,109786
Cloud_Mesh_Distance_BA_R0_R2	21,75359726	-83,15887451	-940,6524048	-0,011287
Cloud_Mesh_Distance_BA_R0_R2	21,89992142	-83,14183044	-940,7393799	0,018285
Cloud_Mesh_Distance_BA_R0_R2	22,19654655	-83,17999268	-940,9350586	0,124878
Cloud_Mesh_Distance_BA_R0_R2	22,29000473	-83,11512756	-940,9678345	0,133874
Cloud_Mesh_Distance_BA_R0_R2	22,08113098	-82,98348999	-940,7858887	0,02071
Cloud_Mesh_Distance_BA_R0_R2	17,05716896	-82,20475769	-940,7775269	-0,098596
Cloud_Mesh_Distance_BA_R0_R2	16,99730682	-82,15760803	-940,8756104	-0,092664
Cloud_Mesh_Distance_BA_R0_R2	17,13554573	-82,30862427	-940,718689	-0,104014
Cloud_Mesh_Distance_BA_R0_R2	21,81934547	-83,04664612	-940,6349487	-0,026226
Cloud_Mesh_Distance_BA_R0_R2	21,85868454	-82,93945313	-940,602478	-0,040474
Cloud_Mesh_Distance_BA_R0_R2	22,16214752	-82,79244995	-940,758606	-0,009144
Cloud_Mesh_Distance_BA_R0_R2	22,24979782	-82,87860107	-940,8641357	0,037513
Cloud_Mesh_Distance_BA_R0_R2	22,20566559	-83,05545044	-940,8985596	0,082445
Cloud_Mesh_Distance_BA_R0_R2	16,9513588	-82,04814148	-940,8522949	-0,084355
Cloud_Mesh_Distance_BA_R0_R2	16,99872398	-82,04554749	-940,7218018	-0,087345
Cloud_Mesh_Distance_BA_R0_R2	16,93621826	-81,95726013	-940,7789917	-0,080337
Cloud_Mesh_Distance_BA_R0_R2	22,10375404	-82,71116638	-940,6694336	-0,040715
Cloud_Mesh_Distance_BA_R0_R2	22,29876328	-82,66970825	-940,8195801	-0,006443
Cloud_Mesh_Distance_BA_R0_R2	16,89022446	-81,96812439	-940,9212647	-0,077494
Cloud_Mesh_Distance_BA_R0_R2	16,88562012	-81,82189941	-940,7614746	-0,071328
Cloud_Mesh_Distance_BA_R0_R2	22,38014221	-82,46794128	-940,8085938	-0,041592
Cloud_Mesh_Distance_BA_R0_R2	16,81892967	-81,80955505	-940,9337769	-0,067053
Cloud_Mesh_Distance_BA_R0_R2	22,50875092	-82,37113953	-940,9140625	-0,038944
Cloud_Mesh_Distance_BA_R0_R2	22,57866669	-82,22044373	-940,9396973	-0,062837
Cloud_Mesh_Distance_BA_R0_R2	16,74413681	-81,38067627	-940,7568359	-0,049413
Cloud_Mesh_Distance_BA_R0_R2	16,77176666	-81,53855896	-940,8019409	-0,055516
Cloud_Mesh_Distance_BA_R0_R2	16,80337524	-81,47312927	-940,6694336	-0,057206
Cloud_Mesh_Distance_BA_R0_R2	22,60141182	-82,07600403	-940,8925171	-0,093571

Cloud_Mesh_Distance_BA_R0_R2	22,49598694	-82,18493652	-940,8024292	-0,08389
Cloud_Mesh_Distance_BA_R0_R2	22,52845573	-81,97491455	-940,7131958	-0,108094
Cloud_Mesh_Distance_BA_R0_R2	16,64037704	-81,21244812	-940,9179077	-0,037011
Cloud_Mesh_Distance_BA_R0_R2	16,67339325	-81,35649109	-940,925415	-0,042463
Cloud_Mesh_Distance_BA_R0_R2	16,64126396	-81,10444641	-940,8539429	-0,032014
Cloud_Mesh_Distance_BA_R0_R2	22,70604324	-81,74545288	-940,8269653	-0,129281
Cloud_Mesh_Distance_BA_R0_R2	22,64844131	-81,90678406	-940,8540039	-0,116368
Cloud_Mesh_Distance_BA_R0_R2	16,53106499	-80,78071594	-940,9707031	-0,011902
Cloud_Mesh_Distance_BA_R0_R2	22,87444305	-81,51417542	-940,9758301	-0,154552
Cloud_Mesh_Distance_BA_R0_R2	22,83030319	-81,47929382	-940,833313	-0,150716
Cloud_Mesh_Distance_BA_R0_R2	22,93970108	-81,43418884	-941,0705566	-0,160649
Cloud_Mesh_Distance_BA_R0_R2	16,53803635	-80,62844849	-940,9029541	0,000133
Cloud_Mesh_Distance_BA_R0_R2	16,54071808	-80,44673157	-940,8521729	0,007545
Cloud_Mesh_Distance_BA_R0_R2	22,91068077	-81,3691864	-940,9096069	-0,164157
Cloud_Mesh_Distance_BA_R0_R2	16,47385406	-80,51168823	-941,0061646	0,012859
Cloud_Mesh_Distance_BA_R0_R2	16,54249382	-80,30088806	-940,8243408	0,019821
Cloud_Mesh_Distance_BA_R0_R2	22,9456501	-81,22583008	-940,8553467	-0,170119
Cloud_Mesh_Distance_BA_R0_R2	22,99498367	-81,24693298	-941,0153198	-0,180337
Cloud_Mesh_Distance_BA_R0_R2	16,51420784	-80,15795898	-940,8648071	0,032517
Cloud_Mesh_Distance_BA_R0_R2	16,59767914	-80,34994507	-940,7212524	0,009144
Cloud_Mesh_Distance_BA_R0_R2	16,57779312	-80,21421814	-940,7484741	0,01948
Cloud_Mesh_Distance_BA_R0_R2	23,09111786	-80,92068481	-941,0066528	-0,194891
Cloud_Mesh_Distance_BA_R0_R2	16,43196106	-80,1884613	-941,0249023	0,03753
Cloud_Mesh_Distance_BA_R0_R2	23,05098915	-80,83830261	-940,8424683	-0,191844
Cloud_Mesh_Distance_BA_R0_R2	23,11658096	-80,75904846	-940,9941406	-0,19908
Cloud_Mesh_Distance_BA_R0_R2	16,53238487	-80,00871277	-940,8259277	0,040861
Cloud_Mesh_Distance_BA_R0_R2	16,53459167	-79,87561035	-940,8276978	0,04827
Cloud_Mesh_Distance_BA_R0_R2	16,47852135	-79,74133301	-940,9383545	0,055559
Cloud_Mesh_Distance_BA_R0_R2	16,42365456	-79,85417175	-941,0256958	0,056132
Cloud_Mesh_Distance_BA_R0_R2	23,10014725	-80,66496277	-940,9110107	-0,194336
Cloud_Mesh_Distance_BA_R0_R2	16,50722313	-79,55303955	-940,9204712	0,05903
Cloud_Mesh_Distance_BA_R0_R2	16,56317902	-79,74336243	-940,7922363	0,049156
Cloud_Mesh_Distance_BA_R0_R2	23,10547638	-80,49687195	-940,9087524	-0,178526
Cloud_Mesh_Distance_BA_R0_R2	16,42140579	-79,54701233	-941,0675659	0,05801
Cloud_Mesh_Distance_BA_R0_R2	16,56392288	-79,61045837	-940,8151856	0,052958
Cloud_Mesh_Distance_BA_R0_R2	16,62955475	-79,51179504	-940,7342529	0,04953
Cloud_Mesh_Distance_BA_R0_R2	23,14950562	-80,34205627	-941,0858154	-0,147668
Cloud_Mesh_Distance_BA_R0_R2	16,50621223	-79,41047668	-940,9586182	0,055926
Cloud_Mesh_Distance_BA_R0_R2	16,47716522	-79,20111084	-941,0750732	0,044264
Cloud_Mesh_Distance_BA_R0_R2	16,51932144	-79,26261902	-940,9867554	0,050266
Cloud_Mesh_Distance_BA_R0_R2	23,03259087	-80,06556702	-940,8660889	-0,097236
Cloud_Mesh_Distance_BA_R0_R2	23,06975555	-80,13917542	-940,9204712	-0,108309
Cloud_Mesh_Distance_BA_R0_R2	16,64168739	-79,07473755	-940,8848267	0,035256
Cloud_Mesh_Distance_BA_R0_R2	16,57627106	-79,02307129	-941,0010986	0,03044
Cloud_Mesh_Distance_BA_R0_R2	16,58294296	-79,17002869	-940,9267578	0,042501
Cloud_Mesh_Distance_BA_R0_R2	16,67442322	-78,93284607	-940,909668	0,02335
Cloud_Mesh_Distance_BA_R0_R2	22,9026413	-79,52703857	-941,1572266	-0,028525
Cloud_Mesh_Distance_BA_R0_R2	22,85804749	-79,54576111	-940,9650269	-0,039285
Cloud_Mesh_Distance_BA_R0_R2	22,93277168	-79,64808655	-941,0343018	-0,041622
Cloud_Mesh_Distance_BA_R0_R2	16,71927643	-78,74960327	-940,9443359	0,003175

Cloud_Mesh_Distance_BA_R0_R2	16,84814072	-78,65879822	-940,8345947	-0,000485
Cloud_Mesh_Distance_BA_R0_R2	22,77989578	-79,46180725	-940,8782959	-0,029846
Cloud_Mesh_Distance_BA_R0_R2	16,88061905	-78,42977905	-940,921936	-0,01003
Cloud_Mesh_Distance_BA_R0_R2	16,75758743	-78,55813599	-940,996521	-0,005473
Cloud_Mesh_Distance_BA_R0_R2	22,7109108	-79,31137085	-940,9324341	-0,014445
Cloud_Mesh_Distance_BA_R0_R2	22,78777504	-79,40229797	-940,9979858	-0,020876
Cloud_Mesh_Distance_BA_R0_R2	22,82624626	-79,4044342	-941,1305542	-0,022677
Cloud_Mesh_Distance_BA_R0_R2	16,7785759	-78,40245056	-941,0528565	-0,00859
Cloud_Mesh_Distance_BA_R0_R2	16,84731293	-78,26896667	-941,0469971	-0,013588
Cloud_Mesh_Distance_BA_R0_R2	22,48236656	-78,93518066	-940,9929199	0,01452
Cloud_Mesh_Distance_BA_R0_R2	22,55080032	-79,04478455	-940,9699097	0,004859
Cloud_Mesh_Distance_BA_R0_R2	22,50539207	-78,89543152	-941,1838989	0,006943
Cloud_Mesh_Distance_BA_R0_R2	22,61229897	-79,144104	-940,9553833	-0,003809
Cloud_Mesh_Distance_BA_R0_R2	22,5752697	-79,03053284	-941,0831299	0,003242
Cloud_Mesh_Distance_BA_R0_R2	22,30870628	-78,7334137	-940,9265747	0,029778
Cloud_Mesh_Distance_BA_R0_R2	22,39667892	-78,77690125	-941,095459	0,019036
Cloud_Mesh_Distance_BA_R0_R2	22,39869308	-78,8364563	-940,9576416	0,023949
Cloud_Mesh_Distance_BA_R0_R2	17,10681915	-77,86094666	-941,0249634	0,010314
Cloud_Mesh_Distance_BA_R0_R2	17,2093029	-77,80465698	-940,9674072	0,018697
Cloud_Mesh_Distance_BA_R0_R2	22,09140778	-78,51820374	-940,8391724	0,031817
Cloud_Mesh_Distance_BA_R0_R2	22,20739365	-78,60012817	-940,9456177	0,031517
Cloud_Mesh_Distance_BA_R0_R2	22,14899635	-78,49546814	-941,0372925	0,027287
Cloud_Mesh_Distance_BA_R0_R2	22,27191353	-78,61630249	-941,1004639	0,024326
Cloud_Mesh_Distance_BA_R0_R2	17,24346733	-77,67196655	-941,0369263	0,02711
Cloud_Mesh_Distance_BA_R0_R2	22,09049416	-78,39962769	-941,1257324	0,02206
Cloud_Mesh_Distance_BA_R0_R2	17,25774765	-77,579422	-941,0985107	0,029176
Cloud_Mesh_Distance_BA_R0_R2	17,36292458	-77,58261108	-941,0081177	0,035606
Cloud_Mesh_Distance_BA_R0_R2	21,67434692	-78,06991577	-940,9508057	-0,001443
Cloud_Mesh_Distance_BA_R0_R2	21,92982292	-78,27374268	-941,019104	0,021663
Cloud_Mesh_Distance_BA_R0_R2	17,42102242	-77,32164001	-941,2011719	0,043051
Cloud_Mesh_Distance_BA_R0_R2	17,46025848	-77,44482422	-941,0531616	0,045547
Cloud_Mesh_Distance_BA_R0_R2	17,67758942	-77,33148193	-941,0059815	0,058622
Cloud_Mesh_Distance_BA_R0_R2	21,41052628	-77,79193115	-941,0617676	-0,018796
Cloud_Mesh_Distance_BA_R0_R2	21,51155853	-77,86021423	-941,1086426	-0,012056
Cloud_Mesh_Distance_BA_R0_R2	21,58016968	-77,87004089	-941,2407227	-0,009716
Cloud_Mesh_Distance_BA_R0_R2	21,61073112	-77,95924377	-941,0817871	-0,007292
Cloud_Mesh_Distance_BA_R0_R2	17,71563339	-77,22085571	-941,0968018	0,059566
Cloud_Mesh_Distance_BA_R0_R2	17,57619095	-77,33097839	-941,078064	0,050157
Cloud_Mesh_Distance_BA_R0_R2	21,28243256	-77,69439697	-941,0339356	-0,018578
Cloud_Mesh_Distance_BA_R0_R2	21,37276459	-77,71318054	-941,1645508	-0,016536
Cloud_Mesh_Distance_BA_R0_R2	17,98032951	-77,13591003	-941,0283203	0,072214
Cloud_Mesh_Distance_BA_R0_R2	18,08882523	-77,15596008	-940,942627	0,074136
Cloud_Mesh_Distance_BA_R0_R2	20,96761894	-77,46611023	-940,9730225	-0,008208
Cloud_Mesh_Distance_BA_R0_R2	21,19199181	-77,60041809	-941,0667114	-0,0163
Cloud_Mesh_Distance_BA_R0_R2	18,11648941	-77,07670593	-941,0205689	0,075659
Cloud_Mesh_Distance_BA_R0_R2	18,26764679	-77,01921082	-941,0125732	0,084408
Cloud_Mesh_Distance_BA_R0_R2	18,13170052	-76,98138428	-941,1282349	0,0749
Cloud_Mesh_Distance_BA_R0_R2	18,57301903	-76,86721802	-941,0856323	0,09078
Cloud_Mesh_Distance_BA_R0_R2	18,29040909	-76,91793823	-941,1303101	0,08412
Cloud_Mesh_Distance_BA_R0_R2	18,72651672	-76,86669922	-941,0400391	0,089251

Cloud_Mesh_Distance_BA_R0_R2	20,69701004	-77,15841675	-941,2171021	0,016293
Cloud_Mesh_Distance_BA_R0_R2	20,5625515	-77,13485718	-941,0991821	0,023196
Cloud_Mesh_Distance_BA_R0_R2	20,80720711	-77,2525177	-941,1620483	0,010261
Cloud_Mesh_Distance_BA_R0_R2	20,77157593	-77,2840271	-941,0464478	0,009935
Cloud_Mesh_Distance_BA_R0_R2	20,70081711	-77,21551514	-941,0942383	0,014933
Cloud_Mesh_Distance_BA_R0_R2	20,88136292	-77,34558105	-941,0765381	0,003438
Cloud_Mesh_Distance_BA_R0_R2	20,99354362	-77,40481567	-941,1305542	-0,002691
Cloud_Mesh_Distance_BA_R0_R2	18,42221069	-76,87165833	-941,1342163	0,086765
Cloud_Mesh_Distance_BA_R0_R2	18,83012772	-76,90896606	-940,9515991	0,088302
Cloud_Mesh_Distance_BA_R0_R2	18,89671707	-76,76225281	-941,1741943	0,078101
Cloud_Mesh_Distance_BA_R0_R2	18,86379051	-76,83872986	-941,0532837	0,086128
Cloud_Mesh_Distance_BA_R0_R2	19,71294212	-76,88877869	-941,0305176	0,046028
Cloud_Mesh_Distance_BA_R0_R2	19,57701111	-76,80938721	-941,1553345	0,042925
Cloud_Mesh_Distance_BA_R0_R2	19,74490738	-76,82949829	-941,1759644	0,031158
Cloud_Mesh_Distance_BA_R0_R2	19,87723351	-76,94273376	-940,9841309	0,040345
Cloud_Mesh_Distance_BA_R0_R2	19,91877556	-76,88423157	-941,1364746	0,032727
Cloud_Mesh_Distance_BA_R0_R2	20,09797287	-76,93980408	-941,1178589	0,027636
Cloud_Mesh_Distance_BA_R0_R2	20,02594376	-76,97103882	-941,0027466	0,035954
Cloud_Mesh_Distance_BA_R0_R2	20,1948719	-77,01313782	-941,0227051	0,032441
Cloud_Mesh_Distance_BA_R0_R2	20,43747139	-77,0269928	-941,2086792	0,023825
Cloud_Mesh_Distance_BA_R0_R2	20,25193405	-76,96327209	-941,1826172	0,02625
Cloud_Mesh_Distance_BA_R0_R2	20,57655144	-77,07795715	-941,2486572	0,021597
Cloud_Mesh_Distance_BA_R0_R2	19,18387604	-76,76994324	-941,1558838	0,065271
Cloud_Mesh_Distance_BA_R0_R2	19,37693214	-76,78903198	-941,1451416	0,053435
Cloud_Mesh_Distance_BA_R0_R2	19,27067757	-76,71784973	-941,2724609	0,049572
Cloud_Mesh_Distance_BA_R0_R2	19,46078682	-76,73866272	-941,2767944	0,036676
Cloud_Mesh_Distance_BA_R0_R2	19,67411041	-76,76069641	-941,3098755	0,022556
Cloud_Mesh_Distance_BA_R0_R2	19,12578773	-84,14135742	-940,725769	-0,048246
Cloud_Mesh_Distance_BA_R0_R2	19,21410179	-84,12373352	-940,6522217	-0,05773
Cloud_Mesh_Distance_BA_R0_R2	19,24269295	-84,18563843	-940,755188	-0,044638
Cloud_Mesh_Distance_BA_R0_R2	19,61453438	-84,25917053	-940,8040161	-0,050258
Cloud_Mesh_Distance_BA_R0_R2	19,54262543	-84,1709137	-940,6563721	-0,066867
Cloud_Mesh_Distance_BA_R0_R2	18,69161987	-83,88220215	-940,5599365	-0,053924
Cloud_Mesh_Distance_BA_R0_R2	19,09991264	-84,07972717	-940,6248169	-0,059042
Cloud_Mesh_Distance_BA_R0_R2	19,35495949	-84,14427185	-940,6431274	-0,06477
Cloud_Mesh_Distance_BA_R0_R2	19,47107887	-84,08068848	-940,5206299	-0,080227
Cloud_Mesh_Distance_BA_R0_R2	19,89404678	-84,16687012	-940,661499	-0,059519
Cloud_Mesh_Distance_BA_R0_R2	19,88836288	-84,06770325	-940,5164185	-0,078211
Cloud_Mesh_Distance_BA_R0_R2	20,08081818	-84,14872742	-940,6720581	-0,058358
Cloud_Mesh_Distance_BA_R0_R2	20,27077103	-83,99661255	-940,5292969	-0,073126
Cloud_Mesh_Distance_BA_R0_R2	20,21423721	-84,08496094	-940,6213989	-0,060377
Cloud_Mesh_Distance_BA_R0_R2	20,37079239	-84,13893127	-940,7502441	-0,035822
Cloud_Mesh_Distance_BA_R0_R2	20,19679642	-84,17153931	-940,7381592	-0,044621
Cloud_Mesh_Distance_BA_R0_R2	20,51486778	-84,06199646	-940,704834	-0,032288
Cloud_Mesh_Distance_BA_R0_R2	18,43325233	-83,7268219	-940,5601807	-0,060698
Cloud_Mesh_Distance_BA_R0_R2	18,48557472	-83,6658783	-940,3963623	-0,077696
Cloud_Mesh_Distance_BA_R0_R2	18,62228775	-83,92289734	-940,7095947	-0,042746
Cloud_Mesh_Distance_BA_R0_R2	18,53863907	-83,83226013	-940,6281738	-0,048956
Cloud_Mesh_Distance_BA_R0_R2	18,60651016	-83,77264404	-940,4510498	-0,065827
Cloud_Mesh_Distance_BA_R0_R2	18,96879005	-83,9599762	-940,4906006	-0,069053

Cloud_Mesh_Distance_BA_R0_R2	18,93750381	-83,89306641	-940,402832	-0,078245
Cloud_Mesh_Distance_BA_R0_R2	18,84622192	-83,9312439	-940,5197754	-0,064373
Cloud_Mesh_Distance_BA_R0_R2	20,38467789	-84,03262329	-940,6160278	-0,05465
Cloud_Mesh_Distance_BA_R0_R2	20,55264091	-83,95335388	-940,5945435	-0,048474
Cloud_Mesh_Distance_BA_R0_R2	20,84906387	-83,86929321	-940,645752	-0,020517
Cloud_Mesh_Distance_BA_R0_R2	20,72675323	-83,93151855	-940,6502686	-0,025758
Cloud_Mesh_Distance_BA_R0_R2	18,10097694	-83,47732544	-940,5871582	-0,081954
Cloud_Mesh_Distance_BA_R0_R2	20,94926071	-83,65649414	-940,5117188	-0,057939
Cloud_Mesh_Distance_BA_R0_R2	20,88792419	-83,77409363	-940,5779419	-0,039875
Cloud_Mesh_Distance_BA_R0_R2	21,26732254	-83,61714172	-940,6494751	0,000687
Cloud_Mesh_Distance_BA_R0_R2	21,5220108	-83,68235779	-940,8238525	0,086555
Cloud_Mesh_Distance_BA_R0_R2	17,69193649	-83,02999878	-940,5575562	-0,109595
Cloud_Mesh_Distance_BA_R0_R2	17,72071648	-82,95909119	-940,4013062	-0,116009
Cloud_Mesh_Distance_BA_R0_R2	17,91588402	-83,19960022	-940,4221191	-0,102212
Cloud_Mesh_Distance_BA_R0_R2	17,85336494	-83,24932861	-940,6154785	-0,093216
Cloud_Mesh_Distance_BA_R0_R2	17,77796745	-83,14788818	-940,5842285	-0,104066
Cloud_Mesh_Distance_BA_R0_R2	21,26525497	-83,46853638	-940,5447388	-0,038108
Cloud_Mesh_Distance_BA_R0_R2	21,11540222	-83,46690369	-940,4594116	-0,0697
Cloud_Mesh_Distance_BA_R0_R2	21,1109581	-83,58665466	-940,5440064	-0,043385
Cloud_Mesh_Distance_BA_R0_R2	21,41772461	-83,50387573	-940,6540527	0,00356
Cloud_Mesh_Distance_BA_R0_R2	21,4156189	-83,62245178	-940,7317505	0,038924
Cloud_Mesh_Distance_BA_R0_R2	17,53874397	-82,81408691	-940,5325317	-0,119368
Cloud_Mesh_Distance_BA_R0_R2	17,63291931	-82,92860413	-940,5161743	-0,115112
Cloud_Mesh_Distance_BA_R0_R2	21,20845795	-83,38677979	-940,4556885	-0,069614
Cloud_Mesh_Distance_BA_R0_R2	21,4319458	-83,35853577	-940,5679932	-0,033804
Cloud_Mesh_Distance_BA_R0_R2	17,43711662	-82,72880554	-940,6171265	-0,115086
Cloud_Mesh_Distance_BA_R0_R2	17,47750282	-82,67997742	-940,467041	-0,120252
Cloud_Mesh_Distance_BA_R0_R2	21,31103325	-83,34080505	-940,4848023	-0,062944
Cloud_Mesh_Distance_BA_R0_R2	21,60253143	-83,21992493	-940,5892334	-0,028414
Cloud_Mesh_Distance_BA_R0_R2	17,35538673	-82,65783691	-940,6931152	-0,111681
Cloud_Mesh_Distance_BA_R0_R2	17,34365845	-82,43785095	-940,4194336	-0,119717
Cloud_Mesh_Distance_BA_R0_R2	17,31851387	-82,53184509	-940,5941162	-0,11593
Cloud_Mesh_Distance_BA_R0_R2	17,41167641	-82,55935669	-940,4381714	-0,122062
Cloud_Mesh_Distance_BA_R0_R2	17,39635658	-82,63288879	-940,5662842	-0,115812
Cloud_Mesh_Distance_BA_R0_R2	21,70499802	-83,08731079	-940,5778809	-0,040613
Cloud_Mesh_Distance_BA_R0_R2	17,24425507	-82,35951233	-940,5289307	-0,11319
Cloud_Mesh_Distance_BA_R0_R2	17,1622448	-82,21383667	-940,5300903	-0,10606
Cloud_Mesh_Distance_BA_R0_R2	21,75701332	-82,97013855	-940,5457153	-0,05447
Cloud_Mesh_Distance_BA_R0_R2	21,93902588	-82,96939087	-940,678833	-0,017269
Cloud_Mesh_Distance_BA_R0_R2	22,03816414	-82,84202576	-940,6864624	-0,026083
Cloud_Mesh_Distance_BA_R0_R2	22,00662422	-82,73348999	-940,6018066	-0,055855
Cloud_Mesh_Distance_BA_R0_R2	17,08061981	-82,13929749	-940,6311035	-0,096073
Cloud_Mesh_Distance_BA_R0_R2	17,04978943	-81,96856689	-940,5062866	-0,090339
Cloud_Mesh_Distance_BA_R0_R2	17,12631798	-82,0867157	-940,4611206	-0,101351
Cloud_Mesh_Distance_BA_R0_R2	22,19270325	-82,65858459	-940,7190552	-0,036557
Cloud_Mesh_Distance_BA_R0_R2	22,24449158	-82,51469421	-940,6936035	-0,056418
Cloud_Mesh_Distance_BA_R0_R2	22,14763641	-82,6027832	-940,6491699	-0,054598
Cloud_Mesh_Distance_BA_R0_R2	16,96160698	-81,88639832	-940,6328125	-0,080473
Cloud_Mesh_Distance_BA_R0_R2	22,07083511	-82,63079834	-940,597168	-0,062814
Cloud_Mesh_Distance_BA_R0_R2	16,92368889	-81,71948242	-940,5649414	-0,071015

Cloud_Mesh_Distance_BA_R0_R2	16,83936882	-81,63337708	-940,7033691	-0,063119
Cloud_Mesh_Distance_BA_R0_R2	22,42639542	-82,31997681	-940,786377	-0,06621
Cloud_Mesh_Distance_BA_R0_R2	22,31546402	-82,20822144	-940,593689	-0,099698
Cloud_Mesh_Distance_BA_R0_R2	22,33436966	-82,34898376	-940,6964722	-0,073732
Cloud_Mesh_Distance_BA_R0_R2	22,40045738	-82,23246765	-940,7070923	-0,088183
Cloud_Mesh_Distance_BA_R0_R2	22,41676903	-82,10896301	-940,6525269	-0,105553
Cloud_Mesh_Distance_BA_R0_R2	16,82907104	-81,26663208	-940,4780884	-0,060096
Cloud_Mesh_Distance_BA_R0_R2	16,6922245	-81,18424988	-940,769104	-0,04127
Cloud_Mesh_Distance_BA_R0_R2	16,79773331	-81,3460083	-940,5991821	-0,055457
Cloud_Mesh_Distance_BA_R0_R2	22,35018158	-82,09214783	-940,5627441	-0,110234
Cloud_Mesh_Distance_BA_R0_R2	16,61862564	-80,9756012	-940,8460083	-0,023926
Cloud_Mesh_Distance_BA_R0_R2	16,66728973	-81,03227234	-940,7548218	-0,034463
Cloud_Mesh_Distance_BA_R0_R2	22,67983818	-81,66221619	-940,7085571	-0,136234
Cloud_Mesh_Distance_BA_R0_R2	22,61702156	-81,63127136	-940,5891724	-0,137242
Cloud_Mesh_Distance_BA_R0_R2	22,61699295	-81,78552246	-940,7067261	-0,126843
Cloud_Mesh_Distance_BA_R0_R2	16,66623116	-80,88745117	-940,697876	-0,031097
Cloud_Mesh_Distance_BA_R0_R2	16,5975647	-80,78012085	-940,8178711	-0,017818
Cloud_Mesh_Distance_BA_R0_R2	22,69952965	-81,55943298	-940,6573486	-0,143994
Cloud_Mesh_Distance_BA_R0_R2	22,74917412	-81,59124756	-940,7719116	-0,137505
Cloud_Mesh_Distance_BA_R0_R2	16,66968727	-80,70344543	-940,6341553	-0,021808
Cloud_Mesh_Distance_BA_R0_R2	16,61162186	-80,55236816	-940,7252808	-0,005937
Cloud_Mesh_Distance_BA_R0_R2	16,68050957	-80,55088806	-940,5794678	-0,016507
Cloud_Mesh_Distance_BA_R0_R2	16,73704529	-80,62654114	-940,4716187	-0,029876
Cloud_Mesh_Distance_BA_R0_R2	16,66436386	-80,40228271	-940,5929565	-0,006454
Cloud_Mesh_Distance_BA_R0_R2	22,93389511	-81,14007568	-940,7578735	-0,173017
Cloud_Mesh_Distance_BA_R0_R2	22,86686325	-81,12498474	-940,6210327	-0,171159
Cloud_Mesh_Distance_BA_R0_R2	22,81546402	-81,27160645	-940,6293335	-0,162116
Cloud_Mesh_Distance_BA_R0_R2	22,86971283	-81,29188538	-940,7452393	-0,161259
Cloud_Mesh_Distance_BA_R0_R2	23,01076126	-81,06568909	-940,8843384	-0,186388
Cloud_Mesh_Distance_BA_R0_R2	16,65400124	-80,17933655	-940,6005249	0,00903
Cloud_Mesh_Distance_BA_R0_R2	16,63493919	-79,9690094	-940,6428833	0,028466
Cloud_Mesh_Distance_BA_R0_R2	16,5812645	-80,08035278	-940,7372437	0,027492
Cloud_Mesh_Distance_BA_R0_R2	16,57711601	-79,95285034	-940,7470093	0,038963
Cloud_Mesh_Distance_BA_R0_R2	22,9642334	-80,98136902	-940,7182007	-0,178705
Cloud_Mesh_Distance_BA_R0_R2	23,03972626	-80,64271545	-940,7521973	-0,182032
Cloud_Mesh_Distance_BA_R0_R2	16,60360718	-79,83357239	-940,7111816	0,040534
Cloud_Mesh_Distance_BA_R0_R2	16,67753601	-79,80667114	-940,5880737	0,031629
Cloud_Mesh_Distance_BA_R0_R2	16,63836288	-79,66090393	-940,6817017	0,045751
Cloud_Mesh_Distance_BA_R0_R2	16,71499252	-79,65829468	-940,5569458	0,035109
Cloud_Mesh_Distance_BA_R0_R2	22,98503494	-80,29350281	-940,6549072	-0,130269
Cloud_Mesh_Distance_BA_R0_R2	23,01143837	-80,39753723	-940,6885986	-0,14768
Cloud_Mesh_Distance_BA_R0_R2	16,57232475	-79,45877075	-940,8394165	0,054711
Cloud_Mesh_Distance_BA_R0_R2	23,08738518	-80,29710388	-940,8937988	-0,132198
Cloud_Mesh_Distance_BA_R0_R2	23,03577423	-80,3291626	-940,7532959	-0,140244
Cloud_Mesh_Distance_BA_R0_R2	23,03563309	-80,19897461	-940,7970581	-0,116975
Cloud_Mesh_Distance_BA_R0_R2	16,65455627	-79,19706726	-940,8111572	0,04118
Cloud_Mesh_Distance_BA_R0_R2	16,58023643	-79,31001282	-940,8756714	0,050013
Cloud_Mesh_Distance_BA_R0_R2	16,6515522	-79,3609314	-940,7493897	0,046649
Cloud_Mesh_Distance_BA_R0_R2	22,97510147	-80,06201172	-940,7307129	-0,101163
Cloud_Mesh_Distance_BA_R0_R2	16,7217083	-79,04804993	-940,7876587	0,027358

Cloud_Mesh_Distance_BA_R0_R2	22,83843994	-79,74490356	-940,6853638	-0,059365
Cloud_Mesh_Distance_BA_R0_R2	22,88545799	-79,70782471	-940,8229981	-0,052272
Cloud_Mesh_Distance_BA_R0_R2	22,88373947	-79,81048584	-940,7200317	-0,063999
Cloud_Mesh_Distance_BA_R0_R2	22,9319458	-79,86195374	-940,7810059	-0,066612
Cloud_Mesh_Distance_BA_R0_R2	23,00310898	-79,91444397	-940,90979	-0,07579
Cloud_Mesh_Distance_BA_R0_R2	16,77996063	-78,86611938	-940,8052979	0,011388
Cloud_Mesh_Distance_BA_R0_R2	16,82841873	-79,00105286	-940,6668091	0,020149
Cloud_Mesh_Distance_BA_R0_R2	22,94537735	-79,75761414	-940,9199829	-0,056675
Cloud_Mesh_Distance_BA_R0_R2	22,90594673	-79,66178894	-940,9341431	-0,047517
Cloud_Mesh_Distance_BA_R0_R2	16,97204971	-78,53091431	-940,7588501	-0,008254
Cloud_Mesh_Distance_BA_R0_R2	22,81246376	-79,59379578	-940,7841187	-0,041856
Cloud_Mesh_Distance_BA_R0_R2	17,04778862	-78,55503845	-940,6522217	-0,004055
Cloud_Mesh_Distance_BA_R0_R2	17,07696152	-78,4322052	-940,6935425	-0,002873
Cloud_Mesh_Distance_BA_R0_R2	22,52393913	-79,07221985	-940,8494263	0,00387
Cloud_Mesh_Distance_BA_R0_R2	22,60807037	-79,25517273	-940,7671509	-0,010221
Cloud_Mesh_Distance_BA_R0_R2	16,95537186	-78,24920654	-940,9403076	-0,00825
Cloud_Mesh_Distance_BA_R0_R2	17,00849152	-78,35115051	-940,8217163	-0,006951
Cloud_Mesh_Distance_BA_R0_R2	17,11677933	-78,27233887	-940,7487183	-0,000315
Cloud_Mesh_Distance_BA_R0_R2	17,18838501	-78,36528015	-940,6052856	0,004862
Cloud_Mesh_Distance_BA_R0_R2	17,01216888	-78,14302063	-940,9407349	-0,002999
Cloud_Mesh_Distance_BA_R0_R2	17,05000687	-78,2076416	-940,8614502	-0,004227
Cloud_Mesh_Distance_BA_R0_R2	17,07855225	-78,10449219	-940,8951416	-0,000459
Cloud_Mesh_Distance_BA_R0_R2	22,39331245	-78,90560913	-940,817627	0,018181
Cloud_Mesh_Distance_BA_R0_R2	22,25813484	-78,76948547	-940,7480469	0,024549
Cloud_Mesh_Distance_BA_R0_R2	22,20215797	-78,64993286	-940,8320923	0,030174
Cloud_Mesh_Distance_BA_R0_R2	17,04164314	-78,05821228	-940,9619141	-0,002075
Cloud_Mesh_Distance_BA_R0_R2	17,16006851	-77,98638916	-940,8886108	0,010397
Cloud_Mesh_Distance_BA_R0_R2	17,40567589	-77,8188324	-940,7757568	0,035857
Cloud_Mesh_Distance_BA_R0_R2	17,28548431	-77,85746765	-940,8569946	0,02274
Cloud_Mesh_Distance_BA_R0_R2	17,24855232	-78,15213013	-940,6837769	0,011257
Cloud_Mesh_Distance_BA_R0_R2	17,13050079	-78,14173889	-940,8164063	0,00472
Cloud_Mesh_Distance_BA_R0_R2	22,13118744	-78,6408844	-940,7041626	0,032188
Cloud_Mesh_Distance_BA_R0_R2	17,33194733	-77,730896	-940,9121704	0,031593
Cloud_Mesh_Distance_BA_R0_R2	21,9781723	-78,40197754	-940,8317261	0,027895
Cloud_Mesh_Distance_BA_R0_R2	22,02347946	-78,38269043	-940,9798584	0,028142
Cloud_Mesh_Distance_BA_R0_R2	21,90958214	-78,30879211	-940,887207	0,025569
Cloud_Mesh_Distance_BA_R0_R2	17,5084362	-77,54057312	-940,9266968	0,045859
Cloud_Mesh_Distance_BA_R0_R2	17,48287201	-77,66700745	-940,8353882	0,043546
Cloud_Mesh_Distance_BA_R0_R2	17,56173325	-77,78320313	-940,6670532	0,047759
Cloud_Mesh_Distance_BA_R0_R2	17,61340523	-77,65432739	-940,7391968	0,056117
Cloud_Mesh_Distance_BA_R0_R2	17,5919323	-77,57298279	-940,8308106	0,052936
Cloud_Mesh_Distance_BA_R0_R2	21,81336403	-78,18896484	-940,9587402	0,01655
Cloud_Mesh_Distance_BA_R0_R2	21,69871521	-78,159729	-940,8017578	0,010033
Cloud_Mesh_Distance_BA_R0_R2	17,7796402	-77,3309021	-940,9378662	0,062284
Cloud_Mesh_Distance_BA_R0_R2	17,64325333	-77,44450378	-940,9170532	0,05591
Cloud_Mesh_Distance_BA_R0_R2	17,92886925	-77,31596375	-940,857666	0,070939
Cloud_Mesh_Distance_BA_R0_R2	18,02988815	-77,38056946	-940,7225952	0,074097
Cloud_Mesh_Distance_BA_R0_R2	18,0479126	-77,28822327	-940,8151245	0,075169
Cloud_Mesh_Distance_BA_R0_R2	21,20092392	-77,71148682	-940,8753662	-0,022502
Cloud_Mesh_Distance_BA_R0_R2	21,24054909	-77,81776428	-940,7507935	-0,018783

Cloud_Mesh_Distance_BA_R0_R2	21,33543968	-77,8033905	-940,9102173	-0,02023
Cloud_Mesh_Distance_BA_R0_R2	21,47649002	-77,89801025	-940,9580078	-0,016298
Cloud_Mesh_Distance_BA_R0_R2	21,56913376	-77,99180603	-940,9251709	-0,007489
Cloud_Mesh_Distance_BA_R0_R2	17,87506104	-77,22851563	-940,9868774	0,064382
Cloud_Mesh_Distance_BA_R0_R2	18,00539398	-77,22331238	-940,9135132	0,072116
Cloud_Mesh_Distance_BA_R0_R2	18,12837601	-77,22013855	-940,8461914	0,07503
Cloud_Mesh_Distance_BA_R0_R2	21,06298447	-77,60487366	-940,8648682	-0,015585
Cloud_Mesh_Distance_BA_R0_R2	21,17080498	-77,63494873	-940,9659424	-0,017716
Cloud_Mesh_Distance_BA_R0_R2	18,22971344	-77,13052368	-940,8966675	0,081232
Cloud_Mesh_Distance_BA_R0_R2	18,25598907	-77,22531128	-940,7695923	0,083708
Cloud_Mesh_Distance_BA_R0_R2	18,51226044	-77,12332153	-940,7686768	0,089093
Cloud_Mesh_Distance_BA_R0_R2	18,39789581	-77,06895447	-940,8886108	0,086606
Cloud_Mesh_Distance_BA_R0_R2	18,67046356	-77,03288269	-940,8236694	0,094399
Cloud_Mesh_Distance_BA_R0_R2	20,80361176	-77,35572815	-940,9540405	0,004212
Cloud_Mesh_Distance_BA_R0_R2	20,70777702	-77,35250854	-940,8510742	-0,000162
Cloud_Mesh_Distance_BA_R0_R2	21,10430908	-77,56175232	-940,9987183	-0,013699
Cloud_Mesh_Distance_BA_R0_R2	18,56221962	-77,03686523	-940,8587647	0,093828
Cloud_Mesh_Distance_BA_R0_R2	18,45512199	-76,95892334	-941,0053101	0,085815
Cloud_Mesh_Distance_BA_R0_R2	18,68488312	-76,95933533	-940,9197388	0,093502
Cloud_Mesh_Distance_BA_R0_R2	18,88964272	-76,98136902	-940,8307495	0,09069
Cloud_Mesh_Distance_BA_R0_R2	18,77762985	-77,04768372	-940,7670288	0,095125
Cloud_Mesh_Distance_BA_R0_R2	18,9885807	-76,90977478	-940,9197998	0,089305
Cloud_Mesh_Distance_BA_R0_R2	19,59757805	-76,94396973	-940,8840332	0,060592
Cloud_Mesh_Distance_BA_R0_R2	19,69742584	-77,02406311	-940,7615967	0,062946
Cloud_Mesh_Distance_BA_R0_R2	19,78028488	-76,97250366	-940,8842163	0,053041
Cloud_Mesh_Distance_BA_R0_R2	19,8508358	-77,04101563	-940,7813721	0,053226
Cloud_Mesh_Distance_BA_R0_R2	19,94335365	-77,01081848	-940,8783569	0,044291
Cloud_Mesh_Distance_BA_R0_R2	20,00676346	-77,10140991	-940,7419434	0,045552
Cloud_Mesh_Distance_BA_R0_R2	20,11350441	-77,05513	-940,8842163	0,03604
Cloud_Mesh_Distance_BA_R0_R2	20,38768768	-77,21400452	-940,7895508	0,021363
Cloud_Mesh_Distance_BA_R0_R2	20,29398155	-77,10771179	-940,9068604	0,030573
Cloud_Mesh_Distance_BA_R0_R2	20,47632217	-77,17326355	-940,9348755	0,021845
Cloud_Mesh_Distance_BA_R0_R2	20,65808868	-77,25502014	-940,9664917	0,014737
Cloud_Mesh_Distance_BA_R0_R2	19,06150818	-76,82514954	-941,0535889	0,077619
Cloud_Mesh_Distance_BA_R0_R2	19,28219986	-76,84590149	-941,0194702	0,069568
Cloud_Mesh_Distance_BA_R0_R2	19,18976402	-76,9152832	-940,8934326	0,079793
Cloud_Mesh_Distance_BA_R0_R2	19,39647484	-76,92633057	-940,8818359	0,073491
Cloud_Mesh_Distance_BA_R0_R2	19,49012375	-76,86439514	-941,0146484	0,055544
Cloud_Mesh_Distance_BA_R0_R2	20,37236404	-77,0652771	-941,0551758	0,025193
Cloud_Mesh_Distance_BA_R0_R2	19,08086395	-84,00601196	-940,5090942	-0,06792
Cloud_Mesh_Distance_BA_R0_R2	19,04847145	-83,94029236	-940,4212036	-0,078151
Cloud_Mesh_Distance_BA_R0_R2	19,18648911	-83,95849609	-940,3982544	-0,085912
Cloud_Mesh_Distance_BA_R0_R2	19,25412369	-84,05825806	-940,5297241	-0,071312
Cloud_Mesh_Distance_BA_R0_R2	19,38352585	-83,98124695	-940,3883667	-0,094844
Cloud_Mesh_Distance_BA_R0_R2	19,69979095	-84,10070801	-940,5426025	-0,075039
Cloud_Mesh_Distance_BA_R0_R2	19,5963192	-83,99797058	-940,397522	-0,094282
Cloud_Mesh_Distance_BA_R0_R2	20,0398159	-84,07923889	-940,5633545	-0,073038
Cloud_Mesh_Distance_BA_R0_R2	18,91329575	-83,79412842	-940,2739258	-0,093643
Cloud_Mesh_Distance_BA_R0_R2	18,78461838	-83,8400116	-940,4151001	-0,076216
Cloud_Mesh_Distance_BA_R0_R2	18,73141861	-83,74333191	-940,3063355	-0,086446

Cloud_Mesh_Distance_BA_R0_R2	19,07758331	-83,87443542	-940,3157959	-0,09769
Cloud_Mesh_Distance_BA_R0_R2	19,28495026	-83,8653717	-940,2518311	-0,107995
Cloud_Mesh_Distance_BA_R0_R2	19,71051025	-83,90660095	-940,289856	-0,107861
Cloud_Mesh_Distance_BA_R0_R2	19,79918671	-83,99565125	-940,4085083	-0,092556
Cloud_Mesh_Distance_BA_R0_R2	19,89117622	-83,89758301	-940,3062134	-0,105586
Cloud_Mesh_Distance_BA_R0_R2	20,13602829	-83,80328369	-940,2731934	-0,110561
Cloud_Mesh_Distance_BA_R0_R2	20,04600525	-83,87576294	-940,3201294	-0,106035
Cloud_Mesh_Distance_BA_R0_R2	20,00593567	-83,97921753	-940,4276733	-0,087386
Cloud_Mesh_Distance_BA_R0_R2	20,2211647	-83,90124512	-940,4035034	-0,095474
Cloud_Mesh_Distance_BA_R0_R2	20,15099525	-84,01062012	-940,5062256	-0,076063
Cloud_Mesh_Distance_BA_R0_R2	18,342659	-83,61694336	-940,4794312	-0,07283
Cloud_Mesh_Distance_BA_R0_R2	18,28376007	-83,52561951	-940,4016724	-0,078868
Cloud_Mesh_Distance_BA_R0_R2	18,53888512	-83,60810852	-940,2593994	-0,084381
Cloud_Mesh_Distance_BA_R0_R2	18,40273666	-83,56608582	-940,3271484	-0,082578
Cloud_Mesh_Distance_BA_R0_R2	20,30091858	-83,73942566	-940,2732544	-0,110063
Cloud_Mesh_Distance_BA_R0_R2	20,38486481	-83,62303162	-940,2077026	-0,126156
Cloud_Mesh_Distance_BA_R0_R2	20,47281647	-83,82391357	-940,4260864	-0,089289
Cloud_Mesh_Distance_BA_R0_R2	20,38056374	-83,94418335	-940,5106812	-0,072021
Cloud_Mesh_Distance_BA_R0_R2	20,49039459	-83,67182922	-940,2961426	-0,111963
Cloud_Mesh_Distance_BA_R0_R2	20,70645523	-83,82582092	-940,536499	-0,055092
Cloud_Mesh_Distance_BA_R0_R2	20,80379486	-83,70651245	-940,4778442	-0,07197
Cloud_Mesh_Distance_BA_R0_R2	20,65493774	-83,69801331	-940,3966675	-0,092974
Cloud_Mesh_Distance_BA_R0_R2	18,05445862	-83,29975891	-940,3606567	-0,093051
Cloud_Mesh_Distance_BA_R0_R2	18,15952301	-83,43144226	-940,4167481	-0,085276
Cloud_Mesh_Distance_BA_R0_R2	18,21072006	-83,54534912	-940,5369873	-0,077037
Cloud_Mesh_Distance_BA_R0_R2	20,63420677	-83,57356262	-940,2844238	-0,119644
Cloud_Mesh_Distance_BA_R0_R2	20,99762344	-83,54277039	-940,4505005	-0,079214
Cloud_Mesh_Distance_BA_R0_R2	17,79551697	-83,07084656	-940,4301758	-0,108551
Cloud_Mesh_Distance_BA_R0_R2	17,88159943	-83,05836487	-940,2764893	-0,11776
Cloud_Mesh_Distance_BA_R0_R2	17,7987709	-83,01098633	-940,3418579	-0,115002
Cloud_Mesh_Distance_BA_R0_R2	18,07994461	-83,23857117	-940,2380981	-0,100233
Cloud_Mesh_Distance_BA_R0_R2	18,01255989	-83,22393799	-940,3103027	-0,101385
Cloud_Mesh_Distance_BA_R0_R2	20,72914696	-83,4775238	-940,2592773	-0,129347
Cloud_Mesh_Distance_BA_R0_R2	20,61508369	-83,4621582	-940,1907959	-0,141276
Cloud_Mesh_Distance_BA_R0_R2	21,12411118	-83,37425232	-940,3989258	-0,089618
Cloud_Mesh_Distance_BA_R0_R2	20,99461937	-83,42913818	-940,3657227	-0,09969
Cloud_Mesh_Distance_BA_R0_R2	20,82651138	-83,56265259	-940,3746338	-0,099623
Cloud_Mesh_Distance_BA_R0_R2	17,6991272	-82,8289032	-940,2711182	-0,130341
Cloud_Mesh_Distance_BA_R0_R2	17,63228989	-82,86170959	-940,4240112	-0,124406
Cloud_Mesh_Distance_BA_R0_R2	20,72173691	-83,3874054	-940,1898193	-0,141449
Cloud_Mesh_Distance_BA_R0_R2	17,58235741	-82,74772644	-940,3638916	-0,128029
Cloud_Mesh_Distance_BA_R0_R2	21,07546616	-83,30638123	-940,3234253	-0,110621
Cloud_Mesh_Distance_BA_R0_R2	21,32389641	-83,12828064	-940,3488159	-0,100327
Cloud_Mesh_Distance_BA_R0_R2	21,42000389	-83,20582581	-940,4625244	-0,071614
Cloud_Mesh_Distance_BA_R0_R2	21,23179245	-83,27325439	-940,3916016	-0,091204
Cloud_Mesh_Distance_BA_R0_R2	17,30800629	-82,33155823	-940,3618164	-0,116407
Cloud_Mesh_Distance_BA_R0_R2	17,36817169	-82,37385559	-940,2970581	-0,121724
Cloud_Mesh_Distance_BA_R0_R2	17,42883682	-82,46339417	-940,2908325	-0,127884
Cloud_Mesh_Distance_BA_R0_R2	21,44306946	-83,07417297	-940,3901367	-0,089921
Cloud_Mesh_Distance_BA_R0_R2	21,48327255	-82,9763031	-940,3515015	-0,095934

Cloud_Mesh_Distance_BA_R0_R2	21,59002304	-83,03038025	-940,4628296	-0,074964
Cloud_Mesh_Distance_BA_R0_R2	21,57591629	-82,84126282	-940,3273315	-0,099149
Cloud_Mesh_Distance_BA_R0_R2	17,24025536	-82,21026611	-940,3581543	-0,112687
Cloud_Mesh_Distance_BA_R0_R2	21,7235775	-82,85441589	-940,4484253	-0,081191
Cloud_Mesh_Distance_BA_R0_R2	21,83990097	-82,85095215	-940,5363159	-0,061413
Cloud_Mesh_Distance_BA_R0_R2	21,93423271	-82,84999084	-940,609314	-0,044743
Cloud_Mesh_Distance_BA_R0_R2	17,11470795	-82,00248718	-940,3955689	-0,097064
Cloud_Mesh_Distance_BA_R0_R2	21,84889603	-82,60490417	-940,3901978	-0,096372
Cloud_Mesh_Distance_BA_R0_R2	21,72977066	-82,6985321	-940,3527832	-0,100228
Cloud_Mesh_Distance_BA_R0_R2	21,9716835	-82,60713196	-940,4968872	-0,083609
Cloud_Mesh_Distance_BA_R0_R2	21,84017754	-82,72883606	-940,461792	-0,083698
Cloud_Mesh_Distance_BA_R0_R2	21,91928864	-82,75480652	-940,5422363	-0,066982
Cloud_Mesh_Distance_BA_R0_R2	16,97717667	-81,68325806	-940,4053345	-0,077209
Cloud_Mesh_Distance_BA_R0_R2	17,11681938	-81,91661072	-940,3039551	-0,093597
Cloud_Mesh_Distance_BA_R0_R2	16,99843216	-81,81018066	-940,4674072	-0,081662
Cloud_Mesh_Distance_BA_R0_R2	21,92033768	-82,47724915	-940,3711548	-0,102814
Cloud_Mesh_Distance_BA_R0_R2	22,12057114	-82,50013733	-940,5664063	-0,075683
Cloud_Mesh_Distance_BA_R0_R2	16,93872643	-81,53637695	-940,3839722	-0,074542
Cloud_Mesh_Distance_BA_R0_R2	22,08525467	-82,29162598	-940,4086914	-0,106539
Cloud_Mesh_Distance_BA_R0_R2	22,14196396	-82,15460205	-940,3810425	-0,114738
Cloud_Mesh_Distance_BA_R0_R2	22,22616005	-82,36064148	-940,5882568	-0,087032
Cloud_Mesh_Distance_BA_R0_R2	22,19662285	-82,24176025	-940,4879761	-0,103814
Cloud_Mesh_Distance_BA_R0_R2	22,13433456	-82,36231995	-940,4981079	-0,09557
Cloud_Mesh_Distance_BA_R0_R2	16,88799667	-81,58174133	-940,5373535	-0,068114
Cloud_Mesh_Distance_BA_R0_R2	16,91137314	-81,38963318	-940,3535767	-0,06833
Cloud_Mesh_Distance_BA_R0_R2	16,85912704	-81,43357849	-940,5039673	-0,062081
Cloud_Mesh_Distance_BA_R0_R2	22,35682297	-81,7797699	-940,376648	-0,130588
Cloud_Mesh_Distance_BA_R0_R2	22,26675415	-82,08920288	-940,4697266	-0,11623
Cloud_Mesh_Distance_BA_R0_R2	22,39268494	-81,97286987	-940,5366821	-0,119153
Cloud_Mesh_Distance_BA_R0_R2	22,5035038	-81,79483032	-940,5536499	-0,12887
Cloud_Mesh_Distance_BA_R0_R2	16,77038193	-81,18296814	-940,5762329	-0,050235
Cloud_Mesh_Distance_BA_R0_R2	16,79773903	-80,951828	-940,4185181	-0,051804
Cloud_Mesh_Distance_BA_R0_R2	16,82807732	-81,13319397	-940,4168701	-0,057752
Cloud_Mesh_Distance_BA_R0_R2	22,39362907	-81,83956909	-940,4525147	-0,128109
Cloud_Mesh_Distance_BA_R0_R2	22,43651009	-81,73880005	-940,4368897	-0,128388
Cloud_Mesh_Distance_BA_R0_R2	16,71907043	-81,01072693	-940,6218262	-0,04299
Cloud_Mesh_Distance_BA_R0_R2	16,72772026	-80,82273865	-940,5370483	-0,037262
Cloud_Mesh_Distance_BA_R0_R2	22,67759514	-81,4989624	-940,5782471	-0,150007
Cloud_Mesh_Distance_BA_R0_R2	22,60489655	-81,49479675	-940,4782105	-0,147274
Cloud_Mesh_Distance_BA_R0_R2	22,51131058	-81,63124084	-940,4542236	-0,139451
Cloud_Mesh_Distance_BA_R0_R2	22,76422882	-81,41854858	-940,65271	-0,155109
Cloud_Mesh_Distance_BA_R0_R2	16,78110504	-80,75694275	-940,4022827	-0,044195
Cloud_Mesh_Distance_BA_R0_R2	22,68945313	-81,39859009	-940,526062	-0,150186
Cloud_Mesh_Distance_BA_R0_R2	16,71900177	-80,46891785	-940,4884033	-0,021089
Cloud_Mesh_Distance_BA_R0_R2	16,72766876	-80,3110199	-940,4608154	-0,009588
Cloud_Mesh_Distance_BA_R0_R2	16,72161865	-79,9666748	-940,4868774	0,015643
Cloud_Mesh_Distance_BA_R0_R2	16,73948097	-80,14332581	-940,4395752	0,000981
Cloud_Mesh_Distance_BA_R0_R2	22,90267181	-80,89019775	-940,5634155	-0,171625
Cloud_Mesh_Distance_BA_R0_R2	22,81440544	-80,86268616	-940,4241943	-0,159009
Cloud_Mesh_Distance_BA_R0_R2	22,98825455	-80,81459045	-940,6898193	-0,178166

Cloud_Mesh_Distance_BA_R0_R2	22,88906097	-80,51519775	-940,465332	-0,135411
Cloud_Mesh_Distance_BA_R0_R2	22,97328758	-80,51022339	-940,6067505	-0,154846
Cloud_Mesh_Distance_BA_R0_R2	23,04706573	-80,44749451	-940,7597656	-0,156789
Cloud_Mesh_Distance_BA_R0_R2	22,96252441	-80,71092224	-940,6099854	-0,173647
Cloud_Mesh_Distance_BA_R0_R2	16,75577164	-79,76243591	-940,4624023	0,026061
Cloud_Mesh_Distance_BA_R0_R2	22,91142654	-80,3694458	-940,5083618	-0,123472
Cloud_Mesh_Distance_BA_R0_R2	22,83658981	-80,27848816	-940,4098511	-0,107804
Cloud_Mesh_Distance_BA_R0_R2	22,84498787	-80,43052673	-940,4033814	-0,123688
Cloud_Mesh_Distance_BA_R0_R2	16,72460938	-79,47465515	-940,597229	0,040328
Cloud_Mesh_Distance_BA_R0_R2	16,78052521	-79,5983429	-940,4658814	0,029198
Cloud_Mesh_Distance_BA_R0_R2	22,90756035	-80,17214966	-940,5502319	-0,107633
Cloud_Mesh_Distance_BA_R0_R2	16,8288269	-79,3321228	-940,4941406	0,032625
Cloud_Mesh_Distance_BA_R0_R2	16,87865829	-79,13818359	-940,5202026	0,021534
Cloud_Mesh_Distance_BA_R0_R2	16,75408745	-79,21984863	-940,6582642	0,033282
Cloud_Mesh_Distance_BA_R0_R2	16,9883709	-79,08982849	-940,3892822	0,018674
Cloud_Mesh_Distance_BA_R0_R2	22,69697189	-79,64466858	-940,5157471	-0,046121
Cloud_Mesh_Distance_BA_R0_R2	22,87198257	-79,9309845	-940,6066284	-0,082089
Cloud_Mesh_Distance_BA_R0_R2	22,78505135	-79,78588867	-940,5527344	-0,061482
Cloud_Mesh_Distance_BA_R0_R2	16,88836098	-78,81210327	-940,6966553	0,005057
Cloud_Mesh_Distance_BA_R0_R2	16,94933701	-78,93293762	-940,5413818	0,012122
Cloud_Mesh_Distance_BA_R0_R2	17,03156281	-78,94633484	-940,4180908	0,013977
Cloud_Mesh_Distance_BA_R0_R2	16,98139191	-78,75109863	-940,6132813	0,000628
Cloud_Mesh_Distance_BA_R0_R2	17,04771042	-78,81130981	-940,4848023	0,008821
Cloud_Mesh_Distance_BA_R0_R2	22,77353859	-79,64758301	-940,6478272	-0,047427
Cloud_Mesh_Distance_BA_R0_R2	22,71827888	-79,47247314	-940,7236328	-0,032549
Cloud_Mesh_Distance_BA_R0_R2	22,60018921	-79,41699219	-940,5634155	-0,025199
Cloud_Mesh_Distance_BA_R0_R2	16,93868446	-78,67453003	-940,7141113	0,000012
Cloud_Mesh_Distance_BA_R0_R2	17,00064087	-78,63172913	-940,6635742	-0,005084
Cloud_Mesh_Distance_BA_R0_R2	17,06026459	-78,66085815	-940,5680542	0,000634
Cloud_Mesh_Distance_BA_R0_R2	17,127491	-78,53364563	-940,565979	0,001693
Cloud_Mesh_Distance_BA_R0_R2	22,21075439	-78,93939209	-940,4604492	0,017545
Cloud_Mesh_Distance_BA_R0_R2	22,45688629	-79,26850891	-940,4832153	-0,009902
Cloud_Mesh_Distance_BA_R0_R2	22,40820503	-79,11779785	-940,564148	0,006704
Cloud_Mesh_Distance_BA_R0_R2	22,51708984	-79,23139954	-940,619751	-0,006083
Cloud_Mesh_Distance_BA_R0_R2	22,45962906	-79,07440186	-940,7069092	0,005265
Cloud_Mesh_Distance_BA_R0_R2	22,53053665	-79,15792847	-940,7357178	-0,001554
Cloud_Mesh_Distance_BA_R0_R2	17,23702431	-78,50343323	-940,449646	0,011784
Cloud_Mesh_Distance_BA_R0_R2	22,35679626	-78,96940613	-940,6531372	0,013976
Cloud_Mesh_Distance_BA_R0_R2	22,31670952	-78,87431335	-940,7072754	0,020205
Cloud_Mesh_Distance_BA_R0_R2	22,24658203	-78,85801697	-940,6055908	0,021318
Cloud_Mesh_Distance_BA_R0_R2	22,29102516	-78,99768066	-940,513916	0,011776
Cloud_Mesh_Distance_BA_R0_R2	22,22883797	-78,90911865	-940,519165	0,016072
Cloud_Mesh_Distance_BA_R0_R2	17,3767643	-78,14918518	-940,5473633	0,030282
Cloud_Mesh_Distance_BA_R0_R2	22,18165016	-78,74406433	-940,6436157	0,030147
Cloud_Mesh_Distance_BA_R0_R2	17,45100403	-77,92840576	-940,6460571	0,03715
Cloud_Mesh_Distance_BA_R0_R2	17,32007599	-77,96211243	-940,746521	0,025491
Cloud_Mesh_Distance_BA_R0_R2	17,3731575	-78,04631042	-940,6304321	0,025793
Cloud_Mesh_Distance_BA_R0_R2	17,47280312	-78,04812622	-940,5283203	0,03617
Cloud_Mesh_Distance_BA_R0_R2	22,00874901	-78,58712769	-940,5769653	0,036197
Cloud_Mesh_Distance_BA_R0_R2	21,96406555	-78,45896912	-940,6991577	0,034823

Cloud_Mesh_Distance_BA_R0_R2	22,10937309	-78,69195557	-940,593811	0,033091
Cloud_Mesh_Distance_BA_R0_R2	22,05016327	-78,54830933	-940,7036743	0,034763
Cloud_Mesh_Distance_BA_R0_R2	17,5751152	-77,92324829	-940,53479	0,050288
Cloud_Mesh_Distance_BA_R0_R2	17,77983093	-77,74935913	-940,5175171	0,063164
Cloud_Mesh_Distance_BA_R0_R2	17,71763611	-77,69017029	-940,6219482	0,060107
Cloud_Mesh_Distance_BA_R0_R2	17,8279438	-77,68241882	-940,545044	0,065742
Cloud_Mesh_Distance_BA_R0_R2	21,58426094	-78,14909363	-940,6473389	0,012434
Cloud_Mesh_Distance_BA_R0_R2	21,71774673	-78,24078369	-940,6792603	0,023445
Cloud_Mesh_Distance_BA_R0_R2	21,51506042	-78,21051025	-940,465271	0,024105
Cloud_Mesh_Distance_BA_R0_R2	21,81891441	-78,35546875	-940,6358032	0,031929
Cloud_Mesh_Distance_BA_R0_R2	21,8968811	-78,35552979	-940,7659912	0,028175
Cloud_Mesh_Distance_BA_R0_R2	17,84150696	-77,59136963	-940,6245117	0,068742
Cloud_Mesh_Distance_BA_R0_R2	17,8582859	-77,73220825	-940,4740601	0,067655
Cloud_Mesh_Distance_BA_R0_R2	21,29791832	-77,95014954	-940,6121826	-0,011649
Cloud_Mesh_Distance_BA_R0_R2	21,29654884	-78,04534912	-940,4725342	-0,001005
Cloud_Mesh_Distance_BA_R0_R2	21,42382813	-78,08529663	-940,5498657	0,00882
Cloud_Mesh_Distance_BA_R0_R2	21,46406174	-78,02049255	-940,7063599	-0,005771
Cloud_Mesh_Distance_BA_R0_R2	21,58253288	-78,05970764	-940,80896	0,000251
Cloud_Mesh_Distance_BA_R0_R2	21,49695396	-77,97567749	-940,8363037	-0,008489
Cloud_Mesh_Distance_BA_R0_R2	21,81046104	-78,26547241	-940,7854614	0,019271
Cloud_Mesh_Distance_BA_R0_R2	17,70946503	-77,56570435	-940,7477417	0,062781
Cloud_Mesh_Distance_BA_R0_R2	18,00893402	-77,51861572	-940,5856934	0,077054
Cloud_Mesh_Distance_BA_R0_R2	17,84778595	-77,44830322	-940,7686157	0,064457
Cloud_Mesh_Distance_BA_R0_R2	18,15737534	-77,30459595	-940,7324219	0,078894
Cloud_Mesh_Distance_BA_R0_R2	21,38745499	-77,90995789	-940,793457	-0,014573
Cloud_Mesh_Distance_BA_R0_R2	18,29413033	-77,30534363	-940,6558838	0,084479
Cloud_Mesh_Distance_BA_R0_R2	18,43030357	-77,2885437	-940,6057739	0,087763
Cloud_Mesh_Distance_BA_R0_R2	20,5452137	-77,45018005	-940,5629883	-0,000529
Cloud_Mesh_Distance_BA_R0_R2	20,67773438	-77,43115234	-940,703125	-0,005013
Cloud_Mesh_Distance_BA_R0_R2	20,78761482	-77,5788269	-940,6015625	-0,019358
Cloud_Mesh_Distance_BA_R0_R2	20,95021057	-77,61978149	-940,7077026	-0,023579
Cloud_Mesh_Distance_BA_R0_R2	21,0092392	-77,71838379	-940,632019	-0,024048
Cloud_Mesh_Distance_BA_R0_R2	21,0977211	-77,71536255	-940,7352295	-0,021361
Cloud_Mesh_Distance_BA_R0_R2	18,37103653	-77,16331482	-940,7851563	0,08524
Cloud_Mesh_Distance_BA_R0_R2	18,36443901	-77,23674011	-940,7001343	0,086052
Cloud_Mesh_Distance_BA_R0_R2	18,47356796	-77,20736694	-940,6826782	0,08805
Cloud_Mesh_Distance_BA_R0_R2	18,80578423	-77,15759277	-940,6124878	0,094928
Cloud_Mesh_Distance_BA_R0_R2	18,95716095	-77,07525635	-940,6791992	0,094379
Cloud_Mesh_Distance_BA_R0_R2	18,65724754	-77,09460449	-940,7470093	0,092588
Cloud_Mesh_Distance_BA_R0_R2	19,05115891	-77,18597412	-940,5108643	0,091144
Cloud_Mesh_Distance_BA_R0_R2	20,2060318	-77,29031372	-940,5501099	0,031351
Cloud_Mesh_Distance_BA_R0_R2	20,12345123	-77,20152283	-940,6367188	0,03901
Cloud_Mesh_Distance_BA_R0_R2	20,36215401	-77,3724823	-940,5341797	0,01224
Cloud_Mesh_Distance_BA_R0_R2	20,56634903	-77,28929138	-940,8164673	0,011054
Cloud_Mesh_Distance_BA_R0_R2	20,47483063	-77,33140564	-940,6742554	0,00981
Cloud_Mesh_Distance_BA_R0_R2	20,83560753	-77,519104	-940,730835	-0,012391
Cloud_Mesh_Distance_BA_R0_R2	20,93002892	-77,5295105	-940,819519	-0,011715
Cloud_Mesh_Distance_BA_R0_R2	20,8228569	-77,4407959	-940,8352661	-0,004272
Cloud_Mesh_Distance_BA_R0_R2	19,10108185	-77,00379944	-940,7576904	0,091569
Cloud_Mesh_Distance_BA_R0_R2	19,11498833	-77,0945282	-940,6219482	0,094322

Cloud_Mesh_Distance_BA_R0_R2	19,25419617	-77,07498169	-940,6350098	0,089515
Cloud_Mesh_Distance_BA_R0_R2	19,50936127	-77,00654602	-940,7542114	0,073773
Cloud_Mesh_Distance_BA_R0_R2	19,4314785	-77,07600403	-940,6330566	0,082947
Cloud_Mesh_Distance_BA_R0_R2	19,31351089	-76,99775696	-940,7545776	0,081088
Cloud_Mesh_Distance_BA_R0_R2	19,8244915	-77,11006165	-940,6536255	0,059727
Cloud_Mesh_Distance_BA_R0_R2	19,97719955	-77,1710968	-940,6132813	0,047482
Cloud_Mesh_Distance_BA_R0_R2	20,29517555	-77,25614929	-940,65625	0,024538
Cloud_Mesh_Distance_BA_R0_R2	20,20537758	-77,15158081	-940,7664185	0,030349
Cloud_Mesh_Distance_BA_R0_R2	18,60538483	-83,68977356	-940,3223267	-0,081494
Cloud_Mesh_Distance_BA_R0_R2	18,67577553	-83,65060425	-940,2146606	-0,089215
Cloud_Mesh_Distance_BA_R0_R2	18,81955147	-83,69290161	-940,185791	-0,100514
Cloud_Mesh_Distance_BA_R0_R2	19,10705566	-83,76948547	-940,1724854	-0,113415
Cloud_Mesh_Distance_BA_R0_R2	19,51373291	-83,89422607	-940,2674561	-0,110917
Cloud_Mesh_Distance_BA_R0_R2	19,45480156	-83,77656555	-940,1334229	-0,127661
Cloud_Mesh_Distance_BA_R0_R2	19,8078537	-83,82527161	-940,2122192	-0,12032
Cloud_Mesh_Distance_BA_R0_R2	19,97521019	-83,78401184	-940,206604	-0,122103
Cloud_Mesh_Distance_BA_R0_R2	18,76148224	-83,59963989	-940,1000977	-0,10818
Cloud_Mesh_Distance_BA_R0_R2	18,95452881	-83,71224976	-940,1507568	-0,111854
Cloud_Mesh_Distance_BA_R0_R2	19,20284843	-83,69657898	-940,0684815	-0,130732
Cloud_Mesh_Distance_BA_R0_R2	19,27916336	-83,76261902	-940,1307983	-0,125309
Cloud_Mesh_Distance_BA_R0_R2	19,48591805	-83,68162537	-940,0357666	-0,137223
Cloud_Mesh_Distance_BA_R0_R2	19,80835724	-83,71643066	-940,1043091	-0,134741
Cloud_Mesh_Distance_BA_R0_R2	19,65265083	-83,80778503	-940,1743164	-0,125243
Cloud_Mesh_Distance_BA_R0_R2	19,99697495	-83,64147949	-940,0813599	-0,137624
Cloud_Mesh_Distance_BA_R0_R2	20,2637825	-83,62252808	-940,1570435	-0,134291
Cloud_Mesh_Distance_BA_R0_R2	20,13722992	-83,70193481	-940,1790772	-0,125745
Cloud_Mesh_Distance_BA_R0_R2	18,19988251	-83,32415771	-940,2073364	-0,098115
Cloud_Mesh_Distance_BA_R0_R2	18,3147316	-83,45928955	-940,2651367	-0,091327
Cloud_Mesh_Distance_BA_R0_R2	19,61889648	-83,70474243	-940,0656738	-0,137944
Cloud_Mesh_Distance_BA_R0_R2	18,00472832	-83,15368652	-940,2254639	-0,111257
Cloud_Mesh_Distance_BA_R0_R2	18,05213737	-83,0969696	-940,0985107	-0,120293
Cloud_Mesh_Distance_BA_R0_R2	18,13734245	-83,18470764	-940,1029663	-0,115563
Cloud_Mesh_Distance_BA_R0_R2	20,3736515	-83,51673889	-940,1188965	-0,143555
Cloud_Mesh_Distance_BA_R0_R2	20,49943542	-83,55213928	-940,2024536	-0,136379
Cloud_Mesh_Distance_BA_R0_R2	17,80187988	-82,8507843	-940,1464233	-0,139514
Cloud_Mesh_Distance_BA_R0_R2	17,90414047	-82,94941711	-940,1183472	-0,130331
Cloud_Mesh_Distance_BA_R0_R2	17,7996254	-82,94636536	-940,258606	-0,124674
Cloud_Mesh_Distance_BA_R0_R2	17,97171021	-83,05558777	-940,1495361	-0,118948
Cloud_Mesh_Distance_BA_R0_R2	20,43947601	-83,39665222	-940,0585938	-0,154993
Cloud_Mesh_Distance_BA_R0_R2	20,50647354	-83,45114136	-940,1293335	-0,146885
Cloud_Mesh_Distance_BA_R0_R2	20,85896492	-83,39175415	-940,2652588	-0,12192
Cloud_Mesh_Distance_BA_R0_R2	20,80487061	-83,27914429	-940,1560059	-0,146565
Cloud_Mesh_Distance_BA_R0_R2	20,97278976	-83,24984741	-940,2260742	-0,128611
Cloud_Mesh_Distance_BA_R0_R2	20,97404099	-83,34538269	-940,2943115	-0,115266
Cloud_Mesh_Distance_BA_R0_R2	17,63567924	-82,69152832	-940,211853	-0,133951
Cloud_Mesh_Distance_BA_R0_R2	17,88225365	-82,79469299	-939,9900513	-0,145633
Cloud_Mesh_Distance_BA_R0_R2	17,73778725	-82,72213745	-940,0977783	-0,144437
Cloud_Mesh_Distance_BA_R0_R2	21,17602348	-83,01229858	-940,1766968	-0,125726
Cloud_Mesh_Distance_BA_R0_R2	21,15927124	-83,15779114	-940,2687378	-0,116665
Cloud_Mesh_Distance_BA_R0_R2	21,29184532	-83,02677917	-940,2591553	-0,113978

Cloud_Mesh_Distance_BA_R0_R2	17,52640533	-82,59611511	-940,2767944	-0,133902
Cloud_Mesh_Distance_BA_R0_R2	21,28434563	-82,92405701	-940,1820679	-0,119901
Cloud_Mesh_Distance_BA_R0_R2	21,39147186	-83,0234375	-940,3210449	-0,10072
Cloud_Mesh_Distance_BA_R0_R2	17,34913063	-82,27035522	-940,2161865	-0,121796
Cloud_Mesh_Distance_BA_R0_R2	17,44494057	-82,29295349	-940,0794067	-0,134476
Cloud_Mesh_Distance_BA_R0_R2	17,41972351	-82,36662292	-940,1985474	-0,129026
Cloud_Mesh_Distance_BA_R0_R2	21,40887451	-82,90786743	-940,2532349	-0,108815
Cloud_Mesh_Distance_BA_R0_R2	21,48121643	-82,77102661	-940,2103272	-0,107228
Cloud_Mesh_Distance_BA_R0_R2	17,29456902	-82,11898804	-940,1550293	-0,112785
Cloud_Mesh_Distance_BA_R0_R2	17,18389511	-82,05674744	-940,3052368	-0,104382
Cloud_Mesh_Distance_BA_R0_R2	21,89222336	-82,36070251	-940,2747803	-0,113918
Cloud_Mesh_Distance_BA_R0_R2	21,77653122	-82,51649475	-940,272583	-0,108548
Cloud_Mesh_Distance_BA_R0_R2	22,03775215	-82,43003845	-940,4473267	-0,097191
Cloud_Mesh_Distance_BA_R0_R2	21,98900223	-82,35401917	-940,3569946	-0,10808
Cloud_Mesh_Distance_BA_R0_R2	17,00607681	-81,61170959	-940,2860107	-0,078759
Cloud_Mesh_Distance_BA_R0_R2	17,06606293	-81,57038879	-940,1314697	-0,08402
Cloud_Mesh_Distance_BA_R0_R2	17,05742264	-81,75418091	-940,286438	-0,083222
Cloud_Mesh_Distance_BA_R0_R2	17,1274395	-81,82437134	-940,1994629	-0,092086
Cloud_Mesh_Distance_BA_R0_R2	21,77045822	-82,3553772	-940,1682129	-0,112451
Cloud_Mesh_Distance_BA_R0_R2	21,88442993	-82,24894714	-940,2010498	-0,117523
Cloud_Mesh_Distance_BA_R0_R2	21,94749832	-82,11795044	-940,1820679	-0,119957
Cloud_Mesh_Distance_BA_R0_R2	22,00617981	-82,22288513	-940,2945557	-0,118294
Cloud_Mesh_Distance_BA_R0_R2	16,99775887	-81,49726868	-940,2263794	-0,07404
Cloud_Mesh_Distance_BA_R0_R2	17,03420067	-81,41944885	-940,1062622	-0,075728
Cloud_Mesh_Distance_BA_R0_R2	22,00364494	-81,98956299	-940,163269	-0,1216
Cloud_Mesh_Distance_BA_R0_R2	22,09547806	-82,04623413	-940,2747192	-0,124294
Cloud_Mesh_Distance_BA_R0_R2	16,96557808	-81,35705566	-940,2158814	-0,069547
Cloud_Mesh_Distance_BA_R0_R2	16,89086914	-81,22377014	-940,3146362	-0,066571
Cloud_Mesh_Distance_BA_R0_R2	22,26271248	-81,9226532	-940,364502	-0,132282
Cloud_Mesh_Distance_BA_R0_R2	22,10585594	-81,85900879	-940,18396	-0,123645
Cloud_Mesh_Distance_BA_R0_R2	22,19302177	-82,05076599	-940,3705444	-0,123655
Cloud_Mesh_Distance_BA_R0_R2	16,86103439	-81,04316711	-940,3080444	-0,061594
Cloud_Mesh_Distance_BA_R0_R2	16,97248459	-81,16482544	-940,1152344	-0,071519
Cloud_Mesh_Distance_BA_R0_R2	22,27726936	-81,74845886	-940,2803345	-0,134764
Cloud_Mesh_Distance_BA_R0_R2	16,84430885	-80,75550842	-940,2666016	-0,049411
Cloud_Mesh_Distance_BA_R0_R2	16,86622238	-80,9122467	-940,2558594	-0,056629
Cloud_Mesh_Distance_BA_R0_R2	22,3517952	-81,47999573	-940,2102051	-0,139408
Cloud_Mesh_Distance_BA_R0_R2	22,3302536	-81,59690857	-940,2491455	-0,141778
Cloud_Mesh_Distance_BA_R0_R2	22,47782898	-81,47683716	-940,3269653	-0,142814
Cloud_Mesh_Distance_BA_R0_R2	22,40164185	-81,66036987	-940,3525391	-0,132158
Cloud_Mesh_Distance_BA_R0_R2	16,80023193	-80,62976074	-940,3378296	-0,03694
Cloud_Mesh_Distance_BA_R0_R2	22,40643311	-81,34902954	-940,1984253	-0,13855
Cloud_Mesh_Distance_BA_R0_R2	22,62872696	-81,33946228	-940,4133301	-0,149015
Cloud_Mesh_Distance_BA_R0_R2	16,85355377	-80,55853271	-940,2201538	-0,039968
Cloud_Mesh_Distance_BA_R0_R2	16,86335182	-80,37039185	-940,1936035	-0,029526
Cloud_Mesh_Distance_BA_R0_R2	22,57796288	-81,09664917	-940,2468262	-0,140879
Cloud_Mesh_Distance_BA_R0_R2	22,54323578	-81,26625061	-940,2853394	-0,142927
Cloud_Mesh_Distance_BA_R0_R2	22,79152298	-81,03938293	-940,4622803	-0,158216
Cloud_Mesh_Distance_BA_R0_R2	22,67180252	-81,15621948	-940,3690796	-0,151567
Cloud_Mesh_Distance_BA_R0_R2	22,75229645	-81,24954224	-940,5195923	-0,1569

Cloud_Mesh_Distance_BA_R0_R2	16,78557205	-80,45454407	-940,3500366	-0,026125
Cloud_Mesh_Distance_BA_R0_R2	22,555336	-80,9553833	-940,1771851	-0,137806
Cloud_Mesh_Distance_BA_R0_R2	16,81188011	-79,87330627	-940,3405762	0,010964
Cloud_Mesh_Distance_BA_R0_R2	16,78956604	-80,04348755	-940,3538818	0,003028
Cloud_Mesh_Distance_BA_R0_R2	16,807024	-80,22033691	-940,3065186	-0,01441
Cloud_Mesh_Distance_BA_R0_R2	16,88442039	-80,20635986	-940,1619873	-0,019031
Cloud_Mesh_Distance_BA_R0_R2	22,69772148	-80,94932556	-940,3139038	-0,146931
Cloud_Mesh_Distance_BA_R0_R2	22,8819828	-80,68766785	-940,4744873	-0,155918
Cloud_Mesh_Distance_BA_R0_R2	22,76808739	-80,74552917	-940,3359375	-0,143105
Cloud_Mesh_Distance_BA_R0_R2	16,83291054	-79,68560791	-940,3518066	0,021541
Cloud_Mesh_Distance_BA_R0_R2	22,81363106	-80,56802368	-940,364624	-0,132946
Cloud_Mesh_Distance_BA_R0_R2	16,90767097	-79,77304077	-940,2003784	0,012066
Cloud_Mesh_Distance_BA_R0_R2	16,81177521	-79,4747467	-940,4591065	0,030676
Cloud_Mesh_Distance_BA_R0_R2	16,84214783	-79,54988098	-940,3815918	0,028345
Cloud_Mesh_Distance_BA_R0_R2	16,9029026	-79,57228088	-940,2752075	0,023415
Cloud_Mesh_Distance_BA_R0_R2	16,88058281	-79,43215942	-940,3685913	0,026193
Cloud_Mesh_Distance_BA_R0_R2	22,64399147	-79,80403137	-940,3286743	-0,047641
Cloud_Mesh_Distance_BA_R0_R2	22,68650627	-79,9450531	-940,3161011	-0,06465
Cloud_Mesh_Distance_BA_R0_R2	22,8187294	-80,08779907	-940,4385376	-0,086609
Cloud_Mesh_Distance_BA_R0_R2	17,06362534	-79,01678467	-940,3275757	0,020376
Cloud_Mesh_Distance_BA_R0_R2	16,92650414	-79,26948547	-940,3777466	0,024856
Cloud_Mesh_Distance_BA_R0_R2	17,05654335	-79,11372375	-940,2768555	0,020902
Cloud_Mesh_Distance_BA_R0_R2	22,77929306	-79,92652893	-940,4536743	-0,064736
Cloud_Mesh_Distance_BA_R0_R2	22,72734833	-79,81619263	-940,4393921	-0,053778
Cloud_Mesh_Distance_BA_R0_R2	22,71623421	-79,90600586	-940,3728638	-0,059185
Cloud_Mesh_Distance_BA_R0_R2	17,10882759	-78,89468384	-940,3446655	0,014311
Cloud_Mesh_Distance_BA_R0_R2	17,15480995	-78,6988678	-940,4178467	0,009555
Cloud_Mesh_Distance_BA_R0_R2	22,41785622	-79,3372345	-940,3656616	-0,010953
Cloud_Mesh_Distance_BA_R0_R2	22,51042557	-79,41720581	-940,4243164	-0,019978
Cloud_Mesh_Distance_BA_R0_R2	22,4524231	-79,46089172	-940,310669	-0,01895
Cloud_Mesh_Distance_BA_R0_R2	22,52423286	-79,33415222	-940,5216675	-0,016946
Cloud_Mesh_Distance_BA_R0_R2	17,35111809	-78,47558594	-940,3312378	0,024016
Cloud_Mesh_Distance_BA_R0_R2	22,22343826	-79,03210449	-940,3866577	0,010667
Cloud_Mesh_Distance_BA_R0_R2	22,14319992	-78,92041016	-940,3897095	0,021887
Cloud_Mesh_Distance_BA_R0_R2	22,12059402	-79,00608826	-940,2854004	0,016904
Cloud_Mesh_Distance_BA_R0_R2	22,34053421	-79,17420959	-940,4082031	-0,004542
Cloud_Mesh_Distance_BA_R0_R2	17,43848419	-78,34590149	-940,3309937	0,029228
Cloud_Mesh_Distance_BA_R0_R2	17,46916962	-78,19354248	-940,4160156	0,035957
Cloud_Mesh_Distance_BA_R0_R2	17,51920891	-78,26742554	-940,307312	0,040222
Cloud_Mesh_Distance_BA_R0_R2	17,57036209	-78,18028259	-940,3253174	0,046094
Cloud_Mesh_Distance_BA_R0_R2	17,59316254	-78,24537659	-940,2521362	0,044582
Cloud_Mesh_Distance_BA_R0_R2	22,08090973	-78,82711792	-940,401062	0,028644
Cloud_Mesh_Distance_BA_R0_R2	17,32770348	-78,31112671	-940,4802246	0,019437
Cloud_Mesh_Distance_BA_R0_R2	21,97475624	-78,76670837	-940,3300171	0,036249
Cloud_Mesh_Distance_BA_R0_R2	21,95278549	-78,64356995	-940,4299316	0,040782
Cloud_Mesh_Distance_BA_R0_R2	22,15414238	-78,77174377	-940,5629883	0,028571
Cloud_Mesh_Distance_BA_R0_R2	22,16255188	-78,84791565	-940,489624	0,023159
Cloud_Mesh_Distance_BA_R0_R2	22,06473923	-78,72969055	-940,4821167	0,031735
Cloud_Mesh_Distance_BA_R0_R2	17,59957886	-78,05279541	-940,4026489	0,047206
Cloud_Mesh_Distance_BA_R0_R2	21,891119	-78,47314453	-940,5615845	0,038786

Cloud_Mesh_Distance_BA_R0_R2	17,68353653	-77,91812134	-940,4428101	0,057811
Cloud_Mesh_Distance_BA_R0_R2	17,75789261	-77,97363281	-940,3322144	0,060841
Cloud_Mesh_Distance_BA_R0_R2	17,68691254	-77,81459045	-940,5327148	0,060964
Cloud_Mesh_Distance_BA_R0_R2	21,36877251	-78,14535522	-940,4066773	0,011184
Cloud_Mesh_Distance_BA_R0_R2	21,23497391	-78,08714294	-940,3598633	0,003095
Cloud_Mesh_Distance_BA_R0_R2	21,29782104	-78,18006897	-940,300354	0,017861
Cloud_Mesh_Distance_BA_R0_R2	21,66951752	-78,28593445	-940,5374146	0,028792
Cloud_Mesh_Distance_BA_R0_R2	21,76728058	-78,43701172	-940,4459229	0,042753
Cloud_Mesh_Distance_BA_R0_R2	17,93675804	-77,65097046	-940,4976807	0,073213
Cloud_Mesh_Distance_BA_R0_R2	20,95735168	-77,95726013	-940,291626	-0,013976
Cloud_Mesh_Distance_BA_R0_R2	21,14396477	-77,9624176	-940,4373779	-0,011448
Cloud_Mesh_Distance_BA_R0_R2	18,05044556	-77,60743713	-940,4667358	0,0757
Cloud_Mesh_Distance_BA_R0_R2	18,19632339	-77,41482544	-940,5856934	0,080044
Cloud_Mesh_Distance_BA_R0_R2	18,16191864	-77,54876709	-940,460083	0,078966
Cloud_Mesh_Distance_BA_R0_R2	18,36188507	-77,48822021	-940,4157105	0,083284
Cloud_Mesh_Distance_BA_R0_R2	18,78549957	-77,44412231	-940,2851563	0,078485
Cloud_Mesh_Distance_BA_R0_R2	20,52277374	-77,66070557	-940,296936	-0,009864
Cloud_Mesh_Distance_BA_R0_R2	20,70141792	-77,76725769	-940,3078613	-0,02066
Cloud_Mesh_Distance_BA_R0_R2	20,98063278	-77,84049988	-940,4447022	-0,023094
Cloud_Mesh_Distance_BA_R0_R2	21,10833168	-77,82185364	-940,592041	-0,023064
Cloud_Mesh_Distance_BA_R0_R2	18,54930496	-77,25926208	-940,5853272	0,090703
Cloud_Mesh_Distance_BA_R0_R2	18,36381721	-77,36981201	-940,5452271	0,084567
Cloud_Mesh_Distance_BA_R0_R2	18,53531075	-77,36019897	-940,4744873	0,087051
Cloud_Mesh_Distance_BA_R0_R2	18,73484802	-77,36973572	-940,3839722	0,083197
Cloud_Mesh_Distance_BA_R0_R2	18,889431	-77,27893066	-940,4370117	0,091751
Cloud_Mesh_Distance_BA_R0_R2	20,66054153	-77,51063538	-940,5778809	-0,010172
Cloud_Mesh_Distance_BA_R0_R2	20,62595367	-77,58816528	-940,4519653	-0,014113
Cloud_Mesh_Distance_BA_R0_R2	20,91215134	-77,67797852	-940,5893555	-0,024145
Cloud_Mesh_Distance_BA_R0_R2	20,83538818	-77,71868896	-940,4678955	-0,024756
Cloud_Mesh_Distance_BA_R0_R2	20,9705677	-77,75544739	-940,543396	-0,025926
Cloud_Mesh_Distance_BA_R0_R2	18,6148243	-77,18234253	-940,6518555	0,092119
Cloud_Mesh_Distance_BA_R0_R2	18,68915749	-77,26530457	-940,5216065	0,090909
Cloud_Mesh_Distance_BA_R0_R2	19,20686722	-77,14161682	-940,5435791	0,092151
Cloud_Mesh_Distance_BA_R0_R2	19,02781296	-77,27082825	-940,4106445	0,087866
Cloud_Mesh_Distance_BA_R0_R2	19,35396194	-77,14666748	-940,5267334	0,084016
Cloud_Mesh_Distance_BA_R0_R2	19,72642326	-77,17614746	-940,5228272	0,065246
Cloud_Mesh_Distance_BA_R0_R2	19,85213661	-77,2796936	-940,4124146	0,056571
Cloud_Mesh_Distance_BA_R0_R2	19,88547897	-77,19645691	-940,5394898	0,056323
Cloud_Mesh_Distance_BA_R0_R2	20,04082298	-77,2623291	-940,5058594	0,041079
Cloud_Mesh_Distance_BA_R0_R2	20,45862198	-77,48526001	-940,4537964	0,003781
Cloud_Mesh_Distance_BA_R0_R2	19,53479767	-77,15783691	-940,5184326	0,079196
Cloud_Mesh_Distance_BA_R0_R2	19,62114334	-77,08828735	-940,6365967	0,071053
Cloud_Mesh_Distance_BA_R0_R2	18,61138916	-83,55586243	-940,1350708	-0,094648
Cloud_Mesh_Distance_BA_R0_R2	18,8928833	-83,64231873	-940,0914307	-0,11425
Cloud_Mesh_Distance_BA_R0_R2	19,34835243	-83,6892395	-940,045105	-0,132169
Cloud_Mesh_Distance_BA_R0_R2	19,04786682	-83,64849854	-940,048584	-0,128187
Cloud_Mesh_Distance_BA_R0_R2	19,23399925	-83,59822083	-939,9628906	-0,138773
Cloud_Mesh_Distance_BA_R0_R2	19,43374252	-83,60380554	-939,9596558	-0,146669
Cloud_Mesh_Distance_BA_R0_R2	20,15015984	-83,60406494	-940,0987549	-0,142557
Cloud_Mesh_Distance_BA_R0_R2	18,54699898	-83,46159363	-940,0654907	-0,102928

Cloud_Mesh_Distance_BA_R0_R2	18,39788055	-83,37989807	-940,0825195	-0,104171
Cloud_Mesh_Distance_BA_R0_R2	18,46607971	-83,50773621	-940,1841431	-0,089894
Cloud_Mesh_Distance_BA_R0_R2	18,69359779	-83,49263	-940,0129395	-0,11853
Cloud_Mesh_Distance_BA_R0_R2	18,88882256	-83,54560852	-939,9863281	-0,12715
Cloud_Mesh_Distance_BA_R0_R2	18,98555756	-83,44696045	-939,8600464	-0,138468
Cloud_Mesh_Distance_BA_R0_R2	19,06489182	-83,54647827	-939,9382324	-0,138063
Cloud_Mesh_Distance_BA_R0_R2	19,37143517	-83,49632263	-939,8613892	-0,149344
Cloud_Mesh_Distance_BA_R0_R2	19,57318497	-83,62339783	-939,984436	-0,148579
Cloud_Mesh_Distance_BA_R0_R2	19,58984184	-83,5124054	-939,8884277	-0,151529
Cloud_Mesh_Distance_BA_R0_R2	19,73748779	-83,60224915	-939,9873047	-0,149579
Cloud_Mesh_Distance_BA_R0_R2	19,86654472	-83,62385559	-940,0323486	-0,14657
Cloud_Mesh_Distance_BA_R0_R2	20,11507416	-83,50941467	-940,0108032	-0,150665
Cloud_Mesh_Distance_BA_R0_R2	18,12923431	-83,07441711	-939,9932251	-0,130359
Cloud_Mesh_Distance_BA_R0_R2	18,22696686	-83,14280701	-939,9691162	-0,124765
Cloud_Mesh_Distance_BA_R0_R2	20,1609478	-83,39457703	-939,9406738	-0,158944
Cloud_Mesh_Distance_BA_R0_R2	20,2378273	-83,53617859	-940,0767822	-0,147908
Cloud_Mesh_Distance_BA_R0_R2	18,02311897	-83,00979614	-940,0392456	-0,130931
Cloud_Mesh_Distance_BA_R0_R2	20,2864399	-83,42076111	-940,0095215	-0,155109
Cloud_Mesh_Distance_BA_R0_R2	20,25190163	-83,3144989	-939,9179688	-0,165151
Cloud_Mesh_Distance_BA_R0_R2	20,60837936	-83,14241028	-939,960083	-0,158914
Cloud_Mesh_Distance_BA_R0_R2	20,42110443	-83,26876831	-939,9584961	-0,164669
Cloud_Mesh_Distance_BA_R0_R2	20,61016273	-83,31959534	-940,0849609	-0,156106
Cloud_Mesh_Distance_BA_R0_R2	20,90281677	-83,16249084	-940,1262817	-0,14482
Cloud_Mesh_Distance_BA_R0_R2	20,76950073	-83,15821838	-940,0518799	-0,151657
Cloud_Mesh_Distance_BA_R0_R2	17,98888779	-82,90892029	-939,9785156	-0,146351
Cloud_Mesh_Distance_BA_R0_R2	18,10090065	-82,9624176	-939,9157105	-0,142245
Cloud_Mesh_Distance_BA_R0_R2	18,0464592	-82,81925964	-939,8444214	-0,155971
Cloud_Mesh_Distance_BA_R0_R2	20,7630291	-83,01831055	-939,9519653	-0,151599
Cloud_Mesh_Distance_BA_R0_R2	21,03783417	-83,09835815	-940,1557007	-0,13491
Cloud_Mesh_Distance_BA_R0_R2	17,64468002	-82,56652832	-940,0662842	-0,148006
Cloud_Mesh_Distance_BA_R0_R2	17,7048645	-82,48959351	-939,9147949	-0,154242
Cloud_Mesh_Distance_BA_R0_R2	17,61997223	-82,43489075	-939,9693604	-0,149839
Cloud_Mesh_Distance_BA_R0_R2	17,79653168	-82,61170959	-939,9202881	-0,156503
Cloud_Mesh_Distance_BA_R0_R2	20,89266014	-82,88873291	-939,9302979	-0,13919
Cloud_Mesh_Distance_BA_R0_R2	21,08096886	-82,97842407	-940,0966797	-0,132716
Cloud_Mesh_Distance_BA_R0_R2	20,930233	-83,02519226	-940,0449829	-0,142553
Cloud_Mesh_Distance_BA_R0_R2	17,38187218	-82,19340515	-940,0789795	-0,12328
Cloud_Mesh_Distance_BA_R0_R2	17,45911789	-82,18603516	-939,9523315	-0,128255
Cloud_Mesh_Distance_BA_R0_R2	17,51797867	-82,43406677	-940,1136475	-0,138675
Cloud_Mesh_Distance_BA_R0_R2	21,05636215	-82,87110901	-940,0070801	-0,129884
Cloud_Mesh_Distance_BA_R0_R2	21,37555504	-82,62586975	-940,0376587	-0,106528
Cloud_Mesh_Distance_BA_R0_R2	21,19388199	-82,88285828	-940,0962524	-0,121973
Cloud_Mesh_Distance_BA_R0_R2	21,20622826	-82,72322083	-939,9936523	-0,112508
Cloud_Mesh_Distance_BA_R0_R2	21,46678162	-82,67326355	-940,1333618	-0,109498
Cloud_Mesh_Distance_BA_R0_R2	21,34635925	-82,78689575	-940,1273804	-0,113201
Cloud_Mesh_Distance_BA_R0_R2	17,3751812	-82,10420227	-940,0026245	-0,114652
Cloud_Mesh_Distance_BA_R0_R2	17,31130219	-81,98001099	-939,9938965	-0,102756
Cloud_Mesh_Distance_BA_R0_R2	17,44594002	-82,04562378	-939,8434448	-0,117833
Cloud_Mesh_Distance_BA_R0_R2	17,56084824	-82,31176758	-939,930542	-0,14384
Cloud_Mesh_Distance_BA_R0_R2	21,31086349	-82,44145203	-939,8806152	-0,090039

Cloud_Mesh_Distance_BA_R0_R2	21,280056	-82,56086731	-939,9324951	-0,101675
Cloud_Mesh_Distance_BA_R0_R2	21,5226593	-82,53926086	-940,0864868	-0,109368
Cloud_Mesh_Distance_BA_R0_R2	21,60343552	-82,645401	-940,2166748	-0,103295
Cloud_Mesh_Distance_BA_R0_R2	17,23067093	-82,00410461	-940,1601563	-0,104883
Cloud_Mesh_Distance_BA_R0_R2	17,19775009	-81,88905334	-940,1177979	-0,100717
Cloud_Mesh_Distance_BA_R0_R2	21,41843033	-82,47599792	-939,9714966	-0,096737
Cloud_Mesh_Distance_BA_R0_R2	21,65704727	-82,47824097	-940,1509399	-0,110211
Cloud_Mesh_Distance_BA_R0_R2	21,60146141	-82,33604431	-940,022583	-0,104232
Cloud_Mesh_Distance_BA_R0_R2	17,14909744	-81,71795654	-940,0712891	-0,090613
Cloud_Mesh_Distance_BA_R0_R2	17,2083149	-81,62646484	-939,9041748	-0,092062
Cloud_Mesh_Distance_BA_R0_R2	21,514431	-82,1696167	-939,8742676	-0,086141
Cloud_Mesh_Distance_BA_R0_R2	17,03206253	-81,27403259	-940,0424194	-0,078119
Cloud_Mesh_Distance_BA_R0_R2	17,14146996	-81,55384827	-939,9780273	-0,085576
Cloud_Mesh_Distance_BA_R0_R2	21,69976234	-82,1131134	-939,9810181	-0,102804
Cloud_Mesh_Distance_BA_R0_R2	21,75536728	-81,9697876	-939,9574585	-0,105988
Cloud_Mesh_Distance_BA_R0_R2	21,79990387	-82,19538879	-940,1013794	-0,11383
Cloud_Mesh_Distance_BA_R0_R2	21,8751297	-82,00364685	-940,0651856	-0,116999
Cloud_Mesh_Distance_BA_R0_R2	17,09158325	-81,18077087	-939,9014282	-0,077922
Cloud_Mesh_Distance_BA_R0_R2	21,83347321	-81,8782196	-939,9771729	-0,112089
Cloud_Mesh_Distance_BA_R0_R2	22,07398224	-81,71151733	-940,088501	-0,129243
Cloud_Mesh_Distance_BA_R0_R2	22,19770432	-81,67132568	-940,1703491	-0,133953
Cloud_Mesh_Distance_BA_R0_R2	17,03388596	-81,13438416	-939,9873657	-0,075621
Cloud_Mesh_Distance_BA_R0_R2	17,07025909	-81,04812622	-939,9004517	-0,074229
Cloud_Mesh_Distance_BA_R0_R2	17,00855637	-81,00349426	-939,996521	-0,070586
Cloud_Mesh_Distance_BA_R0_R2	16,92502213	-80,99267578	-940,1567993	-0,067735
Cloud_Mesh_Distance_BA_R0_R2	16,90644836	-80,82070923	-940,1520386	-0,059993
Cloud_Mesh_Distance_BA_R0_R2	16,98852158	-80,78274536	-939,9901123	-0,061476
Cloud_Mesh_Distance_BA_R0_R2	22,13725281	-81,55433655	-940,0703735	-0,131475
Cloud_Mesh_Distance_BA_R0_R2	22,07805252	-81,35580444	-939,9567261	-0,131982
Cloud_Mesh_Distance_BA_R0_R2	22,25935364	-81,54724121	-940,1633301	-0,134568
Cloud_Mesh_Distance_BA_R0_R2	16,91319847	-80,66311646	-940,1144409	-0,051652
Cloud_Mesh_Distance_BA_R0_R2	22,15886307	-81,38745117	-940,0228882	-0,137093
Cloud_Mesh_Distance_BA_R0_R2	22,2716732	-81,43013	-940,1224976	-0,135327
Cloud_Mesh_Distance_BA_R0_R2	16,9362793	-80,30543518	-940,0625	-0,029044
Cloud_Mesh_Distance_BA_R0_R2	16,99396133	-80,42245483	-939,9594727	-0,040926
Cloud_Mesh_Distance_BA_R0_R2	16,9725914	-80,58337402	-939,9981079	-0,050316
Cloud_Mesh_Distance_BA_R0_R2	16,92092514	-80,48287964	-940,0866089	-0,040534
Cloud_Mesh_Distance_BA_R0_R2	22,30599594	-81,2552948	-940,0802002	-0,13905
Cloud_Mesh_Distance_BA_R0_R2	22,33588028	-80,98519897	-940,0146484	-0,129049
Cloud_Mesh_Distance_BA_R0_R2	22,26147461	-81,11322021	-940,001709	-0,134273
Cloud_Mesh_Distance_BA_R0_R2	22,41470146	-81,06724548	-940,0974731	-0,135661
Cloud_Mesh_Distance_BA_R0_R2	22,46333122	-81,18429565	-940,1775513	-0,137854
Cloud_Mesh_Distance_BA_R0_R2	16,94998741	-80,12068176	-940,0567017	-0,015711
Cloud_Mesh_Distance_BA_R0_R2	22,45028877	-80,90232849	-940,0757446	-0,126521
Cloud_Mesh_Distance_BA_R0_R2	22,50621414	-81,07447815	-940,1742554	-0,139915
Cloud_Mesh_Distance_BA_R0_R2	22,6516571	-80,79051208	-940,2208252	-0,13568
Cloud_Mesh_Distance_BA_R0_R2	16,87160873	-80,02098083	-940,2075195	-0,003419
Cloud_Mesh_Distance_BA_R0_R2	17,01411438	-80,23612976	-939,9385986	-0,031895
Cloud_Mesh_Distance_BA_R0_R2	22,59286499	-80,67002869	-940,1419678	-0,114408
Cloud_Mesh_Distance_BA_R0_R2	22,54959488	-80,81227112	-940,1323242	-0,12414

Cloud_Mesh_Distance_BA_R0_R2	22,5055027	-80,57106018	-940,053772	-0,097681
Cloud_Mesh_Distance_BA_R0_R2	22,70897293	-80,61222839	-940,24646	-0,12282
Cloud_Mesh_Distance_BA_R0_R2	16,96883774	-79,92152405	-940,0632935	-0,004421
Cloud_Mesh_Distance_BA_R0_R2	22,75518036	-80,40763855	-940,289856	-0,105879
Cloud_Mesh_Distance_BA_R0_R2	16,98879623	-79,59806824	-940,1304321	0,016343
Cloud_Mesh_Distance_BA_R0_R2	16,99549866	-79,75613403	-940,0657349	0,005834
Cloud_Mesh_Distance_BA_R0_R2	22,63161087	-80,48602295	-940,157898	-0,105617
Cloud_Mesh_Distance_BA_R0_R2	22,65035439	-80,27587891	-940,1828613	-0,086887
Cloud_Mesh_Distance_BA_R0_R2	16,97197914	-79,41075134	-940,2371216	0,022619
Cloud_Mesh_Distance_BA_R0_R2	17,03930283	-79,46330261	-940,112793	0,018842
Cloud_Mesh_Distance_BA_R0_R2	22,74663734	-80,18630981	-940,3084717	-0,084481
Cloud_Mesh_Distance_BA_R0_R2	17,01426506	-79,23100281	-940,2678223	0,022521
Cloud_Mesh_Distance_BA_R0_R2	17,06201172	-79,32643127	-940,147522	0,023026
Cloud_Mesh_Distance_BA_R0_R2	22,54535484	-79,86395264	-940,1778565	-0,04702
Cloud_Mesh_Distance_BA_R0_R2	22,53417397	-79,99836731	-940,1171265	-0,049785
Cloud_Mesh_Distance_BA_R0_R2	22,64294815	-80,03369141	-940,2268677	-0,064398
Cloud_Mesh_Distance_BA_R0_R2	22,73511887	-80,01777649	-940,3474121	-0,073336
Cloud_Mesh_Distance_BA_R0_R2	17,08422089	-79,19082642	-940,1922607	0,01976
Cloud_Mesh_Distance_BA_R0_R2	17,13366127	-79,05136108	-940,2097778	0,019417
Cloud_Mesh_Distance_BA_R0_R2	22,38391876	-79,60215759	-940,1362305	-0,02318
Cloud_Mesh_Distance_BA_R0_R2	17,33859253	-78,91833496	-940,0422974	0,017792
Cloud_Mesh_Distance_BA_R0_R2	17,22743607	-78,85784912	-940,2124023	0,018223
Cloud_Mesh_Distance_BA_R0_R2	22,26745987	-79,41468811	-940,1329956	-0,005561
Cloud_Mesh_Distance_BA_R0_R2	22,30283928	-79,50636292	-940,1108398	-0,01118
Cloud_Mesh_Distance_BA_R0_R2	22,49588966	-79,63383484	-940,243042	-0,033016
Cloud_Mesh_Distance_BA_R0_R2	22,51310349	-79,52336121	-940,3411865	-0,024583
Cloud_Mesh_Distance_BA_R0_R2	22,58247566	-79,57383728	-940,3980103	-0,031521
Cloud_Mesh_Distance_BA_R0_R2	17,34954453	-78,74824524	-940,1391602	0,022852
Cloud_Mesh_Distance_BA_R0_R2	22,21998787	-79,35704041	-940,1240845	-0,001495
Cloud_Mesh_Distance_BA_R0_R2	22,36482048	-79,45588684	-940,2097168	-0,01855
Cloud_Mesh_Distance_BA_R0_R2	22,33647156	-79,30891418	-940,2862549	-0,008014
Cloud_Mesh_Distance_BA_R0_R2	22,39442825	-79,3916626	-940,2922363	-0,014317
Cloud_Mesh_Distance_BA_R0_R2	17,28270149	-78,64456177	-940,2909546	0,01869
Cloud_Mesh_Distance_BA_R0_R2	17,39551353	-78,58766174	-940,1992798	0,024505
Cloud_Mesh_Distance_BA_R0_R2	17,46063995	-78,67390442	-940,0710449	0,025734
Cloud_Mesh_Distance_BA_R0_R2	22,05831528	-78,88783264	-940,3165283	0,024641
Cloud_Mesh_Distance_BA_R0_R2	21,97243309	-78,93721008	-940,1819458	0,029577
Cloud_Mesh_Distance_BA_R0_R2	22,26425552	-79,3270874	-940,1905518	-0,006271
Cloud_Mesh_Distance_BA_R0_R2	22,22365189	-79,17106628	-940,2655029	0,005889
Cloud_Mesh_Distance_BA_R0_R2	17,4434185	-78,45953369	-940,239563	0,030959
Cloud_Mesh_Distance_BA_R0_R2	17,54064369	-78,36225891	-940,2122192	0,03684
Cloud_Mesh_Distance_BA_R0_R2	21,86613464	-78,76689148	-940,2130737	0,042937
Cloud_Mesh_Distance_BA_R0_R2	21,74715996	-78,706604	-940,1491699	0,052029
Cloud_Mesh_Distance_BA_R0_R2	21,81605911	-78,83718872	-940,1105957	0,042161
Cloud_Mesh_Distance_BA_R0_R2	17,77400589	-78,1782074	-940,1469116	0,053957
Cloud_Mesh_Distance_BA_R0_R2	17,68633652	-78,13496399	-940,2562256	0,051165
Cloud_Mesh_Distance_BA_R0_R2	17,66739655	-78,27163696	-940,1644898	0,047434
Cloud_Mesh_Distance_BA_R0_R2	17,86918449	-78,04014587	-940,1869507	0,06055
Cloud_Mesh_Distance_BA_R0_R2	21,52033806	-78,40725708	-940,227356	0,04724
Cloud_Mesh_Distance_BA_R0_R2	21,47624969	-78,45599365	-940,1453857	0,05252

Cloud_Mesh_Distance_BA_R0_R2	21,82004547	-78,61128235	-940,3071899	0,050154
Cloud_Mesh_Distance_BA_R0_R2	21,66116714	-78,48124695	-940,2776489	0,051229
Cloud_Mesh_Distance_BA_R0_R2	21,88925552	-78,54614258	-940,4613648	0,044353
Cloud_Mesh_Distance_BA_R0_R2	17,92077637	-77,8719635	-940,2973633	0,067926
Cloud_Mesh_Distance_BA_R0_R2	17,80330658	-77,82920837	-940,4248657	0,065883
Cloud_Mesh_Distance_BA_R0_R2	18,08091736	-77,90457153	-940,1605835	0,067414
Cloud_Mesh_Distance_BA_R0_R2	18,41413498	-77,81965637	-940,0582886	0,058102
Cloud_Mesh_Distance_BA_R0_R2	18,47486305	-77,70776367	-940,1380005	0,066628
Cloud_Mesh_Distance_BA_R0_R2	21,17525864	-78,14561462	-940,2457275	0,007315
Cloud_Mesh_Distance_BA_R0_R2	21,37948608	-78,38339233	-940,1455689	0,040769
Cloud_Mesh_Distance_BA_R0_R2	21,4255352	-78,27171326	-940,2983398	0,03275
Cloud_Mesh_Distance_BA_R0_R2	21,26790619	-78,25474548	-940,1965332	0,022314
Cloud_Mesh_Distance_BA_R0_R2	21,59692001	-78,3447113	-940,37146	0,042741
Cloud_Mesh_Distance_BA_R0_R2	17,93870926	-77,75056458	-940,3987427	0,071624
Cloud_Mesh_Distance_BA_R0_R2	18,08512878	-77,7454071	-940,3061523	0,075015
Cloud_Mesh_Distance_BA_R0_R2	18,02607727	-77,67883301	-940,4098511	0,07539
Cloud_Mesh_Distance_BA_R0_R2	18,28120804	-77,75553894	-940,1846924	0,073024
Cloud_Mesh_Distance_BA_R0_R2	18,55470085	-77,76333618	-940,0523682	0,058924
Cloud_Mesh_Distance_BA_R0_R2	18,56474304	-77,62109375	-940,1854858	0,070643
Cloud_Mesh_Distance_BA_R0_R2	18,42313957	-77,61456299	-940,2532959	0,07345
Cloud_Mesh_Distance_BA_R0_R2	18,69976234	-77,60668945	-940,149353	0,064928
Cloud_Mesh_Distance_BA_R0_R2	20,80771446	-77,88566589	-940,2601929	-0,019786
Cloud_Mesh_Distance_BA_R0_R2	20,85733986	-77,83317566	-940,3522949	-0,023502
Cloud_Mesh_Distance_BA_R0_R2	21,11003494	-78,048172	-940,3052368	-0,005575
Cloud_Mesh_Distance_BA_R0_R2	18,11336136	-77,64167786	-940,3919678	0,075693
Cloud_Mesh_Distance_BA_R0_R2	18,23808479	-77,6302948	-940,3319092	0,076792
Cloud_Mesh_Distance_BA_R0_R2	18,61421585	-77,49954224	-940,2895508	0,079798
Cloud_Mesh_Distance_BA_R0_R2	20,26387787	-77,57646179	-940,2341309	0,007483
Cloud_Mesh_Distance_BA_R0_R2	20,38820457	-77,57872009	-940,2997437	0,001043
Cloud_Mesh_Distance_BA_R0_R2	20,4782753	-77,56228638	-940,3753052	-0,003585
Cloud_Mesh_Distance_BA_R0_R2	20,37942123	-77,69285583	-940,180542	-0,009324
Cloud_Mesh_Distance_BA_R0_R2	20,69578171	-77,90856934	-940,1658325	-0,018891
Cloud_Mesh_Distance_BA_R0_R2	18,88570976	-77,38490295	-940,3178711	0,083511
Cloud_Mesh_Distance_BA_R0_R2	18,88017273	-77,45507813	-940,244812	0,077452
Cloud_Mesh_Distance_BA_R0_R2	19,01934052	-77,33908081	-940,3338013	0,082576
Cloud_Mesh_Distance_BA_R0_R2	19,16599846	-77,39700317	-940,2423706	0,075205
Cloud_Mesh_Distance_BA_R0_R2	19,58810043	-77,44035339	-940,1785278	0,055695
Cloud_Mesh_Distance_BA_R0_R2	19,38218689	-77,4175415	-940,1994629	0,066216
Cloud_Mesh_Distance_BA_R0_R2	19,78679276	-77,47369385	-940,1713257	0,042037
Cloud_Mesh_Distance_BA_R0_R2	19,91662598	-77,40386963	-940,2789307	0,04444
Cloud_Mesh_Distance_BA_R0_R2	19,71823883	-77,35675049	-940,2865601	0,056961
Cloud_Mesh_Distance_BA_R0_R2	20,11956787	-77,46951294	-940,2798462	0,024392
Cloud_Mesh_Distance_BA_R0_R2	19,97545052	-77,52742004	-940,1657715	0,028623
Cloud_Mesh_Distance_BA_R0_R2	20,39129829	-77,50999451	-940,3787842	0,005529
Cloud_Mesh_Distance_BA_R0_R2	19,14151764	-77,28326416	-940,3739624	0,086223
Cloud_Mesh_Distance_BA_R0_R2	19,3181839	-77,30758667	-940,3233032	0,079923
Cloud_Mesh_Distance_BA_R0_R2	19,43649101	-77,2303009	-940,4143677	0,080463
Cloud_Mesh_Distance_BA_R0_R2	19,25323677	-77,21324158	-940,4448242	0,089251
Cloud_Mesh_Distance_BA_R0_R2	19,63253784	-77,24990845	-940,4036865	0,071966
Cloud_Mesh_Distance_BA_R0_R2	19,51524734	-77,32875061	-940,2966309	0,070411

Cloud_Mesh_Distance_BA_R0_R2	20,0283165	-77,35359192	-940,3782349	0,039292
Cloud_Mesh_Distance_BA_R0_R2	20,20001984	-77,37818909	-940,4286499	0,026766
Cloud_Mesh_Distance_BA_R0_R2	20,34091187	-77,44444275	-940,4262085	0,011783
Cloud_Mesh_Distance_BA_R0_R2	20,28581238	-77,48936462	-940,3404541	0,011358
Cloud_Mesh_Distance_BA_R0_R2	18,42224312	-83,27102661	-939,9432983	-0,122803
Cloud_Mesh_Distance_BA_R0_R2	18,28553009	-83,2464447	-940,0255737	-0,113432
Cloud_Mesh_Distance_BA_R0_R2	18,57890701	-83,35972595	-939,9349976	-0,120262
Cloud_Mesh_Distance_BA_R0_R2	18,51727867	-83,19604492	-939,8153687	-0,130309
Cloud_Mesh_Distance_BA_R0_R2	18,71064377	-83,27253723	-939,789978	-0,137001
Cloud_Mesh_Distance_BA_R0_R2	18,79099083	-83,41140747	-939,8862305	-0,124697
Cloud_Mesh_Distance_BA_R0_R2	19,1742878	-83,47434998	-939,8530884	-0,144819
Cloud_Mesh_Distance_BA_R0_R2	18,90565109	-83,3213501	-939,7684937	-0,143382
Cloud_Mesh_Distance_BA_R0_R2	19,09990311	-83,35536194	-939,7583008	-0,145164
Cloud_Mesh_Distance_BA_R0_R2	19,9232235	-83,52835083	-939,9664307	-0,1514
Cloud_Mesh_Distance_BA_R0_R2	19,779953	-83,48284912	-939,8952026	-0,154099
Cloud_Mesh_Distance_BA_R0_R2	18,34716415	-83,16253662	-939,8922119	-0,131107
Cloud_Mesh_Distance_BA_R0_R2	18,52683258	-83,08010864	-939,7165527	-0,147802
Cloud_Mesh_Distance_BA_R0_R2	18,65517426	-83,13316345	-939,7006226	-0,145547
Cloud_Mesh_Distance_BA_R0_R2	18,83406067	-83,18392944	-939,6781616	-0,143536
Cloud_Mesh_Distance_BA_R0_R2	19,29660225	-83,3768158	-939,7615967	-0,153616
Cloud_Mesh_Distance_BA_R0_R2	19,50250435	-83,3837738	-939,7741089	-0,153926
Cloud_Mesh_Distance_BA_R0_R2	19,72867393	-83,36299133	-939,7911987	-0,158792
Cloud_Mesh_Distance_BA_R0_R2	19,61072922	-83,25900269	-939,6925049	-0,151633
Cloud_Mesh_Distance_BA_R0_R2	19,76766205	-83,23454285	-939,7045288	-0,153507
Cloud_Mesh_Distance_BA_R0_R2	19,91813278	-83,39369202	-939,8603516	-0,16162
Cloud_Mesh_Distance_BA_R0_R2	20,04470444	-83,42628479	-939,9229736	-0,157226
Cloud_Mesh_Distance_BA_R0_R2	19,92333984	-83,24597168	-939,75354	-0,160632
Cloud_Mesh_Distance_BA_R0_R2	18,2420311	-83,04008484	-939,8619385	-0,135455
Cloud_Mesh_Distance_BA_R0_R2	18,40156937	-83,06822205	-939,7752686	-0,14044
Cloud_Mesh_Distance_BA_R0_R2	19,96183395	-83,12257385	-939,6795654	-0,156887
Cloud_Mesh_Distance_BA_R0_R2	20,10173035	-83,30018616	-939,8499756	-0,166257
Cloud_Mesh_Distance_BA_R0_R2	20,28179932	-83,18778992	-939,8422852	-0,165183
Cloud_Mesh_Distance_BA_R0_R2	17,93301201	-82,69094849	-939,8453369	-0,16356
Cloud_Mesh_Distance_BA_R0_R2	18,21389008	-82,7857666	-939,6894531	-0,165019
Cloud_Mesh_Distance_BA_R0_R2	18,31592178	-82,94189453	-939,7328491	-0,15283
Cloud_Mesh_Distance_BA_R0_R2	18,18322372	-82,91157532	-939,8032227	-0,154846
Cloud_Mesh_Distance_BA_R0_R2	18,39004517	-82,85630798	-939,6323853	-0,158726
Cloud_Mesh_Distance_BA_R0_R2	18,46780396	-82,97470093	-939,6712647	-0,152932
Cloud_Mesh_Distance_BA_R0_R2	20,32358551	-83,01622009	-939,744751	-0,155193
Cloud_Mesh_Distance_BA_R0_R2	20,11683655	-83,12882996	-939,7367554	-0,16266
Cloud_Mesh_Distance_BA_R0_R2	20,46177673	-83,09492493	-939,8579712	-0,161566
Cloud_Mesh_Distance_BA_R0_R2	17,68148994	-82,38563538	-939,8480225	-0,150125
Cloud_Mesh_Distance_BA_R0_R2	17,78937531	-82,46931458	-939,8033447	-0,16048
Cloud_Mesh_Distance_BA_R0_R2	20,26545334	-82,88298035	-939,6361694	-0,13663
Cloud_Mesh_Distance_BA_R0_R2	20,50218582	-82,96080017	-939,7872925	-0,150505
Cloud_Mesh_Distance_BA_R0_R2	20,44894028	-82,85704041	-939,6968994	-0,139917
Cloud_Mesh_Distance_BA_R0_R2	20,64354897	-82,84353638	-939,7763062	-0,139454
Cloud_Mesh_Distance_BA_R0_R2	20,75133133	-82,90484619	-939,8682251	-0,143789
Cloud_Mesh_Distance_BA_R0_R2	20,62282562	-82,99342346	-939,8651733	-0,155351
Cloud_Mesh_Distance_BA_R0_R2	20,89800262	-82,6524353	-939,7770386	-0,109223

Cloud_Mesh_Distance_BA_R0_R2	20,7495575	-82,71261597	-939,7428589	-0,11816
Cloud_Mesh_Distance_BA_R0_R2	20,94024467	-82,51974487	-939,7193604	-0,088584
Cloud_Mesh_Distance_BA_R0_R2	20,8279705	-82,78736877	-939,8288574	-0,130809
Cloud_Mesh_Distance_BA_R0_R2	21,0154705	-82,72976685	-939,8874512	-0,117674
Cloud_Mesh_Distance_BA_R0_R2	21,15276527	-82,61384583	-939,8892822	-0,105517
Cloud_Mesh_Distance_BA_R0_R2	17,3826046	-81,89720154	-939,8134155	-0,10795
Cloud_Mesh_Distance_BA_R0_R2	17,47464561	-81,93913269	-939,7191162	-0,106632
Cloud_Mesh_Distance_BA_R0_R2	17,65773392	-82,28182983	-939,7833252	-0,146633
Cloud_Mesh_Distance_BA_R0_R2	17,72351646	-82,2008667	-939,6467896	-0,137431
Cloud_Mesh_Distance_BA_R0_R2	17,56807137	-82,17601013	-939,7973633	-0,135984
Cloud_Mesh_Distance_BA_R0_R2	21,05224419	-82,57441711	-939,8085938	-0,097606
Cloud_Mesh_Distance_BA_R0_R2	17,27713394	-81,7808075	-939,8919678	-0,095561
Cloud_Mesh_Distance_BA_R0_R2	17,36690331	-81,79225159	-939,7615967	-0,096362
Cloud_Mesh_Distance_BA_R0_R2	17,43920326	-81,83387756	-939,6904907	-0,092295
Cloud_Mesh_Distance_BA_R0_R2	21,1921978	-82,49006653	-939,8364868	-0,087308
Cloud_Mesh_Distance_BA_R0_R2	21,08680344	-82,43281555	-939,7462769	-0,079068
Cloud_Mesh_Distance_BA_R0_R2	21,23863983	-82,32527161	-939,7747192	-0,074046
Cloud_Mesh_Distance_BA_R0_R2	21,40468597	-82,33508301	-939,8826294	-0,088985
Cloud_Mesh_Distance_BA_R0_R2	17,2022419	-81,48497009	-939,8394165	-0,088162
Cloud_Mesh_Distance_BA_R0_R2	21,41529846	-82,1293335	-939,7913818	-0,075113
Cloud_Mesh_Distance_BA_R0_R2	17,11314011	-81,3805542	-939,9404297	-0,081453
Cloud_Mesh_Distance_BA_R0_R2	21,72397995	-81,85325623	-939,8900147	-0,105202
Cloud_Mesh_Distance_BA_R0_R2	21,62556839	-81,9942627	-939,8760376	-0,092946
Cloud_Mesh_Distance_BA_R0_R2	21,56141663	-81,81951904	-939,7739258	-0,091306
Cloud_Mesh_Distance_BA_R0_R2	21,47869492	-82,01768494	-939,7880249	-0,078857
Cloud_Mesh_Distance_BA_R0_R2	17,20035553	-81,34947205	-939,7862549	-0,08683
Cloud_Mesh_Distance_BA_R0_R2	21,95576859	-81,8175354	-940,0426636	-0,122634
Cloud_Mesh_Distance_BA_R0_R2	21,84305954	-81,74523926	-939,9320068	-0,113583
Cloud_Mesh_Distance_BA_R0_R2	17,14811897	-81,02464294	-939,7733154	-0,079537
Cloud_Mesh_Distance_BA_R0_R2	21,71803665	-81,54525757	-939,7890625	-0,109004
Cloud_Mesh_Distance_BA_R0_R2	21,71389771	-81,69081116	-939,8288574	-0,106251
Cloud_Mesh_Distance_BA_R0_R2	22,01405334	-81,46730042	-939,9511719	-0,129514
Cloud_Mesh_Distance_BA_R0_R2	21,97886848	-81,64656067	-939,991394	-0,123507
Cloud_Mesh_Distance_BA_R0_R2	21,85035896	-81,56813049	-939,8770142	-0,119965
Cloud_Mesh_Distance_BA_R0_R2	17,07543945	-80,89733887	-939,8608398	-0,073422
Cloud_Mesh_Distance_BA_R0_R2	17,17270851	-80,79190063	-939,706665	-0,072238
Cloud_Mesh_Distance_BA_R0_R2	21,78791428	-81,4294281	-939,7987061	-0,114112
Cloud_Mesh_Distance_BA_R0_R2	17,08229256	-80,73472595	-939,8294067	-0,064069
Cloud_Mesh_Distance_BA_R0_R2	17,1317749	-80,6341095	-939,751709	-0,060744
Cloud_Mesh_Distance_BA_R0_R2	17,05321503	-80,5705719	-939,8655396	-0,057481
Cloud_Mesh_Distance_BA_R0_R2	21,97278976	-81,10321045	-939,8200684	-0,119755
Cloud_Mesh_Distance_BA_R0_R2	21,94856834	-81,20321655	-939,8314819	-0,12126
Cloud_Mesh_Distance_BA_R0_R2	22,14719009	-81,24485779	-939,9661255	-0,134412
Cloud_Mesh_Distance_BA_R0_R2	22,16570663	-81,12605286	-939,9423218	-0,130619
Cloud_Mesh_Distance_BA_R0_R2	22,0006752	-81,30964661	-939,8928223	-0,129474
Cloud_Mesh_Distance_BA_R0_R2	22,06611061	-81,14675903	-939,8858643	-0,126834
Cloud_Mesh_Distance_BA_R0_R2	22,23675537	-80,79051208	-939,9022827	-0,107589
Cloud_Mesh_Distance_BA_R0_R2	22,19519424	-80,99209595	-939,9239502	-0,122813
Cloud_Mesh_Distance_BA_R0_R2	17,1367054	-79,97866821	-939,81073	-0,02092
Cloud_Mesh_Distance_BA_R0_R2	17,03710365	-80,04125977	-939,9330444	-0,017062

Cloud_Mesh_Distance_BA_R0_R2	22,13315392	-80,71434021	-939,8255615	-0,101584
Cloud_Mesh_Distance_BA_R0_R2	22,26589203	-80,61949158	-939,8899536	-0,091051
Cloud_Mesh_Distance_BA_R0_R2	22,34758377	-80,84895325	-939,9873657	-0,118754
Cloud_Mesh_Distance_BA_R0_R2	22,47483826	-80,74650574	-940,0570068	-0,114651
Cloud_Mesh_Distance_BA_R0_R2	17,17023087	-79,80827332	-939,8153076	-0,014049
Cloud_Mesh_Distance_BA_R0_R2	17,06866455	-79,82626343	-939,9389038	-0,002885
Cloud_Mesh_Distance_BA_R0_R2	22,26456451	-80,48973083	-939,8727417	-0,079746
Cloud_Mesh_Distance_BA_R0_R2	22,29779434	-80,34248352	-939,8845825	-0,067479
Cloud_Mesh_Distance_BA_R0_R2	22,53211784	-80,37057495	-940,06427	-0,081186
Cloud_Mesh_Distance_BA_R0_R2	22,37438011	-80,67536926	-939,9702148	-0,101982
Cloud_Mesh_Distance_BA_R0_R2	22,38760376	-80,47454834	-939,954895	-0,086638
Cloud_Mesh_Distance_BA_R0_R2	17,051754	-79,66949463	-940,0125732	0,009056
Cloud_Mesh_Distance_BA_R0_R2	17,06656265	-79,55523682	-940,0357666	0,014014
Cloud_Mesh_Distance_BA_R0_R2	17,11278343	-79,43321228	-940,027832	0,013699
Cloud_Mesh_Distance_BA_R0_R2	22,42596054	-80,00566101	-940,0140381	-0,045822
Cloud_Mesh_Distance_BA_R0_R2	22,4251461	-80,25003052	-939,9788818	-0,063145
Cloud_Mesh_Distance_BA_R0_R2	22,54330444	-80,15765381	-940,0913696	-0,064544
Cloud_Mesh_Distance_BA_R0_R2	17,15955162	-79,23979187	-940,065979	0,016182
Cloud_Mesh_Distance_BA_R0_R2	22,30714798	-79,81297302	-939,9668579	-0,026873
Cloud_Mesh_Distance_BA_R0_R2	22,48777008	-79,91590881	-940,0979614	-0,044967
Cloud_Mesh_Distance_BA_R0_R2	22,42927742	-79,78158569	-940,0905151	-0,032671
Cloud_Mesh_Distance_BA_R0_R2	17,32375526	-79,10028076	-939,9541626	0,011035
Cloud_Mesh_Distance_BA_R0_R2	17,25120544	-79,05935669	-940,0571289	0,018741
Cloud_Mesh_Distance_BA_R0_R2	17,32216644	-79,02705383	-939,9962769	0,014648
Cloud_Mesh_Distance_BA_R0_R2	22,12637901	-79,68153381	-939,8745728	-0,006538
Cloud_Mesh_Distance_BA_R0_R2	22,30669022	-79,62895203	-940,0452271	-0,016504
Cloud_Mesh_Distance_BA_R0_R2	17,44818115	-78,90356445	-939,9414063	0,015007
Cloud_Mesh_Distance_BA_R0_R2	22,08130264	-79,52589417	-939,9076538	0,00158
Cloud_Mesh_Distance_BA_R0_R2	22,20644951	-79,6408844	-939,9519653	-0,009166
Cloud_Mesh_Distance_BA_R0_R2	17,41780281	-78,80473328	-940,0296631	0,020661
Cloud_Mesh_Distance_BA_R0_R2	17,51397514	-78,77134705	-939,9595947	0,017197
Cloud_Mesh_Distance_BA_R0_R2	17,63038063	-78,75865173	-939,8696899	0,011194
Cloud_Mesh_Distance_BA_R0_R2	22,18000793	-79,42893982	-940,0404663	-0,000906
Cloud_Mesh_Distance_BA_R0_R2	22,0091629	-79,18251038	-940,043457	0,017124
Cloud_Mesh_Distance_BA_R0_R2	17,51023483	-78,51887512	-940,1281128	0,032199
Cloud_Mesh_Distance_BA_R0_R2	17,60785484	-78,60510254	-939,9803467	0,028022
Cloud_Mesh_Distance_BA_R0_R2	17,75965691	-78,63874817	-939,8406982	0,014604
Cloud_Mesh_Distance_BA_R0_R2	17,64433289	-78,41668701	-940,076416	0,037463
Cloud_Mesh_Distance_BA_R0_R2	21,59235382	-78,89472961	-939,9082031	0,044199
Cloud_Mesh_Distance_BA_R0_R2	21,74193001	-79,07807922	-939,9013672	0,032325
Cloud_Mesh_Distance_BA_R0_R2	21,68826485	-78,95205688	-939,9376221	0,038651
Cloud_Mesh_Distance_BA_R0_R2	21,86235619	-79,14579773	-939,9480591	0,024542
Cloud_Mesh_Distance_BA_R0_R2	21,8235817	-78,98733521	-940,0151367	0,033874
Cloud_Mesh_Distance_BA_R0_R2	22,09926224	-79,10803223	-940,1806641	0,012991
Cloud_Mesh_Distance_BA_R0_R2	22,16735458	-79,25996399	-940,1375732	0,002166
Cloud_Mesh_Distance_BA_R0_R2	17,91790771	-78,41098022	-939,871521	0,03315
Cloud_Mesh_Distance_BA_R0_R2	17,91402245	-78,31222534	-939,9415283	0,045684
Cloud_Mesh_Distance_BA_R0_R2	17,78193665	-78,45411682	-939,9398193	0,030818
Cloud_Mesh_Distance_BA_R0_R2	17,79293251	-78,28460693	-940,0496216	0,047714
Cloud_Mesh_Distance_BA_R0_R2	21,54844856	-78,80296326	-939,9364014	0,050385

Cloud_Mesh_Distance_BA_R0_R2	21,49122429	-78,87106323	-939,8640137	0,043001
Cloud_Mesh_Distance_BA_R0_R2	21,54172516	-78,70443726	-939,9943848	0,055734
Cloud_Mesh_Distance_BA_R0_R2	17,87332344	-78,15608215	-940,088562	0,054067
Cloud_Mesh_Distance_BA_R0_R2	17,9294014	-78,20349121	-940,0104981	0,053153
Cloud_Mesh_Distance_BA_R0_R2	20,94374657	-78,35678101	-939,9399414	0,00102
Cloud_Mesh_Distance_BA_R0_R2	21,12932205	-78,42520142	-939,9729004	0,022331
Cloud_Mesh_Distance_BA_R0_R2	21,62176514	-78,65263367	-940,0880737	0,054383
Cloud_Mesh_Distance_BA_R0_R2	21,67254257	-78,8122406	-940,0128174	0,049753
Cloud_Mesh_Distance_BA_R0_R2	18,15237045	-77,97743225	-940,0525513	0,061629
Cloud_Mesh_Distance_BA_R0_R2	18,01716232	-78,07377625	-940,0542603	0,059262
Cloud_Mesh_Distance_BA_R0_R2	18,06627846	-78,19429016	-939,9291992	0,046409
Cloud_Mesh_Distance_BA_R0_R2	18,16755104	-78,05589294	-939,9771118	0,055985
Cloud_Mesh_Distance_BA_R0_R2	20,50090981	-78,12226868	-939,9096069	-0,033663
Cloud_Mesh_Distance_BA_R0_R2	20,66574287	-78,27737427	-939,8803711	-0,030521
Cloud_Mesh_Distance_BA_R0_R2	20,78497314	-78,33375549	-939,892334	-0,019248
Cloud_Mesh_Distance_BA_R0_R2	20,79064369	-78,19625854	-939,9863281	-0,014293
Cloud_Mesh_Distance_BA_R0_R2	20,96619606	-78,24514771	-940,0310059	0,001494
Cloud_Mesh_Distance_BA_R0_R2	21,07184219	-78,31619263	-940,027771	0,012981
Cloud_Mesh_Distance_BA_R0_R2	21,43313026	-78,51449585	-940,06604	0,051333
Cloud_Mesh_Distance_BA_R0_R2	21,28965759	-78,46087646	-940,0252075	0,039385
Cloud_Mesh_Distance_BA_R0_R2	21,20974731	-78,34107971	-940,0796509	0,02853
Cloud_Mesh_Distance_BA_R0_R2	21,56165886	-78,52561951	-940,1464233	0,05687
Cloud_Mesh_Distance_BA_R0_R2	21,68327522	-78,59103394	-940,1901856	0,056457
Cloud_Mesh_Distance_BA_R0_R2	18,24369621	-77,97238159	-940,0057983	0,060569
Cloud_Mesh_Distance_BA_R0_R2	18,28282166	-78,01672363	-939,947937	0,054037
Cloud_Mesh_Distance_BA_R0_R2	18,25504494	-77,89073181	-940,0719605	0,064136
Cloud_Mesh_Distance_BA_R0_R2	18,35827637	-77,93135071	-939,9846191	0,055229
Cloud_Mesh_Distance_BA_R0_R2	18,71689224	-77,72866821	-940,0276489	0,050037
Cloud_Mesh_Distance_BA_R0_R2	18,78907394	-77,84950256	-939,9002686	0,029006
Cloud_Mesh_Distance_BA_R0_R2	18,55643082	-77,88708496	-939,9406738	0,046394
Cloud_Mesh_Distance_BA_R0_R2	20,625597	-78,17086792	-939,9309082	-0,027602
Cloud_Mesh_Distance_BA_R0_R2	20,91992188	-78,14215088	-940,0905151	-0,008727
Cloud_Mesh_Distance_BA_R0_R2	20,82142067	-78,11994934	-940,0582886	-0,013996
Cloud_Mesh_Distance_BA_R0_R2	21,09432983	-78,21815491	-940,1223755	0,010363
Cloud_Mesh_Distance_BA_R0_R2	18,60391808	-77,68409729	-940,1082153	0,065008
Cloud_Mesh_Distance_BA_R0_R2	18,88833237	-77,6015625	-940,0974731	0,058851
Cloud_Mesh_Distance_BA_R0_R2	18,9178772	-77,73062134	-939,9723511	0,039845
Cloud_Mesh_Distance_BA_R0_R2	19,09386063	-77,63519287	-940,0222168	0,04203
Cloud_Mesh_Distance_BA_R0_R2	19,19952965	-77,72822571	-939,9282837	0,022525
Cloud_Mesh_Distance_BA_R0_R2	19,05646896	-77,75541687	-939,9240723	0,025805
Cloud_Mesh_Distance_BA_R0_R2	20,16532707	-77,85655212	-939,9520264	-0,018967
Cloud_Mesh_Distance_BA_R0_R2	20,44069099	-77,8684845	-940,0617065	-0,02086
Cloud_Mesh_Distance_BA_R0_R2	20,62342834	-78,03057861	-940,0279541	-0,023838
Cloud_Mesh_Distance_BA_R0_R2	20,82579994	-77,96464539	-940,1947632	-0,015283
Cloud_Mesh_Distance_BA_R0_R2	20,76131439	-78,09197998	-940,0487061	-0,016255
Cloud_Mesh_Distance_BA_R0_R2	20,77623558	-78,02946472	-940,1073608	-0,017007
Cloud_Mesh_Distance_BA_R0_R2	20,89163017	-78,04450989	-940,1607056	-0,011048
Cloud_Mesh_Distance_BA_R0_R2	21,02683258	-78,09170532	-940,1998291	-0,00148
Cloud_Mesh_Distance_BA_R0_R2	18,80086136	-77,51664734	-940,2049561	0,071142
Cloud_Mesh_Distance_BA_R0_R2	18,92494774	-77,50189209	-940,18396	0,071023

Cloud_Mesh_Distance_BA_R0_R2	19,0528717	-77,51119995	-940,1462402	0,062137
Cloud_Mesh_Distance_BA_R0_R2	19,29026604	-77,62644958	-940,0063477	0,037716
Cloud_Mesh_Distance_BA_R0_R2	19,24112701	-77,51782227	-940,1101074	0,057487
Cloud_Mesh_Distance_BA_R0_R2	19,54831314	-77,6463623	-939,9859009	0,023188
Cloud_Mesh_Distance_BA_R0_R2	19,84292603	-77,59518433	-940,0671387	0,023066
Cloud_Mesh_Distance_BA_R0_R2	19,71546936	-77,67138672	-939,9793091	0,018346
Cloud_Mesh_Distance_BA_R0_R2	20,21822929	-77,75242615	-940,0524902	-0,00641
Cloud_Mesh_Distance_BA_R0_R2	20,0200386	-77,66036987	-940,0562744	0,010178
Cloud_Mesh_Distance_BA_R0_R2	20,25322533	-77,6519165	-940,1550903	0,002426
Cloud_Mesh_Distance_BA_R0_R2	20,55399704	-77,79655457	-940,1835938	-0,018435
Cloud_Mesh_Distance_BA_R0_R2	20,57838631	-77,89938354	-940,1072998	-0,023478
Cloud_Mesh_Distance_BA_R0_R2	18,9906311	-77,43003845	-940,241272	0,076977
Cloud_Mesh_Distance_BA_R0_R2	19,44873047	-77,54516602	-940,072937	0,043127
Cloud_Mesh_Distance_BA_R0_R2	19,65846062	-77,55618286	-940,0723267	0,037903
Cloud_Mesh_Distance_BA_R0_R2	20,14470291	-77,60089111	-940,1557617	0,009097
Cloud_Mesh_Distance_BA_R0_R2	19,02675247	-83,22860718	-939,6697998	-0,143373
Cloud_Mesh_Distance_BA_R0_R2	18,9621048	-83,09309387	-939,5853272	-0,148369
Cloud_Mesh_Distance_BA_R0_R2	19,22036552	-83,25611877	-939,6698608	-0,150223
Cloud_Mesh_Distance_BA_R0_R2	19,15084267	-83,13116455	-939,5852051	-0,14899
Cloud_Mesh_Distance_BA_R0_R2	19,33979607	-83,14933777	-939,5915527	-0,143995
Cloud_Mesh_Distance_BA_R0_R2	19,4160881	-83,26686096	-939,6774292	-0,147163
Cloud_Mesh_Distance_BA_R0_R2	19,85046768	-83,15881348	-939,6717529	-0,155508
Cloud_Mesh_Distance_BA_R0_R2	18,77809906	-82,88191223	-939,4994507	-0,156571
Cloud_Mesh_Distance_BA_R0_R2	18,5798111	-82,86401367	-939,5531616	-0,158252
Cloud_Mesh_Distance_BA_R0_R2	18,59656715	-83,01858521	-939,6425171	-0,148565
Cloud_Mesh_Distance_BA_R0_R2	18,75895119	-83,02401733	-939,5895996	-0,15301
Cloud_Mesh_Distance_BA_R0_R2	18,92246246	-82,95518494	-939,5076904	-0,148645
Cloud_Mesh_Distance_BA_R0_R2	19,27441216	-83,02749634	-939,5127564	-0,14151
Cloud_Mesh_Distance_BA_R0_R2	19,45519066	-83,03866577	-939,5255127	-0,144908
Cloud_Mesh_Distance_BA_R0_R2	19,61860657	-83,04048157	-939,5446777	-0,140908
Cloud_Mesh_Distance_BA_R0_R2	19,52664185	-83,15106201	-939,6044922	-0,147176
Cloud_Mesh_Distance_BA_R0_R2	19,71489525	-83,1333313	-939,6229248	-0,148249
Cloud_Mesh_Distance_BA_R0_R2	19,86962891	-83,04801941	-939,6036987	-0,14612
Cloud_Mesh_Distance_BA_R0_R2	18,0418129	-82,66532898	-939,7288818	-0,166845
Cloud_Mesh_Distance_BA_R0_R2	18,00017166	-82,53694153	-939,6670532	-0,164192
Cloud_Mesh_Distance_BA_R0_R2	18,55024719	-82,69480896	-939,4703369	-0,161613
Cloud_Mesh_Distance_BA_R0_R2	19,74298477	-83,02372742	-939,5577393	-0,140799
Cloud_Mesh_Distance_BA_R0_R2	19,80912209	-82,93844604	-939,5215454	-0,135701
Cloud_Mesh_Distance_BA_R0_R2	20,17069244	-82,99281311	-939,6680908	-0,147329
Cloud_Mesh_Distance_BA_R0_R2	20,1040535	-82,90315247	-939,5883179	-0,137434
Cloud_Mesh_Distance_BA_R0_R2	19,95287704	-82,92442322	-939,5513916	-0,132992
Cloud_Mesh_Distance_BA_R0_R2	20,03230286	-83,02215576	-939,6366577	-0,145645
Cloud_Mesh_Distance_BA_R0_R2	17,9068718	-82,56817627	-939,7692871	-0,166585
Cloud_Mesh_Distance_BA_R0_R2	18,15419579	-82,58349609	-939,592041	-0,171503
Cloud_Mesh_Distance_BA_R0_R2	18,01786423	-82,42485046	-939,5740967	-0,164487
Cloud_Mesh_Distance_BA_R0_R2	18,37561798	-82,69863892	-939,5431519	-0,169256
Cloud_Mesh_Distance_BA_R0_R2	18,46000099	-82,56776428	-939,4359131	-0,164424
Cloud_Mesh_Distance_BA_R0_R2	20,02344513	-82,80363464	-939,5040283	-0,121551
Cloud_Mesh_Distance_BA_R0_R2	20,18028831	-82,77462769	-939,5411377	-0,121003
Cloud_Mesh_Distance_BA_R0_R2	17,76758575	-82,34605408	-939,722229	-0,157318

Cloud_Mesh_Distance_BA_R0_R2	17,87546158	-82,31640625	-939,6018066	-0,156436
Cloud_Mesh_Distance_BA_R0_R2	17,89036179	-82,44085693	-939,684082	-0,163889
Cloud_Mesh_Distance_BA_R0_R2	20,26168442	-82,60696411	-939,4821167	-0,099531
Cloud_Mesh_Distance_BA_R0_R2	20,36442947	-82,73652649	-939,5892944	-0,116571
Cloud_Mesh_Distance_BA_R0_R2	20,46218109	-82,58435059	-939,5456543	-0,098765
Cloud_Mesh_Distance_BA_R0_R2	20,63828278	-82,53523254	-939,5921631	-0,089805
Cloud_Mesh_Distance_BA_R0_R2	20,58462334	-82,69569397	-939,6581421	-0,114091
Cloud_Mesh_Distance_BA_R0_R2	17,58854866	-82,0340271	-939,6561279	-0,120026
Cloud_Mesh_Distance_BA_R0_R2	17,88271713	-82,19355774	-939,5072022	-0,136859
Cloud_Mesh_Distance_BA_R0_R2	17,98219299	-82,30184937	-939,5130005	-0,150834
Cloud_Mesh_Distance_BA_R0_R2	20,75161743	-82,31575012	-939,538208	-0,066875
Cloud_Mesh_Distance_BA_R0_R2	20,77477837	-82,59315491	-939,6831055	-0,099956
Cloud_Mesh_Distance_BA_R0_R2	20,80968857	-82,44413757	-939,6198731	-0,077922
Cloud_Mesh_Distance_BA_R0_R2	17,53690338	-81,88464355	-939,6069946	-0,098292
Cloud_Mesh_Distance_BA_R0_R2	20,90881348	-82,30050659	-939,5979614	-0,063205
Cloud_Mesh_Distance_BA_R0_R2	20,97382736	-82,39788818	-939,6725464	-0,074626
Cloud_Mesh_Distance_BA_R0_R2	21,2266922	-82,15403748	-939,692749	-0,06397
Cloud_Mesh_Distance_BA_R0_R2	17,45273209	-81,58079529	-939,5427246	-0,0792
Cloud_Mesh_Distance_BA_R0_R2	17,37149048	-81,51889038	-939,6143189	-0,083918
Cloud_Mesh_Distance_BA_R0_R2	17,28699303	-81,5905304	-939,7613525	-0,08701
Cloud_Mesh_Distance_BA_R0_R2	17,39929962	-81,69514465	-939,6609497	-0,083354
Cloud_Mesh_Distance_BA_R0_R2	17,49311829	-81,77893066	-939,5924683	-0,085231
Cloud_Mesh_Distance_BA_R0_R2	17,59225082	-81,79270935	-939,4960327	-0,091117
Cloud_Mesh_Distance_BA_R0_R2	21,24037552	-81,98661804	-939,6425171	-0,062985
Cloud_Mesh_Distance_BA_R0_R2	21,35236359	-82,20417786	-939,7849731	-0,073235
Cloud_Mesh_Distance_BA_R0_R2	21,34924889	-82,06614685	-939,7280273	-0,070409
Cloud_Mesh_Distance_BA_R0_R2	21,06738853	-81,90571594	-939,5375977	-0,055349
Cloud_Mesh_Distance_BA_R0_R2	21,11871529	-81,98088074	-939,5809326	-0,056118
Cloud_Mesh_Distance_BA_R0_R2	17,2462635	-81,25790405	-939,6903076	-0,08378
Cloud_Mesh_Distance_BA_R0_R2	17,28503036	-81,40127563	-939,6837769	-0,084725
Cloud_Mesh_Distance_BA_R0_R2	21,17527008	-81,8792572	-939,5802002	-0,059624
Cloud_Mesh_Distance_BA_R0_R2	21,28454971	-81,81896973	-939,6177979	-0,066235
Cloud_Mesh_Distance_BA_R0_R2	21,20353508	-81,77015686	-939,5671387	-0,063518
Cloud_Mesh_Distance_BA_R0_R2	21,39028168	-81,66773987	-939,6360474	-0,078827
Cloud_Mesh_Distance_BA_R0_R2	21,38883209	-81,89874268	-939,6953735	-0,072794
Cloud_Mesh_Distance_BA_R0_R2	17,16985321	-81,21794128	-939,7884522	-0,084228
Cloud_Mesh_Distance_BA_R0_R2	17,24115562	-81,10829163	-939,6618042	-0,083255
Cloud_Mesh_Distance_BA_R0_R2	17,31558228	-81,01324463	-939,5556641	-0,080235
Cloud_Mesh_Distance_BA_R0_R2	21,23894119	-81,47276306	-939,5212402	-0,0725
Cloud_Mesh_Distance_BA_R0_R2	21,26231575	-81,69468689	-939,5776978	-0,06794
Cloud_Mesh_Distance_BA_R0_R2	21,25247192	-81,59634399	-939,5526123	-0,070087
Cloud_Mesh_Distance_BA_R0_R2	21,43812561	-81,51335144	-939,6259766	-0,085596
Cloud_Mesh_Distance_BA_R0_R2	21,58023834	-81,58761597	-939,7202759	-0,096003
Cloud_Mesh_Distance_BA_R0_R2	17,25295448	-80,8109436	-939,6071167	-0,076414
Cloud_Mesh_Distance_BA_R0_R2	17,24206161	-80,92701721	-939,6309815	-0,076896
Cloud_Mesh_Distance_BA_R0_R2	21,40623474	-81,41703796	-939,5901489	-0,084934
Cloud_Mesh_Distance_BA_R0_R2	21,32783508	-81,50740051	-939,5703125	-0,077117
Cloud_Mesh_Distance_BA_R0_R2	21,66118813	-81,42933655	-939,7260742	-0,104644
Cloud_Mesh_Distance_BA_R0_R2	21,60837173	-81,28593445	-939,6657105	-0,101601
Cloud_Mesh_Distance_BA_R0_R2	21,90171814	-81,41978455	-939,8637695	-0,122254

Cloud_Mesh_Distance_BA_R0_R2	21,86890793	-81,301651	-939,8114624	-0,119629
Cloud_Mesh_Distance_BA_R0_R2	17,26082611	-80,71286011	-939,5929565	-0,071308
Cloud_Mesh_Distance_BA_R0_R2	21,54813385	-81,16770935	-939,6107178	-0,09751
Cloud_Mesh_Distance_BA_R0_R2	21,74127769	-81,28538513	-939,7363281	-0,112164
Cloud_Mesh_Distance_BA_R0_R2	21,85469055	-81,13749695	-939,7633057	-0,114102
Cloud_Mesh_Distance_BA_R0_R2	17,2123909	-80,59773254	-939,6478272	-0,064764
Cloud_Mesh_Distance_BA_R0_R2	17,14551926	-80,47961426	-939,7330322	-0,051758
Cloud_Mesh_Distance_BA_R0_R2	17,24188805	-80,41993713	-939,6185913	-0,061271
Cloud_Mesh_Distance_BA_R0_R2	21,68684006	-81,10835266	-939,6690064	-0,10479
Cloud_Mesh_Distance_BA_R0_R2	22,05459023	-81,02613831	-939,8483276	-0,123098
Cloud_Mesh_Distance_BA_R0_R2	17,17134666	-80,29786682	-939,7129517	-0,043172
Cloud_Mesh_Distance_BA_R0_R2	17,07769203	-80,36210632	-939,8322144	-0,041301
Cloud_Mesh_Distance_BA_R0_R2	17,10393906	-80,17097473	-939,8164673	-0,03067
Cloud_Mesh_Distance_BA_R0_R2	21,69314003	-80,90718079	-939,633667	-0,101772
Cloud_Mesh_Distance_BA_R0_R2	21,83604431	-80,74038696	-939,6740112	-0,097159
Cloud_Mesh_Distance_BA_R0_R2	21,8319912	-80,95091248	-939,7109985	-0,109207
Cloud_Mesh_Distance_BA_R0_R2	21,9610672	-80,98881531	-939,7865601	-0,11431
Cloud_Mesh_Distance_BA_R0_R2	22,09249878	-80,88897705	-939,8380127	-0,11261
Cloud_Mesh_Distance_BA_R0_R2	17,25770569	-79,91499329	-939,6871338	-0,028303
Cloud_Mesh_Distance_BA_R0_R2	17,27125931	-80,24359131	-939,6054077	-0,048419
Cloud_Mesh_Distance_BA_R0_R2	17,20546913	-80,114151	-939,6999512	-0,037769
Cloud_Mesh_Distance_BA_R0_R2	17,31311417	-80,08125305	-939,5916748	-0,044346
Cloud_Mesh_Distance_BA_R0_R2	21,73599434	-80,62036133	-939,6085815	-0,086945
Cloud_Mesh_Distance_BA_R0_R2	21,78658867	-80,43888855	-939,6122437	-0,070556
Cloud_Mesh_Distance_BA_R0_R2	21,98088264	-80,81454468	-939,7613525	-0,103232
Cloud_Mesh_Distance_BA_R0_R2	17,39089012	-79,94403076	-939,5517578	-0,046007
Cloud_Mesh_Distance_BA_R0_R2	21,92430496	-80,33872986	-939,6716919	-0,062668
Cloud_Mesh_Distance_BA_R0_R2	21,89687729	-80,52015686	-939,6737671	-0,081066
Cloud_Mesh_Distance_BA_R0_R2	22,17765617	-80,57234192	-939,8292236	-0,085845
Cloud_Mesh_Distance_BA_R0_R2	22,03771973	-80,59861755	-939,7549439	-0,089519
Cloud_Mesh_Distance_BA_R0_R2	17,26930428	-79,53807068	-939,8029175	-0,007888
Cloud_Mesh_Distance_BA_R0_R2	17,25906563	-79,70664978	-939,7501831	-0,015387
Cloud_Mesh_Distance_BA_R0_R2	17,37431335	-79,58766174	-939,6832886	-0,024757
Cloud_Mesh_Distance_BA_R0_R2	17,46154404	-79,67805481	-939,5756836	-0,04099
Cloud_Mesh_Distance_BA_R0_R2	17,36211014	-79,77374268	-939,6280518	-0,035378
Cloud_Mesh_Distance_BA_R0_R2	22,17325211	-80,44078064	-939,8131104	-0,076759
Cloud_Mesh_Distance_BA_R0_R2	22,04810143	-80,3885498	-939,7388916	-0,069583
Cloud_Mesh_Distance_BA_R0_R2	22,31134224	-80,14424133	-939,9002075	-0,048463
Cloud_Mesh_Distance_BA_R0_R2	17,2184639	-79,40367126	-939,9155273	0,007291
Cloud_Mesh_Distance_BA_R0_R2	17,14748955	-79,61669922	-939,9081421	0,000911
Cloud_Mesh_Distance_BA_R0_R2	17,44805145	-79,47557068	-939,6657715	-0,027048
Cloud_Mesh_Distance_BA_R0_R2	21,93618011	-80,08279419	-939,6779785	-0,033199
Cloud_Mesh_Distance_BA_R0_R2	22,18371391	-80,25514221	-939,8113403	-0,056134
Cloud_Mesh_Distance_BA_R0_R2	22,04092216	-80,18365479	-939,7299194	-0,045061
Cloud_Mesh_Distance_BA_R0_R2	22,18360329	-80,03564453	-939,8257446	-0,035739
Cloud_Mesh_Distance_BA_R0_R2	17,36992455	-79,37458801	-939,7755737	-0,009954
Cloud_Mesh_Distance_BA_R0_R2	17,28141403	-79,22311401	-939,934021	0,008414
Cloud_Mesh_Distance_BA_R0_R2	17,49491882	-79,37818909	-939,6690674	-0,028249
Cloud_Mesh_Distance_BA_R0_R2	17,64631271	-79,21505737	-939,6300659	-0,034913
Cloud_Mesh_Distance_BA_R0_R2	17,70887566	-79,25984192	-939,5670776	-0,046597

Cloud_Mesh_Distance_BA_R0_R2	21,71119499	-79,65458679	-939,6378784	0,001331
Cloud_Mesh_Distance_BA_R0_R2	21,8660984	-79,71258545	-939,7025147	-0,003065
Cloud_Mesh_Distance_BA_R0_R2	21,97593117	-79,82725525	-939,736023	-0,013524
Cloud_Mesh_Distance_BA_R0_R2	21,78951454	-79,77330017	-939,6464233	-0,007701
Cloud_Mesh_Distance_BA_R0_R2	21,85591125	-79,85575867	-939,6644287	-0,0183
Cloud_Mesh_Distance_BA_R0_R2	21,9344101	-79,94915771	-939,6898804	-0,022584
Cloud_Mesh_Distance_BA_R0_R2	22,20279694	-79,77029419	-939,899292	-0,016328
Cloud_Mesh_Distance_BA_R0_R2	22,22868919	-79,88511658	-939,8845825	-0,021055
Cloud_Mesh_Distance_BA_R0_R2	22,11577606	-79,8309021	-939,8213501	-0,016206
Cloud_Mesh_Distance_BA_R0_R2	22,03967667	-79,98286438	-939,744751	-0,029433
Cloud_Mesh_Distance_BA_R0_R2	22,29885101	-79,97688293	-939,9152222	-0,034493
Cloud_Mesh_Distance_BA_R0_R2	17,38569641	-79,02381897	-939,9336548	0,010603
Cloud_Mesh_Distance_BA_R0_R2	17,39427376	-79,16242981	-939,8535767	0,0019
Cloud_Mesh_Distance_BA_R0_R2	17,52278328	-79,22538757	-939,7162476	-0,021862
Cloud_Mesh_Distance_BA_R0_R2	17,66232872	-79,07673645	-939,6829834	-0,024923
Cloud_Mesh_Distance_BA_R0_R2	17,7381916	-79,16186523	-939,5926514	-0,043036
Cloud_Mesh_Distance_BA_R0_R2	21,82561874	-79,53370667	-939,7382202	0,005292
Cloud_Mesh_Distance_BA_R0_R2	21,99511147	-79,64396667	-939,8000488	-0,00259
Cloud_Mesh_Distance_BA_R0_R2	17,5125618	-79,0226593	-939,8212891	-0,002821
Cloud_Mesh_Distance_BA_R0_R2	17,54924965	-78,87080383	-939,8712158	0,007502
Cloud_Mesh_Distance_BA_R0_R2	21,7544117	-79,34661865	-939,774353	0,019773
Cloud_Mesh_Distance_BA_R0_R2	22,02631187	-79,36024475	-939,9509888	0,009156
Cloud_Mesh_Distance_BA_R0_R2	21,92137527	-79,41239929	-939,8486328	0,012242
Cloud_Mesh_Distance_BA_R0_R2	17,6652298	-78,88825989	-939,7724609	-0,003029
Cloud_Mesh_Distance_BA_R0_R2	17,79542923	-78,9438324	-939,656311	-0,025436
Cloud_Mesh_Distance_BA_R0_R2	21,32696533	-78,99769592	-939,7201538	0,0322
Cloud_Mesh_Distance_BA_R0_R2	21,38456917	-79,1204071	-939,6859131	0,029631
Cloud_Mesh_Distance_BA_R0_R2	21,62936783	-79,30207825	-939,723999	0,021555
Cloud_Mesh_Distance_BA_R0_R2	21,65172577	-79,16618347	-939,7996826	0,026916
Cloud_Mesh_Distance_BA_R0_R2	21,81086731	-79,23258972	-939,8640137	0,02155
Cloud_Mesh_Distance_BA_R0_R2	21,92706871	-79,26399231	-939,9289551	0,015388
Cloud_Mesh_Distance_BA_R0_R2	17,87506866	-78,51139832	-939,8370972	0,024086
Cloud_Mesh_Distance_BA_R0_R2	17,71606827	-78,76573181	-939,8014526	0,002917
Cloud_Mesh_Distance_BA_R0_R2	17,82520485	-78,76829529	-939,7250977	-0,007484
Cloud_Mesh_Distance_BA_R0_R2	17,91817474	-78,60289001	-939,7549439	0,010661
Cloud_Mesh_Distance_BA_R0_R2	21,33065414	-78,84011841	-939,8026733	0,039542
Cloud_Mesh_Distance_BA_R0_R2	21,54477119	-78,9440155	-939,8534546	0,041376
Cloud_Mesh_Distance_BA_R0_R2	21,61651993	-79,00523376	-939,8617554	0,039107
Cloud_Mesh_Distance_BA_R0_R2	21,48375702	-79,01524353	-939,7839356	0,035014
Cloud_Mesh_Distance_BA_R0_R2	18,018116	-78,32923889	-939,8618774	0,03699
Cloud_Mesh_Distance_BA_R0_R2	18,01719475	-78,4473114	-939,786377	0,020473
Cloud_Mesh_Distance_BA_R0_R2	18,15302467	-78,43617249	-939,7182007	0,009939
Cloud_Mesh_Distance_BA_R0_R2	18,20731735	-78,50842285	-939,6484375	-0,003801
Cloud_Mesh_Distance_BA_R0_R2	18,18958855	-78,28689575	-939,7941895	0,030674
Cloud_Mesh_Distance_BA_R0_R2	21,16888237	-78,82719421	-939,7438355	0,026466
Cloud_Mesh_Distance_BA_R0_R2	21,32610703	-78,66798401	-939,8987427	0,045857
Cloud_Mesh_Distance_BA_R0_R2	21,16328049	-78,69314575	-939,8157349	0,025198
Cloud_Mesh_Distance_BA_R0_R2	21,44724274	-78,74908447	-939,9100952	0,048778
Cloud_Mesh_Distance_BA_R0_R2	18,35572433	-78,26618958	-939,7288208	0,017858
Cloud_Mesh_Distance_BA_R0_R2	20,33233833	-78,40657043	-939,6911621	-0,061321

Cloud_Mesh_Distance_BA_R0_R2	20,40789223	-78,34843445	-939,7459717	-0,052753
Cloud_Mesh_Distance_BA_R0_R2	20,54865646	-78,34814453	-939,7954102	-0,040476
Cloud_Mesh_Distance_BA_R0_R2	20,65363312	-78,50639343	-939,7456665	-0,035105
Cloud_Mesh_Distance_BA_R0_R2	20,82624435	-78,45297241	-939,8343506	-0,013055
Cloud_Mesh_Distance_BA_R0_R2	21,02148819	-78,53494263	-939,855896	0,013846
Cloud_Mesh_Distance_BA_R0_R2	21,21609497	-78,55453491	-939,9212036	0,036353
Cloud_Mesh_Distance_BA_R0_R2	21,43742561	-78,63021851	-939,9816284	0,051871
Cloud_Mesh_Distance_BA_R0_R2	21,52044487	-78,6023407	-940,0536499	0,054607
Cloud_Mesh_Distance_BA_R0_R2	18,24728584	-78,11682129	-939,8864746	0,04439
Cloud_Mesh_Distance_BA_R0_R2	18,39420509	-78,01983643	-939,8936768	0,042511
Cloud_Mesh_Distance_BA_R0_R2	18,46301079	-78,20925903	-939,7243652	0,015159
Cloud_Mesh_Distance_BA_R0_R2	18,38665962	-78,14923096	-939,796936	0,028877
Cloud_Mesh_Distance_BA_R0_R2	18,5293045	-78,03909302	-939,824585	0,03105
Cloud_Mesh_Distance_BA_R0_R2	19,3448391	-78,09794617	-939,6547852	-0,038771
Cloud_Mesh_Distance_BA_R0_R2	19,48504257	-77,97285461	-939,7409058	-0,024159
Cloud_Mesh_Distance_BA_R0_R2	19,54009438	-78,09565735	-939,6658325	-0,040648
Cloud_Mesh_Distance_BA_R0_R2	19,67743874	-78,09266663	-939,6843872	-0,044062
Cloud_Mesh_Distance_BA_R0_R2	20,25859833	-78,16236877	-939,7940674	-0,048483
Cloud_Mesh_Distance_BA_R0_R2	20,29212952	-78,29655457	-939,7338257	-0,057889
Cloud_Mesh_Distance_BA_R0_R2	20,55010414	-78,22938538	-939,8639526	-0,038632
Cloud_Mesh_Distance_BA_R0_R2	20,69576454	-78,38453674	-939,8278198	-0,027069
Cloud_Mesh_Distance_BA_R0_R2	21,34377098	-78,56297302	-939,9776001	0,047523
Cloud_Mesh_Distance_BA_R0_R2	18,61745262	-78,08444214	-939,7602539	0,017068
Cloud_Mesh_Distance_BA_R0_R2	18,689291	-77,98086548	-939,819519	0,022504
Cloud_Mesh_Distance_BA_R0_R2	19,11012077	-77,86367798	-939,8302612	0,006115
Cloud_Mesh_Distance_BA_R0_R2	19,30617523	-77,80021667	-939,8629761	0,006972
Cloud_Mesh_Distance_BA_R0_R2	19,457901	-77,84355164	-939,8300781	-0,004396
Cloud_Mesh_Distance_BA_R0_R2	19,28521538	-77,951828	-939,7528076	-0,016506
Cloud_Mesh_Distance_BA_R0_R2	19,62323761	-77,87487793	-939,8173218	-0,014614
Cloud_Mesh_Distance_BA_R0_R2	19,64809227	-77,99461365	-939,7405396	-0,030815
Cloud_Mesh_Distance_BA_R0_R2	19,95670509	-77,92285156	-939,8433228	-0,024693
Cloud_Mesh_Distance_BA_R0_R2	19,78962708	-77,9198761	-939,8092651	-0,02141
Cloud_Mesh_Distance_BA_R0_R2	19,88285446	-78,03018188	-939,7590332	-0,038698
Cloud_Mesh_Distance_BA_R0_R2	20,4401474	-78,00421143	-939,9606934	-0,029161
Cloud_Mesh_Distance_BA_R0_R2	20,41506767	-78,22640991	-939,8144531	-0,04607
Cloud_Mesh_Distance_BA_R0_R2	20,79878807	-78,08235168	-940,0755615	-0,015538
Cloud_Mesh_Distance_BA_R0_R2	18,95451355	-77,82969666	-939,8800659	0,020884
Cloud_Mesh_Distance_BA_R0_R2	19,3324337	-77,70405579	-939,9385986	0,021544
Cloud_Mesh_Distance_BA_R0_R2	19,44390297	-77,72946167	-939,9156494	0,014699
Cloud_Mesh_Distance_BA_R0_R2	19,59256363	-77,75460815	-939,9012451	0,005196
Cloud_Mesh_Distance_BA_R0_R2	20,03771782	-77,80697632	-939,9459229	-0,009774
Cloud_Mesh_Distance_BA_R0_R2	20,22695541	-78,04147339	-939,8529053	-0,038828
Cloud_Mesh_Distance_BA_R0_R2	20,28158188	-77,93196106	-939,9434815	-0,027788
Cloud_Mesh_Distance_BA_R0_R2	20,35365677	-78,07948303	-939,8774414	-0,041048
Cloud_Mesh_Distance_BA_R0_R2	20,84070587	-78,08435059	-940,0961304	-0,014265
Cloud_Mesh_Distance_BA_R0_R2	19,41465378	-77,64753723	-939,9822998	0,030204
Cloud_Mesh_Distance_BA_R0_R2	19,7509346	-77,78953552	-939,8934937	-0,001596
Cloud_Mesh_Distance_BA_R0_R2	19,8791256	-77,71931458	-939,9701538	0,003711
Cloud_Mesh_Distance_BA_R0_R2	19,8859539	-77,82695007	-939,8919067	-0,010693
Cloud_Mesh_Distance_BA_R0_R2	19,09404373	-83,00299072	-939,5093994	-0,148331

Cloud_Mesh_Distance_BA_R0_R2	19,21716499	-82,90299988	-939,4440308	-0,139732
Cloud_Mesh_Distance_BA_R0_R2	19,69280624	-82,9576416	-939,5072022	-0,133884
Cloud_Mesh_Distance_BA_R0_R2	18,73479271	-82,72001648	-939,4275513	-0,156577
Cloud_Mesh_Distance_BA_R0_R2	18,90289688	-82,82131958	-939,4398193	-0,151327
Cloud_Mesh_Distance_BA_R0_R2	19,16167831	-82,79940796	-939,3945923	-0,138908
Cloud_Mesh_Distance_BA_R0_R2	19,03410721	-82,75598145	-939,387207	-0,145524
Cloud_Mesh_Distance_BA_R0_R2	19,05264282	-82,87763977	-939,444519	-0,14902
Cloud_Mesh_Distance_BA_R0_R2	19,33580208	-82,76898193	-939,3755493	-0,132471
Cloud_Mesh_Distance_BA_R0_R2	19,40116692	-82,91331482	-939,449646	-0,137276
Cloud_Mesh_Distance_BA_R0_R2	19,71436882	-82,85714722	-939,454895	-0,128989
Cloud_Mesh_Distance_BA_R0_R2	19,5739212	-82,93319702	-939,4752197	-0,133587
Cloud_Mesh_Distance_BA_R0_R2	18,28704071	-82,51124573	-939,4755249	-0,164691
Cloud_Mesh_Distance_BA_R0_R2	18,60272026	-82,60548401	-939,4083862	-0,164803
Cloud_Mesh_Distance_BA_R0_R2	18,56011963	-82,48216248	-939,3623657	-0,163896
Cloud_Mesh_Distance_BA_R0_R2	18,70935631	-82,54650879	-939,3538208	-0,158839
Cloud_Mesh_Distance_BA_R0_R2	18,90721893	-82,70941162	-939,3849487	-0,150781
Cloud_Mesh_Distance_BA_R0_R2	19,18337631	-82,66960144	-939,335083	-0,134561
Cloud_Mesh_Distance_BA_R0_R2	19,01274872	-82,63238525	-939,3359985	-0,144386
Cloud_Mesh_Distance_BA_R0_R2	19,4953804	-82,64900208	-939,3282471	-0,118329
Cloud_Mesh_Distance_BA_R0_R2	19,34815407	-82,63005066	-939,3143921	-0,123124
Cloud_Mesh_Distance_BA_R0_R2	19,56113434	-82,78575134	-939,3965454	-0,12745
Cloud_Mesh_Distance_BA_R0_R2	19,75153351	-82,58447266	-939,3322754	-0,101341
Cloud_Mesh_Distance_BA_R0_R2	19,64204788	-82,65403748	-939,345459	-0,113119
Cloud_Mesh_Distance_BA_R0_R2	19,87132835	-82,82162476	-939,4711304	-0,123345
Cloud_Mesh_Distance_BA_R0_R2	19,95590591	-82,69111633	-939,4260864	-0,108743
Cloud_Mesh_Distance_BA_R0_R2	20,09579086	-82,68818665	-939,4666138	-0,108163
Cloud_Mesh_Distance_BA_R0_R2	18,08110428	-82,35202026	-939,484436	-0,152968
Cloud_Mesh_Distance_BA_R0_R2	18,14836121	-82,44692993	-939,507019	-0,159031
Cloud_Mesh_Distance_BA_R0_R2	18,20042992	-82,36125183	-939,428772	-0,149047
Cloud_Mesh_Distance_BA_R0_R2	18,38477135	-82,39608765	-939,3737793	-0,158451
Cloud_Mesh_Distance_BA_R0_R2	18,66889191	-82,41830444	-939,3079834	-0,155998
Cloud_Mesh_Distance_BA_R0_R2	19,12975121	-82,52345276	-939,2808838	-0,133326
Cloud_Mesh_Distance_BA_R0_R2	19,7701664	-82,70909119	-939,3914795	-0,113777
Cloud_Mesh_Distance_BA_R0_R2	20,08909416	-82,53977966	-939,3949585	-0,091154
Cloud_Mesh_Distance_BA_R0_R2	19,89542198	-82,56724548	-939,3552246	-0,096462
Cloud_Mesh_Distance_BA_R0_R2	20,15804863	-82,36553955	-939,3462524	-0,073938
Cloud_Mesh_Distance_BA_R0_R2	20,28100014	-82,42887878	-939,4100342	-0,080109
Cloud_Mesh_Distance_BA_R0_R2	20,403759	-82,47450256	-939,4714356	-0,083624
Cloud_Mesh_Distance_BA_R0_R2	17,65863037	-81,92417908	-939,5094605	-0,103362
Cloud_Mesh_Distance_BA_R0_R2	17,75489044	-82,05982971	-939,5145264	-0,120264
Cloud_Mesh_Distance_BA_R0_R2	18,05742073	-82,22703552	-939,4202881	-0,14146
Cloud_Mesh_Distance_BA_R0_R2	18,24621201	-82,06358337	-939,2557983	-0,12591
Cloud_Mesh_Distance_BA_R0_R2	18,10301018	-82,09225464	-939,3206787	-0,121552
Cloud_Mesh_Distance_BA_R0_R2	18,24838829	-82,22636414	-939,335144	-0,142635
Cloud_Mesh_Distance_BA_R0_R2	20,20915794	-82,2464447	-939,321106	-0,062329
Cloud_Mesh_Distance_BA_R0_R2	20,46527863	-82,31712341	-939,4302368	-0,068069
Cloud_Mesh_Distance_BA_R0_R2	20,31302643	-82,27632141	-939,3641357	-0,065134
Cloud_Mesh_Distance_BA_R0_R2	20,54352951	-82,44825745	-939,5126343	-0,0803
Cloud_Mesh_Distance_BA_R0_R2	20,67147064	-82,4213562	-939,5510254	-0,077486
Cloud_Mesh_Distance_BA_R0_R2	17,78436661	-81,94636536	-939,4193726	-0,102927

Cloud_Mesh_Distance_BA_R0_R2	17,92336273	-82,05233765	-939,3897095	-0,116179
Cloud_Mesh_Distance_BA_R0_R2	20,30722427	-82,16918945	-939,328186	-0,05764
Cloud_Mesh_Distance_BA_R0_R2	20,44242477	-82,14530945	-939,3655396	-0,057654
Cloud_Mesh_Distance_BA_R0_R2	20,61736107	-82,33482361	-939,493103	-0,068809
Cloud_Mesh_Distance_BA_R0_R2	20,98447418	-82,17610168	-939,5828247	-0,055932
Cloud_Mesh_Distance_BA_R0_R2	21,07911682	-82,26435852	-939,6622315	-0,064104
Cloud_Mesh_Distance_BA_R0_R2	21,10840225	-82,0980835	-939,6126099	-0,057588
Cloud_Mesh_Distance_BA_R0_R2	17,51691246	-81,67762756	-939,5151367	-0,080937
Cloud_Mesh_Distance_BA_R0_R2	17,45326233	-81,45605469	-939,49823	-0,076573
Cloud_Mesh_Distance_BA_R0_R2	17,7241478	-81,81988525	-939,3967285	-0,090373
Cloud_Mesh_Distance_BA_R0_R2	20,40293884	-81,89073181	-939,2872925	-0,050251
Cloud_Mesh_Distance_BA_R0_R2	20,6404953	-82,15516663	-939,4390259	-0,05733
Cloud_Mesh_Distance_BA_R0_R2	20,84057808	-82,17753601	-939,5222778	-0,056686
Cloud_Mesh_Distance_BA_R0_R2	17,44996262	-81,30995178	-939,4634399	-0,078055
Cloud_Mesh_Distance_BA_R0_R2	17,37732124	-81,37913513	-939,5604248	-0,078901
Cloud_Mesh_Distance_BA_R0_R2	17,54016495	-81,54246521	-939,4387817	-0,079732
Cloud_Mesh_Distance_BA_R0_R2	20,54931641	-81,8474884	-939,3268433	-0,050002
Cloud_Mesh_Distance_BA_R0_R2	20,65369415	-81,79457092	-939,3512573	-0,050115
Cloud_Mesh_Distance_BA_R0_R2	20,50125122	-81,9879303	-939,3433838	-0,051285
Cloud_Mesh_Distance_BA_R0_R2	20,65971947	-81,93534851	-939,3851929	-0,050346
Cloud_Mesh_Distance_BA_R0_R2	20,80853271	-82,006073	-939,4585571	-0,051392
Cloud_Mesh_Distance_BA_R0_R2	20,76284599	-81,79353333	-939,3902588	-0,051483
Cloud_Mesh_Distance_BA_R0_R2	20,94381332	-81,81903076	-939,4655151	-0,052969
Cloud_Mesh_Distance_BA_R0_R2	20,98272896	-82,0294342	-939,5349731	-0,053737
Cloud_Mesh_Distance_BA_R0_R2	17,34543037	-81,20674133	-939,5547485	-0,080862
Cloud_Mesh_Distance_BA_R0_R2	17,63658905	-81,30845642	-939,3024292	-0,071508
Cloud_Mesh_Distance_BA_R0_R2	17,54149818	-81,3913269	-939,3950806	-0,072324
Cloud_Mesh_Distance_BA_R0_R2	20,58966827	-81,72990417	-939,3158569	-0,051195
Cloud_Mesh_Distance_BA_R0_R2	21,10602188	-81,81159973	-939,5319214	-0,058047
Cloud_Mesh_Distance_BA_R0_R2	17,53632355	-81,09381104	-939,3518677	-0,073512
Cloud_Mesh_Distance_BA_R0_R2	17,45681572	-81,17314148	-939,4313965	-0,077878
Cloud_Mesh_Distance_BA_R0_R2	17,61684036	-81,17300415	-939,2975464	-0,07109
Cloud_Mesh_Distance_BA_R0_R2	17,53801537	-81,2427063	-939,3693848	-0,07303
Cloud_Mesh_Distance_BA_R0_R2	20,98518562	-81,58843994	-939,4346924	-0,059549
Cloud_Mesh_Distance_BA_R0_R2	20,9081192	-81,51664734	-939,390625	-0,05922
Cloud_Mesh_Distance_BA_R0_R2	21,13477516	-81,51678467	-939,4832153	-0,067256
Cloud_Mesh_Distance_BA_R0_R2	21,12707138	-81,68357849	-939,5133667	-0,061467
Cloud_Mesh_Distance_BA_R0_R2	21,17708969	-81,36677551	-939,4740601	-0,072943
Cloud_Mesh_Distance_BA_R0_R2	21,2720623	-81,24156189	-939,4946899	-0,081895
Cloud_Mesh_Distance_BA_R0_R2	21,29510117	-81,38555908	-939,5311279	-0,078828
Cloud_Mesh_Distance_BA_R0_R2	21,48744392	-81,29063416	-939,6048584	-0,093387
Cloud_Mesh_Distance_BA_R0_R2	21,54038239	-81,42247009	-939,6594849	-0,094408
Cloud_Mesh_Distance_BA_R0_R2	17,33742142	-80,55212402	-939,5116577	-0,066313
Cloud_Mesh_Distance_BA_R0_R2	17,44985771	-80,66925049	-939,4039917	-0,080024
Cloud_Mesh_Distance_BA_R0_R2	17,46135521	-80,83288574	-939,3961182	-0,074342
Cloud_Mesh_Distance_BA_R0_R2	17,339468	-80,81518555	-939,5119629	-0,075007
Cloud_Mesh_Distance_BA_R0_R2	21,26602554	-81,06257629	-939,461853	-0,083484
Cloud_Mesh_Distance_BA_R0_R2	21,5561142	-81,01200867	-939,5861816	-0,097333
Cloud_Mesh_Distance_BA_R0_R2	21,37963867	-81,31303406	-939,5573731	-0,086509
Cloud_Mesh_Distance_BA_R0_R2	17,43517685	-80,35316467	-939,4373779	-0,071563

Cloud_Mesh_Distance_BA_R0_R2	17,56008911	-80,37745667	-939,3372192	-0,079328
Cloud_Mesh_Distance_BA_R0_R2	17,46512985	-80,50343323	-939,3986816	-0,076531
Cloud_Mesh_Distance_BA_R0_R2	21,25179482	-80,9153595	-939,4332275	-0,080882
Cloud_Mesh_Distance_BA_R0_R2	21,42650986	-80,89012146	-939,5061646	-0,089423
Cloud_Mesh_Distance_BA_R0_R2	21,29993439	-80,76307678	-939,4331055	-0,080908
Cloud_Mesh_Distance_BA_R0_R2	21,41747856	-81,13894653	-939,5429688	-0,091025
Cloud_Mesh_Distance_BA_R0_R2	21,58571434	-80,85951233	-939,5742798	-0,095108
Cloud_Mesh_Distance_BA_R0_R2	17,33082581	-80,3527832	-939,5314941	-0,059403
Cloud_Mesh_Distance_BA_R0_R2	21,46598816	-80,6566925	-939,4914551	-0,08076
Cloud_Mesh_Distance_BA_R0_R2	21,68445015	-80,7693634	-939,605835	-0,095167
Cloud_Mesh_Distance_BA_R0_R2	17,38189316	-80,19725037	-939,5054932	-0,059507
Cloud_Mesh_Distance_BA_R0_R2	21,51224136	-80,46055603	-939,4905396	-0,070983
Cloud_Mesh_Distance_BA_R0_R2	21,58601189	-80,72125244	-939,5532837	-0,087939
Cloud_Mesh_Distance_BA_R0_R2	17,38871384	-80,07243347	-939,5230103	-0,054982
Cloud_Mesh_Distance_BA_R0_R2	17,47617722	-80,06474304	-939,451355	-0,059925
Cloud_Mesh_Distance_BA_R0_R2	21,62974167	-80,53646851	-939,5491333	-0,078463
Cloud_Mesh_Distance_BA_R0_R2	21,8305645	-80,29655457	-939,6229248	-0,056563
Cloud_Mesh_Distance_BA_R0_R2	21,68840218	-80,32801819	-939,5584717	-0,0578
Cloud_Mesh_Distance_BA_R0_R2	17,4518795	-79,80741882	-939,5400391	-0,045252
Cloud_Mesh_Distance_BA_R0_R2	17,54586029	-79,74467468	-939,4867554	-0,054338
Cloud_Mesh_Distance_BA_R0_R2	21,54229736	-80,28277588	-939,4934082	-0,054699
Cloud_Mesh_Distance_BA_R0_R2	21,53004837	-80,15794373	-939,4871826	-0,04545
Cloud_Mesh_Distance_BA_R0_R2	21,81760216	-80,17260742	-939,6157227	-0,045711
Cloud_Mesh_Distance_BA_R0_R2	21,91524506	-80,21305847	-939,6633911	-0,048327
Cloud_Mesh_Distance_BA_R0_R2	17,47716331	-79,56082153	-939,6069336	-0,036853
Cloud_Mesh_Distance_BA_R0_R2	17,53836441	-79,46760559	-939,5979614	-0,040979
Cloud_Mesh_Distance_BA_R0_R2	17,57672119	-79,58673096	-939,5210571	-0,051773
Cloud_Mesh_Distance_BA_R0_R2	21,55426788	-80,01976013	-939,5039063	-0,033254
Cloud_Mesh_Distance_BA_R0_R2	21,67174339	-80,11901855	-939,5487671	-0,040816
Cloud_Mesh_Distance_BA_R0_R2	21,82667542	-80,00726318	-939,628418	-0,026066
Cloud_Mesh_Distance_BA_R0_R2	21,69751358	-79,87786865	-939,5824585	-0,016284
Cloud_Mesh_Distance_BA_R0_R2	17,61115456	-79,33827209	-939,5999756	-0,040862
Cloud_Mesh_Distance_BA_R0_R2	17,63742638	-79,45375061	-939,53125	-0,050075
Cloud_Mesh_Distance_BA_R0_R2	17,72407913	-79,51274109	-939,4485474	-0,061511
Cloud_Mesh_Distance_BA_R0_R2	17,8087883	-79,2172699	-939,5211792	-0,053289
Cloud_Mesh_Distance_BA_R0_R2	17,88720703	-79,26972961	-939,4497681	-0,062413
Cloud_Mesh_Distance_BA_R0_R2	17,74833298	-79,35295105	-939,499939	-0,060152
Cloud_Mesh_Distance_BA_R0_R2	21,33978271	-79,64283752	-939,4802246	-0,000167
Cloud_Mesh_Distance_BA_R0_R2	21,43907166	-79,81034851	-939,4822388	-0,011744
Cloud_Mesh_Distance_BA_R0_R2	21,54221153	-79,87997437	-939,5131226	-0,016997
Cloud_Mesh_Distance_BA_R0_R2	21,56390381	-79,73321533	-939,5491333	-0,004705
Cloud_Mesh_Distance_BA_R0_R2	17,89102173	-79,01098633	-939,5653076	-0,041522
Cloud_Mesh_Distance_BA_R0_R2	17,78178215	-79,06550598	-939,6079102	-0,039744
Cloud_Mesh_Distance_BA_R0_R2	17,83206177	-79,11891174	-939,5518799	-0,047644
Cloud_Mesh_Distance_BA_R0_R2	21,37089539	-79,46702576	-939,5429688	0,013053
Cloud_Mesh_Distance_BA_R0_R2	21,51478004	-79,52160645	-939,5858154	0,010941
Cloud_Mesh_Distance_BA_R0_R2	21,4383564	-79,6676178	-939,5114136	-0,000275
Cloud_Mesh_Distance_BA_R0_R2	21,66833687	-79,44920349	-939,6842651	0,011146
Cloud_Mesh_Distance_BA_R0_R2	20,88541794	-79,26725769	-939,4520264	-0,002015
Cloud_Mesh_Distance_BA_R0_R2	20,96975708	-79,30540466	-939,4619751	0,003364

Cloud_Mesh_Distance_BA_R0_R2	20,93344116	-79,19442749	-939,4946289	0,000183
Cloud_Mesh_Distance_BA_R0_R2	21,11016655	-79,35723877	-939,4869385	0,0086
Cloud_Mesh_Distance_BA_R0_R2	18,15661621	-78,66970825	-939,5863037	-0,020852
Cloud_Mesh_Distance_BA_R0_R2	17,98447609	-78,68914795	-939,6685791	-0,008917
Cloud_Mesh_Distance_BA_R0_R2	17,96789932	-78,84025574	-939,6014404	-0,028994
Cloud_Mesh_Distance_BA_R0_R2	18,2387886	-78,77668762	-939,4932251	-0,043039
Cloud_Mesh_Distance_BA_R0_R2	18,31849289	-78,6557312	-939,5198975	-0,032495
Cloud_Mesh_Distance_BA_R0_R2	18,11077499	-78,83056641	-939,5290527	-0,038976
Cloud_Mesh_Distance_BA_R0_R2	18,37185669	-78,72413635	-939,4632568	-0,043731
Cloud_Mesh_Distance_BA_R0_R2	20,8656826	-79,12194824	-939,5043945	-0,003103
Cloud_Mesh_Distance_BA_R0_R2	20,84010887	-79,04034424	-939,5330811	-0,004654
Cloud_Mesh_Distance_BA_R0_R2	21,2073307	-79,27160645	-939,5513306	0,016873
Cloud_Mesh_Distance_BA_R0_R2	21,07071114	-79,2118988	-939,5296021	0,011275
Cloud_Mesh_Distance_BA_R0_R2	20,98082733	-79,06906128	-939,5617676	0,00819
Cloud_Mesh_Distance_BA_R0_R2	21,54495811	-79,35368347	-939,6593628	0,019211
Cloud_Mesh_Distance_BA_R0_R2	21,51468658	-79,20689392	-939,7080078	0,027364
Cloud_Mesh_Distance_BA_R0_R2	21,37893295	-79,30215454	-939,6067505	0,018335
Cloud_Mesh_Distance_BA_R0_R2	18,07029533	-78,55909729	-939,6904907	0,001209
Cloud_Mesh_Distance_BA_R0_R2	18,37537766	-78,49569702	-939,5819702	-0,015864
Cloud_Mesh_Distance_BA_R0_R2	18,4550724	-78,60559082	-939,4928589	-0,036761
Cloud_Mesh_Distance_BA_R0_R2	20,19036102	-78,64341736	-939,5297241	-0,06935
Cloud_Mesh_Distance_BA_R0_R2	20,03544235	-78,6105957	-939,4984131	-0,071969
Cloud_Mesh_Distance_BA_R0_R2	20,56605721	-78,91905212	-939,5126343	-0,034704
Cloud_Mesh_Distance_BA_R0_R2	20,71663284	-78,84199524	-939,5922241	-0,023815
Cloud_Mesh_Distance_BA_R0_R2	21,06012917	-78,91162109	-939,6625366	0,013613
Cloud_Mesh_Distance_BA_R0_R2	21,22062111	-78,93161011	-939,7091675	0,027535
Cloud_Mesh_Distance_BA_R0_R2	21,20684624	-79,09777832	-939,6255493	0,022274
Cloud_Mesh_Distance_BA_R0_R2	18,54139137	-78,491745	-939,5251465	-0,029213
Cloud_Mesh_Distance_BA_R0_R2	18,8183136	-78,41394043	-939,5004273	-0,03892
Cloud_Mesh_Distance_BA_R0_R2	18,78631783	-78,32540894	-939,5583496	-0,029634
Cloud_Mesh_Distance_BA_R0_R2	20,12287521	-78,5063324	-939,5744629	-0,070393
Cloud_Mesh_Distance_BA_R0_R2	20,53948975	-78,76773071	-939,576294	-0,043314
Cloud_Mesh_Distance_BA_R0_R2	20,50221634	-78,60899353	-939,6433716	-0,047423
Cloud_Mesh_Distance_BA_R0_R2	20,3603611	-78,69923401	-939,5562744	-0,060673
Cloud_Mesh_Distance_BA_R0_R2	20,87167358	-78,93367004	-939,5928345	-0,00401
Cloud_Mesh_Distance_BA_R0_R2	21,05965042	-78,77339172	-939,7335205	0,017061
Cloud_Mesh_Distance_BA_R0_R2	20,89514923	-78,77249146	-939,6803589	-0,003365
Cloud_Mesh_Distance_BA_R0_R2	18,28755569	-78,3828125	-939,6842651	0,008538
Cloud_Mesh_Distance_BA_R0_R2	18,44662476	-78,3140564	-939,661377	0,002795
Cloud_Mesh_Distance_BA_R0_R2	18,59864807	-78,35134888	-939,5886231	-0,015367
Cloud_Mesh_Distance_BA_R0_R2	18,59423828	-78,19494629	-939,68927	0,005413
Cloud_Mesh_Distance_BA_R0_R2	19,33457184	-78,31306458	-939,5236206	-0,05817
Cloud_Mesh_Distance_BA_R0_R2	19,4393177	-78,2142334	-939,5864258	-0,04996
Cloud_Mesh_Distance_BA_R0_R2	19,86069489	-78,44534302	-939,5290527	-0,070481
Cloud_Mesh_Distance_BA_R0_R2	20,20184708	-78,3911438	-939,6560059	-0,06734
Cloud_Mesh_Distance_BA_R0_R2	20,45801163	-78,45957947	-939,7051392	-0,053426
Cloud_Mesh_Distance_BA_R0_R2	20,30881882	-78,53192139	-939,6206665	-0,063252
Cloud_Mesh_Distance_BA_R0_R2	20,69382477	-78,68325806	-939,6651611	-0,027596
Cloud_Mesh_Distance_BA_R0_R2	21,00016403	-78,67367554	-939,7670898	0,008741
Cloud_Mesh_Distance_BA_R0_R2	20,85005951	-78,60307312	-939,7567749	-0,010871

Cloud_Mesh_Distance_BA_R0_R2	18,52074242	-78,13311768	-939,756958	0,018553
Cloud_Mesh_Distance_BA_R0_R2	18,75917625	-78,20835876	-939,6380005	-0,010839
Cloud_Mesh_Distance_BA_R0_R2	18,94191742	-78,20922852	-939,6050415	-0,024706
Cloud_Mesh_Distance_BA_R0_R2	19,06944847	-78,04386902	-939,7024536	-0,016782
Cloud_Mesh_Distance_BA_R0_R2	19,28857613	-78,2255249	-939,5737305	-0,046922
Cloud_Mesh_Distance_BA_R0_R2	19,15146828	-78,17895508	-939,6056519	-0,035026
Cloud_Mesh_Distance_BA_R0_R2	19,21410942	-78,06646729	-939,6772461	-0,026207
Cloud_Mesh_Distance_BA_R0_R2	19,63578606	-78,2204895	-939,604126	-0,057088
Cloud_Mesh_Distance_BA_R0_R2	19,82733345	-78,28399658	-939,604187	-0,061734
Cloud_Mesh_Distance_BA_R0_R2	20,01679039	-78,36047363	-939,6138306	-0,065972
Cloud_Mesh_Distance_BA_R0_R2	20,1437664	-78,2437439	-939,711853	-0,060015
Cloud_Mesh_Distance_BA_R0_R2	18,8921051	-77,96099854	-939,7878418	0,008321
Cloud_Mesh_Distance_BA_R0_R2	18,88245964	-78,09910583	-939,6879273	-0,00754
Cloud_Mesh_Distance_BA_R0_R2	18,74108315	-78,0854187	-939,7268677	0,004599
Cloud_Mesh_Distance_BA_R0_R2	19,79428864	-78,13415527	-939,6798706	-0,04826
Cloud_Mesh_Distance_BA_R0_R2	19,7603569	-78,03141785	-939,7342529	-0,036437
Cloud_Mesh_Distance_BA_R0_R2	20,01173401	-78,02952576	-939,791687	-0,037554
Cloud_Mesh_Distance_BA_R0_R2	20,12238121	-78,09593201	-939,7855835	-0,045222
Cloud_Mesh_Distance_BA_R0_R2	19,97244453	-78,16494751	-939,7041016	-0,053409
Cloud_Mesh_Distance_BA_R0_R2	20,11642265	-77,95082092	-939,8717651	-0,029026
Cloud_Mesh_Distance_BA_R0_R2	18,86069107	-82,59684753	-939,3446655	-0,155279
Cloud_Mesh_Distance_BA_R0_R2	18,40655327	-82,26405334	-939,3023682	-0,148234
Cloud_Mesh_Distance_BA_R0_R2	18,538311	-82,33930969	-939,3027954	-0,153621
Cloud_Mesh_Distance_BA_R0_R2	18,83531952	-82,43391418	-939,2837524	-0,153668
Cloud_Mesh_Distance_BA_R0_R2	18,97504807	-82,51472473	-939,2938843	-0,142832
Cloud_Mesh_Distance_BA_R0_R2	18,89061546	-82,29225159	-939,2241211	-0,141115
Cloud_Mesh_Distance_BA_R0_R2	19,28041077	-82,54524231	-939,2816162	-0,123128
Cloud_Mesh_Distance_BA_R0_R2	19,04737091	-82,36909485	-939,2341919	-0,13395
Cloud_Mesh_Distance_BA_R0_R2	19,29447556	-82,38587952	-939,2259522	-0,111718
Cloud_Mesh_Distance_BA_R0_R2	19,45800972	-82,49897766	-939,2669678	-0,107774
Cloud_Mesh_Distance_BA_R0_R2	19,63626289	-82,53865051	-939,296875	-0,099662
Cloud_Mesh_Distance_BA_R0_R2	19,65522766	-82,38227844	-939,2431641	-0,08697
Cloud_Mesh_Distance_BA_R0_R2	19,8055439	-82,45539856	-939,2923584	-0,088964
Cloud_Mesh_Distance_BA_R0_R2	18,40836906	-82,06956482	-939,2189331	-0,132663
Cloud_Mesh_Distance_BA_R0_R2	18,36072159	-82,16349792	-939,2687378	-0,138452
Cloud_Mesh_Distance_BA_R0_R2	18,51883507	-82,07460022	-939,2005615	-0,137852
Cloud_Mesh_Distance_BA_R0_R2	18,70814896	-82,27326965	-939,2426758	-0,148856
Cloud_Mesh_Distance_BA_R0_R2	18,51190948	-82,18200684	-939,2425537	-0,142022
Cloud_Mesh_Distance_BA_R0_R2	19,5014267	-82,33834839	-939,2151489	-0,097462
Cloud_Mesh_Distance_BA_R0_R2	19,4424324	-82,2081604	-939,1745606	-0,095244
Cloud_Mesh_Distance_BA_R0_R2	19,98493767	-82,41435242	-939,3165894	-0,078672
Cloud_Mesh_Distance_BA_R0_R2	17,88398933	-81,8690033	-939,3155518	-0,094001
Cloud_Mesh_Distance_BA_R0_R2	18,02945137	-81,8250885	-939,2280884	-0,093013
Cloud_Mesh_Distance_BA_R0_R2	18,03525543	-81,9630127	-939,2840576	-0,107197
Cloud_Mesh_Distance_BA_R0_R2	18,13372803	-82,01002502	-939,2676392	-0,116255
Cloud_Mesh_Distance_BA_R0_R2	18,33480835	-81,94917297	-939,1882324	-0,121132
Cloud_Mesh_Distance_BA_R0_R2	18,66653824	-82,06774902	-939,177124	-0,13742
Cloud_Mesh_Distance_BA_R0_R2	18,86572075	-81,99841309	-939,1369019	-0,129507
Cloud_Mesh_Distance_BA_R0_R2	18,86297226	-82,1541748	-939,1824951	-0,138875
Cloud_Mesh_Distance_BA_R0_R2	19,24074745	-82,02281189	-939,1260376	-0,105688

Cloud_Mesh_Distance_BA_R0_R2	19,21148491	-82,19052124	-939,1695557	-0,1119
Cloud_Mesh_Distance_BA_R0_R2	19,05313873	-82,05871582	-939,1411133	-0,122534
Cloud_Mesh_Distance_BA_R0_R2	19,01078987	-82,21580505	-939,1879883	-0,131406
Cloud_Mesh_Distance_BA_R0_R2	19,64479065	-82,21125793	-939,189148	-0,075652
Cloud_Mesh_Distance_BA_R0_R2	19,5886879	-82,06530762	-939,1459961	-0,076258
Cloud_Mesh_Distance_BA_R0_R2	19,8836937	-82,06257629	-939,1869507	-0,058771
Cloud_Mesh_Distance_BA_R0_R2	19,8324337	-81,96841431	-939,1552124	-0,058197
Cloud_Mesh_Distance_BA_R0_R2	19,85813141	-82,24739075	-939,2327881	-0,068672
Cloud_Mesh_Distance_BA_R0_R2	20,07043457	-82,26350403	-939,2871094	-0,064589
Cloud_Mesh_Distance_BA_R0_R2	20,35041046	-82,04719543	-939,3082886	-0,052325
Cloud_Mesh_Distance_BA_R0_R2	17,65100479	-81,65725708	-939,3831177	-0,07908
Cloud_Mesh_Distance_BA_R0_R2	17,8055191	-81,71324158	-939,2958374	-0,081709
Cloud_Mesh_Distance_BA_R0_R2	17,90491486	-81,61555481	-939,2115479	-0,075111
Cloud_Mesh_Distance_BA_R0_R2	17,77126694	-81,55241394	-939,2647705	-0,072284
Cloud_Mesh_Distance_BA_R0_R2	18,50572968	-81,96903992	-939,1657105	-0,130052
Cloud_Mesh_Distance_BA_R0_R2	19,17695427	-81,92175293	-939,1047363	-0,108916
Cloud_Mesh_Distance_BA_R0_R2	20,00350571	-81,8391571	-939,1640625	-0,051361
Cloud_Mesh_Distance_BA_R0_R2	20,0422821	-82,09066772	-939,2304077	-0,055992
Cloud_Mesh_Distance_BA_R0_R2	20,2232666	-81,96052551	-939,24823	-0,050643
Cloud_Mesh_Distance_BA_R0_R2	20,07177353	-81,92228699	-939,197937	-0,05127
Cloud_Mesh_Distance_BA_R0_R2	20,20178032	-82,13694763	-939,2863159	-0,056645
Cloud_Mesh_Distance_BA_R0_R2	17,75653648	-81,3764801	-939,2351685	-0,070043
Cloud_Mesh_Distance_BA_R0_R2	17,64586258	-81,47471619	-939,3305664	-0,070744
Cloud_Mesh_Distance_BA_R0_R2	17,89116859	-81,45213318	-939,1794434	-0,071349
Cloud_Mesh_Distance_BA_R0_R2	20,11914063	-81,78294373	-939,1823731	-0,050672
Cloud_Mesh_Distance_BA_R0_R2	20,30871391	-81,7361908	-939,2276611	-0,050412
Cloud_Mesh_Distance_BA_R0_R2	20,83916473	-81,65696716	-939,3902588	-0,053833
Cloud_Mesh_Distance_BA_R0_R2	17,88154984	-81,270401	-939,1557617	-0,073153
Cloud_Mesh_Distance_BA_R0_R2	20,33468819	-81,44128418	-939,1899414	-0,059113
Cloud_Mesh_Distance_BA_R0_R2	20,48202515	-81,75724792	-939,2862549	-0,052138
Cloud_Mesh_Distance_BA_R0_R2	20,695961	-81,67932129	-939,3432007	-0,052916
Cloud_Mesh_Distance_BA_R0_R2	20,55998421	-81,57591248	-939,2775879	-0,054502
Cloud_Mesh_Distance_BA_R0_R2	20,76556206	-81,54417419	-939,3422852	-0,056957
Cloud_Mesh_Distance_BA_R0_R2	17,64519691	-81,03572083	-939,2652588	-0,077935
Cloud_Mesh_Distance_BA_R0_R2	17,7366333	-81,17979431	-939,2200928	-0,070687
Cloud_Mesh_Distance_BA_R0_R2	17,76836777	-80,99223328	-939,1880493	-0,075968
Cloud_Mesh_Distance_BA_R0_R2	20,27142334	-81,25965881	-939,1539307	-0,069204
Cloud_Mesh_Distance_BA_R0_R2	20,7341938	-81,38938904	-939,3051148	-0,060693
Cloud_Mesh_Distance_BA_R0_R2	20,87229919	-81,4360199	-939,3622437	-0,060771
Cloud_Mesh_Distance_BA_R0_R2	17,42514992	-81,00891113	-939,4415894	-0,077232
Cloud_Mesh_Distance_BA_R0_R2	17,54811096	-80,92707825	-939,3294067	-0,074837
Cloud_Mesh_Distance_BA_R0_R2	17,87732887	-80,94197083	-939,1323242	-0,084031
Cloud_Mesh_Distance_BA_R0_R2	20,37575912	-81,14437866	-939,1690674	-0,072748
Cloud_Mesh_Distance_BA_R0_R2	20,61281204	-81,19023132	-939,2382202	-0,066143
Cloud_Mesh_Distance_BA_R0_R2	20,56624222	-81,32614136	-939,2418213	-0,062865
Cloud_Mesh_Distance_BA_R0_R2	20,738451	-81,19284058	-939,277771	-0,065728
Cloud_Mesh_Distance_BA_R0_R2	21,01600647	-81,40649414	-939,4135742	-0,065563
Cloud_Mesh_Distance_BA_R0_R2	21,10079002	-81,19335938	-939,4128418	-0,07416
Cloud_Mesh_Distance_BA_R0_R2	17,55871201	-80,74812317	-939,3175659	-0,078045
Cloud_Mesh_Distance_BA_R0_R2	17,6819706	-80,84562683	-939,234314	-0,08262

Cloud_Mesh_Distance_BA_R0_R2	17,81097984	-80,85704041	-939,161499	-0,083109
Cloud_Mesh_Distance_BA_R0_R2	17,79904938	-80,71080017	-939,1688843	-0,09068
Cloud_Mesh_Distance_BA_R0_R2	20,30130959	-80,9495697	-939,1334839	-0,083185
Cloud_Mesh_Distance_BA_R0_R2	20,27043152	-81,1010437	-939,1384277	-0,076863
Cloud_Mesh_Distance_BA_R0_R2	20,75643349	-81,05862427	-939,267395	-0,070554
Cloud_Mesh_Distance_BA_R0_R2	20,77813339	-80,95954895	-939,2639771	-0,072562
Cloud_Mesh_Distance_BA_R0_R2	20,93670464	-81,01472473	-939,3237305	-0,07224
Cloud_Mesh_Distance_BA_R0_R2	20,90024757	-81,27008057	-939,3457642	-0,066073
Cloud_Mesh_Distance_BA_R0_R2	21,13752747	-81,00758362	-939,3997803	-0,078535
Cloud_Mesh_Distance_BA_R0_R2	17,56027794	-80,56915283	-939,3206787	-0,080849
Cloud_Mesh_Distance_BA_R0_R2	17,6721077	-80,64979553	-939,2421265	-0,087804
Cloud_Mesh_Distance_BA_R0_R2	20,48562622	-80,95387268	-939,1813965	-0,080644
Cloud_Mesh_Distance_BA_R0_R2	20,66007423	-80,75630188	-939,2142944	-0,082681
Cloud_Mesh_Distance_BA_R0_R2	17,51532364	-80,27877808	-939,3826904	-0,072624
Cloud_Mesh_Distance_BA_R0_R2	20,45664597	-80,84463501	-939,164856	-0,083328
Cloud_Mesh_Distance_BA_R0_R2	20,5400753	-80,66879272	-939,1751099	-0,082776
Cloud_Mesh_Distance_BA_R0_R2	20,68081474	-80,59777832	-939,2105713	-0,081207
Cloud_Mesh_Distance_BA_R0_R2	20,74143219	-80,45326233	-939,2231445	-0,075082
Cloud_Mesh_Distance_BA_R0_R2	21,02832031	-80,79879761	-939,3330078	-0,077567
Cloud_Mesh_Distance_BA_R0_R2	20,85370636	-80,80488586	-939,2747192	-0,077701
Cloud_Mesh_Distance_BA_R0_R2	21,13779259	-80,8626709	-939,3809204	-0,078982
Cloud_Mesh_Distance_BA_R0_R2	17,59666634	-80,17094421	-939,3408203	-0,075719
Cloud_Mesh_Distance_BA_R0_R2	17,47843361	-80,21316528	-939,4208984	-0,065938
Cloud_Mesh_Distance_BA_R0_R2	17,73600006	-80,15884399	-939,2546387	-0,083586
Cloud_Mesh_Distance_BA_R0_R2	17,81450653	-80,2263031	-939,2006836	-0,089976
Cloud_Mesh_Distance_BA_R0_R2	17,73568344	-80,01507568	-939,2828369	-0,079576
Cloud_Mesh_Distance_BA_R0_R2	20,92058945	-80,3500824	-939,274231	-0,069742
Cloud_Mesh_Distance_BA_R0_R2	21,03553009	-80,63122559	-939,3208008	-0,075837
Cloud_Mesh_Distance_BA_R0_R2	21,07237816	-80,42680359	-939,3233032	-0,071279
Cloud_Mesh_Distance_BA_R0_R2	20,85395622	-80,57444763	-939,260376	-0,078909
Cloud_Mesh_Distance_BA_R0_R2	21,17785263	-80,68716431	-939,376648	-0,076702
Cloud_Mesh_Distance_BA_R0_R2	21,39184189	-80,39071655	-939,4358521	-0,06515
Cloud_Mesh_Distance_BA_R0_R2	21,34520531	-80,5750885	-939,4310303	-0,074006
Cloud_Mesh_Distance_BA_R0_R2	17,61090469	-79,96246338	-939,3757324	-0,068176
Cloud_Mesh_Distance_BA_R0_R2	17,5121479	-79,89111328	-939,46698	-0,057165
Cloud_Mesh_Distance_BA_R0_R2	20,79929352	-80,08006287	-939,24823	-0,05213
Cloud_Mesh_Distance_BA_R0_R2	20,95273209	-80,1492157	-939,2877197	-0,052714
Cloud_Mesh_Distance_BA_R0_R2	21,21905899	-80,498703	-939,376648	-0,072387
Cloud_Mesh_Distance_BA_R0_R2	17,67409515	-79,67875671	-939,4182129	-0,062648
Cloud_Mesh_Distance_BA_R0_R2	17,61637878	-79,81878662	-939,4118042	-0,064502
Cloud_Mesh_Distance_BA_R0_R2	17,75435257	-79,81221008	-939,3234863	-0,073108
Cloud_Mesh_Distance_BA_R0_R2	20,37346268	-79,94300842	-939,1489258	-0,04949
Cloud_Mesh_Distance_BA_R0_R2	20,80794525	-79,92599487	-939,2667236	-0,038637
Cloud_Mesh_Distance_BA_R0_R2	21,27913094	-80,08398438	-939,394043	-0,041666
Cloud_Mesh_Distance_BA_R0_R2	21,12414169	-80,02470398	-939,3474121	-0,038755
Cloud_Mesh_Distance_BA_R0_R2	21,10872269	-80,22047424	-939,3329468	-0,05626
Cloud_Mesh_Distance_BA_R0_R2	21,43695259	-80,24757385	-939,4495239	-0,054105
Cloud_Mesh_Distance_BA_R0_R2	21,27980423	-80,28457642	-939,3901978	-0,060325
Cloud_Mesh_Distance_BA_R0_R2	17,93893623	-79,71528625	-939,2472534	-0,0808
Cloud_Mesh_Distance_BA_R0_R2	17,99668694	-79,572052	-939,263855	-0,079273

Cloud_Mesh_Distance_BA_R0_R2	18,00563812	-79,44313049	-939,3074341	-0,076205
Cloud_Mesh_Distance_BA_R0_R2	17,84888077	-79,59172058	-939,3399048	-0,07278
Cloud_Mesh_Distance_BA_R0_R2	17,87673569	-79,40333557	-939,3980713	-0,068461
Cloud_Mesh_Distance_BA_R0_R2	20,38742065	-79,97462463	-939,1478272	-0,051208
Cloud_Mesh_Distance_BA_R0_R2	20,88161659	-79,76957703	-939,3114624	-0,028156
Cloud_Mesh_Distance_BA_R0_R2	20,73198318	-79,84152222	-939,2589111	-0,035381
Cloud_Mesh_Distance_BA_R0_R2	20,9661026	-79,94293213	-939,3085938	-0,035189
Cloud_Mesh_Distance_BA_R0_R2	21,04473114	-79,78111267	-939,3552856	-0,021962
Cloud_Mesh_Distance_BA_R0_R2	21,42251205	-80,12599182	-939,4451294	-0,044409
Cloud_Mesh_Distance_BA_R0_R2	20,85688782	-79,57226563	-939,3483887	-0,014753
Cloud_Mesh_Distance_BA_R0_R2	20,69406891	-79,52650452	-939,3175049	-0,021262
Cloud_Mesh_Distance_BA_R0_R2	21,02484512	-79,62992859	-939,3809204	-0,009006
Cloud_Mesh_Distance_BA_R0_R2	21,12893867	-79,86613464	-939,3668823	-0,025754
Cloud_Mesh_Distance_BA_R0_R2	21,20579147	-79,69891357	-939,4208984	-0,007231
Cloud_Mesh_Distance_BA_R0_R2	21,34634399	-79,73936462	-939,4602661	-0,005989
Cloud_Mesh_Distance_BA_R0_R2	21,28386116	-79,88343811	-939,4147339	-0,022428
Cloud_Mesh_Distance_BA_R0_R2	21,42592621	-79,96629333	-939,4567871	-0,028105
Cloud_Mesh_Distance_BA_R0_R2	18,06456757	-79,24009705	-939,3626709	-0,070495
Cloud_Mesh_Distance_BA_R0_R2	18,29241371	-79,068573	-939,3310547	-0,070989
Cloud_Mesh_Distance_BA_R0_R2	20,30102921	-79,25236511	-939,3047485	-0,04573
Cloud_Mesh_Distance_BA_R0_R2	21,39510155	-79,56770325	-939,520752	0,00532
Cloud_Mesh_Distance_BA_R0_R2	18,16573334	-78,89561462	-939,4695435	-0,049742
Cloud_Mesh_Distance_BA_R0_R2	17,94804382	-79,12283325	-939,4804688	-0,059023
Cloud_Mesh_Distance_BA_R0_R2	18,03419495	-78,95744324	-939,5083008	-0,047828
Cloud_Mesh_Distance_BA_R0_R2	18,13462639	-79,02798462	-939,4230347	-0,059977
Cloud_Mesh_Distance_BA_R0_R2	18,35222244	-79,1572876	-939,2658691	-0,073796
Cloud_Mesh_Distance_BA_R0_R2	18,59724808	-79,01327515	-939,2503662	-0,074295
Cloud_Mesh_Distance_BA_R0_R2	18,73091125	-78,86851501	-939,2841187	-0,073793
Cloud_Mesh_Distance_BA_R0_R2	20,13006401	-79,19590759	-939,2802734	-0,057811
Cloud_Mesh_Distance_BA_R0_R2	20,27206993	-79,09474182	-939,3557129	-0,050536
Cloud_Mesh_Distance_BA_R0_R2	20,55142593	-79,37101746	-939,3275757	-0,029227
Cloud_Mesh_Distance_BA_R0_R2	20,45534706	-79,30805969	-939,3244629	-0,034581
Cloud_Mesh_Distance_BA_R0_R2	20,63648987	-79,24740601	-939,3933106	-0,021399
Cloud_Mesh_Distance_BA_R0_R2	20,44792557	-79,1615448	-939,37677	-0,03617
Cloud_Mesh_Distance_BA_R0_R2	20,81601715	-79,21463013	-939,4533081	-0,007115
Cloud_Mesh_Distance_BA_R0_R2	20,81473541	-79,36973572	-939,3967285	-0,00929
Cloud_Mesh_Distance_BA_R0_R2	21,14988327	-79,51321411	-939,4489746	0,001594
Cloud_Mesh_Distance_BA_R0_R2	20,99056244	-79,45487976	-939,418457	-0,002101
Cloud_Mesh_Distance_BA_R0_R2	21,25496292	-79,40690613	-939,5189819	0,014582
Cloud_Mesh_Distance_BA_R0_R2	21,29036713	-79,54742432	-939,4874268	0,005036
Cloud_Mesh_Distance_BA_R0_R2	18,31957817	-78,87161255	-939,4115601	-0,05606
Cloud_Mesh_Distance_BA_R0_R2	18,53290939	-78,70294189	-939,4170532	-0,052157
Cloud_Mesh_Distance_BA_R0_R2	18,51883698	-78,89941406	-939,3265381	-0,069248
Cloud_Mesh_Distance_BA_R0_R2	18,86200905	-78,88078308	-939,255249	-0,074312
Cloud_Mesh_Distance_BA_R0_R2	18,93240356	-78,7399292	-939,3126221	-0,069091
Cloud_Mesh_Distance_BA_R0_R2	19,01413345	-78,84828186	-939,2526245	-0,076616
Cloud_Mesh_Distance_BA_R0_R2	19,93396759	-78,9044342	-939,3388672	-0,068829
Cloud_Mesh_Distance_BA_R0_R2	19,82178307	-78,96754456	-939,2853394	-0,070996
Cloud_Mesh_Distance_BA_R0_R2	20,07801819	-79,02177429	-939,3308716	-0,062546
Cloud_Mesh_Distance_BA_R0_R2	20,24671555	-78,93467712	-939,4146118	-0,059185

Cloud_Mesh_Distance_BA_R0_R2	18,59261894	-78,55908203	-939,4736328	-0,040562
Cloud_Mesh_Distance_BA_R0_R2	18,93139648	-78,57939148	-939,3933106	-0,059344
Cloud_Mesh_Distance_BA_R0_R2	18,73211861	-78,72013855	-939,3563843	-0,063115
Cloud_Mesh_Distance_BA_R0_R2	19,11644745	-78,59384155	-939,3698731	-0,066704
Cloud_Mesh_Distance_BA_R0_R2	19,30526543	-78,62376404	-939,3539429	-0,072131
Cloud_Mesh_Distance_BA_R0_R2	19,13436699	-78,72137451	-939,3049316	-0,069709
Cloud_Mesh_Distance_BA_R0_R2	19,25806999	-78,7387085	-939,2971191	-0,072847
Cloud_Mesh_Distance_BA_R0_R2	19,36159134	-78,71801758	-939,3115234	-0,071882
Cloud_Mesh_Distance_BA_R0_R2	19,78299332	-78,8474884	-939,3231812	-0,07108
Cloud_Mesh_Distance_BA_R0_R2	20,08991432	-78,72244263	-939,4624634	-0,06958
Cloud_Mesh_Distance_BA_R0_R2	20,2275219	-78,78433228	-939,4762573	-0,063646
Cloud_Mesh_Distance_BA_R0_R2	20,08232117	-78,84844971	-939,4038696	-0,068116
Cloud_Mesh_Distance_BA_R0_R2	20,39423752	-78,85218811	-939,4944458	-0,051824
Cloud_Mesh_Distance_BA_R0_R2	20,59237289	-79,0690155	-939,4530029	-0,030161
Cloud_Mesh_Distance_BA_R0_R2	20,4211235	-79,00480652	-939,4337158	-0,045998
Cloud_Mesh_Distance_BA_R0_R2	20,74785423	-79,10951233	-939,4771118	-0,014338
Cloud_Mesh_Distance_BA_R0_R2	20,72435379	-78,98332214	-939,5270386	-0,016972
Cloud_Mesh_Distance_BA_R0_R2	18,68227386	-78,44741821	-939,5109253	-0,032727
Cloud_Mesh_Distance_BA_R0_R2	18,74995804	-78,54714966	-939,4412842	-0,050094
Cloud_Mesh_Distance_BA_R0_R2	19,09366226	-78,47349548	-939,4345703	-0,058045
Cloud_Mesh_Distance_BA_R0_R2	18,95748711	-78,42242432	-939,4755859	-0,048539
Cloud_Mesh_Distance_BA_R0_R2	19,58972549	-78,66990662	-939,3615112	-0,073308
Cloud_Mesh_Distance_BA_R0_R2	19,44979477	-78,65458679	-939,3494263	-0,070088
Cloud_Mesh_Distance_BA_R0_R2	19,9143219	-78,72320557	-939,411377	-0,072689
Cloud_Mesh_Distance_BA_R0_R2	19,74299622	-78,57536316	-939,437561	-0,070557
Cloud_Mesh_Distance_BA_R0_R2	19,98841858	-78,49803162	-939,538208	-0,073277
Cloud_Mesh_Distance_BA_R0_R2	19,90843964	-78,56938171	-939,480896	-0,072918
Cloud_Mesh_Distance_BA_R0_R2	18,90790367	-78,33195496	-939,5338135	-0,035915
Cloud_Mesh_Distance_BA_R0_R2	19,02641487	-78,2936554	-939,5436401	-0,041386
Cloud_Mesh_Distance_BA_R0_R2	19,28870773	-78,45462036	-939,4420166	-0,063939
Cloud_Mesh_Distance_BA_R0_R2	19,50764847	-78,5267334	-939,4205322	-0,068746
Cloud_Mesh_Distance_BA_R0_R2	19,4824791	-78,35038757	-939,5120239	-0,061208
Cloud_Mesh_Distance_BA_R0_R2	19,67767715	-78,39660645	-939,5145264	-0,068506
Cloud_Mesh_Distance_BA_R0_R2	19,16474915	-78,3343811	-939,5109863	-0,051293
Cloud_Mesh_Distance_BA_R0_R2	18,1692524	-81,91665649	-939,2159424	-0,107366
Cloud_Mesh_Distance_BA_R0_R2	18,28395844	-81,82821655	-939,1571045	-0,109146
Cloud_Mesh_Distance_BA_R0_R2	18,74959564	-81,9362793	-939,1299439	-0,131163
Cloud_Mesh_Distance_BA_R0_R2	18,86172104	-81,86991882	-939,1056519	-0,125842
Cloud_Mesh_Distance_BA_R0_R2	19,4017334	-82,05015564	-939,1325073	-0,091529
Cloud_Mesh_Distance_BA_R0_R2	19,74939346	-82,06822205	-939,1652222	-0,065093
Cloud_Mesh_Distance_BA_R0_R2	17,92669487	-81,7437439	-939,2416382	-0,082062
Cloud_Mesh_Distance_BA_R0_R2	18,03286171	-81,68141174	-939,1782837	-0,083691
Cloud_Mesh_Distance_BA_R0_R2	18,1664238	-81,75747681	-939,161499	-0,094845
Cloud_Mesh_Distance_BA_R0_R2	18,57405281	-81,84632874	-939,12146	-0,124573
Cloud_Mesh_Distance_BA_R0_R2	18,43260193	-81,82868958	-939,133606	-0,118862
Cloud_Mesh_Distance_BA_R0_R2	18,64113235	-81,92765808	-939,1373291	-0,129138
Cloud_Mesh_Distance_BA_R0_R2	18,95759964	-81,71000671	-939,0682373	-0,120291
Cloud_Mesh_Distance_BA_R0_R2	18,73201561	-81,80519104	-939,098938	-0,126172
Cloud_Mesh_Distance_BA_R0_R2	18,78333664	-81,63993835	-939,0621948	-0,118919
Cloud_Mesh_Distance_BA_R0_R2	19,21177292	-81,74728394	-939,0703735	-0,104617

Cloud_Mesh_Distance_BA_R0_R2	19,01906013	-81,88702393	-939,1014404	-0,119637
Cloud_Mesh_Distance_BA_R0_R2	19,36688614	-81,87976074	-939,0958252	-0,090575
Cloud_Mesh_Distance_BA_R0_R2	19,5596447	-81,76347351	-939,085022	-0,075388
Cloud_Mesh_Distance_BA_R0_R2	19,72076035	-81,94400024	-939,1330566	-0,06341
Cloud_Mesh_Distance_BA_R0_R2	19,56058884	-81,91995239	-939,1125488	-0,075575
Cloud_Mesh_Distance_BA_R0_R2	19,87257385	-81,84367371	-939,1383057	-0,056222
Cloud_Mesh_Distance_BA_R0_R2	19,71952057	-81,80369568	-939,1081543	-0,064013
Cloud_Mesh_Distance_BA_R0_R2	19,9436264	-81,94143677	-939,1716919	-0,053393
Cloud_Mesh_Distance_BA_R0_R2	18,1760273	-81,59753418	-939,118042	-0,091431
Cloud_Mesh_Distance_BA_R0_R2	18,19011688	-81,42381287	-939,0819092	-0,089508
Cloud_Mesh_Distance_BA_R0_R2	18,0315094	-81,52442932	-939,1408081	-0,07798
Cloud_Mesh_Distance_BA_R0_R2	18,58361435	-81,68623352	-939,0829468	-0,119712
Cloud_Mesh_Distance_BA_R0_R2	18,35003471	-81,67131042	-939,1040039	-0,103604
Cloud_Mesh_Distance_BA_R0_R2	18,32615089	-81,49963379	-939,0722656	-0,097738
Cloud_Mesh_Distance_BA_R0_R2	18,94028473	-81,46656799	-939,0304565	-0,121217
Cloud_Mesh_Distance_BA_R0_R2	19,41732788	-81,71330261	-939,0690918	-0,086716
Cloud_Mesh_Distance_BA_R0_R2	19,38008118	-81,56440735	-939,0466919	-0,093522
Cloud_Mesh_Distance_BA_R0_R2	19,8710556	-81,68850708	-939,1138916	-0,059182
Cloud_Mesh_Distance_BA_R0_R2	19,72141647	-81,64950562	-939,0855713	-0,066905
Cloud_Mesh_Distance_BA_R0_R2	18,02457237	-81,15748596	-939,0904541	-0,080183
Cloud_Mesh_Distance_BA_R0_R2	18,03079224	-81,34750366	-939,1109009	-0,075687
Cloud_Mesh_Distance_BA_R0_R2	18,4133091	-81,31686401	-939,0355835	-0,107148
Cloud_Mesh_Distance_BA_R0_R2	18,20014191	-81,23043823	-939,0543213	-0,094195
Cloud_Mesh_Distance_BA_R0_R2	18,56129456	-81,33387756	-939,0264893	-0,118212
Cloud_Mesh_Distance_BA_R0_R2	18,75946617	-81,3249054	-939,0165405	-0,122999
Cloud_Mesh_Distance_BA_R0_R2	18,71256256	-81,51533508	-939,0444946	-0,11821
Cloud_Mesh_Distance_BA_R0_R2	18,52581215	-81,47268677	-939,0487671	-0,117336
Cloud_Mesh_Distance_BA_R0_R2	19,03702545	-81,29399109	-939,0073853	-0,118379
Cloud_Mesh_Distance_BA_R0_R2	18,94375038	-81,3217926	-939,0115356	-0,121902
Cloud_Mesh_Distance_BA_R0_R2	19,12763214	-81,22865295	-939,0002441	-0,116877
Cloud_Mesh_Distance_BA_R0_R2	19,15613174	-81,34359741	-939,0135498	-0,114055
Cloud_Mesh_Distance_BA_R0_R2	19,16717911	-81,53527832	-939,0379639	-0,108455
Cloud_Mesh_Distance_BA_R0_R2	19,73849106	-81,47732544	-939,0673218	-0,072346
Cloud_Mesh_Distance_BA_R0_R2	19,56432152	-81,59880066	-939,0628052	-0,079487
Cloud_Mesh_Distance_BA_R0_R2	19,5598793	-81,4090271	-939,0402832	-0,085356
Cloud_Mesh_Distance_BA_R0_R2	20,00998688	-81,72113037	-939,1463623	-0,053916
Cloud_Mesh_Distance_BA_R0_R2	20,16047859	-81,62309265	-939,1685791	-0,054545
Cloud_Mesh_Distance_BA_R0_R2	20,16812706	-81,50260925	-939,1549683	-0,059316
Cloud_Mesh_Distance_BA_R0_R2	20,0260334	-81,55151367	-939,1271362	-0,059017
Cloud_Mesh_Distance_BA_R0_R2	18,18875122	-81,014328	-939,0367432	-0,095678
Cloud_Mesh_Distance_BA_R0_R2	18,3876667	-81,10003662	-939,0131226	-0,110381
Cloud_Mesh_Distance_BA_R0_R2	18,94643974	-81,22767639	-939,0012817	-0,123934
Cloud_Mesh_Distance_BA_R0_R2	19,29411888	-81,19110107	-938,9995728	-0,106571
Cloud_Mesh_Distance_BA_R0_R2	19,3532486	-81,36888123	-939,0217285	-0,099827
Cloud_Mesh_Distance_BA_R0_R2	19,7611599	-81,26618958	-939,0491943	-0,080561
Cloud_Mesh_Distance_BA_R0_R2	19,86317062	-81,55012512	-939,0949097	-0,063989
Cloud_Mesh_Distance_BA_R0_R2	19,94896317	-81,37313843	-939,0921631	-0,068542
Cloud_Mesh_Distance_BA_R0_R2	20,14674759	-81,39189148	-939,1374512	-0,066114
Cloud_Mesh_Distance_BA_R0_R2	17,87743187	-81,08813477	-939,1401367	-0,078626
Cloud_Mesh_Distance_BA_R0_R2	18,00098419	-80,97436523	-939,0859985	-0,085872

Cloud_Mesh_Distance_BA_R0_R2	18,37156105	-80,91873169	-938,9995117	-0,109231
Cloud_Mesh_Distance_BA_R0_R2	18,25276184	-80,85932922	-939,0145874	-0,100742
Cloud_Mesh_Distance_BA_R0_R2	18,12024307	-80,83958435	-939,0455933	-0,09493
Cloud_Mesh_Distance_BA_R0_R2	18,63238716	-81,1481781	-939	-0,123034
Cloud_Mesh_Distance_BA_R0_R2	18,8650322	-80,96069336	-938,9758301	-0,128798
Cloud_Mesh_Distance_BA_R0_R2	19,05815506	-81,01820374	-938,979126	-0,123282
Cloud_Mesh_Distance_BA_R0_R2	18,87781715	-81,1346283	-938,9916382	-0,126397
Cloud_Mesh_Distance_BA_R0_R2	19,14725685	-81,14491272	-938,9914551	-0,116174
Cloud_Mesh_Distance_BA_R0_R2	19,66185188	-81,00190735	-939,0078735	-0,087752
Cloud_Mesh_Distance_BA_R0_R2	19,55700684	-80,98823547	-938,9954834	-0,091761
Cloud_Mesh_Distance_BA_R0_R2	19,54850388	-81,16213989	-939,0125732	-0,091715
Cloud_Mesh_Distance_BA_R0_R2	20,12629318	-81,21762085	-939,1156616	-0,074625
Cloud_Mesh_Distance_BA_R0_R2	20,41606522	-81,27279663	-939,1919556	-0,065371
Cloud_Mesh_Distance_BA_R0_R2	20,49677658	-81,19836426	-939,2052002	-0,067445
Cloud_Mesh_Distance_BA_R0_R2	17,94640732	-80,79182434	-939,1019287	-0,091318
Cloud_Mesh_Distance_BA_R0_R2	18,11948013	-80,64587402	-939,045166	-0,10095
Cloud_Mesh_Distance_BA_R0_R2	18,91680527	-80,81086731	-938,963562	-0,130915
Cloud_Mesh_Distance_BA_R0_R2	19,51716995	-80,89413452	-938,9836426	-0,094324
Cloud_Mesh_Distance_BA_R0_R2	19,21979332	-81,06385803	-938,9849854	-0,112651
Cloud_Mesh_Distance_BA_R0_R2	19,40386581	-81,00520325	-938,9866943	-0,102983
Cloud_Mesh_Distance_BA_R0_R2	20,03189087	-80,89604187	-939,0630493	-0,080376
Cloud_Mesh_Distance_BA_R0_R2	19,9693985	-81,1390686	-939,0736694	-0,07909
Cloud_Mesh_Distance_BA_R0_R2	19,7990303	-81,04310608	-939,0323486	-0,083243
Cloud_Mesh_Distance_BA_R0_R2	19,85920715	-80,88981628	-939,026062	-0,079814
Cloud_Mesh_Distance_BA_R0_R2	20,15093613	-81,03329468	-939,1038208	-0,081362
Cloud_Mesh_Distance_BA_R0_R2	20,40516853	-81,0014801	-939,1638794	-0,079926
Cloud_Mesh_Distance_BA_R0_R2	20,47765923	-81,06687927	-939,1882324	-0,075316
Cloud_Mesh_Distance_BA_R0_R2	20,57221222	-80,99572754	-939,2073364	-0,076028
Cloud_Mesh_Distance_BA_R0_R2	20,6672802	-81,1131134	-939,2458496	-0,0685
Cloud_Mesh_Distance_BA_R0_R2	20,56846237	-81,11071777	-939,21698	-0,070414
Cloud_Mesh_Distance_BA_R0_R2	20,66885948	-81,02224731	-939,2374268	-0,072579
Cloud_Mesh_Distance_BA_R0_R2	17,93323326	-80,56396484	-939,1136475	-0,093509
Cloud_Mesh_Distance_BA_R0_R2	17,7786808	-80,55633545	-939,1855469	-0,089987
Cloud_Mesh_Distance_BA_R0_R2	17,83359528	-80,38609314	-939,1719971	-0,095138
Cloud_Mesh_Distance_BA_R0_R2	18,26443481	-80,62413025	-939,0081787	-0,105963
Cloud_Mesh_Distance_BA_R0_R2	18,23995399	-80,73826599	-939,0142212	-0,100945
Cloud_Mesh_Distance_BA_R0_R2	18,09611702	-80,47198486	-939,0584717	-0,104329
Cloud_Mesh_Distance_BA_R0_R2	18,26664925	-80,47926331	-939,0113525	-0,110382
Cloud_Mesh_Distance_BA_R0_R2	19,06221199	-80,82743835	-938,9644775	-0,123579
Cloud_Mesh_Distance_BA_R0_R2	19,00232697	-80,71931458	-938,9572754	-0,126588
Cloud_Mesh_Distance_BA_R0_R2	19,11315918	-80,68751526	-938,9560547	-0,121184
Cloud_Mesh_Distance_BA_R0_R2	19,38006592	-80,72824097	-938,9638672	-0,104882
Cloud_Mesh_Distance_BA_R0_R2	19,2542572	-80,88653564	-938,9707031	-0,113618
Cloud_Mesh_Distance_BA_R0_R2	19,42237854	-80,85194397	-938,9748535	-0,101944
Cloud_Mesh_Distance_BA_R0_R2	19,97317886	-80,59516907	-939,0233154	-0,069574
Cloud_Mesh_Distance_BA_R0_R2	20,0880661	-80,67253113	-939,0548096	-0,072783
Cloud_Mesh_Distance_BA_R0_R2	20,20993423	-80,88719177	-939,1043701	-0,082083
Cloud_Mesh_Distance_BA_R0_R2	20,32266045	-80,8273468	-939,1289063	-0,084253
Cloud_Mesh_Distance_BA_R0_R2	20,55528259	-80,90031433	-939,1951904	-0,079781
Cloud_Mesh_Distance_BA_R0_R2	20,68025398	-80,9052887	-939,2306519	-0,076594

Cloud_Mesh_Distance_BA_R0_R2	17,67899513	-80,47224426	-939,248169	-0,085242
Cloud_Mesh_Distance_BA_R0_R2	17,69837761	-80,29823303	-939,2559815	-0,08294
Cloud_Mesh_Distance_BA_R0_R2	17,99883842	-80,38250732	-939,1000977	-0,100507
Cloud_Mesh_Distance_BA_R0_R2	18,32002068	-80,34095764	-939,0059204	-0,109835
Cloud_Mesh_Distance_BA_R0_R2	18,39884377	-80,48374939	-938,9857788	-0,114151
Cloud_Mesh_Distance_BA_R0_R2	19,22514725	-80,7217865	-938,9588013	-0,115769
Cloud_Mesh_Distance_BA_R0_R2	19,21114731	-80,60484314	-938,9533691	-0,118025
Cloud_Mesh_Distance_BA_R0_R2	20,09651375	-80,53361511	-939,0477295	-0,068147
Cloud_Mesh_Distance_BA_R0_R2	20,40096664	-80,72485352	-939,1409302	-0,081875
Cloud_Mesh_Distance_BA_R0_R2	20,24848938	-80,72621155	-939,1005249	-0,07938
Cloud_Mesh_Distance_BA_R0_R2	20,60190773	-80,53300476	-939,1853027	-0,077762
Cloud_Mesh_Distance_BA_R0_R2	17,97296715	-80,20584106	-939,12854	-0,097884
Cloud_Mesh_Distance_BA_R0_R2	18,34369278	-80,23841858	-939,0065308	-0,110491
Cloud_Mesh_Distance_BA_R0_R2	18,18306541	-80,28410339	-939,045044	-0,106901
Cloud_Mesh_Distance_BA_R0_R2	18,51335144	-80,0907135	-938,9838257	-0,113769
Cloud_Mesh_Distance_BA_R0_R2	18,63103104	-80,04862976	-938,9708862	-0,114544
Cloud_Mesh_Distance_BA_R0_R2	18,45668793	-80,19862366	-938,9873657	-0,114451
Cloud_Mesh_Distance_BA_R0_R2	18,59087753	-80,17301941	-938,968811	-0,119824
Cloud_Mesh_Distance_BA_R0_R2	18,70617104	-80,12173462	-938,9599609	-0,119175
Cloud_Mesh_Distance_BA_R0_R2	19,71223831	-80,25213623	-938,9821777	-0,076168
Cloud_Mesh_Distance_BA_R0_R2	20,24269485	-80,51177979	-939,0838623	-0,069648
Cloud_Mesh_Distance_BA_R0_R2	20,43733978	-80,55274963	-939,140564	-0,077711
Cloud_Mesh_Distance_BA_R0_R2	20,60216522	-80,38658142	-939,1825562	-0,071446
Cloud_Mesh_Distance_BA_R0_R2	17,89015579	-79,90267944	-939,22229	-0,083192
Cloud_Mesh_Distance_BA_R0_R2	17,8562851	-80,08352661	-939,2023926	-0,089429
Cloud_Mesh_Distance_BA_R0_R2	18,14498138	-80,08802795	-939,0770264	-0,097039
Cloud_Mesh_Distance_BA_R0_R2	18,02008057	-79,99479675	-939,1412354	-0,094266
Cloud_Mesh_Distance_BA_R0_R2	18,36085892	-79,89631653	-939,0352173	-0,097127
Cloud_Mesh_Distance_BA_R0_R2	18,22073746	-79,89692688	-939,079834	-0,093597
Cloud_Mesh_Distance_BA_R0_R2	18,43761635	-79,92277527	-939,0126343	-0,099566
Cloud_Mesh_Distance_BA_R0_R2	18,35232544	-80,06373596	-939,0184937	-0,105559
Cloud_Mesh_Distance_BA_R0_R2	18,74583435	-80,02305603	-938,9622803	-0,116665
Cloud_Mesh_Distance_BA_R0_R2	19,1305542	-79,9773407	-938,9664917	-0,12042
Cloud_Mesh_Distance_BA_R0_R2	19,21627045	-79,9119873	-938,977417	-0,117953
Cloud_Mesh_Distance_BA_R0_R2	19,74325943	-80,2297821	-938,9864502	-0,073842
Cloud_Mesh_Distance_BA_R0_R2	20,08419228	-80,01403809	-939,0668945	-0,053173
Cloud_Mesh_Distance_BA_R0_R2	20,19586945	-80,07215881	-939,085022	-0,050717
Cloud_Mesh_Distance_BA_R0_R2	20,28073311	-80,25202942	-939,0929565	-0,058422
Cloud_Mesh_Distance_BA_R0_R2	20,41433144	-80,30751038	-939,1289063	-0,06467
Cloud_Mesh_Distance_BA_R0_R2	20,50281143	-80,10803223	-939,1637573	-0,053508
Cloud_Mesh_Distance_BA_R0_R2	20,77661133	-80,2702179	-939,2330322	-0,064412
Cloud_Mesh_Distance_BA_R0_R2	18,06836891	-79,79826355	-939,161438	-0,085643
Cloud_Mesh_Distance_BA_R0_R2	18,36253357	-79,78013611	-939,0545654	-0,088784
Cloud_Mesh_Distance_BA_R0_R2	18,5307579	-79,96536255	-938,9902954	-0,105519
Cloud_Mesh_Distance_BA_R0_R2	18,58620453	-79,85630798	-938,9929199	-0,098105
Cloud_Mesh_Distance_BA_R0_R2	19,12320137	-79,85243225	-938,9771729	-0,114738
Cloud_Mesh_Distance_BA_R0_R2	19,4011898	-79,80476379	-939,0044556	-0,106584
Cloud_Mesh_Distance_BA_R0_R2	19,31907845	-79,90939331	-938,984375	-0,113473
Cloud_Mesh_Distance_BA_R0_R2	20,35579681	-79,91127014	-939,1489258	-0,047688
Cloud_Mesh_Distance_BA_R0_R2	20,24996376	-79,92381287	-939,119751	-0,050754

Cloud_Mesh_Distance_BA_R0_R2	20,34354973	-79,96757507	-939,1375122	-0,051598
Cloud_Mesh_Distance_BA_R0_R2	20,3405056	-80,08485413	-939,1221314	-0,051765
Cloud_Mesh_Distance_BA_R0_R2	20,62682724	-80,192276	-939,1929932	-0,058876
Cloud_Mesh_Distance_BA_R0_R2	18,10331345	-79,61683655	-939,196167	-0,081517
Cloud_Mesh_Distance_BA_R0_R2	18,10413551	-79,48973083	-939,2390137	-0,077797
Cloud_Mesh_Distance_BA_R0_R2	18,27025986	-79,67198181	-939,1107788	-0,088747
Cloud_Mesh_Distance_BA_R0_R2	18,47478867	-79,50645447	-939,0913086	-0,082391
Cloud_Mesh_Distance_BA_R0_R2	18,717453	-79,55403137	-939,0281982	-0,086248
Cloud_Mesh_Distance_BA_R0_R2	19,26902008	-79,78526306	-938,9954834	-0,113592
Cloud_Mesh_Distance_BA_R0_R2	19,42776489	-79,62443542	-939,0343628	-0,097392
Cloud_Mesh_Distance_BA_R0_R2	19,55687141	-79,72901917	-939,0319824	-0,094164
Cloud_Mesh_Distance_BA_R0_R2	19,81486893	-79,74026489	-939,0639038	-0,07243
Cloud_Mesh_Distance_BA_R0_R2	20,19022369	-79,69242859	-939,1502075	-0,048757
Cloud_Mesh_Distance_BA_R0_R2	20,01366043	-79,70336914	-939,1087036	-0,057799
Cloud_Mesh_Distance_BA_R0_R2	20,06477737	-79,84848022	-939,0900269	-0,054228
Cloud_Mesh_Distance_BA_R0_R2	20,40263748	-79,80427551	-939,1796265	-0,042709
Cloud_Mesh_Distance_BA_R0_R2	20,39966202	-79,91830444	-939,1593018	-0,047353
Cloud_Mesh_Distance_BA_R0_R2	20,46981049	-79,95822144	-939,1713867	-0,048396
Cloud_Mesh_Distance_BA_R0_R2	20,64947701	-79,99092102	-939,2155762	-0,047742
Cloud_Mesh_Distance_BA_R0_R2	18,16400146	-79,3553009	-939,262146	-0,075944
Cloud_Mesh_Distance_BA_R0_R2	18,26948357	-79,45872498	-939,177063	-0,080445
Cloud_Mesh_Distance_BA_R0_R2	18,47992706	-79,30755615	-939,1591187	-0,079521
Cloud_Mesh_Distance_BA_R0_R2	18,643116	-79,49241638	-939,0559692	-0,081602
Cloud_Mesh_Distance_BA_R0_R2	19,26344299	-79,5206604	-939,0362549	-0,101153
Cloud_Mesh_Distance_BA_R0_R2	19,60305405	-79,39796448	-939,1051025	-0,081038
Cloud_Mesh_Distance_BA_R0_R2	19,64187622	-79,59516907	-939,0679932	-0,086071
Cloud_Mesh_Distance_BA_R0_R2	19,90690994	-79,6730957	-939,093811	-0,065309
Cloud_Mesh_Distance_BA_R0_R2	19,80212021	-79,61988831	-939,0874634	-0,07397
Cloud_Mesh_Distance_BA_R0_R2	19,98443222	-79,5111084	-939,1486816	-0,06231
Cloud_Mesh_Distance_BA_R0_R2	20,38788223	-79,5643158	-939,2298584	-0,038182
Cloud_Mesh_Distance_BA_R0_R2	20,59091187	-79,82322693	-939,2240601	-0,038199
Cloud_Mesh_Distance_BA_R0_R2	20,55962372	-79,64892578	-939,2524414	-0,034408
Cloud_Mesh_Distance_BA_R0_R2	20,72093391	-79,69618225	-939,2833862	-0,029196
Cloud_Mesh_Distance_BA_R0_R2	18,23677254	-79,2208252	-939,286377	-0,075392
Cloud_Mesh_Distance_BA_R0_R2	18,32589531	-79,27577209	-939,225708	-0,076826
Cloud_Mesh_Distance_BA_R0_R2	18,84273529	-79,32579041	-939,0795288	-0,082541
Cloud_Mesh_Distance_BA_R0_R2	18,68069077	-79,33433533	-939,1000977	-0,080464
Cloud_Mesh_Distance_BA_R0_R2	19,12236404	-79,16213989	-939,12323	-0,082234
Cloud_Mesh_Distance_BA_R0_R2	18,98045731	-79,29814148	-939,079834	-0,085294
Cloud_Mesh_Distance_BA_R0_R2	19,31869507	-79,22669983	-939,1132202	-0,085516
Cloud_Mesh_Distance_BA_R0_R2	19,39813614	-79,39666748	-939,0773926	-0,088662
Cloud_Mesh_Distance_BA_R0_R2	19,52833557	-79,25158691	-939,1321411	-0,081796
Cloud_Mesh_Distance_BA_R0_R2	19,61044312	-79,28425598	-939,1362915	-0,079386
Cloud_Mesh_Distance_BA_R0_R2	19,78969193	-79,28979492	-939,1692505	-0,072633
Cloud_Mesh_Distance_BA_R0_R2	19,76464844	-79,47994995	-939,112854	-0,074678
Cloud_Mesh_Distance_BA_R0_R2	20,00532722	-79,29994202	-939,213562	-0,060909
Cloud_Mesh_Distance_BA_R0_R2	20,2124157	-79,50404358	-939,2033691	-0,047624
Cloud_Mesh_Distance_BA_R0_R2	20,16192436	-79,35925293	-939,2332153	-0,052032
Cloud_Mesh_Distance_BA_R0_R2	20,33055496	-79,40072632	-939,262146	-0,041639
Cloud_Mesh_Distance_BA_R0_R2	20,45656395	-79,42463684	-939,2869873	-0,034375

Cloud_Mesh_Distance_BA_R0_R2	20,65102577	-79,39788818	-939,3447266	-0,022978
Cloud_Mesh_Distance_BA_R0_R2	20,55136871	-79,48704529	-939,2922363	-0,028943
Cloud_Mesh_Distance_BA_R0_R2	18,44467163	-79,05953979	-939,2757568	-0,073053
Cloud_Mesh_Distance_BA_R0_R2	18,47616005	-79,15640259	-939,2224731	-0,07988
Cloud_Mesh_Distance_BA_R0_R2	18,74904251	-78,97399902	-939,2318115	-0,076917
Cloud_Mesh_Distance_BA_R0_R2	18,67758369	-79,13059998	-939,1790161	-0,079036
Cloud_Mesh_Distance_BA_R0_R2	18,88272095	-78,99023438	-939,2038574	-0,077189
Cloud_Mesh_Distance_BA_R0_R2	18,89668655	-79,16719055	-939,1304932	-0,081428
Cloud_Mesh_Distance_BA_R0_R2	19,30522346	-79,02571106	-939,1791992	-0,079601
Cloud_Mesh_Distance_BA_R0_R2	19,62065887	-79,23805237	-939,1516113	-0,077707
Cloud_Mesh_Distance_BA_R0_R2	19,6723938	-79,24586487	-939,1588135	-0,075452
Cloud_Mesh_Distance_BA_R0_R2	19,91887093	-79,026474	-939,286499	-0,06737
Cloud_Mesh_Distance_BA_R0_R2	19,95493698	-79,152771	-939,2504273	-0,065521
Cloud_Mesh_Distance_BA_R0_R2	19,16545105	-78,8180542	-939,2598267	-0,073309
Cloud_Mesh_Distance_BA_R0_R2	19,10027885	-78,97166443	-939,1950073	-0,077516
Cloud_Mesh_Distance_BA_R0_R2	19,30850792	-78,84156799	-939,2525635	-0,075626
Cloud_Mesh_Distance_BA_R0_R2	19,49317932	-79,08656311	-939,1783447	-0,07685
Cloud_Mesh_Distance_BA_R0_R2	19,4798851	-78,91018677	-939,2407227	-0,072793
Cloud_Mesh_Distance_BA_R0_R2	19,80177307	-79,09346008	-939,2338867	-0,07189
Cloud_Mesh_Distance_BA_R0_R2	19,66656494	-78,97784424	-939,2466431	-0,072505
Cloud_Mesh_Distance_BA_R0_R2	19,65351868	-79,16477966	-939,1793823	-0,073901
Cloud_Mesh_Distance_BA_R0_R2	19,73191261	-78,72148132	-939,366394	-0,071906
Cloud_Mesh_Distance_BA_R0_R2	19,62235832	-78,80949402	-939,3049927	-0,071837
Cloud_Mesh_Distance_BA_R0_R2	19,47118759	-78,76104736	-939,3030396	-0,071619
Cloud_Mesh_Distance_BA_R0_R2	18,54555511	-80,95611572	-938,9864502	-0,119301
Cloud_Mesh_Distance_BA_R0_R2	18,68028831	-80,81861877	-938,9689941	-0,125552
Cloud_Mesh_Distance_BA_R0_R2	18,70363808	-80,94708252	-938,9780273	-0,124983
Cloud_Mesh_Distance_BA_R0_R2	19,0280323	-81,16355896	-938,9932251	-0,121667
Cloud_Mesh_Distance_BA_R0_R2	19,64664268	-80,8742218	-938,9938965	-0,0881
Cloud_Mesh_Distance_BA_R0_R2	18,47849083	-80,63772583	-938,9754028	-0,116698
Cloud_Mesh_Distance_BA_R0_R2	18,34023094	-80,67182922	-938,9939575	-0,109381
Cloud_Mesh_Distance_BA_R0_R2	18,36306763	-80,77281189	-938,9928589	-0,112186
Cloud_Mesh_Distance_BA_R0_R2	18,51534653	-80,79536438	-938,9775391	-0,117077
Cloud_Mesh_Distance_BA_R0_R2	18,66991615	-80,65530396	-938,9607544	-0,127396
Cloud_Mesh_Distance_BA_R0_R2	18,80287361	-80,84802246	-938,9678345	-0,127226
Cloud_Mesh_Distance_BA_R0_R2	18,85632324	-80,70497131	-938,9572754	-0,128864
Cloud_Mesh_Distance_BA_R0_R2	19,55064774	-80,74905396	-938,9744263	-0,091344
Cloud_Mesh_Distance_BA_R0_R2	19,75047112	-80,92597961	-939,0122681	-0,083025
Cloud_Mesh_Distance_BA_R0_R2	18,47274208	-80,52038574	-938,9738159	-0,117518
Cloud_Mesh_Distance_BA_R0_R2	18,36862183	-80,57226563	-938,989563	-0,111299
Cloud_Mesh_Distance_BA_R0_R2	19,69949532	-80,73983765	-938,9891968	-0,082473
Cloud_Mesh_Distance_BA_R0_R2	19,68624878	-80,59507751	-938,9784546	-0,080087
Cloud_Mesh_Distance_BA_R0_R2	19,78506851	-80,81053162	-939,0061646	-0,080457
Cloud_Mesh_Distance_BA_R0_R2	19,92750168	-80,75679016	-939,0261841	-0,076434
Cloud_Mesh_Distance_BA_R0_R2	18,40152359	-80,39561462	-938,9883423	-0,113168
Cloud_Mesh_Distance_BA_R0_R2	18,41005707	-80,30006409	-938,9900513	-0,111945
Cloud_Mesh_Distance_BA_R0_R2	18,48695755	-80,43753052	-938,9731445	-0,119044
Cloud_Mesh_Distance_BA_R0_R2	18,86339188	-80,50953674	-938,9499512	-0,132482
Cloud_Mesh_Distance_BA_R0_R2	18,69808769	-80,24841309	-938,9563599	-0,123901
Cloud_Mesh_Distance_BA_R0_R2	18,63336945	-80,45606995	-938,9590454	-0,123156

Cloud_Mesh_Distance_BA_R0_R2	19,06514359	-80,5740509	-938,9509277	-0,124054
Cloud_Mesh_Distance_BA_R0_R2	19,08512878	-80,35643005	-938,9479981	-0,12591
Cloud_Mesh_Distance_BA_R0_R2	19,32755661	-80,60943604	-938,9559326	-0,108704
Cloud_Mesh_Distance_BA_R0_R2	19,4948082	-80,55929565	-938,9615479	-0,094987
Cloud_Mesh_Distance_BA_R0_R2	19,67181778	-80,38870239	-938,9725952	-0,077224
Cloud_Mesh_Distance_BA_R0_R2	19,51481247	-80,40974426	-938,9606934	-0,092858
Cloud_Mesh_Distance_BA_R0_R2	19,82430649	-80,66291809	-939,0009766	-0,0741
Cloud_Mesh_Distance_BA_R0_R2	19,86663055	-80,46929932	-938,9991455	-0,068297
Cloud_Mesh_Distance_BA_R0_R2	18,5265255	-80,31794739	-938,9711304	-0,119763
Cloud_Mesh_Distance_BA_R0_R2	18,84985352	-80,1030426	-938,9539185	-0,121612
Cloud_Mesh_Distance_BA_R0_R2	18,86831284	-80,30285645	-938,9489746	-0,128387
Cloud_Mesh_Distance_BA_R0_R2	19,07068253	-80,12969971	-938,9547119	-0,125638
Cloud_Mesh_Distance_BA_R0_R2	19,31264496	-80,1738739	-938,9608154	-0,117155
Cloud_Mesh_Distance_BA_R0_R2	19,56196213	-80,25376892	-938,96875	-0,09247
Cloud_Mesh_Distance_BA_R0_R2	19,32305908	-80,44160461	-938,9519043	-0,110133
Cloud_Mesh_Distance_BA_R0_R2	19,67608452	-80,27828979	-938,9767456	-0,078436
Cloud_Mesh_Distance_BA_R0_R2	19,72761917	-80,2819519	-938,9819946	-0,073506
Cloud_Mesh_Distance_BA_R0_R2	19,82523346	-80,27418518	-938,9952393	-0,067135
Cloud_Mesh_Distance_BA_R0_R2	20,02651215	-80,46418762	-939,0285034	-0,062401
Cloud_Mesh_Distance_BA_R0_R2	20,25612259	-80,3603363	-939,0840454	-0,061172
Cloud_Mesh_Distance_BA_R0_R2	20,14378548	-80,41444397	-939,0546875	-0,06013
Cloud_Mesh_Distance_BA_R0_R2	18,68611145	-79,91011047	-938,9752808	-0,107747
Cloud_Mesh_Distance_BA_R0_R2	18,84252167	-79,92204285	-938,96521	-0,112021
Cloud_Mesh_Distance_BA_R0_R2	18,99976349	-79,95652771	-938,963501	-0,121283
Cloud_Mesh_Distance_BA_R0_R2	19,236763	-80,02072144	-938,9678955	-0,120923
Cloud_Mesh_Distance_BA_R0_R2	19,69193268	-80,22593689	-938,9820557	-0,079566
Cloud_Mesh_Distance_BA_R0_R2	19,53535461	-80,00335693	-938,9892578	-0,097688
Cloud_Mesh_Distance_BA_R0_R2	19,95672607	-80,13555908	-939,0268555	-0,058504
Cloud_Mesh_Distance_BA_R0_R2	20,09719849	-80,14337158	-939,0550537	-0,053263
Cloud_Mesh_Distance_BA_R0_R2	20,18562317	-80,30574036	-939,0663452	-0,058277
Cloud_Mesh_Distance_BA_R0_R2	20,19303894	-80,19407654	-939,0735474	-0,05453
Cloud_Mesh_Distance_BA_R0_R2	20,04027176	-80,30064392	-939,0322266	-0,057932
Cloud_Mesh_Distance_BA_R0_R2	18,46913528	-79,73674011	-939,0336914	-0,088794
Cloud_Mesh_Distance_BA_R0_R2	18,50702667	-79,86895752	-939,0043945	-0,097905
Cloud_Mesh_Distance_BA_R0_R2	18,44434166	-79,82872009	-939,024353	-0,094096
Cloud_Mesh_Distance_BA_R0_R2	18,54105759	-79,7805481	-939,0105591	-0,093933
Cloud_Mesh_Distance_BA_R0_R2	18,97169113	-79,74081421	-938,9840088	-0,10873
Cloud_Mesh_Distance_BA_R0_R2	19,14829826	-79,72953796	-938,9934692	-0,109061
Cloud_Mesh_Distance_BA_R0_R2	19,42413902	-79,91401672	-938,9920044	-0,108384
Cloud_Mesh_Distance_BA_R0_R2	19,36609268	-80,00611877	-938,9780273	-0,11322
Cloud_Mesh_Distance_BA_R0_R2	19,49908257	-79,83995056	-939,0083618	-0,101455
Cloud_Mesh_Distance_BA_R0_R2	19,92285538	-79,96556091	-939,0421753	-0,062915
Cloud_Mesh_Distance_BA_R0_R2	19,75099945	-80,12020874	-938,9974976	-0,072362
Cloud_Mesh_Distance_BA_R0_R2	18,42846298	-79,66496277	-939,0601807	-0,083567
Cloud_Mesh_Distance_BA_R0_R2	18,58190918	-79,64105225	-939,0292358	-0,08607
Cloud_Mesh_Distance_BA_R0_R2	18,73396111	-79,65324402	-939,005249	-0,092429
Cloud_Mesh_Distance_BA_R0_R2	18,86520767	-79,55679321	-939,0155029	-0,091864
Cloud_Mesh_Distance_BA_R0_R2	19,13016129	-79,61112976	-939,0101318	-0,101655
Cloud_Mesh_Distance_BA_R0_R2	19,24727058	-79,65400696	-939,0115356	-0,105585
Cloud_Mesh_Distance_BA_R0_R2	19,47056961	-79,74960327	-939,0192871	-0,100478

Cloud_Mesh_Distance_BA_R0_R2	19,69604111	-79,73268127	-939,0484619	-0,084935
Cloud_Mesh_Distance_BA_R0_R2	19,60784149	-79,83712769	-939,0202637	-0,091486
Cloud_Mesh_Distance_BA_R0_R2	19,91388512	-79,80059814	-939,0690308	-0,063992
Cloud_Mesh_Distance_BA_R0_R2	19,75041008	-79,88401794	-939,0293579	-0,077854
Cloud_Mesh_Distance_BA_R0_R2	18,77165794	-79,4415741	-939,0519409	-0,0881
Cloud_Mesh_Distance_BA_R0_R2	19,01989365	-79,55949402	-939,0147705	-0,098447
Cloud_Mesh_Distance_BA_R0_R2	19,0027523	-79,43190002	-939,0426636	-0,092948
Cloud_Mesh_Distance_BA_R0_R2	18,89122009	-79,40708923	-939,0515747	-0,085723
Cloud_Mesh_Distance_BA_R0_R2	19,13233376	-79,46861267	-939,038208	-0,09489
Cloud_Mesh_Distance_BA_R0_R2	19,13350677	-79,32365417	-939,0734253	-0,086924
Cloud_Mesh_Distance_BA_R0_R2	19,24868774	-79,383255	-939,0654297	-0,091447
Cloud_Mesh_Distance_BA_R0_R2	19,6623745	-79,29216003	-939,1436157	-0,077229
Cloud_Mesh_Distance_BA_R0_R2	19,64055634	-79,26142883	-939,1486816	-0,07758
Cloud_Mesh_Distance_BA_R0_R2	18,66730881	-79,76344299	-938,9937134	-0,094376
Cloud_Mesh_Distance_BA_R0_R2	18,79115295	-79,79150391	-938,9794312	-0,102606
Cloud_Mesh_Distance_BA_R0_R2	18,82273102	-79,69195557	-938,9920654	-0,099755
Cloud_Mesh_Distance_BA_R0_R2	18,75397873	-79,72381592	-938,9909668	-0,097243
Cloud_Mesh_Distance_BA_R0_R3	18,009655	-81,7982254	-946,1516113	0,151671
Cloud_Mesh_Distance_BA_R0_R3	17,86519051	-79,97463226	-946,2550659	0,004452
Cloud_Mesh_Distance_BA_R0_R3	17,89897728	-79,9409256	-946,2650757	0,005587
Cloud_Mesh_Distance_BA_R0_R3	17,92642975	-79,96808624	-946,2581787	0,008192
Cloud_Mesh_Distance_BA_R0_R3	17,87587738	-79,91968536	-946,2697144	0,002671
Cloud_Mesh_Distance_BA_R0_R3	17,93692398	-79,9131546	-946,2728882	0,00978
Cloud_Mesh_Distance_BA_R0_R3	16,75760841	-79,43525696	-946,2816162	-0,075583
Cloud_Mesh_Distance_BA_R0_R3	16,82849503	-79,56855774	-946,2685547	-0,07272
Cloud_Mesh_Distance_BA_R0_R3	17,3357048	-79,44800568	-946,3547363	-0,053971
Cloud_Mesh_Distance_BA_R0_R3	17,41267204	-79,59668732	-946,3300781	-0,047005
Cloud_Mesh_Distance_BA_R0_R3	17,54554939	-79,46591949	-946,3607178	-0,036732
Cloud_Mesh_Distance_BA_R0_R3	16,63383102	-79,44837189	-946,2463989	-0,079123
Cloud_Mesh_Distance_BA_R0_R3	16,87201691	-79,33811951	-946,3215332	-0,074342
Cloud_Mesh_Distance_BA_R0_R3	16,93922997	-79,46485138	-946,3117065	-0,069867
Cloud_Mesh_Distance_BA_R0_R3	17,12618256	-79,47486115	-946,3338013	-0,064778
Cloud_Mesh_Distance_BA_R0_R3	17,04255295	-79,36313629	-946,3446655	-0,070952
Cloud_Mesh_Distance_BA_R0_R3	17,49288177	-79,2876358	-946,3860474	-0,044006
Cloud_Mesh_Distance_BA_R0_R3	17,32946587	-79,33053589	-946,3739624	-0,056928
Cloud_Mesh_Distance_BA_R0_R3	17,57739258	-79,58623505	-946,3388062	-0,033809
Cloud_Mesh_Distance_BA_R0_R3	18,41703796	-79,39362335	-946,3334351	0,040874
Cloud_Mesh_Distance_BA_R0_R3	18,35696411	-79,48925018	-946,3322754	0,040855
Cloud_Mesh_Distance_BA_R0_R3	16,74011993	-79,31774139	-946,2966919	-0,078671
Cloud_Mesh_Distance_BA_R0_R3	16,64121628	-79,36037445	-946,2633667	-0,084169
Cloud_Mesh_Distance_BA_R0_R3	16,65945816	-79,25650024	-946,2823486	-0,087729
Cloud_Mesh_Distance_BA_R0_R3	16,84880829	-79,08068085	-946,343689	-0,088694
Cloud_Mesh_Distance_BA_R0_R3	17,05966949	-79,02218628	-946,3811035	-0,078517
Cloud_Mesh_Distance_BA_R0_R3	17,23981476	-79,19792938	-946,3847046	-0,065892
Cloud_Mesh_Distance_BA_R0_R3	17,09336472	-79,1571579	-946,3765869	-0,070985
Cloud_Mesh_Distance_BA_R0_R3	17,22105217	-79,06788635	-946,394165	-0,070068
Cloud_Mesh_Distance_BA_R0_R3	17,34339523	-79,2501297	-946,3846436	-0,058295
Cloud_Mesh_Distance_BA_R0_R3	17,39112663	-79,13153076	-946,3986206	-0,055579
Cloud_Mesh_Distance_BA_R0_R3	17,53491592	-79,14044952	-946,4011841	-0,044163
Cloud_Mesh_Distance_BA_R0_R3	17,73698997	-79,36383057	-946,3787842	-0,022484

Cloud_Mesh_Distance_BA_R0_R3	18,11236572	-79,35030365	-946,3693848	0,018701
Cloud_Mesh_Distance_BA_R0_R3	17,9313736	-79,39069366	-946,3731079	-0,001035
Cloud_Mesh_Distance_BA_R0_R3	18,29844666	-79,37793732	-946,3505859	0,03434
Cloud_Mesh_Distance_BA_R0_R3	18,50312424	-79,35881805	-946,3220825	0,040934
Cloud_Mesh_Distance_BA_R0_R3	16,77096176	-79,00466156	-946,3291016	-0,096156
Cloud_Mesh_Distance_BA_R0_R3	16,79149628	-78,85161591	-946,3352051	-0,106726
Cloud_Mesh_Distance_BA_R0_R3	16,91172028	-79,00156403	-946,3590698	-0,091625
Cloud_Mesh_Distance_BA_R0_R3	17,20228958	-78,90563965	-946,4002686	-0,07617
Cloud_Mesh_Distance_BA_R0_R3	17,7406044	-78,98713684	-946,4066773	-0,029659
Cloud_Mesh_Distance_BA_R0_R3	17,69961166	-79,1517868	-946,3998413	-0,029056
Cloud_Mesh_Distance_BA_R0_R3	17,9366169	-79,23390198	-946,387207	-0,003234
Cloud_Mesh_Distance_BA_R0_R3	17,89063263	-79,05561066	-946,3995972	-0,013421
Cloud_Mesh_Distance_BA_R0_R3	16,87189484	-78,90315247	-946,3539429	-0,098439
Cloud_Mesh_Distance_BA_R0_R3	17,0153389	-78,86646271	-946,3794556	-0,08944
Cloud_Mesh_Distance_BA_R0_R3	16,90373993	-78,78424835	-946,3585815	-0,109227
Cloud_Mesh_Distance_BA_R0_R3	17,01983261	-78,725914	-946,3778687	-0,09737
Cloud_Mesh_Distance_BA_R0_R3	17,18016434	-78,71469879	-946,3984375	-0,086095
Cloud_Mesh_Distance_BA_R0_R3	17,5890274	-78,80625153	-946,4127808	-0,049246
Cloud_Mesh_Distance_BA_R0_R3	17,55049515	-78,98194885	-946,4104614	-0,04685
Cloud_Mesh_Distance_BA_R0_R3	17,35284042	-78,97510529	-946,4071655	-0,060384
Cloud_Mesh_Distance_BA_R0_R3	17,6940155	-78,87162781	-946,4102783	-0,038176
Cloud_Mesh_Distance_BA_R0_R3	18,26318169	-78,80501556	-946,357666	0,00247
Cloud_Mesh_Distance_BA_R0_R3	18,20840454	-78,82872772	-946,3678589	0,002089
Cloud_Mesh_Distance_BA_R0_R3	18,28545189	-78,86720276	-946,3571777	0,007091
Cloud_Mesh_Distance_BA_R0_R3	17,22870636	-78,52967834	-946,3943481	-0,087727
Cloud_Mesh_Distance_BA_R0_R3	17,40923882	-78,77890778	-946,4116211	-0,063826
Cloud_Mesh_Distance_BA_R0_R3	17,34072876	-78,60163116	-946,4055786	-0,073404
Cloud_Mesh_Distance_BA_R0_R3	17,57462311	-78,68637085	-946,4119263	-0,050901
Cloud_Mesh_Distance_BA_R0_R3	18,31787109	-78,81240082	-946,3490601	0,004771
Cloud_Mesh_Distance_BA_R0_R3	17,21828842	-78,31483459	-946,3717651	-0,09521
Cloud_Mesh_Distance_BA_R0_R3	17,26646423	-78,39789581	-946,3867188	-0,086144
Cloud_Mesh_Distance_BA_R0_R3	17,50663567	-78,56113434	-946,4075928	-0,062484
Cloud_Mesh_Distance_BA_R0_R3	17,5476284	-78,40467834	-946,3981934	-0,056734
Cloud_Mesh_Distance_BA_R0_R3	17,39332199	-78,43621063	-946,3977051	-0,072796
Cloud_Mesh_Distance_BA_R0_R3	16,89907455	-81,82108307	-946,0808106	-0,282789
Cloud_Mesh_Distance_BA_R0_R3	17,12514687	-81,80257416	-946,1036377	0,146945
Cloud_Mesh_Distance_BA_R0_R3	16,69938469	-81,66530609	-945,9380493	0,294531
Cloud_Mesh_Distance_BA_R0_R3	16,88236618	-81,6657486	-945,9864502	0,159346
Cloud_Mesh_Distance_BA_R0_R3	17,34101486	-81,81127167	-946,130127	0,126498
Cloud_Mesh_Distance_BA_R0_R3	17,52711487	-81,66681671	-946,0980225	0,085179
Cloud_Mesh_Distance_BA_R0_R3	17,86362839	-81,80448151	-946,1524658	0,191945
Cloud_Mesh_Distance_BA_R0_R3	17,74256516	-81,71117401	-946,1269531	0,112498
Cloud_Mesh_Distance_BA_R0_R3	18,124897	-81,68943024	-946,1299439	0,120437
Cloud_Mesh_Distance_BA_R0_R3	18,61191559	-81,75199127	-946,1364746	-0,18876
Cloud_Mesh_Distance_BA_R0_R3	18,46327972	-81,77518463	-946,140564	-0,196619
Cloud_Mesh_Distance_BA_R0_R3	19,04723358	-81,79923248	-946,1481934	-0,238094
Cloud_Mesh_Distance_BA_R0_R3	16,8175869	-81,60706329	-945,9351807	0,175507
Cloud_Mesh_Distance_BA_R0_R3	17,0412178	-81,47145844	-945,9282227	0,138611
Cloud_Mesh_Distance_BA_R0_R3	17,05647659	-81,68953705	-946,0377808	0,096742
Cloud_Mesh_Distance_BA_R0_R3	17,08661652	-81,54743195	-945,9744263	0,109318

Cloud_Mesh_Distance_BA_R0_R3	17,28799057	-81,61267853	-946,0452271	0,097341
Cloud_Mesh_Distance_BA_R0_R3	17,94051361	-81,68082428	-946,1268921	0,064717
Cloud_Mesh_Distance_BA_R0_R3	18,31643295	-81,76010895	-946,1403198	-0,118484
Cloud_Mesh_Distance_BA_R0_R3	18,83822441	-81,74474335	-946,1378174	-0,225551
Cloud_Mesh_Distance_BA_R0_R3	18,86191177	-81,59477997	-946,1159058	-0,116928
Cloud_Mesh_Distance_BA_R0_R3	19,03559875	-81,57123566	-946,1151733	-0,11792
Cloud_Mesh_Distance_BA_R0_R3	19,2653923	-81,67597198	-946,1286011	-0,1831
Cloud_Mesh_Distance_BA_R0_R3	19,63159752	-81,7407608	-946,1154175	-0,21168
Cloud_Mesh_Distance_BA_R0_R3	17,15812683	-81,39006805	-945,9274292	0,167619
Cloud_Mesh_Distance_BA_R0_R3	17,52758598	-81,47919464	-946,0374146	0,123646
Cloud_Mesh_Distance_BA_R0_R3	17,70841408	-81,38404083	-946,0379639	0,114057
Cloud_Mesh_Distance_BA_R0_R3	17,70811844	-81,53575897	-946,0772705	0,098901
Cloud_Mesh_Distance_BA_R0_R3	18,05210686	-81,52216339	-946,0943604	0,044863
Cloud_Mesh_Distance_BA_R0_R3	17,86427307	-81,57102203	-946,0987549	0,075433
Cloud_Mesh_Distance_BA_R0_R3	18,28281784	-81,55489349	-946,1029663	-0,007108
Cloud_Mesh_Distance_BA_R0_R3	18,47709465	-81,63518524	-946,1170044	-0,070208
Cloud_Mesh_Distance_BA_R0_R3	18,84301758	-81,36193085	-946,0869751	-0,084328
Cloud_Mesh_Distance_BA_R0_R3	18,84393692	-81,43199921	-946,093811	-0,092097
Cloud_Mesh_Distance_BA_R0_R3	18,90031052	-81,50113678	-946,1034546	-0,102684
Cloud_Mesh_Distance_BA_R0_R3	18,96600533	-81,38240814	-946,0916748	-0,096712
Cloud_Mesh_Distance_BA_R0_R3	19,12668228	-81,39614105	-946,0951538	-0,104857
Cloud_Mesh_Distance_BA_R0_R3	19,27286911	-81,46141815	-946,1001587	-0,116647
Cloud_Mesh_Distance_BA_R0_R3	19,7460022	-81,63434601	-946,0857544	-0,156912
Cloud_Mesh_Distance_BA_R0_R3	19,77093697	-81,75641632	-946,1026001	-0,252175
Cloud_Mesh_Distance_BA_R0_R3	19,86544991	-81,66703033	-946,0747681	-0,186648
Cloud_Mesh_Distance_BA_R0_R3	17,69309616	-81,27170563	-946,0128174	0,121184
Cloud_Mesh_Distance_BA_R0_R3	17,88912964	-81,41529083	-946,0640259	0,08298
Cloud_Mesh_Distance_BA_R0_R3	18,19312477	-81,1475296	-946,0499878	0,033412
Cloud_Mesh_Distance_BA_R0_R3	18,45166206	-81,47676849	-946,0906372	-0,0389
Cloud_Mesh_Distance_BA_R0_R3	18,4557724	-81,35765839	-946,0759888	-0,030985
Cloud_Mesh_Distance_BA_R0_R3	18,88529205	-81,28148651	-946,0810547	-0,082341
Cloud_Mesh_Distance_BA_R0_R3	19,25123215	-81,29856873	-946,0822144	-0,102785
Cloud_Mesh_Distance_BA_R0_R3	19,42199516	-81,42266083	-946,0888062	-0,11368
Cloud_Mesh_Distance_BA_R0_R3	19,38589859	-81,32759857	-946,0795898	-0,106628
Cloud_Mesh_Distance_BA_R0_R3	19,49370575	-81,3467865	-946,0731201	-0,111479
Cloud_Mesh_Distance_BA_R0_R3	18,20415115	-81,12342072	-946,0491943	0,031796
Cloud_Mesh_Distance_BA_R0_R3	18,227314	-81,13734436	-946,0508423	0,027161
Cloud_Mesh_Distance_BA_R0_R3	18,92927933	-81,15068817	-946,0734863	-0,073527
Cloud_Mesh_Distance_BA_R0_R3	18,99246597	-81,24261475	-946,0791626	-0,087741
Cloud_Mesh_Distance_BA_R0_R3	19,10775948	-81,2669754	-946,0817261	-0,094463
Cloud_Mesh_Distance_BA_R0_R3	19,23379135	-81,15398407	-946,0693359	-0,09183
Cloud_Mesh_Distance_BA_R0_R3	19,48934937	-81,25209045	-946,0623169	-0,105665
Cloud_Mesh_Distance_BA_R0_R3	18,25167084	-80,80514526	-946,0689087	0,02856
Cloud_Mesh_Distance_BA_R0_R3	17,53944016	-80,39121246	-946,1020508	0,016868
Cloud_Mesh_Distance_BA_R0_R3	17,35938454	-80,42475128	-946,0653076	0,017804
Cloud_Mesh_Distance_BA_R0_R3	17,57778931	-80,57172394	-946,0455322	0,036123
Cloud_Mesh_Distance_BA_R0_R3	17,89761353	-80,53492737	-946,0973511	0,038721
Cloud_Mesh_Distance_BA_R0_R3	18,04379272	-80,63770294	-946,0857544	0,044599
Cloud_Mesh_Distance_BA_R0_R3	18,04525566	-80,47057343	-946,1262207	0,039054
Cloud_Mesh_Distance_BA_R0_R3	18,20498276	-80,51163483	-946,1224976	0,034005

Cloud_Mesh_Distance_BA_R0_R3	18,34672546	-80,72274017	-946,0839844	0,02269
Cloud_Mesh_Distance_BA_R0_R3	18,22574234	-80,67888641	-946,0883179	0,035338
Cloud_Mesh_Distance_BA_R0_R3	18,39790916	-80,57629395	-946,111145	0,023221
Cloud_Mesh_Distance_BA_R0_R3	18,47668839	-80,70701599	-946,0883179	0,011396
Cloud_Mesh_Distance_BA_R0_R3	18,63428307	-80,68045807	-946,0895996	-0,002352
Cloud_Mesh_Distance_BA_R0_R3	18,69109344	-80,54381561	-946,1074219	0,004183
Cloud_Mesh_Distance_BA_R0_R3	16,60506821	-80,23513031	-945,9916382	-0,016258
Cloud_Mesh_Distance_BA_R0_R3	16,4977951	-80,23317719	-945,9492188	-0,024532
Cloud_Mesh_Distance_BA_R0_R3	16,60934639	-80,31868744	-945,9569092	-0,005287
Cloud_Mesh_Distance_BA_R0_R3	16,73111916	-80,299263	-946,0044556	-0,001688
Cloud_Mesh_Distance_BA_R0_R3	17,16292953	-80,21990204	-946,119751	-0,008139
Cloud_Mesh_Distance_BA_R0_R3	17,20173645	-80,38558197	-946,0588989	0,009776
Cloud_Mesh_Distance_BA_R0_R3	17,01615524	-80,34978485	-946,0449829	0,005813
Cloud_Mesh_Distance_BA_R0_R3	17,38742447	-80,25962067	-946,132019	-0,002648
Cloud_Mesh_Distance_BA_R0_R3	17,72467041	-80,27180481	-946,1608887	0,014224
Cloud_Mesh_Distance_BA_R0_R3	17,73587799	-80,45568085	-946,1037598	0,031598
Cloud_Mesh_Distance_BA_R0_R3	18,06051254	-80,3768692	-946,1516113	0,033263
Cloud_Mesh_Distance_BA_R0_R3	18,1364975	-80,4141922	-946,1439819	0,034765
Cloud_Mesh_Distance_BA_R0_R3	18,59716606	-80,4234848	-946,133728	0,019617
Cloud_Mesh_Distance_BA_R0_R3	18,56098175	-80,58878326	-946,1057129	0,012857
Cloud_Mesh_Distance_BA_R0_R3	16,86782074	-79,99423981	-946,1529541	-0,040339
Cloud_Mesh_Distance_BA_R0_R3	16,72454834	-80,14850616	-946,0631714	-0,023835
Cloud_Mesh_Distance_BA_R0_R3	16,92434692	-80,20244598	-946,0883789	-0,012471
Cloud_Mesh_Distance_BA_R0_R3	17,57554245	-80,22891998	-946,1618042	0,002171
Cloud_Mesh_Distance_BA_R0_R3	17,3418293	-80,07474518	-946,192749	-0,023419
Cloud_Mesh_Distance_BA_R0_R3	17,93835068	-80,33457184	-946,1569214	0,030448
Cloud_Mesh_Distance_BA_R0_R3	18,10498047	-80,1620636	-946,2098999	0,026469
Cloud_Mesh_Distance_BA_R0_R3	18,32282639	-80,19683075	-946,1972046	0,036534
Cloud_Mesh_Distance_BA_R0_R3	18,07646561	-80,30352783	-946,1712036	0,029707
Cloud_Mesh_Distance_BA_R0_R3	18,17281723	-80,28501129	-946,1782837	0,034064
Cloud_Mesh_Distance_BA_R0_R3	18,3651886	-80,38146973	-946,1524658	0,030942
Cloud_Mesh_Distance_BA_R0_R3	18,68902206	-80,28052521	-946,1542358	0,02301
Cloud_Mesh_Distance_BA_R0_R3	18,75014305	-80,34720612	-946,1361084	0,014586
Cloud_Mesh_Distance_BA_R0_R3	18,56232834	-80,21524811	-946,1784668	0,033043
Cloud_Mesh_Distance_BA_R0_R3	16,48705864	-79,82906342	-946,0973511	-0,066384
Cloud_Mesh_Distance_BA_R0_R3	16,75589371	-79,83070374	-946,1809082	-0,058721
Cloud_Mesh_Distance_BA_R0_R3	16,93707657	-79,8289566	-946,2193604	-0,05308
Cloud_Mesh_Distance_BA_R0_R3	17,11719894	-80,0459671	-946,177063	-0,030761
Cloud_Mesh_Distance_BA_R0_R3	17,31256866	-79,90037537	-946,2451172	-0,036803
Cloud_Mesh_Distance_BA_R0_R3	17,09760094	-79,87470245	-946,2294312	-0,045179
Cloud_Mesh_Distance_BA_R0_R3	17,54548073	-80,09661102	-946,2033691	-0,011584
Cloud_Mesh_Distance_BA_R0_R3	17,53121948	-79,92584229	-946,2539063	-0,023982
Cloud_Mesh_Distance_BA_R0_R3	17,88118935	-80,09174347	-946,2240601	0,012522
Cloud_Mesh_Distance_BA_R0_R3	17,69370461	-80,12985992	-946,2028809	0,000488
Cloud_Mesh_Distance_BA_R0_R3	18,72403526	-80,172966	-946,1705322	0,028586
Cloud_Mesh_Distance_BA_R0_R3	18,7196312	-80,02810669	-946,197937	0,036491
Cloud_Mesh_Distance_BA_R0_R3	18,94169235	-80,04040527	-946,1592407	0,023816
Cloud_Mesh_Distance_BA_R0_R3	16,5842514	-79,7534256	-946,1550293	-0,067394
Cloud_Mesh_Distance_BA_R0_R3	16,70833206	-79,66467285	-946,2166138	-0,06891
Cloud_Mesh_Distance_BA_R0_R3	17,5084095	-79,73688507	-946,3030396	-0,034228

Cloud_Mesh_Distance_BA_R0_R3	17,72138023	-79,78446198	-946,2998047	-0,013047
Cloud_Mesh_Distance_BA_R0_R3	17,75439072	-79,96585846	-946,2540283	-0,001535
Cloud_Mesh_Distance_BA_R0_R3	18,29639816	-79,98323822	-946,2474365	0,035982
Cloud_Mesh_Distance_BA_R0_R3	18,28326225	-79,78401184	-946,2911987	0,035032
Cloud_Mesh_Distance_BA_R0_R3	18,06023788	-79,9333725	-946,2675171	0,02103
Cloud_Mesh_Distance_BA_R0_R3	18,5134964	-80,01345825	-946,2249146	0,042518
Cloud_Mesh_Distance_BA_R0_R3	18,68223953	-79,84575653	-946,2336426	0,042929
Cloud_Mesh_Distance_BA_R0_R3	18,87999153	-79,97193909	-946,180542	0,033039
Cloud_Mesh_Distance_BA_R0_R3	18,96193314	-79,83887482	-946,180481	0,029564
Cloud_Mesh_Distance_BA_R0_R3	18,98315239	-79,95119476	-946,1628418	0,02443
Cloud_Mesh_Distance_BA_R0_R3	16,16741562	-79,3289566	-946,0733643	-0,095584
Cloud_Mesh_Distance_BA_R0_R3	16,265728	-79,50409698	-946,0908814	-0,086913
Cloud_Mesh_Distance_BA_R0_R3	16,67195511	-79,53336334	-946,2390747	-0,074595
Cloud_Mesh_Distance_BA_R0_R3	16,53027344	-79,57732391	-946,1841431	-0,073848
Cloud_Mesh_Distance_BA_R0_R3	16,5881691	-79,493927	-946,2226563	-0,076115
Cloud_Mesh_Distance_BA_R0_R3	16,8862915	-79,70490265	-946,2453003	-0,063888
Cloud_Mesh_Distance_BA_R0_R3	17,01166153	-79,59224701	-946,2949829	-0,065166
Cloud_Mesh_Distance_BA_R0_R3	17,07123566	-79,72562408	-946,2687988	-0,057198
Cloud_Mesh_Distance_BA_R0_R3	17,20835495	-79,59838867	-946,3153076	-0,059495
Cloud_Mesh_Distance_BA_R0_R3	17,27174377	-79,74002838	-946,2858276	-0,049651
Cloud_Mesh_Distance_BA_R0_R3	17,88292313	-79,64382172	-946,333252	-0,000233
Cloud_Mesh_Distance_BA_R0_R3	17,92956161	-79,8174057	-946,2960205	0,009244
Cloud_Mesh_Distance_BA_R0_R3	18,25282097	-79,62586212	-946,3228149	0,034386
Cloud_Mesh_Distance_BA_R0_R3	18,12258911	-79,75774384	-946,3052368	0,022841
Cloud_Mesh_Distance_BA_R0_R3	18,48711205	-79,81092072	-946,2666016	0,043568
Cloud_Mesh_Distance_BA_R0_R3	18,45813751	-79,60095978	-946,3046265	0,043663
Cloud_Mesh_Distance_BA_R0_R3	19,02062607	-79,70285797	-946,1793823	0,027765
Cloud_Mesh_Distance_BA_R0_R3	18,8241291	-79,87703705	-946,2040405	0,036903
Cloud_Mesh_Distance_BA_R0_R3	16,26412582	-79,37172699	-946,1148071	-0,094801
Cloud_Mesh_Distance_BA_R0_R3	16,23253632	-79,25907135	-946,1154785	-0,098161
Cloud_Mesh_Distance_BA_R0_R3	16,38700104	-79,45198822	-946,1559448	-0,085983
Cloud_Mesh_Distance_BA_R0_R3	16,52806282	-79,41156006	-946,2175903	-0,08208
Cloud_Mesh_Distance_BA_R0_R3	16,39374161	-79,25312805	-946,1878662	-0,098196
Cloud_Mesh_Distance_BA_R0_R3	16,79453278	-79,18796539	-946,3241577	-0,081647
Cloud_Mesh_Distance_BA_R0_R3	17,20612717	-79,36585999	-946,3607788	-0,06342
Cloud_Mesh_Distance_BA_R0_R3	17,68560791	-79,50868988	-946,3566895	-0,023668
Cloud_Mesh_Distance_BA_R0_R3	17,70649338	-79,62612915	-946,3346558	-0,021016
Cloud_Mesh_Distance_BA_R0_R3	18,00940323	-79,50260162	-946,3552246	0,010231
Cloud_Mesh_Distance_BA_R0_R3	17,83155823	-79,50479889	-946,3577881	-0,008136
Cloud_Mesh_Distance_BA_R0_R3	18,06990623	-79,63853455	-946,3308716	0,017177
Cloud_Mesh_Distance_BA_R0_R3	18,19024658	-79,4960556	-946,3468628	0,029187
Cloud_Mesh_Distance_BA_R0_R3	18,48080826	-79,44948578	-946,3178101	0,043561
Cloud_Mesh_Distance_BA_R0_R3	18,66623688	-79,644104	-946,2644653	0,0442
Cloud_Mesh_Distance_BA_R0_R3	18,86222458	-79,70682526	-946,2172241	0,038701
Cloud_Mesh_Distance_BA_R0_R3	16,59437561	-79,12290192	-946,2733765	-0,099354
Cloud_Mesh_Distance_BA_R0_R3	16,55716324	-79,28277588	-946,2467041	-0,087376
Cloud_Mesh_Distance_BA_R0_R3	16,97710228	-79,24289703	-946,3521118	-0,076452
Cloud_Mesh_Distance_BA_R0_R3	16,94239044	-79,11833954	-946,3582764	-0,085403
Cloud_Mesh_Distance_BA_R0_R3	17,13542747	-79,27263641	-946,3679199	-0,065956
Cloud_Mesh_Distance_BA_R0_R3	17,25294113	-79,28696442	-946,3758545	-0,061084

Cloud_Mesh_Distance_BA_R0_R3	18,26167107	-79,24478912	-946,3630371	0,026763
Cloud_Mesh_Distance_BA_R0_R3	18,40925598	-79,29550934	-946,3406982	0,036128
Cloud_Mesh_Distance_BA_R0_R3	18,39612007	-79,14893341	-946,3460083	0,02899
Cloud_Mesh_Distance_BA_R0_R3	18,54158592	-79,24437714	-946,317688	0,035215
Cloud_Mesh_Distance_BA_R0_R3	18,84074402	-79,49558258	-946,2393189	0,036329
Cloud_Mesh_Distance_BA_R0_R3	18,63564873	-79,41106415	-946,2914429	0,041209
Cloud_Mesh_Distance_BA_R0_R3	16,18110657	-78,86847687	-946,104126	-0,127358
Cloud_Mesh_Distance_BA_R0_R3	16,32004738	-78,80318451	-946,1668701	-0,134046
Cloud_Mesh_Distance_BA_R0_R3	16,4077282	-78,989151	-946,2088013	-0,118915
Cloud_Mesh_Distance_BA_R0_R3	18,04187202	-79,11192322	-946,3881226	0,002771
Cloud_Mesh_Distance_BA_R0_R3	18,20625496	-79,08332062	-946,3735352	0,015721
Cloud_Mesh_Distance_BA_R0_R3	18,11572647	-79,20609283	-946,3788452	0,013264
Cloud_Mesh_Distance_BA_R0_R3	18,04962349	-78,97133636	-946,3895264	-0,000049
Cloud_Mesh_Distance_BA_R0_R3	18,36329651	-78,96576691	-946,3477783	0,014942
Cloud_Mesh_Distance_BA_R0_R3	18,70372009	-79,19717407	-946,2798462	0,02786
Cloud_Mesh_Distance_BA_R0_R3	18,8622818	-79,12552643	-946,2330933	0,013478
Cloud_Mesh_Distance_BA_R0_R3	18,84073639	-79,2999649	-946,2440186	0,027341
Cloud_Mesh_Distance_BA_R0_R3	16,20820618	-78,74932098	-946,1118164	-0,13513
Cloud_Mesh_Distance_BA_R0_R3	16,45881271	-78,60715485	-946,2114868	-0,145364
Cloud_Mesh_Distance_BA_R0_R3	16,48485565	-78,78362274	-946,2340698	-0,1317
Cloud_Mesh_Distance_BA_R0_R3	16,63455963	-78,87050629	-946,2902222	-0,120115
Cloud_Mesh_Distance_BA_R0_R3	16,95080185	-78,66594696	-946,3635254	-0,107837
Cloud_Mesh_Distance_BA_R0_R3	17,07597351	-78,6006546	-946,3811646	-0,098971
Cloud_Mesh_Distance_BA_R0_R3	17,87026978	-78,86664581	-946,402832	-0,021763
Cloud_Mesh_Distance_BA_R0_R3	18,08835793	-78,77846527	-946,3815918	-0,007409
Cloud_Mesh_Distance_BA_R0_R3	18,21505356	-78,75839233	-946,3630981	-0,00179
Cloud_Mesh_Distance_BA_R0_R3	18,19965744	-78,91867828	-946,3717651	0,005536
Cloud_Mesh_Distance_BA_R0_R3	18,3699913	-78,85243988	-946,34198	0,008469
Cloud_Mesh_Distance_BA_R0_R3	18,50767326	-78,82202911	-946,3100586	0,00503
Cloud_Mesh_Distance_BA_R0_R3	18,74455643	-79,04779816	-946,2630615	0,014503
Cloud_Mesh_Distance_BA_R0_R3	18,70663071	-78,89894867	-946,2641602	0,004591
Cloud_Mesh_Distance_BA_R0_R3	18,56987381	-79,04836273	-946,3087158	0,021421
Cloud_Mesh_Distance_BA_R0_R3	16,40175056	-78,37439728	-946,1580811	-0,155149
Cloud_Mesh_Distance_BA_R0_R3	16,35123825	-78,51212311	-946,156311	-0,149969
Cloud_Mesh_Distance_BA_R0_R3	16,63788223	-78,64538574	-946,2792969	-0,138228
Cloud_Mesh_Distance_BA_R0_R3	16,65121078	-78,44107819	-946,2615356	-0,152494
Cloud_Mesh_Distance_BA_R0_R3	16,80987549	-78,68992615	-946,3326416	-0,120475
Cloud_Mesh_Distance_BA_R0_R3	16,82330513	-78,48246002	-946,3180542	-0,133894
Cloud_Mesh_Distance_BA_R0_R3	17,13194275	-78,4464035	-946,37677	-0,104274
Cloud_Mesh_Distance_BA_R0_R3	17,82188988	-78,48851776	-946,3920288	-0,033007
Cloud_Mesh_Distance_BA_R0_R3	17,72812271	-78,71113586	-946,4078369	-0,036321
Cloud_Mesh_Distance_BA_R0_R3	17,90835571	-78,68161011	-946,3955689	-0,022618
Cloud_Mesh_Distance_BA_R0_R3	18,03998756	-78,56313324	-946,3754883	-0,016122
Cloud_Mesh_Distance_BA_R0_R3	18,3000412	-78,74103546	-946,3480225	0,000968
Cloud_Mesh_Distance_BA_R0_R3	18,224617	-78,62464142	-946,3522339	-0,00595
Cloud_Mesh_Distance_BA_R0_R3	18,37990189	-78,77857208	-946,3353882	0,003236
Cloud_Mesh_Distance_BA_R0_R3	18,68902969	-78,71707153	-946,2520142	-0,007752
Cloud_Mesh_Distance_BA_R0_R3	16,65866089	-78,23950958	-946,232605	-0,154103
Cloud_Mesh_Distance_BA_R0_R3	16,5013237	-78,40860748	-946,2039795	-0,15525
Cloud_Mesh_Distance_BA_R0_R3	16,84902763	-78,27417755	-946,2957153	-0,141538

Cloud_Mesh_Distance_BA_R0_R3	17,04626846	-78,32402802	-946,3470459	-0,117083
Cloud_Mesh_Distance_BA_R0_R3	16,96920395	-78,53276062	-946,3577271	-0,115054
Cloud_Mesh_Distance_BA_R0_R3	17,65285873	-78,57936859	-946,4067993	-0,045541
Cloud_Mesh_Distance_BA_R0_R3	17,64314651	-78,45993805	-946,3999023	-0,047362
Cloud_Mesh_Distance_BA_R0_R3	17,6922493	-78,28352356	-946,3824463	-0,046352
Cloud_Mesh_Distance_BA_R0_R3	17,88705444	-78,28543854	-946,3679199	-0,02973
Cloud_Mesh_Distance_BA_R0_R3	18,56196594	-78,64723969	-946,2815552	-0,005367
Cloud_Mesh_Distance_BA_R0_R3	18,40796089	-78,65016937	-946,3184204	-0,003443
Cloud_Mesh_Distance_BA_R0_R3	16,70320892	-78,0536499	-946,2053833	-0,152688
Cloud_Mesh_Distance_BA_R0_R3	17,06208992	-78,1027298	-946,3131104	-0,120451
Cloud_Mesh_Distance_BA_R0_R3	17,28243256	-78,14418793	-946,3531494	-0,089111
Cloud_Mesh_Distance_BA_R0_R3	17,36687851	-78,28403473	-946,3810425	-0,079234
Cloud_Mesh_Distance_BA_R0_R3	17,5069313	-78,30414581	-946,3880005	-0,062054
Cloud_Mesh_Distance_BA_R0_R3	17,53451538	-78,1391449	-946,3667603	-0,057562
Cloud_Mesh_Distance_BA_R0_R3	17,86347389	-78,13641357	-946,3512573	-0,026915
Cloud_Mesh_Distance_BA_R0_R3	18,02670288	-78,3647995	-946,3601685	-0,021256
Cloud_Mesh_Distance_BA_R0_R3	18,0227375	-78,17714691	-946,3374634	-0,018628
Cloud_Mesh_Distance_BA_R0_R3	18,17116547	-78,44484711	-946,345459	-0,014456
Cloud_Mesh_Distance_BA_R0_R3	18,34975052	-78,43091583	-946,3094482	-0,010877
Cloud_Mesh_Distance_BA_R0_R3	18,66951752	-78,54441071	-946,239502	-0,017397
Cloud_Mesh_Distance_BA_R0_R3	18,52464294	-78,49803162	-946,2747192	-0,012023
Cloud_Mesh_Distance_BA_R0_R3	18,63078117	-78,39868927	-946,2314453	-0,019166
Cloud_Mesh_Distance_BA_R0_R3	16,59109497	-78,012146	-946,1517944	-0,149988
Cloud_Mesh_Distance_BA_R0_R3	16,6384716	-77,91687775	-946,1387329	-0,149651
Cloud_Mesh_Distance_BA_R0_R3	16,86354065	-78,07648468	-946,2605591	-0,140756
Cloud_Mesh_Distance_BA_R0_R3	17,26805496	-77,93508911	-946,307251	-0,092581
Cloud_Mesh_Distance_BA_R0_R3	17,06264496	-77,89547729	-946,2612305	-0,110991
Cloud_Mesh_Distance_BA_R0_R3	17,7267189	-78,09314728	-946,3543091	-0,037736
Cloud_Mesh_Distance_BA_R0_R3	17,6782341	-77,95912933	-946,3306274	-0,042335
Cloud_Mesh_Distance_BA_R0_R3	17,85133362	-77,98155975	-946,3239746	-0,026395
Cloud_Mesh_Distance_BA_R0_R3	18,20813751	-78,24850464	-946,3156738	-0,014558
Cloud_Mesh_Distance_BA_R0_R3	18,14919281	-78,02562714	-946,289856	-0,013498
Cloud_Mesh_Distance_BA_R0_R3	17,98329353	-78,03388214	-946,3193359	-0,021369
Cloud_Mesh_Distance_BA_R0_R3	16,70635033	-77,93840027	-946,1726685	-0,144016
Cloud_Mesh_Distance_BA_R0_R3	16,74344063	-77,80252838	-946,1361084	-0,136935
Cloud_Mesh_Distance_BA_R0_R3	16,84453011	-77,91837311	-946,2124023	-0,13716
Cloud_Mesh_Distance_BA_R0_R3	17,25671577	-77,65370941	-946,2125244	-0,091552
Cloud_Mesh_Distance_BA_R0_R3	17,14191055	-77,73918915	-946,2262573	-0,103249
Cloud_Mesh_Distance_BA_R0_R3	17,44525909	-77,78621674	-946,2821655	-0,065958
Cloud_Mesh_Distance_BA_R0_R3	17,26691818	-77,76951599	-946,2575684	-0,088352
Cloud_Mesh_Distance_BA_R0_R3	17,47687912	-77,95355988	-946,3292847	-0,062406
Cloud_Mesh_Distance_BA_R0_R3	17,63633156	-77,80350494	-946,2919922	-0,042201
Cloud_Mesh_Distance_BA_R0_R3	18,17443085	-77,86695099	-946,2451782	-0,012014
Cloud_Mesh_Distance_BA_R0_R3	18,28379059	-77,93230438	-946,2377319	-0,013908
Cloud_Mesh_Distance_BA_R0_R3	18,01076317	-77,88018036	-946,279541	-0,014034
Cloud_Mesh_Distance_BA_R0_R3	17,408535	-77,64267731	-946,2270508	-0,069787
Cloud_Mesh_Distance_BA_R0_R3	17,82225037	-77,82946777	-946,2893677	-0,026227
Cloud_Mesh_Distance_BA_R0_R3	16,76875114	-81,55588531	-945,8917847	0,191604
Cloud_Mesh_Distance_BA_R0_R3	16,61315155	-81,58382416	-945,8574829	0,26233
Cloud_Mesh_Distance_BA_R0_R3	16,93868637	-81,33573914	-945,8477783	0,206294

Cloud_Mesh_Distance_BA_R0_R3	16,92096329	-81,5313797	-945,9229126	0,110614
Cloud_Mesh_Distance_BA_R0_R3	16,999403	-81,41153717	-945,8922119	0,16493
Cloud_Mesh_Distance_BA_R0_R3	17,38464355	-81,45017242	-946,0026856	0,13965
Cloud_Mesh_Distance_BA_R0_R3	18,73781586	-81,62799835	-946,1177368	-0,111968
Cloud_Mesh_Distance_BA_R0_R3	18,62233734	-81,61299896	-946,1138306	-0,082451
Cloud_Mesh_Distance_BA_R0_R3	18,69259262	-81,53308868	-946,1033325	-0,082201
Cloud_Mesh_Distance_BA_R0_R3	18,79307175	-81,51558685	-946,1034546	-0,092576
Cloud_Mesh_Distance_BA_R0_R3	19,43966675	-81,66083527	-946,1194458	-0,146026
Cloud_Mesh_Distance_BA_R0_R3	19,5365448	-81,68538666	-946,1157227	-0,15758
Cloud_Mesh_Distance_BA_R0_R3	19,88906479	-81,74976349	-946,0864868	-0,257362
Cloud_Mesh_Distance_BA_R0_R3	20,5975132	-81,8534317	-945,9479981	-0,25594
Cloud_Mesh_Distance_BA_R0_R3	17,26573372	-81,21550751	-945,9064941	0,173014
Cloud_Mesh_Distance_BA_R0_R3	17,15402412	-81,16516876	-945,8682861	0,180565
Cloud_Mesh_Distance_BA_R0_R3	17,08511734	-81,28462982	-945,8751221	0,196348
Cloud_Mesh_Distance_BA_R0_R3	17,36390686	-81,33001709	-945,9607544	0,159366
Cloud_Mesh_Distance_BA_R0_R3	17,2567482	-81,46581268	-945,9793091	0,141892
Cloud_Mesh_Distance_BA_R0_R3	17,55769348	-81,24222565	-945,9822998	0,137986
Cloud_Mesh_Distance_BA_R0_R3	17,48589897	-81,36846161	-945,9976807	0,142664
Cloud_Mesh_Distance_BA_R0_R3	17,59450531	-81,34752655	-946,012146	0,132323
Cloud_Mesh_Distance_BA_R0_R3	18,06292534	-81,36322784	-946,0645752	0,050347
Cloud_Mesh_Distance_BA_R0_R3	18,17307281	-81,21664429	-946,0526123	0,033096
Cloud_Mesh_Distance_BA_R0_R3	18,21089935	-81,39142609	-946,0742798	0,016715
Cloud_Mesh_Distance_BA_R0_R3	18,34978485	-81,41254425	-946,0801392	-0,013956
Cloud_Mesh_Distance_BA_R0_R3	18,58714485	-81,5079422	-946,0969849	-0,065408
Cloud_Mesh_Distance_BA_R0_R3	19,41493797	-81,53929901	-946,1035767	-0,124346
Cloud_Mesh_Distance_BA_R0_R3	19,54352188	-81,57305145	-946,0987549	-0,129361
Cloud_Mesh_Distance_BA_R0_R3	19,63149071	-81,62384796	-946,0976563	-0,140233
Cloud_Mesh_Distance_BA_R0_R3	20,32569695	-81,73596954	-946,0027466	-0,207087
Cloud_Mesh_Distance_BA_R0_R3	20,04265213	-81,66492462	-946,0472412	-0,192219
Cloud_Mesh_Distance_BA_R0_R3	20,05641556	-81,44150543	-946,0073242	-0,104626
Cloud_Mesh_Distance_BA_R0_R3	20,46310043	-81,54859161	-945,9358521	-0,075685
Cloud_Mesh_Distance_BA_R0_R3	20,26366425	-81,49869537	-945,9764404	-0,090054
Cloud_Mesh_Distance_BA_R0_R3	20,5692482	-81,60883331	-945,9144287	-0,069339
Cloud_Mesh_Distance_BA_R0_R3	17,25683212	-81,09905243	-945,8886108	0,154906
Cloud_Mesh_Distance_BA_R0_R3	17,43131638	-81,18399811	-945,9441528	0,148753
Cloud_Mesh_Distance_BA_R0_R3	17,99510574	-81,27708435	-946,0475464	0,065497
Cloud_Mesh_Distance_BA_R0_R3	17,7608223	-81,12674713	-946,0041504	0,104658
Cloud_Mesh_Distance_BA_R0_R3	18,01104355	-81,13050079	-946,0343018	0,065358
Cloud_Mesh_Distance_BA_R0_R3	17,84276199	-81,26624298	-946,0320435	0,093558
Cloud_Mesh_Distance_BA_R0_R3	18,34991074	-81,28474426	-946,065918	-0,005324
Cloud_Mesh_Distance_BA_R0_R3	18,55710983	-81,39186859	-946,0818481	-0,052375
Cloud_Mesh_Distance_BA_R0_R3	18,34110641	-81,10876465	-946,055603	0,007925
Cloud_Mesh_Distance_BA_R0_R3	18,57239532	-81,22286987	-946,0686035	-0,041171
Cloud_Mesh_Distance_BA_R0_R3	18,78495026	-81,23194122	-946,0749512	-0,069939
Cloud_Mesh_Distance_BA_R0_R3	18,71959877	-81,39167023	-946,0859985	-0,075342
Cloud_Mesh_Distance_BA_R0_R3	18,80667686	-81,31742096	-946,0824585	-0,07848
Cloud_Mesh_Distance_BA_R0_R3	19,63013649	-81,29612732	-946,0512695	-0,108294
Cloud_Mesh_Distance_BA_R0_R3	19,53464699	-81,44332123	-946,0814209	-0,120369
Cloud_Mesh_Distance_BA_R0_R3	19,88082123	-81,53458405	-946,0509033	-0,120649
Cloud_Mesh_Distance_BA_R0_R3	19,69344711	-81,48778534	-946,0704956	-0,122822

Cloud_Mesh_Distance_BA_R0_R3	19,87926292	-81,3142395	-946,0166626	-0,10667
Cloud_Mesh_Distance_BA_R0_R3	20,20142746	-81,33800507	-945,9608154	-0,089563
Cloud_Mesh_Distance_BA_R0_R3	20,32470703	-81,35799408	-945,9365234	-0,079954
Cloud_Mesh_Distance_BA_R0_R3	20,42564392	-81,40122223	-945,9199219	-0,070708
Cloud_Mesh_Distance_BA_R0_R3	20,56402588	-81,40952301	-945,8793335	-0,061125
Cloud_Mesh_Distance_BA_R0_R3	20,60315323	-81,5144577	-945,8861084	-0,062842
Cloud_Mesh_Distance_BA_R0_R3	16,76183701	-80,87609863	-945,7788696	0,115823
Cloud_Mesh_Distance_BA_R0_R3	16,85700417	-80,90279388	-945,8000488	0,122262
Cloud_Mesh_Distance_BA_R0_R3	17,37597656	-80,95183563	-945,9187012	0,109246
Cloud_Mesh_Distance_BA_R0_R3	17,37419128	-80,86095428	-945,9297485	0,091062
Cloud_Mesh_Distance_BA_R0_R3	17,39206314	-81,06134796	-945,9210205	0,133566
Cloud_Mesh_Distance_BA_R0_R3	17,56673622	-81,0774231	-945,9636841	0,118635
Cloud_Mesh_Distance_BA_R0_R3	17,71353912	-80,96663666	-945,9926148	0,094138
Cloud_Mesh_Distance_BA_R0_R3	18,1687088	-81,11472321	-946,0460815	0,037748
Cloud_Mesh_Distance_BA_R0_R3	18,20235825	-80,97615814	-946,0507202	0,034777
Cloud_Mesh_Distance_BA_R0_R3	18,22852707	-81,09272766	-946,0499268	0,027757
Cloud_Mesh_Distance_BA_R0_R3	17,93096924	-80,93993378	-946,02771	0,071074
Cloud_Mesh_Distance_BA_R0_R3	18,40559387	-80,94759369	-946,0616455	0,002493
Cloud_Mesh_Distance_BA_R0_R3	18,80518913	-81,02558136	-946,0684815	-0,052776
Cloud_Mesh_Distance_BA_R0_R3	19,08330154	-81,13391876	-946,0723267	-0,081424
Cloud_Mesh_Distance_BA_R0_R3	19,38120461	-81,19364166	-946,0645142	-0,097939
Cloud_Mesh_Distance_BA_R0_R3	19,55278397	-81,10604858	-946,0366821	-0,096801
Cloud_Mesh_Distance_BA_R0_R3	19,76095581	-81,13184357	-946,0092163	-0,100334
Cloud_Mesh_Distance_BA_R0_R3	19,91851234	-81,13327026	-945,9822388	-0,098197
Cloud_Mesh_Distance_BA_R0_R3	20,06703949	-81,29459381	-945,9800415	-0,097323
Cloud_Mesh_Distance_BA_R0_R3	20,64580154	-81,29097748	-945,8266602	-0,05062
Cloud_Mesh_Distance_BA_R0_R3	16,73722267	-80,74074554	-945,8126221	0,078238
Cloud_Mesh_Distance_BA_R0_R3	16,95338631	-80,89406586	-945,8266602	0,117304
Cloud_Mesh_Distance_BA_R0_R3	16,89298248	-80,78517151	-945,8397827	0,087995
Cloud_Mesh_Distance_BA_R0_R3	17,22580528	-80,71411896	-945,9354248	0,060963
Cloud_Mesh_Distance_BA_R0_R3	17,13362694	-80,80329132	-945,8891602	0,084129
Cloud_Mesh_Distance_BA_R0_R3	17,52933502	-80,89234161	-945,9605713	0,093291
Cloud_Mesh_Distance_BA_R0_R3	17,60769272	-80,71945953	-946,0092773	0,058644
Cloud_Mesh_Distance_BA_R0_R3	17,94650459	-80,77288055	-946,0494995	0,053736
Cloud_Mesh_Distance_BA_R0_R3	17,88096428	-80,6758194	-946,0609741	0,051308
Cloud_Mesh_Distance_BA_R0_R3	18,10193253	-80,80362701	-946,0591431	0,045394
Cloud_Mesh_Distance_BA_R0_R3	18,37194633	-80,83293915	-946,0703735	0,016673
Cloud_Mesh_Distance_BA_R0_R3	18,56980705	-80,98723602	-946,0640869	-0,019438
Cloud_Mesh_Distance_BA_R0_R3	19,03622055	-80,99214935	-946,06604	-0,064983
Cloud_Mesh_Distance_BA_R0_R3	18,99445724	-80,80672455	-946,0667725	-0,045979
Cloud_Mesh_Distance_BA_R0_R3	19,21175003	-81,0056839	-946,0587158	-0,078244
Cloud_Mesh_Distance_BA_R0_R3	19,37496567	-81,03742218	-946,0494385	-0,085568
Cloud_Mesh_Distance_BA_R0_R3	19,34815216	-80,89276886	-946,0415039	-0,076758
Cloud_Mesh_Distance_BA_R0_R3	19,70520401	-80,95815277	-945,9972534	-0,093597
Cloud_Mesh_Distance_BA_R0_R3	16,54720306	-80,36119843	-945,913147	-0,005193
Cloud_Mesh_Distance_BA_R0_R3	16,63617325	-80,39981842	-945,9296265	0,007584
Cloud_Mesh_Distance_BA_R0_R3	16,8440094	-80,59646606	-945,9005737	0,046251
Cloud_Mesh_Distance_BA_R0_R3	17,06059456	-80,68073273	-945,9136353	0,058028
Cloud_Mesh_Distance_BA_R0_R3	17,42674637	-80,53070831	-946,036377	0,033336
Cloud_Mesh_Distance_BA_R0_R3	17,40949249	-80,70800018	-945,9741211	0,057038

Cloud_Mesh_Distance_BA_R0_R3	17,76689148	-80,79965973	-946,020752	0,069182
Cloud_Mesh_Distance_BA_R0_R3	17,74522591	-80,62669373	-946,0542603	0,047845
Cloud_Mesh_Distance_BA_R0_R3	18,53709793	-80,82564545	-946,0738525	-0,005954
Cloud_Mesh_Distance_BA_R0_R3	18,77471352	-80,61728668	-946,0922852	-0,012864
Cloud_Mesh_Distance_BA_R0_R3	18,96757126	-80,56217194	-946,0843506	-0,019166
Cloud_Mesh_Distance_BA_R0_R3	18,75545883	-80,78749847	-946,0764771	-0,024278
Cloud_Mesh_Distance_BA_R0_R3	19,20275307	-80,8383255	-946,0531006	-0,063664
Cloud_Mesh_Distance_BA_R0_R3	19,20119095	-80,63765717	-946,0539551	-0,045894
Cloud_Mesh_Distance_BA_R0_R3	19,52674484	-80,88884735	-946,019104	-0,082019
Cloud_Mesh_Distance_BA_R0_R3	19,58537865	-80,74253082	-946,0002441	-0,075644
Cloud_Mesh_Distance_BA_R0_R3	16,53651428	-80,29919434	-945,9368286	-0,0139
Cloud_Mesh_Distance_BA_R0_R3	16,76914215	-80,43732452	-945,9527588	0,017564
Cloud_Mesh_Distance_BA_R0_R3	16,86751556	-80,34638214	-946,0181885	0,008192
Cloud_Mesh_Distance_BA_R0_R3	16,90681458	-80,44524384	-945,9832764	0,022538
Cloud_Mesh_Distance_BA_R0_R3	17,24762344	-80,56241608	-945,9945068	0,033632
Cloud_Mesh_Distance_BA_R0_R3	17,04539108	-80,50240326	-945,9844971	0,028346
Cloud_Mesh_Distance_BA_R0_R3	18,17816925	-80,35822296	-946,1589966	0,036112
Cloud_Mesh_Distance_BA_R0_R3	18,22489738	-80,4013443	-946,1486816	0,03485
Cloud_Mesh_Distance_BA_R0_R3	18,2504406	-80,3269577	-946,1674194	0,037716
Cloud_Mesh_Distance_BA_R0_R3	18,81836319	-80,50380707	-946,1049194	-0,002908
Cloud_Mesh_Distance_BA_R0_R3	18,75254631	-80,43601227	-946,1210327	0,009043
Cloud_Mesh_Distance_BA_R0_R3	19,21115112	-80,42946625	-946,0644531	-0,02709
Cloud_Mesh_Distance_BA_R0_R3	19,07686996	-80,35749817	-946,0918579	-0,011488
Cloud_Mesh_Distance_BA_R0_R3	19,39837074	-80,48802948	-946,0302734	-0,044253
Cloud_Mesh_Distance_BA_R0_R3	19,56939888	-80,53359222	-945,9974365	-0,060678
Cloud_Mesh_Distance_BA_R0_R3	19,42877197	-80,70648956	-946,0239258	-0,066598
Cloud_Mesh_Distance_BA_R0_R3	19,69848251	-80,58158112	-945,9708862	-0,070443
Cloud_Mesh_Distance_BA_R0_R3	19,7035656	-80,4487381	-945,966919	-0,058455
Cloud_Mesh_Distance_BA_R0_R3	19,79942703	-80,51477814	-945,9451904	-0,06599
Cloud_Mesh_Distance_BA_R0_R3	16,19761086	-79,91769409	-945,9303589	-0,075532
Cloud_Mesh_Distance_BA_R0_R3	16,04296112	-79,84455109	-945,8684692	-0,091544
Cloud_Mesh_Distance_BA_R0_R3	16,54233551	-80,10160065	-946,0218506	-0,040294
Cloud_Mesh_Distance_BA_R0_R3	16,38317871	-80,17485046	-945,9213867	-0,039288
Cloud_Mesh_Distance_BA_R0_R3	16,6053009	-79,98978424	-946,0857544	-0,046182
Cloud_Mesh_Distance_BA_R0_R3	16,68824768	-80,04098511	-946,0935059	-0,037491
Cloud_Mesh_Distance_BA_R0_R3	16,70437431	-79,95258331	-946,1286621	-0,048596
Cloud_Mesh_Distance_BA_R0_R3	18,12146759	-80,35005951	-946,1606445	0,032148
Cloud_Mesh_Distance_BA_R0_R3	18,89941597	-80,35124969	-946,1187744	0,006415
Cloud_Mesh_Distance_BA_R0_R3	19,22450447	-80,24286652	-946,0778809	-0,01244
Cloud_Mesh_Distance_BA_R0_R3	19,581213	-80,33879852	-945,9952393	-0,048986
Cloud_Mesh_Distance_BA_R0_R3	16,36022949	-80,01329803	-945,9772949	-0,059174
Cloud_Mesh_Distance_BA_R0_R3	16,13508606	-79,71935272	-945,9664917	-0,084611
Cloud_Mesh_Distance_BA_R0_R3	16,42858505	-79,70685577	-946,1109619	-0,076723
Cloud_Mesh_Distance_BA_R0_R3	16,48797798	-79,94624329	-946,0580444	-0,056118
Cloud_Mesh_Distance_BA_R0_R3	16,33594894	-79,83013153	-946,0322266	-0,071909
Cloud_Mesh_Distance_BA_R0_R3	16,60387421	-79,88799286	-946,1192627	-0,058295
Cloud_Mesh_Distance_BA_R0_R3	18,83667755	-80,10141754	-946,1671753	0,02834
Cloud_Mesh_Distance_BA_R0_R3	18,80734634	-80,23625946	-946,1488037	0,020174
Cloud_Mesh_Distance_BA_R0_R3	19,06196404	-80,02147675	-946,1366577	0,015475
Cloud_Mesh_Distance_BA_R0_R3	18,93831635	-80,15864563	-946,1419678	0,016303

Cloud_Mesh_Distance_BA_R0_R3	19,07297897	-80,19890594	-946,112793	0,003529
Cloud_Mesh_Distance_BA_R0_R3	19,22465515	-80,05910492	-946,0951538	-0,001418
Cloud_Mesh_Distance_BA_R0_R3	19,38769913	-80,28878021	-946,0410156	-0,030802
Cloud_Mesh_Distance_BA_R0_R3	19,53499222	-80,11286163	-946,0127564	-0,030476
Cloud_Mesh_Distance_BA_R0_R3	19,3600235	-80,12308502	-946,0570068	-0,017345
Cloud_Mesh_Distance_BA_R0_R3	19,7130127	-80,17980957	-945,9620361	-0,04555
Cloud_Mesh_Distance_BA_R0_R3	19,74137878	-80,32646942	-945,954895	-0,053282
Cloud_Mesh_Distance_BA_R0_R3	15,90000153	-79,61116028	-945,8551025	-0,091483
Cloud_Mesh_Distance_BA_R0_R3	15,98721313	-79,69684601	-945,8864746	-0,090454
Cloud_Mesh_Distance_BA_R0_R3	15,99475384	-79,53968048	-945,9355469	-0,093905
Cloud_Mesh_Distance_BA_R0_R3	16,27668762	-79,64919281	-946,0604858	-0,084005
Cloud_Mesh_Distance_BA_R0_R3	16,37376785	-79,580513	-946,1218872	-0,082639
Cloud_Mesh_Distance_BA_R0_R3	19,05986595	-79,88887024	-946,1525269	0,022038
Cloud_Mesh_Distance_BA_R0_R3	19,19721222	-79,85633087	-946,1199341	0,009188
Cloud_Mesh_Distance_BA_R0_R3	19,58547211	-79,93505096	-946,0023193	-0,026682
Cloud_Mesh_Distance_BA_R0_R3	15,87032604	-79,49207306	-945,8670044	-0,091463
Cloud_Mesh_Distance_BA_R0_R3	16,06604004	-79,36902618	-946,0132446	-0,095674
Cloud_Mesh_Distance_BA_R0_R3	16,17646599	-79,42343903	-946,0633545	-0,091329
Cloud_Mesh_Distance_BA_R0_R3	16,14678955	-79,53623199	-946,0238037	-0,090215
Cloud_Mesh_Distance_BA_R0_R3	19,084095	-79,78371429	-946,1565552	0,024048
Cloud_Mesh_Distance_BA_R0_R3	19,16793442	-79,63970947	-946,1410523	0,013088
Cloud_Mesh_Distance_BA_R0_R3	19,41514969	-79,91381073	-946,0551148	-0,011083
Cloud_Mesh_Distance_BA_R0_R3	15,90249634	-79,34249115	-945,9181519	-0,090942
Cloud_Mesh_Distance_BA_R0_R3	15,86836624	-79,15297699	-945,9180908	-0,091295
Cloud_Mesh_Distance_BA_R0_R3	15,99610329	-79,1869812	-945,9953613	-0,102733
Cloud_Mesh_Distance_BA_R0_R3	16,12821388	-79,2158432	-946,0669556	-0,101216
Cloud_Mesh_Distance_BA_R0_R3	19,15836334	-79,4220047	-946,1467285	0,006396
Cloud_Mesh_Distance_BA_R0_R3	19,48433685	-79,72614288	-946,0372925	-0,01639
Cloud_Mesh_Distance_BA_R0_R3	19,34802818	-79,69525146	-946,0830078	-0,002576
Cloud_Mesh_Distance_BA_R0_R3	19,47456169	-79,57015228	-946,0391846	-0,017788
Cloud_Mesh_Distance_BA_R0_R3	15,84331512	-79,02436066	-945,9075317	-0,095828
Cloud_Mesh_Distance_BA_R0_R3	15,9514122	-79,0120163	-945,9776611	-0,104269
Cloud_Mesh_Distance_BA_R0_R3	16,24427795	-79,11859894	-946,1318359	-0,107523
Cloud_Mesh_Distance_BA_R0_R3	19,01165771	-79,55376434	-946,1908569	0,027708
Cloud_Mesh_Distance_BA_R0_R3	19,33562279	-79,47451019	-946,086731	-0,008054
Cloud_Mesh_Distance_BA_R0_R3	19,2808876	-79,29923248	-946,0997925	-0,009885
Cloud_Mesh_Distance_BA_R0_R3	16,1069603	-79,02431488	-946,0661621	-0,115216
Cloud_Mesh_Distance_BA_R0_R3	16,05921555	-78,79947662	-946,0375366	-0,123371
Cloud_Mesh_Distance_BA_R0_R3	16,2445488	-78,96192169	-946,1368408	-0,12137
Cloud_Mesh_Distance_BA_R0_R3	18,97620201	-79,20671844	-946,2010498	0,011855
Cloud_Mesh_Distance_BA_R0_R3	18,99821472	-79,36373901	-946,1981201	0,019281
Cloud_Mesh_Distance_BA_R0_R3	19,02352333	-79,03445435	-946,1741943	-0,008292
Cloud_Mesh_Distance_BA_R0_R3	19,13957214	-79,20870209	-946,1459961	-0,003328
Cloud_Mesh_Distance_BA_R0_R3	19,33210373	-79,11255646	-946,0667725	-0,029719
Cloud_Mesh_Distance_BA_R0_R3	19,55633545	-79,22265625	-945,9856567	-0,041721
Cloud_Mesh_Distance_BA_R0_R3	19,43122482	-79,28343964	-946,0424194	-0,02672
Cloud_Mesh_Distance_BA_R0_R3	15,92065525	-78,68623352	-945,9476318	-0,105362
Cloud_Mesh_Distance_BA_R0_R3	16,20516396	-78,61477661	-946,0997925	-0,141348
Cloud_Mesh_Distance_BA_R0_R3	16,30364227	-78,65762329	-946,1501465	-0,142945
Cloud_Mesh_Distance_BA_R0_R3	18,86332512	-78,96079254	-946,2214356	-0,00111

Cloud_Mesh_Distance_BA_R0_R3	19,13541794	-78,84662628	-946,1140137	-0,03422
Cloud_Mesh_Distance_BA_R0_R3	19,18901253	-79,02318573	-946,112915	-0,022297
Cloud_Mesh_Distance_BA_R0_R3	16,1167717	-78,35958099	-946,0159302	-0,129203
Cloud_Mesh_Distance_BA_R0_R3	15,99900723	-78,29528809	-945,9331055	-0,107225
Cloud_Mesh_Distance_BA_R0_R3	16,08068657	-78,55834961	-946,0286255	-0,126298
Cloud_Mesh_Distance_BA_R0_R3	18,84716415	-78,78650665	-946,210022	-0,013483
Cloud_Mesh_Distance_BA_R0_R3	18,97755051	-78,86225891	-946,1732178	-0,019256
Cloud_Mesh_Distance_BA_R0_R3	19,30345917	-78,91007233	-946,0563965	-0,043615
Cloud_Mesh_Distance_BA_R0_R3	16,0574913	-78,15158844	-945,9282837	-0,114293
Cloud_Mesh_Distance_BA_R0_R3	16,27639008	-78,22806549	-946,0709839	-0,141641
Cloud_Mesh_Distance_BA_R0_R3	16,50223541	-78,28562927	-946,1834106	-0,158219
Cloud_Mesh_Distance_BA_R0_R3	16,25881958	-78,413414	-946,0994873	-0,144683
Cloud_Mesh_Distance_BA_R0_R3	18,82432175	-78,59624481	-946,196167	-0,025046
Cloud_Mesh_Distance_BA_R0_R3	19,110569	-78,5176239	-946,0794067	-0,052519
Cloud_Mesh_Distance_BA_R0_R3	19,00354004	-78,67562103	-946,1420288	-0,035261
Cloud_Mesh_Distance_BA_R0_R3	16,15109253	-78,19239044	-945,9957275	-0,128886
Cloud_Mesh_Distance_BA_R0_R3	16,16605949	-78,0775528	-945,9690552	-0,129846
Cloud_Mesh_Distance_BA_R0_R3	16,4372139	-78,10276794	-946,1136475	-0,151721
Cloud_Mesh_Distance_BA_R0_R3	16,40350914	-78,25795746	-946,1364136	-0,15367
Cloud_Mesh_Distance_BA_R0_R3	16,54956055	-78,15609741	-946,1748047	-0,152534
Cloud_Mesh_Distance_BA_R0_R3	18,38229752	-78,24105072	-946,2763062	-0,014494
Cloud_Mesh_Distance_BA_R0_R3	18,51102638	-78,32025146	-946,2554321	-0,015557
Cloud_Mesh_Distance_BA_R0_R3	18,76783371	-78,40969086	-946,1898193	-0,026919
Cloud_Mesh_Distance_BA_R0_R3	18,66410828	-78,22890472	-946,1934815	-0,025893
Cloud_Mesh_Distance_BA_R0_R3	18,94818115	-78,44248199	-946,1306763	-0,041133
Cloud_Mesh_Distance_BA_R0_R3	19,07284546	-78,36649323	-946,0695801	-0,054168
Cloud_Mesh_Distance_BA_R0_R3	16,30127907	-78,05876923	-946,0357666	-0,144142
Cloud_Mesh_Distance_BA_R0_R3	16,50249863	-77,92144775	-946,0835571	-0,151289
Cloud_Mesh_Distance_BA_R0_R3	18,33122635	-78,08353424	-946,2606201	-0,011771
Cloud_Mesh_Distance_BA_R0_R3	18,49334335	-78,13944244	-946,2292481	-0,015674
Cloud_Mesh_Distance_BA_R0_R3	18,44627762	-77,94499969	-946,1962891	-0,020258
Cloud_Mesh_Distance_BA_R0_R3	18,76824188	-78,05899811	-946,1203003	-0,03801
Cloud_Mesh_Distance_BA_R0_R3	18,60223198	-78,04391479	-946,1738892	-0,024088
Cloud_Mesh_Distance_BA_R0_R3	18,98403931	-78,28199768	-946,0892334	-0,049467
Cloud_Mesh_Distance_BA_R0_R3	18,8485527	-78,25679016	-946,1346436	-0,039374
Cloud_Mesh_Distance_BA_R0_R3	16,98001862	-77,76683044	-946,197876	-0,121625
Cloud_Mesh_Distance_BA_R0_R3	18,29917908	-77,76892853	-946,1821289	-0,016238
Cloud_Mesh_Distance_BA_R0_R3	18,89008713	-77,99526978	-946,0567627	-0,049869
Cloud_Mesh_Distance_BA_R0_R3	16,77972984	-77,62676239	-946,0703125	-0,12937
Cloud_Mesh_Distance_BA_R0_R3	16,69131088	-77,57205963	-946,0055542	-0,141702
Cloud_Mesh_Distance_BA_R0_R3	16,81259155	-77,51867676	-946,0256348	-0,130027
Cloud_Mesh_Distance_BA_R0_R3	16,93373489	-77,51980591	-946,0692749	-0,120769
Cloud_Mesh_Distance_BA_R0_R3	17,12801361	-77,63205719	-946,178833	-0,102556
Cloud_Mesh_Distance_BA_R0_R3	16,94673729	-77,63952637	-946,1340942	-0,118221
Cloud_Mesh_Distance_BA_R0_R3	17,54584503	-77,52067566	-946,1823731	-0,055838
Cloud_Mesh_Distance_BA_R0_R3	17,59495926	-77,65657806	-946,2419434	-0,048006
Cloud_Mesh_Distance_BA_R0_R3	17,73519516	-77,54734039	-946,192688	-0,036908
Cloud_Mesh_Distance_BA_R0_R3	17,89347839	-77,59233856	-946,1977539	-0,024168
Cloud_Mesh_Distance_BA_R0_R3	17,77679443	-77,68331146	-946,2461548	-0,031669
Cloud_Mesh_Distance_BA_R0_R3	18,09262466	-77,59172821	-946,1623535	-0,018452

Cloud_Mesh_Distance_BA_R0_R3	17,95482635	-77,71534729	-946,2370606	-0,020456
Cloud_Mesh_Distance_BA_R0_R3	18,10989761	-77,75826263	-946,2252197	-0,014189
Cloud_Mesh_Distance_BA_R0_R3	18,30515289	-77,59809875	-946,1098633	-0,023418
Cloud_Mesh_Distance_BA_R0_R3	18,75351715	-77,81252289	-946,0461426	-0,045401
Cloud_Mesh_Distance_BA_R0_R3	18,48846817	-77,69389343	-946,0938721	-0,027828
Cloud_Mesh_Distance_BA_R0_R3	18,62348557	-77,87168884	-946,1158447	-0,031153
Cloud_Mesh_Distance_BA_R0_R3	18,46632385	-77,81496429	-946,149292	-0,020367
Cloud_Mesh_Distance_BA_R0_R3	17,10377121	-77,51052094	-946,1139526	-0,109212
Cloud_Mesh_Distance_BA_R0_R3	17,3223877	-77,50774384	-946,1560059	-0,084544
Cloud_Mesh_Distance_BA_R0_R3	17,68762016	-77,41545105	-946,1288452	-0,046754
Cloud_Mesh_Distance_BA_R0_R3	17,50756073	-77,39575958	-946,1152954	-0,064131
Cloud_Mesh_Distance_BA_R0_R3	17,89256668	-77,4576416	-946,133667	-0,032657
Cloud_Mesh_Distance_BA_R0_R3	18,21048737	-77,46297455	-946,0685425	-0,030259
Cloud_Mesh_Distance_BA_R0_R3	18,65573883	-77,7345047	-946,0512695	-0,042643
Cloud_Mesh_Distance_BA_R0_R3	16,35569	-81,4787674	-945,6775513	0,311924
Cloud_Mesh_Distance_BA_R0_R3	16,56845856	-81,47170258	-945,7738037	0,228824
Cloud_Mesh_Distance_BA_R0_R3	16,41532898	-81,24864197	-945,6165161	0,292232
Cloud_Mesh_Distance_BA_R0_R3	16,54233932	-81,31378937	-945,6974487	0,29168
Cloud_Mesh_Distance_BA_R0_R3	16,71338272	-81,48329926	-945,8341675	0,187516
Cloud_Mesh_Distance_BA_R0_R3	16,85148621	-81,44600677	-945,8617554	0,155318
Cloud_Mesh_Distance_BA_R0_R3	20,74133301	-81,78250885	-945,8839722	-0,13154
Cloud_Mesh_Distance_BA_R0_R3	20,99831772	-81,91666412	-945,8053589	-0,092064
Cloud_Mesh_Distance_BA_R0_R3	21,2162056	-81,90331268	-945,692749	-0,158173
Cloud_Mesh_Distance_BA_R0_R3	21,10629654	-81,85022736	-945,7356567	-0,079322
Cloud_Mesh_Distance_BA_R0_R3	16,76857948	-81,3404007	-945,7942505	0,227791
Cloud_Mesh_Distance_BA_R0_R3	16,5521965	-81,13950348	-945,6712647	0,230285
Cloud_Mesh_Distance_BA_R0_R3	16,98598099	-81,18582916	-945,8261108	0,2124
Cloud_Mesh_Distance_BA_R0_R3	16,7741642	-81,11943054	-945,7561646	0,208737
Cloud_Mesh_Distance_BA_R0_R3	20,49116516	-81,77648163	-945,967041	-0,204316
Cloud_Mesh_Distance_BA_R0_R3	20,48796654	-81,67578888	-945,9505005	-0,116949
Cloud_Mesh_Distance_BA_R0_R3	20,59646225	-81,71446991	-945,9231567	-0,127034
Cloud_Mesh_Distance_BA_R0_R3	20,90472984	-81,79447174	-945,819519	-0,06843
Cloud_Mesh_Distance_BA_R0_R3	16,26385117	-81,01784515	-945,508728	0,141692
Cloud_Mesh_Distance_BA_R0_R3	16,42442513	-81,13718414	-945,6066895	0,225359
Cloud_Mesh_Distance_BA_R0_R3	16,49057579	-80,99397278	-945,6479492	0,157064
Cloud_Mesh_Distance_BA_R0_R3	16,87666321	-80,97986603	-945,7927856	0,149221
Cloud_Mesh_Distance_BA_R0_R3	16,78882027	-80,97616577	-945,7677612	0,150032
Cloud_Mesh_Distance_BA_R0_R3	17,16367149	-81,04447937	-945,8627319	0,147082
Cloud_Mesh_Distance_BA_R0_R3	20,40897751	-81,29708099	-945,9033203	-0,069279
Cloud_Mesh_Distance_BA_R0_R3	20,71853828	-81,4131546	-945,8231201	-0,052513
Cloud_Mesh_Distance_BA_R0_R3	20,6998291	-81,59719086	-945,8666992	-0,062777
Cloud_Mesh_Distance_BA_R0_R3	20,82116318	-81,51070404	-945,8000488	-0,052471
Cloud_Mesh_Distance_BA_R0_R3	20,85002899	-81,64698029	-945,8134155	-0,058488
Cloud_Mesh_Distance_BA_R0_R3	16,2375412	-80,89948273	-945,5096436	0,083327
Cloud_Mesh_Distance_BA_R0_R3	16,3539753	-80,93019867	-945,5819092	0,114282
Cloud_Mesh_Distance_BA_R0_R3	16,46671295	-80,80473328	-945,6791382	0,081765
Cloud_Mesh_Distance_BA_R0_R3	16,66579437	-80,99538422	-945,7232666	0,163095
Cloud_Mesh_Distance_BA_R0_R3	16,62052727	-80,85196686	-945,7349854	0,110712
Cloud_Mesh_Distance_BA_R0_R3	16,99795341	-81,00307465	-945,8223267	0,149505
Cloud_Mesh_Distance_BA_R0_R3	17,27500916	-80,93410492	-945,8961792	0,111417

Cloud_Mesh_Distance_BA_R0_R3	17,29588699	-81,0166626	-945,895752	0,13045
Cloud_Mesh_Distance_BA_R0_R3	20,17273521	-81,17867279	-945,937439	-0,087408
Cloud_Mesh_Distance_BA_R0_R3	20,01928711	-81,1927948	-945,9719849	-0,097266
Cloud_Mesh_Distance_BA_R0_R3	20,21126747	-81,03070068	-945,9015503	-0,082907
Cloud_Mesh_Distance_BA_R0_R3	20,44843674	-81,18075562	-945,867981	-0,065632
Cloud_Mesh_Distance_BA_R0_R3	20,31628799	-81,24446869	-945,9174194	-0,077814
Cloud_Mesh_Distance_BA_R0_R3	20,5201149	-81,28845978	-945,8691406	-0,060923
Cloud_Mesh_Distance_BA_R0_R3	16,31900024	-80,77320099	-945,6035156	0,051998
Cloud_Mesh_Distance_BA_R0_R3	16,23121262	-80,64243317	-945,5982056	-0,000496
Cloud_Mesh_Distance_BA_R0_R3	16,59698868	-80,69345093	-945,7798462	0,060581
Cloud_Mesh_Distance_BA_R0_R3	17,02216721	-80,80895996	-945,8634644	0,088812
Cloud_Mesh_Distance_BA_R0_R3	17,05535126	-80,88419342	-945,852478	0,107393
Cloud_Mesh_Distance_BA_R0_R3	17,15243912	-80,91440582	-945,8704224	0,112018
Cloud_Mesh_Distance_BA_R0_R3	17,2651062	-80,82184601	-945,913208	0,08183
Cloud_Mesh_Distance_BA_R0_R3	19,87545395	-80,98865509	-945,9701538	-0,092905
Cloud_Mesh_Distance_BA_R0_R3	20,05260086	-81,03617859	-945,939209	-0,092516
Cloud_Mesh_Distance_BA_R0_R3	20,32850266	-81,0978775	-945,8841553	-0,075607
Cloud_Mesh_Distance_BA_R0_R3	20,32187843	-80,93709564	-945,854187	-0,079273
Cloud_Mesh_Distance_BA_R0_R3	20,64825439	-80,97277069	-945,7508545	-0,061472
Cloud_Mesh_Distance_BA_R0_R3	20,61501122	-81,14200592	-945,803772	-0,057301
Cloud_Mesh_Distance_BA_R0_R3	20,80073929	-81,19997406	-945,7396851	-0,050358
Cloud_Mesh_Distance_BA_R0_R3	16,14167786	-80,33005524	-945,6979981	-0,057642
Cloud_Mesh_Distance_BA_R0_R3	16,00727081	-80,27108765	-945,6328735	-0,081706
Cloud_Mesh_Distance_BA_R0_R3	16,06319809	-80,41944122	-945,5888672	-0,068427
Cloud_Mesh_Distance_BA_R0_R3	16,17237091	-80,46942902	-945,6431274	-0,040704
Cloud_Mesh_Distance_BA_R0_R3	16,27620316	-80,41855621	-945,7409058	-0,02866
Cloud_Mesh_Distance_BA_R0_R3	16,25611115	-80,53176117	-945,6676025	-0,018455
Cloud_Mesh_Distance_BA_R0_R3	16,4148407	-80,57834625	-945,7424927	0,016154
Cloud_Mesh_Distance_BA_R0_R3	16,53164864	-80,44524384	-945,8662109	0,007705
Cloud_Mesh_Distance_BA_R0_R3	16,70423698	-80,64237213	-945,8406372	0,054458
Cloud_Mesh_Distance_BA_R0_R3	16,64037704	-80,54216766	-945,8632813	0,032963
Cloud_Mesh_Distance_BA_R0_R3	19,69242477	-80,79645538	-945,9842529	-0,084375
Cloud_Mesh_Distance_BA_R0_R3	19,81975555	-80,84701538	-945,9647217	-0,084903
Cloud_Mesh_Distance_BA_R0_R3	19,98957443	-80,82738495	-945,9247437	-0,086943
Cloud_Mesh_Distance_BA_R0_R3	20,17065048	-80,8919754	-945,8882446	-0,083515
Cloud_Mesh_Distance_BA_R0_R3	20,28228569	-80,7947464	-945,8409424	-0,08064
Cloud_Mesh_Distance_BA_R0_R3	20,57660294	-80,86469269	-945,7546997	-0,069647
Cloud_Mesh_Distance_BA_R0_R3	20,48534012	-81,00040436	-945,8163452	-0,066632
Cloud_Mesh_Distance_BA_R0_R3	16,42835617	-80,34627533	-945,8641968	-0,016529
Cloud_Mesh_Distance_BA_R0_R3	19,6815567	-80,68541718	-945,9790649	-0,078901
Cloud_Mesh_Distance_BA_R0_R3	19,59405136	-80,64767456	-945,9946899	-0,071277
Cloud_Mesh_Distance_BA_R0_R3	19,83063889	-80,66978455	-945,9467773	-0,077107
Cloud_Mesh_Distance_BA_R0_R3	20,15430069	-80,71625519	-945,8667603	-0,081513
Cloud_Mesh_Distance_BA_R0_R3	20,01287651	-80,63991547	-945,8983765	-0,08195
Cloud_Mesh_Distance_BA_R0_R3	19,93670273	-80,47466278	-945,9067993	-0,072455
Cloud_Mesh_Distance_BA_R0_R3	20,31521225	-80,62656403	-945,8048706	-0,079813
Cloud_Mesh_Distance_BA_R0_R3	20,48472595	-80,59252167	-945,7376099	-0,077962
Cloud_Mesh_Distance_BA_R0_R3	20,58999634	-80,72602081	-945,7200928	-0,073232
Cloud_Mesh_Distance_BA_R0_R3	20,43872643	-80,79355621	-945,7900391	-0,074382
Cloud_Mesh_Distance_BA_R0_R3	15,73983955	-79,96511078	-945,5888672	-0,101344

Cloud_Mesh_Distance_BA_R0_R3	15,79447746	-80,07953644	-945,5734253	-0,103009
Cloud_Mesh_Distance_BA_R0_R3	15,9230032	-80,1060257	-945,6620483	-0,093626
Cloud_Mesh_Distance_BA_R0_R3	15,97241783	-79,96405792	-945,7711182	-0,089827
Cloud_Mesh_Distance_BA_R0_R3	16,10303497	-79,99360657	-945,843689	-0,079447
Cloud_Mesh_Distance_BA_R0_R3	16,22845078	-80,08558655	-945,8771973	-0,063457
Cloud_Mesh_Distance_BA_R0_R3	16,08727264	-80,14517975	-945,7595215	-0,078328
Cloud_Mesh_Distance_BA_R0_R3	16,25510406	-80,2519455	-945,8140869	-0,047164
Cloud_Mesh_Distance_BA_R0_R3	19,81483078	-80,40496826	-945,9373169	-0,061678
Cloud_Mesh_Distance_BA_R0_R3	19,87784004	-80,26448822	-945,9143677	-0,060306
Cloud_Mesh_Distance_BA_R0_R3	20,19901085	-80,35207367	-945,8145752	-0,072927
Cloud_Mesh_Distance_BA_R0_R3	20,05968857	-80,32055664	-945,8602295	-0,068526
Cloud_Mesh_Distance_BA_R0_R3	20,14224625	-80,51183319	-945,8477783	-0,075296
Cloud_Mesh_Distance_BA_R0_R3	20,30148315	-80,44947815	-945,7875977	-0,076137
Cloud_Mesh_Distance_BA_R0_R3	15,8146925	-79,92188263	-945,6738892	-0,099307
Cloud_Mesh_Distance_BA_R0_R3	15,67671585	-79,84407806	-945,5933838	-0,097069
Cloud_Mesh_Distance_BA_R0_R3	20,01576614	-80,12387848	-945,8634033	-0,059216
Cloud_Mesh_Distance_BA_R0_R3	19,84122467	-80,02477264	-945,9195557	-0,048912
Cloud_Mesh_Distance_BA_R0_R3	20,16969872	-80,19658661	-945,8121338	-0,067737
Cloud_Mesh_Distance_BA_R0_R3	15,60278988	-79,60749054	-945,6170654	-0,08823
Cloud_Mesh_Distance_BA_R0_R3	15,73995209	-79,7250061	-945,6987305	-0,093193
Cloud_Mesh_Distance_BA_R0_R3	15,66303825	-79,57169342	-945,6845093	-0,088729
Cloud_Mesh_Distance_BA_R0_R3	15,88628864	-79,76216125	-945,795044	-0,094382
Cloud_Mesh_Distance_BA_R0_R3	15,78465462	-79,56267548	-945,7865601	-0,092574
Cloud_Mesh_Distance_BA_R0_R3	19,68144989	-80,01540375	-945,9719849	-0,036696
Cloud_Mesh_Distance_BA_R0_R3	15,7966938	-79,40337372	-945,8328857	-0,088098
Cloud_Mesh_Distance_BA_R0_R3	19,56471252	-79,80928802	-946,0106201	-0,023869
Cloud_Mesh_Distance_BA_R0_R3	19,61086655	-79,64919281	-945,991394	-0,027673
Cloud_Mesh_Distance_BA_R0_R3	19,74212646	-79,64473724	-945,9412842	-0,040864
Cloud_Mesh_Distance_BA_R0_R3	19,71779251	-79,82776642	-945,9567871	-0,038482
Cloud_Mesh_Distance_BA_R0_R3	19,87068939	-79,84156799	-945,9020386	-0,045899
Cloud_Mesh_Distance_BA_R0_R3	19,82963753	-79,71173096	-945,9104004	-0,045038
Cloud_Mesh_Distance_BA_R0_R3	19,99198532	-79,92502594	-945,8609619	-0,055201
Cloud_Mesh_Distance_BA_R0_R3	15,67738247	-79,372612	-945,7446899	-0,084866
Cloud_Mesh_Distance_BA_R0_R3	15,67649746	-79,12187195	-945,7749023	-0,078404
Cloud_Mesh_Distance_BA_R0_R3	15,77702236	-79,24897003	-945,8435059	-0,084189
Cloud_Mesh_Distance_BA_R0_R3	15,78067493	-79,10351563	-945,8591309	-0,087309
Cloud_Mesh_Distance_BA_R0_R3	19,84897232	-79,52935028	-945,8887329	-0,05189
Cloud_Mesh_Distance_BA_R0_R3	19,96276093	-79,72364044	-945,857605	-0,054628
Cloud_Mesh_Distance_BA_R0_R3	20,11083221	-79,82750702	-945,8058472	-0,060249
Cloud_Mesh_Distance_BA_R0_R3	15,66547108	-78,91371918	-945,7699585	-0,072048
Cloud_Mesh_Distance_BA_R0_R3	15,76513767	-78,97285461	-945,8516846	-0,084151
Cloud_Mesh_Distance_BA_R0_R3	19,55095291	-79,33614349	-945,9976196	-0,035822
Cloud_Mesh_Distance_BA_R0_R3	19,58660507	-79,46321869	-945,9924316	-0,030537
Cloud_Mesh_Distance_BA_R0_R3	19,47598648	-79,42359161	-946,0338135	-0,021869
Cloud_Mesh_Distance_BA_R0_R3	19,69926453	-79,5147934	-945,9504395	-0,041133
Cloud_Mesh_Distance_BA_R0_R3	19,70672607	-79,29888153	-945,9281616	-0,050039
Cloud_Mesh_Distance_BA_R0_R3	19,99462509	-79,57276154	-945,8297119	-0,059197
Cloud_Mesh_Distance_BA_R0_R3	19,97053528	-79,44704437	-945,8259888	-0,063
Cloud_Mesh_Distance_BA_R0_R3	15,87959576	-78,88153839	-945,9324341	-0,098621
Cloud_Mesh_Distance_BA_R0_R3	15,77978992	-78,79580688	-945,8578491	-0,08968

Cloud_Mesh_Distance_BA_R0_R3	19,6108017	-79,07894135	-945,9467773	-0,056203
Cloud_Mesh_Distance_BA_R0_R3	19,74251366	-79,08001709	-945,8864136	-0,062277
Cloud_Mesh_Distance_BA_R0_R3	15,70713139	-78,53325653	-945,7685547	-0,067379
Cloud_Mesh_Distance_BA_R0_R3	15,67730904	-78,72193146	-945,7697144	-0,065575
Cloud_Mesh_Distance_BA_R0_R3	15,58644295	-78,64243317	-945,6729736	-0,05408
Cloud_Mesh_Distance_BA_R0_R3	15,62494946	-78,46588135	-945,6763916	-0,052717
Cloud_Mesh_Distance_BA_R0_R3	15,80828476	-78,60370636	-945,8607788	-0,086783
Cloud_Mesh_Distance_BA_R0_R3	19,60745239	-78,94696808	-945,9316406	-0,060116
Cloud_Mesh_Distance_BA_R0_R3	19,48356819	-79,12619019	-946,007019	-0,040609
Cloud_Mesh_Distance_BA_R0_R3	15,65878487	-78,35475159	-945,678772	-0,053535
Cloud_Mesh_Distance_BA_R0_R3	15,73412704	-78,36330414	-945,753479	-0,065406
Cloud_Mesh_Distance_BA_R0_R3	15,95643425	-78,48369598	-945,9441528	-0,105544
Cloud_Mesh_Distance_BA_R0_R3	19,27346992	-78,74034882	-946,0465088	-0,051112
Cloud_Mesh_Distance_BA_R0_R3	19,38306427	-78,64189148	-945,9863892	-0,062672
Cloud_Mesh_Distance_BA_R0_R3	19,50693893	-78,72898865	-945,9450684	-0,067166
Cloud_Mesh_Distance_BA_R0_R3	19,39267159	-78,79314423	-946,0037842	-0,05538
Cloud_Mesh_Distance_BA_R0_R3	19,4858284	-78,93963623	-945,984375	-0,054868
Cloud_Mesh_Distance_BA_R0_R3	19,6385231	-78,82344818	-945,8994751	-0,067094
Cloud_Mesh_Distance_BA_R0_R3	19,69332123	-78,95145416	-945,8925171	-0,063412
Cloud_Mesh_Distance_BA_R0_R3	19,73801804	-78,85655975	-945,8572998	-0,070439
Cloud_Mesh_Distance_BA_R0_R3	15,84397697	-78,40962982	-945,8538818	-0,083425
Cloud_Mesh_Distance_BA_R0_R3	19,15803146	-78,67138672	-946,0831299	-0,0472
Cloud_Mesh_Distance_BA_R0_R3	19,25215912	-78,56984711	-946,0299072	-0,060307
Cloud_Mesh_Distance_BA_R0_R3	19,49534607	-78,54810333	-945,9198608	-0,074081
Cloud_Mesh_Distance_BA_R0_R3	15,94867134	-77,99977112	-945,7955933	-0,096347
Cloud_Mesh_Distance_BA_R0_R3	15,89251328	-78,18943787	-945,8284302	-0,082214
Cloud_Mesh_Distance_BA_R0_R3	19,20828247	-78,37812042	-946,0158081	-0,064545
Cloud_Mesh_Distance_BA_R0_R3	19,36045837	-78,46395111	-945,9659424	-0,070203
Cloud_Mesh_Distance_BA_R0_R3	19,21406174	-78,24155426	-945,9849243	-0,070835
Cloud_Mesh_Distance_BA_R0_R3	19,3272438	-78,27344513	-945,9420166	-0,075845
Cloud_Mesh_Distance_BA_R0_R3	15,883358	-77,95010376	-945,7167969	-0,080709
Cloud_Mesh_Distance_BA_R0_R3	16,0242424	-77,83067322	-945,7630615	-0,105347
Cloud_Mesh_Distance_BA_R0_R3	16,23519897	-77,98209381	-945,9732666	-0,135736
Cloud_Mesh_Distance_BA_R0_R3	16,08897018	-78,01526642	-945,8988037	-0,119784
Cloud_Mesh_Distance_BA_R0_R3	16,27441978	-77,84116364	-945,934021	-0,140244
Cloud_Mesh_Distance_BA_R0_R3	16,34652328	-77,92879486	-946,0110474	-0,144922
Cloud_Mesh_Distance_BA_R0_R3	18,93860626	-78,13724518	-946,0750122	-0,048456
Cloud_Mesh_Distance_BA_R0_R3	19,21264458	-78,10346222	-945,9519653	-0,07194
Cloud_Mesh_Distance_BA_R0_R3	19,09712219	-78,21792603	-946,029419	-0,061257
Cloud_Mesh_Distance_BA_R0_R3	16,07061768	-77,88288116	-945,826355	-0,116285
Cloud_Mesh_Distance_BA_R0_R3	16,44846725	-77,5094223	-945,8430176	-0,139737
Cloud_Mesh_Distance_BA_R0_R3	16,37682343	-77,65080261	-945,8907471	-0,14228
Cloud_Mesh_Distance_BA_R0_R3	16,54285431	-77,72386932	-946,0178223	-0,150285
Cloud_Mesh_Distance_BA_R0_R3	16,41733551	-77,85211945	-946,0154419	-0,146887
Cloud_Mesh_Distance_BA_R0_R3	18,79114151	-77,91378021	-946,0690918	-0,043065
Cloud_Mesh_Distance_BA_R0_R3	19,02946854	-78,03504181	-946,0117798	-0,06298
Cloud_Mesh_Distance_BA_R0_R3	19,31639862	-78,0437088	-945,8860474	-0,083284
Cloud_Mesh_Distance_BA_R0_R3	16,59143448	-77,42855072	-945,8675537	-0,135072
Cloud_Mesh_Distance_BA_R0_R3	16,49184036	-77,42432404	-945,8093872	-0,13406
Cloud_Mesh_Distance_BA_R0_R3	16,56648064	-77,5533371	-945,93573	-0,14439

Cloud_Mesh_Distance_BA_R0_R3	16,89976311	-77,43470001	-946,0084839	-0,122626
Cloud_Mesh_Distance_BA_R0_R3	18,91628647	-77,85488129	-945,998291	-0,057798
Cloud_Mesh_Distance_BA_R0_R3	18,78091812	-77,7100296	-945,9919434	-0,054893
Cloud_Mesh_Distance_BA_R0_R3	19,23538971	-77,97014618	-945,9002075	-0,078292
Cloud_Mesh_Distance_BA_R0_R3	19,10980988	-77,92795563	-945,9424439	-0,07088
Cloud_Mesh_Distance_BA_R0_R3	16,70212364	-77,2269516	-945,7738648	-0,139415
Cloud_Mesh_Distance_BA_R0_R3	16,7491951	-77,44416809	-945,9529419	-0,139833
Cloud_Mesh_Distance_BA_R0_R3	16,73296165	-77,31780243	-945,859375	-0,13641
Cloud_Mesh_Distance_BA_R0_R3	17,04893684	-77,40131378	-946,0370483	-0,112362
Cloud_Mesh_Distance_BA_R0_R3	16,94665718	-77,29651642	-945,9331665	-0,125256
Cloud_Mesh_Distance_BA_R0_R3	17,16995811	-77,27867889	-945,9899902	-0,10426
Cloud_Mesh_Distance_BA_R0_R3	17,2305851	-77,38652802	-946,0729981	-0,092509
Cloud_Mesh_Distance_BA_R0_R3	17,37897682	-77,36927032	-946,0878296	-0,078206
Cloud_Mesh_Distance_BA_R0_R3	17,4746666	-77,30060577	-946,0560303	-0,072376
Cloud_Mesh_Distance_BA_R0_R3	18,0524292	-77,43553162	-946,093689	-0,029305
Cloud_Mesh_Distance_BA_R0_R3	18,44783783	-77,36785126	-945,9337158	-0,046596
Cloud_Mesh_Distance_BA_R0_R3	18,4267807	-77,49817657	-946,0178223	-0,039351
Cloud_Mesh_Distance_BA_R0_R3	18,63115692	-77,60482025	-945,9986572	-0,049892
Cloud_Mesh_Distance_BA_R0_R3	18,81411362	-77,57038116	-945,9065552	-0,06984
Cloud_Mesh_Distance_BA_R0_R3	18,91457367	-77,70571136	-945,9348145	-0,06758
Cloud_Mesh_Distance_BA_R0_R3	19,16953087	-77,71268463	-945,8257446	-0,087916
Cloud_Mesh_Distance_BA_R0_R3	19,20259857	-77,8521347	-945,8720703	-0,082242
Cloud_Mesh_Distance_BA_R0_R3	19,05404663	-77,77738953	-945,9074097	-0,07635
Cloud_Mesh_Distance_BA_R0_R3	16,84781647	-77,19586945	-945,8182373	-0,131734
Cloud_Mesh_Distance_BA_R0_R3	17,12871552	-77,16759491	-945,8973999	-0,108702
Cloud_Mesh_Distance_BA_R0_R3	17,50793839	-77,18706512	-945,9814453	-0,071949
Cloud_Mesh_Distance_BA_R0_R3	17,3472023	-77,27732086	-946,024353	-0,084824
Cloud_Mesh_Distance_BA_R0_R3	17,30965042	-77,1671524	-945,9406738	-0,0949
Cloud_Mesh_Distance_BA_R0_R3	17,72943115	-77,18560028	-945,984436	-0,052577
Cloud_Mesh_Distance_BA_R0_R3	17,62104607	-77,29460907	-946,0592041	-0,055507
Cloud_Mesh_Distance_BA_R0_R3	17,96151733	-77,35045624	-946,0632324	-0,031154
Cloud_Mesh_Distance_BA_R0_R3	17,80856895	-77,31603241	-946,0637817	-0,03804
Cloud_Mesh_Distance_BA_R0_R3	18,09972572	-77,3380661	-946,0266113	-0,030825
Cloud_Mesh_Distance_BA_R0_R3	17,94996834	-77,225914	-945,9876099	-0,036899
Cloud_Mesh_Distance_BA_R0_R3	18,37715149	-77,27854156	-945,902771	-0,048804
Cloud_Mesh_Distance_BA_R0_R3	18,26980591	-77,23429871	-945,911377	-0,04377
Cloud_Mesh_Distance_BA_R0_R3	18,28147316	-77,34247589	-945,9767456	-0,037764
Cloud_Mesh_Distance_BA_R0_R3	17,43494987	-77,07561493	-945,8904419	-0,082491
Cloud_Mesh_Distance_BA_R0_R3	17,62882614	-77,04664612	-945,8833008	-0,063449
Cloud_Mesh_Distance_BA_R0_R3	18,05190468	-77,11322784	-945,8898315	-0,035606
Cloud_Mesh_Distance_BA_R0_R3	18,14160156	-77,23241425	-945,9487305	-0,03876
Cloud_Mesh_Distance_BA_R0_R3	21,59967422	-82,0358963	-945,5096436	-0,111685
Cloud_Mesh_Distance_BA_R0_R3	16,09880638	-81,34655762	-945,4248047	0,337604
Cloud_Mesh_Distance_BA_R0_R3	16,36244583	-81,35768127	-945,6210327	0,333581
Cloud_Mesh_Distance_BA_R0_R3	21,19306564	-81,74443817	-945,6594238	-0,05618
Cloud_Mesh_Distance_BA_R0_R3	16,23589134	-81,1383667	-945,4859619	0,205806
Cloud_Mesh_Distance_BA_R0_R3	16,12599945	-81,20122528	-945,4025879	0,226041
Cloud_Mesh_Distance_BA_R0_R3	16,21453476	-81,27455902	-945,4926148	0,295388
Cloud_Mesh_Distance_BA_R0_R3	16,30258179	-81,30574799	-945,5643921	0,327786
Cloud_Mesh_Distance_BA_R0_R3	21,07900047	-81,73035431	-945,7197266	-0,057521

Cloud_Mesh_Distance_BA_R0_R3	20,9828949	-81,67887115	-945,7576294	-0,059199
Cloud_Mesh_Distance_BA_R0_R3	21,01408005	-81,79410553	-945,7679443	-0,064732
Cloud_Mesh_Distance_BA_R0_R3	21,3032856	-81,69207001	-945,578186	-0,055108
Cloud_Mesh_Distance_BA_R0_R3	16,3212738	-81,20250702	-945,5523682	0,257307
Cloud_Mesh_Distance_BA_R0_R3	16,34728241	-81,07482147	-945,5614014	0,184531
Cloud_Mesh_Distance_BA_R0_R3	21,09964371	-81,5858078	-945,6732788	-0,052275
Cloud_Mesh_Distance_BA_R0_R3	16,13323975	-80,85603333	-945,4381714	0,04755
Cloud_Mesh_Distance_BA_R0_R3	16,20867348	-80,77800751	-945,5247192	0,033028
Cloud_Mesh_Distance_BA_R0_R3	20,88571548	-81,37699127	-945,7408447	-0,048181
Cloud_Mesh_Distance_BA_R0_R3	20,94975281	-81,52727509	-945,7420044	-0,050499
Cloud_Mesh_Distance_BA_R0_R3	20,95948029	-81,246315	-945,6716309	-0,047732
Cloud_Mesh_Distance_BA_R0_R3	21,05570984	-81,37438202	-945,6491089	-0,047162
Cloud_Mesh_Distance_BA_R0_R3	21,06710815	-81,20639801	-945,600708	-0,052929
Cloud_Mesh_Distance_BA_R0_R3	16,11363411	-80,56504059	-945,5469971	-0,038991
Cloud_Mesh_Distance_BA_R0_R3	16,02652359	-80,6517868	-945,428833	-0,038519
Cloud_Mesh_Distance_BA_R0_R3	16,19205093	-80,55182648	-945,6137695	-0,027004
Cloud_Mesh_Distance_BA_R0_R3	20,8434391	-81,0025177	-945,6709595	-0,057481
Cloud_Mesh_Distance_BA_R0_R3	20,73483658	-81,05323029	-945,734314	-0,055005
Cloud_Mesh_Distance_BA_R0_R3	20,98108101	-81,07971954	-945,6182251	-0,054689
Cloud_Mesh_Distance_BA_R0_R3	21,12140656	-81,09876251	-945,5401611	-0,057548
Cloud_Mesh_Distance_BA_R0_R3	15,8657856	-80,4223175	-945,4188843	-0,093425
Cloud_Mesh_Distance_BA_R0_R3	15,8803587	-80,24599457	-945,545166	-0,097247
Cloud_Mesh_Distance_BA_R0_R3	15,96023464	-80,37363434	-945,5324707	-0,084498
Cloud_Mesh_Distance_BA_R0_R3	15,99212551	-80,50470734	-945,4800415	-0,068529
Cloud_Mesh_Distance_BA_R0_R3	20,75385094	-80,94486237	-945,6993408	-0,060035
Cloud_Mesh_Distance_BA_R0_R3	15,68071079	-80,01625824	-945,5067749	-0,102746
Cloud_Mesh_Distance_BA_R0_R3	20,6195488	-80,59874725	-945,68396	-0,075158
Cloud_Mesh_Distance_BA_R0_R3	15,59043121	-79,89432526	-945,4849243	-0,100155
Cloud_Mesh_Distance_BA_R0_R3	15,55371094	-79,95265961	-945,4127808	-0,099795
Cloud_Mesh_Distance_BA_R0_R3	15,59796715	-80,00649261	-945,4299927	-0,103678
Cloud_Mesh_Distance_BA_R0_R3	20,38784981	-80,51899719	-945,7636108	-0,077771
Cloud_Mesh_Distance_BA_R0_R3	20,58694458	-80,47048187	-945,6773071	-0,075414
Cloud_Mesh_Distance_BA_R0_R3	20,44242477	-80,40253448	-945,727478	-0,077198
Cloud_Mesh_Distance_BA_R0_R3	15,45405483	-79,6858902	-945,4304199	-0,083891
Cloud_Mesh_Distance_BA_R0_R3	15,49536037	-79,85024261	-945,4035034	-0,091541
Cloud_Mesh_Distance_BA_R0_R3	15,55453205	-79,69835663	-945,5372315	-0,086905
Cloud_Mesh_Distance_BA_R0_R3	20,30915451	-80,27927399	-945,7662964	-0,072077
Cloud_Mesh_Distance_BA_R0_R3	20,58657265	-80,1868515	-945,638916	-0,072532
Cloud_Mesh_Distance_BA_R0_R3	20,44714737	-80,1805191	-945,6994629	-0,071455
Cloud_Mesh_Distance_BA_R0_R3	20,56782913	-80,31659698	-945,6639404	-0,074453
Cloud_Mesh_Distance_BA_R0_R3	20,66516876	-80,26687622	-945,6133423	-0,07408
Cloud_Mesh_Distance_BA_R0_R3	15,38918114	-79,60201263	-945,3782349	-0,072248
Cloud_Mesh_Distance_BA_R0_R3	15,55653191	-79,47999573	-945,6080933	-0,078617
Cloud_Mesh_Distance_BA_R0_R3	15,64444923	-79,68773651	-945,6290283	-0,092258
Cloud_Mesh_Distance_BA_R0_R3	20,13641548	-80,01331329	-945,8108521	-0,061574
Cloud_Mesh_Distance_BA_R0_R3	20,38241577	-79,94304657	-945,7011719	-0,067313
Cloud_Mesh_Distance_BA_R0_R3	20,28211021	-80,08919525	-945,7593994	-0,068005
Cloud_Mesh_Distance_BA_R0_R3	20,54590988	-80,0357132	-945,6390381	-0,069024
Cloud_Mesh_Distance_BA_R0_R3	15,44571495	-79,35079193	-945,5142822	-0,063845
Cloud_Mesh_Distance_BA_R0_R3	15,36492157	-79,44875336	-945,3897705	-0,063231

Cloud_Mesh_Distance_BA_R0_R3	15,35476971	-79,23721313	-945,4100952	-0,056625
Cloud_Mesh_Distance_BA_R0_R3	15,54169559	-79,22248077	-945,6394653	-0,060003
Cloud_Mesh_Distance_BA_R0_R3	15,45586109	-79,54281616	-945,480896	-0,075931
Cloud_Mesh_Distance_BA_R0_R3	20,23350525	-79,91869354	-945,7634277	-0,063494
Cloud_Mesh_Distance_BA_R0_R3	20,40659904	-79,77542114	-945,6689453	-0,064611
Cloud_Mesh_Distance_BA_R0_R3	15,43847847	-79,1840744	-945,5267334	-0,054485
Cloud_Mesh_Distance_BA_R0_R3	15,57697392	-79,02645111	-945,6876831	-0,063823
Cloud_Mesh_Distance_BA_R0_R3	20,08459473	-79,66870117	-945,8005981	-0,060074
Cloud_Mesh_Distance_BA_R0_R3	20,10527039	-79,50823212	-945,7720947	-0,065983
Cloud_Mesh_Distance_BA_R0_R3	20,25172043	-79,51036835	-945,7037964	-0,066024
Cloud_Mesh_Distance_BA_R0_R3	20,2448101	-79,72057343	-945,7352905	-0,067045
Cloud_Mesh_Distance_BA_R0_R3	15,4058857	-78,97546387	-945,489502	-0,044971
Cloud_Mesh_Distance_BA_R0_R3	15,3932724	-78,85144806	-945,461853	-0,038535
Cloud_Mesh_Distance_BA_R0_R3	15,47648335	-79,03181458	-945,5790405	-0,052932
Cloud_Mesh_Distance_BA_R0_R3	19,88490677	-79,34148407	-945,8526001	-0,062676
Cloud_Mesh_Distance_BA_R0_R3	20,34280014	-79,47853851	-945,6542969	-0,06569
Cloud_Mesh_Distance_BA_R0_R3	15,55369854	-78,85076141	-945,6612549	-0,055188
Cloud_Mesh_Distance_BA_R0_R3	15,45785141	-78,8884964	-945,5532227	-0,046504
Cloud_Mesh_Distance_BA_R0_R3	20,01704407	-79,1332016	-945,7604981	-0,070241
Cloud_Mesh_Distance_BA_R0_R3	19,88015747	-79,15048981	-945,8310547	-0,065803
Cloud_Mesh_Distance_BA_R0_R3	20,02090645	-79,30092621	-945,7832031	-0,066546
Cloud_Mesh_Distance_BA_R0_R3	20,16969681	-79,35799408	-945,7194824	-0,068547
Cloud_Mesh_Distance_BA_R0_R3	20,12403107	-79,19962311	-945,716919	-0,067574
Cloud_Mesh_Distance_BA_R0_R3	15,44428539	-78,43367767	-945,4320679	-0,030589
Cloud_Mesh_Distance_BA_R0_R3	15,47812843	-78,74013519	-945,5630493	-0,040941
Cloud_Mesh_Distance_BA_R0_R3	19,96957779	-78,99289703	-945,762146	-0,069962
Cloud_Mesh_Distance_BA_R0_R3	15,51812363	-78,2678299	-945,47052	-0,0319
Cloud_Mesh_Distance_BA_R0_R3	15,57735252	-78,35583496	-945,588501	-0,039687
Cloud_Mesh_Distance_BA_R0_R3	15,51182842	-78,54963684	-945,5679321	-0,036504
Cloud_Mesh_Distance_BA_R0_R3	19,82257843	-78,93688202	-945,8278198	-0,071763
Cloud_Mesh_Distance_BA_R0_R3	19,93721962	-78,82897186	-945,7510376	-0,06976
Cloud_Mesh_Distance_BA_R0_R3	19,79568863	-78,72412872	-945,8060303	-0,072848
Cloud_Mesh_Distance_BA_R0_R3	15,70782757	-78,23155975	-945,6849365	-0,0579
Cloud_Mesh_Distance_BA_R0_R3	15,81417179	-78,0714035	-945,7174683	-0,069528
Cloud_Mesh_Distance_BA_R0_R3	19,63234329	-78,62545013	-945,8701782	-0,073784
Cloud_Mesh_Distance_BA_R0_R3	19,89788628	-78,56812286	-945,7201538	-0,073348
Cloud_Mesh_Distance_BA_R0_R3	19,93763351	-78,69811249	-945,7258301	-0,070313
Cloud_Mesh_Distance_BA_R0_R3	15,64984798	-78,04256439	-945,5252686	-0,043593
Cloud_Mesh_Distance_BA_R0_R3	15,77686596	-77,95957184	-945,6208496	-0,062546
Cloud_Mesh_Distance_BA_R0_R3	15,70769882	-78,1001358	-945,626709	-0,052254
Cloud_Mesh_Distance_BA_R0_R3	19,47570038	-78,36987305	-945,8945313	-0,07711
Cloud_Mesh_Distance_BA_R0_R3	19,5951252	-78,45511627	-945,8543701	-0,075144
Cloud_Mesh_Distance_BA_R0_R3	19,77986526	-78,30531311	-945,7192383	-0,084407
Cloud_Mesh_Distance_BA_R0_R3	19,88377571	-78,40595245	-945,6871948	-0,080892
Cloud_Mesh_Distance_BA_R0_R3	19,74540901	-78,47612762	-945,7822876	-0,079531
Cloud_Mesh_Distance_BA_R0_R3	20,02389145	-78,62935638	-945,6609497	-0,075382
Cloud_Mesh_Distance_BA_R0_R3	15,80083942	-77,83209991	-945,5617676	-0,066777
Cloud_Mesh_Distance_BA_R0_R3	15,96804333	-77,89706421	-945,7572632	-0,096647
Cloud_Mesh_Distance_BA_R0_R3	19,35161972	-78,14203644	-945,8980713	-0,080355
Cloud_Mesh_Distance_BA_R0_R3	19,44490433	-78,22254944	-945,8747559	-0,08036

Cloud_Mesh_Distance_BA_R0_R3	19,61018753	-78,25601959	-945,7993164	-0,085894
Cloud_Mesh_Distance_BA_R0_R3	15,7635231	-77,74944305	-945,4508057	-0,056645
Cloud_Mesh_Distance_BA_R0_R3	15,92778206	-77,81821442	-945,6762085	-0,090036
Cloud_Mesh_Distance_BA_R0_R3	15,86824799	-77,72704315	-945,5543213	-0,073015
Cloud_Mesh_Distance_BA_R0_R3	16,11651039	-77,4969101	-945,6018677	-0,102869
Cloud_Mesh_Distance_BA_R0_R3	16,14135361	-77,71927643	-945,7825928	-0,118407
Cloud_Mesh_Distance_BA_R0_R3	19,32906723	-77,93880463	-945,8449097	-0,086342
Cloud_Mesh_Distance_BA_R0_R3	19,44503021	-78,07508087	-945,8323364	-0,08762
Cloud_Mesh_Distance_BA_R0_R3	19,57073212	-77,89446259	-945,6968994	-0,098135
Cloud_Mesh_Distance_BA_R0_R3	19,59030342	-78,03138733	-945,7390747	-0,090311
Cloud_Mesh_Distance_BA_R0_R3	16,22403336	-77,4413681	-945,6438599	-0,119206
Cloud_Mesh_Distance_BA_R0_R3	16,18988419	-77,54798126	-945,699707	-0,122854
Cloud_Mesh_Distance_BA_R0_R3	16,30234337	-77,57122803	-945,7953491	-0,132376
Cloud_Mesh_Distance_BA_R0_R3	19,50580978	-77,80223846	-945,6930542	-0,102797
Cloud_Mesh_Distance_BA_R0_R3	19,31199646	-77,80384064	-945,798645	-0,091833
Cloud_Mesh_Distance_BA_R0_R3	19,45366478	-77,9008255	-945,7651367	-0,09252
Cloud_Mesh_Distance_BA_R0_R3	16,29879379	-77,35056305	-945,6248169	-0,123163
Cloud_Mesh_Distance_BA_R0_R3	16,56046104	-77,32648468	-945,7756348	-0,134275
Cloud_Mesh_Distance_BA_R0_R3	16,4393158	-77,27437592	-945,6591797	-0,137962
Cloud_Mesh_Distance_BA_R0_R3	16,36704636	-77,43030548	-945,7363892	-0,134578
Cloud_Mesh_Distance_BA_R0_R3	16,60266113	-77,19165802	-945,6900635	-0,136157
Cloud_Mesh_Distance_BA_R0_R3	18,53760719	-77,28220367	-945,843689	-0,059922
Cloud_Mesh_Distance_BA_R0_R3	18,6185894	-77,4416275	-945,9116821	-0,057448
Cloud_Mesh_Distance_BA_R0_R3	19,00721359	-77,63355255	-945,8592529	-0,078404
Cloud_Mesh_Distance_BA_R0_R3	18,95269012	-77,48961639	-945,8029175	-0,081914
Cloud_Mesh_Distance_BA_R0_R3	19,40870094	-77,76570892	-945,7315064	-0,100393
Cloud_Mesh_Distance_BA_R0_R3	16,62386703	-77,03156281	-945,5532837	-0,127763
Cloud_Mesh_Distance_BA_R0_R3	16,64520645	-77,09583282	-945,6288452	-0,13398
Cloud_Mesh_Distance_BA_R0_R3	16,76373672	-77,10971832	-945,7058106	-0,133884
Cloud_Mesh_Distance_BA_R0_R3	16,99224472	-77,16110992	-945,8477783	-0,121615
Cloud_Mesh_Distance_BA_R0_R3	18,67544937	-77,28457642	-945,7903442	-0,068051
Cloud_Mesh_Distance_BA_R0_R3	18,63664055	-77,21320343	-945,7590332	-0,063283
Cloud_Mesh_Distance_BA_R0_R3	16,94498253	-77,07685089	-945,7587891	-0,125238
Cloud_Mesh_Distance_BA_R0_R3	17,08670998	-77,07954407	-945,8135986	-0,112413
Cloud_Mesh_Distance_BA_R0_R3	17,10017776	-76,96193695	-945,711853	-0,119667
Cloud_Mesh_Distance_BA_R0_R3	17,26037407	-77,04320526	-945,831543	-0,101506
Cloud_Mesh_Distance_BA_R0_R3	17,29266739	-76,92456055	-945,7341309	-0,101997
Cloud_Mesh_Distance_BA_R0_R3	17,46020126	-76,95642853	-945,7949219	-0,082147
Cloud_Mesh_Distance_BA_R0_R3	17,97454834	-76,97844696	-945,8057861	-0,035604
Cloud_Mesh_Distance_BA_R0_R3	17,85302162	-77,07880402	-945,8988037	-0,043559
Cloud_Mesh_Distance_BA_R0_R3	18,20112419	-77,15378571	-945,8790283	-0,039246
Cloud_Mesh_Distance_BA_R0_R3	18,35432053	-77,16634369	-945,8364258	-0,044124
Cloud_Mesh_Distance_BA_R0_R3	18,52518845	-77,06406403	-945,703125	-0,050694
Cloud_Mesh_Distance_BA_R0_R3	18,51171875	-77,14954376	-945,7661743	-0,053499
Cloud_Mesh_Distance_BA_R0_R3	18,39583969	-77,04229736	-945,7363892	-0,041355
Cloud_Mesh_Distance_BA_R0_R3	17,62730789	-76,92908478	-945,7873535	-0,062088
Cloud_Mesh_Distance_BA_R0_R3	17,78004074	-76,95104218	-945,805481	-0,050645
Cloud_Mesh_Distance_BA_R0_R3	17,8867321	-76,85248566	-945,7171021	-0,037566
Cloud_Mesh_Distance_BA_R0_R3	18,19409561	-77,02701569	-945,7914429	-0,033575
Cloud_Mesh_Distance_BA_R0_R3	21,68740273	-82,12277985	-945,4918823	-0,140243

Cloud_Mesh_Distance_BA_R0_R3	21,76377487	-82,07558441	-945,4139404	-0,057171
Cloud_Mesh_Distance_BA_R0_R3	21,91001511	-82,14061737	-945,336853	-0,075172
Cloud_Mesh_Distance_BA_R0_R3	22,04126549	-82,22055817	-945,2730713	-0,155696
Cloud_Mesh_Distance_BA_R0_R3	16,02582932	-81,26700592	-945,3222046	0,254242
Cloud_Mesh_Distance_BA_R0_R3	21,3211937	-81,84577179	-945,6135864	-0,059883
Cloud_Mesh_Distance_BA_R0_R3	21,43564415	-81,7282486	-945,5011597	-0,058771
Cloud_Mesh_Distance_BA_R0_R3	21,57668686	-81,85578156	-945,4492798	-0,058716
Cloud_Mesh_Distance_BA_R0_R3	21,45764542	-81,93871307	-945,5612183	-0,058873
Cloud_Mesh_Distance_BA_R0_R3	21,68623352	-82,05059052	-945,4570313	-0,075784
Cloud_Mesh_Distance_BA_R0_R3	16,03277969	-81,11917114	-945,3059692	0,151461
Cloud_Mesh_Distance_BA_R0_R3	15,91663647	-81,22807312	-945,1784058	0,192541
Cloud_Mesh_Distance_BA_R0_R3	21,2286396	-81,60199738	-945,6001587	-0,054957
Cloud_Mesh_Distance_BA_R0_R3	21,47713852	-81,55487823	-945,4173584	-0,061887
Cloud_Mesh_Distance_BA_R0_R3	21,58120728	-81,66706085	-945,376648	-0,061414
Cloud_Mesh_Distance_BA_R0_R3	16,02714729	-80,92578888	-945,3225098	0,048766
Cloud_Mesh_Distance_BA_R0_R3	16,15094757	-81,02666473	-945,4198608	0,127544
Cloud_Mesh_Distance_BA_R0_R3	21,3419075	-81,5402298	-945,5093384	-0,056876
Cloud_Mesh_Distance_BA_R0_R3	21,31028366	-81,37458038	-945,4862671	-0,058248
Cloud_Mesh_Distance_BA_R0_R3	15,91431904	-80,88967133	-945,213562	0,010456
Cloud_Mesh_Distance_BA_R0_R3	16,05203247	-80,78084564	-945,3933716	0,00113
Cloud_Mesh_Distance_BA_R0_R3	21,21248627	-81,23901367	-945,5170288	-0,058561
Cloud_Mesh_Distance_BA_R0_R3	21,3538208	-81,26470184	-945,4266968	-0,065202
Cloud_Mesh_Distance_BA_R0_R3	21,20795059	-81,45121002	-945,574707	-0,052222
Cloud_Mesh_Distance_BA_R0_R3	21,43869019	-81,38538361	-945,3973999	-0,065704
Cloud_Mesh_Distance_BA_R0_R3	15,73491573	-80,39724731	-945,3071899	-0,104092
Cloud_Mesh_Distance_BA_R0_R3	15,93151474	-80,59061432	-945,3752441	-0,067504
Cloud_Mesh_Distance_BA_R0_R3	15,95112228	-80,74873352	-945,3103638	-0,028136
Cloud_Mesh_Distance_BA_R0_R3	16,12659454	-80,71320343	-945,4854736	-0,003381
Cloud_Mesh_Distance_BA_R0_R3	20,95362091	-80,86703491	-945,5803223	-0,065736
Cloud_Mesh_Distance_BA_R0_R3	15,57016182	-80,2436676	-945,2368164	-0,110984
Cloud_Mesh_Distance_BA_R0_R3	15,55745506	-80,3390274	-945,1463623	-0,112661
Cloud_Mesh_Distance_BA_R0_R3	15,64046574	-80,23436737	-945,3242798	-0,11278
Cloud_Mesh_Distance_BA_R0_R3	20,82303238	-80,86788177	-945,6486816	-0,063452
Cloud_Mesh_Distance_BA_R0_R3	20,70778656	-80,82262421	-945,6912231	-0,067383
Cloud_Mesh_Distance_BA_R0_R3	21,08803749	-80,74924469	-945,4772339	-0,073171
Cloud_Mesh_Distance_BA_R0_R3	21,25563049	-80,82852936	-945,388794	-0,077109
Cloud_Mesh_Distance_BA_R0_R3	21,10727119	-80,94212341	-945,5099487	-0,065807
Cloud_Mesh_Distance_BA_R0_R3	15,49276257	-80,20689392	-945,168396	-0,111666
Cloud_Mesh_Distance_BA_R0_R3	15,63753319	-80,10779572	-945,407959	-0,11099
Cloud_Mesh_Distance_BA_R0_R3	15,74494934	-80,21617889	-945,4436035	-0,106103
Cloud_Mesh_Distance_BA_R0_R3	20,69999504	-80,67507172	-945,6636963	-0,073139
Cloud_Mesh_Distance_BA_R0_R3	20,84581757	-80,69841766	-945,5994263	-0,070953
Cloud_Mesh_Distance_BA_R0_R3	20,99795532	-80,59191132	-945,4970703	-0,075479
Cloud_Mesh_Distance_BA_R0_R3	20,97290802	-80,72740173	-945,5377197	-0,073241
Cloud_Mesh_Distance_BA_R0_R3	15,36239243	-79,9569931	-945,1678467	-0,092012
Cloud_Mesh_Distance_BA_R0_R3	15,32854843	-79,81752777	-945,1976318	-0,082174
Cloud_Mesh_Distance_BA_R0_R3	15,42581654	-80,05728149	-945,1901856	-0,103693
Cloud_Mesh_Distance_BA_R0_R3	15,53304672	-80,06447601	-945,3214722	-0,105355
Cloud_Mesh_Distance_BA_R0_R3	15,41867065	-79,9148407	-945,2735596	-0,094796
Cloud_Mesh_Distance_BA_R0_R3	20,72811508	-80,52216339	-945,6218262	-0,076206

Cloud_Mesh_Distance_BA_R0_R3	20,67007446	-80,38039398	-945,6264038	-0,076728
Cloud_Mesh_Distance_BA_R0_R3	20,79598618	-80,31571198	-945,5567017	-0,073954
Cloud_Mesh_Distance_BA_R0_R3	20,86910248	-80,50434113	-945,550415	-0,075276
Cloud_Mesh_Distance_BA_R0_R3	20,9720974	-80,3853302	-945,4749146	-0,075431
Cloud_Mesh_Distance_BA_R0_R3	15,39246273	-79,74375153	-945,3268433	-0,082293
Cloud_Mesh_Distance_BA_R0_R3	20,81640625	-80,15326691	-945,5222778	-0,071298
Cloud_Mesh_Distance_BA_R0_R3	20,8287735	-79,99115753	-945,4916382	-0,066366
Cloud_Mesh_Distance_BA_R0_R3	20,69909096	-80,12310028	-945,5771484	-0,071143
Cloud_Mesh_Distance_BA_R0_R3	20,93769455	-80,17235565	-945,4609985	-0,070606
Cloud_Mesh_Distance_BA_R0_R3	21,11810875	-80,25426483	-945,3675537	-0,076015
Cloud_Mesh_Distance_BA_R0_R3	21,05387497	-80,08706665	-945,3781738	-0,069078
Cloud_Mesh_Distance_BA_R0_R3	20,50246429	-79,87457275	-945,6376953	-0,066843
Cloud_Mesh_Distance_BA_R0_R3	20,64492035	-79,89350128	-945,5716553	-0,065255
Cloud_Mesh_Distance_BA_R0_R3	20,76138115	-79,75583649	-945,4862671	-0,059989
Cloud_Mesh_Distance_BA_R0_R3	20,9575882	-79,81494904	-945,3830566	-0,060908
Cloud_Mesh_Distance_BA_R0_R3	15,27569675	-79,20442963	-945,2883911	-0,044213
Cloud_Mesh_Distance_BA_R0_R3	20,37174225	-79,61958313	-945,6623535	-0,066234
Cloud_Mesh_Distance_BA_R0_R3	20,52223206	-79,71572113	-945,6050415	-0,062595
Cloud_Mesh_Distance_BA_R0_R3	20,63970566	-79,73853302	-945,5495606	-0,061282
Cloud_Mesh_Distance_BA_R0_R3	20,76102448	-79,59525299	-945,4523315	-0,057949
Cloud_Mesh_Distance_BA_R0_R3	15,395854	-79,08870697	-945,4762573	-0,053585
Cloud_Mesh_Distance_BA_R0_R3	20,27384186	-79,39081573	-945,6733398	-0,067325
Cloud_Mesh_Distance_BA_R0_R3	20,45425034	-79,51929474	-945,6048584	-0,061445
Cloud_Mesh_Distance_BA_R0_R3	20,71182251	-79,48343658	-945,4547119	-0,05596
Cloud_Mesh_Distance_BA_R0_R3	20,62478447	-79,41301727	-945,4897461	-0,058636
Cloud_Mesh_Distance_BA_R0_R3	20,49829483	-79,3629303	-945,5494385	-0,061938
Cloud_Mesh_Distance_BA_R0_R3	20,62335396	-79,57607269	-945,5265503	-0,058486
Cloud_Mesh_Distance_BA_R0_R3	15,27775192	-78,65549469	-945,2088013	-0,029486
Cloud_Mesh_Distance_BA_R0_R3	15,27337551	-78,77866364	-945,241333	-0,032284
Cloud_Mesh_Distance_BA_R0_R3	15,32143116	-78,69589996	-945,3116455	-0,030343
Cloud_Mesh_Distance_BA_R0_R3	15,39729977	-78,66407013	-945,4332275	-0,035121
Cloud_Mesh_Distance_BA_R0_R3	20,24535561	-79,26177216	-945,6647339	-0,066221
Cloud_Mesh_Distance_BA_R0_R3	20,3602562	-79,36039734	-945,6237183	-0,063916
Cloud_Mesh_Distance_BA_R0_R3	20,49373245	-79,20775604	-945,5161743	-0,06156
Cloud_Mesh_Distance_BA_R0_R3	15,34832668	-78,55957031	-945,3161621	-0,027917
Cloud_Mesh_Distance_BA_R0_R3	20,09543228	-79,04888153	-945,7062378	-0,068233
Cloud_Mesh_Distance_BA_R0_R3	20,20122528	-79,12595367	-945,663208	-0,066416
Cloud_Mesh_Distance_BA_R0_R3	20,19912338	-78,98325348	-945,6364746	-0,069705
Cloud_Mesh_Distance_BA_R0_R3	20,45430756	-79,05184174	-945,4996338	-0,061927
Cloud_Mesh_Distance_BA_R0_R3	20,3461647	-79,15937042	-945,5905151	-0,065593
Cloud_Mesh_Distance_BA_R0_R3	15,30146885	-78,49640656	-945,1862183	-0,031167
Cloud_Mesh_Distance_BA_R0_R3	15,37024307	-78,41129303	-945,2880249	-0,027783
Cloud_Mesh_Distance_BA_R0_R3	20,0667305	-78,89043427	-945,6919556	-0,070457
Cloud_Mesh_Distance_BA_R0_R3	20,02770233	-78,75749969	-945,687439	-0,071487
Cloud_Mesh_Distance_BA_R0_R3	20,17020798	-78,76194763	-945,6039429	-0,066499
Cloud_Mesh_Distance_BA_R0_R3	20,31749725	-78,93877411	-945,5559082	-0,066063
Cloud_Mesh_Distance_BA_R0_R3	20,41565323	-78,89764404	-945,4810181	-0,064395
Cloud_Mesh_Distance_BA_R0_R3	15,61298656	-78,18856049	-945,5604248	-0,036283
Cloud_Mesh_Distance_BA_R0_R3	15,54672813	-78,12741089	-945,4331055	-0,033577
Cloud_Mesh_Distance_BA_R0_R3	20,11576462	-78,52049255	-945,572937	-0,06996

Cloud_Mesh_Distance_BA_R0_R3	15,59092045	-77,97338104	-945,3915405	-0,036856
Cloud_Mesh_Distance_BA_R0_R3	15,69756031	-77,89488983	-945,4844971	-0,047157
Cloud_Mesh_Distance_BA_R0_R3	15,64331436	-77,79959869	-945,3219605	-0,045644
Cloud_Mesh_Distance_BA_R0_R3	19,8728447	-78,27085876	-945,6519165	-0,082493
Cloud_Mesh_Distance_BA_R0_R3	19,98694229	-78,48926544	-945,6477051	-0,076963
Cloud_Mesh_Distance_BA_R0_R3	20,00463867	-78,33220673	-945,5869141	-0,078493
Cloud_Mesh_Distance_BA_R0_R3	19,91513062	-78,19078827	-945,5960693	-0,08654
Cloud_Mesh_Distance_BA_R0_R3	20,12879562	-78,37815857	-945,5163574	-0,075018
Cloud_Mesh_Distance_BA_R0_R3	15,87045479	-77,59796906	-945,4470215	-0,069482
Cloud_Mesh_Distance_BA_R0_R3	16,01122475	-77,62722778	-945,6147461	-0,091436
Cloud_Mesh_Distance_BA_R0_R3	19,67247391	-77,9457016	-945,6558228	-0,099927
Cloud_Mesh_Distance_BA_R0_R3	19,62385559	-77,78748322	-945,6142578	-0,109946
Cloud_Mesh_Distance_BA_R0_R3	19,7726059	-78,11837769	-945,661438	-0,089941
Cloud_Mesh_Distance_BA_R0_R3	16,0154686	-77,48468781	-945,5	-0,079895
Cloud_Mesh_Distance_BA_R0_R3	16,05887794	-77,27333069	-945,3399048	-0,077916
Cloud_Mesh_Distance_BA_R0_R3	16,04528809	-77,36261749	-945,4168701	-0,082575
Cloud_Mesh_Distance_BA_R0_R3	16,16287994	-77,37554932	-945,5379028	-0,106693
Cloud_Mesh_Distance_BA_R0_R3	18,7907238	-77,42394257	-945,8306274	-0,074229
Cloud_Mesh_Distance_BA_R0_R3	18,68053055	-77,35131073	-945,8305054	-0,070645
Cloud_Mesh_Distance_BA_R0_R3	18,77907372	-77,31965637	-945,7701416	-0,075767
Cloud_Mesh_Distance_BA_R0_R3	19,13422585	-77,56017303	-945,7631226	-0,093757
Cloud_Mesh_Distance_BA_R0_R3	19,46465874	-77,65906525	-945,6468506	-0,104659
Cloud_Mesh_Distance_BA_R0_R3	19,29990768	-77,66347504	-945,7385864	-0,096254
Cloud_Mesh_Distance_BA_R0_R3	19,29749298	-77,48697662	-945,6433106	-0,102791
Cloud_Mesh_Distance_BA_R0_R3	19,63270569	-77,47608185	-945,4350586	-0,11388
Cloud_Mesh_Distance_BA_R0_R3	19,6088295	-77,61611176	-945,5335693	-0,110722
Cloud_Mesh_Distance_BA_R0_R3	16,157444	-77,25948334	-945,4239502	-0,096799
Cloud_Mesh_Distance_BA_R0_R3	16,38919449	-77,09815216	-945,4570923	-0,113063
Cloud_Mesh_Distance_BA_R0_R3	16,50442505	-77,12613678	-945,5686035	-0,129034
Cloud_Mesh_Distance_BA_R0_R3	16,31283569	-77,23046112	-945,5282593	-0,112657
Cloud_Mesh_Distance_BA_R0_R3	18,89624786	-77,37039948	-945,7543335	-0,081715
Cloud_Mesh_Distance_BA_R0_R3	18,88552475	-77,23175812	-945,6713257	-0,075232
Cloud_Mesh_Distance_BA_R0_R3	19,067379	-77,38498688	-945,6918945	-0,089066
Cloud_Mesh_Distance_BA_R0_R3	19,2166996	-77,30860901	-945,5772095	-0,09485
Cloud_Mesh_Distance_BA_R0_R3	19,38738251	-77,34990692	-945,5134888	-0,101846
Cloud_Mesh_Distance_BA_R0_R3	19,48932266	-77,4933548	-945,5404663	-0,109077
Cloud_Mesh_Distance_BA_R0_R3	16,84750748	-76,87187195	-945,5113525	-0,123178
Cloud_Mesh_Distance_BA_R0_R3	16,71572495	-76,9391861	-945,5115967	-0,122325
Cloud_Mesh_Distance_BA_R0_R3	16,75995255	-76,8443985	-945,4313965	-0,119114
Cloud_Mesh_Distance_BA_R0_R3	16,87347031	-76,97059631	-945,6275024	-0,126511
Cloud_Mesh_Distance_BA_R0_R3	16,7256279	-77,02741241	-945,6089478	-0,131909
Cloud_Mesh_Distance_BA_R0_R3	17,22315788	-76,83618927	-945,6279907	-0,105162
Cloud_Mesh_Distance_BA_R0_R3	18,62558746	-77,12267303	-945,7037964	-0,059716
Cloud_Mesh_Distance_BA_R0_R3	18,74154663	-77,24800873	-945,7397461	-0,068244
Cloud_Mesh_Distance_BA_R0_R3	18,72784042	-77,18152618	-945,7015381	-0,06496
Cloud_Mesh_Distance_BA_R0_R3	18,89832687	-77,09677887	-945,5773926	-0,066784
Cloud_Mesh_Distance_BA_R0_R3	17,10616493	-76,66472626	-945,3869019	-0,104225
Cloud_Mesh_Distance_BA_R0_R3	17,01268768	-76,83592987	-945,5501099	-0,116646
Cloud_Mesh_Distance_BA_R0_R3	17,40958405	-76,84220123	-945,6813355	-0,088758
Cloud_Mesh_Distance_BA_R0_R3	17,55494118	-76,86237335	-945,7233887	-0,066398

Cloud_Mesh_Distance_BA_R0_R3	17,56242371	-76,75247192	-945,6171875	-0,064469
Cloud_Mesh_Distance_BA_R0_R3	17,34969711	-76,73978424	-945,5608521	-0,087212
Cloud_Mesh_Distance_BA_R0_R3	17,70350647	-76,8425827	-945,7150879	-0,049951
Cloud_Mesh_Distance_BA_R0_R3	18,08076286	-76,87020111	-945,7019043	-0,028841
Cloud_Mesh_Distance_BA_R0_R3	18,35465431	-76,72652435	-945,5027466	-0,005006
Cloud_Mesh_Distance_BA_R0_R3	18,30521774	-76,88307953	-945,6502686	-0,023137
Cloud_Mesh_Distance_BA_R0_R3	18,49209404	-76,96559906	-945,6459961	-0,037708
Cloud_Mesh_Distance_BA_R0_R3	18,47913361	-76,83916473	-945,5543823	-0,018669
Cloud_Mesh_Distance_BA_R0_R3	18,74839592	-77,08376312	-945,6290894	-0,061143
Cloud_Mesh_Distance_BA_R0_R3	18,60716057	-77,03449249	-945,6503906	-0,049822
Cloud_Mesh_Distance_BA_R0_R3	18,6505928	-76,93222809	-945,5594482	-0,033937
Cloud_Mesh_Distance_BA_R0_R3	17,16266632	-76,7358017	-945,496521	-0,105468
Cloud_Mesh_Distance_BA_R0_R3	17,45829964	-76,6582489	-945,4959106	-0,068834
Cloud_Mesh_Distance_BA_R0_R3	17,64485931	-76,62363434	-945,4888916	-0,041956
Cloud_Mesh_Distance_BA_R0_R3	17,79821777	-76,72681427	-945,607544	-0,037392
Cloud_Mesh_Distance_BA_R0_R3	17,97468185	-76,65692902	-945,527771	-0,012088
Cloud_Mesh_Distance_BA_R0_R3	17,97812653	-76,76576996	-945,6311035	-0,024729
Cloud_Mesh_Distance_BA_R0_R3	18,11421394	-76,63535309	-945,4835205	-0,002618
Cloud_Mesh_Distance_BA_R0_R3	18,14709663	-76,73666382	-945,5735474	-0,008835
Cloud_Mesh_Distance_BA_R0_R3	18,04610062	-76,55960846	-945,4100342	0,000201
Cloud_Mesh_Distance_BA_R0_R3	15,72384453	-81,46840668	-944,8928223	0,296383
Cloud_Mesh_Distance_BA_R0_R3	21,82933807	-81,93584442	-945,2958984	-0,05531
Cloud_Mesh_Distance_BA_R0_R3	21,95664978	-81,9905777	-945,2200317	-0,048373
Cloud_Mesh_Distance_BA_R0_R3	15,84494209	-81,41996002	-945,1599121	0,332637
Cloud_Mesh_Distance_BA_R0_R3	21,69257736	-81,96016693	-945,4100952	-0,054223
Cloud_Mesh_Distance_BA_R0_R3	21,700634	-81,79669952	-945,3354492	-0,058355
Cloud_Mesh_Distance_BA_R0_R3	15,82234287	-81,17128754	-945,0236206	0,112086
Cloud_Mesh_Distance_BA_R0_R3	15,80533218	-81,33634186	-945,026062	0,226816
Cloud_Mesh_Distance_BA_R0_R3	21,82830429	-81,60720062	-945,161499	-0,067273
Cloud_Mesh_Distance_BA_R0_R3	21,71114159	-81,60797882	-945,257019	-0,066143
Cloud_Mesh_Distance_BA_R0_R3	21,82036209	-81,74552155	-945,2202148	-0,060205
Cloud_Mesh_Distance_BA_R0_R3	15,79828167	-80,9250412	-945,0512695	-0,007888
Cloud_Mesh_Distance_BA_R0_R3	15,95096111	-81,03913116	-945,2193604	0,088536
Cloud_Mesh_Distance_BA_R0_R3	15,85936832	-81,01554108	-945,1051025	0,044962
Cloud_Mesh_Distance_BA_R0_R3	15,74561024	-80,8397522	-945,0183106	-0,046158
Cloud_Mesh_Distance_BA_R0_R3	15,71968365	-80,67237091	-945,0925293	-0,087867
Cloud_Mesh_Distance_BA_R0_R3	15,82226944	-80,82061005	-945,1303101	-0,041244
Cloud_Mesh_Distance_BA_R0_R3	15,87800217	-80,77165985	-945,2223511	-0,037392
Cloud_Mesh_Distance_BA_R0_R3	21,46368217	-81,20944214	-945,3314209	-0,072556
Cloud_Mesh_Distance_BA_R0_R3	21,53657913	-81,30693817	-945,302124	-0,074086
Cloud_Mesh_Distance_BA_R0_R3	21,5962944	-81,45749664	-945,2981567	-0,069737
Cloud_Mesh_Distance_BA_R0_R3	21,76924515	-81,49260712	-945,1707764	-0,073679
Cloud_Mesh_Distance_BA_R0_R3	21,69397736	-81,48719025	-945,2300415	-0,071916
Cloud_Mesh_Distance_BA_R0_R3	21,7060318	-81,39385223	-945,1923218	-0,075373
Cloud_Mesh_Distance_BA_R0_R3	15,56622314	-80,53928375	-944,9882813	-0,110101
Cloud_Mesh_Distance_BA_R0_R3	15,82597637	-80,61860657	-945,2525635	-0,079482
Cloud_Mesh_Distance_BA_R0_R3	15,7048769	-80,5125351	-945,1906128	-0,103178
Cloud_Mesh_Distance_BA_R0_R3	21,23344421	-81,03139496	-945,4505615	-0,068411
Cloud_Mesh_Distance_BA_R0_R3	21,3465004	-81,12320709	-945,395874	-0,068235
Cloud_Mesh_Distance_BA_R0_R3	21,56495285	-81,12883759	-945,234375	-0,082212

Cloud_Mesh_Distance_BA_R0_R3	21,5504055	-81,22583008	-945,2698975	-0,077855
Cloud_Mesh_Distance_BA_R0_R3	15,6134243	-80,49576569	-945,0900879	-0,108275
Cloud_Mesh_Distance_BA_R0_R3	15,40342617	-80,15969086	-945,0802612	-0,106529
Cloud_Mesh_Distance_BA_R0_R3	15,63545036	-80,37143707	-945,2160645	-0,11337
Cloud_Mesh_Distance_BA_R0_R3	21,36362076	-80,85830688	-945,3208618	-0,081182
Cloud_Mesh_Distance_BA_R0_R3	21,46165085	-81,04142761	-945,2919312	-0,080385
Cloud_Mesh_Distance_BA_R0_R3	21,45096207	-80,88370514	-945,263794	-0,0862
Cloud_Mesh_Distance_BA_R0_R3	21,35274506	-80,96084595	-945,3521118	-0,077021
Cloud_Mesh_Distance_BA_R0_R3	15,28885078	-79,95638275	-945,0477295	-0,089248
Cloud_Mesh_Distance_BA_R0_R3	15,36324215	-80,04225159	-945,1088867	-0,097048
Cloud_Mesh_Distance_BA_R0_R3	21,19677162	-80,67684937	-945,3947754	-0,079949
Cloud_Mesh_Distance_BA_R0_R3	21,21144485	-80,54673004	-945,3598633	-0,081665
Cloud_Mesh_Distance_BA_R0_R3	21,39150047	-80,75858307	-945,2796631	-0,085583
Cloud_Mesh_Distance_BA_R0_R3	21,10704041	-80,46422577	-945,4100952	-0,0785
Cloud_Mesh_Distance_BA_R0_R3	21,12126923	-80,60680389	-945,4279785	-0,078105
Cloud_Mesh_Distance_BA_R0_R3	21,32038879	-80,48368835	-945,2758789	-0,085141
Cloud_Mesh_Distance_BA_R0_R3	15,2155838	-79,60144806	-945,1036987	-0,062744
Cloud_Mesh_Distance_BA_R0_R3	15,29673481	-79,59733582	-945,2451172	-0,070135
Cloud_Mesh_Distance_BA_R0_R3	15,24345493	-79,73799896	-945,0960083	-0,0763
Cloud_Mesh_Distance_BA_R0_R3	21,21628571	-80,39370728	-945,3296509	-0,080592
Cloud_Mesh_Distance_BA_R0_R3	15,21528149	-79,46141815	-945,1449585	-0,055567
Cloud_Mesh_Distance_BA_R0_R3	15,281394	-79,37120819	-945,2790527	-0,056603
Cloud_Mesh_Distance_BA_R0_R3	20,9707756	-80,00418091	-945,4138794	-0,06515
Cloud_Mesh_Distance_BA_R0_R3	21,22562027	-80,2882843	-945,3044434	-0,078519
Cloud_Mesh_Distance_BA_R0_R3	15,21613312	-79,28662109	-945,1747437	-0,048363
Cloud_Mesh_Distance_BA_R0_R3	21,07242203	-79,94202423	-945,3379517	-0,064129
Cloud_Mesh_Distance_BA_R0_R3	21,08064651	-79,83214569	-945,3069458	-0,05955
Cloud_Mesh_Distance_BA_R0_R3	15,32190037	-79,02509308	-945,3660278	-0,039105
Cloud_Mesh_Distance_BA_R0_R3	15,22729874	-79,10944366	-945,2008667	-0,037332
Cloud_Mesh_Distance_BA_R0_R3	20,86120987	-79,65487671	-945,4059448	-0,056274
Cloud_Mesh_Distance_BA_R0_R3	20,84069824	-79,48241425	-945,3723755	-0,053966
Cloud_Mesh_Distance_BA_R0_R3	21,09243393	-79,71292114	-945,2653198	-0,058214
Cloud_Mesh_Distance_BA_R0_R3	15,17277718	-79,01296234	-945,0703125	-0,044767
Cloud_Mesh_Distance_BA_R0_R3	15,24912548	-78,92359161	-945,2245483	-0,03573
Cloud_Mesh_Distance_BA_R0_R3	15,3223362	-78,82222748	-945,3430786	-0,039126
Cloud_Mesh_Distance_BA_R0_R3	15,2084465	-78,79880524	-945,1004028	-0,037822
Cloud_Mesh_Distance_BA_R0_R3	15,23058987	-78,60533142	-945,0692139	-0,039493
Cloud_Mesh_Distance_BA_R0_R3	20,58756638	-79,16269684	-945,444397	-0,061576
Cloud_Mesh_Distance_BA_R0_R3	20,5819912	-79,2975235	-945,4862671	-0,059295
Cloud_Mesh_Distance_BA_R0_R3	20,72546387	-79,12999725	-945,3365479	-0,061552
Cloud_Mesh_Distance_BA_R0_R3	20,70411301	-79,32733154	-945,4165039	-0,054248
Cloud_Mesh_Distance_BA_R0_R3	20,85042572	-79,29066467	-945,302063	-0,060493
Cloud_Mesh_Distance_BA_R0_R3	20,87438583	-79,13941193	-945,223938	-0,061911
Cloud_Mesh_Distance_BA_R0_R3	20,79120827	-79,03416443	-945,2476196	-0,058707
Cloud_Mesh_Distance_BA_R0_R3	15,34011173	-78,33482361	-945,1729736	-0,031019
Cloud_Mesh_Distance_BA_R0_R3	15,3726902	-78,19506073	-945,1384277	-0,033304
Cloud_Mesh_Distance_BA_R0_R3	20,66381073	-78,8378067	-945,2687378	-0,060922
Cloud_Mesh_Distance_BA_R0_R3	20,70394135	-78,97612762	-945,295105	-0,06055
Cloud_Mesh_Distance_BA_R0_R3	20,56443405	-78,94553375	-945,390686	-0,060985
Cloud_Mesh_Distance_BA_R0_R3	15,43695545	-78,02081299	-945,1270752	-0,031961

Cloud_Mesh_Distance_BA_R0_R3	15,43488312	-78,23013306	-945,3039551	-0,024651
Cloud_Mesh_Distance_BA_R0_R3	20,28110886	-78,61396027	-945,4868164	-0,067686
Cloud_Mesh_Distance_BA_R0_R3	20,32134056	-78,79286957	-945,5144043	-0,066584
Cloud_Mesh_Distance_BA_R0_R3	20,42280388	-78,76166534	-945,4320679	-0,063419
Cloud_Mesh_Distance_BA_R0_R3	20,65090942	-78,66594696	-945,2001343	-0,063401
Cloud_Mesh_Distance_BA_R0_R3	20,5405674	-78,7286911	-945,3260498	-0,062708
Cloud_Mesh_Distance_BA_R0_R3	15,50451565	-78,05345917	-945,3057251	-0,030889
Cloud_Mesh_Distance_BA_R0_R3	20,41709328	-78,59075165	-945,3725586	-0,066669
Cloud_Mesh_Distance_BA_R0_R3	20,30754662	-78,42819214	-945,3964844	-0,069613
Cloud_Mesh_Distance_BA_R0_R3	20,20640182	-78,45667267	-945,487915	-0,072127
Cloud_Mesh_Distance_BA_R0_R3	20,52561569	-78,55905914	-945,2643433	-0,06533
Cloud_Mesh_Distance_BA_R0_R3	20,42687988	-78,45261383	-945,303833	-0,06881
Cloud_Mesh_Distance_BA_R0_R3	15,57739925	-77,78186035	-945,1827393	-0,037397
Cloud_Mesh_Distance_BA_R0_R3	15,53885174	-77,91053009	-945,2415772	-0,033037
Cloud_Mesh_Distance_BA_R0_R3	20,11631012	-78,23706818	-945,4694824	-0,084325
Cloud_Mesh_Distance_BA_R0_R3	20,00298119	-78,17338562	-945,5268555	-0,089634
Cloud_Mesh_Distance_BA_R0_R3	20,1749897	-78,12342834	-945,3662109	-0,084676
Cloud_Mesh_Distance_BA_R0_R3	20,05822372	-78,05017853	-945,427002	-0,095041
Cloud_Mesh_Distance_BA_R0_R3	20,24197769	-78,25688934	-945,3751221	-0,075304
Cloud_Mesh_Distance_BA_R0_R3	20,19946098	-78,35063934	-945,4525757	-0,07664
Cloud_Mesh_Distance_BA_R0_R3	15,82394314	-77,51642609	-945,309021	-0,055334
Cloud_Mesh_Distance_BA_R0_R3	15,74329758	-77,63209534	-945,3126831	-0,046994
Cloud_Mesh_Distance_BA_R0_R3	19,91752434	-78,05561066	-945,5380249	-0,099812
Cloud_Mesh_Distance_BA_R0_R3	20,05819702	-77,90425873	-945,3474731	-0,10401
Cloud_Mesh_Distance_BA_R0_R3	15,92403316	-77,44080353	-945,3596802	-0,068926
Cloud_Mesh_Distance_BA_R0_R3	15,91996002	-77,3529892	-945,260437	-0,069907
Cloud_Mesh_Distance_BA_R0_R3	15,98445034	-77,33377838	-945,3201904	-0,074368
Cloud_Mesh_Distance_BA_R0_R3	19,95253944	-77,90517426	-945,4360352	-0,103591
Cloud_Mesh_Distance_BA_R0_R3	19,88079262	-77,73274231	-945,3967285	-0,116177
Cloud_Mesh_Distance_BA_R0_R3	19,79575729	-77,89217377	-945,5477295	-0,110013
Cloud_Mesh_Distance_BA_R0_R3	19,7341156	-77,69740295	-945,4905396	-0,115041
Cloud_Mesh_Distance_BA_R0_R3	20,00368118	-77,79183197	-945,3287964	-0,112401
Cloud_Mesh_Distance_BA_R0_R3	20,18310928	-77,95352936	-945,263794	-0,097091
Cloud_Mesh_Distance_BA_R0_R3	15,99759007	-77,22850037	-945,2168579	-0,065978
Cloud_Mesh_Distance_BA_R0_R3	19,69655991	-77,57785797	-945,4503174	-0,117104
Cloud_Mesh_Distance_BA_R0_R3	16,13066483	-77,17650604	-945,3101807	-0,080683
Cloud_Mesh_Distance_BA_R0_R3	16,22242737	-77,0605545	-945,2695313	-0,091458
Cloud_Mesh_Distance_BA_R0_R3	16,25841141	-77,14015198	-945,3924561	-0,099805
Cloud_Mesh_Distance_BA_R0_R3	16,35245514	-77,02603912	-945,3488159	-0,105271
Cloud_Mesh_Distance_BA_R0_R3	16,52322578	-76,97974396	-945,4301758	-0,118706
Cloud_Mesh_Distance_BA_R0_R3	19,52769852	-77,37398529	-945,4432983	-0,104586
Cloud_Mesh_Distance_BA_R0_R3	19,73395157	-77,44490051	-945,3395996	-0,112988
Cloud_Mesh_Distance_BA_R0_R3	19,71366119	-77,51929474	-945,4022217	-0,116419
Cloud_Mesh_Distance_BA_R0_R3	16,65008736	-76,87244415	-945,3956909	-0,117151
Cloud_Mesh_Distance_BA_R0_R3	19,07239914	-77,25196075	-945,6067505	-0,082198
Cloud_Mesh_Distance_BA_R0_R3	19,02653503	-77,14451599	-945,5559692	-0,071203
Cloud_Mesh_Distance_BA_R0_R3	19,15647507	-77,19069672	-945,5296021	-0,078908
Cloud_Mesh_Distance_BA_R0_R3	19,30716324	-77,08076477	-945,3762817	-0,072066
Cloud_Mesh_Distance_BA_R0_R3	19,3037796	-77,19330597	-945,458374	-0,082371
Cloud_Mesh_Distance_BA_R0_R3	19,47211838	-77,25063324	-945,3989868	-0,09441

Cloud_Mesh_Distance_BA_R0_R3	16,7111702	-76,76826477	-945,3059692	-0,108855
Cloud_Mesh_Distance_BA_R0_R3	16,87437248	-76,68444061	-945,2958374	-0,107202
Cloud_Mesh_Distance_BA_R0_R3	17,00611496	-76,72332001	-945,4150391	-0,11312
Cloud_Mesh_Distance_BA_R0_R3	16,86943054	-76,78438568	-945,4204712	-0,122365
Cloud_Mesh_Distance_BA_R0_R3	18,60250282	-76,78549957	-945,4612427	-0,016689
Cloud_Mesh_Distance_BA_R0_R3	18,48315048	-76,75391388	-945,4810791	-0,008997
Cloud_Mesh_Distance_BA_R0_R3	18,84446716	-76,9845047	-945,5195923	-0,050276
Cloud_Mesh_Distance_BA_R0_R3	18,98458481	-77,03981781	-945,5010376	-0,061305
Cloud_Mesh_Distance_BA_R0_R3	18,78942871	-76,84299469	-945,4321899	-0,02772
Cloud_Mesh_Distance_BA_R0_R3	18,9980545	-76,91890717	-945,4015503	-0,04056
Cloud_Mesh_Distance_BA_R0_R3	19,15401077	-77,05925751	-945,4383545	-0,068634
Cloud_Mesh_Distance_BA_R0_R3	19,2792511	-76,99269867	-945,3218384	-0,056419
Cloud_Mesh_Distance_BA_R0_R3	17,00399017	-76,65291595	-945,3230591	-0,106523
Cloud_Mesh_Distance_BA_R0_R3	17,0829277	-76,58654022	-945,2699585	-0,098464
Cloud_Mesh_Distance_BA_R0_R3	17,26786423	-76,63472748	-945,411377	-0,087262
Cloud_Mesh_Distance_BA_R0_R3	17,53788376	-76,47537994	-945,2728882	-0,044434
Cloud_Mesh_Distance_BA_R0_R3	17,45395279	-76,55602264	-945,3673096	-0,064792
Cloud_Mesh_Distance_BA_R0_R3	17,82253647	-76,6235733	-945,5012817	-0,021564
Cloud_Mesh_Distance_BA_R0_R3	17,90890503	-76,57378387	-945,4415894	-0,010425
Cloud_Mesh_Distance_BA_R0_R3	18,22621536	-76,65010834	-945,4713745	-0,001107
Cloud_Mesh_Distance_BA_R0_R3	18,71769333	-76,69284821	-945,3248291	-0,005013
Cloud_Mesh_Distance_BA_R0_R3	18,9305191	-76,76535797	-945,2963257	-0,024572
Cloud_Mesh_Distance_BA_R0_R3	19,16516876	-76,93520355	-945,3348389	-0,046251
Cloud_Mesh_Distance_BA_R0_R3	19,27664757	-76,89122009	-945,2332153	-0,045212
Cloud_Mesh_Distance_BA_R0_R3	17,77429962	-76,54641724	-945,4069824	-0,022075
Cloud_Mesh_Distance_BA_R0_R3	17,62635803	-76,5291214	-945,3677979	-0,04081
Cloud_Mesh_Distance_BA_R0_R3	17,91945648	-76,48937225	-945,3314209	-0,005495
Cloud_Mesh_Distance_BA_R0_R3	18,04998779	-76,49362946	-945,3239136	0,004391
Cloud_Mesh_Distance_BA_R0_R3	18,17960548	-76,58364105	-945,4116821	0,005921
Cloud_Mesh_Distance_BA_R0_R3	15,6522398	-81,61306	-944,708252	0,355255
Cloud_Mesh_Distance_BA_R0_R3	15,70518398	-81,4192276	-944,6985474	0,194435
Cloud_Mesh_Distance_BA_R0_R3	22,03992844	-82,0800705	-945,1951904	-0,043099
Cloud_Mesh_Distance_BA_R0_R3	22,13855362	-82,16155243	-945,1488037	-0,042659
Cloud_Mesh_Distance_BA_R0_R3	22,21538734	-82,28151703	-945,142395	-0,089754
Cloud_Mesh_Distance_BA_R0_R3	21,95926666	-81,82595062	-945,1393433	-0,054484
Cloud_Mesh_Distance_BA_R0_R3	22,17197418	-81,83637238	-944,9454346	-0,051379
Cloud_Mesh_Distance_BA_R0_R3	22,13807487	-81,99440765	-945,0571289	-0,045515
Cloud_Mesh_Distance_BA_R0_R3	22,03660583	-81,9560318	-945,1325684	-0,052023
Cloud_Mesh_Distance_BA_R0_R3	22,25542068	-82,09073639	-944,9882813	-0,043299
Cloud_Mesh_Distance_BA_R0_R3	22,22727776	-81,94078827	-944,9395142	-0,046516
Cloud_Mesh_Distance_BA_R0_R3	15,7524786	-81,24414825	-944,8681641	0,123368
Cloud_Mesh_Distance_BA_R0_R3	15,69760895	-81,22412872	-944,6871338	0,064933
Cloud_Mesh_Distance_BA_R0_R3	21,89756775	-81,68975067	-945,1345825	-0,061872
Cloud_Mesh_Distance_BA_R0_R3	21,9747982	-81,65975189	-945,0559082	-0,064134
Cloud_Mesh_Distance_BA_R0_R3	22,07774734	-81,87311554	-945,0546875	-0,05171
Cloud_Mesh_Distance_BA_R0_R3	15,66928959	-80,84223175	-944,894043	-0,063572
Cloud_Mesh_Distance_BA_R0_R3	15,70044804	-81,05252838	-944,8079834	0,007174
Cloud_Mesh_Distance_BA_R0_R3	15,7678299	-81,01483154	-944,9627686	0,017373
Cloud_Mesh_Distance_BA_R0_R3	21,87120056	-81,5006485	-945,0886231	-0,072531
Cloud_Mesh_Distance_BA_R0_R3	21,90515518	-81,6014328	-945,0946045	-0,067158

Cloud_Mesh_Distance_BA_R0_R3	21,95418358	-81,54271698	-945,0308228	-0,070228
Cloud_Mesh_Distance_BA_R0_R3	15,43930912	-80,60445404	-944,6963501	-0,107547
Cloud_Mesh_Distance_BA_R0_R3	15,63261986	-80,65628815	-944,9869385	-0,094906
Cloud_Mesh_Distance_BA_R0_R3	15,69127178	-80,75401306	-944,99646	-0,076331
Cloud_Mesh_Distance_BA_R0_R3	21,81336975	-81,36022949	-945,0941773	-0,078653
Cloud_Mesh_Distance_BA_R0_R3	15,40145874	-80,42932892	-944,8248901	-0,10881
Cloud_Mesh_Distance_BA_R0_R3	15,53823948	-80,62928009	-944,8598023	-0,108208
Cloud_Mesh_Distance_BA_R0_R3	21,590271	-80,94651031	-945,1716919	-0,08845
Cloud_Mesh_Distance_BA_R0_R3	21,64076996	-81,26450348	-945,2092896	-0,077669
Cloud_Mesh_Distance_BA_R0_R3	21,76601028	-81,21413422	-945,0940552	-0,086303
Cloud_Mesh_Distance_BA_R0_R3	21,73041534	-81,30730438	-945,1484375	-0,078873
Cloud_Mesh_Distance_BA_R0_R3	15,30834675	-80,31376648	-944,7646484	-0,107707
Cloud_Mesh_Distance_BA_R0_R3	15,48351383	-80,37496185	-945,0145874	-0,115506
Cloud_Mesh_Distance_BA_R0_R3	21,69651413	-80,79594421	-945,0546875	-0,097817
Cloud_Mesh_Distance_BA_R0_R3	15,22083664	-80,02225494	-944,8646851	-0,092351
Cloud_Mesh_Distance_BA_R0_R3	15,15425491	-79,93579865	-944,7734985	-0,085222
Cloud_Mesh_Distance_BA_R0_R3	15,24377918	-80,18169403	-944,7637329	-0,100449
Cloud_Mesh_Distance_BA_R0_R3	15,3449297	-80,08633423	-945,0446167	-0,099452
Cloud_Mesh_Distance_BA_R0_R3	15,33005047	-80,19152069	-944,9306641	-0,102146
Cloud_Mesh_Distance_BA_R0_R3	21,30148697	-80,64893341	-945,3203735	-0,082575
Cloud_Mesh_Distance_BA_R0_R3	21,42740059	-80,5933609	-945,2213745	-0,089816
Cloud_Mesh_Distance_BA_R0_R3	21,54188538	-80,73400116	-945,1636353	-0,092725
Cloud_Mesh_Distance_BA_R0_R3	21,56869888	-80,39395905	-945,0739746	-0,093046
Cloud_Mesh_Distance_BA_R0_R3	21,55424118	-80,53318787	-945,1136475	-0,094852
Cloud_Mesh_Distance_BA_R0_R3	21,65883255	-80,45597076	-945,0133667	-0,097101
Cloud_Mesh_Distance_BA_R0_R3	21,67307472	-80,61789703	-945,036377	-0,098123
Cloud_Mesh_Distance_BA_R0_R3	15,17261314	-79,79073334	-944,9243774	-0,07316
Cloud_Mesh_Distance_BA_R0_R3	15,10007954	-79,64209747	-944,8190308	-0,067492
Cloud_Mesh_Distance_BA_R0_R3	15,1927557	-79,66815948	-945,0303345	-0,066589
Cloud_Mesh_Distance_BA_R0_R3	21,31211853	-80,31978607	-945,2521362	-0,081277
Cloud_Mesh_Distance_BA_R0_R3	21,44794655	-80,39389801	-945,1674805	-0,087728
Cloud_Mesh_Distance_BA_R0_R3	15,10738754	-79,34755707	-944,9223633	-0,048693
Cloud_Mesh_Distance_BA_R0_R3	15,15062809	-79,54712677	-944,986084	-0,060024
Cloud_Mesh_Distance_BA_R0_R3	15,16501236	-79,36991119	-945,0603027	-0,051873
Cloud_Mesh_Distance_BA_R0_R3	21,25615501	-80,18235016	-945,2645874	-0,076088
Cloud_Mesh_Distance_BA_R0_R3	21,30703735	-80,00318146	-945,1881104	-0,071096
Cloud_Mesh_Distance_BA_R0_R3	21,53213501	-80,05762482	-945,0223999	-0,084919
Cloud_Mesh_Distance_BA_R0_R3	21,4034729	-80,17371368	-945,1547241	-0,080319
Cloud_Mesh_Distance_BA_R0_R3	15,16028118	-79,20970917	-945,0603638	-0,049811
Cloud_Mesh_Distance_BA_R0_R3	15,10688686	-79,13454437	-944,9157715	-0,040854
Cloud_Mesh_Distance_BA_R0_R3	21,16865921	-80,05348969	-945,2981567	-0,069448
Cloud_Mesh_Distance_BA_R0_R3	21,42039871	-80,00923157	-945,1022949	-0,075449
Cloud_Mesh_Distance_BA_R0_R3	21,38692474	-79,8919754	-945,0950317	-0,073208
Cloud_Mesh_Distance_BA_R0_R3	21,31159782	-79,86852264	-945,1471558	-0,069238
Cloud_Mesh_Distance_BA_R0_R3	21,4758358	-79,92595673	-945,0316162	-0,080858
Cloud_Mesh_Distance_BA_R0_R3	15,12268639	-78,90608215	-944,9013062	-0,042611
Cloud_Mesh_Distance_BA_R0_R3	20,97919083	-79,6019516	-945,3128662	-0,056347
Cloud_Mesh_Distance_BA_R0_R3	21,2218132	-79,63195038	-945,1394653	-0,069758
Cloud_Mesh_Distance_BA_R0_R3	21,20195389	-79,84760284	-945,2247315	-0,063427
Cloud_Mesh_Distance_BA_R0_R3	21,45857239	-79,8194809	-945,0110474	-0,081881

Cloud_Mesh_Distance_BA_R0_R3	21,10923004	-79,53283691	-945,1925049	-0,065858
Cloud_Mesh_Distance_BA_R0_R3	21,11998558	-79,41827393	-945,1386719	-0,065126
Cloud_Mesh_Distance_BA_R0_R3	21,23215866	-79,42474365	-945,0457153	-0,072012
Cloud_Mesh_Distance_BA_R0_R3	15,16892052	-78,71596527	-944,9544067	-0,041664
Cloud_Mesh_Distance_BA_R0_R3	15,18740273	-78,55745697	-944,9066162	-0,040267
Cloud_Mesh_Distance_BA_R0_R3	20,94772339	-79,24269867	-945,2072754	-0,061931
Cloud_Mesh_Distance_BA_R0_R3	20,95923996	-79,17015839	-945,1665649	-0,064456
Cloud_Mesh_Distance_BA_R0_R3	21,00561142	-79,38860321	-945,2195435	-0,061414
Cloud_Mesh_Distance_BA_R0_R3	21,1350708	-79,3110733	-945,0786133	-0,068749
Cloud_Mesh_Distance_BA_R0_R3	20,77698517	-78,90795135	-945,2030029	-0,060983
Cloud_Mesh_Distance_BA_R0_R3	15,31723785	-78,26366425	-945,0545654	-0,033645
Cloud_Mesh_Distance_BA_R0_R3	15,26243782	-78,41481781	-945,0281372	-0,032716
Cloud_Mesh_Distance_BA_R0_R3	15,21503544	-78,40727997	-944,8676148	-0,041208
Cloud_Mesh_Distance_BA_R0_R3	20,74818802	-78,77875519	-945,1663208	-0,064205
Cloud_Mesh_Distance_BA_R0_R3	15,348526	-78,12801361	-945,0039673	-0,035239
Cloud_Mesh_Distance_BA_R0_R3	20,7727623	-78,65642548	-945,0789795	-0,0664
Cloud_Mesh_Distance_BA_R0_R3	20,69607925	-78,56874847	-945,1068726	-0,066311
Cloud_Mesh_Distance_BA_R0_R3	15,38609505	-77,991539	-944,9465332	-0,039131
Cloud_Mesh_Distance_BA_R0_R3	20,37912178	-78,28429413	-945,2647095	-0,071545
Cloud_Mesh_Distance_BA_R0_R3	20,50969124	-78,39861298	-945,1991577	-0,068104
Cloud_Mesh_Distance_BA_R0_R3	15,5125103	-77,82829285	-945,0906372	-0,036629
Cloud_Mesh_Distance_BA_R0_R3	15,56897449	-77,716362	-945,0834351	-0,036826
Cloud_Mesh_Distance_BA_R0_R3	15,53050137	-77,69976044	-944,9574585	-0,042715
Cloud_Mesh_Distance_BA_R0_R3	15,76115131	-77,47583771	-945,1636963	-0,045147
Cloud_Mesh_Distance_BA_R0_R3	15,64455032	-77,6460495	-945,1565552	-0,040332
Cloud_Mesh_Distance_BA_R0_R3	20,3886795	-78,11401367	-945,1591797	-0,081393
Cloud_Mesh_Distance_BA_R0_R3	20,27822113	-78,12242889	-945,272522	-0,082965
Cloud_Mesh_Distance_BA_R0_R3	20,465868	-78,08170319	-945,0585938	-0,074716
Cloud_Mesh_Distance_BA_R0_R3	15,85333157	-77,42481232	-945,2502441	-0,058022
Cloud_Mesh_Distance_BA_R0_R3	20,09943008	-77,81828308	-945,258728	-0,104251
Cloud_Mesh_Distance_BA_R0_R3	20,30866241	-78,00624847	-945,1750488	-0,085947
Cloud_Mesh_Distance_BA_R0_R3	20,29499245	-77,8730545	-945,1026611	-0,086689
Cloud_Mesh_Distance_BA_R0_R3	15,91764069	-77,18636322	-945,0445557	-0,049074
Cloud_Mesh_Distance_BA_R0_R3	15,86535454	-77,3265152	-945,1506958	-0,052154
Cloud_Mesh_Distance_BA_R0_R3	19,97363663	-77,68159485	-945,2872315	-0,112796
Cloud_Mesh_Distance_BA_R0_R3	19,78963089	-77,57177734	-945,3748169	-0,116973
Cloud_Mesh_Distance_BA_R0_R3	20,19547081	-77,73723602	-945,1134033	-0,095009
Cloud_Mesh_Distance_BA_R0_R3	20,06407928	-77,70442963	-945,2192993	-0,107409
Cloud_Mesh_Distance_BA_R0_R3	16,07581139	-76,99141693	-944,998291	-0,060676
Cloud_Mesh_Distance_BA_R0_R3	16,11178589	-77,10230255	-945,2020874	-0,072509
Cloud_Mesh_Distance_BA_R0_R3	16,18709183	-76,96964264	-945,112854	-0,070413
Cloud_Mesh_Distance_BA_R0_R3	16,03964424	-77,08591461	-945,0877075	-0,065852
Cloud_Mesh_Distance_BA_R0_R3	16,41874504	-76,93021393	-945,2918701	-0,100731
Cloud_Mesh_Distance_BA_R0_R3	19,76449394	-77,35897064	-945,2567749	-0,107233
Cloud_Mesh_Distance_BA_R0_R3	19,91135979	-77,58496857	-945,2806397	-0,111354
Cloud_Mesh_Distance_BA_R0_R3	16,05073357	-76,95978546	-944,9012451	-0,051507
Cloud_Mesh_Distance_BA_R0_R3	16,25669289	-76,84054565	-945,0003662	-0,07071
Cloud_Mesh_Distance_BA_R0_R3	16,30958557	-76,96414948	-945,2359009	-0,092337
Cloud_Mesh_Distance_BA_R0_R3	16,37487221	-76,85068512	-945,1466675	-0,090624
Cloud_Mesh_Distance_BA_R0_R3	16,53773117	-76,84854889	-945,2850342	-0,107386

Cloud_Mesh_Distance_BA_R0_R3	19,41656494	-77,12836456	-945,3475952	-0,081384
Cloud_Mesh_Distance_BA_R0_R3	19,4940033	-77,0526123	-945,237915	-0,068813
Cloud_Mesh_Distance_BA_R0_R3	19,53781509	-77,16771698	-945,2963867	-0,08963
Cloud_Mesh_Distance_BA_R0_R3	19,65313148	-77,11956787	-945,171936	-0,081147
Cloud_Mesh_Distance_BA_R0_R3	19,64594078	-77,30297089	-945,3128052	-0,100358
Cloud_Mesh_Distance_BA_R0_R3	16,55584908	-76,72167206	-945,1237793	-0,08915
Cloud_Mesh_Distance_BA_R0_R3	16,84907341	-76,61492157	-945,1815186	-0,100419
Cloud_Mesh_Distance_BA_R0_R3	19,49492645	-76,96311188	-945,1577759	-0,059903
Cloud_Mesh_Distance_BA_R0_R3	19,58049011	-77,0085907	-945,1348877	-0,066287
Cloud_Mesh_Distance_BA_R0_R3	19,37800217	-77,04148102	-945,3049316	-0,066272
Cloud_Mesh_Distance_BA_R0_R3	16,94539642	-76,61484528	-945,2388306	-0,104913
Cloud_Mesh_Distance_BA_R0_R3	16,91641617	-76,54575348	-945,116394	-0,092199
Cloud_Mesh_Distance_BA_R0_R3	17,2337532	-76,46234894	-945,1503296	-0,072926
Cloud_Mesh_Distance_BA_R0_R3	17,25153732	-76,52919769	-945,2617188	-0,080385
Cloud_Mesh_Distance_BA_R0_R3	18,48439026	-76,58896637	-945,3133545	0,011316
Cloud_Mesh_Distance_BA_R0_R3	18,50894928	-76,67133331	-945,3920288	-0,002463
Cloud_Mesh_Distance_BA_R0_R3	18,7247963	-76,59300995	-945,2102051	0,01029
Cloud_Mesh_Distance_BA_R0_R3	19,11823082	-76,82862091	-945,2619629	-0,03492
Cloud_Mesh_Distance_BA_R0_R3	19,26421928	-76,77749634	-945,1219482	-0,032939
Cloud_Mesh_Distance_BA_R0_R3	19,39373016	-76,96521759	-945,2294922	-0,055635
Cloud_Mesh_Distance_BA_R0_R3	19,44049072	-76,87008667	-945,104187	-0,047435
Cloud_Mesh_Distance_BA_R0_R3	17,38009644	-76,46576691	-945,2121582	-0,060119
Cloud_Mesh_Distance_BA_R0_R3	17,71095467	-76,45207977	-945,2661743	-0,028603
Cloud_Mesh_Distance_BA_R0_R3	17,87966537	-76,3973465	-945,1838379	-0,008852
Cloud_Mesh_Distance_BA_R0_R3	18,06080246	-76,42865753	-945,2228394	0,007661
Cloud_Mesh_Distance_BA_R0_R3	18,2137413	-76,42649078	-945,1848755	0,012358
Cloud_Mesh_Distance_BA_R0_R3	18,17488098	-76,50183868	-945,3101807	0,009128
Cloud_Mesh_Distance_BA_R0_R3	18,34456253	-76,58241272	-945,3598633	0,008968
Cloud_Mesh_Distance_BA_R0_R3	18,59489441	-76,59580231	-945,2755127	0,012061
Cloud_Mesh_Distance_BA_R0_R3	18,16704941	-76,37355804	-945,1005249	0,005042
Cloud_Mesh_Distance_BA_R0_R3	18,33490944	-76,37813568	-945,0565186	0,009072
Cloud_Mesh_Distance_BA_R0_R3	22,43249321	-82,33887482	-944,9234009	-0,160318
Cloud_Mesh_Distance_BA_R0_R3	15,68735886	-81,57830048	-944,5229492	0,241029
Cloud_Mesh_Distance_BA_R0_R3	22,3935051	-82,06937408	-944,8128052	-0,045403
Cloud_Mesh_Distance_BA_R0_R3	22,34218788	-82,26996613	-944,9934082	-0,156423
Cloud_Mesh_Distance_BA_R0_R3	22,43811607	-82,21839142	-944,8417969	-0,067166
Cloud_Mesh_Distance_BA_R0_R3	15,6926775	-81,37732697	-944,5180054	0,101871
Cloud_Mesh_Distance_BA_R0_R3	15,66268063	-81,25606537	-944,4360352	0,021179
Cloud_Mesh_Distance_BA_R0_R3	22,24464798	-81,8642807	-944,8821411	-0,04908
Cloud_Mesh_Distance_BA_R0_R3	22,31167221	-81,90381622	-944,8251343	-0,048522
Cloud_Mesh_Distance_BA_R0_R3	15,64073372	-81,03751373	-944,6586914	-0,028644
Cloud_Mesh_Distance_BA_R0_R3	15,66872883	-81,20565033	-944,5664063	0,023376
Cloud_Mesh_Distance_BA_R0_R3	22,07096291	-81,73644257	-944,9996338	-0,055667
Cloud_Mesh_Distance_BA_R0_R3	22,25777054	-81,76415253	-944,8226929	-0,051794
Cloud_Mesh_Distance_BA_R0_R3	15,47320557	-80,75205231	-944,5751343	-0,103658
Cloud_Mesh_Distance_BA_R0_R3	15,51700401	-80,89148712	-944,477478	-0,090472
Cloud_Mesh_Distance_BA_R0_R3	15,5572176	-80,88025665	-944,6175537	-0,082504
Cloud_Mesh_Distance_BA_R0_R3	15,65235138	-80,94530487	-944,7810669	-0,040493
Cloud_Mesh_Distance_BA_R0_R3	22,0590229	-81,54839325	-944,9388428	-0,067142
Cloud_Mesh_Distance_BA_R0_R3	22,18935013	-81,45534515	-944,7784424	-0,069142

Cloud_Mesh_Distance_BA_R0_R3	22,17265129	-81,65805817	-944,8693237	-0,058931
Cloud_Mesh_Distance_BA_R0_R3	15,52049732	-80,74219513	-944,7043457	-0,100493
Cloud_Mesh_Distance_BA_R0_R3	15,5976038	-80,8459549	-944,7548218	-0,074307
Cloud_Mesh_Distance_BA_R0_R3	21,96025467	-81,39528656	-944,9783936	-0,074385
Cloud_Mesh_Distance_BA_R0_R3	22,08408546	-81,3759079	-944,8587647	-0,073718
Cloud_Mesh_Distance_BA_R0_R3	22,04247665	-81,21234131	-944,8514404	-0,083843
Cloud_Mesh_Distance_BA_R0_R3	15,2504673	-80,30801392	-944,6260376	-0,110665
Cloud_Mesh_Distance_BA_R0_R3	15,28175735	-80,46531677	-944,4763184	-0,108988
Cloud_Mesh_Distance_BA_R0_R3	15,3389473	-80,45675659	-944,6576538	-0,110535
Cloud_Mesh_Distance_BA_R0_R3	15,38034344	-80,61536407	-944,5309448	-0,117329
Cloud_Mesh_Distance_BA_R0_R3	21,89637566	-81,21872711	-944,9854126	-0,084216
Cloud_Mesh_Distance_BA_R0_R3	21,96295547	-81,08150482	-944,8912964	-0,090854
Cloud_Mesh_Distance_BA_R0_R3	22,14234924	-81,12574005	-944,7315674	-0,086537
Cloud_Mesh_Distance_BA_R0_R3	21,68662071	-81,0909729	-945,12854	-0,088872
Cloud_Mesh_Distance_BA_R0_R3	21,83229828	-81,04547882	-944,9973145	-0,090056
Cloud_Mesh_Distance_BA_R0_R3	21,7265625	-80,94768524	-945,0633545	-0,09489
Cloud_Mesh_Distance_BA_R0_R3	21,9592495	-80,90502167	-944,8519898	-0,097934
Cloud_Mesh_Distance_BA_R0_R3	15,17990589	-80,07108307	-944,7157593	-0,093619
Cloud_Mesh_Distance_BA_R0_R3	15,16291523	-80,12006378	-944,6002808	-0,102757
Cloud_Mesh_Distance_BA_R0_R3	21,82406998	-80,85509491	-944,9606934	-0,099063
Cloud_Mesh_Distance_BA_R0_R3	21,81533432	-80,66530609	-944,9259644	-0,102853
Cloud_Mesh_Distance_BA_R0_R3	15,10580158	-79,80890656	-944,7294922	-0,082465
Cloud_Mesh_Distance_BA_R0_R3	21,75117874	-80,34322357	-944,9074707	-0,101028
Cloud_Mesh_Distance_BA_R0_R3	21,76183319	-80,51076508	-944,9382324	-0,103538
Cloud_Mesh_Distance_BA_R0_R3	21,92858124	-80,64065552	-944,8183594	-0,105085
Cloud_Mesh_Distance_BA_R0_R3	15,03473759	-79,56267548	-944,6074219	-0,070741
Cloud_Mesh_Distance_BA_R0_R3	15,05939865	-79,5328064	-944,7312012	-0,061242
Cloud_Mesh_Distance_BA_R0_R3	15,06131554	-79,67441559	-944,6629028	-0,071803
Cloud_Mesh_Distance_BA_R0_R3	15,06720161	-79,8214798	-944,5552368	-0,087261
Cloud_Mesh_Distance_BA_R0_R3	21,54210472	-80,25359344	-945,0638428	-0,089358
Cloud_Mesh_Distance_BA_R0_R3	21,77830887	-80,18288422	-944,8398438	-0,103801
Cloud_Mesh_Distance_BA_R0_R3	21,63654327	-80,32562256	-945,0028076	-0,09396
Cloud_Mesh_Distance_BA_R0_R3	21,85151672	-80,28362274	-944,800293	-0,104051
Cloud_Mesh_Distance_BA_R0_R3	15,06506062	-79,41027069	-944,7858887	-0,052764
Cloud_Mesh_Distance_BA_R0_R3	15,09207439	-79,49610901	-944,8514404	-0,057888
Cloud_Mesh_Distance_BA_R0_R3	21,72172928	-80,04964447	-944,8521118	-0,10001
Cloud_Mesh_Distance_BA_R0_R3	21,63465691	-80,01564026	-944,9205322	-0,093533
Cloud_Mesh_Distance_BA_R0_R3	21,65771103	-80,17520905	-944,9468384	-0,094002
Cloud_Mesh_Distance_BA_R0_R3	15,05850029	-79,26326752	-944,7713013	-0,048091
Cloud_Mesh_Distance_BA_R0_R3	15,06151295	-79,06809998	-944,7470093	-0,047867
Cloud_Mesh_Distance_BA_R0_R3	21,3351059	-79,53593445	-945,0050659	-0,075717
Cloud_Mesh_Distance_BA_R0_R3	21,35519409	-79,73282623	-945,067688	-0,075423
Cloud_Mesh_Distance_BA_R0_R3	21,49438858	-79,69516754	-944,9320679	-0,08273
Cloud_Mesh_Distance_BA_R0_R3	15,07320499	-78,90048981	-944,7205811	-0,052698
Cloud_Mesh_Distance_BA_R0_R3	21,14879227	-79,20795441	-945,0152588	-0,073713
Cloud_Mesh_Distance_BA_R0_R3	21,33571243	-79,4254837	-944,9529419	-0,080591
Cloud_Mesh_Distance_BA_R0_R3	21,34262085	-79,31932068	-944,8927612	-0,081553
Cloud_Mesh_Distance_BA_R0_R3	15,15348148	-78,49625397	-944,7206421	-0,051946
Cloud_Mesh_Distance_BA_R0_R3	20,96759605	-79,0785675	-945,1168213	-0,067226
Cloud_Mesh_Distance_BA_R0_R3	21,0498085	-79,1940918	-945,0986328	-0,069306

Cloud_Mesh_Distance_BA_R0_R3	21,25112915	-79,2126236	-944,9223023	-0,077064
Cloud_Mesh_Distance_BA_R0_R3	15,21030331	-78,33104706	-944,7608032	-0,052576
Cloud_Mesh_Distance_BA_R0_R3	20,97618866	-78,929039	-945,0314331	-0,067544
Cloud_Mesh_Distance_BA_R0_R3	20,87763023	-78,98751068	-945,152771	-0,062402
Cloud_Mesh_Distance_BA_R0_R3	20,87824821	-78,8017807	-945,0559082	-0,06618
Cloud_Mesh_Distance_BA_R0_R3	21,08145905	-78,83638763	-944,8748779	-0,072633
Cloud_Mesh_Distance_BA_R0_R3	15,27169418	-78,25227356	-944,8983154	-0,04074
Cloud_Mesh_Distance_BA_R0_R3	20,61262512	-78,507164	-945,1556397	-0,067917
Cloud_Mesh_Distance_BA_R0_R3	20,76541519	-78,52090454	-945,0089111	-0,065637
Cloud_Mesh_Distance_BA_R0_R3	20,68231964	-78,47375488	-945,0670166	-0,066216
Cloud_Mesh_Distance_BA_R0_R3	20,98881912	-78,71735382	-944,8969116	-0,067239
Cloud_Mesh_Distance_BA_R0_R3	20,98128891	-78,82519531	-944,9680786	-0,068993
Cloud_Mesh_Distance_BA_R0_R3	15,32278252	-78,05910492	-944,8225098	-0,041754
Cloud_Mesh_Distance_BA_R0_R3	20,61647415	-78,35988617	-945,0692139	-0,068942
Cloud_Mesh_Distance_BA_R0_R3	15,38400936	-77,92060089	-944,8295288	-0,044563
Cloud_Mesh_Distance_BA_R0_R3	15,35021687	-77,9162674	-944,6731567	-0,054534
Cloud_Mesh_Distance_BA_R0_R3	15,44771099	-77,84305573	-944,9259644	-0,04308
Cloud_Mesh_Distance_BA_R0_R3	20,60623169	-78,22322845	-944,9970703	-0,071608
Cloud_Mesh_Distance_BA_R0_R3	15,59592342	-77,6210556	-945,0149536	-0,040333
Cloud_Mesh_Distance_BA_R0_R3	15,58653069	-77,55056	-944,8707275	-0,045391
Cloud_Mesh_Distance_BA_R0_R3	20,55524826	-78,08927155	-944,9662476	-0,072388
Cloud_Mesh_Distance_BA_R0_R3	20,50222397	-78,22176361	-945,1068726	-0,074987
Cloud_Mesh_Distance_BA_R0_R3	15,66713619	-77,49221802	-944,9945068	-0,047702
Cloud_Mesh_Distance_BA_R0_R3	20,29791451	-77,76767731	-945,0283203	-0,088433
Cloud_Mesh_Distance_BA_R0_R3	20,40673828	-77,97145081	-945,0507202	-0,079243
Cloud_Mesh_Distance_BA_R0_R3	20,38641167	-77,80893707	-944,9615479	-0,083262
Cloud_Mesh_Distance_BA_R0_R3	20,52568054	-77,90962219	-944,8768311	-0,072121
Cloud_Mesh_Distance_BA_R0_R3	20,48642731	-78,01273346	-944,9918823	-0,073304
Cloud_Mesh_Distance_BA_R0_R3	15,78430271	-77,29517365	-944,9517822	-0,042801
Cloud_Mesh_Distance_BA_R0_R3	15,82931805	-77,13747406	-944,7542114	-0,053618
Cloud_Mesh_Distance_BA_R0_R3	15,74922085	-77,25592804	-944,7799683	-0,046447
Cloud_Mesh_Distance_BA_R0_R3	15,8804512	-77,14493561	-944,9058228	-0,045975
Cloud_Mesh_Distance_BA_R0_R3	20,10632324	-77,60692596	-945,1130981	-0,103502
Cloud_Mesh_Distance_BA_R0_R3	20,06287384	-77,47690582	-945,06427	-0,1031
Cloud_Mesh_Distance_BA_R0_R3	20,01255226	-77,5909729	-945,192627	-0,108073
Cloud_Mesh_Distance_BA_R0_R3	20,1987114	-77,5296402	-944,9625855	-0,088331
Cloud_Mesh_Distance_BA_R0_R3	20,32114792	-77,65473175	-944,9215698	-0,081924
Cloud_Mesh_Distance_BA_R0_R3	15,97708511	-77,05579376	-944,9411011	-0,049731
Cloud_Mesh_Distance_BA_R0_R3	19,95999527	-77,49248505	-945,175354	-0,106587
Cloud_Mesh_Distance_BA_R0_R3	20,13874817	-77,39942169	-944,9285278	-0,081826
Cloud_Mesh_Distance_BA_R0_R3	16,14156532	-76,90073395	-944,9440918	-0,061804
Cloud_Mesh_Distance_BA_R0_R3	16,38205338	-76,72496796	-944,9541626	-0,072506
Cloud_Mesh_Distance_BA_R0_R3	16,21685219	-76,7844696	-944,8372192	-0,063732
Cloud_Mesh_Distance_BA_R0_R3	19,82285881	-77,2522049	-945,1292114	-0,094817
Cloud_Mesh_Distance_BA_R0_R3	19,85040474	-77,43894196	-945,2373047	-0,108626
Cloud_Mesh_Distance_BA_R0_R3	19,96079254	-77,37375641	-945,0904541	-0,101489
Cloud_Mesh_Distance_BA_R0_R3	20,00009537	-77,23886871	-944,947937	-0,077642
Cloud_Mesh_Distance_BA_R0_R3	16,52261353	-76,64242554	-944,9626465	-0,082957
Cloud_Mesh_Distance_BA_R0_R3	16,73498917	-76,65891266	-945,170105	-0,100515
Cloud_Mesh_Distance_BA_R0_R3	16,7774086	-76,58347321	-945,0828247	-0,096171

Cloud_Mesh_Distance_BA_R0_R3	19,69170189	-76,99147797	-945,0269775	-0,062575
Cloud_Mesh_Distance_BA_R0_R3	16,65049362	-76,60889435	-945,0249634	-0,088641
Cloud_Mesh_Distance_BA_R0_R3	16,58291626	-76,56925201	-944,8876343	-0,075591
Cloud_Mesh_Distance_BA_R0_R3	16,7454071	-76,5058136	-944,9216919	-0,07458
Cloud_Mesh_Distance_BA_R0_R3	17,06168365	-76,42699432	-945,0014648	-0,07795
Cloud_Mesh_Distance_BA_R0_R3	17,09059906	-76,48820496	-945,1243286	-0,083456
Cloud_Mesh_Distance_BA_R0_R3	16,94064903	-76,45819855	-944,9824829	-0,080293
Cloud_Mesh_Distance_BA_R0_R3	18,92743683	-76,55819702	-945,0552368	0,00199
Cloud_Mesh_Distance_BA_R0_R3	19,04824448	-76,60534668	-945,0466919	-0,006175
Cloud_Mesh_Distance_BA_R0_R3	18,88662529	-76,63108063	-945,173584	0,001016
Cloud_Mesh_Distance_BA_R0_R3	19,07817078	-76,70717621	-945,1557007	-0,017805
Cloud_Mesh_Distance_BA_R0_R3	19,444664	-76,73563385	-944,9419556	-0,032118
Cloud_Mesh_Distance_BA_R0_R3	19,56846237	-76,93494415	-945,0739136	-0,057035
Cloud_Mesh_Distance_BA_R0_R3	16,99299812	-76,39653015	-944,8988037	-0,071823
Cloud_Mesh_Distance_BA_R0_R3	17,16085052	-76,42908478	-945,0587158	-0,075163
Cloud_Mesh_Distance_BA_R0_R3	17,51501274	-76,38355255	-945,1104126	-0,04491
Cloud_Mesh_Distance_BA_R0_R3	17,29119682	-76,40305328	-945,0706177	-0,066644
Cloud_Mesh_Distance_BA_R0_R3	17,33491898	-76,32350922	-944,9275513	-0,054616
Cloud_Mesh_Distance_BA_R0_R3	17,72060776	-76,37050629	-945,1248169	-0,026348
Cloud_Mesh_Distance_BA_R0_R3	18,02516556	-76,34606171	-945,0708618	-0,005589
Cloud_Mesh_Distance_BA_R0_R3	18,33895302	-76,46743011	-945,2097168	0,014199
Cloud_Mesh_Distance_BA_R0_R3	18,64841652	-76,53511047	-945,1737061	0,015267
Cloud_Mesh_Distance_BA_R0_R3	18,50192261	-76,5178299	-945,2166138	0,016539
Cloud_Mesh_Distance_BA_R0_R3	18,78948784	-76,52957916	-945,0927734	0,010385
Cloud_Mesh_Distance_BA_R0_R3	18,79788208	-76,46140289	-944,9817505	0,003249
Cloud_Mesh_Distance_BA_R0_R3	17,50259399	-76,30516815	-944,942627	-0,043394
Cloud_Mesh_Distance_BA_R0_R3	17,43148422	-76,27045441	-944,835022	-0,0477
Cloud_Mesh_Distance_BA_R0_R3	17,6595993	-76,311203	-944,9894409	-0,035298
Cloud_Mesh_Distance_BA_R0_R3	17,59327698	-76,24749756	-944,8132324	-0,042384
Cloud_Mesh_Distance_BA_R0_R3	17,80014038	-76,25783539	-944,8635254	-0,030746
Cloud_Mesh_Distance_BA_R0_R3	17,99922562	-76,27570343	-944,9014893	-0,018545
Cloud_Mesh_Distance_BA_R0_R3	17,84531021	-76,32257843	-945,0304565	-0,02108
Cloud_Mesh_Distance_BA_R0_R3	18,20679474	-76,30623627	-944,9378052	-0,003808
Cloud_Mesh_Distance_BA_R0_R3	18,47303581	-76,4318161	-945,098999	0,011342
Cloud_Mesh_Distance_BA_R0_R3	18,55135155	-76,36493683	-944,9327393	0,005039
Cloud_Mesh_Distance_BA_R0_R3	18,64386368	-76,45731354	-945,059021	0,01025
Cloud_Mesh_Distance_BA_R0_R3	18,75024223	-76,39852142	-944,8937988	0,007324
Cloud_Mesh_Distance_BA_R0_R3	18,90852928	-76,4996109	-944,9785156	0,00012
Cloud_Mesh_Distance_BA_R0_R3	22,63869858	-82,52988434	-944,74646	-0,055158
Cloud_Mesh_Distance_BA_R0_R3	22,56810951	-82,49176788	-944,8312988	-0,105817
Cloud_Mesh_Distance_BA_R0_R3	22,76758957	-82,59766388	-944,5699463	-0,118251
Cloud_Mesh_Distance_BA_R0_R3	22,61896133	-82,39260101	-944,6871948	-0,045955
Cloud_Mesh_Distance_BA_R0_R3	22,53303146	-82,28743744	-944,7498779	-0,047488
Cloud_Mesh_Distance_BA_R0_R3	22,6634903	-82,29737091	-944,5498657	-0,03758
Cloud_Mesh_Distance_BA_R0_R3	22,4822998	-82,12645721	-944,7263184	-0,044761
Cloud_Mesh_Distance_BA_R0_R3	22,61636734	-82,19008636	-944,5600586	-0,040418
Cloud_Mesh_Distance_BA_R0_R3	22,60801125	-82,29036713	-944,6365356	-0,041733
Cloud_Mesh_Distance_BA_R0_R3	15,6548481	-81,27248383	-944,2699585	-0,002991
Cloud_Mesh_Distance_BA_R0_R3	15,6140337	-81,13555145	-944,3937378	-0,035819
Cloud_Mesh_Distance_BA_R0_R3	15,63559246	-81,13037872	-944,517395	-0,021278

Cloud_Mesh_Distance_BA_R0_R3	15,69707012	-81,41159821	-944,326416	0,071935
Cloud_Mesh_Distance_BA_R0_R3	22,51565933	-81,91216278	-944,5581055	-0,049997
Cloud_Mesh_Distance_BA_R0_R3	22,56123352	-82,05428314	-944,5654297	-0,042076
Cloud_Mesh_Distance_BA_R0_R3	22,4787674	-82,00521088	-944,6615601	-0,045996
Cloud_Mesh_Distance_BA_R0_R3	15,58722305	-81,01470184	-944,5118408	-0,058367
Cloud_Mesh_Distance_BA_R0_R3	15,43195248	-80,77892303	-944,3961792	-0,108315
Cloud_Mesh_Distance_BA_R0_R3	22,276577	-81,59143829	-944,7315064	-0,056776
Cloud_Mesh_Distance_BA_R0_R3	22,29276657	-81,44080353	-944,6593628	-0,067457
Cloud_Mesh_Distance_BA_R0_R3	22,37875748	-81,50414276	-944,574585	-0,063225
Cloud_Mesh_Distance_BA_R0_R3	15,22041416	-80,3302536	-944,4906006	-0,106891
Cloud_Mesh_Distance_BA_R0_R3	15,33818722	-80,63022614	-944,3565674	-0,108743
Cloud_Mesh_Distance_BA_R0_R3	22,07952118	-81,02058411	-944,7664185	-0,091489
Cloud_Mesh_Distance_BA_R0_R3	22,1652298	-81,04318237	-944,6864624	-0,090501
Cloud_Mesh_Distance_BA_R0_R3	22,16419411	-81,26054382	-944,744751	-0,080909
Cloud_Mesh_Distance_BA_R0_R3	22,25201416	-81,08733368	-944,6035156	-0,087057
Cloud_Mesh_Distance_BA_R0_R3	15,18928909	-80,2248764	-944,546875	-0,105368
Cloud_Mesh_Distance_BA_R0_R3	21,90763664	-80,76094818	-944,8658447	-0,101848
Cloud_Mesh_Distance_BA_R0_R3	22,09186172	-80,83342743	-944,7088013	-0,099674
Cloud_Mesh_Distance_BA_R0_R3	22,18118286	-80,9353714	-944,642395	-0,095832
Cloud_Mesh_Distance_BA_R0_R3	15,13709545	-80,13773346	-944,4559937	-0,109337
Cloud_Mesh_Distance_BA_R0_R3	15,20259476	-80,18090057	-944,6541748	-0,101225
Cloud_Mesh_Distance_BA_R0_R3	22,11122131	-80,64577484	-944,6424561	-0,106986
Cloud_Mesh_Distance_BA_R0_R3	22,00295639	-80,7286911	-944,7704468	-0,10447
Cloud_Mesh_Distance_BA_R0_R3	15,12046719	-79,98259735	-944,6118774	-0,09531
Cloud_Mesh_Distance_BA_R0_R3	22,00183868	-80,48171234	-944,7093506	-0,108541
Cloud_Mesh_Distance_BA_R0_R3	22,00566101	-80,57666779	-944,7301636	-0,108232
Cloud_Mesh_Distance_BA_R0_R3	15,01138306	-79,51935577	-944,4779053	-0,08266
Cloud_Mesh_Distance_BA_R0_R3	15,03277874	-79,67330933	-944,5073853	-0,087824
Cloud_Mesh_Distance_BA_R0_R3	21,8621788	-80,1782608	-944,7591553	-0,107234
Cloud_Mesh_Distance_BA_R0_R3	21,90162277	-80,46149445	-944,8000488	-0,104592
Cloud_Mesh_Distance_BA_R0_R3	22,01787949	-80,39055634	-944,6686401	-0,110997
Cloud_Mesh_Distance_BA_R0_R3	22,07334328	-80,49324799	-944,6416626	-0,109684
Cloud_Mesh_Distance_BA_R0_R3	14,99930573	-79,34557343	-944,4545288	-0,075825
Cloud_Mesh_Distance_BA_R0_R3	15,02890015	-79,40738678	-944,6363525	-0,064889
Cloud_Mesh_Distance_BA_R0_R3	15,02108479	-79,20790863	-944,6000977	-0,063431
Cloud_Mesh_Distance_BA_R0_R3	21,68111801	-79,87421417	-944,8284302	-0,095795
Cloud_Mesh_Distance_BA_R0_R3	21,6763649	-79,94242096	-944,8583374	-0,095097
Cloud_Mesh_Distance_BA_R0_R3	21,81894302	-80,06760406	-944,7654419	-0,105974
Cloud_Mesh_Distance_BA_R0_R3	14,99622917	-79,16173553	-944,416687	-0,069863
Cloud_Mesh_Distance_BA_R0_R3	15,02754307	-78,99668121	-944,5565796	-0,062789
Cloud_Mesh_Distance_BA_R0_R3	21,58613586	-79,86027527	-944,9118042	-0,090229
Cloud_Mesh_Distance_BA_R0_R3	21,70466232	-79,78061676	-944,7697754	-0,098452
Cloud_Mesh_Distance_BA_R0_R3	15,06185246	-78,81702423	-944,6119995	-0,055515
Cloud_Mesh_Distance_BA_R0_R3	21,4288559	-79,58246613	-944,9422607	-0,080971
Cloud_Mesh_Distance_BA_R0_R3	21,61532974	-79,65297699	-944,800354	-0,094531
Cloud_Mesh_Distance_BA_R0_R3	21,52500534	-79,53578949	-944,8313599	-0,090325
Cloud_Mesh_Distance_BA_R0_R3	15,08588219	-78,65595245	-944,5786133	-0,056753
Cloud_Mesh_Distance_BA_R0_R3	15,11620426	-78,71906281	-944,774353	-0,052307
Cloud_Mesh_Distance_BA_R0_R3	21,35004807	-79,21535492	-944,8273315	-0,083153
Cloud_Mesh_Distance_BA_R0_R3	21,44846535	-79,21717072	-944,7315064	-0,087943

Cloud_Mesh_Distance_BA_R0_R3	21,54368973	-79,32243347	-944,6980591	-0,089927
Cloud_Mesh_Distance_BA_R0_R3	21,43708611	-79,41979218	-944,8564453	-0,084754
Cloud_Mesh_Distance_BA_R0_R3	21,53568649	-79,42974854	-944,7661743	-0,088436
Cloud_Mesh_Distance_BA_R0_R3	21,06765747	-79,01800537	-944,9926148	-0,067422
Cloud_Mesh_Distance_BA_R0_R3	21,1600647	-79,10001373	-944,9481201	-0,072618
Cloud_Mesh_Distance_BA_R0_R3	15,24705505	-78,18833923	-944,7096558	-0,051984
Cloud_Mesh_Distance_BA_R0_R3	21,16490936	-78,9489975	-944,8568726	-0,075681
Cloud_Mesh_Distance_BA_R0_R3	21,23303986	-78,87227631	-944,7390747	-0,073149
Cloud_Mesh_Distance_BA_R0_R3	15,29488182	-77,9881134	-944,5360107	-0,059299
Cloud_Mesh_Distance_BA_R0_R3	15,28838158	-78,05171967	-944,6552124	-0,056339
Cloud_Mesh_Distance_BA_R0_R3	20,8972435	-78,57811737	-944,9069214	-0,066039
Cloud_Mesh_Distance_BA_R0_R3	21,09080887	-78,63407135	-944,737793	-0,061896
Cloud_Mesh_Distance_BA_R0_R3	21,17068291	-78,85288239	-944,7920532	-0,072385
Cloud_Mesh_Distance_BA_R0_R3	21,20148087	-78,74822998	-944,6931152	-0,062016
Cloud_Mesh_Distance_BA_R0_R3	20,72493744	-78,3953476	-944,9758911	-0,067359
Cloud_Mesh_Distance_BA_R0_R3	20,82020187	-78,43108368	-944,8965454	-0,068135
Cloud_Mesh_Distance_BA_R0_R3	20,9265976	-78,36295319	-944,7376709	-0,051594
Cloud_Mesh_Distance_BA_R0_R3	20,99894333	-78,61052704	-944,8207398	-0,06443
Cloud_Mesh_Distance_BA_R0_R3	15,4113903	-77,76004028	-944,6018066	-0,062626
Cloud_Mesh_Distance_BA_R0_R3	15,49490356	-77,6505661	-944,7369385	-0,05113
Cloud_Mesh_Distance_BA_R0_R3	15,41657257	-77,81339264	-944,7576294	-0,047947
Cloud_Mesh_Distance_BA_R0_R3	20,68190956	-78,2852478	-944,9542236	-0,069201
Cloud_Mesh_Distance_BA_R0_R3	20,67943573	-78,15403748	-944,8709106	-0,06273
Cloud_Mesh_Distance_BA_R0_R3	20,78421021	-78,28421783	-944,8414307	-0,061721
Cloud_Mesh_Distance_BA_R0_R3	20,86459351	-78,21831512	-944,7072754	-0,052491
Cloud_Mesh_Distance_BA_R0_R3	15,55270386	-77,47225189	-944,5114136	-0,066872
Cloud_Mesh_Distance_BA_R0_R3	15,58475876	-77,4797287	-944,7106934	-0,048679
Cloud_Mesh_Distance_BA_R0_R3	20,71419907	-78,03839874	-944,7514038	-0,055966
Cloud_Mesh_Distance_BA_R0_R3	15,63393021	-77,36966705	-944,6271973	-0,049424
Cloud_Mesh_Distance_BA_R0_R3	15,67092323	-77,37727356	-944,7954712	-0,042938
Cloud_Mesh_Distance_BA_R0_R3	20,47589111	-77,68604279	-944,7679443	-0,06273
Cloud_Mesh_Distance_BA_R0_R3	20,62770653	-78,02204895	-944,8391113	-0,061966
Cloud_Mesh_Distance_BA_R0_R3	20,30569458	-77,50537872	-944,8248901	-0,0701
Cloud_Mesh_Distance_BA_R0_R3	15,91631603	-77,06472778	-944,8297119	-0,045908
Cloud_Mesh_Distance_BA_R0_R3	20,05710602	-77,35611725	-944,9815064	-0,087424
Cloud_Mesh_Distance_BA_R0_R3	20,25137711	-77,34337616	-944,7563477	-0,059603
Cloud_Mesh_Distance_BA_R0_R3	15,92095852	-77,01886749	-944,732666	-0,048261
Cloud_Mesh_Distance_BA_R0_R3	15,98834419	-76,97930145	-944,8134766	-0,045964
Cloud_Mesh_Distance_BA_R0_R3	16,00028992	-76,91057587	-944,6625366	-0,054713
Cloud_Mesh_Distance_BA_R0_R3	16,08393478	-76,88361359	-944,8069458	-0,05383
Cloud_Mesh_Distance_BA_R0_R3	16,23123741	-76,71559906	-944,6973267	-0,058035
Cloud_Mesh_Distance_BA_R0_R3	19,89721298	-77,12132263	-944,9537964	-0,065901
Cloud_Mesh_Distance_BA_R0_R3	19,78153229	-77,09662628	-945,0424194	-0,07272
Cloud_Mesh_Distance_BA_R0_R3	20,11560631	-77,30242157	-944,8775024	-0,074846
Cloud_Mesh_Distance_BA_R0_R3	16,20708847	-76,69123077	-944,5519409	-0,059039
Cloud_Mesh_Distance_BA_R0_R3	16,32421112	-76,67520142	-944,7713623	-0,065726
Cloud_Mesh_Distance_BA_R0_R3	16,45601654	-76,61186981	-944,8250122	-0,07058
Cloud_Mesh_Distance_BA_R0_R3	19,77973938	-76,9348526	-944,8884888	-0,04896
Cloud_Mesh_Distance_BA_R0_R3	19,83034515	-77,02111053	-944,9273071	-0,061701
Cloud_Mesh_Distance_BA_R0_R3	16,66342545	-76,40120697	-944,5838623	-0,055396

Cloud_Mesh_Distance_BA_R0_R3	16,85976982	-76,40888214	-944,8233643	-0,068763
Cloud_Mesh_Distance_BA_R0_R3	19,01392937	-76,53882599	-944,9732056	-0,004189
Cloud_Mesh_Distance_BA_R0_R3	19,22681808	-76,66188049	-945,0054932	-0,018899
Cloud_Mesh_Distance_BA_R0_R3	19,65947151	-76,73705292	-944,7565308	-0,028077
Cloud_Mesh_Distance_BA_R0_R3	19,63469696	-76,85962677	-944,9369507	-0,044845
Cloud_Mesh_Distance_BA_R0_R3	16,7208786	-76,43021393	-944,7364502	-0,06489
Cloud_Mesh_Distance_BA_R0_R3	16,80588913	-76,35894012	-944,6497803	-0,05727
Cloud_Mesh_Distance_BA_R0_R3	17,13903809	-76,36834717	-944,9299927	-0,067714
Cloud_Mesh_Distance_BA_R0_R3	17,25201797	-76,22991943	-944,6433106	-0,048573
Cloud_Mesh_Distance_BA_R0_R3	17,3202343	-76,26496124	-944,7788696	-0,052653
Cloud_Mesh_Distance_BA_R0_R3	17,1793499	-76,29122162	-944,7767944	-0,057538
Cloud_Mesh_Distance_BA_R0_R3	19,15005493	-76,47916412	-944,7753296	-0,006552
Cloud_Mesh_Distance_BA_R0_R3	19,02862167	-76,48642731	-944,8775635	-0,004675
Cloud_Mesh_Distance_BA_R0_R3	19,36168098	-76,61828613	-944,84198	-0,023298
Cloud_Mesh_Distance_BA_R0_R3	19,16460037	-76,55452728	-944,8943481	-0,014729
Cloud_Mesh_Distance_BA_R0_R3	19,3237114	-76,51734161	-944,7019043	-0,01679
Cloud_Mesh_Distance_BA_R0_R3	18,39879036	-76,31519318	-944,8909302	-0,001924
Cloud_Mesh_Distance_BA_R0_R3	18,90153694	-76,43693542	-944,8741455	0,001868
Cloud_Mesh_Distance_BA_R0_R3	18,88750839	-76,36948395	-944,7438355	0,003099
Cloud_Mesh_Distance_BA_R0_R3	19,03580475	-76,42272949	-944,7523804	-0,002604
Cloud_Mesh_Distance_BA_R0_R3	15,9735117	-82,24848175	-944,0293579	0,361511
Cloud_Mesh_Distance_BA_R0_R3	22,9036293	-82,67505646	-944,3470459	-0,125614
Cloud_Mesh_Distance_BA_R0_R3	15,71760273	-81,73854828	-944,3311157	0,27331
Cloud_Mesh_Distance_BA_R0_R3	15,7719202	-81,92494965	-944,2003174	0,329278
Cloud_Mesh_Distance_BA_R0_R3	22,77281189	-82,46103668	-944,4650269	-0,031021
Cloud_Mesh_Distance_BA_R0_R3	22,83466721	-82,54958344	-944,4047852	-0,077328
Cloud_Mesh_Distance_BA_R0_R3	15,77887154	-81,7718277	-944,1691284	0,219079
Cloud_Mesh_Distance_BA_R0_R3	22,70011902	-82,46787262	-944,6008911	-0,040833
Cloud_Mesh_Distance_BA_R0_R3	22,74091339	-82,3204422	-944,4268189	-0,0279
Cloud_Mesh_Distance_BA_R0_R3	22,81827927	-82,37940216	-944,3161011	-0,015559
Cloud_Mesh_Distance_BA_R0_R3	15,7106142	-81,54032135	-944,3799439	0,16023
Cloud_Mesh_Distance_BA_R0_R3	15,74537182	-81,59590912	-944,2006836	0,134169
Cloud_Mesh_Distance_BA_R0_R3	15,70844173	-81,42401886	-944,1407471	0,037497
Cloud_Mesh_Distance_BA_R0_R3	22,62945175	-82,0898056	-944,4746704	-0,041818
Cloud_Mesh_Distance_BA_R0_R3	22,64307404	-81,98842621	-944,392273	-0,040973
Cloud_Mesh_Distance_BA_R0_R3	15,52167988	-80,99044037	-944,2658081	-0,081373
Cloud_Mesh_Distance_BA_R0_R3	15,55356598	-81,01189423	-944,3764038	-0,071591
Cloud_Mesh_Distance_BA_R0_R3	15,58441162	-81,12319183	-944,2206421	-0,058347
Cloud_Mesh_Distance_BA_R0_R3	22,54169083	-81,7004776	-944,4168091	-0,053222
Cloud_Mesh_Distance_BA_R0_R3	22,4336834	-81,86617279	-944,6522217	-0,0484
Cloud_Mesh_Distance_BA_R0_R3	22,46237946	-81,65885162	-944,5197754	-0,054173
Cloud_Mesh_Distance_BA_R0_R3	22,50979233	-81,79535675	-944,5093384	-0,050892
Cloud_Mesh_Distance_BA_R0_R3	15,49694824	-80,9125061	-944,3550415	-0,091519
Cloud_Mesh_Distance_BA_R0_R3	22,35523987	-81,71595001	-944,6868897	-0,055599
Cloud_Mesh_Distance_BA_R0_R3	22,47087097	-81,47788239	-944,4388428	-0,065262
Cloud_Mesh_Distance_BA_R0_R3	15,32472897	-80,68379974	-944,1246948	-0,111298
Cloud_Mesh_Distance_BA_R0_R3	15,37606525	-80,75332642	-944,2084351	-0,105632
Cloud_Mesh_Distance_BA_R0_R3	22,26609421	-81,29961395	-944,6463013	-0,07435
Cloud_Mesh_Distance_BA_R0_R3	22,34581757	-81,34784698	-944,56604	-0,07189
Cloud_Mesh_Distance_BA_R0_R3	22,43953705	-81,29308319	-944,4246826	-0,073498

Cloud_Mesh_Distance_BA_R0_R3	15,15802288	-80,2668457	-944,2913208	-0,118105
Cloud_Mesh_Distance_BA_R0_R3	15,21444035	-80,38211823	-944,3570557	-0,111908
Cloud_Mesh_Distance_BA_R0_R3	15,31400108	-80,63646698	-944,2218018	-0,108877
Cloud_Mesh_Distance_BA_R0_R3	15,2669239	-80,5231781	-944,2843628	-0,111
Cloud_Mesh_Distance_BA_R0_R3	22,34493256	-81,20045471	-944,5246582	-0,079824
Cloud_Mesh_Distance_BA_R0_R3	15,18110657	-80,26940155	-944,4316406	-0,110729
Cloud_Mesh_Distance_BA_R0_R3	22,34344101	-81,08582306	-944,4946899	-0,086246
Cloud_Mesh_Distance_BA_R0_R3	15,10534382	-80,11383057	-944,2614136	-0,121923
Cloud_Mesh_Distance_BA_R0_R3	15,08195686	-79,97171021	-944,4186401	-0,105768
Cloud_Mesh_Distance_BA_R0_R3	22,21140289	-80,61936188	-944,5297852	-0,10912
Cloud_Mesh_Distance_BA_R0_R3	22,30672264	-80,65810394	-944,4301148	-0,107592
Cloud_Mesh_Distance_BA_R0_R3	22,28303909	-80,86536407	-944,5118408	-0,097638
Cloud_Mesh_Distance_BA_R0_R3	22,20095825	-80,75956726	-944,5773926	-0,103641
Cloud_Mesh_Distance_BA_R0_R3	15,04139328	-79,8085556	-944,3733521	-0,106729
Cloud_Mesh_Distance_BA_R0_R3	15,01330566	-79,64395142	-944,3352051	-0,100429
Cloud_Mesh_Distance_BA_R0_R3	22,16950226	-80,50595093	-944,5443115	-0,113801
Cloud_Mesh_Distance_BA_R0_R3	21,90163422	-80,10791779	-944,6977539	-0,110278
Cloud_Mesh_Distance_BA_R0_R3	21,88370705	-79,97159576	-944,6675415	-0,110465
Cloud_Mesh_Distance_BA_R0_R3	21,98282433	-80,07554626	-944,6040649	-0,115012
Cloud_Mesh_Distance_BA_R0_R3	21,95573235	-80,24969482	-944,6890869	-0,110988
Cloud_Mesh_Distance_BA_R0_R3	22,04155731	-80,29930878	-944,6179199	-0,114635
Cloud_Mesh_Distance_BA_R0_R3	22,11707306	-80,3549881	-944,5563965	-0,115624
Cloud_Mesh_Distance_BA_R0_R3	21,76201057	-79,92087555	-944,7692871	-0,101087
Cloud_Mesh_Distance_BA_R0_R3	21,84579659	-79,77313995	-944,6247559	-0,107217
Cloud_Mesh_Distance_BA_R0_R3	15,00098324	-79,00458527	-944,3656006	-0,069367
Cloud_Mesh_Distance_BA_R0_R3	15,03018856	-78,82086182	-944,4223023	-0,06396
Cloud_Mesh_Distance_BA_R0_R3	21,7379818	-79,61547089	-944,6605225	-0,097992
Cloud_Mesh_Distance_BA_R0_R3	21,64160156	-79,44578552	-944,6697998	-0,093816
Cloud_Mesh_Distance_BA_R0_R3	21,98572159	-79,66958618	-944,4210815	-0,113089
Cloud_Mesh_Distance_BA_R0_R3	21,85231018	-79,57666016	-944,5206299	-0,104154
Cloud_Mesh_Distance_BA_R0_R3	15,06875515	-78,60300446	-944,3834229	-0,068814
Cloud_Mesh_Distance_BA_R0_R3	21,75113297	-79,33437347	-944,4890137	-0,090605
Cloud_Mesh_Distance_BA_R0_R3	21,76755905	-79,47636414	-944,5559692	-0,100122
Cloud_Mesh_Distance_BA_R0_R3	15,11714649	-78,49918365	-944,5419312	-0,058457
Cloud_Mesh_Distance_BA_R0_R3	15,12003613	-78,43018341	-944,4298706	-0,063993
Cloud_Mesh_Distance_BA_R0_R3	21,35893059	-79,11074066	-944,7570801	-0,082565
Cloud_Mesh_Distance_BA_R0_R3	21,37524033	-78,97532654	-944,6552124	-0,075969
Cloud_Mesh_Distance_BA_R0_R3	21,45153809	-79,04237366	-944,6182251	-0,078944
Cloud_Mesh_Distance_BA_R0_R3	21,65068054	-79,21729279	-944,5219727	-0,085139
Cloud_Mesh_Distance_BA_R0_R3	21,54779053	-79,21399689	-944,6278076	-0,087128
Cloud_Mesh_Distance_BA_R0_R3	15,09722328	-78,45331573	-944,2433472	-0,074636
Cloud_Mesh_Distance_BA_R0_R3	15,17313004	-78,33285522	-944,5830689	-0,053857
Cloud_Mesh_Distance_BA_R0_R3	15,15944386	-78,28992462	-944,3856812	-0,062964
Cloud_Mesh_Distance_BA_R0_R3	21,27231026	-79,00363922	-944,7807617	-0,077435
Cloud_Mesh_Distance_BA_R0_R3	15,15437031	-78,26885986	-944,2042236	-0,077759
Cloud_Mesh_Distance_BA_R0_R3	15,23002911	-78,13741302	-944,5105591	-0,054879
Cloud_Mesh_Distance_BA_R0_R3	15,26569557	-78,00576019	-944,3466187	-0,068815
Cloud_Mesh_Distance_BA_R0_R3	21,03192902	-78,39539337	-944,6425781	-0,049854
Cloud_Mesh_Distance_BA_R0_R3	21,01601219	-78,50243378	-944,732666	-0,058109
Cloud_Mesh_Distance_BA_R0_R3	15,34262562	-77,86481476	-944,4780884	-0,062896

Cloud_Mesh_Distance_BA_R0_R3	15,47335911	-77,58437347	-944,3612061	-0,07264
Cloud_Mesh_Distance_BA_R0_R3	20,94670486	-78,19681549	-944,5993042	-0,042679
Cloud_Mesh_Distance_BA_R0_R3	21,03790474	-78,2871933	-944,5604248	-0,041247
Cloud_Mesh_Distance_BA_R0_R3	15,46625614	-77,63159943	-944,5281982	-0,064811
Cloud_Mesh_Distance_BA_R0_R3	15,53469563	-77,46751404	-944,2991943	-0,072939
Cloud_Mesh_Distance_BA_R0_R3	20,94198227	-78,04755402	-944,4937744	-0,026749
Cloud_Mesh_Distance_BA_R0_R3	20,87133026	-78,1122818	-944,6241455	-0,041732
Cloud_Mesh_Distance_BA_R0_R3	20,78793144	-78,12971497	-944,7322998	-0,055396
Cloud_Mesh_Distance_BA_R0_R3	15,64603043	-77,31511688	-944,4926758	-0,062364
Cloud_Mesh_Distance_BA_R0_R3	20,60178375	-77,77711487	-944,6884766	-0,051356
Cloud_Mesh_Distance_BA_R0_R3	20,80786514	-77,96718597	-944,5897217	-0,038098
Cloud_Mesh_Distance_BA_R0_R3	20,66631699	-77,91893768	-944,7200317	-0,056366
Cloud_Mesh_Distance_BA_R0_R3	15,71117878	-77,20056915	-944,3903809	-0,070614
Cloud_Mesh_Distance_BA_R0_R3	15,7278204	-77,2258606	-944,6158447	-0,052104
Cloud_Mesh_Distance_BA_R0_R3	15,81065273	-77,11174774	-944,6080322	-0,05037
Cloud_Mesh_Distance_BA_R0_R3	20,38593674	-77,56164551	-944,7763672	-0,06709
Cloud_Mesh_Distance_BA_R0_R3	20,65107346	-77,67380524	-944,5426636	-0,039269
Cloud_Mesh_Distance_BA_R0_R3	20,57018661	-77,68028259	-944,6484375	-0,049194
Cloud_Mesh_Distance_BA_R0_R3	20,5526123	-77,590065	-944,5950928	-0,04737
Cloud_Mesh_Distance_BA_R0_R3	15,80957317	-77,07962799	-944,4613037	-0,056715
Cloud_Mesh_Distance_BA_R0_R3	15,88570404	-77,01694489	-944,5982056	-0,051678
Cloud_Mesh_Distance_BA_R0_R3	20,45724487	-77,52361298	-944,6589966	-0,052548
Cloud_Mesh_Distance_BA_R0_R3	20,36198616	-77,36270905	-944,6368408	-0,050611
Cloud_Mesh_Distance_BA_R0_R3	20,36702728	-77,44788361	-944,7061768	-0,056783
Cloud_Mesh_Distance_BA_R0_R3	15,95947647	-76,91434479	-944,5116577	-0,061537
Cloud_Mesh_Distance_BA_R0_R3	16,13006592	-76,79263306	-944,6755371	-0,053737
Cloud_Mesh_Distance_BA_R0_R3	16,06655121	-76,80278778	-944,4910278	-0,063635
Cloud_Mesh_Distance_BA_R0_R3	20,00456238	-77,10442352	-944,8287964	-0,055641
Cloud_Mesh_Distance_BA_R0_R3	19,98134422	-77,01683807	-944,7684326	-0,048194
Cloud_Mesh_Distance_BA_R0_R3	20,1184082	-77,18883514	-944,7784424	-0,057677
Cloud_Mesh_Distance_BA_R0_R3	16,27963448	-76,60106659	-944,3997192	-0,069035
Cloud_Mesh_Distance_BA_R0_R3	16,29088211	-76,65314484	-944,6488648	-0,062896
Cloud_Mesh_Distance_BA_R0_R3	19,92402077	-77,04206085	-944,8545532	-0,053901
Cloud_Mesh_Distance_BA_R0_R3	19,89270973	-76,96582794	-944,8095093	-0,0448
Cloud_Mesh_Distance_BA_R0_R3	20,07645035	-76,95623016	-944,5910645	-0,022152
Cloud_Mesh_Distance_BA_R0_R3	19,98526764	-76,94658661	-944,687439	-0,033567
Cloud_Mesh_Distance_BA_R0_R3	20,08295059	-77,04473877	-944,6823731	-0,036139
Cloud_Mesh_Distance_BA_R0_R3	20,22891998	-77,1264801	-944,5845337	-0,027014
Cloud_Mesh_Distance_BA_R0_R3	20,15159798	-77,00727081	-944,5557861	-0,019605
Cloud_Mesh_Distance_BA_R0_R3	16,31401062	-76,6078949	-944,5540772	-0,062341
Cloud_Mesh_Distance_BA_R0_R3	16,38946915	-76,59017181	-944,6679688	-0,064315
Cloud_Mesh_Distance_BA_R0_R3	16,5587883	-76,50048065	-944,7052002	-0,062255
Cloud_Mesh_Distance_BA_R0_R3	16,54944038	-76,44049835	-944,5085449	-0,055772
Cloud_Mesh_Distance_BA_R0_R3	19,51306534	-76,66649628	-944,7877808	-0,026584
Cloud_Mesh_Distance_BA_R0_R3	19,70928001	-76,67662048	-944,6137695	-0,018281
Cloud_Mesh_Distance_BA_R0_R3	19,60360336	-76,65571594	-944,6864624	-0,020226
Cloud_Mesh_Distance_BA_R0_R3	19,83646011	-76,85810089	-944,7424316	-0,029825
Cloud_Mesh_Distance_BA_R0_R3	16,74791718	-76,33351898	-944,4899292	-0,041627
Cloud_Mesh_Distance_BA_R0_R3	16,98682404	-76,33067322	-944,7454224	-0,060246
Cloud_Mesh_Distance_BA_R0_R3	17,08268356	-76,2530365	-944,6040649	-0,048604

Cloud_Mesh_Distance_BA_R0_R3	19,37313461	-76,44512177	-944,5056152	-0,018317
Cloud_Mesh_Distance_BA_R0_R3	19,49369812	-76,59700012	-944,692627	-0,020197
Cloud_Mesh_Distance_BA_R0_R3	19,51647949	-76,52848053	-944,539856	-0,01993
Cloud_Mesh_Distance_BA_R0_R3	17,43407822	-76,21796417	-944,6823731	-0,0482
Cloud_Mesh_Distance_BA_R0_R3	17,59909058	-76,19462585	-944,6445313	-0,047499
Cloud_Mesh_Distance_BA_R0_R3	17,96228027	-76,21537781	-944,7183838	-0,038006
Cloud_Mesh_Distance_BA_R0_R3	17,76037216	-76,20083618	-944,6795044	-0,045268
Cloud_Mesh_Distance_BA_R0_R3	17,70547867	-76,15164948	-944,4796753	-0,051986
Cloud_Mesh_Distance_BA_R0_R3	17,92407417	-76,16420746	-944,5240479	-0,049884
Cloud_Mesh_Distance_BA_R0_R3	18,35990715	-76,26024628	-944,7536621	-0,016627
Cloud_Mesh_Distance_BA_R0_R3	18,16566467	-76,23665619	-944,7471314	-0,025848
Cloud_Mesh_Distance_BA_R0_R3	18,51023102	-76,23244476	-944,5948486	-0,022859
Cloud_Mesh_Distance_BA_R0_R3	18,53902435	-76,28881073	-944,7547607	-0,007337
Cloud_Mesh_Distance_BA_R0_R3	18,7155571	-76,3259964	-944,7524414	0,003304
Cloud_Mesh_Distance_BA_R0_R3	19,19707108	-76,42524719	-944,6271973	-0,01051
Cloud_Mesh_Distance_BA_R0_R3	19,04491043	-76,35494232	-944,5913696	-0,008678
Cloud_Mesh_Distance_BA_R0_R3	16,417202	-82,65323639	-943,9301148	0,248418
Cloud_Mesh_Distance_BA_R0_R3	16,52371788	-82,71747589	-943,8955689	0,20004
Cloud_Mesh_Distance_BA_R0_R3	16,21512604	-82,46102142	-943,9260254	0,292694
Cloud_Mesh_Distance_BA_R0_R3	15,96642208	-82,14040375	-943,9730835	0,291685
Cloud_Mesh_Distance_BA_R0_R3	16,04207611	-82,21724701	-943,9175415	0,276545
Cloud_Mesh_Distance_BA_R0_R3	16,05581093	-82,3677597	-944,0171509	0,369507
Cloud_Mesh_Distance_BA_R0_R3	16,13814545	-82,40927887	-943,9622803	0,324941
Cloud_Mesh_Distance_BA_R0_R3	22,96905136	-82,79846954	-944,2822876	-0,193929
Cloud_Mesh_Distance_BA_R0_R3	16,00039101	-82,07450104	-943,8533325	0,210049
Cloud_Mesh_Distance_BA_R0_R3	15,89827442	-82,15532684	-944,074585	0,36433
Cloud_Mesh_Distance_BA_R0_R3	15,91373253	-82,002388	-943,9724731	0,24277
Cloud_Mesh_Distance_BA_R0_R3	15,84287834	-82,02451324	-944,1057129	0,326179
Cloud_Mesh_Distance_BA_R0_R3	22,95960617	-82,64868927	-944,1987915	-0,031131
Cloud_Mesh_Distance_BA_R0_R3	23,01760864	-82,7385025	-944,1154175	0,113876
Cloud_Mesh_Distance_BA_R0_R3	22,99371338	-82,57045746	-944,0516357	0,021849
Cloud_Mesh_Distance_BA_R0_R3	15,80763531	-81,72013092	-944,0418091	0,146638
Cloud_Mesh_Distance_BA_R0_R3	15,83444691	-81,87892914	-944,0700684	0,235085
Cloud_Mesh_Distance_BA_R0_R3	22,79030418	-82,19721222	-944,244751	-0,019534
Cloud_Mesh_Distance_BA_R0_R3	15,76856709	-81,563591	-944,0061646	0,06642
Cloud_Mesh_Distance_BA_R0_R3	22,6955204	-82,13413239	-944,3854981	-0,032083
Cloud_Mesh_Distance_BA_R0_R3	15,65107441	-81,27962494	-944,0794067	-0,025616
Cloud_Mesh_Distance_BA_R0_R3	15,71683598	-81,41725922	-943,9469605	-0,000173
Cloud_Mesh_Distance_BA_R0_R3	22,71983528	-81,93817902	-944,2183228	-0,031324
Cloud_Mesh_Distance_BA_R0_R3	15,42668533	-80,88045502	-944,0422363	-0,094453
Cloud_Mesh_Distance_BA_R0_R3	15,45945454	-80,8894577	-944,2354736	-0,099021
Cloud_Mesh_Distance_BA_R0_R3	15,51268768	-81,01200104	-944,1184692	-0,080749
Cloud_Mesh_Distance_BA_R0_R3	22,53862762	-81,55486298	-944,3622437	-0,060861
Cloud_Mesh_Distance_BA_R0_R3	22,58794594	-81,84438324	-944,4069824	-0,04338
Cloud_Mesh_Distance_BA_R0_R3	22,65534782	-81,88471222	-944,3099976	-0,040538
Cloud_Mesh_Distance_BA_R0_R3	22,66726303	-81,777565	-944,2338257	-0,047434
Cloud_Mesh_Distance_BA_R0_R3	15,36310863	-80,78757477	-943,9134522	-0,092968
Cloud_Mesh_Distance_BA_R0_R3	15,35816193	-80,75871277	-944,0598145	-0,103386
Cloud_Mesh_Distance_BA_R0_R3	22,5308075	-81,38275909	-944,3157349	-0,067952
Cloud_Mesh_Distance_BA_R0_R3	15,30852413	-80,67662811	-944,0006714	-0,098429

Cloud_Mesh_Distance_BA_R0_R3	15,20532417	-80,42646027	-944,1516724	-0,113178
Cloud_Mesh_Distance_BA_R0_R3	15,27383709	-80,58487701	-944,1234131	-0,10781
Cloud_Mesh_Distance_BA_R0_R3	15,24271297	-80,54503632	-943,9466553	-0,098632
Cloud_Mesh_Distance_BA_R0_R3	22,5646286	-81,1483078	-944,1925049	-0,087233
Cloud_Mesh_Distance_BA_R0_R3	22,58986282	-81,27767181	-944,184021	-0,079007
Cloud_Mesh_Distance_BA_R0_R3	22,52552223	-81,24025726	-944,2809448	-0,079382
Cloud_Mesh_Distance_BA_R0_R3	15,09037495	-80,08317566	-944,0874023	-0,12761
Cloud_Mesh_Distance_BA_R0_R3	22,36026573	-80,96710968	-944,4442139	-0,091101
Cloud_Mesh_Distance_BA_R0_R3	22,37905121	-80,78818512	-944,3742676	-0,102828
Cloud_Mesh_Distance_BA_R0_R3	22,41158676	-81,09024048	-944,4073486	-0,087373
Cloud_Mesh_Distance_BA_R0_R3	22,47497559	-80,89220428	-944,2678223	-0,100062
Cloud_Mesh_Distance_BA_R0_R3	22,49837494	-81,10883331	-944,2874146	-0,087331
Cloud_Mesh_Distance_BA_R0_R3	15,06217098	-79,95067596	-944,2240601	-0,120085
Cloud_Mesh_Distance_BA_R0_R3	22,38373947	-80,67241669	-944,3374634	-0,11099
Cloud_Mesh_Distance_BA_R0_R3	15,0608139	-79,932724	-944,0106812	-0,139131
Cloud_Mesh_Distance_BA_R0_R3	15,03368855	-79,70844269	-943,9321899	-0,143521
Cloud_Mesh_Distance_BA_R0_R3	15,02955532	-79,77674103	-944,1743164	-0,121997
Cloud_Mesh_Distance_BA_R0_R3	22,25139999	-80,52950287	-944,4592896	-0,113749
Cloud_Mesh_Distance_BA_R0_R3	22,32028198	-80,47427368	-944,3615112	-0,115602
Cloud_Mesh_Distance_BA_R0_R3	22,39318848	-80,56613159	-944,2953491	-0,114864
Cloud_Mesh_Distance_BA_R0_R3	22,44520187	-80,68353271	-944,2576904	-0,109144
Cloud_Mesh_Distance_BA_R0_R3	15,00953579	-79,56034088	-944,0286865	-0,130229
Cloud_Mesh_Distance_BA_R0_R3	14,99511623	-79,47316742	-944,3025513	-0,095354
Cloud_Mesh_Distance_BA_R0_R3	14,99361324	-79,42396545	-944,1045532	-0,118789
Cloud_Mesh_Distance_BA_R0_R3	15,00704098	-79,59986877	-944,1488648	-0,120544
Cloud_Mesh_Distance_BA_R0_R3	22,23170853	-80,39418793	-944,4419556	-0,118996
Cloud_Mesh_Distance_BA_R0_R3	14,98591328	-79,29470062	-944,274353	-0,089075
Cloud_Mesh_Distance_BA_R0_R3	22,06691933	-80,20212555	-944,5605469	-0,118777
Cloud_Mesh_Distance_BA_R0_R3	22,19473648	-80,20223236	-944,420105	-0,122024
Cloud_Mesh_Distance_BA_R0_R3	22,09312439	-80,0348587	-944,4702148	-0,116507
Cloud_Mesh_Distance_BA_R0_R3	21,98478127	-79,89270782	-944,5302124	-0,112357
Cloud_Mesh_Distance_BA_R0_R3	22,2140522	-79,85897064	-944,2474365	-0,126139
Cloud_Mesh_Distance_BA_R0_R3	22,20706558	-80,00322723	-944,3249512	-0,125997
Cloud_Mesh_Distance_BA_R0_R3	14,99905682	-78,92856598	-944,1948242	-0,086321
Cloud_Mesh_Distance_BA_R0_R3	14,98605633	-79,11659241	-944,2363281	-0,08903
Cloud_Mesh_Distance_BA_R0_R3	22,1063385	-79,84038544	-944,3690186	-0,121007
Cloud_Mesh_Distance_BA_R0_R3	22,16317749	-79,72570038	-944,2387695	-0,120343
Cloud_Mesh_Distance_BA_R0_R3	22,08613014	-79,70334625	-944,3218384	-0,116098
Cloud_Mesh_Distance_BA_R0_R3	14,99732399	-78,95957184	-944,0783081	-0,092698
Cloud_Mesh_Distance_BA_R0_R3	21,920578	-79,51752472	-944,4110718	-0,102064
Cloud_Mesh_Distance_BA_R0_R3	22,09236336	-79,57781219	-944,2393799	-0,109271
Cloud_Mesh_Distance_BA_R0_R3	15,02602291	-78,7551651	-944,2550049	-0,075453
Cloud_Mesh_Distance_BA_R0_R3	15,04798698	-78,63639069	-944,2014771	-0,075064
Cloud_Mesh_Distance_BA_R0_R3	21,74940681	-79,22373199	-944,4178467	-0,085861
Cloud_Mesh_Distance_BA_R0_R3	21,85675621	-79,41643524	-944,4227295	-0,097039
Cloud_Mesh_Distance_BA_R0_R3	21,92578125	-79,44034576	-944,3573608	-0,098871
Cloud_Mesh_Distance_BA_R0_R3	21,54897308	-79,10305023	-944,5557251	-0,081395
Cloud_Mesh_Distance_BA_R0_R3	21,43798828	-78,9176712	-944,5495606	-0,074366
Cloud_Mesh_Distance_BA_R0_R3	21,47389412	-78,83332062	-944,4506226	-0,065403
Cloud_Mesh_Distance_BA_R0_R3	21,53288651	-78,93793488	-944,4597778	-0,074546

Cloud_Mesh_Distance_BA_R0_R3	21,60841179	-78,86423492	-944,3189087	-0,061277
Cloud_Mesh_Distance_BA_R0_R3	15,20181084	-78,15749359	-944,3270874	-0,067317
Cloud_Mesh_Distance_BA_R0_R3	15,20910072	-78,12187958	-944,1775513	-0,085701
Cloud_Mesh_Distance_BA_R0_R3	21,27706909	-78,64424896	-944,5396118	-0,054057
Cloud_Mesh_Distance_BA_R0_R3	21,25765991	-78,50862122	-944,4663696	-0,046063
Cloud_Mesh_Distance_BA_R0_R3	21,35123444	-78,80547333	-944,5684204	-0,065913
Cloud_Mesh_Distance_BA_R0_R3	21,59518051	-78,7676239	-944,2609863	-0,05353
Cloud_Mesh_Distance_BA_R0_R3	21,54296684	-78,81809998	-944,361145	-0,062081
Cloud_Mesh_Distance_BA_R0_R3	15,33670998	-77,84118652	-944,2748413	-0,080022
Cloud_Mesh_Distance_BA_R0_R3	21,19530106	-78,5916214	-944,5949097	-0,055442
Cloud_Mesh_Distance_BA_R0_R3	21,31143951	-78,41165924	-944,3302002	-0,029098
Cloud_Mesh_Distance_BA_R0_R3	15,4053688	-77,72220612	-944,4141846	-0,068649
Cloud_Mesh_Distance_BA_R0_R3	21,14121246	-78,19204712	-944,3688355	-0,015542
Cloud_Mesh_Distance_BA_R0_R3	21,14129257	-78,42566681	-944,5415649	-0,041758
Cloud_Mesh_Distance_BA_R0_R3	21,23733711	-78,31574249	-944,3474731	-0,020188
Cloud_Mesh_Distance_BA_R0_R3	21,23850441	-78,41391754	-944,4192505	-0,033684
Cloud_Mesh_Distance_BA_R0_R3	15,55736732	-77,42090607	-944,1283569	-0,086841
Cloud_Mesh_Distance_BA_R0_R3	20,97643089	-77,95186615	-944,3753052	-0,013991
Cloud_Mesh_Distance_BA_R0_R3	21,02505684	-78,15177155	-944,4762573	-0,025789
Cloud_Mesh_Distance_BA_R0_R3	21,04569817	-78,03916168	-944,3623657	-0,010695
Cloud_Mesh_Distance_BA_R0_R3	20,71887207	-77,80391693	-944,5667114	-0,03762
Cloud_Mesh_Distance_BA_R0_R3	15,61693859	-77,33243561	-944,3102417	-0,072233
Cloud_Mesh_Distance_BA_R0_R3	15,69494438	-77,20737457	-944,2247925	-0,076858
Cloud_Mesh_Distance_BA_R0_R3	15,79289055	-77,0750351	-944,2260132	-0,07953
Cloud_Mesh_Distance_BA_R0_R3	20,52293015	-77,46656036	-944,5231934	-0,033329
Cloud_Mesh_Distance_BA_R0_R3	20,63234138	-77,52422333	-944,4298706	-0,025713
Cloud_Mesh_Distance_BA_R0_R3	20,73792648	-77,72064972	-944,4725342	-0,02863
Cloud_Mesh_Distance_BA_R0_R3	15,870924	-76,98740387	-944,3339844	-0,069999
Cloud_Mesh_Distance_BA_R0_R3	15,89580822	-76,97376251	-944,4511108	-0,059683
Cloud_Mesh_Distance_BA_R0_R3	15,96416092	-76,88475037	-944,3498535	-0,0697
Cloud_Mesh_Distance_BA_R0_R3	20,43297768	-77,40298462	-944,5828857	-0,041019
Cloud_Mesh_Distance_BA_R0_R3	16,07593918	-76,76604462	-944,2905273	-0,07662
Cloud_Mesh_Distance_BA_R0_R3	16,19624138	-76,65006256	-944,2193604	-0,07128
Cloud_Mesh_Distance_BA_R0_R3	16,16178131	-76,69824982	-944,3908691	-0,072686
Cloud_Mesh_Distance_BA_R0_R3	20,39065552	-77,26535797	-944,5073853	-0,030959
Cloud_Mesh_Distance_BA_R0_R3	20,48911285	-77,36607361	-944,4721069	-0,026808
Cloud_Mesh_Distance_BA_R0_R3	20,52576256	-77,30080414	-944,3473511	-0,018549
Cloud_Mesh_Distance_BA_R0_R3	16,30897141	-76,55789185	-944,2438965	-0,067566
Cloud_Mesh_Distance_BA_R0_R3	16,37957764	-76,51457214	-944,3319092	-0,065139
Cloud_Mesh_Distance_BA_R0_R3	20,22185898	-76,99146271	-944,4382935	-0,01164
Cloud_Mesh_Distance_BA_R0_R3	16,41872025	-76,52092743	-944,5022583	-0,06387
Cloud_Mesh_Distance_BA_R0_R3	16,49978828	-76,43399811	-944,3391113	-0,05676
Cloud_Mesh_Distance_BA_R0_R3	19,85266685	-76,74289703	-944,5636597	-0,017671
Cloud_Mesh_Distance_BA_R0_R3	20,1267395	-76,93654633	-944,5005493	-0,0125
Cloud_Mesh_Distance_BA_R0_R3	20,1489048	-76,87944031	-944,3863525	-0,002119
Cloud_Mesh_Distance_BA_R0_R3	20,01386261	-76,86373138	-944,5483398	-0,010229
Cloud_Mesh_Distance_BA_R0_R3	16,63458252	-76,36981964	-944,4136963	-0,047275
Cloud_Mesh_Distance_BA_R0_R3	16,92205429	-76,23347473	-944,3475342	-0,035183
Cloud_Mesh_Distance_BA_R0_R3	16,77217674	-76,28066254	-944,2894287	-0,03716
Cloud_Mesh_Distance_BA_R0_R3	16,89998055	-76,27950287	-944,510437	-0,043405

Cloud_Mesh_Distance_BA_R0_R3	17,18095398	-76,20189667	-944,4995728	-0,044239
Cloud_Mesh_Distance_BA_R0_R3	19,40232086	-76,4033432	-944,3696899	-0,016313
Cloud_Mesh_Distance_BA_R0_R3	19,54412842	-76,46071625	-944,3549194	-0,015472
Cloud_Mesh_Distance_BA_R0_R3	19,63841248	-76,60886383	-944,5698242	-0,017391
Cloud_Mesh_Distance_BA_R0_R3	19,71294212	-76,56000519	-944,3858032	-0,013056
Cloud_Mesh_Distance_BA_R0_R3	19,78247261	-76,64879608	-944,4838257	-0,012493
Cloud_Mesh_Distance_BA_R0_R3	17,02444458	-76,18842316	-944,2648926	-0,028013
Cloud_Mesh_Distance_BA_R0_R3	17,04437828	-76,21477509	-944,4280396	-0,039248
Cloud_Mesh_Distance_BA_R0_R3	17,20621681	-76,1588974	-944,3204956	-0,034177
Cloud_Mesh_Distance_BA_R0_R3	17,35921478	-76,17167664	-944,4842529	-0,046241
Cloud_Mesh_Distance_BA_R0_R3	17,5276947	-76,16999817	-944,5365601	-0,046559
Cloud_Mesh_Distance_BA_R0_R3	17,67700768	-76,13053131	-944,3760986	-0,057451
Cloud_Mesh_Distance_BA_R0_R3	18,13461685	-76,18399811	-944,5609741	-0,041861
Cloud_Mesh_Distance_BA_R0_R3	17,87748718	-76,11960602	-944,3161621	-0,058665
Cloud_Mesh_Distance_BA_R0_R3	18,33031845	-76,20684052	-944,5835571	-0,032134
Cloud_Mesh_Distance_BA_R0_R3	18,30816269	-76,16257477	-944,4100342	-0,049884
Cloud_Mesh_Distance_BA_R0_R3	18,68512344	-76,26082611	-944,5898438	-0,015839
Cloud_Mesh_Distance_BA_R0_R3	18,63734436	-76,21170807	-944,4532471	-0,029661
Cloud_Mesh_Distance_BA_R0_R3	18,86605072	-76,29933929	-944,5834961	-0,007552
Cloud_Mesh_Distance_BA_R0_R3	18,81856155	-76,22782135	-944,3937988	-0,024236
Cloud_Mesh_Distance_BA_R0_R3	16,88961029	-82,97081757	-943,7339478	0,058348
Cloud_Mesh_Distance_BA_R0_R3	16,65392303	-82,74478912	-943,7168579	0,098913
Cloud_Mesh_Distance_BA_R0_R3	16,64099121	-82,82062531	-943,9056397	0,17101
Cloud_Mesh_Distance_BA_R0_R3	16,7769165	-82,87810516	-943,7686157	0,089486
Cloud_Mesh_Distance_BA_R0_R3	16,3571701	-82,54894257	-943,8598633	0,227049
Cloud_Mesh_Distance_BA_R0_R3	16,46472168	-82,62924957	-943,831665	0,187633
Cloud_Mesh_Distance_BA_R0_R3	16,54325867	-82,65810394	-943,7514038	0,136333
Cloud_Mesh_Distance_BA_R0_R3	16,1489296	-82,32212067	-943,8602295	0,251591
Cloud_Mesh_Distance_BA_R0_R3	16,20693588	-82,25991058	-943,6586304	0,149327
Cloud_Mesh_Distance_BA_R0_R3	15,95261574	-81,91584015	-943,7866821	0,136285
Cloud_Mesh_Distance_BA_R0_R3	23,0747261	-82,83039093	-944,0199585	0,171694
Cloud_Mesh_Distance_BA_R0_R3	23,02766228	-82,50873566	-943,9036255	0,040609
Cloud_Mesh_Distance_BA_R0_R3	23,06316757	-82,66233063	-943,9252319	0,040856
Cloud_Mesh_Distance_BA_R0_R3	15,87430573	-81,82874298	-943,9237671	0,155337
Cloud_Mesh_Distance_BA_R0_R3	15,83242035	-81,68093109	-943,8806763	0,083434
Cloud_Mesh_Distance_BA_R0_R3	22,85678482	-82,2926712	-944,1712036	-0,003027
Cloud_Mesh_Distance_BA_R0_R3	22,90897751	-82,4699173	-944,1850586	-0,002342
Cloud_Mesh_Distance_BA_R0_R3	15,78415012	-81,54158783	-943,8201904	0,024044
Cloud_Mesh_Distance_BA_R0_R3	22,84930611	-82,17398834	-944,0993652	-0,003191
Cloud_Mesh_Distance_BA_R0_R3	22,87412453	-82,11061859	-943,9934692	0,003134
Cloud_Mesh_Distance_BA_R0_R3	15,58137321	-81,14933777	-944,005249	-0,058472
Cloud_Mesh_Distance_BA_R0_R3	15,65299225	-81,28281403	-943,8771362	-0,041109
Cloud_Mesh_Distance_BA_R0_R3	22,75484085	-82,07151031	-944,2318726	-0,025005
Cloud_Mesh_Distance_BA_R0_R3	22,80963326	-82,0280838	-944,0864258	-0,017971
Cloud_Mesh_Distance_BA_R0_R3	15,50574207	-81,02733612	-943,9165649	-0,082373
Cloud_Mesh_Distance_BA_R0_R3	22,60509109	-81,65592194	-944,2904663	-0,056694
Cloud_Mesh_Distance_BA_R0_R3	15,30341053	-80,67949677	-943,8283691	-0,090191
Cloud_Mesh_Distance_BA_R0_R3	15,36569023	-80,79534149	-943,7198486	-0,084166
Cloud_Mesh_Distance_BA_R0_R3	22,67865372	-81,60694122	-944,1324463	-0,057124
Cloud_Mesh_Distance_BA_R0_R3	22,61398315	-81,46320343	-944,2000732	-0,066401

Cloud_Mesh_Distance_BA_R0_R3	22,74426842	-81,54769135	-943,9698486	-0,05761
Cloud_Mesh_Distance_BA_R0_R3	22,68684387	-81,46073151	-944,059082	-0,062443
Cloud_Mesh_Distance_BA_R0_R3	22,73110962	-81,3789444	-943,9381714	-0,074099
Cloud_Mesh_Distance_BA_R0_R3	15,15603161	-80,31478119	-943,8908081	-0,118782
Cloud_Mesh_Distance_BA_R0_R3	15,13794613	-80,25469208	-944,0733643	-0,122894
Cloud_Mesh_Distance_BA_R0_R3	15,18474007	-80,40021515	-943,970459	-0,107753
Cloud_Mesh_Distance_BA_R0_R3	15,25498486	-80,57407379	-943,7562866	-0,094122
Cloud_Mesh_Distance_BA_R0_R3	15,16005611	-80,31072235	-943,7628174	-0,113365
Cloud_Mesh_Distance_BA_R0_R3	22,56762886	-80,99970245	-944,1501465	-0,095815
Cloud_Mesh_Distance_BA_R0_R3	22,5710144	-80,83937836	-944,1063232	-0,106551
Cloud_Mesh_Distance_BA_R0_R3	15,05955505	-79,7760849	-943,7763672	-0,153602
Cloud_Mesh_Distance_BA_R0_R3	22,40195847	-80,40213776	-944,2337036	-0,120106
Cloud_Mesh_Distance_BA_R0_R3	22,47840309	-80,51741791	-944,1624756	-0,116227
Cloud_Mesh_Distance_BA_R0_R3	15,01470757	-79,50211334	-943,9191895	-0,137548
Cloud_Mesh_Distance_BA_R0_R3	15,05147457	-79,70146942	-943,7745361	-0,158269
Cloud_Mesh_Distance_BA_R0_R3	15,04226208	-79,59799194	-943,769043	-0,156303
Cloud_Mesh_Distance_BA_R0_R3	22,3125	-80,30469513	-944,315918	-0,123252
Cloud_Mesh_Distance_BA_R0_R3	22,48592567	-80,32796478	-944,0889893	-0,126277
Cloud_Mesh_Distance_BA_R0_R3	14,98528385	-79,23904419	-944,1187134	-0,103007
Cloud_Mesh_Distance_BA_R0_R3	22,30439758	-80,13096619	-944,2595215	-0,127642
Cloud_Mesh_Distance_BA_R0_R3	22,3955822	-80,23271942	-944,1810913	-0,130281
Cloud_Mesh_Distance_BA_R0_R3	22,46424866	-80,14696503	-944,0476685	-0,133845
Cloud_Mesh_Distance_BA_R0_R3	15,00091362	-79,28670502	-943,9296265	-0,125221
Cloud_Mesh_Distance_BA_R0_R3	14,9915657	-79,06851196	-944,0463257	-0,099761
Cloud_Mesh_Distance_BA_R0_R3	22,31225014	-79,95734406	-944,1703491	-0,132143
Cloud_Mesh_Distance_BA_R0_R3	22,41689682	-79,99297333	-944,0429077	-0,136653
Cloud_Mesh_Distance_BA_R0_R3	22,38139343	-80,09166718	-944,1411743	-0,131393
Cloud_Mesh_Distance_BA_R0_R3	14,99961281	-78,96231079	-944,0261231	-0,097282
Cloud_Mesh_Distance_BA_R0_R3	22,27552032	-79,76883698	-944,1151733	-0,124238
Cloud_Mesh_Distance_BA_R0_R3	15,00047779	-78,93561554	-944,0544434	-0,093916
Cloud_Mesh_Distance_BA_R0_R3	15,00459194	-78,91094208	-944,0325317	-0,095928
Cloud_Mesh_Distance_BA_R0_R3	15,00199223	-78,90789032	-944,0847168	-0,090999
Cloud_Mesh_Distance_BA_R0_R3	15,044137	-78,7378006	-943,8745728	-0,106833
Cloud_Mesh_Distance_BA_R0_R3	15,01723194	-78,799263	-944,0757446	-0,090171
Cloud_Mesh_Distance_BA_R0_R3	22,0702858	-79,37610626	-944,1329956	-0,102119
Cloud_Mesh_Distance_BA_R0_R3	22,14817619	-79,48239899	-944,1032715	-0,112167
Cloud_Mesh_Distance_BA_R0_R3	15,06037235	-78,5888443	-944,0496826	-0,088779
Cloud_Mesh_Distance_BA_R0_R3	21,98927689	-79,48642731	-944,3111572	-0,100842
Cloud_Mesh_Distance_BA_R0_R3	21,94389153	-79,34061432	-944,2709961	-0,095101
Cloud_Mesh_Distance_BA_R0_R3	15,11289978	-78,3976059	-944,0447998	-0,089767
Cloud_Mesh_Distance_BA_R0_R3	21,65496826	-78,99893951	-944,3665772	-0,075177
Cloud_Mesh_Distance_BA_R0_R3	21,75767326	-79,11101532	-944,329895	-0,083575
Cloud_Mesh_Distance_BA_R0_R3	21,83898926	-79,23499298	-944,3234253	-0,087413
Cloud_Mesh_Distance_BA_R0_R3	15,21208286	-78,12017059	-944,0344849	-0,085883
Cloud_Mesh_Distance_BA_R0_R3	15,16756153	-78,23469543	-944,0428467	-0,089152
Cloud_Mesh_Distance_BA_R0_R3	21,7625885	-78,96696472	-944,215271	-0,062465
Cloud_Mesh_Distance_BA_R0_R3	21,70729828	-78,80725861	-944,1555786	-0,050766
Cloud_Mesh_Distance_BA_R0_R3	15,23276329	-78,09119415	-943,9169312	-0,092427
Cloud_Mesh_Distance_BA_R0_R3	15,29328918	-77,95709229	-943,9017334	-0,096951
Cloud_Mesh_Distance_BA_R0_R3	15,26702499	-77,98656464	-944,1042481	-0,084538

Cloud_Mesh_Distance_BA_R0_R3	21,49752235	-78,69507599	-944,3220215	-0,052026
Cloud_Mesh_Distance_BA_R0_R3	21,61123276	-78,66210938	-944,1558228	-0,040483
Cloud_Mesh_Distance_BA_R0_R3	15,34522724	-77,82175446	-944,0686035	-0,089642
Cloud_Mesh_Distance_BA_R0_R3	21,52260971	-78,45513916	-944,093811	-0,018296
Cloud_Mesh_Distance_BA_R0_R3	21,37131691	-78,57329559	-944,3798218	-0,046488
Cloud_Mesh_Distance_BA_R0_R3	21,54277229	-78,57756805	-944,1726685	-0,031453
Cloud_Mesh_Distance_BA_R0_R3	21,47179031	-78,5424118	-944,2333984	-0,032071
Cloud_Mesh_Distance_BA_R0_R3	15,40831852	-77,69394684	-944,2212524	-0,084979
Cloud_Mesh_Distance_BA_R0_R3	15,47952557	-77,55826569	-944,1702271	-0,088241
Cloud_Mesh_Distance_BA_R0_R3	20,90442276	-77,9203186	-944,4359131	-0,020437
Cloud_Mesh_Distance_BA_R0_R3	20,94417763	-77,86598969	-944,3406372	-0,013195
Cloud_Mesh_Distance_BA_R0_R3	21,03414536	-77,89490509	-944,2515869	-0,001609
Cloud_Mesh_Distance_BA_R0_R3	21,23939133	-78,21527863	-944,263855	-0,009476
Cloud_Mesh_Distance_BA_R0_R3	15,67136669	-77,24297333	-943,975647	-0,090938
Cloud_Mesh_Distance_BA_R0_R3	15,64049053	-77,2857132	-944,1054688	-0,086431
Cloud_Mesh_Distance_BA_R0_R3	20,84184837	-77,77606964	-944,3894043	-0,020439
Cloud_Mesh_Distance_BA_R0_R3	20,95519447	-77,79605103	-944,2609253	-0,001753
Cloud_Mesh_Distance_BA_R0_R3	15,80928421	-77,05238342	-944,0100098	-0,091276
Cloud_Mesh_Distance_BA_R0_R3	15,7218771	-77,16608429	-944,0787964	-0,088099
Cloud_Mesh_Distance_BA_R0_R3	15,84745312	-77,00448608	-944,1148071	-0,088191
Cloud_Mesh_Distance_BA_R0_R3	20,73721123	-77,63593292	-944,3953247	-0,024665
Cloud_Mesh_Distance_BA_R0_R3	20,82285881	-77,61104584	-944,2514648	-0,005513
Cloud_Mesh_Distance_BA_R0_R3	15,97774982	-76,85817719	-944,0148926	-0,081793
Cloud_Mesh_Distance_BA_R0_R3	15,99938679	-76,83635712	-944,1873169	-0,082393
Cloud_Mesh_Distance_BA_R0_R3	15,90333843	-76,94042206	-944,1814575	-0,084482
Cloud_Mesh_Distance_BA_R0_R3	20,73043442	-77,52474213	-944,2910156	-0,013581
Cloud_Mesh_Distance_BA_R0_R3	20,79868317	-77,47480011	-944,125061	-0,00163
Cloud_Mesh_Distance_BA_R0_R3	20,45875549	-77,17063141	-944,2952271	-0,008751
Cloud_Mesh_Distance_BA_R0_R3	20,34024048	-77,08747101	-944,3804932	-0,007307
Cloud_Mesh_Distance_BA_R0_R3	20,56602478	-77,40740204	-944,4042358	-0,022126
Cloud_Mesh_Distance_BA_R0_R3	16,29444885	-76,5616684	-944,0943604	-0,070502
Cloud_Mesh_Distance_BA_R0_R3	16,40988159	-76,47615814	-944,1708374	-0,063417
Cloud_Mesh_Distance_BA_R0_R3	20,43454742	-77,01706696	-944,1036377	0,000165
Cloud_Mesh_Distance_BA_R0_R3	20,40723038	-77,06198883	-944,2324219	-0,002203
Cloud_Mesh_Distance_BA_R0_R3	16,53199005	-76,39406586	-944,1760864	-0,052253
Cloud_Mesh_Distance_BA_R0_R3	19,90285873	-76,69015503	-944,4115601	-0,007116
Cloud_Mesh_Distance_BA_R0_R3	20,02638245	-76,76488495	-944,3776856	0,002549
Cloud_Mesh_Distance_BA_R0_R3	20,16805267	-76,8081131	-944,2315674	0,008302
Cloud_Mesh_Distance_BA_R0_R3	16,65716934	-76,31690979	-944,0980835	-0,038219
Cloud_Mesh_Distance_BA_R0_R3	16,61318016	-76,35538483	-944,2649536	-0,048365
Cloud_Mesh_Distance_BA_R0_R3	16,97827721	-76,18712616	-944,1531982	-0,023839
Cloud_Mesh_Distance_BA_R0_R3	19,47211647	-76,3928833	-944,256958	-0,017307
Cloud_Mesh_Distance_BA_R0_R3	19,37212753	-76,36756134	-944,3028565	-0,019059
Cloud_Mesh_Distance_BA_R0_R3	19,59077454	-76,42984009	-944,2088623	-0,017166
Cloud_Mesh_Distance_BA_R0_R3	19,85820007	-76,6302948	-944,3505249	-0,006071
Cloud_Mesh_Distance_BA_R0_R3	19,97503662	-76,67604828	-944,2810059	-0,002221
Cloud_Mesh_Distance_BA_R0_R3	16,90684891	-76,21981812	-944,2358398	-0,033256
Cloud_Mesh_Distance_BA_R0_R3	17,56875229	-76,13083649	-944,3635864	-0,052208
Cloud_Mesh_Distance_BA_R0_R3	17,39550781	-76,13141632	-944,307251	-0,043313
Cloud_Mesh_Distance_BA_R0_R3	17,35855293	-76,11188507	-944,1474609	-0,035171

Cloud_Mesh_Distance_BA_R0_R3	17,51818848	-76,10533905	-944,2029419	-0,04597
Cloud_Mesh_Distance_BA_R0_R3	17,72601318	-76,09514618	-944,177124	-0,053712
Cloud_Mesh_Distance_BA_R0_R3	18,11249352	-76,14009857	-944,37146	-0,057556
Cloud_Mesh_Distance_BA_R0_R3	18,48849869	-76,18564606	-944,4303589	-0,039312
Cloud_Mesh_Distance_BA_R0_R3	18,65839005	-76,16623688	-944,2573853	-0,034123
Cloud_Mesh_Distance_BA_R0_R3	19,02198792	-76,28264618	-944,4111328	-0,014477
Cloud_Mesh_Distance_BA_R0_R3	19,15272141	-76,26721954	-944,2333984	-0,016439
Cloud_Mesh_Distance_BA_R0_R3	19,24426079	-76,35378265	-944,4058228	-0,016982
Cloud_Mesh_Distance_BA_R0_R3	19,36499786	-76,32060242	-944,1596069	-0,027328
Cloud_Mesh_Distance_BA_R0_R3	17,68123245	-76,11925507	-944,3155518	-0,056121
Cloud_Mesh_Distance_BA_R0_R3	17,73911667	-76,12818146	-944,3659668	-0,060306
Cloud_Mesh_Distance_BA_R0_R3	17,70713806	-76,12395477	-944,34375	-0,05873
Cloud_Mesh_Distance_BA_R0_R3	17,74333954	-76,1166153	-944,305542	-0,05872
Cloud_Mesh_Distance_BA_R0_R3	18,84195137	-76,18541718	-944,2138672	-0,028096
Cloud_Mesh_Distance_BA_R0_R3	19,07371902	-76,21364594	-944,1125488	-0,024739
Cloud_Mesh_Distance_BA_R0_R3	18,97903442	-76,22142792	-944,2410889	-0,020029
Cloud_Mesh_Distance_BA_R0_R3	17,21116066	-83,24727631	-943,6077271	-0,05568
Cloud_Mesh_Distance_BA_R0_R3	17,10250092	-83,1266098	-943,4725952	-0,025138
Cloud_Mesh_Distance_BA_R0_R3	17,09289742	-83,14481354	-943,6618652	0,083296
Cloud_Mesh_Distance_BA_R0_R3	16,8877964	-82,92472076	-943,5698242	0,016964
Cloud_Mesh_Distance_BA_R0_R3	16,95615768	-83,04994965	-943,7953491	0,1242
Cloud_Mesh_Distance_BA_R0_R3	16,97075653	-83,02472687	-943,6464844	0,023003
Cloud_Mesh_Distance_BA_R0_R3	16,5875206	-82,63690948	-943,5968018	0,07546
Cloud_Mesh_Distance_BA_R0_R3	16,42615891	-82,41718292	-943,4252319	0,060797
Cloud_Mesh_Distance_BA_R0_R3	16,39640808	-82,46460724	-943,6378784	0,125917
Cloud_Mesh_Distance_BA_R0_R3	16,47875214	-82,57498932	-943,7022095	0,132521
Cloud_Mesh_Distance_BA_R0_R3	16,09639931	-82,15085602	-943,7370606	0,173151
Cloud_Mesh_Distance_BA_R0_R3	16,27518272	-82,39705658	-943,753418	0,188245
Cloud_Mesh_Distance_BA_R0_R3	23,16513062	-83,00138092	-943,8543701	0,023327
Cloud_Mesh_Distance_BA_R0_R3	23,18703842	-82,94509125	-943,7380981	0,0403
Cloud_Mesh_Distance_BA_R0_R3	16,02681732	-82,01836395	-943,7150269	0,141687
Cloud_Mesh_Distance_BA_R0_R3	16,07937622	-82,02439117	-943,557312	0,102683
Cloud_Mesh_Distance_BA_R0_R3	23,13195419	-82,82514191	-943,8338013	0,042469
Cloud_Mesh_Distance_BA_R0_R3	23,10565948	-82,66582489	-943,7894898	0,057617
Cloud_Mesh_Distance_BA_R0_R3	15,9365654	-81,7907486	-943,6109009	0,066956
Cloud_Mesh_Distance_BA_R0_R3	15,89161777	-81,77465057	-943,7822266	0,092244
Cloud_Mesh_Distance_BA_R0_R3	16,01589394	-81,88417816	-943,4746704	0,063328
Cloud_Mesh_Distance_BA_R0_R3	16,00265884	-81,92656708	-943,6297607	0,101561
Cloud_Mesh_Distance_BA_R0_R3	22,97665215	-82,40830231	-943,9655151	0,030685
Cloud_Mesh_Distance_BA_R0_R3	23,02231979	-82,40372467	-943,8271484	0,047016
Cloud_Mesh_Distance_BA_R0_R3	15,85393333	-81,65746307	-943,704834	0,045592
Cloud_Mesh_Distance_BA_R0_R3	15,73245811	-81,38686371	-943,5471802	-0,027748
Cloud_Mesh_Distance_BA_R0_R3	15,72469234	-81,40581512	-943,7493897	-0,016319
Cloud_Mesh_Distance_BA_R0_R3	22,87088394	-82,00611115	-943,9249268	-0,004915
Cloud_Mesh_Distance_BA_R0_R3	22,91223717	-82,25655365	-944,0104981	0,011406
Cloud_Mesh_Distance_BA_R0_R3	22,98626328	-82,25775909	-943,8061523	0,042355
Cloud_Mesh_Distance_BA_R0_R3	15,65743542	-81,27584839	-943,6723023	-0,044059
Cloud_Mesh_Distance_BA_R0_R3	22,77862549	-81,89133453	-944,0680542	-0,023331
Cloud_Mesh_Distance_BA_R0_R3	22,83183861	-81,8600235	-943,9239502	-0,021608
Cloud_Mesh_Distance_BA_R0_R3	22,92497063	-82,10417938	-943,8542481	0,016065

Cloud_Mesh_Distance_BA_R0_R3	15,4319458	-80,91066742	-943,8187256	-0,083741
Cloud_Mesh_Distance_BA_R0_R3	15,50855923	-81,03421021	-943,7051392	-0,067853
Cloud_Mesh_Distance_BA_R0_R3	15,58176708	-81,15642548	-943,7957153	-0,06302
Cloud_Mesh_Distance_BA_R0_R3	22,74887276	-81,75101471	-944,0548706	-0,039333
Cloud_Mesh_Distance_BA_R0_R3	15,372612	-80,78102875	-943,5134277	-0,06383
Cloud_Mesh_Distance_BA_R0_R3	15,30429363	-80,66886139	-943,6367188	-0,078642
Cloud_Mesh_Distance_BA_R0_R3	15,43813133	-80,91118622	-943,607605	-0,0702
Cloud_Mesh_Distance_BA_R0_R3	22,66314697	-81,3183136	-944,0601196	-0,078103
Cloud_Mesh_Distance_BA_R0_R3	15,20032978	-80,43886566	-943,7926636	-0,107339
Cloud_Mesh_Distance_BA_R0_R3	22,63842773	-81,12567902	-944,0552979	-0,091166
Cloud_Mesh_Distance_BA_R0_R3	22,71507263	-81,03762054	-943,8850708	-0,09967
Cloud_Mesh_Distance_BA_R0_R3	15,15533352	-80,24903107	-943,6334229	-0,110831
Cloud_Mesh_Distance_BA_R0_R3	15,11250496	-80,14636993	-943,8566284	-0,128921
Cloud_Mesh_Distance_BA_R0_R3	15,18913078	-80,37911224	-943,6500855	-0,097814
Cloud_Mesh_Distance_BA_R0_R3	15,07656384	-79,91029358	-943,7766113	-0,143843
Cloud_Mesh_Distance_BA_R0_R3	22,54227448	-80,67667389	-944,1102295	-0,115763
Cloud_Mesh_Distance_BA_R0_R3	22,61611366	-80,61681366	-943,9736328	-0,120087
Cloud_Mesh_Distance_BA_R0_R3	22,68211937	-80,69061279	-943,8740845	-0,119547
Cloud_Mesh_Distance_BA_R0_R3	22,61332321	-80,74202728	-944,0100098	-0,113888
Cloud_Mesh_Distance_BA_R0_R3	22,64689255	-80,8970871	-943,9866333	-0,106717
Cloud_Mesh_Distance_BA_R0_R3	15,08896542	-79,78565216	-943,5841675	-0,153801
Cloud_Mesh_Distance_BA_R0_R3	15,07465458	-79,74552155	-943,6477661	-0,155176
Cloud_Mesh_Distance_BA_R0_R3	15,06470013	-79,74306488	-943,711853	-0,156376
Cloud_Mesh_Distance_BA_R0_R3	15,07706165	-79,81150055	-943,6779785	-0,154636
Cloud_Mesh_Distance_BA_R0_R3	22,57677841	-80,46079254	-943,9931641	-0,125167
Cloud_Mesh_Distance_BA_R0_R3	15,03363514	-79,42588806	-943,7446289	-0,151916
Cloud_Mesh_Distance_BA_R0_R3	15,03330612	-79,2256546	-943,7085571	-0,142377
Cloud_Mesh_Distance_BA_R0_R3	22,39962769	-79,79109955	-943,9505615	-0,131814
Cloud_Mesh_Distance_BA_R0_R3	22,36792374	-79,85759735	-944,0383301	-0,131849
Cloud_Mesh_Distance_BA_R0_R3	22,44873047	-79,88205719	-943,9321289	-0,136275
Cloud_Mesh_Distance_BA_R0_R3	22,51197433	-80,02399445	-943,9136353	-0,135659
Cloud_Mesh_Distance_BA_R0_R3	15,00728512	-79,11472321	-943,8702393	-0,122289
Cloud_Mesh_Distance_BA_R0_R3	15,0281477	-79,05063629	-943,7532959	-0,128026
Cloud_Mesh_Distance_BA_R0_R3	15,01106644	-78,94163513	-943,9221191	-0,11018
Cloud_Mesh_Distance_BA_R0_R3	22,34727097	-79,75134277	-944,0028076	-0,129104
Cloud_Mesh_Distance_BA_R0_R3	22,20355797	-79,60503387	-944,1104736	-0,118341
Cloud_Mesh_Distance_BA_R0_R3	22,22851181	-79,45140076	-943,9655151	-0,106833
Cloud_Mesh_Distance_BA_R0_R3	21,92803574	-79,19397736	-944,1853027	-0,082319
Cloud_Mesh_Distance_BA_R0_R3	21,99891281	-79,24146271	-944,1290283	-0,086731
Cloud_Mesh_Distance_BA_R0_R3	22,07423019	-79,18019867	-943,9776001	-0,074151
Cloud_Mesh_Distance_BA_R0_R3	22,15075493	-79,40353394	-944,0417481	-0,102608
Cloud_Mesh_Distance_BA_R0_R3	15,08969688	-78,53456116	-943,8642578	-0,104103
Cloud_Mesh_Distance_BA_R0_R3	21,97851944	-79,12236786	-944,0640869	-0,072821
Cloud_Mesh_Distance_BA_R0_R3	21,96564865	-79,03716278	-944,0105591	-0,065311
Cloud_Mesh_Distance_BA_R0_R3	15,14490604	-78,34152985	-943,8655396	-0,103651
Cloud_Mesh_Distance_BA_R0_R3	15,18938065	-78,19606781	-943,9296875	-0,092634
Cloud_Mesh_Distance_BA_R0_R3	15,197505	-78,21971893	-943,791687	-0,107285
Cloud_Mesh_Distance_BA_R0_R3	21,8050766	-78,89093781	-944,1011353	-0,050079
Cloud_Mesh_Distance_BA_R0_R3	21,8790741	-79,03510284	-944,1246948	-0,066289
Cloud_Mesh_Distance_BA_R0_R3	22,02303696	-79,05535126	-943,9464111	-0,064321

Cloud_Mesh_Distance_BA_R0_R3	15,31848812	-77,96348572	-943,7056274	-0,116175
Cloud_Mesh_Distance_BA_R0_R3	21,70940018	-78,59482574	-943,9648438	-0,019128
Cloud_Mesh_Distance_BA_R0_R3	21,7963028	-78,81493378	-944,0472412	-0,044561
Cloud_Mesh_Distance_BA_R0_R3	21,87192726	-78,83917999	-943,9680786	-0,044489
Cloud_Mesh_Distance_BA_R0_R3	21,79501343	-78,72916412	-943,9730835	-0,036015
Cloud_Mesh_Distance_BA_R0_R3	15,36287403	-77,81536865	-943,8718872	-0,103359
Cloud_Mesh_Distance_BA_R0_R3	21,60130882	-78,5442276	-944,0667725	-0,025985
Cloud_Mesh_Distance_BA_R0_R3	15,43106461	-77,68585968	-943,8306274	-0,104259
Cloud_Mesh_Distance_BA_R0_R3	15,41821575	-77,67967987	-944,0238037	-0,095324
Cloud_Mesh_Distance_BA_R0_R3	15,49367428	-77,54264069	-943,966919	-0,095936
Cloud_Mesh_Distance_BA_R0_R3	21,41469574	-78,42255402	-944,2089844	-0,017418
Cloud_Mesh_Distance_BA_R0_R3	21,46128082	-78,31972504	-944,0570679	-0,003171
Cloud_Mesh_Distance_BA_R0_R3	21,23587036	-78,11262512	-944,1823731	-0,000867
Cloud_Mesh_Distance_BA_R0_R3	21,32447433	-78,05010223	-943,9950562	0,021161
Cloud_Mesh_Distance_BA_R0_R3	21,33567047	-78,25029755	-944,1685181	-0,004278
Cloud_Mesh_Distance_BA_R0_R3	21,43342018	-78,1853714	-943,9675293	0,00881
Cloud_Mesh_Distance_BA_R0_R3	15,59244919	-77,37657166	-943,8966065	-0,091572
Cloud_Mesh_Distance_BA_R0_R3	15,67382813	-77,2496109	-943,8602905	-0,09149
Cloud_Mesh_Distance_BA_R0_R3	21,13420105	-77,86016083	-944,0792847	0,02055
Cloud_Mesh_Distance_BA_R0_R3	21,21718979	-77,98057556	-944,0838623	0,017
Cloud_Mesh_Distance_BA_R0_R3	21,13823318	-78,00948334	-944,2198486	0,00043
Cloud_Mesh_Distance_BA_R0_R3	15,73218155	-77,15868378	-943,9191895	-0,091718
Cloud_Mesh_Distance_BA_R0_R3	15,72085953	-77,18909454	-943,786438	-0,093341
Cloud_Mesh_Distance_BA_R0_R3	20,92297554	-77,6920166	-944,1981201	0,005662
Cloud_Mesh_Distance_BA_R0_R3	20,89263153	-77,58423615	-944,1173706	0,008185
Cloud_Mesh_Distance_BA_R0_R3	21,02356339	-77,7642746	-944,1340942	0,014144
Cloud_Mesh_Distance_BA_R0_R3	20,9878788	-77,64232635	-944,0429688	0,020677
Cloud_Mesh_Distance_BA_R0_R3	15,80358505	-77,07576752	-943,7875977	-0,091311
Cloud_Mesh_Distance_BA_R0_R3	15,89532471	-76,95468903	-943,8902588	-0,090176
Cloud_Mesh_Distance_BA_R0_R3	15,88567924	-76,95952606	-944,0421753	-0,085637
Cloud_Mesh_Distance_BA_R0_R3	16,0862999	-76,74731445	-944,1033936	-0,081784
Cloud_Mesh_Distance_BA_R0_R3	16,09286118	-76,74568939	-943,9052734	-0,084249
Cloud_Mesh_Distance_BA_R0_R3	16,19239426	-76,64896393	-944,0079956	-0,078379
Cloud_Mesh_Distance_BA_R0_R3	20,54165268	-77,09698486	-944,0214233	0,002037
Cloud_Mesh_Distance_BA_R0_R3	20,65324402	-77,39336395	-944,2568359	-0,014163
Cloud_Mesh_Distance_BA_R0_R3	20,58217621	-77,22335052	-944,1513672	-0,004187
Cloud_Mesh_Distance_BA_R0_R3	16,38043976	-76,49372101	-944,0064697	-0,059625
Cloud_Mesh_Distance_BA_R0_R3	20,37803268	-76,92373657	-944,0409546	0,002037
Cloud_Mesh_Distance_BA_R0_R3	20,48665619	-77,11068726	-944,1602173	0,000066
Cloud_Mesh_Distance_BA_R0_R3	16,41236115	-76,47673798	-943,8465576	-0,054525
Cloud_Mesh_Distance_BA_R0_R3	20,30198288	-76,94213104	-944,2323608	0,000919
Cloud_Mesh_Distance_BA_R0_R3	16,50532532	-76,40561676	-943,9979248	-0,050302
Cloud_Mesh_Distance_BA_R0_R3	16,53873444	-76,39409637	-943,7897339	-0,041569
Cloud_Mesh_Distance_BA_R0_R3	16,64710808	-76,32262421	-943,8834839	-0,034628
Cloud_Mesh_Distance_BA_R0_R3	16,92678261	-76,19400024	-943,9840088	-0,017373
Cloud_Mesh_Distance_BA_R0_R3	16,8083725	-76,2405777	-943,9421997	-0,0233
Cloud_Mesh_Distance_BA_R0_R3	16,87186432	-76,21981049	-944,1132813	-0,023433
Cloud_Mesh_Distance_BA_R0_R3	16,77127457	-76,26155853	-944,1130981	-0,03011
Cloud_Mesh_Distance_BA_R0_R3	19,74751282	-76,45362091	-944,0377197	-0,013651
Cloud_Mesh_Distance_BA_R0_R3	19,72318649	-76,48683167	-944,1860352	-0,01565

Cloud_Mesh_Distance_BA_R0_R3	19,89790726	-76,58740997	-944,1898193	-0,006462
Cloud_Mesh_Distance_BA_R0_R3	19,88280869	-76,51419067	-943,9926758	-0,002894
Cloud_Mesh_Distance_BA_R0_R3	20,15104294	-76,74326324	-944,1251221	0,008405
Cloud_Mesh_Distance_BA_R0_R3	20,0796051	-76,73007965	-944,2279053	0,004968
Cloud_Mesh_Distance_BA_R0_R3	20,05566216	-76,67180634	-944,1331177	-0,000203
Cloud_Mesh_Distance_BA_R0_R3	20,16364288	-76,7039032	-943,9909668	0,000752
Cloud_Mesh_Distance_BA_R0_R3	20,0351944	-76,60949707	-943,991394	0,001046
Cloud_Mesh_Distance_BA_R0_R3	16,98657799	-76,17703247	-944,0535889	-0,017791
Cloud_Mesh_Distance_BA_R0_R3	17,05244446	-76,15424347	-943,918396	-0,016841
Cloud_Mesh_Distance_BA_R0_R3	17,3650322	-76,09345245	-943,9382324	-0,032048
Cloud_Mesh_Distance_BA_R0_R3	17,1394043	-76,1407547	-944,0882568	-0,027334
Cloud_Mesh_Distance_BA_R0_R3	17,57023621	-76,07889557	-944,0066528	-0,048572
Cloud_Mesh_Distance_BA_R0_R3	17,89097595	-76,06941223	-943,9901733	-0,060717
Cloud_Mesh_Distance_BA_R0_R3	18,02737427	-76,06967926	-943,9719849	-0,061013
Cloud_Mesh_Distance_BA_R0_R3	17,96237564	-76,086586	-944,1085205	-0,059974
Cloud_Mesh_Distance_BA_R0_R3	18,30471039	-76,1257782	-944,2335205	-0,054597
Cloud_Mesh_Distance_BA_R0_R3	18,11805153	-76,10462189	-944,1818237	-0,061329
Cloud_Mesh_Distance_BA_R0_R3	18,13769913	-76,07930756	-944,0150757	-0,063511
Cloud_Mesh_Distance_BA_R0_R3	18,45798492	-76,14946747	-944,286499	-0,047402
Cloud_Mesh_Distance_BA_R0_R3	18,50396538	-76,11885834	-944,1102295	-0,053047
Cloud_Mesh_Distance_BA_R0_R3	18,62050247	-76,10541534	-943,9631958	-0,046316
Cloud_Mesh_Distance_BA_R0_R3	18,78806496	-76,15302277	-944,1084595	-0,038265
Cloud_Mesh_Distance_BA_R0_R3	19,20921516	-76,23738861	-944,0432129	-0,023592
Cloud_Mesh_Distance_BA_R0_R3	19,65716934	-76,43003845	-944,1115723	-0,018608
Cloud_Mesh_Distance_BA_R0_R3	19,54908371	-76,38317108	-944,1192017	-0,022434
Cloud_Mesh_Distance_BA_R0_R3	17,75335884	-76,0681839	-943,9724731	-0,052364
Cloud_Mesh_Distance_BA_R0_R3	18,67939377	-76,1307373	-944,0720215	-0,045164
Cloud_Mesh_Distance_BA_R0_R3	18,93462372	-76,17192841	-944,0705566	-0,033131
Cloud_Mesh_Distance_BA_R0_R3	19,0739975	-76,18366241	-943,9646606	-0,031796
Cloud_Mesh_Distance_BA_R0_R3	17,37314034	-83,38306427	-943,3455811	-0,060498
Cloud_Mesh_Distance_BA_R0_R3	17,015522	-83,03726959	-943,4763184	-0,016926
Cloud_Mesh_Distance_BA_R0_R3	16,93301582	-82,93663788	-943,3946533	-0,026649
Cloud_Mesh_Distance_BA_R0_R3	17,21252251	-83,22473907	-943,3084106	-0,055576
Cloud_Mesh_Distance_BA_R0_R3	17,18683624	-83,2103653	-943,4638672	-0,033645
Cloud_Mesh_Distance_BA_R0_R3	17,27226067	-83,28874969	-943,3774414	-0,049708
Cloud_Mesh_Distance_BA_R0_R3	16,83083153	-82,82756805	-943,3912354	-0,016177
Cloud_Mesh_Distance_BA_R0_R3	16,92261887	-82,90314484	-943,2468262	-0,052656
Cloud_Mesh_Distance_BA_R0_R3	16,70377731	-82,72025299	-943,4944458	0,025692
Cloud_Mesh_Distance_BA_R0_R3	16,77392197	-82,8159256	-943,5872192	0,038075
Cloud_Mesh_Distance_BA_R0_R3	16,31636429	-82,33792877	-943,543396	0,105986
Cloud_Mesh_Distance_BA_R0_R3	16,53340149	-82,49861908	-943,3067627	0,015853
Cloud_Mesh_Distance_BA_R0_R3	16,50935745	-82,53127289	-943,5123291	0,066837
Cloud_Mesh_Distance_BA_R0_R3	16,15756035	-82,14620209	-943,5639648	0,114359
Cloud_Mesh_Distance_BA_R0_R3	16,23937225	-82,21390533	-943,4628906	0,085976
Cloud_Mesh_Distance_BA_R0_R3	23,18193054	-82,83144379	-943,6610107	0,062358
Cloud_Mesh_Distance_BA_R0_R3	23,23209763	-82,9497757	-943,5782471	0,064541
Cloud_Mesh_Distance_BA_R0_R3	16,16410446	-82,08446503	-943,3875732	0,066524
Cloud_Mesh_Distance_BA_R0_R3	16,08586502	-81,96495819	-943,3808594	0,055675
Cloud_Mesh_Distance_BA_R0_R3	23,07840919	-82,51084137	-943,7410278	0,062602
Cloud_Mesh_Distance_BA_R0_R3	23,14587021	-82,67136383	-943,6486206	0,076472

Cloud_Mesh_Distance_BA_R0_R3	15,87335396	-81,63471222	-943,5078735	0,021133
Cloud_Mesh_Distance_BA_R0_R3	15,88700199	-81,59830475	-943,2963867	0,002838
Cloud_Mesh_Distance_BA_R0_R3	15,79718494	-81,52085114	-943,625061	-0,000576
Cloud_Mesh_Distance_BA_R0_R3	15,80831242	-81,49234772	-943,4193726	-0,011092
Cloud_Mesh_Distance_BA_R0_R3	15,95379543	-81,7443924	-943,3869019	0,026721
Cloud_Mesh_Distance_BA_R0_R3	23,04026031	-82,34679413	-943,7147827	0,056239
Cloud_Mesh_Distance_BA_R0_R3	23,02880096	-82,23752594	-943,647522	0,05449
Cloud_Mesh_Distance_BA_R0_R3	15,74088955	-81,35631561	-943,345398	-0,028688
Cloud_Mesh_Distance_BA_R0_R3	23,04257965	-82,18921661	-943,5447388	0,061031
Cloud_Mesh_Distance_BA_R0_R3	22,99041176	-82,07062531	-943,6204224	0,029994
Cloud_Mesh_Distance_BA_R0_R3	15,66475201	-81,25696564	-943,46698	-0,040711
Cloud_Mesh_Distance_BA_R0_R3	15,67674541	-81,22602081	-943,267273	-0,034536
Cloud_Mesh_Distance_BA_R0_R3	15,59686947	-81,1264267	-943,37677	-0,043896
Cloud_Mesh_Distance_BA_R0_R3	15,58678627	-81,14951324	-943,5858765	-0,057588
Cloud_Mesh_Distance_BA_R0_R3	22,90853882	-81,94351959	-943,7750855	0,003905
Cloud_Mesh_Distance_BA_R0_R3	15,51672363	-81,02259064	-943,4924316	-0,060727
Cloud_Mesh_Distance_BA_R0_R3	15,38879967	-80,74636078	-943,3019409	-0,050053
Cloud_Mesh_Distance_BA_R0_R3	22,81490707	-81,65387726	-943,8508911	-0,043126
Cloud_Mesh_Distance_BA_R0_R3	15,3122673	-80,63486481	-943,427002	-0,061402
Cloud_Mesh_Distance_BA_R0_R3	15,2411499	-80,51006317	-943,5818481	-0,080011
Cloud_Mesh_Distance_BA_R0_R3	22,77094269	-81,29453278	-943,8233032	-0,081693
Cloud_Mesh_Distance_BA_R0_R3	22,82952499	-81,28579712	-943,6783447	-0,082808
Cloud_Mesh_Distance_BA_R0_R3	22,80255127	-81,44776154	-943,7938232	-0,066367
Cloud_Mesh_Distance_BA_R0_R3	22,8516407	-81,42057037	-943,6575928	-0,065572
Cloud_Mesh_Distance_BA_R0_R3	15,2021637	-80,35865021	-943,4909668	-0,086187
Cloud_Mesh_Distance_BA_R0_R3	22,77393723	-81,15190125	-943,7827148	-0,091184
Cloud_Mesh_Distance_BA_R0_R3	22,71591949	-81,21607208	-943,9241943	-0,088432
Cloud_Mesh_Distance_BA_R0_R3	15,17114735	-80,18948364	-943,434021	-0,108165
Cloud_Mesh_Distance_BA_R0_R3	15,12163258	-80,07556915	-943,6187134	-0,13182
Cloud_Mesh_Distance_BA_R0_R3	22,77931595	-80,98571014	-943,7381592	-0,102768
Cloud_Mesh_Distance_BA_R0_R3	22,73043442	-80,85899353	-943,8171997	-0,111607
Cloud_Mesh_Distance_BA_R0_R3	22,78832245	-80,84632111	-943,694397	-0,109323
Cloud_Mesh_Distance_BA_R0_R3	22,835392	-80,92771149	-943,6012573	-0,10517
Cloud_Mesh_Distance_BA_R0_R3	15,10456657	-79,90122223	-943,5651245	-0,147729
Cloud_Mesh_Distance_BA_R0_R3	15,14010906	-80,0296936	-943,4557495	-0,128773
Cloud_Mesh_Distance_BA_R0_R3	15,11223221	-79,75693512	-943,4465942	-0,151703
Cloud_Mesh_Distance_BA_R0_R3	15,13677025	-79,92116547	-943,4025269	-0,138346
Cloud_Mesh_Distance_BA_R0_R3	22,6560936	-80,52393341	-943,8781738	-0,125096
Cloud_Mesh_Distance_BA_R0_R3	22,73493767	-80,50283051	-943,7261353	-0,125961
Cloud_Mesh_Distance_BA_R0_R3	15,06367302	-79,67744446	-943,6766968	-0,157153
Cloud_Mesh_Distance_BA_R0_R3	15,07445812	-79,56502533	-943,5667725	-0,159586
Cloud_Mesh_Distance_BA_R0_R3	15,08177376	-79,70702362	-943,5845337	-0,156257
Cloud_Mesh_Distance_BA_R0_R3	22,65880394	-80,35918427	-943,819519	-0,133123
Cloud_Mesh_Distance_BA_R0_R3	22,57196808	-80,21925354	-943,9107666	-0,135769
Cloud_Mesh_Distance_BA_R0_R3	22,65721512	-80,19618988	-943,7557373	-0,135439
Cloud_Mesh_Distance_BA_R0_R3	15,0615263	-79,3711319	-943,5822754	-0,156222
Cloud_Mesh_Distance_BA_R0_R3	15,10906982	-79,38425446	-943,3790894	-0,159327
Cloud_Mesh_Distance_BA_R0_R3	22,61526871	-80,03499603	-943,7473755	-0,135274
Cloud_Mesh_Distance_BA_R0_R3	22,69107628	-80,11256409	-943,6538696	-0,134437
Cloud_Mesh_Distance_BA_R0_R3	22,7349205	-80,26644135	-943,6445313	-0,129672

Cloud_Mesh_Distance_BA_R0_R3	15,07176971	-79,24991608	-943,5267334	-0,155333
Cloud_Mesh_Distance_BA_R0_R3	15,06556129	-79,07604218	-943,569519	-0,14592
Cloud_Mesh_Distance_BA_R0_R3	22,31200027	-79,62625885	-943,9697876	-0,128085
Cloud_Mesh_Distance_BA_R0_R3	22,3999176	-79,62352753	-943,8320313	-0,123337
Cloud_Mesh_Distance_BA_R0_R3	22,45219231	-79,75179291	-943,8409424	-0,133251
Cloud_Mesh_Distance_BA_R0_R3	22,38195801	-79,70922089	-943,9229736	-0,13115
Cloud_Mesh_Distance_BA_R0_R3	15,13914394	-78,66498566	-943,4736328	-0,135014
Cloud_Mesh_Distance_BA_R0_R3	15,09423542	-78,68454742	-943,6433716	-0,128078
Cloud_Mesh_Distance_BA_R0_R3	15,09220886	-78,88977814	-943,5200806	-0,141043
Cloud_Mesh_Distance_BA_R0_R3	15,04862881	-78,89551544	-943,7105103	-0,125027
Cloud_Mesh_Distance_BA_R0_R3	15,1642828	-78,75511932	-943,3308106	-0,142731
Cloud_Mesh_Distance_BA_R0_R3	22,341856	-79,47835541	-943,8117065	-0,10754
Cloud_Mesh_Distance_BA_R0_R3	22,50854111	-79,62298584	-943,6472168	-0,12187
Cloud_Mesh_Distance_BA_R0_R3	22,46644211	-79,65114594	-943,7435303	-0,123534
Cloud_Mesh_Distance_BA_R0_R3	15,17362404	-78,60126495	-943,3989258	-0,138642
Cloud_Mesh_Distance_BA_R0_R3	22,16643524	-79,17089081	-943,8311768	-0,074871
Cloud_Mesh_Distance_BA_R0_R3	22,16176987	-79,30626678	-943,9511108	-0,092255
Cloud_Mesh_Distance_BA_R0_R3	15,12505817	-78,48436737	-943,7177734	-0,118645
Cloud_Mesh_Distance_BA_R0_R3	15,15251637	-78,51796722	-943,5494995	-0,126641
Cloud_Mesh_Distance_BA_R0_R3	15,16786003	-78,36274719	-943,6796875	-0,117796
Cloud_Mesh_Distance_BA_R0_R3	22,1126976	-79,00714111	-943,7685547	-0,054999
Cloud_Mesh_Distance_BA_R0_R3	22,20341492	-79,10831451	-943,71521	-0,067439
Cloud_Mesh_Distance_BA_R0_R3	15,27832317	-78,15714264	-943,5181885	-0,121612
Cloud_Mesh_Distance_BA_R0_R3	15,25225163	-78,10192871	-943,7415772	-0,112167
Cloud_Mesh_Distance_BA_R0_R3	21,98078537	-78,94219208	-943,9078369	-0,049791
Cloud_Mesh_Distance_BA_R0_R3	21,9588623	-78,7877121	-943,7944336	-0,028362
Cloud_Mesh_Distance_BA_R0_R3	15,41351891	-77,8102951	-943,5714722	-0,111167
Cloud_Mesh_Distance_BA_R0_R3	15,38527966	-77,82054901	-943,6948853	-0,11417
Cloud_Mesh_Distance_BA_R0_R3	15,36116505	-77,96105194	-943,5059815	-0,11738
Cloud_Mesh_Distance_BA_R0_R3	21,5405674	-78,35510254	-943,9752808	-0,003387
Cloud_Mesh_Distance_BA_R0_R3	21,61211395	-78,44238281	-943,9563599	-0,010165
Cloud_Mesh_Distance_BA_R0_R3	21,87012482	-78,69536591	-943,8334351	-0,023318
Cloud_Mesh_Distance_BA_R0_R3	21,80105591	-78,63863373	-943,8760376	-0,022748
Cloud_Mesh_Distance_BA_R0_R3	15,43794918	-77,71656036	-943,6771851	-0,11192
Cloud_Mesh_Distance_BA_R0_R3	15,50050449	-77,57416534	-943,7333374	-0,104658
Cloud_Mesh_Distance_BA_R0_R3	21,50956917	-78,26719666	-943,9338379	0,006149
Cloud_Mesh_Distance_BA_R0_R3	21,59038162	-78,30438995	-943,8462524	0,011052
Cloud_Mesh_Distance_BA_R0_R3	21,43821335	-78,06253815	-943,8209839	0,025316
Cloud_Mesh_Distance_BA_R0_R3	21,58522415	-78,20563507	-943,7374268	0,019369
Cloud_Mesh_Distance_BA_R0_R3	21,51644516	-78,18518066	-943,8325806	0,017044
Cloud_Mesh_Distance_BA_R0_R3	15,57666492	-77,44669342	-943,6759644	-0,099546
Cloud_Mesh_Distance_BA_R0_R3	15,61585236	-77,42806244	-943,5091553	-0,095305
Cloud_Mesh_Distance_BA_R0_R3	15,65692043	-77,30238342	-943,7014771	-0,092756
Cloud_Mesh_Distance_BA_R0_R3	21,09087372	-77,72306061	-943,9835815	0,02565
Cloud_Mesh_Distance_BA_R0_R3	21,24005508	-77,89070892	-943,9511108	0,030929
Cloud_Mesh_Distance_BA_R0_R3	21,17209244	-77,7898941	-943,9376831	0,029144
Cloud_Mesh_Distance_BA_R0_R3	21,23456573	-77,78604889	-943,8215942	0,033346
Cloud_Mesh_Distance_BA_R0_R3	21,34245682	-77,90534973	-943,7896118	0,034556
Cloud_Mesh_Distance_BA_R0_R3	15,7441721	-77,17972565	-943,6450806	-0,085426
Cloud_Mesh_Distance_BA_R0_R3	21,15246964	-77,70922089	-943,8590088	0,03337

Cloud_Mesh_Distance_BA_R0_R3	21,29284096	-77,77866364	-943,6935425	0,034323
Cloud_Mesh_Distance_BA_R0_R3	15,99915218	-76,8483963	-943,7954102	-0,083103
Cloud_Mesh_Distance_BA_R0_R3	20,76865196	-77,29598236	-943,899353	0,003789
Cloud_Mesh_Distance_BA_R0_R3	20,95062256	-77,43479156	-943,7774658	0,015239
Cloud_Mesh_Distance_BA_R0_R3	20,83509064	-77,39398956	-943,9389648	0,009439
Cloud_Mesh_Distance_BA_R0_R3	20,92288971	-77,51743317	-943,9755859	0,011222
Cloud_Mesh_Distance_BA_R0_R3	16,11346626	-76,74434662	-943,6974487	-0,065742
Cloud_Mesh_Distance_BA_R0_R3	20,58807755	-77,06920624	-943,8460693	-0,0032
Cloud_Mesh_Distance_BA_R0_R3	20,6901741	-77,13951874	-943,7431641	-0,000519
Cloud_Mesh_Distance_BA_R0_R3	20,71962357	-77,32580566	-944,0495606	0,000105
Cloud_Mesh_Distance_BA_R0_R3	20,66831779	-77,19576263	-943,9265137	-0,004259
Cloud_Mesh_Distance_BA_R0_R3	20,77476501	-77,24269104	-943,7727661	-0,000258
Cloud_Mesh_Distance_BA_R0_R3	16,29754829	-76,5618515	-943,9171753	-0,065952
Cloud_Mesh_Distance_BA_R0_R3	16,20462799	-76,64817047	-943,8130493	-0,067641
Cloud_Mesh_Distance_BA_R0_R3	16,31983948	-76,5587616	-943,7294922	-0,056675
Cloud_Mesh_Distance_BA_R0_R3	20,47806358	-76,98664093	-943,9393311	-0,003122
Cloud_Mesh_Distance_BA_R0_R3	16,64337349	-76,33611298	-943,7346802	-0,030684
Cloud_Mesh_Distance_BA_R0_R3	16,59615707	-76,37711334	-943,630127	-0,029143
Cloud_Mesh_Distance_BA_R0_R3	20,26771736	-76,81710815	-944,0502319	0,007524
Cloud_Mesh_Distance_BA_R0_R3	16,76755524	-76,27297211	-943,7191162	-0,023234
Cloud_Mesh_Distance_BA_R0_R3	17,07215881	-76,15836334	-943,6821289	-0,017318
Cloud_Mesh_Distance_BA_R0_R3	20,01888275	-76,55890656	-943,8413086	-0,004615
Cloud_Mesh_Distance_BA_R0_R3	16,94558716	-76,1883316	-943,8764648	-0,01872
Cloud_Mesh_Distance_BA_R0_R3	17,03083038	-76,16353607	-943,7905884	-0,019315
Cloud_Mesh_Distance_BA_R0_R3	17,21300888	-76,11634827	-943,8842163	-0,023456
Cloud_Mesh_Distance_BA_R0_R3	17,72709084	-76,05809784	-943,8344727	-0,045963
Cloud_Mesh_Distance_BA_R0_R3	17,58267593	-76,06434631	-943,7561646	-0,036961
Cloud_Mesh_Distance_BA_R0_R3	18,46341705	-76,0853653	-943,9230347	-0,050523
Cloud_Mesh_Distance_BA_R0_R3	18,30416679	-76,09441376	-944,0566406	-0,060462
Cloud_Mesh_Distance_BA_R0_R3	19,20600128	-76,20136261	-943,8505249	-0,030806
Cloud_Mesh_Distance_BA_R0_R3	19,52540779	-76,29764557	-943,760376	-0,028218
Cloud_Mesh_Distance_BA_R0_R3	19,36651039	-76,2642746	-943,914917	-0,025093
Cloud_Mesh_Distance_BA_R0_R3	19,34864807	-76,22519684	-943,6852417	-0,034285
Cloud_Mesh_Distance_BA_R0_R3	19,53055191	-76,3363266	-943,961853	-0,024275
Cloud_Mesh_Distance_BA_R0_R3	19,69513321	-76,3758316	-943,8032227	-0,025806
Cloud_Mesh_Distance_BA_R0_R3	19,66529465	-76,40139008	-943,9908447	-0,021242
Cloud_Mesh_Distance_BA_R0_R3	17,85851669	-76,04795837	-943,7214356	-0,046799
Cloud_Mesh_Distance_BA_R0_R3	18,01786995	-76,05741119	-943,8623657	-0,057159
Cloud_Mesh_Distance_BA_R0_R3	17,87205887	-76,0555954	-943,8504639	-0,050891
Cloud_Mesh_Distance_BA_R0_R3	18,00434875	-76,04659271	-943,7275391	-0,051673
Cloud_Mesh_Distance_BA_R0_R3	18,27594757	-76,06965637	-943,8942871	-0,05826
Cloud_Mesh_Distance_BA_R0_R3	18,13016129	-76,06363678	-943,8956299	-0,057192
Cloud_Mesh_Distance_BA_R0_R3	18,18991852	-76,0513382	-943,7478027	-0,053704
Cloud_Mesh_Distance_BA_R0_R3	18,63371658	-76,08424377	-943,7902222	-0,046153
Cloud_Mesh_Distance_BA_R0_R3	18,80057144	-76,10154724	-943,7575684	-0,044991
Cloud_Mesh_Distance_BA_R0_R3	18,7947197	-76,12291718	-943,9337158	-0,039621
Cloud_Mesh_Distance_BA_R0_R3	18,95477867	-76,14763641	-943,9117432	-0,032226
Cloud_Mesh_Distance_BA_R0_R3	19,04738808	-76,15068817	-943,7825928	-0,034169
Cloud_Mesh_Distance_BA_R0_R3	17,28750992	-83,29701996	-943,2369995	-0,064794
Cloud_Mesh_Distance_BA_R0_R3	16,99887276	-82,99474335	-943,3048706	-0,046582

Cloud_Mesh_Distance_BA_R0_R3	17,10785294	-83,11292267	-943,2930298	-0,053867
Cloud_Mesh_Distance_BA_R0_R3	16,91566277	-82,87760162	-943,100708	-0,071212
Cloud_Mesh_Distance_BA_R0_R3	16,73571396	-82,70589447	-943,2802734	-0,025672
Cloud_Mesh_Distance_BA_R0_R3	16,76109695	-82,69722748	-943,0496826	-0,06231
Cloud_Mesh_Distance_BA_R0_R3	16,83939362	-82,80200958	-943,1801758	-0,053594
Cloud_Mesh_Distance_BA_R0_R3	16,34418678	-82,28739166	-943,2997437	0,039737
Cloud_Mesh_Distance_BA_R0_R3	16,45083618	-82,39212799	-943,2355347	0,014504
Cloud_Mesh_Distance_BA_R0_R3	16,45092773	-82,36248016	-943,1085815	0,000974
Cloud_Mesh_Distance_BA_R0_R3	16,64302444	-82,58887482	-943,1810913	-0,026211
Cloud_Mesh_Distance_BA_R0_R3	16,62124252	-82,61118317	-943,3960571	0,015838
Cloud_Mesh_Distance_BA_R0_R3	16,54087448	-82,4600296	-943,0904541	-0,019672
Cloud_Mesh_Distance_BA_R0_R3	16,24718094	-82,17031097	-943,3076782	0,049786
Cloud_Mesh_Distance_BA_R0_R3	16,38116074	-82,27169037	-943,0736694	0,00911
Cloud_Mesh_Distance_BA_R0_R3	16,28306961	-82,1714859	-943,1549683	0,028411
Cloud_Mesh_Distance_BA_R0_R3	16,40671539	-82,32874298	-943,1806641	0,015391
Cloud_Mesh_Distance_BA_R0_R3	16,20846939	-82,09352875	-943,2265625	0,039393
Cloud_Mesh_Distance_BA_R0_R3	16,13366508	-81,97858429	-943,2026367	0,037068
Cloud_Mesh_Distance_BA_R0_R3	23,17708397	-82,59888458	-943,4450684	0,106895
Cloud_Mesh_Distance_BA_R0_R3	23,26377106	-82,86626434	-943,3762207	0,08246
Cloud_Mesh_Distance_BA_R0_R3	23,20687103	-82,76808929	-943,5040283	0,083311
Cloud_Mesh_Distance_BA_R0_R3	15,97266102	-81,70159149	-943,1508179	0,015862
Cloud_Mesh_Distance_BA_R0_R3	16,03794098	-81,85297394	-943,2780762	0,036347
Cloud_Mesh_Distance_BA_R0_R3	23,12678909	-82,50972748	-943,5574951	0,093079
Cloud_Mesh_Distance_BA_R0_R3	15,81938076	-81,44664764	-943,1932373	-0,012451
Cloud_Mesh_Distance_BA_R0_R3	23,14365005	-82,43976593	-943,4037476	0,110864
Cloud_Mesh_Distance_BA_R0_R3	15,74664402	-81,31226349	-943,1721802	-0,021508
Cloud_Mesh_Distance_BA_R0_R3	23,08244896	-82,33917999	-943,5496216	0,084427
Cloud_Mesh_Distance_BA_R0_R3	23,0742836	-82,19101715	-943,4129028	0,078589
Cloud_Mesh_Distance_BA_R0_R3	22,88499832	-81,76607513	-943,7226563	-0,021415
Cloud_Mesh_Distance_BA_R0_R3	22,94319344	-81,87300873	-943,614563	0,005476
Cloud_Mesh_Distance_BA_R0_R3	23,03856659	-82,0558548	-943,418457	0,054041
Cloud_Mesh_Distance_BA_R0_R3	23,03650284	-82,12352753	-943,4984741	0,055208
Cloud_Mesh_Distance_BA_R0_R3	15,47903442	-80,84237671	-943,147583	-0,0379
Cloud_Mesh_Distance_BA_R0_R3	15,45065403	-80,88936615	-943,3904419	-0,050009
Cloud_Mesh_Distance_BA_R0_R3	15,61457443	-81,08174896	-943,1567383	-0,037277
Cloud_Mesh_Distance_BA_R0_R3	15,53227615	-80,99263	-943,2827759	-0,040537
Cloud_Mesh_Distance_BA_R0_R3	22,87385559	-81,56412506	-943,6494751	-0,044279
Cloud_Mesh_Distance_BA_R0_R3	22,97959518	-81,66452789	-943,3380127	-0,021177
Cloud_Mesh_Distance_BA_R0_R3	22,99568939	-81,88098907	-943,4287109	0,014107
Cloud_Mesh_Distance_BA_R0_R3	22,93463135	-81,71686554	-943,5374146	-0,020834
Cloud_Mesh_Distance_BA_R0_R3	22,92798233	-81,56517792	-943,4799194	-0,039255
Cloud_Mesh_Distance_BA_R0_R3	22,95198822	-81,4977951	-943,3691406	-0,049044
Cloud_Mesh_Distance_BA_R0_R3	15,33887005	-80,58991241	-943,2070923	-0,050507
Cloud_Mesh_Distance_BA_R0_R3	15,21978855	-80,31778717	-943,3318481	-0,086149
Cloud_Mesh_Distance_BA_R0_R3	15,26304531	-80,46599579	-943,3347778	-0,072343
Cloud_Mesh_Distance_BA_R0_R3	15,30120087	-80,46840668	-943,1790161	-0,058355
Cloud_Mesh_Distance_BA_R0_R3	15,26795292	-80,32842255	-943,1516724	-0,067684
Cloud_Mesh_Distance_BA_R0_R3	22,87524033	-81,20506287	-943,5413818	-0,085521
Cloud_Mesh_Distance_BA_R0_R3	15,21206474	-80,21366119	-943,2622681	-0,094652
Cloud_Mesh_Distance_BA_R0_R3	15,181777	-80,04044342	-943,2685547	-0,113052

Cloud_Mesh_Distance_BA_R0_R3	22,8299942	-81,09928131	-943,640686	-0,094786
Cloud_Mesh_Distance_BA_R0_R3	22,88563156	-81,04584503	-943,4915161	-0,098417
Cloud_Mesh_Distance_BA_R0_R3	22,76507759	-80,70601654	-943,7164307	-0,117078
Cloud_Mesh_Distance_BA_R0_R3	22,82987785	-80,75966644	-943,5887451	-0,112755
Cloud_Mesh_Distance_BA_R0_R3	22,89591599	-80,83744049	-943,4428711	-0,108872
Cloud_Mesh_Distance_BA_R0_R3	15,12002754	-79,58982086	-943,3640747	-0,159212
Cloud_Mesh_Distance_BA_R0_R3	15,16008949	-79,5266037	-943,2062378	-0,152376
Cloud_Mesh_Distance_BA_R0_R3	22,75420761	-80,08150482	-943,5159302	-0,12536
Cloud_Mesh_Distance_BA_R0_R3	15,10053444	-79,18863678	-943,4136963	-0,155534
Cloud_Mesh_Distance_BA_R0_R3	15,16501236	-79,37674713	-943,1819458	-0,156559
Cloud_Mesh_Distance_BA_R0_R3	15,15217304	-79,19726563	-943,2327271	-0,154181
Cloud_Mesh_Distance_BA_R0_R3	22,63088417	-79,83712006	-943,5911865	-0,126597
Cloud_Mesh_Distance_BA_R0_R3	22,68347168	-79,99742889	-943,6027832	-0,13092
Cloud_Mesh_Distance_BA_R0_R3	15,11601162	-79,03240204	-943,3838501	-0,14961
Cloud_Mesh_Distance_BA_R0_R3	22,53402328	-79,8757019	-943,7904663	-0,138662
Cloud_Mesh_Distance_BA_R0_R3	15,1314497	-78,88188171	-943,3787231	-0,144736
Cloud_Mesh_Distance_BA_R0_R3	15,1221714	-78,77414703	-943,4644775	-0,137136
Cloud_Mesh_Distance_BA_R0_R3	15,21065617	-78,79969025	-943,170166	-0,142391
Cloud_Mesh_Distance_BA_R0_R3	22,4423027	-79,52994537	-943,6855469	-0,11317
Cloud_Mesh_Distance_BA_R0_R3	22,52853775	-79,73361969	-943,699646	-0,127024
Cloud_Mesh_Distance_BA_R0_R3	15,22098541	-78,61006927	-943,2448731	-0,136683
Cloud_Mesh_Distance_BA_R0_R3	22,27765846	-79,2689743	-943,7380981	-0,087617
Cloud_Mesh_Distance_BA_R0_R3	22,38504028	-79,38295746	-943,656311	-0,099428
Cloud_Mesh_Distance_BA_R0_R3	22,36393356	-79,28707123	-943,6043701	-0,085549
Cloud_Mesh_Distance_BA_R0_R3	15,2027483	-78,46547699	-943,4163208	-0,133225
Cloud_Mesh_Distance_BA_R0_R3	15,26739216	-78,37056732	-943,3002319	-0,136647
Cloud_Mesh_Distance_BA_R0_R3	22,26313019	-79,08359528	-943,5880127	-0,055759
Cloud_Mesh_Distance_BA_R0_R3	22,42567444	-79,32169342	-943,5244751	-0,090528
Cloud_Mesh_Distance_BA_R0_R3	22,36903954	-79,1873703	-943,4966431	-0,06707
Cloud_Mesh_Distance_BA_R0_R3	15,19182587	-78,38218689	-943,5470581	-0,127823
Cloud_Mesh_Distance_BA_R0_R3	15,22922897	-78,32359314	-943,4780884	-0,126013
Cloud_Mesh_Distance_BA_R0_R3	15,21636963	-78,25424957	-943,6241455	-0,121258
Cloud_Mesh_Distance_BA_R0_R3	15,34298897	-78,15581512	-943,2928467	-0,129835
Cloud_Mesh_Distance_BA_R0_R3	22,03754425	-78,85954285	-943,7442627	-0,031166
Cloud_Mesh_Distance_BA_R0_R3	22,02185822	-78,7614975	-943,6662598	-0,020212
Cloud_Mesh_Distance_BA_R0_R3	22,1045208	-78,82173157	-943,5935669	-0,024236
Cloud_Mesh_Distance_BA_R0_R3	22,204422	-78,94025421	-943,5454712	-0,033121
Cloud_Mesh_Distance_BA_R0_R3	22,1959343	-79,03069305	-943,6525879	-0,054279
Cloud_Mesh_Distance_BA_R0_R3	15,39345837	-77,99221039	-943,3388672	-0,127782
Cloud_Mesh_Distance_BA_R0_R3	22,04563904	-78,70700836	-943,5618897	-0,014086
Cloud_Mesh_Distance_BA_R0_R3	15,42481327	-77,85866547	-943,4283447	-0,114632
Cloud_Mesh_Distance_BA_R0_R3	21,82285309	-78,54529572	-943,7433472	-0,007696
Cloud_Mesh_Distance_BA_R0_R3	21,69765091	-78,41687775	-943,798523	-0,001286
Cloud_Mesh_Distance_BA_R0_R3	21,95585442	-78,65331268	-943,6521606	-0,008679
Cloud_Mesh_Distance_BA_R0_R3	15,46434212	-77,71315002	-943,5454712	-0,110343
Cloud_Mesh_Distance_BA_R0_R3	15,49287891	-77,76599884	-943,328186	-0,115778
Cloud_Mesh_Distance_BA_R0_R3	15,58235741	-77,58126068	-943,3156738	-0,100423
Cloud_Mesh_Distance_BA_R0_R3	15,53442955	-77,5797348	-943,5203857	-0,104653
Cloud_Mesh_Distance_BA_R0_R3	21,66112518	-78,27863312	-943,6941528	0,012371
Cloud_Mesh_Distance_BA_R0_R3	15,67381096	-77,40618134	-943,3116455	-0,081657

Cloud_Mesh_Distance_BA_R0_R3	21,53503609	-78,10404205	-943,696228	0,023443
Cloud_Mesh_Distance_BA_R0_R3	15,7504158	-77,25153351	-943,3685913	-0,07303
Cloud_Mesh_Distance_BA_R0_R3	15,69791031	-77,28600311	-943,5095215	-0,083508
Cloud_Mesh_Distance_BA_R0_R3	15,7672205	-77,18122101	-943,5021362	-0,076404
Cloud_Mesh_Distance_BA_R0_R3	15,83377552	-77,07372284	-943,565918	-0,078402
Cloud_Mesh_Distance_BA_R0_R3	15,87893963	-77,05168915	-943,4242554	-0,065365
Cloud_Mesh_Distance_BA_R0_R3	21,05996323	-77,59758759	-943,8537598	0,024005
Cloud_Mesh_Distance_BA_R0_R3	21,19646454	-77,66812134	-943,7022095	0,036621
Cloud_Mesh_Distance_BA_R0_R3	21,30197525	-77,7175293	-943,5541382	0,035116
Cloud_Mesh_Distance_BA_R0_R3	15,9331913	-76,96805573	-943,4937744	-0,067432
Cloud_Mesh_Distance_BA_R0_R3	16,02038574	-76,85451508	-943,5821533	-0,068879
Cloud_Mesh_Distance_BA_R0_R3	15,91116619	-76,95761108	-943,6862183	-0,08181
Cloud_Mesh_Distance_BA_R0_R3	16,22913361	-76,64860535	-943,6221924	-0,056058
Cloud_Mesh_Distance_BA_R0_R3	16,26286697	-76,64531708	-943,4735107	-0,042076
Cloud_Mesh_Distance_BA_R0_R3	16,15288925	-76,74452972	-943,4697266	-0,052675
Cloud_Mesh_Distance_BA_R0_R3	20,62203979	-77,0533905	-943,7059937	-0,002556
Cloud_Mesh_Distance_BA_R0_R3	20,79271317	-77,19091034	-943,5889893	-0,004752
Cloud_Mesh_Distance_BA_R0_R3	20,8426857	-77,33377075	-943,8105469	0,009862
Cloud_Mesh_Distance_BA_R0_R3	20,51990509	-76,95833588	-943,7409668	-0,00754
Cloud_Mesh_Distance_BA_R0_R3	20,61845779	-77,01070404	-943,579773	-0,009098
Cloud_Mesh_Distance_BA_R0_R3	16,45815659	-76,46773529	-943,6292725	-0,038492
Cloud_Mesh_Distance_BA_R0_R3	16,69002151	-76,3227005	-943,6347656	-0,027659
Cloud_Mesh_Distance_BA_R0_R3	16,6920929	-76,34220123	-943,5087891	-0,020665
Cloud_Mesh_Distance_BA_R0_R3	16,77282333	-76,2912674	-943,5631104	-0,020963
Cloud_Mesh_Distance_BA_R0_R3	20,4015255	-76,82326508	-943,6783447	-0,007546
Cloud_Mesh_Distance_BA_R0_R3	20,283535	-76,72119904	-943,6924439	-0,008887
Cloud_Mesh_Distance_BA_R0_R3	20,39146423	-76,87441254	-943,8832398	-0,003246
Cloud_Mesh_Distance_BA_R0_R3	16,91736603	-76,23310089	-943,522644	-0,016029
Cloud_Mesh_Distance_BA_R0_R3	17,06843948	-76,17293549	-943,5552979	-0,013751
Cloud_Mesh_Distance_BA_R0_R3	16,93805695	-76,19913483	-943,7306519	-0,020156
Cloud_Mesh_Distance_BA_R0_R3	17,23047638	-76,14287567	-943,4276123	-0,011558
Cloud_Mesh_Distance_BA_R0_R3	19,79538155	-76,44280243	-943,8977051	-0,013915
Cloud_Mesh_Distance_BA_R0_R3	19,83430099	-76,43511963	-943,7583618	-0,02103
Cloud_Mesh_Distance_BA_R0_R3	19,92463684	-76,49178314	-943,7960815	-0,011791
Cloud_Mesh_Distance_BA_R0_R3	20,06821823	-76,54917908	-943,6271362	-0,017014
Cloud_Mesh_Distance_BA_R0_R3	20,15835953	-76,64664459	-943,8012085	-0,003068
Cloud_Mesh_Distance_BA_R0_R3	20,28221703	-76,76042938	-943,848877	-0,003602
Cloud_Mesh_Distance_BA_R0_R3	17,17463684	-76,1383667	-943,5862427	-0,01637
Cloud_Mesh_Distance_BA_R0_R3	17,17435074	-76,12755585	-943,7329102	-0,020497
Cloud_Mesh_Distance_BA_R0_R3	17,3690815	-76,08944702	-943,7302246	-0,027392
Cloud_Mesh_Distance_BA_R0_R3	17,35253143	-76,10419464	-943,5200806	-0,020087
Cloud_Mesh_Distance_BA_R0_R3	17,76873779	-76,05099487	-943,5449829	-0,03505
Cloud_Mesh_Distance_BA_R0_R3	19,18182373	-76,17524719	-943,6902466	-0,03773
Cloud_Mesh_Distance_BA_R0_R3	19,67268181	-76,33818817	-943,6103516	-0,022836
Cloud_Mesh_Distance_BA_R0_R3	19,8164196	-76,40637207	-943,630188	-0,021168
Cloud_Mesh_Distance_BA_R0_R3	17,75282669	-76,05136871	-943,6949463	-0,045418
Cloud_Mesh_Distance_BA_R0_R3	17,84995079	-76,04567719	-943,616394	-0,042852
Cloud_Mesh_Distance_BA_R0_R3	17,96868896	-76,0421524	-943,6040649	-0,043855
Cloud_Mesh_Distance_BA_R0_R3	18,13319397	-76,04116821	-943,5842285	-0,048959
Cloud_Mesh_Distance_BA_R0_R3	18,41692924	-76,06182098	-943,7453003	-0,055601

Cloud_Mesh_Distance_BA_R0_R3	18,73894882	-76,0838089	-943,6403809	-0,042443
Cloud_Mesh_Distance_BA_R0_R3	18,9054203	-76,12346649	-943,802063	-0,036472
Cloud_Mesh_Distance_BA_R0_R3	17,64027786	-83,60785675	-942,9815674	-0,126926
Cloud_Mesh_Distance_BA_R0_R3	17,53388214	-83,51931	-943,0371094	-0,09117
Cloud_Mesh_Distance_BA_R0_R3	17,38194656	-83,38404083	-943,1192627	-0,078352
Cloud_Mesh_Distance_BA_R0_R3	17,42529488	-83,41582489	-942,9353027	-0,09356
Cloud_Mesh_Distance_BA_R0_R3	17,51728058	-83,49124908	-942,8190308	-0,10105
Cloud_Mesh_Distance_BA_R0_R3	17,69978333	-83,65221405	-942,8972778	-0,190362
Cloud_Mesh_Distance_BA_R0_R3	17,28108978	-83,28368378	-943,0983887	-0,076861
Cloud_Mesh_Distance_BA_R0_R3	17,31046677	-83,30432892	-942,9386597	-0,091332
Cloud_Mesh_Distance_BA_R0_R3	17,03744316	-83,00452423	-942,9454346	-0,090276
Cloud_Mesh_Distance_BA_R0_R3	17,00795937	-82,98535919	-943,1221314	-0,072022
Cloud_Mesh_Distance_BA_R0_R3	17,2059021	-83,20929718	-943,1668091	-0,069904
Cloud_Mesh_Distance_BA_R0_R3	17,22326088	-83,21866608	-943,0205078	-0,083724
Cloud_Mesh_Distance_BA_R0_R3	17,12523651	-83,11203766	-943,0379639	-0,082732
Cloud_Mesh_Distance_BA_R0_R3	17,19765663	-83,18038177	-942,8587036	-0,093278
Cloud_Mesh_Distance_BA_R0_R3	16,94251633	-82,89322662	-942,9421997	-0,087106
Cloud_Mesh_Distance_BA_R0_R3	16,86067963	-82,80660248	-943,0250855	-0,076117
Cloud_Mesh_Distance_BA_R0_R3	16,83972931	-82,76464081	-942,8615112	-0,083033
Cloud_Mesh_Distance_BA_R0_R3	16,74216843	-82,65094757	-942,8643189	-0,071975
Cloud_Mesh_Distance_BA_R0_R3	16,65598297	-82,56737518	-942,9689331	-0,052717
Cloud_Mesh_Distance_BA_R0_R3	16,40547943	-82,24999237	-942,8647461	-0,015186
Cloud_Mesh_Distance_BA_R0_R3	16,15758896	-81,94844818	-942,9950562	0,020568
Cloud_Mesh_Distance_BA_R0_R3	16,21795654	-82,0611496	-943,0748901	0,022047
Cloud_Mesh_Distance_BA_R0_R3	23,19882584	-82,51953888	-943,2436523	0,136875
Cloud_Mesh_Distance_BA_R0_R3	23,25023079	-82,63622284	-943,1560059	0,138827
Cloud_Mesh_Distance_BA_R0_R3	23,23185158	-82,69431305	-943,3153076	0,114239
Cloud_Mesh_Distance_BA_R0_R3	16,0251255	-81,72180939	-942,9563599	0,015965
Cloud_Mesh_Distance_BA_R0_R3	16,07138824	-81,83971405	-943,0718994	0,019507
Cloud_Mesh_Distance_BA_R0_R3	16,11153412	-81,82584381	-942,850769	0,021654
Cloud_Mesh_Distance_BA_R0_R3	15,89801598	-81,56130219	-943,1220703	0,002135
Cloud_Mesh_Distance_BA_R0_R3	15,86889744	-81,4701004	-943,0205689	-0,006166
Cloud_Mesh_Distance_BA_R0_R3	23,11626053	-82,31214142	-943,3688965	0,107906
Cloud_Mesh_Distance_BA_R0_R3	23,10013771	-82,08644867	-943,1465454	0,08391
Cloud_Mesh_Distance_BA_R0_R3	23,07797813	-82,10651398	-943,2925415	0,075879
Cloud_Mesh_Distance_BA_R0_R3	23,12294006	-82,23149872	-943,2265015	0,105625
Cloud_Mesh_Distance_BA_R0_R3	15,70420742	-81,19956207	-943,0765381	-0,02595
Cloud_Mesh_Distance_BA_R0_R3	15,5924654	-80,96263885	-943,0005493	-0,030126
Cloud_Mesh_Distance_BA_R0_R3	15,5413866	-80,90035248	-943,0507813	-0,036903
Cloud_Mesh_Distance_BA_R0_R3	15,55100918	-80,96025848	-943,1282349	-0,036748
Cloud_Mesh_Distance_BA_R0_R3	15,38861847	-80,60234833	-943,059082	-0,046148
Cloud_Mesh_Distance_BA_R0_R3	15,40696335	-80,69788361	-943,1361084	-0,041374
Cloud_Mesh_Distance_BA_R0_R3	22,99869537	-81,49744415	-943,1845093	-0,047786
Cloud_Mesh_Distance_BA_R0_R3	15,40843391	-80,55377197	-942,9533081	-0,040014
Cloud_Mesh_Distance_BA_R0_R3	22,95248032	-81,37953949	-943,3353882	-0,068198
Cloud_Mesh_Distance_BA_R0_R3	22,89910698	-81,38982391	-943,510376	-0,067732
Cloud_Mesh_Distance_BA_R0_R3	22,93125534	-81,21041107	-943,3786621	-0,08347
Cloud_Mesh_Distance_BA_R0_R3	22,97825623	-81,30957794	-943,2306519	-0,074741
Cloud_Mesh_Distance_BA_R0_R3	15,34483433	-80,46278381	-943,0346069	-0,050387
Cloud_Mesh_Distance_BA_R0_R3	15,31677914	-80,32334137	-943,0015259	-0,062459

Cloud_Mesh_Distance_BA_R0_R3	15,23501015	-80,16181946	-943,1427612	-0,088689
Cloud_Mesh_Distance_BA_R0_R3	15,29625034	-80,15679932	-942,9636231	-0,082078
Cloud_Mesh_Distance_BA_R0_R3	15,23459721	-80,04140472	-943,085022	-0,096775
Cloud_Mesh_Distance_BA_R0_R3	22,81586456	-80,58820343	-943,5852661	-0,118978
Cloud_Mesh_Distance_BA_R0_R3	22,87232971	-80,66350555	-943,4764404	-0,114515
Cloud_Mesh_Distance_BA_R0_R3	15,15666771	-79,86145782	-943,2892456	-0,138531
Cloud_Mesh_Distance_BA_R0_R3	15,20839596	-79,8647995	-943,1086426	-0,122679
Cloud_Mesh_Distance_BA_R0_R3	15,15890217	-79,70761871	-943,2371216	-0,147513
Cloud_Mesh_Distance_BA_R0_R3	22,87269211	-80,50868988	-943,4422607	-0,113071
Cloud_Mesh_Distance_BA_R0_R3	22,86782074	-80,34299469	-943,4015503	-0,117098
Cloud_Mesh_Distance_BA_R0_R3	22,91766548	-80,4387207	-943,3168945	-0,109015
Cloud_Mesh_Distance_BA_R0_R3	15,21860218	-79,63143158	-943,0370483	-0,136823
Cloud_Mesh_Distance_BA_R0_R3	22,8094635	-80,40348053	-943,5482788	-0,124267
Cloud_Mesh_Distance_BA_R0_R3	22,8127327	-80,23239899	-943,4758911	-0,123126
Cloud_Mesh_Distance_BA_R0_R3	22,86362457	-80,21839142	-943,3580933	-0,117381
Cloud_Mesh_Distance_BA_R0_R3	15,20989227	-79,30940247	-943,0523682	-0,146687
Cloud_Mesh_Distance_BA_R0_R3	22,71659088	-79,93460846	-943,4978638	-0,123995
Cloud_Mesh_Distance_BA_R0_R3	22,78897667	-79,9484787	-943,3570557	-0,118272
Cloud_Mesh_Distance_BA_R0_R3	15,21941376	-79,12346649	-943,0477295	-0,144058
Cloud_Mesh_Distance_BA_R0_R3	15,2428503	-78,931633	-943,0335693	-0,139987
Cloud_Mesh_Distance_BA_R0_R3	22,71594238	-79,82524109	-943,4156494	-0,118633
Cloud_Mesh_Distance_BA_R0_R3	22,57201958	-79,67301178	-943,5727539	-0,120074
Cloud_Mesh_Distance_BA_R0_R3	22,65302658	-79,64765167	-943,3887329	-0,112832
Cloud_Mesh_Distance_BA_R0_R3	15,17270088	-78,96022797	-943,2186279	-0,150142
Cloud_Mesh_Distance_BA_R0_R3	22,53249741	-79,53665924	-943,5272827	-0,108476
Cloud_Mesh_Distance_BA_R0_R3	15,26576996	-78,74131012	-943,0513916	-0,136843
Cloud_Mesh_Distance_BA_R0_R3	22,45815849	-79,43038177	-943,5679932	-0,10501
Cloud_Mesh_Distance_BA_R0_R3	15,29422092	-78,54549408	-943,0875855	-0,136289
Cloud_Mesh_Distance_BA_R0_R3	15,3835659	-78,20749664	-943,1207886	-0,126246
Cloud_Mesh_Distance_BA_R0_R3	22,24282074	-78,86864471	-943,387085	-0,019663
Cloud_Mesh_Distance_BA_R0_R3	15,40928841	-78,0684967	-943,1905518	-0,122571
Cloud_Mesh_Distance_BA_R0_R3	15,48116302	-77,96720886	-943,0988159	-0,116538
Cloud_Mesh_Distance_BA_R0_R3	22,02421188	-78,6186142	-943,4865112	-0,001759
Cloud_Mesh_Distance_BA_R0_R3	22,17660141	-78,81472778	-943,4516602	-0,0146
Cloud_Mesh_Distance_BA_R0_R3	15,43816757	-77,92842865	-943,2762451	-0,121714
Cloud_Mesh_Distance_BA_R0_R3	21,85474396	-78,3300705	-943,3800049	0,013899
Cloud_Mesh_Distance_BA_R0_R3	21,77420044	-78,35695648	-943,5899658	0,012291
Cloud_Mesh_Distance_BA_R0_R3	21,9272728	-78,49215698	-943,4909058	0,005064
Cloud_Mesh_Distance_BA_R0_R3	15,56312752	-77,76248932	-943,1080933	-0,098808
Cloud_Mesh_Distance_BA_R0_R3	21,69329453	-78,23538208	-943,5712891	0,016151
Cloud_Mesh_Distance_BA_R0_R3	15,64958	-77,57494354	-943,107544	-0,086383
Cloud_Mesh_Distance_BA_R0_R3	21,46264648	-77,97083282	-943,6452026	0,029605
Cloud_Mesh_Distance_BA_R0_R3	21,57969666	-78,05124664	-943,5180664	0,024812
Cloud_Mesh_Distance_BA_R0_R3	21,62014198	-78,16919708	-943,6203613	0,018504
Cloud_Mesh_Distance_BA_R0_R3	21,6818161	-78,10710907	-943,3716431	0,020821
Cloud_Mesh_Distance_BA_R0_R3	21,66419411	-78,15591431	-943,5064087	0,019086
Cloud_Mesh_Distance_BA_R0_R3	15,70749569	-77,4356308	-943,1495972	-0,075379
Cloud_Mesh_Distance_BA_R0_R3	15,81854057	-77,13796997	-943,4046631	-0,071176
Cloud_Mesh_Distance_BA_R0_R3	21,48620224	-77,89212036	-943,4437256	0,031647
Cloud_Mesh_Distance_BA_R0_R3	15,93576908	-77,00461578	-943,3490601	-0,05702

Cloud_Mesh_Distance_BA_R0_R3	15,87898922	-77,09430695	-943,2971802	-0,056791
Cloud_Mesh_Distance_BA_R0_R3	15,96383095	-77,03721619	-943,1717529	-0,043835
Cloud_Mesh_Distance_BA_R0_R3	21,21761894	-77,59712219	-943,4973755	0,027467
Cloud_Mesh_Distance_BA_R0_R3	21,38097763	-77,83582306	-943,5969849	0,03368
Cloud_Mesh_Distance_BA_R0_R3	21,32482147	-77,67176056	-943,3793335	0,030872
Cloud_Mesh_Distance_BA_R0_R3	16,03412819	-76,89427185	-943,3452759	-0,0487
Cloud_Mesh_Distance_BA_R0_R3	21,09527206	-77,52140045	-943,6313477	0,024764
Cloud_Mesh_Distance_BA_R0_R3	21,00014877	-77,39300537	-943,56073	0,015883
Cloud_Mesh_Distance_BA_R0_R3	21,12657928	-77,46263885	-943,3911133	0,017065
Cloud_Mesh_Distance_BA_R0_R3	16,17513657	-76,78517914	-943,2338867	-0,025706
Cloud_Mesh_Distance_BA_R0_R3	16,26716995	-76,67601776	-943,3260498	-0,028663
Cloud_Mesh_Distance_BA_R0_R3	20,69024277	-77,09531403	-943,617981	-0,004664
Cloud_Mesh_Distance_BA_R0_R3	20,91988373	-77,28582001	-943,4882813	-0,00018
Cloud_Mesh_Distance_BA_R0_R3	20,88137817	-77,3116684	-943,6669312	0,004866
Cloud_Mesh_Distance_BA_R0_R3	16,34018707	-76,56929016	-943,5429688	-0,043487
Cloud_Mesh_Distance_BA_R0_R3	16,33912277	-76,59925079	-943,3984985	-0,032968
Cloud_Mesh_Distance_BA_R0_R3	20,52304268	-76,90911865	-943,5540161	-0,014733
Cloud_Mesh_Distance_BA_R0_R3	20,44668198	-76,82654572	-943,5095825	-0,017836
Cloud_Mesh_Distance_BA_R0_R3	20,5177269	-76,87372589	-943,4193726	-0,017593
Cloud_Mesh_Distance_BA_R0_R3	20,62417793	-76,96800995	-943,3894653	-0,021418
Cloud_Mesh_Distance_BA_R0_R3	20,72456551	-77,08568573	-943,4686279	-0,010779
Cloud_Mesh_Distance_BA_R0_R3	16,6079216	-76,447052	-943,2646484	-0,009892
Cloud_Mesh_Distance_BA_R0_R3	16,58248901	-76,41508484	-943,4619751	-0,025392
Cloud_Mesh_Distance_BA_R0_R3	16,45612717	-76,51390839	-943,3919067	-0,025672
Cloud_Mesh_Distance_BA_R0_R3	16,71965408	-76,3664856	-943,3268433	-0,010885
Cloud_Mesh_Distance_BA_R0_R3	16,82149506	-76,29595947	-943,4094849	-0,013196
Cloud_Mesh_Distance_BA_R0_R3	20,33983612	-76,73412323	-943,5203247	-0,017721
Cloud_Mesh_Distance_BA_R0_R3	20,21831703	-76,65341949	-943,6164551	-0,011303
Cloud_Mesh_Distance_BA_R0_R3	16,87468338	-76,31789398	-943,2159424	-0,000255
Cloud_Mesh_Distance_BA_R0_R3	17,10887527	-76,189888	-943,3649292	-0,006305
Cloud_Mesh_Distance_BA_R0_R3	17,41489601	-76,11975861	-943,2909546	-0,004449
Cloud_Mesh_Distance_BA_R0_R3	17,56468391	-76,07007599	-943,536438	-0,026984
Cloud_Mesh_Distance_BA_R0_R3	17,97123718	-76,04808807	-943,3535156	-0,031679
Cloud_Mesh_Distance_BA_R0_R3	17,92123795	-76,04364777	-943,5162964	-0,036801
Cloud_Mesh_Distance_BA_R0_R3	18,23093796	-76,04190826	-943,4283447	-0,042907
Cloud_Mesh_Distance_BA_R0_R3	18,42643547	-76,05593109	-943,2470703	-0,034862
Cloud_Mesh_Distance_BA_R0_R3	19,04919815	-76,13113403	-943,49823	-0,042377
Cloud_Mesh_Distance_BA_R0_R3	19,08316612	-76,14463043	-943,6202393	-0,039647
Cloud_Mesh_Distance_BA_R0_R3	19,2250824	-76,17411041	-943,4936523	-0,036973
Cloud_Mesh_Distance_BA_R0_R3	19,26025391	-76,17897797	-943,2836304	-0,034152
Cloud_Mesh_Distance_BA_R0_R3	19,5386734	-76,26746368	-943,3864136	-0,036166
Cloud_Mesh_Distance_BA_R0_R3	19,40538216	-76,22808075	-943,496521	-0,038162
Cloud_Mesh_Distance_BA_R0_R3	19,39213943	-76,21704865	-943,3347168	-0,03608
Cloud_Mesh_Distance_BA_R0_R3	19,65231323	-76,31484985	-943,4538574	-0,028564
Cloud_Mesh_Distance_BA_R0_R3	19,52486038	-76,27501678	-943,5681763	-0,02796
Cloud_Mesh_Distance_BA_R0_R3	19,9219017	-76,46630096	-943,6630249	-0,018552
Cloud_Mesh_Distance_BA_R0_R3	19,94615746	-76,4585495	-943,5064087	-0,020398
Cloud_Mesh_Distance_BA_R0_R3	18,06395721	-76,0406723	-943,4642334	-0,036583
Cloud_Mesh_Distance_BA_R0_R3	18,35081482	-76,04683685	-943,5458374	-0,045313
Cloud_Mesh_Distance_BA_R0_R3	18,36932945	-76,04750824	-943,3758545	-0,041665

Cloud_Mesh_Distance_BA_R0_R3	18,53907394	-76,0581665	-943,3705444	-0,044082
Cloud_Mesh_Distance_BA_R0_R3	18,57482338	-76,06337738	-943,5880737	-0,045863
Cloud_Mesh_Distance_BA_R0_R3	18,76127625	-76,08058929	-943,449646	-0,04359
Cloud_Mesh_Distance_BA_R0_R3	18,91646957	-76,11011505	-943,6235352	-0,041806
Cloud_Mesh_Distance_BA_R0_R3	18,94124413	-76,10848236	-943,4317627	-0,044644
Cloud_Mesh_Distance_BA_R0_R3	17,72117424	-83,65906525	-942,7591553	-0,144667
Cloud_Mesh_Distance_BA_R0_R3	17,62144852	-83,58475494	-942,8652954	-0,116768
Cloud_Mesh_Distance_BA_R0_R3	17,09930611	-83,06987762	-942,8583374	-0,09361
Cloud_Mesh_Distance_BA_R0_R3	17,12774277	-83,08899689	-942,6776733	-0,100149
Cloud_Mesh_Distance_BA_R0_R3	17,01304626	-82,96225739	-942,770874	-0,094696
Cloud_Mesh_Distance_BA_R0_R3	17,22480774	-83,19826508	-942,6970825	-0,096394
Cloud_Mesh_Distance_BA_R0_R3	17,28439522	-83,26680756	-942,7759399	-0,094898
Cloud_Mesh_Distance_BA_R0_R3	16,86846161	-82,78179169	-942,7000122	-0,089885
Cloud_Mesh_Distance_BA_R0_R3	16,93972397	-82,86200714	-942,6342163	-0,094192
Cloud_Mesh_Distance_BA_R0_R3	16,92057037	-82,85269928	-942,7805786	-0,093034
Cloud_Mesh_Distance_BA_R0_R3	16,77940559	-82,67072296	-942,6777344	-0,080081
Cloud_Mesh_Distance_BA_R0_R3	16,67196274	-82,55376434	-942,7666016	-0,064741
Cloud_Mesh_Distance_BA_R0_R3	16,49723625	-82,34108734	-942,7657471	-0,030293
Cloud_Mesh_Distance_BA_R0_R3	16,57228661	-82,45349884	-942,8679199	-0,043438
Cloud_Mesh_Distance_BA_R0_R3	16,30419159	-82,14057159	-942,9463501	0,00458
Cloud_Mesh_Distance_BA_R0_R3	16,35165787	-82,15380096	-942,7702637	-0,001618
Cloud_Mesh_Distance_BA_R0_R3	16,47449493	-82,35739899	-942,9620361	-0,017062
Cloud_Mesh_Distance_BA_R0_R3	16,22751808	-82,02835846	-942,9243774	0,016829
Cloud_Mesh_Distance_BA_R0_R3	16,19132996	-81,94876862	-942,8443604	0,016224
Cloud_Mesh_Distance_BA_R0_R3	16,19455719	-81,89647675	-942,6895752	0,019403
Cloud_Mesh_Distance_BA_R0_R3	16,27030563	-82,03734589	-942,7613525	0,009645
Cloud_Mesh_Distance_BA_R0_R3	23,23601532	-82,53461456	-943,0752564	0,162949
Cloud_Mesh_Distance_BA_R0_R3	23,18995094	-82,37111664	-943,0650024	0,153125
Cloud_Mesh_Distance_BA_R0_R3	23,29436493	-82,65557098	-942,9765625	0,167827
Cloud_Mesh_Distance_BA_R0_R3	15,95711613	-81,51267242	-942,7626953	0,010476
Cloud_Mesh_Distance_BA_R0_R3	15,94698524	-81,58106232	-942,9476318	0,008359
Cloud_Mesh_Distance_BA_R0_R3	23,1550293	-82,37268829	-943,2597046	0,124179
Cloud_Mesh_Distance_BA_R0_R3	23,1439209	-82,19704437	-943,0510864	0,119865
Cloud_Mesh_Distance_BA_R0_R3	15,85142994	-81,31253815	-942,7781982	-0,001421
Cloud_Mesh_Distance_BA_R0_R3	15,91924858	-81,3907547	-942,6844482	0,006886
Cloud_Mesh_Distance_BA_R0_R3	15,88848877	-81,43312836	-942,8624268	0,000613
Cloud_Mesh_Distance_BA_R0_R3	23,0538311	-81,98038483	-943,263794	0,045579
Cloud_Mesh_Distance_BA_R0_R3	23,07818222	-81,94406891	-943,088501	0,047319
Cloud_Mesh_Distance_BA_R0_R3	15,73259449	-81,1687088	-942,920166	-0,024094
Cloud_Mesh_Distance_BA_R0_R3	15,78697872	-81,17238617	-942,7645264	-0,012684
Cloud_Mesh_Distance_BA_R0_R3	15,71498013	-81,07004547	-942,8223267	-0,023329
Cloud_Mesh_Distance_BA_R0_R3	15,79697037	-81,31771851	-942,9733887	-0,014254
Cloud_Mesh_Distance_BA_R0_R3	23,03139496	-81,8225174	-943,2175293	0,0086
Cloud_Mesh_Distance_BA_R0_R3	15,66582298	-81,07038116	-942,9606934	-0,02537
Cloud_Mesh_Distance_BA_R0_R3	23,02241516	-81,68193817	-943,1566162	-0,016118
Cloud_Mesh_Distance_BA_R0_R3	15,54485226	-80,81996918	-942,9243164	-0,035223
Cloud_Mesh_Distance_BA_R0_R3	15,4543848	-80,67876434	-942,9733887	-0,035385
Cloud_Mesh_Distance_BA_R0_R3	15,52773857	-80,65306854	-942,7853394	-0,031658
Cloud_Mesh_Distance_BA_R0_R3	23,039814	-81,49927521	-942,987854	-0,048463
Cloud_Mesh_Distance_BA_R0_R3	15,4600668	-80,54046631	-942,8264771	-0,035611

Cloud_Mesh_Distance_BA_R0_R3	22,98436546	-81,1496048	-943,1998901	-0,090831
Cloud_Mesh_Distance_BA_R0_R3	23,02296448	-81,3210907	-943,048523	-0,07161
Cloud_Mesh_Distance_BA_R0_R3	15,44702244	-80,3995285	-942,7555542	-0,049138
Cloud_Mesh_Distance_BA_R0_R3	15,37022305	-80,32136536	-942,8692017	-0,057711
Cloud_Mesh_Distance_BA_R0_R3	15,40371037	-80,46056366	-942,885376	-0,044732
Cloud_Mesh_Distance_BA_R0_R3	22,99317551	-80,96141815	-943,1712647	-0,10203
Cloud_Mesh_Distance_BA_R0_R3	15,38407707	-80,14492035	-942,762085	-0,071333
Cloud_Mesh_Distance_BA_R0_R3	22,99792862	-80,77802277	-943,1535645	-0,108626
Cloud_Mesh_Distance_BA_R0_R3	22,94217491	-81,00484467	-943,3319702	-0,101559
Cloud_Mesh_Distance_BA_R0_R3	22,95541382	-80,84021759	-943,2854614	-0,105339
Cloud_Mesh_Distance_BA_R0_R3	23,03889847	-80,9123764	-943,0079956	-0,106016
Cloud_Mesh_Distance_BA_R0_R3	15,2687645	-79,95899963	-942,9630737	-0,103566
Cloud_Mesh_Distance_BA_R0_R3	15,27467823	-79,78488922	-942,9118652	-0,113811
Cloud_Mesh_Distance_BA_R0_R3	22,93984032	-80,63728333	-943,3052979	-0,110252
Cloud_Mesh_Distance_BA_R0_R3	23,00173569	-80,62065887	-943,1265869	-0,10981
Cloud_Mesh_Distance_BA_R0_R3	15,20287228	-79,45884705	-943,0686646	-0,146537
Cloud_Mesh_Distance_BA_R0_R3	15,32953453	-79,53929901	-942,7703247	-0,11932
Cloud_Mesh_Distance_BA_R0_R3	15,28484344	-79,61950684	-942,8693848	-0,12143
Cloud_Mesh_Distance_BA_R0_R3	15,26932907	-79,44068146	-942,8981934	-0,134308
Cloud_Mesh_Distance_BA_R0_R3	22,8832283	-80,15117645	-943,2761231	-0,113912
Cloud_Mesh_Distance_BA_R0_R3	22,83015251	-80,10639191	-943,3726196	-0,118366
Cloud_Mesh_Distance_BA_R0_R3	22,932024	-80,13932037	-943,1402588	-0,10285
Cloud_Mesh_Distance_BA_R0_R3	22,91867828	-80,28259277	-943,2556763	-0,11619
Cloud_Mesh_Distance_BA_R0_R3	15,26261806	-79,24906158	-942,9226074	-0,137568
Cloud_Mesh_Distance_BA_R0_R3	15,29607105	-79,08557129	-942,8703003	-0,132869
Cloud_Mesh_Distance_BA_R0_R3	22,86565399	-80,0345993	-943,2420654	-0,111784
Cloud_Mesh_Distance_BA_R0_R3	22,9050827	-79,98145294	-943,093811	-0,102267
Cloud_Mesh_Distance_BA_R0_R3	22,73210526	-79,61519623	-943,1749268	-0,096218
Cloud_Mesh_Distance_BA_R0_R3	22,7765007	-79,77683258	-943,2374268	-0,103286
Cloud_Mesh_Distance_BA_R0_R3	22,84223557	-79,911026	-943,201294	-0,105916
Cloud_Mesh_Distance_BA_R0_R3	22,86441422	-79,83582306	-943,0668335	-0,097273
Cloud_Mesh_Distance_BA_R0_R3	15,33280659	-78,68418121	-942,9187012	-0,126296
Cloud_Mesh_Distance_BA_R0_R3	22,50492096	-79,39557648	-943,4441528	-0,093424
Cloud_Mesh_Distance_BA_R0_R3	22,58122253	-79,47841644	-943,3722534	-0,094918
Cloud_Mesh_Distance_BA_R0_R3	22,6522789	-79,46920013	-943,2005005	-0,090189
Cloud_Mesh_Distance_BA_R0_R3	22,55669022	-79,3582077	-943,2942505	-0,082832
Cloud_Mesh_Distance_BA_R0_R3	22,52444839	-79,24422455	-943,2329102	-0,065831
Cloud_Mesh_Distance_BA_R0_R3	15,34383392	-78,35398865	-943,0977783	-0,126562
Cloud_Mesh_Distance_BA_R0_R3	22,39265442	-78,95793915	-943,1678467	-0,024238
Cloud_Mesh_Distance_BA_R0_R3	22,48207092	-79,0437851	-943,0640869	-0,032457
Cloud_Mesh_Distance_BA_R0_R3	22,31975555	-78,99564362	-943,3833618	-0,039973
Cloud_Mesh_Distance_BA_R0_R3	22,44625473	-79,12047577	-943,2593384	-0,052332
Cloud_Mesh_Distance_BA_R0_R3	22,47229767	-79,26763916	-943,3751831	-0,073174
Cloud_Mesh_Distance_BA_R0_R3	22,54331589	-79,17658234	-943,0960083	-0,052854
Cloud_Mesh_Distance_BA_R0_R3	22,59836388	-79,31144714	-943,1376953	-0,070205
Cloud_Mesh_Distance_BA_R0_R3	15,39754391	-78,29261017	-943,0101929	-0,12141
Cloud_Mesh_Distance_BA_R0_R3	15,43324757	-78,16869354	-943,0251465	-0,121197
Cloud_Mesh_Distance_BA_R0_R3	15,50386524	-78,14443207	-942,8770752	-0,107977
Cloud_Mesh_Distance_BA_R0_R3	22,209095	-78,76740265	-943,3210449	-0,008233
Cloud_Mesh_Distance_BA_R0_R3	22,2975235	-78,85192108	-943,2433472	-0,016449

Cloud_Mesh_Distance_BA_R0_R3	21,96789932	-78,41538239	-943,2734375	0,011576
Cloud_Mesh_Distance_BA_R0_R3	22,16453552	-78,66835785	-943,2678223	0,000763
Cloud_Mesh_Distance_BA_R0_R3	22,11529541	-78,70302582	-943,4259033	-0,004852
Cloud_Mesh_Distance_BA_R0_R3	22,06696701	-78,56139374	-943,3094482	0,009144
Cloud_Mesh_Distance_BA_R0_R3	15,69544029	-77,6112442	-942,9433594	-0,073763
Cloud_Mesh_Distance_BA_R0_R3	21,90152359	-78,31026459	-943,2263794	0,011904
Cloud_Mesh_Distance_BA_R0_R3	15,72050095	-77,49656677	-943,0239868	-0,070773
Cloud_Mesh_Distance_BA_R0_R3	15,74379349	-77,55007935	-942,902771	-0,064922
Cloud_Mesh_Distance_BA_R0_R3	21,59804344	-77,9829483	-943,3320923	0,024699
Cloud_Mesh_Distance_BA_R0_R3	21,60361099	-77,93657684	-943,1969605	0,017183
Cloud_Mesh_Distance_BA_R0_R3	21,74612808	-78,22777557	-943,4451294	0,018457
Cloud_Mesh_Distance_BA_R0_R3	15,78648376	-77,43006134	-942,9526367	-0,060283
Cloud_Mesh_Distance_BA_R0_R3	15,75453663	-77,39186096	-943,0852051	-0,065071
Cloud_Mesh_Distance_BA_R0_R3	15,75001621	-77,3250351	-943,2111816	-0,069073
Cloud_Mesh_Distance_BA_R0_R3	15,87603378	-77,15882874	-943,1591797	-0,048979
Cloud_Mesh_Distance_BA_R0_R3	15,81275749	-77,20410156	-943,2564087	-0,060341
Cloud_Mesh_Distance_BA_R0_R3	15,81849957	-77,28731537	-943,081604	-0,054492
Cloud_Mesh_Distance_BA_R0_R3	21,53611374	-77,88385773	-943,2764893	0,021935
Cloud_Mesh_Distance_BA_R0_R3	21,54670715	-77,84698486	-943,1321411	0,018031
Cloud_Mesh_Distance_BA_R0_R3	21,44345093	-77,77174377	-943,274353	0,02236
Cloud_Mesh_Distance_BA_R0_R3	16,04348373	-77,04894257	-942,9520264	-0,019565
Cloud_Mesh_Distance_BA_R0_R3	21,19701958	-77,4980545	-943,2702026	0,016965
Cloud_Mesh_Distance_BA_R0_R3	21,24575043	-77,52799988	-943,1984863	0,014102
Cloud_Mesh_Distance_BA_R0_R3	21,24373817	-77,56213379	-943,3190918	0,020628
Cloud_Mesh_Distance_BA_R0_R3	21,3888092	-77,77471161	-943,4506836	0,033278
Cloud_Mesh_Distance_BA_R0_R3	21,34154129	-77,62494659	-943,1763916	0,016693
Cloud_Mesh_Distance_BA_R0_R3	16,09454727	-76,91222382	-943,1080322	-0,023581
Cloud_Mesh_Distance_BA_R0_R3	21,01441574	-77,35494232	-943,4093628	0,006046
Cloud_Mesh_Distance_BA_R0_R3	16,16267395	-76,93403625	-942,9116821	-0,002541
Cloud_Mesh_Distance_BA_R0_R3	16,22566223	-76,81873322	-943,0187988	-0,004412
Cloud_Mesh_Distance_BA_R0_R3	16,31166267	-76,70360565	-943,1182251	-0,010556
Cloud_Mesh_Distance_BA_R0_R3	16,38808632	-76,60222626	-943,2429199	-0,015222
Cloud_Mesh_Distance_BA_R0_R3	20,85706711	-77,18566132	-943,3588867	-0,004311
Cloud_Mesh_Distance_BA_R0_R3	20,75575638	-77,06735992	-943,2736816	-0,017521
Cloud_Mesh_Distance_BA_R0_R3	20,96372986	-77,28485107	-943,3428345	-0,000781
Cloud_Mesh_Distance_BA_R0_R3	20,96478271	-77,24701691	-943,1922607	-0,006106
Cloud_Mesh_Distance_BA_R0_R3	21,05432701	-77,35172272	-943,2628784	0,001387
Cloud_Mesh_Distance_BA_R0_R3	16,63266754	-76,47653961	-943,1196289	-0,000834
Cloud_Mesh_Distance_BA_R0_R3	20,54467392	-76,87033844	-943,2696533	-0,016931
Cloud_Mesh_Distance_BA_R0_R3	16,51959991	-76,52765656	-943,1807861	-0,004057
Cloud_Mesh_Distance_BA_R0_R3	16,77640152	-76,40903473	-943,0804443	0,005939
Cloud_Mesh_Distance_BA_R0_R3	20,22109413	-76,62824249	-943,4474487	-0,019885
Cloud_Mesh_Distance_BA_R0_R3	20,34201622	-76,70471191	-943,3234253	-0,01944
Cloud_Mesh_Distance_BA_R0_R3	20,44168663	-76,7964859	-943,3738403	-0,016064
Cloud_Mesh_Distance_BA_R0_R3	20,34379196	-76,68520355	-943,1482544	-0,026171
Cloud_Mesh_Distance_BA_R0_R3	16,94120598	-76,33588409	-943,0568237	0,007634
Cloud_Mesh_Distance_BA_R0_R3	16,98612022	-76,24951935	-943,2954712	-0,004412
Cloud_Mesh_Distance_BA_R0_R3	17,35522079	-76,17760468	-943,0794067	0,00491
Cloud_Mesh_Distance_BA_R0_R3	17,18910408	-76,21422577	-943,1366577	0,005555
Cloud_Mesh_Distance_BA_R0_R3	19,94701958	-76,44260406	-943,3369751	-0,026994

Cloud_Mesh_Distance_BA_R0_R3	20,00596619	-76,46192169	-943,1497803	-0,02157
Cloud_Mesh_Distance_BA_R0_R3	20,08969498	-76,52687836	-943,3490601	-0,025015
Cloud_Mesh_Distance_BA_R0_R3	17,28953552	-76,15990448	-943,2365112	-0,001727
Cloud_Mesh_Distance_BA_R0_R3	17,45626831	-76,14467621	-943,1091919	0,002656
Cloud_Mesh_Distance_BA_R0_R3	17,77674294	-76,08507538	-943,1392212	-0,009144
Cloud_Mesh_Distance_BA_R0_R3	17,57658768	-76,08660126	-943,3255615	-0,01721
Cloud_Mesh_Distance_BA_R0_R3	17,76754189	-76,06247711	-943,3412476	-0,025426
Cloud_Mesh_Distance_BA_R0_R3	18,17193604	-76,04588318	-943,3122559	-0,034493
Cloud_Mesh_Distance_BA_R0_R3	18,30180359	-76,04781342	-943,3058472	-0,039842
Cloud_Mesh_Distance_BA_R0_R3	19,08843994	-76,13796234	-943,314209	-0,041138
Cloud_Mesh_Distance_BA_R0_R3	19,38590813	-76,21370697	-943,147644	-0,030381
Cloud_Mesh_Distance_BA_R0_R3	19,86795425	-76,39646149	-943,2529297	-0,025453
Cloud_Mesh_Distance_BA_R0_R3	19,80552292	-76,38006592	-943,4360352	-0,028611
Cloud_Mesh_Distance_BA_R0_R3	18,46512985	-76,07067108	-943,0988159	-0,031127
Cloud_Mesh_Distance_BA_R0_R3	18,57201004	-76,07054901	-943,1732178	-0,0325
Cloud_Mesh_Distance_BA_R0_R3	18,07110977	-83,91397858	-942,4469605	-0,096195
Cloud_Mesh_Distance_BA_R0_R3	17,83991241	-83,74581146	-942,6557007	-0,110211
Cloud_Mesh_Distance_BA_R0_R3	17,74108505	-83,65796661	-942,5722046	-0,110038
Cloud_Mesh_Distance_BA_R0_R3	17,39360809	-83,3727951	-942,7515259	-0,097931
Cloud_Mesh_Distance_BA_R0_R3	17,51711273	-83,4744339	-942,6224976	-0,103755
Cloud_Mesh_Distance_BA_R0_R3	17,62436295	-83,57285309	-942,6906738	-0,107281
Cloud_Mesh_Distance_BA_R0_R3	17,63637924	-83,56456757	-942,5009155	-0,106302
Cloud_Mesh_Distance_BA_R0_R3	17,30622673	-83,27700043	-942,6254883	-0,098939
Cloud_Mesh_Distance_BA_R0_R3	17,41334915	-83,37241364	-942,5355835	-0,101824
Cloud_Mesh_Distance_BA_R0_R3	17,12665367	-83,06726837	-942,4543457	-0,092685
Cloud_Mesh_Distance_BA_R0_R3	16,94810486	-82,85231781	-942,4515381	-0,087781
Cloud_Mesh_Distance_BA_R0_R3	16,86406708	-82,75862885	-942,5457153	-0,084333
Cloud_Mesh_Distance_BA_R0_R3	16,59175873	-82,43663788	-942,6616211	-0,049217
Cloud_Mesh_Distance_BA_R0_R3	16,68889999	-82,5391922	-942,5591431	-0,064307
Cloud_Mesh_Distance_BA_R0_R3	16,4264946	-82,22690582	-942,6748657	-0,015895
Cloud_Mesh_Distance_BA_R0_R3	16,51444054	-82,31351471	-942,5630493	-0,029515
Cloud_Mesh_Distance_BA_R0_R3	16,13220406	-81,70368195	-942,5010986	0,026869
Cloud_Mesh_Distance_BA_R0_R3	16,20322037	-81,82144928	-942,4857788	0,021164
Cloud_Mesh_Distance_BA_R0_R3	16,12330246	-81,75930023	-942,6447144	0,020591
Cloud_Mesh_Distance_BA_R0_R3	23,2468853	-82,49626923	-942,9520264	0,182395
Cloud_Mesh_Distance_BA_R0_R3	23,28583336	-82,5280838	-942,7889404	0,214998
Cloud_Mesh_Distance_BA_R0_R3	16,09508514	-81,59622955	-942,4403687	0,032416
Cloud_Mesh_Distance_BA_R0_R3	16,00833321	-81,49576569	-942,5608521	0,024457
Cloud_Mesh_Distance_BA_R0_R3	16,07674789	-81,64340973	-942,5811768	0,025213
Cloud_Mesh_Distance_BA_R0_R3	16,03270721	-81,65125275	-942,7575684	0,016045
Cloud_Mesh_Distance_BA_R0_R3	23,22925186	-82,3973465	-942,8691406	0,186414
Cloud_Mesh_Distance_BA_R0_R3	23,24173546	-82,36371613	-942,694519	0,208314
Cloud_Mesh_Distance_BA_R0_R3	15,94637871	-81,3405304	-942,532959	0,013065
Cloud_Mesh_Distance_BA_R0_R3	23,12462997	-82,07106781	-942,9693604	0,095866
Cloud_Mesh_Distance_BA_R0_R3	23,10103989	-81,9197464	-942,9078369	0,054107
Cloud_Mesh_Distance_BA_R0_R3	23,1817894	-82,23322296	-942,8577881	0,149715
Cloud_Mesh_Distance_BA_R0_R3	15,87537384	-81,23699951	-942,6036377	-0,00089
Cloud_Mesh_Distance_BA_R0_R3	23,05974197	-81,79387665	-943,0389404	0,016177
Cloud_Mesh_Distance_BA_R0_R3	15,74329376	-81,01131439	-942,6810913	-0,018748
Cloud_Mesh_Distance_BA_R0_R3	15,81657505	-81,06945801	-942,5682983	-0,00923

Cloud_Mesh_Distance_BA_R0_R3	23,08273697	-81,76413727	-942,8624268	0,010408
Cloud_Mesh_Distance_BA_R0_R3	23,04549789	-81,63895416	-943,0116577	-0,017475
Cloud_Mesh_Distance_BA_R0_R3	15,59703732	-80,75126648	-942,7357788	-0,033282
Cloud_Mesh_Distance_BA_R0_R3	15,59311867	-80,63311768	-942,6459351	-0,037729
Cloud_Mesh_Distance_BA_R0_R3	15,65279579	-80,91549683	-942,7832642	-0,031148
Cloud_Mesh_Distance_BA_R0_R3	23,06251907	-81,44274139	-942,8504639	-0,056789
Cloud_Mesh_Distance_BA_R0_R3	15,5446167	-80,46923065	-942,6235352	-0,048336
Cloud_Mesh_Distance_BA_R0_R3	23,06322479	-81,26025391	-942,8516235	-0,084545
Cloud_Mesh_Distance_BA_R0_R3	23,03070068	-81,10739899	-943,0241699	-0,09609
Cloud_Mesh_Distance_BA_R0_R3	15,44601822	-80,15293121	-942,6513672	-0,065751
Cloud_Mesh_Distance_BA_R0_R3	15,43438339	-80,26275635	-942,7114258	-0,059796
Cloud_Mesh_Distance_BA_R0_R3	15,35091877	-79,9263916	-942,7723999	-0,093977
Cloud_Mesh_Distance_BA_R0_R3	15,43584442	-80,0246048	-942,6360474	-0,078611
Cloud_Mesh_Distance_BA_R0_R3	15,4388113	-79,8824234	-942,6053467	-0,087646
Cloud_Mesh_Distance_BA_R0_R3	23,0802269	-80,86893463	-942,8312988	-0,114246
Cloud_Mesh_Distance_BA_R0_R3	15,48672962	-79,78629303	-942,5171509	-0,091546
Cloud_Mesh_Distance_BA_R0_R3	22,97705269	-80,4458847	-943,1591187	-0,111632
Cloud_Mesh_Distance_BA_R0_R3	23,0249424	-80,35902405	-942,9724731	-0,110802
Cloud_Mesh_Distance_BA_R0_R3	23,0372963	-80,54416656	-942,9923706	-0,111029
Cloud_Mesh_Distance_BA_R0_R3	23,04364395	-80,72559357	-942,9933472	-0,111371
Cloud_Mesh_Distance_BA_R0_R3	15,34228611	-79,68941498	-942,7548218	-0,107514
Cloud_Mesh_Distance_BA_R0_R3	22,9895668	-80,20824432	-943,0097656	-0,105951
Cloud_Mesh_Distance_BA_R0_R3	15,343853	-79,42493439	-942,7406616	-0,116639
Cloud_Mesh_Distance_BA_R0_R3	22,97079849	-80,28090668	-943,1113281	-0,110261
Cloud_Mesh_Distance_BA_R0_R3	15,35928059	-79,14122772	-942,7305298	-0,118523
Cloud_Mesh_Distance_BA_R0_R3	15,32741833	-79,2766037	-942,7791138	-0,12214
Cloud_Mesh_Distance_BA_R0_R3	15,36958408	-78,9888382	-942,7394409	-0,12134
Cloud_Mesh_Distance_BA_R0_R3	22,93855286	-79,92151642	-942,9257202	-0,102068
Cloud_Mesh_Distance_BA_R0_R3	15,3121376	-78,87361145	-942,8890381	-0,13181
Cloud_Mesh_Distance_BA_R0_R3	22,81271362	-79,68370819	-943,039856	-0,093638
Cloud_Mesh_Distance_BA_R0_R3	22,89362335	-79,76529694	-942,8881226	-0,08609
Cloud_Mesh_Distance_BA_R0_R3	15,44562531	-78,71339417	-942,6776733	-0,113036
Cloud_Mesh_Distance_BA_R0_R3	15,38393211	-78,80043793	-942,7650757	-0,11922
Cloud_Mesh_Distance_BA_R0_R3	22,67379379	-79,37942505	-943,0285645	-0,069951
Cloud_Mesh_Distance_BA_R0_R3	15,47028637	-78,52484894	-942,7094116	-0,107688
Cloud_Mesh_Distance_BA_R0_R3	15,39823723	-78,62160492	-942,807312	-0,117385
Cloud_Mesh_Distance_BA_R0_R3	15,35928726	-78,51461792	-942,9477539	-0,126071
Cloud_Mesh_Distance_BA_R0_R3	15,38994312	-78,39614105	-942,9524536	-0,123488
Cloud_Mesh_Distance_BA_R0_R3	15,41082954	-78,48003387	-942,8538208	-0,118564
Cloud_Mesh_Distance_BA_R0_R3	15,44393826	-78,33386993	-942,8718872	-0,119641
Cloud_Mesh_Distance_BA_R0_R3	22,49354172	-78,98560333	-942,9337158	-0,021107
Cloud_Mesh_Distance_BA_R0_R3	15,5286417	-78,32401276	-942,703125	-0,100503
Cloud_Mesh_Distance_BA_R0_R3	22,43613434	-78,93690491	-943,019165	-0,014928
Cloud_Mesh_Distance_BA_R0_R3	22,35717773	-78,82437897	-943,0452271	-0,004156
Cloud_Mesh_Distance_BA_R0_R3	22,24922943	-78,72818756	-943,1641235	-0,000547
Cloud_Mesh_Distance_BA_R0_R3	22,27855301	-78,67977905	-942,9874878	0,006319
Cloud_Mesh_Distance_BA_R0_R3	15,56747532	-77,94658661	-942,8996582	-0,09939
Cloud_Mesh_Distance_BA_R0_R3	15,64414597	-77,75791168	-942,9031982	-0,084281
Cloud_Mesh_Distance_BA_R0_R3	15,69082546	-77,81015778	-942,7532349	-0,073618
Cloud_Mesh_Distance_BA_R0_R3	21,97294235	-78,35686493	-943,1370239	0,010705

Cloud_Mesh_Distance_BA_R0_R3	22,07479858	-78,4631424	-943,0955811	0,00709
Cloud_Mesh_Distance_BA_R0_R3	22,19667435	-78,55194855	-942,9431152	0,011032
Cloud_Mesh_Distance_BA_R0_R3	22,17432785	-78,60467529	-943,1275635	0,006474
Cloud_Mesh_Distance_BA_R0_R3	15,73286819	-77,68388367	-942,7834473	-0,0666
Cloud_Mesh_Distance_BA_R0_R3	15,79400158	-77,53578949	-942,8118897	-0,057386
Cloud_Mesh_Distance_BA_R0_R3	21,90825462	-78,2614212	-943,0904541	0,011197
Cloud_Mesh_Distance_BA_R0_R3	21,80256653	-78,19864655	-943,2459717	0,01189
Cloud_Mesh_Distance_BA_R0_R3	22,01057053	-78,34435272	-942,9900513	0,012426
Cloud_Mesh_Distance_BA_R0_R3	21,81985664	-78,13214874	-943,0170288	0,012695
Cloud_Mesh_Distance_BA_R0_R3	21,69654465	-78,04202271	-943,1774902	0,014247
Cloud_Mesh_Distance_BA_R0_R3	15,85345364	-77,33850861	-942,9246826	-0,046378
Cloud_Mesh_Distance_BA_R0_R3	15,92130089	-77,19783783	-942,9769287	-0,03657
Cloud_Mesh_Distance_BA_R0_R3	16,13793945	-77,06122589	-942,7545776	0,002
Cloud_Mesh_Distance_BA_R0_R3	16,24568176	-76,9471283	-942,7470703	0,008555
Cloud_Mesh_Distance_BA_R0_R3	21,16804123	-77,44114685	-943,1723023	0,008947
Cloud_Mesh_Distance_BA_R0_R3	16,26374817	-76,85865021	-942,8643799	0,008279
Cloud_Mesh_Distance_BA_R0_R3	16,47101021	-76,61912537	-943,019043	0,008121
Cloud_Mesh_Distance_BA_R0_R3	16,3865242	-76,76050568	-942,8493652	0,015725
Cloud_Mesh_Distance_BA_R0_R3	20,86379814	-77,14279938	-943,1535645	-0,015481
Cloud_Mesh_Distance_BA_R0_R3	21,07562065	-77,32486725	-943,0692749	-0,005448
Cloud_Mesh_Distance_BA_R0_R3	16,53149414	-76,66454315	-942,8234863	0,017219
Cloud_Mesh_Distance_BA_R0_R3	16,59960938	-76,53113556	-943,0236206	0,008766
Cloud_Mesh_Distance_BA_R0_R3	16,6877594	-76,47816467	-943,0241089	0,009758
Cloud_Mesh_Distance_BA_R0_R3	20,67096138	-76,96419525	-943,1519775	-0,01884
Cloud_Mesh_Distance_BA_R0_R3	20,55447388	-76,84933472	-943,0765991	-0,020591
Cloud_Mesh_Distance_BA_R0_R3	20,78275681	-77,0590744	-943,1132813	-0,0197
Cloud_Mesh_Distance_BA_R0_R3	20,76529694	-77,01720428	-942,9647217	-0,016995
Cloud_Mesh_Distance_BA_R0_R3	16,98708534	-76,37575531	-942,8894653	0,013268
Cloud_Mesh_Distance_BA_R0_R3	17,05327988	-76,31477356	-942,9741211	0,015209
Cloud_Mesh_Distance_BA_R0_R3	16,74956894	-76,46625519	-942,965332	0,014124
Cloud_Mesh_Distance_BA_R0_R3	20,24078369	-76,61855316	-943,2498169	-0,024905
Cloud_Mesh_Distance_BA_R0_R3	20,26359558	-76,62155914	-943,1049194	-0,021467
Cloud_Mesh_Distance_BA_R0_R3	20,32284927	-76,65756989	-943,019043	-0,018796
Cloud_Mesh_Distance_BA_R0_R3	20,44570351	-76,77327728	-943,1975708	-0,018829
Cloud_Mesh_Distance_BA_R0_R3	17,12465477	-76,32577515	-942,8654785	0,015341
Cloud_Mesh_Distance_BA_R0_R3	17,19241714	-76,27144623	-942,9408569	0,015347
Cloud_Mesh_Distance_BA_R0_R3	17,03551102	-76,27953339	-943,1056519	0,009584
Cloud_Mesh_Distance_BA_R0_R3	17,53704453	-76,19036865	-942,8589478	0,014038
Cloud_Mesh_Distance_BA_R0_R3	19,97777939	-76,43898773	-942,9381714	-0,010969
Cloud_Mesh_Distance_BA_R0_R3	20,1619339	-76,55447388	-943,1316528	-0,021939
Cloud_Mesh_Distance_BA_R0_R3	20,11309814	-76,51509857	-942,963562	-0,014613
Cloud_Mesh_Distance_BA_R0_R3	17,46842575	-76,17243195	-942,9819946	0,010827
Cloud_Mesh_Distance_BA_R0_R3	17,60315323	-76,11212158	-943,1236572	-0,004067
Cloud_Mesh_Distance_BA_R0_R3	17,78894234	-76,11817932	-942,9455566	0,003055
Cloud_Mesh_Distance_BA_R0_R3	17,96528625	-76,06743622	-943,1504517	-0,018914
Cloud_Mesh_Distance_BA_R0_R3	18,15670013	-76,05941772	-943,156311	-0,023923
Cloud_Mesh_Distance_BA_R0_R3	18,31383133	-76,05710602	-943,1824341	-0,030182
Cloud_Mesh_Distance_BA_R0_R3	18,36369324	-76,08061218	-942,9929199	-0,020324
Cloud_Mesh_Distance_BA_R0_R3	18,85643005	-76,10851288	-943,083252	-0,036491
Cloud_Mesh_Distance_BA_R0_R3	19,03839493	-76,13582611	-943,1004639	-0,035193

Cloud_Mesh_Distance_BA_R0_R3	19,22038651	-76,17211151	-943,1212158	-0,032265
Cloud_Mesh_Distance_BA_R0_R3	19,3694191	-76,21474457	-942,9561157	-0,025033
Cloud_Mesh_Distance_BA_R0_R3	19,53584671	-76,26013947	-942,9943237	-0,022732
Cloud_Mesh_Distance_BA_R0_R3	19,54397964	-76,26274872	-943,194519	-0,03198
Cloud_Mesh_Distance_BA_R0_R3	19,69694138	-76,31568146	-943,0420532	-0,022206
Cloud_Mesh_Distance_BA_R0_R3	19,85300446	-76,38206482	-943,0861816	-0,024003
Cloud_Mesh_Distance_BA_R0_R3	19,71095657	-76,32733917	-943,263855	-0,027553
Cloud_Mesh_Distance_BA_R0_R3	18,89904594	-76,10381317	-943,2681885	-0,0379
Cloud_Mesh_Distance_BA_R0_R3	18,72002792	-76,08092499	-943,2327881	-0,039928
Cloud_Mesh_Distance_BA_R0_R3	17,96160507	-83,83426666	-942,5512695	-0,103497
Cloud_Mesh_Distance_BA_R0_R3	17,7537384	-83,65021515	-942,3875732	-0,108718
Cloud_Mesh_Distance_BA_R0_R3	17,98666382	-83,8411026	-942,401062	-0,098784
Cloud_Mesh_Distance_BA_R0_R3	17,87226105	-83,7518692	-942,4416504	-0,108438
Cloud_Mesh_Distance_BA_R0_R3	17,53115654	-83,46735382	-942,4248047	-0,103549
Cloud_Mesh_Distance_BA_R0_R3	17,31903267	-83,27204132	-942,4406738	-0,097035
Cloud_Mesh_Distance_BA_R0_R3	17,33872986	-83,26810455	-942,2439575	-0,089639
Cloud_Mesh_Distance_BA_R0_R3	17,21889114	-83,17847443	-942,5417481	-0,095799
Cloud_Mesh_Distance_BA_R0_R3	17,13739395	-83,05315399	-942,2468872	-0,089658
Cloud_Mesh_Distance_BA_R0_R3	17,0300045	-82,96411896	-942,5568237	-0,094181
Cloud_Mesh_Distance_BA_R0_R3	16,78307152	-82,64203644	-942,454773	-0,075698
Cloud_Mesh_Distance_BA_R0_R3	16,70361519	-82,51438141	-942,3453369	-0,057149
Cloud_Mesh_Distance_BA_R0_R3	16,79924774	-82,62276459	-942,244751	-0,064592
Cloud_Mesh_Distance_BA_R0_R3	16,60818481	-82,41080475	-942,4526978	-0,047197
Cloud_Mesh_Distance_BA_R0_R3	16,44924927	-82,14652252	-942,3293457	-0,007541
Cloud_Mesh_Distance_BA_R0_R3	16,35450363	-82,10630035	-942,6062012	0,000236
Cloud_Mesh_Distance_BA_R0_R3	16,43917084	-82,18674469	-942,4845581	-0,016886
Cloud_Mesh_Distance_BA_R0_R3	16,2887001	-81,91584015	-942,3825073	0,017024
Cloud_Mesh_Distance_BA_R0_R3	16,27814865	-81,97316742	-942,5531006	0,011009
Cloud_Mesh_Distance_BA_R0_R3	16,2515831	-81,7967453	-942,2762451	0,032241
Cloud_Mesh_Distance_BA_R0_R3	16,12998199	-81,55484772	-942,2755737	0,038044
Cloud_Mesh_Distance_BA_R0_R3	16,1686554	-81,68802643	-942,3551636	0,033575
Cloud_Mesh_Distance_BA_R0_R3	16,00400734	-81,32871246	-942,3590088	0,020926
Cloud_Mesh_Distance_BA_R0_R3	23,14522552	-82,05381012	-942,7752075	0,100787
Cloud_Mesh_Distance_BA_R0_R3	23,15317917	-82,01804352	-942,6067505	0,097545
Cloud_Mesh_Distance_BA_R0_R3	23,19620705	-82,18624115	-942,5977783	0,171424
Cloud_Mesh_Distance_BA_R0_R3	15,93287563	-81,24207306	-942,4559326	0,009131
Cloud_Mesh_Distance_BA_R0_R3	15,77650642	-80,94483185	-942,543335	-0,022691
Cloud_Mesh_Distance_BA_R0_R3	15,72189236	-80,88562775	-942,6072998	-0,027327
Cloud_Mesh_Distance_BA_R0_R3	23,10382843	-81,73513031	-942,6587524	0,009528
Cloud_Mesh_Distance_BA_R0_R3	15,76228905	-80,83069611	-942,4801636	-0,027461
Cloud_Mesh_Distance_BA_R0_R3	23,07019806	-81,60590363	-942,8442993	-0,023511
Cloud_Mesh_Distance_BA_R0_R3	23,08763123	-81,56481171	-942,6982422	-0,035147
Cloud_Mesh_Distance_BA_R0_R3	15,62463665	-80,56248474	-942,5443115	-0,040505
Cloud_Mesh_Distance_BA_R0_R3	15,68755341	-80,50931549	-942,411438	-0,040178
Cloud_Mesh_Distance_BA_R0_R3	15,68064785	-80,72060394	-942,5510254	-0,034447
Cloud_Mesh_Distance_BA_R0_R3	15,75320148	-80,73973846	-942,43396	-0,029424
Cloud_Mesh_Distance_BA_R0_R3	15,77025986	-80,63954926	-942,3436279	-0,033125
Cloud_Mesh_Distance_BA_R0_R3	15,80006504	-80,76992798	-942,3672485	-0,022808
Cloud_Mesh_Distance_BA_R0_R3	15,48996735	-80,32074738	-942,6405029	-0,052454
Cloud_Mesh_Distance_BA_R0_R3	15,62131119	-80,47717285	-942,5021362	-0,044216

Cloud_Mesh_Distance_BA_R0_R3	23,07127762	-81,06380463	-942,8435059	-0,102802
Cloud_Mesh_Distance_BA_R0_R3	15,48302841	-80,21810913	-942,6099243	-0,057457
Cloud_Mesh_Distance_BA_R0_R3	15,55113411	-80,24295807	-942,5115967	-0,053093
Cloud_Mesh_Distance_BA_R0_R3	23,11471939	-80,84725189	-942,6454468	-0,124821
Cloud_Mesh_Distance_BA_R0_R3	15,49985218	-80,10158539	-942,5482788	-0,068876
Cloud_Mesh_Distance_BA_R0_R3	15,58504295	-80,03607941	-942,4072876	-0,064979
Cloud_Mesh_Distance_BA_R0_R3	15,5801506	-79,89641571	-942,3913574	-0,07288
Cloud_Mesh_Distance_BA_R0_R3	15,41790104	-79,74118805	-942,6233521	-0,1001
Cloud_Mesh_Distance_BA_R0_R3	15,51304817	-79,92470551	-942,4943848	-0,079079
Cloud_Mesh_Distance_BA_R0_R3	23,06797218	-80,48694611	-942,8561401	-0,116067
Cloud_Mesh_Distance_BA_R0_R3	23,06932068	-80,3770752	-942,802002	-0,113154
Cloud_Mesh_Distance_BA_R0_R3	23,08652687	-80,66443634	-942,8085327	-0,116218
Cloud_Mesh_Distance_BA_R0_R3	15,48991489	-79,64012909	-942,5006714	-0,09425
Cloud_Mesh_Distance_BA_R0_R3	23,10766983	-80,52118683	-942,671936	-0,125655
Cloud_Mesh_Distance_BA_R0_R3	15,38759899	-79,23239899	-942,6687012	-0,113953
Cloud_Mesh_Distance_BA_R0_R3	15,38400745	-79,34626007	-942,6676025	-0,111927
Cloud_Mesh_Distance_BA_R0_R3	15,4150877	-79,51107025	-942,614563	-0,107061
Cloud_Mesh_Distance_BA_R0_R3	15,44918442	-79,27558136	-942,562439	-0,109214
Cloud_Mesh_Distance_BA_R0_R3	15,49268723	-79,52344513	-942,4918823	-0,096959
Cloud_Mesh_Distance_BA_R0_R3	15,5113802	-79,40363312	-942,4634399	-0,097927
Cloud_Mesh_Distance_BA_R0_R3	22,97170067	-80,07709503	-942,9682617	-0,101389
Cloud_Mesh_Distance_BA_R0_R3	15,41889286	-79,10935211	-942,6286011	-0,11157
Cloud_Mesh_Distance_BA_R0_R3	15,49429417	-79,09360504	-942,5081787	-0,099859
Cloud_Mesh_Distance_BA_R0_R3	22,96874237	-79,86604309	-942,734436	-0,090395
Cloud_Mesh_Distance_BA_R0_R3	15,46171761	-78,90727997	-942,5941162	-0,106459
Cloud_Mesh_Distance_BA_R0_R3	22,83935928	-79,61356354	-942,8590698	-0,077534
Cloud_Mesh_Distance_BA_R0_R3	22,7506752	-79,51386261	-942,9966431	-0,080333
Cloud_Mesh_Distance_BA_R0_R3	22,8497467	-79,54440308	-942,7041626	-0,067193
Cloud_Mesh_Distance_BA_R0_R3	22,91727066	-79,69236755	-942,6813965	-0,074309
Cloud_Mesh_Distance_BA_R0_R3	15,5439682	-78,8582077	-942,4723511	-0,095849
Cloud_Mesh_Distance_BA_R0_R3	15,59558773	-78,77283478	-942,4137573	-0,089669
Cloud_Mesh_Distance_BA_R0_R3	22,79501915	-79,49346161	-942,8238525	-0,068238
Cloud_Mesh_Distance_BA_R0_R3	22,6292572	-79,25322723	-942,9705811	-0,052533
Cloud_Mesh_Distance_BA_R0_R3	15,56639099	-78,48822784	-942,552124	-0,093767
Cloud_Mesh_Distance_BA_R0_R3	15,61502457	-78,31720734	-942,5473633	-0,087135
Cloud_Mesh_Distance_BA_R0_R3	22,65219116	-79,20197296	-942,8099976	-0,043512
Cloud_Mesh_Distance_BA_R0_R3	22,56640244	-79,10028839	-942,9105835	-0,034458
Cloud_Mesh_Distance_BA_R0_R3	15,59979916	-78,12653351	-942,6878662	-0,092754
Cloud_Mesh_Distance_BA_R0_R3	22,44589043	-78,88434601	-942,8905029	-0,008457
Cloud_Mesh_Distance_BA_R0_R3	22,52776527	-78,96998596	-942,7896118	-0,016949
Cloud_Mesh_Distance_BA_R0_R3	15,64678383	-77,92586517	-942,7434082	-0,081254
Cloud_Mesh_Distance_BA_R0_R3	15,71237469	-77,88328552	-942,6491699	-0,071515
Cloud_Mesh_Distance_BA_R0_R3	15,68768978	-78,02631378	-942,5893555	-0,073424
Cloud_Mesh_Distance_BA_R0_R3	15,75739574	-77,94486237	-942,5239258	-0,063265
Cloud_Mesh_Distance_BA_R0_R3	22,3771553	-78,75901031	-942,8471069	0,001244
Cloud_Mesh_Distance_BA_R0_R3	22,11750031	-78,43321228	-942,8833008	0,015936
Cloud_Mesh_Distance_BA_R0_R3	22,22025681	-78,51689911	-942,7597656	0,016081
Cloud_Mesh_Distance_BA_R0_R3	15,72689819	-77,79336548	-942,6964722	-0,067765
Cloud_Mesh_Distance_BA_R0_R3	15,77513218	-77,7891922	-942,6112061	-0,061624
Cloud_Mesh_Distance_BA_R0_R3	15,81936646	-77,62872314	-942,6709595	-0,052745

Cloud_Mesh_Distance_BA_R0_R3	15,85512733	-77,50033569	-942,7322998	-0,045147
Cloud_Mesh_Distance_BA_R0_R3	22,04453278	-78,32534027	-942,8099976	0,013273
Cloud_Mesh_Distance_BA_R0_R3	21,93995857	-78,23764801	-942,9146729	0,012436
Cloud_Mesh_Distance_BA_R0_R3	15,83903885	-77,43138885	-942,8381348	-0,047106
Cloud_Mesh_Distance_BA_R0_R3	15,91717529	-77,55106354	-942,5755615	-0,038863
Cloud_Mesh_Distance_BA_R0_R3	16,02650833	-77,36449432	-942,5784302	-0,01985
Cloud_Mesh_Distance_BA_R0_R3	21,7244854	-78,016716	-943,0038452	0,011052
Cloud_Mesh_Distance_BA_R0_R3	21,64051437	-77,9356308	-943,0614014	0,012412
Cloud_Mesh_Distance_BA_R0_R3	21,78134155	-78,03500366	-942,8356323	0,006378
Cloud_Mesh_Distance_BA_R0_R3	21,88043213	-78,14728546	-942,8358765	0,009013
Cloud_Mesh_Distance_BA_R0_R3	15,914258	-77,36382294	-942,7701416	-0,0342
Cloud_Mesh_Distance_BA_R0_R3	16,01989555	-77,19939423	-942,7758179	-0,018847
Cloud_Mesh_Distance_BA_R0_R3	16,11165619	-77,20374298	-942,6166382	-0,006811
Cloud_Mesh_Distance_BA_R0_R3	21,46595001	-77,73009491	-943,0523071	0,016649
Cloud_Mesh_Distance_BA_R0_R3	21,58636093	-77,83560944	-942,9280396	0,009848
Cloud_Mesh_Distance_BA_R0_R3	21,68352509	-77,94524384	-942,9194336	0,008364
Cloud_Mesh_Distance_BA_R0_R3	16,20794296	-77,11788177	-942,574707	0,004055
Cloud_Mesh_Distance_BA_R0_R3	16,21506119	-77,02986145	-942,6748657	0,009287
Cloud_Mesh_Distance_BA_R0_R3	21,29668045	-77,5013504	-942,8540039	0,002333
Cloud_Mesh_Distance_BA_R0_R3	21,40172768	-77,59262085	-942,7492065	0,003805
Cloud_Mesh_Distance_BA_R0_R3	21,37947845	-77,61009979	-942,9544067	0,007414
Cloud_Mesh_Distance_BA_R0_R3	16,38161087	-76,83592987	-942,7237549	0,017263
Cloud_Mesh_Distance_BA_R0_R3	21,18937111	-77,41348267	-942,9606934	-0,000068
Cloud_Mesh_Distance_BA_R0_R3	21,27210808	-77,51830292	-943,0531616	0,008294
Cloud_Mesh_Distance_BA_R0_R3	16,32475281	-76,85916901	-942,7670898	0,012971
Cloud_Mesh_Distance_BA_R0_R3	16,57985687	-76,73484039	-942,6368408	0,020732
Cloud_Mesh_Distance_BA_R0_R3	16,46265984	-76,82215118	-942,6383057	0,019539
Cloud_Mesh_Distance_BA_R0_R3	20,96136856	-77,19484711	-942,9624634	-0,011686
Cloud_Mesh_Distance_BA_R0_R3	20,84927177	-77,10228729	-943,019165	-0,016922
Cloud_Mesh_Distance_BA_R0_R3	20,8648262	-77,09554291	-942,8995361	-0,013659
Cloud_Mesh_Distance_BA_R0_R3	21,10017776	-77,30728912	-942,8630981	-0,002672
Cloud_Mesh_Distance_BA_R0_R3	16,63762093	-76,64472198	-942,7246704	0,018656
Cloud_Mesh_Distance_BA_R0_R3	16,70419312	-76,66883087	-942,6063232	0,020758
Cloud_Mesh_Distance_BA_R0_R3	16,61520767	-76,58875275	-942,8650513	0,014347
Cloud_Mesh_Distance_BA_R0_R3	16,78340912	-76,58393097	-942,6702881	0,021225
Cloud_Mesh_Distance_BA_R0_R3	16,70755005	-76,51567078	-942,9016113	0,01767
Cloud_Mesh_Distance_BA_R0_R3	16,93946838	-76,51416779	-942,6403198	0,020549
Cloud_Mesh_Distance_BA_R0_R3	20,70497131	-76,94025421	-942,7841187	-0,018997
Cloud_Mesh_Distance_BA_R0_R3	20,6541214	-76,9207077	-942,9715576	-0,02161
Cloud_Mesh_Distance_BA_R0_R3	16,84692955	-76,46723175	-942,8364868	0,018844
Cloud_Mesh_Distance_BA_R0_R3	17,17141724	-76,37030792	-942,7128906	0,019374
Cloud_Mesh_Distance_BA_R0_R3	17,22924805	-76,3110733	-942,7974243	0,016898
Cloud_Mesh_Distance_BA_R0_R3	20,43172264	-76,73839569	-942,9897461	-0,02098
Cloud_Mesh_Distance_BA_R0_R3	17,56548309	-76,24529266	-942,6795654	0,020755
Cloud_Mesh_Distance_BA_R0_R3	17,37910843	-76,215065	-942,921875	0,013361
Cloud_Mesh_Distance_BA_R0_R3	17,35268402	-76,27535248	-942,7745972	0,020204
Cloud_Mesh_Distance_BA_R0_R3	20,23498154	-76,59326935	-942,9796753	-0,015239
Cloud_Mesh_Distance_BA_R0_R3	20,07295799	-76,4887619	-942,8070068	-0,005181
Cloud_Mesh_Distance_BA_R0_R3	20,32751083	-76,65159607	-942,8711548	-0,012909
Cloud_Mesh_Distance_BA_R0_R3	17,61519241	-76,15055084	-942,9348145	0,010219

Cloud_Mesh_Distance_BA_R0_R3	17,80017853	-76,16484833	-942,7544556	0,018863
Cloud_Mesh_Distance_BA_R0_R3	17,65249252	-76,18810272	-942,7764282	0,018821
Cloud_Mesh_Distance_BA_R0_R3	17,96870422	-76,09663391	-942,9569702	-0,004038
Cloud_Mesh_Distance_BA_R0_R3	18,34959412	-76,11466217	-942,7929688	-0,006407
Cloud_Mesh_Distance_BA_R0_R3	18,15926743	-76,0848465	-942,9679565	-0,014282
Cloud_Mesh_Distance_BA_R0_R3	18,53071785	-76,09004974	-942,9726563	-0,024106
Cloud_Mesh_Distance_BA_R0_R3	18,57149887	-76,11840057	-942,8096924	-0,017623
Cloud_Mesh_Distance_BA_R0_R3	18,82120132	-76,14225006	-942,7779541	-0,018602
Cloud_Mesh_Distance_BA_R0_R3	18,66840363	-76,10468292	-942,9337769	-0,020634
Cloud_Mesh_Distance_BA_R0_R3	19,19143295	-76,17703247	-942,925415	-0,025581
Cloud_Mesh_Distance_BA_R0_R3	18,98699951	-76,14581299	-942,8930664	-0,022149
Cloud_Mesh_Distance_BA_R0_R3	19,18079376	-76,19594574	-942,7282715	-0,013681
Cloud_Mesh_Distance_BA_R0_R3	19,68642426	-76,31401825	-942,8416748	-0,010626
Cloud_Mesh_Distance_BA_R0_R3	18,58640099	-76,08408356	-943,0447388	-0,027861
Cloud_Mesh_Distance_BA_R0_R3	18,7000885	-76,09156799	-943,065918	-0,030682
Cloud_Mesh_Distance_BA_R0_R3	18,80526352	-76,11579132	-942,9480591	-0,025022
Cloud_Mesh_Distance_BA_R0_R3	18,17345428	-83,98337555	-942,2901001	-0,170876
Cloud_Mesh_Distance_BA_R0_R3	17,85165977	-83,71843719	-942,258667	-0,102785
Cloud_Mesh_Distance_BA_R0_R3	17,76287842	-83,64446259	-942,2487793	-0,105927
Cloud_Mesh_Distance_BA_R0_R3	18,08965302	-83,90283966	-942,1304321	-0,089464
Cloud_Mesh_Distance_BA_R0_R3	18,05802917	-83,8911972	-942,3067017	-0,089103
Cloud_Mesh_Distance_BA_R0_R3	17,96589279	-83,81270599	-942,2659302	-0,094529
Cloud_Mesh_Distance_BA_R0_R3	17,54528427	-83,45986176	-942,2421265	-0,103512
Cloud_Mesh_Distance_BA_R0_R3	17,43080521	-83,3683548	-942,3417969	-0,097947
Cloud_Mesh_Distance_BA_R0_R3	17,65673065	-83,56137848	-942,3010864	-0,105647
Cloud_Mesh_Distance_BA_R0_R3	17,64017487	-83,53121185	-942,1481323	-0,098748
Cloud_Mesh_Distance_BA_R0_R3	17,22741699	-83,16661835	-942,3461304	-0,091256
Cloud_Mesh_Distance_BA_R0_R3	17,37566376	-83,27133942	-942,0270996	-0,085017
Cloud_Mesh_Distance_BA_R0_R3	17,03811073	-82,95046234	-942,3522339	-0,089993
Cloud_Mesh_Distance_BA_R0_R3	16,96304893	-82,81403351	-942,1101685	-0,074162
Cloud_Mesh_Distance_BA_R0_R3	16,95713615	-82,83615875	-942,2633057	-0,080194
Cloud_Mesh_Distance_BA_R0_R3	16,88966751	-82,73020172	-942,171936	-0,069839
Cloud_Mesh_Distance_BA_R0_R3	16,87159348	-82,74091339	-942,3554688	-0,080308
Cloud_Mesh_Distance_BA_R0_R3	16,72311401	-82,47989655	-942,1119995	-0,048958
Cloud_Mesh_Distance_BA_R0_R3	16,62904358	-82,38361359	-942,2471314	-0,039893
Cloud_Mesh_Distance_BA_R0_R3	16,64473343	-82,35353851	-942,0844116	-0,030018
Cloud_Mesh_Distance_BA_R0_R3	16,36717606	-82,04813385	-942,3977051	0,002232
Cloud_Mesh_Distance_BA_R0_R3	16,42108917	-82,06438446	-942,2365723	0,006118
Cloud_Mesh_Distance_BA_R0_R3	16,49341965	-82,1583786	-942,1862183	-0,008153
Cloud_Mesh_Distance_BA_R0_R3	16,52843475	-82,26830292	-942,3430786	-0,027906
Cloud_Mesh_Distance_BA_R0_R3	16,3503685	-81,92989349	-942,2005615	0,018439
Cloud_Mesh_Distance_BA_R0_R3	23,30341911	-82,49199677	-942,5755005	0,251524
Cloud_Mesh_Distance_BA_R0_R3	23,26668549	-82,35614777	-942,3329468	0,258164
Cloud_Mesh_Distance_BA_R0_R3	23,25653076	-82,34931183	-942,4901123	0,236461
Cloud_Mesh_Distance_BA_R0_R3	16,06371117	-81,47888947	-942,3692017	0,032037
Cloud_Mesh_Distance_BA_R0_R3	16,0912323	-81,4081955	-942,2105103	0,035769
Cloud_Mesh_Distance_BA_R0_R3	16,04979706	-81,29631042	-942,2080078	0,029893
Cloud_Mesh_Distance_BA_R0_R3	23,11951065	-81,89774323	-942,7227783	0,05357
Cloud_Mesh_Distance_BA_R0_R3	23,22231674	-82,2345047	-942,3687744	0,207136
Cloud_Mesh_Distance_BA_R0_R3	15,95320988	-81,1813736	-942,3459473	0,009802

Cloud_Mesh_Distance_BA_R0_R3	23,17127037	-82,05370331	-942,4101563	0,124868
Cloud_Mesh_Distance_BA_R0_R3	23,12366676	-81,78992462	-942,4793091	0,027643
Cloud_Mesh_Distance_BA_R0_R3	23,13511658	-81,89881134	-942,5397339	0,058421
Cloud_Mesh_Distance_BA_R0_R3	15,93336296	-81,04465485	-942,2807007	0,004201
Cloud_Mesh_Distance_BA_R0_R3	15,89038849	-81,11754608	-942,4390259	0,000725
Cloud_Mesh_Distance_BA_R0_R3	15,8439579	-80,92433929	-942,3839722	-0,013228
Cloud_Mesh_Distance_BA_R0_R3	15,98837376	-80,97829437	-942,1152344	0,010187
Cloud_Mesh_Distance_BA_R0_R3	23,12788963	-81,78865814	-942,3624268	0,027424
Cloud_Mesh_Distance_BA_R0_R3	23,11135864	-81,62885284	-942,4763184	-0,022326
Cloud_Mesh_Distance_BA_R0_R3	15,91963577	-80,89492035	-942,210022	-0,002956
Cloud_Mesh_Distance_BA_R0_R3	15,87320805	-80,71799469	-942,1992798	-0,017763
Cloud_Mesh_Distance_BA_R0_R3	23,0891304	-81,41925812	-942,6676025	-0,063021
Cloud_Mesh_Distance_BA_R0_R3	23,09487724	-81,22777557	-942,6602173	-0,093929
Cloud_Mesh_Distance_BA_R0_R3	15,65242195	-80,1760025	-942,3407593	-0,057941
Cloud_Mesh_Distance_BA_R0_R3	15,62947178	-80,36591339	-942,4381104	-0,046181
Cloud_Mesh_Distance_BA_R0_R3	15,73358059	-80,31587219	-942,2615967	-0,046417
Cloud_Mesh_Distance_BA_R0_R3	23,10492325	-81,03353882	-942,6560669	-0,115109
Cloud_Mesh_Distance_BA_R0_R3	23,13103104	-81,01357269	-942,4641113	-0,130155
Cloud_Mesh_Distance_BA_R0_R3	15,66951084	-80,02004242	-942,2819214	-0,058393
Cloud_Mesh_Distance_BA_R0_R3	15,75546074	-80,07376862	-942,1655884	-0,046062
Cloud_Mesh_Distance_BA_R0_R3	23,14103699	-80,83247375	-942,4602661	-0,137477
Cloud_Mesh_Distance_BA_R0_R3	15,58130741	-79,73790741	-942,3734741	-0,081423
Cloud_Mesh_Distance_BA_R0_R3	15,6431427	-79,88626862	-942,3004761	-0,068005
Cloud_Mesh_Distance_BA_R0_R3	23,12205124	-80,69911957	-942,6160889	-0,129718
Cloud_Mesh_Distance_BA_R0_R3	23,14359283	-80,65447235	-942,4642944	-0,140371
Cloud_Mesh_Distance_BA_R0_R3	15,70924091	-79,57280731	-942,1873169	-0,068885
Cloud_Mesh_Distance_BA_R0_R3	15,64169407	-79,54741669	-942,2806397	-0,08105
Cloud_Mesh_Distance_BA_R0_R3	15,55521107	-79,53464508	-942,4011231	-0,093559
Cloud_Mesh_Distance_BA_R0_R3	15,66736603	-79,66239929	-942,2487793	-0,073892
Cloud_Mesh_Distance_BA_R0_R3	23,06943893	-80,1576004	-942,6216431	-0,113429
Cloud_Mesh_Distance_BA_R0_R3	23,11992455	-80,290802	-942,4451904	-0,126633
Cloud_Mesh_Distance_BA_R0_R3	23,09464264	-80,3332901	-942,6461792	-0,117018
Cloud_Mesh_Distance_BA_R0_R3	15,61970615	-79,35138702	-942,3110352	-0,08629
Cloud_Mesh_Distance_BA_R0_R3	23,03707504	-80,21460724	-942,8348999	-0,108291
Cloud_Mesh_Distance_BA_R0_R3	23,00783157	-80,03203583	-942,7815552	-0,101839
Cloud_Mesh_Distance_BA_R0_R3	15,54542637	-79,21735382	-942,4212036	-0,09445
Cloud_Mesh_Distance_BA_R0_R3	23,03620148	-79,98740387	-942,5787354	-0,102888
Cloud_Mesh_Distance_BA_R0_R3	15,56404114	-79,06478119	-942,4082642	-0,091681
Cloud_Mesh_Distance_BA_R0_R3	15,53375912	-78,97248077	-942,4660645	-0,097208
Cloud_Mesh_Distance_BA_R0_R3	15,64208031	-79,10497284	-942,2943726	-0,084541
Cloud_Mesh_Distance_BA_R0_R3	15,60630608	-78,92443848	-942,3670654	-0,088978
Cloud_Mesh_Distance_BA_R0_R3	15,68795013	-78,92923737	-942,2518311	-0,077938
Cloud_Mesh_Distance_BA_R0_R3	22,79896545	-79,44110107	-942,7171021	-0,06149
Cloud_Mesh_Distance_BA_R0_R3	22,89540482	-79,57862091	-942,5651245	-0,069042
Cloud_Mesh_Distance_BA_R0_R3	15,52658558	-78,6997757	-942,5421143	-0,097776
Cloud_Mesh_Distance_BA_R0_R3	15,62342548	-78,61513519	-942,413147	-0,086758
Cloud_Mesh_Distance_BA_R0_R3	15,65449905	-78,43493652	-942,4276123	-0,078573
Cloud_Mesh_Distance_BA_R0_R3	22,72645569	-79,35681915	-942,8300781	-0,060648
Cloud_Mesh_Distance_BA_R0_R3	22,77514648	-79,35765076	-942,6484375	-0,050038
Cloud_Mesh_Distance_BA_R0_R3	15,67169094	-78,32091522	-942,4499512	-0,075868

Cloud_Mesh_Distance_BA_R0_R3	22,61493301	-79,08144379	-942,704773	-0,023524
Cloud_Mesh_Distance_BA_R0_R3	15,68589211	-78,1947403	-942,4897461	-0,074401
Cloud_Mesh_Distance_BA_R0_R3	15,77089024	-78,16028595	-942,3713379	-0,061927
Cloud_Mesh_Distance_BA_R0_R3	22,46778107	-78,84209442	-942,7175293	0,002191
Cloud_Mesh_Distance_BA_R0_R3	22,3940773	-78,70752716	-942,6444702	0,018323
Cloud_Mesh_Distance_BA_R0_R3	22,55849266	-78,93466187	-942,5813599	-0,000679
Cloud_Mesh_Distance_BA_R0_R3	15,80369949	-78,02085114	-942,3989868	-0,056166
Cloud_Mesh_Distance_BA_R0_R3	15,74010563	-78,07169342	-942,4699097	-0,065965
Cloud_Mesh_Distance_BA_R0_R3	22,30510902	-78,59002686	-942,6495361	0,020205
Cloud_Mesh_Distance_BA_R0_R3	15,83579636	-77,8675766	-942,4502564	-0,053009
Cloud_Mesh_Distance_BA_R0_R3	15,88383389	-77,96929932	-942,3103027	-0,046274
Cloud_Mesh_Distance_BA_R0_R3	22,2431469	-78,4943161	-942,579895	0,018968
Cloud_Mesh_Distance_BA_R0_R3	22,14574432	-78,41065216	-942,6995239	0,01549
Cloud_Mesh_Distance_BA_R0_R3	22,29837799	-78,63185883	-942,8037109	0,011137
Cloud_Mesh_Distance_BA_R0_R3	15,85718918	-77,71868134	-942,5293579	-0,048929
Cloud_Mesh_Distance_BA_R0_R3	21,97252274	-78,22348785	-942,7346191	0,007259
Cloud_Mesh_Distance_BA_R0_R3	16,09452629	-77,42404175	-942,4223633	-0,018117
Cloud_Mesh_Distance_BA_R0_R3	16,1333847	-77,30371857	-942,4813843	-0,011115
Cloud_Mesh_Distance_BA_R0_R3	16,17026138	-77,21239471	-942,5230103	-0,002711
Cloud_Mesh_Distance_BA_R0_R3	16,23827744	-77,24239349	-942,4033814	-0,00234
Cloud_Mesh_Distance_BA_R0_R3	16,17763519	-77,40361786	-942,3303833	-0,013595
Cloud_Mesh_Distance_BA_R0_R3	21,68466377	-77,90883636	-942,7648926	0,002464
Cloud_Mesh_Distance_BA_R0_R3	21,58781433	-77,79030609	-942,7178345	0,003363
Cloud_Mesh_Distance_BA_R0_R3	21,78500748	-77,99111176	-942,6264038	0,004875
Cloud_Mesh_Distance_BA_R0_R3	16,28273392	-77,01448822	-942,5993652	0,010471
Cloud_Mesh_Distance_BA_R0_R3	21,48831558	-77,70494843	-942,8413696	0,002964
Cloud_Mesh_Distance_BA_R0_R3	21,42029953	-77,5812149	-942,5507202	0,001741
Cloud_Mesh_Distance_BA_R0_R3	16,35153389	-77,00530243	-942,5236816	0,012411
Cloud_Mesh_Distance_BA_R0_R3	16,37139702	-76,91727448	-942,6138916	0,014796
Cloud_Mesh_Distance_BA_R0_R3	16,55380249	-76,81552124	-942,5427856	0,019011
Cloud_Mesh_Distance_BA_R0_R3	21,21030426	-77,39662933	-942,7573853	-0,001085
Cloud_Mesh_Distance_BA_R0_R3	21,12244797	-77,29711151	-942,6665039	-0,004827
Cloud_Mesh_Distance_BA_R0_R3	16,64843941	-76,814888	-942,4420166	0,018336
Cloud_Mesh_Distance_BA_R0_R3	16,7631073	-76,73645782	-942,440979	0,023483
Cloud_Mesh_Distance_BA_R0_R3	16,87415504	-76,65978241	-942,4508667	0,024455
Cloud_Mesh_Distance_BA_R0_R3	20,92292786	-77,12347412	-942,7417603	-0,011239
Cloud_Mesh_Distance_BA_R0_R3	21,02896118	-77,22336578	-942,763794	-0,008185
Cloud_Mesh_Distance_BA_R0_R3	17,00344467	-76,58773041	-942,4507446	0,021507
Cloud_Mesh_Distance_BA_R0_R3	16,77811241	-76,66034698	-942,5447388	0,022002
Cloud_Mesh_Distance_BA_R0_R3	20,71037292	-76,92430878	-942,5627441	-0,006985
Cloud_Mesh_Distance_BA_R0_R3	20,81697083	-77,03907776	-942,807312	-0,013565
Cloud_Mesh_Distance_BA_R0_R3	17,02766228	-76,44423676	-942,6917725	0,019516
Cloud_Mesh_Distance_BA_R0_R3	17,05632782	-76,50500488	-942,5473023	0,023188
Cloud_Mesh_Distance_BA_R0_R3	17,13611221	-76,53157043	-942,4319458	0,022372
Cloud_Mesh_Distance_BA_R0_R3	17,18654442	-76,45697021	-942,5230713	0,02436
Cloud_Mesh_Distance_BA_R0_R3	17,35030746	-76,38233185	-942,5356445	0,026029
Cloud_Mesh_Distance_BA_R0_R3	17,44168854	-76,30916595	-942,6192017	0,024474
Cloud_Mesh_Distance_BA_R0_R3	17,52013016	-76,34722137	-942,4804077	0,029785
Cloud_Mesh_Distance_BA_R0_R3	17,5440979	-76,30010986	-942,5648804	0,026734
Cloud_Mesh_Distance_BA_R0_R3	20,23538971	-76,58666992	-942,621582	0,006882

Cloud_Mesh_Distance_BA_R0_R3	20,44185829	-76,72856903	-942,7390137	-0,011021
Cloud_Mesh_Distance_BA_R0_R3	20,35024261	-76,66228485	-942,515625	0,008364
Cloud_Mesh_Distance_BA_R0_R3	20,56504822	-76,83405304	-942,8721314	-0,019058
Cloud_Mesh_Distance_BA_R0_R3	17,3092308	-76,32656097	-942,6876831	0,021028
Cloud_Mesh_Distance_BA_R0_R3	17,63760757	-76,30698395	-942,4837036	0,02855
Cloud_Mesh_Distance_BA_R0_R3	17,95236969	-76,22694397	-942,4923706	0,026512
Cloud_Mesh_Distance_BA_R0_R3	19,96143723	-76,43136597	-942,7519531	-0,000376
Cloud_Mesh_Distance_BA_R0_R3	20,2022438	-76,56530762	-942,7922363	-0,008367
Cloud_Mesh_Distance_BA_R0_R3	20,31373596	-76,6370697	-942,7064209	-0,004871
Cloud_Mesh_Distance_BA_R0_R3	17,73626328	-76,20993042	-942,6554565	0,026158
Cloud_Mesh_Distance_BA_R0_R3	17,85435867	-76,20624542	-942,5961914	0,027418
Cloud_Mesh_Distance_BA_R0_R3	17,97176933	-76,1818161	-942,612854	0,023466
Cloud_Mesh_Distance_BA_R0_R3	17,9707756	-76,13593292	-942,7739868	0,008963
Cloud_Mesh_Distance_BA_R0_R3	18,15190125	-76,12050629	-942,7820435	0,001158
Cloud_Mesh_Distance_BA_R0_R3	18,13577271	-76,16468811	-942,6126709	0,020321
Cloud_Mesh_Distance_BA_R0_R3	18,52469254	-76,15724945	-942,6162109	0,001007
Cloud_Mesh_Distance_BA_R0_R3	18,74014664	-76,16966248	-942,6064453	-0,003741
Cloud_Mesh_Distance_BA_R0_R3	19,02267075	-76,17744446	-942,6998901	-0,011985
Cloud_Mesh_Distance_BA_R0_R3	18,95343018	-76,19345856	-942,5751953	-0,006353
Cloud_Mesh_Distance_BA_R0_R3	19,35816956	-76,22590637	-942,7622681	-0,010324
Cloud_Mesh_Distance_BA_R0_R3	19,35298538	-76,24824524	-942,5730591	0,00456
Cloud_Mesh_Distance_BA_R0_R3	19,52700424	-76,26537323	-942,7987671	-0,010751
Cloud_Mesh_Distance_BA_R0_R3	19,5212841	-76,28058624	-942,6088867	0,00652
Cloud_Mesh_Distance_BA_R0_R3	19,68038559	-76,32268524	-942,651062	0,008749
Cloud_Mesh_Distance_BA_R0_R3	19,82560349	-76,37242889	-942,7027588	0,00585
Cloud_Mesh_Distance_BA_R0_R3	19,83696747	-76,37229919	-942,8901978	-0,008429
Cloud_Mesh_Distance_BA_R0_R3	18,29333878	-84,05115509	-942,0214233	-0,051045
Cloud_Mesh_Distance_BA_R0_R3	18,34089661	-84,06472015	-941,8462524	-0,040389
Cloud_Mesh_Distance_BA_R0_R3	18,41568375	-84,13578033	-942,0072022	-0,084696
Cloud_Mesh_Distance_BA_R0_R3	18,21182632	-83,9985733	-942,1157837	-0,068584
Cloud_Mesh_Distance_BA_R0_R3	18,2039299	-83,97879791	-941,9663696	-0,054619
Cloud_Mesh_Distance_BA_R0_R3	18,30189705	-84,07242584	-942,1687012	-0,055688
Cloud_Mesh_Distance_BA_R0_R3	17,80747604	-83,66162872	-942,0404053	-0,090555
Cloud_Mesh_Distance_BA_R0_R3	17,81781006	-83,68091583	-942,1508179	-0,098534
Cloud_Mesh_Distance_BA_R0_R3	17,93159103	-83,76735687	-942,0583496	-0,085829
Cloud_Mesh_Distance_BA_R0_R3	18,0838871	-83,8791275	-941,9092407	-0,062525
Cloud_Mesh_Distance_BA_R0_R3	17,70158768	-83,56360626	-941,9753418	-0,089509
Cloud_Mesh_Distance_BA_R0_R3	17,56899643	-83,45970917	-942,0714111	-0,092506
Cloud_Mesh_Distance_BA_R0_R3	17,73139954	-83,60805511	-942,1448975	-0,098872
Cloud_Mesh_Distance_BA_R0_R3	17,27016068	-83,14484406	-941,9433594	-0,07891
Cloud_Mesh_Distance_BA_R0_R3	17,24731827	-83,15891266	-942,1435547	-0,086219
Cloud_Mesh_Distance_BA_R0_R3	17,45552826	-83,36829376	-942,1559448	-0,092364
Cloud_Mesh_Distance_BA_R0_R3	17,49109268	-83,37882233	-942,0038452	-0,085144
Cloud_Mesh_Distance_BA_R0_R3	17,37811279	-83,2314682	-941,824707	-0,079809
Cloud_Mesh_Distance_BA_R0_R3	17,16135406	-83,04540253	-942,0474243	-0,07701
Cloud_Mesh_Distance_BA_R0_R3	17,09994507	-82,9512558	-941,954773	-0,07109
Cloud_Mesh_Distance_BA_R0_R3	17,04834366	-82,92716217	-942,1322632	-0,077254
Cloud_Mesh_Distance_BA_R0_R3	16,83872986	-82,62245941	-942,026062	-0,055196
Cloud_Mesh_Distance_BA_R0_R3	16,9233284	-82,74121857	-942,027832	-0,063491
Cloud_Mesh_Distance_BA_R0_R3	16,77469254	-82,49593353	-941,9246216	-0,040966

Cloud_Mesh_Distance_BA_R0_R3	16,6134243	-82,27584076	-942,0084839	-0,018141
Cloud_Mesh_Distance_BA_R0_R3	16,54615974	-82,16257477	-941,9985962	-0,006669
Cloud_Mesh_Distance_BA_R0_R3	16,57453537	-82,26786041	-942,1443481	-0,023015
Cloud_Mesh_Distance_BA_R0_R3	16,50496674	-82,01387787	-941,8485718	0,013073
Cloud_Mesh_Distance_BA_R0_R3	16,44920731	-82,033638	-942,071228	0,007343
Cloud_Mesh_Distance_BA_R0_R3	16,21307373	-81,64098358	-942,15271	0,03822
Cloud_Mesh_Distance_BA_R0_R3	16,29902649	-81,77143097	-942,0889282	0,030814
Cloud_Mesh_Distance_BA_R0_R3	16,1473217	-81,40753937	-942,0621338	0,04569
Cloud_Mesh_Distance_BA_R0_R3	16,16043091	-81,50406647	-942,1290283	0,042702
Cloud_Mesh_Distance_BA_R0_R3	16,16643143	-81,34358978	-941,9561157	0,048869
Cloud_Mesh_Distance_BA_R0_R3	16,22806931	-81,50074005	-941,9459229	0,050791
Cloud_Mesh_Distance_BA_R0_R3	23,19398117	-82,15213776	-942,1959839	0,170956
Cloud_Mesh_Distance_BA_R0_R3	23,15555573	-81,98404694	-942,2119141	0,097881
Cloud_Mesh_Distance_BA_R0_R3	23,1376915	-81,86443329	-942,2650757	0,050006
Cloud_Mesh_Distance_BA_R0_R3	23,14191628	-81,89756012	-942,3746948	0,065157
Cloud_Mesh_Distance_BA_R0_R3	16,01193428	-81,16927338	-942,1929932	0,024683
Cloud_Mesh_Distance_BA_R0_R3	16,07441521	-81,07302094	-941,9799805	0,032364
Cloud_Mesh_Distance_BA_R0_R3	23,12718582	-81,75858307	-942,2345581	0,015433
Cloud_Mesh_Distance_BA_R0_R3	15,96246243	-80,81125641	-942,0753174	0,002641
Cloud_Mesh_Distance_BA_R0_R3	16,00591469	-80,73497772	-941,9491577	0,011935
Cloud_Mesh_Distance_BA_R0_R3	23,11048889	-81,40462494	-942,4654541	-0,075689
Cloud_Mesh_Distance_BA_R0_R3	23,11903381	-81,57341766	-942,263794	-0,043984
Cloud_Mesh_Distance_BA_R0_R3	23,12337494	-81,38103485	-942,2623901	-0,08696
Cloud_Mesh_Distance_BA_R0_R3	15,83158207	-80,54341125	-942,1917114	-0,025867
Cloud_Mesh_Distance_BA_R0_R3	15,76980782	-80,47905731	-942,2667847	-0,036288
Cloud_Mesh_Distance_BA_R0_R3	15,95270157	-80,67003632	-942,0264282	-0,001823
Cloud_Mesh_Distance_BA_R0_R3	23,11909103	-81,20546722	-942,4644775	-0,105598
Cloud_Mesh_Distance_BA_R0_R3	15,82736683	-80,39124298	-942,1383667	-0,031599
Cloud_Mesh_Distance_BA_R0_R3	15,88631916	-80,30397797	-942,0108643	-0,015903
Cloud_Mesh_Distance_BA_R0_R3	15,83129311	-80,0999527	-942,0532227	-0,031834
Cloud_Mesh_Distance_BA_R0_R3	15,82269382	-80,2179718	-942,0939331	-0,028588
Cloud_Mesh_Distance_BA_R0_R3	23,16168594	-80,80609894	-942,2705689	-0,157508
Cloud_Mesh_Distance_BA_R0_R3	15,71187305	-79,93076324	-942,2073975	-0,058826
Cloud_Mesh_Distance_BA_R0_R3	15,77967834	-79,8539505	-942,0986328	-0,050158
Cloud_Mesh_Distance_BA_R0_R3	15,66737366	-79,76085663	-942,2543335	-0,072969
Cloud_Mesh_Distance_BA_R0_R3	15,70802307	-79,82463837	-942,2011108	-0,065654
Cloud_Mesh_Distance_BA_R0_R3	23,16534042	-80,61859894	-942,2680054	-0,153076
Cloud_Mesh_Distance_BA_R0_R3	15,71829224	-79,42694855	-942,1738892	-0,07246
Cloud_Mesh_Distance_BA_R0_R3	15,73449707	-79,69660187	-942,1550903	-0,061455
Cloud_Mesh_Distance_BA_R0_R3	23,13669586	-80,47112274	-942,4644165	-0,136014
Cloud_Mesh_Distance_BA_R0_R3	15,73160934	-79,22185516	-942,163269	-0,073084
Cloud_Mesh_Distance_BA_R0_R3	15,74748421	-79,01856232	-942,1585083	-0,070101
Cloud_Mesh_Distance_BA_R0_R3	22,99542046	-79,82426453	-942,5274658	-0,089332
Cloud_Mesh_Distance_BA_R0_R3	15,82252884	-78,92147064	-942,067627	-0,058592
Cloud_Mesh_Distance_BA_R0_R3	22,95247078	-79,67623138	-942,4674683	-0,076967
Cloud_Mesh_Distance_BA_R0_R3	15,76313782	-78,83976746	-942,1616821	-0,066652
Cloud_Mesh_Distance_BA_R0_R3	15,85299683	-78,75920868	-942,0512695	-0,050521
Cloud_Mesh_Distance_BA_R0_R3	15,72814846	-78,52880096	-942,2832031	-0,068948
Cloud_Mesh_Distance_BA_R0_R3	15,68308067	-78,753685	-942,2896118	-0,078033
Cloud_Mesh_Distance_BA_R0_R3	15,78813553	-78,65671539	-942,163208	-0,061644

Cloud_Mesh_Distance_BA_R0_R3	22,73347473	-79,17874908	-942,4036255	-0,023217
Cloud_Mesh_Distance_BA_R0_R3	22,78873634	-79,31674957	-942,491272	-0,039128
Cloud_Mesh_Distance_BA_R0_R3	22,84020805	-79,45308685	-942,5619507	-0,056183
Cloud_Mesh_Distance_BA_R0_R3	22,80900955	-79,29242706	-942,2955322	-0,030551
Cloud_Mesh_Distance_BA_R0_R3	15,81577778	-78,51369476	-942,1619263	-0,05474
Cloud_Mesh_Distance_BA_R0_R3	15,85868835	-78,44275665	-942,1246338	-0,048676
Cloud_Mesh_Distance_BA_R0_R3	22,6069603	-78,95563507	-942,4019775	0,001283
Cloud_Mesh_Distance_BA_R0_R3	22,7099514	-79,22180939	-942,6295776	-0,033919
Cloud_Mesh_Distance_BA_R0_R3	22,67311096	-79,04297638	-942,3117676	-0,001532
Cloud_Mesh_Distance_BA_R0_R3	22,65307045	-79,06749725	-942,5056152	-0,011951
Cloud_Mesh_Distance_BA_R0_R3	15,81246853	-78,39473724	-942,2052612	-0,056215
Cloud_Mesh_Distance_BA_R0_R3	15,73414135	-78,32593536	-942,3483887	-0,065119
Cloud_Mesh_Distance_BA_R0_R3	15,82860088	-78,2364502	-942,246521	-0,051104
Cloud_Mesh_Distance_BA_R0_R3	22,48211098	-78,80766296	-942,5601196	0,013384
Cloud_Mesh_Distance_BA_R0_R3	15,83883476	-78,10141754	-942,2997437	-0,051657
Cloud_Mesh_Distance_BA_R0_R3	22,44563484	-78,72940063	-942,473938	0,021258
Cloud_Mesh_Distance_BA_R0_R3	22,5291214	-78,83057404	-942,3944702	0,014244
Cloud_Mesh_Distance_BA_R0_R3	16,07865906	-77,66857147	-942,2442017	-0,027559
Cloud_Mesh_Distance_BA_R0_R3	16,0387001	-77,82102203	-942,1888428	-0,030767
Cloud_Mesh_Distance_BA_R0_R3	15,94855976	-77,7594986	-942,3574219	-0,038595
Cloud_Mesh_Distance_BA_R0_R3	22,16379166	-78,39033508	-942,5324097	0,018109
Cloud_Mesh_Distance_BA_R0_R3	22,09954071	-78,31026459	-942,4981079	0,017635
Cloud_Mesh_Distance_BA_R0_R3	22,25139618	-78,46008301	-942,3783569	0,027362
Cloud_Mesh_Distance_BA_R0_R3	16,11761856	-77,52138519	-942,3073731	-0,020164
Cloud_Mesh_Distance_BA_R0_R3	21,886549	-78,11306	-942,6723023	0,006257
Cloud_Mesh_Distance_BA_R0_R3	21,89057541	-78,07502747	-942,4598389	0,008101
Cloud_Mesh_Distance_BA_R0_R3	22,07322502	-78,31127167	-942,6348267	0,013114
Cloud_Mesh_Distance_BA_R0_R3	22,00425339	-78,21225739	-942,5322876	0,013952
Cloud_Mesh_Distance_BA_R0_R3	22,15794945	-78,3591156	-942,4126587	0,022111
Cloud_Mesh_Distance_BA_R0_R3	16,02758408	-77,54471588	-942,4121704	-0,028189
Cloud_Mesh_Distance_BA_R0_R3	21,78155518	-77,95727539	-942,4605713	0,00324
Cloud_Mesh_Distance_BA_R0_R3	21,71713638	-77,87441254	-942,3634033	0,003424
Cloud_Mesh_Distance_BA_R0_R3	16,31744766	-77,26378632	-942,2884522	-0,002195
Cloud_Mesh_Distance_BA_R0_R3	16,31608582	-77,20030975	-942,3511963	0,002136
Cloud_Mesh_Distance_BA_R0_R3	21,68023491	-77,86316681	-942,5596314	0,00319
Cloud_Mesh_Distance_BA_R0_R3	21,58159828	-77,75591278	-942,5630493	0,003682
Cloud_Mesh_Distance_BA_R0_R3	16,33463097	-77,10627747	-942,4268189	0,005809
Cloud_Mesh_Distance_BA_R0_R3	16,43259621	-77,00231171	-942,4317627	0,012922
Cloud_Mesh_Distance_BA_R0_R3	16,50506973	-77,11438751	-942,2335815	0,005108
Cloud_Mesh_Distance_BA_R0_R3	16,5218544	-77,00281525	-942,3378906	0,013379
Cloud_Mesh_Distance_BA_R0_R3	21,5008812	-77,68210602	-942,6483765	0,004612
Cloud_Mesh_Distance_BA_R0_R3	21,44384003	-77,58374786	-942,3388062	-0,000021
Cloud_Mesh_Distance_BA_R0_R3	16,5381813	-76,90487671	-942,4375	0,017481
Cloud_Mesh_Distance_BA_R0_R3	16,73155212	-76,90824127	-942,2427368	0,016325
Cloud_Mesh_Distance_BA_R0_R3	16,61997223	-77,00525665	-942,2359009	0,010346
Cloud_Mesh_Distance_BA_R0_R3	21,13006592	-77,28165436	-942,4732056	-0,000312
Cloud_Mesh_Distance_BA_R0_R3	21,22564316	-77,38317108	-942,5581055	0,000196
Cloud_Mesh_Distance_BA_R0_R3	21,31584549	-77,48674774	-942,6516724	-0,000121
Cloud_Mesh_Distance_BA_R0_R3	21,33068466	-77,47689056	-942,4500732	0,000301
Cloud_Mesh_Distance_BA_R0_R3	16,7456131	-76,81822968	-942,3441162	0,017303

Cloud_Mesh_Distance_BA_R0_R3	16,95851707	-76,74529266	-942,2510376	0,016462
Cloud_Mesh_Distance_BA_R0_R3	20,81888771	-77,02071381	-942,6448364	-0,011783
Cloud_Mesh_Distance_BA_R0_R3	20,92562294	-77,1004715	-942,5247192	-0,00236
Cloud_Mesh_Distance_BA_R0_R3	21,0282917	-77,19946289	-942,5927124	-0,0048
Cloud_Mesh_Distance_BA_R0_R3	21,02797318	-77,18109894	-942,3972778	-0,000233
Cloud_Mesh_Distance_BA_R0_R3	17,20439911	-76,59664917	-942,2719116	0,023408
Cloud_Mesh_Distance_BA_R0_R3	17,23694229	-76,51726532	-942,3758545	0,021854
Cloud_Mesh_Distance_BA_R0_R3	16,97790146	-76,66512299	-942,3508911	0,021265
Cloud_Mesh_Distance_BA_R0_R3	20,820858	-77,00595856	-942,4483643	-0,002135
Cloud_Mesh_Distance_BA_R0_R3	17,32195282	-76,53507233	-942,2821655	0,022118
Cloud_Mesh_Distance_BA_R0_R3	17,33808899	-76,46865082	-942,3834229	0,023584
Cloud_Mesh_Distance_BA_R0_R3	17,73405075	-76,32653046	-942,3829956	0,033309
Cloud_Mesh_Distance_BA_R0_R3	20,22660065	-76,60086823	-942,3215332	0,022735
Cloud_Mesh_Distance_BA_R0_R3	20,37361908	-76,68743896	-942,3126221	0,018844
Cloud_Mesh_Distance_BA_R0_R3	20,5886116	-76,83544159	-942,6641235	-0,010208
Cloud_Mesh_Distance_BA_R0_R3	20,495224	-76,76125336	-942,5551758	-0,001625
Cloud_Mesh_Distance_BA_R0_R3	18,10221481	-76,21619415	-942,4627686	0,029527
Cloud_Mesh_Distance_BA_R0_R3	17,79548264	-76,26576996	-942,4796143	0,031885
Cloud_Mesh_Distance_BA_R0_R3	18,28426743	-76,20480347	-942,4536743	0,021104
Cloud_Mesh_Distance_BA_R0_R3	18,47772217	-76,20178223	-942,4494629	0,013866
Cloud_Mesh_Distance_BA_R0_R3	18,67987442	-76,20789337	-942,4448242	0,003057
Cloud_Mesh_Distance_BA_R0_R3	18,8833847	-76,22229767	-942,4338379	0,002186
Cloud_Mesh_Distance_BA_R0_R3	19,07524109	-76,24284363	-942,4133911	0,004538
Cloud_Mesh_Distance_BA_R0_R3	18,82130051	-76,26202393	-942,2891235	0,011914
Cloud_Mesh_Distance_BA_R0_R3	19,22270393	-76,26663208	-942,3822632	0,009443
Cloud_Mesh_Distance_BA_R0_R3	19,94054794	-76,42836761	-942,5977173	0,015143
Cloud_Mesh_Distance_BA_R0_R3	19,82614326	-76,38692474	-942,5219727	0,016757
Cloud_Mesh_Distance_BA_R0_R3	20,20642471	-76,57444	-942,4946899	0,014692
Cloud_Mesh_Distance_BA_R0_R3	20,09304428	-76,50414276	-942,6103516	0,009225
Cloud_Mesh_Distance_BA_R0_R3	18,32214737	-76,15615082	-942,6135864	0,008889
Cloud_Mesh_Distance_BA_R0_R3	19,16880798	-76,22577667	-942,5355835	0,001989
Cloud_Mesh_Distance_BA_R0_R3	19,67557144	-76,3422699	-942,466919	0,022721
Cloud_Mesh_Distance_BA_R0_R3	19,51506805	-76,30542755	-942,4324341	0,01937
Cloud_Mesh_Distance_BA_R0_R3	18,8082695	-84,29297638	-941,6950684	-0,044442
Cloud_Mesh_Distance_BA_R0_R3	18,979702	-84,3470993	-941,638855	-0,054365
Cloud_Mesh_Distance_BA_R0_R3	18,34087372	-84,03522491	-941,642273	-0,030764
Cloud_Mesh_Distance_BA_R0_R3	18,47120667	-84,14694977	-941,8312378	-0,034241
Cloud_Mesh_Distance_BA_R0_R3	18,58901024	-84,19503021	-941,7245483	-0,03498
Cloud_Mesh_Distance_BA_R0_R3	18,55634308	-84,20816803	-941,8967285	-0,040732
Cloud_Mesh_Distance_BA_R0_R3	18,21267509	-83,96405792	-941,7719116	-0,041557
Cloud_Mesh_Distance_BA_R0_R3	17,67628479	-83,52239227	-941,8446655	-0,083422
Cloud_Mesh_Distance_BA_R0_R3	17,85283279	-83,68106842	-941,8659668	-0,07563
Cloud_Mesh_Distance_BA_R0_R3	17,75086784	-83,58019257	-941,7880249	-0,078182
Cloud_Mesh_Distance_BA_R0_R3	17,98233414	-83,7881546	-941,8378906	-0,063701
Cloud_Mesh_Distance_BA_R0_R3	17,58986664	-83,45148468	-941,8917847	-0,083799
Cloud_Mesh_Distance_BA_R0_R3	17,29386711	-83,12323761	-941,7563477	-0,072527
Cloud_Mesh_Distance_BA_R0_R3	17,39085579	-83,19794464	-941,6531982	-0,075447
Cloud_Mesh_Distance_BA_R0_R3	17,47704697	-83,340065	-941,8662109	-0,080766
Cloud_Mesh_Distance_BA_R0_R3	17,18980408	-83,03281403	-941,8561401	-0,071603
Cloud_Mesh_Distance_BA_R0_R3	17,10869408	-82,91376495	-941,7841797	-0,067941

Cloud_Mesh_Distance_BA_R0_R3	16,87175369	-82,53452301	-941,6439209	-0,037719
Cloud_Mesh_Distance_BA_R0_R3	16,85310745	-82,5761795	-941,8200684	-0,046272
Cloud_Mesh_Distance_BA_R0_R3	16,92789459	-82,70336151	-941,8763428	-0,054754
Cloud_Mesh_Distance_BA_R0_R3	17,00936699	-82,83457184	-941,9476929	-0,067038
Cloud_Mesh_Distance_BA_R0_R3	17,02017593	-82,78400421	-941,7398682	-0,055832
Cloud_Mesh_Distance_BA_R0_R3	16,78108978	-82,43949127	-941,7520142	-0,030436
Cloud_Mesh_Distance_BA_R0_R3	16,58357048	-82,14838409	-941,8365479	-0,004983
Cloud_Mesh_Distance_BA_R0_R3	16,61755371	-82,23683929	-941,9011231	-0,011867
Cloud_Mesh_Distance_BA_R0_R3	16,68947792	-82,3584671	-941,9116211	-0,025397
Cloud_Mesh_Distance_BA_R0_R3	16,53388405	-81,94571686	-941,6437378	0,025928
Cloud_Mesh_Distance_BA_R0_R3	16,59584045	-82,08989716	-941,6841431	0,00632
Cloud_Mesh_Distance_BA_R0_R3	16,32731247	-81,73059845	-941,9469605	0,041014
Cloud_Mesh_Distance_BA_R0_R3	16,39140129	-81,8663559	-941,9587402	0,025371
Cloud_Mesh_Distance_BA_R0_R3	16,45244598	-81,84856415	-941,7470093	0,032548
Cloud_Mesh_Distance_BA_R0_R3	16,37388992	-81,72980499	-941,8134155	0,042807
Cloud_Mesh_Distance_BA_R0_R3	16,2732048	-81,65525055	-942,001709	0,04235
Cloud_Mesh_Distance_BA_R0_R3	16,30621338	-81,62708282	-941,8761597	0,048401
Cloud_Mesh_Distance_BA_R0_R3	23,24602127	-82,30821991	-942,1975708	0,253203
Cloud_Mesh_Distance_BA_R0_R3	23,20399857	-82,25714874	-941,9831543	0,237114
Cloud_Mesh_Distance_BA_R0_R3	23,15824127	-82,06879425	-942,0289307	0,131797
Cloud_Mesh_Distance_BA_R0_R3	16,14895439	-81,20776367	-941,8948975	0,043772
Cloud_Mesh_Distance_BA_R0_R3	16,09638596	-81,27396393	-942,0714111	0,039618
Cloud_Mesh_Distance_BA_R0_R3	16,2941761	-81,54389191	-941,8167114	0,050972
Cloud_Mesh_Distance_BA_R0_R3	16,20320511	-81,15494537	-941,7317505	0,053436
Cloud_Mesh_Distance_BA_R0_R3	23,12026978	-81,70549774	-942,0957642	-0,009058
Cloud_Mesh_Distance_BA_R0_R3	23,12703323	-81,85391998	-942,0481567	0,043337
Cloud_Mesh_Distance_BA_R0_R3	23,13735199	-81,88544464	-942,1630859	0,058415
Cloud_Mesh_Distance_BA_R0_R3	23,14207077	-81,95438385	-942,0828857	0,079983
Cloud_Mesh_Distance_BA_R0_R3	16,14294434	-81,07207489	-941,8217163	0,042638
Cloud_Mesh_Distance_BA_R0_R3	16,1102829	-80,92707062	-941,8167725	0,037234
Cloud_Mesh_Distance_BA_R0_R3	16,03536034	-80,87510681	-941,9553223	0,022079
Cloud_Mesh_Distance_BA_R0_R3	16,07506752	-80,72544861	-941,7982788	0,027755
Cloud_Mesh_Distance_BA_R0_R3	16,00369263	-80,59632111	-941,8942261	0,006604
Cloud_Mesh_Distance_BA_R0_R3	15,91704178	-80,49446106	-942,0197144	-0,011575
Cloud_Mesh_Distance_BA_R0_R3	23,13570023	-81,1855545	-942,2652588	-0,124078
Cloud_Mesh_Distance_BA_R0_R3	15,96566582	-80,29191589	-941,8642578	0,004803
Cloud_Mesh_Distance_BA_R0_R3	15,99885845	-80,44947052	-941,8496094	0,010891
Cloud_Mesh_Distance_BA_R0_R3	23,15028763	-80,99390411	-942,2689819	-0,143786
Cloud_Mesh_Distance_BA_R0_R3	23,17733002	-80,77631378	-942,0689087	-0,174261
Cloud_Mesh_Distance_BA_R0_R3	15,84612656	-79,98239899	-942,0106201	-0,032628
Cloud_Mesh_Distance_BA_R0_R3	15,87494183	-79,80418396	-941,9469605	-0,026246
Cloud_Mesh_Distance_BA_R0_R3	15,89627647	-80,13134003	-941,9515381	-0,015791
Cloud_Mesh_Distance_BA_R0_R3	15,93233585	-79,94037628	-941,8594971	-0,004762
Cloud_Mesh_Distance_BA_R0_R3	23,18166733	-80,58307648	-942,0595703	-0,16794
Cloud_Mesh_Distance_BA_R0_R3	23,15802956	-80,43279266	-942,2582398	-0,148133
Cloud_Mesh_Distance_BA_R0_R3	15,89671707	-79,65557098	-941,9067993	-0,026673
Cloud_Mesh_Distance_BA_R0_R3	15,87476349	-79,51103973	-941,9449463	-0,037848
Cloud_Mesh_Distance_BA_R0_R3	15,82436752	-79,67446136	-942,0210571	-0,044876
Cloud_Mesh_Distance_BA_R0_R3	15,78136063	-79,53517151	-942,0838013	-0,057241
Cloud_Mesh_Distance_BA_R0_R3	23,11194229	-80,09414673	-942,1906128	-0,123405

Cloud_Mesh_Distance_BA_R0_R3	23,09341431	-80,11898804	-942,4124146	-0,114883
Cloud_Mesh_Distance_BA_R0_R3	23,13861084	-80,25759125	-942,2427368	-0,132649
Cloud_Mesh_Distance_BA_R0_R3	23,14810371	-80,23789215	-942,0875244	-0,14016
Cloud_Mesh_Distance_BA_R0_R3	23,05805588	-79,95475769	-942,3722534	-0,105777
Cloud_Mesh_Distance_BA_R0_R3	23,07168961	-79,93796539	-942,1970215	-0,106173
Cloud_Mesh_Distance_BA_R0_R3	15,82386684	-79,33718109	-942,0281372	-0,055133
Cloud_Mesh_Distance_BA_R0_R3	15,83955002	-79,09455109	-942,0231934	-0,055261
Cloud_Mesh_Distance_BA_R0_R3	15,91293812	-79,21489716	-941,9066162	-0,037733
Cloud_Mesh_Distance_BA_R0_R3	23,01683426	-79,79441071	-942,3068237	-0,08806
Cloud_Mesh_Distance_BA_R0_R3	15,91112709	-78,89994049	-941,9467163	-0,041222
Cloud_Mesh_Distance_BA_R0_R3	15,92949009	-79,05791473	-941,8988037	-0,03578
Cloud_Mesh_Distance_BA_R0_R3	22,96591187	-79,65181732	-942,3095093	-0,067983
Cloud_Mesh_Distance_BA_R0_R3	23,05194092	-79,85045624	-942,1176148	-0,09897
Cloud_Mesh_Distance_BA_R0_R3	22,9444046	-79,57289886	-942,2247315	-0,058581
Cloud_Mesh_Distance_BA_R0_R3	22,90768051	-79,5362854	-942,3930054	-0,058784
Cloud_Mesh_Distance_BA_R0_R3	23,00023079	-79,69901276	-942,1347046	-0,074601
Cloud_Mesh_Distance_BA_R0_R3	15,94678593	-78,69944	-941,9330444	-0,034674
Cloud_Mesh_Distance_BA_R0_R3	22,84902	-79,40505219	-942,3930664	-0,045537
Cloud_Mesh_Distance_BA_R0_R3	15,98630524	-78,42597198	-941,9528198	-0,028936
Cloud_Mesh_Distance_BA_R0_R3	15,98122311	-78,5575943	-941,9199219	-0,028528
Cloud_Mesh_Distance_BA_R0_R3	15,89155102	-78,54517365	-942,0474243	-0,043352
Cloud_Mesh_Distance_BA_R0_R3	22,6759243	-79,01480865	-942,1569214	0,007581
Cloud_Mesh_Distance_BA_R0_R3	22,74645233	-79,14469147	-942,1791382	-0,008526
Cloud_Mesh_Distance_BA_R0_R3	15,91324425	-78,31838226	-942,0928345	-0,040887
Cloud_Mesh_Distance_BA_R0_R3	15,90812111	-78,11457062	-942,1929321	-0,043825
Cloud_Mesh_Distance_BA_R0_R3	16,01800919	-78,28423309	-941,963562	-0,026055
Cloud_Mesh_Distance_BA_R0_R3	16,10394287	-78,20516968	-941,8851318	-0,017384
Cloud_Mesh_Distance_BA_R0_R3	16,06206512	-77,93487549	-942,0849609	-0,028599
Cloud_Mesh_Distance_BA_R0_R3	16,01672173	-78,11322784	-942,0443726	-0,029683
Cloud_Mesh_Distance_BA_R0_R3	22,52648926	-78,78832245	-942,215271	0,025242
Cloud_Mesh_Distance_BA_R0_R3	22,45166588	-78,70134735	-942,3181763	0,022472
Cloud_Mesh_Distance_BA_R0_R3	15,97515678	-77,95104218	-942,1921387	-0,035927
Cloud_Mesh_Distance_BA_R0_R3	16,13320541	-77,79895782	-942,0808716	-0,023356
Cloud_Mesh_Distance_BA_R0_R3	22,35541153	-78,60647583	-942,4682007	0,025035
Cloud_Mesh_Distance_BA_R0_R3	22,27075005	-78,44876862	-942,145874	0,031067
Cloud_Mesh_Distance_BA_R0_R3	16,37388229	-77,46562958	-942,0508423	-0,012477
Cloud_Mesh_Distance_BA_R0_R3	16,36719322	-77,64862823	-941,9144898	-0,016802
Cloud_Mesh_Distance_BA_R0_R3	16,21971893	-77,58150482	-942,1327515	-0,019174
Cloud_Mesh_Distance_BA_R0_R3	22,00453949	-78,17948151	-942,3446045	0,01471
Cloud_Mesh_Distance_BA_R0_R3	22,09095001	-78,27936554	-942,3822632	0,017687
Cloud_Mesh_Distance_BA_R0_R3	16,47159576	-77,45755768	-941,9554443	-0,011856
Cloud_Mesh_Distance_BA_R0_R3	16,4721508	-77,35928345	-942,0379028	-0,010665
Cloud_Mesh_Distance_BA_R0_R3	21,82990265	-77,98621368	-942,2939453	0,008649
Cloud_Mesh_Distance_BA_R0_R3	21,91180801	-78,05939484	-942,1514893	0,01216
Cloud_Mesh_Distance_BA_R0_R3	22,00628853	-78,16123199	-942,1802368	0,016935
Cloud_Mesh_Distance_BA_R0_R3	21,93027115	-78,09194183	-942,286499	0,010098
Cloud_Mesh_Distance_BA_R0_R3	16,2809906	-77,37449646	-942,2299805	-0,009333
Cloud_Mesh_Distance_BA_R0_R3	16,38361359	-77,20407104	-942,2709961	0,001401
Cloud_Mesh_Distance_BA_R0_R3	16,37976074	-77,31587219	-942,171875	-0,008438
Cloud_Mesh_Distance_BA_R0_R3	16,55439758	-77,4144516	-941,9093628	-0,014367

Cloud_Mesh_Distance_BA_R0_R3	21,6160965	-77,77289581	-942,4176636	-0,000015
Cloud_Mesh_Distance_BA_R0_R3	21,5197525	-77,67671967	-942,4727173	0,001514
Cloud_Mesh_Distance_BA_R0_R3	21,54979324	-77,69300079	-942,3232422	-0,000452
Cloud_Mesh_Distance_BA_R0_R3	21,74576759	-77,8901062	-942,2139893	0,005797
Cloud_Mesh_Distance_BA_R0_R3	16,71303558	-77,12029266	-942,0286865	-0,000994
Cloud_Mesh_Distance_BA_R0_R3	16,46575165	-77,24913025	-942,1428833	-0,001868
Cloud_Mesh_Distance_BA_R0_R3	21,52230644	-77,65608215	-942,1623535	0,006214
Cloud_Mesh_Distance_BA_R0_R3	16,92499161	-76,91535187	-942,0627441	0,011178
Cloud_Mesh_Distance_BA_R0_R3	16,84329414	-76,82249451	-942,2470093	0,018631
Cloud_Mesh_Distance_BA_R0_R3	16,82104874	-77,00843048	-942,0472412	0,006923
Cloud_Mesh_Distance_BA_R0_R3	16,72203827	-77,00821686	-942,1380615	0,007463
Cloud_Mesh_Distance_BA_R0_R3	21,13090897	-77,26987457	-942,2755737	0,005897
Cloud_Mesh_Distance_BA_R0_R3	21,23319626	-77,37227631	-942,3614502	0,00044
Cloud_Mesh_Distance_BA_R0_R3	21,33472061	-77,46776581	-942,2574463	0,002718
Cloud_Mesh_Distance_BA_R0_R3	16,94124985	-76,82889557	-942,1549072	0,014851
Cloud_Mesh_Distance_BA_R0_R3	17,29577827	-76,6735611	-942,0874023	0,022529
Cloud_Mesh_Distance_BA_R0_R3	20,92202759	-77,08463287	-942,3311768	0,005011
Cloud_Mesh_Distance_BA_R0_R3	21,01772308	-77,16527557	-942,2006226	0,010446
Cloud_Mesh_Distance_BA_R0_R3	17,08079147	-76,67064667	-942,2549439	0,023779
Cloud_Mesh_Distance_BA_R0_R3	17,18569946	-76,67632294	-942,1660156	0,019376
Cloud_Mesh_Distance_BA_R0_R3	17,59193802	-76,46403503	-942,2158203	0,028227
Cloud_Mesh_Distance_BA_R0_R3	17,5596199	-76,55594635	-942,0852051	0,027621
Cloud_Mesh_Distance_BA_R0_R3	17,71663094	-76,43173981	-942,1939087	0,031634
Cloud_Mesh_Distance_BA_R0_R3	20,60160065	-76,83666229	-942,4680786	0,001586
Cloud_Mesh_Distance_BA_R0_R3	20,71596909	-76,91981506	-942,3648071	0,006327
Cloud_Mesh_Distance_BA_R0_R3	20,82078934	-76,99991608	-942,2675781	0,013059
Cloud_Mesh_Distance_BA_R0_R3	17,447155	-76,50527191	-942,2420654	0,025924
Cloud_Mesh_Distance_BA_R0_R3	17,48431396	-76,42249298	-942,3614502	0,027064
Cloud_Mesh_Distance_BA_R0_R3	17,64916229	-76,37807465	-942,3335571	0,027714
Cloud_Mesh_Distance_BA_R0_R3	17,80605316	-76,46215057	-942,088562	0,033259
Cloud_Mesh_Distance_BA_R0_R3	17,94250107	-76,42806244	-942,0748901	0,039157
Cloud_Mesh_Distance_BA_R0_R3	20,1157074	-76,52991486	-942,4232788	0,019814
Cloud_Mesh_Distance_BA_R0_R3	20,13376617	-76,59008789	-942,1005859	0,033021
Cloud_Mesh_Distance_BA_R0_R3	20,32240677	-76,67646027	-942,1472778	0,028225
Cloud_Mesh_Distance_BA_R0_R3	20,60415268	-76,83934784	-942,2768555	0,014332
Cloud_Mesh_Distance_BA_R0_R3	20,48485756	-76,75480652	-942,387085	0,009328
Cloud_Mesh_Distance_BA_R0_R3	17,83963776	-76,36347961	-942,2476196	0,03342
Cloud_Mesh_Distance_BA_R0_R3	18,03180504	-76,36728668	-942,145813	0,040657
Cloud_Mesh_Distance_BA_R0_R3	18,23149109	-76,26244354	-942,3063355	0,033473
Cloud_Mesh_Distance_BA_R0_R3	18,02192307	-76,28427887	-942,3236084	0,034508
Cloud_Mesh_Distance_BA_R0_R3	18,18212509	-76,33027649	-942,1654663	0,038968
Cloud_Mesh_Distance_BA_R0_R3	18,42328644	-76,253685	-942,2982788	0,024147
Cloud_Mesh_Distance_BA_R0_R3	18,5527153	-76,3085022	-942,1503296	0,026756
Cloud_Mesh_Distance_BA_R0_R3	18,62041473	-76,25418854	-942,2938232	0,0169
Cloud_Mesh_Distance_BA_R0_R3	19,01629257	-76,27495575	-942,2841187	0,008919
Cloud_Mesh_Distance_BA_R0_R3	18,75442123	-76,31124115	-942,1438599	0,01646
Cloud_Mesh_Distance_BA_R0_R3	19,18836975	-76,29013824	-942,2827148	0,014659
Cloud_Mesh_Distance_BA_R0_R3	19,15110016	-76,32923126	-942,1513062	0,016428
Cloud_Mesh_Distance_BA_R0_R3	19,30499077	-76,29917145	-942,296814	0,016199
Cloud_Mesh_Distance_BA_R0_R3	19,39953613	-76,3218689	-942,2640381	0,022764

Cloud_Mesh_Distance_BA_R0_R3	19,33400917	-76,33945465	-942,1762085	0,021496
Cloud_Mesh_Distance_BA_R0_R3	19,95533371	-76,4721756	-942,2926636	0,032045
Cloud_Mesh_Distance_BA_R0_R3	19,97859955	-76,46175385	-942,4295044	0,025861
Cloud_Mesh_Distance_BA_R0_R3	20,06110764	-76,52126312	-942,2818604	0,031484
Cloud_Mesh_Distance_BA_R0_R3	19,35693359	-76,28308868	-942,392334	0,016951
Cloud_Mesh_Distance_BA_R0_R3	19,47199059	-76,35709381	-942,1842651	0,028042
Cloud_Mesh_Distance_BA_R0_R3	19,50452995	-76,33516693	-942,2858276	0,027683
Cloud_Mesh_Distance_BA_R0_R3	19,65298462	-76,3664093	-942,3013916	0,029464
Cloud_Mesh_Distance_BA_R0_R3	18,85991669	-84,28229523	-941,5710449	-0,051896
Cloud_Mesh_Distance_BA_R0_R3	19,21133041	-84,37247467	-941,4795532	-0,079883
Cloud_Mesh_Distance_BA_R0_R3	19,65177155	-84,47306061	-941,473877	-0,095403
Cloud_Mesh_Distance_BA_R0_R3	18,4715004	-84,12070465	-941,6732788	-0,030579
Cloud_Mesh_Distance_BA_R0_R3	18,46905518	-84,08039093	-941,4768066	-0,030419
Cloud_Mesh_Distance_BA_R0_R3	18,68719482	-84,2589035	-941,7910767	-0,037047
Cloud_Mesh_Distance_BA_R0_R3	18,72719383	-84,24280548	-941,6340332	-0,042531
Cloud_Mesh_Distance_BA_R0_R3	18,96621132	-84,3013382	-941,5014648	-0,06328
Cloud_Mesh_Distance_BA_R0_R3	18,137043	-83,84705353	-941,4418335	-0,03992
Cloud_Mesh_Distance_BA_R0_R3	18,23252678	-83,9489975	-941,5766602	-0,031085
Cloud_Mesh_Distance_BA_R0_R3	18,09081841	-83,85697174	-941,684021	-0,047134
Cloud_Mesh_Distance_BA_R0_R3	18,04724503	-83,80191803	-941,5737305	-0,048101
Cloud_Mesh_Distance_BA_R0_R3	18,34234238	-84,01016998	-941,5023193	-0,028922
Cloud_Mesh_Distance_BA_R0_R3	17,77082443	-83,56635284	-941,6226807	-0,070085
Cloud_Mesh_Distance_BA_R0_R3	17,88760376	-83,64199066	-941,4867554	-0,059244
Cloud_Mesh_Distance_BA_R0_R3	18,00809479	-83,75287628	-941,4993897	-0,047582
Cloud_Mesh_Distance_BA_R0_R3	17,95724869	-83,74187469	-941,6633911	-0,057849
Cloud_Mesh_Distance_BA_R0_R3	17,84303093	-83,64530182	-941,6872559	-0,068535
Cloud_Mesh_Distance_BA_R0_R3	17,70931625	-83,45311737	-941,4246826	-0,070235
Cloud_Mesh_Distance_BA_R0_R3	17,66424179	-83,48778534	-941,7199707	-0,07575
Cloud_Mesh_Distance_BA_R0_R3	17,70458603	-83,4895401	-941,564148	-0,070238
Cloud_Mesh_Distance_BA_R0_R3	17,48093414	-83,30330658	-941,6936646	-0,080589
Cloud_Mesh_Distance_BA_R0_R3	17,60310745	-83,39099884	-941,5681763	-0,074984
Cloud_Mesh_Distance_BA_R0_R3	17,5614624	-83,39647675	-941,7486572	-0,078386
Cloud_Mesh_Distance_BA_R0_R3	17,2287178	-83,01241302	-941,642273	-0,070592
Cloud_Mesh_Distance_BA_R0_R3	17,32509804	-83,11344147	-941,6130371	-0,07249
Cloud_Mesh_Distance_BA_R0_R3	17,24983406	-82,95987701	-941,4387207	-0,070266
Cloud_Mesh_Distance_BA_R0_R3	17,3914032	-83,15753937	-941,5278931	-0,073996
Cloud_Mesh_Distance_BA_R0_R3	17,33032227	-83,07163239	-941,480835	-0,074381
Cloud_Mesh_Distance_BA_R0_R3	16,82274246	-82,43785858	-941,5974731	-0,028605
Cloud_Mesh_Distance_BA_R0_R3	16,96395302	-82,60340118	-941,4888306	-0,044935
Cloud_Mesh_Distance_BA_R0_R3	16,94117355	-82,65619659	-941,6904297	-0,048945
Cloud_Mesh_Distance_BA_R0_R3	16,71932983	-82,34175873	-941,7566528	-0,022831
Cloud_Mesh_Distance_BA_R0_R3	16,83543777	-82,39479828	-941,4654541	-0,023538
Cloud_Mesh_Distance_BA_R0_R3	16,66106606	-82,24063873	-941,7504273	-0,012815
Cloud_Mesh_Distance_BA_R0_R3	16,49376488	-81,82984161	-941,5993042	0,033802
Cloud_Mesh_Distance_BA_R0_R3	16,4317131	-81,70549774	-941,6220093	0,042999
Cloud_Mesh_Distance_BA_R0_R3	16,51091385	-81,76967621	-941,473999	0,036323
Cloud_Mesh_Distance_BA_R0_R3	23,18606567	-82,30925751	-941,8183594	0,255915
Cloud_Mesh_Distance_BA_R0_R3	16,38941956	-81,5318985	-941,5570679	0,052524
Cloud_Mesh_Distance_BA_R0_R3	23,16016769	-82,22286224	-941,8288574	0,203915
Cloud_Mesh_Distance_BA_R0_R3	23,14767838	-82,13303375	-941,87677	0,159022

Cloud_Mesh_Distance_BA_R0_R3	16,21794128	-81,3208313	-941,8106079	0,050996
Cloud_Mesh_Distance_BA_R0_R3	16,29876328	-81,44681549	-941,7138672	0,051786
Cloud_Mesh_Distance_BA_R0_R3	16,34727097	-81,59824371	-941,7329102	0,050832
Cloud_Mesh_Distance_BA_R0_R3	23,13439178	-81,9651413	-941,9901733	0,083128
Cloud_Mesh_Distance_BA_R0_R3	23,12882614	-82,01706696	-941,8987427	0,103908
Cloud_Mesh_Distance_BA_R0_R3	16,26013565	-81,06712341	-941,5481567	0,057626
Cloud_Mesh_Distance_BA_R0_R3	23,1192894	-81,91426849	-941,9242554	0,061521
Cloud_Mesh_Distance_BA_R0_R3	16,18183708	-80,99214172	-941,6876221	0,052437
Cloud_Mesh_Distance_BA_R0_R3	23,1057682	-81,53601837	-941,8529053	-0,078113
Cloud_Mesh_Distance_BA_R0_R3	23,11818695	-81,55706024	-942,0663452	-0,055028
Cloud_Mesh_Distance_BA_R0_R3	23,12800598	-81,35939789	-942,0592651	-0,105076
Cloud_Mesh_Distance_BA_R0_R3	16,06901932	-80,5087738	-941,725769	0,033059
Cloud_Mesh_Distance_BA_R0_R3	23,14543533	-81,16138458	-942,0653076	-0,143922
Cloud_Mesh_Distance_BA_R0_R3	16,03979301	-80,21870422	-941,7022705	0,029157
Cloud_Mesh_Distance_BA_R0_R3	16,03520966	-80,36125183	-941,7489014	0,026991
Cloud_Mesh_Distance_BA_R0_R3	16,10052109	-80,34204865	-941,6084595	0,045487
Cloud_Mesh_Distance_BA_R0_R3	23,14892578	-81,12690735	-941,8746338	-0,162228
Cloud_Mesh_Distance_BA_R0_R3	23,16356468	-80,96842194	-942,071167	-0,164478
Cloud_Mesh_Distance_BA_R0_R3	16,0495491	-80,07402802	-941,6548462	0,03765
Cloud_Mesh_Distance_BA_R0_R3	15,98489666	-80,09345245	-941,7842407	0,010622
Cloud_Mesh_Distance_BA_R0_R3	23,17022705	-80,93793488	-941,8743897	-0,186379
Cloud_Mesh_Distance_BA_R0_R3	16,02526474	-79,91265106	-941,6846924	0,027324
Cloud_Mesh_Distance_BA_R0_R3	23,18838501	-80,54125977	-941,8422852	-0,18427
Cloud_Mesh_Distance_BA_R0_R3	15,94098568	-79,5692215	-941,8361206	-0,0145
Cloud_Mesh_Distance_BA_R0_R3	15,96380329	-79,72823334	-941,7932739	-0,002278
Cloud_Mesh_Distance_BA_R0_R3	16,05523682	-79,72049713	-941,6256104	0,03097
Cloud_Mesh_Distance_BA_R0_R3	23,16482544	-80,29733276	-941,9299927	-0,150074
Cloud_Mesh_Distance_BA_R0_R3	23,17181969	-80,3927536	-942,0459595	-0,155561
Cloud_Mesh_Distance_BA_R0_R3	15,90643215	-79,36563873	-941,902771	-0,035009
Cloud_Mesh_Distance_BA_R0_R3	15,94422722	-79,45306396	-941,8373413	-0,018978
Cloud_Mesh_Distance_BA_R0_R3	15,97492218	-79,32180786	-941,7996826	-0,016879
Cloud_Mesh_Distance_BA_R0_R3	23,13786697	-80,16316986	-942,0187378	-0,136318
Cloud_Mesh_Distance_BA_R0_R3	16,00281143	-79,12414551	-941,7807617	-0,015717
Cloud_Mesh_Distance_BA_R0_R3	16,10168839	-79,09844208	-941,6340332	0,007677
Cloud_Mesh_Distance_BA_R0_R3	16,06303024	-78,94800568	-941,7202759	-0,007595
Cloud_Mesh_Distance_BA_R0_R3	15,98266697	-78,966362	-941,8345947	-0,026445
Cloud_Mesh_Distance_BA_R0_R3	23,00014687	-79,67456818	-941,942627	-0,079057
Cloud_Mesh_Distance_BA_R0_R3	16,12524605	-78,75246429	-941,6748047	-0,003713
Cloud_Mesh_Distance_BA_R0_R3	16,01029205	-78,81412506	-941,8216553	-0,021666
Cloud_Mesh_Distance_BA_R0_R3	22,90478134	-79,43904877	-941,9488525	-0,044291
Cloud_Mesh_Distance_BA_R0_R3	22,95201492	-79,56033325	-942,064209	-0,059975
Cloud_Mesh_Distance_BA_R0_R3	16,04008293	-78,63382721	-941,8200684	-0,018067
Cloud_Mesh_Distance_BA_R0_R3	16,14149475	-78,50066376	-941,7232666	-0,006391
Cloud_Mesh_Distance_BA_R0_R3	22,82965469	-79,29064941	-942,0846558	-0,026361
Cloud_Mesh_Distance_BA_R0_R3	22,77613449	-79,17546844	-941,9909058	-0,008735
Cloud_Mesh_Distance_BA_R0_R3	22,88855362	-79,43779755	-942,2039795	-0,044516
Cloud_Mesh_Distance_BA_R0_R3	16,04401398	-78,49485016	-941,8533936	-0,019322
Cloud_Mesh_Distance_BA_R0_R3	16,07393074	-78,36852264	-941,8565064	-0,017251
Cloud_Mesh_Distance_BA_R0_R3	22,64060211	-78,94382477	-942,0705566	0,016106
Cloud_Mesh_Distance_BA_R0_R3	22,60587502	-78,91120148	-942,2237549	0,015897

Cloud_Mesh_Distance_BA_R0_R3	16,15092278	-77,99867249	-941,9329834	-0,017515
Cloud_Mesh_Distance_BA_R0_R3	22,36686325	-78,57722473	-942,2455444	0,028445
Cloud_Mesh_Distance_BA_R0_R3	22,46385574	-78,68793488	-942,1297607	0,034598
Cloud_Mesh_Distance_BA_R0_R3	16,35337448	-77,76734161	-941,8438721	-0,012788
Cloud_Mesh_Distance_BA_R0_R3	16,26383972	-77,80466461	-941,9192505	-0,014847
Cloud_Mesh_Distance_BA_R0_R3	22,09749031	-78,24983978	-942,0673218	0,024854
Cloud_Mesh_Distance_BA_R0_R3	22,17184639	-78,33604431	-942,1385498	0,026597
Cloud_Mesh_Distance_BA_R0_R3	22,17517471	-78,35666656	-942,2799072	0,025102
Cloud_Mesh_Distance_BA_R0_R3	22,3815918	-78,57341003	-942,0466309	0,040139
Cloud_Mesh_Distance_BA_R0_R3	16,44643784	-77,54396057	-941,9121704	-0,01591
Cloud_Mesh_Distance_BA_R0_R3	16,42278481	-77,75216675	-941,7804565	-0,013428
Cloud_Mesh_Distance_BA_R0_R3	21,98630142	-78,12987518	-941,9991455	0,017993
Cloud_Mesh_Distance_BA_R0_R3	22,09328842	-78,25999451	-942,2310181	0,022008
Cloud_Mesh_Distance_BA_R0_R3	16,51740837	-77,51293182	-941,8644409	-0,016352
Cloud_Mesh_Distance_BA_R0_R3	21,8189373	-77,96039581	-942,1196289	0,006872
Cloud_Mesh_Distance_BA_R0_R3	21,71343803	-77,85036469	-942,0591431	0,007111
Cloud_Mesh_Distance_BA_R0_R3	21,88731956	-78,02796936	-942,0274048	0,013257
Cloud_Mesh_Distance_BA_R0_R3	16,66164017	-77,38556671	-941,8319702	-0,014389
Cloud_Mesh_Distance_BA_R0_R3	16,59815598	-77,26867676	-941,9956055	-0,008252
Cloud_Mesh_Distance_BA_R0_R3	21,60915756	-77,74394989	-942,0735474	0,006698
Cloud_Mesh_Distance_BA_R0_R3	21,62008286	-77,76021576	-941,9107666	0,003781
Cloud_Mesh_Distance_BA_R0_R3	21,64091873	-77,78177643	-942,2304077	0,002774
Cloud_Mesh_Distance_BA_R0_R3	16,71178055	-77,23204041	-941,9226074	-0,006825
Cloud_Mesh_Distance_BA_R0_R3	16,90939713	-77,10366058	-941,8699341	0,002459
Cloud_Mesh_Distance_BA_R0_R3	21,3368988	-77,46897888	-942,0762329	0,004907
Cloud_Mesh_Distance_BA_R0_R3	21,41746902	-77,54897308	-942,1657715	0,006445
Cloud_Mesh_Distance_BA_R0_R3	21,54010773	-77,67519379	-942,0055542	0,006
Cloud_Mesh_Distance_BA_R0_R3	21,44178963	-77,57650757	-942,0056763	0,004991
Cloud_Mesh_Distance_BA_R0_R3	16,92021179	-77,0087204	-941,9603882	0,007398
Cloud_Mesh_Distance_BA_R0_R3	17,0344162	-76,83493805	-942,0687866	0,015022
Cloud_Mesh_Distance_BA_R0_R3	17,0164566	-77,01040649	-941,8764648	0,009198
Cloud_Mesh_Distance_BA_R0_R3	17,13169861	-76,84937286	-941,9723511	0,01796
Cloud_Mesh_Distance_BA_R0_R3	21,12687683	-77,26663208	-942,0744629	0,012188
Cloud_Mesh_Distance_BA_R0_R3	21,23594284	-77,36846161	-942,168457	0,00636
Cloud_Mesh_Distance_BA_R0_R3	21,24015427	-77,3789978	-941,9838257	0,009155
Cloud_Mesh_Distance_BA_R0_R3	17,16286087	-76,76171112	-942,0627441	0,01885
Cloud_Mesh_Distance_BA_R0_R3	17,24071503	-76,88380432	-941,8450317	0,017696
Cloud_Mesh_Distance_BA_R0_R3	17,46520424	-76,6541214	-941,9973755	0,024955
Cloud_Mesh_Distance_BA_R0_R3	17,60754395	-76,62742615	-941,9453735	0,028798
Cloud_Mesh_Distance_BA_R0_R3	20,82257843	-77,00347137	-942,1382446	0,020391
Cloud_Mesh_Distance_BA_R0_R3	20,86858177	-77,04385376	-942,0549927	0,02469
Cloud_Mesh_Distance_BA_R0_R3	20,90655708	-77,07024384	-942,1593628	0,016855
Cloud_Mesh_Distance_BA_R0_R3	17,38219833	-76,58698273	-942,1554565	0,022752
Cloud_Mesh_Distance_BA_R0_R3	17,68371582	-76,49453735	-942,1065064	0,030382
Cloud_Mesh_Distance_BA_R0_R3	17,73648453	-76,55760956	-941,9732666	0,03484
Cloud_Mesh_Distance_BA_R0_R3	20,60927773	-76,85652924	-942,072937	0,027238
Cloud_Mesh_Distance_BA_R0_R3	20,72628784	-76,92977142	-942,1722412	0,018052
Cloud_Mesh_Distance_BA_R0_R3	20,78499985	-76,98055267	-942,0466919	0,025494
Cloud_Mesh_Distance_BA_R0_R3	18,01176834	-76,46115875	-941,9841919	0,041989
Cloud_Mesh_Distance_BA_R0_R3	19,96775627	-76,56908417	-941,9330444	0,036771

Cloud_Mesh_Distance_BA_R0_R3	20,44325638	-76,76352692	-942,0158081	0,03195
Cloud_Mesh_Distance_BA_R0_R3	20,47497177	-76,75915527	-942,2006226	0,020802
Cloud_Mesh_Distance_BA_R0_R3	18,04672241	-76,41912079	-942,041626	0,041077
Cloud_Mesh_Distance_BA_R0_R3	18,3605423	-76,31357574	-942,1586304	0,033951
Cloud_Mesh_Distance_BA_R0_R3	18,95723152	-76,3188324	-942,144165	0,016056
Cloud_Mesh_Distance_BA_R0_R3	18,88637161	-76,37328339	-941,9976196	0,021821
Cloud_Mesh_Distance_BA_R0_R3	19,09320068	-76,37906647	-942,0081177	0,019409
Cloud_Mesh_Distance_BA_R0_R3	19,29076767	-76,38973999	-942,0232544	0,022375
Cloud_Mesh_Distance_BA_R0_R3	19,60052872	-76,38860321	-942,1655884	0,032706
Cloud_Mesh_Distance_BA_R0_R3	19,68623734	-76,45066071	-942,0357666	0,03443
Cloud_Mesh_Distance_BA_R0_R3	19,9383812	-76,49435425	-942,1573486	0,034821
Cloud_Mesh_Distance_BA_R0_R3	19,83152199	-76,41768646	-942,3265381	0,030241
Cloud_Mesh_Distance_BA_R0_R3	19,76154518	-76,43108368	-942,164856	0,036078
Cloud_Mesh_Distance_BA_R0_R3	18,90235138	-84,2642746	-941,4512329	-0,06108
Cloud_Mesh_Distance_BA_R0_R3	19,40247726	-84,37000275	-941,34198	-0,09582
Cloud_Mesh_Distance_BA_R0_R3	19,42083359	-84,43990326	-941,5092773	-0,079384
Cloud_Mesh_Distance_BA_R0_R3	19,81659698	-84,43541718	-941,3366089	-0,077178
Cloud_Mesh_Distance_BA_R0_R3	20,30974197	-84,45186615	-941,3743286	-0,03106
Cloud_Mesh_Distance_BA_R0_R3	20,76570892	-84,45162201	-941,4785156	0,119732
Cloud_Mesh_Distance_BA_R0_R3	18,39328194	-84,01876068	-941,3994141	-0,028441
Cloud_Mesh_Distance_BA_R0_R3	18,48283005	-84,04947662	-941,321106	-0,030902
Cloud_Mesh_Distance_BA_R0_R3	18,64664078	-84,1778183	-941,5148315	-0,040387
Cloud_Mesh_Distance_BA_R0_R3	18,79741859	-84,23731232	-941,4981079	-0,051641
Cloud_Mesh_Distance_BA_R0_R3	19,06844711	-84,32605743	-941,4727173	-0,072131
Cloud_Mesh_Distance_BA_R0_R3	19,03523636	-84,28211212	-941,3670654	-0,078652
Cloud_Mesh_Distance_BA_R0_R3	19,07507324	-84,24289703	-941,2172852	-0,091965
Cloud_Mesh_Distance_BA_R0_R3	19,27325821	-84,33576202	-941,3338013	-0,097156
Cloud_Mesh_Distance_BA_R0_R3	19,22283554	-84,28887177	-941,2391968	-0,101691
Cloud_Mesh_Distance_BA_R0_R3	19,16082764	-84,32202911	-941,3779907	-0,086497
Cloud_Mesh_Distance_BA_R0_R3	20,42182541	-84,39415741	-941,281311	-0,021572
Cloud_Mesh_Distance_BA_R0_R3	20,64396286	-84,43943024	-941,4263306	0,03469
Cloud_Mesh_Distance_BA_R0_R3	20,48913193	-84,44519806	-941,3980103	-0,004473
Cloud_Mesh_Distance_BA_R0_R3	20,58259964	-84,36737823	-941,2766113	0,001649
Cloud_Mesh_Distance_BA_R0_R3	20,79133224	-84,37592316	-941,3550415	0,043672
Cloud_Mesh_Distance_BA_R0_R3	20,93103409	-84,43198395	-941,4805298	0,091001
Cloud_Mesh_Distance_BA_R0_R3	21,06705856	-84,28694916	-941,2957153	0,094281
Cloud_Mesh_Distance_BA_R0_R3	20,96138954	-84,35094452	-941,3614502	0,079462
Cloud_Mesh_Distance_BA_R0_R3	21,12525368	-84,44579315	-941,5314941	0,13269
Cloud_Mesh_Distance_BA_R0_R3	21,64273453	-84,40818024	-941,4830322	0,267554
Cloud_Mesh_Distance_BA_R0_R3	18,29293823	-83,95624542	-941,4099121	-0,027795
Cloud_Mesh_Distance_BA_R0_R3	18,39636803	-83,98757172	-941,277771	-0,02862
Cloud_Mesh_Distance_BA_R0_R3	21,09889603	-84,36249542	-941,4087524	0,113544
Cloud_Mesh_Distance_BA_R0_R3	21,389534	-84,29369354	-941,3636475	0,183756
Cloud_Mesh_Distance_BA_R0_R3	21,23336792	-84,28348541	-941,3290405	0,138289
Cloud_Mesh_Distance_BA_R0_R3	21,44795609	-84,37741852	-941,461853	0,216634
Cloud_Mesh_Distance_BA_R0_R3	21,26750183	-84,37030792	-941,4447022	0,16139
Cloud_Mesh_Distance_BA_R0_R3	17,62566566	-83,3340683	-941,3269043	-0,079921
Cloud_Mesh_Distance_BA_R0_R3	17,77813339	-83,53816986	-941,4828491	-0,065459
Cloud_Mesh_Distance_BA_R0_R3	17,51709366	-83,2204361	-941,3305664	-0,078902
Cloud_Mesh_Distance_BA_R0_R3	17,48915672	-83,25232697	-941,4988403	-0,076616

Cloud_Mesh_Distance_BA_R0_R3	17,18861771	-82,85942841	-941,380188	-0,064301
Cloud_Mesh_Distance_BA_R0_R3	17,36976242	-83,03011322	-941,2699585	-0,079268
Cloud_Mesh_Distance_BA_R0_R3	17,42780685	-83,1476059	-941,3934937	-0,076555
Cloud_Mesh_Distance_BA_R0_R3	17,12780762	-82,85858917	-941,5626831	-0,061265
Cloud_Mesh_Distance_BA_R0_R3	16,88174629	-82,4905777	-941,5108643	-0,032249
Cloud_Mesh_Distance_BA_R0_R3	16,92338371	-82,48038483	-941,3597412	-0,029612
Cloud_Mesh_Distance_BA_R0_R3	17,09555244	-82,7360611	-941,3765259	-0,05813
Cloud_Mesh_Distance_BA_R0_R3	17,02703667	-82,72914886	-941,5722046	-0,052414
Cloud_Mesh_Distance_BA_R0_R3	16,7522316	-82,32956696	-941,6123657	-0,018975
Cloud_Mesh_Distance_BA_R0_R3	16,68529129	-82,18018341	-941,5491333	0,000869
Cloud_Mesh_Distance_BA_R0_R3	16,7766037	-82,2720108	-941,4190674	-0,009793
Cloud_Mesh_Distance_BA_R0_R3	16,72195625	-82,13549042	-941,3533936	0,004857
Cloud_Mesh_Distance_BA_R0_R3	16,6831665	-81,99880219	-941,2628784	0,016544
Cloud_Mesh_Distance_BA_R0_R3	16,54833031	-81,88240814	-941,5075073	0,030346
Cloud_Mesh_Distance_BA_R0_R3	16,62010956	-82,01294708	-941,4776611	0,016307
Cloud_Mesh_Distance_BA_R0_R3	16,47115898	-81,6279068	-941,4307861	0,047486
Cloud_Mesh_Distance_BA_R0_R3	23,13422966	-82,24684906	-941,7233276	0,20245
Cloud_Mesh_Distance_BA_R0_R3	16,35365105	-81,36235809	-941,5102539	0,057824
Cloud_Mesh_Distance_BA_R0_R3	23,08771515	-82,12519073	-941,670105	0,133235
Cloud_Mesh_Distance_BA_R0_R3	23,12710571	-82,15937042	-941,7702637	0,160453
Cloud_Mesh_Distance_BA_R0_R3	23,11043549	-82,0558548	-941,7826538	0,111741
Cloud_Mesh_Distance_BA_R0_R3	16,27529907	-81,26031494	-941,6288452	0,056411
Cloud_Mesh_Distance_BA_R0_R3	16,32966042	-81,19913483	-941,460083	0,060911
Cloud_Mesh_Distance_BA_R0_R3	16,32532883	-81,06287384	-941,3950195	0,059244
Cloud_Mesh_Distance_BA_R0_R3	23,10523796	-81,77323151	-941,8892212	0,001484
Cloud_Mesh_Distance_BA_R0_R3	23,07267761	-81,93671417	-941,6863403	0,047854
Cloud_Mesh_Distance_BA_R0_R3	23,10560417	-81,94330597	-941,8296509	0,0666
Cloud_Mesh_Distance_BA_R0_R3	16,15797234	-80,81980133	-941,657959	0,044443
Cloud_Mesh_Distance_BA_R0_R3	16,22155762	-80,90387726	-941,553894	0,058186
Cloud_Mesh_Distance_BA_R0_R3	23,06824112	-81,71051788	-941,663269	-0,038037
Cloud_Mesh_Distance_BA_R0_R3	16,14751053	-80,61559296	-941,5968628	0,046519
Cloud_Mesh_Distance_BA_R0_R3	16,27131653	-80,66609955	-941,3450317	0,066631
Cloud_Mesh_Distance_BA_R0_R3	16,21683121	-80,74895477	-941,4967651	0,053988
Cloud_Mesh_Distance_BA_R0_R3	23,12531662	-81,32254791	-941,8566895	-0,129369
Cloud_Mesh_Distance_BA_R0_R3	23,12230301	-81,25737762	-941,7002564	-0,152485
Cloud_Mesh_Distance_BA_R0_R3	16,24787521	-80,52211761	-941,3471069	0,07289
Cloud_Mesh_Distance_BA_R0_R3	16,1823349	-80,4308548	-941,4594116	0,066318
Cloud_Mesh_Distance_BA_R0_R3	16,12475395	-80,47426605	-941,5969238	0,049784
Cloud_Mesh_Distance_BA_R0_R3	16,21246338	-80,59752655	-941,4483643	0,065292
Cloud_Mesh_Distance_BA_R0_R3	16,10058212	-80,15651703	-941,5657349	0,050738
Cloud_Mesh_Distance_BA_R0_R3	16,09642029	-79,99500275	-941,5517578	0,052423
Cloud_Mesh_Distance_BA_R0_R3	16,1659584	-80,06707764	-941,4194946	0,07228
Cloud_Mesh_Distance_BA_R0_R3	23,18661118	-80,56269073	-941,722168	-0,198939
Cloud_Mesh_Distance_BA_R0_R3	23,1857338	-80,74112701	-941,8623047	-0,193291
Cloud_Mesh_Distance_BA_R0_R3	16,17146111	-79,70381927	-941,4106445	0,070112
Cloud_Mesh_Distance_BA_R0_R3	16,11276245	-79,59939575	-941,5296021	0,046887
Cloud_Mesh_Distance_BA_R0_R3	23,18622589	-80,51370239	-941,7503662	-0,191319
Cloud_Mesh_Distance_BA_R0_R3	23,17907143	-80,41361237	-941,8948975	-0,165372
Cloud_Mesh_Distance_BA_R0_R3	23,18443298	-80,50403595	-941,7216797	-0,193141
Cloud_Mesh_Distance_BA_R0_R3	16,02145576	-79,50806427	-941,7026367	0,010396

Cloud_Mesh_Distance_BA_R0_R3	16,19341278	-79,52840424	-941,3978272	0,06216
Cloud_Mesh_Distance_BA_R0_R3	23,16566467	-80,32534027	-941,6904297	-0,174732
Cloud_Mesh_Distance_BA_R0_R3	23,18070221	-80,42800903	-941,7641602	-0,176912
Cloud_Mesh_Distance_BA_R0_R3	23,16272354	-80,27910614	-941,8046265	-0,158079
Cloud_Mesh_Distance_BA_R0_R3	16,15136337	-79,22280121	-941,5310059	0,029161
Cloud_Mesh_Distance_BA_R0_R3	16,0615654	-79,27849579	-941,6654663	0,008563
Cloud_Mesh_Distance_BA_R0_R3	16,12432671	-79,409935	-941,538269	0,035354
Cloud_Mesh_Distance_BA_R0_R3	23,10594368	-80,01984406	-941,9844971	-0,120529
Cloud_Mesh_Distance_BA_R0_R3	23,14090919	-80,1717453	-941,7440186	-0,152738
Cloud_Mesh_Distance_BA_R0_R3	23,14499092	-80,18334198	-941,8966675	-0,143528
Cloud_Mesh_Distance_BA_R0_R3	23,05149841	-79,82657623	-941,9514771	-0,099338
Cloud_Mesh_Distance_BA_R0_R3	16,17546654	-79,08405304	-941,5245361	0,02323
Cloud_Mesh_Distance_BA_R0_R3	16,22554779	-79,0174942	-941,4671021	0,028187
Cloud_Mesh_Distance_BA_R0_R3	16,16565704	-78,94797516	-941,5714722	0,013443
Cloud_Mesh_Distance_BA_R0_R3	16,24121284	-79,14481354	-941,4105835	0,037528
Cloud_Mesh_Distance_BA_R0_R3	22,98954391	-79,64681244	-941,8175049	-0,077077
Cloud_Mesh_Distance_BA_R0_R3	23,03431702	-79,77849579	-941,7601318	-0,10014
Cloud_Mesh_Distance_BA_R0_R3	22,9529438	-79,55835724	-941,7539673	-0,061745
Cloud_Mesh_Distance_BA_R0_R3	22,96080208	-79,57061005	-941,909668	-0,066718
Cloud_Mesh_Distance_BA_R0_R3	16,22678566	-78,62751007	-941,5736084	0,008477
Cloud_Mesh_Distance_BA_R0_R3	22,83317947	-79,2841568	-941,8746948	-0,023648
Cloud_Mesh_Distance_BA_R0_R3	22,82514381	-79,28430939	-941,6713867	-0,024086
Cloud_Mesh_Distance_BA_R0_R3	22,88954353	-79,41321564	-941,7294312	-0,042071
Cloud_Mesh_Distance_BA_R0_R3	16,2442627	-78,48807526	-941,5960083	0,003345
Cloud_Mesh_Distance_BA_R0_R3	16,31006241	-78,54560852	-941,4953003	0,010362
Cloud_Mesh_Distance_BA_R0_R3	22,75529861	-79,13491821	-941,8071899	0,000098
Cloud_Mesh_Distance_BA_R0_R3	16,3010788	-78,22304535	-941,6356201	-0,001759
Cloud_Mesh_Distance_BA_R0_R3	16,21996117	-78,16603088	-941,7592773	-0,008805
Cloud_Mesh_Distance_BA_R0_R3	16,15474319	-78,31491852	-941,7738037	-0,009052
Cloud_Mesh_Distance_BA_R0_R3	16,40933609	-78,06699371	-941,5949707	-0,001904
Cloud_Mesh_Distance_BA_R0_R3	16,38247681	-78,18876648	-941,5601807	0,000769
Cloud_Mesh_Distance_BA_R0_R3	22,61626053	-78,89662933	-941,828064	0,024285
Cloud_Mesh_Distance_BA_R0_R3	22,68158913	-79,00189209	-941,8572388	0,015366
Cloud_Mesh_Distance_BA_R0_R3	22,70494843	-79,04631805	-942,0028076	0,006528
Cloud_Mesh_Distance_BA_R0_R3	22,64292145	-78,93899536	-941,9556885	0,019384
Cloud_Mesh_Distance_BA_R0_R3	16,43285751	-77,9562149	-941,6342773	-0,008496
Cloud_Mesh_Distance_BA_R0_R3	16,36055756	-77,9952774	-941,6886597	-0,0062
Cloud_Mesh_Distance_BA_R0_R3	16,32388878	-78,10302734	-941,6693726	-0,002783
Cloud_Mesh_Distance_BA_R0_R3	16,46788597	-78,02599335	-941,555542	-0,003427
Cloud_Mesh_Distance_BA_R0_R3	22,46162605	-78,67342377	-941,8269653	0,040503
Cloud_Mesh_Distance_BA_R0_R3	22,56551933	-78,82259369	-942,0219116	0,0268
Cloud_Mesh_Distance_BA_R0_R3	22,4678688	-78,6804657	-941,947876	0,04172
Cloud_Mesh_Distance_BA_R0_R3	16,27829933	-77,98729706	-941,7860107	-0,009812
Cloud_Mesh_Distance_BA_R0_R3	16,46477127	-77,84081268	-941,6749268	-0,009887
Cloud_Mesh_Distance_BA_R0_R3	16,36602402	-77,87010956	-941,760376	-0,010915
Cloud_Mesh_Distance_BA_R0_R3	16,55347633	-77,7771225	-941,6278687	-0,010851
Cloud_Mesh_Distance_BA_R0_R3	16,48633766	-77,7522049	-941,7141113	-0,013734
Cloud_Mesh_Distance_BA_R0_R3	22,20979691	-78,36850739	-941,9764404	0,032718
Cloud_Mesh_Distance_BA_R0_R3	22,28883171	-78,45865631	-941,8493042	0,035575
Cloud_Mesh_Distance_BA_R0_R3	22,3105011	-78,48360443	-941,9726563	0,038503

Cloud_Mesh_Distance_BA_R0_R3	22,38714981	-78,57650757	-941,8554077	0,041008
Cloud_Mesh_Distance_BA_R0_R3	16,69382858	-77,65855408	-941,5779419	-0,014712
Cloud_Mesh_Distance_BA_R0_R3	16,73640823	-77,47091675	-941,6879883	-0,016319
Cloud_Mesh_Distance_BA_R0_R3	16,51015091	-77,64306641	-941,7704468	-0,016623
Cloud_Mesh_Distance_BA_R0_R3	21,98196983	-78,13173676	-941,7837524	0,021745
Cloud_Mesh_Distance_BA_R0_R3	22,10040665	-78,26203156	-941,7003784	0,029143
Cloud_Mesh_Distance_BA_R0_R3	22,10015106	-78,24941254	-941,8851929	0,028541
Cloud_Mesh_Distance_BA_R0_R3	16,61100769	-77,51276398	-941,7735596	-0,017226
Cloud_Mesh_Distance_BA_R0_R3	16,75922585	-77,35674286	-941,7655029	-0,011323
Cloud_Mesh_Distance_BA_R0_R3	16,86350632	-77,38726044	-941,642395	-0,008322
Cloud_Mesh_Distance_BA_R0_R3	21,89742279	-78,03958893	-941,8948975	0,017009
Cloud_Mesh_Distance_BA_R0_R3	21,81209564	-77,95085907	-941,9668579	0,012416
Cloud_Mesh_Distance_BA_R0_R3	16,71112442	-77,29701996	-941,8636475	-0,011118
Cloud_Mesh_Distance_BA_R0_R3	16,82062912	-77,24723053	-941,8082886	-0,006327
Cloud_Mesh_Distance_BA_R0_R3	21,4991436	-77,64263153	-941,8773193	0,003376
Cloud_Mesh_Distance_BA_R0_R3	21,7305584	-77,871315	-941,8961792	0,008154
Cloud_Mesh_Distance_BA_R0_R3	21,6840477	-77,84001923	-941,737793	0,006556
Cloud_Mesh_Distance_BA_R0_R3	16,95845032	-77,20420074	-941,7268066	-0,002603
Cloud_Mesh_Distance_BA_R0_R3	21,33981323	-77,4838028	-941,8903809	0,005707
Cloud_Mesh_Distance_BA_R0_R3	17,08355331	-76,91384125	-941,9317017	0,014455
Cloud_Mesh_Distance_BA_R0_R3	17,14422226	-76,98963165	-941,7949219	0,015825
Cloud_Mesh_Distance_BA_R0_R3	21,24733734	-77,39788818	-941,855896	0,0107
Cloud_Mesh_Distance_BA_R0_R3	17,36219215	-76,9285202	-941,7003784	0,024935
Cloud_Mesh_Distance_BA_R0_R3	17,57124519	-76,71163177	-941,8450317	0,029368
Cloud_Mesh_Distance_BA_R0_R3	17,37850571	-76,76529694	-941,8991699	0,026415
Cloud_Mesh_Distance_BA_R0_R3	17,39430046	-76,85900116	-941,7640991	0,025448
Cloud_Mesh_Distance_BA_R0_R3	17,88333511	-76,60884857	-941,8117676	0,036365
Cloud_Mesh_Distance_BA_R0_R3	17,95419121	-76,69153595	-941,6507568	0,04475
Cloud_Mesh_Distance_BA_R0_R3	20,97623444	-77,13780975	-941,9820557	0,022601
Cloud_Mesh_Distance_BA_R0_R3	21,00143814	-77,18908691	-941,7508545	0,024879
Cloud_Mesh_Distance_BA_R0_R3	21,141819	-77,29853058	-941,8549194	0,015745
Cloud_Mesh_Distance_BA_R0_R3	17,7081604	-76,65198517	-941,8465576	0,033361
Cloud_Mesh_Distance_BA_R0_R3	18,08060455	-76,60700989	-941,7163086	0,044486
Cloud_Mesh_Distance_BA_R0_R3	18,46980286	-76,49945831	-941,7622681	0,041204
Cloud_Mesh_Distance_BA_R0_R3	18,5574913	-76,5467453	-941,6552124	0,041535
Cloud_Mesh_Distance_BA_R0_R3	20,56041908	-76,85167694	-941,8911743	0,035912
Cloud_Mesh_Distance_BA_R0_R3	20,51832581	-76,86232758	-941,7351074	0,040827
Cloud_Mesh_Distance_BA_R0_R3	20,63945007	-76,92115784	-941,7770386	0,039031
Cloud_Mesh_Distance_BA_R0_R3	20,712677	-76,92834473	-942,0500488	0,027429
Cloud_Mesh_Distance_BA_R0_R3	20,84649658	-77,03469086	-941,9620361	0,028349
Cloud_Mesh_Distance_BA_R0_R3	20,72465324	-76,95238495	-941,9160156	0,031735
Cloud_Mesh_Distance_BA_R0_R3	17,90736389	-76,50371552	-941,9649048	0,03894
Cloud_Mesh_Distance_BA_R0_R3	18,06230354	-76,51152802	-941,8775024	0,043823
Cloud_Mesh_Distance_BA_R0_R3	18,2617836	-76,52558899	-941,7753906	0,04587
Cloud_Mesh_Distance_BA_R0_R3	18,6469593	-76,49643707	-941,7366943	0,037527
Cloud_Mesh_Distance_BA_R0_R3	18,79831505	-76,44328308	-941,8370972	0,027123
Cloud_Mesh_Distance_BA_R0_R3	19,02580261	-76,43708038	-941,8624878	0,022978
Cloud_Mesh_Distance_BA_R0_R3	19,16550064	-76,49973297	-941,7423096	0,020738
Cloud_Mesh_Distance_BA_R0_R3	19,22470856	-76,44226837	-941,8809204	0,017303
Cloud_Mesh_Distance_BA_R0_R3	19,52023697	-76,54576874	-941,7279663	0,018052

Cloud_Mesh_Distance_BA_R0_R3	19,78527832	-76,58359528	-941,7568359	0,025488
Cloud_Mesh_Distance_BA_R0_R3	19,61880493	-76,49001312	-941,8939819	0,030834
Cloud_Mesh_Distance_BA_R0_R3	20,12754822	-76,62535095	-941,9466553	0,037233
Cloud_Mesh_Distance_BA_R0_R3	20,40948105	-76,7796402	-941,8444214	0,037376
Cloud_Mesh_Distance_BA_R0_R3	20,26708984	-76,68622589	-941,9487915	0,038676
Cloud_Mesh_Distance_BA_R0_R3	18,09453583	-76,44606781	-941,973877	0,042359
Cloud_Mesh_Distance_BA_R0_R3	18,14323044	-76,39636993	-942,0458374	0,039834
Cloud_Mesh_Distance_BA_R0_R3	18,291008	-76,37934113	-942,0335693	0,040663
Cloud_Mesh_Distance_BA_R0_R3	18,20486832	-76,44672394	-941,932312	0,042425
Cloud_Mesh_Distance_BA_R0_R3	18,47240067	-76,36995697	-942,0174561	0,032058
Cloud_Mesh_Distance_BA_R0_R3	18,37813568	-76,43849945	-941,8979492	0,042099
Cloud_Mesh_Distance_BA_R0_R3	18,67427254	-76,36958313	-942,0020752	0,027873
Cloud_Mesh_Distance_BA_R0_R3	18,57301521	-76,43256378	-941,8737793	0,035209
Cloud_Mesh_Distance_BA_R0_R3	19,41802597	-76,45988464	-941,8876343	0,023537
Cloud_Mesh_Distance_BA_R0_R3	19,49422073	-76,40703583	-942,0482178	0,02611
Cloud_Mesh_Distance_BA_R0_R3	19,83091736	-76,4895401	-942,0389404	0,036405
Cloud_Mesh_Distance_BA_R0_R3	19,78165627	-76,5138855	-941,9281006	0,032811
Cloud_Mesh_Distance_BA_R0_R3	19,37096024	-84,32091522	-941,2366943	-0,105448
Cloud_Mesh_Distance_BA_R0_R3	19,52453232	-84,33483124	-941,1981201	-0,106266
Cloud_Mesh_Distance_BA_R0_R3	19,61443329	-84,39945221	-941,3134766	-0,093609
Cloud_Mesh_Distance_BA_R0_R3	19,99791336	-84,45771027	-941,3639526	-0,061048
Cloud_Mesh_Distance_BA_R0_R3	20,16586304	-84,41051483	-941,2682495	-0,056435
Cloud_Mesh_Distance_BA_R0_R3	20,14406586	-84,47414398	-941,4012451	-0,047091
Cloud_Mesh_Distance_BA_R0_R3	20,31387329	-84,38704681	-941,2442017	-0,041272
Cloud_Mesh_Distance_BA_R0_R3	18,86902237	-84,21193695	-941,3139648	-0,066301
Cloud_Mesh_Distance_BA_R0_R3	18,64401245	-84,11560822	-941,2782593	-0,043518
Cloud_Mesh_Distance_BA_R0_R3	18,85215378	-84,13785553	-941,098877	-0,069228
Cloud_Mesh_Distance_BA_R0_R3	20,25818062	-84,36142731	-941,1845093	-0,051336
Cloud_Mesh_Distance_BA_R0_R3	20,5831871	-84,30469513	-941,1663208	-0,010792
Cloud_Mesh_Distance_BA_R0_R3	20,73019028	-84,28992462	-941,190979	0,020955
Cloud_Mesh_Distance_BA_R0_R3	18,08482361	-83,69884491	-941,1164551	-0,051239
Cloud_Mesh_Distance_BA_R0_R3	18,02098083	-83,71527863	-941,3190918	-0,047388
Cloud_Mesh_Distance_BA_R0_R3	18,16124535	-83,79819489	-941,2091065	-0,039352
Cloud_Mesh_Distance_BA_R0_R3	18,27888107	-83,90117645	-941,2501221	-0,029638
Cloud_Mesh_Distance_BA_R0_R3	18,42245865	-83,94530487	-941,1048584	-0,038882
Cloud_Mesh_Distance_BA_R0_R3	21,17295074	-84,20153046	-941,211731	0,105781
Cloud_Mesh_Distance_BA_R0_R3	21,39691162	-84,18027496	-941,2387085	0,167327
Cloud_Mesh_Distance_BA_R0_R3	21,81916809	-84,19484711	-941,2645874	0,310043
Cloud_Mesh_Distance_BA_R0_R3	21,59807205	-84,25688934	-941,3339844	0,241905
Cloud_Mesh_Distance_BA_R0_R3	17,90100098	-83,57141876	-941,2227173	-0,063627
Cloud_Mesh_Distance_BA_R0_R3	17,91950607	-83,61756134	-941,3030396	-0,059644
Cloud_Mesh_Distance_BA_R0_R3	17,97498703	-83,62030792	-941,1675415	-0,060087
Cloud_Mesh_Distance_BA_R0_R3	21,5748291	-84,14464569	-941,225647	0,217059
Cloud_Mesh_Distance_BA_R0_R3	17,72743988	-83,42528534	-941,2910767	-0,072824
Cloud_Mesh_Distance_BA_R0_R3	17,70602417	-83,37703705	-941,2228394	-0,079161
Cloud_Mesh_Distance_BA_R0_R3	17,77568817	-83,42832184	-941,1724243	-0,075742
Cloud_Mesh_Distance_BA_R0_R3	17,82193756	-83,52681732	-941,3109131	-0,064414
Cloud_Mesh_Distance_BA_R0_R3	17,89193344	-83,51636505	-941,105957	-0,068619
Cloud_Mesh_Distance_BA_R0_R3	17,4753952	-83,14388275	-941,2553101	-0,08154
Cloud_Mesh_Distance_BA_R0_R3	17,69487762	-83,31674957	-941,1042481	-0,083559

Cloud_Mesh_Distance_BA_R0_R3	17,56983376	-83,2165451	-941,1812134	-0,081554
Cloud_Mesh_Distance_BA_R0_R3	17,31327438	-82,90630341	-941,147583	-0,075375
Cloud_Mesh_Distance_BA_R0_R3	17,4936142	-83,0971756	-941,102417	-0,086711
Cloud_Mesh_Distance_BA_R0_R3	17,26570892	-82,91648102	-941,2937622	-0,070513
Cloud_Mesh_Distance_BA_R0_R3	17,21121597	-82,81757355	-941,2282715	-0,067182
Cloud_Mesh_Distance_BA_R0_R3	17,06830597	-82,55991364	-941,1057129	-0,048357
Cloud_Mesh_Distance_BA_R0_R3	17,02666473	-82,58621979	-941,2662354	-0,045142
Cloud_Mesh_Distance_BA_R0_R3	16,8671875	-82,35651398	-941,2943726	-0,019714
Cloud_Mesh_Distance_BA_R0_R3	16,88949966	-82,30951691	-941,1485596	-0,019404
Cloud_Mesh_Distance_BA_R0_R3	16,77055359	-82,11264801	-941,1729126	0,004797
Cloud_Mesh_Distance_BA_R0_R3	22,95016479	-82,43241119	-941,3222046	0,197505
Cloud_Mesh_Distance_BA_R0_R3	16,53818893	-81,7165451	-941,336731	0,038164
Cloud_Mesh_Distance_BA_R0_R3	16,59224701	-81,85688019	-941,3432007	0,030684
Cloud_Mesh_Distance_BA_R0_R3	16,44789886	-81,44400787	-941,3345947	0,053237
Cloud_Mesh_Distance_BA_R0_R3	16,57344055	-81,54445648	-941,0874634	0,041536
Cloud_Mesh_Distance_BA_R0_R3	16,52288437	-81,59470367	-941,2617798	0,047244
Cloud_Mesh_Distance_BA_R0_R3	16,60292435	-81,73015594	-941,1706543	0,036798
Cloud_Mesh_Distance_BA_R0_R3	16,51338577	-81,49651337	-941,2059326	0,050565
Cloud_Mesh_Distance_BA_R0_R3	22,99699783	-82,22248077	-941,4393311	0,131939
Cloud_Mesh_Distance_BA_R0_R3	23,02995682	-82,38582611	-941,4423828	0,222508
Cloud_Mesh_Distance_BA_R0_R3	23,07756805	-82,26677704	-941,5775147	0,186108
Cloud_Mesh_Distance_BA_R0_R3	16,39068604	-81,28205109	-941,3651123	0,057378
Cloud_Mesh_Distance_BA_R0_R3	16,37930489	-81,14963531	-941,3157959	0,057736
Cloud_Mesh_Distance_BA_R0_R3	16,44822311	-81,24637604	-941,2062988	0,051448
Cloud_Mesh_Distance_BA_R0_R3	16,5127964	-81,39574432	-941,1393433	0,048191
Cloud_Mesh_Distance_BA_R0_R3	23,01741791	-81,85443878	-941,5010376	-0,005106
Cloud_Mesh_Distance_BA_R0_R3	23,02828407	-81,66692352	-941,4764404	-0,069487
Cloud_Mesh_Distance_BA_R0_R3	16,29471588	-80,88414764	-941,3806763	0,059506
Cloud_Mesh_Distance_BA_R0_R3	16,41904449	-81,09118652	-941,1948242	0,056039
Cloud_Mesh_Distance_BA_R0_R3	16,36689758	-81,00524139	-941,2730713	0,059825
Cloud_Mesh_Distance_BA_R0_R3	23,03332138	-81,54337311	-941,4250488	-0,107967
Cloud_Mesh_Distance_BA_R0_R3	23,08140182	-81,469841	-941,6061401	-0,112108
Cloud_Mesh_Distance_BA_R0_R3	16,366045	-80,87633514	-941,2197266	0,062993
Cloud_Mesh_Distance_BA_R0_R3	16,34211731	-80,72933197	-941,216919	0,061744
Cloud_Mesh_Distance_BA_R0_R3	16,40939331	-80,79891205	-941,0996094	0,058348
Cloud_Mesh_Distance_BA_R0_R3	23,05332565	-81,42881012	-941,4244385	-0,13594
Cloud_Mesh_Distance_BA_R0_R3	23,09731674	-81,25101471	-941,4909668	-0,170878
Cloud_Mesh_Distance_BA_R0_R3	16,25447845	-80,40516663	-941,300354	0,076352
Cloud_Mesh_Distance_BA_R0_R3	23,1450119	-81,09230804	-941,690979	-0,18334
Cloud_Mesh_Distance_BA_R0_R3	23,1372261	-81,04810333	-941,541626	-0,208124
Cloud_Mesh_Distance_BA_R0_R3	16,15435219	-80,24701691	-941,4712524	0,059842
Cloud_Mesh_Distance_BA_R0_R3	16,32224846	-80,50782013	-941,1885986	0,075994
Cloud_Mesh_Distance_BA_R0_R3	23,1681366	-80,90512085	-941,6845093	-0,207219
Cloud_Mesh_Distance_BA_R0_R3	23,15204811	-80,89701843	-941,5015259	-0,225201
Cloud_Mesh_Distance_BA_R0_R3	16,25008965	-80,04349518	-941,2503662	0,091653
Cloud_Mesh_Distance_BA_R0_R3	23,18237114	-80,69468689	-941,6511231	-0,213332
Cloud_Mesh_Distance_BA_R0_R3	16,14130402	-79,91853333	-941,4586792	0,068236
Cloud_Mesh_Distance_BA_R0_R3	16,09656715	-79,84828949	-941,5443115	0,053307
Cloud_Mesh_Distance_BA_R0_R3	16,11608696	-79,73605347	-941,5105591	0,053974
Cloud_Mesh_Distance_BA_R0_R3	16,14562607	-79,81467438	-941,4503784	0,067556

Cloud_Mesh_Distance_BA_R0_R3	16,20307922	-79,872612	-941,3395386	0,08148
Cloud_Mesh_Distance_BA_R0_R3	23,15375519	-80,46465302	-941,428833	-0,218869
Cloud_Mesh_Distance_BA_R0_R3	23,16881943	-80,60163116	-941,4927979	-0,224784
Cloud_Mesh_Distance_BA_R0_R3	23,18362808	-80,537117	-941,663208	-0,204378
Cloud_Mesh_Distance_BA_R0_R3	23,18546295	-80,52950287	-941,713562	-0,197294
Cloud_Mesh_Distance_BA_R0_R3	16,26478577	-79,67848969	-941,2468262	0,086804
Cloud_Mesh_Distance_BA_R0_R3	23,1520195	-80,31075287	-941,5516968	-0,189718
Cloud_Mesh_Distance_BA_R0_R3	23,17268753	-80,44395447	-941,5960083	-0,198501
Cloud_Mesh_Distance_BA_R0_R3	16,28343582	-79,45378113	-941,2632446	0,073443
Cloud_Mesh_Distance_BA_R0_R3	16,21502304	-79,33448029	-941,4037476	0,051925
Cloud_Mesh_Distance_BA_R0_R3	23,09245682	-79,97153473	-941,7512207	-0,131409
Cloud_Mesh_Distance_BA_R0_R3	23,11703491	-80,13579559	-941,5340576	-0,162735
Cloud_Mesh_Distance_BA_R0_R3	16,3103447	-79,25835419	-941,274231	0,058129
Cloud_Mesh_Distance_BA_R0_R3	23,06347656	-79,92662048	-941,5286255	-0,132837
Cloud_Mesh_Distance_BA_R0_R3	22,99653625	-79,67766571	-941,7060547	-0,082534
Cloud_Mesh_Distance_BA_R0_R3	23,01970482	-79,76422882	-941,6054688	-0,102646
Cloud_Mesh_Distance_BA_R0_R3	16,30448532	-78,67559052	-941,4594116	0,016896
Cloud_Mesh_Distance_BA_R0_R3	16,24614334	-78,81302643	-941,4945068	0,015593
Cloud_Mesh_Distance_BA_R0_R3	16,2368927	-78,93837738	-941,4718018	0,024453
Cloud_Mesh_Distance_BA_R0_R3	16,38657379	-78,61682129	-941,3779907	0,017644
Cloud_Mesh_Distance_BA_R0_R3	22,82323837	-79,31557465	-941,5107422	-0,032265
Cloud_Mesh_Distance_BA_R0_R3	22,87181473	-79,40879822	-941,5471802	-0,047575
Cloud_Mesh_Distance_BA_R0_R3	16,24251175	-78,34645081	-941,6524048	0,000628
Cloud_Mesh_Distance_BA_R0_R3	16,30691528	-78,42743683	-941,5425415	0,007395
Cloud_Mesh_Distance_BA_R0_R3	16,34279251	-78,31856537	-941,5448608	0,005678
Cloud_Mesh_Distance_BA_R0_R3	16,3996582	-78,44245148	-941,4279175	0,011601
Cloud_Mesh_Distance_BA_R0_R3	16,48838043	-78,54932404	-941,2832031	0,013928
Cloud_Mesh_Distance_BA_R0_R3	22,68083191	-79,01302338	-941,6925659	0,011201
Cloud_Mesh_Distance_BA_R0_R3	22,58072662	-78,86281586	-941,6248169	0,027471
Cloud_Mesh_Distance_BA_R0_R3	22,66915131	-79,03356171	-941,4863281	0,00988
Cloud_Mesh_Distance_BA_R0_R3	16,43132019	-78,28775787	-941,458374	0,005703
Cloud_Mesh_Distance_BA_R0_R3	16,49575996	-78,13877106	-941,4638672	0,00213
Cloud_Mesh_Distance_BA_R0_R3	22,52817535	-78,76542664	-941,8217163	0,034503
Cloud_Mesh_Distance_BA_R0_R3	16,61200333	-78,06084442	-941,3867798	-0,001655
Cloud_Mesh_Distance_BA_R0_R3	22,31770706	-78,50758362	-941,6662598	0,040833
Cloud_Mesh_Distance_BA_R0_R3	22,40280151	-78,63234711	-941,541687	0,039172
Cloud_Mesh_Distance_BA_R0_R3	22,45678329	-78,67870331	-941,6884766	0,034778
Cloud_Mesh_Distance_BA_R0_R3	16,538311	-77,94529724	-941,5321045	-0,0076
Cloud_Mesh_Distance_BA_R0_R3	16,64138603	-77,83249664	-941,5025024	-0,008637
Cloud_Mesh_Distance_BA_R0_R3	16,65556908	-77,94024658	-941,4167481	-0,007285
Cloud_Mesh_Distance_BA_R0_R3	16,7747097	-77,84729767	-941,3613892	-0,003813
Cloud_Mesh_Distance_BA_R0_R3	22,2501812	-78,45305634	-941,5247192	0,035537
Cloud_Mesh_Distance_BA_R0_R3	16,90805244	-77,66777039	-941,3672485	-0,007927
Cloud_Mesh_Distance_BA_R0_R3	22,00086594	-78,17871857	-941,567688	0,023199
Cloud_Mesh_Distance_BA_R0_R3	22,10678673	-78,28913879	-941,5631714	0,029671
Cloud_Mesh_Distance_BA_R0_R3	22,2047081	-78,36710358	-941,7821655	0,034702
Cloud_Mesh_Distance_BA_R0_R3	22,19278908	-78,37206268	-941,6196289	0,034441
Cloud_Mesh_Distance_BA_R0_R3	16,99287224	-77,34374237	-941,5639038	-0,000423
Cloud_Mesh_Distance_BA_R0_R3	16,83305359	-77,5315094	-941,5464478	-0,00874
Cloud_Mesh_Distance_BA_R0_R3	16,96875572	-77,47847748	-941,4665527	-0,005463

Cloud_Mesh_Distance_BA_R0_R3	17,07313538	-77,47867584	-941,3707886	0,00517
Cloud_Mesh_Distance_BA_R0_R3	17,08675766	-77,3826828	-941,4453125	0,003068
Cloud_Mesh_Distance_BA_R0_R3	21,76768875	-77,95826721	-941,5153809	0,006578
Cloud_Mesh_Distance_BA_R0_R3	21,76834869	-77,93302917	-941,659668	0,010505
Cloud_Mesh_Distance_BA_R0_R3	21,81080055	-77,95825958	-941,7992554	0,01123
Cloud_Mesh_Distance_BA_R0_R3	21,89263916	-78,04141998	-941,7825928	0,015754
Cloud_Mesh_Distance_BA_R0_R3	16,93177414	-77,28630066	-941,6717529	-0,004175
Cloud_Mesh_Distance_BA_R0_R3	21,41152191	-77,57306671	-941,7420654	0,003504
Cloud_Mesh_Distance_BA_R0_R3	21,54606438	-77,70644379	-941,7269287	0,002848
Cloud_Mesh_Distance_BA_R0_R3	21,69686127	-77,87551117	-941,5891724	0,005911
Cloud_Mesh_Distance_BA_R0_R3	17,01663589	-77,24983215	-941,6314697	0,002596
Cloud_Mesh_Distance_BA_R0_R3	17,10696411	-77,13622284	-941,6677856	0,00894
Cloud_Mesh_Distance_BA_R0_R3	17,32348251	-77,11269379	-941,5166016	0,021487
Cloud_Mesh_Distance_BA_R0_R3	17,26944733	-77,00634003	-941,6777954	0,020118
Cloud_Mesh_Distance_BA_R0_R3	17,08769226	-77,27852631	-941,5420532	0,008074
Cloud_Mesh_Distance_BA_R0_R3	21,26115799	-77,4331131	-941,7063599	0,007859
Cloud_Mesh_Distance_BA_R0_R3	21,42443085	-77,56859589	-941,8776856	0,004297
Cloud_Mesh_Distance_BA_R0_R3	17,46632195	-76,89821625	-941,6641846	0,028343
Cloud_Mesh_Distance_BA_R0_R3	17,58983994	-77,01239014	-941,4384155	0,037553
Cloud_Mesh_Distance_BA_R0_R3	17,4405117	-76,99011993	-941,5686646	0,030266
Cloud_Mesh_Distance_BA_R0_R3	17,74650764	-76,9489212	-941,4173584	0,041898
Cloud_Mesh_Distance_BA_R0_R3	21,11375427	-77,30643463	-941,6500244	0,019892
Cloud_Mesh_Distance_BA_R0_R3	17,74948692	-76,75266266	-941,6773071	0,037341
Cloud_Mesh_Distance_BA_R0_R3	17,52707672	-76,81400299	-941,7327881	0,027569
Cloud_Mesh_Distance_BA_R0_R3	17,61302567	-76,89510345	-941,5701294	0,0355
Cloud_Mesh_Distance_BA_R0_R3	17,78848457	-76,85823822	-941,5100098	0,040285
Cloud_Mesh_Distance_BA_R0_R3	17,97459221	-76,80444336	-941,4788208	0,04629
Cloud_Mesh_Distance_BA_R0_R3	20,86716461	-77,0692215	-941,8225708	0,029739
Cloud_Mesh_Distance_BA_R0_R3	20,75049973	-77,00487518	-941,71875	0,039644
Cloud_Mesh_Distance_BA_R0_R3	20,73949051	-77,03961945	-941,5559082	0,039535
Cloud_Mesh_Distance_BA_R0_R3	18,10037613	-76,69419098	-941,5736084	0,047621
Cloud_Mesh_Distance_BA_R0_R3	18,22818375	-76,69773102	-941,5099487	0,051663
Cloud_Mesh_Distance_BA_R0_R3	18,24134827	-76,62638855	-941,616394	0,046788
Cloud_Mesh_Distance_BA_R0_R3	18,41353607	-76,57794189	-941,6369019	0,047601
Cloud_Mesh_Distance_BA_R0_R3	19,65467644	-76,62831116	-941,5979614	0,017787
Cloud_Mesh_Distance_BA_R0_R3	19,55783463	-76,68432617	-941,4520874	0,015196
Cloud_Mesh_Distance_BA_R0_R3	19,75777817	-76,70165253	-941,4849854	0,015885
Cloud_Mesh_Distance_BA_R0_R3	19,80444336	-76,65636444	-941,600708	0,020711
Cloud_Mesh_Distance_BA_R0_R3	20,28222466	-76,73348236	-941,7937012	0,040495
Cloud_Mesh_Distance_BA_R0_R3	20,3716774	-76,80283356	-941,692688	0,040437
Cloud_Mesh_Distance_BA_R0_R3	20,30056763	-76,82769012	-941,5355835	0,035223
Cloud_Mesh_Distance_BA_R0_R3	20,48256302	-76,8897934	-941,5792236	0,041643
Cloud_Mesh_Distance_BA_R0_R3	18,68616104	-76,55605316	-941,616394	0,04093
Cloud_Mesh_Distance_BA_R0_R3	18,8014698	-76,5688858	-941,5803833	0,037617
Cloud_Mesh_Distance_BA_R0_R3	18,97200203	-76,50119781	-941,71875	0,023158
Cloud_Mesh_Distance_BA_R0_R3	18,92750931	-76,56174469	-941,5913086	0,033347
Cloud_Mesh_Distance_BA_R0_R3	18,8046608	-76,51895905	-941,6797485	0,032409
Cloud_Mesh_Distance_BA_R0_R3	19,33267975	-76,50144958	-941,7716065	0,019599
Cloud_Mesh_Distance_BA_R0_R3	19,31904221	-76,56780243	-941,6271973	0,016588
Cloud_Mesh_Distance_BA_R0_R3	19,48996735	-76,61186218	-941,5785523	0,016235

Cloud_Mesh_Distance_BA_R0_R3	19,92695427	-76,66790009	-941,6408081	0,027118
Cloud_Mesh_Distance_BA_R0_R3	19,97981644	-76,63445282	-941,7600708	0,033464
Cloud_Mesh_Distance_BA_R0_R3	20,13353539	-76,66718292	-941,8157959	0,034896
Cloud_Mesh_Distance_BA_R0_R3	19,80132675	-84,37162018	-941,2001343	-0,09408
Cloud_Mesh_Distance_BA_R0_R3	19,97805023	-84,39215851	-941,2245483	-0,078119
Cloud_Mesh_Distance_BA_R0_R3	19,93015671	-84,32711029	-941,0946655	-0,094709
Cloud_Mesh_Distance_BA_R0_R3	18,69705963	-84,06726837	-941,0579834	-0,053996
Cloud_Mesh_Distance_BA_R0_R3	18,5816803	-83,9897995	-940,9927979	-0,051599
Cloud_Mesh_Distance_BA_R0_R3	19,02495956	-84,12828827	-940,9393921	-0,101436
Cloud_Mesh_Distance_BA_R0_R3	19,03517914	-84,19037628	-941,0979614	-0,093866
Cloud_Mesh_Distance_BA_R0_R3	19,4296875	-84,26978302	-941,0845947	-0,119265
Cloud_Mesh_Distance_BA_R0_R3	19,22217178	-84,22626495	-941,0758057	-0,111132
Cloud_Mesh_Distance_BA_R0_R3	19,17772293	-84,16880035	-940,9536133	-0,113319
Cloud_Mesh_Distance_BA_R0_R3	19,78004837	-84,32007599	-941,0959473	-0,107669
Cloud_Mesh_Distance_BA_R0_R3	19,64626312	-84,29425812	-941,069397	-0,119464
Cloud_Mesh_Distance_BA_R0_R3	19,67546082	-84,34470367	-941,1705933	-0,104929
Cloud_Mesh_Distance_BA_R0_R3	20,03026581	-84,27368927	-940,9912109	-0,097145
Cloud_Mesh_Distance_BA_R0_R3	20,1419754	-84,33434296	-941,1151733	-0,070002
Cloud_Mesh_Distance_BA_R0_R3	20,40149117	-84,31090546	-941,1216431	-0,037865
Cloud_Mesh_Distance_BA_R0_R3	20,49211502	-84,22133636	-940,9935303	-0,038582
Cloud_Mesh_Distance_BA_R0_R3	20,60969543	-84,24520111	-941,0742188	-0,010854
Cloud_Mesh_Distance_BA_R0_R3	20,69220352	-84,19194794	-941,0206299	-0,000887
Cloud_Mesh_Distance_BA_R0_R3	20,90916824	-84,29210663	-941,2562866	0,061217
Cloud_Mesh_Distance_BA_R0_R3	21,01642418	-84,22611237	-941,1955566	0,072025
Cloud_Mesh_Distance_BA_R0_R3	18,18046379	-83,75893402	-941,0668945	-0,048694
Cloud_Mesh_Distance_BA_R0_R3	18,14536667	-83,6954422	-940,9891968	-0,060067
Cloud_Mesh_Distance_BA_R0_R3	18,29379272	-83,83965302	-941,0548096	-0,044467
Cloud_Mesh_Distance_BA_R0_R3	18,53277969	-84,03107452	-941,1714478	-0,038696
Cloud_Mesh_Distance_BA_R0_R3	20,45466995	-84,15103912	-940,8671875	-0,054603
Cloud_Mesh_Distance_BA_R0_R3	20,37540054	-84,22154999	-940,9570313	-0,055959
Cloud_Mesh_Distance_BA_R0_R3	20,74309921	-84,21628571	-941,0783081	0,014259
Cloud_Mesh_Distance_BA_R0_R3	20,87609863	-84,21590424	-941,1300659	0,041881
Cloud_Mesh_Distance_BA_R0_R3	21,41460037	-84,0769577	-941,1344605	0,151793
Cloud_Mesh_Distance_BA_R0_R3	21,69639587	-84,14453888	-941,2302246	0,259955
Cloud_Mesh_Distance_BA_R0_R3	18,23519135	-83,75275421	-940,9577026	-0,058638
Cloud_Mesh_Distance_BA_R0_R3	21,51532745	-84,0896225	-941,1671753	0,182661
Cloud_Mesh_Distance_BA_R0_R3	21,66140366	-84,0502243	-941,1509399	0,22913
Cloud_Mesh_Distance_BA_R0_R3	21,80683517	-83,96549225	-941,0945435	0,267426
Cloud_Mesh_Distance_BA_R0_R3	21,82094193	-84,07491302	-941,1728516	0,290541
Cloud_Mesh_Distance_BA_R0_R3	17,92128372	-83,46651459	-940,9323731	-0,079442
Cloud_Mesh_Distance_BA_R0_R3	18,05081177	-83,58666229	-940,9347534	-0,072425
Cloud_Mesh_Distance_BA_R0_R3	17,76310921	-83,2728653	-940,8557739	-0,090388
Cloud_Mesh_Distance_BA_R0_R3	17,52151489	-83,02417755	-940,8847656	-0,09129
Cloud_Mesh_Distance_BA_R0_R3	17,41241455	-82,98006439	-941,0535278	-0,082793
Cloud_Mesh_Distance_BA_R0_R3	17,20999146	-82,68264008	-940,9730225	-0,067361
Cloud_Mesh_Distance_BA_R0_R3	17,1432457	-82,68125153	-941,1364136	-0,059258
Cloud_Mesh_Distance_BA_R0_R3	17,26805496	-82,79251862	-941,0349121	-0,076883
Cloud_Mesh_Distance_BA_R0_R3	17,35560226	-82,88803864	-941,0068359	-0,080246
Cloud_Mesh_Distance_BA_R0_R3	16,97913933	-82,4429245	-941,1328125	-0,034822
Cloud_Mesh_Distance_BA_R0_R3	17,06243515	-82,50359344	-941,0231323	-0,047962

Cloud_Mesh_Distance_BA_R0_R3	16,85312462	-82,20702362	-941,0846558	-0,008474
Cloud_Mesh_Distance_BA_R0_R3	16,81129456	-82,23308563	-941,2440186	-0,007914
Cloud_Mesh_Distance_BA_R0_R3	16,94437981	-82,30127716	-940,9905396	-0,024404
Cloud_Mesh_Distance_BA_R0_R3	17,13365936	-82,57619476	-940,9708252	-0,057304
Cloud_Mesh_Distance_BA_R0_R3	16,7476387	-81,98653412	-941,0640869	0,01414
Cloud_Mesh_Distance_BA_R0_R3	16,78356171	-81,91210175	-940,8855591	0,00966
Cloud_Mesh_Distance_BA_R0_R3	16,69595718	-81,8282547	-941,0194702	0,024521
Cloud_Mesh_Distance_BA_R0_R3	16,81894875	-82,10642242	-941,0305786	0,001968
Cloud_Mesh_Distance_BA_R0_R3	16,83718872	-82,04616547	-940,9063721	0,001792
Cloud_Mesh_Distance_BA_R0_R3	22,9343338	-82,54898834	-941,270752	0,245862
Cloud_Mesh_Distance_BA_R0_R3	16,66208267	-81,88187408	-941,1717529	0,027245
Cloud_Mesh_Distance_BA_R0_R3	22,82316208	-82,24115753	-941,1738281	0,065201
Cloud_Mesh_Distance_BA_R0_R3	16,63850403	-81,52994537	-940,9147949	0,03196
Cloud_Mesh_Distance_BA_R0_R3	22,94973755	-82,11811066	-941,3641968	0,067346
Cloud_Mesh_Distance_BA_R0_R3	22,9199295	-82,26944733	-941,3110352	0,115869
Cloud_Mesh_Distance_BA_R0_R3	16,51698112	-81,258461	-941,0505371	0,041606
Cloud_Mesh_Distance_BA_R0_R3	22,89006805	-81,83209991	-941,1790772	-0,054973
Cloud_Mesh_Distance_BA_R0_R3	22,95132065	-81,95407867	-941,3505859	0,009116
Cloud_Mesh_Distance_BA_R0_R3	23,01885223	-82,07035065	-941,5094605	0,085752
Cloud_Mesh_Distance_BA_R0_R3	16,56093216	-81,17863464	-940,9124756	0,034503
Cloud_Mesh_Distance_BA_R0_R3	22,97166634	-81,76720428	-941,3432007	-0,053065
Cloud_Mesh_Distance_BA_R0_R3	16,48389626	-81,08892059	-941,0476074	0,047655
Cloud_Mesh_Distance_BA_R0_R3	16,48291779	-80,87224579	-940,9701538	0,045016
Cloud_Mesh_Distance_BA_R0_R3	22,99651527	-81,62851715	-941,3477173	-0,094579
Cloud_Mesh_Distance_BA_R0_R3	22,94884109	-81,64707184	-941,2221069	-0,094155
Cloud_Mesh_Distance_BA_R0_R3	23,00466347	-81,49793243	-941,2925415	-0,128611
Cloud_Mesh_Distance_BA_R0_R3	16,42050171	-80,9569931	-941,1337891	0,056455
Cloud_Mesh_Distance_BA_R0_R3	23,04515457	-81,35620117	-941,3324585	-0,152121
Cloud_Mesh_Distance_BA_R0_R3	16,2276001	-80,24051666	-941,3183594	0,078493
Cloud_Mesh_Distance_BA_R0_R3	16,29680061	-80,32288361	-941,1964111	0,078728
Cloud_Mesh_Distance_BA_R0_R3	16,38197708	-80,29264069	-941,0287476	0,082261
Cloud_Mesh_Distance_BA_R0_R3	16,40485382	-80,4839325	-941,0204468	0,068474
Cloud_Mesh_Distance_BA_R0_R3	23,10464478	-81,07337189	-941,362915	-0,211739
Cloud_Mesh_Distance_BA_R0_R3	16,36657333	-80,08092499	-941,0379639	0,092077
Cloud_Mesh_Distance_BA_R0_R3	16,30452347	-80,16779327	-941,1574707	0,089055
Cloud_Mesh_Distance_BA_R0_R3	23,1590004	-80,76041412	-941,4586792	-0,233289
Cloud_Mesh_Distance_BA_R0_R3	23,13998795	-80,71775055	-941,3342896	-0,245724
Cloud_Mesh_Distance_BA_R0_R3	16,3135643	-80,02761841	-941,1318359	0,094969
Cloud_Mesh_Distance_BA_R0_R3	16,29901505	-79,88776398	-941,1619263	0,092191
Cloud_Mesh_Distance_BA_R0_R3	23,12123108	-80,33325958	-941,3270264	-0,203732
Cloud_Mesh_Distance_BA_R0_R3	16,35900497	-79,5563736	-941,1154175	0,083262
Cloud_Mesh_Distance_BA_R0_R3	16,37356949	-79,37560272	-941,1436768	0,071891
Cloud_Mesh_Distance_BA_R0_R3	16,45909119	-79,28227997	-941,0458374	0,065937
Cloud_Mesh_Distance_BA_R0_R3	23,08120918	-80,11951447	-941,3131714	-0,172556
Cloud_Mesh_Distance_BA_R0_R3	16,3874321	-79,2480011	-941,1625366	0,060535
Cloud_Mesh_Distance_BA_R0_R3	16,42369461	-79,19612122	-941,1273193	0,05806
Cloud_Mesh_Distance_BA_R0_R3	22,9811821	-79,66254425	-941,5813599	-0,083189
Cloud_Mesh_Distance_BA_R0_R3	23,01210785	-79,79470825	-941,4428711	-0,115405
Cloud_Mesh_Distance_BA_R0_R3	16,42384338	-79,01865387	-941,1893311	0,043966
Cloud_Mesh_Distance_BA_R0_R3	16,37670898	-79,13523865	-941,2155151	0,052629

Cloud_Mesh_Distance_BA_R0_R3	16,31672668	-78,98210144	-941,3487549	0,035836
Cloud_Mesh_Distance_BA_R0_R3	16,3094635	-79,12850189	-941,3140259	0,046904
Cloud_Mesh_Distance_BA_R0_R3	16,44974518	-78,87619781	-941,2061157	0,03277
Cloud_Mesh_Distance_BA_R0_R3	16,50591087	-78,97888184	-941,0939941	0,040246
Cloud_Mesh_Distance_BA_R0_R3	16,52757645	-78,88794708	-941,1014404	0,035191
Cloud_Mesh_Distance_BA_R0_R3	22,92911148	-79,53510284	-941,5664063	-0,063962
Cloud_Mesh_Distance_BA_R0_R3	22,89751816	-79,52306366	-941,3773804	-0,068983
Cloud_Mesh_Distance_BA_R0_R3	16,46434021	-78,70802307	-941,2497559	0,024605
Cloud_Mesh_Distance_BA_R0_R3	16,3597374	-78,77825928	-941,3554077	0,025228
Cloud_Mesh_Distance_BA_R0_R3	16,55751801	-78,77902985	-941,1066895	0,029399
Cloud_Mesh_Distance_BA_R0_R3	22,85042953	-79,40083313	-941,4212036	-0,046032
Cloud_Mesh_Distance_BA_R0_R3	16,5473938	-78,64263916	-941,1757202	0,019075
Cloud_Mesh_Distance_BA_R0_R3	22,75873375	-79,17742157	-941,5530396	-0,010426
Cloud_Mesh_Distance_BA_R0_R3	22,80016327	-79,31905365	-941,3570557	-0,035155
Cloud_Mesh_Distance_BA_R0_R3	16,66933632	-78,25624847	-941,2185669	0,003642
Cloud_Mesh_Distance_BA_R0_R3	16,54338455	-78,35198975	-941,3067627	0,008857
Cloud_Mesh_Distance_BA_R0_R3	22,66050148	-79,06912231	-941,3273315	0,002008
Cloud_Mesh_Distance_BA_R0_R3	16,76890182	-78,14447784	-941,1768189	0,003134
Cloud_Mesh_Distance_BA_R0_R3	22,49067116	-78,76061249	-941,4880981	0,035864
Cloud_Mesh_Distance_BA_R0_R3	16,59809113	-78,18897247	-941,329773	0,002206
Cloud_Mesh_Distance_BA_R0_R3	16,67288399	-78,13433075	-941,2821045	0,000867
Cloud_Mesh_Distance_BA_R0_R3	16,71754456	-78,01869202	-941,3049316	-0,004821
Cloud_Mesh_Distance_BA_R0_R3	22,32637405	-78,55395508	-941,472229	0,035849
Cloud_Mesh_Distance_BA_R0_R3	22,24059677	-78,47646332	-941,3863525	0,03251
Cloud_Mesh_Distance_BA_R0_R3	16,88826561	-77,80318451	-941,2827759	-0,003051
Cloud_Mesh_Distance_BA_R0_R3	16,82597733	-77,98668671	-941,2167358	-0,003466
Cloud_Mesh_Distance_BA_R0_R3	22,16576767	-78,37017059	-941,4762573	0,031919
Cloud_Mesh_Distance_BA_R0_R3	17,09587288	-77,65077972	-941,2032471	0,006108
Cloud_Mesh_Distance_BA_R0_R3	22,0693779	-78,27552032	-941,4441528	0,025533
Cloud_Mesh_Distance_BA_R0_R3	17,03106689	-77,56157684	-941,3375244	0,003706
Cloud_Mesh_Distance_BA_R0_R3	17,11564636	-77,53253174	-941,2848511	0,008945
Cloud_Mesh_Distance_BA_R0_R3	21,87356186	-78,03835297	-941,6483154	0,014751
Cloud_Mesh_Distance_BA_R0_R3	17,42937279	-77,18930817	-941,3522949	0,031145
Cloud_Mesh_Distance_BA_R0_R3	17,23447037	-77,28044128	-941,414856	0,014797
Cloud_Mesh_Distance_BA_R0_R3	17,29366302	-77,40979767	-941,239563	0,018311
Cloud_Mesh_Distance_BA_R0_R3	17,16847992	-77,43656158	-941,3239136	0,00996
Cloud_Mesh_Distance_BA_R0_R3	17,39308167	-77,30770111	-941,2574463	0,028095
Cloud_Mesh_Distance_BA_R0_R3	21,44751358	-77,64849091	-941,5249634	0,001878
Cloud_Mesh_Distance_BA_R0_R3	21,34765434	-77,53636932	-941,6008301	0,004078
Cloud_Mesh_Distance_BA_R0_R3	21,33766174	-77,58634186	-941,3591919	0,005959
Cloud_Mesh_Distance_BA_R0_R3	21,54798889	-77,77360535	-941,4025879	0,003435
Cloud_Mesh_Distance_BA_R0_R3	21,59871292	-77,78382874	-941,5729981	0,001243
Cloud_Mesh_Distance_BA_R0_R3	17,4685936	-77,08268738	-941,4407349	0,029666
Cloud_Mesh_Distance_BA_R0_R3	17,54249763	-77,18930054	-941,2677002	0,038429
Cloud_Mesh_Distance_BA_R0_R3	21,25032997	-77,48716736	-941,423523	0,008662
Cloud_Mesh_Distance_BA_R0_R3	21,33333969	-77,54766846	-941,4899292	0,005594
Cloud_Mesh_Distance_BA_R0_R3	21,27755356	-77,48093414	-941,5482788	0,008526
Cloud_Mesh_Distance_BA_R0_R3	21,1870079	-77,40111542	-941,5276489	0,015765
Cloud_Mesh_Distance_BA_R0_R3	17,56640434	-77,11328888	-941,3365479	0,036281
Cloud_Mesh_Distance_BA_R0_R3	17,87273216	-76,89733124	-941,4082642	0,045934

Cloud_Mesh_Distance_BA_R0_R3	17,95280075	-76,89511871	-941,366394	0,049152
Cloud_Mesh_Distance_BA_R0_R3	21,06563759	-77,30458832	-941,4842529	0,023428
Cloud_Mesh_Distance_BA_R0_R3	18,14869499	-76,75222015	-941,4644775	0,05115
Cloud_Mesh_Distance_BA_R0_R3	18,05945587	-76,89785767	-941,3046265	0,055085
Cloud_Mesh_Distance_BA_R0_R3	20,6280899	-76,94898224	-941,6365356	0,040886
Cloud_Mesh_Distance_BA_R0_R3	20,8144989	-77,11940765	-941,4581299	0,036963
Cloud_Mesh_Distance_BA_R0_R3	20,7239151	-77,06465912	-941,4473877	0,038486
Cloud_Mesh_Distance_BA_R0_R3	21,00367165	-77,22396088	-941,5922241	0,027027
Cloud_Mesh_Distance_BA_R0_R3	20,94047165	-77,20745087	-941,4679565	0,030683
Cloud_Mesh_Distance_BA_R0_R3	20,87890816	-77,11870575	-941,6212158	0,036228
Cloud_Mesh_Distance_BA_R0_R3	18,38740158	-76,67333221	-941,4841919	0,052267
Cloud_Mesh_Distance_BA_R0_R3	18,28476334	-76,75324249	-941,4001465	0,057075
Cloud_Mesh_Distance_BA_R0_R3	18,42061806	-76,75136566	-941,3469238	0,05893
Cloud_Mesh_Distance_BA_R0_R3	18,5077858	-76,78993988	-941,2572022	0,063447
Cloud_Mesh_Distance_BA_R0_R3	18,56855774	-76,71668243	-941,3543701	0,059845
Cloud_Mesh_Distance_BA_R0_R3	18,58346367	-76,61653137	-941,5233154	0,047987
Cloud_Mesh_Distance_BA_R0_R3	18,73575211	-76,60269928	-941,5219727	0,04252
Cloud_Mesh_Distance_BA_R0_R3	18,86129761	-76,61252594	-941,4931641	0,039604
Cloud_Mesh_Distance_BA_R0_R3	19,10883713	-76,69985199	-941,3362427	0,032954
Cloud_Mesh_Distance_BA_R0_R3	19,21763992	-76,63069916	-941,4810181	0,023847
Cloud_Mesh_Distance_BA_R0_R3	19,02435875	-76,61969757	-941,480957	0,031931
Cloud_Mesh_Distance_BA_R0_R3	19,70595551	-76,76061249	-941,3494873	0,008752
Cloud_Mesh_Distance_BA_R0_R3	20,01898384	-76,7358551	-941,5389404	0,026041
Cloud_Mesh_Distance_BA_R0_R3	19,88840675	-76,7075119	-941,529419	0,018854
Cloud_Mesh_Distance_BA_R0_R3	20,13571167	-76,80921173	-941,446106	0,025183
Cloud_Mesh_Distance_BA_R0_R3	20,22080421	-76,74602509	-941,6771851	0,03588
Cloud_Mesh_Distance_BA_R0_R3	20,43837547	-76,92658234	-941,4238281	0,036254
Cloud_Mesh_Distance_BA_R0_R3	19,10495758	-76,5611496	-941,6046753	0,024565
Cloud_Mesh_Distance_BA_R0_R3	19,38944626	-76,63955688	-941,4968872	0,015217
Cloud_Mesh_Distance_BA_R0_R3	20,15177727	-76,76139069	-941,5715332	0,030219
Cloud_Mesh_Distance_BA_R0_R3	20,07378769	-76,69948578	-941,6653442	0,03324
Cloud_Mesh_Distance_BA_R0_R3	18,64565659	-83,95847321	-940,8518677	-0,070804
Cloud_Mesh_Distance_BA_R0_R3	18,75162506	-83,97431183	-940,7865601	-0,083923
Cloud_Mesh_Distance_BA_R0_R3	18,73152924	-84,01758575	-940,8973389	-0,070907
Cloud_Mesh_Distance_BA_R0_R3	18,8615818	-84,01624298	-940,7880249	-0,091211
Cloud_Mesh_Distance_BA_R0_R3	18,86216927	-84,0762558	-940,9245606	-0,080275
Cloud_Mesh_Distance_BA_R0_R3	19,21142387	-84,10752106	-940,7956543	-0,123362
Cloud_Mesh_Distance_BA_R0_R3	19,39445877	-84,17479706	-940,8791504	-0,129448
Cloud_Mesh_Distance_BA_R0_R3	19,8347435	-84,2609024	-940,9702759	-0,114108
Cloud_Mesh_Distance_BA_R0_R3	19,62096405	-84,22348785	-940,9250488	-0,130729
Cloud_Mesh_Distance_BA_R0_R3	19,79820633	-84,17470551	-940,8063355	-0,129705
Cloud_Mesh_Distance_BA_R0_R3	19,61037445	-84,14842987	-940,7739868	-0,140213
Cloud_Mesh_Distance_BA_R0_R3	20,21229172	-84,24207306	-940,9549561	-0,07815
Cloud_Mesh_Distance_BA_R0_R3	20,30938339	-84,17655182	-940,8640747	-0,072084
Cloud_Mesh_Distance_BA_R0_R3	20,19425583	-84,16724396	-940,8218994	-0,087891
Cloud_Mesh_Distance_BA_R0_R3	18,48228836	-83,88526154	-940,8829956	-0,059677
Cloud_Mesh_Distance_BA_R0_R3	19,47386932	-84,0568924	-940,6191406	-0,145358
Cloud_Mesh_Distance_BA_R0_R3	19,37989807	-84,10443878	-940,7328491	-0,138326
Cloud_Mesh_Distance_BA_R0_R3	20,46393204	-84,08615875	-940,7700195	-0,064468
Cloud_Mesh_Distance_BA_R0_R3	20,30628204	-84,09616852	-940,734436	-0,081811

Cloud_Mesh_Distance_BA_R0_R3	20,61215973	-84,17093658	-940,9564209	-0,022225
Cloud_Mesh_Distance_BA_R0_R3	20,61920929	-84,07419586	-940,8146362	-0,038348
Cloud_Mesh_Distance_BA_R0_R3	20,79740143	-84,03479767	-940,8407593	-0,013693
Cloud_Mesh_Distance_BA_R0_R3	20,72562218	-83,99567413	-940,75354	-0,030987
Cloud_Mesh_Distance_BA_R0_R3	20,79785919	-84,1319809	-940,975708	0,008859
Cloud_Mesh_Distance_BA_R0_R3	21,04754639	-84,12889862	-941,0772095	0,056956
Cloud_Mesh_Distance_BA_R0_R3	21,2065239	-84,00904083	-940,9955444	0,074383
Cloud_Mesh_Distance_BA_R0_R3	21,26813507	-84,10219574	-941,1223755	0,110915
Cloud_Mesh_Distance_BA_R0_R3	18,15134811	-83,60417938	-940,8031616	-0,078096
Cloud_Mesh_Distance_BA_R0_R3	18,19388199	-83,67234039	-940,8636475	-0,068416
Cloud_Mesh_Distance_BA_R0_R3	18,28691864	-83,7400589	-940,8538818	-0,066691
Cloud_Mesh_Distance_BA_R0_R3	18,33399582	-83,80094147	-940,9078369	-0,059968
Cloud_Mesh_Distance_BA_R0_R3	20,9843483	-84,00959015	-940,8974609	0,021458
Cloud_Mesh_Distance_BA_R0_R3	21,00455093	-83,90210724	-940,779419	0,005249
Cloud_Mesh_Distance_BA_R0_R3	21,3633194	-84,01631927	-941,0593262	0,1216
Cloud_Mesh_Distance_BA_R0_R3	21,3980217	-83,8996048	-940,9569092	0,104778
Cloud_Mesh_Distance_BA_R0_R3	21,52299881	-83,99160004	-941,0786133	0,164128
Cloud_Mesh_Distance_BA_R0_R3	21,61836624	-83,86579132	-940,9952393	0,167084
Cloud_Mesh_Distance_BA_R0_R3	21,68194389	-83,94565582	-941,0696411	0,21005
Cloud_Mesh_Distance_BA_R0_R3	18,15683365	-83,53174591	-940,6652832	-0,095641
Cloud_Mesh_Distance_BA_R0_R3	21,16949081	-83,89225006	-940,8511353	0,039647
Cloud_Mesh_Distance_BA_R0_R3	21,1587944	-83,78609467	-940,7344971	0,015997
Cloud_Mesh_Distance_BA_R0_R3	21,79230881	-83,85723114	-941,019104	0,236167
Cloud_Mesh_Distance_BA_R0_R3	21,74540901	-83,76313019	-940,9475098	0,190025
Cloud_Mesh_Distance_BA_R0_R3	21,92012596	-83,88254547	-941,0426025	0,291492
Cloud_Mesh_Distance_BA_R0_R3	17,61639023	-83,17621613	-940,9782715	-0,08968
Cloud_Mesh_Distance_BA_R0_R3	17,66449356	-83,11692047	-940,7523193	-0,098604
Cloud_Mesh_Distance_BA_R0_R3	17,81819534	-83,39824677	-941,0021362	-0,082844
Cloud_Mesh_Distance_BA_R0_R3	17,8741951	-83,38083649	-940,8504639	-0,091448
Cloud_Mesh_Distance_BA_R0_R3	17,89528275	-83,31827545	-940,6913452	-0,098319
Cloud_Mesh_Distance_BA_R0_R3	17,79804993	-83,18804169	-940,6257935	-0,103373
Cloud_Mesh_Distance_BA_R0_R3	21,33445358	-83,78119659	-940,8192139	0,061177
Cloud_Mesh_Distance_BA_R0_R3	21,62093925	-83,60440826	-940,8008423	0,10408
Cloud_Mesh_Distance_BA_R0_R3	21,77747154	-83,63352203	-940,871582	0,166487
Cloud_Mesh_Distance_BA_R0_R3	21,56217575	-83,74329376	-940,881958	0,11927
Cloud_Mesh_Distance_BA_R0_R3	21,97120285	-83,72597504	-940,9561768	0,280519
Cloud_Mesh_Distance_BA_R0_R3	17,58794785	-82,97995758	-940,6574097	-0,096269
Cloud_Mesh_Distance_BA_R0_R3	17,29187012	-82,65294647	-940,7351074	-0,076092
Cloud_Mesh_Distance_BA_R0_R3	17,28603363	-82,72563934	-940,8699951	-0,075794
Cloud_Mesh_Distance_BA_R0_R3	17,42241669	-82,92220306	-940,9140625	-0,08526
Cloud_Mesh_Distance_BA_R0_R3	17,36680222	-82,75626373	-940,7404785	-0,085175
Cloud_Mesh_Distance_BA_R0_R3	17,36025238	-82,8294754	-940,8843994	-0,083805
Cloud_Mesh_Distance_BA_R0_R3	17,16635895	-82,53606415	-940,8287354	-0,062539
Cloud_Mesh_Distance_BA_R0_R3	17,21965027	-82,62602997	-940,852478	-0,067355
Cloud_Mesh_Distance_BA_R0_R3	17,22970009	-82,56946564	-940,7399902	-0,069783
Cloud_Mesh_Distance_BA_R0_R3	17,0693779	-82,42798615	-940,8834229	-0,04546
Cloud_Mesh_Distance_BA_R0_R3	22,596241	-82,78116608	-940,956543	0,194447
Cloud_Mesh_Distance_BA_R0_R3	22,5900383	-82,93718719	-940,9384766	0,2686
Cloud_Mesh_Distance_BA_R0_R3	16,89266968	-82,16915131	-940,9286499	-0,01202
Cloud_Mesh_Distance_BA_R0_R3	16,82204819	-81,84052277	-940,7281494	0,002108

Cloud_Mesh_Distance_BA_R0_R3	16,87409401	-81,97957611	-940,7476807	-0,007847
Cloud_Mesh_Distance_BA_R0_R3	22,61050797	-82,61859894	-940,9680176	0,12796
Cloud_Mesh_Distance_BA_R0_R3	22,52954102	-82,53699493	-940,8894653	0,065985
Cloud_Mesh_Distance_BA_R0_R3	22,73920822	-82,54354095	-941,0831909	0,152847
Cloud_Mesh_Distance_BA_R0_R3	22,85267448	-82,57619476	-941,1843872	0,21759
Cloud_Mesh_Distance_BA_R0_R3	22,70101166	-82,70494843	-941,036438	0,204713
Cloud_Mesh_Distance_BA_R0_R3	16,73927689	-81,76581573	-940,8515625	0,0187
Cloud_Mesh_Distance_BA_R0_R3	16,65683746	-81,67240143	-940,9747925	0,03032
Cloud_Mesh_Distance_BA_R0_R3	22,62020302	-82,31919098	-940,9375	0,016501
Cloud_Mesh_Distance_BA_R0_R3	22,62587738	-82,45424652	-940,9695435	0,067837
Cloud_Mesh_Distance_BA_R0_R3	22,72252083	-82,32318878	-941,0577393	0,055369
Cloud_Mesh_Distance_BA_R0_R3	22,84692001	-82,41817474	-941,2033081	0,144626
Cloud_Mesh_Distance_BA_R0_R3	16,68120766	-81,42967987	-940,7576904	0,019104
Cloud_Mesh_Distance_BA_R0_R3	22,76473236	-82,12821198	-941,0700073	0,004873
Cloud_Mesh_Distance_BA_R0_R3	16,58553123	-81,37129211	-940,9472656	0,034895
Cloud_Mesh_Distance_BA_R0_R3	22,86882019	-82,07086945	-941,2182007	0,018205
Cloud_Mesh_Distance_BA_R0_R3	22,80137062	-81,95200348	-941,0643311	-0,042673
Cloud_Mesh_Distance_BA_R0_R3	16,6039238	-81,09474945	-940,7865601	0,022985
Cloud_Mesh_Distance_BA_R0_R3	16,55000305	-81,00525665	-940,8720703	0,03624
Cloud_Mesh_Distance_BA_R0_R3	16,56149864	-80,83466339	-940,7987061	0,027228
Cloud_Mesh_Distance_BA_R0_R3	22,9529705	-81,51371002	-941,1500855	-0,132903
Cloud_Mesh_Distance_BA_R0_R3	16,46035194	-80,57689667	-940,9349365	0,057964
Cloud_Mesh_Distance_BA_R0_R3	16,45193481	-80,71663666	-940,9866943	0,052788
Cloud_Mesh_Distance_BA_R0_R3	16,51264	-80,69757843	-940,861145	0,041861
Cloud_Mesh_Distance_BA_R0_R3	22,93053436	-81,4462204	-941,048645	-0,157216
Cloud_Mesh_Distance_BA_R0_R3	16,39748383	-80,63597107	-941,0726929	0,065585
Cloud_Mesh_Distance_BA_R0_R3	23,00128174	-81,33856964	-941,1694946	-0,166077
Cloud_Mesh_Distance_BA_R0_R3	22,94529343	-81,31825256	-941,003479	-0,191418
Cloud_Mesh_Distance_BA_R0_R3	23,06207085	-81,24095917	-941,3088379	-0,182485
Cloud_Mesh_Distance_BA_R0_R3	16,46806717	-80,40647888	-940,8886108	0,05948
Cloud_Mesh_Distance_BA_R0_R3	16,53242493	-80,52217865	-940,7892456	0,042987
Cloud_Mesh_Distance_BA_R0_R3	16,47280502	-80,25582123	-940,8624878	0,069106
Cloud_Mesh_Distance_BA_R0_R3	23,05149078	-81,16342163	-941,210022	-0,206496
Cloud_Mesh_Distance_BA_R0_R3	23,0376358	-80,92243958	-941,0324097	-0,25108
Cloud_Mesh_Distance_BA_R0_R3	16,56297684	-80,31102753	-940,7110596	0,045863
Cloud_Mesh_Distance_BA_R0_R3	23,07476616	-81,00332642	-941,1894531	-0,238428
Cloud_Mesh_Distance_BA_R0_R3	23,12637901	-80,88101196	-941,3337402	-0,241197
Cloud_Mesh_Distance_BA_R0_R3	23,0983696	-80,81865692	-941,1880493	-0,258926
Cloud_Mesh_Distance_BA_R0_R3	16,35092545	-79,95963287	-941,0662842	0,09444
Cloud_Mesh_Distance_BA_R0_R3	16,35864449	-79,88085938	-941,0588379	0,094599
Cloud_Mesh_Distance_BA_R0_R3	16,44155121	-80,11768341	-940,9092407	0,078972
Cloud_Mesh_Distance_BA_R0_R3	23,06515312	-80,72927094	-941,0534668	-0,269102
Cloud_Mesh_Distance_BA_R0_R3	16,45988274	-79,73983002	-940,913269	0,08382
Cloud_Mesh_Distance_BA_R0_R3	16,36737061	-79,75440979	-941,060669	0,093496
Cloud_Mesh_Distance_BA_R0_R3	16,50112534	-79,58807373	-940,8856201	0,076342
Cloud_Mesh_Distance_BA_R0_R3	23,14472389	-80,58892059	-941,3460083	-0,240854
Cloud_Mesh_Distance_BA_R0_R3	23,12815666	-80,50996399	-941,2802734	-0,239194
Cloud_Mesh_Distance_BA_R0_R3	23,11506844	-80,64720154	-941,2128906	-0,256981
Cloud_Mesh_Distance_BA_R0_R3	16,42661858	-79,61954498	-940,9933472	0,0845
Cloud_Mesh_Distance_BA_R0_R3	16,45305443	-79,46369171	-940,9956665	0,076869

Cloud_Mesh_Distance_BA_R0_R3	23,06433487	-80,28695679	-941,1031494	-0,219652
Cloud_Mesh_Distance_BA_R0_R3	16,57971954	-79,4140625	-940,8233032	0,062198
Cloud_Mesh_Distance_BA_R0_R3	16,53100586	-79,36289978	-940,9130249	0,063643
Cloud_Mesh_Distance_BA_R0_R3	16,53090477	-79,47090149	-940,8761597	0,071151
Cloud_Mesh_Distance_BA_R0_R3	16,5904007	-79,5326004	-940,7670898	0,064159
Cloud_Mesh_Distance_BA_R0_R3	23,03198624	-80,09642029	-941,1102295	-0,174857
Cloud_Mesh_Distance_BA_R0_R3	16,57966232	-79,2156601	-940,8982544	0,053158
Cloud_Mesh_Distance_BA_R0_R3	16,66197586	-79,02718353	-940,8637085	0,040289
Cloud_Mesh_Distance_BA_R0_R3	16,56381035	-79,05171967	-940,9864502	0,04354
Cloud_Mesh_Distance_BA_R0_R3	22,98499298	-79,81356812	-941,2503662	-0,121467
Cloud_Mesh_Distance_BA_R0_R3	22,95933914	-79,66680145	-941,3987427	-0,097048
Cloud_Mesh_Distance_BA_R0_R3	22,99444199	-79,94750977	-941,1123657	-0,148122
Cloud_Mesh_Distance_BA_R0_R3	23,03493881	-79,94863129	-941,2920532	-0,145886
Cloud_Mesh_Distance_BA_R0_R3	16,47543335	-79,12459564	-941,0801392	0,052684
Cloud_Mesh_Distance_BA_R0_R3	22,82018089	-79,44001007	-941,1887817	-0,057289
Cloud_Mesh_Distance_BA_R0_R3	22,9188385	-79,68228912	-941,1713257	-0,097301
Cloud_Mesh_Distance_BA_R0_R3	16,59687042	-78,9149704	-941,000061	0,03489
Cloud_Mesh_Distance_BA_R0_R3	22,85031319	-79,44368744	-941,3131714	-0,056898
Cloud_Mesh_Distance_BA_R0_R3	22,87097549	-79,53409576	-941,2253418	-0,071298
Cloud_Mesh_Distance_BA_R0_R3	16,63269615	-78,64048004	-941,0748291	0,015972
Cloud_Mesh_Distance_BA_R0_R3	16,59332275	-78,52646637	-941,1715088	0,013823
Cloud_Mesh_Distance_BA_R0_R3	22,66770554	-79,19004822	-941,0958862	-0,02107
Cloud_Mesh_Distance_BA_R0_R3	22,68699265	-79,15726471	-941,2276611	-0,014454
Cloud_Mesh_Distance_BA_R0_R3	22,72397995	-79,17311859	-941,3516846	-0,011272
Cloud_Mesh_Distance_BA_R0_R3	16,75382042	-78,29177094	-941,1089478	0,00253
Cloud_Mesh_Distance_BA_R0_R3	16,68191147	-78,41996765	-941,1213379	0,010767
Cloud_Mesh_Distance_BA_R0_R3	16,66448975	-78,54097748	-941,0827026	0,016134
Cloud_Mesh_Distance_BA_R0_R3	22,62434387	-79,060112	-941,2042236	-0,002858
Cloud_Mesh_Distance_BA_R0_R3	22,58386803	-78,92870331	-941,3756714	0,017523
Cloud_Mesh_Distance_BA_R0_R3	16,89496994	-78,0812912	-941,0841675	0,001884
Cloud_Mesh_Distance_BA_R0_R3	16,8504715	-78,24026489	-941,0334473	0,004125
Cloud_Mesh_Distance_BA_R0_R3	22,40840149	-78,67708588	-941,3926392	0,036002
Cloud_Mesh_Distance_BA_R0_R3	22,48649216	-78,80797577	-941,3126831	0,026131
Cloud_Mesh_Distance_BA_R0_R3	22,32126236	-78,63547516	-941,2061157	0,025291
Cloud_Mesh_Distance_BA_R0_R3	22,32475281	-78,59752655	-941,31604	0,031288
Cloud_Mesh_Distance_BA_R0_R3	16,88040924	-77,88079834	-941,2340698	-0,001297
Cloud_Mesh_Distance_BA_R0_R3	16,92290688	-77,95037842	-941,1434937	0,001769
Cloud_Mesh_Distance_BA_R0_R3	16,99285698	-77,81983185	-941,1672974	0,00283
Cloud_Mesh_Distance_BA_R0_R3	22,02828979	-78,26480865	-941,3280029	0,021613
Cloud_Mesh_Distance_BA_R0_R3	22,12794113	-78,37462616	-941,3133545	0,025512
Cloud_Mesh_Distance_BA_R0_R3	22,23725891	-78,53253174	-941,2136841	0,027535
Cloud_Mesh_Distance_BA_R0_R3	17,1019516	-77,86421967	-941,0275879	0,009705
Cloud_Mesh_Distance_BA_R0_R3	17,21694946	-77,5349884	-941,190979	0,015125
Cloud_Mesh_Distance_BA_R0_R3	17,44503593	-77,56041718	-940,9705811	0,034831
Cloud_Mesh_Distance_BA_R0_R3	21,98285675	-78,19550323	-941,4074707	0,019831
Cloud_Mesh_Distance_BA_R0_R3	17,40692139	-77,39746094	-941,1565552	0,030468
Cloud_Mesh_Distance_BA_R0_R3	17,35383987	-77,50366974	-941,1000977	0,026524
Cloud_Mesh_Distance_BA_R0_R3	21,52552414	-77,79824066	-941,243103	0,003274
Cloud_Mesh_Distance_BA_R0_R3	21,61095238	-77,90128326	-941,1775513	0,006808
Cloud_Mesh_Distance_BA_R0_R3	21,61341667	-77,85912323	-941,3185425	0,003539

Cloud_Mesh_Distance_BA_R0_R3	21,87489319	-78,08452606	-941,4210815	0,012908
Cloud_Mesh_Distance_BA_R0_R3	21,7236042	-77,96511078	-941,3173218	0,008717
Cloud_Mesh_Distance_BA_R0_R3	21,82263184	-78,09516144	-941,2127075	0,0115
Cloud_Mesh_Distance_BA_R0_R3	17,51624489	-77,32405853	-941,1442871	0,036293
Cloud_Mesh_Distance_BA_R0_R3	17,48902321	-77,248909	-941,2433472	0,034531
Cloud_Mesh_Distance_BA_R0_R3	21,21908569	-77,50914764	-941,2589111	0,012591
Cloud_Mesh_Distance_BA_R0_R3	21,32601929	-77,63967133	-941,161377	0,007792
Cloud_Mesh_Distance_BA_R0_R3	21,4383316	-77,6958847	-941,3075562	0,00491
Cloud_Mesh_Distance_BA_R0_R3	21,65890503	-77,8674469	-941,4530029	0,003801
Cloud_Mesh_Distance_BA_R0_R3	17,65312767	-77,26506805	-941,1066284	0,04616
Cloud_Mesh_Distance_BA_R0_R3	17,6631813	-77,16291046	-941,2124634	0,046502
Cloud_Mesh_Distance_BA_R0_R3	17,57129288	-77,23695374	-941,1948853	0,039966
Cloud_Mesh_Distance_BA_R0_R3	17,92125511	-77,14083099	-941,074707	0,058609
Cloud_Mesh_Distance_BA_R0_R3	17,91891098	-77,07277679	-941,1577759	0,055229
Cloud_Mesh_Distance_BA_R0_R3	17,7869854	-77,19923401	-941,0892944	0,052523
Cloud_Mesh_Distance_BA_R0_R3	21,13637161	-77,4010849	-941,3657227	0,018186
Cloud_Mesh_Distance_BA_R0_R3	17,91286087	-76,98838043	-941,2676392	0,051433
Cloud_Mesh_Distance_BA_R0_R3	17,71449661	-77,05181885	-941,3092041	0,044403
Cloud_Mesh_Distance_BA_R0_R3	17,8186245	-77,09996796	-941,1862793	0,050282
Cloud_Mesh_Distance_BA_R0_R3	18,23069382	-76,90702057	-941,2053223	0,063385
Cloud_Mesh_Distance_BA_R0_R3	18,32105637	-76,99822235	-941,0422363	0,068374
Cloud_Mesh_Distance_BA_R0_R3	18,34211349	-76,91597748	-941,1413574	0,067006
Cloud_Mesh_Distance_BA_R0_R3	20,86445236	-77,19083405	-941,3514404	0,031624
Cloud_Mesh_Distance_BA_R0_R3	20,98965454	-77,29692078	-941,3143921	0,025081
Cloud_Mesh_Distance_BA_R0_R3	20,95803833	-77,34328461	-941,1397095	0,022849
Cloud_Mesh_Distance_BA_R0_R3	18,17534637	-76,82265472	-941,3494873	0,05435
Cloud_Mesh_Distance_BA_R0_R3	18,37100601	-76,83853912	-941,2373047	0,065922
Cloud_Mesh_Distance_BA_R0_R3	19,35663033	-76,86594391	-941,0758057	0,023182
Cloud_Mesh_Distance_BA_R0_R3	19,24688721	-76,78161621	-941,2043457	0,030012
Cloud_Mesh_Distance_BA_R0_R3	19,55718613	-76,87830353	-941,0939331	0,010047
Cloud_Mesh_Distance_BA_R0_R3	19,74391937	-76,89315033	-941,1168213	0,003834
Cloud_Mesh_Distance_BA_R0_R3	19,81111908	-76,83530426	-941,241394	0,008139
Cloud_Mesh_Distance_BA_R0_R3	19,88983154	-76,90234375	-941,1463623	0,006779
Cloud_Mesh_Distance_BA_R0_R3	20,45586014	-77,03542328	-941,1979981	0,027631
Cloud_Mesh_Distance_BA_R0_R3	20,53694916	-77,01622009	-941,3149414	0,033252
Cloud_Mesh_Distance_BA_R0_R3	20,58167839	-77,08644867	-941,208313	0,031399
Cloud_Mesh_Distance_BA_R0_R3	20,711483	-77,10683441	-941,3182373	0,034391
Cloud_Mesh_Distance_BA_R0_R3	18,70323372	-76,82154083	-941,1543579	0,066074
Cloud_Mesh_Distance_BA_R0_R3	18,61489868	-76,78819275	-941,2277222	0,065041
Cloud_Mesh_Distance_BA_R0_R3	18,73081207	-76,75559235	-941,2537231	0,05755
Cloud_Mesh_Distance_BA_R0_R3	18,75834274	-76,66901398	-941,3981323	0,049747
Cloud_Mesh_Distance_BA_R0_R3	18,91623306	-76,73913574	-941,2609253	0,050197
Cloud_Mesh_Distance_BA_R0_R3	18,93181419	-76,66551971	-941,3916016	0,044098
Cloud_Mesh_Distance_BA_R0_R3	19,35856438	-76,7098465	-941,354187	0,017706
Cloud_Mesh_Distance_BA_R0_R3	19,4487381	-76,79917145	-941,2091675	0,014552
Cloud_Mesh_Distance_BA_R0_R3	19,5414257	-76,7529068	-941,3156128	0,014253
Cloud_Mesh_Distance_BA_R0_R3	19,6350174	-76,81809235	-941,2197876	0,009929
Cloud_Mesh_Distance_BA_R0_R3	19,95862961	-76,8660202	-941,2379761	0,011976
Cloud_Mesh_Distance_BA_R0_R3	19,92665672	-76,78372955	-941,3873901	0,016388
Cloud_Mesh_Distance_BA_R0_R3	20,03626442	-76,93684387	-941,1397095	0,008262

Cloud_Mesh_Distance_BA_R0_R3	20,39067841	-76,96429443	-941,2922974	0,028847
Cloud_Mesh_Distance_BA_R0_R3	20,2796917	-76,88799286	-941,3713989	0,027056
Cloud_Mesh_Distance_BA_R0_R3	20,12004852	-76,88130188	-941,2835083	0,017981
Cloud_Mesh_Distance_BA_R0_R3	20,6138382	-76,99546051	-941,4650879	0,040757
Cloud_Mesh_Distance_BA_R0_R3	19,01706505	-84,06182098	-940,7870483	-0,107306
Cloud_Mesh_Distance_BA_R0_R3	18,98049927	-83,9872818	-940,6558228	-0,113252
Cloud_Mesh_Distance_BA_R0_R3	19,4934082	-84,11606598	-940,7298584	-0,141872
Cloud_Mesh_Distance_BA_R0_R3	20,02071571	-84,19496918	-940,8464966	-0,105452
Cloud_Mesh_Distance_BA_R0_R3	18,54249573	-83,81009674	-940,6769409	-0,085885
Cloud_Mesh_Distance_BA_R0_R3	18,58040237	-83,73807526	-940,5245972	-0,108561
Cloud_Mesh_Distance_BA_R0_R3	18,83313751	-83,95943451	-940,6951294	-0,096969
Cloud_Mesh_Distance_BA_R0_R3	18,68962097	-83,90630341	-940,7095947	-0,087018
Cloud_Mesh_Distance_BA_R0_R3	18,88562393	-83,89720917	-940,5515137	-0,120795
Cloud_Mesh_Distance_BA_R0_R3	19,34231567	-83,98801422	-940,5214844	-0,147074
Cloud_Mesh_Distance_BA_R0_R3	19,16568947	-84,02715302	-940,6464233	-0,130568
Cloud_Mesh_Distance_BA_R0_R3	19,31620216	-84,05893707	-940,6589966	-0,139581
Cloud_Mesh_Distance_BA_R0_R3	19,59653854	-84,09137726	-940,6662598	-0,141966
Cloud_Mesh_Distance_BA_R0_R3	19,74490356	-84,09708405	-940,6658936	-0,134495
Cloud_Mesh_Distance_BA_R0_R3	19,94435501	-84,10797882	-940,689209	-0,119496
Cloud_Mesh_Distance_BA_R0_R3	20,11647034	-84,03040314	-940,5877686	-0,11036
Cloud_Mesh_Distance_BA_R0_R3	19,85971069	-84,01352692	-940,5270996	-0,133328
Cloud_Mesh_Distance_BA_R0_R3	20,11728287	-84,12094879	-940,730835	-0,102378
Cloud_Mesh_Distance_BA_R0_R3	20,62446022	-83,99106598	-940,6993408	-0,045809
Cloud_Mesh_Distance_BA_R0_R3	18,30524826	-83,62471771	-940,6300659	-0,099492
Cloud_Mesh_Distance_BA_R0_R3	18,21733284	-83,62549591	-940,7439575	-0,082155
Cloud_Mesh_Distance_BA_R0_R3	18,39116096	-83,77593231	-940,7835693	-0,075015
Cloud_Mesh_Distance_BA_R0_R3	18,29931259	-83,69268036	-940,7509766	-0,07965
Cloud_Mesh_Distance_BA_R0_R3	18,4281311	-83,70912933	-940,6268921	-0,095612
Cloud_Mesh_Distance_BA_R0_R3	20,85716248	-83,95569611	-940,7670288	-0,012839
Cloud_Mesh_Distance_BA_R0_R3	21,02253342	-83,82819366	-940,7037964	-0,005418
Cloud_Mesh_Distance_BA_R0_R3	21,08994865	-83,85266876	-940,7677002	0,012914
Cloud_Mesh_Distance_BA_R0_R3	18,47949982	-83,6135025	-940,4297485	-0,128093
Cloud_Mesh_Distance_BA_R0_R3	18,40839195	-83,63401031	-940,5289917	-0,111423
Cloud_Mesh_Distance_BA_R0_R3	20,94663239	-83,81189728	-940,6435547	-0,021736
Cloud_Mesh_Distance_BA_R0_R3	21,14205742	-83,66883087	-940,6044922	-0,006286
Cloud_Mesh_Distance_BA_R0_R3	21,17904091	-83,69965363	-940,6575317	0,005892
Cloud_Mesh_Distance_BA_R0_R3	21,03213501	-83,74124908	-940,6148071	-0,016085
Cloud_Mesh_Distance_BA_R0_R3	21,27831268	-83,70470428	-940,718689	0,031413
Cloud_Mesh_Distance_BA_R0_R3	17,71668625	-83,04553986	-940,5241089	-0,105393
Cloud_Mesh_Distance_BA_R0_R3	18,04091072	-83,37429047	-940,5596924	-0,108481
Cloud_Mesh_Distance_BA_R0_R3	17,98552322	-83,4447403	-940,7719727	-0,091165
Cloud_Mesh_Distance_BA_R0_R3	17,93290901	-83,23983002	-940,4932251	-0,10947
Cloud_Mesh_Distance_BA_R0_R3	21,69005013	-83,50006866	-940,7583618	0,098541
Cloud_Mesh_Distance_BA_R0_R3	21,52307129	-83,53733063	-940,7074585	0,053321
Cloud_Mesh_Distance_BA_R0_R3	21,43367767	-83,66014862	-940,7597656	0,059023
Cloud_Mesh_Distance_BA_R0_R3	21,7358532	-83,55570221	-940,8105469	0,129054
Cloud_Mesh_Distance_BA_R0_R3	22,07631493	-83,57190704	-940,8933716	0,281538
Cloud_Mesh_Distance_BA_R0_R3	22,06653595	-83,44353485	-940,8428955	0,232826
Cloud_Mesh_Distance_BA_R0_R3	21,94960403	-83,57012177	-940,8746948	0,222993
Cloud_Mesh_Distance_BA_R0_R3	17,46225739	-82,87928009	-940,7443237	-0,092289

Cloud_Mesh_Distance_BA_R0_R3	21,847229	-83,2733078	-940,694458	0,087468
Cloud_Mesh_Distance_BA_R0_R3	21,77674866	-83,3857193	-940,7244263	0,095558
Cloud_Mesh_Distance_BA_R0_R3	21,85171127	-83,48589325	-940,8081665	0,152571
Cloud_Mesh_Distance_BA_R0_R3	21,96305847	-83,32236481	-940,7648315	0,144972
Cloud_Mesh_Distance_BA_R0_R3	22,14066315	-83,29781342	-940,8113403	0,216942
Cloud_Mesh_Distance_BA_R0_R3	17,44338989	-82,76267242	-940,5891113	-0,092532
Cloud_Mesh_Distance_BA_R0_R3	21,95627213	-83,20664215	-940,711853	0,104471
Cloud_Mesh_Distance_BA_R0_R3	22,09989929	-83,17450714	-940,7592163	0,150864
Cloud_Mesh_Distance_BA_R0_R3	17,35978889	-82,674263	-940,6239624	-0,085521
Cloud_Mesh_Distance_BA_R0_R3	17,29032707	-82,55197906	-940,5865479	-0,080691
Cloud_Mesh_Distance_BA_R0_R3	17,40991211	-82,63684845	-940,4702148	-0,091281
Cloud_Mesh_Distance_BA_R0_R3	22,10542297	-83,05628204	-940,7235107	0,108967
Cloud_Mesh_Distance_BA_R0_R3	22,28397942	-83,18904877	-940,828064	0,243277
Cloud_Mesh_Distance_BA_R0_R3	22,23606491	-83,04209137	-940,7810059	0,157175
Cloud_Mesh_Distance_BA_R0_R3	22,43129921	-83,02939606	-940,8641968	0,23673
Cloud_Mesh_Distance_BA_R0_R3	17,11043167	-82,33330536	-940,6599731	-0,054243
Cloud_Mesh_Distance_BA_R0_R3	17,1800499	-82,46889496	-940,6978149	-0,064587
Cloud_Mesh_Distance_BA_R0_R3	22,16420937	-82,94080353	-940,7165527	0,091301
Cloud_Mesh_Distance_BA_R0_R3	22,32581139	-82,87273407	-940,7927246	0,12353
Cloud_Mesh_Distance_BA_R0_R3	22,48197174	-82,86284637	-940,8843384	0,184352
Cloud_Mesh_Distance_BA_R0_R3	16,99586105	-82,23401642	-940,7759399	-0,033418
Cloud_Mesh_Distance_BA_R0_R3	16,99058723	-82,06247711	-940,5924072	-0,032077
Cloud_Mesh_Distance_BA_R0_R3	16,91629028	-82,10233307	-940,7897339	-0,016374
Cloud_Mesh_Distance_BA_R0_R3	17,06678391	-82,21112823	-940,600769	-0,04766
Cloud_Mesh_Distance_BA_R0_R3	22,4634285	-82,69971466	-940,859314	0,105689
Cloud_Mesh_Distance_BA_R0_R3	22,41087914	-82,52814484	-940,7791748	0,022915
Cloud_Mesh_Distance_BA_R0_R3	16,78536987	-81,7004776	-940,6959229	0,00679
Cloud_Mesh_Distance_BA_R0_R3	16,84392929	-81,63381195	-940,5335693	-0,012546
Cloud_Mesh_Distance_BA_R0_R3	22,56482315	-82,26415253	-940,8561401	-0,018926
Cloud_Mesh_Distance_BA_R0_R3	22,49181747	-82,29296112	-940,7827148	-0,028626
Cloud_Mesh_Distance_BA_R0_R3	22,43549347	-82,38248444	-940,7550049	-0,015389
Cloud_Mesh_Distance_BA_R0_R3	22,52824783	-82,39464569	-940,8557739	0,013734
Cloud_Mesh_Distance_BA_R0_R3	22,65032005	-82,16861725	-940,9275513	-0,026162
Cloud_Mesh_Distance_BA_R0_R3	16,74843979	-81,41355133	-940,6062012	0,001755
Cloud_Mesh_Distance_BA_R0_R3	16,70750999	-81,61261749	-940,8068237	0,019191
Cloud_Mesh_Distance_BA_R0_R3	16,80998611	-81,46598053	-940,5085449	-0,011606
Cloud_Mesh_Distance_BA_R0_R3	16,76012802	-81,54782867	-940,6503906	0,004594
Cloud_Mesh_Distance_BA_R0_R3	22,63409042	-81,98358917	-940,8204346	-0,077623
Cloud_Mesh_Distance_BA_R0_R3	22,71970367	-82,01830292	-940,9632568	-0,044948
Cloud_Mesh_Distance_BA_R0_R3	16,78155518	-81,3408432	-940,508728	-0,011665
Cloud_Mesh_Distance_BA_R0_R3	16,6140995	-80,95456696	-940,7230225	0,01609
Cloud_Mesh_Distance_BA_R0_R3	16,63360596	-81,2400589	-940,7764893	0,019288
Cloud_Mesh_Distance_BA_R0_R3	22,82112885	-81,79994965	-941,0273438	-0,085292
Cloud_Mesh_Distance_BA_R0_R3	22,74186516	-81,86353302	-940,9223023	-0,0895
Cloud_Mesh_Distance_BA_R0_R3	22,67657471	-81,82762909	-940,7979126	-0,104967
Cloud_Mesh_Distance_BA_R0_R3	16,68589783	-80,88768768	-940,5647583	-0,00236
Cloud_Mesh_Distance_BA_R0_R3	22,7923584	-81,72534943	-940,9321899	-0,112241
Cloud_Mesh_Distance_BA_R0_R3	22,86967468	-81,42508698	-940,9006348	-0,169077
Cloud_Mesh_Distance_BA_R0_R3	22,87479401	-81,62471771	-941,0358887	-0,118564
Cloud_Mesh_Distance_BA_R0_R3	16,65969276	-80,72310638	-940,5847778	0,005021

Cloud_Mesh_Distance_BA_R0_R3	16,62245178	-80,81267548	-940,6723633	0,012079
Cloud_Mesh_Distance_BA_R0_R3	22,9121418	-81,22727203	-940,8759155	-0,205358
Cloud_Mesh_Distance_BA_R0_R3	16,59243393	-80,66336823	-940,7007446	0,025314
Cloud_Mesh_Distance_BA_R0_R3	23,00073624	-81,14309692	-941,0421143	-0,219942
Cloud_Mesh_Distance_BA_R0_R3	16,60423279	-80,48163605	-940,6539307	0,024302
Cloud_Mesh_Distance_BA_R0_R3	16,62803459	-80,30900574	-940,598938	0,02949
Cloud_Mesh_Distance_BA_R0_R3	16,51292038	-80,15535736	-940,7892456	0,064077
Cloud_Mesh_Distance_BA_R0_R3	16,5811882	-80,09397125	-940,6768799	0,050966
Cloud_Mesh_Distance_BA_R0_R3	16,6687603	-80,3456192	-940,5296631	0,015973
Cloud_Mesh_Distance_BA_R0_R3	16,41775894	-79,9227066	-940,9556885	0,089325
Cloud_Mesh_Distance_BA_R0_R3	16,50766182	-80,00272369	-940,802124	0,074129
Cloud_Mesh_Distance_BA_R0_R3	23,08388519	-80,51327515	-941,1043091	-0,252824
Cloud_Mesh_Distance_BA_R0_R3	16,50489426	-79,85031891	-940,822876	0,078703
Cloud_Mesh_Distance_BA_R0_R3	23,00881577	-80,43809509	-940,8864136	-0,242022
Cloud_Mesh_Distance_BA_R0_R3	16,55037689	-79,70867157	-940,7788086	0,068894
Cloud_Mesh_Distance_BA_R0_R3	16,65885162	-79,66580963	-940,6219482	0,053767
Cloud_Mesh_Distance_BA_R0_R3	16,65729523	-79,51831055	-940,6706543	0,05604
Cloud_Mesh_Distance_BA_R0_R3	22,95140457	-79,92780304	-940,9865112	-0,151157
Cloud_Mesh_Distance_BA_R0_R3	22,97494125	-80,05073547	-940,9542847	-0,16782
Cloud_Mesh_Distance_BA_R0_R3	16,65800476	-79,37184906	-940,7232056	0,051776
Cloud_Mesh_Distance_BA_R0_R3	16,58730316	-79,33961487	-940,8387451	0,059022
Cloud_Mesh_Distance_BA_R0_R3	16,81441498	-79,13219452	-940,6027832	0,031412
Cloud_Mesh_Distance_BA_R0_R3	22,95541954	-79,83966827	-941,093689	-0,130371
Cloud_Mesh_Distance_BA_R0_R3	16,82789612	-78,97478485	-940,6630859	0,029086
Cloud_Mesh_Distance_BA_R0_R3	16,67583847	-78,90263367	-940,9014282	0,032667
Cloud_Mesh_Distance_BA_R0_R3	16,75391006	-79,03986359	-940,7322388	0,032307
Cloud_Mesh_Distance_BA_R0_R3	22,85124969	-79,57382965	-941,085144	-0,08223
Cloud_Mesh_Distance_BA_R0_R3	22,87029076	-79,6855545	-941,0012817	-0,106624
Cloud_Mesh_Distance_BA_R0_R3	16,68333435	-78,77353668	-940,951294	0,022316
Cloud_Mesh_Distance_BA_R0_R3	22,74451065	-79,29450989	-941,168335	-0,034339
Cloud_Mesh_Distance_BA_R0_R3	22,77625084	-79,46875763	-940,9967041	-0,067442
Cloud_Mesh_Distance_BA_R0_R3	16,84945679	-78,47112274	-940,9039917	0,009417
Cloud_Mesh_Distance_BA_R0_R3	16,74756432	-78,5666275	-940,9735107	0,01329
Cloud_Mesh_Distance_BA_R0_R3	16,85572624	-78,57068634	-940,8426514	0,012609
Cloud_Mesh_Distance_BA_R0_R3	16,81149673	-78,70968628	-940,8228149	0,020336
Cloud_Mesh_Distance_BA_R0_R3	22,64716148	-79,21863556	-940,9901733	-0,028507
Cloud_Mesh_Distance_BA_R0_R3	16,79828835	-78,39109802	-941,0060425	0,008706
Cloud_Mesh_Distance_BA_R0_R3	16,90579987	-78,34798431	-940,9102783	0,007124
Cloud_Mesh_Distance_BA_R0_R3	22,49006844	-79,01363373	-940,913147	-0,004884
Cloud_Mesh_Distance_BA_R0_R3	22,58215332	-79,08975983	-941,0180664	-0,01269
Cloud_Mesh_Distance_BA_R0_R3	16,98119545	-78,18157196	-940,9295654	0,008883
Cloud_Mesh_Distance_BA_R0_R3	22,46180725	-78,87955475	-941,072937	0,01038
Cloud_Mesh_Distance_BA_R0_R3	22,47462463	-78,83611298	-941,2011719	0,015454
Cloud_Mesh_Distance_BA_R0_R3	22,53985214	-78,93183136	-941,1957398	0,009498
Cloud_Mesh_Distance_BA_R0_R3	17,10322762	-78,08688354	-940,8654785	0,011792
Cloud_Mesh_Distance_BA_R0_R3	17,02338028	-77,98760223	-941,0160523	0,006029
Cloud_Mesh_Distance_BA_R0_R3	17,21255302	-78,05879974	-940,776062	0,016795
Cloud_Mesh_Distance_BA_R0_R3	22,10697174	-78,48214722	-941,0029297	0,01604
Cloud_Mesh_Distance_BA_R0_R3	22,165802	-78,55887604	-940,9841919	0,015451
Cloud_Mesh_Distance_BA_R0_R3	22,31529617	-78,6925354	-941,0653076	0,019628

Cloud_Mesh_Distance_BA_R0_R3	22,4023304	-78,72919464	-941,223999	0,021936
Cloud_Mesh_Distance_BA_R0_R3	17,17279243	-77,94033051	-940,9008789	0,018219
Cloud_Mesh_Distance_BA_R0_R3	17,26977539	-77,83535004	-940,888916	0,022043
Cloud_Mesh_Distance_BA_R0_R3	17,19178581	-77,81544495	-940,9788818	0,015599
Cloud_Mesh_Distance_BA_R0_R3	21,89794159	-78,22763062	-941,0695801	0,014776
Cloud_Mesh_Distance_BA_R0_R3	22,02383041	-78,30509186	-941,1979981	0,019447
Cloud_Mesh_Distance_BA_R0_R3	22,19480896	-78,55019379	-941,0640259	0,018695
Cloud_Mesh_Distance_BA_R0_R3	22,0272789	-78,36840057	-941,0579834	0,017204
Cloud_Mesh_Distance_BA_R0_R3	22,12059402	-78,4310379	-941,1415405	0,019941
Cloud_Mesh_Distance_BA_R0_R3	17,13248062	-77,76467896	-941,0754395	0,010003
Cloud_Mesh_Distance_BA_R0_R3	17,28695297	-77,67993927	-941,0021973	0,022149
Cloud_Mesh_Distance_BA_R0_R3	17,39038658	-77,80158234	-940,8069458	0,031544
Cloud_Mesh_Distance_BA_R0_R3	17,50899315	-77,66927338	-940,8184815	0,040073
Cloud_Mesh_Distance_BA_R0_R3	21,93894577	-78,19094849	-941,2730103	0,017665
Cloud_Mesh_Distance_BA_R0_R3	17,5995903	-77,51128387	-940,8927612	0,045333
Cloud_Mesh_Distance_BA_R0_R3	17,64437103	-77,59656525	-940,7755127	0,053757
Cloud_Mesh_Distance_BA_R0_R3	17,75592995	-77,53014374	-940,7568359	0,058281
Cloud_Mesh_Distance_BA_R0_R3	17,74743271	-77,41442108	-940,881958	0,05515
Cloud_Mesh_Distance_BA_R0_R3	21,50402069	-77,83098602	-941,0910645	0,008095
Cloud_Mesh_Distance_BA_R0_R3	21,78830719	-78,11336517	-941,076294	0,010988
Cloud_Mesh_Distance_BA_R0_R3	21,70916748	-78,01593781	-941,1226196	0,010446
Cloud_Mesh_Distance_BA_R0_R3	17,48885727	-77,4444809	-941,0453491	0,037628
Cloud_Mesh_Distance_BA_R0_R3	17,62611961	-77,37545013	-941,0092773	0,047783
Cloud_Mesh_Distance_BA_R0_R3	21,43805695	-77,741745	-941,1653442	0,005473
Cloud_Mesh_Distance_BA_R0_R3	21,39636421	-77,75401306	-941,0320435	0,009127
Cloud_Mesh_Distance_BA_R0_R3	17,76571846	-77,30348969	-940,9871826	0,055258
Cloud_Mesh_Distance_BA_R0_R3	17,90621185	-77,23877716	-940,9690552	0,058579
Cloud_Mesh_Distance_BA_R0_R3	18,02353287	-77,07165527	-941,1000977	0,060994
Cloud_Mesh_Distance_BA_R0_R3	18,19091606	-77,0790329	-941,0018921	0,065529
Cloud_Mesh_Distance_BA_R0_R3	18,29560089	-77,17262268	-940,8340454	0,068964
Cloud_Mesh_Distance_BA_R0_R3	18,30457687	-77,08248901	-940,9411621	0,070979
Cloud_Mesh_Distance_BA_R0_R3	21,03516769	-77,43210602	-941,067688	0,019201
Cloud_Mesh_Distance_BA_R0_R3	20,94546127	-77,39012909	-941,0203247	0,020912
Cloud_Mesh_Distance_BA_R0_R3	21,01060486	-77,48197174	-940,9300537	0,019701
Cloud_Mesh_Distance_BA_R0_R3	21,08665848	-77,41770172	-941,1981812	0,017887
Cloud_Mesh_Distance_BA_R0_R3	18,12260818	-76,98694611	-941,1533203	0,058159
Cloud_Mesh_Distance_BA_R0_R3	18,44724274	-76,92842865	-941,0810547	0,066707
Cloud_Mesh_Distance_BA_R0_R3	18,55211258	-76,9434433	-941,0216675	0,069291
Cloud_Mesh_Distance_BA_R0_R3	20,48542023	-77,183815	-940,9523926	0,009412
Cloud_Mesh_Distance_BA_R0_R3	20,5124588	-77,10587311	-941,1083984	0,023631
Cloud_Mesh_Distance_BA_R0_R3	20,66782379	-77,17815399	-941,1112671	0,025545
Cloud_Mesh_Distance_BA_R0_R3	20,84180069	-77,23305511	-941,2092285	0,027734
Cloud_Mesh_Distance_BA_R0_R3	18,76502609	-76,88980865	-941,0409546	0,063145
Cloud_Mesh_Distance_BA_R0_R3	18,57637596	-76,86717224	-941,121521	0,069981
Cloud_Mesh_Distance_BA_R0_R3	18,6626091	-76,96091461	-940,9642944	0,069533
Cloud_Mesh_Distance_BA_R0_R3	18,84038925	-76,81691742	-941,1393433	0,057903
Cloud_Mesh_Distance_BA_R0_R3	19,14062119	-76,85507965	-941,0704956	0,036176
Cloud_Mesh_Distance_BA_R0_R3	19,26788521	-76,93888092	-940,9473267	0,028445
Cloud_Mesh_Distance_BA_R0_R3	19,67406273	-76,94741058	-941,0053101	0,003772
Cloud_Mesh_Distance_BA_R0_R3	20,27147675	-76,93773651	-941,2589111	0,024381

Cloud_Mesh_Distance_BA_R0_R3	20,33907318	-76,99193573	-941,1962891	0,022774
Cloud_Mesh_Distance_BA_R0_R3	20,34824181	-77,05809021	-941,0803223	0,01386
Cloud_Mesh_Distance_BA_R0_R3	20,21098328	-76,97013855	-941,1615601	0,01601
Cloud_Mesh_Distance_BA_R0_R3	20,13074112	-77,02878571	-941,0197144	0,001715
Cloud_Mesh_Distance_BA_R0_R3	19,07711029	-76,77597809	-941,1983643	0,039004
Cloud_Mesh_Distance_BA_R0_R3	18,98406982	-76,81447601	-941,1333008	0,050068
Cloud_Mesh_Distance_BA_R0_R3	18,70715714	-83,81880951	-940,5466919	-0,107964
Cloud_Mesh_Distance_BA_R0_R3	18,70930099	-83,74300385	-940,4279175	-0,12823
Cloud_Mesh_Distance_BA_R0_R3	18,85093307	-83,79346466	-940,4085083	-0,134721
Cloud_Mesh_Distance_BA_R0_R3	19,02745819	-83,84046173	-940,3857422	-0,143078
Cloud_Mesh_Distance_BA_R0_R3	19,12513733	-83,93341827	-940,4939575	-0,135669
Cloud_Mesh_Distance_BA_R0_R3	19,5261879	-83,99202728	-940,4976196	-0,150716
Cloud_Mesh_Distance_BA_R0_R3	19,6483078	-84,03504181	-940,5603027	-0,142002
Cloud_Mesh_Distance_BA_R0_R3	19,6786747	-83,94731903	-940,416626	-0,147994
Cloud_Mesh_Distance_BA_R0_R3	19,85394669	-83,92188263	-940,3859863	-0,133826
Cloud_Mesh_Distance_BA_R0_R3	20,31565094	-84,00101471	-940,593689	-0,090987
Cloud_Mesh_Distance_BA_R0_R3	20,47659492	-84,01920319	-940,677063	-0,065676
Cloud_Mesh_Distance_BA_R0_R3	18,80392075	-83,69237518	-940,2923584	-0,147412
Cloud_Mesh_Distance_BA_R0_R3	18,61447906	-83,66175079	-940,3823242	-0,135329
Cloud_Mesh_Distance_BA_R0_R3	19,93716431	-83,83800507	-940,2807007	-0,134708
Cloud_Mesh_Distance_BA_R0_R3	20,02417183	-83,93021393	-940,4208984	-0,120469
Cloud_Mesh_Distance_BA_R0_R3	20,13318062	-83,84017181	-940,3221436	-0,114872
Cloud_Mesh_Distance_BA_R0_R3	20,41471863	-83,84090424	-940,4119873	-0,086999
Cloud_Mesh_Distance_BA_R0_R3	20,24510193	-83,91719818	-940,4544678	-0,103194
Cloud_Mesh_Distance_BA_R0_R3	20,4376564	-83,93094635	-940,5383911	-0,078388
Cloud_Mesh_Distance_BA_R0_R3	20,73105049	-83,91764069	-940,6539307	-0,036462
Cloud_Mesh_Distance_BA_R0_R3	20,57419968	-83,94330597	-940,6127319	-0,057772
Cloud_Mesh_Distance_BA_R0_R3	20,89921761	-83,8707962	-940,6852417	-0,019168
Cloud_Mesh_Distance_BA_R0_R3	20,83347702	-83,80579376	-940,5722656	-0,037048
Cloud_Mesh_Distance_BA_R0_R3	18,20583344	-83,4210434	-940,4226685	-0,121784
Cloud_Mesh_Distance_BA_R0_R3	18,37402725	-83,43837738	-940,2739868	-0,141986
Cloud_Mesh_Distance_BA_R0_R3	18,34780121	-83,54759979	-940,4598999	-0,119789
Cloud_Mesh_Distance_BA_R0_R3	18,49911499	-83,67209625	-940,4984131	-0,115146
Cloud_Mesh_Distance_BA_R0_R3	20,60781097	-83,85100555	-940,5092163	-0,061109
Cloud_Mesh_Distance_BA_R0_R3	20,89902878	-83,68314362	-940,4724731	-0,041277
Cloud_Mesh_Distance_BA_R0_R3	21,07592392	-83,63799286	-940,532898	-0,021641
Cloud_Mesh_Distance_BA_R0_R3	17,83847427	-83,12256622	-940,446167	-0,111772
Cloud_Mesh_Distance_BA_R0_R3	21,10682297	-83,52387238	-940,4413452	-0,028025
Cloud_Mesh_Distance_BA_R0_R3	21,28077698	-83,59072113	-940,6147461	0,008931
Cloud_Mesh_Distance_BA_R0_R3	21,6303978	-83,43312836	-940,6864624	0,061642
Cloud_Mesh_Distance_BA_R0_R3	17,63428116	-82,93601227	-940,4938965	-0,10449
Cloud_Mesh_Distance_BA_R0_R3	21,30567551	-83,4371109	-940,4949341	-0,009381
Cloud_Mesh_Distance_BA_R0_R3	21,48332596	-83,43524933	-940,6063843	0,021555
Cloud_Mesh_Distance_BA_R0_R3	21,48134804	-83,29781342	-940,5003662	-0,003623
Cloud_Mesh_Distance_BA_R0_R3	17,60966492	-82,85837555	-940,4188843	-0,109019
Cloud_Mesh_Distance_BA_R0_R3	17,5423851	-82,86334991	-940,5509033	-0,099439
Cloud_Mesh_Distance_BA_R0_R3	21,53162575	-83,18097687	-940,4492188	-0,013786
Cloud_Mesh_Distance_BA_R0_R3	21,66997147	-83,15355682	-940,5233765	0,008002
Cloud_Mesh_Distance_BA_R0_R3	21,59406281	-83,11569977	-940,4469605	-0,015249
Cloud_Mesh_Distance_BA_R0_R3	21,67857933	-83,30489349	-940,6258545	0,04526

Cloud_Mesh_Distance_BA_R0_R3	22,0080986	-83,11911774	-940,6994629	0,095684
Cloud_Mesh_Distance_BA_R0_R3	21,86257553	-83,15164948	-940,6380615	0,056417
Cloud_Mesh_Distance_BA_R0_R3	22,01466751	-83,01432037	-940,6581421	0,065938
Cloud_Mesh_Distance_BA_R0_R3	17,35445595	-82,46294403	-940,3520508	-0,091415
Cloud_Mesh_Distance_BA_R0_R3	17,220438	-82,37662506	-940,4963379	-0,074331
Cloud_Mesh_Distance_BA_R0_R3	17,28105545	-82,32207489	-940,326355	-0,085323
Cloud_Mesh_Distance_BA_R0_R3	21,85908508	-82,99077606	-940,5491943	0,017812
Cloud_Mesh_Distance_BA_R0_R3	21,79704285	-82,93149567	-940,4728394	-0,008473
Cloud_Mesh_Distance_BA_R0_R3	22,08123016	-82,83315277	-940,6231079	0,03262
Cloud_Mesh_Distance_BA_R0_R3	17,20459366	-82,21147919	-940,3483276	-0,079156
Cloud_Mesh_Distance_BA_R0_R3	17,10189438	-82,14720917	-940,46698	-0,059239
Cloud_Mesh_Distance_BA_R0_R3	17,13991165	-82,25940704	-940,5135498	-0,063524
Cloud_Mesh_Distance_BA_R0_R3	22,21292305	-82,49514008	-940,5829468	-0,026606
Cloud_Mesh_Distance_BA_R0_R3	22,16458321	-82,64521027	-940,607666	0,000749
Cloud_Mesh_Distance_BA_R0_R3	22,28027344	-82,70900726	-940,7233276	0,047052
Cloud_Mesh_Distance_BA_R0_R3	22,21434021	-82,83689117	-940,7157593	0,071627
Cloud_Mesh_Distance_BA_R0_R3	16,90925026	-81,88472748	-940,5872803	-0,0189
Cloud_Mesh_Distance_BA_R0_R3	16,91641235	-81,7559433	-940,4769287	-0,025786
Cloud_Mesh_Distance_BA_R0_R3	16,85376358	-81,76857758	-940,6037598	-0,006595
Cloud_Mesh_Distance_BA_R0_R3	17,07360649	-82,02965546	-940,4108276	-0,058041
Cloud_Mesh_Distance_BA_R0_R3	22,28383446	-82,55921173	-940,6750488	0,002251
Cloud_Mesh_Distance_BA_R0_R3	22,33757401	-82,4300766	-940,673584	-0,020825
Cloud_Mesh_Distance_BA_R0_R3	16,91499329	-81,643013	-940,4089966	-0,034182
Cloud_Mesh_Distance_BA_R0_R3	22,2706337	-82,35135651	-940,5691528	-0,050197
Cloud_Mesh_Distance_BA_R0_R3	22,37651062	-82,11203766	-940,5570679	-0,079956
Cloud_Mesh_Distance_BA_R0_R3	22,39593124	-82,27933502	-940,6680908	-0,048542
Cloud_Mesh_Distance_BA_R0_R3	22,52211952	-82,15570831	-940,7573242	-0,055411
Cloud_Mesh_Distance_BA_R0_R3	16,85521698	-81,31595612	-940,3635864	-0,036624
Cloud_Mesh_Distance_BA_R0_R3	22,46200371	-81,89704132	-940,5358276	-0,104599
Cloud_Mesh_Distance_BA_R0_R3	22,59100914	-81,85808563	-940,6884155	-0,102434
Cloud_Mesh_Distance_BA_R0_R3	22,52037048	-82,00034332	-940,6730347	-0,08375
Cloud_Mesh_Distance_BA_R0_R3	16,78351593	-81,22872925	-940,4647217	-0,018607
Cloud_Mesh_Distance_BA_R0_R3	16,7491188	-81,06951904	-940,484436	-0,016177
Cloud_Mesh_Distance_BA_R0_R3	16,71777916	-81,26177216	-940,6055908	0,000202
Cloud_Mesh_Distance_BA_R0_R3	22,56376266	-81,74819183	-940,5838013	-0,121195
Cloud_Mesh_Distance_BA_R0_R3	16,66553879	-81,08661652	-940,6546631	0,006506
Cloud_Mesh_Distance_BA_R0_R3	22,605896	-81,57912445	-940,5459595	-0,147015
Cloud_Mesh_Distance_BA_R0_R3	22,79286194	-81,44467926	-940,7645264	-0,167372
Cloud_Mesh_Distance_BA_R0_R3	22,71686554	-81,52014923	-940,6791382	-0,156105
Cloud_Mesh_Distance_BA_R0_R3	22,78212166	-81,59726715	-940,8366699	-0,135113
Cloud_Mesh_Distance_BA_R0_R3	22,67056274	-81,69577789	-940,7097168	-0,129582
Cloud_Mesh_Distance_BA_R0_R3	22,73445511	-81,74131012	-940,8405762	-0,113808
Cloud_Mesh_Distance_BA_R0_R3	22,83092308	-81,30863953	-940,7581177	-0,192972
Cloud_Mesh_Distance_BA_R0_R3	22,75348663	-81,37145996	-940,6572266	-0,184435
Cloud_Mesh_Distance_BA_R0_R3	16,65981674	-80,57527924	-940,5633545	0,009608
Cloud_Mesh_Distance_BA_R0_R3	22,78506851	-81,22926331	-940,6400757	-0,207875
Cloud_Mesh_Distance_BA_R0_R3	22,94726372	-81,008461	-940,8461304	-0,245485
Cloud_Mesh_Distance_BA_R0_R3	16,62427902	-80,37760162	-940,6088257	0,025911
Cloud_Mesh_Distance_BA_R0_R3	16,65385437	-80,44739532	-940,5620728	0,012652
Cloud_Mesh_Distance_BA_R0_R3	22,85694504	-81,15000153	-940,7285156	-0,218593

Cloud_Mesh_Distance_BA_R0_R3	23,00419426	-80,82006073	-940,9092407	-0,266764
Cloud_Mesh_Distance_BA_R0_R3	16,74096298	-80,27680206	-940,404541	0,002994
Cloud_Mesh_Distance_BA_R0_R3	16,65269279	-80,20694733	-940,5556641	0,02745
Cloud_Mesh_Distance_BA_R0_R3	23,01460457	-80,64617157	-940,9012451	-0,270644
Cloud_Mesh_Distance_BA_R0_R3	22,92913818	-80,78765106	-940,7376099	-0,256564
Cloud_Mesh_Distance_BA_R0_R3	16,66448975	-80,01241302	-940,5476074	0,036546
Cloud_Mesh_Distance_BA_R0_R3	22,94116592	-80,46555328	-940,729187	-0,238873
Cloud_Mesh_Distance_BA_R0_R3	22,90871048	-80,52831268	-940,6659546	-0,243444
Cloud_Mesh_Distance_BA_R0_R3	16,59287643	-79,88103485	-940,6786499	0,059529
Cloud_Mesh_Distance_BA_R0_R3	16,74022484	-79,62441254	-940,5098267	0,040155
Cloud_Mesh_Distance_BA_R0_R3	16,79985046	-79,67778778	-940,401062	0,028355
Cloud_Mesh_Distance_BA_R0_R3	22,94628906	-80,3454361	-940,7545776	-0,225366
Cloud_Mesh_Distance_BA_R0_R3	22,89089584	-80,27974701	-940,6586914	-0,204868
Cloud_Mesh_Distance_BA_R0_R3	22,9939537	-80,2252121	-940,9057617	-0,198688
Cloud_Mesh_Distance_BA_R0_R3	16,75176239	-79,38237762	-940,579834	0,040419
Cloud_Mesh_Distance_BA_R0_R3	22,80462265	-79,94363403	-940,6426392	-0,156798
Cloud_Mesh_Distance_BA_R0_R3	22,84650421	-79,84506989	-940,7876587	-0,13348
Cloud_Mesh_Distance_BA_R0_R3	22,90463829	-79,97255707	-940,8268433	-0,153653
Cloud_Mesh_Distance_BA_R0_R3	16,70342445	-79,17550659	-940,7394409	0,042947
Cloud_Mesh_Distance_BA_R0_R3	16,75222397	-79,27387238	-940,6257324	0,039745
Cloud_Mesh_Distance_BA_R0_R3	22,9037056	-79,81385803	-940,9569702	-0,129255
Cloud_Mesh_Distance_BA_R0_R3	22,83282089	-79,72290802	-940,8600464	-0,114159
Cloud_Mesh_Distance_BA_R0_R3	22,7630806	-79,59749603	-940,8166504	-0,093605
Cloud_Mesh_Distance_BA_R0_R3	22,8230896	-79,6142807	-940,9477539	-0,097049
Cloud_Mesh_Distance_BA_R0_R3	16,75841904	-78,89280701	-940,7979126	0,025976
Cloud_Mesh_Distance_BA_R0_R3	16,89123344	-78,82113647	-940,6617432	0,01983
Cloud_Mesh_Distance_BA_R0_R3	16,92937088	-78,62711334	-940,7230225	0,015681
Cloud_Mesh_Distance_BA_R0_R3	22,5802269	-79,29284668	-940,7427368	-0,041855
Cloud_Mesh_Distance_BA_R0_R3	22,62940025	-79,26883698	-940,8745728	-0,039978
Cloud_Mesh_Distance_BA_R0_R3	22,68689537	-79,430336	-940,8161011	-0,066384
Cloud_Mesh_Distance_BA_R0_R3	16,93793678	-78,47206879	-940,802124	0,011029
Cloud_Mesh_Distance_BA_R0_R3	17,01207352	-78,70368195	-940,5784912	0,014452
Cloud_Mesh_Distance_BA_R0_R3	17,06944275	-78,80782318	-940,4474487	0,015404
Cloud_Mesh_Distance_BA_R0_R3	22,44239235	-79,05973053	-940,7562866	-0,019876
Cloud_Mesh_Distance_BA_R0_R3	22,69736862	-79,31401062	-940,9855347	-0,039377
Cloud_Mesh_Distance_BA_R0_R3	17,20976639	-78,34901428	-940,579895	0,012575
Cloud_Mesh_Distance_BA_R0_R3	17,06873703	-78,48440552	-940,6451416	0,014136
Cloud_Mesh_Distance_BA_R0_R3	17,18467522	-78,48844147	-940,5153198	0,011337
Cloud_Mesh_Distance_BA_R0_R3	22,54547119	-79,15859222	-940,8304443	-0,025022
Cloud_Mesh_Distance_BA_R0_R3	17,01669312	-78,32823944	-940,7995606	0,008996
Cloud_Mesh_Distance_BA_R0_R3	17,07696152	-78,20592499	-940,8128052	0,012595
Cloud_Mesh_Distance_BA_R0_R3	17,12845421	-78,28115845	-940,7090454	0,012913
Cloud_Mesh_Distance_BA_R0_R3	17,35215759	-78,25016785	-940,5061646	0,019748
Cloud_Mesh_Distance_BA_R0_R3	22,26408386	-78,74130249	-940,8723145	0,007811
Cloud_Mesh_Distance_BA_R0_R3	22,21804047	-78,62369537	-940,9760132	0,016117
Cloud_Mesh_Distance_BA_R0_R3	22,18033981	-78,70676422	-940,7746582	-0,003028
Cloud_Mesh_Distance_BA_R0_R3	22,24402428	-78,8090744	-940,7388916	-0,005015
Cloud_Mesh_Distance_BA_R0_R3	22,33064651	-78,90312958	-940,7599487	-0,008089
Cloud_Mesh_Distance_BA_R0_R3	22,38976479	-78,88008881	-940,9047852	0,005968
Cloud_Mesh_Distance_BA_R0_R3	17,2455349	-78,17206573	-940,6633911	0,016917

Cloud_Mesh_Distance_BA_R0_R3	17,28700829	-78,05902863	-940,703125	0,020438
Cloud_Mesh_Distance_BA_R0_R3	17,15381432	-78,15895844	-940,7648926	0,017655
Cloud_Mesh_Distance_BA_R0_R3	21,96343613	-78,51146698	-940,7034302	-0,000426
Cloud_Mesh_Distance_BA_R0_R3	22,14401245	-78,58860779	-940,890564	0,00951
Cloud_Mesh_Distance_BA_R0_R3	17,48957253	-77,78381348	-940,7349854	0,038372
Cloud_Mesh_Distance_BA_R0_R3	17,312397	-77,9493103	-940,7603149	0,026749
Cloud_Mesh_Distance_BA_R0_R3	17,48267937	-77,90310669	-940,6425171	0,035942
Cloud_Mesh_Distance_BA_R0_R3	21,78995514	-78,21781158	-940,8674316	0,010154
Cloud_Mesh_Distance_BA_R0_R3	21,98517799	-78,41834259	-940,8800659	0,007316
Cloud_Mesh_Distance_BA_R0_R3	17,65508461	-77,668396	-940,7003174	0,052831
Cloud_Mesh_Distance_BA_R0_R3	17,79931641	-77,63894653	-940,6164551	0,058792
Cloud_Mesh_Distance_BA_R0_R3	21,55923462	-78,01798248	-940,8051758	0,014793
Cloud_Mesh_Distance_BA_R0_R3	21,61856079	-78,09331512	-940,7776489	0,012721
Cloud_Mesh_Distance_BA_R0_R3	21,67469978	-78,15467072	-940,7716065	0,009718
Cloud_Mesh_Distance_BA_R0_R3	21,72108841	-78,08746338	-940,9824219	0,010034
Cloud_Mesh_Distance_BA_R0_R3	21,65772247	-78,08492279	-940,8615723	0,011015
Cloud_Mesh_Distance_BA_R0_R3	21,79771423	-78,15816498	-940,998291	0,013089
Cloud_Mesh_Distance_BA_R0_R3	17,71211433	-77,60820007	-940,7127686	0,056903
Cloud_Mesh_Distance_BA_R0_R3	17,93665314	-77,51450348	-940,6447754	0,069992
Cloud_Mesh_Distance_BA_R0_R3	17,86616516	-77,43545532	-940,7775879	0,061196
Cloud_Mesh_Distance_BA_R0_R3	18,0854969	-77,45952606	-940,6081543	0,072312
Cloud_Mesh_Distance_BA_R0_R3	21,46619797	-77,84905243	-940,9616089	0,009029
Cloud_Mesh_Distance_BA_R0_R3	21,58898544	-77,95821381	-940,9794922	0,007444
Cloud_Mesh_Distance_BA_R0_R3	21,47987366	-77,91330719	-940,8568726	0,014923
Cloud_Mesh_Distance_BA_R0_R3	17,8840065	-77,34169769	-940,868042	0,06165
Cloud_Mesh_Distance_BA_R0_R3	18,01549721	-77,38563538	-940,7357178	0,067689
Cloud_Mesh_Distance_BA_R0_R3	18,16325378	-77,38990021	-940,6413574	0,072762
Cloud_Mesh_Distance_BA_R0_R3	18,17061996	-77,28980255	-940,7560425	0,070624
Cloud_Mesh_Distance_BA_R0_R3	21,16153336	-77,53632355	-941,0586548	0,016332
Cloud_Mesh_Distance_BA_R0_R3	21,2776947	-77,67643738	-940,9658814	0,014572
Cloud_Mesh_Distance_BA_R0_R3	18,00968552	-77,28458405	-940,854187	0,067516
Cloud_Mesh_Distance_BA_R0_R3	18,07406425	-77,17926788	-940,9408569	0,063058
Cloud_Mesh_Distance_BA_R0_R3	18,40449524	-77,08690643	-940,8912964	0,069568
Cloud_Mesh_Distance_BA_R0_R3	18,49943161	-77,09617615	-940,8401489	0,071202
Cloud_Mesh_Distance_BA_R0_R3	18,47423935	-77,15814209	-940,7719727	0,068825
Cloud_Mesh_Distance_BA_R0_R3	18,30709839	-77,26909637	-940,7097168	0,074474
Cloud_Mesh_Distance_BA_R0_R3	19,05171776	-77,07175446	-940,7473755	0,04682
Cloud_Mesh_Distance_BA_R0_R3	19,03794098	-77,1681366	-940,6137085	0,046385
Cloud_Mesh_Distance_BA_R0_R3	19,30045509	-77,14532471	-940,6422119	0,023708
Cloud_Mesh_Distance_BA_R0_R3	20,68663025	-77,39096832	-940,7626953	0,001851
Cloud_Mesh_Distance_BA_R0_R3	20,57907104	-77,34631348	-940,7572632	-0,003423
Cloud_Mesh_Distance_BA_R0_R3	20,76180267	-77,34903717	-940,890686	0,01323
Cloud_Mesh_Distance_BA_R0_R3	20,86424446	-77,46111298	-940,8019409	0,014737
Cloud_Mesh_Distance_BA_R0_R3	18,52301788	-77,02237701	-940,9277954	0,070671
Cloud_Mesh_Distance_BA_R0_R3	18,70409775	-77,06132507	-940,8182983	0,067667
Cloud_Mesh_Distance_BA_R0_R3	18,58999252	-77,11878204	-940,7781982	0,069932
Cloud_Mesh_Distance_BA_R0_R3	18,87420464	-77,12839508	-940,6904297	0,060299
Cloud_Mesh_Distance_BA_R0_R3	19,23198318	-77,08389282	-940,7281494	0,031045
Cloud_Mesh_Distance_BA_R0_R3	19,35467339	-77,10269928	-940,7089233	0,018837
Cloud_Mesh_Distance_BA_R0_R3	19,3561039	-77,03025055	-940,8162842	0,020376

Cloud_Mesh_Distance_BA_R0_R3	19,47682571	-77,09819031	-940,7297363	0,010968
Cloud_Mesh_Distance_BA_R0_R3	19,96197891	-77,12702179	-940,7989502	-0,013569
Cloud_Mesh_Distance_BA_R0_R3	19,97048569	-77,06661224	-940,8967896	-0,007518
Cloud_Mesh_Distance_BA_R0_R3	20,10680389	-77,1081543	-940,8796997	-0,008076
Cloud_Mesh_Distance_BA_R0_R3	20,02669907	-77,04724884	-940,9474487	-0,004751
Cloud_Mesh_Distance_BA_R0_R3	20,09369278	-77,18292236	-940,7601929	-0,014827
Cloud_Mesh_Distance_BA_R0_R3	20,27324677	-77,1135788	-940,9447022	0,002989
Cloud_Mesh_Distance_BA_R0_R3	20,61366272	-77,30084229	-940,8497315	0,006107
Cloud_Mesh_Distance_BA_R0_R3	20,67521858	-77,26008606	-940,9665527	0,017563
Cloud_Mesh_Distance_BA_R0_R3	20,82415581	-77,30374908	-941,0366211	0,023714
Cloud_Mesh_Distance_BA_R0_R3	20,88444138	-77,39511871	-940,9352417	0,019888
Cloud_Mesh_Distance_BA_R0_R3	18,8613224	-76,99517059	-940,8743286	0,058814
Cloud_Mesh_Distance_BA_R0_R3	19,1104126	-76,9328537	-940,9475098	0,042545
Cloud_Mesh_Distance_BA_R0_R3	18,95874023	-76,89464569	-941,0082398	0,05571
Cloud_Mesh_Distance_BA_R0_R3	19,17126083	-77,00431061	-940,841919	0,037193
Cloud_Mesh_Distance_BA_R0_R3	19,02794266	-76,97848511	-940,8812866	0,050061
Cloud_Mesh_Distance_BA_R0_R3	19,48766708	-76,95879364	-940,9462891	0,011712
Cloud_Mesh_Distance_BA_R0_R3	19,52262115	-77,03999329	-940,8250122	0,007483
Cloud_Mesh_Distance_BA_R0_R3	19,68934441	-77,02937317	-940,8757324	-0,000227
Cloud_Mesh_Distance_BA_R0_R3	19,89263153	-76,98807526	-940,9993897	-0,003106
Cloud_Mesh_Distance_BA_R0_R3	19,1755867	-83,85965729	-940,3578491	-0,151493
Cloud_Mesh_Distance_BA_R0_R3	19,30305672	-83,89801788	-940,3817749	-0,154796
Cloud_Mesh_Distance_BA_R0_R3	19,48048592	-83,92307281	-940,3909912	-0,15257
Cloud_Mesh_Distance_BA_R0_R3	19,60840988	-83,85706329	-940,2835083	-0,149385
Cloud_Mesh_Distance_BA_R0_R3	19,14958191	-83,79033661	-940,2654419	-0,15497
Cloud_Mesh_Distance_BA_R0_R3	18,98317719	-83,7485733	-940,2731323	-0,153595
Cloud_Mesh_Distance_BA_R0_R3	19,31860733	-83,75096893	-940,1682739	-0,159643
Cloud_Mesh_Distance_BA_R0_R3	19,42053795	-83,83971405	-940,2738037	-0,154821
Cloud_Mesh_Distance_BA_R0_R3	19,27332306	-83,82874298	-940,2844238	-0,15516
Cloud_Mesh_Distance_BA_R0_R3	19,76107407	-83,77848053	-940,1838989	-0,147427
Cloud_Mesh_Distance_BA_R0_R3	19,5394516	-83,76584625	-940,1624756	-0,154831
Cloud_Mesh_Distance_BA_R0_R3	19,76811218	-83,87058258	-940,3059692	-0,14106
Cloud_Mesh_Distance_BA_R0_R3	20,08290863	-83,75756073	-940,2080689	-0,116791
Cloud_Mesh_Distance_BA_R0_R3	19,9373436	-83,74732208	-940,1665649	-0,130464
Cloud_Mesh_Distance_BA_R0_R3	20,27941132	-83,82413483	-940,3428955	-0,098106
Cloud_Mesh_Distance_BA_R0_R3	18,26269531	-83,29177094	-940,1778565	-0,145673
Cloud_Mesh_Distance_BA_R0_R3	18,24782944	-83,34287262	-940,2623901	-0,135908
Cloud_Mesh_Distance_BA_R0_R3	18,36846161	-83,33931732	-940,144104	-0,149368
Cloud_Mesh_Distance_BA_R0_R3	18,54371834	-83,54813385	-940,2792358	-0,141308
Cloud_Mesh_Distance_BA_R0_R3	18,70623016	-83,60990143	-940,2426148	-0,146831
Cloud_Mesh_Distance_BA_R0_R3	20,37733841	-83,75580597	-940,295166	-0,087929
Cloud_Mesh_Distance_BA_R0_R3	20,55075455	-83,75041962	-940,3607178	-0,073263
Cloud_Mesh_Distance_BA_R0_R3	20,72130966	-83,74925995	-940,4441528	-0,059449
Cloud_Mesh_Distance_BA_R0_R3	20,47022438	-83,64218903	-940,2063599	-0,084005
Cloud_Mesh_Distance_BA_R0_R3	20,82322693	-83,53598785	-940,2748413	-0,058497
Cloud_Mesh_Distance_BA_R0_R3	20,998312	-83,608284	-940,4541016	-0,037763
Cloud_Mesh_Distance_BA_R0_R3	17,79138184	-83,03514862	-940,3833618	-0,110424
Cloud_Mesh_Distance_BA_R0_R3	17,86245728	-83,07176971	-940,3325806	-0,117323
Cloud_Mesh_Distance_BA_R0_R3	17,92084122	-83,0442276	-940,2174683	-0,124804
Cloud_Mesh_Distance_BA_R0_R3	17,95245743	-83,14829254	-940,3233643	-0,125257

Cloud_Mesh_Distance_BA_R0_R3	18,1026535	-83,27428436	-940,3226929	-0,123166
Cloud_Mesh_Distance_BA_R0_R3	18,19958687	-83,28588104	-940,2323608	-0,134814
Cloud_Mesh_Distance_BA_R0_R3	18,19852066	-83,23587799	-940,1655273	-0,141649
Cloud_Mesh_Distance_BA_R0_R3	20,96735382	-83,47928619	-940,3082886	-0,044955
Cloud_Mesh_Distance_BA_R0_R3	20,91793442	-83,58170319	-940,3771362	-0,048354
Cloud_Mesh_Distance_BA_R0_R3	17,82621193	-83,00823212	-940,2938843	-0,117566
Cloud_Mesh_Distance_BA_R0_R3	21,28348351	-83,32968903	-940,3902588	-0,029396
Cloud_Mesh_Distance_BA_R0_R3	21,11729622	-83,40807343	-940,3414307	-0,039434
Cloud_Mesh_Distance_BA_R0_R3	21,20165253	-83,42749786	-940,4168091	-0,024879
Cloud_Mesh_Distance_BA_R0_R3	17,62684631	-82,80884552	-940,3212891	-0,109297
Cloud_Mesh_Distance_BA_R0_R3	17,59137726	-82,7201767	-940,2626343	-0,107344
Cloud_Mesh_Distance_BA_R0_R3	17,85008621	-82,86730194	-940,0872803	-0,1328
Cloud_Mesh_Distance_BA_R0_R3	17,69167137	-82,78194427	-940,1900635	-0,116681
Cloud_Mesh_Distance_BA_R0_R3	17,7273941	-82,92528534	-940,3233032	-0,112438
Cloud_Mesh_Distance_BA_R0_R3	17,84407806	-82,9633255	-940,2110596	-0,12555
Cloud_Mesh_Distance_BA_R0_R3	21,22431183	-83,15726471	-940,2108765	-0,046365
Cloud_Mesh_Distance_BA_R0_R3	21,42171478	-83,22553253	-940,4047241	-0,02425
Cloud_Mesh_Distance_BA_R0_R3	21,32050133	-83,20269012	-940,31427	-0,037764
Cloud_Mesh_Distance_BA_R0_R3	17,56505585	-82,51490021	-940,0762329	-0,11872
Cloud_Mesh_Distance_BA_R0_R3	17,4995842	-82,59899139	-940,263794	-0,107432
Cloud_Mesh_Distance_BA_R0_R3	17,52871704	-82,75226593	-940,4105835	-0,101931
Cloud_Mesh_Distance_BA_R0_R3	21,35680389	-83,09468842	-940,2595215	-0,043128
Cloud_Mesh_Distance_BA_R0_R3	21,74760437	-82,88576508	-940,4091797	-0,026955
Cloud_Mesh_Distance_BA_R0_R3	21,68146324	-83,01909637	-940,4454346	-0,017092
Cloud_Mesh_Distance_BA_R0_R3	21,49615288	-83,09864044	-940,3643189	-0,031176
Cloud_Mesh_Distance_BA_R0_R3	21,69055176	-82,82791901	-940,3289185	-0,043183
Cloud_Mesh_Distance_BA_R0_R3	21,89732933	-82,82501984	-940,4869385	-0,011304
Cloud_Mesh_Distance_BA_R0_R3	21,94628525	-82,6685257	-940,437439	-0,033406
Cloud_Mesh_Distance_BA_R0_R3	21,80894089	-82,75562286	-940,3777466	-0,039189
Cloud_Mesh_Distance_BA_R0_R3	17,12402344	-81,96399689	-940,274231	-0,072958
Cloud_Mesh_Distance_BA_R0_R3	17,14112854	-82,08579254	-940,3421021	-0,071577
Cloud_Mesh_Distance_BA_R0_R3	17,28006172	-82,25019073	-940,2585449	-0,090816
Cloud_Mesh_Distance_BA_R0_R3	17,28346252	-82,16286469	-940,1763306	-0,09787
Cloud_Mesh_Distance_BA_R0_R3	21,85763168	-82,61312103	-940,331665	-0,051199
Cloud_Mesh_Distance_BA_R0_R3	22,03579712	-82,70890045	-940,5319824	-0,010788
Cloud_Mesh_Distance_BA_R0_R3	17,01613808	-81,86746979	-940,3812256	-0,049554
Cloud_Mesh_Distance_BA_R0_R3	17,20715141	-81,88886261	-940,095459	-0,09816
Cloud_Mesh_Distance_BA_R0_R3	21,99031067	-82,53304291	-940,3990479	-0,048754
Cloud_Mesh_Distance_BA_R0_R3	22,0809536	-82,57714081	-940,503418	-0,028939
Cloud_Mesh_Distance_BA_R0_R3	22,12482071	-82,42031097	-940,4599609	-0,051654
Cloud_Mesh_Distance_BA_R0_R3	17,0632515	-81,66220856	-940,1798096	-0,075038
Cloud_Mesh_Distance_BA_R0_R3	16,97304153	-81,70609283	-940,3478394	-0,04554
Cloud_Mesh_Distance_BA_R0_R3	17,11030388	-81,83034515	-940,204895	-0,077292
Cloud_Mesh_Distance_BA_R0_R3	22,3176918	-82,24604034	-940,5644531	-0,064714
Cloud_Mesh_Distance_BA_R0_R3	16,96252823	-81,57949066	-940,2952271	-0,052652
Cloud_Mesh_Distance_BA_R0_R3	22,13383102	-82,21651459	-940,3520508	-0,073918
Cloud_Mesh_Distance_BA_R0_R3	22,21821594	-82,24932098	-940,4588623	-0,068712
Cloud_Mesh_Distance_BA_R0_R3	22,3385582	-81,98635101	-940,4370117	-0,093737
Cloud_Mesh_Distance_BA_R0_R3	16,88542366	-81,51563263	-940,394165	-0,033241
Cloud_Mesh_Distance_BA_R0_R3	16,94063187	-81,42760468	-940,2617188	-0,050652

Cloud_Mesh_Distance_BA_R0_R3	22,34758377	-81,8645401	-940,3746338	-0,102623
Cloud_Mesh_Distance_BA_R0_R3	16,82172394	-81,14123535	-940,367981	-0,034324
Cloud_Mesh_Distance_BA_R0_R3	22,51540375	-81,54810333	-940,4069214	-0,143348
Cloud_Mesh_Distance_BA_R0_R3	16,82289505	-80,98230743	-940,3270264	-0,039327
Cloud_Mesh_Distance_BA_R0_R3	16,7626152	-80,88375854	-940,4177246	-0,023732
Cloud_Mesh_Distance_BA_R0_R3	16,88490677	-80,89627838	-940,2045898	-0,055298
Cloud_Mesh_Distance_BA_R0_R3	16,82302094	-80,79682922	-940,2943115	-0,039245
Cloud_Mesh_Distance_BA_R0_R3	22,67799187	-81,43199921	-940,5714722	-0,172646
Cloud_Mesh_Distance_BA_R0_R3	16,73959351	-80,67792511	-940,4296265	-0,016841
Cloud_Mesh_Distance_BA_R0_R3	16,82902908	-80,63334656	-940,265625	-0,035009
Cloud_Mesh_Distance_BA_R0_R3	22,64525986	-81,20375824	-940,4240112	-0,190567
Cloud_Mesh_Distance_BA_R0_R3	22,70341492	-81,29318237	-940,5419312	-0,19077
Cloud_Mesh_Distance_BA_R0_R3	22,68083572	-81,08150482	-940,428894	-0,205462
Cloud_Mesh_Distance_BA_R0_R3	22,74463272	-81,12418365	-940,5340576	-0,211052
Cloud_Mesh_Distance_BA_R0_R3	16,71017838	-80,46294403	-940,4630127	-0,002925
Cloud_Mesh_Distance_BA_R0_R3	16,80067253	-80,45931244	-940,3040772	-0,021893
Cloud_Mesh_Distance_BA_R0_R3	16,82694817	-80,27298737	-940,2596436	-0,016494
Cloud_Mesh_Distance_BA_R0_R3	22,77154541	-80,74649811	-940,4691162	-0,234239
Cloud_Mesh_Distance_BA_R0_R3	22,83099174	-80,82053375	-940,5727539	-0,244687
Cloud_Mesh_Distance_BA_R0_R3	22,8405838	-80,99463654	-940,6381836	-0,238868
Cloud_Mesh_Distance_BA_R0_R3	22,7280159	-80,9360733	-940,4500732	-0,225153
Cloud_Mesh_Distance_BA_R0_R3	22,80807495	-81,12503815	-940,6329346	-0,224786
Cloud_Mesh_Distance_BA_R0_R3	16,72297668	-80,10637665	-940,4429932	0,018925
Cloud_Mesh_Distance_BA_R0_R3	22,86058044	-80,63217926	-940,588501	-0,241369
Cloud_Mesh_Distance_BA_R0_R3	16,74725723	-79,95111847	-940,4211426	0,022296
Cloud_Mesh_Distance_BA_R0_R3	16,76121902	-79,80869293	-940,4266968	0,029318
Cloud_Mesh_Distance_BA_R0_R3	16,69268036	-79,83956909	-940,5285645	0,040825
Cloud_Mesh_Distance_BA_R0_R3	16,8097229	-80,08808136	-940,3017578	0,000911
Cloud_Mesh_Distance_BA_R0_R3	16,83260727	-79,85480499	-940,3034668	0,013509
Cloud_Mesh_Distance_BA_R0_R3	22,7317028	-80,4920578	-940,3979492	-0,206793
Cloud_Mesh_Distance_BA_R0_R3	22,73846436	-80,57572174	-940,4072266	-0,216632
Cloud_Mesh_Distance_BA_R0_R3	22,77826691	-80,63030243	-940,4643555	-0,233493
Cloud_Mesh_Distance_BA_R0_R3	22,87341499	-80,43215179	-940,6056519	-0,220211
Cloud_Mesh_Distance_BA_R0_R3	22,94969177	-80,58330536	-940,7470093	-0,256836
Cloud_Mesh_Distance_BA_R0_R3	16,73142242	-79,7226181	-940,4942627	0,038494
Cloud_Mesh_Distance_BA_R0_R3	22,79548454	-80,52515411	-940,4833984	-0,220969
Cloud_Mesh_Distance_BA_R0_R3	22,77207947	-80,38770294	-940,4571533	-0,209467
Cloud_Mesh_Distance_BA_R0_R3	16,79289246	-79,4504776	-940,4912109	0,035574
Cloud_Mesh_Distance_BA_R0_R3	16,78560257	-79,55145264	-940,4645386	0,034541
Cloud_Mesh_Distance_BA_R0_R3	22,93590927	-80,23232269	-940,7615356	-0,20391
Cloud_Mesh_Distance_BA_R0_R3	22,89739227	-80,18370819	-940,6992188	-0,191373
Cloud_Mesh_Distance_BA_R0_R3	16,72101593	-79,50797272	-940,5777588	0,047067
Cloud_Mesh_Distance_BA_R0_R3	16,83226967	-79,31491852	-940,4917603	0,034907
Cloud_Mesh_Distance_BA_R0_R3	22,71203995	-79,86069489	-940,5349731	-0,134773
Cloud_Mesh_Distance_BA_R0_R3	22,84410667	-80,19086456	-940,6010742	-0,190871
Cloud_Mesh_Distance_BA_R0_R3	22,86743546	-80,08569336	-940,6831665	-0,17544
Cloud_Mesh_Distance_BA_R0_R3	22,93419456	-80,12632751	-940,8023071	-0,188254
Cloud_Mesh_Distance_BA_R0_R3	16,98216248	-79,18878174	-940,3432617	0,01841
Cloud_Mesh_Distance_BA_R0_R3	16,9122467	-79,16495514	-940,4505615	0,024778
Cloud_Mesh_Distance_BA_R0_R3	16,93296814	-79,30884552	-940,3502808	0,023006

Cloud_Mesh_Distance_BA_R0_R3	17,05918503	-79,14203644	-940,2692871	0,013307
Cloud_Mesh_Distance_BA_R0_R3	22,757267	-79,75549316	-940,6744995	-0,122165
Cloud_Mesh_Distance_BA_R0_R3	16,99631882	-79,06188202	-940,3920898	0,019733
Cloud_Mesh_Distance_BA_R0_R3	16,91896439	-78,99045563	-940,5333862	0,024316
Cloud_Mesh_Distance_BA_R0_R3	17,01219177	-78,93302155	-940,4441528	0,017613
Cloud_Mesh_Distance_BA_R0_R3	17,09867096	-78,9565506	-940,3250732	0,01411
Cloud_Mesh_Distance_BA_R0_R3	22,60496902	-79,41214752	-940,6696777	-0,060745
Cloud_Mesh_Distance_BA_R0_R3	22,57182121	-79,4805069	-940,5570679	-0,076497
Cloud_Mesh_Distance_BA_R0_R3	16,98059464	-78,85085297	-940,5314941	0,016865
Cloud_Mesh_Distance_BA_R0_R3	22,49628067	-79,34571838	-940,5501099	-0,055401
Cloud_Mesh_Distance_BA_R0_R3	22,53779221	-79,40163422	-940,5661621	-0,063518
Cloud_Mesh_Distance_BA_R0_R3	22,5324173	-79,31900787	-940,6308594	-0,047493
Cloud_Mesh_Distance_BA_R0_R3	22,62891388	-79,3562851	-940,7715454	-0,052169
Cloud_Mesh_Distance_BA_R0_R3	22,51266861	-79,21326447	-940,7023926	-0,032042
Cloud_Mesh_Distance_BA_R0_R3	17,13727188	-78,63432312	-940,4751587	0,011428
Cloud_Mesh_Distance_BA_R0_R3	17,22691345	-78,54972076	-940,4310303	0,010368
Cloud_Mesh_Distance_BA_R0_R3	17,28210068	-78,42978668	-940,453125	0,012514
Cloud_Mesh_Distance_BA_R0_R3	22,05401421	-78,79393768	-940,4867554	-0,014481
Cloud_Mesh_Distance_BA_R0_R3	22,26935196	-78,90080261	-940,663147	-0,010965
Cloud_Mesh_Distance_BA_R0_R3	17,47105026	-78,23969269	-940,4020996	0,020315
Cloud_Mesh_Distance_BA_R0_R3	22,15889549	-78,79252625	-940,6304321	-0,009106
Cloud_Mesh_Distance_BA_R0_R3	22,0766449	-78,6084671	-940,7446899	-0,000693
Cloud_Mesh_Distance_BA_R0_R3	17,47187424	-78,12136078	-940,4864502	0,026138
Cloud_Mesh_Distance_BA_R0_R3	17,37570763	-78,06537628	-940,6147461	0,025743
Cloud_Mesh_Distance_BA_R0_R3	21,85651588	-78,38307953	-940,7177734	0,003489
Cloud_Mesh_Distance_BA_R0_R3	21,87925339	-78,51230621	-940,5811768	-0,001894
Cloud_Mesh_Distance_BA_R0_R3	22,01255417	-78,64743805	-940,5999756	-0,009484
Cloud_Mesh_Distance_BA_R0_R3	17,48036194	-78,02266693	-940,552002	0,029234
Cloud_Mesh_Distance_BA_R0_R3	21,72293472	-78,26831818	-940,677124	0,010193
Cloud_Mesh_Distance_BA_R0_R3	21,72509575	-78,3587265	-940,5610352	0,005772
Cloud_Mesh_Distance_BA_R0_R3	21,79265976	-78,39195251	-940,6124268	0,00279
Cloud_Mesh_Distance_BA_R0_R3	17,57941055	-77,76650238	-940,6739502	0,044407
Cloud_Mesh_Distance_BA_R0_R3	17,69538879	-77,73605347	-940,6057129	0,053472
Cloud_Mesh_Distance_BA_R0_R3	21,28157616	-77,89154816	-940,6010132	0,021318
Cloud_Mesh_Distance_BA_R0_R3	21,33476257	-78,01707458	-940,4989624	0,025115
Cloud_Mesh_Distance_BA_R0_R3	21,58874321	-78,1251297	-940,6817627	0,014864
Cloud_Mesh_Distance_BA_R0_R3	18,21839523	-77,45995331	-940,5267334	0,07849
Cloud_Mesh_Distance_BA_R0_R3	18,09730339	-77,55673981	-940,4918823	0,077362
Cloud_Mesh_Distance_BA_R0_R3	18,32578278	-77,45439911	-940,4724121	0,082996
Cloud_Mesh_Distance_BA_R0_R3	21,23900795	-77,77953339	-940,7145386	0,022601
Cloud_Mesh_Distance_BA_R0_R3	21,36392212	-77,80277252	-940,8633423	0,016219
Cloud_Mesh_Distance_BA_R0_R3	21,42025757	-77,95560455	-940,6901245	0,019139
Cloud_Mesh_Distance_BA_R0_R3	18,43045425	-77,25566864	-940,6679077	0,073492
Cloud_Mesh_Distance_BA_R0_R3	18,32709885	-77,36345673	-940,5825195	0,077638
Cloud_Mesh_Distance_BA_R0_R3	20,92312622	-77,58113861	-940,6712647	0,009381
Cloud_Mesh_Distance_BA_R0_R3	20,83339691	-77,62749481	-940,5387573	-0,001545
Cloud_Mesh_Distance_BA_R0_R3	21,13039017	-77,77545929	-940,5941773	0,019547
Cloud_Mesh_Distance_BA_R0_R3	21,15024948	-77,62695313	-940,8533325	0,020638
Cloud_Mesh_Distance_BA_R0_R3	21,25843239	-77,71901703	-940,8466797	0,016696
Cloud_Mesh_Distance_BA_R0_R3	18,71685219	-77,15149689	-940,6953735	0,066197

Cloud_Mesh_Distance_BA_R0_R3	18,61691284	-77,22490692	-940,6306763	0,07175
Cloud_Mesh_Distance_BA_R0_R3	18,79987144	-77,22562408	-940,5749512	0,065236
Cloud_Mesh_Distance_BA_R0_R3	18,88738251	-77,29968262	-940,4546509	0,065417
Cloud_Mesh_Distance_BA_R0_R3	18,95945358	-77,2332077	-940,5336914	0,056459
Cloud_Mesh_Distance_BA_R0_R3	19,17853737	-77,1570816	-940,6221924	0,037196
Cloud_Mesh_Distance_BA_R0_R3	19,50346565	-77,23230743	-940,5358887	0,00839
Cloud_Mesh_Distance_BA_R0_R3	19,32992172	-77,22629547	-940,5269165	0,02265
Cloud_Mesh_Distance_BA_R0_R3	19,67907906	-77,28652954	-940,4887085	-0,007151
Cloud_Mesh_Distance_BA_R0_R3	19,87925148	-77,30162811	-940,5167236	-0,017585
Cloud_Mesh_Distance_BA_R0_R3	19,95837593	-77,21877289	-940,6606445	-0,019978
Cloud_Mesh_Distance_BA_R0_R3	19,77895546	-77,21922302	-940,6092529	-0,012137
Cloud_Mesh_Distance_BA_R0_R3	20,0588131	-77,3249588	-940,5420532	-0,025202
Cloud_Mesh_Distance_BA_R0_R3	20,15934753	-77,26586151	-940,663208	-0,019359
Cloud_Mesh_Distance_BA_R0_R3	20,2257843	-77,36067963	-940,5567627	-0,024885
Cloud_Mesh_Distance_BA_R0_R3	20,33995247	-77,31926727	-940,6652222	-0,018312
Cloud_Mesh_Distance_BA_R0_R3	20,53431702	-77,41005707	-940,6386108	-0,014772
Cloud_Mesh_Distance_BA_R0_R3	20,47435379	-77,29076385	-940,7763672	-0,009028
Cloud_Mesh_Distance_BA_R0_R3	20,74940109	-77,50170135	-940,6473999	-0,002823
Cloud_Mesh_Distance_BA_R0_R3	21,00756836	-77,55976105	-940,7884522	0,017901
Cloud_Mesh_Distance_BA_R0_R3	21,076334	-77,66571808	-940,6954346	0,018946
Cloud_Mesh_Distance_BA_R0_R3	19,42528343	-77,16112518	-940,630188	0,013626
Cloud_Mesh_Distance_BA_R0_R3	19,6131897	-77,09207916	-940,7619019	-0,001696
Cloud_Mesh_Distance_BA_R0_R3	19,60589409	-77,17166901	-940,6410523	-0,000799
Cloud_Mesh_Distance_BA_R0_R3	19,780159	-77,12961578	-940,7434082	-0,008615
Cloud_Mesh_Distance_BA_R0_R3	20,27848434	-77,20523834	-940,8035889	-0,01081
Cloud_Mesh_Distance_BA_R0_R3	19,85925293	-77,06654358	-940,8612671	-0,008271
Cloud_Mesh_Distance_BA_R0_R3	18,95245934	-83,65729523	-940,1646729	-0,161939
Cloud_Mesh_Distance_BA_R0_R3	19,12244415	-83,70058441	-940,1528931	-0,161944
Cloud_Mesh_Distance_BA_R0_R3	18,91480255	-83,57720184	-940,081665	-0,165211
Cloud_Mesh_Distance_BA_R0_R3	19,07183838	-83,60041046	-940,0446167	-0,166429
Cloud_Mesh_Distance_BA_R0_R3	19,26456833	-83,6391983	-940,0383301	-0,160061
Cloud_Mesh_Distance_BA_R0_R3	19,46430206	-83,66791534	-940,0457764	-0,15404
Cloud_Mesh_Distance_BA_R0_R3	20,23538589	-83,75876617	-940,2501831	-0,10192
Cloud_Mesh_Distance_BA_R0_R3	20,3247757	-83,70523834	-940,218689	-0,093662
Cloud_Mesh_Distance_BA_R0_R3	18,31652832	-83,25417328	-940,0801392	-0,151129
Cloud_Mesh_Distance_BA_R0_R3	18,47340584	-83,28324127	-939,9918213	-0,164057
Cloud_Mesh_Distance_BA_R0_R3	18,7694931	-83,41175079	-939,9559326	-0,163922
Cloud_Mesh_Distance_BA_R0_R3	18,55980682	-83,41365814	-940,0881958	-0,155736
Cloud_Mesh_Distance_BA_R0_R3	18,82512283	-83,61356354	-940,1729736	-0,158075
Cloud_Mesh_Distance_BA_R0_R3	18,97385406	-83,48104095	-939,9415283	-0,166807
Cloud_Mesh_Distance_BA_R0_R3	18,75372887	-83,52631378	-940,1028442	-0,163179
Cloud_Mesh_Distance_BA_R0_R3	19,20694351	-83,53089142	-939,9263306	-0,162697
Cloud_Mesh_Distance_BA_R0_R3	19,40539932	-83,56478119	-939,9324951	-0,157044
Cloud_Mesh_Distance_BA_R0_R3	19,75211906	-83,59978485	-939,9745483	-0,140179
Cloud_Mesh_Distance_BA_R0_R3	19,59434891	-83,58646393	-939,9500122	-0,147695
Cloud_Mesh_Distance_BA_R0_R3	19,66403961	-83,68039703	-940,0578613	-0,14949
Cloud_Mesh_Distance_BA_R0_R3	19,89303017	-83,55440521	-939,9473877	-0,127839
Cloud_Mesh_Distance_BA_R0_R3	19,87174988	-83,66587067	-940,0622559	-0,134665
Cloud_Mesh_Distance_BA_R0_R3	20,05780792	-83,59055328	-940,0180054	-0,116855
Cloud_Mesh_Distance_BA_R0_R3	20,03437805	-83,68634796	-940,114563	-0,118973

Cloud_Mesh_Distance_BA_R0_R3	20,21681023	-83,55043793	-940,0209351	-0,105329
Cloud_Mesh_Distance_BA_R0_R3	20,22309113	-83,65854645	-940,1342773	-0,106997
Cloud_Mesh_Distance_BA_R0_R3	20,52916145	-83,50727081	-940,0969849	-0,081189
Cloud_Mesh_Distance_BA_R0_R3	20,72150803	-83,64316559	-940,3277588	-0,062724
Cloud_Mesh_Distance_BA_R0_R3	18,08920288	-83,12522125	-940,1339722	-0,141509
Cloud_Mesh_Distance_BA_R0_R3	18,37104034	-83,1901474	-939,9605103	-0,163244
Cloud_Mesh_Distance_BA_R0_R3	18,6613121	-83,31687164	-939,9086304	-0,163108
Cloud_Mesh_Distance_BA_R0_R3	20,02285004	-83,49919891	-939,9194336	-0,112738
Cloud_Mesh_Distance_BA_R0_R3	20,55286026	-83,41902924	-940,0281982	-0,079972
Cloud_Mesh_Distance_BA_R0_R3	20,66889572	-83,4285202	-940,0913696	-0,074305
Cloud_Mesh_Distance_BA_R0_R3	20,67510605	-83,53452301	-940,1928711	-0,072423
Cloud_Mesh_Distance_BA_R0_R3	20,84161377	-83,42758942	-940,1848755	-0,057699
Cloud_Mesh_Distance_BA_R0_R3	17,99173737	-83,01786041	-940,1033936	-0,13484
Cloud_Mesh_Distance_BA_R0_R3	17,91220665	-82,96834564	-940,1326904	-0,12974
Cloud_Mesh_Distance_BA_R0_R3	18,08481026	-83,0018692	-939,9923096	-0,150411
Cloud_Mesh_Distance_BA_R0_R3	18,24479294	-83,17740631	-940,0496216	-0,15331
Cloud_Mesh_Distance_BA_R0_R3	18,26500511	-83,07538605	-939,9171753	-0,157397
Cloud_Mesh_Distance_BA_R0_R3	21,00727844	-83,38126373	-940,2451172	-0,046413
Cloud_Mesh_Distance_BA_R0_R3	21,14543343	-83,28598785	-940,2572022	-0,043972
Cloud_Mesh_Distance_BA_R0_R3	20,97121048	-83,2882309	-940,1446533	-0,053704
Cloud_Mesh_Distance_BA_R0_R3	17,9716568	-82,94722748	-940,043335	-0,140246
Cloud_Mesh_Distance_BA_R0_R3	21,13787651	-83,11156464	-940,1176758	-0,052867
Cloud_Mesh_Distance_BA_R0_R3	21,28879929	-83,01728058	-940,1549683	-0,0502
Cloud_Mesh_Distance_BA_R0_R3	17,62664795	-82,65058136	-940,1300659	-0,115617
Cloud_Mesh_Distance_BA_R0_R3	21,42398834	-82,85958099	-940,145752	-0,053433
Cloud_Mesh_Distance_BA_R0_R3	21,45810509	-82,97863007	-940,2520142	-0,043452
Cloud_Mesh_Distance_BA_R0_R3	21,26173782	-82,88446808	-940,0463257	-0,051309
Cloud_Mesh_Distance_BA_R0_R3	21,60818863	-82,94391632	-940,3416748	-0,038042
Cloud_Mesh_Distance_BA_R0_R3	17,45545578	-82,43192291	-940,1536255	-0,109002
Cloud_Mesh_Distance_BA_R0_R3	17,45947456	-82,2997818	-940,0273438	-0,119365
Cloud_Mesh_Distance_BA_R0_R3	17,5446167	-82,32427216	-939,9332886	-0,128166
Cloud_Mesh_Distance_BA_R0_R3	17,52671051	-82,40096283	-940,0217896	-0,121122
Cloud_Mesh_Distance_BA_R0_R3	21,38907433	-82,73639679	-940,0406494	-0,051278
Cloud_Mesh_Distance_BA_R0_R3	21,57675934	-82,83640289	-940,2459106	-0,046797
Cloud_Mesh_Distance_BA_R0_R3	17,28218269	-82,02268219	-940,0734253	-0,100078
Cloud_Mesh_Distance_BA_R0_R3	17,35388184	-82,2960434	-940,182312	-0,101366
Cloud_Mesh_Distance_BA_R0_R3	21,56846619	-82,68456268	-940,1414795	-0,052659
Cloud_Mesh_Distance_BA_R0_R3	21,71235657	-82,70752716	-940,2704468	-0,050162
Cloud_Mesh_Distance_BA_R0_R3	17,20507813	-82,05928802	-940,21698	-0,088268
Cloud_Mesh_Distance_BA_R0_R3	21,77202225	-82,51737213	-940,2006226	-0,062466
Cloud_Mesh_Distance_BA_R0_R3	21,91486931	-82,49449921	-940,3096314	-0,060399
Cloud_Mesh_Distance_BA_R0_R3	21,94369125	-82,38257599	-940,269043	-0,068008
Cloud_Mesh_Distance_BA_R0_R3	22,01060104	-82,42853546	-940,3569946	-0,060696
Cloud_Mesh_Distance_BA_R0_R3	17,14809227	-81,75292206	-940,1026611	-0,093688
Cloud_Mesh_Distance_BA_R0_R3	17,30656052	-81,83112335	-939,9221191	-0,11792
Cloud_Mesh_Distance_BA_R0_R3	17,28254318	-81,92790985	-940,0115967	-0,106561
Cloud_Mesh_Distance_BA_R0_R3	21,91827393	-82,20694733	-940,1393433	-0,07135
Cloud_Mesh_Distance_BA_R0_R3	21,86084938	-82,3249588	-940,1593628	-0,069008
Cloud_Mesh_Distance_BA_R0_R3	22,02539063	-82,21381378	-940,2426148	-0,077643
Cloud_Mesh_Distance_BA_R0_R3	22,07701492	-82,31742096	-940,3547974	-0,067755

Cloud_Mesh_Distance_BA_R0_R3	17,00857544	-81,4986496	-940,1843262	-0,067164
Cloud_Mesh_Distance_BA_R0_R3	17,14570236	-81,67197418	-940,064209	-0,097885
Cloud_Mesh_Distance_BA_R0_R3	21,98220825	-82,14220428	-940,1591187	-0,07392
Cloud_Mesh_Distance_BA_R0_R3	22,22639275	-81,94181061	-940,2834473	-0,090249
Cloud_Mesh_Distance_BA_R0_R3	22,21133995	-82,08994293	-940,3567505	-0,08527
Cloud_Mesh_Distance_BA_R0_R3	16,99520874	-81,19616699	-940,0965576	-0,079845
Cloud_Mesh_Distance_BA_R0_R3	16,93880081	-81,28096771	-940,210144	-0,058975
Cloud_Mesh_Distance_BA_R0_R3	17,00582123	-81,3549881	-940,1315918	-0,072938
Cloud_Mesh_Distance_BA_R0_R3	22,36353683	-81,7334671	-940,3185425	-0,108611
Cloud_Mesh_Distance_BA_R0_R3	22,26798439	-81,81053925	-940,2528076	-0,096172
Cloud_Mesh_Distance_BA_R0_R3	22,28245544	-81,97011566	-940,3630371	-0,09757
Cloud_Mesh_Distance_BA_R0_R3	22,47154045	-81,71129608	-940,4381104	-0,118904
Cloud_Mesh_Distance_BA_R0_R3	16,99492645	-81,0592041	-940,062561	-0,0834
Cloud_Mesh_Distance_BA_R0_R3	16,97300339	-80,89530945	-940,0635986	-0,077414
Cloud_Mesh_Distance_BA_R0_R3	16,90752411	-81,09793854	-940,2089233	-0,059541
Cloud_Mesh_Distance_BA_R0_R3	17,05550003	-80,98442841	-939,9585571	-0,096418
Cloud_Mesh_Distance_BA_R0_R3	22,34892654	-81,51837921	-940,1958618	-0,117702
Cloud_Mesh_Distance_BA_R0_R3	22,47099876	-81,4467392	-940,303772	-0,143575
Cloud_Mesh_Distance_BA_R0_R3	22,41537094	-81,60564423	-940,3118897	-0,122218
Cloud_Mesh_Distance_BA_R0_R3	16,90843964	-80,70317078	-940,1394653	-0,058835
Cloud_Mesh_Distance_BA_R0_R3	22,59659576	-81,39600372	-940,4398193	-0,166959
Cloud_Mesh_Distance_BA_R0_R3	22,52830505	-81,26473999	-940,2996216	-0,16307
Cloud_Mesh_Distance_BA_R0_R3	16,88908768	-80,36467743	-940,1552734	-0,034851
Cloud_Mesh_Distance_BA_R0_R3	16,87940788	-80,52759552	-940,1738892	-0,045667
Cloud_Mesh_Distance_BA_R0_R3	16,97598267	-80,47592926	-940,0183106	-0,056393
Cloud_Mesh_Distance_BA_R0_R3	22,63486099	-80,71055603	-940,2927856	-0,205741
Cloud_Mesh_Distance_BA_R0_R3	22,62237549	-80,86585999	-940,3006592	-0,202126
Cloud_Mesh_Distance_BA_R0_R3	22,59675026	-81,05963898	-940,3167114	-0,18857
Cloud_Mesh_Distance_BA_R0_R3	22,69671631	-80,7864151	-940,3773193	-0,220093
Cloud_Mesh_Distance_BA_R0_R3	16,89850044	-80,17716217	-940,1487427	-0,02126
Cloud_Mesh_Distance_BA_R0_R3	16,90913582	-79,98169708	-940,1575928	-0,007741
Cloud_Mesh_Distance_BA_R0_R3	22,71593475	-80,66894531	-940,3857422	-0,218592
Cloud_Mesh_Distance_BA_R0_R3	16,93146896	-79,79484558	-940,1652832	0,004024
Cloud_Mesh_Distance_BA_R0_R3	16,98841095	-79,89762115	-940,0552979	-0,013031
Cloud_Mesh_Distance_BA_R0_R3	22,70538712	-80,27033234	-940,3864746	-0,185549
Cloud_Mesh_Distance_BA_R0_R3	22,67980957	-80,41156769	-940,3377686	-0,192809
Cloud_Mesh_Distance_BA_R0_R3	16,87850952	-79,68740082	-940,2774658	0,017184
Cloud_Mesh_Distance_BA_R0_R3	16,96252823	-79,61318207	-940,178772	0,008651
Cloud_Mesh_Distance_BA_R0_R3	17,08723068	-79,54160309	-940,036438	0,000353
Cloud_Mesh_Distance_BA_R0_R3	16,99324036	-79,42022705	-940,2148438	0,014652
Cloud_Mesh_Distance_BA_R0_R3	22,7027626	-80,12175751	-940,4171143	-0,168996
Cloud_Mesh_Distance_BA_R0_R3	22,6210041	-80,17108917	-940,3056641	-0,166629
Cloud_Mesh_Distance_BA_R0_R3	22,77046776	-80,29019165	-940,4674683	-0,19534
Cloud_Mesh_Distance_BA_R0_R3	22,7752018	-80,20934296	-940,4911499	-0,18424
Cloud_Mesh_Distance_BA_R0_R3	22,82425117	-80,29737091	-940,5440064	-0,198584
Cloud_Mesh_Distance_BA_R0_R3	17,01358223	-79,27011108	-940,2599487	0,016468
Cloud_Mesh_Distance_BA_R0_R3	16,87086105	-79,5016098	-940,3557739	0,026709
Cloud_Mesh_Distance_BA_R0_R3	17,10756683	-79,29464722	-940,1278076	0,006492
Cloud_Mesh_Distance_BA_R0_R3	22,638134	-79,88034821	-940,4199219	-0,13353
Cloud_Mesh_Distance_BA_R0_R3	22,7109642	-79,98474884	-940,4763184	-0,150007

Cloud_Mesh_Distance_BA_R0_R3	22,78839493	-80,08965302	-940,5477905	-0,171686
Cloud_Mesh_Distance_BA_R0_R3	22,36232376	-79,54509735	-940,2401123	-0,07813
Cloud_Mesh_Distance_BA_R0_R3	22,62965965	-79,72786713	-940,4837647	-0,116141
Cloud_Mesh_Distance_BA_R0_R3	22,50097466	-79,63074493	-940,3622437	-0,096558
Cloud_Mesh_Distance_BA_R0_R3	22,52624512	-79,81368256	-940,307251	-0,123131
Cloud_Mesh_Distance_BA_R0_R3	22,42544365	-79,697258	-940,2380981	-0,10159
Cloud_Mesh_Distance_BA_R0_R3	22,57014275	-79,57016754	-940,4920654	-0,088544
Cloud_Mesh_Distance_BA_R0_R3	22,46347618	-79,42365265	-940,44104	-0,064206
Cloud_Mesh_Distance_BA_R0_R3	22,6618309	-79,57105255	-940,6383057	-0,090989
Cloud_Mesh_Distance_BA_R0_R3	17,26285934	-78,68387604	-940,3083496	0,005549
Cloud_Mesh_Distance_BA_R0_R3	22,31045151	-79,22650909	-940,3917847	-0,042179
Cloud_Mesh_Distance_BA_R0_R3	22,43513298	-79,2165451	-940,5731201	-0,034586
Cloud_Mesh_Distance_BA_R0_R3	17,1808033	-78,77071381	-940,3424683	0,006978
Cloud_Mesh_Distance_BA_R0_R3	17,22276878	-78,63944244	-940,3779297	0,007315
Cloud_Mesh_Distance_BA_R0_R3	17,3091774	-78,55864716	-940,3416138	0,007201
Cloud_Mesh_Distance_BA_R0_R3	17,34157562	-78,67656708	-940,2346191	0,001023
Cloud_Mesh_Distance_BA_R0_R3	22,31559753	-79,09990692	-940,5126343	-0,026362
Cloud_Mesh_Distance_BA_R0_R3	17,49714279	-78,43032074	-940,2496948	0,00605
Cloud_Mesh_Distance_BA_R0_R3	17,41235161	-78,39043427	-940,3535156	0,009516
Cloud_Mesh_Distance_BA_R0_R3	17,42246246	-78,54235077	-940,2437134	0,001577
Cloud_Mesh_Distance_BA_R0_R3	22,20928574	-79,05137634	-940,4202881	-0,026742
Cloud_Mesh_Distance_BA_R0_R3	22,1522541	-78,91002655	-940,4889526	-0,018006
Cloud_Mesh_Distance_BA_R0_R3	22,24694633	-78,96553802	-940,5567627	-0,020617
Cloud_Mesh_Distance_BA_R0_R3	22,34590149	-79,02610016	-940,6344605	-0,01717
Cloud_Mesh_Distance_BA_R0_R3	17,5575676	-78,29060364	-940,2907105	0,016444
Cloud_Mesh_Distance_BA_R0_R3	17,66555786	-78,29528046	-940,1939087	0,016209
Cloud_Mesh_Distance_BA_R0_R3	21,81238174	-78,54607391	-940,4555664	-0,000197
Cloud_Mesh_Distance_BA_R0_R3	21,84050751	-78,65709686	-940,3719482	-0,004088
Cloud_Mesh_Distance_BA_R0_R3	21,92826843	-78,67177582	-940,4615479	-0,007291
Cloud_Mesh_Distance_BA_R0_R3	21,90302849	-78,7450943	-940,355835	-0,008275
Cloud_Mesh_Distance_BA_R0_R3	17,56113434	-78,15496826	-940,3829956	0,026442
Cloud_Mesh_Distance_BA_R0_R3	17,71580124	-78,12384796	-940,2733154	0,031251
Cloud_Mesh_Distance_BA_R0_R3	17,90576935	-77,96979523	-940,2366333	0,05396
Cloud_Mesh_Distance_BA_R0_R3	21,69868851	-78,42546082	-940,4505005	0,008061
Cloud_Mesh_Distance_BA_R0_R3	21,65196037	-78,46927643	-940,3508911	0,011337
Cloud_Mesh_Distance_BA_R0_R3	17,58994675	-78,00965881	-940,4659424	0,036261
Cloud_Mesh_Distance_BA_R0_R3	17,77072144	-77,91388702	-940,3914185	0,050393
Cloud_Mesh_Distance_BA_R0_R3	17,65147018	-77,84700775	-940,545105	0,051268
Cloud_Mesh_Distance_BA_R0_R3	21,41312408	-78,16327667	-940,4143677	0,021418
Cloud_Mesh_Distance_BA_R0_R3	21,54385376	-78,32923889	-940,3756104	0,019091
Cloud_Mesh_Distance_BA_R0_R3	21,63004684	-78,27121735	-940,5436401	0,011771
Cloud_Mesh_Distance_BA_R0_R3	21,78149033	-78,44571686	-940,5307617	0,003184
Cloud_Mesh_Distance_BA_R0_R3	17,79817963	-77,76691437	-940,496521	0,058349
Cloud_Mesh_Distance_BA_R0_R3	17,90674973	-77,69107056	-940,4853516	0,067054
Cloud_Mesh_Distance_BA_R0_R3	17,97322464	-77,78779602	-940,3427734	0,070688
Cloud_Mesh_Distance_BA_R0_R3	18,16867065	-77,78105927	-940,2064819	0,082451
Cloud_Mesh_Distance_BA_R0_R3	18,2977829	-77,6320343	-940,2788696	0,089341
Cloud_Mesh_Distance_BA_R0_R3	21,19303131	-77,88774872	-940,5119629	0,021015
Cloud_Mesh_Distance_BA_R0_R3	21,49113464	-78,1397934	-940,5306397	0,02263
Cloud_Mesh_Distance_BA_R0_R3	17,95023346	-77,61035919	-940,5346069	0,070944

Cloud_Mesh_Distance_BA_R0_R3	18,01793289	-77,65945435	-940,4362183	0,074466
Cloud_Mesh_Distance_BA_R0_R3	18,16653824	-77,66255951	-940,329895	0,081698
Cloud_Mesh_Distance_BA_R0_R3	18,59027672	-77,45367432	-940,3427734	0,087066
Cloud_Mesh_Distance_BA_R0_R3	18,31148911	-77,54653168	-940,3690186	0,088013
Cloud_Mesh_Distance_BA_R0_R3	20,65106201	-77,69721222	-940,3461304	-0,016704
Cloud_Mesh_Distance_BA_R0_R3	20,77126503	-77,69545746	-940,4168091	-0,006646
Cloud_Mesh_Distance_BA_R0_R3	20,84284592	-77,79859924	-940,3470459	-0,00469
Cloud_Mesh_Distance_BA_R0_R3	21,05715942	-77,80850983	-940,4856567	0,013358
Cloud_Mesh_Distance_BA_R0_R3	21,21201897	-77,83205414	-940,6037598	0,020289
Cloud_Mesh_Distance_BA_R0_R3	18,44229889	-77,4522934	-940,4140015	0,082222
Cloud_Mesh_Distance_BA_R0_R3	18,55458069	-77,35316467	-940,4888916	0,078757
Cloud_Mesh_Distance_BA_R0_R3	18,72078323	-77,41718292	-940,3421021	0,077951
Cloud_Mesh_Distance_BA_R0_R3	19,02757263	-77,32570648	-940,3963013	0,053712
Cloud_Mesh_Distance_BA_R0_R3	18,90396881	-77,39478302	-940,3225098	0,069655
Cloud_Mesh_Distance_BA_R0_R3	18,74777412	-77,31845856	-940,4650269	0,069671
Cloud_Mesh_Distance_BA_R0_R3	19,11643982	-77,4320755	-940,2405396	0,053931
Cloud_Mesh_Distance_BA_R0_R3	19,16445541	-77,34496307	-940,3571167	0,044773
Cloud_Mesh_Distance_BA_R0_R3	19,82369804	-77,38037109	-940,3903198	-0,015585
Cloud_Mesh_Distance_BA_R0_R3	19,79751968	-77,43993378	-940,3032837	-0,013393
Cloud_Mesh_Distance_BA_R0_R3	19,985569	-77,39630127	-940,4188232	-0,024151
Cloud_Mesh_Distance_BA_R0_R3	20,36312675	-77,40280151	-940,5596924	-0,024581
Cloud_Mesh_Distance_BA_R0_R3	20,48975182	-77,6186142	-940,3514404	-0,025099
Cloud_Mesh_Distance_BA_R0_R3	20,43074036	-77,49524689	-940,4723511	-0,023402
Cloud_Mesh_Distance_BA_R0_R3	20,63809204	-77,57249451	-940,4846191	-0,015388
Cloud_Mesh_Distance_BA_R0_R3	20,99168777	-77,69670868	-940,5708618	0,011961
Cloud_Mesh_Distance_BA_R0_R3	20,91586113	-77,74427032	-940,4545898	0,003419
Cloud_Mesh_Distance_BA_R0_R3	19,29314232	-77,32357788	-940,3869019	0,031758
Cloud_Mesh_Distance_BA_R0_R3	19,13914108	-77,25267792	-940,4881592	0,042958
Cloud_Mesh_Distance_BA_R0_R3	19,49101257	-77,3270874	-940,3983765	0,010937
Cloud_Mesh_Distance_BA_R0_R3	19,68520927	-77,39500427	-940,336853	-0,004508
Cloud_Mesh_Distance_BA_R0_R3	20,1515274	-77,42333984	-940,4428101	-0,028023
Cloud_Mesh_Distance_BA_R0_R3	20,28775215	-77,44346619	-940,4727783	-0,028607
Cloud_Mesh_Distance_BA_R0_R3	19,73587799	-83,50199127	-939,8725586	-0,132085
Cloud_Mesh_Distance_BA_R0_R3	19,91541672	-83,45359039	-939,8540039	-0,119287
Cloud_Mesh_Distance_BA_R0_R3	20,36392593	-83,54651642	-940,0670166	-0,091962
Cloud_Mesh_Distance_BA_R0_R3	18,55823517	-83,18518829	-939,8300781	-0,162847
Cloud_Mesh_Distance_BA_R0_R3	18,56885719	-83,24359894	-939,883728	-0,162508
Cloud_Mesh_Distance_BA_R0_R3	18,88832092	-83,36873627	-939,8522339	-0,159822
Cloud_Mesh_Distance_BA_R0_R3	18,78420258	-83,3449173	-939,8740234	-0,161633
Cloud_Mesh_Distance_BA_R0_R3	19,79682922	-83,39493561	-939,7810669	-0,120835
Cloud_Mesh_Distance_BA_R0_R3	20,29801559	-83,44589996	-939,9505005	-0,09333
Cloud_Mesh_Distance_BA_R0_R3	20,1405468	-83,49753571	-939,9481812	-0,105591
Cloud_Mesh_Distance_BA_R0_R3	20,4462471	-83,45558929	-940,0151367	-0,085203
Cloud_Mesh_Distance_BA_R0_R3	20,48764038	-83,34725189	-939,9393921	-0,085504
Cloud_Mesh_Distance_BA_R0_R3	18,29233551	-82,96872711	-939,7912598	-0,166492
Cloud_Mesh_Distance_BA_R0_R3	18,43647003	-83,02002716	-939,744751	-0,166833
Cloud_Mesh_Distance_BA_R0_R3	18,45409393	-83,13425446	-939,8430176	-0,16263
Cloud_Mesh_Distance_BA_R0_R3	18,48489189	-83,20778656	-939,8991699	-0,162541
Cloud_Mesh_Distance_BA_R0_R3	20,0031395	-83,27625275	-939,7210083	-0,100248
Cloud_Mesh_Distance_BA_R0_R3	19,93541336	-83,36669159	-939,7801514	-0,113891

Cloud_Mesh_Distance_BA_R0_R3	20,10535431	-83,38864899	-939,8404541	-0,103977
Cloud_Mesh_Distance_BA_R0_R3	20,32078934	-83,31012726	-939,843811	-0,088885
Cloud_Mesh_Distance_BA_R0_R3	20,63484573	-83,34671783	-940,0054321	-0,073862
Cloud_Mesh_Distance_BA_R0_R3	20,68212509	-83,20598602	-939,920166	-0,065452
Cloud_Mesh_Distance_BA_R0_R3	20,47307968	-83,22156525	-939,8347168	-0,074119
Cloud_Mesh_Distance_BA_R0_R3	20,78277206	-83,31848907	-940,0587158	-0,067856
Cloud_Mesh_Distance_BA_R0_R3	21,10025024	-83,20774078	-940,1640015	-0,048236
Cloud_Mesh_Distance_BA_R0_R3	17,7579422	-82,70490265	-940,019165	-0,12867
Cloud_Mesh_Distance_BA_R0_R3	17,95204163	-82,64632416	-939,769165	-0,152753
Cloud_Mesh_Distance_BA_R0_R3	17,82132149	-82,6011734	-939,852478	-0,143907
Cloud_Mesh_Distance_BA_R0_R3	17,91083336	-82,74170685	-939,8914185	-0,144522
Cloud_Mesh_Distance_BA_R0_R3	18,08078766	-82,73313141	-939,7369385	-0,160622
Cloud_Mesh_Distance_BA_R0_R3	18,17844009	-82,95861053	-939,866272	-0,158295
Cloud_Mesh_Distance_BA_R0_R3	18,03761673	-82,87680817	-939,9041138	-0,152403
Cloud_Mesh_Distance_BA_R0_R3	20,55536652	-83,08022308	-939,7700806	-0,064947
Cloud_Mesh_Distance_BA_R0_R3	20,73123741	-83,05510712	-939,8395996	-0,06245
Cloud_Mesh_Distance_BA_R0_R3	20,89681244	-83,19095612	-940,0249023	-0,05879
Cloud_Mesh_Distance_BA_R0_R3	21,03030777	-83,08072662	-940,0265503	-0,055523
Cloud_Mesh_Distance_BA_R0_R3	20,88406754	-83,05036163	-939,9188843	-0,058269
Cloud_Mesh_Distance_BA_R0_R3	21,02691841	-83,17896271	-940,0952148	-0,054796
Cloud_Mesh_Distance_BA_R0_R3	17,62742996	-82,42292023	-939,9119263	-0,129563
Cloud_Mesh_Distance_BA_R0_R3	17,68380737	-82,56720734	-939,9723511	-0,134041
Cloud_Mesh_Distance_BA_R0_R3	20,82660675	-82,95253754	-939,8244019	-0,056372
Cloud_Mesh_Distance_BA_R0_R3	21,15891266	-83,00719452	-940,0586548	-0,052986
Cloud_Mesh_Distance_BA_R0_R3	17,75584793	-82,3607254	-939,7252808	-0,145766
Cloud_Mesh_Distance_BA_R0_R3	17,73759079	-82,49100494	-939,8442383	-0,137424
Cloud_Mesh_Distance_BA_R0_R3	17,89945221	-82,43442535	-939,6486816	-0,149365
Cloud_Mesh_Distance_BA_R0_R3	17,8229084	-82,48940277	-939,7574463	-0,147862
Cloud_Mesh_Distance_BA_R0_R3	20,93964386	-82,91667938	-939,8654785	-0,055415
Cloud_Mesh_Distance_BA_R0_R3	21,07555389	-82,92525482	-939,9509277	-0,053432
Cloud_Mesh_Distance_BA_R0_R3	21,36279297	-82,64667511	-939,9671631	-0,050616
Cloud_Mesh_Distance_BA_R0_R3	17,38917923	-82,12030792	-939,9883423	-0,113909
Cloud_Mesh_Distance_BA_R0_R3	17,61611176	-82,27806854	-939,8121948	-0,134635
Cloud_Mesh_Distance_BA_R0_R3	17,51120758	-82,20821381	-939,8880005	-0,128971
Cloud_Mesh_Distance_BA_R0_R3	17,58795547	-82,13663483	-939,7480469	-0,136243
Cloud_Mesh_Distance_BA_R0_R3	21,57398987	-82,47383881	-940,015686	-0,056818
Cloud_Mesh_Distance_BA_R0_R3	21,61888123	-82,53177643	-940,0859375	-0,060561
Cloud_Mesh_Distance_BA_R0_R3	21,4852047	-82,58692169	-940,0185547	-0,056599
Cloud_Mesh_Distance_BA_R0_R3	21,48620987	-82,46962738	-939,949707	-0,048951
Cloud_Mesh_Distance_BA_R0_R3	21,40016937	-82,55712128	-939,9407349	-0,050076
Cloud_Mesh_Distance_BA_R0_R3	21,69790268	-82,39167023	-940,0631714	-0,060953
Cloud_Mesh_Distance_BA_R0_R3	17,49374771	-82,07303619	-939,8187256	-0,132207
Cloud_Mesh_Distance_BA_R0_R3	21,58722687	-82,38744354	-939,9747315	-0,053447
Cloud_Mesh_Distance_BA_R0_R3	21,80864334	-82,23709869	-940,0614624	-0,062881
Cloud_Mesh_Distance_BA_R0_R3	17,37853622	-81,74221039	-939,7838745	-0,131276
Cloud_Mesh_Distance_BA_R0_R3	17,30751801	-81,67516327	-939,8440552	-0,12754
Cloud_Mesh_Distance_BA_R0_R3	17,33664322	-81,96373749	-939,9576416	-0,112597
Cloud_Mesh_Distance_BA_R0_R3	17,43087006	-81,9094162	-939,8021851	-0,134636
Cloud_Mesh_Distance_BA_R0_R3	17,07884979	-81,455513	-940,0620117	-0,091505
Cloud_Mesh_Distance_BA_R0_R3	17,26488495	-81,51255035	-939,8327026	-0,128351

Cloud_Mesh_Distance_BA_R0_R3	17,15474701	-81,56545258	-940,0010376	-0,105098
Cloud_Mesh_Distance_BA_R0_R3	17,21713066	-81,70119476	-939,9777222	-0,111117
Cloud_Mesh_Distance_BA_R0_R3	21,9401741	-81,98668671	-940,0303345	-0,062247
Cloud_Mesh_Distance_BA_R0_R3	22,06966209	-82,03153229	-940,1762085	-0,075691
Cloud_Mesh_Distance_BA_R0_R3	21,91844368	-82,11952972	-940,0872192	-0,06901
Cloud_Mesh_Distance_BA_R0_R3	17,17054176	-81,38111115	-939,9083252	-0,115837
Cloud_Mesh_Distance_BA_R0_R3	17,17559052	-81,22129059	-939,8535767	-0,121323
Cloud_Mesh_Distance_BA_R0_R3	17,08436394	-81,25907135	-939,9857178	-0,103785
Cloud_Mesh_Distance_BA_R0_R3	22,07318687	-81,87186432	-940,0883179	-0,070627
Cloud_Mesh_Distance_BA_R0_R3	22,09422112	-81,733284	-940,0367432	-0,071539
Cloud_Mesh_Distance_BA_R0_R3	22,18835831	-81,76378632	-940,1436768	-0,08455
Cloud_Mesh_Distance_BA_R0_R3	22,16676331	-81,88401031	-940,1871338	-0,080176
Cloud_Mesh_Distance_BA_R0_R3	17,22014236	-81,11629486	-939,7717285	-0,13034
Cloud_Mesh_Distance_BA_R0_R3	17,13063049	-81,08138275	-939,8771362	-0,1104
Cloud_Mesh_Distance_BA_R0_R3	17,0508728	-81,11665344	-939,9936523	-0,095272
Cloud_Mesh_Distance_BA_R0_R3	22,30223656	-81,67359161	-940,2172852	-0,105341
Cloud_Mesh_Distance_BA_R0_R3	22,21113968	-81,60660553	-940,0909424	-0,087785
Cloud_Mesh_Distance_BA_R0_R3	17,06815338	-80,82659149	-939,9137573	-0,095859
Cloud_Mesh_Distance_BA_R0_R3	22,27282333	-81,42969513	-940,081604	-0,103979
Cloud_Mesh_Distance_BA_R0_R3	17,09196091	-80,68849945	-939,8652344	-0,092234
Cloud_Mesh_Distance_BA_R0_R3	17,01211548	-80,69112396	-939,9771729	-0,076831
Cloud_Mesh_Distance_BA_R0_R3	22,23939133	-81,236763	-939,9916382	-0,10851
Cloud_Mesh_Distance_BA_R0_R3	22,30821609	-81,26779938	-940,0643311	-0,121837
Cloud_Mesh_Distance_BA_R0_R3	22,4332962	-81,13431549	-940,1540527	-0,153774
Cloud_Mesh_Distance_BA_R0_R3	22,39812851	-81,34410858	-940,1812134	-0,132787
Cloud_Mesh_Distance_BA_R0_R3	17,0709343	-80,55190277	-939,8840332	-0,080848
Cloud_Mesh_Distance_BA_R0_R3	22,28327942	-81,12047577	-940,0044556	-0,122895
Cloud_Mesh_Distance_BA_R0_R3	22,37273026	-80,95441437	-940,0560303	-0,146417
Cloud_Mesh_Distance_BA_R0_R3	17,06703949	-80,41480255	-939,8861084	-0,067138
Cloud_Mesh_Distance_BA_R0_R3	17,11304855	-80,32746124	-939,8256226	-0,063258
Cloud_Mesh_Distance_BA_R0_R3	17,13639832	-80,44194794	-939,7946777	-0,075509
Cloud_Mesh_Distance_BA_R0_R3	22,5063858	-80,92484283	-940,1833496	-0,175931
Cloud_Mesh_Distance_BA_R0_R3	22,55120087	-80,77339935	-940,2077637	-0,187197
Cloud_Mesh_Distance_BA_R0_R3	17,00447655	-80,08332062	-939,9971314	-0,030511
Cloud_Mesh_Distance_BA_R0_R3	16,96076965	-80,2752533	-940,0441284	-0,039803
Cloud_Mesh_Distance_BA_R0_R3	17,08396149	-80,14173889	-939,8775635	-0,0422
Cloud_Mesh_Distance_BA_R0_R3	22,54339027	-80,60475922	-940,1871338	-0,183216
Cloud_Mesh_Distance_BA_R0_R3	17,14796257	-79,95911407	-939,8234863	-0,033096
Cloud_Mesh_Distance_BA_R0_R3	17,07812691	-79,89347839	-939,9295654	-0,019973
Cloud_Mesh_Distance_BA_R0_R3	17,09415436	-80,03329468	-939,8795166	-0,034079
Cloud_Mesh_Distance_BA_R0_R3	22,42689133	-80,58106232	-940,0724487	-0,158548
Cloud_Mesh_Distance_BA_R0_R3	22,33354568	-80,49210358	-939,9891357	-0,143562
Cloud_Mesh_Distance_BA_R0_R3	22,47161674	-80,39214325	-940,1206055	-0,164375
Cloud_Mesh_Distance_BA_R0_R3	22,66499519	-80,56002045	-940,3167725	-0,203439
Cloud_Mesh_Distance_BA_R0_R3	17,02666092	-79,74208832	-940,0427856	-0,004813
Cloud_Mesh_Distance_BA_R0_R3	22,5966568	-80,30771637	-940,255249	-0,180804
Cloud_Mesh_Distance_BA_R0_R3	22,49187469	-80,15166473	-940,1706543	-0,153185
Cloud_Mesh_Distance_BA_R0_R3	22,59354401	-80,45495605	-940,239502	-0,188234
Cloud_Mesh_Distance_BA_R0_R3	17,20728302	-79,40836334	-939,9508667	-0,006447
Cloud_Mesh_Distance_BA_R0_R3	22,46236801	-79,94664764	-940,1894531	-0,134255

Cloud_Mesh_Distance_BA_R0_R3	22,25726318	-79,77384949	-940,0372315	-0,092868
Cloud_Mesh_Distance_BA_R0_R3	22,40435028	-79,80615997	-940,1728516	-0,115644
Cloud_Mesh_Distance_BA_R0_R3	22,32770157	-79,80168915	-940,0971069	-0,104876
Cloud_Mesh_Distance_BA_R0_R3	22,59701538	-80,00913239	-940,3206787	-0,155167
Cloud_Mesh_Distance_BA_R0_R3	17,16800308	-79,0827713	-940,1712036	0,003257
Cloud_Mesh_Distance_BA_R0_R3	17,25367355	-79,20375824	-940,0077515	-0,007069
Cloud_Mesh_Distance_BA_R0_R3	17,31082916	-79,33647919	-939,8754883	-0,016659
Cloud_Mesh_Distance_BA_R0_R3	17,37472534	-79,0825119	-939,9536133	-0,017405
Cloud_Mesh_Distance_BA_R0_R3	22,24554825	-79,67136383	-940,0617065	-0,079416
Cloud_Mesh_Distance_BA_R0_R3	22,3235321	-79,69282532	-940,1308594	-0,092964
Cloud_Mesh_Distance_BA_R0_R3	17,21964645	-78,90076447	-940,2210083	0,000868
Cloud_Mesh_Distance_BA_R0_R3	17,29043007	-78,99878693	-940,0874634	-0,006016
Cloud_Mesh_Distance_BA_R0_R3	17,41909218	-78,89597321	-940,0236816	-0,014784
Cloud_Mesh_Distance_BA_R0_R3	22,31779099	-79,37848663	-940,2869263	-0,056237
Cloud_Mesh_Distance_BA_R0_R3	17,26542664	-78,77152252	-940,2514038	0,003964
Cloud_Mesh_Distance_BA_R0_R3	17,33112144	-78,82118988	-940,1539307	-0,003342
Cloud_Mesh_Distance_BA_R0_R3	22,0223465	-79,20713043	-940,095459	-0,023742
Cloud_Mesh_Distance_BA_R0_R3	22,07999039	-79,09024811	-940,2385864	-0,02443
Cloud_Mesh_Distance_BA_R0_R3	22,18842506	-79,26258087	-940,2223511	-0,038406
Cloud_Mesh_Distance_BA_R0_R3	22,19091797	-79,14105988	-940,3189697	-0,029934
Cloud_Mesh_Distance_BA_R0_R3	22,23480988	-79,42915344	-940,1644898	-0,052433
Cloud_Mesh_Distance_BA_R0_R3	17,51183701	-78,51843262	-940,1777954	0,000658
Cloud_Mesh_Distance_BA_R0_R3	17,60108948	-78,64199829	-940,0193481	-0,01192
Cloud_Mesh_Distance_BA_R0_R3	17,46925163	-78,67804718	-940,1130981	-0,008254
Cloud_Mesh_Distance_BA_R0_R3	17,70495987	-78,52541351	-940,0059204	-0,004984
Cloud_Mesh_Distance_BA_R0_R3	17,59156799	-78,52915955	-940,1003418	-0,003845
Cloud_Mesh_Distance_BA_R0_R3	21,94560623	-78,94210052	-940,2214966	-0,013064
Cloud_Mesh_Distance_BA_R0_R3	21,8863678	-78,99514008	-940,1211548	-0,011218
Cloud_Mesh_Distance_BA_R0_R3	22,14144516	-79,05882263	-940,3329468	-0,025642
Cloud_Mesh_Distance_BA_R0_R3	22,06840515	-78,93746185	-940,359436	-0,02002
Cloud_Mesh_Distance_BA_R0_R3	17,58447266	-78,41034698	-940,185669	0,004352
Cloud_Mesh_Distance_BA_R0_R3	17,68784332	-78,39962006	-940,1033936	0,005399
Cloud_Mesh_Distance_BA_R0_R3	21,753479	-78,83487701	-940,1225586	0,002857
Cloud_Mesh_Distance_BA_R0_R3	21,94449234	-78,83750153	-940,314148	-0,011716
Cloud_Mesh_Distance_BA_R0_R3	17,86313629	-78,27857208	-940,0410767	0,022213
Cloud_Mesh_Distance_BA_R0_R3	17,78590202	-78,25811768	-940,1190796	0,021672
Cloud_Mesh_Distance_BA_R0_R3	17,87257195	-78,2161026	-940,0770264	0,028514
Cloud_Mesh_Distance_BA_R0_R3	21,74068451	-78,56739044	-940,3496094	0,003895
Cloud_Mesh_Distance_BA_R0_R3	21,8103714	-78,75276947	-940,2480469	-0,000466
Cloud_Mesh_Distance_BA_R0_R3	21,98210907	-78,79920197	-940,3934937	-0,012296
Cloud_Mesh_Distance_BA_R0_R3	17,90169334	-78,1231308	-940,1206055	0,039284
Cloud_Mesh_Distance_BA_R0_R3	18,01712799	-78,00397491	-940,1204224	0,060606
Cloud_Mesh_Distance_BA_R0_R3	18,20860672	-77,99222565	-939,9884033	0,073115
Cloud_Mesh_Distance_BA_R0_R3	21,12406731	-78,21051788	-940,1321411	0,021688
Cloud_Mesh_Distance_BA_R0_R3	21,3446064	-78,44244385	-940,0896606	0,030383
Cloud_Mesh_Distance_BA_R0_R3	21,44506836	-78,39923859	-940,210022	0,023743
Cloud_Mesh_Distance_BA_R0_R3	21,62000465	-78,55768585	-940,2322998	0,013571
Cloud_Mesh_Distance_BA_R0_R3	18,18856239	-77,86400604	-940,112854	0,079714
Cloud_Mesh_Distance_BA_R0_R3	18,06460381	-77,89011383	-940,1801758	0,070234
Cloud_Mesh_Distance_BA_R0_R3	18,25488663	-77,90511322	-940,0298462	0,08308

Cloud_Mesh_Distance_BA_R0_R3	18,13589478	-77,95542145	-940,0700684	0,070378
Cloud_Mesh_Distance_BA_R0_R3	20,91361237	-78,03573608	-940,1582031	0,006074
Cloud_Mesh_Distance_BA_R0_R3	21,37194061	-78,23387909	-940,3005981	0,025335
Cloud_Mesh_Distance_BA_R0_R3	21,2867012	-78,29319763	-940,173584	0,025177
Cloud_Mesh_Distance_BA_R0_R3	18,30304527	-77,69963837	-940,2008667	0,093014
Cloud_Mesh_Distance_BA_R0_R3	18,40390396	-77,62671661	-940,2224121	0,091632
Cloud_Mesh_Distance_BA_R0_R3	20,58446503	-77,83112335	-940,1740723	-0,023012
Cloud_Mesh_Distance_BA_R0_R3	20,66302872	-77,91177368	-940,1373901	-0,015975
Cloud_Mesh_Distance_BA_R0_R3	20,791008	-77,96821594	-940,1516113	-0,003594
Cloud_Mesh_Distance_BA_R0_R3	20,91314888	-77,9315033	-940,2549439	0,004716
Cloud_Mesh_Distance_BA_R0_R3	21,03869247	-78,06929779	-940,2033691	0,015065
Cloud_Mesh_Distance_BA_R0_R3	21,20976067	-78,00128937	-940,3959351	0,022452
Cloud_Mesh_Distance_BA_R0_R3	21,20814705	-78,12555695	-940,2692871	0,021948
Cloud_Mesh_Distance_BA_R0_R3	21,31081009	-78,10655975	-940,3745117	0,022457
Cloud_Mesh_Distance_BA_R0_R3	18,5029068	-77,63311005	-940,1624146	0,095144
Cloud_Mesh_Distance_BA_R0_R3	18,68654633	-77,49850464	-940,2478638	0,085635
Cloud_Mesh_Distance_BA_R0_R3	18,53676605	-77,55254364	-940,2424927	0,091633
Cloud_Mesh_Distance_BA_R0_R3	18,99085808	-77,49049377	-940,1782227	0,070223
Cloud_Mesh_Distance_BA_R0_R3	18,74062729	-77,58306122	-940,1227417	0,086242
Cloud_Mesh_Distance_BA_R0_R3	18,83106041	-77,49118805	-940,2111206	0,080722
Cloud_Mesh_Distance_BA_R0_R3	19,19563293	-77,54668427	-940,0873413	0,053604
Cloud_Mesh_Distance_BA_R0_R3	19,76124763	-77,5125351	-940,1984253	-0,006145
Cloud_Mesh_Distance_BA_R0_R3	20,54931259	-77,73821259	-940,2498169	-0,025554
Cloud_Mesh_Distance_BA_R0_R3	20,46158981	-77,78788757	-940,1573486	-0,028558
Cloud_Mesh_Distance_BA_R0_R3	20,74490356	-77,75231171	-940,3388062	-0,010853
Cloud_Mesh_Distance_BA_R0_R3	20,717556	-77,82593536	-940,2480469	-0,013993
Cloud_Mesh_Distance_BA_R0_R3	21,08148575	-77,97987366	-940,3181152	0,013172
Cloud_Mesh_Distance_BA_R0_R3	20,98897743	-77,85868073	-940,3773193	0,009252
Cloud_Mesh_Distance_BA_R0_R3	21,10871506	-77,90535736	-940,4159546	0,017807
Cloud_Mesh_Distance_BA_R0_R3	19,09255219	-77,50577545	-940,145813	0,058685
Cloud_Mesh_Distance_BA_R0_R3	19,56633377	-77,44167328	-940,250061	0,010481
Cloud_Mesh_Distance_BA_R0_R3	19,92528152	-77,47994232	-940,288147	-0,019409
Cloud_Mesh_Distance_BA_R0_R3	19,93539047	-77,57909393	-940,166687	-0,017145
Cloud_Mesh_Distance_BA_R0_R3	20,08522415	-77,4845047	-940,3370972	-0,030238
Cloud_Mesh_Distance_BA_R0_R3	20,27218437	-77,6852951	-940,1774902	-0,030767
Cloud_Mesh_Distance_BA_R0_R3	20,2755146	-77,5487442	-940,3347778	-0,03384
Cloud_Mesh_Distance_BA_R0_R3	20,4006958	-77,68469238	-940,2352295	-0,030393
Cloud_Mesh_Distance_BA_R0_R3	19,25256348	-77,39437103	-940,2870483	0,041016
Cloud_Mesh_Distance_BA_R0_R3	19,39342499	-77,40695953	-940,2753906	0,025393
Cloud_Mesh_Distance_BA_R0_R3	19,12015915	-83,29987335	-939,7133179	-0,146775
Cloud_Mesh_Distance_BA_R0_R3	19,02319336	-83,37717438	-939,8126221	-0,162538
Cloud_Mesh_Distance_BA_R0_R3	19,16762924	-83,41931915	-939,8166504	-0,153518
Cloud_Mesh_Distance_BA_R0_R3	19,35854149	-83,4587326	-939,826416	-0,1479
Cloud_Mesh_Distance_BA_R0_R3	19,31982613	-83,34650421	-939,7228394	-0,144225
Cloud_Mesh_Distance_BA_R0_R3	19,50618362	-83,38388824	-939,7476807	-0,133808
Cloud_Mesh_Distance_BA_R0_R3	19,54268646	-83,48807526	-939,8475952	-0,139382
Cloud_Mesh_Distance_BA_R0_R3	18,81243896	-83,28052521	-939,7942505	-0,16281
Cloud_Mesh_Distance_BA_R0_R3	18,66523361	-83,22187042	-939,8068237	-0,165273
Cloud_Mesh_Distance_BA_R0_R3	18,96035767	-83,30826569	-939,7648315	-0,163591
Cloud_Mesh_Distance_BA_R0_R3	19,12757492	-83,18883514	-939,6173096	-0,137417

Cloud_Mesh_Distance_BA_R0_R3	19,65328979	-83,41738129	-939,7841797	-0,132515
Cloud_Mesh_Distance_BA_R0_R3	18,36115646	-82,92267609	-939,7041016	-0,161506
Cloud_Mesh_Distance_BA_R0_R3	18,77098274	-83,18048859	-939,7180176	-0,160676
Cloud_Mesh_Distance_BA_R0_R3	18,61402893	-83,1160202	-939,7328491	-0,167033
Cloud_Mesh_Distance_BA_R0_R3	18,94933701	-83,20522308	-939,6751709	-0,151884
Cloud_Mesh_Distance_BA_R0_R3	19,03839493	-83,08426666	-939,5567017	-0,140286
Cloud_Mesh_Distance_BA_R0_R3	19,28600121	-83,22879791	-939,6265869	-0,130638
Cloud_Mesh_Distance_BA_R0_R3	19,63611031	-83,1654129	-939,5780029	-0,103752
Cloud_Mesh_Distance_BA_R0_R3	19,43048096	-83,13898468	-939,5523682	-0,115153
Cloud_Mesh_Distance_BA_R0_R3	19,6836853	-83,30776215	-939,6923828	-0,115739
Cloud_Mesh_Distance_BA_R0_R3	19,47711372	-83,26598358	-939,6474609	-0,120008
Cloud_Mesh_Distance_BA_R0_R3	19,85571671	-83,30950165	-939,7175903	-0,10792
Cloud_Mesh_Distance_BA_R0_R3	19,85982132	-83,19025421	-939,6248779	-0,098298
Cloud_Mesh_Distance_BA_R0_R3	20,19989014	-83,22719574	-939,7368774	-0,087261
Cloud_Mesh_Distance_BA_R0_R3	20,35557556	-83,20815277	-939,77771	-0,078534
Cloud_Mesh_Distance_BA_R0_R3	20,19897079	-83,11304474	-939,652771	-0,073787
Cloud_Mesh_Distance_BA_R0_R3	20,34996033	-83,11185455	-939,704895	-0,073018
Cloud_Mesh_Distance_BA_R0_R3	18,16637039	-82,64368439	-939,6048584	-0,157194
Cloud_Mesh_Distance_BA_R0_R3	18,25733948	-82,74027252	-939,619751	-0,159326
Cloud_Mesh_Distance_BA_R0_R3	18,22458267	-82,86461639	-939,7438965	-0,162372
Cloud_Mesh_Distance_BA_R0_R3	18,46956253	-82,92224884	-939,6415405	-0,161451
Cloud_Mesh_Distance_BA_R0_R3	20,26117897	-83,00003815	-939,5985718	-0,057556
Cloud_Mesh_Distance_BA_R0_R3	20,43326187	-83,15773773	-939,7720337	-0,072577
Cloud_Mesh_Distance_BA_R0_R3	20,7065773	-82,97063446	-939,7719727	-0,056708
Cloud_Mesh_Distance_BA_R0_R3	20,61712074	-82,91477203	-939,6942139	-0,051467
Cloud_Mesh_Distance_BA_R0_R3	18,0462265	-82,59860992	-939,6543579	-0,150922
Cloud_Mesh_Distance_BA_R0_R3	17,92110825	-82,54679108	-939,7141113	-0,148159
Cloud_Mesh_Distance_BA_R0_R3	20,89585495	-82,87119293	-939,8128662	-0,052774
Cloud_Mesh_Distance_BA_R0_R3	18,00853157	-82,49762726	-939,6083374	-0,149376
Cloud_Mesh_Distance_BA_R0_R3	20,80281067	-82,83512115	-939,741272	-0,050042
Cloud_Mesh_Distance_BA_R0_R3	21,23159409	-82,74178314	-939,9372559	-0,049734
Cloud_Mesh_Distance_BA_R0_R3	21,06829071	-82,58350372	-939,75	-0,03892
Cloud_Mesh_Distance_BA_R0_R3	21,01643562	-82,77141571	-939,822937	-0,048968
Cloud_Mesh_Distance_BA_R0_R3	17,69881058	-82,19617462	-939,6687622	-0,137799
Cloud_Mesh_Distance_BA_R0_R3	17,79494286	-82,08126068	-939,5109863	-0,134847
Cloud_Mesh_Distance_BA_R0_R3	21,21546364	-82,44438934	-939,762146	-0,031935
Cloud_Mesh_Distance_BA_R0_R3	21,35953712	-82,47383881	-939,8658447	-0,044847
Cloud_Mesh_Distance_BA_R0_R3	21,20041466	-82,60489655	-939,8388672	-0,045688
Cloud_Mesh_Distance_BA_R0_R3	21,31048393	-82,60569	-939,9082642	-0,05023
Cloud_Mesh_Distance_BA_R0_R3	17,56746101	-81,97811127	-939,6767578	-0,136306
Cloud_Mesh_Distance_BA_R0_R3	17,66333771	-82,06906891	-939,6258545	-0,133743
Cloud_Mesh_Distance_BA_R0_R3	21,38771629	-82,29483795	-939,7890625	-0,031304
Cloud_Mesh_Distance_BA_R0_R3	21,66724014	-82,26612091	-939,965271	-0,051607
Cloud_Mesh_Distance_BA_R0_R3	17,53001785	-81,84339142	-939,649231	-0,131037
Cloud_Mesh_Distance_BA_R0_R3	21,43672943	-82,15044403	-939,7460938	-0,020949
Cloud_Mesh_Distance_BA_R0_R3	21,5151062	-82,3368454	-939,8950195	-0,045072
Cloud_Mesh_Distance_BA_R0_R3	17,37803078	-81,58722687	-939,71875	-0,13488
Cloud_Mesh_Distance_BA_R0_R3	17,3675499	-81,40526581	-939,6696777	-0,140519
Cloud_Mesh_Distance_BA_R0_R3	17,47938728	-81,70615387	-939,645752	-0,128096
Cloud_Mesh_Distance_BA_R0_R3	21,60208893	-82,12960052	-939,8433228	-0,033744

Cloud_Mesh_Distance_BA_R0_R3	21,79215622	-82,1149826	-939,9776611	-0,052215
Cloud_Mesh_Distance_BA_R0_R3	21,81031227	-81,95121002	-939,9030762	-0,040767
Cloud_Mesh_Distance_BA_R0_R3	17,44181824	-81,29985809	-939,5581665	-0,137705
Cloud_Mesh_Distance_BA_R0_R3	21,67819023	-81,90851593	-939,7841797	-0,019651
Cloud_Mesh_Distance_BA_R0_R3	21,68971252	-81,7543869	-939,7250977	-0,010911
Cloud_Mesh_Distance_BA_R0_R3	21,96805382	-81,8303299	-939,9716187	-0,055387
Cloud_Mesh_Distance_BA_R0_R3	21,8632431	-81,73575592	-939,8424072	-0,034321
Cloud_Mesh_Distance_BA_R0_R3	17,3047924	-81,06487274	-939,6610107	-0,135019
Cloud_Mesh_Distance_BA_R0_R3	17,27954483	-81,26959991	-939,7366943	-0,135134
Cloud_Mesh_Distance_BA_R0_R3	17,36800003	-81,28575134	-939,6369629	-0,139683
Cloud_Mesh_Distance_BA_R0_R3	22,01120377	-81,7014389	-939,947937	-0,054947
Cloud_Mesh_Distance_BA_R0_R3	22,09886742	-81,62453461	-939,9915772	-0,070546
Cloud_Mesh_Distance_BA_R0_R3	17,12516975	-80,92295074	-939,8532715	-0,109958
Cloud_Mesh_Distance_BA_R0_R3	17,21673203	-80,94709015	-939,7432251	-0,124796
Cloud_Mesh_Distance_BA_R0_R3	21,90135956	-81,36571503	-939,7522583	-0,039009
Cloud_Mesh_Distance_BA_R0_R3	21,91013336	-81,53350067	-939,8029175	-0,038708
Cloud_Mesh_Distance_BA_R0_R3	22,01801109	-81,58254242	-939,9052734	-0,054754
Cloud_Mesh_Distance_BA_R0_R3	22,19999695	-81,34169769	-939,984436	-0,096236
Cloud_Mesh_Distance_BA_R0_R3	22,05473328	-81,39885712	-939,8760376	-0,065298
Cloud_Mesh_Distance_BA_R0_R3	22,14660263	-81,49784088	-939,9858398	-0,081049
Cloud_Mesh_Distance_BA_R0_R3	17,15273094	-80,5966568	-939,7799683	-0,091939
Cloud_Mesh_Distance_BA_R0_R3	17,16315079	-80,77328491	-939,7832642	-0,106225
Cloud_Mesh_Distance_BA_R0_R3	17,24163055	-80,64302826	-939,6781006	-0,108636
Cloud_Mesh_Distance_BA_R0_R3	17,25993347	-80,7868042	-939,6708374	-0,123814
Cloud_Mesh_Distance_BA_R0_R3	22,15460014	-81,21852875	-939,913269	-0,093056
Cloud_Mesh_Distance_BA_R0_R3	17,22223663	-80,47997284	-939,6920776	-0,091463
Cloud_Mesh_Distance_BA_R0_R3	22,17981339	-81,05849457	-939,9038086	-0,103707
Cloud_Mesh_Distance_BA_R0_R3	21,97567368	-80,61728668	-939,7185669	-0,083873
Cloud_Mesh_Distance_BA_R0_R3	22,18842506	-80,91609955	-939,8912964	-0,107822
Cloud_Mesh_Distance_BA_R0_R3	22,28581238	-80,81330109	-939,9602051	-0,130532
Cloud_Mesh_Distance_BA_R0_R3	22,43581772	-80,75870514	-940,0907593	-0,164522
Cloud_Mesh_Distance_BA_R0_R3	22,26539612	-80,97278595	-939,961792	-0,121914
Cloud_Mesh_Distance_BA_R0_R3	17,04466248	-80,2768631	-939,9210205	-0,054508
Cloud_Mesh_Distance_BA_R0_R3	17,11981201	-80,22415924	-939,8223267	-0,053843
Cloud_Mesh_Distance_BA_R0_R3	17,20106888	-80,28279114	-939,7195435	-0,069205
Cloud_Mesh_Distance_BA_R0_R3	17,16032028	-80,09818268	-939,7844238	-0,048596
Cloud_Mesh_Distance_BA_R0_R3	22,07824707	-80,5887146	-939,788269	-0,099784
Cloud_Mesh_Distance_BA_R0_R3	22,18631744	-80,63239288	-939,8688965	-0,114098
Cloud_Mesh_Distance_BA_R0_R3	22,06637955	-80,67079926	-939,7824097	-0,098777
Cloud_Mesh_Distance_BA_R0_R3	22,18582916	-80,79749298	-939,8774414	-0,110667
Cloud_Mesh_Distance_BA_R0_R3	22,33698654	-80,65624237	-939,9938965	-0,142073
Cloud_Mesh_Distance_BA_R0_R3	22,21656036	-80,38498688	-939,8963013	-0,122326
Cloud_Mesh_Distance_BA_R0_R3	17,12736893	-79,7216568	-939,9158325	-0,014907
Cloud_Mesh_Distance_BA_R0_R3	17,17433929	-79,83286285	-939,8225098	-0,027202
Cloud_Mesh_Distance_BA_R0_R3	17,26890945	-79,75956726	-939,7358398	-0,028417
Cloud_Mesh_Distance_BA_R0_R3	17,23785782	-79,94908905	-939,7189331	-0,040161
Cloud_Mesh_Distance_BA_R0_R3	22,34266281	-80,30325317	-940,008667	-0,140944
Cloud_Mesh_Distance_BA_R0_R3	22,41666985	-80,24555969	-940,083313	-0,151445
Cloud_Mesh_Distance_BA_R0_R3	22,35962677	-80,13832092	-940,0452881	-0,138957
Cloud_Mesh_Distance_BA_R0_R3	17,1959343	-79,72789764	-939,8293457	-0,021255

Cloud_Mesh_Distance_BA_R0_R3	17,29501724	-79,47324371	-939,8230591	-0,018502
Cloud_Mesh_Distance_BA_R0_R3	17,3720665	-79,60268402	-939,6871948	-0,030257
Cloud_Mesh_Distance_BA_R0_R3	17,38706398	-79,41422272	-939,7588501	-0,024835
Cloud_Mesh_Distance_BA_R0_R3	17,21782684	-79,60364532	-939,8512573	-0,015615
Cloud_Mesh_Distance_BA_R0_R3	17,47771072	-79,46720123	-939,6484985	-0,033011
Cloud_Mesh_Distance_BA_R0_R3	22,24669456	-80,16999817	-939,9414673	-0,122235
Cloud_Mesh_Distance_BA_R0_R3	22,16726303	-80,04347992	-939,8959351	-0,104019
Cloud_Mesh_Distance_BA_R0_R3	22,38782883	-80,01494598	-940,0963745	-0,133854
Cloud_Mesh_Distance_BA_R0_R3	22,14837456	-79,92119598	-939,9041748	-0,088688
Cloud_Mesh_Distance_BA_R0_R3	22,30102158	-79,85323334	-940,0553589	-0,108218
Cloud_Mesh_Distance_BA_R0_R3	22,2669487	-79,95415497	-939,998291	-0,112286
Cloud_Mesh_Distance_BA_R0_R3	22,35774994	-79,89382172	-940,0982666	-0,1186
Cloud_Mesh_Distance_BA_R0_R3	17,44578934	-79,17346954	-939,8323975	-0,023526
Cloud_Mesh_Distance_BA_R0_R3	17,35167694	-79,21954346	-939,8971558	-0,019258
Cloud_Mesh_Distance_BA_R0_R3	17,39451981	-79,29209137	-939,8144531	-0,022928
Cloud_Mesh_Distance_BA_R0_R3	22,0721817	-79,71102142	-939,8995361	-0,064977
Cloud_Mesh_Distance_BA_R0_R3	22,16826248	-79,6612854	-939,9949951	-0,069797
Cloud_Mesh_Distance_BA_R0_R3	22,16663742	-79,80625153	-939,9485474	-0,085695
Cloud_Mesh_Distance_BA_R0_R3	17,51641083	-78,99445343	-939,8744507	-0,026877
Cloud_Mesh_Distance_BA_R0_R3	17,69915962	-78,9593277	-939,7391968	-0,033203
Cloud_Mesh_Distance_BA_R0_R3	22,23392868	-79,56848907	-940,0931397	-0,067623
Cloud_Mesh_Distance_BA_R0_R3	17,6608963	-78,86484528	-939,8280029	-0,026956
Cloud_Mesh_Distance_BA_R0_R3	17,87135696	-78,83661652	-939,6738892	-0,027687
Cloud_Mesh_Distance_BA_R0_R3	21,7788105	-79,09189606	-939,9633179	-0,003164
Cloud_Mesh_Distance_BA_R0_R3	22,00783539	-79,35049438	-939,9957886	-0,027846
Cloud_Mesh_Distance_BA_R0_R3	22,16266251	-79,46366119	-940,0739136	-0,04922
Cloud_Mesh_Distance_BA_R0_R3	22,12116623	-79,35811615	-940,092041	-0,037759
Cloud_Mesh_Distance_BA_R0_R3	17,55683899	-78,79366302	-939,9619751	-0,021407
Cloud_Mesh_Distance_BA_R0_R3	17,69528008	-78,68061829	-939,9138794	-0,016283
Cloud_Mesh_Distance_BA_R0_R3	17,8011322	-78,7383728	-939,7894898	-0,02472
Cloud_Mesh_Distance_BA_R0_R3	21,67378616	-78,98695374	-939,9476318	0,008223
Cloud_Mesh_Distance_BA_R0_R3	21,68440247	-79,10652924	-939,8833618	0,006089
Cloud_Mesh_Distance_BA_R0_R3	21,88731766	-79,09295654	-940,0517578	-0,011279
Cloud_Mesh_Distance_BA_R0_R3	21,96756554	-79,06777954	-940,1420288	-0,017793
Cloud_Mesh_Distance_BA_R0_R3	17,86481094	-78,51914215	-939,8761597	-0,001548
Cloud_Mesh_Distance_BA_R0_R3	21,6428833	-78,82751465	-940,0313721	0,01459
Cloud_Mesh_Distance_BA_R0_R3	21,68393135	-78,72902679	-940,1433716	0,008221
Cloud_Mesh_Distance_BA_R0_R3	21,78214264	-78,96221924	-940,052124	0,000556
Cloud_Mesh_Distance_BA_R0_R3	21,83781433	-78,89911652	-940,1499023	-0,005539
Cloud_Mesh_Distance_BA_R0_R3	17,80236053	-78,38397217	-940,0184937	0,007536
Cloud_Mesh_Distance_BA_R0_R3	17,91796494	-78,33119965	-939,9593506	0,02119
Cloud_Mesh_Distance_BA_R0_R3	18,02140236	-78,36296844	-939,8551636	0,020465
Cloud_Mesh_Distance_BA_R0_R3	21,58203506	-78,6733017	-940,0964966	0,017816
Cloud_Mesh_Distance_BA_R0_R3	21,47721863	-78,55461884	-940,1033936	0,026831
Cloud_Mesh_Distance_BA_R0_R3	17,94218445	-78,21257782	-940,0229492	0,033522
Cloud_Mesh_Distance_BA_R0_R3	18,02475739	-78,24331665	-939,93573	0,034477
Cloud_Mesh_Distance_BA_R0_R3	18,08036423	-78,09848785	-939,9989014	0,050153
Cloud_Mesh_Distance_BA_R0_R3	18,2536869	-78,06309509	-939,9006958	0,066344
Cloud_Mesh_Distance_BA_R0_R3	18,38275146	-78,00528717	-939,8659668	0,074485
Cloud_Mesh_Distance_BA_R0_R3	20,84493637	-78,29214478	-939,9116211	0,013356

Cloud_Mesh_Distance_BA_R0_R3	21,19618607	-78,35890961	-940,0534058	0,030831
Cloud_Mesh_Distance_BA_R0_R3	21,01533699	-78,28500366	-940,0043335	0,021616
Cloud_Mesh_Distance_BA_R0_R3	21,37658501	-78,5632782	-940,0166626	0,032954
Cloud_Mesh_Distance_BA_R0_R3	18,35799408	-77,87677765	-939,9904785	0,086859
Cloud_Mesh_Distance_BA_R0_R3	18,31671524	-77,95648956	-939,9462891	0,080485
Cloud_Mesh_Distance_BA_R0_R3	20,49082565	-77,99565125	-939,984314	-0,023619
Cloud_Mesh_Distance_BA_R0_R3	20,72260666	-78,18265533	-939,9389038	-0,00039
Cloud_Mesh_Distance_BA_R0_R3	20,85004044	-78,16850281	-940,0112305	0,00663
Cloud_Mesh_Distance_BA_R0_R3	20,96817017	-78,13977051	-940,0981445	0,00999
Cloud_Mesh_Distance_BA_R0_R3	18,31416512	-77,80395508	-940,0862427	0,088949
Cloud_Mesh_Distance_BA_R0_R3	18,45853043	-77,81678772	-939,989502	0,091049
Cloud_Mesh_Distance_BA_R0_R3	18,59895325	-77,6490097	-940,0988159	0,097266
Cloud_Mesh_Distance_BA_R0_R3	18,61517334	-77,75415802	-939,9783936	0,093967
Cloud_Mesh_Distance_BA_R0_R3	18,45898819	-77,70874786	-940,1004028	0,094939
Cloud_Mesh_Distance_BA_R0_R3	18,72042465	-77,67577362	-940,0211792	0,090712
Cloud_Mesh_Distance_BA_R0_R3	19,71285248	-77,70462799	-939,9609375	0,006736
Cloud_Mesh_Distance_BA_R0_R3	19,80464172	-77,80051422	-939,8878784	-0,000243
Cloud_Mesh_Distance_BA_R0_R3	20,35688782	-78,00115204	-939,9211426	-0,027908
Cloud_Mesh_Distance_BA_R0_R3	20,53723526	-77,8993988	-940,0883179	-0,025304
Cloud_Mesh_Distance_BA_R0_R3	20,68502808	-78,03951263	-940,0368652	-0,010164
Cloud_Mesh_Distance_BA_R0_R3	20,8440609	-78,07068634	-940,0896606	-0,00027
Cloud_Mesh_Distance_BA_R0_R3	18,76857567	-77,7385788	-939,9379273	0,088002
Cloud_Mesh_Distance_BA_R0_R3	18,92874527	-77,57117462	-940,0877075	0,079116
Cloud_Mesh_Distance_BA_R0_R3	19,058918	-77,55368042	-940,0881958	0,064969
Cloud_Mesh_Distance_BA_R0_R3	19,20939445	-77,76465607	-939,8414917	0,056095
Cloud_Mesh_Distance_BA_R0_R3	19,54941559	-77,71727753	-939,9141846	0,020228
Cloud_Mesh_Distance_BA_R0_R3	19,34487534	-77,67223358	-939,9405518	0,04312
Cloud_Mesh_Distance_BA_R0_R3	19,6997509	-77,60062408	-940,074585	0,00468
Cloud_Mesh_Distance_BA_R0_R3	19,60652733	-77,52928925	-940,1425781	0,010257
Cloud_Mesh_Distance_BA_R0_R3	19,83455658	-77,74549866	-939,9507446	-0,006542
Cloud_Mesh_Distance_BA_R0_R3	20,02256775	-77,66104889	-940,1030884	-0,021504
Cloud_Mesh_Distance_BA_R0_R3	19,87139511	-77,66796112	-940,0437622	-0,00967
Cloud_Mesh_Distance_BA_R0_R3	20,18055725	-77,82077789	-940,0020142	-0,028096
Cloud_Mesh_Distance_BA_R0_R3	20,34072304	-77,83839417	-940,0543213	-0,030525
Cloud_Mesh_Distance_BA_R0_R3	20,16013336	-77,7226944	-940,09021	-0,026855
Cloud_Mesh_Distance_BA_R0_R3	20,36678123	-77,74750519	-940,1547852	-0,033593
Cloud_Mesh_Distance_BA_R0_R3	19,3700943	-77,55567169	-940,078186	0,038276
Cloud_Mesh_Distance_BA_R0_R3	19,29913712	-77,46998596	-940,1853027	0,041841
Cloud_Mesh_Distance_BA_R0_R3	19,53778458	-77,6067276	-940,0350342	0,019979
Cloud_Mesh_Distance_BA_R0_R3	19,46304131	-77,49895477	-940,1589966	0,022254
Cloud_Mesh_Distance_BA_R0_R3	20,09593773	-77,59044647	-940,2113648	-0,028986
Cloud_Mesh_Distance_BA_R0_R3	18,60418892	-82,98470306	-939,6254273	-0,155338
Cloud_Mesh_Distance_BA_R0_R3	18,73350906	-82,92606354	-939,5286865	-0,150907
Cloud_Mesh_Distance_BA_R0_R3	18,56097412	-82,8436203	-939,5371094	-0,151519
Cloud_Mesh_Distance_BA_R0_R3	18,80713654	-83,0655899	-939,607544	-0,155523
Cloud_Mesh_Distance_BA_R0_R3	19,23774338	-83,10897064	-939,5431519	-0,127813
Cloud_Mesh_Distance_BA_R0_R3	18,93559837	-82,92896271	-939,4741211	-0,134671
Cloud_Mesh_Distance_BA_R0_R3	19,37299347	-83,00835419	-939,4693604	-0,111189
Cloud_Mesh_Distance_BA_R0_R3	19,79323959	-83,03105927	-939,5078735	-0,085915
Cloud_Mesh_Distance_BA_R0_R3	19,98490143	-83,0561142	-939,5573731	-0,074113

Cloud_Mesh_Distance_BA_R0_R3	20,06152534	-83,15628815	-939,6439819	-0,086566
Cloud_Mesh_Distance_BA_R0_R3	20,12387466	-83,05698395	-939,5921631	-0,068881
Cloud_Mesh_Distance_BA_R0_R3	18,38089752	-82,83751678	-939,6220703	-0,15812
Cloud_Mesh_Distance_BA_R0_R3	18,28849411	-82,62847137	-939,5200195	-0,149841
Cloud_Mesh_Distance_BA_R0_R3	19,16807556	-82,97759247	-939,4650269	-0,1231
Cloud_Mesh_Distance_BA_R0_R3	19,5742588	-83,02918243	-939,4851074	-0,100452
Cloud_Mesh_Distance_BA_R0_R3	19,84248734	-82,90312958	-939,4431763	-0,074322
Cloud_Mesh_Distance_BA_R0_R3	19,93836975	-82,83332062	-939,4244995	-0,060523
Cloud_Mesh_Distance_BA_R0_R3	19,95645905	-82,93573761	-939,4802856	-0,065297
Cloud_Mesh_Distance_BA_R0_R3	20,24043655	-82,89644623	-939,5308838	-0,049486
Cloud_Mesh_Distance_BA_R0_R3	20,44381523	-82,97098541	-939,6502686	-0,054715
Cloud_Mesh_Distance_BA_R0_R3	20,35849762	-82,87985992	-939,5621338	-0,044436
Cloud_Mesh_Distance_BA_R0_R3	20,52127457	-82,81368256	-939,5933228	-0,040514
Cloud_Mesh_Distance_BA_R0_R3	18,56937599	-82,70029449	-939,4385376	-0,137844
Cloud_Mesh_Distance_BA_R0_R3	18,41305923	-82,72559357	-939,5223389	-0,152256
Cloud_Mesh_Distance_BA_R0_R3	18,93733978	-82,78749847	-939,3893433	-0,126201
Cloud_Mesh_Distance_BA_R0_R3	18,85337067	-82,69028473	-939,3525391	-0,121547
Cloud_Mesh_Distance_BA_R0_R3	18,75584984	-82,79184723	-939,4337769	-0,135269
Cloud_Mesh_Distance_BA_R0_R3	20,11624146	-82,94753265	-939,5228882	-0,0585
Cloud_Mesh_Distance_BA_R0_R3	20,67438507	-82,79431915	-939,6533203	-0,041733
Cloud_Mesh_Distance_BA_R0_R3	17,80057907	-82,23145294	-939,595459	-0,142033
Cloud_Mesh_Distance_BA_R0_R3	17,8901329	-82,30918121	-939,5701294	-0,143172
Cloud_Mesh_Distance_BA_R0_R3	18,02391052	-82,38547516	-939,5203247	-0,142978
Cloud_Mesh_Distance_BA_R0_R3	18,16935158	-82,51238251	-939,5106812	-0,145264
Cloud_Mesh_Distance_BA_R0_R3	18,34632111	-82,46233368	-939,3880615	-0,132428
Cloud_Mesh_Distance_BA_R0_R3	18,42462921	-82,59650421	-939,4321289	-0,138136
Cloud_Mesh_Distance_BA_R0_R3	18,30656052	-82,54973602	-939,4597778	-0,144938
Cloud_Mesh_Distance_BA_R0_R3	18,49081802	-82,51303864	-939,3577271	-0,128988
Cloud_Mesh_Distance_BA_R0_R3	20,4977417	-82,65164948	-939,505188	-0,027834
Cloud_Mesh_Distance_BA_R0_R3	20,66067886	-82,67928314	-939,5881348	-0,031439
Cloud_Mesh_Distance_BA_R0_R3	20,52802658	-82,4693222	-939,4445801	-0,018868
Cloud_Mesh_Distance_BA_R0_R3	20,91253281	-82,66851044	-939,7091065	-0,04056
Cloud_Mesh_Distance_BA_R0_R3	20,77563858	-82,71039581	-939,6593628	-0,038576
Cloud_Mesh_Distance_BA_R0_R3	20,8339138	-82,55748749	-939,6138916	-0,028105
Cloud_Mesh_Distance_BA_R0_R3	17,88032913	-82,19629669	-939,5079956	-0,135154
Cloud_Mesh_Distance_BA_R0_R3	18,00510216	-82,22863007	-939,4380493	-0,135381
Cloud_Mesh_Distance_BA_R0_R3	20,66867828	-82,52381134	-939,5220947	-0,020928
Cloud_Mesh_Distance_BA_R0_R3	20,59075165	-82,32538605	-939,4157715	-0,011159
Cloud_Mesh_Distance_BA_R0_R3	20,83048248	-82,36777496	-939,5291748	-0,014353
Cloud_Mesh_Distance_BA_R0_R3	20,98190117	-82,53568268	-939,678894	-0,031866
Cloud_Mesh_Distance_BA_R0_R3	21,08573532	-82,46637726	-939,7003174	-0,030124
Cloud_Mesh_Distance_BA_R0_R3	17,6057148	-81,85933685	-939,5744629	-0,135541
Cloud_Mesh_Distance_BA_R0_R3	17,57710648	-81,75925446	-939,5595703	-0,131756
Cloud_Mesh_Distance_BA_R0_R3	17,68513107	-81,94013214	-939,5348511	-0,13226
Cloud_Mesh_Distance_BA_R0_R3	17,92418671	-82,12039948	-939,4317627	-0,128055
Cloud_Mesh_Distance_BA_R0_R3	20,6687851	-82,39406586	-939,4708252	-0,014778
Cloud_Mesh_Distance_BA_R0_R3	20,87202263	-82,21535492	-939,4865112	-0,00352
Cloud_Mesh_Distance_BA_R0_R3	21,01473999	-82,39279938	-939,6287231	-0,02235
Cloud_Mesh_Distance_BA_R0_R3	21,15237427	-82,3711319	-939,6904297	-0,025789
Cloud_Mesh_Distance_BA_R0_R3	21,25493813	-82,31279755	-939,7185059	-0,024001

Cloud_Mesh_Distance_BA_R0_R3	21,19387817	-82,23822784	-939,6499023	-0,015424
Cloud_Mesh_Distance_BA_R0_R3	17,600914	-81,61412811	-939,4792481	-0,133809
Cloud_Mesh_Distance_BA_R0_R3	17,70750809	-81,6374588	-939,3873901	-0,119936
Cloud_Mesh_Distance_BA_R0_R3	17,67852402	-81,77144623	-939,4641724	-0,128205
Cloud_Mesh_Distance_BA_R0_R3	21,11082077	-82,02555084	-939,5181885	0,008132
Cloud_Mesh_Distance_BA_R0_R3	21,02927017	-82,20102692	-939,5512695	-0,003959
Cloud_Mesh_Distance_BA_R0_R3	21,32553864	-82,21845245	-939,7126465	-0,019598
Cloud_Mesh_Distance_BA_R0_R3	21,35195541	-82,10201263	-939,6730957	-0,011508
Cloud_Mesh_Distance_BA_R0_R3	17,59514236	-81,45172882	-939,4362793	-0,126595
Cloud_Mesh_Distance_BA_R0_R3	17,70800018	-81,48841095	-939,3450317	-0,116389
Cloud_Mesh_Distance_BA_R0_R3	21,22629547	-82,06916046	-939,5917969	-0,001513
Cloud_Mesh_Distance_BA_R0_R3	21,40929604	-81,9283371	-939,6286011	0,001847
Cloud_Mesh_Distance_BA_R0_R3	21,51079941	-82,01357269	-939,7242432	-0,012304
Cloud_Mesh_Distance_BA_R0_R3	17,54880905	-81,32232666	-939,4514771	-0,132276
Cloud_Mesh_Distance_BA_R0_R3	17,48420334	-81,48316193	-939,5595703	-0,135073
Cloud_Mesh_Distance_BA_R0_R3	21,25488281	-81,92166901	-939,5457764	0,011904
Cloud_Mesh_Distance_BA_R0_R3	21,37709236	-81,78339386	-939,555481	0,01605
Cloud_Mesh_Distance_BA_R0_R3	21,57089424	-81,86891937	-939,6964111	-0,006761
Cloud_Mesh_Distance_BA_R0_R3	21,54876518	-81,7452774	-939,6345215	0,006214
Cloud_Mesh_Distance_BA_R0_R3	21,70776558	-81,54152679	-939,6658325	-0,006563
Cloud_Mesh_Distance_BA_R0_R3	17,38378143	-81,17510986	-939,5940552	-0,139159
Cloud_Mesh_Distance_BA_R0_R3	21,7804451	-81,61862946	-939,7363892	-0,01773
Cloud_Mesh_Distance_BA_R0_R3	21,81463242	-81,4935379	-939,7223511	-0,02096
Cloud_Mesh_Distance_BA_R0_R3	17,41030121	-81,00201416	-939,5370483	-0,140443
Cloud_Mesh_Distance_BA_R0_R3	17,50693893	-81,13726044	-939,4602051	-0,134553
Cloud_Mesh_Distance_BA_R0_R3	21,65071106	-81,16979218	-939,569397	-0,018872
Cloud_Mesh_Distance_BA_R0_R3	21,92217255	-81,23999786	-939,7421875	-0,047701
Cloud_Mesh_Distance_BA_R0_R3	17,42874146	-80,82833862	-939,4990234	-0,131625
Cloud_Mesh_Distance_BA_R0_R3	17,32812119	-80,88523865	-939,6082764	-0,131078
Cloud_Mesh_Distance_BA_R0_R3	21,80519485	-81,23403168	-939,6657715	-0,031929
Cloud_Mesh_Distance_BA_R0_R3	22,01524734	-81,24433899	-939,8092041	-0,063975
Cloud_Mesh_Distance_BA_R0_R3	22,08088493	-81,12598419	-939,8374023	-0,082691
Cloud_Mesh_Distance_BA_R0_R3	17,30392456	-80,53586578	-939,6045532	-0,103035
Cloud_Mesh_Distance_BA_R0_R3	17,30761337	-80,38285828	-939,5993652	-0,092199
Cloud_Mesh_Distance_BA_R0_R3	17,35804749	-80,68701935	-939,5574341	-0,11835
Cloud_Mesh_Distance_BA_R0_R3	17,47417068	-80,61284637	-939,4451904	-0,124941
Cloud_Mesh_Distance_BA_R0_R3	17,44467926	-80,71955872	-939,4760742	-0,127317
Cloud_Mesh_Distance_BA_R0_R3	21,66381454	-80,93224335	-939,5562744	-0,039567
Cloud_Mesh_Distance_BA_R0_R3	21,76263237	-81,01943207	-939,6176758	-0,04399
Cloud_Mesh_Distance_BA_R0_R3	21,8171463	-80,86852264	-939,6362305	-0,058005
Cloud_Mesh_Distance_BA_R0_R3	21,95252609	-81,10905457	-939,7433472	-0,05945
Cloud_Mesh_Distance_BA_R0_R3	22,08346367	-80,99401855	-939,8203125	-0,089065
Cloud_Mesh_Distance_BA_R0_R3	17,39551926	-80,49003601	-939,5126343	-0,10529
Cloud_Mesh_Distance_BA_R0_R3	21,62188339	-80,83181	-939,5271606	-0,041267
Cloud_Mesh_Distance_BA_R0_R3	21,9963665	-80,73989105	-939,7384644	-0,084232
Cloud_Mesh_Distance_BA_R0_R3	22,08109283	-80,76118469	-939,7976685	-0,096924
Cloud_Mesh_Distance_BA_R0_R3	21,96451569	-80,88948822	-939,7283936	-0,072624
Cloud_Mesh_Distance_BA_R0_R3	17,32063675	-80,20676422	-939,5944214	-0,072968
Cloud_Mesh_Distance_BA_R0_R3	17,42968941	-80,1313858	-939,4991455	-0,078319
Cloud_Mesh_Distance_BA_R0_R3	22,14350891	-80,76351166	-939,8433838	-0,1062

Cloud_Mesh_Distance_BA_R0_R3	22,1156311	-80,52184296	-939,8148804	-0,106178
Cloud_Mesh_Distance_BA_R0_R3	22,03255272	-80,48749542	-939,755188	-0,093038
Cloud_Mesh_Distance_BA_R0_R3	22,11110115	-80,85541534	-939,8258667	-0,096743
Cloud_Mesh_Distance_BA_R0_R3	17,24940872	-80,11186218	-939,678833	-0,057306
Cloud_Mesh_Distance_BA_R0_R3	17,33618736	-80,03404999	-939,5993652	-0,056854
Cloud_Mesh_Distance_BA_R0_R3	17,44578171	-79,95226288	-939,5133667	-0,058668
Cloud_Mesh_Distance_BA_R0_R3	22,15897369	-80,48706055	-939,8479614	-0,113113
Cloud_Mesh_Distance_BA_R0_R3	17,35874557	-79,74788666	-939,6473999	-0,038103
Cloud_Mesh_Distance_BA_R0_R3	17,3475647	-79,86629486	-939,6239624	-0,046853
Cloud_Mesh_Distance_BA_R0_R3	21,87072754	-80,24820709	-939,6596069	-0,070244
Cloud_Mesh_Distance_BA_R0_R3	22,00213623	-80,32263947	-939,7400513	-0,090257
Cloud_Mesh_Distance_BA_R0_R3	22,11310005	-80,20412445	-939,8309326	-0,10085
Cloud_Mesh_Distance_BA_R0_R3	22,10965347	-80,40283966	-939,8130493	-0,106519
Cloud_Mesh_Distance_BA_R0_R3	21,89559555	-79,93972015	-939,7178345	-0,062949
Cloud_Mesh_Distance_BA_R0_R3	22,05429459	-79,97899628	-939,8201294	-0,084738
Cloud_Mesh_Distance_BA_R0_R3	21,96949768	-80,13013458	-939,7363281	-0,076105
Cloud_Mesh_Distance_BA_R0_R3	17,49848938	-79,30606842	-939,7110596	-0,033077
Cloud_Mesh_Distance_BA_R0_R3	17,59240913	-79,41082001	-939,5774536	-0,039481
Cloud_Mesh_Distance_BA_R0_R3	17,6687336	-79,47754669	-939,4883423	-0,046008
Cloud_Mesh_Distance_BA_R0_R3	17,66025925	-79,23738861	-939,611145	-0,039037
Cloud_Mesh_Distance_BA_R0_R3	17,71253586	-79,36605072	-939,507019	-0,04785
Cloud_Mesh_Distance_BA_R0_R3	22,08542252	-79,83876801	-939,8739014	-0,076805
Cloud_Mesh_Distance_BA_R0_R3	21,7825737	-79,49498749	-939,769104	-0,023176
Cloud_Mesh_Distance_BA_R0_R3	21,98299789	-79,79419708	-939,8095093	-0,062876
Cloud_Mesh_Distance_BA_R0_R3	21,82221603	-79,721138	-939,7215576	-0,041125
Cloud_Mesh_Distance_BA_R0_R3	21,95354843	-79,59577179	-939,8480225	-0,043507
Cloud_Mesh_Distance_BA_R0_R3	17,56671524	-79,13367462	-939,7476807	-0,033806
Cloud_Mesh_Distance_BA_R0_R3	17,62178802	-79,00658417	-939,7750855	-0,032647
Cloud_Mesh_Distance_BA_R0_R3	17,68346024	-79,07112885	-939,6859131	-0,037513
Cloud_Mesh_Distance_BA_R0_R3	17,78844643	-79,10636139	-939,5836182	-0,042371
Cloud_Mesh_Distance_BA_R0_R3	17,89332581	-79,12065125	-939,5012817	-0,04405
Cloud_Mesh_Distance_BA_R0_R3	17,78389549	-78,97142792	-939,6636963	-0,037515
Cloud_Mesh_Distance_BA_R0_R3	21,89686394	-79,4002533	-939,8850708	-0,023246
Cloud_Mesh_Distance_BA_R0_R3	22,07015991	-79,59282684	-939,9377441	-0,052432
Cloud_Mesh_Distance_BA_R0_R3	22,02608109	-79,48202515	-939,9471436	-0,039005
Cloud_Mesh_Distance_BA_R0_R3	22,12623024	-79,53145599	-940,0092773	-0,056491
Cloud_Mesh_Distance_BA_R0_R3	22,09021568	-79,43604279	-940,0221558	-0,041302
Cloud_Mesh_Distance_BA_R0_R3	17,76046944	-78,87488556	-939,7398071	-0,031685
Cloud_Mesh_Distance_BA_R0_R3	21,46525955	-79,18615723	-939,7047119	0,018852
Cloud_Mesh_Distance_BA_R0_R3	21,71989441	-79,26778412	-939,8245239	-0,004384
Cloud_Mesh_Distance_BA_R0_R3	21,76457405	-79,17316437	-939,90448	-0,003245
Cloud_Mesh_Distance_BA_R0_R3	21,87144089	-79,22550201	-939,9556274	-0,014468
Cloud_Mesh_Distance_BA_R0_R3	17,97263336	-78,65653229	-939,7042236	-0,010258
Cloud_Mesh_Distance_BA_R0_R3	18,15697861	-78,53183746	-939,6471558	0,007545
Cloud_Mesh_Distance_BA_R0_R3	18,24841881	-78,67671967	-939,5057983	-0,005385
Cloud_Mesh_Distance_BA_R0_R3	18,3098259	-78,51131439	-939,5610352	0,014395
Cloud_Mesh_Distance_BA_R0_R3	21,40056419	-78,95301056	-939,781311	0,033638
Cloud_Mesh_Distance_BA_R0_R3	21,40862083	-79,08202362	-939,7206421	0,026825
Cloud_Mesh_Distance_BA_R0_R3	21,53353882	-78,90895844	-939,8931885	0,022361
Cloud_Mesh_Distance_BA_R0_R3	21,61129189	-79,02457428	-939,8789673	0,014654

Cloud_Mesh_Distance_BA_R0_R3	21,55035019	-79,09645081	-939,7988892	0,014372
Cloud_Mesh_Distance_BA_R0_R3	18,02777863	-78,48889923	-939,7675781	0,006222
Cloud_Mesh_Distance_BA_R0_R3	18,32697296	-78,33538055	-939,6613159	0,035542
Cloud_Mesh_Distance_BA_R0_R3	18,43058777	-78,44869232	-939,5333862	0,027161
Cloud_Mesh_Distance_BA_R0_R3	18,40132141	-78,57527924	-939,4772949	0,010959
Cloud_Mesh_Distance_BA_R0_R3	21,24976921	-78,85347748	-939,7486572	0,042831
Cloud_Mesh_Distance_BA_R0_R3	21,02628326	-78,66461945	-939,7422485	0,043129
Cloud_Mesh_Distance_BA_R0_R3	21,36205673	-78,78551483	-939,8533936	0,036525
Cloud_Mesh_Distance_BA_R0_R3	18,13773346	-78,3859787	-939,7536011	0,020965
Cloud_Mesh_Distance_BA_R0_R3	18,17051697	-78,22618866	-939,8392944	0,043541
Cloud_Mesh_Distance_BA_R0_R3	18,67180061	-78,2492218	-939,5574951	0,048642
Cloud_Mesh_Distance_BA_R0_R3	20,16786194	-78,39274597	-939,5918579	-0,023322
Cloud_Mesh_Distance_BA_R0_R3	20,31787682	-78,32534027	-939,6819458	-0,020606
Cloud_Mesh_Distance_BA_R0_R3	20,51202774	-78,36881256	-939,7236328	-0,005972
Cloud_Mesh_Distance_BA_R0_R3	21,03289604	-78,53598785	-939,8271484	0,040077
Cloud_Mesh_Distance_BA_R0_R3	21,18386841	-78,68428802	-939,8104858	0,041785
Cloud_Mesh_Distance_BA_R0_R3	21,18472099	-78,55426788	-939,8973999	0,040152
Cloud_Mesh_Distance_BA_R0_R3	21,31058502	-78,6286087	-939,9237061	0,040564
Cloud_Mesh_Distance_BA_R0_R3	21,47307396	-78,70346832	-939,9855957	0,029337
Cloud_Mesh_Distance_BA_R0_R3	18,3685112	-78,12458038	-939,7833252	0,060588
Cloud_Mesh_Distance_BA_R0_R3	18,74873734	-78,0122757	-939,7001953	0,069525
Cloud_Mesh_Distance_BA_R0_R3	18,53787804	-77,98504639	-939,8027954	0,079178
Cloud_Mesh_Distance_BA_R0_R3	18,5541687	-78,16304779	-939,6609497	0,057193
Cloud_Mesh_Distance_BA_R0_R3	18,85554504	-78,09323883	-939,6119385	0,058609
Cloud_Mesh_Distance_BA_R0_R3	18,72501755	-78,13999176	-939,6141357	0,057763
Cloud_Mesh_Distance_BA_R0_R3	20,18247223	-78,2645874	-939,6708374	-0,024973
Cloud_Mesh_Distance_BA_R0_R3	20,20475769	-78,34889984	-939,6293335	-0,024316
Cloud_Mesh_Distance_BA_R0_R3	20,39711952	-78,17238617	-939,8099365	-0,021502
Cloud_Mesh_Distance_BA_R0_R3	20,67347717	-78,42828369	-939,7449951	0,009557
Cloud_Mesh_Distance_BA_R0_R3	20,65247345	-78,2864151	-939,8323364	-0,001969
Cloud_Mesh_Distance_BA_R0_R3	20,97279739	-78,3963089	-939,8956299	0,027841
Cloud_Mesh_Distance_BA_R0_R3	20,83354187	-78,43578339	-939,803894	0,021982
Cloud_Mesh_Distance_BA_R0_R3	21,11528397	-78,43629456	-939,9428101	0,034203
Cloud_Mesh_Distance_BA_R0_R3	21,25921631	-78,49578094	-939,986084	0,036051
Cloud_Mesh_Distance_BA_R0_R3	18,43637085	-77,91417694	-939,9127808	0,084838
Cloud_Mesh_Distance_BA_R0_R3	18,7248745	-77,85162354	-939,8432617	0,083541
Cloud_Mesh_Distance_BA_R0_R3	19,12693405	-77,86411285	-939,7518311	0,05971
Cloud_Mesh_Distance_BA_R0_R3	18,91551208	-78,00075531	-939,6678467	0,064912
Cloud_Mesh_Distance_BA_R0_R3	18,93375397	-77,88660431	-939,7573853	0,071037
Cloud_Mesh_Distance_BA_R0_R3	19,05075836	-77,96588898	-939,6743164	0,057702
Cloud_Mesh_Distance_BA_R0_R3	19,63476753	-78,01953888	-939,6635132	0,012608
Cloud_Mesh_Distance_BA_R0_R3	20,21324158	-78,02249908	-939,8452148	-0,026208
Cloud_Mesh_Distance_BA_R0_R3	20,45380974	-78,07401276	-939,9052124	-0,021912
Cloud_Mesh_Distance_BA_R0_R3	20,56844139	-78,12501526	-939,9144898	-0,013074
Cloud_Mesh_Distance_BA_R0_R3	18,55632019	-77,86122894	-939,8997192	0,091345
Cloud_Mesh_Distance_BA_R0_R3	18,90493584	-77,75988007	-939,8817749	0,07952
Cloud_Mesh_Distance_BA_R0_R3	19,33396912	-77,77243805	-939,8358154	0,0447
Cloud_Mesh_Distance_BA_R0_R3	19,57184982	-77,90856171	-939,7388306	0,018724
Cloud_Mesh_Distance_BA_R0_R3	19,4630146	-77,80373383	-939,8161011	0,03298
Cloud_Mesh_Distance_BA_R0_R3	19,70340347	-77,93650055	-939,7422485	0,009534

Cloud_Mesh_Distance_BA_R0_R3	19,84990883	-77,92806244	-939,7895508	-0,005325
Cloud_Mesh_Distance_BA_R0_R3	19,66923714	-77,82341766	-939,8329468	0,012854
Cloud_Mesh_Distance_BA_R0_R3	20,02958298	-77,97141266	-939,8150635	-0,017727
Cloud_Mesh_Distance_BA_R0_R3	19,9386673	-77,81780243	-939,9138184	-0,011246
Cloud_Mesh_Distance_BA_R0_R3	20,24099541	-77,91133881	-939,9457398	-0,027667
Cloud_Mesh_Distance_BA_R0_R3	18,85944748	-77,66205597	-939,9956665	0,08736
Cloud_Mesh_Distance_BA_R0_R3	19,07231903	-77,65904999	-939,9625244	0,067819
Cloud_Mesh_Distance_BA_R0_R3	19,05263901	-77,78359222	-939,8336182	0,068404
Cloud_Mesh_Distance_BA_R0_R3	20,04867554	-77,7588501	-940,0089722	-0,019613
Cloud_Mesh_Distance_BA_R0_R3	20,11041832	-77,88124847	-939,9192505	-0,023056
Cloud_Mesh_Distance_BA_R0_R3	19,31503296	-82,8695755	-939,3947144	-0,112712
Cloud_Mesh_Distance_BA_R0_R3	19,10808372	-82,83202362	-939,3896484	-0,120568
Cloud_Mesh_Distance_BA_R0_R3	19,51480675	-82,89519501	-939,4091797	-0,098358
Cloud_Mesh_Distance_BA_R0_R3	19,46924973	-82,75051117	-939,3399658	-0,095319
Cloud_Mesh_Distance_BA_R0_R3	19,70126915	-82,90869904	-939,4281006	-0,084524
Cloud_Mesh_Distance_BA_R0_R3	20,40172195	-82,77411652	-939,5233765	-0,033315
Cloud_Mesh_Distance_BA_R0_R3	18,70100784	-82,64110565	-939,3617554	-0,125656
Cloud_Mesh_Distance_BA_R0_R3	19,03466606	-82,67350006	-939,3186646	-0,113456
Cloud_Mesh_Distance_BA_R0_R3	18,84070587	-82,53884125	-939,2820435	-0,116439
Cloud_Mesh_Distance_BA_R0_R3	19,04030609	-82,53307343	-939,256897	-0,104343
Cloud_Mesh_Distance_BA_R0_R3	19,20903778	-82,57344818	-939,2663574	-0,106122
Cloud_Mesh_Distance_BA_R0_R3	19,26067352	-82,71985626	-939,3255615	-0,108134
Cloud_Mesh_Distance_BA_R0_R3	19,634552	-82,62778473	-939,3025513	-0,081334
Cloud_Mesh_Distance_BA_R0_R3	19,81491089	-82,65335846	-939,3318481	-0,067654
Cloud_Mesh_Distance_BA_R0_R3	19,66000175	-82,77576447	-939,3623657	-0,083711
Cloud_Mesh_Distance_BA_R0_R3	19,82638168	-82,79691315	-939,390686	-0,070472
Cloud_Mesh_Distance_BA_R0_R3	20,06666946	-82,8289566	-939,4494019	-0,052563
Cloud_Mesh_Distance_BA_R0_R3	20,18283463	-82,83957672	-939,4835815	-0,047248
Cloud_Mesh_Distance_BA_R0_R3	20,28873634	-82,80358124	-939,4985962	-0,038485
Cloud_Mesh_Distance_BA_R0_R3	20,20496368	-82,70975494	-939,4315796	-0,038003
Cloud_Mesh_Distance_BA_R0_R3	20,34951782	-82,70435333	-939,4727173	-0,030456
Cloud_Mesh_Distance_BA_R0_R3	18,35396194	-82,34999847	-939,3241577	-0,125028
Cloud_Mesh_Distance_BA_R0_R3	18,56249619	-82,59391022	-939,3770142	-0,131869
Cloud_Mesh_Distance_BA_R0_R3	18,62850952	-82,51634979	-939,3162842	-0,122124
Cloud_Mesh_Distance_BA_R0_R3	18,82405663	-82,39684296	-939,2240601	-0,108179
Cloud_Mesh_Distance_BA_R0_R3	18,68723297	-82,40712738	-939,25177	-0,11117
Cloud_Mesh_Distance_BA_R0_R3	19,15375328	-82,43547821	-939,2157593	-0,09842
Cloud_Mesh_Distance_BA_R0_R3	19,43360138	-82,58342743	-939,2738648	-0,09295
Cloud_Mesh_Distance_BA_R0_R3	19,48695374	-82,45191193	-939,2324219	-0,085414
Cloud_Mesh_Distance_BA_R0_R3	20,00211334	-82,69245148	-939,3775635	-0,05311
Cloud_Mesh_Distance_BA_R0_R3	20,18204308	-82,50986481	-939,3521118	-0,03454
Cloud_Mesh_Distance_BA_R0_R3	19,97369385	-82,49672699	-939,303833	-0,048049
Cloud_Mesh_Distance_BA_R0_R3	18,11187363	-82,17511749	-939,3447266	-0,116616
Cloud_Mesh_Distance_BA_R0_R3	18,1231308	-82,10533905	-939,3049316	-0,111187
Cloud_Mesh_Distance_BA_R0_R3	18,19664383	-82,31319427	-939,3757324	-0,123159
Cloud_Mesh_Distance_BA_R0_R3	18,38392448	-82,2296524	-939,2547607	-0,109452
Cloud_Mesh_Distance_BA_R0_R3	18,49620819	-82,39429474	-939,295044	-0,118143
Cloud_Mesh_Distance_BA_R0_R3	18,74662399	-82,31073761	-939,2008667	-0,103168
Cloud_Mesh_Distance_BA_R0_R3	18,59722137	-82,27205658	-939,2136231	-0,107778
Cloud_Mesh_Distance_BA_R0_R3	19,05939293	-82,13178253	-939,1168823	-0,082046

Cloud_Mesh_Distance_BA_R0_R3	19,04235458	-82,1674881	-939,1281128	-0,084577
Cloud_Mesh_Distance_BA_R0_R3	19,1187439	-82,13388824	-939,1175537	-0,080687
Cloud_Mesh_Distance_BA_R0_R3	19,57154083	-82,3789444	-939,2157593	-0,075678
Cloud_Mesh_Distance_BA_R0_R3	19,68575287	-82,33777618	-939,2150879	-0,065371
Cloud_Mesh_Distance_BA_R0_R3	19,63993454	-82,46529388	-939,2487793	-0,072845
Cloud_Mesh_Distance_BA_R0_R3	19,7885704	-82,51796722	-939,2825928	-0,065771
Cloud_Mesh_Distance_BA_R0_R3	20,22019196	-82,36878204	-939,3178711	-0,028425
Cloud_Mesh_Distance_BA_R0_R3	20,35876465	-82,56632233	-939,4210205	-0,026496
Cloud_Mesh_Distance_BA_R0_R3	17,91880035	-82,00270844	-939,3759155	-0,119012
Cloud_Mesh_Distance_BA_R0_R3	18,03305054	-82,08585358	-939,3446655	-0,116935
Cloud_Mesh_Distance_BA_R0_R3	19,06550598	-82,10144806	-939,1083374	-0,080813
Cloud_Mesh_Distance_BA_R0_R3	20,04298019	-82,29328156	-939,2568359	-0,036191
Cloud_Mesh_Distance_BA_R0_R3	20,05320168	-82,18611908	-939,2296753	-0,032414
Cloud_Mesh_Distance_BA_R0_R3	19,89109802	-82,18225861	-939,199646	-0,0435
Cloud_Mesh_Distance_BA_R0_R3	19,87390709	-82,34105682	-939,2414551	-0,052876
Cloud_Mesh_Distance_BA_R0_R3	20,14751434	-82,26261139	-939,2695313	-0,028705
Cloud_Mesh_Distance_BA_R0_R3	20,26514053	-82,17404938	-939,2711182	-0,015739
Cloud_Mesh_Distance_BA_R0_R3	20,38705826	-82,33358002	-939,3516235	-0,017546
Cloud_Mesh_Distance_BA_R0_R3	20,73485374	-82,26815033	-939,4501953	-0,006326
Cloud_Mesh_Distance_BA_R0_R3	20,6412735	-82,14722443	-939,373291	0,001333
Cloud_Mesh_Distance_BA_R0_R3	20,9577446	-82,08776093	-939,4742432	0,006186
Cloud_Mesh_Distance_BA_R0_R3	17,77125549	-81,71511078	-939,3617554	-0,116403
Cloud_Mesh_Distance_BA_R0_R3	17,86993408	-81,69895172	-939,2857056	-0,10675
Cloud_Mesh_Distance_BA_R0_R3	17,82295609	-81,86847687	-939,383728	-0,117531
Cloud_Mesh_Distance_BA_R0_R3	17,93120766	-81,90799713	-939,3253174	-0,114423
Cloud_Mesh_Distance_BA_R0_R3	17,96577835	-81,81612396	-939,2677612	-0,103633
Cloud_Mesh_Distance_BA_R0_R3	18,02130699	-81,94425201	-939,2867432	-0,109308
Cloud_Mesh_Distance_BA_R0_R3	20,56648636	-82,00656891	-939,3045044	0,009369
Cloud_Mesh_Distance_BA_R0_R3	20,39352226	-82,00775909	-939,2571411	-0,000265
Cloud_Mesh_Distance_BA_R0_R3	20,45009995	-82,13581085	-939,3083496	-0,004324
Cloud_Mesh_Distance_BA_R0_R3	20,71145248	-81,97447968	-939,341919	0,014343
Cloud_Mesh_Distance_BA_R0_R3	21,04148674	-81,96295929	-939,4649658	0,014781
Cloud_Mesh_Distance_BA_R0_R3	20,81580353	-82,14040375	-939,4360962	0,002818
Cloud_Mesh_Distance_BA_R0_R3	17,78662872	-81,60019684	-939,3130493	-0,112197
Cloud_Mesh_Distance_BA_R0_R3	20,80616379	-81,84528351	-939,3358765	0,023445
Cloud_Mesh_Distance_BA_R0_R3	20,88087654	-81,97063446	-939,4024658	0,016688
Cloud_Mesh_Distance_BA_R0_R3	21,15173912	-81,90894318	-939,4929199	0,016632
Cloud_Mesh_Distance_BA_R0_R3	21,10440826	-81,81816864	-939,4421997	0,024503
Cloud_Mesh_Distance_BA_R0_R3	17,63269997	-81,21388245	-939,3553467	-0,121816
Cloud_Mesh_Distance_BA_R0_R3	17,64235687	-81,3564682	-939,3710327	-0,120809
Cloud_Mesh_Distance_BA_R0_R3	17,79693985	-81,51494598	-939,282959	-0,106553
Cloud_Mesh_Distance_BA_R0_R3	20,9679985	-81,77179718	-939,3734741	0,029011
Cloud_Mesh_Distance_BA_R0_R3	20,88240051	-81,71555328	-939,3267212	0,031064
Cloud_Mesh_Distance_BA_R0_R3	21,24050903	-81,81830597	-939,5020752	0,019969
Cloud_Mesh_Distance_BA_R0_R3	21,29999542	-81,66347504	-939,4818115	0,025892
Cloud_Mesh_Distance_BA_R0_R3	21,67065811	-81,65254974	-939,675354	-0,003906
Cloud_Mesh_Distance_BA_R0_R3	21,6057663	-81,60709381	-939,6224976	0,00529
Cloud_Mesh_Distance_BA_R0_R3	21,15824127	-81,48749542	-939,3795776	0,029885
Cloud_Mesh_Distance_BA_R0_R3	21,21008492	-81,38831329	-939,3847046	0,024656
Cloud_Mesh_Distance_BA_R0_R3	21,12971497	-81,62854767	-939,3979492	0,03228

Cloud_Mesh_Distance_BA_R0_R3	21,29302597	-81,46773529	-939,4332886	0,026095
Cloud_Mesh_Distance_BA_R0_R3	21,43415833	-81,5036087	-939,5066528	0,018934
Cloud_Mesh_Distance_BA_R0_R3	21,48232651	-81,62827301	-939,560791	0,014444
Cloud_Mesh_Distance_BA_R0_R3	21,75217819	-81,4046402	-939,661438	-0,016151
Cloud_Mesh_Distance_BA_R0_R3	17,51863861	-80,92848206	-939,4246216	-0,128812
Cloud_Mesh_Distance_BA_R0_R3	17,62939835	-81,02774048	-939,3375244	-0,126696
Cloud_Mesh_Distance_BA_R0_R3	21,32036972	-81,21578217	-939,4109497	0,007698
Cloud_Mesh_Distance_BA_R0_R3	21,46894836	-81,29153442	-939,4885864	0,004268
Cloud_Mesh_Distance_BA_R0_R3	21,65963364	-81,31504059	-939,5911255	-0,008641
Cloud_Mesh_Distance_BA_R0_R3	21,5937767	-81,47058868	-939,5822754	0,005669
Cloud_Mesh_Distance_BA_R0_R3	17,51697922	-80,75402069	-939,4133911	-0,126635
Cloud_Mesh_Distance_BA_R0_R3	17,62873077	-80,82814789	-939,326416	-0,12694
Cloud_Mesh_Distance_BA_R0_R3	21,3634243	-81,08000946	-939,4195557	-0,007578
Cloud_Mesh_Distance_BA_R0_R3	21,53927612	-81,08420563	-939,5037842	-0,0176
Cloud_Mesh_Distance_BA_R0_R3	17,61197853	-80,61210632	-939,3328247	-0,123419
Cloud_Mesh_Distance_BA_R0_R3	17,52602005	-80,41892242	-939,3994751	-0,110561
Cloud_Mesh_Distance_BA_R0_R3	17,65503311	-80,43000031	-939,3035278	-0,114355
Cloud_Mesh_Distance_BA_R0_R3	17,75040817	-80,71948242	-939,2387695	-0,124986
Cloud_Mesh_Distance_BA_R0_R3	21,10558128	-80,87549591	-939,302063	-0,014247
Cloud_Mesh_Distance_BA_R0_R3	21,11380768	-80,96671295	-939,3101807	-0,007597
Cloud_Mesh_Distance_BA_R0_R3	21,04398918	-80,91221619	-939,2816773	-0,010694
Cloud_Mesh_Distance_BA_R0_R3	21,61445618	-80,98925018	-939,5340576	-0,030976
Cloud_Mesh_Distance_BA_R0_R3	21,59379959	-80,91673279	-939,519043	-0,034762
Cloud_Mesh_Distance_BA_R0_R3	21,64392853	-81,0672226	-939,5559082	-0,026869
Cloud_Mesh_Distance_BA_R0_R3	21,87985229	-80,74375153	-939,6646118	-0,069263
Cloud_Mesh_Distance_BA_R0_R3	20,99642181	-80,84468079	-939,2599487	-0,013649
Cloud_Mesh_Distance_BA_R0_R3	21,29711914	-80,72229004	-939,3685303	-0,024256
Cloud_Mesh_Distance_BA_R0_R3	21,24424362	-80,90532684	-939,357666	-0,014668
Cloud_Mesh_Distance_BA_R0_R3	21,49411964	-80,71604156	-939,4555054	-0,033395
Cloud_Mesh_Distance_BA_R0_R3	21,47319412	-80,9058609	-939,4588013	-0,026563
Cloud_Mesh_Distance_BA_R0_R3	21,72327232	-80,89014435	-939,5848999	-0,047559
Cloud_Mesh_Distance_BA_R0_R3	21,74712753	-80,78183746	-939,5900879	-0,054051
Cloud_Mesh_Distance_BA_R0_R3	21,65259743	-80,66179657	-939,5321655	-0,046589
Cloud_Mesh_Distance_BA_R0_R3	21,86021423	-80,61048126	-939,6463013	-0,069654
Cloud_Mesh_Distance_BA_R0_R3	21,781353	-80,67215729	-939,602478	-0,059646
Cloud_Mesh_Distance_BA_R0_R3	17,41337204	-80,30913544	-939,4989624	-0,091892
Cloud_Mesh_Distance_BA_R0_R3	17,63723373	-80,25180054	-939,3252564	-0,102547
Cloud_Mesh_Distance_BA_R0_R3	17,5121994	-80,23357391	-939,4195557	-0,090307
Cloud_Mesh_Distance_BA_R0_R3	21,53649902	-80,45830536	-939,4656372	-0,038388
Cloud_Mesh_Distance_BA_R0_R3	21,52124405	-80,56726837	-939,4614868	-0,037241
Cloud_Mesh_Distance_BA_R0_R3	21,89286423	-80,45653534	-939,6645508	-0,073057
Cloud_Mesh_Distance_BA_R0_R3	17,56217575	-79,86249542	-939,4378052	-0,063367
Cloud_Mesh_Distance_BA_R0_R3	17,56417465	-80,06281281	-939,3977661	-0,076325
Cloud_Mesh_Distance_BA_R0_R3	21,60060501	-80,39625549	-939,498291	-0,040347
Cloud_Mesh_Distance_BA_R0_R3	21,59477806	-80,30522156	-939,4971924	-0,038238
Cloud_Mesh_Distance_BA_R0_R3	21,63362885	-80,49834442	-939,5151367	-0,046301
Cloud_Mesh_Distance_BA_R0_R3	21,75940323	-80,54419708	-939,5858765	-0,061418
Cloud_Mesh_Distance_BA_R0_R3	17,56118774	-79,73932648	-939,4732666	-0,052851
Cloud_Mesh_Distance_BA_R0_R3	17,67729187	-79,72988129	-939,3911743	-0,059798
Cloud_Mesh_Distance_BA_R0_R3	17,69099808	-79,92711639	-939,3324585	-0,073116

Cloud_Mesh_Distance_BA_R0_R3	21,39115715	-80,16031647	-939,407959	-0,024855
Cloud_Mesh_Distance_BA_R0_R3	21,50628281	-80,23326111	-939,4562378	-0,032902
Cloud_Mesh_Distance_BA_R0_R3	21,50644302	-80,0787735	-939,472168	-0,030524
Cloud_Mesh_Distance_BA_R0_R3	21,62496567	-80,17765045	-939,5222778	-0,042602
Cloud_Mesh_Distance_BA_R0_R3	21,72052383	-80,34731293	-939,564148	-0,050848
Cloud_Mesh_Distance_BA_R0_R3	21,85882187	-80,09067535	-939,6685181	-0,060668
Cloud_Mesh_Distance_BA_R0_R3	17,45457649	-79,76599884	-939,5527344	-0,044604
Cloud_Mesh_Distance_BA_R0_R3	17,72272491	-79,56059265	-939,4172974	-0,053821
Cloud_Mesh_Distance_BA_R0_R3	17,56087112	-79,59520721	-939,5237427	-0,047151
Cloud_Mesh_Distance_BA_R0_R3	21,35846519	-79,78614044	-939,453186	-0,00802
Cloud_Mesh_Distance_BA_R0_R3	21,35159874	-79,91591644	-939,4233398	-0,014127
Cloud_Mesh_Distance_BA_R0_R3	21,38583565	-80,02462769	-939,4208984	-0,020254
Cloud_Mesh_Distance_BA_R0_R3	21,46339035	-79,91158295	-939,4770508	-0,022891
Cloud_Mesh_Distance_BA_R0_R3	21,76951981	-79,876091	-939,6508789	-0,047002
Cloud_Mesh_Distance_BA_R0_R3	21,64253998	-79,9562912	-939,562561	-0,039313
Cloud_Mesh_Distance_BA_R0_R3	21,78758621	-79,99949646	-939,6386108	-0,050963
Cloud_Mesh_Distance_BA_R0_R3	21,75509644	-80,13715363	-939,6000366	-0,054146
Cloud_Mesh_Distance_BA_R0_R3	17,83970261	-79,48934174	-939,3690186	-0,056715
Cloud_Mesh_Distance_BA_R0_R3	17,79201126	-79,35718536	-939,4554443	-0,051264
Cloud_Mesh_Distance_BA_R0_R3	17,76802063	-79,43830872	-939,4353638	-0,051644
Cloud_Mesh_Distance_BA_R0_R3	17,88429642	-79,35461426	-939,397583	-0,054804
Cloud_Mesh_Distance_BA_R0_R3	21,66070938	-79,59654999	-939,6608276	-0,02055
Cloud_Mesh_Distance_BA_R0_R3	21,68439484	-79,76920319	-939,6254883	-0,034375
Cloud_Mesh_Distance_BA_R0_R3	17,80925751	-79,25188446	-939,4932251	-0,047315
Cloud_Mesh_Distance_BA_R0_R3	17,95171738	-79,20026398	-939,425415	-0,049986
Cloud_Mesh_Distance_BA_R0_R3	18,07226563	-79,1335144	-939,3859253	-0,048925
Cloud_Mesh_Distance_BA_R0_R3	21,35978317	-79,46399689	-939,5447388	0,00872
Cloud_Mesh_Distance_BA_R0_R3	21,34200668	-79,63616943	-939,4837036	-0,000705
Cloud_Mesh_Distance_BA_R0_R3	21,50917053	-79,7015152	-939,5480347	-0,015619
Cloud_Mesh_Distance_BA_R0_R3	18,00815201	-79,05994415	-939,4573975	-0,045426
Cloud_Mesh_Distance_BA_R0_R3	17,917099	-78,97325897	-939,5622559	-0,035532
Cloud_Mesh_Distance_BA_R0_R3	21,08019638	-79,33434296	-939,4656982	0,025771
Cloud_Mesh_Distance_BA_R0_R3	21,21670532	-79,2742691	-939,5452881	0,024237
Cloud_Mesh_Distance_BA_R0_R3	21,34461021	-79,30968475	-939,5914307	0,016835
Cloud_Mesh_Distance_BA_R0_R3	21,52528763	-79,49771881	-939,6174316	-0,003761
Cloud_Mesh_Distance_BA_R0_R3	21,62161255	-79,40477753	-939,7049561	-0,007884
Cloud_Mesh_Distance_BA_R0_R3	21,4613266	-79,32657623	-939,6453857	0,007826
Cloud_Mesh_Distance_BA_R0_R3	18,00896835	-78,80619049	-939,5899658	-0,026416
Cloud_Mesh_Distance_BA_R0_R3	18,08158875	-78,9078598	-939,4873047	-0,030388
Cloud_Mesh_Distance_BA_R0_R3	18,19403839	-78,90399933	-939,4238281	-0,030797
Cloud_Mesh_Distance_BA_R0_R3	18,18735504	-78,81787109	-939,4696045	-0,023182
Cloud_Mesh_Distance_BA_R0_R3	20,50826645	-78,90200043	-939,4474487	0,00913
Cloud_Mesh_Distance_BA_R0_R3	20,5848999	-79,01807404	-939,4213257	0,013828
Cloud_Mesh_Distance_BA_R0_R3	20,73990631	-79,0840683	-939,4403076	0,024468
Cloud_Mesh_Distance_BA_R0_R3	20,87633324	-78,99933624	-939,519043	0,035444
Cloud_Mesh_Distance_BA_R0_R3	21,0265255	-79,15494537	-939,5110474	0,034231
Cloud_Mesh_Distance_BA_R0_R3	21,01470184	-79,02780151	-939,5576172	0,039582
Cloud_Mesh_Distance_BA_R0_R3	21,3348465	-79,17655182	-939,6392822	0,024563
Cloud_Mesh_Distance_BA_R0_R3	21,5652771	-79,25501251	-939,7329102	0,007495
Cloud_Mesh_Distance_BA_R0_R3	18,12413025	-78,77890778	-939,5276489	-0,019173

Cloud_Mesh_Distance_BA_R0_R3	18,0998764	-78,68852997	-939,5934448	-0,010612
Cloud_Mesh_Distance_BA_R0_R3	18,49084091	-78,59889221	-939,4263306	0,005525
Cloud_Mesh_Distance_BA_R0_R3	18,29125214	-78,82110596	-939,4127808	-0,02329
Cloud_Mesh_Distance_BA_R0_R3	20,38828278	-78,84369659	-939,4384155	-0,002076
Cloud_Mesh_Distance_BA_R0_R3	20,65244865	-78,78207397	-939,5427856	0,018663
Cloud_Mesh_Distance_BA_R0_R3	20,66489029	-78,9026413	-939,4925537	0,021631
Cloud_Mesh_Distance_BA_R0_R3	21,15812492	-78,91997528	-939,6675415	0,042867
Cloud_Mesh_Distance_BA_R0_R3	21,00341797	-78,90098572	-939,6091919	0,04023
Cloud_Mesh_Distance_BA_R0_R3	21,15026474	-79,06331635	-939,5998535	0,038065
Cloud_Mesh_Distance_BA_R0_R3	21,28547287	-79,00574493	-939,6903076	0,035763
Cloud_Mesh_Distance_BA_R0_R3	18,55161285	-78,6304245	-939,3896484	0,001879
Cloud_Mesh_Distance_BA_R0_R3	19,99797821	-78,55998993	-939,4499512	-0,018745
Cloud_Mesh_Distance_BA_R0_R3	20,11015701	-78,46260834	-939,5349121	-0,021066
Cloud_Mesh_Distance_BA_R0_R3	20,49930954	-78,60330963	-939,5847778	0,003005
Cloud_Mesh_Distance_BA_R0_R3	20,49933052	-78,75215149	-939,512085	0,004577
Cloud_Mesh_Distance_BA_R0_R3	20,76477051	-78,69688416	-939,6202393	0,030892
Cloud_Mesh_Distance_BA_R0_R3	20,79567719	-78,79821777	-939,5809326	0,031152
Cloud_Mesh_Distance_BA_R0_R3	20,82300949	-78,7117691	-939,6333008	0,035097
Cloud_Mesh_Distance_BA_R0_R3	20,92440033	-78,72985077	-939,6618042	0,040991
Cloud_Mesh_Distance_BA_R0_R3	21,09336281	-78,78925323	-939,7035523	0,047529
Cloud_Mesh_Distance_BA_R0_R3	18,52323914	-78,3309021	-939,5618286	0,038798
Cloud_Mesh_Distance_BA_R0_R3	18,80165863	-78,19086456	-939,5578003	0,050521
Cloud_Mesh_Distance_BA_R0_R3	18,63969803	-78,37285614	-939,4924316	0,035346
Cloud_Mesh_Distance_BA_R0_R3	18,99835968	-78,31232452	-939,4517822	0,024502
Cloud_Mesh_Distance_BA_R0_R3	19,18605804	-78,32590485	-939,4326782	0,016561
Cloud_Mesh_Distance_BA_R0_R3	19,14961243	-78,19749451	-939,507019	0,032434
Cloud_Mesh_Distance_BA_R0_R3	19,9418354	-78,45343018	-939,4840088	-0,020098
Cloud_Mesh_Distance_BA_R0_R3	19,93792725	-78,31560516	-939,5571289	-0,015679
Cloud_Mesh_Distance_BA_R0_R3	20,25526047	-78,43122101	-939,5993042	-0,019856
Cloud_Mesh_Distance_BA_R0_R3	20,32072067	-78,58487701	-939,5391846	-0,012054
Cloud_Mesh_Distance_BA_R0_R3	20,41823196	-78,47342682	-939,6295776	-0,010759
Cloud_Mesh_Distance_BA_R0_R3	20,65363121	-78,68202972	-939,5923462	0,01821
Cloud_Mesh_Distance_BA_R0_R3	20,60964203	-78,52823639	-939,6616821	0,008857
Cloud_Mesh_Distance_BA_R0_R3	20,75705338	-78,62288666	-939,6574097	0,027811
Cloud_Mesh_Distance_BA_R0_R3	20,75268936	-78,52184296	-939,7158814	0,020023
Cloud_Mesh_Distance_BA_R0_R3	20,88263321	-78,59529877	-939,7213745	0,03401
Cloud_Mesh_Distance_BA_R0_R3	18,96103668	-78,17058563	-939,5407715	0,045355
Cloud_Mesh_Distance_BA_R0_R3	19,31770325	-78,22303009	-939,4910889	0,020653
Cloud_Mesh_Distance_BA_R0_R3	19,33775711	-78,32909393	-939,4335938	0,006558
Cloud_Mesh_Distance_BA_R0_R3	19,28860092	-78,11605072	-939,5574951	0,029265
Cloud_Mesh_Distance_BA_R0_R3	19,75379944	-78,16747284	-939,5914917	-0,001316
Cloud_Mesh_Distance_BA_R0_R3	19,48694611	-78,12601471	-939,5656738	0,018948
Cloud_Mesh_Distance_BA_R0_R3	19,76556396	-78,35413361	-939,4851685	-0,006263
Cloud_Mesh_Distance_BA_R0_R3	20,10722351	-78,32789612	-939,6069946	-0,021347
Cloud_Mesh_Distance_BA_R0_R3	20,2590065	-78,21652985	-939,7290649	-0,024685
Cloud_Mesh_Distance_BA_R0_R3	20,16670227	-78,17988586	-939,7174683	-0,024578
Cloud_Mesh_Distance_BA_R0_R3	18,98478317	-78,05596161	-939,6152954	0,054182
Cloud_Mesh_Distance_BA_R0_R3	19,13955688	-78,05953217	-939,5971069	0,045593
Cloud_Mesh_Distance_BA_R0_R3	19,33876038	-77,99490356	-939,6437988	0,036114
Cloud_Mesh_Distance_BA_R0_R3	19,4978447	-77,98683929	-939,6652222	0,025297

Cloud_Mesh_Distance_BA_R0_R3	19,77202606	-78,02206421	-939,6943359	0,001758
Cloud_Mesh_Distance_BA_R0_R3	19,91070938	-78,18444061	-939,6257935	-0,015535
Cloud_Mesh_Distance_BA_R0_R3	19,95022202	-78,00927734	-939,7582398	-0,012346
Cloud_Mesh_Distance_BA_R0_R3	20,01007843	-78,06619263	-939,7375488	-0,016086
Cloud_Mesh_Distance_BA_R0_R3	20,0428009	-78,18698883	-939,668396	-0,019473
Cloud_Mesh_Distance_BA_R0_R3	20,23111534	-78,13724518	-939,7702637	-0,024323
Cloud_Mesh_Distance_BA_R0_R3	20,11687088	-78,09320068	-939,7576904	-0,022627
Cloud_Mesh_Distance_BA_R0_R3	19,19440079	-77,94532776	-939,6801148	0,05119
Cloud_Mesh_Distance_BA_R0_R3	19,4389267	-77,90692902	-939,7210083	0,033672
Cloud_Mesh_Distance_BA_R0_R3	19,30420303	-77,86025238	-939,7523193	0,042656
Cloud_Mesh_Distance_BA_R0_R3	19,90119553	-78,07761383	-939,6935425	-0,010624
Cloud_Mesh_Distance_BA_R0_R3	18,96951294	-82,41680145	-939,2160645	-0,09924
Cloud_Mesh_Distance_BA_R0_R3	19,33714104	-82,45905304	-939,2260742	-0,089208
Cloud_Mesh_Distance_BA_R0_R3	19,28955841	-82,32413483	-939,1794434	-0,083961
Cloud_Mesh_Distance_BA_R0_R3	19,08259583	-82,2707901	-939,1600952	-0,091958
Cloud_Mesh_Distance_BA_R0_R3	19,45346069	-82,35459137	-939,1981812	-0,082222
Cloud_Mesh_Distance_BA_R0_R3	18,53488922	-82,10709381	-939,1630859	-0,09645
Cloud_Mesh_Distance_BA_R0_R3	18,74882889	-82,17063141	-939,1491699	-0,091972
Cloud_Mesh_Distance_BA_R0_R3	18,88394547	-82,2939682	-939,1784668	-0,09658
Cloud_Mesh_Distance_BA_R0_R3	18,94094658	-82,1724472	-939,1335449	-0,088081
Cloud_Mesh_Distance_BA_R0_R3	19,268013	-82,17584991	-939,133667	-0,076686
Cloud_Mesh_Distance_BA_R0_R3	19,59072876	-82,27259064	-939,18573	-0,06871
Cloud_Mesh_Distance_BA_R0_R3	19,44517517	-82,21692657	-939,15625	-0,075343
Cloud_Mesh_Distance_BA_R0_R3	20,02441788	-82,3673172	-939,274292	-0,040267
Cloud_Mesh_Distance_BA_R0_R3	20,11463928	-82,39577484	-939,3012085	-0,036346
Cloud_Mesh_Distance_BA_R0_R3	20,16765213	-82,32486725	-939,2921143	-0,029898
Cloud_Mesh_Distance_BA_R0_R3	20,23939896	-82,29064178	-939,298584	-0,022674
Cloud_Mesh_Distance_BA_R0_R3	18,14098358	-82,02094269	-939,2588501	-0,107901
Cloud_Mesh_Distance_BA_R0_R3	18,21044159	-82,14337921	-939,2824097	-0,111959
Cloud_Mesh_Distance_BA_R0_R3	18,32155228	-82,0157547	-939,1864624	-0,098279
Cloud_Mesh_Distance_BA_R0_R3	18,15330696	-81,82846832	-939,180603	-0,091719
Cloud_Mesh_Distance_BA_R0_R3	18,67031288	-82,013237	-939,1080933	-0,083935
Cloud_Mesh_Distance_BA_R0_R3	19,0104084	-82,1032486	-939,1095581	-0,081241
Cloud_Mesh_Distance_BA_R0_R3	18,99491119	-81,94437408	-939,0665283	-0,07536
Cloud_Mesh_Distance_BA_R0_R3	18,88236237	-82,00089264	-939,0843506	-0,078215
Cloud_Mesh_Distance_BA_R0_R3	19,0730381	-82,01651764	-939,0841065	-0,076358
Cloud_Mesh_Distance_BA_R0_R3	19,24055672	-82,03647614	-939,0935059	-0,071201
Cloud_Mesh_Distance_BA_R0_R3	19,20530128	-81,90583038	-939,05896	-0,065326
Cloud_Mesh_Distance_BA_R0_R3	19,81227684	-82,10559845	-939,1680298	-0,048264
Cloud_Mesh_Distance_BA_R0_R3	19,74914169	-82,22516632	-939,190979	-0,055476
Cloud_Mesh_Distance_BA_R0_R3	20,10253143	-82,32480621	-939,2781982	-0,034057
Cloud_Mesh_Distance_BA_R0_R3	18,0173645	-81,64286041	-939,1849976	-0,092031
Cloud_Mesh_Distance_BA_R0_R3	18,29630089	-81,67285919	-939,0892334	-0,078848
Cloud_Mesh_Distance_BA_R0_R3	18,48968506	-81,75647736	-939,0651856	-0,076575
Cloud_Mesh_Distance_BA_R0_R3	18,34939957	-81,84116364	-939,1184692	-0,085392
Cloud_Mesh_Distance_BA_R0_R3	18,69205856	-81,84679413	-939,0588989	-0,076732
Cloud_Mesh_Distance_BA_R0_R3	18,50618362	-81,93100739	-939,1096191	-0,086207
Cloud_Mesh_Distance_BA_R0_R3	19,34695816	-81,91928864	-939,0702515	-0,060932
Cloud_Mesh_Distance_BA_R0_R3	19,47421265	-81,8935318	-939,0750732	-0,055255
Cloud_Mesh_Distance_BA_R0_R3	19,43214035	-82,0427475	-939,1077271	-0,064863

Cloud_Mesh_Distance_BA_R0_R3	19,63758659	-82,10813141	-939,1456299	-0,057744
Cloud_Mesh_Distance_BA_R0_R3	19,82023621	-81,9535141	-939,1309204	-0,040018
Cloud_Mesh_Distance_BA_R0_R3	20,02664375	-81,90097809	-939,1519775	-0,022611
Cloud_Mesh_Distance_BA_R0_R3	19,9636631	-82,05554962	-939,1782837	-0,032514
Cloud_Mesh_Distance_BA_R0_R3	20,17923546	-82,00052643	-939,2056274	-0,015059
Cloud_Mesh_Distance_BA_R0_R3	20,66906738	-81,88892365	-939,3031616	0,018765
Cloud_Mesh_Distance_BA_R0_R3	18,15390396	-81,66501617	-939,1317139	-0,083761
Cloud_Mesh_Distance_BA_R0_R3	18,46956635	-81,58478546	-939,0335693	-0,07372
Cloud_Mesh_Distance_BA_R0_R3	18,32846069	-81,51386261	-939,0498047	-0,074152
Cloud_Mesh_Distance_BA_R0_R3	18,64915657	-81,65836334	-939,022583	-0,070695
Cloud_Mesh_Distance_BA_R0_R3	18,8633728	-81,83444977	-939,0436401	-0,071209
Cloud_Mesh_Distance_BA_R0_R3	18,80309296	-81,72245026	-939,0227661	-0,06768
Cloud_Mesh_Distance_BA_R0_R3	19,41844559	-81,80992889	-939,0509644	-0,05279
Cloud_Mesh_Distance_BA_R0_R3	20,19208145	-81,81937408	-939,1638184	-0,006953
Cloud_Mesh_Distance_BA_R0_R3	20,1744194	-81,70296478	-939,1357422	-0,005975
Cloud_Mesh_Distance_BA_R0_R3	20,34255981	-81,66791534	-939,1645508	0,005551
Cloud_Mesh_Distance_BA_R0_R3	20,36375999	-81,84734344	-939,2089233	0,003525
Cloud_Mesh_Distance_BA_R0_R3	20,54200363	-81,86785126	-939,260376	0,013361
Cloud_Mesh_Distance_BA_R0_R3	20,72571945	-81,73636627	-939,2807617	0,026108
Cloud_Mesh_Distance_BA_R0_R3	17,73273468	-81,31233215	-939,2901001	-0,116407
Cloud_Mesh_Distance_BA_R0_R3	17,82133484	-81,42420197	-939,2462769	-0,101908
Cloud_Mesh_Distance_BA_R0_R3	17,87468338	-81,55002594	-939,2393189	-0,098572
Cloud_Mesh_Distance_BA_R0_R3	18,12866211	-81,32025909	-939,0809326	-0,083921
Cloud_Mesh_Distance_BA_R0_R3	18,00063705	-81,21504974	-939,1208496	-0,09372
Cloud_Mesh_Distance_BA_R0_R3	17,97503662	-81,41956329	-939,1574707	-0,090532
Cloud_Mesh_Distance_BA_R0_R3	18,16370392	-81,51644135	-939,0964966	-0,080559
Cloud_Mesh_Distance_BA_R0_R3	19,81319237	-81,46738434	-939,0344238	-0,028087
Cloud_Mesh_Distance_BA_R0_R3	20,21310425	-81,57076263	-939,1190796	-0,002563
Cloud_Mesh_Distance_BA_R0_R3	20,53951263	-81,70110321	-939,2197266	0,016926
Cloud_Mesh_Distance_BA_R0_R3	20,992836	-81,64707184	-939,3492432	0,033538
Cloud_Mesh_Distance_BA_R0_R3	20,91152191	-81,57169342	-939,3044434	0,032055
Cloud_Mesh_Distance_BA_R0_R3	20,72364807	-81,54653168	-939,2410889	0,024668
Cloud_Mesh_Distance_BA_R0_R3	17,85871887	-81,26018524	-939,1980591	-0,101364
Cloud_Mesh_Distance_BA_R0_R3	17,7481823	-81,13295746	-939,2573242	-0,113203
Cloud_Mesh_Distance_BA_R0_R3	18,13454247	-81,15520477	-939,0663452	-0,093369
Cloud_Mesh_Distance_BA_R0_R3	18,24573517	-81,23282623	-939,0383301	-0,089914
Cloud_Mesh_Distance_BA_R0_R3	19,7624073	-81,42394257	-939,0196533	-0,029857
Cloud_Mesh_Distance_BA_R0_R3	19,87213898	-81,469841	-939,0431519	-0,023616
Cloud_Mesh_Distance_BA_R0_R3	20,38111496	-81,48514557	-939,1412354	0,006369
Cloud_Mesh_Distance_BA_R0_R3	20,34646225	-81,2748642	-939,1062012	-0,001675
Cloud_Mesh_Distance_BA_R0_R3	20,53847122	-81,52161407	-939,185669	0,015272
Cloud_Mesh_Distance_BA_R0_R3	20,52388191	-81,3650589	-939,1594849	0,008541
Cloud_Mesh_Distance_BA_R0_R3	21,05762863	-81,5487442	-939,352478	0,032385
Cloud_Mesh_Distance_BA_R0_R3	21,07269669	-81,41538239	-939,3345947	0,028184
Cloud_Mesh_Distance_BA_R0_R3	17,75614548	-80,92993927	-939,239502	-0,120162
Cloud_Mesh_Distance_BA_R0_R3	17,8986969	-81,05321503	-939,15979	-0,108942
Cloud_Mesh_Distance_BA_R0_R3	18,03471947	-81,07410431	-939,0986328	-0,101211
Cloud_Mesh_Distance_BA_R0_R3	18,0197773	-80,93482208	-939,100708	-0,109203
Cloud_Mesh_Distance_BA_R0_R3	18,99498749	-81,17577362	-938,9475708	-0,075881
Cloud_Mesh_Distance_BA_R0_R3	19,00224304	-81,14124298	-938,9442749	-0,076535

Cloud_Mesh_Distance_BA_R0_R3	19,05696106	-81,18778229	-938,9468994	-0,071657
Cloud_Mesh_Distance_BA_R0_R3	20,39991951	-81,35683441	-939,1281128	0,003712
Cloud_Mesh_Distance_BA_R0_R3	20,62405014	-81,23090363	-939,1707153	0,006802
Cloud_Mesh_Distance_BA_R0_R3	20,74798775	-81,19412231	-939,203125	0,00755
Cloud_Mesh_Distance_BA_R0_R3	20,7114296	-81,34888458	-939,2076416	0,016604
Cloud_Mesh_Distance_BA_R0_R3	20,89774704	-81,39054108	-939,2703247	0,024289
Cloud_Mesh_Distance_BA_R0_R3	21,19099808	-81,26351929	-939,361023	0,01602
Cloud_Mesh_Distance_BA_R0_R3	21,30315781	-81,32233429	-939,414917	0,017692
Cloud_Mesh_Distance_BA_R0_R3	18,0319252	-80,79969788	-939,0958252	-0,116686
Cloud_Mesh_Distance_BA_R0_R3	17,90121078	-80,81629181	-939,1533203	-0,119206
Cloud_Mesh_Distance_BA_R0_R3	18,00342178	-80,66414642	-939,107605	-0,120117
Cloud_Mesh_Distance_BA_R0_R3	18,49319077	-80,73265839	-938,9768066	-0,117072
Cloud_Mesh_Distance_BA_R0_R3	18,52832031	-80,81006622	-938,9728394	-0,113288
Cloud_Mesh_Distance_BA_R0_R3	18,56636047	-80,7261734	-938,9641113	-0,115629
Cloud_Mesh_Distance_BA_R0_R3	20,40648842	-80,83235168	-939,0756836	-0,01208
Cloud_Mesh_Distance_BA_R0_R3	20,94767952	-80,98680878	-939,2520142	-0,005035
Cloud_Mesh_Distance_BA_R0_R3	21,03794098	-81,27309418	-939,3045044	0,017875
Cloud_Mesh_Distance_BA_R0_R3	20,89681625	-81,19547272	-939,2490234	0,01104
Cloud_Mesh_Distance_BA_R0_R3	21,09901047	-81,11803436	-939,3132324	0,005585
Cloud_Mesh_Distance_BA_R0_R3	21,24433517	-81,13844299	-939,3722534	0,003707
Cloud_Mesh_Distance_BA_R0_R3	17,74855423	-80,52437592	-939,2404785	-0,119828
Cloud_Mesh_Distance_BA_R0_R3	17,87199974	-80,60424042	-939,1694336	-0,119662
Cloud_Mesh_Distance_BA_R0_R3	18,42857552	-80,69094086	-938,987854	-0,119785
Cloud_Mesh_Distance_BA_R0_R3	18,61595917	-80,65775299	-938,9526978	-0,114033
Cloud_Mesh_Distance_BA_R0_R3	18,51889038	-80,6528244	-938,9696655	-0,116528
Cloud_Mesh_Distance_BA_R0_R3	18,67663193	-80,76674652	-938,9486694	-0,109434
Cloud_Mesh_Distance_BA_R0_R3	20,4834938	-80,85831451	-939,0995483	-0,012167
Cloud_Mesh_Distance_BA_R0_R3	20,50127411	-80,79175568	-939,0988159	-0,01264
Cloud_Mesh_Distance_BA_R0_R3	20,57748413	-80,72853851	-939,1156006	-0,012808
Cloud_Mesh_Distance_BA_R0_R3	20,61019135	-80,83571625	-939,1342773	-0,012598
Cloud_Mesh_Distance_BA_R0_R3	20,5067215	-80,93261719	-939,1126099	-0,011116
Cloud_Mesh_Distance_BA_R0_R3	20,59236908	-80,92056274	-939,1361694	-0,010909
Cloud_Mesh_Distance_BA_R0_R3	21,21792793	-81,03690338	-939,3544922	-0,004769
Cloud_Mesh_Distance_BA_R0_R3	17,76005554	-80,34506989	-939,239563	-0,107738
Cloud_Mesh_Distance_BA_R0_R3	18,12482262	-80,41030121	-939,0664063	-0,118105
Cloud_Mesh_Distance_BA_R0_R3	18,05566978	-80,30562592	-939,0962524	-0,112992
Cloud_Mesh_Distance_BA_R0_R3	17,85440826	-80,43560028	-939,1826782	-0,119197
Cloud_Mesh_Distance_BA_R0_R3	17,99020386	-80,4620285	-939,1174316	-0,117265
Cloud_Mesh_Distance_BA_R0_R3	18,12854195	-80,57233429	-939,0622559	-0,120261
Cloud_Mesh_Distance_BA_R0_R3	20,81411743	-80,84893036	-939,1983032	-0,012709
Cloud_Mesh_Distance_BA_R0_R3	21,11632156	-80,77645874	-939,2996826	-0,020781
Cloud_Mesh_Distance_BA_R0_R3	21,24281693	-80,52727509	-939,3364868	-0,024912
Cloud_Mesh_Distance_BA_R0_R3	21,10978317	-80,63323212	-939,2889404	-0,021478
Cloud_Mesh_Distance_BA_R0_R3	17,76599503	-80,1889267	-939,2476196	-0,098934
Cloud_Mesh_Distance_BA_R0_R3	17,83745384	-80,01911163	-939,229187	-0,090161
Cloud_Mesh_Distance_BA_R0_R3	17,69629669	-80,0893631	-939,3023071	-0,08823
Cloud_Mesh_Distance_BA_R0_R3	17,91389465	-80,24240875	-939,1641235	-0,108102
Cloud_Mesh_Distance_BA_R0_R3	18,00413513	-80,03443146	-939,1425781	-0,096355
Cloud_Mesh_Distance_BA_R0_R3	21,08378983	-80,51384735	-939,2729492	-0,021511
Cloud_Mesh_Distance_BA_R0_R3	21,41229057	-80,45848846	-939,4069214	-0,033502

Cloud_Mesh_Distance_BA_R0_R3	21,39366913	-80,59908295	-939,4033203	-0,029318
Cloud_Mesh_Distance_BA_R0_R3	18,11485291	-80,03116608	-939,0962524	-0,096095
Cloud_Mesh_Distance_BA_R0_R3	18,19695663	-79,97472382	-939,0737915	-0,094957
Cloud_Mesh_Distance_BA_R0_R3	20,9620018	-80,33817291	-939,2240601	-0,008567
Cloud_Mesh_Distance_BA_R0_R3	20,95125198	-80,1036911	-939,2323608	0,004285
Cloud_Mesh_Distance_BA_R0_R3	20,92471123	-80,23726654	-939,2130737	0,00072
Cloud_Mesh_Distance_BA_R0_R3	21,28308487	-80,38053131	-939,3495483	-0,023221
Cloud_Mesh_Distance_BA_R0_R3	21,12057114	-80,38132477	-939,2843628	-0,017382
Cloud_Mesh_Distance_BA_R0_R3	21,50911903	-80,36138153	-939,4525147	-0,033608
Cloud_Mesh_Distance_BA_R0_R3	21,39289093	-80,30477905	-939,3994751	-0,027124
Cloud_Mesh_Distance_BA_R0_R3	17,83394623	-79,80200958	-939,2738648	-0,073247
Cloud_Mesh_Distance_BA_R0_R3	17,98912048	-79,85765839	-939,1812744	-0,082887
Cloud_Mesh_Distance_BA_R0_R3	17,8404541	-79,61854553	-939,3231812	-0,063723
Cloud_Mesh_Distance_BA_R0_R3	18,17248917	-79,72660828	-939,1345215	-0,085836
Cloud_Mesh_Distance_BA_R0_R3	18,1172905	-79,91201019	-939,1146851	-0,092328
Cloud_Mesh_Distance_BA_R0_R3	18,24921799	-79,86406708	-939,0753784	-0,092505
Cloud_Mesh_Distance_BA_R0_R3	20,42680359	-79,95287323	-939,0872192	0,020741
Cloud_Mesh_Distance_BA_R0_R3	21,05927467	-80,21917725	-939,263794	-0,005733
Cloud_Mesh_Distance_BA_R0_R3	21,29362297	-80,08525085	-939,3719482	-0,016631
Cloud_Mesh_Distance_BA_R0_R3	21,24317551	-80,22377014	-939,3380127	-0,018281
Cloud_Mesh_Distance_BA_R0_R3	17,99732971	-79,66188812	-939,2271729	-0,078926
Cloud_Mesh_Distance_BA_R0_R3	20,45165253	-79,84236908	-939,1174927	0,021205
Cloud_Mesh_Distance_BA_R0_R3	20,53296471	-79,82500458	-939,1433716	0,021742
Cloud_Mesh_Distance_BA_R0_R3	20,51652527	-79,88926697	-939,1242676	0,022361
Cloud_Mesh_Distance_BA_R0_R3	20,93319702	-79,7522583	-939,2869873	0,014157
Cloud_Mesh_Distance_BA_R0_R3	21,28764343	-79,98451996	-939,3823853	-0,009963
Cloud_Mesh_Distance_BA_R0_R3	21,2444458	-79,86339569	-939,3851318	-0,004276
Cloud_Mesh_Distance_BA_R0_R3	21,14440536	-80,02312469	-939,3166504	-0,003461
Cloud_Mesh_Distance_BA_R0_R3	18,1813755	-79,51016998	-939,1953735	-0,079575
Cloud_Mesh_Distance_BA_R0_R3	18,20313644	-79,30770111	-939,2567749	-0,070093
Cloud_Mesh_Distance_BA_R0_R3	18,05141449	-79,26425934	-939,3417358	-0,057797
Cloud_Mesh_Distance_BA_R0_R3	17,99569511	-79,45914459	-939,2943115	-0,066332
Cloud_Mesh_Distance_BA_R0_R3	18,34616661	-79,56121063	-939,12323	-0,089613
Cloud_Mesh_Distance_BA_R0_R3	18,40117645	-79,34923553	-939,1754761	-0,083966
Cloud_Mesh_Distance_BA_R0_R3	18,5126915	-79,45773315	-939,1120606	-0,09792
Cloud_Mesh_Distance_BA_R0_R3	20,23093796	-79,42179108	-939,1863403	-0,00521
Cloud_Mesh_Distance_BA_R0_R3	20,2623558	-79,48723602	-939,1729126	-0,001377
Cloud_Mesh_Distance_BA_R0_R3	20,56993484	-79,77864838	-939,164978	0,020527
Cloud_Mesh_Distance_BA_R0_R3	20,84479523	-79,66757965	-939,2774658	0,019929
Cloud_Mesh_Distance_BA_R0_R3	21,21595764	-79,68178558	-939,414978	0,006258
Cloud_Mesh_Distance_BA_R0_R3	21,08291626	-79,78089142	-939,336731	0,006662
Cloud_Mesh_Distance_BA_R0_R3	18,39410973	-79,14266968	-939,2466431	-0,06651
Cloud_Mesh_Distance_BA_R0_R3	18,84018326	-79,11374664	-939,1668091	-0,088503
Cloud_Mesh_Distance_BA_R0_R3	20,9556675	-79,58745575	-939,3377075	0,020884
Cloud_Mesh_Distance_BA_R0_R3	20,78528595	-79,40673828	-939,3364258	0,02274
Cloud_Mesh_Distance_BA_R0_R3	21,1114006	-79,51483917	-939,4190064	0,015034
Cloud_Mesh_Distance_BA_R0_R3	20,95419121	-79,38889313	-939,3995972	0,025579
Cloud_Mesh_Distance_BA_R0_R3	21,24985504	-79,54511261	-939,4685669	0,008167
Cloud_Mesh_Distance_BA_R0_R3	21,2059021	-79,42635345	-939,4863281	0,018818
Cloud_Mesh_Distance_BA_R0_R3	21,09515762	-79,64162445	-939,3760986	0,013608

Cloud_Mesh_Distance_BA_R0_R3	18,13953781	-79,01435089	-939,4031372	-0,040539
Cloud_Mesh_Distance_BA_R0_R3	18,27054977	-78,95549774	-939,3634644	-0,037686
Cloud_Mesh_Distance_BA_R0_R3	18,21347809	-79,11830902	-939,3233643	-0,057314
Cloud_Mesh_Distance_BA_R0_R3	18,37187767	-79,0029068	-939,3036499	-0,046905
Cloud_Mesh_Distance_BA_R0_R3	18,8134346	-78,95014191	-939,2196655	-0,06509
Cloud_Mesh_Distance_BA_R0_R3	18,61645126	-79,15090942	-939,189148	-0,082184
Cloud_Mesh_Distance_BA_R0_R3	18,97588348	-79,12532806	-939,151062	-0,091942
Cloud_Mesh_Distance_BA_R0_R3	20,18975067	-79,24347687	-939,2347412	-0,013637
Cloud_Mesh_Distance_BA_R0_R3	20,1187439	-79,31197357	-939,1968384	-0,017697
Cloud_Mesh_Distance_BA_R0_R3	20,40504456	-79,33799744	-939,2543945	0,003369
Cloud_Mesh_Distance_BA_R0_R3	20,22556114	-79,33268738	-939,2132568	-0,008693
Cloud_Mesh_Distance_BA_R0_R3	20,50008011	-79,34029388	-939,2785645	0,00935
Cloud_Mesh_Distance_BA_R0_R3	20,8218956	-79,24567413	-939,4042358	0,02823
Cloud_Mesh_Distance_BA_R0_R3	20,95456505	-79,25255585	-939,4476929	0,028846
Cloud_Mesh_Distance_BA_R0_R3	20,88447571	-79,14552307	-939,4625244	0,030003
Cloud_Mesh_Distance_BA_R0_R3	18,57078171	-78,92731476	-939,2705078	-0,04683
Cloud_Mesh_Distance_BA_R0_R3	18,41343689	-78,86699677	-939,340271	-0,031261
Cloud_Mesh_Distance_BA_R0_R3	19,03433037	-78,79345703	-939,2486572	-0,049853
Cloud_Mesh_Distance_BA_R0_R3	18,79053879	-78,73847198	-939,2918701	-0,028978
Cloud_Mesh_Distance_BA_R0_R3	19,19472504	-79,00020599	-939,1795044	-0,074566
Cloud_Mesh_Distance_BA_R0_R3	19,03095245	-78,98590851	-939,1890259	-0,07724
Cloud_Mesh_Distance_BA_R0_R3	19,35671234	-78,97818756	-939,1876831	-0,064926
Cloud_Mesh_Distance_BA_R0_R3	19,5447464	-78,96826935	-939,2035523	-0,05312
Cloud_Mesh_Distance_BA_R0_R3	19,67443275	-78,82630157	-939,2666016	-0,033233
Cloud_Mesh_Distance_BA_R0_R3	20,17569733	-79,15576172	-939,2615356	-0,014425
Cloud_Mesh_Distance_BA_R0_R3	20,05288315	-79,08422089	-939,2571411	-0,023553
Cloud_Mesh_Distance_BA_R0_R3	20,20692444	-79,03407288	-939,3134155	-0,012753
Cloud_Mesh_Distance_BA_R0_R3	20,60373306	-79,20989227	-939,3528442	0,016808
Cloud_Mesh_Distance_BA_R0_R3	20,34086609	-79,22307587	-939,2786865	-0,000771
Cloud_Mesh_Distance_BA_R0_R3	18,43828201	-78,70584106	-939,3978272	-0,006781
Cloud_Mesh_Distance_BA_R0_R3	18,58045578	-78,6958847	-939,3530884	-0,009471
Cloud_Mesh_Distance_BA_R0_R3	18,54066086	-78,78853607	-939,3284302	-0,025096
Cloud_Mesh_Distance_BA_R0_R3	18,65916634	-78,71129608	-939,326416	-0,015552
Cloud_Mesh_Distance_BA_R0_R3	18,88295746	-78,56253052	-939,3460083	-0,00359
Cloud_Mesh_Distance_BA_R0_R3	19,25880432	-78,8433075	-939,2272949	-0,054298
Cloud_Mesh_Distance_BA_R0_R3	19,62503815	-78,64840698	-939,322937	-0,024359
Cloud_Mesh_Distance_BA_R0_R3	19,42861938	-78,69634247	-939,2822266	-0,029837
Cloud_Mesh_Distance_BA_R0_R3	19,48610687	-78,82201385	-939,2442627	-0,041084
Cloud_Mesh_Distance_BA_R0_R3	19,85241699	-78,85855103	-939,2894898	-0,029547
Cloud_Mesh_Distance_BA_R0_R3	20,21382141	-78,84003448	-939,3912354	-0,014159
Cloud_Mesh_Distance_BA_R0_R3	20,0358429	-78,92188263	-939,3112183	-0,023079
Cloud_Mesh_Distance_BA_R0_R3	20,40753174	-79,00649261	-939,3773193	-0,000579
Cloud_Mesh_Distance_BA_R0_R3	18,56570244	-78,56372833	-939,4158936	0,010441
Cloud_Mesh_Distance_BA_R0_R3	18,68738174	-78,58435822	-939,371582	0,002593
Cloud_Mesh_Distance_BA_R0_R3	19,26351166	-78,46453094	-939,3664551	-0,002774
Cloud_Mesh_Distance_BA_R0_R3	19,04607201	-78,60713959	-939,3131714	-0,022046
Cloud_Mesh_Distance_BA_R0_R3	19,25756454	-78,65271759	-939,2908936	-0,029368
Cloud_Mesh_Distance_BA_R0_R3	19,49341583	-78,53792572	-939,3482056	-0,011361
Cloud_Mesh_Distance_BA_R0_R3	19,6758194	-78,51004791	-939,3899536	-0,011996
Cloud_Mesh_Distance_BA_R0_R3	19,98016357	-78,72000885	-939,3745728	-0,021492

Cloud_Mesh_Distance_BA_R0_R3	19,78966904	-78,7121048	-939,3303223	-0,024161
Cloud_Mesh_Distance_BA_R0_R3	20,31498718	-78,73149872	-939,4675903	-0,009232
Cloud_Mesh_Distance_BA_R0_R3	20,15647125	-78,64717865	-939,4581909	-0,018669
Cloud_Mesh_Distance_BA_R0_R3	18,50309372	-78,51712799	-939,4622192	0,016357
Cloud_Mesh_Distance_BA_R0_R3	18,53759003	-78,43078613	-939,4955444	0,029231
Cloud_Mesh_Distance_BA_R0_R3	18,60545349	-78,48456573	-939,4419556	0,022002
Cloud_Mesh_Distance_BA_R0_R3	18,80344963	-78,30846405	-939,4855347	0,034303
Cloud_Mesh_Distance_BA_R0_R3	18,85305023	-78,440979	-939,4061279	0,016471
Cloud_Mesh_Distance_BA_R0_R3	18,71543121	-78,43782806	-939,4355469	0,02353
Cloud_Mesh_Distance_BA_R0_R3	19,0389576	-78,44943237	-939,3792114	0,004827
Cloud_Mesh_Distance_BA_R0_R3	19,46442413	-78,38254547	-939,4157715	0,002538
Cloud_Mesh_Distance_BA_R0_R3	19,6331749	-78,44031525	-939,4134522	-0,009254
Cloud_Mesh_Distance_BA_R0_R3	19,79684448	-78,53154755	-939,4074707	-0,014784
Cloud_Mesh_Distance_BA_R0_R3	19,86188507	-78,41094971	-939,4818726	-0,01324
Cloud_Mesh_Distance_BA_R0_R3	20,83658409	-78,66631317	-939,6625366	0,035476
Cloud_Mesh_Distance_BA_R0_R3	20,79531097	-78,67431641	-939,6425781	0,033347
Cloud_Mesh_Distance_BA_R0_R3	19,59439468	-78,2843399	-939,486023	0,003883
Cloud_Mesh_Distance_BA_R0_R3	19,44310951	-78,26540375	-939,4758911	0,011904
Cloud_Mesh_Distance_BA_R0_R3	18,9586525	-81,87622833	-939,0499878	-0,072782
Cloud_Mesh_Distance_BA_R0_R3	19,0683403	-81,87274933	-939,048584	-0,068049
Cloud_Mesh_Distance_BA_R0_R3	19,31204987	-81,81226349	-939,0445557	-0,058785
Cloud_Mesh_Distance_BA_R0_R3	19,19220734	-81,76021576	-939,0272827	-0,061253
Cloud_Mesh_Distance_BA_R0_R3	19,6131897	-81,92523956	-939,0963745	-0,050083
Cloud_Mesh_Distance_BA_R0_R3	19,75509644	-81,78011322	-939,0828247	-0,03718
Cloud_Mesh_Distance_BA_R0_R3	20,07563782	-81,77715302	-939,1321411	-0,014682
Cloud_Mesh_Distance_BA_R0_R3	18,62909698	-81,48468781	-938,9980469	-0,073471
Cloud_Mesh_Distance_BA_R0_R3	18,98934364	-81,71768951	-939,0164185	-0,067486
Cloud_Mesh_Distance_BA_R0_R3	19,39847565	-81,6558609	-939,0195923	-0,049939
Cloud_Mesh_Distance_BA_R0_R3	19,56192398	-81,75997162	-939,0551148	-0,046485
Cloud_Mesh_Distance_BA_R0_R3	19,70771408	-81,61591339	-939,0444946	-0,034564
Cloud_Mesh_Distance_BA_R0_R3	19,56868172	-81,62627411	-939,0302124	-0,042637
Cloud_Mesh_Distance_BA_R0_R3	19,93697357	-81,78578949	-939,1108398	-0,025814
Cloud_Mesh_Distance_BA_R0_R3	20,05783463	-81,65991974	-939,1060791	-0,014886
Cloud_Mesh_Distance_BA_R0_R3	18,45298958	-81,41791534	-939,0126953	-0,077055
Cloud_Mesh_Distance_BA_R0_R3	18,284338	-81,3736496	-939,0415649	-0,080248
Cloud_Mesh_Distance_BA_R0_R3	18,41915894	-81,23133087	-939,0026245	-0,088868
Cloud_Mesh_Distance_BA_R0_R3	18,8330307	-81,54930878	-938,9929199	-0,067471
Cloud_Mesh_Distance_BA_R0_R3	18,77545547	-81,38043976	-938,9755859	-0,07474
Cloud_Mesh_Distance_BA_R0_R3	19,02413177	-81,52648163	-938,9854126	-0,0658
Cloud_Mesh_Distance_BA_R0_R3	19,14418411	-81,42932892	-938,9732666	-0,062054
Cloud_Mesh_Distance_BA_R0_R3	19,34582901	-81,41191864	-938,9777222	-0,051552
Cloud_Mesh_Distance_BA_R0_R3	19,18201065	-81,58473969	-938,9959106	-0,058499
Cloud_Mesh_Distance_BA_R0_R3	19,60711098	-81,46778107	-939,0077515	-0,037815
Cloud_Mesh_Distance_BA_R0_R3	19,74326324	-81,50357819	-939,0299072	-0,031153
Cloud_Mesh_Distance_BA_R0_R3	19,82477379	-81,53437042	-939,0466919	-0,027835
Cloud_Mesh_Distance_BA_R0_R3	19,8939743	-81,62589264	-939,0725098	-0,024295
Cloud_Mesh_Distance_BA_R0_R3	18,13886261	-81,002388	-939,0592041	-0,106095
Cloud_Mesh_Distance_BA_R0_R3	18,2882576	-81,07324219	-939,0219116	-0,100334
Cloud_Mesh_Distance_BA_R0_R3	18,46391678	-81,060112	-938,9888306	-0,101167
Cloud_Mesh_Distance_BA_R0_R3	18,61740112	-81,31084442	-938,982666	-0,083903

Cloud_Mesh_Distance_BA_R0_R3	18,61652946	-81,13555908	-938,9713135	-0,094589
Cloud_Mesh_Distance_BA_R0_R3	19,19362259	-81,27370453	-938,954895	-0,061725
Cloud_Mesh_Distance_BA_R0_R3	19,1596241	-81,18827057	-938,9452515	-0,064551
Cloud_Mesh_Distance_BA_R0_R3	18,96360588	-81,21520233	-938,9524536	-0,076626
Cloud_Mesh_Distance_BA_R0_R3	18,9771843	-81,34671783	-938,9647217	-0,071092
Cloud_Mesh_Distance_BA_R0_R3	19,54311371	-81,21487427	-938,9640503	-0,038802
Cloud_Mesh_Distance_BA_R0_R3	19,86201477	-81,39540863	-939,0303345	-0,024099
Cloud_Mesh_Distance_BA_R0_R3	19,99676323	-81,29128265	-939,0367432	-0,016353
Cloud_Mesh_Distance_BA_R0_R3	19,78613281	-81,27817535	-939,0015259	-0,027899
Cloud_Mesh_Distance_BA_R0_R3	19,94180107	-81,4307785	-939,0478516	-0,018947
Cloud_Mesh_Distance_BA_R0_R3	19,91846085	-81,50984955	-939,0570068	-0,021383
Cloud_Mesh_Distance_BA_R0_R3	20,20161819	-81,27735138	-939,0741577	-0,00886
Cloud_Mesh_Distance_BA_R0_R3	20,0609436	-81,47505951	-939,0755005	-0,013369
Cloud_Mesh_Distance_BA_R0_R3	20,26716995	-81,40964508	-939,1051636	-0,001206
Cloud_Mesh_Distance_BA_R0_R3	18,60972214	-80,94707489	-938,9648438	-0,107762
Cloud_Mesh_Distance_BA_R0_R3	18,95828629	-81,15673065	-938,947937	-0,079083
Cloud_Mesh_Distance_BA_R0_R3	18,83358955	-81,20552826	-938,9581299	-0,084759
Cloud_Mesh_Distance_BA_R0_R3	18,77396393	-81,04659271	-938,9514771	-0,093636
Cloud_Mesh_Distance_BA_R0_R3	19,38770676	-81,16014862	-938,9464111	-0,04852
Cloud_Mesh_Distance_BA_R0_R3	19,65065002	-81,02070618	-938,9481201	-0,02871
Cloud_Mesh_Distance_BA_R0_R3	19,76113892	-81,09477997	-938,9723511	-0,026481
Cloud_Mesh_Distance_BA_R0_R3	19,9733429	-81,05237579	-939,0009766	-0,018391
Cloud_Mesh_Distance_BA_R0_R3	20,38399506	-81,08402252	-939,0941773	-0,007526
Cloud_Mesh_Distance_BA_R0_R3	20,47719574	-81,23847961	-939,1325073	0,002503
Cloud_Mesh_Distance_BA_R0_R3	20,79683685	-81,08072662	-939,2088013	-0,000262
Cloud_Mesh_Distance_BA_R0_R3	18,13550186	-80,85048676	-939,05896	-0,11159
Cloud_Mesh_Distance_BA_R0_R3	18,28413773	-80,71775818	-939,0187378	-0,118147
Cloud_Mesh_Distance_BA_R0_R3	18,24639511	-80,91159821	-939,0285645	-0,109336
Cloud_Mesh_Distance_BA_R0_R3	18,42629242	-80,77268219	-938,9900513	-0,118359
Cloud_Mesh_Distance_BA_R0_R3	18,40389252	-80,90137482	-938,9961548	-0,111993
Cloud_Mesh_Distance_BA_R0_R3	18,8180809	-80,89348602	-938,9378052	-0,094273
Cloud_Mesh_Distance_BA_R0_R3	18,98470497	-80,83410645	-938,9186401	-0,081731
Cloud_Mesh_Distance_BA_R0_R3	18,99975967	-81,04180145	-938,9356079	-0,078981
Cloud_Mesh_Distance_BA_R0_R3	19,25814819	-80,97805023	-938,921814	-0,055533
Cloud_Mesh_Distance_BA_R0_R3	19,45768738	-81,02966309	-938,9332275	-0,040688
Cloud_Mesh_Distance_BA_R0_R3	19,85974312	-80,90774536	-938,9619751	-0,015422
Cloud_Mesh_Distance_BA_R0_R3	19,97466278	-80,92299652	-938,9848633	-0,014427
Cloud_Mesh_Distance_BA_R0_R3	20,22066498	-80,88889313	-939,0336914	-0,010931
Cloud_Mesh_Distance_BA_R0_R3	20,39909554	-80,91656494	-939,0818481	-0,011685
Cloud_Mesh_Distance_BA_R0_R3	20,15451241	-81,1188736	-939,0455322	-0,013254
Cloud_Mesh_Distance_BA_R0_R3	20,57185936	-80,99979401	-939,1363525	-0,008418
Cloud_Mesh_Distance_BA_R0_R3	20,68966675	-81,12834167	-939,1802368	0,002265
Cloud_Mesh_Distance_BA_R0_R3	20,57468796	-81,11719513	-939,1461182	-0,000662
Cloud_Mesh_Distance_BA_R0_R3	18,11371422	-80,72260284	-939,0662842	-0,116946
Cloud_Mesh_Distance_BA_R0_R3	18,42446899	-80,56977081	-938,9851074	-0,118445
Cloud_Mesh_Distance_BA_R0_R3	18,58584976	-80,52915192	-938,9530029	-0,11264
Cloud_Mesh_Distance_BA_R0_R3	18,7320118	-80,60489655	-938,9326172	-0,105109
Cloud_Mesh_Distance_BA_R0_R3	18,86926079	-80,71178436	-938,9215698	-0,093287
Cloud_Mesh_Distance_BA_R0_R3	19,43523407	-80,8640976	-938,9122925	-0,037416
Cloud_Mesh_Distance_BA_R0_R3	19,55329895	-80,73106384	-938,9047241	-0,022868

Cloud_Mesh_Distance_BA_R0_R3	19,7008934	-80,70740509	-938,9167481	-0,012346
Cloud_Mesh_Distance_BA_R0_R3	19,64005089	-80,85665131	-938,9262695	-0,024047
Cloud_Mesh_Distance_BA_R0_R3	19,83757591	-80,7338028	-938,9373779	-0,00652
Cloud_Mesh_Distance_BA_R0_R3	20,06665421	-80,72911835	-938,9797363	-0,002169
Cloud_Mesh_Distance_BA_R0_R3	20,06557465	-80,85412598	-938,9941406	-0,009645
Cloud_Mesh_Distance_BA_R0_R3	20,67863083	-80,93042755	-939,1618652	-0,008953
Cloud_Mesh_Distance_BA_R0_R3	20,68619156	-81,03391266	-939,1713867	-0,003481
Cloud_Mesh_Distance_BA_R0_R3	20,78777313	-80,971138	-939,1985474	-0,006344
Cloud_Mesh_Distance_BA_R0_R3	18,27429581	-80,45357513	-939,0194092	-0,118623
Cloud_Mesh_Distance_BA_R0_R3	18,18857574	-80,26177216	-939,0499878	-0,111107
Cloud_Mesh_Distance_BA_R0_R3	18,32603073	-80,27703094	-939,006958	-0,108977
Cloud_Mesh_Distance_BA_R0_R3	20,26989365	-80,52492523	-939,0085449	0,009507
Cloud_Mesh_Distance_BA_R0_R3	20,28915405	-80,67934418	-939,0268555	-0,000005
Cloud_Mesh_Distance_BA_R0_R3	20,43701172	-80,72141266	-939,0720215	-0,006492
Cloud_Mesh_Distance_BA_R0_R3	20,61697006	-80,60399628	-939,1166382	-0,00814
Cloud_Mesh_Distance_BA_R0_R3	20,93900299	-80,70038605	-939,2306519	-0,01808
Cloud_Mesh_Distance_BA_R0_R3	20,72966385	-80,70714569	-939,1604614	-0,014255
Cloud_Mesh_Distance_BA_R0_R3	20,8241787	-80,54698181	-939,1809082	-0,01306
Cloud_Mesh_Distance_BA_R0_R3	20,98366928	-80,56933594	-939,2380981	-0,017537
Cloud_Mesh_Distance_BA_R0_R3	18,19521523	-80,09165192	-939,0595703	-0,098636
Cloud_Mesh_Distance_BA_R0_R3	18,07538223	-80,158638	-939,0980835	-0,102592
Cloud_Mesh_Distance_BA_R0_R3	18,28957748	-80,15290833	-939,0239868	-0,10651
Cloud_Mesh_Distance_BA_R0_R3	18,4274559	-80,15827179	-938,9865112	-0,103755
Cloud_Mesh_Distance_BA_R0_R3	18,46234131	-80,37059021	-938,9725342	-0,109762
Cloud_Mesh_Distance_BA_R0_R3	18,62491417	-80,37963867	-938,9412842	-0,104453
Cloud_Mesh_Distance_BA_R0_R3	18,58636856	-80,23471832	-938,9492798	-0,102737
Cloud_Mesh_Distance_BA_R0_R3	20,30583954	-80,39007568	-939,0120239	0,019596
Cloud_Mesh_Distance_BA_R0_R3	20,4570961	-80,54473114	-939,0622559	0,003241
Cloud_Mesh_Distance_BA_R0_R3	20,68166733	-80,39133453	-939,1260986	0,003823
Cloud_Mesh_Distance_BA_R0_R3	20,85565376	-80,41022491	-939,1864014	-0,007809
Cloud_Mesh_Distance_BA_R0_R3	20,97009277	-80,45977783	-939,2288208	-0,014881
Cloud_Mesh_Distance_BA_R0_R3	18,47021866	-80,00935364	-938,9897461	-0,10191
Cloud_Mesh_Distance_BA_R0_R3	18,55368996	-80,09203339	-938,9631958	-0,100234
Cloud_Mesh_Distance_BA_R0_R3	19,50912094	-80,00683594	-938,930481	-0,045193
Cloud_Mesh_Distance_BA_R0_R3	19,70495605	-80,02105713	-938,9454346	-0,021443
Cloud_Mesh_Distance_BA_R0_R3	19,77599335	-80,13459778	-938,9364014	-0,009401
Cloud_Mesh_Distance_BA_R0_R3	20,44846344	-80,36265564	-939,052124	0,01605
Cloud_Mesh_Distance_BA_R0_R3	20,45009232	-80,24253082	-939,055542	0,021025
Cloud_Mesh_Distance_BA_R0_R3	20,59187317	-80,1545639	-939,1043701	0,020975
Cloud_Mesh_Distance_BA_R0_R3	20,84521103	-80,31633759	-939,1824951	-0,001833
Cloud_Mesh_Distance_BA_R0_R3	20,80198669	-80,20349884	-939,1709595	0,009073
Cloud_Mesh_Distance_BA_R0_R3	18,32488251	-79,99292755	-939,0296021	-0,098741
Cloud_Mesh_Distance_BA_R0_R3	18,87398529	-79,84899139	-938,9561768	-0,093689
Cloud_Mesh_Distance_BA_R0_R3	19,03537369	-79,87574005	-938,9420166	-0,087105
Cloud_Mesh_Distance_BA_R0_R3	18,82240295	-79,72846985	-938,9869385	-0,100959
Cloud_Mesh_Distance_BA_R0_R3	18,99697495	-79,69716644	-938,9836426	-0,100081
Cloud_Mesh_Distance_BA_R0_R3	19,19996262	-79,93375397	-938,9296875	-0,074381
Cloud_Mesh_Distance_BA_R0_R3	19,19783783	-79,75481415	-938,9666138	-0,085134
Cloud_Mesh_Distance_BA_R0_R3	19,6693573	-79,91399384	-938,961731	-0,033814
Cloud_Mesh_Distance_BA_R0_R3	19,88968277	-79,99027252	-938,9743042	-0,007271

Cloud_Mesh_Distance_BA_R0_R3	20,1168499	-80,0399704	-939,0030518	0,013439
Cloud_Mesh_Distance_BA_R0_R3	20,11749268	-79,89354706	-939,0316773	0,00528
Cloud_Mesh_Distance_BA_R0_R3	20,32916641	-79,89066315	-939,0766602	0,01764
Cloud_Mesh_Distance_BA_R0_R3	20,54287529	-79,97901154	-939,114624	0,020145
Cloud_Mesh_Distance_BA_R0_R3	20,41950607	-80,09896088	-939,0615234	0,024903
Cloud_Mesh_Distance_BA_R0_R3	20,461586	-80,17797852	-939,0635986	0,023954
Cloud_Mesh_Distance_BA_R0_R3	18,34480286	-79,86106873	-939,0457764	-0,094654
Cloud_Mesh_Distance_BA_R0_R3	18,36318398	-79,73683929	-939,0686035	-0,091885
Cloud_Mesh_Distance_BA_R0_R3	18,45761108	-79,86380768	-939,0161743	-0,096376
Cloud_Mesh_Distance_BA_R0_R3	18,64504623	-79,53775787	-939,0622559	-0,100688
Cloud_Mesh_Distance_BA_R0_R3	18,53223991	-79,7045517	-939,0362549	-0,100187
Cloud_Mesh_Distance_BA_R0_R3	18,85934067	-79,56483459	-939,02771	-0,105318
Cloud_Mesh_Distance_BA_R0_R3	19,04288483	-79,53298187	-939,0256348	-0,102098
Cloud_Mesh_Distance_BA_R0_R3	19,41438675	-79,8323288	-938,9580689	-0,064762
Cloud_Mesh_Distance_BA_R0_R3	19,38009262	-79,61071014	-939,008606	-0,076296
Cloud_Mesh_Distance_BA_R0_R3	19,57356644	-79,78516388	-938,9803467	-0,050986
Cloud_Mesh_Distance_BA_R0_R3	19,5809269	-79,65646362	-939,012146	-0,057816
Cloud_Mesh_Distance_BA_R0_R3	19,65626335	-79,7334671	-939,0007324	-0,046344
Cloud_Mesh_Distance_BA_R0_R3	20,15626144	-79,61487579	-939,1116943	-0,006986
Cloud_Mesh_Distance_BA_R0_R3	20,67002487	-79,93213654	-939,1604004	0,021645
Cloud_Mesh_Distance_BA_R0_R3	20,58860779	-79,86100006	-939,1504517	0,022682
Cloud_Mesh_Distance_BA_R0_R3	20,80091476	-80,01938629	-939,189148	0,013433
Cloud_Mesh_Distance_BA_R0_R3	20,80121613	-79,81295013	-939,227417	0,019207
Cloud_Mesh_Distance_BA_R0_R3	20,95735168	-79,91521454	-939,2609863	0,012252
Cloud_Mesh_Distance_BA_R0_R3	18,47477341	-79,59758759	-939,0783081	-0,094807
Cloud_Mesh_Distance_BA_R0_R3	19,02653885	-79,39925385	-939,0643311	-0,103869
Cloud_Mesh_Distance_BA_R0_R3	19,18616486	-79,57057953	-939,0128174	-0,092914
Cloud_Mesh_Distance_BA_R0_R3	19,16747665	-79,43398285	-939,0507202	-0,09549
Cloud_Mesh_Distance_BA_R0_R3	19,56134415	-79,45497894	-939,0629883	-0,064235
Cloud_Mesh_Distance_BA_R0_R3	19,35352898	-79,39430237	-939,0649414	-0,081977
Cloud_Mesh_Distance_BA_R0_R3	19,71959686	-79,65898132	-939,0278931	-0,044422
Cloud_Mesh_Distance_BA_R0_R3	19,97297478	-79,53554535	-939,0997925	-0,025561
Cloud_Mesh_Distance_BA_R0_R3	19,7708931	-79,51509857	-939,0719605	-0,043439
Cloud_Mesh_Distance_BA_R0_R3	20,12639999	-79,43322754	-939,1605225	-0,01334
Cloud_Mesh_Distance_BA_R0_R3	20,19680786	-79,4859848	-939,1591187	-0,006201
Cloud_Mesh_Distance_BA_R0_R3	20,49827576	-79,76943207	-939,1473999	0,020109
Cloud_Mesh_Distance_BA_R0_R3	20,36000252	-79,74355316	-939,1196899	0,01431
Cloud_Mesh_Distance_BA_R0_R3	20,53964615	-79,67398834	-939,1841431	0,016035
Cloud_Mesh_Distance_BA_R0_R3	20,65295982	-79,77962494	-939,1879273	0,021777
Cloud_Mesh_Distance_BA_R0_R3	20,70643044	-79,63666534	-939,2420044	0,021227
Cloud_Mesh_Distance_BA_R0_R3	18,5800209	-79,32039642	-939,1421509	-0,09381
Cloud_Mesh_Distance_BA_R0_R3	18,65549469	-79,38776398	-939,1070557	-0,099676
Cloud_Mesh_Distance_BA_R0_R3	18,88079834	-79,21762085	-939,1306152	-0,097435
Cloud_Mesh_Distance_BA_R0_R3	18,7309227	-79,25911713	-939,1358032	-0,098293
Cloud_Mesh_Distance_BA_R0_R3	18,82971573	-79,36882782	-939,0891724	-0,104834
Cloud_Mesh_Distance_BA_R0_R3	19,41068268	-79,2820816	-939,100708	-0,077734
Cloud_Mesh_Distance_BA_R0_R3	19,74922371	-79,3122406	-939,1268921	-0,048716
Cloud_Mesh_Distance_BA_R0_R3	19,96380234	-79,35459137	-939,1508179	-0,029544
Cloud_Mesh_Distance_BA_R0_R3	20,43907547	-79,41652679	-939,2373047	0,008458
Cloud_Mesh_Distance_BA_R0_R3	20,32966995	-79,39006805	-939,2190552	0,00058

Cloud_Mesh_Distance_BA_R0_R3	20,38283539	-79,54177094	-939,184021	0,008642
Cloud_Mesh_Distance_BA_R0_R3	20,56189346	-79,49414825	-939,2434692	0,016929
Cloud_Mesh_Distance_BA_R0_R3	20,63353348	-79,38540649	-939,2993774	0,016186
Cloud_Mesh_Distance_BA_R0_R3	20,70184708	-79,49292755	-939,2833862	0,021403
Cloud_Mesh_Distance_BA_R0_R3	20,80289078	-79,5562973	-939,2949829	0,023074
Cloud_Mesh_Distance_BA_R0_R3	19,09312439	-79,08904266	-939,1552124	-0,086054
Cloud_Mesh_Distance_BA_R0_R3	19,22405434	-79,11588287	-939,1447144	-0,083073
Cloud_Mesh_Distance_BA_R0_R3	19,03761292	-79,2257309	-939,116272	-0,099244
Cloud_Mesh_Distance_BA_R0_R3	19,36540604	-79,23680878	-939,1112061	-0,080324
Cloud_Mesh_Distance_BA_R0_R3	19,24217987	-79,27698517	-939,0967407	-0,09208
Cloud_Mesh_Distance_BA_R0_R3	19,42316055	-79,23149872	-939,1152344	-0,074273
Cloud_Mesh_Distance_BA_R0_R3	19,41244888	-79,14059448	-939,1420898	-0,071647
Cloud_Mesh_Distance_BA_R0_R3	19,53207397	-79,25206757	-939,1177979	-0,067997
Cloud_Mesh_Distance_BA_R0_R3	18,63350677	-78,78359985	-939,3058472	-0,026823
Cloud_Mesh_Distance_BA_R0_R3	19,7198143	-78,9787674	-939,2243652	-0,041208
Cloud_Mesh_Distance_BA_R0_R3	19,59307861	-79,09079742	-939,1717529	-0,055845
Cloud_Mesh_Distance_BA_R0_R3	19,73703384	-79,13523102	-939,1776733	-0,046082
Cloud_Mesh_Distance_BA_R0_R3	19,88979721	-79,02205658	-939,2416382	-0,033302
Cloud_Mesh_Distance_BA_R0_R3	19,914711	-79,18304443	-939,1948242	-0,033412
Cloud_Mesh_Distance_BA_R0_R3	20,07273865	-79,21752167	-939,2174072	-0,022846
Cloud_Mesh_Distance_BA_R0_R3	18,6045723	-78,74687958	-939,3261108	-0,018914
Cloud_Mesh_Distance_BA_R0_R3	19,67466736	-78,54933167	-939,3720703	-0,01408
Cloud_Mesh_Distance_BA_R0_R3	19,64945221	-78,52753448	-939,3768311	-0,013135
Cloud_Mesh_Distance_BA_R0_R3	19,61694717	-78,55090332	-939,3602295	-0,014976
Cloud_Mesh_Distance_BA_R0_R3	19,61869431	-78,51100922	-939,3780518	-0,012457
Cloud_Mesh_Distance_BA_R0_R3	19,35218811	-81,25127411	-938,9569702	-0,051055
Cloud_Mesh_Distance_BA_R0_R3	19,29044914	-81,19428253	-938,9477539	-0,055393
Cloud_Mesh_Distance_BA_R0_R3	19,22140884	-81,17624664	-938,9445801	-0,060048
Cloud_Mesh_Distance_BA_R0_R3	19,16534042	-81,11434174	-938,937561	-0,064699
Cloud_Mesh_Distance_BA_R0_R3	19,29658508	-81,10929108	-938,937561	-0,054261
Cloud_Mesh_Distance_BA_R0_R3	19,30573463	-80,83177948	-938,9059448	-0,050675
Cloud_Mesh_Distance_BA_R0_R3	19,15211487	-80,84597015	-938,9102783	-0,063176
Cloud_Mesh_Distance_BA_R0_R3	19,28284454	-80,71166229	-938,8960571	-0,050668
Cloud_Mesh_Distance_BA_R0_R3	20,04701233	-80,91910553	-938,9989624	-0,01279
Cloud_Mesh_Distance_BA_R0_R3	20,01638031	-80,88645172	-938,9890137	-0,012632
Cloud_Mesh_Distance_BA_R0_R3	19,12942123	-80,49906158	-938,8884888	-0,062593
Cloud_Mesh_Distance_BA_R0_R3	19,08743286	-80,66681671	-938,8994751	-0,067418
Cloud_Mesh_Distance_BA_R0_R3	19,40790939	-80,75048065	-938,8999634	-0,036813
Cloud_Mesh_Distance_BA_R0_R3	19,28032684	-80,55542755	-938,8869629	-0,04859
Cloud_Mesh_Distance_BA_R0_R3	19,99321556	-80,85806274	-938,9810181	-0,012372
Cloud_Mesh_Distance_BA_R0_R3	18,72570419	-80,46234894	-938,9279175	-0,100338
Cloud_Mesh_Distance_BA_R0_R3	18,7689991	-80,30177307	-938,9194946	-0,095225
Cloud_Mesh_Distance_BA_R0_R3	18,9196701	-80,49079132	-938,9041748	-0,085641
Cloud_Mesh_Distance_BA_R0_R3	19,09887695	-80,3639679	-938,8891602	-0,066577
Cloud_Mesh_Distance_BA_R0_R3	18,95395279	-80,29807281	-938,9002075	-0,080483
Cloud_Mesh_Distance_BA_R0_R3	19,6772995	-80,59648895	-938,9063721	-0,010901
Cloud_Mesh_Distance_BA_R0_R3	19,66187668	-80,44384003	-938,9020386	-0,011023
Cloud_Mesh_Distance_BA_R0_R3	19,47210312	-80,60494232	-938,892334	-0,027425
Cloud_Mesh_Distance_BA_R0_R3	19,80904007	-80,61841583	-938,9231567	-0,002176
Cloud_Mesh_Distance_BA_R0_R3	19,96287918	-80,5881424	-938,944519	0,004837

Cloud_Mesh_Distance_BA_R0_R3	20,11553192	-80,54366302	-938,9725342	0,009042
Cloud_Mesh_Distance_BA_R0_R3	19,23273087	-80,27996063	-938,8886108	-0,056128
Cloud_Mesh_Distance_BA_R0_R3	19,26597214	-80,4026413	-938,8845825	-0,050836
Cloud_Mesh_Distance_BA_R0_R3	19,6247139	-80,28432465	-938,9046021	-0,017936
Cloud_Mesh_Distance_BA_R0_R3	19,45162582	-80,42945099	-938,8877564	-0,029264
Cloud_Mesh_Distance_BA_R0_R3	19,87626457	-80,46710968	-938,9266968	0,004699
Cloud_Mesh_Distance_BA_R0_R3	20,20223427	-80,44359589	-938,9872437	0,017484
Cloud_Mesh_Distance_BA_R0_R3	20,07859421	-80,444664	-938,9605103	0,016104
Cloud_Mesh_Distance_BA_R0_R3	20,36473846	-80,30647278	-939,0284424	0,020855
Cloud_Mesh_Distance_BA_R0_R3	20,23697662	-80,32711792	-938,9959106	0,020962
Cloud_Mesh_Distance_BA_R0_R3	18,69432831	-80,1140213	-938,9379273	-0,095356
Cloud_Mesh_Distance_BA_R0_R3	18,89242172	-80,12864685	-938,9139404	-0,085612
Cloud_Mesh_Distance_BA_R0_R3	18,78011894	-79,97626495	-938,9422607	-0,093576
Cloud_Mesh_Distance_BA_R0_R3	18,92198944	-79,97438049	-938,9302368	-0,08804
Cloud_Mesh_Distance_BA_R0_R3	19,05290985	-80,03237152	-938,9153442	-0,079908
Cloud_Mesh_Distance_BA_R0_R3	19,10022736	-80,19307709	-938,8967285	-0,071221
Cloud_Mesh_Distance_BA_R0_R3	19,43502998	-80,13558197	-938,9066773	-0,044755
Cloud_Mesh_Distance_BA_R0_R3	19,39079285	-80,08325958	-938,9115601	-0,052508
Cloud_Mesh_Distance_BA_R0_R3	19,41064072	-80,26560211	-938,8925781	-0,037864
Cloud_Mesh_Distance_BA_R0_R3	19,57624435	-80,14524078	-938,9151611	-0,031479
Cloud_Mesh_Distance_BA_R0_R3	19,87146378	-80,26866913	-938,9335938	0,002469
Cloud_Mesh_Distance_BA_R0_R3	19,98107338	-80,08129883	-938,9724121	0,005779
Cloud_Mesh_Distance_BA_R0_R3	20,09462166	-80,15293121	-938,9817505	0,015757
Cloud_Mesh_Distance_BA_R0_R3	20,24602509	-80,1886673	-939,0071411	0,022642
Cloud_Mesh_Distance_BA_R0_R3	20,0704174	-80,30762482	-938,9615479	0,017522
Cloud_Mesh_Distance_BA_R0_R3	20,36775589	-80,22197723	-939,0343018	0,023319
Cloud_Mesh_Distance_BA_R0_R3	18,60585022	-79,95519257	-938,9699707	-0,096887
Cloud_Mesh_Distance_BA_R0_R3	18,72230721	-79,8351059	-938,973877	-0,097595
Cloud_Mesh_Distance_BA_R0_R3	19,19281197	-80,07022095	-938,9085083	-0,068718
Cloud_Mesh_Distance_BA_R0_R3	19,2967453	-80,15380096	-938,9001465	-0,057558
Cloud_Mesh_Distance_BA_R0_R3	19,33115196	-80,01753235	-938,9185791	-0,060517
Cloud_Mesh_Distance_BA_R0_R3	19,56384659	-79,87425995	-938,960144	-0,047524
Cloud_Mesh_Distance_BA_R0_R3	19,77208138	-79,83049774	-938,9919434	-0,029424
Cloud_Mesh_Distance_BA_R0_R3	19,93771744	-79,88284302	-939,0037231	-0,01118
Cloud_Mesh_Distance_BA_R0_R3	19,80867767	-79,91485596	-938,9786377	-0,020983
Cloud_Mesh_Distance_BA_R0_R3	20,25627899	-80,03018951	-939,0324707	0,019202
Cloud_Mesh_Distance_BA_R0_R3	18,69016647	-79,69553375	-939,0106812	-0,101037
Cloud_Mesh_Distance_BA_R0_R3	18,59619904	-79,80908966	-938,9983521	-0,096654
Cloud_Mesh_Distance_BA_R0_R3	19,66299057	-79,8159256	-938,9821777	-0,040512
Cloud_Mesh_Distance_BA_R0_R3	19,75229645	-79,74052429	-939,0106812	-0,037488
Cloud_Mesh_Distance_BA_R0_R3	19,87534904	-79,83239746	-939,0062256	-0,019889
Cloud_Mesh_Distance_BA_R0_R3	19,98864746	-79,77313232	-939,0373535	-0,009545
Cloud_Mesh_Distance_BA_R0_R3	19,84184647	-79,64401245	-939,0481567	-0,032115
Cloud_Mesh_Distance_BA_R0_R3	19,96666336	-79,66301727	-939,0629273	-0,019197
Cloud_Mesh_Distance_BA_R0_R3	19,86649704	-79,74880219	-939,0248413	-0,024069
Cloud_Mesh_Distance_BA_R0_R3	19,38909149	-79,25733185	-939,1065674	-0,079263
Cloud_Mesh_Distance_BA_R0_R3	19,35284042	-79,28735352	-939,0957642	-0,082028
Cloud_Mesh_Distance_ICA_R0_R1	3,79118347	-92,97956848	-948,7341919	0,040273
Cloud_Mesh_Distance_ICA_R0_R1	4,41677856	-93,02081299	-948,7346802	-0,107881
Cloud_Mesh_Distance_ICA_R0_R1	3,7816925	-92,79997253	-948,7312622	0,055314

Cloud_Mesh_Distance_ICA_R0_R1	4,03831482	-93,04948425	-948,742981	-0,02605
Cloud_Mesh_Distance_ICA_R0_R1	4,25830078	-92,9921875	-948,746521	-0,068255
Cloud_Mesh_Distance_ICA_R0_R1	4,56227112	-92,88023376	-948,7228394	-0,115851
Cloud_Mesh_Distance_ICA_R0_R1	4,36196899	-92,79341125	-948,7434082	-0,06403
Cloud_Mesh_Distance_ICA_R0_R1	4,74928284	-93,09288025	-948,6883545	-0,205092
Cloud_Mesh_Distance_ICA_R0_R1	4,58815002	-93,04994202	-948,7158203	-0,167934
Cloud_Mesh_Distance_ICA_R0_R1	5,04016113	-92,95536804	-948,6254273	-0,162537
Cloud_Mesh_Distance_ICA_R0_R1	4,2023468	-92,84983826	-948,7511597	-0,041833
Cloud_Mesh_Distance_ICA_R0_R1	3,99452209	-92,80953979	-948,7476807	0,010491
Cloud_Mesh_Distance_ICA_R0_R1	4,2013092	-92,60066223	-948,7362671	-0,000736
Cloud_Mesh_Distance_ICA_R0_R1	3,96427917	-92,60380554	-948,730957	0,042806
Cloud_Mesh_Distance_ICA_R0_R1	4,87194824	-92,97877502	-948,6679077	-0,160355
Cloud_Mesh_Distance_ICA_R0_R1	4,73548889	-92,92393494	-948,6953735	-0,143745
Cloud_Mesh_Distance_ICA_R0_R1	4,71621704	-92,74595642	-948,6889038	-0,12063
Cloud_Mesh_Distance_ICA_R0_R1	4,51837158	-92,69584656	-948,7190552	-0,079986
Cloud_Mesh_Distance_ICA_R0_R1	4,68328857	-92,56747437	-948,6724243	-0,082135
Cloud_Mesh_Distance_ICA_R0_R1	4,90811157	-92,80943298	-948,6504517	-0,139564
Cloud_Mesh_Distance_ICA_R0_R1	5,12762451	-92,82019043	-948,5872803	-0,138307
Cloud_Mesh_Distance_ICA_R0_R1	3,60194397	-92,95503235	-948,7150879	0,086406
Cloud_Mesh_Distance_ICA_R0_R1	3,46559143	-92,76457214	-948,6844482	0,11913
Cloud_Mesh_Distance_ICA_R0_R1	3,69142151	-92,68339539	-948,7111816	0,082791
Cloud_Mesh_Distance_ICA_R0_R1	5,2203064	-93,00216675	-948,5730591	-0,267774
Cloud_Mesh_Distance_ICA_R0_R1	5,34591675	-92,84805298	-948,5117798	-0,207449
Cloud_Mesh_Distance_ICA_R0_R1	5,49620056	-92,93669128	-948,4630127	-0,26253
Cloud_Mesh_Distance_ICA_R0_R1	5,66017151	-92,94520569	-948,3899536	-0,351544
Cloud_Mesh_Distance_ICA_R0_R1	5,2024231	-92,67333984	-948,5338745	-0,126752
Cloud_Mesh_Distance_ICA_R0_R1	4,25100708	-92,41931152	-948,7042236	0,006541
Cloud_Mesh_Distance_ICA_R0_R1	4,47010803	-92,52474976	-948,7055664	-0,040556
Cloud_Mesh_Distance_ICA_R0_R1	4,60279846	-92,33164978	-948,6381836	-0,032996
Cloud_Mesh_Distance_ICA_R0_R1	4,82150269	-92,43476868	-948,6072388	-0,076812
Cloud_Mesh_Distance_ICA_R0_R1	4,86932373	-92,61885071	-948,6339111	-0,109577
Cloud_Mesh_Distance_ICA_R0_R1	5,02272034	-92,67538452	-948,5984497	-0,122568
Cloud_Mesh_Distance_ICA_R0_R1	5,03913879	-92,50302124	-948,5518799	-0,098947
Cloud_Mesh_Distance_ICA_R0_R1	5,52598572	-92,76972961	-948,4168091	-0,162367
Cloud_Mesh_Distance_ICA_R0_R1	5,6245575	-92,64225769	-948,3253174	-0,147244
Cloud_Mesh_Distance_ICA_R0_R1	5,71369934	-92,74128723	-948,3104248	-0,266532
Cloud_Mesh_Distance_ICA_R0_R1	3,64221191	-92,2492218	-948,6290283	0,126246
Cloud_Mesh_Distance_ICA_R0_R1	4,06314087	-92,42008972	-948,7050781	0,043879
Cloud_Mesh_Distance_ICA_R0_R1	3,88172913	-92,38063049	-948,6868897	0,075062
Cloud_Mesh_Distance_ICA_R0_R1	4,0426178	-92,24287415	-948,6671753	0,065735
Cloud_Mesh_Distance_ICA_R0_R1	4,22805786	-92,27407837	-948,6731567	0,028869
Cloud_Mesh_Distance_ICA_R0_R1	5,21691895	-92,4985199	-948,4797974	-0,088828
Cloud_Mesh_Distance_ICA_R0_R1	4,9553833	-92,30097961	-948,5205689	-0,052379
Cloud_Mesh_Distance_ICA_R0_R1	5,3523407	-92,62446594	-948,4588623	-0,111051
Cloud_Mesh_Distance_ICA_R0_R1	5,15350342	-92,34631348	-948,4530029	-0,063212
Cloud_Mesh_Distance_ICA_R0_R1	5,34347534	-92,41827393	-948,388794	-0,069492
Cloud_Mesh_Distance_ICA_R0_R1	4,3639679	-92,15814209	-948,6335449	0,025997
Cloud_Mesh_Distance_ICA_R0_R1	4,20249939	-92,10993958	-948,6339722	0,051567
Cloud_Mesh_Distance_ICA_R0_R1	4,43319702	-92,31399536	-948,6638794	-0,005989
Cloud_Mesh_Distance_ICA_R0_R1	4,73731995	-92,08570862	-948,5279541	-0,012997

Cloud_Mesh_Distance_ICA_R0_R1	4,74183655	-92,24423218	-948,5773315	-0,033652
Cloud_Mesh_Distance_ICA_R0_R1	5,06655884	-92,1681366	-948,4212036	-0,028045
Cloud_Mesh_Distance_ICA_R0_R1	4,88500977	-92,12870789	-948,4864502	-0,019614
Cloud_Mesh_Distance_ICA_R0_R1	5,44979858	-92,31216431	-948,2769775	-0,045546
Cloud_Mesh_Distance_ICA_R0_R1	4,56149292	-92,12083435	-948,5881348	0,004505
Cloud_Mesh_Distance_ICA_R0_R1	4,17250061	-91,94630432	-948,5934448	0,071468
Cloud_Mesh_Distance_ICA_R0_R1	3,97587585	-91,94868469	-948,59375	0,091941
Cloud_Mesh_Distance_ICA_R0_R1	4,07888794	-91,78352356	-948,5554199	0,088418
Cloud_Mesh_Distance_ICA_R0_R1	4,38093567	-91,98988342	-948,5864868	0,034344
Cloud_Mesh_Distance_ICA_R0_R1	4,47926331	-91,85368347	-948,531311	0,045533
Cloud_Mesh_Distance_ICA_R0_R1	4,6098938	-91,96897888	-948,5314331	0,021432
Cloud_Mesh_Distance_ICA_R0_R1	3,15206909	-92,93289185	-948,6326294	0,366475
Cloud_Mesh_Distance_ICA_R0_R1	2,70599365	-92,86219788	-948,5311279	0,788275
Cloud_Mesh_Distance_ICA_R0_R1	3,2052002	-92,75994873	-948,6307983	0,295706
Cloud_Mesh_Distance_ICA_R0_R1	3,29772949	-92,90016174	-948,6607666	0,231786
Cloud_Mesh_Distance_ICA_R0_R1	3,05622864	-92,67378235	-948,5895996	0,450995
Cloud_Mesh_Distance_ICA_R0_R1	2,95220947	-92,90290833	-948,5891724	0,54765
Cloud_Mesh_Distance_ICA_R0_R1	2,81526184	-92,66665649	-948,532959	0,689139
Cloud_Mesh_Distance_ICA_R0_R1	3,22285461	-92,53623962	-948,6055298	0,291897
Cloud_Mesh_Distance_ICA_R0_R1	3,40908813	-92,59931946	-948,6535645	0,153793
Cloud_Mesh_Distance_ICA_R0_R1	5,79882813	-92,85249329	-948,295044	-0,40044
Cloud_Mesh_Distance_ICA_R0_R1	5,91291809	-92,72236633	-948,1747437	-0,264192
Cloud_Mesh_Distance_ICA_R0_R1	3,03036499	-92,49691772	-948,555603	0,431982
Cloud_Mesh_Distance_ICA_R0_R1	2,88002014	-92,45239258	-948,5092163	0,53745
Cloud_Mesh_Distance_ICA_R0_R1	3,43717957	-92,36924744	-948,6168823	0,148172
Cloud_Mesh_Distance_ICA_R0_R1	3,64219666	-92,52122498	-948,6829224	0,105725
Cloud_Mesh_Distance_ICA_R0_R1	5,88246155	-92,49946594	-948,0731201	-0,091634
Cloud_Mesh_Distance_ICA_R0_R1	3,00810242	-92,26203918	-948,4952393	0,371438
Cloud_Mesh_Distance_ICA_R0_R1	3,21151733	-92,32258606	-948,5599365	0,231155
Cloud_Mesh_Distance_ICA_R0_R1	3,41874695	-92,16651917	-948,5643921	0,161463
Cloud_Mesh_Distance_ICA_R0_R1	5,50335693	-92,52546692	-948,3491211	-0,083495
Cloud_Mesh_Distance_ICA_R0_R1	5,7245636	-92,52496338	-948,2072754	-0,110958
Cloud_Mesh_Distance_ICA_R0_R1	3,39784241	-91,98568726	-948,5055542	0,173903
Cloud_Mesh_Distance_ICA_R0_R1	4,9750824	-92,02429199	-948,4046631	-0,005515
Cloud_Mesh_Distance_ICA_R0_R1	5,31060791	-92,03770447	-948,2038574	0,002553
Cloud_Mesh_Distance_ICA_R0_R1	5,27288818	-92,20651245	-948,3265991	-0,03009
Cloud_Mesh_Distance_ICA_R0_R1	5,46702576	-92,10707092	-948,1333008	-0,002499
Cloud_Mesh_Distance_ICA_R0_R1	5,62536621	-92,38166809	-948,1980591	-0,054544
Cloud_Mesh_Distance_ICA_R0_R1	3,8006897	-92,09191895	-948,6143799	0,113381
Cloud_Mesh_Distance_ICA_R0_R1	3,60076904	-92,01519775	-948,5595703	0,143547
Cloud_Mesh_Distance_ICA_R0_R1	3,50074768	-91,82287598	-948,479187	0,155565
Cloud_Mesh_Distance_ICA_R0_R1	4,96018982	-91,85523987	-948,3406982	0,025395
Cloud_Mesh_Distance_ICA_R0_R1	5,15177917	-91,95671082	-948,2675781	0,008788
Cloud_Mesh_Distance_ICA_R0_R1	3,68359375	-91,68479919	-948,4802856	0,132611
Cloud_Mesh_Distance_ICA_R0_R1	4,05204773	-91,5458374	-948,5061646	0,08413
Cloud_Mesh_Distance_ICA_R0_R1	3,92390442	-91,65736389	-948,5164185	0,091824
Cloud_Mesh_Distance_ICA_R0_R1	3,81468201	-91,86102295	-948,5536499	0,120827
Cloud_Mesh_Distance_ICA_R0_R1	4,31575012	-91,81364441	-948,5498657	0,052677
Cloud_Mesh_Distance_ICA_R0_R1	4,6388855	-91,61927795	-948,4272461	0,06253
Cloud_Mesh_Distance_ICA_R0_R1	4,65837097	-91,82435608	-948,472229	0,031003

Cloud_Mesh_Distance_ICA_R0_R1	4,81391907	-91,89802551	-948,4329224	0,026348
Cloud_Mesh_Distance_ICA_R0_R1	4,04804993	-91,39682007	-948,482544	0,062465
Cloud_Mesh_Distance_ICA_R0_R1	4,265625	-91,39929199	-948,4824219	0,045992
Cloud_Mesh_Distance_ICA_R0_R1	4,29069519	-91,62475586	-948,5126343	0,060457
Cloud_Mesh_Distance_ICA_R0_R1	4,47981262	-91,70213318	-948,4948731	0,054077
Cloud_Mesh_Distance_ICA_R0_R1	4,80711365	-91,70140076	-948,3709106	0,046711
Cloud_Mesh_Distance_ICA_R0_R1	4,68502808	-91,43527222	-948,3725586	0,067458
Cloud_Mesh_Distance_ICA_R0_R1	4,90896606	-91,5448761	-948,2619629	0,076298
Cloud_Mesh_Distance_ICA_R0_R1	4,48721313	-91,48974609	-948,453125	0,062929
Cloud_Mesh_Distance_ICA_R0_R1	4,40830994	-91,24385071	-948,4519653	0,048156
Cloud_Mesh_Distance_ICA_R0_R1	4,61524963	-91,15290833	-948,3800049	0,062095
Cloud_Mesh_Distance_ICA_R0_R1	4,47550964	-91,09230042	-948,4307861	0,033036
Cloud_Mesh_Distance_ICA_R0_R1	4,26004028	-91,03715515	-948,4664307	-0,003812
Cloud_Mesh_Distance_ICA_R0_R1	4,55273438	-90,75598145	-948,4241333	0,017102
Cloud_Mesh_Distance_ICA_R0_R1	4,48136902	-90,90979004	-948,435669	0,009218
Cloud_Mesh_Distance_ICA_R0_R1	4,47732544	-90,55943298	-948,4621582	-0,036009
Cloud_Mesh_Distance_ICA_R0_R1	2,63926697	-92,49520874	-948,4483643	0,767558
Cloud_Mesh_Distance_ICA_R0_R1	2,80369568	-92,32775879	-948,4539185	0,569852
Cloud_Mesh_Distance_ICA_R0_R1	5,88887024	-92,33592224	-947,940918	-0,045612
Cloud_Mesh_Distance_ICA_R0_R1	2,73887634	-92,06498718	-948,3378906	0,656268
Cloud_Mesh_Distance_ICA_R0_R1	5,77041626	-92,37030029	-948,0759888	-0,051895
Cloud_Mesh_Distance_ICA_R0_R1	5,76239014	-92,23078918	-947,9697876	-0,020654
Cloud_Mesh_Distance_ICA_R0_R1	3,17166138	-92,04881287	-948,4700317	0,284276
Cloud_Mesh_Distance_ICA_R0_R1	3,26164246	-91,89683533	-948,4379883	0,244133
Cloud_Mesh_Distance_ICA_R0_R1	5,24252319	-91,8762207	-948,1542969	0,034545
Cloud_Mesh_Distance_ICA_R0_R1	5,41546631	-91,96348572	-948,0662842	0,024732
Cloud_Mesh_Distance_ICA_R0_R1	5,71704102	-92,10719299	-947,9000244	0,003379
Cloud_Mesh_Distance_ICA_R0_R1	5,6161499	-92,23414612	-948,105957	-0,01839
Cloud_Mesh_Distance_ICA_R0_R1	3,49154663	-91,63589478	-948,4127197	0,155105
Cloud_Mesh_Distance_ICA_R0_R1	3,70233154	-91,4972229	-948,4365845	0,11116
Cloud_Mesh_Distance_ICA_R0_R1	3,84916687	-91,46366882	-948,463562	0,093965
Cloud_Mesh_Distance_ICA_R0_R1	3,73020935	-91,33465576	-948,4086304	0,082598
Cloud_Mesh_Distance_ICA_R0_R1	3,56059265	-91,43510437	-948,3739624	0,12266
Cloud_Mesh_Distance_ICA_R0_R1	3,58480835	-91,26560974	-948,3380127	0,092757
Cloud_Mesh_Distance_ICA_R0_R1	3,76159668	-91,15812683	-948,3874512	0,039914
Cloud_Mesh_Distance_ICA_R0_R1	4,87734985	-91,38034058	-948,2440796	0,08778
Cloud_Mesh_Distance_ICA_R0_R1	3,61961365	-91,08706665	-948,3122559	0,042908
Cloud_Mesh_Distance_ICA_R0_R1	3,93373108	-91,23284912	-948,4459839	0,041341
Cloud_Mesh_Distance_ICA_R0_R1	4,13847351	-91,22650146	-948,4732666	0,03537
Cloud_Mesh_Distance_ICA_R0_R1	4,7711792	-91,23268127	-948,3004761	0,085915
Cloud_Mesh_Distance_ICA_R0_R1	3,92912292	-91,02919006	-948,4258423	-0,017407
Cloud_Mesh_Distance_ICA_R0_R1	3,94093323	-90,83442688	-948,4185181	-0,079729
Cloud_Mesh_Distance_ICA_R0_R1	3,78015137	-90,97738647	-948,3707275	-0,019613
Cloud_Mesh_Distance_ICA_R0_R1	4,30708313	-90,81755066	-948,4694214	-0,042138
Cloud_Mesh_Distance_ICA_R0_R1	4,07264709	-91,0657959	-948,4577637	-0,005748
Cloud_Mesh_Distance_ICA_R0_R1	4,09269714	-90,88961792	-948,4556274	-0,053581
Cloud_Mesh_Distance_ICA_R0_R1	4,6124115	-90,96072388	-948,3850708	0,051112
Cloud_Mesh_Distance_ICA_R0_R1	4,74066162	-91,01800537	-948,3151245	0,076412
Cloud_Mesh_Distance_ICA_R0_R1	3,71946716	-90,68615723	-948,3051758	-0,107161
Cloud_Mesh_Distance_ICA_R0_R1	3,92402649	-90,62016296	-948,4013672	-0,156598

Cloud_Mesh_Distance_ICA_R0_R1	4,73805237	-90,82493591	-948,3273926	0,078988
Cloud_Mesh_Distance_ICA_R0_R1	4,88560486	-90,91452026	-948,2057495	0,12388
Cloud_Mesh_Distance_ICA_R0_R1	4,14572144	-90,41848755	-948,4662476	-0,198691
Cloud_Mesh_Distance_ICA_R0_R1	4,35079956	-90,66468811	-948,4746704	-0,067292
Cloud_Mesh_Distance_ICA_R0_R1	4,14854431	-90,66252136	-948,4639893	-0,125369
Cloud_Mesh_Distance_ICA_R0_R1	4,37161255	-90,45594788	-948,4802246	-0,099173
Cloud_Mesh_Distance_ICA_R0_R1	4,73817444	-90,62806702	-948,3445435	0,071617
Cloud_Mesh_Distance_ICA_R0_R1	4,71582031	-90,41134644	-948,3721924	0,047807
Cloud_Mesh_Distance_ICA_R0_R1	4,86650085	-90,47795105	-948,2581177	0,111018
Cloud_Mesh_Distance_ICA_R0_R1	4,85742188	-90,69293213	-948,2480469	0,11435
Cloud_Mesh_Distance_ICA_R0_R1	3,88948059	-90,38633728	-948,3724365	-0,238835
Cloud_Mesh_Distance_ICA_R0_R1	4,31144714	-90,22950745	-948,4885254	-0,168674
Cloud_Mesh_Distance_ICA_R0_R1	4,57804871	-90,25137329	-948,4379273	-0,03288
Cloud_Mesh_Distance_ICA_R0_R1	4,75637817	-90,20503235	-948,3477783	0,041855
Cloud_Mesh_Distance_ICA_R0_R1	3,91107178	-90,22398376	-948,362854	-0,29798
Cloud_Mesh_Distance_ICA_R0_R1	4,16247559	-90,24380493	-948,4655151	-0,237663
Cloud_Mesh_Distance_ICA_R0_R1	3,99064636	-90,03712463	-948,3719482	-0,363012
Cloud_Mesh_Distance_ICA_R0_R1	4,24134827	-90,04949951	-948,4571533	-0,243313
Cloud_Mesh_Distance_ICA_R0_R1	4,56420898	-90,0171051	-948,4249268	-0,060267
Cloud_Mesh_Distance_ICA_R0_R1	4,72207642	-90,04495239	-948,3565064	0,016047
Cloud_Mesh_Distance_ICA_R0_R1	4,86521912	-90,26609802	-948,2665405	0,11017
Cloud_Mesh_Distance_ICA_R0_R1	4,85681152	-90,07171631	-948,2625122	0,100309
Cloud_Mesh_Distance_ICA_R0_R1	4,9871521	-90,31843567	-948,1329346	0,170733
Cloud_Mesh_Distance_ICA_R0_R1	4,21876526	-89,84434509	-948,4025269	-0,284624
Cloud_Mesh_Distance_ICA_R0_R1	4,40000916	-90,09492493	-948,4736328	-0,14328
Cloud_Mesh_Distance_ICA_R0_R1	4,8276062	-89,86506653	-948,2508545	0,073818
Cloud_Mesh_Distance_ICA_R0_R1	4,95046997	-90,13679504	-948,1723023	0,150027
Cloud_Mesh_Distance_ICA_R0_R1	4,95773315	-89,94364929	-948,1310425	0,142381
Cloud_Mesh_Distance_ICA_R0_R1	4,40344238	-89,71127319	-948,3763428	-0,182194
Cloud_Mesh_Distance_ICA_R0_R1	4,42320251	-89,90983582	-948,43396	-0,156217
Cloud_Mesh_Distance_ICA_R0_R1	4,68937683	-89,88926697	-948,3494873	-0,008256
Cloud_Mesh_Distance_ICA_R0_R1	4,92468262	-89,73670959	-948,1057739	0,12486
Cloud_Mesh_Distance_ICA_R0_R1	4,54611206	-89,58666992	-948,2996216	-0,09944
Cloud_Mesh_Distance_ICA_R0_R1	4,7013855	-89,70066833	-948,2875366	-0,009496
Cloud_Mesh_Distance_ICA_R0_R1	4,56495667	-89,79475403	-948,3759766	-0,078716
Cloud_Mesh_Distance_ICA_R0_R1	4,36146545	-89,54670715	-948,3012695	-0,215207
Cloud_Mesh_Distance_ICA_R0_R1	6,03372192	-92,43887329	-947,8919678	-0,103396
Cloud_Mesh_Distance_ICA_R0_R1	2,76173401	-91,85552979	-948,2372437	0,543641
Cloud_Mesh_Distance_ICA_R0_R1	2,60548401	-91,93553162	-948,215271	0,711944
Cloud_Mesh_Distance_ICA_R0_R1	2,92364502	-91,97546387	-948,3630371	0,502207
Cloud_Mesh_Distance_ICA_R0_R1	5,970047	-92,24349976	-947,7620239	-0,034072
Cloud_Mesh_Distance_ICA_R0_R1	3,05395508	-91,82852173	-948,3400269	0,328317
Cloud_Mesh_Distance_ICA_R0_R1	3,24040222	-91,65116882	-948,3294678	0,191354
Cloud_Mesh_Distance_ICA_R0_R1	5,31698608	-91,80204773	-948,0370483	0,051174
Cloud_Mesh_Distance_ICA_R0_R1	5,58830261	-92,00334167	-947,9332886	0,030261
Cloud_Mesh_Distance_ICA_R0_R1	5,4683075	-91,8240509	-947,8928833	0,062505
Cloud_Mesh_Distance_ICA_R0_R1	5,62420654	-91,87129211	-947,7489014	0,057594
Cloud_Mesh_Distance_ICA_R0_R1	3,31585693	-91,76283264	-948,4022217	0,188753
Cloud_Mesh_Distance_ICA_R0_R1	5,34106445	-91,6502533	-947,8948975	0,089956
Cloud_Mesh_Distance_ICA_R0_R1	3,25047302	-91,26179504	-948,1563721	0,149196

Cloud_Mesh_Distance_ICA_R0_R1	3,21453857	-91,48808289	-948,2424927	0,181148
Cloud_Mesh_Distance_ICA_R0_R1	3,40985107	-91,37294006	-948,2904053	0,140967
Cloud_Mesh_Distance_ICA_R0_R1	3,3952179	-91,53355408	-948,3427124	0,162602
Cloud_Mesh_Distance_ICA_R0_R1	5,05015564	-91,6967926	-948,2114868	0,061182
Cloud_Mesh_Distance_ICA_R0_R1	5,1865387	-91,65002441	-948,0683594	0,07904
Cloud_Mesh_Distance_ICA_R0_R1	5,21592712	-91,47984314	-947,934021	0,111051
Cloud_Mesh_Distance_ICA_R0_R1	5,3170929	-91,49780273	-947,8016357	0,125455
Cloud_Mesh_Distance_ICA_R0_R1	3,4309845	-91,18278503	-948,2352905	0,102384
Cloud_Mesh_Distance_ICA_R0_R1	5,06558228	-91,43493652	-948,0900269	0,107418
Cloud_Mesh_Distance_ICA_R0_R1	5,25296021	-91,3526001	-947,7978516	0,152157
Cloud_Mesh_Distance_ICA_R0_R1	5,10250854	-91,27548218	-947,9921875	0,135696
Cloud_Mesh_Distance_ICA_R0_R1	3,72605896	-90,8394928	-948,3279419	-0,056643
Cloud_Mesh_Distance_ICA_R0_R1	5,05563354	-91,12052917	-948,0178833	0,150254
Cloud_Mesh_Distance_ICA_R0_R1	4,97737122	-91,2545929	-948,1329346	0,114229
Cloud_Mesh_Distance_ICA_R0_R1	3,4393158	-90,88571167	-948,1392822	0,023674
Cloud_Mesh_Distance_ICA_R0_R1	3,47029114	-90,73475647	-948,1149292	-0,030733
Cloud_Mesh_Distance_ICA_R0_R1	3,57991028	-90,81524658	-948,2313843	-0,033438
Cloud_Mesh_Distance_ICA_R0_R1	3,5096283	-90,99240112	-948,225647	0,030947
Cloud_Mesh_Distance_ICA_R0_R1	4,91299438	-91,0997467	-948,1797485	0,124338
Cloud_Mesh_Distance_ICA_R0_R1	3,62483215	-90,5869751	-948,2202759	-0,116502
Cloud_Mesh_Distance_ICA_R0_R1	3,80410767	-90,51403809	-948,3375855	-0,177304
Cloud_Mesh_Distance_ICA_R0_R1	4,94595337	-90,78477478	-948,1513062	0,143158
Cloud_Mesh_Distance_ICA_R0_R1	3,75547791	-90,36595154	-948,2826538	-0,226954
Cloud_Mesh_Distance_ICA_R0_R1	3,78779602	-90,1491394	-948,2573853	-0,309151
Cloud_Mesh_Distance_ICA_R0_R1	3,66764832	-90,16468811	-948,1380005	-0,277668
Cloud_Mesh_Distance_ICA_R0_R1	5,14280701	-90,33860779	-947,8101807	0,256692
Cloud_Mesh_Distance_ICA_R0_R1	5,0867157	-90,22712708	-947,9589233	0,223724
Cloud_Mesh_Distance_ICA_R0_R1	5,03947449	-90,10882568	-948,0335083	0,192414
Cloud_Mesh_Distance_ICA_R0_R1	4,01071167	-89,73628235	-948,2702637	-0,43268
Cloud_Mesh_Distance_ICA_R0_R1	4,01600647	-89,875	-948,3342896	-0,407104
Cloud_Mesh_Distance_ICA_R0_R1	3,88052368	-89,98345947	-948,282898	-0,381496
Cloud_Mesh_Distance_ICA_R0_R1	4,18829346	-89,66870117	-948,3304443	-0,320228
Cloud_Mesh_Distance_ICA_R0_R1	4,09353638	-89,59738159	-948,2392578	-0,413105
Cloud_Mesh_Distance_ICA_R0_R1	4,05751038	-89,48027039	-948,1003418	-0,484442
Cloud_Mesh_Distance_ICA_R0_R1	4,24513245	-89,49087524	-948,2348633	-0,307903
Cloud_Mesh_Distance_ICA_R0_R1	4,9264679	-89,63504028	-948,0029907	0,130042
Cloud_Mesh_Distance_ICA_R0_R1	4,38471985	-89,39877319	-948,1742554	-0,207132
Cloud_Mesh_Distance_ICA_R0_R1	4,52500916	-89,46974182	-948,2314453	-0,112551
Cloud_Mesh_Distance_ICA_R0_R1	4,17897034	-89,3633728	-948,0448608	-0,376426
Cloud_Mesh_Distance_ICA_R0_R1	5,86132813	-92,14459229	-947,7751465	0,00059
Cloud_Mesh_Distance_ICA_R0_R1	6,10409546	-92,31764221	-947,6825562	-0,101552
Cloud_Mesh_Distance_ICA_R0_R1	2,58192444	-91,67892456	-948,0307007	0,665492
Cloud_Mesh_Distance_ICA_R0_R1	2,61198425	-91,80482483	-948,1362915	0,660991
Cloud_Mesh_Distance_ICA_R0_R1	2,8162384	-91,72839355	-948,1867676	0,451332
Cloud_Mesh_Distance_ICA_R0_R1	5,76452637	-91,96980286	-947,68573	0,04479
Cloud_Mesh_Distance_ICA_R0_R1	5,96150208	-92,09934998	-947,5831299	0,012137
Cloud_Mesh_Distance_ICA_R0_R1	2,9811554	-91,58236694	-948,1782837	0,283305
Cloud_Mesh_Distance_ICA_R0_R1	2,7587738	-91,4715271	-947,9753418	0,452356
Cloud_Mesh_Distance_ICA_R0_R1	5,49201965	-91,73617554	-947,7741089	0,081693
Cloud_Mesh_Distance_ICA_R0_R1	5,72891235	-91,8384552	-947,5510254	0,086638

Cloud_Mesh_Distance_ICA_R0_R1	2,90518188	-91,47904968	-948,0682373	0,322568
Cloud_Mesh_Distance_ICA_R0_R1	3,08569336	-91,37861633	-948,1143799	0,199408
Cloud_Mesh_Distance_ICA_R0_R1	3,05072021	-91,25267029	-948,0125732	0,194699
Cloud_Mesh_Distance_ICA_R0_R1	5,35720825	-91,41035461	-947,6555786	0,145331
Cloud_Mesh_Distance_ICA_R0_R1	5,28556824	-91,24537659	-947,6628418	0,166312
Cloud_Mesh_Distance_ICA_R0_R1	3,17703247	-91,02516174	-947,9683838	0,127701
Cloud_Mesh_Distance_ICA_R0_R1	3,17747498	-91,1463623	-948,0427856	0,157198
Cloud_Mesh_Distance_ICA_R0_R1	3,33818054	-91,03678894	-948,1154175	0,091238
Cloud_Mesh_Distance_ICA_R0_R1	5,01676941	-90,91329956	-948,0567017	0,161658
Cloud_Mesh_Distance_ICA_R0_R1	5,11943054	-91,01843262	-947,897522	0,176227
Cloud_Mesh_Distance_ICA_R0_R1	3,51596069	-90,48278809	-948,078125	-0,123562
Cloud_Mesh_Distance_ICA_R0_R1	5,07861328	-90,7381134	-947,9640503	0,198675
Cloud_Mesh_Distance_ICA_R0_R1	5,02224731	-90,57112122	-948,0726929	0,188581
Cloud_Mesh_Distance_ICA_R0_R1	5,08724976	-90,47462463	-947,9649048	0,21102
Cloud_Mesh_Distance_ICA_R0_R1	5,1398468	-90,65762329	-947,8291626	0,222407
Cloud_Mesh_Distance_ICA_R0_R1	3,66673279	-90,32072449	-948,1924439	-0,219783
Cloud_Mesh_Distance_ICA_R0_R1	3,74189758	-89,97355652	-948,137146	-0,353367
Cloud_Mesh_Distance_ICA_R0_R1	5,09931946	-89,94709778	-947,7784424	0,237187
Cloud_Mesh_Distance_ICA_R0_R1	5,09492493	-90,03970337	-947,8773193	0,234369
Cloud_Mesh_Distance_ICA_R0_R1	3,85574341	-89,81715393	-948,1807251	-0,435484
Cloud_Mesh_Distance_ICA_R0_R1	3,8321228	-89,70661926	-948,0564575	-0,48106
Cloud_Mesh_Distance_ICA_R0_R1	3,93412781	-89,67945862	-948,1647339	-0,493024
Cloud_Mesh_Distance_ICA_R0_R1	5,04066467	-89,90885925	-947,9642944	0,199201
Cloud_Mesh_Distance_ICA_R0_R1	4,7668457	-89,5163269	-948,1453247	0,038011
Cloud_Mesh_Distance_ICA_R0_R1	4,90478516	-89,54475403	-947,8991089	0,131641
Cloud_Mesh_Distance_ICA_R0_R1	4,50337219	-89,35652161	-948,1130981	-0,118746
Cloud_Mesh_Distance_ICA_R0_R1	4,64039612	-89,38189697	-948,0900879	-0,02904
Cloud_Mesh_Distance_ICA_R0_R1	6,10830688	-92,15861511	-947,4355469	0,129507
Cloud_Mesh_Distance_ICA_R0_R1	6,22338867	-92,15629578	-947,1662598	0,351996
Cloud_Mesh_Distance_ICA_R0_R1	6,23287964	-92,29270935	-947,4483643	-0,27283
Cloud_Mesh_Distance_ICA_R0_R1	2,44599915	-91,59346008	-947,8724365	0,786214
Cloud_Mesh_Distance_ICA_R0_R1	2,60322571	-91,43254089	-947,8297119	0,58821
Cloud_Mesh_Distance_ICA_R0_R1	5,78804016	-91,79283142	-947,3646851	0,109842
Cloud_Mesh_Distance_ICA_R0_R1	2,86280823	-91,25427246	-947,8648682	0,299045
Cloud_Mesh_Distance_ICA_R0_R1	2,93302917	-91,34558105	-947,9921265	0,271475
Cloud_Mesh_Distance_ICA_R0_R1	5,43760681	-91,4460144	-947,5323486	0,159152
Cloud_Mesh_Distance_ICA_R0_R1	5,59573364	-91,74684143	-947,6356201	0,09744
Cloud_Mesh_Distance_ICA_R0_R1	5,501297	-91,62931824	-947,6409302	0,121022
Cloud_Mesh_Distance_ICA_R0_R1	3,02578735	-91,13432312	-947,9054565	0,181784
Cloud_Mesh_Distance_ICA_R0_R1	5,48852539	-91,41174316	-947,3689575	0,181866
Cloud_Mesh_Distance_ICA_R0_R1	2,90319824	-91,07339478	-947,7285156	0,220619
Cloud_Mesh_Distance_ICA_R0_R1	3,04360962	-90,97750854	-947,7912598	0,146752
Cloud_Mesh_Distance_ICA_R0_R1	5,35862732	-91,28025818	-947,522583	0,185557
Cloud_Mesh_Distance_ICA_R0_R1	3,30915833	-90,83099365	-947,9886475	0,036981
Cloud_Mesh_Distance_ICA_R0_R1	5,21633911	-91,13644409	-947,7463989	0,173499
Cloud_Mesh_Distance_ICA_R0_R1	5,26843262	-90,90412903	-947,4537354	0,25219
Cloud_Mesh_Distance_ICA_R0_R1	5,23336792	-90,98149109	-947,6281738	0,221215
Cloud_Mesh_Distance_ICA_R0_R1	3,40122986	-90,63426208	-947,9974976	-0,03413
Cloud_Mesh_Distance_ICA_R0_R1	5,16836548	-90,87295532	-947,7709961	0,202335
Cloud_Mesh_Distance_ICA_R0_R1	3,47514343	-90,37994385	-947,9703369	-0,138344

Cloud_Mesh_Distance_ICA_R0_R1	3,57095337	-90,27172852	-948,0588989	-0,207164
Cloud_Mesh_Distance_ICA_R0_R1	3,4100647	-90,34858704	-947,8231201	-0,117305
Cloud_Mesh_Distance_ICA_R0_R1	3,50285339	-90,2311554	-947,9190674	-0,185462
Cloud_Mesh_Distance_ICA_R0_R1	3,45335388	-90,19856262	-947,7641602	-0,160617
Cloud_Mesh_Distance_ICA_R0_R1	3,52856445	-90,04423523	-947,7754517	-0,240311
Cloud_Mesh_Distance_ICA_R0_R1	3,63304138	-89,96000671	-947,9490356	-0,321604
Cloud_Mesh_Distance_ICA_R0_R1	3,60163879	-90,11198425	-948,0159912	-0,270099
Cloud_Mesh_Distance_ICA_R0_R1	5,1388855	-90,17855835	-947,7700195	0,258897
Cloud_Mesh_Distance_ICA_R0_R1	5,17068481	-90,24812317	-947,555481	0,288278
Cloud_Mesh_Distance_ICA_R0_R1	3,73622131	-89,81956482	-948,010376	-0,410042
Cloud_Mesh_Distance_ICA_R0_R1	3,78485107	-89,68495178	-947,9248047	-0,471329
Cloud_Mesh_Distance_ICA_R0_R1	3,91462708	-89,573349	-948,0231323	-0,562338
Cloud_Mesh_Distance_ICA_R0_R1	5,10882568	-89,95317078	-947,5809326	0,275187
Cloud_Mesh_Distance_ICA_R0_R1	5,05480957	-89,78390503	-947,682312	0,238908
Cloud_Mesh_Distance_ICA_R0_R1	3,86477661	-89,55500793	-947,8488159	-0,536318
Cloud_Mesh_Distance_ICA_R0_R1	3,98620605	-89,4493866	-947,9144898	-0,584344
Cloud_Mesh_Distance_ICA_R0_R1	4,86090088	-89,47816467	-947,8050537	0,116841
Cloud_Mesh_Distance_ICA_R0_R1	4,94787598	-89,59011841	-947,6988525	0,180175
Cloud_Mesh_Distance_ICA_R0_R1	5,01794434	-89,73258972	-947,8474121	0,189661
Cloud_Mesh_Distance_ICA_R0_R1	4,84625244	-89,4781189	-947,6641235	0,139216
Cloud_Mesh_Distance_ICA_R0_R1	4,76545715	-89,40226746	-947,9351807	0,051083
Cloud_Mesh_Distance_ICA_R0_R1	4,36369324	-89,29173279	-947,9984131	-0,208487
Cloud_Mesh_Distance_ICA_R0_R1	4,13839722	-89,3278656	-947,8175659	-0,379293
Cloud_Mesh_Distance_ICA_R0_R1	4,36080933	-89,26069641	-947,8029785	-0,175176
Cloud_Mesh_Distance_ICA_R0_R1	6,07585144	-91,9752655	-947,0501709	0,253733
Cloud_Mesh_Distance_ICA_R0_R1	5,88415527	-91,93266296	-947,4367065	0,073504
Cloud_Mesh_Distance_ICA_R0_R1	6,03260803	-92,02281189	-947,3109131	0,096874
Cloud_Mesh_Distance_ICA_R0_R1	5,64271545	-91,68986511	-947,4727783	0,12448
Cloud_Mesh_Distance_ICA_R0_R1	2,50048828	-91,36050415	-947,6585083	0,670556
Cloud_Mesh_Distance_ICA_R0_R1	5,57157898	-91,53985596	-947,3770142	0,162718
Cloud_Mesh_Distance_ICA_R0_R1	2,98535156	-90,8866272	-947,6162109	0,153607
Cloud_Mesh_Distance_ICA_R0_R1	5,29801941	-91,10768127	-947,5245361	0,216329
Cloud_Mesh_Distance_ICA_R0_R1	3,11100769	-90,81060791	-947,7183228	0,104885
Cloud_Mesh_Distance_ICA_R0_R1	3,0160675	-90,75508118	-947,4814453	0,131621
Cloud_Mesh_Distance_ICA_R0_R1	3,190979	-90,83204651	-947,8486328	0,087389
Cloud_Mesh_Distance_ICA_R0_R1	3,28646851	-90,64849854	-947,843689	0,009061
Cloud_Mesh_Distance_ICA_R0_R1	3,30569458	-90,47921753	-947,7210693	-0,036544
Cloud_Mesh_Distance_ICA_R0_R1	5,19636536	-90,62870789	-947,6247559	0,261826
Cloud_Mesh_Distance_ICA_R0_R1	5,23034668	-90,78282166	-947,5371704	0,260895
Cloud_Mesh_Distance_ICA_R0_R1	3,3855896	-90,49932861	-947,8972778	-0,068916
Cloud_Mesh_Distance_ICA_R0_R1	5,17166138	-90,45483398	-947,7103272	0,267722
Cloud_Mesh_Distance_ICA_R0_R1	3,57385254	-89,93534851	-947,6751099	-0,274025
Cloud_Mesh_Distance_ICA_R0_R1	5,14578247	-90,11071777	-947,607605	0,281561
Cloud_Mesh_Distance_ICA_R0_R1	5,12792969	-90,07095337	-947,4552612	0,293063
Cloud_Mesh_Distance_ICA_R0_R1	5,0617981	-89,86538696	-947,4699707	0,264702
Cloud_Mesh_Distance_ICA_R0_R1	3,66238403	-89,82766724	-947,8226929	-0,359618
Cloud_Mesh_Distance_ICA_R0_R1	3,66197205	-89,80131531	-947,609314	-0,327922
Cloud_Mesh_Distance_ICA_R0_R1	4,97889709	-89,68809509	-947,515686	0,225194
Cloud_Mesh_Distance_ICA_R0_R1	4,27462769	-89,29310608	-947,6867065	-0,214996
Cloud_Mesh_Distance_ICA_R0_R1	3,98709106	-89,42857361	-947,6932373	-0,471764

Cloud_Mesh_Distance_ICA_R0_R1	4,74246216	-89,3821106	-947,7301025	0,073974
Cloud_Mesh_Distance_ICA_R0_R1	4,60112	-89,29548645	-947,911438	-0,019392
Cloud_Mesh_Distance_ICA_R0_R1	5,97021484	-91,84043884	-946,9528809	0,217923
Cloud_Mesh_Distance_ICA_R0_R1	5,93348694	-91,8709259	-947,1884155	0,115627
Cloud_Mesh_Distance_ICA_R0_R1	5,83181763	-91,73136902	-947,1141968	0,167556
Cloud_Mesh_Distance_ICA_R0_R1	2,3860321	-91,30517578	-947,4503174	0,803639
Cloud_Mesh_Distance_ICA_R0_R1	2,55685425	-91,23135376	-947,5386353	0,601958
Cloud_Mesh_Distance_ICA_R0_R1	2,63069153	-91,11247253	-947,444519	0,540355
Cloud_Mesh_Distance_ICA_R0_R1	2,71382141	-91,20245361	-947,6734009	0,418733
Cloud_Mesh_Distance_ICA_R0_R1	5,48208618	-91,25146484	-947,0974121	0,245459
Cloud_Mesh_Distance_ICA_R0_R1	5,69972229	-91,62440491	-947,2275391	0,170703
Cloud_Mesh_Distance_ICA_R0_R1	5,58473206	-91,44721985	-947,1616821	0,212166
Cloud_Mesh_Distance_ICA_R0_R1	2,71824646	-90,99801636	-947,3731079	0,443361
Cloud_Mesh_Distance_ICA_R0_R1	2,89320374	-90,87358093	-947,4526367	0,233727
Cloud_Mesh_Distance_ICA_R0_R1	2,77391052	-91,05419922	-947,5473633	0,365126
Cloud_Mesh_Distance_ICA_R0_R1	5,40574646	-91,24455261	-947,3467407	0,210435
Cloud_Mesh_Distance_ICA_R0_R1	3,17552185	-90,66542053	-947,6676636	0,047986
Cloud_Mesh_Distance_ICA_R0_R1	3,2097168	-90,53666687	-947,5604858	0,025436
Cloud_Mesh_Distance_ICA_R0_R1	5,31260681	-90,96905518	-947,3249512	0,250704
Cloud_Mesh_Distance_ICA_R0_R1	5,30743408	-90,84785461	-947,1364136	0,281775
Cloud_Mesh_Distance_ICA_R0_R1	3,28773499	-90,40608215	-947,5530396	-0,03663
Cloud_Mesh_Distance_ICA_R0_R1	5,22344971	-90,57781982	-947,3822632	0,2808
Cloud_Mesh_Distance_ICA_R0_R1	5,25799561	-90,72639465	-947,2981567	0,277331
Cloud_Mesh_Distance_ICA_R0_R1	5,25015259	-90,66090393	-947,0595703	0,310261
Cloud_Mesh_Distance_ICA_R0_R1	3,36701965	-90,31069946	-947,65448	-0,098913
Cloud_Mesh_Distance_ICA_R0_R1	3,34953308	-90,27658081	-947,4516602	-0,085844
Cloud_Mesh_Distance_ICA_R0_R1	3,44200134	-90,14404297	-947,3718262	-0,130995
Cloud_Mesh_Distance_ICA_R0_R1	3,43110657	-90,16790771	-947,5985107	-0,149927
Cloud_Mesh_Distance_ICA_R0_R1	5,1953125	-90,41848755	-947,4662476	0,291445
Cloud_Mesh_Distance_ICA_R0_R1	5,14956665	-90,23976135	-947,3156128	0,303487
Cloud_Mesh_Distance_ICA_R0_R1	5,2179718	-90,55140686	-947,1656494	0,30681
Cloud_Mesh_Distance_ICA_R0_R1	5,19259644	-90,42326355	-947,2811279	0,309235
Cloud_Mesh_Distance_ICA_R0_R1	5,15800476	-90,42073059	-947,052124	0,304764
Cloud_Mesh_Distance_ICA_R0_R1	3,53656006	-90,02090454	-947,3752441	-0,197897
Cloud_Mesh_Distance_ICA_R0_R1	3,53881836	-89,98440552	-947,5425415	-0,228812
Cloud_Mesh_Distance_ICA_R0_R1	3,756073	-89,67237854	-947,7495728	-0,434408
Cloud_Mesh_Distance_ICA_R0_R1	3,77490234	-89,66038513	-947,5876465	-0,391819
Cloud_Mesh_Distance_ICA_R0_R1	3,85342407	-89,554245	-947,69104	-0,481274
Cloud_Mesh_Distance_ICA_R0_R1	3,93447876	-89,53382874	-947,4940186	-0,380962
Cloud_Mesh_Distance_ICA_R0_R1	4,95533752	-89,78401184	-947,3245239	0,246645
Cloud_Mesh_Distance_ICA_R0_R1	4,87911987	-89,54830933	-947,5459595	0,177582
Cloud_Mesh_Distance_ICA_R0_R1	4,15922546	-89,345047	-947,6447144	-0,301759
Cloud_Mesh_Distance_ICA_R0_R1	4,30215454	-89,34260559	-947,553894	-0,171083
Cloud_Mesh_Distance_ICA_R0_R1	4,62866211	-89,35832214	-947,5985107	0,035517
Cloud_Mesh_Distance_ICA_R0_R1	4,59895325	-89,30718994	-947,7317505	-0,013902
Cloud_Mesh_Distance_ICA_R0_R1	4,46720886	-89,28219604	-947,6847534	-0,073327
Cloud_Mesh_Distance_ICA_R0_R1	4,7633667	-89,44921875	-947,5501709	0,11526
Cloud_Mesh_Distance_ICA_R0_R1	4,63818359	-89,43548584	-947,4552612	0,062956
Cloud_Mesh_Distance_ICA_R0_R1	6,02520752	-91,87652588	-946,8073731	0,331767
Cloud_Mesh_Distance_ICA_R0_R1	5,77674866	-91,6267395	-946,9824829	0,204

Cloud_Mesh_Distance_ICA_R0_R1	5,63217163	-91,42990112	-946,9517212	0,246243
Cloud_Mesh_Distance_ICA_R0_R1	5,47096252	-91,15414429	-946,9138794	0,278981
Cloud_Mesh_Distance_ICA_R0_R1	5,36035156	-90,94534302	-946,9934082	0,291841
Cloud_Mesh_Distance_ICA_R0_R1	5,38026428	-90,95573425	-946,8386231	0,299946
Cloud_Mesh_Distance_ICA_R0_R1	5,36247253	-91,03205872	-947,1882935	0,256036
Cloud_Mesh_Distance_ICA_R0_R1	2,85848999	-90,7895813	-947,1968384	0,255794
Cloud_Mesh_Distance_ICA_R0_R1	3,08987427	-90,6219635	-947,3977051	0,086892
Cloud_Mesh_Distance_ICA_R0_R1	3,23008728	-90,43321228	-947,3672485	-0,003772
Cloud_Mesh_Distance_ICA_R0_R1	3,1396637	-90,50242615	-947,1722412	0,054909
Cloud_Mesh_Distance_ICA_R0_R1	5,06900024	-90,18969727	-947,1003418	0,297159
Cloud_Mesh_Distance_ICA_R0_R1	5,04920959	-89,95552063	-947,3099976	0,281355
Cloud_Mesh_Distance_ICA_R0_R1	3,64611816	-89,87283325	-947,4167481	-0,26884
Cloud_Mesh_Distance_ICA_R0_R1	3,77285767	-89,7915802	-947,3156738	-0,2876
Cloud_Mesh_Distance_ICA_R0_R1	3,77616882	-89,70428467	-947,4587402	-0,346764
Cloud_Mesh_Distance_ICA_R0_R1	4,97917175	-89,96034241	-947,1759033	0,266818
Cloud_Mesh_Distance_ICA_R0_R1	4,11889648	-89,53495789	-947,3429565	-0,228172
Cloud_Mesh_Distance_ICA_R0_R1	4,34306335	-89,55137634	-947,2623291	-0,073769
Cloud_Mesh_Distance_ICA_R0_R1	4,0644989	-89,70765686	-947,1710815	-0,181728
Cloud_Mesh_Distance_ICA_R0_R1	4,19987488	-89,65332031	-947,1802368	-0,133261
Cloud_Mesh_Distance_ICA_R0_R1	4,83375549	-89,57691956	-947,4118652	0,176854
Cloud_Mesh_Distance_ICA_R0_R1	4,79295349	-89,8249054	-947,1135254	0,188486
Cloud_Mesh_Distance_ICA_R0_R1	4,11326599	-89,41738892	-947,5101929	-0,283876
Cloud_Mesh_Distance_ICA_R0_R1	4,31756592	-89,42936707	-947,4119263	-0,124895
Cloud_Mesh_Distance_ICA_R0_R1	4,53630066	-89,60424805	-947,2174072	0,042201
Cloud_Mesh_Distance_ICA_R0_R1	4,50230408	-89,47427368	-947,355835	0,003027
Cloud_Mesh_Distance_ICA_R0_R1	4,81419373	-89,66139221	-947,2943115	0,174947
Cloud_Mesh_Distance_ICA_R0_R1	4,69903564	-89,68588257	-947,1915894	0,136189
Cloud_Mesh_Distance_ICA_R0_R1	4,47576904	-89,36044312	-947,5140991	-0,040425
Cloud_Mesh_Distance_ICA_R0_R1	4,68141174	-89,55003357	-947,324707	0,111575
Cloud_Mesh_Distance_ICA_R0_R1	5,86080933	-91,66816711	-946,5997925	0,328894
Cloud_Mesh_Distance_ICA_R0_R1	2,30485535	-91,2900238	-947,3034668	0,927207
Cloud_Mesh_Distance_ICA_R0_R1	2,46699524	-91,18266296	-947,3339233	0,744883
Cloud_Mesh_Distance_ICA_R0_R1	2,33366394	-91,21615601	-947,1693726	0,904201
Cloud_Mesh_Distance_ICA_R0_R1	5,64326477	-91,39382935	-946,7635498	0,281179
Cloud_Mesh_Distance_ICA_R0_R1	5,88587952	-91,71922302	-946,8233032	0,235434
Cloud_Mesh_Distance_ICA_R0_R1	5,76292419	-91,55780029	-946,7615356	0,251949
Cloud_Mesh_Distance_ICA_R0_R1	2,57720947	-91,05381775	-947,239502	0,613924
Cloud_Mesh_Distance_ICA_R0_R1	5,54910278	-91,27684021	-946,8704224	0,274
Cloud_Mesh_Distance_ICA_R0_R1	5,58869934	-91,27540588	-946,5230103	0,313281
Cloud_Mesh_Distance_ICA_R0_R1	5,53700256	-91,21134949	-946,6693115	0,302745
Cloud_Mesh_Distance_ICA_R0_R1	2,73925781	-90,92295837	-947,2546997	0,41187
Cloud_Mesh_Distance_ICA_R0_R1	2,74282837	-90,86830139	-947,1151733	0,37488
Cloud_Mesh_Distance_ICA_R0_R1	2,9382782	-90,68609619	-947,1003418	0,151905
Cloud_Mesh_Distance_ICA_R0_R1	2,99830627	-90,66456604	-947,2375488	0,122258
Cloud_Mesh_Distance_ICA_R0_R1	5,31518555	-90,82235718	-946,7282715	0,310079
Cloud_Mesh_Distance_ICA_R0_R1	5,28971863	-90,76881409	-946,9515991	0,308225
Cloud_Mesh_Distance_ICA_R0_R1	3,33679199	-90,31504822	-947,0080566	-0,028331
Cloud_Mesh_Distance_ICA_R0_R1	3,1837616	-90,4467926	-947,0239258	0,04867
Cloud_Mesh_Distance_ICA_R0_R1	3,2858429	-90,35035706	-947,2070313	-0,020053
Cloud_Mesh_Distance_ICA_R0_R1	3,47846985	-90,20614624	-946,9902344	-0,085967

Cloud_Mesh_Distance_ICA_R0_R1	5,20295715	-90,60528564	-946,8269653	0,307183
Cloud_Mesh_Distance_ICA_R0_R1	3,40734863	-90,22621155	-947,1430054	-0,080503
Cloud_Mesh_Distance_ICA_R0_R1	5,0916748	-90,37556458	-946,9052124	0,301187
Cloud_Mesh_Distance_ICA_R0_R1	3,5158844	-90,11323547	-947,1676025	-0,13786
Cloud_Mesh_Distance_ICA_R0_R1	3,66920471	-89,96101379	-947,19104	-0,206496
Cloud_Mesh_Distance_ICA_R0_R1	3,99229431	-89,93678284	-946,9415283	-0,146195
Cloud_Mesh_Distance_ICA_R0_R1	4,92918396	-90,02301025	-947,0458374	0,246419
Cloud_Mesh_Distance_ICA_R0_R1	4,93334961	-90,16275024	-946,9075317	0,248899
Cloud_Mesh_Distance_ICA_R0_R1	3,80732727	-89,86209106	-947,1699829	-0,228012
Cloud_Mesh_Distance_ICA_R0_R1	3,94137573	-89,83634949	-947,0925293	-0,203977
Cloud_Mesh_Distance_ICA_R0_R1	3,92338562	-89,71340942	-947,2531738	-0,257097
Cloud_Mesh_Distance_ICA_R0_R1	4,11013794	-89,85145569	-946,9989014	-0,123301
Cloud_Mesh_Distance_ICA_R0_R1	4,28349304	-89,92417908	-946,8855591	-0,04439
Cloud_Mesh_Distance_ICA_R0_R1	4,56004333	-89,95909119	-946,8780518	0,089287
Cloud_Mesh_Distance_ICA_R0_R1	4,65093994	-89,86190796	-947,0030518	0,128012
Cloud_Mesh_Distance_ICA_R0_R1	4,24719238	-89,77116394	-947,0497437	-0,076958
Cloud_Mesh_Distance_ICA_R0_R1	4,39239502	-89,68534851	-947,1240234	-0,018682
Cloud_Mesh_Distance_ICA_R0_R1	4,57777405	-89,73851013	-947,0957031	0,0794
Cloud_Mesh_Distance_ICA_R0_R1	5,68841553	-91,43867493	-946,6383057	0,290427
Cloud_Mesh_Distance_ICA_R0_R1	5,68742371	-91,42375183	-946,4545898	0,35068
Cloud_Mesh_Distance_ICA_R0_R1	5,8396759	-91,64517212	-946,3966065	0,425248
Cloud_Mesh_Distance_ICA_R0_R1	5,76730347	-91,54122925	-946,5302734	0,32018
Cloud_Mesh_Distance_ICA_R0_R1	2,43409729	-91,10700989	-947,0709229	0,745603
Cloud_Mesh_Distance_ICA_R0_R1	2,6068573	-90,96238708	-947,0457153	0,523012
Cloud_Mesh_Distance_ICA_R0_R1	2,82437134	-90,76689148	-947,0080566	0,248605
Cloud_Mesh_Distance_ICA_R0_R1	3,04328918	-90,56944275	-946,9998169	0,116929
Cloud_Mesh_Distance_ICA_R0_R1	2,91897583	-90,66424561	-946,8978882	0,147459
Cloud_Mesh_Distance_ICA_R0_R1	5,41259766	-90,99191284	-946,6582642	0,310913
Cloud_Mesh_Distance_ICA_R0_R1	3,01965332	-90,57067871	-946,7727051	0,111735
Cloud_Mesh_Distance_ICA_R0_R1	5,05693054	-90,4981842	-946,5886231	0,271836
Cloud_Mesh_Distance_ICA_R0_R1	5,25846863	-90,74357605	-946,6028442	0,30383
Cloud_Mesh_Distance_ICA_R0_R1	5,15063477	-90,63739014	-946,4699707	0,281862
Cloud_Mesh_Distance_ICA_R0_R1	5,36039734	-90,90632629	-946,4787598	0,309961
Cloud_Mesh_Distance_ICA_R0_R1	5,25389099	-90,77684021	-946,3546753	0,289081
Cloud_Mesh_Distance_ICA_R0_R1	5,15888977	-90,59391785	-946,644104	0,303785
Cloud_Mesh_Distance_ICA_R0_R1	5,10542297	-90,48655701	-946,7381592	0,296473
Cloud_Mesh_Distance_ICA_R0_R1	4,99545288	-90,35954285	-946,734436	0,264995
Cloud_Mesh_Distance_ICA_R0_R1	3,5770874	-90,2051239	-946,7998047	-0,077065
Cloud_Mesh_Distance_ICA_R0_R1	3,97920227	-90,10517883	-946,7009888	-0,099858
Cloud_Mesh_Distance_ICA_R0_R1	4,07182312	-90,02590942	-946,7967529	-0,087001
Cloud_Mesh_Distance_ICA_R0_R1	4,38223267	-90,10836792	-946,6596069	0,023716
Cloud_Mesh_Distance_ICA_R0_R1	4,26977539	-90,03540039	-946,75354	-0,028182
Cloud_Mesh_Distance_ICA_R0_R1	4,69543457	-90,17279053	-946,68927	0,157183
Cloud_Mesh_Distance_ICA_R0_R1	4,83625793	-90,24029541	-946,7059326	0,21493
Cloud_Mesh_Distance_ICA_R0_R1	4,92210388	-90,38905334	-946,5662842	0,229536
Cloud_Mesh_Distance_ICA_R0_R1	3,66680908	-90,06842041	-946,9953613	-0,151771
Cloud_Mesh_Distance_ICA_R0_R1	3,88713074	-90,0279541	-946,8703003	-0,133478
Cloud_Mesh_Distance_ICA_R0_R1	3,82640076	-89,94316101	-947,0390015	-0,181307
Cloud_Mesh_Distance_ICA_R0_R1	4,42935181	-90,00436401	-946,8026733	0,041258
Cloud_Mesh_Distance_ICA_R0_R1	4,74638367	-90,03974915	-946,8777466	0,176743

Cloud_Mesh_Distance_ICA_R0_R1	4,57241821	-90,09204102	-946,7327271	0,095388
Cloud_Mesh_Distance_ICA_R0_R1	4,45309448	-89,83818054	-946,979126	0,030437
Cloud_Mesh_Distance_ICA_R0_R1	2,25228882	-91,2199707	-946,942688	0,933442
Cloud_Mesh_Distance_ICA_R0_R1	5,75198364	-91,52388	-946,3338013	0,41552
Cloud_Mesh_Distance_ICA_R0_R1	2,36335754	-91,11470032	-946,8218384	0,765526
Cloud_Mesh_Distance_ICA_R0_R1	2,4822998	-91,03404236	-946,9182739	0,641352
Cloud_Mesh_Distance_ICA_R0_R1	2,5639801	-90,93605042	-946,6035156	0,510063
Cloud_Mesh_Distance_ICA_R0_R1	2,58041382	-90,93766785	-946,8192749	0,49479
Cloud_Mesh_Distance_ICA_R0_R1	2,80076599	-90,7510376	-946,8007202	0,232607
Cloud_Mesh_Distance_ICA_R0_R1	2,70309448	-90,85173035	-946,9176025	0,369148
Cloud_Mesh_Distance_ICA_R0_R1	2,79046631	-90,74536133	-946,5800781	0,249617
Cloud_Mesh_Distance_ICA_R0_R1	2,68202209	-90,84062195	-946,7023315	0,354638
Cloud_Mesh_Distance_ICA_R0_R1	5,47599792	-91,0925293	-946,5030518	0,309967
Cloud_Mesh_Distance_ICA_R0_R1	2,90583801	-90,65525818	-946,6800537	0,144756
Cloud_Mesh_Distance_ICA_R0_R1	3,18240356	-90,44758606	-946,8664551	0,058626
Cloud_Mesh_Distance_ICA_R0_R1	5,08349609	-90,62864685	-946,2570801	0,238066
Cloud_Mesh_Distance_ICA_R0_R1	5,35639954	-90,91436768	-946,3179932	0,292691
Cloud_Mesh_Distance_ICA_R0_R1	3,23493958	-90,41932678	-946,7012329	0,054749
Cloud_Mesh_Distance_ICA_R0_R1	3,35298157	-90,34111023	-946,7570801	0,003572
Cloud_Mesh_Distance_ICA_R0_R1	4,98495483	-90,51651001	-946,3794556	0,22666
Cloud_Mesh_Distance_ICA_R0_R1	3,77757263	-90,12203979	-946,7801514	-0,108744
Cloud_Mesh_Distance_ICA_R0_R1	4,23692322	-90,20387268	-946,4766846	-0,011479
Cloud_Mesh_Distance_ICA_R0_R1	4,51112366	-90,16714478	-946,6056519	0,079852
Cloud_Mesh_Distance_ICA_R0_R1	4,79005432	-90,3434906	-946,4904175	0,184632
Cloud_Mesh_Distance_ICA_R0_R1	2,1884613	-91,28764343	-946,3994141	1,037085
Cloud_Mesh_Distance_ICA_R0_R1	2,24813843	-91,20085144	-946,7098999	0,901013
Cloud_Mesh_Distance_ICA_R0_R1	2,35691833	-91,1295929	-946,4177246	0,810816
Cloud_Mesh_Distance_ICA_R0_R1	2,35916138	-91,11009216	-946,6204224	0,765286
Cloud_Mesh_Distance_ICA_R0_R1	2,4647522	-91,02354431	-946,7197266	0,625442
Cloud_Mesh_Distance_ICA_R0_R1	2,45539856	-91,03068542	-946,5274048	0,659872
Cloud_Mesh_Distance_ICA_R0_R1	5,56808472	-91,25073242	-946,0941162	0,296032
Cloud_Mesh_Distance_ICA_R0_R1	5,66046143	-91,39118958	-946,2145996	0,338232
Cloud_Mesh_Distance_ICA_R0_R1	5,57449341	-91,24942017	-946,3129273	0,309496
Cloud_Mesh_Distance_ICA_R0_R1	2,67396545	-90,84547424	-946,4754028	0,399277
Cloud_Mesh_Distance_ICA_R0_R1	2,79138184	-90,75540161	-946,3604126	0,285678
Cloud_Mesh_Distance_ICA_R0_R1	5,43914795	-91,03691101	-946,3134155	0,297243
Cloud_Mesh_Distance_ICA_R0_R1	2,96572876	-90,61204529	-946,4243774	0,14568
Cloud_Mesh_Distance_ICA_R0_R1	3,01525879	-90,57170105	-946,5608521	0,135712
Cloud_Mesh_Distance_ICA_R0_R1	3,12417603	-90,49458313	-946,6342773	0,101055
Cloud_Mesh_Distance_ICA_R0_R1	3,18798828	-90,47169495	-946,3783569	0,100771
Cloud_Mesh_Distance_ICA_R0_R1	3,40901184	-90,36759949	-946,3823242	0,04689
Cloud_Mesh_Distance_ICA_R0_R1	3,29364014	-90,40107727	-946,5302124	0,061318
Cloud_Mesh_Distance_ICA_R0_R1	3,70727539	-90,21685791	-946,6092529	-0,067435
Cloud_Mesh_Distance_ICA_R0_R1	3,49211121	-90,3039856	-946,5750732	-0,00834
Cloud_Mesh_Distance_ICA_R0_R1	4,79403687	-90,42591858	-946,3049316	0,164782
Cloud_Mesh_Distance_ICA_R0_R1	3,85845947	-90,20152283	-946,548523	-0,070233
Cloud_Mesh_Distance_ICA_R0_R1	4,12886047	-90,13983154	-946,6028442	-0,047641
Cloud_Mesh_Distance_ICA_R0_R1	4,62187195	-90,27314758	-946,4837647	0,108142
Cloud_Mesh_Distance_ICA_R0_R1	4,40991211	-90,22251892	-946,475708	0,041572
Cloud_Mesh_Distance_ICA_R0_R1	2,16209412	-91,35470581	-946,269165	1,118032

Cloud_Mesh_Distance_ICA_R0_R1	5,57618713	-91,2744751	-945,9005737	0,314525
Cloud_Mesh_Distance_ICA_R0_R1	2,69322205	-90,86155701	-946,230835	0,428893
Cloud_Mesh_Distance_ICA_R0_R1	5,46336365	-91,08999634	-945,9285889	0,213058
Cloud_Mesh_Distance_ICA_R0_R1	2,54866028	-90,96655273	-946,3694458	0,576835
Cloud_Mesh_Distance_ICA_R0_R1	5,32516479	-90,90155029	-945,9702759	0,210082
Cloud_Mesh_Distance_ICA_R0_R1	5,47956848	-91,10658264	-946,1574707	0,279875
Cloud_Mesh_Distance_ICA_R0_R1	5,23542786	-90,80769348	-945,8191528	0,144884
Cloud_Mesh_Distance_ICA_R0_R1	5,11915588	-90,70593262	-945,8806152	0,153264
Cloud_Mesh_Distance_ICA_R0_R1	5,34677124	-90,91685486	-946,1607056	0,264877
Cloud_Mesh_Distance_ICA_R0_R1	5,21478271	-90,76544189	-946,15979	0,235695
Cloud_Mesh_Distance_ICA_R0_R1	3,02941895	-90,57951355	-946,2962647	0,133787
Cloud_Mesh_Distance_ICA_R0_R1	5,01991272	-90,62443542	-946,0330811	0,172024
Cloud_Mesh_Distance_ICA_R0_R1	4,85948181	-90,54721069	-945,9147339	0,109832
Cloud_Mesh_Distance_ICA_R0_R1	4,89816284	-90,52844238	-946,1488648	0,160605
Cloud_Mesh_Distance_ICA_R0_R1	3,60606384	-90,33796692	-946,2072754	0,027878
Cloud_Mesh_Distance_ICA_R0_R1	3,39456177	-90,40405273	-946,1619873	0,084432
Cloud_Mesh_Distance_ICA_R0_R1	3,63929749	-90,29656982	-946,3841553	-0,006514
Cloud_Mesh_Distance_ICA_R0_R1	4,43559265	-90,33799744	-946,2076416	0,04141
Cloud_Mesh_Distance_ICA_R0_R1	4,5919342	-90,41853333	-946,0668335	0,07515
Cloud_Mesh_Distance_ICA_R0_R1	4,76078796	-90,47489929	-946,0908814	0,118198
Cloud_Mesh_Distance_ICA_R0_R1	4,58824158	-90,33679199	-946,3234863	0,100045
Cloud_Mesh_Distance_ICA_R0_R1	3,94116211	-90,24198914	-946,3990479	-0,038989
Cloud_Mesh_Distance_ICA_R0_R1	4,13102722	-90,27056885	-946,2998047	-0,019368
Cloud_Mesh_Distance_ICA_R0_R1	4,37846375	-90,27770996	-946,3411255	0,032617
Cloud_Mesh_Distance_ICA_R0_R1	4,26028442	-90,30549622	-946,2224121	0,007192
Cloud_Mesh_Distance_ICA_R0_R1	2,29699707	-91,25186157	-946,203125	0,96231
Cloud_Mesh_Distance_ICA_R0_R1	2,45812988	-91,08847046	-946,2190552	0,738695
Cloud_Mesh_Distance_ICA_R0_R1	2,5381012	-91,09986877	-946,0083008	0,717224
Cloud_Mesh_Distance_ICA_R0_R1	5,46044922	-91,05218506	-945,6401978	0,124136
Cloud_Mesh_Distance_ICA_R0_R1	2,8503418	-90,72892761	-946,2102661	0,249502
Cloud_Mesh_Distance_ICA_R0_R1	5,33436584	-90,9100647	-945,8225708	0,161476
Cloud_Mesh_Distance_ICA_R0_R1	3,27664185	-90,4433136	-946,2386475	0,096426
Cloud_Mesh_Distance_ICA_R0_R1	3,1124115	-90,54325867	-946,1806641	0,121961
Cloud_Mesh_Distance_ICA_R0_R1	3,17356873	-90,55274963	-945,9417114	0,126343
Cloud_Mesh_Distance_ICA_R0_R1	4,51741028	-90,42796326	-945,8985596	0,035577
Cloud_Mesh_Distance_ICA_R0_R1	4,67729187	-90,47148132	-945,932251	0,071798
Cloud_Mesh_Distance_ICA_R0_R1	4,99638367	-90,62536621	-945,7144775	0,087841
Cloud_Mesh_Distance_ICA_R0_R1	3,48377991	-90,39982605	-945,9958496	0,078859
Cloud_Mesh_Distance_ICA_R0_R1	4,01135254	-90,29910278	-946,1913452	-0,013381
Cloud_Mesh_Distance_ICA_R0_R1	3,79089355	-90,31071472	-946,1958008	0,003282
Cloud_Mesh_Distance_ICA_R0_R1	3,93685913	-90,33699036	-946,0064697	0,010726
Cloud_Mesh_Distance_ICA_R0_R1	4,17538452	-90,35240173	-945,9844971	0,003587
Cloud_Mesh_Distance_ICA_R0_R1	4,38566589	-90,37394714	-946,032898	0,032477
Cloud_Mesh_Distance_ICA_R0_R1	2,40231323	-91,20680237	-946,0792847	0,873094
Cloud_Mesh_Distance_ICA_R0_R1	2,67190552	-90,94494629	-946,0241699	0,523362
Cloud_Mesh_Distance_ICA_R0_R1	2,94781494	-90,69093323	-946,0393677	0,196411
Cloud_Mesh_Distance_ICA_R0_R1	2,79550171	-90,83903503	-946,0009155	0,37617
Cloud_Mesh_Distance_ICA_R0_R1	2,96679688	-90,7454834	-945,8526611	0,229753
Cloud_Mesh_Distance_ICA_R0_R1	3,25863647	-90,57099915	-945,7366943	0,117664
Cloud_Mesh_Distance_ICA_R0_R1	3,44015503	-90,46290588	-945,7843628	0,100377

Cloud_Mesh_Distance_ICA_R0_R1	5,12478638	-90,70681763	-945,7280273	0,101391
Cloud_Mesh_Distance_ICA_R0_R1	3,7350769	-90,34616089	-946,0273438	0,030878
Cloud_Mesh_Distance_ICA_R0_R1	2,79292297	-90,92555237	-945,8365479	0,451102
Cloud_Mesh_Distance_ICA_R0_R1	2,9493866	-90,87205505	-945,6713257	0,292813
Cloud_Mesh_Distance_ICA_R0_R1	3,18939209	-90,69908142	-945,5678711	0,105786
Cloud_Mesh_Distance_ICA_R0_R1	3,63450623	-90,39387512	-945,8204956	0,068421
Cloud_Mesh_Distance_ICA_R0_R1	3,23426819	-90,7613678	-945,3986206	0,092791
Cloud_Mesh_Distance_ICA_R0_R1	3,09448242	-90,81025696	-945,5421143	0,137525
Cloud_Mesh_Distance_ICA_R0_R1	3,09362793	-90,70158386	-945,7160034	0,137024
Cloud_Mesh_Distance_ICA_R0_R2	3,65478134	-92,99913025	-948,8163452	0,162305
Cloud_Mesh_Distance_ICA_R0_R2	3,99612808	-92,92596436	-948,8585815	0,105746
Cloud_Mesh_Distance_ICA_R0_R2	4,18083572	-92,98607635	-948,8724976	0,069774
Cloud_Mesh_Distance_ICA_R0_R2	4,38303757	-93,04194641	-948,8760376	0,032894
Cloud_Mesh_Distance_ICA_R0_R2	4,62558365	-93,02924347	-948,8566284	-0,085172
Cloud_Mesh_Distance_ICA_R0_R2	3,66471481	-92,81743622	-948,8104858	0,165862
Cloud_Mesh_Distance_ICA_R0_R2	3,67618179	-92,68205261	-948,7942505	0,165248
Cloud_Mesh_Distance_ICA_R0_R2	4,00018692	-92,73327637	-948,835144	0,105454
Cloud_Mesh_Distance_ICA_R0_R2	3,82117081	-92,86949158	-948,836792	0,137813
Cloud_Mesh_Distance_ICA_R0_R2	4,18488312	-92,78395844	-948,8491821	0,066191
Cloud_Mesh_Distance_ICA_R0_R2	4,34732056	-92,85037231	-948,8569336	0,042448
Cloud_Mesh_Distance_ICA_R0_R2	4,79211044	-92,91704559	-948,8189087	-0,013833
Cloud_Mesh_Distance_ICA_R0_R2	4,92055893	-92,92572021	-948,7894898	-0,025057
Cloud_Mesh_Distance_ICA_R0_R2	4,00080872	-92,57242584	-948,8018799	0,108335
Cloud_Mesh_Distance_ICA_R0_R2	3,88918304	-92,54143524	-948,7888794	0,129326
Cloud_Mesh_Distance_ICA_R0_R2	3,82625961	-92,68197632	-948,8132935	0,138971
Cloud_Mesh_Distance_ICA_R0_R2	4,58698273	-92,59277344	-948,7797852	0,007325
Cloud_Mesh_Distance_ICA_R0_R2	4,58595657	-92,80403137	-948,8309937	0,004632
Cloud_Mesh_Distance_ICA_R0_R2	4,80083466	-92,73069763	-948,7800293	-0,015983
Cloud_Mesh_Distance_ICA_R0_R2	4,95019913	-92,80348206	-948,7606812	-0,025256
Cloud_Mesh_Distance_ICA_R0_R2	5,08488464	-92,80125427	-948,7207031	-0,021309
Cloud_Mesh_Distance_ICA_R0_R2	5,17352676	-92,87030029	-948,7032471	-0,086655
Cloud_Mesh_Distance_ICA_R0_R2	5,04312515	-92,938797	-948,7565308	-0,035223
Cloud_Mesh_Distance_ICA_R0_R2	4,1072998	-92,45706177	-948,7748413	0,089786
Cloud_Mesh_Distance_ICA_R0_R2	4,39353561	-92,46499634	-948,763916	0,035764
Cloud_Mesh_Distance_ICA_R0_R2	4,19063568	-92,57518005	-948,8046265	0,072498
Cloud_Mesh_Distance_ICA_R0_R2	4,58657837	-92,41552734	-948,7255859	0,009167
Cloud_Mesh_Distance_ICA_R0_R2	4,396698	-92,65684509	-948,8157959	0,030189
Cloud_Mesh_Distance_ICA_R0_R2	4,81412125	-92,60069275	-948,7424927	-0,009875
Cloud_Mesh_Distance_ICA_R0_R2	5,04616547	-92,70100403	-948,7093506	-0,021645
Cloud_Mesh_Distance_ICA_R0_R2	5,33046341	-92,89511108	-948,6473999	-0,232387
Cloud_Mesh_Distance_ICA_R0_R2	3,60256958	-92,58728027	-948,7645264	0,178687
Cloud_Mesh_Distance_ICA_R0_R2	3,75898743	-92,56227112	-948,7811279	0,152497
Cloud_Mesh_Distance_ICA_R0_R2	3,87869644	-92,43183899	-948,7592163	0,127544
Cloud_Mesh_Distance_ICA_R0_R2	3,99040604	-92,46304321	-948,7741699	0,110939
Cloud_Mesh_Distance_ICA_R0_R2	4,70846558	-92,64678955	-948,7768555	-0,00736
Cloud_Mesh_Distance_ICA_R0_R2	4,72713852	-92,52245331	-948,7369385	-0,001121
Cloud_Mesh_Distance_ICA_R0_R2	5,18600845	-92,71588135	-948,6638794	-0,022792
Cloud_Mesh_Distance_ICA_R0_R2	5,35042191	-92,68593597	-948,5877075	-0,044413
Cloud_Mesh_Distance_ICA_R0_R2	5,4917984	-92,81396484	-948,5576172	-0,116915
Cloud_Mesh_Distance_ICA_R0_R2	3,70259857	-92,4249115	-948,7391968	0,163677

Cloud_Mesh_Distance_ICA_R0_R2	3,66746521	-92,23204041	-948,6768189	0,167024
Cloud_Mesh_Distance_ICA_R0_R2	4,23921204	-92,38632202	-948,7506104	0,062078
Cloud_Mesh_Distance_ICA_R0_R2	4,88806534	-92,48687744	-948,6879883	-0,011523
Cloud_Mesh_Distance_ICA_R0_R2	4,75879288	-92,40422821	-948,6896973	0,002358
Cloud_Mesh_Distance_ICA_R0_R2	4,93016434	-92,6426239	-948,7269898	-0,021176
Cloud_Mesh_Distance_ICA_R0_R2	5,05658722	-92,56748962	-948,6659546	-0,014837
Cloud_Mesh_Distance_ICA_R0_R2	3,54970169	-92,12135315	-948,619751	0,182599
Cloud_Mesh_Distance_ICA_R0_R2	3,87958527	-92,26911926	-948,7107544	0,129863
Cloud_Mesh_Distance_ICA_R0_R2	4,08349228	-92,13743591	-948,6740723	0,095432
Cloud_Mesh_Distance_ICA_R0_R2	4,06987762	-92,32859802	-948,7363892	0,099343
Cloud_Mesh_Distance_ICA_R0_R2	4,41688538	-92,34377289	-948,7235107	0,039691
Cloud_Mesh_Distance_ICA_R0_R2	4,30149078	-92,19671631	-948,6843872	0,063274
Cloud_Mesh_Distance_ICA_R0_R2	4,51358414	-92,23795319	-948,6741943	0,027598
Cloud_Mesh_Distance_ICA_R0_R2	5,22037125	-92,52355957	-948,5889893	-0,012901
Cloud_Mesh_Distance_ICA_R0_R2	3,68356323	-92,07771301	-948,6271973	0,161362
Cloud_Mesh_Distance_ICA_R0_R2	3,88022614	-92,05766296	-948,6416626	0,126545
Cloud_Mesh_Distance_ICA_R0_R2	4,10936356	-91,8519516	-948,5783081	0,092806
Cloud_Mesh_Distance_ICA_R0_R2	4,44915009	-92,13572693	-948,6468506	0,040616
Cloud_Mesh_Distance_ICA_R0_R2	4,41718292	-92,0431366	-948,6187134	0,04558
Cloud_Mesh_Distance_ICA_R0_R2	4,89561462	-92,2113266	-948,5764771	0,017523
Cloud_Mesh_Distance_ICA_R0_R2	4,72327805	-92,26730347	-948,6460571	0,016159
Cloud_Mesh_Distance_ICA_R0_R2	5,04099274	-92,36812592	-948,5961304	0,000144
Cloud_Mesh_Distance_ICA_R0_R2	4,86404037	-92,34277344	-948,6405029	0,002929
Cloud_Mesh_Distance_ICA_R0_R2	5,20350266	-92,37302399	-948,532898	0,006859
Cloud_Mesh_Distance_ICA_R0_R2	3,74929047	-91,95839691	-948,5935059	0,147319
Cloud_Mesh_Distance_ICA_R0_R2	4,09862518	-91,97332764	-948,6195068	0,0934
Cloud_Mesh_Distance_ICA_R0_R2	4,24569702	-91,87677002	-948,5791016	0,074495
Cloud_Mesh_Distance_ICA_R0_R2	4,25774002	-92,02189636	-948,6286621	0,071399
Cloud_Mesh_Distance_ICA_R0_R2	5,7237854	-92,76926422	-948,4178467	-0,254297
Cloud_Mesh_Distance_ICA_R0_R2	5,47755051	-92,63986969	-948,5095215	-0,026185
Cloud_Mesh_Distance_ICA_R0_R2	5,59070587	-92,74229431	-948,4848023	-0,125906
Cloud_Mesh_Distance_ICA_R0_R2	5,60163879	-92,59729004	-948,4205322	-0,054834
Cloud_Mesh_Distance_ICA_R0_R2	3,60118866	-91,9979248	-948,5841065	0,170799
Cloud_Mesh_Distance_ICA_R0_R2	4,55309677	-92,0796814	-948,6086426	0,038787
Cloud_Mesh_Distance_ICA_R0_R2	5,28650665	-92,27386475	-948,4395142	0,013353
Cloud_Mesh_Distance_ICA_R0_R2	5,3168335	-92,43000793	-948,5054932	0,004918
Cloud_Mesh_Distance_ICA_R0_R2	5,36358261	-92,54763794	-948,5332642	-0,012268
Cloud_Mesh_Distance_ICA_R0_R2	5,45051193	-92,47225952	-948,451416	-0,006346
Cloud_Mesh_Distance_ICA_R0_R2	5,59141159	-92,40341949	-948,3210449	-0,006178
Cloud_Mesh_Distance_ICA_R0_R2	5,84162521	-92,47160339	-948,1712647	-0,034518
Cloud_Mesh_Distance_ICA_R0_R2	5,76935959	-92,58406067	-948,2998047	-0,172884
Cloud_Mesh_Distance_ICA_R0_R2	3,88261032	-91,90460205	-948,5891113	0,120855
Cloud_Mesh_Distance_ICA_R0_R2	3,99476624	-91,91187286	-948,5971069	0,102682
Cloud_Mesh_Distance_ICA_R0_R2	3,99185181	-91,78245544	-948,5528565	0,092606
Cloud_Mesh_Distance_ICA_R0_R2	4,65893936	-92,14451599	-948,6118774	0,028664
Cloud_Mesh_Distance_ICA_R0_R2	4,77275848	-92,13954163	-948,5817871	0,018134
Cloud_Mesh_Distance_ICA_R0_R2	5,13787842	-92,2677002	-948,5116577	0,015036
Cloud_Mesh_Distance_ICA_R0_R2	5,44052887	-92,34037781	-948,3856812	0,004683
Cloud_Mesh_Distance_ICA_R0_R2	5,55129242	-92,2616272	-948,2507935	0,016469
Cloud_Mesh_Distance_ICA_R0_R2	5,64951706	-92,302948	-948,2015381	0,009841

Cloud_Mesh_Distance_ICA_R0_R2	5,74674988	-92,39820862	-948,1942139	-0,012552
Cloud_Mesh_Distance_ICA_R0_R2	3,88367844	-91,64102936	-948,4951172	0,088331
Cloud_Mesh_Distance_ICA_R0_R2	3,50323868	-91,89686584	-948,5232544	0,173821
Cloud_Mesh_Distance_ICA_R0_R2	3,66296005	-91,88267517	-948,5526123	0,158409
Cloud_Mesh_Distance_ICA_R0_R2	3,8013649	-91,81625366	-948,5489502	0,13099
Cloud_Mesh_Distance_ICA_R0_R2	4,06801605	-91,64312744	-948,5064087	0,066258
Cloud_Mesh_Distance_ICA_R0_R2	4,19987869	-91,72874451	-948,5324707	0,065777
Cloud_Mesh_Distance_ICA_R0_R2	4,37789536	-91,92524719	-948,5812988	0,051426
Cloud_Mesh_Distance_ICA_R0_R2	4,54180527	-91,92129517	-948,5512085	0,048903
Cloud_Mesh_Distance_ICA_R0_R2	4,85565186	-92,07472992	-948,5271606	0,032475
Cloud_Mesh_Distance_ICA_R0_R2	4,71847534	-92,01557922	-948,5445557	0,028043
Cloud_Mesh_Distance_ICA_R0_R2	4,96355438	-92,10345459	-948,5023804	0,029099
Cloud_Mesh_Distance_ICA_R0_R2	5,03149033	-92,21051025	-948,5284424	0,016523
Cloud_Mesh_Distance_ICA_R0_R2	5,10874939	-92,11535645	-948,4425049	0,034112
Cloud_Mesh_Distance_ICA_R0_R2	5,26988983	-91,93405914	-948,2088013	0,048903
Cloud_Mesh_Distance_ICA_R0_R2	5,1312294	-91,95011902	-948,3262329	0,044541
Cloud_Mesh_Distance_ICA_R0_R2	5,4262619	-92,20735168	-948,3071899	0,024324
Cloud_Mesh_Distance_ICA_R0_R2	5,27314377	-92,08968353	-948,3302002	0,037054
Cloud_Mesh_Distance_ICA_R0_R2	5,39426422	-92,05514526	-948,208252	0,037506
Cloud_Mesh_Distance_ICA_R0_R2	3,88649368	-91,46977234	-948,430542	0,049752
Cloud_Mesh_Distance_ICA_R0_R2	3,7276535	-91,55516052	-948,4378052	0,098638
Cloud_Mesh_Distance_ICA_R0_R2	4,04663849	-91,51438141	-948,4596558	0,040289
Cloud_Mesh_Distance_ICA_R0_R2	4,34687805	-91,62860107	-948,4810181	0,038515
Cloud_Mesh_Distance_ICA_R0_R2	4,20840073	-91,56467438	-948,4744873	0,034984
Cloud_Mesh_Distance_ICA_R0_R2	4,40756989	-91,77696228	-948,5245972	0,04928
Cloud_Mesh_Distance_ICA_R0_R2	4,58779144	-91,76896667	-948,4801025	0,038365
Cloud_Mesh_Distance_ICA_R0_R2	4,95190048	-91,67086792	-948,2597656	0,043103
Cloud_Mesh_Distance_ICA_R0_R2	4,74226761	-91,68886566	-948,388855	0,040601
Cloud_Mesh_Distance_ICA_R0_R2	4,91736221	-91,91714478	-948,4238281	0,04771
Cloud_Mesh_Distance_ICA_R0_R2	4,70472717	-91,85971069	-948,4822388	0,043848
Cloud_Mesh_Distance_ICA_R0_R2	3,86672211	-91,32443237	-948,3706665	-0,001488
Cloud_Mesh_Distance_ICA_R0_R2	3,75771713	-91,4237442	-948,3909302	0,058531
Cloud_Mesh_Distance_ICA_R0_R2	4,19691467	-91,4141922	-948,4210205	-0,00848
Cloud_Mesh_Distance_ICA_R0_R2	4,02768707	-91,36650085	-948,4046631	-0,015249
Cloud_Mesh_Distance_ICA_R0_R2	4,52987289	-91,45496368	-948,3720093	-0,001286
Cloud_Mesh_Distance_ICA_R0_R2	4,37667847	-91,47593689	-948,4202881	0,007369
Cloud_Mesh_Distance_ICA_R0_R2	4,52616882	-91,60960388	-948,4346924	0,026393
Cloud_Mesh_Distance_ICA_R0_R2	4,66768646	-91,49232483	-948,3326416	0,01464
Cloud_Mesh_Distance_ICA_R0_R2	4,17446899	-91,2611084	-948,3633423	-0,070103
Cloud_Mesh_Distance_ICA_R0_R2	4,00403595	-91,2149353	-948,3422241	-0,081625
Cloud_Mesh_Distance_ICA_R0_R2	5,9295311	-92,46736145	-948,091919	-0,036179
Cloud_Mesh_Distance_ICA_R0_R2	5,93600845	-92,6163559	-948,1959839	-0,163976
Cloud_Mesh_Distance_ICA_R0_R2	6,02098846	-92,54840088	-948,0792236	-0,12652
Cloud_Mesh_Distance_ICA_R0_R2	5,73258591	-92,25410461	-948,0778198	0,012254
Cloud_Mesh_Distance_ICA_R0_R2	5,83726883	-92,36691284	-948,086853	-0,013595
Cloud_Mesh_Distance_ICA_R0_R2	3,46469879	-91,74209595	-948,447937	0,167436
Cloud_Mesh_Distance_ICA_R0_R2	3,57178879	-91,61743164	-948,4258423	0,135382
Cloud_Mesh_Distance_ICA_R0_R2	3,66028595	-91,73161316	-948,4934082	0,140324
Cloud_Mesh_Distance_ICA_R0_R2	5,49438477	-92,02597046	-948,0789185	0,03132
Cloud_Mesh_Distance_ICA_R0_R2	5,5179863	-92,14610291	-948,1807861	0,030278

Cloud_Mesh_Distance_ICA_R0_R2	5,61483002	-92,21444702	-948,154541	0,019748
Cloud_Mesh_Distance_ICA_R0_R2	5,6145134	-92,12013245	-948,0547485	0,023564
Cloud_Mesh_Distance_ICA_R0_R2	3,59014893	-91,46500397	-948,3650513	0,095631
Cloud_Mesh_Distance_ICA_R0_R2	5,05230713	-91,81600952	-948,2861328	0,05083
Cloud_Mesh_Distance_ICA_R0_R2	5,15498352	-91,84446716	-948,2305908	0,053495
Cloud_Mesh_Distance_ICA_R0_R2	5,38768387	-91,95357513	-948,1140137	0,044653
Cloud_Mesh_Distance_ICA_R0_R2	3,65351868	-91,19213867	-948,2619629	-0,011111
Cloud_Mesh_Distance_ICA_R0_R2	3,70586777	-91,31057739	-948,3304443	0,01591
Cloud_Mesh_Distance_ICA_R0_R2	4,77365112	-91,34645081	-948,1984863	-0,009506
Cloud_Mesh_Distance_ICA_R0_R2	4,81885147	-91,52731323	-948,2651978	0,026745
Cloud_Mesh_Distance_ICA_R0_R2	5,07048798	-91,60144043	-948,1071167	0,051289
Cloud_Mesh_Distance_ICA_R0_R2	5,10079575	-91,7314682	-948,1843872	0,060858
Cloud_Mesh_Distance_ICA_R0_R2	5,21871948	-91,79437256	-948,1274414	0,054088
Cloud_Mesh_Distance_ICA_R0_R2	5,16894531	-91,68637848	-948,0758057	0,056236
Cloud_Mesh_Distance_ICA_R0_R2	4,00091553	-91,07066345	-948,2789917	-0,167974
Cloud_Mesh_Distance_ICA_R0_R2	4,15687943	-91,10812378	-948,300415	-0,155647
Cloud_Mesh_Distance_ICA_R0_R2	4,34061813	-91,31469727	-948,3657227	-0,04664
Cloud_Mesh_Distance_ICA_R0_R2	4,46850967	-91,3624115	-948,3543091	-0,023023
Cloud_Mesh_Distance_ICA_R0_R2	4,48476028	-91,2315979	-948,2955933	-0,084343
Cloud_Mesh_Distance_ICA_R0_R2	4,5985527	-91,34155273	-948,2978516	-0,020036
Cloud_Mesh_Distance_ICA_R0_R2	4,00539017	-90,96313477	-948,2269898	-0,239412
Cloud_Mesh_Distance_ICA_R0_R2	4,12988281	-90,97723389	-948,2400513	-0,239823
Cloud_Mesh_Distance_ICA_R0_R2	4,33005524	-91,15596008	-948,3033447	-0,123233
Cloud_Mesh_Distance_ICA_R0_R2	4,71770477	-91,06925964	-948,0880737	-0,081948
Cloud_Mesh_Distance_ICA_R0_R2	4,64970779	-91,17515564	-948,1942749	-0,070151
Cloud_Mesh_Distance_ICA_R0_R2	4,30907822	-90,9851532	-948,2276611	-0,225192
Cloud_Mesh_Distance_ICA_R0_R2	4,45643616	-90,90455627	-948,1439209	-0,240775
Cloud_Mesh_Distance_ICA_R0_R2	4,58252716	-90,94242859	-948,1066284	-0,176595
Cloud_Mesh_Distance_ICA_R0_R2	4,63119507	-91,03962708	-948,1342773	-0,120311
Cloud_Mesh_Distance_ICA_R0_R2	4,49825287	-91,05703735	-948,210022	-0,143623
Cloud_Mesh_Distance_ICA_R0_R2	4,41772079	-90,782547	-948,0785523	-0,327419
Cloud_Mesh_Distance_ICA_R0_R2	4,54710007	-90,82920837	-948,0518189	-0,239289
Cloud_Mesh_Distance_ICA_R0_R2	5,59427261	-92,00046539	-947,9196167	0,028712
Cloud_Mesh_Distance_ICA_R0_R2	5,73797226	-92,13204956	-947,9215088	0,008418
Cloud_Mesh_Distance_ICA_R0_R2	5,83953094	-92,28173828	-947,9958496	-0,003244
Cloud_Mesh_Distance_ICA_R0_R2	5,26771927	-91,70934296	-947,9763794	0,040684
Cloud_Mesh_Distance_ICA_R0_R2	5,33300781	-91,84980774	-948,0587158	0,045608
Cloud_Mesh_Distance_ICA_R0_R2	5,46401978	-91,89611816	-947,9476929	0,037099
Cloud_Mesh_Distance_ICA_R0_R2	3,54014587	-91,31268311	-948,2805176	0,07417
Cloud_Mesh_Distance_ICA_R0_R2	5,07053757	-91,49349976	-948,0124512	0,04041
Cloud_Mesh_Distance_ICA_R0_R2	5,16475677	-91,59034729	-947,9832764	0,038478
Cloud_Mesh_Distance_ICA_R0_R2	3,37256241	-90,94779968	-948,0119629	0,007989
Cloud_Mesh_Distance_ICA_R0_R2	3,51655579	-90,98426819	-948,1056519	-0,050861
Cloud_Mesh_Distance_ICA_R0_R2	3,48832703	-91,16854095	-948,1911011	0,031679
Cloud_Mesh_Distance_ICA_R0_R2	3,83032227	-91,16158295	-948,2941895	-0,070701
Cloud_Mesh_Distance_ICA_R0_R2	4,89453888	-91,20572662	-947,9978638	-0,018491
Cloud_Mesh_Distance_ICA_R0_R2	4,90296173	-91,29414368	-948,0519409	-0,000885
Cloud_Mesh_Distance_ICA_R0_R2	4,9475975	-91,4631958	-948,1237793	0,039485
Cloud_Mesh_Distance_ICA_R0_R2	5,0288353	-91,36541748	-947,9522095	0,023062
Cloud_Mesh_Distance_ICA_R0_R2	3,51054764	-90,84501648	-948,0184937	-0,1096

Cloud_Mesh_Distance_ICA_R0_R2	3,89248657	-91,01657867	-948,237793	-0,17441
Cloud_Mesh_Distance_ICA_R0_R2	3,711689	-91,02816772	-948,2000732	-0,108118
Cloud_Mesh_Distance_ICA_R0_R2	4,6858902	-90,96633911	-948,0499268	-0,129813
Cloud_Mesh_Distance_ICA_R0_R2	4,79721069	-91,00777435	-947,9686279	-0,083969
Cloud_Mesh_Distance_ICA_R0_R2	4,80874252	-91,1701355	-948,0681763	-0,034882
Cloud_Mesh_Distance_ICA_R0_R2	4,96711731	-91,24363708	-947,9327393	0,011464
Cloud_Mesh_Distance_ICA_R0_R2	3,72950745	-90,68916321	-948,001709	-0,306494
Cloud_Mesh_Distance_ICA_R0_R2	3,6070137	-90,74906921	-947,99823	-0,208725
Cloud_Mesh_Distance_ICA_R0_R2	3,69753647	-90,83340454	-948,0878906	-0,213673
Cloud_Mesh_Distance_ICA_R0_R2	4,08106613	-90,88607788	-948,1901245	-0,306319
Cloud_Mesh_Distance_ICA_R0_R2	3,91713333	-90,87702942	-948,168396	-0,275608
Cloud_Mesh_Distance_ICA_R0_R2	4,12029648	-90,75679016	-948,1105347	-0,423823
Cloud_Mesh_Distance_ICA_R0_R2	4,28915787	-90,77838135	-948,1099243	-0,382082
Cloud_Mesh_Distance_ICA_R0_R2	4,34331894	-90,860672	-948,1522827	-0,305079
Cloud_Mesh_Distance_ICA_R0_R2	4,21663666	-90,87033081	-948,1774902	-0,324183
Cloud_Mesh_Distance_ICA_R0_R2	4,7218132	-90,8762207	-947,9483032	-0,146415
Cloud_Mesh_Distance_ICA_R0_R2	3,90390778	-90,69963074	-948,0529175	-0,406464
Cloud_Mesh_Distance_ICA_R0_R2	3,94287491	-90,58596802	-947,9691162	-0,51223
Cloud_Mesh_Distance_ICA_R0_R2	4,31590271	-90,64328766	-947,9981079	-0,470183
Cloud_Mesh_Distance_ICA_R0_R2	4,50971603	-90,70993042	-947,9762573	-0,322986
Cloud_Mesh_Distance_ICA_R0_R2	4,63911057	-90,87741089	-948,0250855	-0,181302
Cloud_Mesh_Distance_ICA_R0_R2	4,0052681	-90,50563812	-947,8963623	-0,621691
Cloud_Mesh_Distance_ICA_R0_R2	4,08443069	-90,60426331	-947,994812	-0,576947
Cloud_Mesh_Distance_ICA_R0_R2	6,02898407	-92,44483948	-947,9807129	-0,076506
Cloud_Mesh_Distance_ICA_R0_R2	6,05149078	-92,34845734	-947,8480835	-0,049419
Cloud_Mesh_Distance_ICA_R0_R2	6,16117477	-92,46396637	-947,8757935	-0,200605
Cloud_Mesh_Distance_ICA_R0_R2	5,8154068	-92,09596252	-947,7489624	0,002831
Cloud_Mesh_Distance_ICA_R0_R2	5,93021011	-92,35348511	-947,9821167	-0,015076
Cloud_Mesh_Distance_ICA_R0_R2	5,90641403	-92,22787476	-947,84729	-0,011467
Cloud_Mesh_Distance_ICA_R0_R2	6,08666992	-92,26052856	-947,6660767	-0,052694
Cloud_Mesh_Distance_ICA_R0_R2	6,20116425	-92,37688446	-947,7214966	-0,196072
Cloud_Mesh_Distance_ICA_R0_R2	5,60131073	-91,89518738	-947,7078857	0,01169
Cloud_Mesh_Distance_ICA_R0_R2	5,69647217	-92,02102661	-947,7979126	0,012875
Cloud_Mesh_Distance_ICA_R0_R2	5,24095535	-91,57827759	-947,8477783	0,042625
Cloud_Mesh_Distance_ICA_R0_R2	5,37014389	-91,77901459	-947,9176636	0,039744
Cloud_Mesh_Distance_ICA_R0_R2	5,14091492	-91,4683075	-947,8834839	0,04151
Cloud_Mesh_Distance_ICA_R0_R2	5,22527313	-91,45468903	-947,6932373	0,036213
Cloud_Mesh_Distance_ICA_R0_R2	3,39712906	-90,83984375	-947,9543457	-0,057729
Cloud_Mesh_Distance_ICA_R0_R2	4,90925217	-91,12156677	-947,9139404	-0,022739
Cloud_Mesh_Distance_ICA_R0_R2	5,03510666	-91,28407288	-947,862854	0,018247
Cloud_Mesh_Distance_ICA_R0_R2	5,02194977	-91,19378662	-947,7910767	0,009998
Cloud_Mesh_Distance_ICA_R0_R2	3,483284	-90,73092651	-947,921875	-0,136833
Cloud_Mesh_Distance_ICA_R0_R2	3,62229156	-90,65881348	-947,9352417	-0,25994
Cloud_Mesh_Distance_ICA_R0_R2	3,72262955	-90,61074066	-947,9354858	-0,356252
Cloud_Mesh_Distance_ICA_R0_R2	4,88335037	-90,99771881	-947,8414307	-0,05403
Cloud_Mesh_Distance_ICA_R0_R2	3,7769165	-90,54073334	-947,8873291	-0,43053
Cloud_Mesh_Distance_ICA_R0_R2	3,65423584	-90,56995392	-947,867981	-0,321784
Cloud_Mesh_Distance_ICA_R0_R2	3,82766724	-90,60653687	-947,9642944	-0,422268
Cloud_Mesh_Distance_ICA_R0_R2	4,48059464	-90,59436798	-947,8758545	-0,38785
Cloud_Mesh_Distance_ICA_R0_R2	4,63189316	-90,78398895	-947,9550171	-0,220845

Cloud_Mesh_Distance_ICA_R0_R2	4,72045517	-90,7651825	-947,8416748	-0,17981
Cloud_Mesh_Distance_ICA_R0_R2	3,88418579	-90,53514099	-947,9109497	-0,513093
Cloud_Mesh_Distance_ICA_R0_R2	4,19585037	-90,49395752	-947,8731079	-0,653412
Cloud_Mesh_Distance_ICA_R0_R2	4,05197144	-90,40621948	-947,7706909	-0,739984
Cloud_Mesh_Distance_ICA_R0_R2	4,35939026	-90,527565	-947,8637695	-0,504413
Cloud_Mesh_Distance_ICA_R0_R2	4,32109833	-90,44536591	-947,7679443	-0,570219
Cloud_Mesh_Distance_ICA_R0_R2	4,61109161	-90,67362976	-947,8609009	-0,27284
Cloud_Mesh_Distance_ICA_R0_R2	4,19863129	-90,40635681	-947,7518311	-0,690861
Cloud_Mesh_Distance_ICA_R0_R2	5,94916534	-92,14832306	-947,6395264	-0,02244
Cloud_Mesh_Distance_ICA_R0_R2	5,94124222	-92,09458923	-947,5090332	-0,010616
Cloud_Mesh_Distance_ICA_R0_R2	6,09014893	-92,17733002	-947,449585	-0,115792
Cloud_Mesh_Distance_ICA_R0_R2	6,2664299	-92,27946472	-947,4228516	-0,304952
Cloud_Mesh_Distance_ICA_R0_R2	6,23838806	-92,3193512	-947,5722656	-0,225384
Cloud_Mesh_Distance_ICA_R0_R2	5,75867844	-92,0032196	-947,6425781	-0,000293
Cloud_Mesh_Distance_ICA_R0_R2	5,86200333	-92,07012939	-947,6079102	-0,010398
Cloud_Mesh_Distance_ICA_R0_R2	5,33832932	-91,67609406	-947,8144531	0,038912
Cloud_Mesh_Distance_ICA_R0_R2	5,39454269	-91,65279388	-947,6425171	0,028747
Cloud_Mesh_Distance_ICA_R0_R2	5,44416046	-91,77239227	-947,774353	0,029014
Cloud_Mesh_Distance_ICA_R0_R2	5,5047493	-91,74832153	-947,5710449	0,020557
Cloud_Mesh_Distance_ICA_R0_R2	5,25871658	-91,39874268	-947,4801636	0,032179
Cloud_Mesh_Distance_ICA_R0_R2	5,30615234	-91,5775528	-947,7111816	0,038541
Cloud_Mesh_Distance_ICA_R0_R2	5,03548813	-91,12552643	-947,6760254	-0,001374
Cloud_Mesh_Distance_ICA_R0_R2	5,10786819	-91,32494354	-947,772522	0,027082
Cloud_Mesh_Distance_ICA_R0_R2	5,09248352	-91,19673157	-947,6309204	0,011894
Cloud_Mesh_Distance_ICA_R0_R2	5,17559814	-91,30706787	-947,5705566	0,024597
Cloud_Mesh_Distance_ICA_R0_R2	3,39393997	-90,64285278	-947,7850952	-0,113071
Cloud_Mesh_Distance_ICA_R0_R2	3,5407753	-90,62037659	-947,8587036	-0,21834
Cloud_Mesh_Distance_ICA_R0_R2	4,94684982	-90,91261292	-947,5939941	-0,039148
Cloud_Mesh_Distance_ICA_R0_R2	4,96922684	-91,04881287	-947,7338867	-0,031405
Cloud_Mesh_Distance_ICA_R0_R2	4,90295792	-90,9373703	-947,7387695	-0,068924
Cloud_Mesh_Distance_ICA_R0_R2	5,04224014	-91,05462646	-947,5444336	0,006978
Cloud_Mesh_Distance_ICA_R0_R2	3,40431976	-90,52690125	-947,6575317	-0,149729
Cloud_Mesh_Distance_ICA_R0_R2	3,55631256	-90,5193634	-947,758667	-0,277799
Cloud_Mesh_Distance_ICA_R0_R2	3,7083931	-90,48953247	-947,803833	-0,402813
Cloud_Mesh_Distance_ICA_R0_R2	4,87430573	-90,85320282	-947,6864014	-0,08347
Cloud_Mesh_Distance_ICA_R0_R2	4,820961	-90,8795929	-947,8209229	-0,101115
Cloud_Mesh_Distance_ICA_R0_R2	3,51597214	-90,44355774	-947,6258545	-0,255209
Cloud_Mesh_Distance_ICA_R0_R2	3,86190796	-90,45217133	-947,8097534	-0,545766
Cloud_Mesh_Distance_ICA_R0_R2	3,91039658	-90,35612488	-947,6739502	-0,635587
Cloud_Mesh_Distance_ICA_R0_R2	4,03988647	-90,33926392	-947,6546021	-0,764967
Cloud_Mesh_Distance_ICA_R0_R2	4,59799194	-90,50843811	-947,6130371	-0,315108
Cloud_Mesh_Distance_ICA_R0_R2	4,58897781	-90,56671143	-947,737793	-0,316132
Cloud_Mesh_Distance_ICA_R0_R2	4,69461823	-90,66435242	-947,7459106	-0,225224
Cloud_Mesh_Distance_ICA_R0_R2	3,97694778	-90,30316162	-947,5726318	-0,705396
Cloud_Mesh_Distance_ICA_R0_R2	4,13764191	-90,35735321	-947,6798096	-0,753588
Cloud_Mesh_Distance_ICA_R0_R2	4,26989365	-90,3625412	-947,6390381	-0,619909
Cloud_Mesh_Distance_ICA_R0_R2	4,45318604	-90,50093842	-947,7711182	-0,442362
Cloud_Mesh_Distance_ICA_R0_R2	4,41905975	-90,42602539	-947,6658936	-0,482084
Cloud_Mesh_Distance_ICA_R0_R2	4,51922226	-90,4838562	-947,6765747	-0,385286
Cloud_Mesh_Distance_ICA_R0_R2	4,51794815	-90,4379425	-947,5695801	-0,384184

Cloud_Mesh_Distance_ICA_R0_R2	5,61449051	-91,82168579	-947,4564209	0,00642
Cloud_Mesh_Distance_ICA_R0_R2	5,70610046	-91,86484528	-947,2612305	-0,003371
Cloud_Mesh_Distance_ICA_R0_R2	5,85208511	-92,02301025	-947,4835205	-0,004762
Cloud_Mesh_Distance_ICA_R0_R2	5,73210526	-91,92921448	-947,4775391	-0,002412
Cloud_Mesh_Distance_ICA_R0_R2	5,53709412	-91,7310791	-947,4008789	0,008403
Cloud_Mesh_Distance_ICA_R0_R2	5,21013641	-91,29458618	-947,4143677	0,033754
Cloud_Mesh_Distance_ICA_R0_R2	5,3289032	-91,52818298	-947,5460815	0,029696
Cloud_Mesh_Distance_ICA_R0_R2	5,20939255	-91,24757385	-947,2712402	0,030571
Cloud_Mesh_Distance_ICA_R0_R2	5,13944626	-91,17463684	-947,4216309	0,031078
Cloud_Mesh_Distance_ICA_R0_R2	5,07024002	-91,01443481	-947,3206787	0,009738
Cloud_Mesh_Distance_ICA_R0_R2	3,4000473	-90,44904327	-947,5308228	-0,152389
Cloud_Mesh_Distance_ICA_R0_R2	3,3078804	-90,5266571	-947,5648804	-0,067657
Cloud_Mesh_Distance_ICA_R0_R2	3,27587509	-90,48343658	-947,4502564	-0,042958
Cloud_Mesh_Distance_ICA_R0_R2	4,79945374	-90,74797058	-947,6869507	-0,13969
Cloud_Mesh_Distance_ICA_R0_R2	4,83406448	-90,71302795	-947,5298462	-0,11697
Cloud_Mesh_Distance_ICA_R0_R2	4,87908936	-90,80056763	-947,5859375	-0,079807
Cloud_Mesh_Distance_ICA_R0_R2	4,91749191	-90,79301453	-947,4293823	-0,057474
Cloud_Mesh_Distance_ICA_R0_R2	4,99859238	-90,92234802	-947,4241943	-0,009861
Cloud_Mesh_Distance_ICA_R0_R2	3,54078293	-90,36160278	-947,4899292	-0,293718
Cloud_Mesh_Distance_ICA_R0_R2	3,6896019	-90,31231689	-947,4902344	-0,421239
Cloud_Mesh_Distance_ICA_R0_R2	3,62179947	-90,29301453	-947,3824463	-0,338457
Cloud_Mesh_Distance_ICA_R0_R2	3,69818878	-90,39234924	-947,6577148	-0,429927
Cloud_Mesh_Distance_ICA_R0_R2	3,83559799	-90,30085754	-947,53479	-0,56319
Cloud_Mesh_Distance_ICA_R0_R2	4,82370758	-90,65048218	-947,3704224	-0,11803
Cloud_Mesh_Distance_ICA_R0_R2	4,71934509	-90,59962463	-947,5737305	-0,20853
Cloud_Mesh_Distance_ICA_R0_R2	4,11825943	-90,30214691	-947,5552368	-0,732742
Cloud_Mesh_Distance_ICA_R0_R2	4,14822388	-90,26290131	-947,4066162	-0,626856
Cloud_Mesh_Distance_ICA_R0_R2	4,26569366	-90,30392456	-947,4752197	-0,573988
Cloud_Mesh_Distance_ICA_R0_R2	4,40149307	-90,36573792	-947,5291748	-0,477133
Cloud_Mesh_Distance_ICA_R0_R2	4,62400818	-90,48308563	-947,482605	-0,277874
Cloud_Mesh_Distance_ICA_R0_R2	4,51947784	-90,39736938	-947,4212647	-0,35362
Cloud_Mesh_Distance_ICA_R0_R2	5,96397781	-92,07439423	-947,362915	-0,024523
Cloud_Mesh_Distance_ICA_R0_R2	6,037117	-92,09225464	-947,1071167	-0,221972
Cloud_Mesh_Distance_ICA_R0_R2	6,07883453	-92,12713623	-947,2310181	-0,196957
Cloud_Mesh_Distance_ICA_R0_R2	5,59989548	-91,77442932	-947,3034058	-0,00156
Cloud_Mesh_Distance_ICA_R0_R2	5,85345459	-91,98438263	-947,2869263	-0,016646
Cloud_Mesh_Distance_ICA_R0_R2	5,97358322	-92,05885315	-947,2147217	-0,100878
Cloud_Mesh_Distance_ICA_R0_R2	5,92937088	-92,02037048	-947,0789795	-0,135802
Cloud_Mesh_Distance_ICA_R0_R2	5,43964767	-91,62553406	-947,4163208	0,02265
Cloud_Mesh_Distance_ICA_R0_R2	5,52674484	-91,68917847	-947,2515259	0,008285
Cloud_Mesh_Distance_ICA_R0_R2	5,33904266	-91,46453857	-947,3148804	0,025594
Cloud_Mesh_Distance_ICA_R0_R2	5,43968201	-91,57359314	-947,1817627	0,022002
Cloud_Mesh_Distance_ICA_R0_R2	5,14598846	-91,11959839	-947,2016602	0,025872
Cloud_Mesh_Distance_ICA_R0_R2	5,26006699	-91,34811401	-947,3308716	0,032262
Cloud_Mesh_Distance_ICA_R0_R2	5,21300125	-91,21551514	-947,0905151	0,031978
Cloud_Mesh_Distance_ICA_R0_R2	5,07539368	-90,97885132	-947,1027832	0,018978
Cloud_Mesh_Distance_ICA_R0_R2	4,98840332	-90,85395813	-947,2348023	-0,017692
Cloud_Mesh_Distance_ICA_R0_R2	4,91986084	-90,7358551	-947,121521	-0,026949
Cloud_Mesh_Distance_ICA_R0_R2	3,38021088	-90,38352203	-947,3622437	-0,138486
Cloud_Mesh_Distance_ICA_R0_R2	4,71972275	-90,54271698	-947,3894043	-0,187458

Cloud_Mesh_Distance_ICA_R0_R2	4,8560257	-90,66101074	-947,2033081	-0,073112
Cloud_Mesh_Distance_ICA_R0_R2	4,90532684	-90,73483276	-947,269165	-0,056611
Cloud_Mesh_Distance_ICA_R0_R2	3,61461639	-90,25462341	-947,2207031	-0,295104
Cloud_Mesh_Distance_ICA_R0_R2	3,514534	-90,31320953	-947,3327026	-0,238256
Cloud_Mesh_Distance_ICA_R0_R2	3,98358154	-90,25297546	-947,4177856	-0,678632
Cloud_Mesh_Distance_ICA_R0_R2	3,77239609	-90,25222778	-947,3535767	-0,472023
Cloud_Mesh_Distance_ICA_R0_R2	3,92565536	-90,21495056	-947,2130127	-0,508223
Cloud_Mesh_Distance_ICA_R0_R2	4,24542999	-90,25548553	-947,2022095	-0,450075
Cloud_Mesh_Distance_ICA_R0_R2	4,12122726	-90,23249817	-947,241333	-0,532919
Cloud_Mesh_Distance_ICA_R0_R2	4,25562286	-90,2696991	-947,3166504	-0,507141
Cloud_Mesh_Distance_ICA_R0_R2	4,3903923	-90,32292938	-947,3657227	-0,433129
Cloud_Mesh_Distance_ICA_R0_R2	5,70485306	-91,8431778	-946,8310547	-0,070645
Cloud_Mesh_Distance_ICA_R0_R2	5,80793381	-91,93389893	-947,1173096	-0,012257
Cloud_Mesh_Distance_ICA_R0_R2	5,71405029	-91,8520813	-947,0369263	-0,004119
Cloud_Mesh_Distance_ICA_R0_R2	5,52769089	-91,66505432	-947,0599365	0,012074
Cloud_Mesh_Distance_ICA_R0_R2	5,52627945	-91,65016174	-946,8615112	0,024536
Cloud_Mesh_Distance_ICA_R0_R2	5,61484528	-91,76708221	-947,1436768	0,002304
Cloud_Mesh_Distance_ICA_R0_R2	5,61553955	-91,75434875	-946,9481201	0,003579
Cloud_Mesh_Distance_ICA_R0_R2	5,27762604	-91,3379364	-947,1730957	0,031913
Cloud_Mesh_Distance_ICA_R0_R2	5,36652374	-91,43383789	-946,8703613	0,034511
Cloud_Mesh_Distance_ICA_R0_R2	5,35826874	-91,44538879	-947,0776367	0,031055
Cloud_Mesh_Distance_ICA_R0_R2	5,14986801	-91,09561157	-947,0089111	0,03491
Cloud_Mesh_Distance_ICA_R0_R2	4,98926544	-90,82918549	-946,9901123	0,007599
Cloud_Mesh_Distance_ICA_R0_R2	3,53982162	-90,2513504	-947,0752564	-0,183486
Cloud_Mesh_Distance_ICA_R0_R2	3,30329895	-90,37467957	-947,2039185	-0,043549
Cloud_Mesh_Distance_ICA_R0_R2	4,63753128	-90,45855713	-947,3101807	-0,239852
Cloud_Mesh_Distance_ICA_R0_R2	4,64717484	-90,45181274	-947,1158447	-0,173
Cloud_Mesh_Distance_ICA_R0_R2	4,76363754	-90,55885315	-947,203186	-0,128444
Cloud_Mesh_Distance_ICA_R0_R2	3,43860245	-90,30453491	-947,1813355	-0,145706
Cloud_Mesh_Distance_ICA_R0_R2	3,76948166	-90,21804047	-947,1693115	-0,388453
Cloud_Mesh_Distance_ICA_R0_R2	3,87265015	-90,20009613	-947,0247192	-0,34997
Cloud_Mesh_Distance_ICA_R0_R2	4,51779175	-90,37117004	-947,242981	-0,297001
Cloud_Mesh_Distance_ICA_R0_R2	4,36853027	-90,29489136	-947,1791382	-0,367804
Cloud_Mesh_Distance_ICA_R0_R2	5,83195114	-91,9499054	-946,9156494	-0,138732
Cloud_Mesh_Distance_ICA_R0_R2	5,44360352	-91,55215454	-946,9699097	0,022458
Cloud_Mesh_Distance_ICA_R0_R2	5,60719681	-91,7430954	-946,7542114	0,024666
Cloud_Mesh_Distance_ICA_R0_R2	5,44447327	-91,53886414	-946,7692871	0,035127
Cloud_Mesh_Distance_ICA_R0_R2	5,36855316	-91,42082977	-946,6746826	0,044117
Cloud_Mesh_Distance_ICA_R0_R2	5,20732117	-91,18545532	-946,9366455	0,036515
Cloud_Mesh_Distance_ICA_R0_R2	5,16231155	-91,10269165	-946,8563843	0,037725
Cloud_Mesh_Distance_ICA_R0_R2	5,22675323	-91,2028656	-946,7724609	0,043878
Cloud_Mesh_Distance_ICA_R0_R2	5,27996063	-91,30793762	-946,9562378	0,037332
Cloud_Mesh_Distance_ICA_R0_R2	5,00156021	-90,84483337	-946,7984009	0,023687
Cloud_Mesh_Distance_ICA_R0_R2	5,08383942	-90,97402954	-946,8764038	0,035095
Cloud_Mesh_Distance_ICA_R0_R2	3,31079102	-90,33441925	-947,0209961	-0,011764
Cloud_Mesh_Distance_ICA_R0_R2	4,90806198	-90,71439362	-946,9862061	-0,017911
Cloud_Mesh_Distance_ICA_R0_R2	3,28219223	-90,32995605	-946,8045044	0,047831
Cloud_Mesh_Distance_ICA_R0_R2	3,41287231	-90,27574158	-946,8695679	-0,037978
Cloud_Mesh_Distance_ICA_R0_R2	3,44463348	-90,27557373	-947,0184937	-0,100512
Cloud_Mesh_Distance_ICA_R0_R2	4,84872437	-90,64276123	-947,0675049	-0,059297

Cloud_Mesh_Distance_ICA_R0_R2	4,85519409	-90,65009308	-946,937561	-0,033361
Cloud_Mesh_Distance_ICA_R0_R2	4,76640701	-90,55567932	-946,9793091	-0,077362
Cloud_Mesh_Distance_ICA_R0_R2	3,54088211	-90,23779297	-946,9237671	-0,131451
Cloud_Mesh_Distance_ICA_R0_R2	3,82435226	-90,19948578	-946,8379517	-0,215643
Cloud_Mesh_Distance_ICA_R0_R2	3,68445587	-90,21414185	-947,0105591	-0,248747
Cloud_Mesh_Distance_ICA_R0_R2	4,05506516	-90,21155548	-947,045166	-0,38695
Cloud_Mesh_Distance_ICA_R0_R2	3,99849701	-90,20744324	-946,8550415	-0,277348
Cloud_Mesh_Distance_ICA_R0_R2	4,1775589	-90,23910522	-946,8605957	-0,268782
Cloud_Mesh_Distance_ICA_R0_R2	4,24514389	-90,25022888	-947,0567627	-0,354827
Cloud_Mesh_Distance_ICA_R0_R2	4,51900864	-90,36634827	-947,0482788	-0,226926
Cloud_Mesh_Distance_ICA_R0_R2	4,40050888	-90,30734253	-946,9958496	-0,265365
Cloud_Mesh_Distance_ICA_R0_R2	4,64436722	-90,45396423	-946,9193726	-0,127623
Cloud_Mesh_Distance_ICA_R0_R2	5,67787933	-91,82394409	-946,6567993	-0,166975
Cloud_Mesh_Distance_ICA_R0_R2	5,44393921	-91,52676392	-946,5727539	0,074776
Cloud_Mesh_Distance_ICA_R0_R2	5,52223969	-91,63956451	-946,6642456	0,03665
Cloud_Mesh_Distance_ICA_R0_R2	5,60014725	-91,73928833	-946,5571899	0,153622
Cloud_Mesh_Distance_ICA_R0_R2	5,28458786	-91,2792511	-946,586853	0,050906
Cloud_Mesh_Distance_ICA_R0_R2	5,37081909	-91,40384674	-946,4709473	0,059709
Cloud_Mesh_Distance_ICA_R0_R2	5,30524063	-91,33047485	-946,772522	0,043945
Cloud_Mesh_Distance_ICA_R0_R2	5,20530701	-91,15345764	-946,605896	0,049661
Cloud_Mesh_Distance_ICA_R0_R2	5,15545273	-91,06686401	-946,526062	0,052037
Cloud_Mesh_Distance_ICA_R0_R2	5,21953964	-91,15675354	-946,4492188	0,056133
Cloud_Mesh_Distance_ICA_R0_R2	5,05202103	-90,91888428	-946,6976929	0,034801
Cloud_Mesh_Distance_ICA_R0_R2	5,13802719	-91,05233765	-946,6931152	0,042372
Cloud_Mesh_Distance_ICA_R0_R2	4,83574677	-90,64038849	-946,6994019	-0,004199
Cloud_Mesh_Distance_ICA_R0_R2	3,39849472	-90,28004456	-946,6831055	0,038904
Cloud_Mesh_Distance_ICA_R0_R2	3,52610397	-90,23912811	-946,7453613	-0,048473
Cloud_Mesh_Distance_ICA_R0_R2	3,51671982	-90,25047302	-946,5674439	0,017516
Cloud_Mesh_Distance_ICA_R0_R2	4,72260666	-90,5405426	-946,6226807	-0,025763
Cloud_Mesh_Distance_ICA_R0_R2	4,74391174	-90,54403687	-946,798523	-0,048329
Cloud_Mesh_Distance_ICA_R0_R2	4,91363525	-90,72410583	-946,8331909	-0,000348
Cloud_Mesh_Distance_ICA_R0_R2	4,82600784	-90,62142944	-946,8447876	-0,025276
Cloud_Mesh_Distance_ICA_R0_R2	3,79419708	-90,20898438	-946,652771	-0,112037
Cloud_Mesh_Distance_ICA_R0_R2	3,66518021	-90,2118454	-946,7994995	-0,13504
Cloud_Mesh_Distance_ICA_R0_R2	4,12100983	-90,24061584	-946,6774902	-0,172186
Cloud_Mesh_Distance_ICA_R0_R2	4,34802246	-90,29387665	-946,8526611	-0,220941
Cloud_Mesh_Distance_ICA_R0_R2	4,29821777	-90,29211426	-946,6740723	-0,149273
Cloud_Mesh_Distance_ICA_R0_R2	4,44210434	-90,35025024	-946,6953735	-0,128584
Cloud_Mesh_Distance_ICA_R0_R2	4,64577866	-90,46725464	-946,7556763	-0,069398
Cloud_Mesh_Distance_ICA_R0_R2	4,51538086	-90,3755722	-946,8387451	-0,14669
Cloud_Mesh_Distance_ICA_R0_R2	5,51709747	-91,628685	-946,4680786	0,179033
Cloud_Mesh_Distance_ICA_R0_R2	5,44722748	-91,51617432	-946,381897	0,130341
Cloud_Mesh_Distance_ICA_R0_R2	5,50822449	-91,6071167	-946,3013916	0,216578
Cloud_Mesh_Distance_ICA_R0_R2	5,30026245	-91,27003479	-946,3568726	0,064783
Cloud_Mesh_Distance_ICA_R0_R2	5,07117462	-90,94088745	-946,5234375	0,044521
Cloud_Mesh_Distance_ICA_R0_R2	4,99454117	-90,83314514	-946,4318237	0,039553
Cloud_Mesh_Distance_ICA_R0_R2	5,05427933	-90,90361023	-946,3594971	0,052863
Cloud_Mesh_Distance_ICA_R0_R2	3,2795639	-90,35157776	-946,3982544	0,131197
Cloud_Mesh_Distance_ICA_R0_R2	4,82863617	-90,64518738	-946,5213623	0,012803
Cloud_Mesh_Distance_ICA_R0_R2	4,95368576	-90,78303528	-946,6090088	0,033519

Cloud_Mesh_Distance_ICA_R0_R2	3,27436447	-90,33540344	-946,6055298	0,099535
Cloud_Mesh_Distance_ICA_R0_R2	3,40135193	-90,29199219	-946,4912109	0,079416
Cloud_Mesh_Distance_ICA_R0_R2	3,77040482	-90,22594452	-946,4759522	-0,028827
Cloud_Mesh_Distance_ICA_R0_R2	3,64691544	-90,22242737	-946,616394	-0,047315
Cloud_Mesh_Distance_ICA_R0_R2	3,63018799	-90,2409668	-946,4450073	0,020179
Cloud_Mesh_Distance_ICA_R0_R2	3,95223236	-90,21360016	-946,6762085	-0,159082
Cloud_Mesh_Distance_ICA_R0_R2	3,92538071	-90,22808838	-946,5026856	-0,078728
Cloud_Mesh_Distance_ICA_R0_R2	4,66590881	-90,50415039	-946,5341797	-0,020502
Cloud_Mesh_Distance_ICA_R0_R2	4,58419037	-90,43885803	-946,6243286	-0,061155
Cloud_Mesh_Distance_ICA_R0_R2	5,46352768	-91,52316284	-946,2230835	0,155195
Cloud_Mesh_Distance_ICA_R0_R2	5,38891983	-91,39544678	-946,244751	0,071628
Cloud_Mesh_Distance_ICA_R0_R2	5,44862747	-91,46380615	-946,0821533	0,200322
Cloud_Mesh_Distance_ICA_R0_R2	5,23090363	-91,13650513	-946,2597656	0,063146
Cloud_Mesh_Distance_ICA_R0_R2	5,14004898	-91,02127075	-946,355957	0,058956
Cloud_Mesh_Distance_ICA_R0_R2	4,85429764	-90,66983032	-946,2011719	0,031663
Cloud_Mesh_Distance_ICA_R0_R2	4,9049263	-90,72833252	-946,3984985	0,03393
Cloud_Mesh_Distance_ICA_R0_R2	4,98196793	-90,80640411	-946,2805176	0,04915
Cloud_Mesh_Distance_ICA_R0_R2	4,36087036	-90,37445068	-946,1118774	0,003635
Cloud_Mesh_Distance_ICA_R0_R2	4,40101624	-90,37561035	-946,3033447	-0,02668
Cloud_Mesh_Distance_ICA_R0_R2	4,70809937	-90,55213928	-946,3027954	0,015781
Cloud_Mesh_Distance_ICA_R0_R2	4,72777939	-90,55834961	-946,4616699	0,004148
Cloud_Mesh_Distance_ICA_R0_R2	4,61942291	-90,4796524	-946,4517212	-0,017409
Cloud_Mesh_Distance_ICA_R0_R2	4,80665588	-90,6307373	-946,3707275	0,019753
Cloud_Mesh_Distance_ICA_R0_R2	3,51651382	-90,26651001	-946,4129028	0,064967
Cloud_Mesh_Distance_ICA_R0_R2	3,60523224	-90,26722717	-946,2813721	0,072566
Cloud_Mesh_Distance_ICA_R0_R2	3,74430084	-90,24798584	-946,3114014	0,025961
Cloud_Mesh_Distance_ICA_R0_R2	3,90078354	-90,24757385	-946,3280029	-0,012094
Cloud_Mesh_Distance_ICA_R0_R2	4,24026489	-90,29582214	-946,4721069	-0,085567
Cloud_Mesh_Distance_ICA_R0_R2	4,08530426	-90,27013397	-946,3501587	-0,039955
Cloud_Mesh_Distance_ICA_R0_R2	4,06350327	-90,24346924	-946,534729	-0,110845
Cloud_Mesh_Distance_ICA_R0_R2	4,56075287	-90,45503235	-946,3151245	-0,010964
Cloud_Mesh_Distance_ICA_R0_R2	4,45256805	-90,37808228	-946,5007935	-0,067463
Cloud_Mesh_Distance_ICA_R0_R2	5,24250031	-91,10688782	-946,1005249	0,066253
Cloud_Mesh_Distance_ICA_R0_R2	5,16923904	-91,01875305	-946,1600342	0,062947
Cloud_Mesh_Distance_ICA_R0_R2	5,20837021	-91,02380371	-946,0154419	0,059316
Cloud_Mesh_Distance_ICA_R0_R2	5,31615067	-91,23457336	-946,1240234	0,072228
Cloud_Mesh_Distance_ICA_R0_R2	5,4331131	-91,38746643	-945,9552612	0,218608
Cloud_Mesh_Distance_ICA_R0_R2	5,39159393	-91,34765625	-946,0623169	0,12843
Cloud_Mesh_Distance_ICA_R0_R2	5,4550705	-91,37120056	-945,8302612	0,264135
Cloud_Mesh_Distance_ICA_R0_R2	3,17821884	-90,46336365	-946,0778198	0,165018
Cloud_Mesh_Distance_ICA_R0_R2	3,29483795	-90,37864685	-946,1882324	0,151679
Cloud_Mesh_Distance_ICA_R0_R2	4,73068619	-90,56895447	-946,1583862	0,024639
Cloud_Mesh_Distance_ICA_R0_R2	4,7829628	-90,597229	-946,0407715	0,037027
Cloud_Mesh_Distance_ICA_R0_R2	5,07738495	-90,90923309	-946,2109985	0,053098
Cloud_Mesh_Distance_ICA_R0_R2	5,02382278	-90,7710495	-945,9192505	0,049475
Cloud_Mesh_Distance_ICA_R0_R2	4,99514389	-90,79161072	-946,1008911	0,044897
Cloud_Mesh_Distance_ICA_R0_R2	3,44206238	-90,30791473	-946,2731934	0,107874
Cloud_Mesh_Distance_ICA_R0_R2	3,71765518	-90,2718811	-946,1679688	0,058068
Cloud_Mesh_Distance_ICA_R0_R2	4,03454208	-90,28456116	-946,1645508	0,011781
Cloud_Mesh_Distance_ICA_R0_R2	3,86015701	-90,26922607	-946,1615601	0,042943

Cloud_Mesh_Distance_ICA_R0_R2	4,20069504	-90,33487701	-945,989502	0,025814
Cloud_Mesh_Distance_ICA_R0_R2	4,39387894	-90,39239502	-945,923523	0,022789
Cloud_Mesh_Distance_ICA_R0_R2	4,63938904	-90,50816345	-946,1971436	0,00827
Cloud_Mesh_Distance_ICA_R0_R2	4,53082275	-90,447258	-946,1590576	0,009642
Cloud_Mesh_Distance_ICA_R0_R2	4,90616989	-90,68606567	-945,9953003	0,045233
Cloud_Mesh_Distance_ICA_R0_R2	4,82731628	-90,60413361	-945,8876343	0,041231
Cloud_Mesh_Distance_ICA_R0_R2	4,25436401	-90,32264709	-946,281189	-0,027418
Cloud_Mesh_Distance_ICA_R0_R2	4,18621445	-90,31384277	-946,1782227	-0,008301
Cloud_Mesh_Distance_ICA_R0_R2	5,38656235	-91,26847839	-945,9094849	0,139973
Cloud_Mesh_Distance_ICA_R0_R2	5,13256073	-90,83000183	-945,7907105	0,047345
Cloud_Mesh_Distance_ICA_R0_R2	5,22183228	-90,970047	-945,861145	0,059894
Cloud_Mesh_Distance_ICA_R0_R2	5,29590607	-91,11625671	-945,9285889	0,067124
Cloud_Mesh_Distance_ICA_R0_R2	3,27534866	-90,46989441	-945,8723755	0,154431
Cloud_Mesh_Distance_ICA_R0_R2	4,65982819	-90,51896667	-946,0750732	0,02123
Cloud_Mesh_Distance_ICA_R0_R2	4,94779968	-90,67948914	-945,8374634	0,055671
Cloud_Mesh_Distance_ICA_R0_R2	5,03755569	-90,73577881	-945,7848511	0,052038
Cloud_Mesh_Distance_ICA_R0_R2	5,11789703	-90,90364075	-946,0092163	0,057486
Cloud_Mesh_Distance_ICA_R0_R2	3,31080246	-90,4081955	-946,0252686	0,153001
Cloud_Mesh_Distance_ICA_R0_R2	3,39117432	-90,3992157	-945,9266357	0,140192
Cloud_Mesh_Distance_ICA_R0_R2	3,42364883	-90,35414124	-946,0627441	0,132767
Cloud_Mesh_Distance_ICA_R0_R2	3,59807968	-90,30575562	-946,0739746	0,096989
Cloud_Mesh_Distance_ICA_R0_R2	3,79409409	-90,29439545	-946,005188	0,067023
Cloud_Mesh_Distance_ICA_R0_R2	4,12155151	-90,33584595	-945,7911377	0,057202
Cloud_Mesh_Distance_ICA_R0_R2	3,98557281	-90,30103302	-945,9868164	0,048305
Cloud_Mesh_Distance_ICA_R0_R2	4,55693054	-90,4596405	-945,9663086	0,021434
Cloud_Mesh_Distance_ICA_R0_R2	4,71010971	-90,53886414	-945,9485474	0,039113
Cloud_Mesh_Distance_ICA_R0_R2	5,38210678	-91,17218781	-945,7709961	0,084978
Cloud_Mesh_Distance_ICA_R0_R2	5,43800354	-91,16157532	-945,6171265	0,139658
Cloud_Mesh_Distance_ICA_R0_R2	5,44994736	-91,28083801	-945,7198486	0,206001
Cloud_Mesh_Distance_ICA_R0_R2	3,15341568	-90,59011841	-945,7510986	0,155801
Cloud_Mesh_Distance_ICA_R0_R2	5,33401108	-91,03886414	-945,7022095	0,048801
Cloud_Mesh_Distance_ICA_R0_R2	5,24397278	-90,90587616	-945,696167	0,04638
Cloud_Mesh_Distance_ICA_R0_R2	5,33227158	-90,89569092	-945,5185547	0,027458
Cloud_Mesh_Distance_ICA_R0_R2	5,4992981	-91,1523056	-945,4747925	-0,217906
Cloud_Mesh_Distance_ICA_R0_R2	4,99509048	-90,67484283	-945,7173462	0,050935
Cloud_Mesh_Distance_ICA_R0_R2	5,20072174	-90,79659271	-945,5948486	0,03813
Cloud_Mesh_Distance_ICA_R0_R2	3,43321228	-90,42459106	-945,77771	0,138316
Cloud_Mesh_Distance_ICA_R0_R2	3,51929092	-90,36199951	-945,8985596	0,121703
Cloud_Mesh_Distance_ICA_R0_R2	3,57791901	-90,38928223	-945,7250977	0,119336
Cloud_Mesh_Distance_ICA_R0_R2	3,70407867	-90,33686829	-945,8286743	0,099783
Cloud_Mesh_Distance_ICA_R0_R2	3,91357422	-90,32301331	-945,8068848	0,076471
Cloud_Mesh_Distance_ICA_R0_R2	4,26356506	-90,36253357	-945,5939941	0,054288
Cloud_Mesh_Distance_ICA_R0_R2	4,52287674	-90,43211365	-945,7734985	0,040094
Cloud_Mesh_Distance_ICA_R0_R2	4,32812119	-90,37392426	-945,7774048	0,038606
Cloud_Mesh_Distance_ICA_R0_R2	4,71818161	-90,5191803	-945,7844849	0,041257
Cloud_Mesh_Distance_ICA_R0_R2	4,47128296	-90,40057373	-945,5922241	0,042751
Cloud_Mesh_Distance_ICA_R0_R2	4,8915863	-90,60686493	-945,730957	0,04824
Cloud_Mesh_Distance_ICA_R0_R2	3,3000145	-90,65460205	-945,4189453	0,127175
Cloud_Mesh_Distance_ICA_R0_R2	3,22565842	-90,63430023	-945,5545044	0,138984
Cloud_Mesh_Distance_ICA_R0_R2	3,39566422	-90,5763855	-945,4337769	0,127879

Cloud_Mesh_Distance_ICA_R0_R2	3,31679916	-90,59313965	-945,5072022	0,133033
Cloud_Mesh_Distance_ICA_R0_R2	5,42857742	-91,03994751	-945,5101318	0,085822
Cloud_Mesh_Distance_ICA_R0_R2	5,52823257	-91,05674744	-945,3465576	-0,21837
Cloud_Mesh_Distance_ICA_R0_R2	5,44011688	-90,94462585	-945,3942871	0,084986
Cloud_Mesh_Distance_ICA_R0_R2	3,34946442	-90,52093506	-945,6223755	0,137984
Cloud_Mesh_Distance_ICA_R0_R2	3,49923706	-90,44451141	-945,6308594	0,127749
Cloud_Mesh_Distance_ICA_R0_R2	3,6222496	-90,41955566	-945,565918	0,114005
Cloud_Mesh_Distance_ICA_R0_R2	3,80884552	-90,41641998	-945,3643189	0,100715
Cloud_Mesh_Distance_ICA_R0_R2	3,76307678	-90,39376831	-945,5126953	0,108839
Cloud_Mesh_Distance_ICA_R0_R2	5,0866394	-90,72554779	-945,6636353	0,043075
Cloud_Mesh_Distance_ICA_R0_R2	3,70009232	-90,37530518	-945,6550903	0,107261
Cloud_Mesh_Distance_ICA_R0_R2	4,07037735	-90,36332703	-945,4299316	0,073762
Cloud_Mesh_Distance_ICA_R0_R2	4,04713821	-90,34912872	-945,5894165	0,083059
Cloud_Mesh_Distance_ICA_R0_R2	3,84168243	-90,35484314	-945,638916	0,100007
Cloud_Mesh_Distance_ICA_R0_R2	4,66632843	-90,46138	-945,5963745	0,044441
Cloud_Mesh_Distance_ICA_R0_R2	4,84607697	-90,53787231	-945,5875244	0,041851
Cloud_Mesh_Distance_ICA_R0_R2	4,97602844	-90,5448761	-945,4115601	0,075346
Cloud_Mesh_Distance_ICA_R0_R2	4,8091011	-90,47454834	-945,4202881	0,082764
Cloud_Mesh_Distance_ICA_R0_R2	5,00960541	-90,62878418	-945,5748901	0,039465
Cloud_Mesh_Distance_ICA_R0_R2	5,13999939	-90,69895935	-945,5209961	0,034106
Cloud_Mesh_Distance_ICA_R0_R2	5,12615204	-90,62445068	-945,3930664	0,080099
Cloud_Mesh_Distance_ICA_R0_R2	3,14666367	-90,89381409	-945,3136597	0,090615
Cloud_Mesh_Distance_ICA_R0_R2	3,18264389	-90,75515747	-945,4238281	0,116455
Cloud_Mesh_Distance_ICA_R0_R2	3,28433228	-90,72627258	-945,3286133	0,117675
Cloud_Mesh_Distance_ICA_R0_R2	3,49305725	-90,56292725	-945,3327026	0,122812
Cloud_Mesh_Distance_ICA_R0_R2	3,53708267	-90,58920288	-945,2157593	0,119423
Cloud_Mesh_Distance_ICA_R0_R2	3,52073288	-90,49267578	-945,4683838	0,118583
Cloud_Mesh_Distance_ICA_R0_R2	3,6790657	-90,44606018	-945,4088135	0,111183
Cloud_Mesh_Distance_ICA_R0_R2	3,74774933	-90,46832275	-945,2456055	0,104317
Cloud_Mesh_Distance_ICA_R0_R2	5,40158081	-90,83396149	-945,3306885	0,14702
Cloud_Mesh_Distance_ICA_R0_R2	5,51209259	-90,91676331	-945,2553101	0,23874
Cloud_Mesh_Distance_ICA_R0_R2	5,59511185	-91,00619507	-945,2040405	-0,336263
Cloud_Mesh_Distance_ICA_R0_R2	4,20172119	-90,36126709	-945,4204102	0,076089
Cloud_Mesh_Distance_ICA_R0_R2	3,93507767	-90,37770081	-945,4337158	0,090985
Cloud_Mesh_Distance_ICA_R0_R2	4,62708664	-90,41703796	-945,4160767	0,115474
Cloud_Mesh_Distance_ICA_R0_R2	5,27452087	-90,73377991	-945,3816528	0,085297
Cloud_Mesh_Distance_ICA_R0_R2	3,14393997	-91,05389404	-945,1965942	0,152304
Cloud_Mesh_Distance_ICA_R0_R2	3,23430634	-90,80256653	-945,2980347	0,108208
Cloud_Mesh_Distance_ICA_R0_R2	3,37958145	-90,78260803	-945,1300049	0,114436
Cloud_Mesh_Distance_ICA_R0_R2	3,25751495	-90,88249969	-945,1864014	0,094792
Cloud_Mesh_Distance_ICA_R0_R2	3,30918121	-90,7684021	-945,2410889	0,114066
Cloud_Mesh_Distance_ICA_R0_R2	3,40189362	-90,66275024	-945,2683716	0,122286
Cloud_Mesh_Distance_ICA_R0_R2	3,60706711	-90,51451111	-945,2977905	0,116926
Cloud_Mesh_Distance_ICA_R0_R2	3,65335846	-90,55198669	-945,1364136	0,098607
Cloud_Mesh_Distance_ICA_R0_R2	3,34346771	-90,89359283	-945,0670776	0,096518
Cloud_Mesh_Distance_ICA_R0_R2	3,45631027	-90,84177399	-944,9609985	0,099093
Cloud_Mesh_Distance_ICA_R0_R2	3,53084946	-90,67359161	-945,071228	0,103455
Cloud_Mesh_Distance_ICA_R0_R3	3,80027771	-92,85809326	-948,7197876	0,0337
Cloud_Mesh_Distance_ICA_R0_R3	4,28671265	-92,94000244	-948,7653198	-0,050562
Cloud_Mesh_Distance_ICA_R0_R3	4,28998566	-93,13349915	-948,7531738	-0,096875

Cloud_Mesh_Distance_ICA_R0_R3	4,24546051	-93,07064819	-948,7579346	-0,060991
Cloud_Mesh_Distance_ICA_R0_R3	4,55498505	-93,02685547	-948,7546387	-0,114816
Cloud_Mesh_Distance_ICA_R0_R3	4,67299652	-93,09057617	-948,7399292	-0,176338
Cloud_Mesh_Distance_ICA_R0_R3	3,82926178	-92,89239502	-948,7235718	0,025871
Cloud_Mesh_Distance_ICA_R0_R3	4,39548492	-93,03730774	-948,7620239	-0,080606
Cloud_Mesh_Distance_ICA_R0_R3	4,42986298	-92,84373474	-948,7617188	-0,061488
Cloud_Mesh_Distance_ICA_R0_R3	4,53671265	-92,91149902	-948,7577515	-0,084622
Cloud_Mesh_Distance_ICA_R0_R3	4,8470993	-92,97463989	-948,7183838	-0,114245
Cloud_Mesh_Distance_ICA_R0_R3	5,28631592	-93,04246521	-948,616394	-0,305402
Cloud_Mesh_Distance_ICA_R0_R3	4,37891388	-92,73948669	-948,7554321	-0,043967
Cloud_Mesh_Distance_ICA_R0_R3	4,59515381	-92,7684021	-948,7426148	-0,075688
Cloud_Mesh_Distance_ICA_R0_R3	4,68691254	-92,90023804	-948,7410889	-0,097211
Cloud_Mesh_Distance_ICA_R0_R3	4,57254791	-92,64266968	-948,7270508	-0,05735
Cloud_Mesh_Distance_ICA_R0_R3	3,39144897	-92,99281311	-948,6355591	0,210452
Cloud_Mesh_Distance_ICA_R0_R3	3,54871368	-93,04916382	-948,6634522	0,147673
Cloud_Mesh_Distance_ICA_R0_R3	3,98313141	-93,12684631	-948,727417	-0,057536
Cloud_Mesh_Distance_ICA_R0_R3	4,14322662	-93,13491821	-948,7442017	-0,07226
Cloud_Mesh_Distance_ICA_R0_R3	3,41520691	-92,76370239	-948,6480713	0,113408
Cloud_Mesh_Distance_ICA_R0_R3	3,60520172	-92,83129883	-948,6880493	0,068711
Cloud_Mesh_Distance_ICA_R0_R3	3,39614868	-92,86630249	-948,6437378	0,132273
Cloud_Mesh_Distance_ICA_R0_R3	3,75285339	-92,88447571	-948,7121582	0,038459
Cloud_Mesh_Distance_ICA_R0_R3	3,7733078	-92,8228302	-948,7160034	0,041105
Cloud_Mesh_Distance_ICA_R0_R3	3,99034119	-92,8722229	-948,7453003	0,003613
Cloud_Mesh_Distance_ICA_R0_R3	3,76059723	-93,01683044	-948,7051392	0,026591
Cloud_Mesh_Distance_ICA_R0_R3	4,14031982	-93,00317383	-948,7557373	-0,035575
Cloud_Mesh_Distance_ICA_R0_R3	5,03961945	-93,07608032	-948,6798096	-0,170798
Cloud_Mesh_Distance_ICA_R0_R3	3,26151276	-92,57608032	-948,5895386	0,265276
Cloud_Mesh_Distance_ICA_R0_R3	3,48261261	-92,60585022	-948,6529541	0,111574
Cloud_Mesh_Distance_ICA_R0_R3	3,86171722	-92,70535278	-948,725708	0,042415
Cloud_Mesh_Distance_ICA_R0_R3	3,84965515	-92,83074951	-948,7283936	0,029364
Cloud_Mesh_Distance_ICA_R0_R3	4,30627441	-92,82226563	-948,7632446	-0,040587
Cloud_Mesh_Distance_ICA_R0_R3	4,19224548	-92,87210083	-948,7612305	-0,032268
Cloud_Mesh_Distance_ICA_R0_R3	4,07385254	-92,67466736	-948,7439575	0,013769
Cloud_Mesh_Distance_ICA_R0_R3	4,2306366	-92,73077393	-948,7557983	-0,018488
Cloud_Mesh_Distance_ICA_R0_R3	5,19510651	-93,0556488	-948,6424561	-0,246317
Cloud_Mesh_Distance_ICA_R0_R3	4,99937439	-92,81800842	-948,6730957	-0,09651
Cloud_Mesh_Distance_ICA_R0_R3	5,38581085	-92,97021484	-948,5757446	-0,290842
Cloud_Mesh_Distance_ICA_R0_R3	3,3265152	-92,59927368	-948,6124878	0,204163
Cloud_Mesh_Distance_ICA_R0_R3	3,67466736	-92,43734741	-948,6708984	0,106138
Cloud_Mesh_Distance_ICA_R0_R3	3,50965118	-92,43850708	-948,6364136	0,124138
Cloud_Mesh_Distance_ICA_R0_R3	3,695961	-92,61158752	-948,6965332	0,081151
Cloud_Mesh_Distance_ICA_R0_R3	4,0501709	-92,52444458	-948,7250977	0,037429
Cloud_Mesh_Distance_ICA_R0_R3	4,48461151	-92,71485901	-948,746521	-0,058733
Cloud_Mesh_Distance_ICA_R0_R3	4,73513794	-92,74952698	-948,7197876	-0,088317
Cloud_Mesh_Distance_ICA_R0_R3	4,82958984	-92,81173706	-948,7104492	-0,093778
Cloud_Mesh_Distance_ICA_R0_R3	5,20809174	-92,9147644	-948,6292725	-0,172896
Cloud_Mesh_Distance_ICA_R0_R3	5,4912796	-92,84884644	-948,5115356	-0,162553
Cloud_Mesh_Distance_ICA_R0_R3	5,51987457	-92,98530579	-948,5244141	-0,292948
Cloud_Mesh_Distance_ICA_R0_R3	3,86565399	-92,48632813	-948,7056274	0,068584
Cloud_Mesh_Distance_ICA_R0_R3	4,05796051	-92,33653259	-948,6922607	0,058103

Cloud_Mesh_Distance_ICA_R0_R3	4,42562866	-92,6056366	-948,7354126	-0,03456
Cloud_Mesh_Distance_ICA_R0_R3	4,23362732	-92,55337524	-948,7333984	0,00171
Cloud_Mesh_Distance_ICA_R0_R3	4,40396118	-92,40063477	-948,6950073	-0,006685
Cloud_Mesh_Distance_ICA_R0_R3	4,20500183	-92,41047668	-948,7081299	0,021854
Cloud_Mesh_Distance_ICA_R0_R3	4,68565369	-92,6541748	-948,713501	-0,072052
Cloud_Mesh_Distance_ICA_R0_R3	5,01230621	-92,66001892	-948,640625	-0,082311
Cloud_Mesh_Distance_ICA_R0_R3	4,85222626	-92,64117432	-948,6773682	-0,078646
Cloud_Mesh_Distance_ICA_R0_R3	4,84805298	-92,46386719	-948,6324463	-0,063058
Cloud_Mesh_Distance_ICA_R0_R3	5,16352081	-92,67976379	-948,5983276	-0,080884
Cloud_Mesh_Distance_ICA_R0_R3	5,34479523	-92,78283691	-948,5583496	-0,138117
Cloud_Mesh_Distance_ICA_R0_R3	5,32512665	-92,61135864	-948,5151367	-0,07129
Cloud_Mesh_Distance_ICA_R0_R3	3,72621155	-92,29182434	-948,6506958	0,109877
Cloud_Mesh_Distance_ICA_R0_R3	3,68005371	-92,20838928	-948,6226196	0,12061
Cloud_Mesh_Distance_ICA_R0_R3	3,85832977	-92,31765747	-948,6741943	0,087915
Cloud_Mesh_Distance_ICA_R0_R3	3,82717896	-92,18017578	-948,6394653	0,104106
Cloud_Mesh_Distance_ICA_R0_R3	4,14840698	-92,19595337	-948,6608276	0,053414
Cloud_Mesh_Distance_ICA_R0_R3	4,58747101	-92,28509521	-948,6372681	-0,016248
Cloud_Mesh_Distance_ICA_R0_R3	4,64829254	-92,4967804	-948,6854248	-0,05134
Cloud_Mesh_Distance_ICA_R0_R3	4,77017212	-92,33053589	-948,6105957	-0,036434
Cloud_Mesh_Distance_ICA_R0_R3	5,01537323	-92,51881409	-948,5999756	-0,067945
Cloud_Mesh_Distance_ICA_R0_R3	4,26982117	-92,26036072	-948,6719971	0,030321
Cloud_Mesh_Distance_ICA_R0_R3	3,15422821	-92,94342041	-948,579773	0,363432
Cloud_Mesh_Distance_ICA_R0_R3	3,2492218	-93,00721741	-948,6004639	0,314934
Cloud_Mesh_Distance_ICA_R0_R3	2,96427155	-92,90361023	-948,5267334	0,537657
Cloud_Mesh_Distance_ICA_R0_R3	3,293396	-92,92532349	-948,6164551	0,234301
Cloud_Mesh_Distance_ICA_R0_R3	3,15672302	-92,75894165	-948,5772705	0,338085
Cloud_Mesh_Distance_ICA_R0_R3	3,00797272	-92,73152161	-948,5292358	0,491138
Cloud_Mesh_Distance_ICA_R0_R3	2,93144226	-92,62646484	-948,4880981	0,563621
Cloud_Mesh_Distance_ICA_R0_R3	3,23987579	-92,64173889	-948,5925293	0,275931
Cloud_Mesh_Distance_ICA_R0_R3	3,28678894	-92,82421875	-948,6168823	0,209787
Cloud_Mesh_Distance_ICA_R0_R3	3,31639862	-92,69734192	-948,6188355	0,197659
Cloud_Mesh_Distance_ICA_R0_R3	3,33921051	-92,48217773	-948,5977783	0,177083
Cloud_Mesh_Distance_ICA_R0_R3	3,14260864	-92,58584595	-948,5539551	0,366215
Cloud_Mesh_Distance_ICA_R0_R3	3,35249329	-92,30892944	-948,5617676	0,142611
Cloud_Mesh_Distance_ICA_R0_R3	3,46385956	-92,35148621	-948,6065674	0,135007
Cloud_Mesh_Distance_ICA_R0_R3	5,62094116	-92,90805054	-948,4639893	-0,270973
Cloud_Mesh_Distance_ICA_R0_R3	3,22355652	-92,24346924	-948,4979248	0,176024
Cloud_Mesh_Distance_ICA_R0_R3	3,28100586	-92,11914063	-948,4768677	0,167404
Cloud_Mesh_Distance_ICA_R0_R3	3,59575653	-92,32194519	-948,632019	0,124066
Cloud_Mesh_Distance_ICA_R0_R3	5,20289612	-92,4826355	-948,519104	-0,055871
Cloud_Mesh_Distance_ICA_R0_R3	5,46742249	-92,69308472	-948,4781494	-0,078541
Cloud_Mesh_Distance_ICA_R0_R3	5,32469177	-92,5062561	-948,4751587	-0,051816
Cloud_Mesh_Distance_ICA_R0_R3	5,58969116	-92,77249146	-948,4431763	-0,165545
Cloud_Mesh_Distance_ICA_R0_R3	5,60822296	-92,62094116	-948,3740845	-0,097542
Cloud_Mesh_Distance_ICA_R0_R3	5,76960754	-92,64292908	-948,2821655	-0,222715
Cloud_Mesh_Distance_ICA_R0_R3	5,75118256	-92,80805969	-948,3638916	-0,315013
Cloud_Mesh_Distance_ICA_R0_R3	3,37906647	-92,16925049	-948,5291138	0,141492
Cloud_Mesh_Distance_ICA_R0_R3	3,52796173	-92,20921326	-948,5869141	0,134721
Cloud_Mesh_Distance_ICA_R0_R3	3,72717285	-92,04141235	-948,5848389	0,120691
Cloud_Mesh_Distance_ICA_R0_R3	3,97190094	-92,04396057	-948,6176758	0,089327

Cloud_Mesh_Distance_ICA_R0_R3	4,00244904	-92,1889801	-948,6567383	0,079677
Cloud_Mesh_Distance_ICA_R0_R3	4,52528381	-92,1085968	-948,59552	0,011582
Cloud_Mesh_Distance_ICA_R0_R3	4,71997833	-92,17382813	-948,569519	-0,013919
Cloud_Mesh_Distance_ICA_R0_R3	5,00373077	-92,23617554	-948,4963989	-0,029635
Cloud_Mesh_Distance_ICA_R0_R3	5,06665039	-92,378479	-948,5317993	-0,048788
Cloud_Mesh_Distance_ICA_R0_R3	4,9310379	-92,35697937	-948,5718384	-0,042145
Cloud_Mesh_Distance_ICA_R0_R3	5,43095398	-92,54182434	-948,4373169	-0,059012
Cloud_Mesh_Distance_ICA_R0_R3	5,51778412	-92,43804932	-948,3355103	-0,045623
Cloud_Mesh_Distance_ICA_R0_R3	5,71634674	-92,478302	-948,2221069	-0,067682
Cloud_Mesh_Distance_ICA_R0_R3	5,88285828	-92,73112488	-948,2481079	-0,291295
Cloud_Mesh_Distance_ICA_R0_R3	5,88742065	-92,5944519	-948,1655884	-0,141334
Cloud_Mesh_Distance_ICA_R0_R3	3,50534058	-92,06706238	-948,5367432	0,137386
Cloud_Mesh_Distance_ICA_R0_R3	3,77835083	-91,86523438	-948,5427856	0,120214
Cloud_Mesh_Distance_ICA_R0_R3	4,16067505	-91,92678833	-948,5899658	0,075902
Cloud_Mesh_Distance_ICA_R0_R3	4,36701965	-92,14581299	-948,6331787	0,029413
Cloud_Mesh_Distance_ICA_R0_R3	4,25606537	-92,13504028	-948,6404419	0,046419
Cloud_Mesh_Distance_ICA_R0_R3	4,14761353	-92,08000183	-948,6311035	0,063638
Cloud_Mesh_Distance_ICA_R0_R3	4,43379974	-92,23469543	-948,6486816	0,008264
Cloud_Mesh_Distance_ICA_R0_R3	5,1105423	-92,1784668	-948,4212036	-0,016516
Cloud_Mesh_Distance_ICA_R0_R3	4,86642456	-92,09701538	-948,4907227	-0,005246
Cloud_Mesh_Distance_ICA_R0_R3	4,86131287	-92,23571777	-948,5489502	-0,026784
Cloud_Mesh_Distance_ICA_R0_R3	3,88928986	-91,74380493	-948,5276489	0,097932
Cloud_Mesh_Distance_ICA_R0_R3	3,97450256	-91,63543701	-948,510376	0,077802
Cloud_Mesh_Distance_ICA_R0_R3	3,96395874	-91,86682129	-948,5690308	0,094465
Cloud_Mesh_Distance_ICA_R0_R3	4,33672333	-92,01539612	-948,5995483	0,041192
Cloud_Mesh_Distance_ICA_R0_R3	4,53443146	-91,79391479	-948,49646	0,038334
Cloud_Mesh_Distance_ICA_R0_R3	4,66372681	-91,85783386	-948,475647	0,023079
Cloud_Mesh_Distance_ICA_R0_R3	4,52108002	-91,92988586	-948,5404053	0,032756
Cloud_Mesh_Distance_ICA_R0_R3	4,68843842	-92,01229858	-948,5212402	0,004495
Cloud_Mesh_Distance_ICA_R0_R3	4,81723022	-91,92910767	-948,4417725	0,020109
Cloud_Mesh_Distance_ICA_R0_R3	4,15538025	-91,53088379	-948,4950562	0,062934
Cloud_Mesh_Distance_ICA_R0_R3	4,17611694	-91,62619019	-948,5150757	0,070454
Cloud_Mesh_Distance_ICA_R0_R3	4,0432663	-91,70372009	-948,5319824	0,08243
Cloud_Mesh_Distance_ICA_R0_R3	4,17206573	-91,76239014	-948,5471802	0,075599
Cloud_Mesh_Distance_ICA_R0_R3	4,31693268	-91,65446472	-948,5072632	0,05113
Cloud_Mesh_Distance_ICA_R0_R3	4,36210632	-91,81771851	-948,5408936	0,045724
Cloud_Mesh_Distance_ICA_R0_R3	4,2849884	-91,5206604	-948,4833374	0,039562
Cloud_Mesh_Distance_ICA_R0_R3	4,41947174	-91,54733276	-948,4641113	0,05072
Cloud_Mesh_Distance_ICA_R0_R3	3,02626038	-92,48304749	-948,492981	0,41611
Cloud_Mesh_Distance_ICA_R0_R3	3,18710327	-92,40429688	-948,531189	0,254344
Cloud_Mesh_Distance_ICA_R0_R3	2,86705017	-92,13568115	-948,3068237	0,519123
Cloud_Mesh_Distance_ICA_R0_R3	2,89646912	-92,27423096	-948,3769531	0,469692
Cloud_Mesh_Distance_ICA_R0_R3	3,04364014	-92,33349609	-948,4570923	0,337738
Cloud_Mesh_Distance_ICA_R0_R3	3,09822845	-92,13369751	-948,4081421	0,2926
Cloud_Mesh_Distance_ICA_R0_R3	3,00844574	-92,0151062	-948,3170776	0,435073
Cloud_Mesh_Distance_ICA_R0_R3	3,07777405	-91,93540955	-948,3133545	0,341738
Cloud_Mesh_Distance_ICA_R0_R3	3,13842773	-91,9808197	-948,3615112	0,331734
Cloud_Mesh_Distance_ICA_R0_R3	3,38569641	-92,06939697	-948,4979248	0,141822
Cloud_Mesh_Distance_ICA_R0_R3	3,31554413	-91,98200989	-948,4381104	0,195869
Cloud_Mesh_Distance_ICA_R0_R3	5,42874908	-92,30023193	-948,3101807	-0,02688

Cloud_Mesh_Distance_ICA_R0_R3	5,33547974	-92,39553833	-948,4174194	-0,039943
Cloud_Mesh_Distance_ICA_R0_R3	3,44472504	-92,00692749	-948,4953003	0,136744
Cloud_Mesh_Distance_ICA_R0_R3	3,56938934	-91,90612793	-948,5018921	0,132732
Cloud_Mesh_Distance_ICA_R0_R3	3,7392807	-91,77171326	-948,5059204	0,1196
Cloud_Mesh_Distance_ICA_R0_R3	3,62339783	-91,71711731	-948,4558106	0,119543
Cloud_Mesh_Distance_ICA_R0_R3	5,16627502	-92,31057739	-948,4594116	-0,036227
Cloud_Mesh_Distance_ICA_R0_R3	5,26670837	-92,2023468	-948,3497925	-0,01481
Cloud_Mesh_Distance_ICA_R0_R3	5,56906891	-92,20149231	-948,1343384	-0,008853
Cloud_Mesh_Distance_ICA_R0_R3	5,58057404	-92,30741882	-948,206604	-0,026665
Cloud_Mesh_Distance_ICA_R0_R3	3,67937469	-91,55003357	-948,4234009	0,100295
Cloud_Mesh_Distance_ICA_R0_R3	3,8300705	-91,62442017	-948,4853516	0,099989
Cloud_Mesh_Distance_ICA_R0_R3	4,77220154	-91,78918457	-948,4085693	0,033559
Cloud_Mesh_Distance_ICA_R0_R3	4,95030975	-91,99990845	-948,4128418	0,006435
Cloud_Mesh_Distance_ICA_R0_R3	5,00797272	-92,10897827	-948,4360352	-0,014299
Cloud_Mesh_Distance_ICA_R0_R3	4,95146942	-91,83032227	-948,3321533	0,026476
Cloud_Mesh_Distance_ICA_R0_R3	5,150177	-91,89854431	-948,2409058	0,024816
Cloud_Mesh_Distance_ICA_R0_R3	5,27417755	-92,0377655	-948,2416992	0,005537
Cloud_Mesh_Distance_ICA_R0_R3	5,10186768	-92,03720093	-948,3504639	0,004699
Cloud_Mesh_Distance_ICA_R0_R3	5,44035339	-92,16334534	-948,2078247	-0,003641
Cloud_Mesh_Distance_ICA_R0_R3	3,71845245	-91,37808228	-948,3920288	0,073293
Cloud_Mesh_Distance_ICA_R0_R3	3,87045288	-91,44268799	-948,4498901	0,073109
Cloud_Mesh_Distance_ICA_R0_R3	4,0764389	-91,59973145	-948,5099487	0,076769
Cloud_Mesh_Distance_ICA_R0_R3	4,47744751	-91,67233276	-948,4785156	0,046399
Cloud_Mesh_Distance_ICA_R0_R3	4,63739014	-91,70928955	-948,4365845	0,036885
Cloud_Mesh_Distance_ICA_R0_R3	4,78450012	-91,62580872	-948,3433838	0,041021
Cloud_Mesh_Distance_ICA_R0_R3	5,2184906	-91,81637573	-948,1345215	0,045069
Cloud_Mesh_Distance_ICA_R0_R3	4,2073288	-91,40701294	-948,4714356	0,040861
Cloud_Mesh_Distance_ICA_R0_R3	4,0262146	-91,50834656	-948,4870606	0,070006
Cloud_Mesh_Distance_ICA_R0_R3	4,06216431	-91,32389832	-948,458374	0,035563
Cloud_Mesh_Distance_ICA_R0_R3	4,42793274	-91,37110901	-948,4298706	0,038759
Cloud_Mesh_Distance_ICA_R0_R3	4,58356476	-91,53045654	-948,4094849	0,036419
Cloud_Mesh_Distance_ICA_R0_R3	3,67533112	-91,13334656	-948,3236084	0,022863
Cloud_Mesh_Distance_ICA_R0_R3	3,78974152	-91,10559082	-948,3696899	0,002806
Cloud_Mesh_Distance_ICA_R0_R3	3,75933075	-91,21517944	-948,3746338	0,032589
Cloud_Mesh_Distance_ICA_R0_R3	3,90049744	-91,26445007	-948,4250488	0,035196
Cloud_Mesh_Distance_ICA_R0_R3	3,90470123	-91,08888245	-948,404419	-0,014299
Cloud_Mesh_Distance_ICA_R0_R3	4,08302307	-91,12284851	-948,4389038	-0,012188
Cloud_Mesh_Distance_ICA_R0_R3	4,60269928	-91,36773682	-948,3677979	0,038401
Cloud_Mesh_Distance_ICA_R0_R3	4,57385254	-91,23406982	-948,3616943	0,020783
Cloud_Mesh_Distance_ICA_R0_R3	4,80984497	-91,31918335	-948,2409668	0,050942
Cloud_Mesh_Distance_ICA_R0_R3	4,72497559	-91,43058777	-948,319519	0,044833
Cloud_Mesh_Distance_ICA_R0_R3	4,86831665	-91,46302795	-948,2382813	0,059129
Cloud_Mesh_Distance_ICA_R0_R3	3,83842468	-91,04330444	-948,3799439	-0,020289
Cloud_Mesh_Distance_ICA_R0_R3	4,0691452	-90,90863037	-948,4280396	-0,072068
Cloud_Mesh_Distance_ICA_R0_R3	4,45930481	-91,17817688	-948,3994751	0,005086
Cloud_Mesh_Distance_ICA_R0_R3	4,28009796	-91,1955719	-948,43927	-0,004546
Cloud_Mesh_Distance_ICA_R0_R3	3,98758698	-90,73501587	-948,4099121	-0,132797
Cloud_Mesh_Distance_ICA_R0_R3	3,98480988	-90,81340027	-948,4107666	-0,108206
Cloud_Mesh_Distance_ICA_R0_R3	4,25566864	-90,73338318	-948,4351196	-0,110656
Cloud_Mesh_Distance_ICA_R0_R3	4,27709198	-90,94763184	-948,4310913	-0,052814

Cloud_Mesh_Distance_ICA_R0_R3	4,09015656	-90,56150818	-948,4295044	-0,191766
Cloud_Mesh_Distance_ICA_R0_R3	4,2213974	-90,54664612	-948,4383545	-0,171639
Cloud_Mesh_Distance_ICA_R0_R3	4,08598328	-90,70629883	-948,4293213	-0,14045
Cloud_Mesh_Distance_ICA_R0_R3	4,35636139	-90,57125854	-948,4252319	-0,12949
Cloud_Mesh_Distance_ICA_R0_R3	4,25282288	-90,40391541	-948,4337158	-0,20721
Cloud_Mesh_Distance_ICA_R0_R3	4,34255219	-90,43711853	-948,4259644	-0,163037
Cloud_Mesh_Distance_ICA_R0_R3	4,32450867	-90,30860901	-948,4203491	-0,204173
Cloud_Mesh_Distance_ICA_R0_R3	2,87937927	-92,00973511	-948,2537231	0,524559
Cloud_Mesh_Distance_ICA_R0_R3	2,8032074	-91,94108582	-948,1812744	0,534198
Cloud_Mesh_Distance_ICA_R0_R3	5,89563751	-92,44343567	-948,0407105	-0,063772
Cloud_Mesh_Distance_ICA_R0_R3	5,85007477	-92,34843445	-947,9977417	-0,043214
Cloud_Mesh_Distance_ICA_R0_R3	2,97116852	-91,90687561	-948,2473755	0,392279
Cloud_Mesh_Distance_ICA_R0_R3	3,42055511	-91,80107117	-948,4107056	0,138881
Cloud_Mesh_Distance_ICA_R0_R3	3,19430542	-91,67706299	-948,2510376	0,142168
Cloud_Mesh_Distance_ICA_R0_R3	3,17891693	-91,84295654	-948,3201294	0,224139
Cloud_Mesh_Distance_ICA_R0_R3	5,63270569	-92,17919922	-948,0551758	-0,002774
Cloud_Mesh_Distance_ICA_R0_R3	5,64761353	-92,24891663	-948,1048584	-0,010586
Cloud_Mesh_Distance_ICA_R0_R3	5,82306671	-92,27859497	-947,9552612	-0,030751
Cloud_Mesh_Distance_ICA_R0_R3	5,74650574	-92,33081055	-948,0848389	-0,030423
Cloud_Mesh_Distance_ICA_R0_R3	3,13682556	-91,53341675	-948,1495972	0,142644
Cloud_Mesh_Distance_ICA_R0_R3	3,22478485	-91,54972839	-948,2084961	0,127805
Cloud_Mesh_Distance_ICA_R0_R3	3,33242798	-91,71133423	-948,3356323	0,139022
Cloud_Mesh_Distance_ICA_R0_R3	3,35959625	-91,58511353	-948,2973023	0,127303
Cloud_Mesh_Distance_ICA_R0_R3	3,49340057	-91,64318848	-948,3817749	0,125891
Cloud_Mesh_Distance_ICA_R0_R3	5,40871429	-91,99285889	-948,0999146	0,021966
Cloud_Mesh_Distance_ICA_R0_R3	5,54989624	-92,10437012	-948,065918	0,003355
Cloud_Mesh_Distance_ICA_R0_R3	5,68476868	-92,11242676	-947,9304199	-0,000479
Cloud_Mesh_Distance_ICA_R0_R3	5,56053162	-91,97549438	-947,925293	0,032911
Cloud_Mesh_Distance_ICA_R0_R3	5,71897125	-92,22407532	-948,0111694	-0,011697
Cloud_Mesh_Distance_ICA_R0_R3	3,53128815	-91,4834137	-948,3441773	0,106178
Cloud_Mesh_Distance_ICA_R0_R3	3,36180878	-91,43130493	-948,2370606	0,114388
Cloud_Mesh_Distance_ICA_R0_R3	5,09845734	-91,63011169	-948,123169	0,066326
Cloud_Mesh_Distance_ICA_R0_R3	5,0828476	-91,73112488	-948,1934815	0,055301
Cloud_Mesh_Distance_ICA_R0_R3	5,27442932	-91,92706299	-948,164856	0,029807
Cloud_Mesh_Distance_ICA_R0_R3	5,3253479	-91,87516785	-948,0809326	0,03966
Cloud_Mesh_Distance_ICA_R0_R3	3,56280518	-91,29516602	-948,3041382	0,080006
Cloud_Mesh_Distance_ICA_R0_R3	5,0338974	-91,61053467	-948,1687012	0,063411
Cloud_Mesh_Distance_ICA_R0_R3	4,9500885	-91,62826538	-948,2410278	0,049728
Cloud_Mesh_Distance_ICA_R0_R3	3,76768494	-91,02281189	-948,3502808	-0,02349
Cloud_Mesh_Distance_ICA_R0_R3	4,57403564	-91,06997681	-948,3482666	0,013513
Cloud_Mesh_Distance_ICA_R0_R3	4,69828033	-91,27478027	-948,3035278	0,04462
Cloud_Mesh_Distance_ICA_R0_R3	3,64574432	-90,99855042	-948,2863159	-0,005722
Cloud_Mesh_Distance_ICA_R0_R3	3,88868713	-90,93713379	-948,388916	-0,054288
Cloud_Mesh_Distance_ICA_R0_R3	3,89144897	-90,76989746	-948,3816528	-0,114419
Cloud_Mesh_Distance_ICA_R0_R3	4,4348526	-91,02096558	-948,4006958	-0,021472
Cloud_Mesh_Distance_ICA_R0_R3	4,67406464	-91,14169312	-948,3009033	0,031431
Cloud_Mesh_Distance_ICA_R0_R3	4,79994965	-91,15052795	-948,2167969	0,05304
Cloud_Mesh_Distance_ICA_R0_R3	4,90090179	-91,07196045	-948,1140747	0,077226
Cloud_Mesh_Distance_ICA_R0_R3	3,77230072	-90,75021362	-948,3289795	-0,102527
Cloud_Mesh_Distance_ICA_R0_R3	3,71659851	-90,65847778	-948,2926025	-0,122718

Cloud_Mesh_Distance_ICA_R0_R3	3,98632813	-90,64411926	-948,4088745	-0,16004
Cloud_Mesh_Distance_ICA_R0_R3	4,37119293	-90,78559875	-948,4188843	-0,077988
Cloud_Mesh_Distance_ICA_R0_R3	4,43173981	-90,86625671	-948,4014893	-0,053141
Cloud_Mesh_Distance_ICA_R0_R3	4,57377625	-90,86885071	-948,3458252	-0,014884
Cloud_Mesh_Distance_ICA_R0_R3	4,71901703	-90,97369385	-948,2605591	0,025428
Cloud_Mesh_Distance_ICA_R0_R3	4,72292328	-90,75488281	-948,2515869	0,016088
Cloud_Mesh_Distance_ICA_R0_R3	4,61919403	-90,6829834	-948,3234863	-0,029736
Cloud_Mesh_Distance_ICA_R0_R3	4,83618164	-90,98965454	-948,1662598	0,060024
Cloud_Mesh_Distance_ICA_R0_R3	4,83296967	-90,83041382	-948,1575928	0,052657
Cloud_Mesh_Distance_ICA_R0_R3	3,77588654	-90,29696655	-948,302124	-0,24633
Cloud_Mesh_Distance_ICA_R0_R3	3,88948822	-90,39251709	-948,366272	-0,241633
Cloud_Mesh_Distance_ICA_R0_R3	3,85536957	-90,60066223	-948,3608398	-0,162449
Cloud_Mesh_Distance_ICA_R0_R3	4,14392853	-90,43032837	-948,4328003	-0,227479
Cloud_Mesh_Distance_ICA_R0_R3	4,38861084	-90,67570496	-948,4180298	-0,097166
Cloud_Mesh_Distance_ICA_R0_R3	4,45481873	-90,58712769	-948,4004517	-0,093098
Cloud_Mesh_Distance_ICA_R0_R3	4,49030304	-90,71151733	-948,3847656	-0,059171
Cloud_Mesh_Distance_ICA_R0_R3	4,57227325	-90,52423096	-948,348999	-0,066757
Cloud_Mesh_Distance_ICA_R0_R3	3,91228485	-90,20399475	-948,3592529	-0,305789
Cloud_Mesh_Distance_ICA_R0_R3	4,00525665	-90,48497009	-948,4092407	-0,220699
Cloud_Mesh_Distance_ICA_R0_R3	4,45159912	-90,46452332	-948,4008789	-0,118782
Cloud_Mesh_Distance_ICA_R0_R3	4,69592285	-90,3420105	-948,2595215	-0,044139
Cloud_Mesh_Distance_ICA_R0_R3	4,8070755	-90,43457031	-948,1635742	0,018871
Cloud_Mesh_Distance_ICA_R0_R3	4,71710205	-90,55783081	-948,2543945	-0,006481
Cloud_Mesh_Distance_ICA_R0_R3	3,92525482	-89,99255371	-948,3225708	-0,384294
Cloud_Mesh_Distance_ICA_R0_R3	4,21208191	-90,31349182	-948,4280396	-0,244109
Cloud_Mesh_Distance_ICA_R0_R3	4,06549072	-90,2855072	-948,4111328	-0,287824
Cloud_Mesh_Distance_ICA_R0_R3	4,42243958	-90,3487854	-948,4039307	-0,156467
Cloud_Mesh_Distance_ICA_R0_R3	4,43544006	-90,21217346	-948,3842773	-0,178018
Cloud_Mesh_Distance_ICA_R0_R3	4,59737396	-90,14215088	-948,2992554	-0,119154
Cloud_Mesh_Distance_ICA_R0_R3	4,53594208	-90,33581543	-948,3596191	-0,108725
Cloud_Mesh_Distance_ICA_R0_R3	3,89540863	-89,92955017	-948,2876587	-0,387468
Cloud_Mesh_Distance_ICA_R0_R3	3,97758484	-89,91191101	-948,3154297	-0,417347
Cloud_Mesh_Distance_ICA_R0_R3	4,04140472	-90,04826355	-948,3724976	-0,37168
Cloud_Mesh_Distance_ICA_R0_R3	4,2539444	-90,15341187	-948,4072876	-0,27771
Cloud_Mesh_Distance_ICA_R0_R3	4,23357391	-89,94171143	-948,3604736	-0,324141
Cloud_Mesh_Distance_ICA_R0_R3	4,43530273	-90,00906372	-948,3416748	-0,212078
Cloud_Mesh_Distance_ICA_R0_R3	4,67604065	-90,01519775	-948,2079468	-0,086633
Cloud_Mesh_Distance_ICA_R0_R3	4,0067215	-89,77481079	-948,2620239	-0,448063
Cloud_Mesh_Distance_ICA_R0_R3	4,08934021	-89,88513184	-948,333313	-0,40211
Cloud_Mesh_Distance_ICA_R0_R3	4,1975174	-89,78593445	-948,2998657	-0,370077
Cloud_Mesh_Distance_ICA_R0_R3	4,51131439	-89,87033081	-948,2620239	-0,199936
Cloud_Mesh_Distance_ICA_R0_R3	6,04095459	-92,3704834	-947,795105	-0,085791
Cloud_Mesh_Distance_ICA_R0_R3	6,07038116	-92,44828796	-947,857666	-0,137364
Cloud_Mesh_Distance_ICA_R0_R3	2,96192169	-91,75460815	-948,1638184	0,311968
Cloud_Mesh_Distance_ICA_R0_R3	2,86336517	-91,86032104	-948,1694946	0,442043
Cloud_Mesh_Distance_ICA_R0_R3	2,9416275	-91,62295532	-948,0789185	0,302563
Cloud_Mesh_Distance_ICA_R0_R3	5,94593048	-92,34410095	-947,8843994	-0,051508
Cloud_Mesh_Distance_ICA_R0_R3	6,0473938	-92,30143738	-947,685791	-0,078853
Cloud_Mesh_Distance_ICA_R0_R3	3,08605194	-91,610672	-948,157959	0,177072
Cloud_Mesh_Distance_ICA_R0_R3	5,78827667	-92,19418335	-947,9000244	-0,020124

Cloud_Mesh_Distance_ICA_R0_R3	3,03449249	-91,51812744	-948,0778809	0,201304
Cloud_Mesh_Distance_ICA_R0_R3	3,04060364	-91,37565613	-948,0028076	0,173752
Cloud_Mesh_Distance_ICA_R0_R3	3,18690491	-91,445755	-948,1384277	0,134889
Cloud_Mesh_Distance_ICA_R0_R3	5,18299866	-91,68545532	-948,078186	0,067087
Cloud_Mesh_Distance_ICA_R0_R3	5,30058289	-91,76243591	-948,015686	0,052022
Cloud_Mesh_Distance_ICA_R0_R3	5,42869568	-91,86109924	-947,9609985	0,044613
Cloud_Mesh_Distance_ICA_R0_R3	5,49433899	-91,80270386	-947,8188477	0,059189
Cloud_Mesh_Distance_ICA_R0_R3	5,5217514	-91,8825531	-947,8713379	0,046746
Cloud_Mesh_Distance_ICA_R0_R3	3,36367798	-91,26359558	-948,1762695	0,089371
Cloud_Mesh_Distance_ICA_R0_R3	3,28294373	-91,36985779	-948,1640015	0,119754
Cloud_Mesh_Distance_ICA_R0_R3	3,20752716	-91,32220459	-948,0922241	0,122049
Cloud_Mesh_Distance_ICA_R0_R3	3,52263641	-91,13046265	-948,2388916	0,045999
Cloud_Mesh_Distance_ICA_R0_R3	5,06270599	-91,54905701	-948,1131592	0,074145
Cloud_Mesh_Distance_ICA_R0_R3	5,06501007	-91,4347229	-948,055603	0,084202
Cloud_Mesh_Distance_ICA_R0_R3	5,17333221	-91,41664124	-947,9163818	0,106247
Cloud_Mesh_Distance_ICA_R0_R3	5,13937378	-91,5559082	-948,0407105	0,073819
Cloud_Mesh_Distance_ICA_R0_R3	5,27888489	-91,45755005	-947,7922363	0,114204
Cloud_Mesh_Distance_ICA_R0_R3	5,26325226	-91,59803772	-947,9268189	0,07935
Cloud_Mesh_Distance_ICA_R0_R3	3,2862854	-90,82499695	-947,9562988	0,045307
Cloud_Mesh_Distance_ICA_R0_R3	3,28952026	-91,04589844	-948,0400391	0,083199
Cloud_Mesh_Distance_ICA_R0_R3	3,36994171	-90,93069458	-948,0725708	0,044225
Cloud_Mesh_Distance_ICA_R0_R3	3,37918854	-91,09088135	-948,1279907	0,061504
Cloud_Mesh_Distance_ICA_R0_R3	5,08240509	-91,30162048	-947,9735107	0,100662
Cloud_Mesh_Distance_ICA_R0_R3	4,98671722	-91,49572754	-948,1561279	0,079399
Cloud_Mesh_Distance_ICA_R0_R3	5,15246582	-91,23425293	-947,8411865	0,12152
Cloud_Mesh_Distance_ICA_R0_R3	3,38728333	-90,7885437	-948,0499878	0,000641
Cloud_Mesh_Distance_ICA_R0_R3	3,48754883	-90,80158997	-948,1434326	-0,020255
Cloud_Mesh_Distance_ICA_R0_R3	3,50621796	-90,96702576	-948,1904297	0,010243
Cloud_Mesh_Distance_ICA_R0_R3	3,7664032	-90,88723755	-948,3369141	-0,062274
Cloud_Mesh_Distance_ICA_R0_R3	3,63843536	-90,81617737	-948,2583008	-0,060857
Cloud_Mesh_Distance_ICA_R0_R3	4,95350647	-91,26947021	-948,1069946	0,076912
Cloud_Mesh_Distance_ICA_R0_R3	5,0691452	-91,14120483	-947,9265137	0,120464
Cloud_Mesh_Distance_ICA_R0_R3	3,44657135	-90,66275024	-948,0794678	-0,043006
Cloud_Mesh_Distance_ICA_R0_R3	3,58976746	-90,61767578	-948,2015381	-0,094419
Cloud_Mesh_Distance_ICA_R0_R3	4,92047119	-90,91195679	-948,0690308	0,077141
Cloud_Mesh_Distance_ICA_R0_R3	5,01441193	-90,81152344	-947,9179077	0,120623
Cloud_Mesh_Distance_ICA_R0_R3	3,73916626	-90,49307251	-948,2962647	-0,177911
Cloud_Mesh_Distance_ICA_R0_R3	3,63292694	-90,38986206	-948,2091675	-0,168925
Cloud_Mesh_Distance_ICA_R0_R3	4,84558105	-90,61141968	-948,1317749	0,044652
Cloud_Mesh_Distance_ICA_R0_R3	4,94080353	-90,57659912	-947,9985962	0,085826
Cloud_Mesh_Distance_ICA_R0_R3	4,92940521	-90,73208618	-948,0362549	0,086564
Cloud_Mesh_Distance_ICA_R0_R3	3,6919632	-90,17050171	-948,220459	-0,250386
Cloud_Mesh_Distance_ICA_R0_R3	3,54139709	-90,28601074	-948,0994873	-0,162601
Cloud_Mesh_Distance_ICA_R0_R3	3,71704102	-90,00828552	-948,1913452	-0,305655
Cloud_Mesh_Distance_ICA_R0_R3	3,81780243	-90,14961243	-948,3051758	-0,303239
Cloud_Mesh_Distance_ICA_R0_R3	3,82225037	-90,02223206	-948,2772217	-0,339628
Cloud_Mesh_Distance_ICA_R0_R3	3,90126801	-90,07815552	-948,3336182	-0,346062
Cloud_Mesh_Distance_ICA_R0_R3	4,82644653	-90,13549805	-948,0821533	0,000496
Cloud_Mesh_Distance_ICA_R0_R3	4,74328613	-90,14318848	-948,1838989	-0,035769
Cloud_Mesh_Distance_ICA_R0_R3	4,82072449	-90,2749176	-948,1271362	0,013975

Cloud_Mesh_Distance_ICA_R0_R3	3,82567596	-89,87045288	-948,2166138	-0,389268
Cloud_Mesh_Distance_ICA_R0_R3	3,92594147	-89,8706665	-948,276062	-0,415984
Cloud_Mesh_Distance_ICA_R0_R3	4,58013916	-89,97523499	-948,2645264	-0,148951
Cloud_Mesh_Distance_ICA_R0_R3	3,87918091	-89,69720459	-948,1365967	-0,481639
Cloud_Mesh_Distance_ICA_R0_R3	4,36989594	-89,82580566	-948,2958984	-0,278529
Cloud_Mesh_Distance_ICA_R0_R3	4,3138504	-89,69792175	-948,2411499	-0,338912
Cloud_Mesh_Distance_ICA_R0_R3	4,63592529	-89,86581421	-948,1763306	-0,131405
Cloud_Mesh_Distance_ICA_R0_R3	4,7748642	-89,97323608	-948,0812378	-0,04558
Cloud_Mesh_Distance_ICA_R0_R3	4,70970917	-89,81976318	-948,0684815	-0,105429
Cloud_Mesh_Distance_ICA_R0_R3	4,05885315	-89,52589417	-948,0761108	-0,526537
Cloud_Mesh_Distance_ICA_R0_R3	4,03192139	-89,61074829	-948,1542969	-0,498839
Cloud_Mesh_Distance_ICA_R0_R3	4,17105103	-89,65014648	-948,2180176	-0,415742
Cloud_Mesh_Distance_ICA_R0_R3	4,49081421	-89,70887756	-948,1813965	-0,240884
Cloud_Mesh_Distance_ICA_R0_R3	4,32472229	-89,57096863	-948,1397705	-0,353927
Cloud_Mesh_Distance_ICA_R0_R3	4,5849762	-89,62799072	-948,0340576	-0,190682
Cloud_Mesh_Distance_ICA_R0_R3	4,68592072	-89,72444153	-948,0103149	-0,119934
Cloud_Mesh_Distance_ICA_R0_R3	4,63118744	-89,74362183	-948,1001587	-0,155831
Cloud_Mesh_Distance_ICA_R0_R3	4,15670013	-89,53686523	-948,1175537	-0,457139
Cloud_Mesh_Distance_ICA_R0_R3	4,24760437	-89,45568848	-948,0139771	-0,397926
Cloud_Mesh_Distance_ICA_R0_R3	4,40032959	-89,4894104	-948,0145264	-0,298693
Cloud_Mesh_Distance_ICA_R0_R3	4,46734619	-89,57562256	-948,0819092	-0,26287
Cloud_Mesh_Distance_ICA_R0_R3	6,10856628	-92,38912964	-947,7293091	-0,147502
Cloud_Mesh_Distance_ICA_R0_R3	2,87519836	-91,5486145	-947,993103	0,352242
Cloud_Mesh_Distance_ICA_R0_R3	5,89707184	-92,23446655	-947,80896	-0,040033
Cloud_Mesh_Distance_ICA_R0_R3	6,16295624	-92,37208557	-947,6211548	-0,193137
Cloud_Mesh_Distance_ICA_R0_R3	6,07994843	-92,22462463	-947,4831543	-0,119272
Cloud_Mesh_Distance_ICA_R0_R3	2,84513855	-91,440979	-947,9025269	0,358942
Cloud_Mesh_Distance_ICA_R0_R3	2,75094604	-91,3682251	-947,7785645	0,429657
Cloud_Mesh_Distance_ICA_R0_R3	2,95091248	-91,47322083	-947,9973755	0,264883
Cloud_Mesh_Distance_ICA_R0_R3	5,60144043	-91,88061523	-947,7571411	0,040962
Cloud_Mesh_Distance_ICA_R0_R3	5,71274567	-91,99359131	-947,7407837	0,020857
Cloud_Mesh_Distance_ICA_R0_R3	5,82023621	-92,12187195	-947,7614136	-0,00912
Cloud_Mesh_Distance_ICA_R0_R3	2,89295197	-91,28274536	-947,8304443	0,26928
Cloud_Mesh_Distance_ICA_R0_R3	3,06271362	-91,22262573	-947,9315796	0,144479
Cloud_Mesh_Distance_ICA_R0_R3	3,13478088	-91,27780151	-948,019043	0,131305
Cloud_Mesh_Distance_ICA_R0_R3	5,46369934	-91,69955444	-947,7449341	0,075726
Cloud_Mesh_Distance_ICA_R0_R3	5,55510712	-91,76065063	-947,6713867	0,07034
Cloud_Mesh_Distance_ICA_R0_R3	5,68309784	-91,91065979	-947,6656494	0,034009
Cloud_Mesh_Distance_ICA_R0_R3	5,66743469	-91,83241272	-947,5715332	0,061207
Cloud_Mesh_Distance_ICA_R0_R3	3,22065735	-91,17373657	-948,036499	0,114031
Cloud_Mesh_Distance_ICA_R0_R3	5,33796692	-91,37710571	-947,6066895	0,132244
Cloud_Mesh_Distance_ICA_R0_R3	5,38858032	-91,71865845	-947,8729858	0,070489
Cloud_Mesh_Distance_ICA_R0_R3	5,38143158	-91,57066345	-947,7331543	0,101164
Cloud_Mesh_Distance_ICA_R0_R3	5,43421173	-91,49902344	-947,5442505	0,125923
Cloud_Mesh_Distance_ICA_R0_R3	3,21166992	-91,0456543	-947,97052	0,099971
Cloud_Mesh_Distance_ICA_R0_R3	3,26473999	-90,96179199	-947,9858398	0,077631
Cloud_Mesh_Distance_ICA_R0_R3	5,23705292	-91,31539917	-947,7498169	0,133498
Cloud_Mesh_Distance_ICA_R0_R3	3,28481293	-90,65896606	-947,8885498	0,029247
Cloud_Mesh_Distance_ICA_R0_R3	4,99087524	-91,02758789	-948,0005493	0,103126
Cloud_Mesh_Distance_ICA_R0_R3	3,38343048	-90,56613159	-947,9846191	-0,02851

Cloud_Mesh_Distance_ICA_R0_R3	3,35552216	-90,70281982	-947,9924927	0,008876
Cloud_Mesh_Distance_ICA_R0_R3	5,07653046	-90,95106506	-947,8474731	0,12885
Cloud_Mesh_Distance_ICA_R0_R3	3,42196655	-90,39343262	-947,9795532	-0,084252
Cloud_Mesh_Distance_ICA_R0_R3	3,49691772	-90,48532104	-948,0960693	-0,099066
Cloud_Mesh_Distance_ICA_R0_R3	5,08261871	-90,79655457	-947,7748413	0,140487
Cloud_Mesh_Distance_ICA_R0_R3	3,36201477	-90,32804871	-947,8477173	-0,06432
Cloud_Mesh_Distance_ICA_R0_R3	3,46295929	-90,21948242	-947,9664917	-0,139315
Cloud_Mesh_Distance_ICA_R0_R3	4,99518585	-90,5012207	-947,8796387	0,106697
Cloud_Mesh_Distance_ICA_R0_R3	5,06460571	-90,61862183	-947,7453003	0,145639
Cloud_Mesh_Distance_ICA_R0_R3	4,99391937	-90,64718628	-947,918335	0,104712
Cloud_Mesh_Distance_ICA_R0_R3	3,49468231	-90,08216858	-947,9329834	-0,179785
Cloud_Mesh_Distance_ICA_R0_R3	3,58123016	-89,92993164	-947,9598389	-0,269044
Cloud_Mesh_Distance_ICA_R0_R3	3,58696747	-90,08198547	-948,0801392	-0,234278
Cloud_Mesh_Distance_ICA_R0_R3	4,88114929	-90,21498108	-948,0230713	0,028045
Cloud_Mesh_Distance_ICA_R0_R3	4,93627167	-90,20225525	-947,899231	0,069866
Cloud_Mesh_Distance_ICA_R0_R3	4,91388702	-90,3924408	-948,0129395	0,061882
Cloud_Mesh_Distance_ICA_R0_R3	4,98918915	-90,3392334	-947,8280029	0,10603
Cloud_Mesh_Distance_ICA_R0_R3	3,68178558	-89,93017578	-948,1141968	-0,313264
Cloud_Mesh_Distance_ICA_R0_R3	4,84627533	-89,95285034	-947,937561	0,000444
Cloud_Mesh_Distance_ICA_R0_R3	4,91003418	-90,02362061	-947,8179932	0,045512
Cloud_Mesh_Distance_ICA_R0_R3	4,87238312	-90,08074951	-947,9778442	0,022247
Cloud_Mesh_Distance_ICA_R0_R3	3,72661591	-89,79693604	-948,0650024	-0,385456
Cloud_Mesh_Distance_ICA_R0_R3	3,65483093	-89,76321411	-947,885376	-0,352803
Cloud_Mesh_Distance_ICA_R0_R3	3,94232178	-89,55610657	-948,0320435	-0,578243
Cloud_Mesh_Distance_ICA_R0_R3	3,85360718	-89,55325317	-947,8980713	-0,529807
Cloud_Mesh_Distance_ICA_R0_R3	4,04494476	-89,48117065	-947,9994507	-0,558033
Cloud_Mesh_Distance_ICA_R0_R3	4,12481689	-89,47770691	-948,0349731	-0,492745
Cloud_Mesh_Distance_ICA_R0_R3	4,56304169	-89,54397583	-947,9361572	-0,189979
Cloud_Mesh_Distance_ICA_R0_R3	4,68190765	-89,63894653	-947,8684692	-0,108055
Cloud_Mesh_Distance_ICA_R0_R3	4,50224304	-89,5348053	-948,0013428	-0,235509
Cloud_Mesh_Distance_ICA_R0_R3	5,95399475	-92,17404175	-947,6254273	-0,04418
Cloud_Mesh_Distance_ICA_R0_R3	6,07792664	-92,16676331	-947,3421021	-0,158311
Cloud_Mesh_Distance_ICA_R0_R3	5,99737549	-92,13494873	-947,4610596	-0,032344
Cloud_Mesh_Distance_ICA_R0_R3	6,17781067	-92,318573	-947,491272	-0,228472
Cloud_Mesh_Distance_ICA_R0_R3	5,86857605	-92,03189087	-947,5233765	0,006207
Cloud_Mesh_Distance_ICA_R0_R3	5,90112305	-91,96907043	-947,2888184	0,023013
Cloud_Mesh_Distance_ICA_R0_R3	2,73678589	-91,25665283	-947,6737671	0,41243
Cloud_Mesh_Distance_ICA_R0_R3	2,9697113	-91,1184845	-947,7827148	0,165901
Cloud_Mesh_Distance_ICA_R0_R3	2,87162781	-91,09196472	-947,6612549	0,252028
Cloud_Mesh_Distance_ICA_R0_R3	5,59699249	-91,70611572	-947,5028076	0,091924
Cloud_Mesh_Distance_ICA_R0_R3	5,74614716	-91,9283905	-947,5791626	0,032474
Cloud_Mesh_Distance_ICA_R0_R3	3,05670929	-90,99502563	-947,7871094	0,127804
Cloud_Mesh_Distance_ICA_R0_R3	3,11468506	-91,08578491	-947,9002686	0,125624
Cloud_Mesh_Distance_ICA_R0_R3	3,17131805	-90,93887329	-947,8796387	0,096434
Cloud_Mesh_Distance_ICA_R0_R3	5,40184784	-91,41049194	-947,4961548	0,14705
Cloud_Mesh_Distance_ICA_R0_R3	5,44689941	-91,43782043	-947,418396	0,14563
Cloud_Mesh_Distance_ICA_R0_R3	5,49628448	-91,633255	-947,600708	0,104645
Cloud_Mesh_Distance_ICA_R0_R3	3,08581543	-90,86520386	-947,7339478	0,125323
Cloud_Mesh_Distance_ICA_R0_R3	3,03791809	-90,7855072	-947,5964356	0,127416
Cloud_Mesh_Distance_ICA_R0_R3	5,26821136	-91,23698425	-947,62323	0,145902

Cloud_Mesh_Distance_ICA_R0_R3	5,20981598	-91,1620636	-947,6912231	0,140393
Cloud_Mesh_Distance_ICA_R0_R3	5,38983154	-91,32281494	-947,4017334	0,166723
Cloud_Mesh_Distance_ICA_R0_R3	3,12226105	-90,73776245	-947,6918945	0,094866
Cloud_Mesh_Distance_ICA_R0_R3	3,18882751	-90,7709198	-947,8115234	0,081172
Cloud_Mesh_Distance_ICA_R0_R3	5,24918365	-91,13171387	-947,5726929	0,169937
Cloud_Mesh_Distance_ICA_R0_R3	5,14427948	-91,05891418	-947,7640381	0,135565
Cloud_Mesh_Distance_ICA_R0_R3	3,31775665	-90,49215698	-947,8613281	-0,020249
Cloud_Mesh_Distance_ICA_R0_R3	3,27703857	-90,423172	-947,7391357	-0,003371
Cloud_Mesh_Distance_ICA_R0_R3	5,10887909	-90,73582458	-947,6749878	0,165171
Cloud_Mesh_Distance_ICA_R0_R3	5,10773468	-90,66296387	-947,6234741	0,16945
Cloud_Mesh_Distance_ICA_R0_R3	5,13687134	-90,87413025	-947,6796875	0,162209
Cloud_Mesh_Distance_ICA_R0_R3	5,14470673	-90,74093628	-947,5499268	0,186417
Cloud_Mesh_Distance_ICA_R0_R3	3,40673828	-90,17672729	-947,8237305	-0,106302
Cloud_Mesh_Distance_ICA_R0_R3	5,03101349	-90,44223022	-947,7553711	0,133468
Cloud_Mesh_Distance_ICA_R0_R3	3,46469879	-90,03227234	-947,7998047	-0,174362
Cloud_Mesh_Distance_ICA_R0_R3	5,02172089	-90,33164978	-947,6954956	0,125293
Cloud_Mesh_Distance_ICA_R0_R3	3,79114532	-89,65377808	-947,9837036	-0,463083
Cloud_Mesh_Distance_ICA_R0_R3	3,74169922	-89,63038635	-947,7998657	-0,420039
Cloud_Mesh_Distance_ICA_R0_R3	4,84788513	-89,88656616	-947,8425903	-0,001708
Cloud_Mesh_Distance_ICA_R0_R3	4,77651215	-89,81871033	-947,9494019	-0,052622
Cloud_Mesh_Distance_ICA_R0_R3	4,86258698	-89,87704468	-947,6888428	0,033492
Cloud_Mesh_Distance_ICA_R0_R3	4,75606537	-89,71060181	-947,6479492	-0,023972
Cloud_Mesh_Distance_ICA_R0_R3	4,79784393	-89,77980042	-947,8032227	-0,027312
Cloud_Mesh_Distance_ICA_R0_R3	4,13381958	-89,4203949	-947,9232178	-0,472215
Cloud_Mesh_Distance_ICA_R0_R3	3,97505951	-89,46406555	-947,8764038	-0,596888
Cloud_Mesh_Distance_ICA_R0_R3	4,08219147	-89,39732361	-947,7667847	-0,442833
Cloud_Mesh_Distance_ICA_R0_R3	4,23638153	-89,37553406	-947,7904053	-0,332401
Cloud_Mesh_Distance_ICA_R0_R3	4,34888458	-89,40867615	-947,8825073	-0,29029
Cloud_Mesh_Distance_ICA_R0_R3	4,24243164	-89,40428162	-947,907959	-0,376296
Cloud_Mesh_Distance_ICA_R0_R3	4,47303009	-89,47311401	-947,9197388	-0,227601
Cloud_Mesh_Distance_ICA_R0_R3	4,44567871	-89,42944336	-947,8260498	-0,221341
Cloud_Mesh_Distance_ICA_R0_R3	4,54608917	-89,49453735	-947,8279419	-0,168063
Cloud_Mesh_Distance_ICA_R0_R3	4,33003235	-89,38156128	-947,7852173	-0,268474
Cloud_Mesh_Distance_ICA_R0_R3	5,78449249	-91,87306213	-947,3786621	0,048365
Cloud_Mesh_Distance_ICA_R0_R3	5,9896698	-92,01599121	-947,1151123	0,154065
Cloud_Mesh_Distance_ICA_R0_R3	5,99320984	-92,07556152	-947,3183594	-0,066323
Cloud_Mesh_Distance_ICA_R0_R3	2,85135651	-91,18215942	-947,716919	0,278583
Cloud_Mesh_Distance_ICA_R0_R3	2,79100037	-91,14865112	-947,6271362	0,342268
Cloud_Mesh_Distance_ICA_R0_R3	5,63490295	-91,67910767	-947,3527832	0,095866
Cloud_Mesh_Distance_ICA_R0_R3	5,68170929	-91,7674408	-947,4157105	0,077275
Cloud_Mesh_Distance_ICA_R0_R3	5,7137146	-91,74435425	-947,2637329	0,094187
Cloud_Mesh_Distance_ICA_R0_R3	5,85047913	-91,89784241	-947,2376099	0,049515
Cloud_Mesh_Distance_ICA_R0_R3	2,69927979	-91,15673828	-947,5355835	0,455998
Cloud_Mesh_Distance_ICA_R0_R3	5,53617096	-91,55795288	-947,3830566	0,128213
Cloud_Mesh_Distance_ICA_R0_R3	5,55437469	-91,49839783	-947,1807251	0,157491
Cloud_Mesh_Distance_ICA_R0_R3	2,8038559	-91,04702759	-947,5369263	0,338083
Cloud_Mesh_Distance_ICA_R0_R3	2,95030975	-90,95623779	-947,6313477	0,177998
Cloud_Mesh_Distance_ICA_R0_R3	2,94049072	-90,81790161	-947,4625244	0,184513
Cloud_Mesh_Distance_ICA_R0_R3	5,40602875	-91,26881409	-947,2385254	0,180593
Cloud_Mesh_Distance_ICA_R0_R3	5,306633	-91,1963501	-947,4796753	0,17621

Cloud_Mesh_Distance_ICA_R0_R3	5,25819397	-91,07106018	-947,482544	0,191908
Cloud_Mesh_Distance_ICA_R0_R3	5,20472717	-90,99481201	-947,5765381	0,183034
Cloud_Mesh_Distance_ICA_R0_R3	5,28821564	-91,06285095	-947,3554688	0,201974
Cloud_Mesh_Distance_ICA_R0_R3	3,10478973	-90,67138672	-947,600769	0,102081
Cloud_Mesh_Distance_ICA_R0_R3	3,03895569	-90,66810608	-947,4515991	0,130881
Cloud_Mesh_Distance_ICA_R0_R3	3,13130951	-90,57478333	-947,5460815	0,092681
Cloud_Mesh_Distance_ICA_R0_R3	3,19962311	-90,59281921	-947,7146606	0,044322
Cloud_Mesh_Distance_ICA_R0_R3	5,24992371	-90,92692566	-947,2807617	0,199174
Cloud_Mesh_Distance_ICA_R0_R3	5,17280579	-90,86050415	-947,5587158	0,187577
Cloud_Mesh_Distance_ICA_R0_R3	5,24172211	-90,97721863	-947,4246216	0,198413
Cloud_Mesh_Distance_ICA_R0_R3	3,20986176	-90,42941284	-947,5618286	0,040789
Cloud_Mesh_Distance_ICA_R0_R3	3,31448364	-90,28840637	-947,6937866	-0,041061
Cloud_Mesh_Distance_ICA_R0_R3	5,19909668	-90,8447876	-947,4256592	0,202808
Cloud_Mesh_Distance_ICA_R0_R3	3,28652954	-90,25764465	-947,5027466	-0,026727
Cloud_Mesh_Distance_ICA_R0_R3	5,10561371	-90,56799316	-947,5218506	0,170764
Cloud_Mesh_Distance_ICA_R0_R3	5,09231567	-90,5	-947,3041382	0,189561
Cloud_Mesh_Distance_ICA_R0_R3	5,15151978	-90,66906738	-947,3641968	0,187123
Cloud_Mesh_Distance_ICA_R0_R3	3,37372589	-90,14251709	-947,65979	-0,098423
Cloud_Mesh_Distance_ICA_R0_R3	3,53504181	-89,88815308	-947,7514038	-0,233606
Cloud_Mesh_Distance_ICA_R0_R3	3,4404068	-90,01193237	-947,6182861	-0,146279
Cloud_Mesh_Distance_ICA_R0_R3	5,05838013	-90,43302917	-947,6205444	0,147564
Cloud_Mesh_Distance_ICA_R0_R3	5,02082825	-90,27497864	-947,5604248	0,136938
Cloud_Mesh_Distance_ICA_R0_R3	4,97994232	-90,18188477	-947,7105713	0,09481
Cloud_Mesh_Distance_ICA_R0_R3	4,93049622	-90,0211792	-947,647644	0,079953
Cloud_Mesh_Distance_ICA_R0_R3	3,62448883	-89,75109863	-947,6691895	-0,306478
Cloud_Mesh_Distance_ICA_R0_R3	3,74753571	-89,62800598	-947,5541382	-0,347649
Cloud_Mesh_Distance_ICA_R0_R3	3,85301971	-89,52015686	-947,7400513	-0,49256
Cloud_Mesh_Distance_ICA_R0_R3	4,38449097	-89,4105835	-947,6226196	-0,179532
Cloud_Mesh_Distance_ICA_R0_R3	4,46193695	-89,43127441	-947,7121582	-0,172724
Cloud_Mesh_Distance_ICA_R0_R3	4,60787964	-89,54293823	-947,6776123	-0,095762
Cloud_Mesh_Distance_ICA_R0_R3	4,82337952	-89,82458496	-947,555542	0,032374
Cloud_Mesh_Distance_ICA_R0_R3	4,63748169	-89,63264465	-947,472168	-0,03377
Cloud_Mesh_Distance_ICA_R0_R3	3,96710968	-89,44241333	-947,6956177	-0,492444
Cloud_Mesh_Distance_ICA_R0_R3	4,05429077	-89,40914917	-947,6296387	-0,389543
Cloud_Mesh_Distance_ICA_R0_R3	4,15234375	-89,38098145	-947,670166	-0,342979
Cloud_Mesh_Distance_ICA_R0_R3	4,31468964	-89,37643433	-947,7346191	-0,255627
Cloud_Mesh_Distance_ICA_R0_R3	4,2716217	-89,38363647	-947,6307373	-0,247785
Cloud_Mesh_Distance_ICA_R0_R3	4,36199188	-89,38815308	-947,7067871	-0,218224
Cloud_Mesh_Distance_ICA_R0_R3	4,38343811	-89,39520264	-947,777832	-0,236242
Cloud_Mesh_Distance_ICA_R0_R3	4,34758759	-89,38278198	-947,7521362	-0,243514
Cloud_Mesh_Distance_ICA_R0_R3	5,72055054	-91,70593262	-947,1137085	0,115391
Cloud_Mesh_Distance_ICA_R0_R3	5,81214905	-91,8218689	-947,130188	0,083103
Cloud_Mesh_Distance_ICA_R0_R3	5,89527893	-91,92373657	-947,1549683	0,054287
Cloud_Mesh_Distance_ICA_R0_R3	2,65756989	-91,03062439	-947,3015747	0,522294
Cloud_Mesh_Distance_ICA_R0_R3	2,70374298	-91,06997681	-947,4334717	0,468469
Cloud_Mesh_Distance_ICA_R0_R3	5,51628113	-91,40840149	-947,0828857	0,178122
Cloud_Mesh_Distance_ICA_R0_R3	5,60254669	-91,51647949	-946,9886475	0,166634
Cloud_Mesh_Distance_ICA_R0_R3	5,47113037	-91,39526367	-947,255188	0,161474
Cloud_Mesh_Distance_ICA_R0_R3	5,67952728	-91,63356018	-947,0324097	0,136525
Cloud_Mesh_Distance_ICA_R0_R3	5,63683319	-91,61842346	-947,1884766	0,130009

Cloud_Mesh_Distance_ICA_R0_R3	2,7648468	-90,97311401	-947,3848267	0,38968
Cloud_Mesh_Distance_ICA_R0_R3	2,85820007	-90,92910767	-947,4710693	0,278653
Cloud_Mesh_Distance_ICA_R0_R3	2,84253693	-90,85644531	-947,3309326	0,284924
Cloud_Mesh_Distance_ICA_R0_R3	2,93520355	-90,7253418	-947,2861939	0,178445
Cloud_Mesh_Distance_ICA_R0_R3	3,02976227	-90,59593201	-947,2612305	0,120517
Cloud_Mesh_Distance_ICA_R0_R3	5,37549591	-91,17672729	-947,1563721	0,195437
Cloud_Mesh_Distance_ICA_R0_R3	5,34810638	-91,17706299	-947,3001709	0,188324
Cloud_Mesh_Distance_ICA_R0_R3	3,11510468	-90,52110291	-947,3968506	0,095811
Cloud_Mesh_Distance_ICA_R0_R3	3,19262695	-90,37916565	-947,3153076	0,044624
Cloud_Mesh_Distance_ICA_R0_R3	5,31270599	-91,04379272	-947,1654053	0,205814
Cloud_Mesh_Distance_ICA_R0_R3	3,36615753	-90,12062073	-947,4810791	-0,064542
Cloud_Mesh_Distance_ICA_R0_R3	3,30700684	-90,2170105	-947,276001	-0,005542
Cloud_Mesh_Distance_ICA_R0_R3	5,02078247	-90,34288025	-947,2580566	0,162758
Cloud_Mesh_Distance_ICA_R0_R3	5,03411102	-90,4480896	-947,0888672	0,175598
Cloud_Mesh_Distance_ICA_R0_R3	5,1300354	-90,61643982	-947,1618042	0,20405
Cloud_Mesh_Distance_ICA_R0_R3	3,43144226	-90,02297974	-947,4564209	-0,11306
Cloud_Mesh_Distance_ICA_R0_R3	3,51860809	-89,89796448	-947,5134277	-0,19294
Cloud_Mesh_Distance_ICA_R0_R3	3,58238983	-89,89219666	-947,2861328	-0,163122
Cloud_Mesh_Distance_ICA_R0_R3	5,05728912	-90,38650513	-947,4398193	0,167659
Cloud_Mesh_Distance_ICA_R0_R3	4,9924469	-90,22775269	-947,3812256	0,144405
Cloud_Mesh_Distance_ICA_R0_R3	3,63328552	-89,77101135	-947,4612427	-0,244776
Cloud_Mesh_Distance_ICA_R0_R3	3,70193481	-89,71353149	-947,421875	-0,276409
Cloud_Mesh_Distance_ICA_R0_R3	4,79281616	-89,86871338	-947,3607178	0,064391
Cloud_Mesh_Distance_ICA_R0_R3	4,88545227	-90,05770874	-947,2963867	0,11137
Cloud_Mesh_Distance_ICA_R0_R3	4,96192932	-90,11125183	-947,5158081	0,115688
Cloud_Mesh_Distance_ICA_R0_R3	3,89318085	-89,50201416	-947,5737915	-0,423814
Cloud_Mesh_Distance_ICA_R0_R3	3,76639557	-89,68197632	-947,3638306	-0,269719
Cloud_Mesh_Distance_ICA_R0_R3	4,87989044	-89,93771362	-947,5037842	0,068209
Cloud_Mesh_Distance_ICA_R0_R3	4,81887054	-89,8475647	-947,4727173	0,052339
Cloud_Mesh_Distance_ICA_R0_R3	3,97438812	-89,48088074	-947,4847412	-0,342059
Cloud_Mesh_Distance_ICA_R0_R3	4,34636688	-89,42897034	-947,5300903	-0,177884
Cloud_Mesh_Distance_ICA_R0_R3	4,47512817	-89,48265076	-947,5357666	-0,116448
Cloud_Mesh_Distance_ICA_R0_R3	4,36406708	-89,48605347	-947,4243164	-0,135828
Cloud_Mesh_Distance_ICA_R0_R3	4,60040283	-89,68856812	-947,3201294	-0,005513
Cloud_Mesh_Distance_ICA_R0_R3	4,69443512	-89,75370789	-947,3539429	0,016657
Cloud_Mesh_Distance_ICA_R0_R3	4,76248932	-89,76834106	-947,4743652	0,026573
Cloud_Mesh_Distance_ICA_R0_R3	3,98231506	-89,44689941	-947,5817261	-0,40474
Cloud_Mesh_Distance_ICA_R0_R3	4,03701019	-89,43588257	-947,5354004	-0,347473
Cloud_Mesh_Distance_ICA_R0_R3	4,06298065	-89,45954895	-947,4557495	-0,292846
Cloud_Mesh_Distance_ICA_R0_R3	4,13024902	-89,411026	-947,5406494	-0,293496
Cloud_Mesh_Distance_ICA_R0_R3	5,91001129	-91,91064453	-947,0054932	0,132309
Cloud_Mesh_Distance_ICA_R0_R3	5,71834564	-91,65361023	-946,8237305	0,148176
Cloud_Mesh_Distance_ICA_R0_R3	5,75198364	-91,71360779	-946,9575195	0,12141
Cloud_Mesh_Distance_ICA_R0_R3	5,83848572	-91,81726074	-946,9312744	0,116447
Cloud_Mesh_Distance_ICA_R0_R3	5,65306091	-91,56216431	-946,8104248	0,177741
Cloud_Mesh_Distance_ICA_R0_R3	5,57627106	-91,4466095	-946,7814331	0,19598
Cloud_Mesh_Distance_ICA_R0_R3	5,68147278	-91,61560059	-946,9210815	0,153027
Cloud_Mesh_Distance_ICA_R0_R3	2,73780823	-90,91436768	-947,2230835	0,414364
Cloud_Mesh_Distance_ICA_R0_R3	2,83171844	-90,79116821	-947,1491089	0,272711
Cloud_Mesh_Distance_ICA_R0_R3	5,29514313	-90,97854614	-946,96698	0,217956

Cloud_Mesh_Distance_ICA_R0_R3	5,44030762	-91,27653503	-947,0748901	0,195217
Cloud_Mesh_Distance_ICA_R0_R3	5,36699677	-91,12452698	-947,0091553	0,207376
Cloud_Mesh_Distance_ICA_R0_R3	3,1027832	-90,49279785	-947,239502	0,09098
Cloud_Mesh_Distance_ICA_R0_R3	3,23386383	-90,32601929	-947,1224365	0,048523
Cloud_Mesh_Distance_ICA_R0_R3	5,19691467	-90,77392578	-947,2244873	0,205597
Cloud_Mesh_Distance_ICA_R0_R3	5,24534607	-90,87976074	-947,095398	0,214584
Cloud_Mesh_Distance_ICA_R0_R3	5,17479706	-90,73475647	-947,0307617	0,212826
Cloud_Mesh_Distance_ICA_R0_R3	3,39203644	-90,09564209	-947,3309326	-0,068424
Cloud_Mesh_Distance_ICA_R0_R3	3,47210693	-90,00559998	-947,3070679	-0,112279
Cloud_Mesh_Distance_ICA_R0_R3	3,41918182	-90,12960815	-947,1095581	-0,04149
Cloud_Mesh_Distance_ICA_R0_R3	4,95768738	-90,2971344	-947,1203613	0,154138
Cloud_Mesh_Distance_ICA_R0_R3	3,65611267	-89,86573792	-947,2034912	-0,158188
Cloud_Mesh_Distance_ICA_R0_R3	4,86180115	-90,16874695	-947,09552	0,122576
Cloud_Mesh_Distance_ICA_R0_R3	4,95031738	-90,21673584	-947,2277222	0,143231
Cloud_Mesh_Distance_ICA_R0_R3	4,79654694	-90,04838562	-947,1327515	0,092556
Cloud_Mesh_Distance_ICA_R0_R3	3,79462433	-89,74200439	-947,2233887	-0,208177
Cloud_Mesh_Distance_ICA_R0_R3	3,68647766	-89,78349304	-947,2994995	-0,21224
Cloud_Mesh_Distance_ICA_R0_R3	3,83197784	-89,66958618	-947,2965088	-0,248918
Cloud_Mesh_Distance_ICA_R0_R3	4,54251862	-89,82325745	-947,1077271	0,010462
Cloud_Mesh_Distance_ICA_R0_R3	4,60892487	-89,91567993	-947,0633545	0,04253
Cloud_Mesh_Distance_ICA_R0_R3	4,70307159	-89,82611084	-947,2644653	0,039667
Cloud_Mesh_Distance_ICA_R0_R3	4,71179962	-90,00445557	-947,0715942	0,072816
Cloud_Mesh_Distance_ICA_R0_R3	4,7407074	-89,93103027	-947,1879883	0,065898
Cloud_Mesh_Distance_ICA_R0_R3	4,00717926	-89,51478577	-947,387146	-0,272276
Cloud_Mesh_Distance_ICA_R0_R3	4,01644897	-89,58956909	-947,2711792	-0,21018
Cloud_Mesh_Distance_ICA_R0_R3	4,12110901	-89,50395203	-947,3546753	-0,219033
Cloud_Mesh_Distance_ICA_R0_R3	3,88317871	-89,58102417	-947,3882446	-0,302716
Cloud_Mesh_Distance_ICA_R0_R3	4,63569641	-89,76306152	-947,2603149	0,019506
Cloud_Mesh_Distance_ICA_R0_R3	4,63059998	-89,82875061	-947,1768799	0,034057
Cloud_Mesh_Distance_ICA_R0_R3	4,47589874	-89,59020996	-947,3411255	-0,061767
Cloud_Mesh_Distance_ICA_R0_R3	4,53302002	-89,72920227	-947,2046509	-0,00809
Cloud_Mesh_Distance_ICA_R0_R3	4,13243866	-89,44892883	-947,4475098	-0,248508
Cloud_Mesh_Distance_ICA_R0_R3	4,23361969	-89,44389343	-947,4539185	-0,202035
Cloud_Mesh_Distance_ICA_R0_R3	5,79827118	-91,7540741	-946,7559815	0,180061
Cloud_Mesh_Distance_ICA_R0_R3	2,62358856	-90,98287964	-947,1260986	0,532748
Cloud_Mesh_Distance_ICA_R0_R3	2,72036743	-90,86791992	-947,0368042	0,380768
Cloud_Mesh_Distance_ICA_R0_R3	5,48625183	-91,30032349	-946,7034912	0,210492
Cloud_Mesh_Distance_ICA_R0_R3	2,81946564	-90,75784302	-946,9488525	0,241352
Cloud_Mesh_Distance_ICA_R0_R3	2,94963837	-90,64389038	-947,0562744	0,150288
Cloud_Mesh_Distance_ICA_R0_R3	5,50879669	-91,35923767	-946,9162598	0,19351
Cloud_Mesh_Distance_ICA_R0_R3	3,05245972	-90,53930664	-947,1289063	0,12099
Cloud_Mesh_Distance_ICA_R0_R3	2,89974976	-90,67364502	-946,8505859	0,154603
Cloud_Mesh_Distance_ICA_R0_R3	5,34493256	-91,06863403	-946,8294678	0,213084
Cloud_Mesh_Distance_ICA_R0_R3	5,39726257	-91,15670776	-946,7110596	0,212766
Cloud_Mesh_Distance_ICA_R0_R3	5,4254303	-91,21304321	-946,8720703	0,208717
Cloud_Mesh_Distance_ICA_R0_R3	5,30968475	-91,02105713	-946,6297607	0,207252
Cloud_Mesh_Distance_ICA_R0_R3	3,05082703	-90,53164673	-946,9976196	0,128612
Cloud_Mesh_Distance_ICA_R0_R3	3,11306763	-90,47192383	-946,892334	0,09931
Cloud_Mesh_Distance_ICA_R0_R3	3,23770905	-90,35971069	-946,8810425	0,064017
Cloud_Mesh_Distance_ICA_R0_R3	3,13621521	-90,43966675	-947,0845947	0,087797

Cloud_Mesh_Distance_ICA_R0_R3	5,20333099	-90,83068848	-946,8005981	0,208469
Cloud_Mesh_Distance_ICA_R0_R3	5,13489532	-90,7179718	-946,8261719	0,195873
Cloud_Mesh_Distance_ICA_R0_R3	5,22210693	-90,84246826	-946,9177856	0,21872
Cloud_Mesh_Distance_ICA_R0_R3	3,32073212	-90,24822998	-947,0431519	0,003683
Cloud_Mesh_Distance_ICA_R0_R3	5,06267548	-90,6386261	-946,7626343	0,180669
Cloud_Mesh_Distance_ICA_R0_R3	3,53872681	-90,0135498	-947,1182861	-0,102592
Cloud_Mesh_Distance_ICA_R0_R3	3,38598633	-90,20556641	-946,9832153	-0,005108
Cloud_Mesh_Distance_ICA_R0_R3	3,60134125	-90,02124023	-947,0066528	-0,089937
Cloud_Mesh_Distance_ICA_R0_R3	4,86496735	-90,43029785	-946,7284546	0,123167
Cloud_Mesh_Distance_ICA_R0_R3	4,88936615	-90,31884766	-946,9423828	0,137325
Cloud_Mesh_Distance_ICA_R0_R3	5,05893707	-90,60186768	-946,8376465	0,182718
Cloud_Mesh_Distance_ICA_R0_R3	4,99730682	-90,56417847	-946,7462769	0,164131
Cloud_Mesh_Distance_ICA_R0_R3	4,99504852	-90,49188232	-946,8847046	0,170732
Cloud_Mesh_Distance_ICA_R0_R3	5,09185028	-90,59970093	-946,9701538	0,194425
Cloud_Mesh_Distance_ICA_R0_R3	3,64523315	-89,94068909	-947,0882568	-0,125695
Cloud_Mesh_Distance_ICA_R0_R3	3,8949585	-89,81845093	-947,0493164	-0,144644
Cloud_Mesh_Distance_ICA_R0_R3	3,95923615	-89,97602844	-946,8334351	-0,093295
Cloud_Mesh_Distance_ICA_R0_R3	3,78218842	-89,93745422	-946,9638672	-0,11176
Cloud_Mesh_Distance_ICA_R0_R3	4,31745148	-89,97225952	-946,8495483	-0,032963
Cloud_Mesh_Distance_ICA_R0_R3	4,63743591	-90,04170227	-946,9571533	0,055974
Cloud_Mesh_Distance_ICA_R0_R3	4,46176147	-89,93357849	-946,9465942	0,003275
Cloud_Mesh_Distance_ICA_R0_R3	4,75628662	-90,08566284	-947,0354614	0,086488
Cloud_Mesh_Distance_ICA_R0_R3	4,75135803	-90,16601563	-946,9372559	0,094031
Cloud_Mesh_Distance_ICA_R0_R3	3,73452759	-89,84538269	-947,1347656	-0,165813
Cloud_Mesh_Distance_ICA_R0_R3	3,90733337	-89,6794281	-947,2157593	-0,205689
Cloud_Mesh_Distance_ICA_R0_R3	4,07505035	-89,87322998	-946,9310303	-0,088009
Cloud_Mesh_Distance_ICA_R0_R3	4,19052887	-89,93527222	-946,8621216	-0,054871
Cloud_Mesh_Distance_ICA_R0_R3	4,07324219	-89,71586609	-947,1018066	-0,127977
Cloud_Mesh_Distance_ICA_R0_R3	4,25558472	-89,83694458	-946,973938	-0,063172
Cloud_Mesh_Distance_ICA_R0_R3	4,50951385	-89,86416626	-947,0437622	0,003347
Cloud_Mesh_Distance_ICA_R0_R3	4,14710999	-89,60005188	-947,2226563	-0,161324
Cloud_Mesh_Distance_ICA_R0_R3	4,26183319	-89,6831665	-947,1344605	-0,08653
Cloud_Mesh_Distance_ICA_R0_R3	4,39694977	-89,66023254	-947,201355	-0,059813
Cloud_Mesh_Distance_ICA_R0_R3	4,42432404	-89,78515625	-947,078186	-0,020874
Cloud_Mesh_Distance_ICA_R0_R3	4,28457642	-89,55213928	-947,2940674	-0,124362
Cloud_Mesh_Distance_ICA_R0_R3	5,63108063	-91,51834106	-946,3746948	0,302024
Cloud_Mesh_Distance_ICA_R0_R3	5,70343018	-91,62475586	-946,6797485	0,178282
Cloud_Mesh_Distance_ICA_R0_R3	2,61084747	-90,9185791	-946,7171631	0,448342
Cloud_Mesh_Distance_ICA_R0_R3	2,60964966	-90,94232178	-946,9277344	0,490364
Cloud_Mesh_Distance_ICA_R0_R3	5,65177917	-91,54844666	-946,5119629	0,235894
Cloud_Mesh_Distance_ICA_R0_R3	5,56861877	-91,42127991	-946,413208	0,242182
Cloud_Mesh_Distance_ICA_R0_R3	5,63129425	-91,52102661	-946,675354	0,195602
Cloud_Mesh_Distance_ICA_R0_R3	5,5700531	-91,42617798	-946,6003418	0,205616
Cloud_Mesh_Distance_ICA_R0_R3	2,71356201	-90,84002686	-946,8383789	0,341223
Cloud_Mesh_Distance_ICA_R0_R3	2,81303406	-90,74804688	-946,753479	0,212046
Cloud_Mesh_Distance_ICA_R0_R3	5,47834015	-91,28227234	-946,374939	0,212853
Cloud_Mesh_Distance_ICA_R0_R3	5,50385284	-91,32275391	-946,5237427	0,216193
Cloud_Mesh_Distance_ICA_R0_R3	5,18772888	-90,83789063	-946,6629028	0,193651
Cloud_Mesh_Distance_ICA_R0_R3	5,2188797	-90,91897583	-946,4442139	0,184097
Cloud_Mesh_Distance_ICA_R0_R3	5,41397858	-91,18475342	-946,5297241	0,209404

Cloud_Mesh_Distance_ICA_R0_R3	3,01062775	-90,57061768	-946,8203735	0,123536
Cloud_Mesh_Distance_ICA_R0_R3	3,14603424	-90,47012329	-946,6693726	0,09771
Cloud_Mesh_Distance_ICA_R0_R3	5,0006485	-90,68360901	-946,4341431	0,136019
Cloud_Mesh_Distance_ICA_R0_R3	5,11495972	-90,78302002	-946,5057983	0,163511
Cloud_Mesh_Distance_ICA_R0_R3	5,26271057	-90,92823792	-946,7996826	0,211675
Cloud_Mesh_Distance_ICA_R0_R3	3,36706543	-90,25549316	-946,8704224	0,008711
Cloud_Mesh_Distance_ICA_R0_R3	4,89736176	-90,55201721	-946,5457153	0,115446
Cloud_Mesh_Distance_ICA_R0_R3	5,05190277	-90,67149353	-946,638855	0,174851
Cloud_Mesh_Distance_ICA_R0_R3	3,45115662	-90,18423462	-946,8997192	-0,02542
Cloud_Mesh_Distance_ICA_R0_R3	3,56047821	-90,10147095	-946,9088135	-0,052917
Cloud_Mesh_Distance_ICA_R0_R3	3,68408203	-90,02522278	-946,9138794	-0,099026
Cloud_Mesh_Distance_ICA_R0_R3	3,73040771	-90,11300659	-946,7304077	-0,05679
Cloud_Mesh_Distance_ICA_R0_R3	3,52042389	-90,17532349	-946,8064575	-0,024641
Cloud_Mesh_Distance_ICA_R0_R3	3,89730835	-90,10386658	-946,6687012	-0,071187
Cloud_Mesh_Distance_ICA_R0_R3	4,50783539	-90,21836853	-946,6478882	0,021093
Cloud_Mesh_Distance_ICA_R0_R3	4,54766846	-90,15020752	-946,7657471	0,026118
Cloud_Mesh_Distance_ICA_R0_R3	4,93483734	-90,5512085	-946,6282959	0,135688
Cloud_Mesh_Distance_ICA_R0_R3	4,6710968	-90,36123657	-946,5770264	0,063608
Cloud_Mesh_Distance_ICA_R0_R3	4,70012665	-90,25210571	-946,7730103	0,082472
Cloud_Mesh_Distance_ICA_R0_R3	3,82349396	-90,03561401	-946,8029785	-0,074368
Cloud_Mesh_Distance_ICA_R0_R3	4,10543823	-89,9702301	-946,8197022	-0,054581
Cloud_Mesh_Distance_ICA_R0_R3	3,9187851	-90,05151367	-946,7420044	-0,084505
Cloud_Mesh_Distance_ICA_R0_R3	4,22216034	-90,08726501	-946,689209	-0,03972
Cloud_Mesh_Distance_ICA_R0_R3	4,37785339	-90,08688354	-946,7426758	-0,009126
Cloud_Mesh_Distance_ICA_R0_R3	4,4160614	-90,01548767	-946,8399048	0,004006
Cloud_Mesh_Distance_ICA_R0_R3	4,51280212	-90,061203	-946,8441773	0,026443
Cloud_Mesh_Distance_ICA_R0_R3	4,63198853	-90,13484192	-946,850708	0,057042
Cloud_Mesh_Distance_ICA_R0_R3	4,21681213	-90,01635742	-946,7752075	-0,037211
Cloud_Mesh_Distance_ICA_R0_R3	2,74065399	-90,81837463	-946,4918213	0,325794
Cloud_Mesh_Distance_ICA_R0_R3	2,63008881	-90,90927124	-946,4840698	0,460522
Cloud_Mesh_Distance_ICA_R0_R3	5,61194611	-91,487854	-946,241394	0,277963
Cloud_Mesh_Distance_ICA_R0_R3	2,72005463	-90,82723999	-946,642273	0,321959
Cloud_Mesh_Distance_ICA_R0_R3	5,38964844	-91,15238953	-946,3273315	0,197864
Cloud_Mesh_Distance_ICA_R0_R3	5,5329895	-91,3629303	-946,2612305	0,217545
Cloud_Mesh_Distance_ICA_R0_R3	3,07460785	-90,55825806	-946,4401245	0,112225
Cloud_Mesh_Distance_ICA_R0_R3	3,10132599	-90,52224731	-946,5475464	0,109015
Cloud_Mesh_Distance_ICA_R0_R3	3,02841949	-90,57279968	-946,5962524	0,121565
Cloud_Mesh_Distance_ICA_R0_R3	2,93553162	-90,668396	-946,4196167	0,140083
Cloud_Mesh_Distance_ICA_R0_R3	2,91571045	-90,66046143	-946,6687622	0,134716
Cloud_Mesh_Distance_ICA_R0_R3	5,10462952	-90,80465698	-946,3497925	0,146655
Cloud_Mesh_Distance_ICA_R0_R3	5,32595062	-91,06002808	-946,4422607	0,196859
Cloud_Mesh_Distance_ICA_R0_R3	5,18601227	-90,90322876	-946,2417603	0,151492
Cloud_Mesh_Distance_ICA_R0_R3	3,27555847	-90,42001343	-946,467041	0,068103
Cloud_Mesh_Distance_ICA_R0_R3	3,35058594	-90,35644531	-946,553894	0,04838
Cloud_Mesh_Distance_ICA_R0_R3	4,86752319	-90,56472778	-946,4480591	0,101726
Cloud_Mesh_Distance_ICA_R0_R3	4,95256042	-90,60424805	-946,5400391	0,13631
Cloud_Mesh_Distance_ICA_R0_R3	3,27011108	-90,38182068	-946,6554565	0,056815
Cloud_Mesh_Distance_ICA_R0_R3	3,35800171	-90,31265259	-946,6973267	0,037061
Cloud_Mesh_Distance_ICA_R0_R3	3,48246002	-90,234375	-946,7127075	-0,004294
Cloud_Mesh_Distance_ICA_R0_R3	3,47200012	-90,3087616	-946,5075684	0,018552

Cloud_Mesh_Distance_ICA_R0_R3	4,62770844	-90,45843506	-946,3018189	0,024773
Cloud_Mesh_Distance_ICA_R0_R3	4,76961517	-90,53224182	-946,333252	0,070252
Cloud_Mesh_Distance_ICA_R0_R3	4,82914734	-90,48724365	-946,5620117	0,099385
Cloud_Mesh_Distance_ICA_R0_R3	3,60579681	-90,17715454	-946,706665	-0,038788
Cloud_Mesh_Distance_ICA_R0_R3	3,72375488	-90,18695068	-946,5897217	-0,041746
Cloud_Mesh_Distance_ICA_R0_R3	4,28830719	-90,17327881	-946,5910645	-0,030075
Cloud_Mesh_Distance_ICA_R0_R3	4,45227051	-90,29803467	-946,4786377	0,001181
Cloud_Mesh_Distance_ICA_R0_R3	4,7273941	-90,46557617	-946,4413452	0,070641
Cloud_Mesh_Distance_ICA_R0_R3	4,5946579	-90,39219666	-946,4281006	0,026623
Cloud_Mesh_Distance_ICA_R0_R3	4,01042938	-90,07974243	-946,6861572	-0,073367
Cloud_Mesh_Distance_ICA_R0_R3	4,10478973	-90,04139709	-946,7332764	-0,054205
Cloud_Mesh_Distance_ICA_R0_R3	4,13572693	-90,11225891	-946,6398926	-0,046761
Cloud_Mesh_Distance_ICA_R0_R3	2,58095551	-90,96440125	-946,3613892	0,54879
Cloud_Mesh_Distance_ICA_R0_R3	2,72679901	-90,84703064	-946,3547363	0,371474
Cloud_Mesh_Distance_ICA_R0_R3	5,54771423	-91,37339783	-946,0788574	0,266796
Cloud_Mesh_Distance_ICA_R0_R3	2,80934143	-90,7711792	-946,4138794	0,261962
Cloud_Mesh_Distance_ICA_R0_R3	2,82066345	-90,74630737	-946,5650635	0,22237
Cloud_Mesh_Distance_ICA_R0_R3	2,82853699	-90,78582764	-946,2349243	0,277679
Cloud_Mesh_Distance_ICA_R0_R3	5,36522675	-91,11479187	-946,1418457	0,175585
Cloud_Mesh_Distance_ICA_R0_R3	5,45975494	-91,24893188	-946,1953735	0,197224
Cloud_Mesh_Distance_ICA_R0_R3	3,16218567	-90,49113464	-946,4747315	0,094799
Cloud_Mesh_Distance_ICA_R0_R3	3,16686249	-90,51559448	-946,3241577	0,088292
Cloud_Mesh_Distance_ICA_R0_R3	5,25615692	-90,98214722	-946,0823364	0,13737
Cloud_Mesh_Distance_ICA_R0_R3	5,29093933	-91,02249146	-946,2798462	0,174214
Cloud_Mesh_Distance_ICA_R0_R3	3,22119141	-90,46972656	-946,3817139	0,077579
Cloud_Mesh_Distance_ICA_R0_R3	3,39320374	-90,36486816	-946,4359131	0,046284
Cloud_Mesh_Distance_ICA_R0_R3	3,48160553	-90,35055542	-946,3486328	0,035994
Cloud_Mesh_Distance_ICA_R0_R3	4,73593903	-90,5473175	-946,1994629	0,044136
Cloud_Mesh_Distance_ICA_R0_R3	4,8861618	-90,62985229	-946,272644	0,080486
Cloud_Mesh_Distance_ICA_R0_R3	4,83708954	-90,63009644	-946,0770874	0,044339
Cloud_Mesh_Distance_ICA_R0_R3	5,01163483	-90,72969055	-946,2766113	0,119378
Cloud_Mesh_Distance_ICA_R0_R3	3,6437912	-90,24505615	-946,5078125	-0,012271
Cloud_Mesh_Distance_ICA_R0_R3	3,62456512	-90,31895447	-946,2996216	0,014822
Cloud_Mesh_Distance_ICA_R0_R3	3,83132172	-90,28540039	-946,298523	-0,019266
Cloud_Mesh_Distance_ICA_R0_R3	3,95895386	-90,23953247	-946,402832	-0,038913
Cloud_Mesh_Distance_ICA_R0_R3	4,49554443	-90,37783813	-946,348877	0,012309
Cloud_Mesh_Distance_ICA_R0_R3	4,46195221	-90,41365051	-946,2046509	-0,006713
Cloud_Mesh_Distance_ICA_R0_R3	3,80006409	-90,22584534	-946,4674072	-0,031224
Cloud_Mesh_Distance_ICA_R0_R3	4,06804657	-90,17376709	-946,5374146	-0,052569
Cloud_Mesh_Distance_ICA_R0_R3	3,85929108	-90,17266846	-946,5596924	-0,053088
Cloud_Mesh_Distance_ICA_R0_R3	4,15597534	-90,27416992	-946,355835	-0,036303
Cloud_Mesh_Distance_ICA_R0_R3	4,26079559	-90,24783325	-946,4541626	-0,031217
Cloud_Mesh_Distance_ICA_R0_R3	4,34606171	-90,32678223	-946,3377686	-0,020811
Cloud_Mesh_Distance_ICA_R0_R3	2,6370697	-90,9302063	-946,2962647	0,495562
Cloud_Mesh_Distance_ICA_R0_R3	2,71928406	-90,8807373	-946,2123413	0,415989
Cloud_Mesh_Distance_ICA_R0_R3	5,44600677	-91,20571899	-945,9831543	0,181423
Cloud_Mesh_Distance_ICA_R0_R3	2,96446991	-90,69024658	-946,170166	0,14454
Cloud_Mesh_Distance_ICA_R0_R3	3,06728363	-90,59547424	-946,2589111	0,098557
Cloud_Mesh_Distance_ICA_R0_R3	3,20188141	-90,53752136	-946,1156006	0,084645
Cloud_Mesh_Distance_ICA_R0_R3	3,28924561	-90,45935059	-946,2493897	0,074371

Cloud_Mesh_Distance_ICA_R0_R3	5,15528107	-90,87808228	-946,1028442	0,117138
Cloud_Mesh_Distance_ICA_R0_R3	5,08545685	-90,80749512	-946,1896973	0,110661
Cloud_Mesh_Distance_ICA_R0_R3	5,34638214	-91,07618713	-945,9790649	0,141099
Cloud_Mesh_Distance_ICA_R0_R3	3,45370483	-90,42817688	-946,0690918	0,054334
Cloud_Mesh_Distance_ICA_R0_R3	3,53249359	-90,37081909	-946,1994019	0,032098
Cloud_Mesh_Distance_ICA_R0_R3	3,40097046	-90,39562988	-946,2965698	0,05182
Cloud_Mesh_Distance_ICA_R0_R3	5,07330322	-90,8052063	-946,0344849	0,077274
Cloud_Mesh_Distance_ICA_R0_R3	4,9603653	-90,72108459	-945,9276733	0,045196
Cloud_Mesh_Distance_ICA_R0_R3	4,9759903	-90,72099304	-946,1303711	0,088295
Cloud_Mesh_Distance_ICA_R0_R3	3,97471619	-90,34507751	-946,0910645	-0,024537
Cloud_Mesh_Distance_ICA_R0_R3	4,05646515	-90,30914307	-946,230835	-0,034448
Cloud_Mesh_Distance_ICA_R0_R3	4,25865936	-90,35151672	-946,2037354	-0,029954
Cloud_Mesh_Distance_ICA_R0_R3	4,60895538	-90,48898315	-946,1598511	0,014735
Cloud_Mesh_Distance_ICA_R0_R3	4,53695679	-90,48110962	-946,0460205	-0,006064
Cloud_Mesh_Distance_ICA_R0_R3	4,68610382	-90,54858398	-946,0472412	0,014844
Cloud_Mesh_Distance_ICA_R0_R3	2,64230347	-90,96801758	-946,1220093	0,538872
Cloud_Mesh_Distance_ICA_R0_R3	2,7634201	-90,88430786	-946,0604858	0,408902
Cloud_Mesh_Distance_ICA_R0_R3	2,91254425	-90,82312012	-945,8555298	0,298913
Cloud_Mesh_Distance_ICA_R0_R3	2,89476776	-90,78552246	-946,0157471	0,264495
Cloud_Mesh_Distance_ICA_R0_R3	2,98905182	-90,74694824	-945,8870239	0,199669
Cloud_Mesh_Distance_ICA_R0_R3	2,99745178	-90,7020874	-946,0153809	0,154609
Cloud_Mesh_Distance_ICA_R0_R3	5,38572693	-91,0941925	-945,8150024	0,106237
Cloud_Mesh_Distance_ICA_R0_R3	3,06713867	-90,67396545	-945,9262085	0,119109
Cloud_Mesh_Distance_ICA_R0_R3	3,07837677	-90,62960815	-946,0652466	0,100957
Cloud_Mesh_Distance_ICA_R0_R3	3,31969452	-90,51641846	-945,9292603	0,078988
Cloud_Mesh_Distance_ICA_R0_R3	5,17108154	-90,89002991	-945,961792	0,097752
Cloud_Mesh_Distance_ICA_R0_R3	5,27777863	-90,984375	-945,8650513	0,095493
Cloud_Mesh_Distance_ICA_R0_R3	5,3293457	-91,01750183	-945,758667	0,079801
Cloud_Mesh_Distance_ICA_R0_R3	3,32846069	-90,47949219	-946,0718384	0,074751
Cloud_Mesh_Distance_ICA_R0_R3	3,39072418	-90,47236633	-945,9781494	0,068586
Cloud_Mesh_Distance_ICA_R0_R3	3,47118378	-90,47546387	-945,8290405	0,065127
Cloud_Mesh_Distance_ICA_R0_R3	3,73755646	-90,37991333	-945,982666	0,014214
Cloud_Mesh_Distance_ICA_R0_R3	5,08695984	-90,81401062	-945,8928223	0,060912
Cloud_Mesh_Distance_ICA_R0_R3	3,60120392	-90,38941956	-946,048645	0,029567
Cloud_Mesh_Distance_ICA_R0_R3	3,78557587	-90,34150696	-946,1218262	-0,006736
Cloud_Mesh_Distance_ICA_R0_R3	3,65310669	-90,35882568	-946,1300659	0,013912
Cloud_Mesh_Distance_ICA_R0_R3	4,17459106	-90,37411499	-946,0604248	-0,034969
Cloud_Mesh_Distance_ICA_R0_R3	4,36723328	-90,42163086	-946,0498657	-0,021191
Cloud_Mesh_Distance_ICA_R0_R3	2,68250275	-91,01367188	-945,9023438	0,573981
Cloud_Mesh_Distance_ICA_R0_R3	2,78909302	-91,00534058	-945,7126465	0,496306
Cloud_Mesh_Distance_ICA_R0_R3	2,82002258	-90,89595032	-945,8812256	0,404472
Cloud_Mesh_Distance_ICA_R0_R3	2,88417053	-90,89094543	-945,7537231	0,36589
Cloud_Mesh_Distance_ICA_R0_R3	2,92396545	-90,93736267	-945,5864868	0,330324
Cloud_Mesh_Distance_ICA_R0_R3	3,07028198	-90,80465698	-945,5643311	0,160372
Cloud_Mesh_Distance_ICA_R0_R3	3,21340942	-90,64016724	-945,6983643	0,098612
Cloud_Mesh_Distance_ICA_R0_R3	3,09281158	-90,70617676	-945,7587891	0,130055
Cloud_Mesh_Distance_ICA_R0_R3	2,97672272	-90,81283569	-945,7345581	0,252357
Cloud_Mesh_Distance_ICA_R0_R3	3,16923523	-90,70553589	-945,602417	0,101971
Cloud_Mesh_Distance_ICA_R0_R3	3,18658447	-90,60293579	-945,8845825	0,093566
Cloud_Mesh_Distance_ICA_R0_R3	3,35945129	-90,56256104	-945,6782227	0,084826

Cloud_Mesh_Distance_ICA_R0_R3	5,17366791	-90,87632751	-945,7893677	0,049578
Cloud_Mesh_Distance_ICA_R0_R3	2,68050385	-91,10258484	-945,7352905	0,640357
Cloud_Mesh_Distance_ICA_R0_R3	3,11682892	-90,8404541	-945,4147339	0,112312
Cloud_Mesh_Distance_ICA_R0_R3	3,20924377	-90,75396729	-945,4258423	0,10076
Cloud_Mesh_Distance_MCA_R0_R1	-10,72263718	-101,1112671	-943,9700317	0,0071
Cloud_Mesh_Distance_MCA_R0_R1	-11,27487755	-102,1688843	-944,7310791	0,270376
Cloud_Mesh_Distance_MCA_R0_R1	-11,58548355	-102,2949829	-944,7932129	0,309726
Cloud_Mesh_Distance_MCA_R0_R1	-11,44116211	-102,1785889	-944,7524414	0,288292
Cloud_Mesh_Distance_MCA_R0_R1	-11,57439804	-102,0835571	-944,7064209	0,27006
Cloud_Mesh_Distance_MCA_R0_R1	-11,39210129	-102,0361328	-944,6871338	0,26199
Cloud_Mesh_Distance_MCA_R0_R1	-11,04656219	-102,1783447	-944,6759644	0,218514
Cloud_Mesh_Distance_MCA_R0_R1	-11,50387955	-101,9020996	-944,6141968	0,229927
Cloud_Mesh_Distance_MCA_R0_R1	-11,73482895	-101,9428101	-944,6022949	0,217057
Cloud_Mesh_Distance_MCA_R0_R1	-11,17650604	-101,8200073	-944,5454102	0,192553
Cloud_Mesh_Distance_MCA_R0_R1	-11,20691681	-102,0045776	-944,6484375	0,231911
Cloud_Mesh_Distance_MCA_R0_R1	-11,00873566	-101,9535523	-944,5721436	0,173515
Cloud_Mesh_Distance_MCA_R0_R1	-10,77180862	-101,7110596	-944,364502	0,04939
Cloud_Mesh_Distance_MCA_R0_R1	-10,81404114	-101,8995972	-944,4746704	0,09136
Cloud_Mesh_Distance_MCA_R0_R1	-10,97711563	-101,7513428	-944,4578247	0,13035
Cloud_Mesh_Distance_MCA_R0_R1	-11,77649498	-101,7782593	-944,4771729	0,158371
Cloud_Mesh_Distance_MCA_R0_R1	-11,3330574	-101,8939819	-944,6085205	0,227069
Cloud_Mesh_Distance_MCA_R0_R1	-11,14951324	-101,6268921	-944,4203491	0,144483
Cloud_Mesh_Distance_MCA_R0_R1	-10,64928818	-101,8655396	-944,3880615	0,009253
Cloud_Mesh_Distance_MCA_R0_R1	-10,93784714	-101,5341797	-944,3127441	0,081952
Cloud_Mesh_Distance_MCA_R0_R1	-11,89266396	-101,6934815	-944,3626709	0,116214
Cloud_Mesh_Distance_MCA_R0_R1	-11,94335938	-101,8804321	-944,4877319	0,159687
Cloud_Mesh_Distance_MCA_R0_R1	-12,1044693	-101,7877197	-944,3283081	0,082375
Cloud_Mesh_Distance_MCA_R0_R1	-11,60215187	-101,7542725	-944,5037842	0,182826
Cloud_Mesh_Distance_MCA_R0_R1	-11,37057304	-101,7199707	-944,5010376	0,18636
Cloud_Mesh_Distance_MCA_R0_R1	-11,51052856	-101,5733643	-944,3797607	0,144105
Cloud_Mesh_Distance_MCA_R0_R1	-11,02006721	-101,375	-944,2186279	0,080063
Cloud_Mesh_Distance_MCA_R0_R1	-11,1214695	-101,4677124	-944,3028565	0,109784
Cloud_Mesh_Distance_MCA_R0_R1	-10,69179726	-101,5232544	-944,2332764	-0,001018
Cloud_Mesh_Distance_MCA_R0_R1	-10,83447647	-101,3232422	-944,1436768	0,035236
Cloud_Mesh_Distance_MCA_R0_R1	-10,35458374	-101,4727173	-944,1218262	-0,114941
Cloud_Mesh_Distance_MCA_R0_R1	-12,31282997	-101,888855	-944,2659912	0,06695
Cloud_Mesh_Distance_MCA_R0_R1	-11,70302391	-101,6300049	-944,3825684	0,136842
Cloud_Mesh_Distance_MCA_R0_R1	-11,30078888	-101,5078125	-944,3430786	0,135442
Cloud_Mesh_Distance_MCA_R0_R1	-11,19137383	-101,3186646	-944,185791	0,090965
Cloud_Mesh_Distance_MCA_R0_R1	-10,91187286	-101,1708374	-944,0411987	0,040432
Cloud_Mesh_Distance_MCA_R0_R1	-10,55378151	-101,3299561	-944,0845337	-0,046558
Cloud_Mesh_Distance_MCA_R0_R1	-10,46653748	-101,0892334	-943,9262695	-0,040237
Cloud_Mesh_Distance_MCA_R0_R1	-12,25291634	-101,6817627	-944,1220703	0,020066
Cloud_Mesh_Distance_MCA_R0_R1	-12,01651001	-101,5897827	-944,2003174	0,052162
Cloud_Mesh_Distance_MCA_R0_R1	-11,62626648	-101,4821777	-944,2744751	0,106769
Cloud_Mesh_Distance_MCA_R0_R1	-11,8030777	-101,5163574	-944,2409058	0,077665
Cloud_Mesh_Distance_MCA_R0_R1	-11,4496727	-101,3712158	-944,2115479	0,100858
Cloud_Mesh_Distance_MCA_R0_R1	-11,00958061	-101,2578125	-944,1240845	0,0633
Cloud_Mesh_Distance_MCA_R0_R1	-10,58943176	-100,9157715	-943,8079224	0,005954
Cloud_Mesh_Distance_MCA_R0_R1	-10,2533226	-101,0511475	-943,9052124	-0,058145

Cloud_Mesh_Distance_MCA_R0_R1	-10,36596298	-100,880188	-943,7854004	-0,003911
Cloud_Mesh_Distance_MCA_R0_R1	-11,50212479	-101,2561646	-944,0864258	0,068915
Cloud_Mesh_Distance_MCA_R0_R1	-11,65906525	-101,3360596	-944,1188965	0,065571
Cloud_Mesh_Distance_MCA_R0_R1	-11,75715637	-101,2147217	-943,9367065	0,028571
Cloud_Mesh_Distance_MCA_R0_R1	-11,5518074	-101,1630249	-943,968811	0,047906
Cloud_Mesh_Distance_MCA_R0_R1	-11,34214401	-101,1687012	-944,0313721	0,070519
Cloud_Mesh_Distance_MCA_R0_R1	-11,08417892	-101,1287231	-944,0136108	0,067627
Cloud_Mesh_Distance_MCA_R0_R1	-11,20146561	-101,013794	-943,8893433	0,058424
Cloud_Mesh_Distance_MCA_R0_R1	-10,90813828	-101,0564575	-943,9420776	0,040657
Cloud_Mesh_Distance_MCA_R0_R1	-10,70295334	-100,7714844	-943,6777344	0,034737
Cloud_Mesh_Distance_MCA_R0_R1	-10,80881882	-100,9318848	-943,8270264	0,027885
Cloud_Mesh_Distance_MCA_R0_R1	-10,48598862	-100,7328491	-943,656189	0,026524
Cloud_Mesh_Distance_MCA_R0_R1	-10,27695084	-100,6799316	-943,637085	0,048852
Cloud_Mesh_Distance_MCA_R0_R1	-11,92684174	-101,2548828	-943,8829956	0,002154
Cloud_Mesh_Distance_MCA_R0_R1	-11,3973217	-100,986084	-943,8130493	0,051098
Cloud_Mesh_Distance_MCA_R0_R1	-11,15907288	-100,8585815	-943,7208252	0,06346
Cloud_Mesh_Distance_MCA_R0_R1	-11,01469994	-100,9607544	-943,8508911	0,0481
Cloud_Mesh_Distance_MCA_R0_R1	-10,82544899	-100,6561279	-943,541687	0,062775
Cloud_Mesh_Distance_MCA_R0_R1	-10,92497444	-100,8096314	-943,6999512	0,049082
Cloud_Mesh_Distance_MCA_R0_R1	-10,60195923	-100,609375	-943,5224609	0,064607
Cloud_Mesh_Distance_MCA_R0_R1	-11,64149475	-101,0512085	-943,793396	0,027093
Cloud_Mesh_Distance_MCA_R0_R1	-11,74858856	-100,9675293	-943,6108398	0,026181
Cloud_Mesh_Distance_MCA_R0_R1	-11,32686234	-100,8157349	-943,6176148	0,063735
Cloud_Mesh_Distance_MCA_R0_R1	-10,71866608	-100,505188	-943,3807373	0,087826
Cloud_Mesh_Distance_MCA_R0_R1	-10,31212234	-100,3982544	-943,3450928	0,346008
Cloud_Mesh_Distance_MCA_R0_R1	-10,39546585	-100,5580444	-943,5018921	0,13843
Cloud_Mesh_Distance_MCA_R0_R1	-11,536129	-100,8925171	-943,6317749	0,043829
Cloud_Mesh_Distance_MCA_R0_R1	-11,44196701	-100,7528687	-943,4726563	0,059858
Cloud_Mesh_Distance_MCA_R0_R1	-11,2207756	-100,6759033	-943,461731	0,070025
Cloud_Mesh_Distance_MCA_R0_R1	-11,19002533	-100,7612915	-943,5917969	0,064382
Cloud_Mesh_Distance_MCA_R0_R1	-11,04849243	-100,7143555	-943,5678711	0,058546
Cloud_Mesh_Distance_MCA_R0_R1	-10,81840897	-100,4153442	-943,2260742	0,113102
Cloud_Mesh_Distance_MCA_R0_R1	-10,50465393	-100,4545288	-943,3654175	0,176467
Cloud_Mesh_Distance_MCA_R0_R1	-10,41085434	-100,3137207	-943,2109375	0,335335
Cloud_Mesh_Distance_MCA_R0_R1	-11,37187958	-100,6306763	-943,3153687	0,08651
Cloud_Mesh_Distance_MCA_R0_R1	-10,97269821	-100,5593262	-943,3833008	0,080695
Cloud_Mesh_Distance_MCA_R0_R1	-10,6069603	-100,3671875	-943,2255859	0,162819
Cloud_Mesh_Distance_MCA_R0_R1	-10,50757027	-100,2440796	-943,0730591	0,299226
Cloud_Mesh_Distance_MCA_R0_R1	-10,70127487	-100,2962647	-943,0811157	0,150587
Cloud_Mesh_Distance_MCA_R0_R1	-10,32045364	-100,1898804	-943,0556641	0,481206
Cloud_Mesh_Distance_MCA_R0_R1	-10,42029572	-100,1339722	-942,9171143	0,44642
Cloud_Mesh_Distance_MCA_R0_R1	-10,60567093	-100,1915283	-942,9377441	0,283891
Cloud_Mesh_Distance_MCA_R0_R1	-10,23143387	-100,0806885	-942,899292	0,627751
Cloud_Mesh_Distance_MCA_R0_R1	-11,37085724	-102,6363525	-944,8546753	0,322032
Cloud_Mesh_Distance_MCA_R0_R1	-11,55072021	-102,6593628	-944,8701172	0,335827
Cloud_Mesh_Distance_MCA_R0_R1	-11,46309471	-102,4817505	-944,8423462	0,326893
Cloud_Mesh_Distance_MCA_R0_R1	-11,01912689	-102,611023	-944,7599487	0,242855
Cloud_Mesh_Distance_MCA_R0_R1	-10,86643219	-102,5227051	-944,6855469	0,182263
Cloud_Mesh_Distance_MCA_R0_R1	-11,23129654	-102,4951172	-944,8106079	0,293118
Cloud_Mesh_Distance_MCA_R0_R1	-11,18809509	-102,3112793	-944,7592163	0,273386

Cloud_Mesh_Distance_MCA_R0_R1	-11,3653717	-102,3196411	-944,7938843	0,303673
Cloud_Mesh_Distance_MCA_R0_R1	-11,02256393	-102,407959	-944,7305908	0,236322
Cloud_Mesh_Distance_MCA_R0_R1	-10,83012581	-102,1016235	-944,5632324	0,129246
Cloud_Mesh_Distance_MCA_R0_R1	-12,13780212	-102,6820679	-944,7507324	0,28398
Cloud_Mesh_Distance_MCA_R0_R1	-11,66914368	-102,4995728	-944,8430176	0,328141
Cloud_Mesh_Distance_MCA_R0_R1	-11,8354454	-102,5202637	-944,8220825	0,319898
Cloud_Mesh_Distance_MCA_R0_R1	-11,96427155	-102,4396362	-944,7665405	0,288254
Cloud_Mesh_Distance_MCA_R0_R1	-11,79897308	-102,3607178	-944,7868652	0,303884
Cloud_Mesh_Distance_MCA_R0_R1	-10,66719818	-102,2186279	-944,5222778	0,057955
Cloud_Mesh_Distance_MCA_R0_R1	-10,64788055	-102,0431519	-944,4581299	0,030695
Cloud_Mesh_Distance_MCA_R0_R1	-12,22584534	-102,5344849	-944,6787109	0,24706
Cloud_Mesh_Distance_MCA_R0_R1	-12,08756256	-102,5261841	-944,7434082	0,280206
Cloud_Mesh_Distance_MCA_R0_R1	-12,12719727	-102,3657837	-944,675354	0,246955
Cloud_Mesh_Distance_MCA_R0_R1	-11,76960564	-102,1655273	-944,7153931	0,268367
Cloud_Mesh_Distance_MCA_R0_R1	-10,4484005	-101,9697876	-944,3408203	-0,085203
Cloud_Mesh_Distance_MCA_R0_R1	-12,22421265	-102,2409058	-944,5662231	0,185361
Cloud_Mesh_Distance_MCA_R0_R1	-12,09194374	-102,1916504	-944,6132813	0,212283
Cloud_Mesh_Distance_MCA_R0_R1	-11,95033264	-102,0739136	-944,6115723	0,211439
Cloud_Mesh_Distance_MCA_R0_R1	-11,95441437	-102,2615356	-944,7050171	0,255661
Cloud_Mesh_Distance_MCA_R0_R1	-10,56543732	-101,7210693	-944,2906494	-0,041853
Cloud_Mesh_Distance_MCA_R0_R1	-10,49888611	-101,5524902	-944,1896973	-0,071482
Cloud_Mesh_Distance_MCA_R0_R1	-9,94391441	-101,5708618	-944,206604	-0,167693
Cloud_Mesh_Distance_MCA_R0_R1	-10,07050133	-101,421875	-944,1115723	-0,146757
Cloud_Mesh_Distance_MCA_R0_R1	-9,90634537	-101,3274536	-944,152832	-0,095384
Cloud_Mesh_Distance_MCA_R0_R1	-10,00379944	-101,1912842	-944,0412598	-0,081341
Cloud_Mesh_Distance_MCA_R0_R1	-12,38932037	-102,0598755	-944,336731	0,093213
Cloud_Mesh_Distance_MCA_R0_R1	-12,18258095	-102,0370483	-944,4703369	0,140353
Cloud_Mesh_Distance_MCA_R0_R1	-10,33156204	-101,2590332	-944,0167847	-0,088368
Cloud_Mesh_Distance_MCA_R0_R1	-12,49552536	-101,9896851	-944,1838379	0,051241
Cloud_Mesh_Distance_MCA_R0_R1	-10,15783691	-101,2244263	-944,0107422	-0,101644
Cloud_Mesh_Distance_MCA_R0_R1	-12,30831528	-101,513794	-943,8846436	-0,032902
Cloud_Mesh_Distance_MCA_R0_R1	-11,91256142	-101,3903198	-944,0522461	0,026575
Cloud_Mesh_Distance_MCA_R0_R1	-12,11877823	-101,5018311	-944,0375366	-0,001351
Cloud_Mesh_Distance_MCA_R0_R1	-12,08839417	-101,3588257	-943,8914185	-0,013876
Cloud_Mesh_Distance_MCA_R0_R1	-11,84326744	-101,1212769	-943,7655029	0,009275
Cloud_Mesh_Distance_MCA_R0_R1	-12,02648163	-101,2078857	-943,7432251	-0,014503
Cloud_Mesh_Distance_MCA_R0_R1	-12,08906174	-101,0461426	-943,4277344	0,006993
Cloud_Mesh_Distance_MCA_R0_R1	-12,12046051	-101,163269	-943,5908814	-0,009443
Cloud_Mesh_Distance_MCA_R0_R1	-11,95064545	-101,0608521	-943,5927734	-0,000342
Cloud_Mesh_Distance_MCA_R0_R1	-12,21376801	-101,0611572	-943,302063	0,024481
Cloud_Mesh_Distance_MCA_R0_R1	-11,68302727	-100,7327881	-943,2611084	0,057756
Cloud_Mesh_Distance_MCA_R0_R1	-11,53352737	-100,7071533	-943,340271	0,069284
Cloud_Mesh_Distance_MCA_R0_R1	-11,63908768	-100,8189087	-943,4551392	0,045752
Cloud_Mesh_Distance_MCA_R0_R1	-11,8666153	-100,8936768	-943,3898315	0,026171
Cloud_Mesh_Distance_MCA_R0_R1	-11,86404419	-100,7706909	-943,1390991	0,05912
Cloud_Mesh_Distance_MCA_R0_R1	-11,1739502	-100,5396118	-943,2714233	0,093731
Cloud_Mesh_Distance_MCA_R0_R1	-11,00375748	-100,4526978	-943,2089844	0,104034
Cloud_Mesh_Distance_MCA_R0_R1	-11,32327271	-100,5219116	-943,1447754	0,099638
Cloud_Mesh_Distance_MCA_R0_R1	-11,50935745	-100,6177368	-943,1869507	0,081327
Cloud_Mesh_Distance_MCA_R0_R1	-11,66407776	-100,6219482	-943,0311279	0,075772

Cloud_Mesh_Distance_MCA_R0_R1	-11,13178062	-100,4307251	-943,0974731	0,110292
Cloud_Mesh_Distance_MCA_R0_R1	-11,45496368	-100,517273	-943,0136719	0,090704
Cloud_Mesh_Distance_MCA_R0_R1	-10,91573334	-100,3394165	-943,0541992	0,13005
Cloud_Mesh_Distance_MCA_R0_R1	-11,27243805	-100,423584	-942,9716797	0,115096
Cloud_Mesh_Distance_MCA_R0_R1	-10,76713181	-100,2433472	-942,951294	0,180775
Cloud_Mesh_Distance_MCA_R0_R1	-10,90005684	-100,2437134	-942,8596802	0,165522
Cloud_Mesh_Distance_MCA_R0_R1	-10,72206879	-100,1635132	-942,8041992	0,302699
Cloud_Mesh_Distance_MCA_R0_R1	-11,0839119	-100,3324585	-942,9216309	0,134545
Cloud_Mesh_Distance_MCA_R0_R1	-11,04782867	-100,2495728	-942,7350464	0,16707
Cloud_Mesh_Distance_MCA_R0_R1	-11,22571564	-100,3399048	-942,7945557	0,143034
Cloud_Mesh_Distance_MCA_R0_R1	-10,53359222	-100,0968628	-942,7764893	0,457039
Cloud_Mesh_Distance_MCA_R0_R1	-11,41183662	-102,828125	-944,8653565	0,315983
Cloud_Mesh_Distance_MCA_R0_R1	-11,36477661	-103,0071411	-944,8458252	0,29662
Cloud_Mesh_Distance_MCA_R0_R1	-11,20266533	-102,7192383	-944,8255615	0,292054
Cloud_Mesh_Distance_MCA_R0_R1	-9,76109314	-102,0853272	-944,3584595	-0,23326
Cloud_Mesh_Distance_MCA_R0_R1	-9,65048409	-102,2789307	-944,4039307	-0,233209
Cloud_Mesh_Distance_MCA_R0_R1	-11,97854233	-102,8442383	-944,8171387	0,310373
Cloud_Mesh_Distance_MCA_R0_R1	-11,77017212	-102,6930542	-944,857544	0,335887
Cloud_Mesh_Distance_MCA_R0_R1	-11,61667061	-102,8214111	-944,8756104	0,334243
Cloud_Mesh_Distance_MCA_R0_R1	-11,7620163	-102,8914795	-944,8618164	0,326724
Cloud_Mesh_Distance_MCA_R0_R1	-10,82179451	-102,7069702	-944,6795654	0,178667
Cloud_Mesh_Distance_MCA_R0_R1	-10,80843544	-102,3383179	-944,623291	0,143826
Cloud_Mesh_Distance_MCA_R0_R1	-11,96552658	-102,616333	-944,8049316	0,302293
Cloud_Mesh_Distance_MCA_R0_R1	-12,41765404	-102,7457886	-944,5969849	0,228004
Cloud_Mesh_Distance_MCA_R0_R1	-12,28777695	-102,684082	-944,673645	0,253068
Cloud_Mesh_Distance_MCA_R0_R1	-9,83972168	-101,7201538	-944,302124	-0,165394
Cloud_Mesh_Distance_MCA_R0_R1	-12,37787628	-102,5636597	-944,5914917	0,221072
Cloud_Mesh_Distance_MCA_R0_R1	-10,34274673	-101,7264404	-944,2214356	-0,136684
Cloud_Mesh_Distance_MCA_R0_R1	-10,21492004	-101,9868774	-944,2683106	-0,190822
Cloud_Mesh_Distance_MCA_R0_R1	-12,30435944	-102,3929443	-944,5849609	0,207238
Cloud_Mesh_Distance_MCA_R0_R1	-10,19721222	-101,5792847	-944,1497192	-0,16803
Cloud_Mesh_Distance_MCA_R0_R1	-10,06291389	-101,642334	-944,1816406	-0,193672
Cloud_Mesh_Distance_MCA_R0_R1	-12,38531113	-102,2400513	-944,4539795	0,143184
Cloud_Mesh_Distance_MCA_R0_R1	-12,44131851	-102,4320068	-944,5011597	0,178479
Cloud_Mesh_Distance_MCA_R0_R1	-10,22058487	-101,3927002	-944,078186	-0,133898
Cloud_Mesh_Distance_MCA_R0_R1	-12,55391312	-102,3776245	-944,3773193	0,135384
Cloud_Mesh_Distance_MCA_R0_R1	-12,53974915	-102,1802368	-944,2807617	0,091027
Cloud_Mesh_Distance_MCA_R0_R1	-12,42173004	-101,8278809	-944,1135254	0,01982
Cloud_Mesh_Distance_MCA_R0_R1	-12,57255554	-101,8925781	-944,0149536	0,01434
Cloud_Mesh_Distance_MCA_R0_R1	-12,60580635	-101,7907105	-943,8717041	0,00464
Cloud_Mesh_Distance_MCA_R0_R1	-12,45592499	-101,7171021	-943,9672852	-0,010583
Cloud_Mesh_Distance_MCA_R0_R1	-12,53974533	-101,6361694	-943,7774658	-0,018815
Cloud_Mesh_Distance_MCA_R0_R1	-12,62050629	-101,3797607	-943,2890015	0,067335
Cloud_Mesh_Distance_MCA_R0_R1	-12,67077827	-101,6018677	-943,5536499	0,027431
Cloud_Mesh_Distance_MCA_R0_R1	-12,21942902	-101,3112183	-943,7075195	-0,028877
Cloud_Mesh_Distance_MCA_R0_R1	-12,30882645	-101,2176514	-943,4621582	0,004962
Cloud_Mesh_Distance_MCA_R0_R1	-12,43823624	-101,4390869	-943,6426392	-0,023467
Cloud_Mesh_Distance_MCA_R0_R1	-12,53980637	-101,3934937	-943,4387207	0,025464
Cloud_Mesh_Distance_MCA_R0_R1	-12,48085976	-101,2544556	-943,2852783	0,04381
Cloud_Mesh_Distance_MCA_R0_R1	-12,34080124	-101,1331177	-943,2632446	0,030144

Cloud_Mesh_Distance_MCA_R0_R1	-12,06900406	-100,9196167	-943,2064819	0,033489
Cloud_Mesh_Distance_MCA_R0_R1	-12,16801071	-100,9035645	-942,9979248	0,072118
Cloud_Mesh_Distance_MCA_R0_R1	-12,00883293	-100,8115234	-943,026001	0,058508
Cloud_Mesh_Distance_MCA_R0_R1	-12,06646729	-100,8029175	-942,8824463	0,07572
Cloud_Mesh_Distance_MCA_R0_R1	-11,68492508	-100,5621338	-942,8094482	0,099123
Cloud_Mesh_Distance_MCA_R0_R1	-11,54571915	-100,513855	-942,8884888	0,104909
Cloud_Mesh_Distance_MCA_R0_R1	-11,39742661	-100,4313355	-942,847229	0,123504
Cloud_Mesh_Distance_MCA_R0_R1	-11,35797119	-100,3652344	-942,6729736	0,138177
Cloud_Mesh_Distance_MCA_R0_R1	-11,88123322	-100,6957398	-942,8988648	0,074196
Cloud_Mesh_Distance_MCA_R0_R1	-12,03365326	-100,7476196	-942,7111206	0,087699
Cloud_Mesh_Distance_MCA_R0_R1	-11,83651733	-100,6189575	-942,6740112	0,09406
Cloud_Mesh_Distance_MCA_R0_R1	-11,52123833	-100,4586792	-942,7335815	0,115294
Cloud_Mesh_Distance_MCA_R0_R1	-11,65443039	-100,5064697	-942,6167603	0,106495
Cloud_Mesh_Distance_MCA_R0_R1	-11,48855972	-100,4076538	-942,5581055	0,125596
Cloud_Mesh_Distance_MCA_R0_R1	-11,33131027	-100,3172607	-942,4875488	0,145906
Cloud_Mesh_Distance_MCA_R0_R1	-11,19033432	-100,2738037	-942,6089478	0,156935
Cloud_Mesh_Distance_MCA_R0_R1	-10,86701584	-100,1626587	-942,671875	0,2902
Cloud_Mesh_Distance_MCA_R0_R1	-11,01858139	-100,1853027	-942,5423584	0,257248
Cloud_Mesh_Distance_MCA_R0_R1	-11,85226059	-103,0568237	-944,8301392	0,301625
Cloud_Mesh_Distance_MCA_R0_R1	-11,58639526	-103,0341187	-944,8586426	0,309714
Cloud_Mesh_Distance_MCA_R0_R1	-11,39340973	-103,1651001	-944,8243408	0,276826
Cloud_Mesh_Distance_MCA_R0_R1	-11,20548248	-102,9232178	-944,8220215	0,279953
Cloud_Mesh_Distance_MCA_R0_R1	-11,24078751	-103,0819702	-944,8120117	0,270785
Cloud_Mesh_Distance_MCA_R0_R1	-11,00452805	-102,8301392	-944,7609863	0,235732
Cloud_Mesh_Distance_MCA_R0_R1	-11,03956604	-103,0773926	-944,7529297	0,233489
Cloud_Mesh_Distance_MCA_R0_R1	-10,61403847	-102,7772217	-944,5680542	0,090977
Cloud_Mesh_Distance_MCA_R0_R1	-10,6878624	-102,5580444	-944,5985718	0,103267
Cloud_Mesh_Distance_MCA_R0_R1	-10,47892189	-102,6159058	-944,486084	0,016106
Cloud_Mesh_Distance_MCA_R0_R1	-9,62378311	-102,4613648	-944,3352051	-0,269034
Cloud_Mesh_Distance_MCA_R0_R1	-12,07521439	-103,067749	-944,7676392	0,279353
Cloud_Mesh_Distance_MCA_R0_R1	-10,5770359	-102,397583	-944,5147705	0,036442
Cloud_Mesh_Distance_MCA_R0_R1	-10,48778534	-102,2182617	-944,4312744	-0,042487
Cloud_Mesh_Distance_MCA_R0_R1	-9,83726501	-101,914978	-944,3122559	-0,221318
Cloud_Mesh_Distance_MCA_R0_R1	-12,38010216	-103,0665894	-944,6124268	0,225679
Cloud_Mesh_Distance_MCA_R0_R1	-12,25234222	-102,8902588	-944,7050781	0,259593
Cloud_Mesh_Distance_MCA_R0_R1	-9,88237381	-102,0646362	-944,2943115	-0,259626
Cloud_Mesh_Distance_MCA_R0_R1	-10,03521347	-102,0137329	-944,2593994	-0,244665
Cloud_Mesh_Distance_MCA_R0_R1	-9,96118164	-101,8208008	-944,2466431	-0,221525
Cloud_Mesh_Distance_MCA_R0_R1	-12,43539047	-102,9113159	-944,590332	0,225498
Cloud_Mesh_Distance_MCA_R0_R1	-12,67687607	-102,9419556	-944,3705444	0,157541
Cloud_Mesh_Distance_MCA_R0_R1	-10,13471603	-101,7982788	-944,2142944	-0,204671
Cloud_Mesh_Distance_MCA_R0_R1	-12,69297028	-102,7293091	-944,3436279	0,149877
Cloud_Mesh_Distance_MCA_R0_R1	-12,55973816	-102,8256226	-944,4884033	0,191558
Cloud_Mesh_Distance_MCA_R0_R1	-12,80519295	-102,8552856	-944,2159424	0,122612
Cloud_Mesh_Distance_MCA_R0_R1	-12,54484558	-102,5971069	-944,4660645	0,178467
Cloud_Mesh_Distance_MCA_R0_R1	-12,81287003	-102,6442261	-944,1772461	0,112264
Cloud_Mesh_Distance_MCA_R0_R1	-12,66669464	-102,3189087	-944,2283325	0,100422
Cloud_Mesh_Distance_MCA_R0_R1	-12,69044495	-102,5114746	-944,2892456	0,13213
Cloud_Mesh_Distance_MCA_R0_R1	-12,69865227	-102,1305542	-944,06427	0,055082
Cloud_Mesh_Distance_MCA_R0_R1	-12,85528564	-102,2625732	-943,9398804	0,073941

Cloud_Mesh_Distance_MCA_R0_R1	-12,80818939	-102,409729	-944,0942993	0,093907
Cloud_Mesh_Distance_MCA_R0_R1	-12,71769333	-101,9378052	-943,8742676	0,032931
Cloud_Mesh_Distance_MCA_R0_R1	-12,71211433	-101,7883301	-943,7246094	0,017395
Cloud_Mesh_Distance_MCA_R0_R1	-12,84607697	-101,6619263	-943,3463745	0,094791
Cloud_Mesh_Distance_MCA_R0_R1	-12,69099426	-101,4984741	-943,3720093	0,06036
Cloud_Mesh_Distance_MCA_R0_R1	-12,75286865	-101,4732056	-943,2150269	0,100407
Cloud_Mesh_Distance_MCA_R0_R1	-12,78282356	-101,4259033	-943,0479736	0,137645
Cloud_Mesh_Distance_MCA_R0_R1	-12,62425041	-101,2996826	-943,1168213	0,092143
Cloud_Mesh_Distance_MCA_R0_R1	-12,71937943	-101,3129273	-942,9061279	0,146412
Cloud_Mesh_Distance_MCA_R0_R1	-12,43719101	-101,1407471	-943,1165772	0,065381
Cloud_Mesh_Distance_MCA_R0_R1	-12,26391029	-101,0219116	-943,1418457	0,05357
Cloud_Mesh_Distance_MCA_R0_R1	-12,35600281	-101,0211182	-942,9525147	0,084087
Cloud_Mesh_Distance_MCA_R0_R1	-12,46104622	-101,0552979	-942,7461548	0,119861
Cloud_Mesh_Distance_MCA_R0_R1	-12,24120331	-100,8954468	-942,7719116	0,092457
Cloud_Mesh_Distance_MCA_R0_R1	-11,97297668	-100,687561	-942,5089111	0,101776
Cloud_Mesh_Distance_MCA_R0_R1	-12,19168091	-100,8359375	-942,5125122	0,103688
Cloud_Mesh_Distance_MCA_R0_R1	-11,90450287	-100,6436157	-942,2962647	0,097572
Cloud_Mesh_Distance_MCA_R0_R1	-12,07295609	-100,7553101	-942,3546753	0,097643
Cloud_Mesh_Distance_MCA_R0_R1	-11,78503036	-100,5670776	-942,479126	0,106235
Cloud_Mesh_Distance_MCA_R0_R1	-11,72480392	-100,5270386	-942,3212891	0,109183
Cloud_Mesh_Distance_MCA_R0_R1	-11,61214447	-100,4631348	-942,4366455	0,118139
Cloud_Mesh_Distance_MCA_R0_R1	-11,46336365	-100,3768311	-942,3988037	0,137254
Cloud_Mesh_Distance_MCA_R0_R1	-11,33169556	-100,2973633	-942,2733765	0,156915
Cloud_Mesh_Distance_MCA_R0_R1	-11,16523743	-100,2266235	-942,4143677	0,210426
Cloud_Mesh_Distance_MCA_R0_R1	-11,55163193	-103,2467041	-944,8168945	0,277834
Cloud_Mesh_Distance_MCA_R0_R1	-11,40833664	-103,3719482	-944,7730103	0,250611
Cloud_Mesh_Distance_MCA_R0_R1	-11,62720108	-103,413269	-944,7649536	0,244395
Cloud_Mesh_Distance_MCA_R0_R1	-11,51148605	-103,5397339	-944,7194214	0,224595
Cloud_Mesh_Distance_MCA_R0_R1	-11,20665359	-103,427002	-944,7228394	0,223014
Cloud_Mesh_Distance_MCA_R0_R1	-11,21755409	-103,2404175	-944,7758179	0,250442
Cloud_Mesh_Distance_MCA_R0_R1	-12,14520454	-103,2456055	-944,6991577	0,233996
Cloud_Mesh_Distance_MCA_R0_R1	-12,21510506	-103,3776856	-944,6184692	0,193897
Cloud_Mesh_Distance_MCA_R0_R1	-12,05461502	-103,4143677	-944,6749878	0,208939
Cloud_Mesh_Distance_MCA_R0_R1	-11,95542908	-103,2463989	-944,7657471	0,256175
Cloud_Mesh_Distance_MCA_R0_R1	-11,73818588	-103,2415772	-944,8087769	0,275495
Cloud_Mesh_Distance_MCA_R0_R1	-10,78318405	-102,9581909	-944,6528931	0,165656
Cloud_Mesh_Distance_MCA_R0_R1	-10,83864212	-103,2021484	-944,6462402	0,181252
Cloud_Mesh_Distance_MCA_R0_R1	-10,6661377	-103,1373291	-944,5664673	0,131753
Cloud_Mesh_Distance_MCA_R0_R1	-9,68117714	-102,5809326	-944,2556152	-0,26164
Cloud_Mesh_Distance_MCA_R0_R1	-9,4464016	-102,6346436	-944,315979	-0,22732
Cloud_Mesh_Distance_MCA_R0_R1	-12,30904198	-103,2437134	-944,6148071	0,210798
Cloud_Mesh_Distance_MCA_R0_R1	-12,23681641	-103,1060181	-944,6901856	0,246816
Cloud_Mesh_Distance_MCA_R0_R1	-9,86224556	-102,4832153	-944,2643433	-0,267662
Cloud_Mesh_Distance_MCA_R0_R1	-10,26675224	-102,6784058	-944,37146	-0,091342
Cloud_Mesh_Distance_MCA_R0_R1	-10,36293411	-102,4442139	-944,4085083	-0,084996
Cloud_Mesh_Distance_MCA_R0_R1	-10,09252739	-102,1706543	-944,2798462	-0,233857
Cloud_Mesh_Distance_MCA_R0_R1	-10,25172806	-102,2388306	-944,3302002	-0,168873
Cloud_Mesh_Distance_MCA_R0_R1	-9,94247055	-102,2376099	-944,2802734	-0,272189
Cloud_Mesh_Distance_MCA_R0_R1	-9,7699852	-102,3137817	-944,3143311	-0,269378
Cloud_Mesh_Distance_MCA_R0_R1	-12,63106346	-103,1472778	-944,3833618	0,151626

Cloud_Mesh_Distance_MCA_R0_R1	-12,77428627	-103,0698853	-944,2341919	0,113424
Cloud_Mesh_Distance_MCA_R0_R1	-12,53416824	-103,0192871	-944,5029297	0,193054
Cloud_Mesh_Distance_MCA_R0_R1	-12,89872169	-102,7699585	-944,072937	0,094816
Cloud_Mesh_Distance_MCA_R0_R1	-12,9352169	-102,421814	-943,9043579	0,0832
Cloud_Mesh_Distance_MCA_R0_R1	-12,91910172	-102,5975952	-944,0040283	0,092962
Cloud_Mesh_Distance_MCA_R0_R1	-12,84319115	-102,1008301	-943,8371582	0,057394
Cloud_Mesh_Distance_MCA_R0_R1	-12,95874977	-102,0829468	-943,6257935	0,087082
Cloud_Mesh_Distance_MCA_R0_R1	-12,95184326	-102,262146	-943,78125	0,081897
Cloud_Mesh_Distance_MCA_R0_R1	-12,86221504	-101,835083	-943,5419922	0,07235
Cloud_Mesh_Distance_MCA_R0_R1	-13,00715637	-101,9675293	-943,4187012	0,116968
Cloud_Mesh_Distance_MCA_R0_R1	-12,93962479	-101,822876	-943,3869019	0,107834
Cloud_Mesh_Distance_MCA_R0_R1	-12,82693481	-101,9465942	-943,7210693	0,053327
Cloud_Mesh_Distance_MCA_R0_R1	-13,01716423	-101,8349609	-943,234375	0,156619
Cloud_Mesh_Distance_MCA_R0_R1	-12,92032433	-101,6060181	-943,0907593	0,157615
Cloud_Mesh_Distance_MCA_R0_R1	-12,87402344	-101,4714966	-942,8896484	0,182413
Cloud_Mesh_Distance_MCA_R0_R1	-12,54584312	-101,1637573	-942,9285889	0,115663
Cloud_Mesh_Distance_MCA_R0_R1	-12,64608383	-101,208252	-942,7211914	0,153637
Cloud_Mesh_Distance_MCA_R0_R1	-12,37481689	-100,9729004	-942,5835571	0,120704
Cloud_Mesh_Distance_MCA_R0_R1	-12,05511856	-100,7741089	-942,1053467	0,147582
Cloud_Mesh_Distance_MCA_R0_R1	-11,56247139	-100,4295654	-942,2371826	0,126254
Cloud_Mesh_Distance_MCA_R0_R1	-11,75740623	-100,5585327	-942,1412354	0,101688
Cloud_Mesh_Distance_MCA_R0_R1	-11,69578934	-103,6286621	-944,6674194	0,198164
Cloud_Mesh_Distance_MCA_R0_R1	-11,34378052	-103,5575562	-944,7009277	0,218315
Cloud_Mesh_Distance_MCA_R0_R1	-11,46808624	-103,7156982	-944,6397705	0,191384
Cloud_Mesh_Distance_MCA_R0_R1	-11,1906662	-103,5734863	-944,6674805	0,20784
Cloud_Mesh_Distance_MCA_R0_R1	-11,03229523	-103,3112183	-944,7037354	0,215506
Cloud_Mesh_Distance_MCA_R0_R1	-10,67494583	-103,3244019	-944,5324097	0,148688
Cloud_Mesh_Distance_MCA_R0_R1	-12,17526436	-103,5323486	-944,5639648	0,15707
Cloud_Mesh_Distance_MCA_R0_R1	-11,84314728	-103,4338989	-944,7287598	0,226097
Cloud_Mesh_Distance_MCA_R0_R1	-10,54862595	-102,9974976	-944,5169678	0,080972
Cloud_Mesh_Distance_MCA_R0_R1	-10,4017067	-102,8421021	-944,4415894	0,006113
Cloud_Mesh_Distance_MCA_R0_R1	-10,3515625	-103,0679321	-944,3842163	0,044045
Cloud_Mesh_Distance_MCA_R0_R1	-10,05773926	-102,7324829	-944,2705689	-0,146826
Cloud_Mesh_Distance_MCA_R0_R1	-9,99307632	-102,9028931	-944,2105103	-0,08032
Cloud_Mesh_Distance_MCA_R0_R1	-9,8066864	-102,791687	-944,177124	-0,143364
Cloud_Mesh_Distance_MCA_R0_R1	-12,27377319	-103,6086426	-944,4613037	0,113178
Cloud_Mesh_Distance_MCA_R0_R1	-9,97683334	-102,581604	-944,2626953	-0,225865
Cloud_Mesh_Distance_MCA_R0_R1	-12,55905914	-103,3440552	-944,3811646	0,127035
Cloud_Mesh_Distance_MCA_R0_R1	-12,37010574	-103,4342041	-944,4949951	0,14554
Cloud_Mesh_Distance_MCA_R0_R1	-10,1525116	-102,510376	-944,3195801	-0,175045
Cloud_Mesh_Distance_MCA_R0_R1	-10,04030228	-102,3717651	-944,2848511	-0,240259
Cloud_Mesh_Distance_MCA_R0_R1	-12,47270393	-103,2127075	-944,5093994	0,174077
Cloud_Mesh_Distance_MCA_R0_R1	-12,95144272	-103,1593628	-943,9285278	0,032995
Cloud_Mesh_Distance_MCA_R0_R1	-12,91017914	-102,9766235	-944,0506592	0,077043
Cloud_Mesh_Distance_MCA_R0_R1	-12,84326935	-103,2092285	-944,0910034	0,066161
Cloud_Mesh_Distance_MCA_R0_R1	-13,01125336	-102,9918823	-943,8637695	0,04862
Cloud_Mesh_Distance_MCA_R0_R1	-13,00178146	-102,7780762	-943,8928223	0,07358
Cloud_Mesh_Distance_MCA_R0_R1	-13,00187302	-102,5723877	-943,8499756	0,082217
Cloud_Mesh_Distance_MCA_R0_R1	-13,02997589	-102,4353027	-943,7384644	0,09202
Cloud_Mesh_Distance_MCA_R0_R1	-13,04056549	-102,28479	-943,6286621	0,099143

Cloud_Mesh_Distance_MCA_R0_R1	-13,11861801	-102,4469605	-943,5529785	0,094891
Cloud_Mesh_Distance_MCA_R0_R1	-13,10394859	-102,208374	-943,4331665	0,121738
Cloud_Mesh_Distance_MCA_R0_R1	-13,20574951	-102,2201538	-943,1719971	0,164988
Cloud_Mesh_Distance_MCA_R0_R1	-13,105896	-102,039856	-943,2731323	0,161731
Cloud_Mesh_Distance_MCA_R0_R1	-13,08005905	-101,7979126	-942,9976807	0,205262
Cloud_Mesh_Distance_MCA_R0_R1	-13,14743423	-101,9890747	-943,09375	0,197004
Cloud_Mesh_Distance_MCA_R0_R1	-13,13518333	-101,8168335	-942,7764893	0,249238
Cloud_Mesh_Distance_MCA_R0_R1	-13,01436234	-101,6434326	-942,864502	0,221781
Cloud_Mesh_Distance_MCA_R0_R1	-13,07279396	-101,6964722	-942,6817627	0,252561
Cloud_Mesh_Distance_MCA_R0_R1	-12,95597458	-101,5343628	-942,6940308	0,226634
Cloud_Mesh_Distance_MCA_R0_R1	-12,88178253	-101,4434204	-942,5183716	0,215789
Cloud_Mesh_Distance_MCA_R0_R1	-12,80957031	-101,3657227	-942,7103272	0,188035
Cloud_Mesh_Distance_MCA_R0_R1	-12,68675995	-101,2628784	-942,3251343	0,164681
Cloud_Mesh_Distance_MCA_R0_R1	-12,55656052	-101,121521	-942,5030518	0,145853
Cloud_Mesh_Distance_MCA_R0_R1	-12,38266945	-100,9871216	-942,371521	0,123686
Cloud_Mesh_Distance_MCA_R0_R1	-12,17706299	-100,892273	-942,0153809	0,142362
Cloud_Mesh_Distance_MCA_R0_R1	-12,21571732	-100,8706055	-942,2595825	0,097814
Cloud_Mesh_Distance_MCA_R0_R1	-11,93139076	-100,6900024	-942,0710449	0,135427
Cloud_Mesh_Distance_MCA_R0_R1	-11,33960533	-103,8354492	-944,5690308	0,181455
Cloud_Mesh_Distance_MCA_R0_R1	-11,26454544	-103,704773	-944,6261597	0,193141
Cloud_Mesh_Distance_MCA_R0_R1	-10,90039063	-103,6054077	-944,5579834	0,182294
Cloud_Mesh_Distance_MCA_R0_R1	-10,95093536	-103,7793579	-944,5007324	0,183499
Cloud_Mesh_Distance_MCA_R0_R1	-11,08359337	-103,6730957	-944,5982666	0,192762
Cloud_Mesh_Distance_MCA_R0_R1	-9,21054649	-102,8678589	-944,0026856	-0,115665
Cloud_Mesh_Distance_MCA_R0_R1	-9,33519554	-102,7655029	-944,2019043	-0,19739
Cloud_Mesh_Distance_MCA_R0_R1	-11,04566383	-103,50177	-944,6519775	0,201635
Cloud_Mesh_Distance_MCA_R0_R1	-10,86359215	-103,4138794	-944,605957	0,180539
Cloud_Mesh_Distance_MCA_R0_R1	-10,70853806	-103,5321045	-944,4863281	0,164124
Cloud_Mesh_Distance_MCA_R0_R1	-10,19148827	-103,1318359	-944,263855	0,043596
Cloud_Mesh_Distance_MCA_R0_R1	-10,31366348	-103,3023682	-944,2966919	0,095981
Cloud_Mesh_Distance_MCA_R0_R1	-10,50318909	-103,2140503	-944,4532471	0,100009
Cloud_Mesh_Distance_MCA_R0_R1	-10,09925652	-103,2885132	-944,1334839	0,083291
Cloud_Mesh_Distance_MCA_R0_R1	-9,89982033	-102,9983521	-944,1296387	-0,031798
Cloud_Mesh_Distance_MCA_R0_R1	-9,39989662	-102,8673706	-944,0588989	-0,107891
Cloud_Mesh_Distance_MCA_R0_R1	-9,05217934	-102,8657837	-943,8477783	-0,083211
Cloud_Mesh_Distance_MCA_R0_R1	-12,14156723	-103,7029419	-944,477478	0,112158
Cloud_Mesh_Distance_MCA_R0_R1	-11,9642067	-103,6290894	-944,6019287	0,16628
Cloud_Mesh_Distance_MCA_R0_R1	-10,18096161	-102,9191895	-944,3051758	-0,041888
Cloud_Mesh_Distance_MCA_R0_R1	-10,04143524	-103,0696411	-944,1854248	0,003933
Cloud_Mesh_Distance_MCA_R0_R1	-9,54480934	-102,7709351	-944,1625977	-0,166874
Cloud_Mesh_Distance_MCA_R0_R1	-9,55918884	-102,960022	-944,0036621	-0,039254
Cloud_Mesh_Distance_MCA_R0_R1	-12,27079391	-103,7372437	-944,37146	0,06756
Cloud_Mesh_Distance_MCA_R0_R1	-12,49640274	-103,5014038	-944,354126	0,090332
Cloud_Mesh_Distance_MCA_R0_R1	-12,39051437	-103,6146851	-944,3705444	0,079693
Cloud_Mesh_Distance_MCA_R0_R1	-12,70966721	-103,28125	-944,2442017	0,092162
Cloud_Mesh_Distance_MCA_R0_R1	-12,88551712	-103,322937	-943,9634399	0,019241
Cloud_Mesh_Distance_MCA_R0_R1	-13,04066467	-103,1360474	-943,7565308	0,009606
Cloud_Mesh_Distance_MCA_R0_R1	-13,18428993	-102,6912842	-943,4667969	0,069512
Cloud_Mesh_Distance_MCA_R0_R1	-13,08509445	-102,6359253	-943,699707	0,081234
Cloud_Mesh_Distance_MCA_R0_R1	-13,32299805	-102,4608765	-942,8987427	0,174209

Cloud_Mesh_Distance_MCA_R0_R1	-13,28101349	-102,2921143	-942,956604	0,193977
Cloud_Mesh_Distance_MCA_R0_R1	-13,22685242	-102,1174927	-942,9840088	0,20816
Cloud_Mesh_Distance_MCA_R0_R1	-13,2682724	-102,1491699	-942,8065796	0,226995
Cloud_Mesh_Distance_MCA_R0_R1	-13,18952942	-101,9658203	-942,8841553	0,228121
Cloud_Mesh_Distance_MCA_R0_R1	-13,22659683	-101,9974365	-942,6663208	0,253424
Cloud_Mesh_Distance_MCA_R0_R1	-13,15082359	-101,8339233	-942,5527344	0,263432
Cloud_Mesh_Distance_MCA_R0_R1	-13,02703094	-101,6386108	-942,4779053	0,24593
Cloud_Mesh_Distance_MCA_R0_R1	-12,73812485	-101,2880249	-942,5247803	0,181321
Cloud_Mesh_Distance_MCA_R0_R1	-12,80735397	-101,3783569	-942,3760376	0,195241
Cloud_Mesh_Distance_MCA_R0_R1	-12,79714394	-101,4187012	-942,1929932	0,176909
Cloud_Mesh_Distance_MCA_R0_R1	-12,53390503	-101,1360474	-942,2485352	0,128112
Cloud_Mesh_Distance_MCA_R0_R1	-12,51814651	-101,1760254	-942,0653687	0,087249
Cloud_Mesh_Distance_MCA_R0_R1	-12,3097744	-101,0397339	-941,9127808	0,109712
Cloud_Mesh_Distance_MCA_R0_R1	-12,37319374	-101,029419	-942,1137085	0,084306
Cloud_Mesh_Distance_MCA_R0_R1	-10,98173904	-103,942688	-944,4175415	0,181145
Cloud_Mesh_Distance_MCA_R0_R1	-10,83633614	-103,9863892	-944,3130493	0,180001
Cloud_Mesh_Distance_MCA_R0_R1	-10,83826447	-103,861084	-944,4019775	0,178574
Cloud_Mesh_Distance_MCA_R0_R1	-11,48335075	-103,9006958	-944,5339966	0,160584
Cloud_Mesh_Distance_MCA_R0_R1	-11,32372665	-103,9813232	-944,4768677	0,166392
Cloud_Mesh_Distance_MCA_R0_R1	-11,15445709	-103,857666	-944,5251465	0,180474
Cloud_Mesh_Distance_MCA_R0_R1	-10,58023643	-103,6650391	-944,3447876	0,165755
Cloud_Mesh_Distance_MCA_R0_R1	-10,67987061	-103,8641968	-944,2980957	0,179119
Cloud_Mesh_Distance_MCA_R0_R1	-10,77007484	-103,7210083	-944,4401245	0,17666
Cloud_Mesh_Distance_MCA_R0_R1	-10,49714851	-103,4468994	-944,3807373	0,135021
Cloud_Mesh_Distance_MCA_R0_R1	-11,62436104	-103,8137207	-944,5772705	0,165674
Cloud_Mesh_Distance_MCA_R0_R1	-11,65924263	-103,9835815	-944,4556885	0,125771
Cloud_Mesh_Distance_MCA_R0_R1	-10,35829926	-103,4609375	-944,2680664	0,137182
Cloud_Mesh_Distance_MCA_R0_R1	-10,247509	-103,4834595	-944,1593018	0,138444
Cloud_Mesh_Distance_MCA_R0_R1	-10,09615326	-103,4937134	-943,9921875	0,155319
Cloud_Mesh_Distance_MCA_R0_R1	-9,76454926	-103,0561523	-944,0256348	0,014596
Cloud_Mesh_Distance_MCA_R0_R1	-9,9206543	-103,1643677	-944,0605469	0,028179
Cloud_Mesh_Distance_MCA_R0_R1	-9,64862251	-103,1629639	-943,8386231	0,089905
Cloud_Mesh_Distance_MCA_R0_R1	-9,36410522	-102,946167	-943,9283447	-0,033646
Cloud_Mesh_Distance_MCA_R0_R1	-9,2308197	-102,9589233	-943,7752686	0,01144
Cloud_Mesh_Distance_MCA_R0_R1	-8,9192009	-102,8210449	-943,7102051	-0,054308
Cloud_Mesh_Distance_MCA_R0_R1	-12,02173424	-103,9563599	-944,3446045	0,053871
Cloud_Mesh_Distance_MCA_R0_R1	-12,14358521	-103,8543091	-944,3619995	0,059099
Cloud_Mesh_Distance_MCA_R0_R1	-11,99398041	-103,8276978	-944,4624023	0,099995
Cloud_Mesh_Distance_MCA_R0_R1	-11,80414772	-103,8156128	-944,5387573	0,138341
Cloud_Mesh_Distance_MCA_R0_R1	-9,06625366	-102,9000244	-943,68396	0,001029
Cloud_Mesh_Distance_MCA_R0_R1	-12,387146	-103,7598267	-944,2592163	0,027322
Cloud_Mesh_Distance_MCA_R0_R1	-12,26391029	-103,8594971	-944,2728882	0,02269
Cloud_Mesh_Distance_MCA_R0_R1	-8,87443542	-102,7908325	-943,373169	0,06074
Cloud_Mesh_Distance_MCA_R0_R1	-8,70561218	-102,6800537	-943,4074097	0,021809
Cloud_Mesh_Distance_MCA_R0_R1	-8,81044769	-102,7598267	-943,5568237	-0,014418
Cloud_Mesh_Distance_MCA_R0_R1	-12,51275253	-103,6268921	-944,2490845	0,037096
Cloud_Mesh_Distance_MCA_R0_R1	-12,63917351	-103,6141968	-944,1132202	0,006914
Cloud_Mesh_Distance_MCA_R0_R1	-12,62174606	-103,4695435	-944,2444458	0,066266
Cloud_Mesh_Distance_MCA_R0_R1	-12,50850296	-103,776062	-944,1207275	-0,019313
Cloud_Mesh_Distance_MCA_R0_R1	-12,76698112	-103,4317017	-944,0805054	0,028669

Cloud_Mesh_Distance_MCA_R0_R1	-12,96662903	-103,3118286	-943,817627	-0,013298
Cloud_Mesh_Distance_MCA_R0_R1	-13,11714172	-103,1483765	-943,5654907	-0,017207
Cloud_Mesh_Distance_MCA_R0_R1	-13,10423279	-102,9065552	-943,6734009	0,040114
Cloud_Mesh_Distance_MCA_R0_R1	-13,24957848	-102,8215332	-943,2802124	0,054656
Cloud_Mesh_Distance_MCA_R0_R1	-13,29705429	-102,7208862	-943,1124268	0,083663
Cloud_Mesh_Distance_MCA_R0_R1	-13,20288467	-102,4425659	-943,3354492	0,12055
Cloud_Mesh_Distance_MCA_R0_R1	-13,28188896	-102,4823608	-943,0998535	0,138557
Cloud_Mesh_Distance_MCA_R0_R1	-13,25079727	-102,6295776	-943,2617798	0,100059
Cloud_Mesh_Distance_MCA_R0_R1	-13,33400345	-102,6356201	-942,9346924	0,132851
Cloud_Mesh_Distance_MCA_R0_R1	-13,35866165	-102,562439	-942,7305298	0,166231
Cloud_Mesh_Distance_MCA_R0_R1	-13,32254982	-102,3452148	-942,7578125	0,206461
Cloud_Mesh_Distance_MCA_R0_R1	-13,29738235	-102,2042236	-942,5881958	0,244105
Cloud_Mesh_Distance_MCA_R0_R1	-13,11817551	-101,8271484	-942,3268433	0,252509
Cloud_Mesh_Distance_MCA_R0_R1	-13,00544739	-101,6685791	-942,2493286	0,229308
Cloud_Mesh_Distance_MCA_R0_R1	-12,90891647	-101,5623169	-942,1754761	0,204081
Cloud_Mesh_Distance_MCA_R0_R1	-12,91132355	-101,5115967	-942,3293457	0,217243
Cloud_Mesh_Distance_MCA_R0_R1	-12,9572258	-101,6797485	-942,0748291	0,19053
Cloud_Mesh_Distance_MCA_R0_R1	-12,85084724	-101,5507202	-942,0406494	0,157454
Cloud_Mesh_Distance_MCA_R0_R1	-12,64606476	-101,2810669	-942,1237183	0,129308
Cloud_Mesh_Distance_MCA_R0_R1	-12,58552933	-101,3035889	-941,921875	0,070922
Cloud_Mesh_Distance_MCA_R0_R1	-12,43972397	-101,1555176	-941,9258423	0,041103
Cloud_Mesh_Distance_MCA_R0_R1	-12,48102951	-101,2601929	-941,7910156	0,093732
Cloud_Mesh_Distance_MCA_R0_R1	-12,35944366	-101,1517334	-941,762146	-0,151741
Cloud_Mesh_Distance_MCA_R0_R1	-12,00628662	-100,7969971	-941,8657227	0,33745
Cloud_Mesh_Distance_MCA_R0_R1	-11,78898621	-100,6210327	-941,8952637	0,182052
Cloud_Mesh_Distance_MCA_R0_R1	-11,28484344	-104,3054199	-944,1743164	0,133364
Cloud_Mesh_Distance_MCA_R0_R1	-11,15225983	-104,0474854	-944,3997803	0,168565
Cloud_Mesh_Distance_MCA_R0_R1	-10,98720932	-104,093811	-944,3030396	0,176579
Cloud_Mesh_Distance_MCA_R0_R1	-11,14545059	-104,2005005	-944,2627564	0,154161
Cloud_Mesh_Distance_MCA_R0_R1	-10,72518539	-104,0127564	-944,2095947	0,179848
Cloud_Mesh_Distance_MCA_R0_R1	-11,57893753	-104,177124	-944,3059692	0,105966
Cloud_Mesh_Distance_MCA_R0_R1	-11,477211	-104,0477905	-944,4313965	0,143158
Cloud_Mesh_Distance_MCA_R0_R1	-10,39772797	-103,6013794	-944,2290649	0,154141
Cloud_Mesh_Distance_MCA_R0_R1	-11,93979073	-104,093811	-944,2590942	0,034262
Cloud_Mesh_Distance_MCA_R0_R1	-11,86658478	-103,970459	-944,4053955	0,086853
Cloud_Mesh_Distance_MCA_R0_R1	-9,9716301	-103,4733276	-943,8499756	0,169462
Cloud_Mesh_Distance_MCA_R0_R1	-9,81220627	-103,218811	-943,93396	0,091815
Cloud_Mesh_Distance_MCA_R0_R1	-9,94478035	-103,3377075	-943,9596558	0,117142
Cloud_Mesh_Distance_MCA_R0_R1	-9,81838989	-103,352417	-943,7859497	0,144908
Cloud_Mesh_Distance_MCA_R0_R1	-9,67438889	-103,2842407	-943,6691895	0,146351
Cloud_Mesh_Distance_MCA_R0_R1	-9,43995857	-103,0528565	-943,8019409	0,056131
Cloud_Mesh_Distance_MCA_R0_R1	-9,45930481	-103,1755981	-943,454773	0,152728
Cloud_Mesh_Distance_MCA_R0_R1	-9,52589035	-103,1787109	-943,647583	0,134344
Cloud_Mesh_Distance_MCA_R0_R1	-9,34672928	-103,0718994	-943,6068115	0,109356
Cloud_Mesh_Distance_MCA_R0_R1	-9,16208458	-102,9698486	-943,5515137	0,079142
Cloud_Mesh_Distance_MCA_R0_R1	-8,98309326	-102,8641968	-943,5300293	0,039877
Cloud_Mesh_Distance_MCA_R0_R1	-9,08040619	-102,9238281	-943,3041382	0,114296
Cloud_Mesh_Distance_MCA_R0_R1	-12,15089607	-104,0072022	-944,2142334	-0,000169
Cloud_Mesh_Distance_MCA_R0_R1	-12,34963989	-103,9147949	-944,1435547	-0,024699
Cloud_Mesh_Distance_MCA_R0_R1	-8,90984344	-102,8011475	-943,2115479	0,098135

Cloud_Mesh_Distance_MCA_R0_R1	-8,68924332	-102,6261597	-943,0973511	0,162856
Cloud_Mesh_Distance_MCA_R0_R1	-8,60041428	-102,5830078	-943,2468262	0,060029
Cloud_Mesh_Distance_MCA_R0_R1	-8,52216339	-102,4788818	-942,9989014	0,297698
Cloud_Mesh_Distance_MCA_R0_R1	-12,74299622	-103,6004639	-943,9804077	-0,028797
Cloud_Mesh_Distance_MCA_R0_R1	-12,64167213	-103,7573853	-943,9718018	-0,059792
Cloud_Mesh_Distance_MCA_R0_R1	-12,77681351	-103,6906738	-943,8347168	-0,085627
Cloud_Mesh_Distance_MCA_R0_R1	-12,86428452	-103,4925537	-943,8751831	-0,033423
Cloud_Mesh_Distance_MCA_R0_R1	-12,88739014	-103,6070557	-943,7215576	-0,091056
Cloud_Mesh_Distance_MCA_R0_R1	-12,97428703	-103,4511719	-943,6929321	-0,068023
Cloud_Mesh_Distance_MCA_R0_R1	-13,04191971	-103,2887573	-943,6689453	-0,034622
Cloud_Mesh_Distance_MCA_R0_R1	-13,08015823	-103,3829346	-943,5003662	-0,087423
Cloud_Mesh_Distance_MCA_R0_R1	-13,18735123	-103,1962891	-943,3394775	-0,047366
Cloud_Mesh_Distance_MCA_R0_R1	-13,19099808	-102,9624023	-943,4348145	0,014654
Cloud_Mesh_Distance_MCA_R0_R1	-13,24168015	-103,0068359	-943,2648926	0,009028
Cloud_Mesh_Distance_MCA_R0_R1	-13,36379814	-102,7648926	-942,7880249	0,116689
Cloud_Mesh_Distance_MCA_R0_R1	-13,37992477	-102,7212524	-942,6392822	0,13735
Cloud_Mesh_Distance_MCA_R0_R1	-13,34760094	-102,4197998	-942,5516357	0,215673
Cloud_Mesh_Distance_MCA_R0_R1	-13,30615234	-102,2962036	-942,3413086	0,243236
Cloud_Mesh_Distance_MCA_R0_R1	-13,0604744	-101,817627	-942,1230469	0,227078
Cloud_Mesh_Distance_MCA_R0_R1	-12,89388275	-101,6854858	-941,9112549	0,139457
Cloud_Mesh_Distance_MCA_R0_R1	-12,79290009	-101,5553589	-941,9022217	0,100777
Cloud_Mesh_Distance_MCA_R0_R1	-12,73061371	-101,425415	-941,9998779	0,119033
Cloud_Mesh_Distance_MCA_R0_R1	-12,69043159	-101,4726563	-941,8291016	0,064631
Cloud_Mesh_Distance_MCA_R0_R1	-12,18897438	-100,9948731	-941,7507324	-0,30529
Cloud_Mesh_Distance_MCA_R0_R1	-12,15058136	-101,057312	-941,5362549	-0,468732
Cloud_Mesh_Distance_MCA_R0_R1	-10,84415436	-104,116394	-944,1962891	0,174742
Cloud_Mesh_Distance_MCA_R0_R1	-10,48566055	-103,7940674	-944,1795654	0,178236
Cloud_Mesh_Distance_MCA_R0_R1	-10,59173965	-103,9639282	-944,1320801	0,184075
Cloud_Mesh_Distance_MCA_R0_R1	-11,34145737	-104,1606445	-944,3361816	0,144092
Cloud_Mesh_Distance_MCA_R0_R1	-11,44106293	-104,281189	-944,2133789	0,116279
Cloud_Mesh_Distance_MCA_R0_R1	-11,7667141	-104,1034546	-944,326416	0,074455
Cloud_Mesh_Distance_MCA_R0_R1	-9,94352722	-103,5325317	-943,6831665	0,167782
Cloud_Mesh_Distance_MCA_R0_R1	-10,08971024	-103,6226807	-943,8277588	0,172092
Cloud_Mesh_Distance_MCA_R0_R1	-12,03179932	-104,2189331	-944,0578613	-0,031538
Cloud_Mesh_Distance_MCA_R0_R1	-12,27037811	-104,0933228	-944,0141602	-0,071679
Cloud_Mesh_Distance_MCA_R0_R1	-9,2645874	-103,0431519	-943,2216187	0,147431
Cloud_Mesh_Distance_MCA_R0_R1	-9,28618431	-103,0611572	-943,4007568	0,136939
Cloud_Mesh_Distance_MCA_R0_R1	-8,99858475	-102,8443604	-943,0759277	0,121433
Cloud_Mesh_Distance_MCA_R0_R1	-8,76676559	-102,703186	-943,234436	0,072519
Cloud_Mesh_Distance_MCA_R0_R1	-12,48444748	-103,9527588	-943,9532471	-0,092745
Cloud_Mesh_Distance_MCA_R0_R1	-8,83328247	-102,7283936	-943,0770874	0,164586
Cloud_Mesh_Distance_MCA_R0_R1	-8,81210709	-102,6613159	-942,7894287	0,290347
Cloud_Mesh_Distance_MCA_R0_R1	-8,66279221	-102,5477295	-942,7753296	0,466728
Cloud_Mesh_Distance_MCA_R0_R1	-8,73564911	-102,633728	-942,9394531	0,281958
Cloud_Mesh_Distance_MCA_R0_R1	-8,49797058	-102,4043579	-942,7166748	0,582276
Cloud_Mesh_Distance_MCA_R0_R1	-12,97038269	-103,59552	-943,5571289	-0,129045
Cloud_Mesh_Distance_MCA_R0_R1	-13,26145935	-103,1256714	-943,140686	-0,023587
Cloud_Mesh_Distance_MCA_R0_R1	-13,28903198	-102,9265747	-943,1234741	0,031089
Cloud_Mesh_Distance_MCA_R0_R1	-13,31594086	-103,0432129	-942,9733887	0,00319
Cloud_Mesh_Distance_MCA_R0_R1	-13,33255005	-102,8405151	-942,9587402	0,073558

Cloud_Mesh_Distance_MCA_R0_R1	-13,35722351	-102,960083	-942,8073731	0,05551
Cloud_Mesh_Distance_MCA_R0_R1	-13,38376999	-102,8804321	-942,6468506	0,096423
Cloud_Mesh_Distance_MCA_R0_R1	-13,39352798	-102,793457	-942,498169	0,135703
Cloud_Mesh_Distance_MCA_R0_R1	-13,37692261	-102,6158447	-942,5238037	0,180221
Cloud_Mesh_Distance_MCA_R0_R1	-13,22925568	-102,0385132	-942,4052734	0,261699
Cloud_Mesh_Distance_MCA_R0_R1	-13,23666	-102,1499634	-942,2052002	0,252754
Cloud_Mesh_Distance_MCA_R0_R1	-13,16047287	-101,9857788	-942,1716309	0,246777
Cloud_Mesh_Distance_MCA_R0_R1	-12,99388504	-101,8046265	-941,96521	0,182604
Cloud_Mesh_Distance_MCA_R0_R1	-12,95301628	-101,8894043	-941,7626953	0,120436
Cloud_Mesh_Distance_MCA_R0_R1	-12,65934372	-101,5267944	-941,6856079	0,161183
Cloud_Mesh_Distance_MCA_R0_R1	-12,78593445	-101,6529541	-941,7407837	0,073842
Cloud_Mesh_Distance_MCA_R0_R1	-12,56168747	-101,482605	-941,5758057	-0,285199
Cloud_Mesh_Distance_MCA_R0_R1	-12,57210541	-101,390625	-941,7354736	0,130516
Cloud_Mesh_Distance_MCA_R0_R1	-12,44124985	-101,3135376	-941,6207275	-0,264939
Cloud_Mesh_Distance_MCA_R0_R1	-12,28461075	-101,1696167	-941,5804443	-0,34327
Cloud_Mesh_Distance_MCA_R0_R1	-12,46250153	-101,4351196	-941,4712524	-0,409047
Cloud_Mesh_Distance_MCA_R0_R1	-11,13363647	-104,3084717	-944,1282959	0,143265
Cloud_Mesh_Distance_MCA_R0_R1	-10,99318695	-104,2244873	-944,1688232	0,159903
Cloud_Mesh_Distance_MCA_R0_R1	-10,85368729	-104,2298584	-944,0534058	0,165548
Cloud_Mesh_Distance_MCA_R0_R1	-10,5585804	-104,0527954	-943,9634399	0,177258
Cloud_Mesh_Distance_MCA_R0_R1	-10,70686531	-104,1055298	-944,0844727	0,174225
Cloud_Mesh_Distance_MCA_R0_R1	-10,44958115	-103,9029541	-944,0264893	0,183034
Cloud_Mesh_Distance_MCA_R0_R1	-11,58083344	-104,3275757	-944,1382446	0,078446
Cloud_Mesh_Distance_MCA_R0_R1	-10,2801857	-103,7139893	-944,0102539	0,172922
Cloud_Mesh_Distance_MCA_R0_R1	-11,69767761	-104,3743286	-944,0365601	0,040705
Cloud_Mesh_Distance_MCA_R0_R1	-9,95432281	-103,5864868	-943,427124	0,138764
Cloud_Mesh_Distance_MCA_R0_R1	-11,78080368	-104,2496338	-944,1663818	0,043685
Cloud_Mesh_Distance_MCA_R0_R1	-9,78627777	-103,4157105	-943,5717163	0,157719
Cloud_Mesh_Distance_MCA_R0_R1	-9,62377167	-103,2927246	-943,5020142	0,149749
Cloud_Mesh_Distance_MCA_R0_R1	-9,74529076	-103,4042358	-943,3652954	0,149243
Cloud_Mesh_Distance_MCA_R0_R1	-9,84846115	-103,4811401	-943,2212524	0,118437
Cloud_Mesh_Distance_MCA_R0_R1	-9,57677841	-103,2709351	-943,3096314	0,155491
Cloud_Mesh_Distance_MCA_R0_R1	-9,53155899	-103,2219849	-943,114502	0,151936
Cloud_Mesh_Distance_MCA_R0_R1	-9,41301727	-103,1489258	-943,2616577	0,156971
Cloud_Mesh_Distance_MCA_R0_R1	-9,17994308	-102,9679565	-943,0701294	0,141621
Cloud_Mesh_Distance_MCA_R0_R1	-12,53303146	-104,0636597	-943,7405396	-0,156714
Cloud_Mesh_Distance_MCA_R0_R1	-12,3886261	-104,2432251	-943,6572876	-0,167051
Cloud_Mesh_Distance_MCA_R0_R1	-12,37831497	-104,1321411	-943,8441773	-0,12607
Cloud_Mesh_Distance_MCA_R0_R1	-9,65568542	-103,2894898	-942,9642334	0,116688
Cloud_Mesh_Distance_MCA_R0_R1	-9,69777298	-103,354187	-943,1650391	0,133239
Cloud_Mesh_Distance_MCA_R0_R1	-9,48337173	-103,1555786	-942,9206543	0,136272
Cloud_Mesh_Distance_MCA_R0_R1	-9,36042404	-103,0947876	-943,0744629	0,150774
Cloud_Mesh_Distance_MCA_R0_R1	-9,28949356	-103,008728	-942,8602295	0,135451
Cloud_Mesh_Distance_MCA_R0_R1	-9,08431244	-102,8722534	-942,8895874	0,129381
Cloud_Mesh_Distance_MCA_R0_R1	-12,66503334	-103,8781738	-943,7949219	-0,124952
Cloud_Mesh_Distance_MCA_R0_R1	-12,82967758	-103,7810669	-943,6312256	-0,147664
Cloud_Mesh_Distance_MCA_R0_R1	-8,97150993	-102,7574463	-942,7026367	0,148326
Cloud_Mesh_Distance_MCA_R0_R1	-8,9056015	-102,7520142	-942,9110107	0,173825
Cloud_Mesh_Distance_MCA_R0_R1	-9,15623093	-102,888794	-942,7194214	0,124648
Cloud_Mesh_Distance_MCA_R0_R1	-8,78757477	-102,6108398	-942,6251221	0,324553

Cloud_Mesh_Distance_MCA_R0_R1	-8,6353035	-102,4908447	-942,5966797	0,499655
Cloud_Mesh_Distance_MCA_R0_R1	-8,92106819	-102,6755371	-942,4760132	0,168323
Cloud_Mesh_Distance_MCA_R0_R1	-12,94830513	-103,7509155	-943,4217529	-0,18734
Cloud_Mesh_Distance_MCA_R0_R1	-13,06258774	-103,5703735	-943,3609009	-0,142423
Cloud_Mesh_Distance_MCA_R0_R1	-13,14860344	-103,5284424	-943,1726074	-0,147972
Cloud_Mesh_Distance_MCA_R0_R1	-13,14456558	-103,4036255	-943,305481	-0,105532
Cloud_Mesh_Distance_MCA_R0_R1	-13,21498489	-103,3278198	-943,156189	-0,087528
Cloud_Mesh_Distance_MCA_R0_R1	-13,28277206	-103,2476196	-942,9845581	-0,059358
Cloud_Mesh_Distance_MCA_R0_R1	-13,38699722	-102,7697144	-942,1612549	0,197392
Cloud_Mesh_Distance_MCA_R0_R1	-13,36406898	-102,6101074	-942,2226563	0,20888
Cloud_Mesh_Distance_MCA_R0_R1	-13,35831833	-102,5150757	-942,3620606	0,21324
Cloud_Mesh_Distance_MCA_R0_R1	-13,38601303	-102,6950684	-942,3508301	0,180528
Cloud_Mesh_Distance_MCA_R0_R1	-13,32688522	-102,463501	-942,1802368	0,231184
Cloud_Mesh_Distance_MCA_R0_R1	-13,26551819	-102,3030396	-942,0996704	0,244925
Cloud_Mesh_Distance_MCA_R0_R1	-13,18148804	-102,1369629	-942,0174561	0,24065
Cloud_Mesh_Distance_MCA_R0_R1	-13,0810318	-101,9560547	-941,9719238	0,217062
Cloud_Mesh_Distance_MCA_R0_R1	-13,09650612	-102,1120606	-941,8170166	0,200731
Cloud_Mesh_Distance_MCA_R0_R1	-12,80275726	-101,8213501	-941,5604248	0,140966
Cloud_Mesh_Distance_MCA_R0_R1	-12,67527008	-101,6417236	-941,5565186	-0,242089
Cloud_Mesh_Distance_MCA_R0_R1	-12,54509735	-101,5960083	-941,3968506	-0,446548
Cloud_Mesh_Distance_MCA_R0_R1	-12,34975815	-101,3223267	-941,4448242	-0,459069
Cloud_Mesh_Distance_MCA_R0_R1	-11,23506927	-104,4213867	-943,9800415	0,115843
Cloud_Mesh_Distance_MCA_R0_R1	-11,42176437	-104,3834229	-944,078125	0,100469
Cloud_Mesh_Distance_MCA_R0_R1	-11,02140045	-104,3356323	-944,0186768	0,144974
Cloud_Mesh_Distance_MCA_R0_R1	-10,88307953	-104,3247681	-943,8874512	0,143341
Cloud_Mesh_Distance_MCA_R0_R1	-10,71212578	-104,2019043	-943,9199219	0,158767
Cloud_Mesh_Distance_MCA_R0_R1	-11,56420326	-104,4292603	-943,9881592	0,059596
Cloud_Mesh_Distance_MCA_R0_R1	-11,54742813	-104,5075073	-943,8254395	0,043235
Cloud_Mesh_Distance_MCA_R0_R1	-11,42592239	-104,4628296	-943,937561	0,078771
Cloud_Mesh_Distance_MCA_R0_R1	-10,20868683	-103,7756958	-943,7870483	0,16383
Cloud_Mesh_Distance_MCA_R0_R1	-10,37887383	-103,9371948	-943,828125	0,166167
Cloud_Mesh_Distance_MCA_R0_R1	-10,24732208	-103,8567505	-943,6224976	0,136081
Cloud_Mesh_Distance_MCA_R0_R1	-10,42271423	-104,0240479	-943,5924683	0,1118
Cloud_Mesh_Distance_MCA_R0_R1	-10,08987808	-103,6963501	-943,638794	0,158093
Cloud_Mesh_Distance_MCA_R0_R1	-10,13669586	-103,7582398	-943,4816895	0,122304
Cloud_Mesh_Distance_MCA_R0_R1	-12,04523468	-104,4212036	-943,7196045	-0,087559
Cloud_Mesh_Distance_MCA_R0_R1	-11,850914	-104,3619995	-943,977417	-0,004586
Cloud_Mesh_Distance_MCA_R0_R1	-10,28834534	-103,8924561	-943,4001465	0,075671
Cloud_Mesh_Distance_MCA_R0_R1	-12,00133514	-104,3547363	-943,8817139	-0,056571
Cloud_Mesh_Distance_MCA_R0_R1	-12,19385529	-104,2749634	-943,833374	-0,106304
Cloud_Mesh_Distance_MCA_R0_R1	-10,12325478	-103,7328491	-943,3215942	0,091022
Cloud_Mesh_Distance_MCA_R0_R1	-9,83221054	-103,4336548	-943,0204468	0,095811
Cloud_Mesh_Distance_MCA_R0_R1	-12,56509972	-104,1517944	-943,5567627	-0,201997
Cloud_Mesh_Distance_MCA_R0_R1	-12,46971512	-104,2737427	-943,4918213	-0,197964
Cloud_Mesh_Distance_MCA_R0_R1	-12,34362984	-104,3674316	-943,486145	-0,176304
Cloud_Mesh_Distance_MCA_R0_R1	-12,20898056	-104,385376	-943,6192017	-0,136522
Cloud_Mesh_Distance_MCA_R0_R1	-10,02096176	-103,6087647	-943,133606	0,072716
Cloud_Mesh_Distance_MCA_R0_R1	-10,2349987	-103,7976685	-943,1640625	0,034619
Cloud_Mesh_Distance_MCA_R0_R1	-9,98426819	-103,5175781	-942,8707275	0,046783
Cloud_Mesh_Distance_MCA_R0_R1	-9,60283279	-103,2156982	-942,7902222	0,108339

Cloud_Mesh_Distance_MCA_R0_R1	-12,69151306	-103,9848633	-943,6060181	-0,185111
Cloud_Mesh_Distance_MCA_R0_R1	-12,82578659	-103,9206543	-943,4517822	-0,215444
Cloud_Mesh_Distance_MCA_R0_R1	-9,44067383	-103,0440674	-942,5021973	0,114594
Cloud_Mesh_Distance_MCA_R0_R1	-9,45852852	-103,097229	-942,7141113	0,119098
Cloud_Mesh_Distance_MCA_R0_R1	-9,21659851	-102,8471069	-942,2920532	0,146404
Cloud_Mesh_Distance_MCA_R0_R1	-9,29754639	-102,9678345	-942,6282959	0,122683
Cloud_Mesh_Distance_MCA_R0_R1	-9,12958145	-102,828186	-942,5106812	0,113458
Cloud_Mesh_Distance_MCA_R0_R1	-12,83144188	-104,0255737	-943,289856	-0,245791
Cloud_Mesh_Distance_MCA_R0_R1	-12,92485046	-103,8776856	-943,3076172	-0,225254
Cloud_Mesh_Distance_MCA_R0_R1	-9,0335083	-102,7200928	-942,3001099	0,109905
Cloud_Mesh_Distance_MCA_R0_R1	-13,04363632	-103,742981	-943,2086792	-0,200757
Cloud_Mesh_Distance_MCA_R0_R1	-13,23089981	-103,4545898	-942,996582	-0,118929
Cloud_Mesh_Distance_MCA_R0_R1	-13,24857712	-103,5830689	-942,8346558	-0,142149
Cloud_Mesh_Distance_MCA_R0_R1	-13,30213547	-103,3702393	-942,8250732	-0,075181
Cloud_Mesh_Distance_MCA_R0_R1	-13,3377533	-103,1623535	-942,8157959	-0,018211
Cloud_Mesh_Distance_MCA_R0_R1	-13,37824249	-103,0736694	-942,6486206	0,038762
Cloud_Mesh_Distance_MCA_R0_R1	-13,40190125	-102,9770508	-942,4859619	0,088152
Cloud_Mesh_Distance_MCA_R0_R1	-13,42031288	-103,06427	-942,3048706	0,105897
Cloud_Mesh_Distance_MCA_R0_R1	-13,41384125	-102,9437256	-942,1804199	0,161737
Cloud_Mesh_Distance_MCA_R0_R1	-13,40519524	-102,8745728	-942,3311768	0,150199
Cloud_Mesh_Distance_MCA_R0_R1	-13,34236336	-102,6071777	-942,0595703	0,232359
Cloud_Mesh_Distance_MCA_R0_R1	-13,20702362	-102,328186	-941,8654175	0,238312
Cloud_Mesh_Distance_MCA_R0_R1	-13,29149628	-102,4594116	-942,0030518	0,247439
Cloud_Mesh_Distance_MCA_R0_R1	-13,01153374	-102,1146851	-941,6251221	0,210709
Cloud_Mesh_Distance_MCA_R0_R1	-13,11115646	-102,2573242	-941,6879883	0,224043
Cloud_Mesh_Distance_MCA_R0_R1	-12,90262604	-101,9898071	-941,5508423	0,184912
Cloud_Mesh_Distance_MCA_R0_R1	-12,79664993	-101,9793091	-941,3751221	-0,329875
Cloud_Mesh_Distance_MCA_R0_R1	-12,67689514	-101,7805176	-941,3939819	-0,342239
Cloud_Mesh_Distance_MCA_R0_R1	-12,44911957	-101,6273804	-941,2094727	-0,643487
Cloud_Mesh_Distance_MCA_R0_R1	-12,40433121	-101,468811	-941,3302002	-0,562995
Cloud_Mesh_Distance_MCA_R0_R1	-11,05715561	-104,4208374	-943,8527832	0,110647
Cloud_Mesh_Distance_MCA_R0_R1	-10,93986511	-104,3990479	-943,7168579	0,103506
Cloud_Mesh_Distance_MCA_R0_R1	-10,75434685	-104,2827759	-943,7328491	0,119946
Cloud_Mesh_Distance_MCA_R0_R1	-10,84988022	-104,3549194	-943,5510864	0,057877
Cloud_Mesh_Distance_MCA_R0_R1	-10,56625748	-104,1321411	-943,7643433	0,143817
Cloud_Mesh_Distance_MCA_R0_R1	-11,70890808	-104,4545288	-943,8920898	0,015034
Cloud_Mesh_Distance_MCA_R0_R1	-11,71579742	-104,531189	-943,7029419	-0,015322
Cloud_Mesh_Distance_MCA_R0_R1	-11,91016769	-104,5183106	-943,6077271	-0,071549
Cloud_Mesh_Distance_MCA_R0_R1	-10,63061905	-104,1982422	-943,5256348	0,06606
Cloud_Mesh_Distance_MCA_R0_R1	-11,87343597	-104,4509277	-943,8005371	-0,036968
Cloud_Mesh_Distance_MCA_R0_R1	-10,79453659	-104,2957764	-943,3592529	-0,011925
Cloud_Mesh_Distance_MCA_R0_R1	-12,0737133	-104,4793091	-943,5571899	-0,111281
Cloud_Mesh_Distance_MCA_R0_R1	-10,61883926	-104,1410523	-943,2593994	-0,025474
Cloud_Mesh_Distance_MCA_R0_R1	-10,421278	-103,9683228	-943,2144775	0,002314
Cloud_Mesh_Distance_MCA_R0_R1	-10,47184372	-104,0530396	-943,3994751	0,049597
Cloud_Mesh_Distance_MCA_R0_R1	-10,5527153	-104,0272827	-943,0571289	-0,066479
Cloud_Mesh_Distance_MCA_R0_R1	-10,38364792	-103,8728638	-943,0019531	-0,038967
Cloud_Mesh_Distance_MCA_R0_R1	-12,19613457	-104,4708252	-943,4393921	-0,148705
Cloud_Mesh_Distance_MCA_R0_R1	-12,32918167	-104,4422607	-943,3408203	-0,181368
Cloud_Mesh_Distance_MCA_R0_R1	-10,53067589	-103,9469605	-942,8665772	-0,09896

Cloud_Mesh_Distance_MCA_R0_R1	-10,19384766	-103,6936035	-942,9125977	-0,002926
Cloud_Mesh_Distance_MCA_R0_R1	-12,70493126	-104,0864868	-943,4307251	-0,229283
Cloud_Mesh_Distance_MCA_R0_R1	-12,58512878	-104,2315064	-943,3920288	-0,223993
Cloud_Mesh_Distance_MCA_R0_R1	-12,71318245	-104,1796265	-943,2683106	-0,245187
Cloud_Mesh_Distance_MCA_R0_R1	-10,10862732	-103,5789795	-942,7313232	0,003892
Cloud_Mesh_Distance_MCA_R0_R1	-9,78657913	-103,345459	-942,7719727	0,083181
Cloud_Mesh_Distance_MCA_R0_R1	-9,9822979	-103,4299316	-942,4508057	0,056936
Cloud_Mesh_Distance_MCA_R0_R1	-9,82816696	-103,3283081	-942,5293579	0,078968
Cloud_Mesh_Distance_MCA_R0_R1	-9,97089386	-103,4571533	-942,6503296	0,040755
Cloud_Mesh_Distance_MCA_R0_R1	-9,87083054	-103,3346558	-942,3577881	0,09189
Cloud_Mesh_Distance_MCA_R0_R1	-9,63008118	-103,1922607	-942,5740356	0,102924
Cloud_Mesh_Distance_MCA_R0_R1	-9,29717255	-102,9331665	-942,4487305	0,115537
Cloud_Mesh_Distance_MCA_R0_R1	-9,55971718	-103,1021118	-942,3376465	0,122977
Cloud_Mesh_Distance_MCA_R0_R1	-12,84697151	-104,1298218	-943,1101685	-0,244494
Cloud_Mesh_Distance_MCA_R0_R1	-12,95396805	-103,9435425	-943,159668	-0,236638
Cloud_Mesh_Distance_MCA_R0_R1	-9,38923836	-102,9728394	-942,298523	0,124616
Cloud_Mesh_Distance_MCA_R0_R1	-9,30956268	-102,8834229	-942,0786743	0,219835
Cloud_Mesh_Distance_MCA_R0_R1	-13,06733131	-103,8572388	-943,0213623	-0,220279
Cloud_Mesh_Distance_MCA_R0_R1	-13,15712738	-103,6608276	-943,0133057	-0,167465
Cloud_Mesh_Distance_MCA_R0_R1	-13,17102814	-103,7921143	-942,8555298	-0,181599
Cloud_Mesh_Distance_MCA_R0_R1	-13,35974693	-103,2797241	-942,6539307	-0,028211
Cloud_Mesh_Distance_MCA_R0_R1	-13,38877106	-103,3970337	-942,4888916	-0,008438
Cloud_Mesh_Distance_MCA_R0_R1	-13,40206146	-103,1818848	-942,4751587	0,041363
Cloud_Mesh_Distance_MCA_R0_R1	-13,4019146	-102,9077759	-942,0098877	0,212781
Cloud_Mesh_Distance_MCA_R0_R1	-13,36191559	-102,7439575	-941,9749756	0,229926
Cloud_Mesh_Distance_MCA_R0_R1	-13,2321701	-102,6466065	-941,5595093	0,293174
Cloud_Mesh_Distance_MCA_R0_R1	-13,1712532	-102,4386597	-941,6359863	0,254189
Cloud_Mesh_Distance_MCA_R0_R1	-12,9322834	-102,1449585	-941,4375	0,349582
Cloud_Mesh_Distance_MCA_R0_R1	-13,05215073	-102,2977295	-941,5131226	0,258996
Cloud_Mesh_Distance_MCA_R0_R1	-12,65495682	-101,9011231	-941,2293091	-0,488869
Cloud_Mesh_Distance_MCA_R0_R1	-12,56797409	-101,7451172	-941,2600098	-0,515197
Cloud_Mesh_Distance_MCA_R0_R1	-12,5016346	-101,8262329	-941,0802612	-0,689179
Cloud_Mesh_Distance_MCA_R0_R1	-11,14654541	-104,4816895	-943,7468262	0,093564
Cloud_Mesh_Distance_MCA_R0_R1	-11,3611145	-104,5128784	-943,7960205	0,070912
Cloud_Mesh_Distance_MCA_R0_R1	-11,25572777	-104,5319824	-943,6100464	0,049445
Cloud_Mesh_Distance_MCA_R0_R1	-11,5453949	-104,5977783	-943,3965454	-0,043716
Cloud_Mesh_Distance_MCA_R0_R1	-11,74211884	-104,5903931	-943,4567261	-0,062628
Cloud_Mesh_Distance_MCA_R0_R1	-11,50801849	-104,5679932	-943,607666	0,009924
Cloud_Mesh_Distance_MCA_R0_R1	-11,04862404	-104,4627075	-943,5570068	0,044793
Cloud_Mesh_Distance_MCA_R0_R1	-10,9164238	-104,3517456	-943,2438965	-0,060569
Cloud_Mesh_Distance_MCA_R0_R1	-11,0625782	-104,4282227	-943,2070313	-0,075593
Cloud_Mesh_Distance_MCA_R0_R1	-10,9773407	-104,4142456	-943,3861084	-0,012254
Cloud_Mesh_Distance_MCA_R0_R1	-11,09306335	-104,4041748	-943,0388794	-0,133957
Cloud_Mesh_Distance_MCA_R0_R1	-12,00370407	-104,5582886	-943,3895264	-0,118834
Cloud_Mesh_Distance_MCA_R0_R1	-11,00827789	-104,3053589	-942,8812256	-0,165345
Cloud_Mesh_Distance_MCA_R0_R1	-10,79807281	-104,2514038	-943,170105	-0,079216
Cloud_Mesh_Distance_MCA_R0_R1	-10,69378281	-104,1452026	-943,0786743	-0,091091
Cloud_Mesh_Distance_MCA_R0_R1	-10,68218803	-104,0822754	-942,9197388	-0,123593
Cloud_Mesh_Distance_MCA_R0_R1	-12,43409729	-104,4500732	-943,1826782	-0,195091
Cloud_Mesh_Distance_MCA_R0_R1	-12,20912933	-104,5386963	-943,2302856	-0,158754

Cloud_Mesh_Distance_MCA_R0_R1	-10,9502449	-104,3309937	-943,0872192	-0,116789
Cloud_Mesh_Distance_MCA_R0_R1	-10,82563591	-104,213562	-942,9872437	-0,132287
Cloud_Mesh_Distance_MCA_R0_R1	-10,38137436	-103,8075562	-942,7969971	-0,066944
Cloud_Mesh_Distance_MCA_R0_R1	-10,66735077	-104,0125732	-942,7404785	-0,137898
Cloud_Mesh_Distance_MCA_R0_R1	-12,7085762	-104,2654419	-943,1357422	-0,238194
Cloud_Mesh_Distance_MCA_R0_R1	-12,45943642	-104,3521729	-943,3565674	-0,206719
Cloud_Mesh_Distance_MCA_R0_R1	-12,58737183	-104,3161011	-943,2357178	-0,226183
Cloud_Mesh_Distance_MCA_R0_R1	-10,25943756	-103,6828003	-942,6858521	-0,036708
Cloud_Mesh_Distance_MCA_R0_R1	-10,40350723	-103,7800903	-942,6329346	-0,071277
Cloud_Mesh_Distance_MCA_R0_R1	-10,14227676	-103,5632324	-942,5335693	0,002955
Cloud_Mesh_Distance_MCA_R0_R1	-10,3336525	-103,7000732	-942,5024414	-0,039284
Cloud_Mesh_Distance_MCA_R0_R1	-9,98588753	-103,4085083	-942,2559815	0,088625
Cloud_Mesh_Distance_MCA_R0_R1	-9,73009491	-103,230835	-942,3583374	0,112504
Cloud_Mesh_Distance_MCA_R0_R1	-9,49919319	-103,0310669	-942,1276856	0,148177
Cloud_Mesh_Distance_MCA_R0_R1	-12,98050499	-104,0164185	-943,015625	-0,24122
Cloud_Mesh_Distance_MCA_R0_R1	-13,07308388	-103,9800415	-942,8822632	-0,221904
Cloud_Mesh_Distance_MCA_R0_R1	-12,94437027	-104,1623535	-942,9066773	-0,223161
Cloud_Mesh_Distance_MCA_R0_R1	-13,17598343	-103,9414673	-942,7268677	-0,171697
Cloud_Mesh_Distance_MCA_R0_R1	-13,27787209	-103,7263184	-942,6611939	-0,11647
Cloud_Mesh_Distance_MCA_R0_R1	-13,32701492	-103,4901733	-942,6641235	-0,07383
Cloud_Mesh_Distance_MCA_R0_R1	-13,36986351	-103,7739868	-942,4420166	-0,017684
Cloud_Mesh_Distance_MCA_R0_R1	-13,35995483	-103,5855103	-942,5220947	-0,050405
Cloud_Mesh_Distance_MCA_R0_R1	-13,42253113	-103,546814	-942,3493652	0,033354
Cloud_Mesh_Distance_MCA_R0_R1	-13,44081688	-103,3025513	-942,2570801	0,087325
Cloud_Mesh_Distance_MCA_R0_R1	-13,42638397	-103,0545044	-941,8234863	0,27682
Cloud_Mesh_Distance_MCA_R0_R1	-13,43770218	-103,0988159	-942,0881958	0,168592
Cloud_Mesh_Distance_MCA_R0_R1	-13,31272697	-102,7697754	-941,6713257	0,303789
Cloud_Mesh_Distance_MCA_R0_R1	-13,36327553	-102,8326416	-941,828064	0,270007
Cloud_Mesh_Distance_MCA_R0_R1	-13,29112816	-102,602417	-941,8225098	0,262634
Cloud_Mesh_Distance_MCA_R0_R1	-12,84291649	-102,1677856	-941,2583618	-0,357781
Cloud_Mesh_Distance_MCA_R0_R1	-12,62926292	-102,0281372	-941,0681152	-0,57486
Cloud_Mesh_Distance_MCA_R0_R1	-12,73689461	-102,052002	-941,2050781	-0,485941
Cloud_Mesh_Distance_MCA_R0_R1	-11,38889694	-104,5729981	-943,4421997	-0,011667
Cloud_Mesh_Distance_MCA_R0_R1	-11,19949722	-104,5151978	-943,3643799	-0,025461
Cloud_Mesh_Distance_MCA_R0_R1	-11,85491371	-104,616333	-943,2310181	-0,110557
Cloud_Mesh_Distance_MCA_R0_R1	-11,23630142	-104,4939575	-943,1165161	-0,113478
Cloud_Mesh_Distance_MCA_R0_R1	-11,20618057	-104,432251	-942,9111939	-0,165078
Cloud_Mesh_Distance_MCA_R0_R1	-12,32966805	-104,5596924	-943,0270996	-0,16136
Cloud_Mesh_Distance_MCA_R0_R1	-11,15320969	-104,3582153	-942,7436523	-0,180593
Cloud_Mesh_Distance_MCA_R0_R1	-10,82991028	-104,1508789	-942,7901611	-0,159098
Cloud_Mesh_Distance_MCA_R0_R1	-11,0052948	-104,2268677	-942,623291	-0,173259
Cloud_Mesh_Distance_MCA_R0_R1	-10,79406738	-104,0662231	-942,5963135	-0,152295
Cloud_Mesh_Distance_MCA_R0_R1	-10,64387894	-103,9521484	-942,5915527	-0,126623
Cloud_Mesh_Distance_MCA_R0_R1	-10,52500725	-103,8862915	-942,6859131	-0,100699
Cloud_Mesh_Distance_MCA_R0_R1	-12,77344513	-104,2946167	-942,9920654	-0,213587
Cloud_Mesh_Distance_MCA_R0_R1	-12,61219406	-104,3840942	-943,0718384	-0,206577
Cloud_Mesh_Distance_MCA_R0_R1	-10,70881271	-103,9667358	-942,4421387	-0,108088
Cloud_Mesh_Distance_MCA_R0_R1	-10,52305031	-103,8398438	-942,4951172	-0,082291
Cloud_Mesh_Distance_MCA_R0_R1	-10,2473259	-103,6117554	-942,3410645	0,014961
Cloud_Mesh_Distance_MCA_R0_R1	-10,33158302	-103,6556397	-942,142395	0,065774

Cloud_Mesh_Distance_MCA_R0_R1	-10,4299736	-103,743042	-942,3174439	-0,01582
Cloud_Mesh_Distance_MCA_R0_R1	-10,10004044	-103,5050659	-942,3557129	0,044602
Cloud_Mesh_Distance_MCA_R0_R1	-9,75686264	-103,2138672	-941,9745483	0,197613
Cloud_Mesh_Distance_MCA_R0_R1	-9,83149719	-103,2874146	-942,1835327	0,128421
Cloud_Mesh_Distance_MCA_R0_R1	-12,84519005	-104,3331909	-942,8418579	-0,172122
Cloud_Mesh_Distance_MCA_R0_R1	-9,83143997	-103,258728	-941,7216797	0,281524
Cloud_Mesh_Distance_MCA_R0_R1	-9,60250854	-103,0966797	-941,9935303	0,187228
Cloud_Mesh_Distance_MCA_R0_R1	-9,6634922	-103,1577759	-942,1605225	0,145573
Cloud_Mesh_Distance_MCA_R0_R1	-13,07413483	-104,0975952	-942,7717896	-0,190051
Cloud_Mesh_Distance_MCA_R0_R1	-13,26801682	-103,9197998	-942,5786133	-0,097246
Cloud_Mesh_Distance_MCA_R0_R1	-13,4671669	-103,7125855	-942,2369995	0,105594
Cloud_Mesh_Distance_MCA_R0_R1	-11,753582	-104,6296387	-943,0627441	-0,122087
Cloud_Mesh_Distance_MCA_R0_R1	-11,65239716	-104,6147461	-943,243103	-0,095446
Cloud_Mesh_Distance_MCA_R0_R1	-11,43272018	-104,5778809	-943,227356	-0,081691
Cloud_Mesh_Distance_MCA_R0_R1	-11,42222214	-104,5417481	-942,956604	-0,149084
Cloud_Mesh_Distance_MCA_R0_R1	-11,58186722	-104,6036987	-943,0775147	-0,114549
Cloud_Mesh_Distance_MCA_R0_R1	-11,94251633	-104,6351318	-943,0440064	-0,134615
Cloud_Mesh_Distance_MCA_R0_R1	-11,64452362	-104,6069336	-942,9019775	-0,143707
Cloud_Mesh_Distance_MCA_R0_R1	-12,13280106	-104,6138306	-943,0356445	-0,146554
Cloud_Mesh_Distance_MCA_R0_R1	-12,02913094	-104,6016846	-943,1866455	-0,134029
Cloud_Mesh_Distance_MCA_R0_R1	-11,32670975	-104,4560547	-942,7411499	-0,177269
Cloud_Mesh_Distance_MCA_R0_R1	-12,49916458	-104,491394	-943,003479	-0,179608
Cloud_Mesh_Distance_MCA_R0_R1	-11,22496033	-104,3587647	-942,5706177	-0,158642
Cloud_Mesh_Distance_MCA_R0_R1	-11,12049103	-104,2538452	-942,3996582	-0,119067
Cloud_Mesh_Distance_MCA_R0_R1	-12,45356369	-104,5785523	-942,8548584	-0,125941
Cloud_Mesh_Distance_MCA_R0_R1	-12,66334534	-104,456604	-942,8804321	-0,160103
Cloud_Mesh_Distance_MCA_R0_R1	-11,42553139	-104,4724121	-942,5500488	-0,139007
Cloud_Mesh_Distance_MCA_R0_R1	-10,76675415	-103,9812012	-942,2818604	-0,06283
Cloud_Mesh_Distance_MCA_R0_R1	-11,32350922	-104,3829956	-942,3730469	-0,101916
Cloud_Mesh_Distance_MCA_R0_R1	-10,90654373	-104,100769	-942,3898315	-0,113744
Cloud_Mesh_Distance_MCA_R0_R1	-10,703825	-103,920166	-942,1275635	0,012059
Cloud_Mesh_Distance_MCA_R0_R1	-10,61139107	-103,8726196	-942,2993774	-0,050129
Cloud_Mesh_Distance_MCA_R0_R1	-10,14374161	-103,5203247	-942,1790161	0,086226
Cloud_Mesh_Distance_MCA_R0_R1	-10,42785645	-103,7134399	-941,9050293	0,164349
Cloud_Mesh_Distance_MCA_R0_R1	-12,83742142	-104,4664307	-942,692749	-0,077543
Cloud_Mesh_Distance_MCA_R0_R1	-9,98370743	-103,3897095	-941,9889526	0,175392
Cloud_Mesh_Distance_MCA_R0_R1	-12,99249268	-104,2649536	-942,7346802	-0,141857
Cloud_Mesh_Distance_MCA_R0_R1	-13,1709404	-104,1315918	-942,6102295	-0,102876
Cloud_Mesh_Distance_MCA_R0_R1	-13,2706871	-104,2904053	-942,4257813	0,086935
Cloud_Mesh_Distance_MCA_R0_R1	-13,32884407	-104,026062	-942,4442749	0,001925
Cloud_Mesh_Distance_MCA_R0_R1	-12,03942108	-104,6546631	-942,8671875	-0,125037
Cloud_Mesh_Distance_MCA_R0_R1	-11,84239006	-104,6438599	-942,8796387	-0,13228
Cloud_Mesh_Distance_MCA_R0_R1	-11,93546677	-104,6656494	-942,699585	-0,103159
Cloud_Mesh_Distance_MCA_R0_R1	-11,53007507	-104,5502319	-942,730896	-0,150349
Cloud_Mesh_Distance_MCA_R0_R1	-12,24394417	-104,6368408	-942,8555908	-0,118497
Cloud_Mesh_Distance_MCA_R0_R1	-12,13709259	-104,6843872	-942,6915894	-0,075712
Cloud_Mesh_Distance_MCA_R0_R1	-12,02922058	-104,6995239	-942,5297852	-0,036416
Cloud_Mesh_Distance_MCA_R0_R1	-11,8283329	-104,6467285	-942,5284424	-0,0762
Cloud_Mesh_Distance_MCA_R0_R1	-11,73488617	-104,6204834	-942,7111206	-0,134862
Cloud_Mesh_Distance_MCA_R0_R1	-12,57096672	-104,7089844	-942,529419	0,049311

Cloud_Mesh_Distance_MCA_R0_R1	-12,36925316	-104,6787109	-942,659729	-0,043602
Cloud_Mesh_Distance_MCA_R0_R1	-12,74383736	-104,6199341	-942,5802612	0,015411
Cloud_Mesh_Distance_MCA_R0_R1	-12,92616844	-104,5764771	-942,5201416	0,066538
Cloud_Mesh_Distance_MCA_R0_R1	-12,60839844	-104,5941162	-942,7018433	-0,067789
Cloud_Mesh_Distance_MCA_R0_R1	-11,21527481	-104,2880249	-942,2022095	-0,038024
Cloud_Mesh_Distance_MCA_R0_R1	-10,89264679	-104,0548096	-942,1375122	-0,009007
Cloud_Mesh_Distance_MCA_R0_R1	-11,04532242	-104,1738892	-942,2388916	-0,061266
Cloud_Mesh_Distance_MCA_R0_R1	-10,62430573	-103,8535156	-941,9615479	0,108041
Cloud_Mesh_Distance_MCA_R0_R1	-10,77765274	-103,9591675	-941,9315796	0,11653
Cloud_Mesh_Distance_MCA_R0_R1	-10,52532196	-103,793396	-942,1259766	0,038893
Cloud_Mesh_Distance_MCA_R0_R1	-13,05946159	-104,3778076	-942,5744629	-0,012694
Cloud_Mesh_Distance_MCA_R0_R1	-10,20760345	-103,5564575	-941,9683228	0,157949
Cloud_Mesh_Distance_MCA_R0_R1	-10,06510925	-103,4435425	-941,7597656	0,264029
Cloud_Mesh_Distance_MCA_R0_R1	-10,01771164	-103,3895874	-941,5180664	0,374576
Cloud_Mesh_Distance_MCA_R0_R1	-12,20707512	-104,7230835	-942,5402222	-0,010852
Cloud_Mesh_Distance_MCA_R0_R1	-11,62686729	-104,5695801	-942,5370483	-0,115266
Cloud_Mesh_Distance_MCA_R0_R1	-12,14264679	-104,7533569	-942,3781128	0,067666
Cloud_Mesh_Distance_MCA_R0_R1	-12,77032471	-104,725647	-942,4486084	0,142738
Cloud_Mesh_Distance_MCA_R0_R1	-11,61169815	-104,5421143	-942,2107544	0,016717
Cloud_Mesh_Distance_MCA_R0_R1	-11,52268982	-104,5012207	-942,3624878	-0,075125
Cloud_Mesh_Distance_MCA_R0_R1	-11,44701576	-104,4317627	-941,973877	0,120752
Cloud_Mesh_Distance_MCA_R0_R1	-11,42915726	-104,4268799	-942,1621094	0,010301
Cloud_Mesh_Distance_MCA_R0_R1	-11,28157425	-104,3161011	-941,9824219	0,093339
Cloud_Mesh_Distance_MCA_R0_R1	-11,08543777	-104,1830444	-942,0549316	0,035357
Cloud_Mesh_Distance_MCA_R0_R1	-11,27120781	-104,305481	-941,7861939	0,223367
Cloud_Mesh_Distance_MCA_R0_R1	-11,12408447	-104,2002564	-941,8496094	0,160432
Cloud_Mesh_Distance_MCA_R0_R1	-10,84624481	-103,9987183	-941,7283325	0,234346
Cloud_Mesh_Distance_MCA_R0_R1	-10,95156479	-104,079773	-941,9080811	0,120897
Cloud_Mesh_Distance_MCA_R0_R1	-10,25661087	-103,5861206	-941,7730103	0,253868
Cloud_Mesh_Distance_MCA_R0_R1	-10,21580124	-103,5441284	-941,5830689	0,349454
Cloud_Mesh_Distance_MCA_R0_R1	-13,13318253	-104,5210571	-942,4320068	0,150266
Cloud_Mesh_Distance_MCA_R0_R1	-10,40438843	-103,6843872	-941,6403198	0,310253
Cloud_Mesh_Distance_MCA_R0_R1	-10,34182167	-103,6201172	-941,427063	0,424629
Cloud_Mesh_Distance_MCA_R0_R1	-10,18295479	-103,5026245	-941,4153442	0,429922
Cloud_Mesh_Distance_MCA_R0_R1	-12,35987663	-104,7655029	-942,4450073	0,07534
Cloud_Mesh_Distance_MCA_R0_R1	-11,92427444	-104,6907349	-942,3515625	0,017926
Cloud_Mesh_Distance_MCA_R0_R1	-11,71808434	-104,6026001	-942,3538818	-0,029124
Cloud_Mesh_Distance_MCA_R0_R1	-12,99727821	-104,7290039	-942,3713989	0,257994
Cloud_Mesh_Distance_MCA_R0_R1	-12,8031292	-104,8422241	-942,322876	0,282839
Cloud_Mesh_Distance_MCA_R0_R1	-12,03757095	-104,7636108	-942,1807861	0,160377
Cloud_Mesh_Distance_MCA_R0_R1	-11,7256813	-104,6282349	-941,9053345	0,245748
Cloud_Mesh_Distance_MCA_R0_R1	-11,92939186	-104,7510986	-941,9729004	0,268094
Cloud_Mesh_Distance_MCA_R0_R1	-11,591362	-104,5293579	-942,0396118	0,115364
Cloud_Mesh_Distance_MCA_R0_R1	-11,79187775	-104,6465454	-942,1435547	0,103023
Cloud_Mesh_Distance_MCA_R0_R1	-11,53565979	-104,4990845	-941,8422852	0,23474
Cloud_Mesh_Distance_MCA_R0_R1	-10,95487213	-104,0687866	-941,5620117	0,352348
Cloud_Mesh_Distance_MCA_R0_R1	-11,15399933	-104,2183838	-941,6248169	0,320037
Cloud_Mesh_Distance_MCA_R0_R1	-11,00412369	-104,111145	-941,7352905	0,232016
Cloud_Mesh_Distance_MCA_R0_R1	-11,40343857	-104,4102173	-941,6258545	0,363313
Cloud_Mesh_Distance_MCA_R0_R1	-11,38915253	-104,3911133	-941,8432007	0,201889

Cloud_Mesh_Distance_MCA_R0_R1	-10,73726082	-103,8887329	-941,3314209	0,489246
Cloud_Mesh_Distance_MCA_R0_R1	-10,56268692	-103,7786255	-941,4376221	0,425332
Cloud_Mesh_Distance_MCA_R0_R1	-10,76891327	-103,9334717	-941,5233765	0,36963
Cloud_Mesh_Distance_MCA_R0_R1	-10,63097763	-103,8479614	-941,7278442	0,243409
Cloud_Mesh_Distance_MCA_R0_R1	-12,57349777	-104,8370972	-942,3344116	0,214825
Cloud_Mesh_Distance_MCA_R0_R1	-12,29876709	-104,8480835	-942,2233887	0,219916
Cloud_Mesh_Distance_MCA_R0_R1	-12,71744537	-104,977356	-942,184021	0,427601
Cloud_Mesh_Distance_MCA_R0_R1	-12,72993469	-105,0977783	-942,0673828	0,580062
Cloud_Mesh_Distance_MCA_R0_R1	-12,49828529	-104,9415894	-942,15979	0,357693
Cloud_Mesh_Distance_MCA_R0_R1	-12,02978325	-104,8567505	-941,8217773	0,431617
Cloud_Mesh_Distance_MCA_R0_R1	-11,84887314	-104,7394409	-941,7669678	0,399661
Cloud_Mesh_Distance_MCA_R0_R1	-11,65097427	-104,6044312	-941,6638184	0,400538
Cloud_Mesh_Distance_MCA_R0_R1	-11,8112793	-104,7528687	-941,567627	0,54898
Cloud_Mesh_Distance_MCA_R0_R1	-11,68092918	-104,6582642	-941,4581909	0,577469
Cloud_Mesh_Distance_MCA_R0_R1	-11,25282669	-104,2935181	-941,4125366	0,488344
Cloud_Mesh_Distance_MCA_R0_R1	-10,89440918	-104,0088501	-941,3598633	0,484541
Cloud_Mesh_Distance_MCA_R0_R1	-11,01490021	-104,0795288	-941,1991577	0,59727
Cloud_Mesh_Distance_MCA_R0_R1	-12,55683517	-105,0704956	-942,0153809	0,538092
Cloud_Mesh_Distance_MCA_R0_R1	-12,38039398	-105,06073	-941,888916	0,573877
Cloud_Mesh_Distance_MCA_R0_R1	-12,21384811	-104,9701538	-941,857605	0,507395
Cloud_Mesh_Distance_MCA_R0_R1	-12,34756851	-104,9616089	-942,0285034	0,412869
Cloud_Mesh_Distance_MCA_R0_R1	-12,14181709	-104,8621826	-942,0158691	0,328764
Cloud_Mesh_Distance_MCA_R0_R1	-12,09808922	-104,9724121	-941,6517334	0,627948
Cloud_Mesh_Distance_MCA_R0_R1	-11,94563484	-104,8421021	-941,663208	0,532316
Cloud_Mesh_Distance_MCA_R0_R1	-11,3067627	-104,3311157	-941,2359619	0,626559
Cloud_Mesh_Distance_MCA_R0_R1	-11,07136917	-104,1477661	-941,3983154	0,471528
Cloud_Mesh_Distance_MCA_R0_R1	-11,17189789	-104,2131348	-941,2265015	0,603841
Cloud_Mesh_Distance_MCA_R0_R1	-12,49516296	-105,2006226	-941,802124	0,749583
Cloud_Mesh_Distance_MCA_R0_R1	-12,29185486	-105,0933228	-941,7287598	0,682599
Cloud_Mesh_Distance_MCA_R0_R1	-11,94163132	-104,8866577	-941,5020142	0,676601
Cloud_Mesh_Distance_MCA_R0_R1	-11,59561157	-104,6015015	-941,2409668	0,723643
Cloud_Mesh_Distance_MCA_R0_R1	-11,42100906	-104,43573	-941,355896	0,56747
Cloud_Mesh_Distance_MCA_R0_R1	-11,55021667	-104,5415649	-941,4705811	0,521257
Cloud_Mesh_Distance_MCA_R0_R1	-11,42408371	-104,4329224	-941,1373901	0,734291
Cloud_Mesh_Distance_MCA_R0_R1	-11,52993393	-104,5305786	-941,0354004	0,849865
Cloud_Mesh_Distance_MCA_R0_R1	-11,27493477	-104,2747192	-941,039856	0,757057
Cloud_Mesh_Distance_MCA_R0_R1	-12,36181068	-105,2213135	-941,6177979	0,852441
Cloud_Mesh_Distance_MCA_R0_R1	-12,21037102	-105,1235962	-941,5411987	0,814226
Cloud_Mesh_Distance_MCA_R0_R1	-12,06167793	-105,0251465	-941,4373779	0,813001
Cloud_Mesh_Distance_MCA_R0_R1	-11,79892731	-104,789917	-941,3411865	0,735612
Cloud_Mesh_Distance_MCA_R0_R1	-11,94094467	-104,93396	-941,3418579	0,821781
Cloud_Mesh_Distance_MCA_R0_R1	-11,63085938	-104,638855	-941,0178223	0,909554
Cloud_Mesh_Distance_MCA_R0_R2	-9,91326141	-101,8422241	-944,5302124	0,01931
Cloud_Mesh_Distance_MCA_R0_R2	-9,7016449	-101,9011688	-944,6048584	0,015872
Cloud_Mesh_Distance_MCA_R0_R2	-9,81649399	-102,0139084	-944,5668335	-0,012006
Cloud_Mesh_Distance_MCA_R0_R2	-9,83420181	-101,8982391	-944,5592041	0,013796
Cloud_Mesh_Distance_MCA_R0_R2	-9,66815186	-102,0602493	-944,6191406	-0,016722
Cloud_Mesh_Distance_MCA_R0_R2	-9,79959297	-101,8003922	-944,55896	0,025656
Cloud_Mesh_Distance_MCA_R0_R2	-9,54931831	-101,5607758	-944,5577393	0,045736
Cloud_Mesh_Distance_MCA_R0_R2	-11,8690815	-103,2528534	-944,5882568	0,070277

Cloud_Mesh_Distance_MCA_R0_R2	-11,72317123	-103,2997665	-944,5704346	0,044909
Cloud_Mesh_Distance_MCA_R0_R2	-11,85388756	-103,4010468	-944,5621338	0,057429
Cloud_Mesh_Distance_MCA_R0_R2	-12,03859138	-103,0598831	-944,5847778	0,092882
Cloud_Mesh_Distance_MCA_R0_R2	-11,88154411	-103,09301	-944,6008911	0,077339
Cloud_Mesh_Distance_MCA_R0_R2	-12,01444626	-103,2152557	-944,583252	0,088771
Cloud_Mesh_Distance_MCA_R0_R2	-11,72415924	-103,4224777	-944,5452271	0,036201
Cloud_Mesh_Distance_MCA_R0_R2	-11,54673386	-103,2108688	-944,5526123	0,011327
Cloud_Mesh_Distance_MCA_R0_R2	-11,3923645	-103,2181015	-944,5040894	-0,035402
Cloud_Mesh_Distance_MCA_R0_R2	-11,71985435	-103,1389999	-944,5927734	0,05222
Cloud_Mesh_Distance_MCA_R0_R2	-11,37449837	-103,0607605	-944,5287476	-0,020778
Cloud_Mesh_Distance_MCA_R0_R2	-12,04877853	-102,8898163	-944,5761719	0,086911
Cloud_Mesh_Distance_MCA_R0_R2	-11,92617798	-102,7855759	-944,5871582	0,075508
Cloud_Mesh_Distance_MCA_R0_R2	-11,73229218	-102,9571381	-944,6034546	0,065652
Cloud_Mesh_Distance_MCA_R0_R2	-11,89953995	-102,924202	-944,5999146	0,080937
Cloud_Mesh_Distance_MCA_R0_R2	-11,55834007	-103,0104675	-944,5812988	0,031757
Cloud_Mesh_Distance_MCA_R0_R2	-11,57232094	-102,8204651	-944,5914917	0,048312
Cloud_Mesh_Distance_MCA_R0_R2	-11,18958664	-103,0525589	-944,4624634	-0,072256
Cloud_Mesh_Distance_MCA_R0_R2	-11,40908241	-102,8758087	-944,56073	0,010375
Cloud_Mesh_Distance_MCA_R0_R2	-11,12427711	-102,9117355	-944,466919	-0,067586
Cloud_Mesh_Distance_MCA_R0_R2	-12,12075615	-102,4669647	-944,4789429	0,041229
Cloud_Mesh_Distance_MCA_R0_R2	-12,16008568	-102,6504593	-944,5103149	0,059042
Cloud_Mesh_Distance_MCA_R0_R2	-12,03572083	-102,723526	-944,5596924	0,071403
Cloud_Mesh_Distance_MCA_R0_R2	-11,88132858	-102,6405869	-944,5749512	0,065489
Cloud_Mesh_Distance_MCA_R0_R2	-11,99846077	-102,5496521	-944,536377	0,057473
Cloud_Mesh_Distance_MCA_R0_R2	-11,75808525	-102,757164	-944,5978394	0,072437
Cloud_Mesh_Distance_MCA_R0_R2	-11,5558548	-102,6055069	-944,5802002	0,052724
Cloud_Mesh_Distance_MCA_R0_R2	-11,73441887	-102,5765152	-944,5795288	0,063996
Cloud_Mesh_Distance_MCA_R0_R2	-11,42510986	-102,7215881	-944,569458	0,029972
Cloud_Mesh_Distance_MCA_R0_R2	-11,25154114	-102,9032593	-944,5137329	-0,029947
Cloud_Mesh_Distance_MCA_R0_R2	-11,2827034	-102,7484512	-944,5378418	0,000032
Cloud_Mesh_Distance_MCA_R0_R2	-11,82754135	-102,4666519	-944,5512085	0,058758
Cloud_Mesh_Distance_MCA_R0_R2	-11,64532089	-102,4252472	-944,5593262	0,059362
Cloud_Mesh_Distance_MCA_R0_R2	-11,16789627	-102,6520462	-944,5148315	-0,011491
Cloud_Mesh_Distance_MCA_R0_R2	-11,9500885	-102,3670044	-944,5029297	0,041016
Cloud_Mesh_Distance_MCA_R0_R2	-12,07475662	-102,2895889	-944,4382935	0,020476
Cloud_Mesh_Distance_MCA_R0_R2	-11,38892555	-102,206398	-944,519104	0,055206
Cloud_Mesh_Distance_MCA_R0_R2	-11,44550133	-102,3958283	-944,5518799	0,05308
Cloud_Mesh_Distance_MCA_R0_R2	-11,31164742	-102,5626755	-944,5482178	0,024184
Cloud_Mesh_Distance_MCA_R0_R2	-11,10100555	-102,5076141	-944,5062866	-0,003507
Cloud_Mesh_Distance_MCA_R0_R2	-11,20894241	-102,3199768	-944,5198975	0,035487
Cloud_Mesh_Distance_MCA_R0_R2	-10,83917236	-102,4556122	-944,4517822	-0,04148
Cloud_Mesh_Distance_MCA_R0_R2	-10,79380417	-102,3413544	-944,4554443	-0,022915
Cloud_Mesh_Distance_MCA_R0_R2	-12,02881813	-102,1511688	-944,4019165	0,006509
Cloud_Mesh_Distance_MCA_R0_R2	-11,76161575	-102,0407105	-944,4342651	0,028115
Cloud_Mesh_Distance_MCA_R0_R2	-11,91705513	-102,1924973	-944,4540405	0,026522
Cloud_Mesh_Distance_MCA_R0_R2	-11,74487305	-102,2593689	-944,5125732	0,048738
Cloud_Mesh_Distance_MCA_R0_R2	-11,55473518	-102,2712479	-944,5322876	0,055961
Cloud_Mesh_Distance_MCA_R0_R2	-11,53753662	-102,0883942	-944,4852905	0,054909
Cloud_Mesh_Distance_MCA_R0_R2	-11,22880363	-102,1273651	-944,498169	0,055682
Cloud_Mesh_Distance_MCA_R0_R2	-11,34325981	-102,0554123	-944,484436	0,061011

Cloud_Mesh_Distance_MCA_R0_R2	-10,95684433	-102,3456802	-944,4829102	0,002478
Cloud_Mesh_Distance_MCA_R0_R2	-10,77234268	-102,1807556	-944,4602051	0,009672
Cloud_Mesh_Distance_MCA_R0_R2	-9,64214706	-102,3195343	-944,5847168	-0,066938
Cloud_Mesh_Distance_MCA_R0_R2	-9,65617371	-102,4187164	-944,545166	-0,076395
Cloud_Mesh_Distance_MCA_R0_R2	-11,73348236	-101,8629227	-944,3574829	0,014136
Cloud_Mesh_Distance_MCA_R0_R2	-11,02200699	-102,1144638	-944,4804688	0,045021
Cloud_Mesh_Distance_MCA_R0_R2	-10,60280991	-102,2019348	-944,4453735	-0,018105
Cloud_Mesh_Distance_MCA_R0_R2	-10,67445374	-102,3078156	-944,4408569	-0,030296
Cloud_Mesh_Distance_MCA_R0_R2	-10,53463364	-102,0298615	-944,4490356	0,015325
Cloud_Mesh_Distance_MCA_R0_R2	-10,32468796	-102,1504059	-944,4486084	-0,031391
Cloud_Mesh_Distance_MCA_R0_R2	-10,02720642	-102,2542572	-944,4707031	-0,069896
Cloud_Mesh_Distance_MCA_R0_R2	-10,14299774	-102,1958618	-944,4603272	-0,051562
Cloud_Mesh_Distance_MCA_R0_R2	-10,16983604	-102,0574417	-944,4741211	-0,01689
Cloud_Mesh_Distance_MCA_R0_R2	-9,89840889	-102,2881165	-944,4966431	-0,072037
Cloud_Mesh_Distance_MCA_R0_R2	-9,61071396	-102,190979	-944,62854	-0,031228
Cloud_Mesh_Distance_MCA_R0_R2	-11,75793648	-101,7453995	-944,2805176	-0,001079
Cloud_Mesh_Distance_MCA_R0_R2	-11,57045555	-101,8977432	-944,4103394	0,043744
Cloud_Mesh_Distance_MCA_R0_R2	-11,42361832	-101,781044	-944,3798828	0,051999
Cloud_Mesh_Distance_MCA_R0_R2	-11,16893768	-101,9546738	-944,458374	0,065021
Cloud_Mesh_Distance_MCA_R0_R2	-11,37952805	-101,9169083	-944,4404907	0,061134
Cloud_Mesh_Distance_MCA_R0_R2	-11,22173882	-101,7498245	-944,3883057	0,069451
Cloud_Mesh_Distance_MCA_R0_R2	-10,99594116	-101,9485397	-944,454773	0,061155
Cloud_Mesh_Distance_MCA_R0_R2	-10,80937958	-101,9446869	-944,4482422	0,055623
Cloud_Mesh_Distance_MCA_R0_R2	-10,29939651	-101,9753952	-944,4608765	0,009477
Cloud_Mesh_Distance_MCA_R0_R2	-9,92930603	-101,9438934	-944,5319214	0,003138
Cloud_Mesh_Distance_MCA_R0_R2	-9,982481	-102,1000443	-944,508667	-0,03316
Cloud_Mesh_Distance_MCA_R0_R2	-9,76876831	-102,1894226	-944,5625	-0,041513
Cloud_Mesh_Distance_MCA_R0_R2	-11,50045395	-101,5749893	-944,243042	0,021258
Cloud_Mesh_Distance_MCA_R0_R2	-11,57530212	-101,727356	-944,3222656	0,028043
Cloud_Mesh_Distance_MCA_R0_R2	-11,19371796	-101,5887909	-944,3146362	0,069132
Cloud_Mesh_Distance_MCA_R0_R2	-10,97679901	-101,7928543	-944,4155884	0,075807
Cloud_Mesh_Distance_MCA_R0_R2	-10,85094261	-101,6257248	-944,3652954	0,085419
Cloud_Mesh_Distance_MCA_R0_R2	-10,60361862	-101,8298111	-944,4310303	0,05861
Cloud_Mesh_Distance_MCA_R0_R2	-10,74834824	-101,740654	-944,4076538	0,076313
Cloud_Mesh_Distance_MCA_R0_R2	-10,69208336	-101,5976334	-944,3660278	0,086289
Cloud_Mesh_Distance_MCA_R0_R2	-10,36808777	-101,77742	-944,4371338	0,048875
Cloud_Mesh_Distance_MCA_R0_R2	-10,55018044	-101,6657105	-944,3979492	0,076348
Cloud_Mesh_Distance_MCA_R0_R2	-10,31158638	-101,5869446	-944,3989868	0,074021
Cloud_Mesh_Distance_MCA_R0_R2	-10,08900642	-101,8754501	-944,4910889	0,01829
Cloud_Mesh_Distance_MCA_R0_R2	-9,74718857	-101,6995392	-944,5526733	0,039576
Cloud_Mesh_Distance_MCA_R0_R2	-9,68470383	-101,7732391	-944,5913086	0,030891
Cloud_Mesh_Distance_MCA_R0_R2	-11,3670845	-101,6339035	-944,3110962	0,05131
Cloud_Mesh_Distance_MCA_R0_R2	-11,27255249	-101,5029373	-944,2485962	0,052854
Cloud_Mesh_Distance_MCA_R0_R2	-11,38531876	-101,5377731	-944,2470703	0,038654
Cloud_Mesh_Distance_MCA_R0_R2	-10,41910362	-101,6445465	-944,4025879	0,070819
Cloud_Mesh_Distance_MCA_R0_R2	-10,12281227	-101,6283646	-944,4404297	0,058831
Cloud_Mesh_Distance_MCA_R0_R2	-9,90327454	-101,7001953	-944,5081787	0,04278
Cloud_Mesh_Distance_MCA_R0_R2	-9,6205616	-101,6722488	-944,5837402	0,043877
Cloud_Mesh_Distance_MCA_R0_R2	-9,71648216	-101,5599899	-944,5122681	0,049405
Cloud_Mesh_Distance_MCA_R0_R2	-10,57005882	-101,5346069	-944,3519898	0,090483

Cloud_Mesh_Distance_MCA_R0_R2	-10,48116684	-101,4533997	-944,3272095	0,091796
Cloud_Mesh_Distance_MCA_R0_R2	-10,06235504	-101,4547806	-944,3890991	0,06813
Cloud_Mesh_Distance_MCA_R0_R2	-9,91275978	-101,5110626	-944,4431763	0,056312
Cloud_Mesh_Distance_MCA_R0_R2	-9,91531372	-101,3539429	-944,3666382	0,071704
Cloud_Mesh_Distance_MCA_R0_R2	-9,73482132	-101,3826828	-944,4191284	0,060674
Cloud_Mesh_Distance_MCA_R0_R2	-9,38243866	-101,2914352	-944,416687	0,038515
Cloud_Mesh_Distance_MCA_R0_R2	-9,52503967	-101,4159851	-944,4830322	0,050609
Cloud_Mesh_Distance_MCA_R0_R2	-9,76833534	-101,2370148	-944,3209229	0,062606
Cloud_Mesh_Distance_MCA_R0_R2	-9,55041504	-101,2403107	-944,3567505	0,049
Cloud_Mesh_Distance_MCA_R0_R2	-9,46079826	-101,1079025	-944,2564697	0,195987
Cloud_Mesh_Distance_MCA_R0_R2	-9,33719254	-101,164505	-944,3121338	0,168268
Cloud_Mesh_Distance_MCA_R0_R2	-11,66542816	-103,8834229	-944,3212891	-0,040439
Cloud_Mesh_Distance_MCA_R0_R2	-12,24848175	-103,7257996	-944,4119873	0,093252
Cloud_Mesh_Distance_MCA_R0_R2	-11,93292236	-103,7988434	-944,4190064	0,040731
Cloud_Mesh_Distance_MCA_R0_R2	-11,828228	-103,8342133	-944,3907471	0,011433
Cloud_Mesh_Distance_MCA_R0_R2	-11,72681618	-103,8244629	-944,3798828	-0,013402
Cloud_Mesh_Distance_MCA_R0_R2	-11,6714592	-103,7506867	-944,4099731	-0,011738
Cloud_Mesh_Distance_MCA_R0_R2	-11,6180191	-103,8112335	-944,3569336	-0,038865
Cloud_Mesh_Distance_MCA_R0_R2	-12,13093185	-103,6171112	-944,4842529	0,088108
Cloud_Mesh_Distance_MCA_R0_R2	-11,79326248	-103,7303696	-944,4452515	0,020453
Cloud_Mesh_Distance_MCA_R0_R2	-11,95721436	-103,6676025	-944,4829712	0,063892
Cloud_Mesh_Distance_MCA_R0_R2	-12,35137939	-103,4564056	-944,4674683	0,116923
Cloud_Mesh_Distance_MCA_R0_R2	-12,33488846	-103,3149338	-944,4940796	0,113777
Cloud_Mesh_Distance_MCA_R0_R2	-12,09637833	-103,3033829	-944,5614014	0,095131
Cloud_Mesh_Distance_MCA_R0_R2	-12,00409508	-103,3532944	-944,5666504	0,083797
Cloud_Mesh_Distance_MCA_R0_R2	-12,17835617	-103,423523	-944,526062	0,103339
Cloud_Mesh_Distance_MCA_R0_R2	-11,99039078	-103,5074234	-944,5355225	0,080727
Cloud_Mesh_Distance_MCA_R0_R2	-11,79772186	-103,5561905	-944,5162354	0,040291
Cloud_Mesh_Distance_MCA_R0_R2	-11,58228302	-103,7326431	-944,390686	-0,037194
Cloud_Mesh_Distance_MCA_R0_R2	-11,47286987	-103,6664124	-944,3792725	-0,068313
Cloud_Mesh_Distance_MCA_R0_R2	-11,63526154	-103,6421814	-944,451416	-0,01009
Cloud_Mesh_Distance_MCA_R0_R2	-11,50965309	-103,5445709	-944,4492798	-0,037605
Cloud_Mesh_Distance_MCA_R0_R2	-12,46860027	-103,0657883	-944,4359741	0,102203
Cloud_Mesh_Distance_MCA_R0_R2	-12,46691418	-103,2401962	-944,4403687	0,115012
Cloud_Mesh_Distance_MCA_R0_R2	-12,17479134	-103,2131424	-944,5525513	0,101846
Cloud_Mesh_Distance_MCA_R0_R2	-12,19049072	-103,0155258	-944,5482788	0,095028
Cloud_Mesh_Distance_MCA_R0_R2	-12,33421326	-103,1363144	-944,5020752	0,108442
Cloud_Mesh_Distance_MCA_R0_R2	-11,4489994	-103,3811417	-944,4812012	-0,038824
Cloud_Mesh_Distance_MCA_R0_R2	-11,61430931	-103,3616409	-944,5378418	0,012473
Cloud_Mesh_Distance_MCA_R0_R2	-11,6223526	-103,4873657	-944,505188	0,004631
Cloud_Mesh_Distance_MCA_R0_R2	-11,31143761	-103,2751923	-944,4558716	-0,071845
Cloud_Mesh_Distance_MCA_R0_R2	-11,23781395	-103,4009171	-944,3757324	-0,120981
Cloud_Mesh_Distance_MCA_R0_R2	-11,37053299	-103,5344849	-944,390686	-0,087965
Cloud_Mesh_Distance_MCA_R0_R2	-12,45951939	-102,8791809	-944,4179077	0,078631
Cloud_Mesh_Distance_MCA_R0_R2	-12,59030724	-102,9966965	-944,3545532	0,097874
Cloud_Mesh_Distance_MCA_R0_R2	-11,29021072	-103,1623993	-944,4775391	-0,060808
Cloud_Mesh_Distance_MCA_R0_R2	-11,19774055	-103,2176361	-944,4207153	-0,100733
Cloud_Mesh_Distance_MCA_R0_R2	-12,54244804	-102,6085815	-944,2978516	0,036676
Cloud_Mesh_Distance_MCA_R0_R2	-12,31445313	-102,7622986	-944,4708252	0,071583
Cloud_Mesh_Distance_MCA_R0_R2	-12,43472481	-102,687851	-944,3914795	0,051365

Cloud_Mesh_Distance_MCA_R0_R2	-12,18468094	-102,8323364	-944,5327759	0,076389
Cloud_Mesh_Distance_MCA_R0_R2	-12,33147049	-102,9498825	-944,4922485	0,094873
Cloud_Mesh_Distance_MCA_R0_R2	-11,03512573	-102,9888229	-944,4121094	-0,108064
Cloud_Mesh_Distance_MCA_R0_R2	-12,49723053	-102,41922	-944,2612305	0,004762
Cloud_Mesh_Distance_MCA_R0_R2	-12,39554024	-102,4976273	-944,3578491	0,021116
Cloud_Mesh_Distance_MCA_R0_R2	-11,13975716	-102,7847519	-944,4937744	-0,040479
Cloud_Mesh_Distance_MCA_R0_R2	-11,0090065	-102,8398514	-944,4381714	-0,085466
Cloud_Mesh_Distance_MCA_R0_R2	-12,28219032	-102,5748901	-944,4418945	0,045197
Cloud_Mesh_Distance_MCA_R0_R2	-10,95254707	-102,5119553	-944,4715576	-0,031619
Cloud_Mesh_Distance_MCA_R0_R2	-10,75344086	-102,5281677	-944,4206543	-0,077556
Cloud_Mesh_Distance_MCA_R0_R2	-10,84282875	-102,7717362	-944,3929443	-0,109518
Cloud_Mesh_Distance_MCA_R0_R2	-10,85420609	-102,5943832	-944,4348755	-0,067794
Cloud_Mesh_Distance_MCA_R0_R2	-11,00886536	-102,6641083	-944,4690552	-0,050218
Cloud_Mesh_Distance_MCA_R0_R2	-10,72596359	-102,642395	-944,3892212	-0,106884
Cloud_Mesh_Distance_MCA_R0_R2	-9,5763855	-102,8689346	-944,3250122	-0,007632
Cloud_Mesh_Distance_MCA_R0_R2	-9,6182003	-102,7217865	-944,414856	-0,057838
Cloud_Mesh_Distance_MCA_R0_R2	-9,43147659	-102,9416351	-944,3161011	0,022512
Cloud_Mesh_Distance_MCA_R0_R2	-9,43388748	-102,8172836	-944,4376831	-0,031704
Cloud_Mesh_Distance_MCA_R0_R2	-12,30677414	-102,1538849	-944,2658081	-0,030803
Cloud_Mesh_Distance_MCA_R0_R2	-12,44280434	-102,2395477	-944,2190552	-0,026503
Cloud_Mesh_Distance_MCA_R0_R2	-12,2371521	-102,3867188	-944,40448	0,017495
Cloud_Mesh_Distance_MCA_R0_R2	-12,34701347	-102,3125	-944,3158569	-0,008224
Cloud_Mesh_Distance_MCA_R0_R2	-12,17781067	-102,1914825	-944,3543091	-0,01084
Cloud_Mesh_Distance_MCA_R0_R2	-10,71715736	-102,4252777	-944,4316406	-0,053967
Cloud_Mesh_Distance_MCA_R0_R2	-10,47908783	-102,6303101	-944,340332	-0,125938
Cloud_Mesh_Distance_MCA_R0_R2	-10,62493896	-102,5322647	-944,392395	-0,09992
Cloud_Mesh_Distance_MCA_R0_R2	-10,60810471	-102,4021912	-944,4180908	-0,061076
Cloud_Mesh_Distance_MCA_R0_R2	-10,36240196	-102,553833	-944,3533936	-0,128714
Cloud_Mesh_Distance_MCA_R0_R2	-9,6883564	-102,5342026	-944,4835815	-0,092982
Cloud_Mesh_Distance_MCA_R0_R2	-9,82626534	-102,5650482	-944,4188843	-0,095068
Cloud_Mesh_Distance_MCA_R0_R2	-9,50450897	-102,6393204	-944,5205078	-0,072337
Cloud_Mesh_Distance_MCA_R0_R2	-12,19096375	-102,0454941	-944,2750244	-0,034799
Cloud_Mesh_Distance_MCA_R0_R2	-11,92711067	-102,0691299	-944,4021606	0,010122
Cloud_Mesh_Distance_MCA_R0_R2	-11,88058853	-101,9492874	-944,3589478	0,00507
Cloud_Mesh_Distance_MCA_R0_R2	-10,47501183	-102,4509888	-944,3918457	-0,09452
Cloud_Mesh_Distance_MCA_R0_R2	-10,39550972	-102,3064499	-944,4206543	-0,066233
Cloud_Mesh_Distance_MCA_R0_R2	-10,1073494	-102,3166275	-944,4400635	-0,078362
Cloud_Mesh_Distance_MCA_R0_R2	-10,23548317	-102,3091278	-944,4260254	-0,078301
Cloud_Mesh_Distance_MCA_R0_R2	-10,30484581	-102,4407349	-944,3875122	-0,107551
Cloud_Mesh_Distance_MCA_R0_R2	-9,95656013	-102,5247574	-944,3998413	-0,116795
Cloud_Mesh_Distance_MCA_R0_R2	-9,54169846	-102,4547501	-944,5881348	-0,071282
Cloud_Mesh_Distance_MCA_R0_R2	-12,10433006	-101,922081	-944,246582	-0,042415
Cloud_Mesh_Distance_MCA_R0_R2	-12,04058838	-102,0259171	-944,3376465	-0,012483
Cloud_Mesh_Distance_MCA_R0_R2	-11,96710587	-101,8813858	-944,2872315	-0,017542
Cloud_Mesh_Distance_MCA_R0_R2	-10,48165894	-102,1963654	-944,4398193	-0,03031
Cloud_Mesh_Distance_MCA_R0_R2	-10,54130936	-102,3084793	-944,427002	-0,048509
Cloud_Mesh_Distance_MCA_R0_R2	-9,99616432	-102,3791733	-944,4431763	-0,095665
Cloud_Mesh_Distance_MCA_R0_R2	-9,85357094	-102,4427567	-944,4610596	-0,085709
Cloud_Mesh_Distance_MCA_R0_R2	-9,75235939	-102,3653946	-944,5241089	-0,073152
Cloud_Mesh_Distance_MCA_R0_R2	-11,84787369	-101,8383637	-944,3074951	-0,004907

Cloud_Mesh_Distance_MCA_R0_R2	-11,9007206	-101,7655487	-944,2392578	-0,022649
Cloud_Mesh_Distance_MCA_R0_R2	-11,81882477	-101,6716385	-944,2041016	-0,026371
Cloud_Mesh_Distance_MCA_R0_R2	-11,67182159	-101,6266861	-944,2265015	-0,001378
Cloud_Mesh_Distance_MCA_R0_R2	-11,60377312	-101,5010757	-944,1491089	-0,008707
Cloud_Mesh_Distance_MCA_R0_R2	-10,88746643	-101,4975739	-944,303833	0,088385
Cloud_Mesh_Distance_MCA_R0_R2	-11,03457451	-101,6160049	-944,3468018	0,079321
Cloud_Mesh_Distance_MCA_R0_R2	-10,76229286	-101,5259247	-944,3309326	0,09162
Cloud_Mesh_Distance_MCA_R0_R2	-11,40935707	-101,4503784	-944,1726074	0,02189
Cloud_Mesh_Distance_MCA_R0_R2	-11,07471657	-101,4620667	-944,2584229	0,07224
Cloud_Mesh_Distance_MCA_R0_R2	-11,03590775	-101,3279953	-944,1741333	0,067822
Cloud_Mesh_Distance_MCA_R0_R2	-10,8889122	-101,3604965	-944,223877	0,083366
Cloud_Mesh_Distance_MCA_R0_R2	-10,71387291	-101,4137268	-944,2799683	0,092292
Cloud_Mesh_Distance_MCA_R0_R2	-10,66529465	-101,5019455	-944,3295898	0,092145
Cloud_Mesh_Distance_MCA_R0_R2	-10,58493805	-101,4435425	-944,3112183	0,092636
Cloud_Mesh_Distance_MCA_R0_R2	-10,44190979	-101,551384	-944,3708496	0,082576
Cloud_Mesh_Distance_MCA_R0_R2	-10,31504631	-101,4262466	-944,3361206	0,085821
Cloud_Mesh_Distance_MCA_R0_R2	-10,1951828	-101,5011978	-944,3850708	0,071313
Cloud_Mesh_Distance_MCA_R0_R2	-11,2125206	-101,3666611	-944,1604614	0,044916
Cloud_Mesh_Distance_MCA_R0_R2	-11,24700928	-101,2687073	-944,0628662	0,022136
Cloud_Mesh_Distance_MCA_R0_R2	-10,91112518	-101,2230072	-944,1179199	0,074271
Cloud_Mesh_Distance_MCA_R0_R2	-11,09979439	-101,2474213	-944,0894775	0,048692
Cloud_Mesh_Distance_MCA_R0_R2	-10,71501541	-101,2599564	-944,18573	0,091649
Cloud_Mesh_Distance_MCA_R0_R2	-10,05295181	-101,3244553	-944,3254395	0,078286
Cloud_Mesh_Distance_MCA_R0_R2	-10,15863419	-101,4076386	-944,3508911	0,07939
Cloud_Mesh_Distance_MCA_R0_R2	-10,27347183	-101,2777939	-944,2644043	0,090616
Cloud_Mesh_Distance_MCA_R0_R2	-10,8910923	-101,1172714	-944,0288696	0,063233
Cloud_Mesh_Distance_MCA_R0_R2	-10,74697876	-101,1257782	-944,0753784	0,082096
Cloud_Mesh_Distance_MCA_R0_R2	-10,49245453	-101,3182678	-944,2559204	0,096785
Cloud_Mesh_Distance_MCA_R0_R2	-10,31763077	-101,1547012	-944,178772	0,100679
Cloud_Mesh_Distance_MCA_R0_R2	-10,17141724	-101,3217468	-944,3049316	0,086358
Cloud_Mesh_Distance_MCA_R0_R2	-10,53960609	-101,1401901	-944,1306152	0,098137
Cloud_Mesh_Distance_MCA_R0_R2	-10,08400917	-101,1782074	-944,2311401	0,08686
Cloud_Mesh_Distance_MCA_R0_R2	-9,8355484	-101,1096115	-944,2186279	0,072375
Cloud_Mesh_Distance_MCA_R0_R2	-9,93105698	-101,2361984	-944,2935791	0,073975
Cloud_Mesh_Distance_MCA_R0_R2	-9,67552948	-101,0098724	-944,1553955	0,063577
Cloud_Mesh_Distance_MCA_R0_R2	-9,63139343	-101,117775	-944,2515869	0,062924
Cloud_Mesh_Distance_MCA_R0_R2	-9,80840874	-100,9924545	-944,1274414	0,072619
Cloud_Mesh_Distance_MCA_R0_R2	-12,4431591	-103,9129791	-944,2224731	0,076335
Cloud_Mesh_Distance_MCA_R0_R2	-12,1055851	-103,9734573	-944,2765503	0,025586
Cloud_Mesh_Distance_MCA_R0_R2	-11,90044975	-103,8856354	-944,3609009	0,01576
Cloud_Mesh_Distance_MCA_R0_R2	-11,78208351	-103,919838	-944,3210449	-0,019911
Cloud_Mesh_Distance_MCA_R0_R2	-11,54814529	-103,8842468	-944,274353	-0,080354
Cloud_Mesh_Distance_MCA_R0_R2	-12,40720749	-103,6865311	-944,3734741	0,110233
Cloud_Mesh_Distance_MCA_R0_R2	-12,35091209	-103,8229446	-944,3241577	0,084672
Cloud_Mesh_Distance_MCA_R0_R2	-12,1863575	-103,8582764	-944,3526001	0,06608
Cloud_Mesh_Distance_MCA_R0_R2	-12,02512169	-103,8817062	-944,3618774	0,035186
Cloud_Mesh_Distance_MCA_R0_R2	-12,08358574	-103,7635117	-944,4277344	0,06886
Cloud_Mesh_Distance_MCA_R0_R2	-11,43499374	-103,8304901	-944,2570801	-0,112983
Cloud_Mesh_Distance_MCA_R0_R2	-12,713871	-103,4989471	-944,2558594	0,134355
Cloud_Mesh_Distance_MCA_R0_R2	-12,45072079	-103,544075	-944,4042358	0,120477

Cloud_Mesh_Distance_MCA_R0_R2	-12,59547901	-103,4785004	-944,3411255	0,131284
Cloud_Mesh_Distance_MCA_R0_R2	-12,54976082	-103,6391525	-944,3229981	0,120223
Cloud_Mesh_Distance_MCA_R0_R2	-12,30021477	-103,5799179	-944,4533691	0,108115
Cloud_Mesh_Distance_MCA_R0_R2	-11,3453331	-103,7828217	-944,2384033	-0,13784
Cloud_Mesh_Distance_MCA_R0_R2	-11,34851456	-103,8700485	-944,1730957	-0,150839
Cloud_Mesh_Distance_MCA_R0_R2	-12,5711441	-103,3256302	-944,3768311	0,128354
Cloud_Mesh_Distance_MCA_R0_R2	-12,58902168	-103,1783524	-944,3694458	0,119123
Cloud_Mesh_Distance_MCA_R0_R2	-12,70986366	-103,3141098	-944,2819214	0,126037
Cloud_Mesh_Distance_MCA_R0_R2	-12,46704578	-103,3975372	-944,4264526	0,121067
Cloud_Mesh_Distance_MCA_R0_R2	-11,51127625	-103,7809677	-944,3319092	-0,07222
Cloud_Mesh_Distance_MCA_R0_R2	-11,26737595	-103,5353241	-944,3338623	-0,132959
Cloud_Mesh_Distance_MCA_R0_R2	-11,41173744	-103,7460632	-944,302124	-0,105005
Cloud_Mesh_Distance_MCA_R0_R2	-11,16911125	-103,5420761	-944,2710571	-0,169407
Cloud_Mesh_Distance_MCA_R0_R2	-12,80918884	-103,1330032	-944,1907959	0,109999
Cloud_Mesh_Distance_MCA_R0_R2	-11,10067749	-103,2876282	-944,3460693	-0,147047
Cloud_Mesh_Distance_MCA_R0_R2	-10,95101357	-103,2537689	-944,2759399	-0,190219
Cloud_Mesh_Distance_MCA_R0_R2	-10,89479256	-103,3933182	-944,1762695	-0,221835
Cloud_Mesh_Distance_MCA_R0_R2	-12,71663094	-102,9336014	-944,2449341	0,080202
Cloud_Mesh_Distance_MCA_R0_R2	-12,68951702	-103,1125031	-944,293457	0,107538
Cloud_Mesh_Distance_MCA_R0_R2	-12,57699013	-102,804718	-944,3283081	0,069796
Cloud_Mesh_Distance_MCA_R0_R2	-11,06535339	-103,1437836	-944,380127	-0,133223
Cloud_Mesh_Distance_MCA_R0_R2	-10,91534233	-103,0737152	-944,3296509	-0,16031
Cloud_Mesh_Distance_MCA_R0_R2	-10,79891014	-103,1678467	-944,2347412	-0,202877
Cloud_Mesh_Distance_MCA_R0_R2	-12,67690086	-102,718483	-944,2250366	0,045279
Cloud_Mesh_Distance_MCA_R0_R2	-10,64204979	-103,1514206	-944,1715698	-0,21295
Cloud_Mesh_Distance_MCA_R0_R2	-9,28258514	-103,0543518	-944,1852417	0,060107
Cloud_Mesh_Distance_MCA_R0_R2	-12,63642406	-102,5269699	-944,1932373	0,022122
Cloud_Mesh_Distance_MCA_R0_R2	-10,90680695	-102,9273758	-944,3748779	-0,132411
Cloud_Mesh_Distance_MCA_R0_R2	-10,73249054	-102,970108	-944,2886963	-0,177038
Cloud_Mesh_Distance_MCA_R0_R2	-9,83759308	-102,9146576	-944,2145996	-0,020785
Cloud_Mesh_Distance_MCA_R0_R2	-9,68245697	-102,9359512	-944,2336426	-0,002534
Cloud_Mesh_Distance_MCA_R0_R2	-9,4260006	-103,0232468	-944,2119141	0,07344
Cloud_Mesh_Distance_MCA_R0_R2	-9,54461288	-102,9781342	-944,2335205	0,04953
Cloud_Mesh_Distance_MCA_R0_R2	-9,32253265	-102,9988556	-944,2866821	0,028616
Cloud_Mesh_Distance_MCA_R0_R2	-10,66825867	-102,7758484	-944,3341675	-0,147451
Cloud_Mesh_Distance_MCA_R0_R2	-10,54470253	-102,7459717	-944,3120117	-0,151291
Cloud_Mesh_Distance_MCA_R0_R2	-10,55392456	-102,8742065	-944,2653809	-0,183097
Cloud_Mesh_Distance_MCA_R0_R2	-10,06297684	-102,7814102	-944,2628784	-0,131636
Cloud_Mesh_Distance_MCA_R0_R2	-12,58767319	-102,3359604	-944,1501465	-0,011661
Cloud_Mesh_Distance_MCA_R0_R2	-12,51350975	-102,1263962	-944,0992432	-0,041269
Cloud_Mesh_Distance_MCA_R0_R2	-12,68152809	-102,2620087	-944,0206299	-0,016312
Cloud_Mesh_Distance_MCA_R0_R2	-12,38096523	-102,1046829	-944,1901245	-0,047937
Cloud_Mesh_Distance_MCA_R0_R2	-10,60910606	-102,6634064	-944,354126	-0,130146
Cloud_Mesh_Distance_MCA_R0_R2	-10,1809864	-102,7220001	-944,2849121	-0,153105
Cloud_Mesh_Distance_MCA_R0_R2	-9,93111038	-102,7844467	-944,2801514	-0,102562
Cloud_Mesh_Distance_MCA_R0_R2	-9,75371361	-102,7954941	-944,3170776	-0,059945
Cloud_Mesh_Distance_MCA_R0_R2	-9,75316429	-102,6584778	-944,3978272	-0,086309
Cloud_Mesh_Distance_MCA_R0_R2	-12,64515781	-102,0561447	-943,9273071	-0,036483
Cloud_Mesh_Distance_MCA_R0_R2	-12,54867363	-101,875351	-943,887146	-0,06657
Cloud_Mesh_Distance_MCA_R0_R2	-12,47321129	-101,942276	-944,0106812	-0,066564

Cloud_Mesh_Distance_MCA_R0_R2	-12,28918266	-102,044899	-944,2158814	-0,044758
Cloud_Mesh_Distance_MCA_R0_R2	-12,34329224	-101,9725723	-944,133606	-0,061253
Cloud_Mesh_Distance_MCA_R0_R2	-12,22105217	-101,9598007	-944,2059937	-0,048625
Cloud_Mesh_Distance_MCA_R0_R2	-10,05285072	-102,6451263	-944,3313599	-0,131903
Cloud_Mesh_Distance_MCA_R0_R2	-10,22601318	-102,5808182	-944,3414307	-0,141221
Cloud_Mesh_Distance_MCA_R0_R2	-10,12683296	-102,4551163	-944,3956299	-0,107277
Cloud_Mesh_Distance_MCA_R0_R2	-9,87931061	-102,6632385	-944,3565064	-0,097388
Cloud_Mesh_Distance_MCA_R0_R2	-12,43317604	-101,8223801	-943,9492798	-0,084507
Cloud_Mesh_Distance_MCA_R0_R2	-12,33000374	-101,8289948	-944,0379639	-0,084098
Cloud_Mesh_Distance_MCA_R0_R2	-12,19693184	-101,7015686	-944,0230713	-0,088818
Cloud_Mesh_Distance_MCA_R0_R2	-12,02709389	-101,8096085	-944,2112427	-0,043994
Cloud_Mesh_Distance_MCA_R0_R2	-12,17506027	-101,8320084	-944,1451416	-0,064784
Cloud_Mesh_Distance_MCA_R0_R2	-11,98612785	-101,6702728	-944,1229248	-0,060375
Cloud_Mesh_Distance_MCA_R0_R2	-12,24887466	-101,6265488	-943,9118042	-0,1054
Cloud_Mesh_Distance_MCA_R0_R2	-11,89051056	-101,5123215	-944,0249634	-0,073744
Cloud_Mesh_Distance_MCA_R0_R2	-11,82537079	-101,4189529	-943,9597168	-0,07932
Cloud_Mesh_Distance_MCA_R0_R2	-11,78147888	-101,5675507	-944,1334229	-0,04164
Cloud_Mesh_Distance_MCA_R0_R2	-11,72525215	-101,4491577	-944,0466309	-0,051091
Cloud_Mesh_Distance_MCA_R0_R2	-11,71641159	-101,3617935	-943,9541626	-0,06994
Cloud_Mesh_Distance_MCA_R0_R2	-11,54616737	-101,3633652	-944,0404663	-0,025153
Cloud_Mesh_Distance_MCA_R0_R2	-11,38466454	-101,2335281	-943,9696045	-0,014855
Cloud_Mesh_Distance_MCA_R0_R2	-11,35807228	-101,332695	-944,0859375	0,011914
Cloud_Mesh_Distance_MCA_R0_R2	-11,27932739	-101,1284638	-943,8913574	-0,010712
Cloud_Mesh_Distance_MCA_R0_R2	-11,11707306	-101,0779495	-943,9042969	0,021906
Cloud_Mesh_Distance_MCA_R0_R2	-11,02223206	-101,1433945	-944,013916	0,050851
Cloud_Mesh_Distance_MCA_R0_R2	-11,18875313	-101,1791534	-943,991333	0,024353
Cloud_Mesh_Distance_MCA_R0_R2	-10,97143173	-101,0596848	-943,9417114	0,044933
Cloud_Mesh_Distance_MCA_R0_R2	-10,8279171	-101,0296097	-943,9597168	0,062903
Cloud_Mesh_Distance_MCA_R0_R2	-10,59832954	-100,9944229	-943,9923706	0,087912
Cloud_Mesh_Distance_MCA_R0_R2	-10,36846161	-101,0128479	-944,0601807	0,09677
Cloud_Mesh_Distance_MCA_R0_R2	-10,15491295	-101,0233231	-944,1056519	0,094077
Cloud_Mesh_Distance_MCA_R0_R2	-10,3986721	-100,8899994	-943,9387207	0,096252
Cloud_Mesh_Distance_MCA_R0_R2	-10,00535011	-100,9030457	-944,0236206	0,092149
Cloud_Mesh_Distance_MCA_R0_R2	-9,95636749	-101,0182953	-944,1312256	0,085556
Cloud_Mesh_Distance_MCA_R0_R2	-10,20542526	-100,8969727	-943,9859619	0,100472
Cloud_Mesh_Distance_MCA_R0_R2	-9,85889053	-100,9244995	-944,0612183	0,101337
Cloud_Mesh_Distance_MCA_R0_R2	-10,55890274	-100,8674851	-943,8696289	0,080083
Cloud_Mesh_Distance_MCA_R0_R2	-10,50033951	-100,7365112	-943,7244873	0,071536
Cloud_Mesh_Distance_MCA_R0_R2	-10,40860748	-100,7812119	-943,8132324	0,09085
Cloud_Mesh_Distance_MCA_R0_R2	-10,37797165	-100,6867142	-943,699707	0,079285
Cloud_Mesh_Distance_MCA_R0_R2	-10,05542564	-100,7721176	-943,8847046	0,098979
Cloud_Mesh_Distance_MCA_R0_R2	-10,25637054	-100,8054581	-943,8817139	0,101886
Cloud_Mesh_Distance_MCA_R0_R2	-10,2186203	-100,7031021	-943,7686157	0,092329
Cloud_Mesh_Distance_MCA_R0_R2	-12,52792454	-103,998642	-944,1026001	0,058563
Cloud_Mesh_Distance_MCA_R0_R2	-12,3561306	-104,0373077	-944,1428223	0,032101
Cloud_Mesh_Distance_MCA_R0_R2	-12,42500687	-104,1217575	-944,012207	0,000721
Cloud_Mesh_Distance_MCA_R0_R2	-12,24550056	-104,1468048	-944,0456543	-0,026988
Cloud_Mesh_Distance_MCA_R0_R2	-12,0706501	-104,1584473	-944,067688	-0,051349
Cloud_Mesh_Distance_MCA_R0_R2	-11,91160583	-104,1523666	-944,081665	-0,074073
Cloud_Mesh_Distance_MCA_R0_R2	-11,88115311	-104,0772018	-944,1780396	-0,04674

Cloud_Mesh_Distance_MCA_R0_R2	-11,77356339	-104,1251221	-944,0992432	-0,087938
Cloud_Mesh_Distance_MCA_R0_R2	-12,59856224	-103,8631744	-944,1757813	0,102039
Cloud_Mesh_Distance_MCA_R0_R2	-12,73924255	-103,796196	-944,121582	0,121423
Cloud_Mesh_Distance_MCA_R0_R2	-12,18225479	-104,0619659	-944,1712647	0,000203
Cloud_Mesh_Distance_MCA_R0_R2	-12,27639389	-103,9493179	-944,2572632	0,052638
Cloud_Mesh_Distance_MCA_R0_R2	-12,01506805	-104,0728455	-944,184082	-0,022446
Cloud_Mesh_Distance_MCA_R0_R2	-11,77301025	-104,0308456	-944,2131348	-0,058719
Cloud_Mesh_Distance_MCA_R0_R2	-11,92743683	-103,983635	-944,2797852	-0,009628
Cloud_Mesh_Distance_MCA_R0_R2	-11,62096405	-104,0812454	-944,1063843	-0,111204
Cloud_Mesh_Distance_MCA_R0_R2	-11,65037918	-103,968132	-944,2418823	-0,068375
Cloud_Mesh_Distance_MCA_R0_R2	-12,86079216	-103,7154388	-944,0595093	0,128092
Cloud_Mesh_Distance_MCA_R0_R2	-12,64326572	-103,7212219	-944,2310181	0,123822
Cloud_Mesh_Distance_MCA_R0_R2	-12,50578022	-103,7785111	-944,2814941	0,10735
Cloud_Mesh_Distance_MCA_R0_R2	-11,51492882	-103,9844589	-944,1658325	-0,116794
Cloud_Mesh_Distance_MCA_R0_R2	-11,38075638	-103,968895	-944,102356	-0,15935
Cloud_Mesh_Distance_MCA_R0_R2	-11,44536018	-103,9097519	-944,199646	-0,121604
Cloud_Mesh_Distance_MCA_R0_R2	-12,88703537	-103,5578384	-944,0891724	0,140629
Cloud_Mesh_Distance_MCA_R0_R2	-12,98533726	-103,502388	-943,9905396	0,133069
Cloud_Mesh_Distance_MCA_R0_R2	-12,76655388	-103,6252136	-944,1793823	0,137874
Cloud_Mesh_Distance_MCA_R0_R2	-12,65889645	-103,597168	-944,2697144	0,13314
Cloud_Mesh_Distance_MCA_R0_R2	-12,81025314	-103,4530563	-944,1832886	0,133461
Cloud_Mesh_Distance_MCA_R0_R2	-12,8340168	-103,3067856	-944,1742554	0,125479
Cloud_Mesh_Distance_MCA_R0_R2	-12,91018105	-103,3921356	-944,0917969	0,13259
Cloud_Mesh_Distance_MCA_R0_R2	-11,29546738	-103,6620407	-944,2843628	-0,140084
Cloud_Mesh_Distance_MCA_R0_R2	-11,10340881	-103,6696701	-944,1507568	-0,206106
Cloud_Mesh_Distance_MCA_R0_R2	-11,23785782	-103,7953491	-944,1581421	-0,183527
Cloud_Mesh_Distance_MCA_R0_R2	-11,0678196	-103,547142	-944,2018433	-0,205535
Cloud_Mesh_Distance_MCA_R0_R2	-12,90244198	-103,2359467	-944,1016846	0,11711
Cloud_Mesh_Distance_MCA_R0_R2	-13,00256348	-103,2878647	-943,9780273	0,109601
Cloud_Mesh_Distance_MCA_R0_R2	-13,03939629	-103,0923691	-943,8950806	0,087306
Cloud_Mesh_Distance_MCA_R0_R2	-11,05186081	-103,4251175	-944,2554321	-0,190534
Cloud_Mesh_Distance_MCA_R0_R2	-10,80134773	-103,4730759	-944,0751343	-0,231279
Cloud_Mesh_Distance_MCA_R0_R2	-12,92812347	-103,0629578	-944,0492554	0,089255
Cloud_Mesh_Distance_MCA_R0_R2	-12,83077812	-102,9626465	-944,1417847	0,085417
Cloud_Mesh_Distance_MCA_R0_R2	-12,79339123	-102,809967	-944,1415405	0,061991
Cloud_Mesh_Distance_MCA_R0_R2	-10,83058167	-103,2899628	-944,192627	-0,218816
Cloud_Mesh_Distance_MCA_R0_R2	-10,78688431	-103,3651199	-944,1292725	-0,226862
Cloud_Mesh_Distance_MCA_R0_R2	-10,68668365	-103,3946915	-944,0593872	-0,220724
Cloud_Mesh_Distance_MCA_R0_R2	-12,88269424	-102,8837967	-944,0623169	0,065406
Cloud_Mesh_Distance_MCA_R0_R2	-12,75906754	-102,6329575	-944,1174927	0,033485
Cloud_Mesh_Distance_MCA_R0_R2	-12,86438084	-102,7341309	-944,0345459	0,048943
Cloud_Mesh_Distance_MCA_R0_R2	-10,7218132	-103,276329	-944,144104	-0,219942
Cloud_Mesh_Distance_MCA_R0_R2	-10,58689117	-103,2905579	-944,0739136	-0,204192
Cloud_Mesh_Distance_MCA_R0_R2	-9,97788048	-103,1686554	-944,0046997	-0,017684
Cloud_Mesh_Distance_MCA_R0_R2	-9,58627129	-103,0851898	-944,0933228	0,095102
Cloud_Mesh_Distance_MCA_R0_R2	-9,45693588	-103,0897522	-944,0903931	0,115705
Cloud_Mesh_Distance_MCA_R0_R2	-9,32450104	-103,106575	-944,03302	0,125587
Cloud_Mesh_Distance_MCA_R0_R2	-9,15011406	-103,069458	-944,1039429	0,040166
Cloud_Mesh_Distance_MCA_R0_R2	-12,83217144	-102,5651856	-944,0048828	0,030398
Cloud_Mesh_Distance_MCA_R0_R2	-12,91766548	-102,6671066	-943,9348145	0,040587

Cloud_Mesh_Distance_MCA_R0_R2	-10,53486633	-103,0281677	-944,1925659	-0,200317
Cloud_Mesh_Distance_MCA_R0_R2	-10,37064743	-103,0638504	-944,1306152	-0,173896
Cloud_Mesh_Distance_MCA_R0_R2	-10,0609417	-103,0998688	-944,065979	-0,067407
Cloud_Mesh_Distance_MCA_R0_R2	-10,0997448	-102,987854	-944,1428223	-0,097478
Cloud_Mesh_Distance_MCA_R0_R2	-9,90752983	-103,0504227	-944,1036377	-0,017097
Cloud_Mesh_Distance_MCA_R0_R2	-9,75443649	-103,0072784	-944,1566773	0,013021
Cloud_Mesh_Distance_MCA_R0_R2	-9,7162838	-103,0913086	-944,0767212	0,069604
Cloud_Mesh_Distance_MCA_R0_R2	-9,63354492	-103,0269241	-944,1577759	0,046757
Cloud_Mesh_Distance_MCA_R0_R2	-9,52750015	-103,0441284	-944,1567993	0,08374
Cloud_Mesh_Distance_MCA_R0_R2	-12,80400372	-102,3948898	-943,9538574	0,013052
Cloud_Mesh_Distance_MCA_R0_R2	-12,7217989	-102,4507828	-944,0783691	0,007752
Cloud_Mesh_Distance_MCA_R0_R2	-10,26863098	-102,7966614	-944,2515869	-0,160919
Cloud_Mesh_Distance_MCA_R0_R2	-10,42980576	-102,9385071	-944,2076416	-0,178793
Cloud_Mesh_Distance_MCA_R0_R2	-10,27658844	-102,9315414	-944,1858521	-0,154575
Cloud_Mesh_Distance_MCA_R0_R2	-10,14852142	-102,8536835	-944,2210083	-0,143124
Cloud_Mesh_Distance_MCA_R0_R2	-9,99824905	-102,8976975	-944,2036133	-0,089968
Cloud_Mesh_Distance_MCA_R0_R2	-9,07909012	-103,048111	-943,9755859	0,038918
Cloud_Mesh_Distance_MCA_R0_R2	-12,78139305	-102,2270813	-943,881958	-0,005543
Cloud_Mesh_Distance_MCA_R0_R2	-12,87849808	-102,3600159	-943,8280029	0,007995
Cloud_Mesh_Distance_MCA_R0_R2	-10,41117859	-102,798233	-944,2669678	-0,170069
Cloud_Mesh_Distance_MCA_R0_R2	-10,32223129	-102,6789475	-944,3053589	-0,157942
Cloud_Mesh_Distance_MCA_R0_R2	-8,85087204	-102,8943634	-943,9052124	-0,086782
Cloud_Mesh_Distance_MCA_R0_R2	-8,75953102	-102,7705917	-943,8359375	-0,14506
Cloud_Mesh_Distance_MCA_R0_R2	-12,79013824	-102,0775833	-943,7622681	-0,013502
Cloud_Mesh_Distance_MCA_R0_R2	-12,67602539	-101,91996	-943,7839356	-0,036028
Cloud_Mesh_Distance_MCA_R0_R2	-12,4624691	-101,7527084	-943,8598633	-0,091919
Cloud_Mesh_Distance_MCA_R0_R2	-8,66852951	-102,6177521	-943,7694702	-0,193712
Cloud_Mesh_Distance_MCA_R0_R2	-12,48890781	-101,6820831	-943,7600098	-0,086658
Cloud_Mesh_Distance_MCA_R0_R2	-12,34200668	-101,7264099	-943,9390259	-0,098817
Cloud_Mesh_Distance_MCA_R0_R2	-12,3709774	-101,6440888	-943,8300171	-0,106568
Cloud_Mesh_Distance_MCA_R0_R2	-12,08339882	-101,5514679	-943,9484863	-0,105603
Cloud_Mesh_Distance_MCA_R0_R2	-12,24352074	-101,5221176	-943,7949219	-0,119257
Cloud_Mesh_Distance_MCA_R0_R2	-11,94309235	-101,446228	-943,9202881	-0,09962
Cloud_Mesh_Distance_MCA_R0_R2	-11,85680389	-101,3460922	-943,8483276	-0,107097
Cloud_Mesh_Distance_MCA_R0_R2	-12,05611038	-101,4199905	-943,8079834	-0,120987
Cloud_Mesh_Distance_MCA_R0_R2	-11,63300133	-101,2545395	-943,8646851	-0,071132
Cloud_Mesh_Distance_MCA_R0_R2	-11,63660812	-101,1528854	-943,7078857	-0,103779
Cloud_Mesh_Distance_MCA_R0_R2	-11,44774246	-101,1405029	-943,8146362	-0,061105
Cloud_Mesh_Distance_MCA_R0_R2	-11,74120331	-101,1535492	-943,6287231	-0,122114
Cloud_Mesh_Distance_MCA_R0_R2	-11,6689682	-101,0913162	-943,5680542	-0,118454
Cloud_Mesh_Distance_MCA_R0_R2	-11,38187981	-100,9721832	-943,5760498	-0,078486
Cloud_Mesh_Distance_MCA_R0_R2	-11,48983955	-101,0650635	-943,6643677	-0,084516
Cloud_Mesh_Distance_MCA_R0_R2	-11,26456451	-101,0303879	-943,7592163	-0,033047
Cloud_Mesh_Distance_MCA_R0_R2	-10,98108482	-100,9579086	-943,8114014	0,01761
Cloud_Mesh_Distance_MCA_R0_R2	-10,73869133	-100,9200897	-943,8677368	0,057141
Cloud_Mesh_Distance_MCA_R0_R2	-10,79799271	-100,8456726	-943,7454834	0,035653
Cloud_Mesh_Distance_MCA_R0_R2	-10,65834808	-100,8367157	-943,796936	0,062616
Cloud_Mesh_Distance_MCA_R0_R2	-11,05311203	-100,8233948	-943,5473633	-0,036851
Cloud_Mesh_Distance_MCA_R0_R2	-10,93937111	-100,7678757	-943,5287476	-0,021503
Cloud_Mesh_Distance_MCA_R0_R2	-10,95554924	-100,8413315	-943,6502686	-0,002809

Cloud_Mesh_Distance_MCA_R0_R2	-10,55369377	-100,7959061	-943,7842407	0,073848
Cloud_Mesh_Distance_MCA_R0_R2	-10,80901146	-100,7686157	-943,6187134	0,012884
Cloud_Mesh_Distance_MCA_R0_R2	-10,69158745	-100,6918182	-943,5565186	0,02792
Cloud_Mesh_Distance_MCA_R0_R2	-10,64657021	-100,7613525	-943,6954346	0,047647
Cloud_Mesh_Distance_MCA_R0_R2	-10,37217331	-100,6230774	-943,6045532	0,091457
Cloud_Mesh_Distance_MCA_R0_R2	-12,75253677	-104,0029907	-943,951416	0,083055
Cloud_Mesh_Distance_MCA_R0_R2	-12,61263466	-104,0845413	-943,9593506	0,039216
Cloud_Mesh_Distance_MCA_R0_R2	-11,9396019	-104,2376862	-943,9473267	-0,107315
Cloud_Mesh_Distance_MCA_R0_R2	-12,11723328	-104,2404862	-943,9290772	-0,086757
Cloud_Mesh_Distance_MCA_R0_R2	-11,78491974	-104,2117157	-943,973999	-0,121012
Cloud_Mesh_Distance_MCA_R0_R2	-11,66893768	-104,1710129	-944,0028687	-0,136319
Cloud_Mesh_Distance_MCA_R0_R2	-12,68550301	-103,9414368	-944,0566406	0,093806
Cloud_Mesh_Distance_MCA_R0_R2	-11,44867897	-104,0684662	-944,0335083	-0,158908
Cloud_Mesh_Distance_MCA_R0_R2	-12,94732094	-103,7694473	-943,9451294	0,12849
Cloud_Mesh_Distance_MCA_R0_R2	-12,83230209	-103,8794403	-943,9902954	0,111998
Cloud_Mesh_Distance_MCA_R0_R2	-11,37829971	-104,1348953	-943,9039307	-0,187943
Cloud_Mesh_Distance_MCA_R0_R2	-11,28240585	-104,0422897	-943,9541626	-0,198431
Cloud_Mesh_Distance_MCA_R0_R2	-11,24950409	-103,9185257	-944,0609741	-0,194535
Cloud_Mesh_Distance_MCA_R0_R2	-12,94721222	-103,6410523	-944,0009155	0,136471
Cloud_Mesh_Distance_MCA_R0_R2	-13,02775764	-103,6482849	-943,8990479	0,133896
Cloud_Mesh_Distance_MCA_R0_R2	-13,08932114	-103,4736099	-943,8449097	0,112286
Cloud_Mesh_Distance_MCA_R0_R2	-10,94991112	-103,5488892	-944,1224976	-0,226462
Cloud_Mesh_Distance_MCA_R0_R2	-10,92234612	-103,730545	-943,979187	-0,232289
Cloud_Mesh_Distance_MCA_R0_R2	-10,81793594	-103,5981674	-944,0048218	-0,227727
Cloud_Mesh_Distance_MCA_R0_R2	-13,10976791	-103,121872	-943,7748413	0,07732
Cloud_Mesh_Distance_MCA_R0_R2	-13,09971142	-103,2622604	-943,8228149	0,097884
Cloud_Mesh_Distance_MCA_R0_R2	-10,67302704	-103,5425873	-943,9570313	-0,202378
Cloud_Mesh_Distance_MCA_R0_R2	-10,55195427	-103,4236069	-943,9755859	-0,184765
Cloud_Mesh_Distance_MCA_R0_R2	-10,51085281	-103,5463028	-943,8690796	-0,143504
Cloud_Mesh_Distance_MCA_R0_R2	-13,07963657	-102,9718857	-943,7854004	0,061347
Cloud_Mesh_Distance_MCA_R0_R2	-13,15741062	-103,0662308	-943,6426392	0,057634
Cloud_Mesh_Distance_MCA_R0_R2	-10,43953133	-103,3210983	-943,9961548	-0,157115
Cloud_Mesh_Distance_MCA_R0_R2	-9,9285183	-103,3738403	-943,7789307	0,095759
Cloud_Mesh_Distance_MCA_R0_R2	-13,05063057	-102,8229294	-943,7789917	0,050823
Cloud_Mesh_Distance_MCA_R0_R2	-12,97648144	-102,8602295	-943,9246216	0,063243
Cloud_Mesh_Distance_MCA_R0_R2	-10,30640984	-103,3674851	-943,916626	-0,096658
Cloud_Mesh_Distance_MCA_R0_R2	-10,41423225	-103,4461823	-943,8995361	-0,127558
Cloud_Mesh_Distance_MCA_R0_R2	-10,29751015	-103,2208786	-944,0186157	-0,116936
Cloud_Mesh_Distance_MCA_R0_R2	-10,11569023	-103,1992188	-943,9967651	-0,073355
Cloud_Mesh_Distance_MCA_R0_R2	-10,16641617	-103,3226624	-943,9086304	-0,035249
Cloud_Mesh_Distance_MCA_R0_R2	-9,9890995	-103,2857971	-943,8961792	0,032878
Cloud_Mesh_Distance_MCA_R0_R2	-9,84484863	-103,2986221	-943,8417358	0,106665
Cloud_Mesh_Distance_MCA_R0_R2	-9,81863976	-103,1929855	-943,9641724	0,077621
Cloud_Mesh_Distance_MCA_R0_R2	-9,60706902	-103,1579666	-943,9850464	0,136967
Cloud_Mesh_Distance_MCA_R0_R2	-9,6991806	-103,2605438	-943,8430176	0,151687
Cloud_Mesh_Distance_MCA_R0_R2	-9,45693588	-103,1472473	-943,9718628	0,155826
Cloud_Mesh_Distance_MCA_R0_R2	-10,47887039	-103,1783676	-944,0958862	-0,188604
Cloud_Mesh_Distance_MCA_R0_R2	-10,20385742	-103,08564	-944,0896606	-0,110873
Cloud_Mesh_Distance_MCA_R0_R2	-9,22973251	-103,1004028	-943,9454956	0,122236
Cloud_Mesh_Distance_MCA_R0_R2	-9,28477097	-103,1323471	-943,7998657	0,169217

Cloud_Mesh_Distance_MCA_R0_R2	-12,90284824	-102,5204086	-943,8842773	0,025715
Cloud_Mesh_Distance_MCA_R0_R2	-12,96690559	-102,4803391	-943,7510986	0,031177
Cloud_Mesh_Distance_MCA_R0_R2	-12,95877075	-102,1388168	-943,4901733	0,023132
Cloud_Mesh_Distance_MCA_R0_R2	-12,93562317	-102,3227997	-943,7034302	0,021205
Cloud_Mesh_Distance_MCA_R0_R2	-12,85989571	-102,2301331	-943,7702637	0,005918
Cloud_Mesh_Distance_MCA_R0_R2	-12,90020561	-102,1755676	-943,6563721	0,018195
Cloud_Mesh_Distance_MCA_R0_R2	-12,80631256	-101,8732376	-943,5400391	0,008335
Cloud_Mesh_Distance_MCA_R0_R2	-12,69428253	-101,8174286	-943,6607666	-0,029264
Cloud_Mesh_Distance_MCA_R0_R2	-12,7687149	-101,938324	-943,6732788	-0,008596
Cloud_Mesh_Distance_MCA_R0_R2	-8,57655334	-102,4811401	-943,7700195	-0,237542
Cloud_Mesh_Distance_MCA_R0_R2	-8,72533989	-102,6197281	-943,6478272	-0,146114
Cloud_Mesh_Distance_MCA_R0_R2	-12,71523952	-101,7418289	-943,5396118	-0,010719
Cloud_Mesh_Distance_MCA_R0_R2	-12,58025742	-101,7825241	-943,7680054	-0,062101
Cloud_Mesh_Distance_MCA_R0_R2	-8,64801216	-102,4984665	-943,6160278	-0,181069
Cloud_Mesh_Distance_MCA_R0_R2	-12,5906601	-101,6759491	-943,6359253	-0,049585
Cloud_Mesh_Distance_MCA_R0_R2	-12,42939377	-101,571579	-943,6866455	-0,092122
Cloud_Mesh_Distance_MCA_R0_R2	-12,53287983	-101,5652695	-943,5574951	-0,057391
Cloud_Mesh_Distance_MCA_R0_R2	-12,31429291	-101,4547424	-943,6365967	-0,109997
Cloud_Mesh_Distance_MCA_R0_R2	-12,09130287	-101,3574295	-943,6894531	-0,128041
Cloud_Mesh_Distance_MCA_R0_R2	-12,03573227	-101,2845612	-943,6149902	-0,129847
Cloud_Mesh_Distance_MCA_R0_R2	-12,18212509	-101,4179001	-943,7043457	-0,124285
Cloud_Mesh_Distance_MCA_R0_R2	-11,82121468	-101,1473312	-943,5448608	-0,13722
Cloud_Mesh_Distance_MCA_R0_R2	-11,97626877	-101,1983948	-943,503418	-0,138472
Cloud_Mesh_Distance_MCA_R0_R2	-11,97724152	-101,3244553	-943,730896	-0,124061
Cloud_Mesh_Distance_MCA_R0_R2	-11,83693314	-101,2279511	-943,6829224	-0,12176
Cloud_Mesh_Distance_MCA_R0_R2	-11,64792442	-101,0312653	-943,4580689	-0,127501
Cloud_Mesh_Distance_MCA_R0_R2	-11,69522858	-101,0129242	-943,361145	-0,147284
Cloud_Mesh_Distance_MCA_R0_R2	-11,53169632	-100,953331	-943,390625	-0,125643
Cloud_Mesh_Distance_MCA_R0_R2	-11,54319572	-101,0207214	-943,5354004	-0,108194
Cloud_Mesh_Distance_MCA_R0_R2	-11,15826797	-100,8346863	-943,4823608	-0,065292
Cloud_Mesh_Distance_MCA_R0_R2	-11,30793571	-100,8829041	-943,4525147	-0,090385
Cloud_Mesh_Distance_MCA_R0_R2	-11,2163887	-100,7953415	-943,3358765	-0,091935
Cloud_Mesh_Distance_MCA_R0_R2	-11,15715981	-100,9024887	-943,6176758	-0,033885
Cloud_Mesh_Distance_MCA_R0_R2	-10,82538033	-100,7122955	-943,5050659	-0,003584
Cloud_Mesh_Distance_MCA_R0_R2	-10,59691811	-100,6143036	-943,4691773	0,029548
Cloud_Mesh_Distance_MCA_R0_R2	-10,75304794	-100,6408997	-943,4111328	-0,0055
Cloud_Mesh_Distance_MCA_R0_R2	-10,64854813	-100,5885468	-943,3781128	0,005015
Cloud_Mesh_Distance_MCA_R0_R2	-10,5175972	-100,6677933	-943,6115723	0,056917
Cloud_Mesh_Distance_MCA_R0_R2	-12,90781116	-104,0577698	-943,7570801	0,077641
Cloud_Mesh_Distance_MCA_R0_R2	-12,75361061	-104,1044998	-943,836731	0,051423
Cloud_Mesh_Distance_MCA_R0_R2	-12,52570915	-104,2725143	-943,7276611	-0,043707
Cloud_Mesh_Distance_MCA_R0_R2	-12,61532021	-104,1797943	-943,8284302	0,00895
Cloud_Mesh_Distance_MCA_R0_R2	-12,47499466	-104,2031479	-943,8704834	-0,020409
Cloud_Mesh_Distance_MCA_R0_R2	-12,29807663	-104,2280579	-943,9039917	-0,05886
Cloud_Mesh_Distance_MCA_R0_R2	-12,16169739	-104,3152237	-943,7809448	-0,114811
Cloud_Mesh_Distance_MCA_R0_R2	-11,97855949	-104,3200989	-943,7924194	-0,140109
Cloud_Mesh_Distance_MCA_R0_R2	-12,93919945	-103,9104691	-943,8618774	0,109306
Cloud_Mesh_Distance_MCA_R0_R2	-12,84357166	-103,9928742	-943,8852539	0,094109
Cloud_Mesh_Distance_MCA_R0_R2	-11,64556122	-104,244072	-943,878479	-0,159554
Cloud_Mesh_Distance_MCA_R0_R2	-11,51704788	-104,2650681	-943,7861328	-0,179825

Cloud_Mesh_Distance_MCA_R0_R2	-11,52262688	-104,1735306	-943,9332275	-0,164735
Cloud_Mesh_Distance_MCA_R0_R2	-11,38829613	-104,2187653	-943,7883911	-0,191389
Cloud_Mesh_Distance_MCA_R0_R2	-13,02644157	-103,9445953	-943,7336426	0,10528
Cloud_Mesh_Distance_MCA_R0_R2	-13,03312302	-103,8023758	-943,8237305	0,127233
Cloud_Mesh_Distance_MCA_R0_R2	-11,11468315	-104,0572662	-943,8121948	-0,204781
Cloud_Mesh_Distance_MCA_R0_R2	-11,05822945	-103,9522018	-943,8853149	-0,217125
Cloud_Mesh_Distance_MCA_R0_R2	-13,15953445	-103,5455322	-943,710144	0,106169
Cloud_Mesh_Distance_MCA_R0_R2	-13,18385029	-103,6590653	-943,633728	0,097444
Cloud_Mesh_Distance_MCA_R0_R2	-13,11648178	-103,6844025	-943,7539063	0,118215
Cloud_Mesh_Distance_MCA_R0_R2	-11,15911484	-103,9668732	-943,9470825	-0,215843
Cloud_Mesh_Distance_MCA_R0_R2	-11,09619713	-103,8486481	-944,0104981	-0,216898
Cloud_Mesh_Distance_MCA_R0_R2	-10,95544434	-103,8859024	-943,8721314	-0,216139
Cloud_Mesh_Distance_MCA_R0_R2	-13,21349525	-103,5267792	-943,5856934	0,084626
Cloud_Mesh_Distance_MCA_R0_R2	-13,16428852	-103,3862457	-943,7054443	0,092654
Cloud_Mesh_Distance_MCA_R0_R2	-10,79850388	-103,8525009	-943,7905884	-0,178684
Cloud_Mesh_Distance_MCA_R0_R2	-13,22357178	-103,2946625	-943,5271606	0,05797
Cloud_Mesh_Distance_MCA_R0_R2	-13,21208191	-103,0622482	-943,460022	0,031179
Cloud_Mesh_Distance_MCA_R0_R2	-13,15566254	-103,224411	-943,7020264	0,075432
Cloud_Mesh_Distance_MCA_R0_R2	-10,7453537	-103,6936112	-943,8895874	-0,199495
Cloud_Mesh_Distance_MCA_R0_R2	-10,598629	-103,6622772	-943,8270874	-0,148962
Cloud_Mesh_Distance_MCA_R0_R2	-10,18638611	-103,6290436	-943,6213989	0,041166
Cloud_Mesh_Distance_MCA_R0_R2	-10,22333145	-103,5261841	-943,7487183	-0,010164
Cloud_Mesh_Distance_MCA_R0_R2	-10,09471893	-103,5392761	-943,6661377	0,057376
Cloud_Mesh_Distance_MCA_R0_R2	-10,37104797	-103,5472717	-943,800293	-0,078753
Cloud_Mesh_Distance_MCA_R0_R2	-10,21552849	-103,423317	-943,8395386	-0,029858
Cloud_Mesh_Distance_MCA_R0_R2	-10,05347443	-103,6194687	-943,5305176	0,105218
Cloud_Mesh_Distance_MCA_R0_R2	-10,07823181	-103,4247437	-943,7841797	0,04753
Cloud_Mesh_Distance_MCA_R0_R2	-13,0702858	-102,7428894	-943,6934815	0,045695
Cloud_Mesh_Distance_MCA_R0_R2	-13,11237526	-102,8786087	-943,668335	0,049886
Cloud_Mesh_Distance_MCA_R0_R2	-13,16529655	-102,9243469	-943,5425415	0,03652
Cloud_Mesh_Distance_MCA_R0_R2	-12,99530029	-102,6679688	-943,8074951	0,039564
Cloud_Mesh_Distance_MCA_R0_R2	-10,3057766	-103,4639969	-943,8406372	-0,069206
Cloud_Mesh_Distance_MCA_R0_R2	-9,61180878	-103,305603	-943,6796875	0,199097
Cloud_Mesh_Distance_MCA_R0_R2	-9,79707527	-103,3624039	-943,7253418	0,144371
Cloud_Mesh_Distance_MCA_R0_R2	-9,43070793	-103,2066193	-943,7402344	0,204426
Cloud_Mesh_Distance_MCA_R0_R2	-9,46567345	-103,2531128	-943,5603027	0,218131
Cloud_Mesh_Distance_MCA_R0_R2	-9,5285244	-103,2098999	-943,8536377	0,179221
Cloud_Mesh_Distance_MCA_R0_R2	-13,12544823	-102,7504578	-943,5475464	0,032003
Cloud_Mesh_Distance_MCA_R0_R2	-13,04947662	-102,6018066	-943,6530762	0,038655
Cloud_Mesh_Distance_MCA_R0_R2	-9,22564888	-103,0826492	-943,5344849	0,167654
Cloud_Mesh_Distance_MCA_R0_R2	-9,32411575	-103,1596375	-943,6400757	0,196978
Cloud_Mesh_Distance_MCA_R0_R2	-9,37530708	-103,1530075	-943,8929443	0,171655
Cloud_Mesh_Distance_MCA_R0_R2	-9,1613121	-103,0731964	-943,8537598	0,110772
Cloud_Mesh_Distance_MCA_R0_R2	-9,18712807	-103,0750656	-943,7141724	0,141283
Cloud_Mesh_Distance_MCA_R0_R2	-13,01615715	-102,4279404	-943,6062622	0,028833
Cloud_Mesh_Distance_MCA_R0_R2	-8,9943161	-102,9176407	-943,6643066	0,037327
Cloud_Mesh_Distance_MCA_R0_R2	-9,0295639	-102,9040756	-943,5119019	0,067649
Cloud_Mesh_Distance_MCA_R0_R2	-9,09966087	-102,9967041	-943,6239014	0,102902
Cloud_Mesh_Distance_MCA_R0_R2	-9,02437019	-102,985054	-943,8145142	0,039496
Cloud_Mesh_Distance_MCA_R0_R2	-12,97559643	-102,2787705	-943,5822144	0,027265

Cloud_Mesh_Distance_MCA_R0_R2	-8,9222641	-102,8273392	-943,6065064	-0,006016
Cloud_Mesh_Distance_MCA_R0_R2	-8,89225006	-102,8572311	-943,7518311	-0,039463
Cloud_Mesh_Distance_MCA_R0_R2	-12,89624977	-101,9820404	-943,4754028	0,02404
Cloud_Mesh_Distance_MCA_R0_R2	-12,85804176	-102,02668	-943,6038208	0,01302
Cloud_Mesh_Distance_MCA_R0_R2	-8,80288696	-102,7262802	-943,6710205	-0,096097
Cloud_Mesh_Distance_MCA_R0_R2	-12,87540627	-101,8886566	-943,4080811	0,024911
Cloud_Mesh_Distance_MCA_R0_R2	-8,77945709	-102,6344605	-943,5297241	-0,096966
Cloud_Mesh_Distance_MCA_R0_R2	-8,71623039	-102,5227509	-943,4418335	-0,119872
Cloud_Mesh_Distance_MCA_R0_R2	-12,80001068	-101,755806	-943,3944092	0,016449
Cloud_Mesh_Distance_MCA_R0_R2	-12,75198364	-101,626442	-943,2762451	0,023981
Cloud_Mesh_Distance_MCA_R0_R2	-12,65774345	-101,6166458	-943,4514771	-0,019099
Cloud_Mesh_Distance_MCA_R0_R2	-12,52830887	-101,4928436	-943,4434204	-0,047206
Cloud_Mesh_Distance_MCA_R0_R2	-12,37858391	-101,3359833	-943,3334351	-0,071246
Cloud_Mesh_Distance_MCA_R0_R2	-12,44233322	-101,3275299	-943,1999512	-0,048009
Cloud_Mesh_Distance_MCA_R0_R2	-12,37730026	-101,3992844	-943,4711304	-0,080499
Cloud_Mesh_Distance_MCA_R0_R2	-12,43928051	-101,492424	-943,5606079	-0,079494
Cloud_Mesh_Distance_MCA_R0_R2	-12,19903755	-101,3495941	-943,5783081	-0,123683
Cloud_Mesh_Distance_MCA_R0_R2	-12,25563622	-101,3000412	-943,4158325	-0,101563
Cloud_Mesh_Distance_MCA_R0_R2	-12,13600159	-101,2075043	-943,3457031	-0,120556
Cloud_Mesh_Distance_MCA_R0_R2	-12,12255096	-101,264595	-943,493103	-0,127408
Cloud_Mesh_Distance_MCA_R0_R2	-11,92267799	-101,0751572	-943,2540894	-0,151914
Cloud_Mesh_Distance_MCA_R0_R2	-11,89384842	-101,1397247	-943,4594727	-0,141404
Cloud_Mesh_Distance_MCA_R0_R2	-11,96965027	-101,1373138	-943,3670654	-0,144691
Cloud_Mesh_Distance_MCA_R0_R2	-11,80289078	-101,0805588	-943,4152832	-0,143296
Cloud_Mesh_Distance_MCA_R0_R2	-11,79498291	-101,030098	-943,2879639	-0,155304
Cloud_Mesh_Distance_MCA_R0_R2	-11,85186005	-101,0131836	-943,152771	-0,157112
Cloud_Mesh_Distance_MCA_R0_R2	-11,69953537	-100,9720917	-943,2387695	-0,15022
Cloud_Mesh_Distance_MCA_R0_R2	-11,40444183	-100,8572846	-943,2808838	-0,118178
Cloud_Mesh_Distance_MCA_R0_R2	-11,06281281	-100,7737961	-943,4376831	-0,057221
Cloud_Mesh_Distance_MCA_R0_R2	-11,0796833	-100,7307205	-943,3183594	-0,073949
Cloud_Mesh_Distance_MCA_R0_R2	-11,32954025	-100,783638	-943,1612549	-0,12069
Cloud_Mesh_Distance_MCA_R0_R2	-10,93396378	-100,6992188	-943,388916	-0,040511
Cloud_Mesh_Distance_MCA_R0_R2	-10,71196938	-100,5731506	-943,2880859	-0,014142
Cloud_Mesh_Distance_MCA_R0_R2	-13,00724411	-104,1665878	-943,5126343	0,055685
Cloud_Mesh_Distance_MCA_R0_R2	-12,90313911	-104,1862106	-943,5974731	0,044593
Cloud_Mesh_Distance_MCA_R0_R2	-13,01699162	-104,0728302	-943,6207275	0,079085
Cloud_Mesh_Distance_MCA_R0_R2	-12,80842018	-104,1675873	-943,710083	0,041199
Cloud_Mesh_Distance_MCA_R0_R2	-12,76209641	-104,2551041	-943,6081543	-0,005056
Cloud_Mesh_Distance_MCA_R0_R2	-12,68443775	-104,2198257	-943,7232666	0,002227
Cloud_Mesh_Distance_MCA_R0_R2	-12,60949993	-104,3162308	-943,5980225	-0,044647
Cloud_Mesh_Distance_MCA_R0_R2	-12,34445572	-104,3037872	-943,750061	-0,084136
Cloud_Mesh_Distance_MCA_R0_R2	-12,40104294	-104,3807755	-943,5639648	-0,107436
Cloud_Mesh_Distance_MCA_R0_R2	-11,77382851	-104,3074799	-943,8001709	-0,15837
Cloud_Mesh_Distance_MCA_R0_R2	-11,62038803	-104,3781891	-943,6167603	-0,173586
Cloud_Mesh_Distance_MCA_R0_R2	-11,52597618	-104,3252335	-943,6835327	-0,176068
Cloud_Mesh_Distance_MCA_R0_R2	-13,11414337	-103,963562	-943,5934448	0,096483
Cloud_Mesh_Distance_MCA_R0_R2	-13,09753895	-103,8425217	-943,708252	0,115596
Cloud_Mesh_Distance_MCA_R0_R2	-11,61459732	-104,3071823	-943,755127	-0,175491
Cloud_Mesh_Distance_MCA_R0_R2	-11,41481972	-104,301651	-943,6677246	-0,179831
Cloud_Mesh_Distance_MCA_R0_R2	-11,25024605	-104,2615051	-943,6305542	-0,174988

Cloud_Mesh_Distance_MCA_R0_R2	-11,25495911	-104,1510544	-943,7976074	-0,198563
Cloud_Mesh_Distance_MCA_R0_R2	-10,9601078	-104,1102142	-943,6315308	-0,159741
Cloud_Mesh_Distance_MCA_R0_R2	-10,97052956	-103,9895325	-943,7806397	-0,194138
Cloud_Mesh_Distance_MCA_R0_R2	-11,12644768	-104,1695023	-943,6804199	-0,185325
Cloud_Mesh_Distance_MCA_R0_R2	-13,23271751	-103,7350769	-943,4829712	0,085365
Cloud_Mesh_Distance_MCA_R0_R2	-13,16448212	-103,8034439	-943,6130371	0,099801
Cloud_Mesh_Distance_MCA_R0_R2	-10,89269638	-103,9299622	-943,7843018	-0,191934
Cloud_Mesh_Distance_MCA_R0_R2	-13,26518631	-103,5434876	-943,4078369	0,054601
Cloud_Mesh_Distance_MCA_R0_R2	-10,84857178	-103,9856873	-943,6939697	-0,161084
Cloud_Mesh_Distance_MCA_R0_R2	-10,66469193	-103,767395	-943,7783814	-0,148708
Cloud_Mesh_Distance_MCA_R0_R2	-10,63999557	-103,8599243	-943,6759033	-0,119004
Cloud_Mesh_Distance_MCA_R0_R2	-10,27222252	-103,725708	-943,5686646	0,02106
Cloud_Mesh_Distance_MCA_R0_R2	-10,22184372	-103,7737274	-943,4666748	0,066559
Cloud_Mesh_Distance_MCA_R0_R2	-13,2541275	-103,1916199	-943,3422241	0,014897
Cloud_Mesh_Distance_MCA_R0_R2	-13,27737617	-103,2634277	-943,2052612	-0,01162
Cloud_Mesh_Distance_MCA_R0_R2	-13,27708912	-103,3794251	-943,3109131	0,01894
Cloud_Mesh_Distance_MCA_R0_R2	-10,45700455	-103,6528702	-943,7542725	-0,091027
Cloud_Mesh_Distance_MCA_R0_R2	-10,54334831	-103,7604294	-943,7086182	-0,102465
Cloud_Mesh_Distance_MCA_R0_R2	-10,31788826	-103,6412659	-943,6873779	-0,02993
Cloud_Mesh_Distance_MCA_R0_R2	-10,40436363	-103,750618	-943,6318359	-0,040987
Cloud_Mesh_Distance_MCA_R0_R2	-10,16836929	-103,6996307	-943,5200195	0,069695
Cloud_Mesh_Distance_MCA_R0_R2	-13,26095867	-103,1414871	-943,2253418	-0,006981
Cloud_Mesh_Distance_MCA_R0_R2	-13,19382572	-102,874855	-943,3825684	0,019479
Cloud_Mesh_Distance_MCA_R0_R2	-9,89589691	-103,5551376	-943,4677734	0,168166
Cloud_Mesh_Distance_MCA_R0_R2	-9,94199944	-103,4780731	-943,645752	0,121876
Cloud_Mesh_Distance_MCA_R0_R2	-9,76015091	-103,4990082	-943,3540039	0,20376
Cloud_Mesh_Distance_MCA_R0_R2	-9,76653099	-103,4336472	-943,5577393	0,181365
Cloud_Mesh_Distance_MCA_R0_R2	-13,15241051	-102,6908569	-943,3877564	0,021314
Cloud_Mesh_Distance_MCA_R0_R2	-13,13052464	-102,5369415	-943,3011475	0,020196
Cloud_Mesh_Distance_MCA_R0_R2	-13,0929594	-102,5559692	-943,4909668	0,023134
Cloud_Mesh_Distance_MCA_R0_R2	-9,354496	-103,1783447	-943,4742432	0,207507
Cloud_Mesh_Distance_MCA_R0_R2	-9,19938469	-103,03508	-943,3858643	0,147259
Cloud_Mesh_Distance_MCA_R0_R2	-9,13249588	-102,9951096	-943,4900513	0,123636
Cloud_Mesh_Distance_MCA_R0_R2	-13,04961967	-102,3480988	-943,4326782	0,02851
Cloud_Mesh_Distance_MCA_R0_R2	-9,10715866	-102,9456177	-943,3848877	0,108798
Cloud_Mesh_Distance_MCA_R0_R2	-8,94252777	-102,8042221	-943,4828491	0,013283
Cloud_Mesh_Distance_MCA_R0_R2	-13,02646637	-102,1896286	-943,3269653	0,03533
Cloud_Mesh_Distance_MCA_R0_R2	-13,03789616	-102,1375961	-943,1803589	0,047192
Cloud_Mesh_Distance_MCA_R0_R2	-8,88396072	-102,7187958	-943,4222412	-0,02194
Cloud_Mesh_Distance_MCA_R0_R2	-8,86696625	-102,7430725	-943,5442505	-0,041049
Cloud_Mesh_Distance_MCA_R0_R2	-12,8923893	-101,8490448	-943,2974854	0,045207
Cloud_Mesh_Distance_MCA_R0_R2	-12,96121025	-102,0061035	-943,3143921	0,048044
Cloud_Mesh_Distance_MCA_R0_R2	-12,99928284	-102,018692	-943,1628418	0,060619
Cloud_Mesh_Distance_MCA_R0_R2	-12,85182381	-101,7377243	-943,2172852	0,05004
Cloud_Mesh_Distance_MCA_R0_R2	-12,633255	-101,5104828	-943,2943726	-0,003206
Cloud_Mesh_Distance_MCA_R0_R2	-12,49807262	-101,4084549	-943,3135376	-0,041136
Cloud_Mesh_Distance_MCA_R0_R2	-12,36590195	-101,2578354	-943,1256714	-0,065446
Cloud_Mesh_Distance_MCA_R0_R2	-12,30438995	-101,2655945	-943,262146	-0,088668
Cloud_Mesh_Distance_MCA_R0_R2	-12,07313538	-101,1326675	-943,2178345	-0,132387
Cloud_Mesh_Distance_MCA_R0_R2	-11,90758514	-101,001976	-943,0146484	-0,155352

Cloud_Mesh_Distance_MCA_R0_R2	-11,7477932	-100,9358978	-943,0338745	-0,163102
Cloud_Mesh_Distance_MCA_R0_R2	-11,5720768	-100,9001236	-943,1864624	-0,152592
Cloud_Mesh_Distance_MCA_R0_R2	-11,57801056	-100,8553391	-943,0187378	-0,164045
Cloud_Mesh_Distance_MCA_R0_R2	-11,46540451	-100,8361893	-943,1339722	-0,143663
Cloud_Mesh_Distance_MCA_R0_R2	-11,1690712	-100,7206497	-943,1860962	-0,100063
Cloud_Mesh_Distance_MCA_R0_R2	-11,01277733	-100,6668701	-943,2260132	-0,075418
Cloud_Mesh_Distance_MCA_R0_R2	-10,85962868	-100,6214523	-943,2694092	-0,045383
Cloud_Mesh_Distance_MCA_R0_R2	-10,9335041	-100,5980377	-943,1201782	-0,076233
Cloud_Mesh_Distance_MCA_R0_R2	-10,98866272	-100,5723114	-942,9559937	-0,101637
Cloud_Mesh_Distance_MCA_R0_R2	-10,80324936	-100,5640106	-943,1723633	-0,04458
Cloud_Mesh_Distance_MCA_R0_R2	-13,05455017	-104,1969299	-943,4001465	0,053933
Cloud_Mesh_Distance_MCA_R0_R2	-12,9355669	-104,2689285	-943,4261475	0,021912
Cloud_Mesh_Distance_MCA_R0_R2	-12,7063427	-104,3406296	-943,4865723	-0,03912
Cloud_Mesh_Distance_MCA_R0_R2	-12,8271637	-104,2839508	-943,5040283	-0,000387
Cloud_Mesh_Distance_MCA_R0_R2	-12,79958725	-104,3543854	-943,3840942	-0,02607
Cloud_Mesh_Distance_MCA_R0_R2	-13,10255432	-104,2079468	-943,2984619	0,048699
Cloud_Mesh_Distance_MCA_R0_R2	-13,08609009	-104,0955124	-943,4991455	0,075825
Cloud_Mesh_Distance_MCA_R0_R2	-12,55170822	-104,4012528	-943,4467163	-0,090857
Cloud_Mesh_Distance_MCA_R0_R2	-12,18928909	-104,4669724	-943,4299927	-0,157024
Cloud_Mesh_Distance_MCA_R0_R2	-12,19100189	-104,3870926	-943,619751	-0,140764
Cloud_Mesh_Distance_MCA_R0_R2	-12,03676605	-104,3792343	-943,6641235	-0,152358
Cloud_Mesh_Distance_MCA_R0_R2	-11,84474945	-104,4059067	-943,6040649	-0,177488
Cloud_Mesh_Distance_MCA_R0_R2	-11,84891701	-104,4863129	-943,4205933	-0,176882
Cloud_Mesh_Distance_MCA_R0_R2	-11,3456192	-104,3610535	-943,5206299	-0,162078
Cloud_Mesh_Distance_MCA_R0_R2	-11,47348595	-104,3617477	-943,5894165	-0,176906
Cloud_Mesh_Distance_MCA_R0_R2	-11,47233963	-104,4265518	-943,4589233	-0,163311
Cloud_Mesh_Distance_MCA_R0_R2	-13,19199371	-103,9205933	-943,477478	0,086914
Cloud_Mesh_Distance_MCA_R0_R2	-13,24352264	-103,9152222	-943,3310547	0,063864
Cloud_Mesh_Distance_MCA_R0_R2	-13,13097191	-104,1247406	-943,3823853	0,069133
Cloud_Mesh_Distance_MCA_R0_R2	-13,14888382	-104,0324554	-943,463501	0,081323
Cloud_Mesh_Distance_MCA_R0_R2	-11,17718697	-104,351326	-943,4299927	-0,142676
Cloud_Mesh_Distance_MCA_R0_R2	-11,06178856	-104,2465057	-943,5192871	-0,146169
Cloud_Mesh_Distance_MCA_R0_R2	-10,67890739	-104,1562882	-943,3323364	-0,040438
Cloud_Mesh_Distance_MCA_R0_R2	-10,58428955	-104,0510559	-943,4067383	-0,0289
Cloud_Mesh_Distance_MCA_R0_R2	-10,55292702	-104,1076279	-943,2802124	0,001933
Cloud_Mesh_Distance_MCA_R0_R2	-10,45913506	-104,0317154	-943,3110962	0,022987
Cloud_Mesh_Distance_MCA_R0_R2	-10,46306229	-104,096962	-943,1769409	0,051555
Cloud_Mesh_Distance_MCA_R0_R2	-13,29612064	-103,5783997	-943,2145996	0,010728
Cloud_Mesh_Distance_MCA_R0_R2	-10,72820091	-103,9479675	-943,6470337	-0,127461
Cloud_Mesh_Distance_MCA_R0_R2	-10,750597	-104,0671921	-943,5239258	-0,106259
Cloud_Mesh_Distance_MCA_R0_R2	-10,61059761	-103,9604034	-943,5482788	-0,073468
Cloud_Mesh_Distance_MCA_R0_R2	-10,35342979	-104,0073319	-943,2151489	0,079411
Cloud_Mesh_Distance_MCA_R0_R2	-10,47190857	-103,9521484	-943,4463501	-0,005945
Cloud_Mesh_Distance_MCA_R0_R2	-13,29422188	-103,4230576	-943,1351929	-0,020775
Cloud_Mesh_Distance_MCA_R0_R2	-10,5006485	-103,8581467	-943,583252	-0,055374
Cloud_Mesh_Distance_MCA_R0_R2	-10,34949112	-103,9413834	-943,3422241	0,053197
Cloud_Mesh_Distance_MCA_R0_R2	-10,34487915	-103,8411026	-943,484436	0,021131
Cloud_Mesh_Distance_MCA_R0_R2	-10,21795082	-103,8473129	-943,3447266	0,094514
Cloud_Mesh_Distance_MCA_R0_R2	-10,11635208	-103,7353058	-943,4136963	0,110084
Cloud_Mesh_Distance_MCA_R0_R2	-10,06522179	-103,7779541	-943,2415161	0,156454

Cloud_Mesh_Distance_MCA_R0_R2	-9,94370651	-103,671463	-943,2642822	0,183014
Cloud_Mesh_Distance_MCA_R0_R2	-9,88541603	-103,6022339	-943,336853	0,187791
Cloud_Mesh_Distance_MCA_R0_R2	-9,99028778	-103,6656418	-943,3759766	0,15704
Cloud_Mesh_Distance_MCA_R0_R2	-9,8477478	-103,5990524	-943,2215576	0,199984
Cloud_Mesh_Distance_MCA_R0_R2	-13,23786926	-103,0380249	-943,2964478	0,008133
Cloud_Mesh_Distance_MCA_R0_R2	-9,61222458	-103,3655167	-943,4648438	0,212169
Cloud_Mesh_Distance_MCA_R0_R2	-9,47265625	-103,2693481	-943,3540039	0,219682
Cloud_Mesh_Distance_MCA_R0_R2	-9,35687065	-103,1692581	-943,3403931	0,201375
Cloud_Mesh_Distance_MCA_R0_R2	-13,09353065	-102,3365021	-943,2006226	0,028562
Cloud_Mesh_Distance_MCA_R0_R2	-9,28191948	-103,0898514	-943,2796021	0,172821
Cloud_Mesh_Distance_MCA_R0_R2	-9,28361702	-103,1136627	-943,4039917	0,186565
Cloud_Mesh_Distance_MCA_R0_R2	-13,11824417	-102,3795548	-943,0148315	0,014739
Cloud_Mesh_Distance_MCA_R0_R2	-9,00595474	-102,8224564	-943,3206177	0,052382
Cloud_Mesh_Distance_MCA_R0_R2	-13,03238583	-102,0615616	-943,0087891	0,052229
Cloud_Mesh_Distance_MCA_R0_R2	-12,94812584	-101,8836746	-943,137085	0,063876
Cloud_Mesh_Distance_MCA_R0_R2	-8,95029068	-102,7125549	-943,1489258	0,039651
Cloud_Mesh_Distance_MCA_R0_R2	-8,83973885	-102,6036911	-943,197876	-0,045067
Cloud_Mesh_Distance_MCA_R0_R2	-8,89795113	-102,6943512	-943,2908936	-0,009021
Cloud_Mesh_Distance_MCA_R0_R2	-8,80791092	-102,6103516	-943,3590698	-0,060242
Cloud_Mesh_Distance_MCA_R0_R2	-12,80431175	-101,6377335	-943,144165	0,048399
Cloud_Mesh_Distance_MCA_R0_R2	-12,87701797	-101,7124939	-943,0372315	0,064829
Cloud_Mesh_Distance_MCA_R0_R2	-12,89505005	-101,7128372	-942,8973389	0,083096
Cloud_Mesh_Distance_MCA_R0_R2	-12,79771996	-101,597023	-943,0409546	0,056158
Cloud_Mesh_Distance_MCA_R0_R2	-12,72457218	-101,5416565	-943,1421509	0,029868
Cloud_Mesh_Distance_MCA_R0_R2	-12,72755814	-101,4954529	-942,9608765	0,049774
Cloud_Mesh_Distance_MCA_R0_R2	-12,60693932	-101,3784027	-942,9411621	0,018765
Cloud_Mesh_Distance_MCA_R0_R2	-12,59594917	-101,4169006	-943,1279297	0,000152
Cloud_Mesh_Distance_MCA_R0_R2	-12,48393059	-101,3078613	-943,0344849	-0,024786
Cloud_Mesh_Distance_MCA_R0_R2	-12,35185051	-101,2141419	-942,9801025	-0,05894
Cloud_Mesh_Distance_MCA_R0_R2	-12,22716141	-101,1894913	-943,151001	-0,100343
Cloud_Mesh_Distance_MCA_R0_R2	-12,19749641	-101,1383362	-943,0118408	-0,095251
Cloud_Mesh_Distance_MCA_R0_R2	-12,13484573	-101,1322556	-943,1132202	-0,114294
Cloud_Mesh_Distance_MCA_R0_R2	-12,00372314	-101,0265656	-942,9244995	-0,133516
Cloud_Mesh_Distance_MCA_R0_R2	-12,03226852	-101,0733795	-943,0708618	-0,133557
Cloud_Mesh_Distance_MCA_R0_R2	-11,89567947	-100,9692307	-942,8863525	-0,154259
Cloud_Mesh_Distance_MCA_R0_R2	-11,68044281	-100,8746033	-942,9002075	-0,17467
Cloud_Mesh_Distance_MCA_R0_R2	-11,44699097	-100,7397308	-942,7731934	-0,158664
Cloud_Mesh_Distance_MCA_R0_R2	-11,55761147	-100,8045349	-942,8361816	-0,165741
Cloud_Mesh_Distance_MCA_R0_R2	-11,43775177	-100,7894745	-943,0090332	-0,158891
Cloud_Mesh_Distance_MCA_R0_R2	-11,41011429	-100,7471237	-942,8877564	-0,15931
Cloud_Mesh_Distance_MCA_R0_R2	-11,27507591	-100,7115479	-942,9994507	-0,137179
Cloud_Mesh_Distance_MCA_R0_R2	-11,32398224	-100,6890869	-942,8082275	-0,14696
Cloud_Mesh_Distance_MCA_R0_R2	-11,09028625	-100,6463471	-943,0601196	-0,105557
Cloud_Mesh_Distance_MCA_R0_R2	-13,11386871	-104,2548828	-943,1730347	0,04159
Cloud_Mesh_Distance_MCA_R0_R2	-13,01343536	-104,3019333	-943,257019	0,030844
Cloud_Mesh_Distance_MCA_R0_R2	-12,88535595	-104,3611832	-943,2886353	-0,007584
Cloud_Mesh_Distance_MCA_R0_R2	-12,60590744	-104,5189972	-943,1175537	-0,086567
Cloud_Mesh_Distance_MCA_R0_R2	-12,74602509	-104,4413147	-943,2211304	-0,051456
Cloud_Mesh_Distance_MCA_R0_R2	-12,60617161	-104,4727707	-943,241272	-0,091911
Cloud_Mesh_Distance_MCA_R0_R2	-12,66101933	-104,4136276	-943,3526001	-0,070256

Cloud_Mesh_Distance_MCA_R0_R2	-13,16392708	-104,152771	-943,2591553	0,058357
Cloud_Mesh_Distance_MCA_R0_R2	-12,46910477	-104,5213852	-943,1895142	-0,117623
Cloud_Mesh_Distance_MCA_R0_R2	-12,53541565	-104,4586868	-943,3166504	-0,105515
Cloud_Mesh_Distance_MCA_R0_R2	-12,37928391	-104,4777908	-943,3423462	-0,133632
Cloud_Mesh_Distance_MCA_R0_R2	-12,18296051	-104,5444565	-943,2376709	-0,155608
Cloud_Mesh_Distance_MCA_R0_R2	-11,97136307	-104,537529	-943,2941895	-0,175013
Cloud_Mesh_Distance_MCA_R0_R2	-12,01216698	-104,4481354	-943,5074463	-0,166814
Cloud_Mesh_Distance_MCA_R0_R2	-11,65735245	-104,4797134	-943,4083252	-0,169317
Cloud_Mesh_Distance_MCA_R0_R2	-11,30252457	-104,4391098	-943,3418579	-0,133999
Cloud_Mesh_Distance_MCA_R0_R2	-13,19111633	-104,0415726	-943,3571777	0,077284
Cloud_Mesh_Distance_MCA_R0_R2	-11,27439499	-104,5183945	-943,1464233	-0,100403
Cloud_Mesh_Distance_MCA_R0_R2	-13,28064728	-103,7507095	-943,2843628	0,043996
Cloud_Mesh_Distance_MCA_R0_R2	-11,00844574	-104,3301621	-943,3353272	-0,109624
Cloud_Mesh_Distance_MCA_R0_R2	-10,8566246	-104,3562088	-943,1174316	-0,038174
Cloud_Mesh_Distance_MCA_R0_R2	-10,88634872	-104,3151016	-943,2488403	-0,075664
Cloud_Mesh_Distance_MCA_R0_R2	-10,86629486	-104,2209854	-943,4008789	-0,101555
Cloud_Mesh_Distance_MCA_R0_R2	-10,59424019	-104,1816406	-943,1757202	0,009792
Cloud_Mesh_Distance_MCA_R0_R2	-13,29910564	-103,7721787	-943,0904541	0,004291
Cloud_Mesh_Distance_MCA_R0_R2	-10,2698288	-103,9320755	-943,2505493	0,100982
Cloud_Mesh_Distance_MCA_R0_R2	-10,18677139	-103,8845596	-943,2126465	0,131661
Cloud_Mesh_Distance_MCA_R0_R2	-13,3014946	-103,6015015	-943,0267334	-0,034651
Cloud_Mesh_Distance_MCA_R0_R2	-13,28236866	-103,291832	-943,0661621	-0,048027
Cloud_Mesh_Distance_MCA_R0_R2	-13,2701664	-103,1802216	-943,1339722	-0,025655
Cloud_Mesh_Distance_MCA_R0_R2	-13,28900528	-103,4387589	-942,9631348	-0,072059
Cloud_Mesh_Distance_MCA_R0_R2	-13,2702446	-103,2890396	-942,9191284	-0,088566
Cloud_Mesh_Distance_MCA_R0_R2	-9,94185257	-103,6941071	-943,1473999	0,188257
Cloud_Mesh_Distance_MCA_R0_R2	-10,02859306	-103,7802658	-943,0795898	0,175025
Cloud_Mesh_Distance_MCA_R0_R2	-9,76801682	-103,5209274	-943,0115967	0,203062
Cloud_Mesh_Distance_MCA_R0_R2	-13,22490883	-102,9232483	-943,2213135	-0,003147
Cloud_Mesh_Distance_MCA_R0_R2	-13,25175476	-103,054039	-943,1389771	-0,021698
Cloud_Mesh_Distance_MCA_R0_R2	-13,1909256	-102,7505112	-943,2072144	-0,001174
Cloud_Mesh_Distance_MCA_R0_R2	-13,22677994	-102,9090118	-943,0823975	-0,03531
Cloud_Mesh_Distance_MCA_R0_R2	-9,72852325	-103,4961014	-943,1525269	0,212904
Cloud_Mesh_Distance_MCA_R0_R2	-9,61738586	-103,3926697	-943,2716065	0,219328
Cloud_Mesh_Distance_MCA_R0_R2	-9,62242508	-103,3919678	-943,1303101	0,212996
Cloud_Mesh_Distance_MCA_R0_R2	-13,19962311	-102,7745209	-943,0023804	-0,032441
Cloud_Mesh_Distance_MCA_R0_R2	-13,15695763	-102,5603714	-943,0721436	-0,00583
Cloud_Mesh_Distance_MCA_R0_R2	-9,57748413	-103,3331833	-943,0536499	0,202433
Cloud_Mesh_Distance_MCA_R0_R2	-9,52663994	-103,3051148	-943,1714478	0,216324
Cloud_Mesh_Distance_MCA_R0_R2	-9,4889679	-103,236908	-943,0332642	0,193064
Cloud_Mesh_Distance_MCA_R0_R2	-13,17397976	-102,6683273	-942,901062	-0,031351
Cloud_Mesh_Distance_MCA_R0_R2	-9,39575958	-103,1809845	-943,1877441	0,194804
Cloud_Mesh_Distance_MCA_R0_R2	-9,37073135	-103,1024094	-942,9970093	0,155418
Cloud_Mesh_Distance_MCA_R0_R2	-13,14135742	-102,5026856	-942,8812866	-0,011686
Cloud_Mesh_Distance_MCA_R0_R2	-13,09481239	-102,2824631	-942,8634033	0,018681
Cloud_Mesh_Distance_MCA_R0_R2	-9,15730667	-102,90065	-943,050293	0,097146
Cloud_Mesh_Distance_MCA_R0_R2	-9,16835785	-102,9616928	-943,2131348	0,12427
Cloud_Mesh_Distance_MCA_R0_R2	-9,29141235	-103,0657578	-943,1422119	0,157676
Cloud_Mesh_Distance_MCA_R0_R2	-9,0672245	-102,8255234	-943,1089478	0,069152
Cloud_Mesh_Distance_MCA_R0_R2	-13,08055592	-102,2357636	-943,0548706	0,031391

Cloud_Mesh_Distance_MCA_R0_R2	-13,03761196	-102,0656509	-942,7473145	0,050422
Cloud_Mesh_Distance_MCA_R0_R2	-9,01246262	-102,7220764	-942,9515991	0,059108
Cloud_Mesh_Distance_MCA_R0_R2	-12,96119785	-101,8497467	-942,8973389	0,076401
Cloud_Mesh_Distance_MCA_R0_R2	-8,87847137	-102,6048813	-943,0250244	-0,19763
Cloud_Mesh_Distance_MCA_R0_R2	-12,83556747	-101,6203995	-942,9138184	0,074747
Cloud_Mesh_Distance_MCA_R0_R2	-12,50703526	-101,2821732	-942,8299561	0,004156
Cloud_Mesh_Distance_MCA_R0_R2	-12,68693733	-101,4175415	-942,7435303	0,057178
Cloud_Mesh_Distance_MCA_R0_R2	-12,42651367	-101,2044373	-942,6677856	-0,009035
Cloud_Mesh_Distance_MCA_R0_R2	-12,57221794	-101,3012924	-942,6182251	0,037569
Cloud_Mesh_Distance_MCA_R0_R2	-12,27400208	-101,1512756	-942,8856201	-0,074179
Cloud_Mesh_Distance_MCA_R0_R2	-12,37289047	-101,195343	-942,8215332	-0,035493
Cloud_Mesh_Distance_MCA_R0_R2	-12,14464378	-101,0825729	-942,871521	-0,100247
Cloud_Mesh_Distance_MCA_R0_R2	-12,02749062	-101,0103684	-942,7786865	-0,125927
Cloud_Mesh_Distance_MCA_R0_R2	-12,04882813	-100,994278	-942,6053467	-0,109172
Cloud_Mesh_Distance_MCA_R0_R2	-11,72561646	-100,8666077	-942,7532959	-0,169034
Cloud_Mesh_Distance_MCA_R0_R2	-11,78871346	-100,9144974	-942,855957	-0,166192
Cloud_Mesh_Distance_MCA_R0_R2	-11,64141846	-100,8096008	-942,6456909	-0,167321
Cloud_Mesh_Distance_MCA_R0_R2	-11,52404404	-100,7605209	-942,6871338	-0,161022
Cloud_Mesh_Distance_MCA_R0_R2	-11,14853477	-100,6206207	-942,8673706	-0,12646
Cloud_Mesh_Distance_MCA_R0_R2	-11,23847008	-100,6247559	-942,6798706	-0,139003
Cloud_Mesh_Distance_MCA_R0_R2	-11,07625961	-100,5592499	-942,7244873	-0,119938
Cloud_Mesh_Distance_MCA_R0_R2	-11,02692032	-100,5608902	-942,8408203	-0,112051
Cloud_Mesh_Distance_MCA_R0_R2	-13,17730427	-104,2425385	-942,9779053	0,020782
Cloud_Mesh_Distance_MCA_R0_R2	-13,13115978	-104,2842484	-943,0415649	0,027388
Cloud_Mesh_Distance_MCA_R0_R2	-13,03583717	-104,3684845	-943,0438232	0,016551
Cloud_Mesh_Distance_MCA_R0_R2	-12,8991394	-104,4826202	-942,9348145	-0,003481
Cloud_Mesh_Distance_MCA_R0_R2	-12,89501762	-104,4192658	-943,1306763	-0,01724
Cloud_Mesh_Distance_MCA_R0_R2	-12,73150826	-104,5229187	-943,0090942	-0,046811
Cloud_Mesh_Distance_MCA_R0_R2	-12,58336639	-104,5691681	-942,9900513	-0,080672
Cloud_Mesh_Distance_MCA_R0_R2	-13,22471523	-104,0673752	-943,2070313	0,056504
Cloud_Mesh_Distance_MCA_R0_R2	-13,18204498	-104,1927185	-943,1143189	0,041319
Cloud_Mesh_Distance_MCA_R0_R2	-12,48236847	-104,5737076	-943,0396729	-0,103916
Cloud_Mesh_Distance_MCA_R0_R2	-12,21575165	-104,5935211	-943,0998535	-0,14113
Cloud_Mesh_Distance_MCA_R0_R2	-12,31867599	-104,5462036	-943,1879273	-0,141568
Cloud_Mesh_Distance_MCA_R0_R2	-12,02832603	-104,6257782	-943,0634155	-0,136148
Cloud_Mesh_Distance_MCA_R0_R2	-11,7711277	-104,541069	-943,284668	-0,168374
Cloud_Mesh_Distance_MCA_R0_R2	-11,7810955	-104,6160507	-943,1013794	-0,134952
Cloud_Mesh_Distance_MCA_R0_R2	-11,45433807	-104,5104828	-943,2685547	-0,137472
Cloud_Mesh_Distance_MCA_R0_R2	-11,61170578	-104,566597	-943,1936035	-0,139621
Cloud_Mesh_Distance_MCA_R0_R2	-11,23724174	-104,5806809	-942,9354248	-0,04647
Cloud_Mesh_Distance_MCA_R0_R2	-11,08814621	-104,5211334	-942,9438477	-0,031518
Cloud_Mesh_Distance_MCA_R0_R2	-13,27350426	-103,9370041	-943,1486206	0,026993
Cloud_Mesh_Distance_MCA_R0_R2	-11,13452721	-104,5052567	-943,0548096	-0,065253
Cloud_Mesh_Distance_MCA_R0_R2	-11,11532593	-104,432106	-943,2199097	-0,097632
Cloud_Mesh_Distance_MCA_R0_R2	-10,97979164	-104,4467087	-943,0316773	-0,042075
Cloud_Mesh_Distance_MCA_R0_R2	-10,75631142	-104,3190079	-943,0617676	-0,009622
Cloud_Mesh_Distance_MCA_R0_R2	-10,96566582	-104,3806076	-943,1901856	-0,068833
Cloud_Mesh_Distance_MCA_R0_R2	-10,71677017	-104,3185577	-942,965332	0,01925
Cloud_Mesh_Distance_MCA_R0_R2	-13,28160286	-103,9535217	-942,9514771	-0,017158
Cloud_Mesh_Distance_MCA_R0_R2	-10,7508316	-104,2656479	-943,1983032	-0,034635

Cloud_Mesh_Distance_MCA_R0_R2	-10,49705505	-104,1636505	-943,0148926	0,063285
Cloud_Mesh_Distance_MCA_R0_R2	-10,34852982	-104,0483246	-943,065918	0,105164
Cloud_Mesh_Distance_MCA_R0_R2	-13,29318905	-103,7820435	-942,8948975	-0,055805
Cloud_Mesh_Distance_MCA_R0_R2	-10,24101448	-103,9685516	-943,0390015	0,132346
Cloud_Mesh_Distance_MCA_R0_R2	-10,25411606	-103,9586334	-943,1442871	0,119936
Cloud_Mesh_Distance_MCA_R0_R2	-10,28370476	-104,0069199	-942,9451904	0,12287
Cloud_Mesh_Distance_MCA_R0_R2	-10,18940544	-103,9274826	-942,9623413	0,145188
Cloud_Mesh_Distance_MCA_R0_R2	-10,14293289	-103,880928	-943,085083	0,151942
Cloud_Mesh_Distance_MCA_R0_R2	-10,07359505	-103,8147659	-942,9231567	0,165898
Cloud_Mesh_Distance_MCA_R0_R2	-13,24601173	-103,2593231	-942,7890625	-0,123357
Cloud_Mesh_Distance_MCA_R0_R2	-13,2595787	-103,4226151	-942,7775879	-0,126951
Cloud_Mesh_Distance_MCA_R0_R2	-9,94609261	-103,7008667	-943,0044556	0,193607
Cloud_Mesh_Distance_MCA_R0_R2	-9,84379578	-103,6048431	-943,0751953	0,205542
Cloud_Mesh_Distance_MCA_R0_R2	-13,26548004	-103,1663742	-943,022583	-0,053769
Cloud_Mesh_Distance_MCA_R0_R2	-13,25012875	-103,1545334	-942,8868408	-0,096018
Cloud_Mesh_Distance_MCA_R0_R2	-13,22598743	-103,0232086	-942,8441773	-0,086519
Cloud_Mesh_Distance_MCA_R0_R2	-9,853302	-103,5899277	-942,9211426	0,189789
Cloud_Mesh_Distance_MCA_R0_R2	-9,75605965	-103,4811401	-942,9104614	0,188231
Cloud_Mesh_Distance_MCA_R0_R2	-13,24442959	-103,0377426	-942,9863892	-0,061497
Cloud_Mesh_Distance_MCA_R0_R2	-13,22175884	-102,918335	-942,9448853	-0,055437
Cloud_Mesh_Distance_MCA_R0_R2	-13,19498634	-102,8457642	-942,815979	-0,066554
Cloud_Mesh_Distance_MCA_R0_R2	-9,67165947	-103,4188004	-943,0031128	0,197776
Cloud_Mesh_Distance_MCA_R0_R2	-9,585186	-103,3041611	-942,9325562	0,176531
Cloud_Mesh_Distance_MCA_R0_R2	-9,69026184	-103,3770523	-942,8372192	0,166525
Cloud_Mesh_Distance_MCA_R0_R2	-13,12047386	-102,4766388	-942,6972046	-0,025522
Cloud_Mesh_Distance_MCA_R0_R2	-9,39629364	-103,0649262	-942,8391724	0,117964
Cloud_Mesh_Distance_MCA_R0_R2	-9,24604607	-102,9863281	-943,0341797	0,124648
Cloud_Mesh_Distance_MCA_R0_R2	-9,29955482	-102,9856415	-942,883667	0,108658
Cloud_Mesh_Distance_MCA_R0_R2	-9,2426548	-102,8858719	-942,7788696	0,069391
Cloud_Mesh_Distance_MCA_R0_R2	-9,19683647	-102,898201	-942,9245606	0,088003
Cloud_Mesh_Distance_MCA_R0_R2	-13,08155727	-102,2901382	-942,6377564	0,009288
Cloud_Mesh_Distance_MCA_R0_R2	-9,13017273	-102,8002167	-942,835083	0,051195
Cloud_Mesh_Distance_MCA_R0_R2	-9,11201668	-102,8297195	-942,9733276	0,068813
Cloud_Mesh_Distance_MCA_R0_R2	-9,04405594	-102,688736	-942,7416992	0,069787
Cloud_Mesh_Distance_MCA_R0_R2	-12,97660637	-101,8759842	-942,6949463	0,084294
Cloud_Mesh_Distance_MCA_R0_R2	-12,89187145	-101,6900482	-942,7345581	0,094904
Cloud_Mesh_Distance_MCA_R0_R2	-12,75135708	-101,4674912	-942,5527954	0,091076
Cloud_Mesh_Distance_MCA_R0_R2	-12,70433903	-101,4060211	-942,4580689	0,089031
Cloud_Mesh_Distance_MCA_R0_R2	-12,80203819	-101,5413818	-942,6617432	0,089497
Cloud_Mesh_Distance_MCA_R0_R2	-12,79483986	-101,5471191	-942,8040772	0,077268
Cloud_Mesh_Distance_MCA_R0_R2	-12,67070293	-101,3809433	-942,5656738	0,071018
Cloud_Mesh_Distance_MCA_R0_R2	-12,16696739	-101,0627899	-942,6792603	-0,087287
Cloud_Mesh_Distance_MCA_R0_R2	-12,30644608	-101,1177979	-942,5437012	-0,031009
Cloud_Mesh_Distance_MCA_R0_R2	-12,28583717	-101,133873	-942,7444458	-0,058396
Cloud_Mesh_Distance_MCA_R0_R2	-12,16887283	-101,0440903	-942,5314331	-0,068858
Cloud_Mesh_Distance_MCA_R0_R2	-12,08657074	-100,9922104	-942,43396	-0,082783
Cloud_Mesh_Distance_MCA_R0_R2	-11,88947678	-100,9343948	-942,7069092	-0,146913
Cloud_Mesh_Distance_MCA_R0_R2	-11,93970299	-100,9295349	-942,5028076	-0,120934
Cloud_Mesh_Distance_MCA_R0_R2	-11,83022881	-100,8646011	-942,3738403	-0,127319
Cloud_Mesh_Distance_MCA_R0_R2	-11,78743362	-100,8675766	-942,5748291	-0,146799

Cloud_Mesh_Distance_MCA_R0_R2	-11,68712425	-100,8082581	-942,4734497	-0,152046
Cloud_Mesh_Distance_MCA_R0_R2	-11,40900993	-100,6972733	-942,6335449	-0,160203
Cloud_Mesh_Distance_MCA_R0_R2	-11,55318642	-100,7528915	-942,5429077	-0,163379
Cloud_Mesh_Distance_MCA_R0_R2	-11,45263672	-100,6936798	-942,4355469	-0,157665
Cloud_Mesh_Distance_MCA_R0_R2	-11,30387115	-100,6296005	-942,4959717	-0,152562
Cloud_Mesh_Distance_MCA_R0_R2	-13,11023331	-104,3479614	-942,8788452	0,017427
Cloud_Mesh_Distance_MCA_R0_R2	-13,19097137	-104,2437668	-942,8289185	-0,000874
Cloud_Mesh_Distance_MCA_R0_R2	-13,00557709	-104,4410782	-942,8632202	0,014453
Cloud_Mesh_Distance_MCA_R0_R2	-12,90760708	-104,5263214	-942,744751	0,008763
[truncated: 1,436,500 more chars]
